# Uncovering a bias in estimated treatment effects on PIRA in multiple sclerosis clinical trials

**DOI:** 10.1016/j.ebiom.2025.105802

**Published:** 2025-06-18

**Authors:** Noemi Montobbio, Francesca Bovis, Alessio Signori, Luca Carmisciano, Irene Schiavetti, Marta Ponzano, Carmen Tur, Cristina Granziera, Alessandro Cagol, Douglas L. Arnold, Ludwig Kappos, Maria Pia Sormani

**Affiliations:** a Department of Health Sciences (DISSAL), University of Genoa, Genoa, Italy; b Department of Clinical and Experimental Medicine, University of Pisa, Italy; c Neurologic Clinic and Policlinic, MS Centre, and Research Centre for Clinical Neuroimmunology and Neuroscience Basel (RC2NB), University Hospital and University of Basel, Basel, Switzerland; d Multiple Sclerosis Centre of Catalonia (Cemcat), Hospital Universitari Vall d'Hebron, Universitat Autònoma de Barcelona, Barcelona, Spain; e NeuroRx Research, Montréal, QC, Canada; f Montréal Neurological Institute, McGill University, Montréal, QC, Canada; g IRCCS Ospedale Policlinico San Martino, Genoa, Italy

**Keywords:** Multiple sclerosis, PIRA, Clinical trials, Treatment effect bias

## Abstract

**Background:**

Interest in progression independent of relapse activity (PIRA) as an endpoint in multiple sclerosis (MS) clinical trials is surging. However, established definitions of PIRA may produce biased treatment effect estimates in the presence of a treatment-induced relapse reduction.

**Methods:**

We applied different definitions of PIRA to pooled data from the OPERA I/II clinical trials (clinicaltrials.gov identifiers: NCT01247324, NCT01412333). Treatment effects on PIRA according to different methods were quantified by hazard ratios (HRs) and risk ratios (RRs). Next, we evaluated the bias in each definition using synthetic Expanded Disability Status Scale (EDSS) data simulating a control and an experimental arm with varying treatment effects on relapses and on PIRA. We quantified the bias by comparing the estimated effect on PIRA with the known true effect.

**Findings:**

The pooled OPERA I/II population included 1656 participants. Estimated treatment effects on PIRA varied from a non-significant HR of 0.83 (CI = 0.66–1.04) to an HR of 0.73 (CI = 0.59–0.90) depending on the definition used. Follow-up analyses on simulated data (n = 800 per arm) revealed an underestimation of the true treatment effect on PIRA when using established definitions, with increasing bias as treatment effect on relapses increased. Defining PIRA as complementary to relapse-associated worsening (RAW) provided a less biased and operationally simple alternative.

**Interpretation:**

For clinical trials with PIRA as an endpoint, we suggest a “complementary” definition of PIRA, relying on accurate exclusion of RAW promoted by appropriate visit timing.

**Funding:**

10.13039/501100003407Italian Ministry of University and Research.

**Research in context**

**Evidence before this study**

Established definitions of progression independent of relapse activity (PIRA) in multiple sclerosis have attempted to increase specificity by requiring wide relapse-free intervals surrounding a disability worsening event.

**Added value of this study**

This study featuring real and simulated clinical trial data showed that, in the context of a clinical trial where the treatment reduces relapses, the treatment's effect on PIRA is underestimated by established definitions. Defining PIRA as complementary to relapse-associated worsening (RAW) considerably reduced such bias.

**Implications of all the available evidence**

For clinical trials aiming to apply PIRA as an endpoint of interest, we recommend defining PIRA as complementary to RAW, with the critical requirement that visit schedules are appropriately timed to ensure accurate detection of all RAW events.

## Introduction

Clinical trials for relapsing multiple sclerosis (MS) have historically used annualised relapse rate (ARR) or confirmed disability accrual (CDA) as endpoints. Recently, several studies^1^, ^2^, ^3^ have introduced the notion of progression independent of relapse activity (PIRA), indicating a clinical deterioration unrelated to acute episodes already in the earliest phase of the disease. PIRA has raised growing interest as a trial endpoint for drugs with potential mechanisms of action targeting progression. While CDA remains the most sensible endpoint to assess the overall clinical benefit of a therapy, disentangling treatment effects on different sources of disability worsening is critical for our understanding of mechanisms of action. However, separating the relapse-associated and relapse-independent contributions to disability worsening only based on the available assessments is a challenging task. In principle, the notion of PIRA is complementary to the notion of relapse-associated worsening (RAW). As such, their operational definitions should ideally be based on antithetical criteria. As long as they are not, some CDA events may potentially be left “undefined” as they cannot be classified as either PIRA or RAW. In practice, the feasibility of defining PIRA as operationally complementary to RAW rests on the possibility to correctly detect all RAW events—which in turn depends on the relative timing of visits and relapses within the available data. A RAW event is generally defined by the onset of a relapse within a pre-specified time interval preceding the event. On the other hand, established definitions of PIRA typically categorise an event as PIRA only in the absence of relapses within wider intervals, defined not only by the date of the event but also by the baseline and confirmation visits.^2^, ^3^, ^4^, ^5^ This conservative approach (i.e., prioritising specificity while potentially losing in sensitivity) is justified when the information available does not allow accurate detection of all RAW events. However, such approach is often presented in the literature as a general recommendation, regardless of the spacing of visits in the data under study. Critically, the probability of discarding true PIRA events grows as the frequency of relapses increases, translating into a lower sensitivity of conservative PIRA definitions in patients experiencing more relapses. On top of this, assuming the presence of an underlying progression independent of relapses does not preclude its occurrence *during* acute events. The impossibility of disentangling simultaneous relapse-independent and relapse-associated disability accrual events affects any clinical-based definition of PIRA. This generates an intrinsic distortion, which is larger when the relapse rate is higher, and is further amplified by conservative definitions.

In the context of a clinical trial where the treatment impacts both relapses and PIRA, a lower post-randomisation relapse rate in the treated arm can create an imbalance in the sensitivity of PIRA detection between the two arms, with more true PIRA events overlooked in the control arm. When comparing the two arms, this would lead to an underestimation of treatment effect on PIRA. Minimising this bias is imperative for evaluating new therapies in terms of their effect on PIRA.

A similar reasoning applies when estimating the effect of treatment on progression independent of relapse *and* magnetic resonance imaging (MRI) activity (PIRMA,^6^ or PIA^7^). Considering lesion activity in addition to clinical relapses increases the complexity of the issue, as the spacing of MRI assessments does not typically permit precise determination of the onset of new lesion formation. To isolate the analysis of bias in PIRA definitions from additional variability introduced by the detection of MRI lesions, the present study will focus exclusively on clinical relapses and PIRA.

Herein, we compare estimates of treatment effect on PIRA obtained by different methods used in previous publications.^2^^,^^5^^,^^8^ We also introduce and evaluate two additional methods, defining PIRA as complementary to RAW. We first compare all the examined methods on the pooled OPERA I and OPERA II trial data^9^ to demonstrate the impact of varying the definition of PIRA in a scenario with a post-randomisation imbalance in relapse rate between the two trial arms. Next, we use artificial data simulating a randomised controlled clinical trial where the “true effect” is known by construction, to quantify the bias introduced by the different approaches.

## Methods

### Ethics

The present study re-analysed data from the OPERA I (clinicaltrials.gov identifier, NCT01247324) and OPERA II (NCT01412333) clinical trials. Trial protocols (Supplementary Data) have been approved by the relevant institutional review boards and ethics committees.

### Main definitions

The endpoint of interest was 12-week-confirmed PIRA. All the alternative definitions of PIRA detailed below build on the common definition of CDA as a worsening in the disability score (as detailed in the next sections) confirmed over 12 or more weeks. A visit could not be used for confirmation if occurring within 30 days from the onset of a relapse. We adopted a roving baseline scheme^10^ where the baseline was reset at the confirmation of every improvement or worsening event. When analysing the OPERA I/II data, to reproduce the results of a previous re-analysis^2^ (see next section), we also implemented a relapse-based re-baseline scheme resetting the reference value with the first available assessment 30 or more days after the onset of each relapse with a value not lower than the original baseline value. We defined a RAW event as a CDA event occurring within 90 days from the onset of a relapse. RAW events are further categorised into transient (followed by complete recovery) and sustained (leading to permanent disability accumulation).

The methods that we evaluated for estimating treatment effect on PIRA can be grouped into the following three main approaches.

The first approach relied on the classification of certain CDA events as PIRA based on a relapse-independence constraint. Treatment effect was quantified as both the hazard ratio (HR) for the time to first PIRA, and the risk ratio (RR) for PIRA. We tested the following definitions adopted in previous studies, requiring an absence of relapses in the intervals specified as follows:

- between the reference visit and the confirmation visit, as recommended by Müller et al.^5^ for scenarios where a high specificity is desired (Standard1);
- between the reference visit and 30 days after the event, and during the 30 days before and 30 days after the confirmation visit, as used by Kappos et al.^2^ (Standard2);
- during the 90 days before and 30 days after the event, and during the 90 days before and 30 days after the confirmation visit, as recommended by Müller et al.^5^ (Standard3).

The Standard1–3 definitions are sorted roughly from the most conservative to the least conservative in terms of the width of relapse-free intervals (Supplementary Figure S1). Additionally, we introduced and tested a fourth, less conservative definition only requiring an absence of relapses in the 90 days preceding the CDA event. This definition, hereafter termed Non-RAW, identifies PIRA as complementary to RAW.

As a second approach, instead of directly searching for events from the whole disability trajectory, we first extracted the relapse-independent component (RIC) of the trajectory, and then detected CDA events from it. Treatment effect was quantified as both an HR and an RR based on those events. We introduced the trajectory decomposition rationale in a previous study on observational data.^11^ The procedure, described in detail in the original publication, isolates the relapse-independent component of the disability trajectory by taking into account the magnitude of transient and sustained RAW events. Hereafter, this method will be referred to as RIC.

The third approach that we tested is based on a Bayesian Principal Stratum (BPS) model. The method had been applied in previous work^8^^,^^12^ to estimate the effect of siponimod on PIRA in secondary progressive MS, using data from the EXPAND trial.^13^ This technique estimates treatment effect on PIRA as the RR for CDA in the theoretical subgroup of “non-relapsing” patients (individuals who would not relapse irrespective of treatment assignment). Herein, we adapted the method for application in a relapsing population. Please refer to Supplementary Methods S1 for details on model assumptions and inference.

To summarise, we compared the following six alternative methods to estimate treatment effect on PIRA: Standard1, Standard2, Standard3, Non-RAW, RIC, and BPS (RR only). Note that this study compares different strategies for estimating treatment effect on PIRA *once the definition of CDA is set*. Whichever of the six methods is used to estimate treatment effect on PIRA, the estimated overall effect on CDA remains unchanged.

### Estimating treatment effects on PIRA in the OPERA I/II data

We tested all methods on pooled data from the OPERA I and OPERA II trials, which assessed the effect of Ocrelizumab vs. Interferon β-1a in relapsing MS. Study details and eligibility criteria have been previously reported.^9^ The data have been re-analysed by Kappos et al.^2^ to characterise the contribution of PIRA to CDA using a composite endpoint. Specifically, CDA was defined as an increase of ≥1.0 or ≥0.5 EDSS points if baseline EDSS was ≤5.5 or >5.5, respectively, or an increase of ≥20% in the Timed 25-Foot Walk score, or an increase of ≥20% in the Nine-Hole Peg Test score, confirmed over 12 or more weeks. The authors defined composite PIRA (i.e., PIRA based on the composite endpoint) by applying the above definition of CDA coupled with the Standard2 constraint for relapse-independence and relapse-based re-baseline.

Herein, we re-computed the treatment effect on composite PIRA with the same criteria for CDA adopted in the previous publication,^2^ but varying the PIRA definition (Standard1–3, Non-RAW, RIC, or BPS) and the baseline scheme (relapse-based re-baseline, or roving baseline). HRs were estimated from time-to-event data using Cox proportional hazards models stratified by study and by baseline EDSS dichotomised into <4.0 or ≥4.0 points (different than in the previous publications,^2^^,^^9^ geographic region data were not available for this study). RRs were estimated from the detected events via log-binomial regression (or by Bayesian inference in the BPS method) adjusted for the same covariates.

### Quantifying the bias in estimated treatment effects

Analyses on the OPERA I/II data can highlight potential differences in estimated treatment effects across methods. However, in the absence of a “ground truth”, we cannot establish which of these methods are biased and in which direction. To address this question, we tested all methods on artificial EDSS data simulating a clinical trial where the true treatment effect was known by construction. We generated different scenarios varying the treatment effect on PIRA and on relapses (Supplementary Methods S3 and Supplementary Table S1).

For each of the artificial datasets, we estimated treatment effects on PIRA using all methods, adopting a roving baseline scheme. We chose to re-baseline only after confirmed non-PIRA events rather than after any relapse (possibly not causing CDA) to reduce the bias introduced by moving the baseline based on post-randomisation events. The effect of treatment on PIRA was quantified by HRs (using Cox models with time to first PIRA as a dependent variable, and no additional covariates) and RRs (computed as the ratio between estimated PIRA probabilities in the treated vs. control arm).

**Fig. 1:**
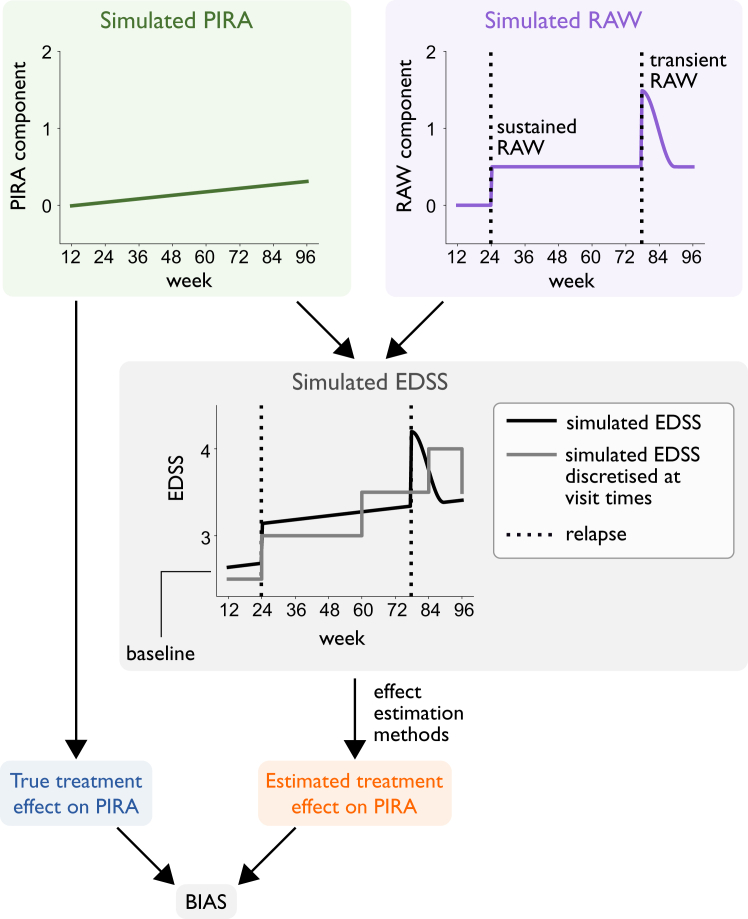
**Schematic illustration of the simulation framework used to quantify the bias in estimated treatment effect on PIRA.** Abbreviations: PIRA, progression independent of relapse activity; RAW, relapse-associated worsening; EDSS, Expanded Disability Status Scale.

In the artificial data, the relapse-independent and relapse-associated EDSS worsening trajectories were simulated separately. The true treatment effect on PIRA could therefore be estimated as the effect of treatment on CDA based on the relapse-independent trajectory alone. The true treatment effect was then compared to the effect estimated by each method from the combined trajectory (Fig. 1). Bias was quantified as the difference between the estimated effect and the true effect (for both HRs and RRs). For each configuration of simulation parameters, all results were averaged over 200 data simulations with different random seeds to ensure stability.

### Statistics

We used the *msprog* R package^14^ for event detection and categorisation. In the re-analysis of the OPERA data, 95% confidence intervals (CIs) for HRs and RRs were estimated using the standard error of the estimated coefficients (from Cox and log-binomial models, respectively), except for the BPS method, where CIs for RRs were estimated via bootstrap as detailed in the original publication.^12^ In the analysis on simulated data, all CIs were computed via bootstrap over the 200 data simulations.

### Role of funders

The study sponsors were not involved in study design, in the collection, analysis, or interpretation of data, in the writing of the report, or in the decision to submit the paper for publication.

## Results

### Variations in estimated treatment effect on PIRA in the OPERA I/II data

In the pooled OPERA I/II trial data, relapse rate was reduced by 45% in the Ocrelizumab arm (ARR = 0.16) with respect to the Interferon arm (ARR = 0.29),^9^ providing a representative example of relapse rate imbalance between trial arms. The OPERA I and OPERA II clinical trials, conducted between April 2011 and August 2015, included 1656 (out of 2096 eligible) participants with relapsing MS from 56 countries (Supplementary Figure S2). Details on participant flow, recruitment, baseline demographics, and trial results have been previously reported.^9^ The re-analysis by Kappos et al.,^2^ using the Standard2 definition, estimated an HR of 0.78 (95% CI = (0.63, 0.98)) for composite PIRA.

**Fig. 2:**
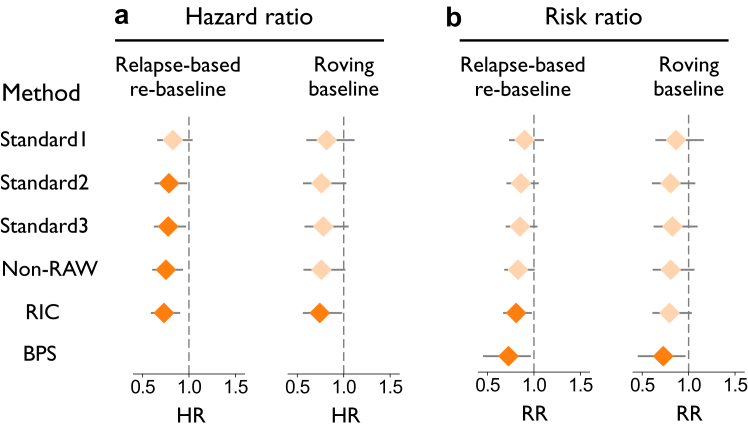
**Estimated treatment effects on PIRA on the OPERA I/II data (n = 1656), obtained using different methods.** Panel a shows HRs as obtained with the Standard1–3 and Non-RAW methods. Panel b shows RRs as obtained with the Standard1–3, Non-RAW, and BPS methods. Markers in a lighter shade indicate that 1.00 is included in the 95% confidence interval. Abbreviations: RR, risk ratio; HR, hazard ratio; PIRA, progression independent of relapse activity; RAW, relapse-associated worsening; RIC, relapse-independent component; BPS, Bayesian Principal Stratum.

Estimated effects of treatment on composite PIRA in the OPERA I/II data showed considerable variation depending on the method used. When applying the original relapse-based re-baseline scheme, HRs ranged from a non-significant 0.83 (CI = (0.66, 1.04)) with the Standard1 definition, to 0.73 (CI = (0.59, 0.90)) with the RIC definition (Fig. 2a). Similarly, RRs ranged from 0.90 (CI = (0.73, 1.11)) with the Standard1 definition, to 0.81 (CI = (0.67, 0.97)) with the RIC definition (Fig. 2b). The BPS method estimated an even lower RR of 0.73 (CI = (0.45, 0.97)), although with a wider CI. A comparable pattern of results was observed when applying a roving baseline scheme (Fig. 2).

Our results show that different methods for estimating treatment effect on PIRA lead to different conclusions on the OPERA I/II data. These differences are likely driven by the imbalance in relapse rate between trial arms, and may be sharpened in situations with a larger imbalance. These observations motivated our next analysis, using simulated data to assess bias and its relationship with relapse rate imbalance.

### A systematic bias in estimated treatment effect on PIRA

**Fig. 3:**
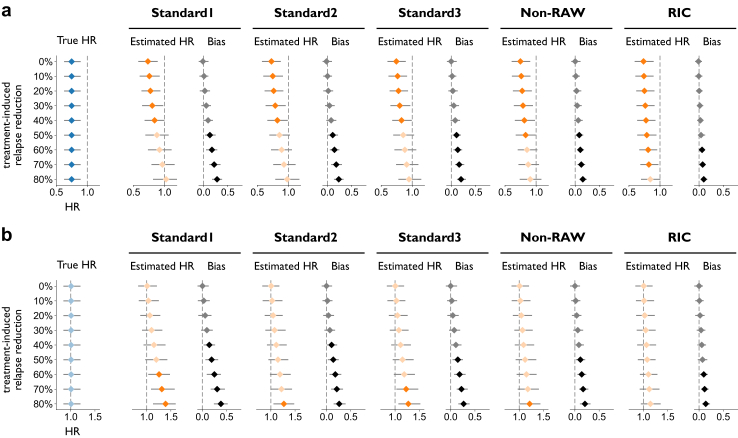
**True vs. estimated HR for PIRA as a function of treatment-induced relapse reduction in simulated data (n = 1600), for a scenario with a mean relapse rate of 0.5 events/year in the control arm, reduced by 0–80% in the treated arm.** True and estimated HRs are displayed in blue and orange, respectively. Results are reported for **(a)** a true HR of 0.75 for PIRA, or **(b)** no true effect on PIRA. All HRs are displayed with bootstrapped 95% confidence intervals; markers in a lighter shade indicate that 1.00 is included in the confidence interval of the HR. Estimated HRs are obtained by using, respectively, the Standard1–3, Non-RAW, and RIC methods detailed in the Methods. The bias on HR for each definition and scenario is also reported (in grey), with its relative confidence interval; here, markers in a lighter shade indicate that 0.00 is included in the confidence interval. Abbreviations: HR, hazard ratio; PIRA, progression independent of relapse activity; RAW, relapse-associated worsening; RIC, relapse-independent component.

Simulated data included 800 subjects per arm. We compared the true HR for PIRA against the estimates obtained with the Standard1–3, Non-RAW, and RIC definitions, in two simulated scenarios. Fig. 3a reports the results in a situation with a true HR for PIRA of 0.75 (CI = (0.62, 0.89)) and where the control arm had a mean ARR of 0.5, which was reduced by 0–80% in the treated arm (“scenario A”). Note that this includes nine sub-scenarios, only differing for treatment effect on relapses.

The Standard1 method progressively underestimated the true treatment effect on PIRA as the effect on relapses increased, until no significant treatment effect on PIRA was detected already for a 50% relapse reduction (estimated HR = 0.88, CI = (0.69, 1.07), bias = 0.13, CI = (0.03, 0.25)). The distortion increased as the simulated effect on relapses was further raised, with a bias as large as 0.28 (CI = (0.18, 0.38)) at 80%. A similar picture was found when applying definitions Standard2 and Standard3, both failing to detect a significant effect on PIRA once simulated relapse reduction reached 50%, although with a less severe bias (maximum bias = 0.23, CI = (0.13, 0.33) for the Standard2 method; 0.20, CI = (0.12, 0.30) for the Standard3 method). As expected, a further decrease in bias was observed when applying the Non-RAW definition (maximum bias = 0.16, CI = (0.09, 0.24)). Finally, the RIC method correctly detected a significant treatment effect on PIRA up to a relapse reduction rate of 70%. The bias was significant, although of limited extent, for a relapse reduction of 60% or more (maximum bias = 0.10, CI = (0.04, 0.17)).

Fig. 3b reports the results obtained in a simulated scenario with no true effect on PIRA (true HR = 1.00, CI = (0.85, 1.20)) and a mean ARR of 0.5 in the control arm, reduced by 0–80% in the treated arm (“scenario B”). The results obtained using the Standard1 definition were significantly biased starting from a 40% relapse reduction, reaching a bias of 0.38 (CI = (0.24, 0.52)) at 80%. Notably, they mistakenly indicated a harmful effect of treatment on PIRA once relapse reduction reached 60% (estimated HR = 1.26, CI = (1.03, 1.48)). Similar to scenario A, an intermediate situation was observed for definitions Standard2 (maximum bias = 0.26, CI = (0.15, 0.39)), Standard3 (maximum bias = 0.27, CI = (0.16, 0.39)), and Non-RAW (maximum bias = 0.21, CI = (0.10, 0.32)), again estimating a detrimental effect of treatment on PIRA for relapse reductions of 70–80%. As before, the RIC definition was the most effective at limiting the bias on HR (maximum bias = 0.14, CI = (0.06, 0.23)).

**Fig. 4:**
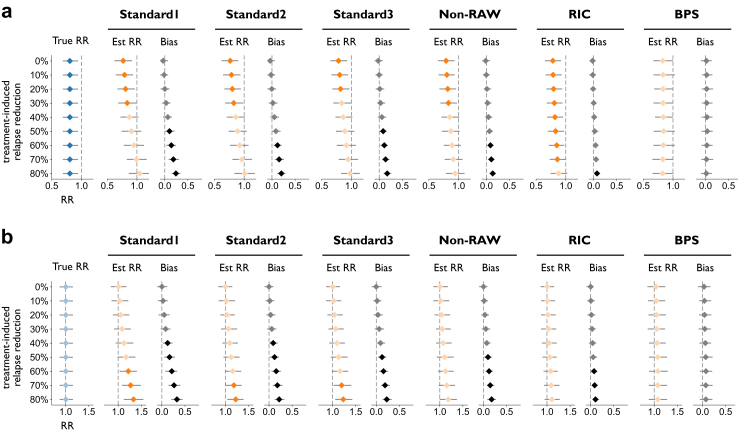
**True vs. estimated RR for PIRA as a function of treatment-induced relapse reduction in simulated data (n = 1600), for a scenario with a mean relapse rate of 0.5 events/year in the control arm, reduced by 0–80% in the treated arm.** True and estimated RRs are displayed in blue and orange, respectively. Results are reported **(a)** a true RR of 0.81 for PIRA, or **(b)** no true effect on PIRA. All RRs are displayed with bootstrapped 95% confidence intervals; markers in a lighter shade indicate that 1.00 is included in the confidence interval. Estimated RRs are obtained by using, respectively, the Standard1–3, Non-RAW, RIC, and BPS definitions detailed in the Methods. The RR bias for each definition and scenario is also reported (in black), with its relative confidence interval; here, markers in a lighter shade indicate that 0.00 is included in the confidence interval. Abbreviations: Est, estimated; RR, risk ratio; PIRA, progression independent of relapse activity; RAW, relapse-associated worsening; RIC, relapse-independent component; BPS, Bayesian Principal Stratum.

Fig. 4 compares the true RR for PIRA with the RR estimated using the Standard1–3, Non-RAW, RIC, and BPS methods in the same two simulated scenarios examined above. In scenario A (Fig. 4a), the true RR for PIRA was 0.81 (CI = (0.69, 0.94)). In scenario B (Fig. 4b), the true RR for PIRA was 1.00 (CI = (0.87, 1.16)). As already observed for HRs from the Standard1–3, Non-RAW, and RIC methods, treatment effect was increasingly underestimated as the simulated treatment effect on relapses increased, with the RIC method minimising the bias (maximum bias = 0.07, CI = (0.02, 0.14) in scenario A; maximum bias = 0.10, CI = (0.01, 0.18) in scenario B). An even more accurate treatment effect estimate was provided by the BPS method, which was completely unbiased even for the strongest relapse rate imbalance (maximum bias = 0.02, CI = (−0.09, 0.14) in scenario A; maximum bias = 0.07, CI = (−0.06, 0.21) in scenario B). It should be noted that the BPS method tends to produce wider CIs as compared to the other examined methods, therefore estimating a non-significant treatment effect on PIRA in scenario A, even though the point estimate of the RR is the closest to the true effect. Moreover, treatment effects on PIRA estimated by the BPS method in the presence of a strong effect on relapses may deviate drastically depending on model assumptions (see Supplementary Methods S2 and Supplementary Tables S2 and S3).

The results remained comparable when the mean ARR in the control arm was set to 0.4 or 0.3 (Supplementary Figure S3), although overall less biased due to the smaller absolute difference in relapses between the two arms. The observed trend suggests that an even larger bias would be observed for a mean ARR higher than 0.5 in the control group.

## Discussion

This study analysed how the sensitivity of different PIRA definitions is affected by relapse rate, and the implications of this when estimating treatment effects on PIRA in clinical trials where relapse rate is reduced by the treatment. Results on the OPERA I/II data highlighted substantial differences in the effects estimated using different methods, with the weakest effects emerging for highly conservative definitions, and the strongest effect arising when using a BPS framework. Our follow-up analysis on artificial data uncovered an intrinsic bias in established definitions of PIRA based on relapse-free intervals, which are less sensitive in trial arms with higher post-randomisation relapse rate. This observation implies that, for example, the effect of Ocrelizumab on PIRA computed in previous analyses^2^ may be underestimated, due to the strong effect of Ocrelizumab on relapses.

To evaluate treatment effect on PIRA in a clinical trial context, we recommend using the RIC method, which defines PIRA as complementary to RAW in a trajectory-based manner, by detecting CDA events after subtracting all relapse-associated contributions to disability worsening from the overall trajectory.^11^ The method provides a finer characterisation of relapse-independence as compared to post-hoc event categorisation, thus limiting the bias while keeping the estimation of HRs and RRs operationally simple.

The BPS method yields an unbiased estimate of treatment effect on PIRA, providing the most accurate point estimates of RRs among the examined methods, although with wider CIs. The algorithm quantifies treatment effects as RRs, but may be extended to time-to-event data, expressing the results as HRs. While out of scope for the present study, this may be the object of future work. The BPS method was originally applied^8^^,^^12^ on data from a population of patients with progressive MS. When applied to a relapsing population where the treatment has a strong effect on relapses, the identifiability of the estimand is challenged. In the present study, we established a reasonable set of additional assumptions to narrow the range of feasible values in such a setting. Our sensitivity analyses showed that results may deviate drastically when removing these constraints. Moreover, despite providing an unbiased estimate of treatment effect on PIRA in our analyses, this may not generalise to different data distributions. The BPS method is based on Markov chain Monte Carlo estimation of RRs in a theoretical subgroup without directly detecting individual-patient PIRA events, thus preventing full interpretability of the results; deviations originating from improper model specification may therefore go unnoticed in real data where no ground truth is available to test the method's accuracy. While the BPS method is a powerful statistical tool, these observations call for caution when applying it to data from a relapsing population.

Within the familiar context of PIRA definitions based on relapse-free intervals, our results showed that the bias on estimated treatment effect on PIRA is minimised by applying the Non-RAW method. Similar to the RIC method, this definition is based on a principle of complementarity of PIRA and RAW, but implements it in an event-based manner, without taking into account the magnitude of relapse-associated changes. This approach may be preferable when a simpler algorithm that aligns with established practices is desired.

Any operational definition of PIRA should be based on (and only be applicable under) assumptions on the relative timing of visits and relapses. It is important to note that, to implement PIRA as complementary to RAW, the RIC and Non-RAW methods rely on accurate detection of relapse-associated events, which requires appropriate visit timing in trial design. This applies if patients always have a visit within the predetermined influence period of each relapse (the time interval used to define RAW, e.g., 90 days from relapse onset). Such a condition is guaranteed, for instance, when the visit spacing is equal to or shorter than such interval. On the other hand, if visit timing does not allow correct identification of all RAW events, a complementary definition of PIRA could potentially lead to many false positives. This can be the case in large observational cohorts, where relapses are not as well characterised as in clinical trials, and the space between visits is usually large (6 months–1 year). The consequent risk of an overestimation of PIRA events in these contexts led to the introduction of highly conservative definitions of PIRA.^5^ While these approaches may help address specificity issues in observational studies, their use should not be extended to clinical trials, where sufficient specificity is promoted by regular and frequent assessments. More generally, the *least conservative* definition that is justifiable based on the available data and chosen criteria should be selected. This last point is also relevant in the clinical practice, where the characterisation of a patient's disease course may influence treatment strategies.

One limitation of this study is that it focused solely on clinical relapses and PIRA. Nonetheless, its implications extend to the broader concept of PIRMA,^6^ or PIA.^7^ Similar to effects on clinical relapses, a treatment-induced reduction of lesion formation will generate a post-randomisation imbalance in lesion activity between trial arms. In our simulation analysis, the rationale articulated throughout the paper would still be applicable if acute events included both relapse onset dates and new lesion formation dates. However, the latter are not easily determined from existing clinical trial data, due to the coarseness of MRI assessments: the formation of newly detected lesions may have occurred at any time in between the previous MRI assessment and the current one. This poses an additional challenge to the detection of “activity-associated worsening” events, a prerequisite for a complementary definition of PIRMA. Future clinical trials aiming to analyse PIRMA as an endpoint will need to first ensure sufficient longitudinal characterisation of lesion activity by increasing the frequency of MRI assessments.

Comparing treatment effects on PIRA estimated by different definitions on real data does not give an indication on which method is most appropriate. For instance, selecting the method which estimates the strongest effect may lead to inflating the true effect. A strength of our study lies in also testing the different definitions on simulated data, which allows to access the ground-truth treatment effect and compare it with the effect estimated by each definition. We are aware that artificially generated data always depend on several assumptions about the mechanism under study (e.g., the linear course of PIRA). However, our results on simulated data show a pattern consistent with the treatment effects estimated on the OPERA I/II trial data, demonstrating how relapse rate imbalance across trial arms can affect the estimation of treatment effect on PIRA.

In conclusion, for clinical trials aiming to analyse PIRA as an endpoint of interest, we suggest adopting a complementary definition of PIRA, relying on accurate identification of all RAW events based on appropriate predetermined visit timing. As a trade-off between accuracy and feasibility, we recommend the “hybrid” RIC method, which computes event-based HRs for PIRA after removing relapse-associated changes in a trajectory-based manner.

## Contributors

All authors read and approved the final version of the manuscript and take responsibility for the decision to submit it. The underlying data was directly accessed and verified by NM and FB.

Conceptualisation: MPS, NM; data curation: DLA, FB, MPS; formal analysis: NM, FB, AS, LC, IS, MP, MPS; funding acquisition: MPS, FB; investigation: all authors; methodology: all authors; project administration: DLA, MPS; resources: MPS, DLA; software: NM; supervision: MPS, DLA, LK; validation: AC, CT, CG, FB, AS, LC, IS, MP, MPS; visualisation: NM; writing – original draft: NM, MPS; writing – review & editing: all authors.

## Data sharing statement

Data used in this work were collected within the International Progressive MS Alliance project (IPMSA, award reference number PA-1603-08175). Access requests should be forwarded to the data controller (https://www.progressivemsalliance.org/contact-us/). Queries and requests may also be directed to mariapia.sormani@unige.it.

## Declaration of interests

NM, AS, LC, IS, MP have nothing to disclose. FB received the 2022 Biostatistics/Informatics Junior Faculty Award (grant code BI-2107-38160) awarded by the National MS Society. The University of Genoa, as the employer of AS, received grants from Roche. AS reported receiving consultancy fees from Horizon and Novartis, and participating on boards for Argenx Italy s.r.l and Roche. CT reported receiving the following grants or contracts: Miguel Servet contract, awarded by the Instituto de Salud Carlos III (ISCIII) (CP23/00117), ‘Proyecto de Investigación’ Research Grants (PI21/01860 and PI24/01277) and FORTALECE research grant (FORT23/00034) awarded by the ISCIII, Ministerio de Ciencia e Innovación de España, a 2020 Junior Leader La Caixa Fellowship (fellowship code: LCF/BQ/PI20/11760008), awarded by “la Caixa” Foundation (ID100010434), the 2021 Merck's Award for the Investigation in MS, awarded by Fundación Merck Salud (Spain), a 2015 ECTRIMS Post-doctoral Research Fellowship, and funding from the UK MS Society; payments/honoraria and travel support from Roche, Novartis, Merck, Sanofi, Immunic Therapeutics, Bristol Myers Squibb. The University Hospital Basel (USB) and the Research Center for Clinical Neuroimmunology and Neuroscience (RC2NB), as the employers of CG, have received fees, which were used exclusively for research support, from Siemens, GeNeuro, Novartis, Biogen, Sanofi-Genzyme and Hoffmann La Roche; and advisory board and consultancy fees from Actelion, Genzyme-Sanofi, Hoffmann La Roche, Biogen, Novartis, GeNeuro. AC reported receiving grants from the Horizon 2020 Eurostar program (grant E!113682), and payment/honoraria from Roche. DLA reported receiving the Collaborative Network Award from the International Progressive MS Alliance, the AI & MS Discovery Grant from the Multiple Sclerosis Society of Canada, and consulting fees from Biogen, BMS, EMD Serono, Frequency Therapeutics, Idorsia Pharmaceuticals, Merck, Race to Erase MS, Sanofi. The University Hospital Basel (USB) and the Research Center for Clinical Neuroimmunology and Neuroscience (RC2NB), as the employers of LK, received grants from the European Union and Innosuisse; consulting fees from Bayer, Biogen, Celltrion Inc, Eli Lilly (Suisse) SA, Galapagos NV, Immunic AG, Janssen, Kiniksa Pharmaceuticals, Laboratoires Juvise Pharmaceuticals, Merck Healthcare AG, MSD Merck Sharp & Dohme AG, Novartis, Sanofi, Shionogi BV, Wellmera AG, Zai Lab; payment/honoraria from Bristol Myers Squibb, Janssen, Novartis, Roche; Df-mp Molina & Pohlman; participation on boards or committees from Clene Nanomedicine Inc., Minorix Therapeutics S.L., Neurostatus, Sanofi, EMD Serono Research and Development, Genentech, Roche. MPS reported receiving consulting fees from Biogen, Merck, Novartis, Sanofi, Roche, Immunic, participating on boards for Novartis, Roche, Orchard, Boeringher, and serving as Vice president of the Clinical Trial Committee (USMS national Society and ECTRIMS) and Chair of the ECTRIMS Educational Committee.

## Appendix A Supplementary data

Supplementary Materials

OPERA Protocol
