# Supplementary material for: Uncovering a bias in estimated treatment effects on PIRA in multiple sclerosis clinical trials
Source: eBioMedicine. 2025 Jun 18;117:105802. doi: 10.1016/j.ebiom.2025.105802 (PMC12219353; doi:10.1016/j.ebiom.2025.105802)
Supplement: Supplementary Materials [file mmc1.docx]

**Supplement to:**

**Uncovering a bias in estimated treatment effects on PIRA in multiple sclerosis clinical trials**

Noemi Montobbio, Francesca Bovis, Alessio Signori, Luca Carmisciano, Irene Schiavetti, Marta Ponzano, Carmen Tur, Cristina Granziera, Alessandro Cagol, Douglas L. Arnold, Ludwig Kappos, Maria Pia Sormani

**Contents:**

**Supplementary Methods.**

1. Bayesian Principal Stratum: mathematical setting and model assumptions
2. Bayesian Principal Stratum: sensitivity analysis
3. Generation of synthetic trial data

**Supplementary Table S1.** Parameters used for simulating EDSS data in all examined scenarios.

**Supplementary Table S2.** Sensitivity analysis for BPS assumptions on OPERA I/II trial data.

**Supplementary Table S3.** Sensitivity analysis for BPS assumptions on synthetic trial data.

**Supplementary Figure S1.** Relapse-free intervals in definitions Standard 1-3 and Non-RAW.

**Supplementary Figure S2.** Patient disposition: pooled OPERA I and OPERA II studies.

**Supplementary Figure S3.** Summary of the bias on estimated treatment effect on PIRA obtained in all examined scenarios, for a reduction in relapses of 30%, 50%, or 70%.

**Supplementary Methods S1. Bayesian Principal Stratum: mathematical setting and model assumptions**

Among the methods tested in the present study to estimate treatment effect on PIRA, we examined the Bayesian Principal Stratum (BPS) approach applied by Magnusson *et al.*^1^ and Cree *et al.*^2^ to estimate the effect of siponimod on PIRA in data from the EXPAND trial.^3^ Here, we shall give a short introduction of the general framework, and discuss model assumptions in our specific setting. Please refer to the original publications for a more detailed description of the method.

In a two-arm randomised controlled clinical trial, every patient has two potential binary outcomes $Y$ for CDA, independent of treatment assignment:

- $Y(0)$: the occurrence of CDA under control treatment;
- $Y(1)$: the occurrence of CDA under experimental treatment.

For each treatment $z\in\{0,1\}$,

$$Y\left( z \right)=\left\{ \begin{aligned} 0 \left( no CDA \right) \\ 1 \left( CDA \right) \end{aligned} \right. . (1)$$

Clearly, for each patient, only the outcome corresponding to the treatment actually assigned is observed.

Similarly, the potential occurrence of post-randomisation relapses subdivides the patients into four mutually exclusive *principal strata*:

- Non-relapsing, or “immune” stratum ($\mathcal{I}$): patients who would not relapse regardless of treatment assignment;
- Definite-relapsing, or “doomed” stratum ($\mathcal{D}$): patients who would relapse regardless of treatment assignment;
- Benefiter stratum ($\mathcal{B}$): patients who would only relapse if assigned to placebo;
- Harmed stratum ($\mathcal{H}$): patients who would only relapse if assigned to treatment.

Being based on *potential* relapse occurrence, belonging to each principal stratum can be considered as a baseline covariate, independent of observed relapses. In BPS analysis, treatment effect on PIRA in the whole population is estimated by restricting the analysis to the non-relapsing stratum, where such estimate is not confounded by post-randomisation imbalances in relapse rate. Clearly, this relies on the assumption that the action of treatment on PIRA does not differ between relapsing and non-relapsing patients. Within the subgroup of non-relapsing patients, the effect on PIRA coincides with the effect on overall CDA. Treatment effect on PIRA is thus estimated as the treatment effect on CDA in the subgroup of non-relapsing patients. Specifically, the estimand of interest is the RR in the non-relapsing stratum $\mathcal{I}$, i.e., the ratio between the CDA probabilities within the non-relapsing stratum in the two trial arms ($z=0,1$):

$$RR= \frac{\mathbb{P}\left( Y\left( 1 \right)=1 \right|\mathcal{I)}}{\mathbb{P}\left( Y\left( 0 \right)=1 \right|\mathcal{I)}} . (2)$$

As patients were not observed under both treatments, membership within the non-relapsing stratum (and CDA probability therein) cannot be established directly, and needs to be estimated via Bayesian inference. If the non-relapsing stratum $\mathcal{I}$ is large relative to the benefiter stratum $\mathcal{B}$, then the range of feasible values for $\mathbb{P}\left( Y\left( 1 \right)=1 \right|\mathcal{I)}$will be narrow, thus leading to accurate estimates without making further assumptions. This is verified when treatment effect on relapses is negligible, or when very few on-study relapses are observed in both trial arms (as was the case in the previous studies on progressive patients^1,2^). Conversely, in a relapsing population where the treatment has a strong effect on relapses, the benefiter stratum $\mathcal{B}$ will be large relative to the non-relapsing stratum $\mathcal{I}$. This is the case in the OPERA I/II data analysed in the present study, and is also relevant in our follow-up analysis on synthetic data. In this situation, the range of feasible values for the numerator of Eq. (2) will be wider, thus requiring additional assumptions to further identify it. A reasonable option, as proposed by Magnusson et al.^1^, is to assume that CDA probability under treatment $z=1$ is lower in the benefiter stratum than in the definite-relapsing stratum:

$$\mathbb{P}\left( Y\left( 1 \right)=1 \right|\mathcal{B)}\leq\mathbb{P}\left( Y\left( 1 \right)=1 \right|\mathcal{D).} (3)$$

Additionally, in the context of a strong treatment-induced reduction in relapses, we may provide the model with some prior information on the expected effect of relapse removal on disability. In the original publication^1^, the effect of treatment on CDA is parameterised via an additive term $\Delta_{g}$ on the logit scale:

$$logit\left[ \mathbb{P}\left( Y\left( 1 \right)=1 \right|stratum=g) \right]= logit\left[ \mathbb{P}\left( Y\left( 0 \right)=1 \right|stratum=g) \right]+\Delta_{g} (g=\mathcal{I,D,B,H)}$$

with $\Delta_{g} \sim N(0, \sigma)$ (where $\sigma$ depends on the number of covariates). This means assuming no prior effect of treatment on CDA in all strata. However, in the benefiter ($\mathcal{B}$) stratum, we can assume that treatment effect on CDA is at least the same as the difference in CDA probability between non-relapsing and relapsing patients observed *in the placebo arm* ($z=0$). A similar reasoning with opposite sign applies to the harmed ($\mathcal{H}$) stratum. This results in the following stratum-specific priors for $\Delta$:

$$\Delta_{\mathcal{I}} \sim N\left( 0, \sigma\right), \Delta_{\mathcal{D}} \sim N(0, \sigma)$$

$$\Delta_{\mathcal{B}}\sim N\left( logit\left[ \mathbb{P}\left( Y\left( 0 \right)=1 \right|not relapsing) \right]- logit\left[ \mathbb{P}\left( Y\left( 0 \right)=1 \right|relapsing) \right], \sigma\right) (4)$$

$$\Delta_{\mathcal{H}}\sim N(logit\left[ \mathbb{P}\left( Y\left( 0 \right)=1 \right|relapsing) \right]- logit\left[ \mathbb{P}\left( Y\left( 0 \right)=1 \right|not relapsing) \right], \sigma)$$

To summarise, in our main analyses we introduced the following two modifications to the original model to adapt it to our setting:

1. we added a penalty term to the likelihood function of the model to encode the constraint in Eq. (3);
2. we set stratum-specific priors for treatment effect on CDA as in Eq. (4).

Except for these modifications, model specification and fitting were carried out following the same procedure detailed by Magnusson et al.^1^. In particular, Bayesian inference was performed via Markov chain Monte Carlo (MCMC) methods with the No-U-Turn (NUTS) sampler. This was implemented in Python using the *PyMC* library (version 7.17.0).

All other methods tested in the study explicitly detect PIRA events for each individual patient. This provides full information on time to event and whether an event was observed, thus allowing for straightforward calculation of both RRs and HRs. In the BPS method, treatment effect on PIRA is modelled indirectly by estimating the probability of belonging to the non-relapsing stratum and the CDA probability therein, obtaining the RR in Eq. (2). The method may be extended to include time-to-event information by integrating Bayesian survival modelling into the principal stratum framework, thus replacing the estimand in Eq. (2) with an HR. However, this was out of the scope of the present study.

**Supplementary Methods S2. Bayesian Principal Stratum: sensitivity analysis**

To study the stability of BPS results with respect to the assumptions, we conducted a sensitivity analysis by individually removing the assumptions described by Eqs. (3-4).

Supplementary Table S2 reports the RRs for PIRA estimated on the OPERA I/II data with and without each of the assumptions. Notably, removing all additional assumptions yielded an RR of 0.52 (95%CI=(0.19, 0.83)), thus estimating a much stronger effect as compared to the result of our main analysis (RR=0.73, 95%CI=(0.45,0.97)).

We conducted the same sensitivity analysis on synthetic data in the two main scenarios examined in the text (scenario A: true RR of 0.81 for PIRA; scenario B: no true effect on PIRA), by keeping the simulated effect on relapses fixed at a 70% reduction. The results are summarised in Supplementary Table S3. Similar to what we observed on the OPERA I/II data, removing any of the assumptions reduced the RRs computed by the BPS method. This corresponded to an overestimation of the true effect of treatment on PIRA (negative bias on RR). Removing both assumptions led to considerable bias (–0.27, 95%CI=(–0.35, –0.19) in scenario A; –0.30, 95%CI=(–0.40, –0.20) in scenario B).

**Supplementary Methods S3. Generation of synthetic trial data**

The artificial data generated in the present study consisted of 12-week-spaced EDSS values simulated over a follow-up of 96 weeks. The EDSS course was generated as a relapse-independent linear growth combined with relapse-associated jumps sampled from a negative binomial distribution. EDSS worsening events linked to relapses were designed to be either sustained (a jump in EDSS leading to permanent disability accumulation, 30% of relapses), or transient (a jump in EDSS followed by complete recovery, 70% of relapses).^4^ The generated EDSS values were constrained to fall within the 0-10 range, and subsequently discretized to multiples of 0.5 (Figure 1).

A similar procedure could be applied to simulate any type of (single or composite) progression measures, or acute inflammatory activity. We chose to focus on EDSS and clinical relapses solely for simplicity in description.

We simulated a control arm and an experimental arm (n=800 per arm, a typical size for a clinical trial) with the following varying parameters:

- mean ARR in the control arm (0.3, 0.4, or 0.5 events per year);
- treatment effect on relapses (0%, 10%, …, or 80% reduction);
- treatment effect on PIRA (25% hazard rate reduction, or no effect).

Within each arm, the patient-specific EDSS at baseline, relapse rate, and relapse-independent progression rate were sampled from a normal distribution, and truncated to fall within a reasonable interval (e.g., excluding negative or extremely high positive rates). Supplementary Table S1 summarises the distribution parameters used for each configuration of the two arms, and the thresholds applied to truncate the sampled values.

The two options for treatment effect on PIRA define the two main scenarios under study (“scenario A”: 25% hazard rate reduction; “scenario B”: no effect). In the main analysis (Figures 3-4), the mean ARR in the control arm was kept fixed at 0.5, while the ARR in the treated arm varied (0-80% reduction compared to the control arm). Alternative scenarios with a control-arm mean ARR of 0.4 or 0.3 were explored in additional analyses (Supplementary Figure S3).

**References**

1. Magnusson BP, Schmidli H, Rouyrre N, Scharfstein DO. Bayesian inference for a principal stratum estimand to assess the treatment effect in a subgroup characterized by postrandomization event occurrence. Stat Med. 2019;38(23):4761-4771. doi:10.1002/sim.8333.
2. Cree BA, Magnusson B, Rouyrre N, et al. Siponimod: Disentangling disability and relapses in secondary progressive multiple sclerosis. Mult Scler. 2021;27(10):1564-1576. doi:10.1177/1352458520971819.
3. Kappos L, Bar-Or A, Cree BAC, et al. Siponimod versus placebo in secondary progressive multiple sclerosis (EXPAND): a double-blind, randomised, phase 3 study [published correction appears in Lancet. 2018 Nov 17;392(10160):2170. doi: 10.1016/S0140-6736(18)32834-4]. Lancet. 2018;391(10127):1263-1273. doi:10.1016/S0140-6736(18)30475-6.
4. Lublin FD, Häring DA, Ganjgahi H, et al. How patients with multiple sclerosis acquire disability. Brain. 2022;145(9):3147-3161. doi:10.1093/brain/awac016.

**Supplementary Table S1. Parameters used for simulating EDSS data in all examined scenarios.**

|  | **Control arm** | | **Experimental arm** | | **Sampled values truncated to interval** |
| --- | --- | --- | --- | --- | --- |
| **Variable** | **mean** | **SD** | **mean** | **SD** |  |
| EDSS at baseline | 3.0 | 2.0 | 3.0 | 2.0 | [0.0, 5.0] |
| Relapse-independent progression rate (EDSS points per year) | 0.15 | 1.00 | 0.15; 0.11 | 1.00; 0.73 | [0.01, 5.00] |
| Relapse rate (events per year) | 0.30  0.40  0.50 | 1.00 | 0.30; …; 0.06^a^  0.40; …; 0.08^b^  0.50; …; 0.10^c^ | 1.00; …; 0.20 | [0.01, 5.00] |

^a^Relapse reduction of 0%,…,80% with respect to a relapse rate of 0.30 in the control arm.

^b^Relapse reduction of 0%,…,80% with respect to a relapse rate of 0.40 in the control arm.

^c^Relapse reduction of 0%,…,80% with respect to a relapse rate of 0.50 in the control arm.

Abbreviations: EDSS, Expanded Disability Status Scale; SD, standard deviation.

**Supplementary Table S2. Results of the sensitivity analysis for BPS assumptions on OPERA I/II trial data.**

|  | | **Eq. (3) constraint** | |
| --- | --- | --- | --- |
|  |  | **yes** | **no** |
| **Eq. (4) priors** | **yes** | 0.73 (0.45, 0.97)^a^ | 0.56 (0.23, 0.90) |
|  | **no** | 0.72 (0.49, 0.95) | 0.52 (0.19, 0.83) |

^a^Configuration adopted in the main analysis.

Abbreviations: BPS, Bayesian Principal Stratum.

**Supplementary Table S3. Results of the sensitivity analysis for BPS assumptions on synthetic trial data.**

| **True treatment effect on PIRA** | **Eq. (3) constraint** | **Eq. (4) priors** | **Estimated RR (95% CI)** | **Bias (95% CI)  against true effect** |
| --- | --- | --- | --- | --- |
| True RR=0.81 (scenario A) | yes | yes | 0.83 (0.65, 1.00)^a^ | 0.02 (–0.09, 0.17)^a^ |
|  | yes | no | 0.75 (0.59, 0.91) | –0.05 (–0.17, 0.09) |
|  | no | yes | 0.70 (0.59, 0.83) | –0.10 (–0.17, –0.02) |
|  | no | no | 0.54 (0.46, 0.63) | –0.27 (–0.35, –0.19) |
| True RR = 1.00 (scenario B) | yes | yes | 1.07 (0.88, 1.28)^a^ | 0.07 (–0.05, 0.22)^a^ |
|  | yes | no | 0.98 (0.80, 1.19) | –0.02 (–0.15, 0.14) |
|  | no | yes | 0.92 (0.75, 1.10) | –0.08 (–0.18, 0.03) |
|  | no | no | 0.70 (0.58, 0.86) | –0.30 (–0.40, –0.20) |

^a^Configuration adopted in the main analysis.

Abbreviations: BPS, Bayesian Principal Stratum; PIRA, progression independent of relapse activity; RR, risk ratio; CI, confidence interval.

**Supplementary Figure S1. Relapse-free intervals in definitions Standard 1-3 and Non-RAW.**


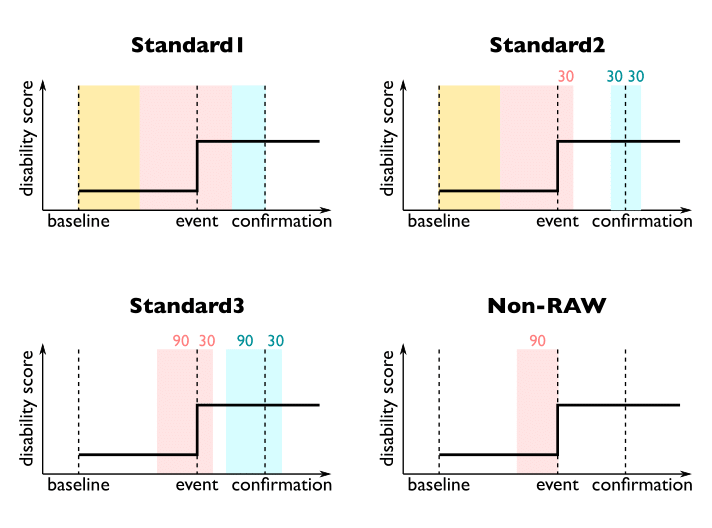


Numbers over intervals represent days. Abbreviations: RAW, relapse-associated worsening.

**Supplementary Figure S2. Patient disposition: pooled OPERA I and OPERA II studies.**


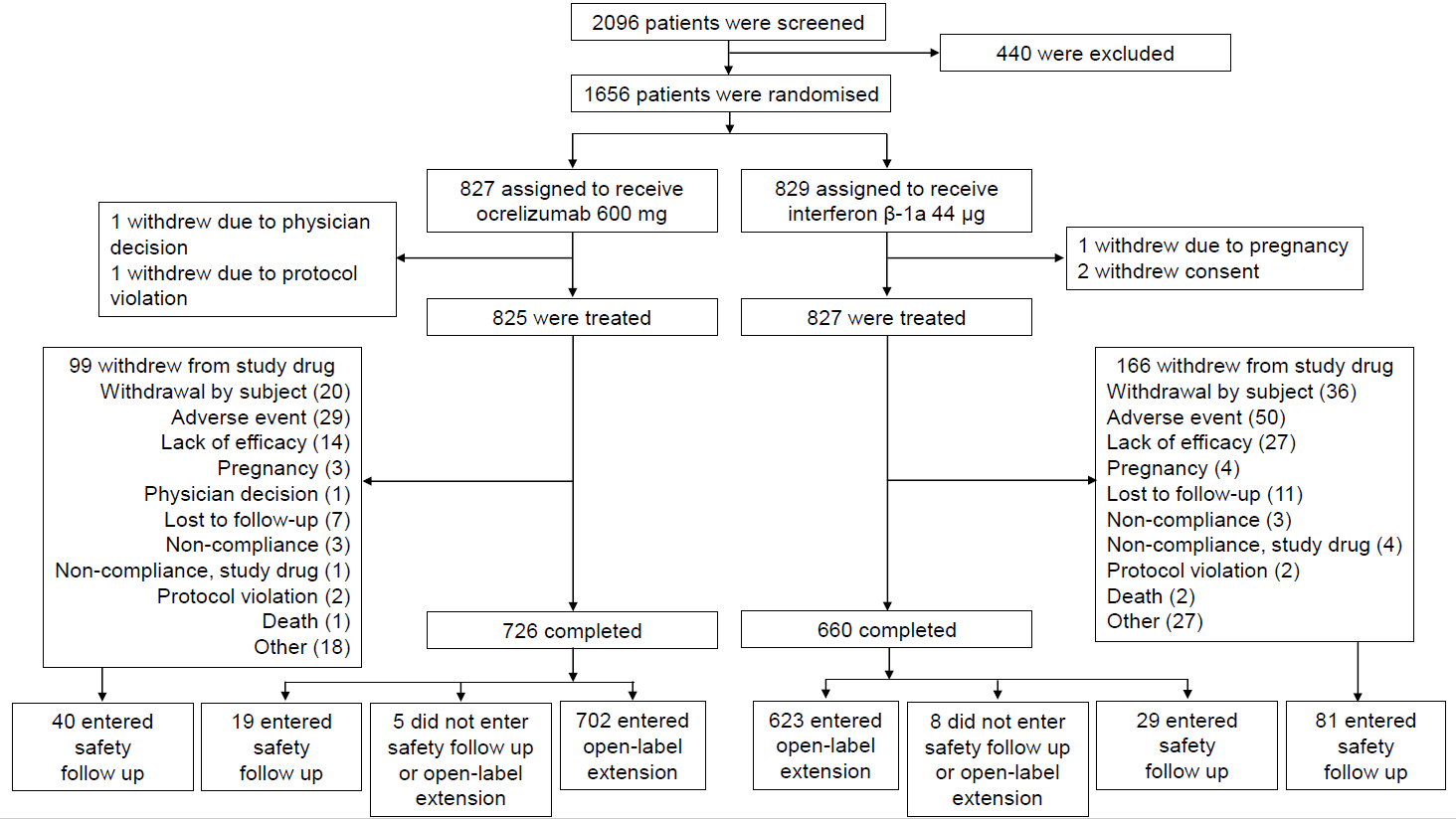


The intent-to-treat population consisted of all randomized patients, including those who prematurely withdrew from the study for any reason and for whom an assessment was not performed for whatever reason; in addition, patients who received an incorrect therapy from that which was intended were summarized according to their randomized treatment. Four patients in total (1 patient in each arm of the two trials, OPERA I and OPERA II) withdrew from study drug due to protocol violation.

**Supplementary Figure S3. Summary of the bias on estimated treatment effect on PIRA obtained in all examined scenarios, for a reduction in relapses of 30%, 50%, or 70%**


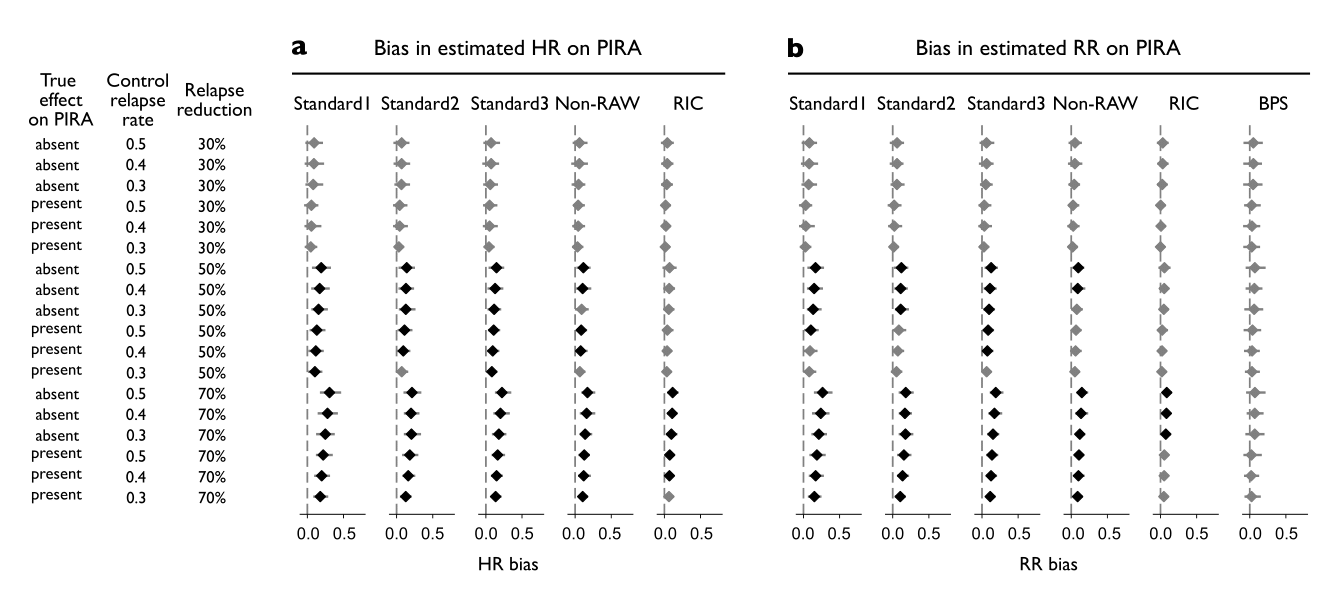


Markers in grey indicate that 0.00 is included in the confidence interval. Abbreviations: PIRA, progression independent of relapse activity; RAW, relapse-associated worsening; RIC, relapse-independent component; BPS, Bayesian Principal Stratum; HR, hazard ratio; RR, risk ratio.
