# Supplementary material for: Uncovering a bias in estimated treatment effects on PIRA in multiple sclerosis clinical trials
Source: eBioMedicine. 2025 Jun 18;117:105802. doi: 10.1016/j.ebiom.2025.105802 (PMC12219353; doi:10.1016/j.ebiom.2025.105802)
Supplement: OPERA Protocol [file mmc2.pdf]

Study codes: NCT01247324, NCT01412333

**A Study of Ocrelizumab in Comparison With Interferon Beta-1a (Rebif) in Patients With Relapsing Multiple Sclerosis**

This supplement contains the following items:


1. Original OPERA I study protocol = PDF pages 2–152
2. Final OPERA I study protocol = PDF pages 153–346
3. Summary of changes to OPERA I study protocol = PDF pages 347–348
4. Original OPERA II study protocol = PDF pages 349–498
5. Final OPERA II study protocol = PDF pages 499–692
6. Summary of changes to OPERA II study protocol = PDF pages 693–694
7. Original individual study statistical analysis plan = PDF pages 695–747
8. Final individual study statistical analysis plan = PDF pages 748–815
9. Summary of changes made to the individual statistical analysis plans = PDF pages 749–750
10. Original pooled study statistical analysis plan = PDF pages 816–830
11. Final pooled study statistical analysis plan = PDF pages 831–858
12. Summary of changes made to the pooled statistical analysis plans = PDF page 832

# F. HOFFMANN-LA ROCHE LTD
# CLINICAL STUDY PROTOCOL
# PROTOCOL NUMBER WA21092B
# RO 4964913

# EUDRACT NUMBER 2010-020337-99

## PROTOCOL APPROVAL

Protocol Number / Version: WA21092 / B

Date: See last date in electronic signature manifestation below.

Protocol approved by: See electronic signature manifestation below.

| Name | Reason for Signing | Date and Time (UTC) |
|---|---|---|
| Glanzman, Robert | Clinical Science Leader | 01-Jun-2011 23:40:52 |

**Confidentiality Statement**

The information contained in this document, especially unpublished data, is the property of F. Hoffmann-La Roche Ltd (or under its control), and therefore provided to you in confidence as an investigator, potential investigator or consultant, for review by you, your staff and an applicable Independent Ethics Committee/Institutional Review Board. It is understood that this information will not be disclosed to others without written authorization from Roche except to the extent necessary to obtain informed consent from those persons to whom the drug may be administered.

## SPECIAL NOTE REGARDING PROTOCOL NUMBER WA21092/B

Because of the great extent of changes to the previous version of Protocol WA21092 (Version A) made in this amendment (Version B), this protocol does not specify new or deleted text herein.

For a detailed summary of the changes, please refer to the separate amendment history.

# SYNOPSIS OF PROTOCOL NUMBER WA21092/B

| | | | |
|---|---|---|---|
| TITLE | **A Randomized, Double-Blind, Double-Dummy, Parallel-Group Study To Evaluate The Efficacy And Safety Of Ocrelizumab In Comparison To Interferon Beta-1a (Rebif®) In Patients With Relapsing Multiple Sclerosis** | | |
| SPONSOR | F.Hoffmann-La Roche Ltd<br>Genentech Inc | CLINICAL PHASE | III |
| INDICATION | Relapsing Multiple Sclerosis | | |
| OBJECTIVES | **Primary:**<br>The primary objective of this study is to assess whether the efficacy of ocrelizumab 600 mg (given as dual infusions of 300 mg on Days 1 and 15 of the first 24-week treatment cycle and as a single infusion of 600 mg on Day 1 of each 24-week treatment cycle thereafter) intravenously every 24 weeks is superior to Rebif® as measured by the annualized <u>protocol-defined*</u> relapse rate by two years (96 weeks) in patients with relapsing multiple sclerosis.<br><br>**Secondary:**<br>To evaluate whether the efficacy of ocrelizumab is superior to Rebif®, as reflected by the following measures:<br>• The time to onset of sustained disability progression for at least 12 weeks during the 96-week comparative treatment period.*<br>• The time to onset of sustained disability progression for at least 24 weeks during the 96-week comparative treatment period.*<br>• The proportion of relapse-free patients by 96 weeks.<br>• The change in total T2 lesion volume as detected by brain MRI from baseline to Week 96.<br>• The total number of new, and/or enlarging T2 hyperintense lesions as detected by brain MRI at weeks 24, 48 and 96.<br>• The change in Multiple Sclerosis Functional Composite Scale (MSFCS) score from baseline to Week 96.<br>• The change in brain volume as detected by brain MRI from Week 24 to Week 96.<br>**See section: "Assessments of efficacy" for the definition*<br><br>**Safety:**<br>To evaluate the safety and tolerability of ocrelizumab 600 mg (given as dual infusions of 300 mg on Days 1 and 15 of the first 24-week treatment cycle and as a single infusion of 600 mg on Day 1 of each 24-week treatment cycle thereafter) intravenously every 24 weeks in patients with relapsing MS.<br><br>**Pharmacokinetics/Pharmacodynamics:**<br>To explore the pharmacokinetics, immunogenicity and pharmacodynamics of ocrelizumab in patients with relapsing MS.<br><br>**Exploratory objectives:**<br>• The change in low contrast visual acuity from baseline to Weeks 48 and 96.<br>• The change in the Symbol Digit Modalities Test from baseline to Weeks 48 and 96.<br>• The annualized relapse rate, based on clinical and protocol-defined relapses at the end of the 96-week comparative treatment period.<br>• The total number of T1 gadolinium-enhanced lesions as detected by brain MRI at weeks 24, 48 and 96. | | |

| | |
|---|---|
| | • The change in brain volume as detected by brain MRI from baseline to Week 96.<br>• The change in brain volume as detected by brain MRI from week 48 to Week 96.<br>• The change in Multiple Sclerosis Functional Composite Scale (MSFCS) score from baseline to Week 48.<br>• The cumulative change in EDSS scores, measured in area under the curve (AUC) by Week 96.<br>• The change in EDSS from baseline to Weeks 48 and 96.<br>• The change in timed 25 foot walk from baseline to Weeks 48 and 96.<br>• The change in 9-hole peg test from baseline to Weeks 48 and 96.<br>• The change in paced auditory serial addition test (PASAT) from baseline to Weeks 48 and 96.<br>• The time to onset of sustained 20% increase in 9-hole peg test for at least 12 weeks.<br>• The time to onset of sustained 20% increase in timed 25 foot walk for at least 12 weeks.<br>• Patient Reported Outcomes (PROs): Modified Fatigue Impact Scale (MFIS), EuroQol instrument (EQ-5D), Center for Epidemiology Studies Depression Scale (CES-D) and Medical Outcomes Study 36-Item Short Form Survey Instrument (SF-36) at baseline, Week 48 and Week 96.<br>• The change in Karnofsky Performance Status Scale (clinician-reported version) from baseline to Weeks 48 and 96. |
| TRIAL DESIGN | Multicentre, randomized, double-blind, double-dummy, parallel-group study |
| NUMBER OF SUBJECTS | 800 patients in total, 400 patients per group using a 1:1 randomization ratio. Please refer to Sample Size and Statistical Analyses section of the synopsis for more details. |
| TARGET POPULATION | **Inclusion criteria:**<br>1. Ability to provide written, informed consent and to be compliant with the schedule of protocol assessments.<br>2. Ages 18-55 years at screening, inclusive.<br>3. Diagnosis of MS, in accordance with the revised McDonald criteria (2010).<br>4. At least 2 documented clinical attacks within the last 2 years prior to screening or one clinical attack in the year prior to screening (but not within 30 days prior to screening).<br>5. Neurological stability for ≥ 30 days prior to both screening and baseline.<br>6. EDSS, at screening, from 0 to 5.5 inclusive.<br>7. Documented MRI of brain with abnormalities consistent with MS prior to screening.<br>8. Patients of reproductive potential must use reliable means of contraception as described below as a minimum (adherence to local requirements, if more stringent, is required*):<br>  • Two methods of contraception throughout the trial, including the active treatment phase AND for 48 weeks after the last dose of ocrelizumab, or until their B-cells have repleted, whichever is longer. Acceptable methods of contraception include one primary (e.g. systemic hormonal contraception or tubal ligation of the female partner, vasectomy of the male partner) AND one secondary barrier method (e.g. latex condoms, spermicide) OR a double barrier method (e.g. latex condom, intrauterine device, vaginal ring or pessary plus spermicide [e.g. foam, vaginal suppository, gel, cream]). |

9. For patients of <u>non reproductive potential</u> (adherence to local requirements, if more stringent, is required*):
   - Women may be enrolled if postmenopausal (i.e. spontaneous amenorrhea for the past year confirmed by an FSH level greater than 40 mIU/mL) unless the patient is receiving a hormonal therapy for their menopause or surgically sterile (i.e. hysterectomy, complete bilateral oophorectomy);
   - Men may be enrolled if they are surgically sterile (castration).

* Based on local Ethics Committees or National Competent Authority feedback additional requirements to assure contraception or to confirm menopause may be required (e.g. serum estradiol compatible with post-menopause status, longer duration of amenorrhea, higher level of FSH).

**Exclusion Criteria**

Patients who meet the following criteria must be excluded from study entry:

1. Diagnosis of primary progressive MS.
2. Disease duration of more than 10 years in patients with an EDSS $\leq 2.0$ at screening.
3. Inability to complete an MRI (contraindications for MRI include but are not restricted to claustrophobia, weight $\geq$ 140 kg, pacemaker, cochlear implants, presence of foreign substances in the eye, intracranial vascular clips, surgery within 6 weeks of entry into the study, coronary stent implanted within 8 weeks prior to the time of the intended MRI, etc).
4. Known presence of other neurological disorders which may mimic MS including but not limited to: neuromeylitis optica, Lyme disease, untreated vitamin B12 deficiency, neurosarcoidosis and cerebrovascular disorders.

**Exclusions Related to General Health**

5. Pregnancy or lactation.
6. Any concomitant disease that may require chronic treatment with systemic corticosteroids or immunosuppressants during the course of the study.
7. History or currently active primary or secondary immunodeficiency.
8. Lack of peripheral venous access.
9. History of severe allergic or anaphylactic reactions to humanized or murine monoclonal antibodies.
10. Significant or uncontrolled somatic disease or any other significant disease that may preclude patient from participating in the study.
11. Congestive heart failure (NYHA III or IV functional severity).
12. Known active bacterial, viral, fungal, mycobacterial infection or other infection, excluding fungal infection of nail beds.
13. Infection requiring hospitalization or treatment with i.v. antibiotics within 4 weeks prior to baseline visit or oral antibiotics within 2 weeks prior to baseline visit.
14. History or known presence of recurrent or chronic infection (e.g., hepatitis B or C, HIV, syphilis, tuberculosis).
15. History of progressive multifocal leukoencephalopathy (PML)
16. History of malignancy, including solid tumors and hematological malignancies, except basal cell carcinoma, *in situ* squamous cell carcinoma of the skin, and *in situ* carcinoma of the cervix of the uterus that have been previously completely excised with documented, clear margins.
17. History of alcohol or drug abuse within 24 weeks prior to baseline.
18. History or laboratory evidence of coagulation disorders.

**Exclusions Related to Medications***

19. Receipt of a live vaccine within 6 weeks prior to baseline.
    *In rare cases when patient requires vaccination with a live vaccine, the screening period may be extended but cannot exceed 8 weeks.*
20. Treatment with any investigational agent within 24 weeks of screening (Visit 1) or five half-lives of the investigational drug (whichever is longer).
21. Contraindications to or intolerance of oral or i.v. corticosteroids, including methylprednisolone administered i.v., according to the country label, including:
    a) Psychosis not yet controlled by a treatment;
    b) Hypersensitivity to any of the constituents.
22. Contraindication to Rebif® or incompatibility with Rebif® use, including:
    a) Current severe depression and/or suicidal ideation;
    b) Hypersensitivity to natural or recombinant interferon-β, or to any excipients;
    c) Previous suboptimal response to High Dose High Frequency (HDHF) interferon or cessation of HDHF interferon therapy due to poor tolerability;
    d) Prior cessation of Rebif® therapy due to toxicity, which is likely to recur.
23. Treatment with β interferons (with exemptions for HDHF interferon as listed above), glatiramer acetate, plasmapheresis, or other immunomodulatory therapies within 4 weeks prior to baseline.
24. Treatment with dalfampridine (Ampyra®) unless on stable dose for ≥ 30 days prior to screening. Patients should remain on stable doses throughout the 96 week treatment period.
25. Previous treatment with B-cell targeted therapies (i.e. rituximab, ocrelizumab, atacicept, belimumab or ofatumumab).
26. Systemic corticosteroid therapy within 4 weeks prior to screening.**
27. Any previous treatment with alemtuzumab (Campath), anti-CD4, cladribine, mitoxantrone, daclizumab, BG12, teriflunomide, laquinimod, total body irradiation or bone marrow transplantation.
28. Treatment with cyclophosphamide, azathioprine, mycophenolate mofetil (MMF), cyclosporine, methotrexate or natalizumab within 24 months prior to screening. NB. P*atients previously treated with natalizumab will be eligible for this study only if duration of treatment with natalizumab was < 1 year.*
29. Treatment with fingolimod (FTY720, Gilenya®) or other S1P receptor modulator (i.e. BAF312) within 24 weeks prior to screening. NB. *Only patients with T lymphocyte count ≥ LLN will be eligible for this study.*
30. Treatment with i.v. immunoglobulin within 12 weeks prior to baseline.

** Patients screened for this study should not be withdrawn from therapies for the sole purpose of meeting eligibility for the trial. Patients, who discontinue their current therapy for non-medical reasons, should specifically be informed before deciding to enter the study of their treatment options.*
*** The screening period may be extended (but cannot exceed 8 weeks) for patients who have used systemic corticosteroids for their MS before screening. For a patient to be eligible, systemic corticosteroids should not have been administered also between screening and baseline.*

| | |
|---|---|
| | **<u>Exclusions Related to Laboratory Findings*</u>**<br>31. Positive serum β hCG measured at screening.<br>32. Positive screening tests for hepatitis B (hepatitis B surface antigen [HBsAg] positive, or positive hepatitis B core antibody [total HBcAb] confirmed by a positive viral deoxyribonucleic acid [DNA] polymerase chain reaction [PCR]) or hepatitis C (HepCAb).<br>33. Positive rapid plasma reagin (RPR).<br>34. CD4 count < 300/µL.<br>35. AST/SGOT or ALT/SGPT ≥ 2.0 Upper Limit of Normal (ULN).<br>36. Platelet count <100,000/µL (<100 x $10^9$/L).<br>37. Levels of serum IgG <5.65 g/L.<br>38. Levels of serum IgM < 0.55 g/L.<br>39. Total neutrophil count <1.5 x $10^3$/µL.<br><br>*<u>*Re-testing before baseline</u>: in rare cases in which the screening laboratory samples are rejected by the central laboratory (example: hemolyzed sample) or the results are not assessable (example: indeterminate) or abnormal, the tests need to be repeated within 4 weeks. The last value before randomization must meet study criteria. In such circumstances, the screening period may need to be prolonged but should not exceed 8 weeks.*<br><br><u>Please note:</u> based on local Ethics Committees or National Competent Authority requirements, additional diagnostic testing may be required for selected patients or selected centers to exclude tuberculosis, Lyme disease, HTLV-1 associated myelopathy (HAM), acquired immune deficiency syndrome (AIDS), hereditary disorders, connective tissue disorders, or sarcoidosis. Other specific diagnostic tests may be requested when deemed necessary by the investigator**.** |
| LENGTH OF STUDY | The study will consist of the following periods:<br>- **Screening period**: 2 weeks.<br><br>*In rare cases (i.e. if the screening laboratory samples are rejected by the central laboratory) the screening period may be extended but cannot exceed 8 weeks.*<br><br>**- Treatment period:** 96 weeks<br><br>The double-blind comparative treatment period will consist of 4 treatment cycles 24 weeks apart.<br><br>*Patients who complete the 96 week treatment period may become eligible for a separate, open-label extension study, under a separate protocol.*<br><br>**-Safety Follow up Period**: at least 48 weeks;<br><br>Patients who withdraw from study treatment will be observed for a period of at least 48 weeks counting from the date of the last infusion of study drug. If at this time the peripheral blood B-cells remain depleted patient should continue to be monitored at 24-week intervals until B-cell count has returned to the baseline value or to the lower limit of the normal range (whichever is the lower).<br><br>See section “Procedures” below for more details. |

| | |
|---|---|
| END OF STUDY | The end of the study has been defined as the date at which the last data point from the last patient, which was required for statistical analysis as defined in Data Analysis Plan (DAP), was received. |
| INVESTIGATIONAL MEDICAL PRODUCT(S) DOSE/ ROUTE/ REGIMEN | – Group A (Ocrelizumab): Ocrelizumab 600 mg (given as dual infusions of ocrelizumab 300 mg on Days 1 and 15 of the first 24-week treatment cycle and as single infusions of 600 mg on Day 1 for each 24-week treatment cycle, thereafter) every 24 weeks.<br><br>– Group B (Interferon beta-1a (Rebif®))<br>Rebif® will be administered as follows:<br>▪ Treatment initiation:<br>⇒ during weeks one and two, Rebif® 8.8 µg (one pre-filled syringe (0.2 mL) containing 8.8 µg (2.4 MIU) of interferon beta-1a given via subcutaneous injection three times per week<br>⇒ during weeks three and four, Rebif® 22 µg (one pre-filled syringe (0.5 mL) containing 22 µg (6 MIU) of interferon beta-1a) given via subcutaneous injection three times per week<br>▪ Treatment continuation:<br>⇒ From the fifth week onwards, Rebif® 44 µg (one pre-filled syringe (0.5 mL) containing 44 µg (12 MIU) of interferon beta-1a) given via subcutaneous injection three times per week<br>⇒ A lower dose of 22 µg, given via subcutaneous injection three times per week, will be available for patients who cannot tolerate the 44 µg dose of Rebif®. *Please refer to detailed guidelines in the study protocol.*<br><br>Patients randomized to active ocrelizumab group will also receive dummy placebo of Rebif® (administered via subcutaneous injection three times per week).<br>Patients randomized to active Rebif® group will also receive dummy placebo of ocrelizumab (administered as intravenous infusions at similar time points to those of the ocrelizumab group).<br>Dummy placebos of ocrelizumab and of Rebif® will be similar in appearance and administration as the investigational product. |
| NON-INVESTIGATIONAL MEDICAL PRODUCT(S) | Ocrelizumab/ocrelizumab dummy placebo: Thirty minutes prior to the start of each infusion, patients will receive a methylprednisolone 100 mg i.v. infusion. Pre-infusion treatment with an oral analgesic/antipyretic (e.g.: acetaminophen), and an oral antihistamine (e.g. diphenhydramine) is also recommended.<br>Rebif®/ Rebif® dummy placebo: Pre-treatment with non-steroid anti-inflammatory drugs (ibuprofen) or acetaminophen are recommended; investigators should follow their local label for further information. |
| ASSESSMENTS OF:<br>- EFFICACY | – **Assessment of clinical and protocol defined relapses**<br>▪ Protocol-defined relapse is the occurrence of new or worsening neurological symptoms attributable to MS. Symptoms must persist for >24 hours and should not be attributable to confounding clinical factors (e.g. fever, infection, injury, adverse reactions to medications) and immediately preceded by a stable or improving neurological state for least 30 days. The new or worsening neurological symptoms must be accompanied by objective neurological worsening consistent with an increase of at least half a step on the EDSS scale, or 2 points on one of the appropriate FSS, or 1 point on two or more of the appropriate FSS. The change must affect the selected FSS (i.e., pyramidal, ambulation, |

cerebellar, brainstem, sensory, or visual). Episodic spasms, sexual dysfunction, fatigue, mood change or bladder or bowel urgency or incontinence will not suffice to establish a relapse. NB: Sexual dysfunction and Fatigue will not be scored.

⇨ *Please note: adjudication of protocol-defined relapses will be performed by the Sponsor based on pre-specified criteria, applied to data collected by investigator, in a blinded fashion.*

– **Brain MRI acquisition sequences**
  - T1-weighted MRI (without gadolinium-enhancement)
  - T1-weighted MRI (with gadolinium-enhancement)
  - Fluid-attenuated, inversion recovery (FLAIR), Proton-density–weighted and/or T2-weighted MRI

– **Assessment of sustained disability progression**
  - Disability progression is defined as an increase of ≥ 1.0 point from the baseline EDSS score that is not attributable to another etiology (e.g. fever, concurrent illness, or concomitant medication) when the baseline score is 5.5 or less, and ≥ 0.5 when the baseline score is above 5.5. Disability progression is considered sustained when the increase in the EDSS is confirmed at regularly scheduled visits at least 12 and 24 weeks after the initial documentation of neurological worsening.
  - Sustained disability progression, confirmed for both 12 and 24 weeks after the initial documentation of neurological worsening, will be analyzed.

The following instruments will be used to assess the patient's functional ability: Low Contrast Letter Acuity Charts (LCVA/Sloan charts), Symbol Digit Modalities Test (SDMT), Kurtzke Expanded Disability Status Scale (EDSS), Multiple Sclerosis Functional Composite Scale (MSFCS) and Karnofsky Performance Status Scale (clinician-reported version).

| | |
|---|---|
| - SAFETY | Adverse events, vital signs, weight, physical and neurological examination, clinical laboratory tests, 12 lead ECG, locally reviewed MRI for safety (non-MS CNS pathology), concomitant medications.<br><br>Pregnancy tests [serum/urine beta subunit human chorionic gonadotropin (beta hCG)] will be performed in women of childbearing potential. Plasma and urine samples will be collected for JCV assessments.<br><br>Columbia-Suicide Severity Rating Scale (C-SSRS) will be used for prospective suicidality assessment. |
| - PHARMACOKINETICS/ PHARMACODYNAMICS | Blood samples will be taken for measurement of ocrelizumab serum concentration at the time points detailed in the Schedule of Assessments. Nonlinear mixed effects modeling will be used to analyze the sparse sampling dose-concentration-time data of ocrelizumab in order to assess the pharmacokinetics. Population PK parameters will be estimated and the influence of covariates, such as age, gender, weight, HAHA, and CD19 lymphocyte count, investigated. The relationship between ocrelizumab exposure and selected safety and efficacy parameters will be analyzed and explored in order to characterize the exposure-response curve of ocrelizumab |
| –IMMUNOGENICITY | Serum samples for measurement of HAHA (Human Anti-human Antibodies) to ocrelizumab are collected according to the Schedule of Assessments. |

| | |
|---|---|
| – PATIENT REPORTED OUTCOMES (PRO) | ▪ Modified Fatigue Impact Scale (MFIS Standard)<br>▪ Center for Epidemiology Studies Depression Scale (CES-D3)<br>▪ Medical Outcomes Study 36-Item Short Form Survey Instrument (SF-36: SF-36v2 Standard)<br>▪ EuroQol instrument (EQ-5D-3L-Tablet) |
| – PROTEIN BIOMARKER SAMPLES | Specimens for protein biomarker discovery and validation will be collected from all patients. These specimens will be used for research purposes to identify and/or verify protein biomarkers that are predictive of response to ocrelizumab treatment (in terms of dose, safety and tolerability) and will help understand the pathogenesis, course and outcome of multiple sclerosis and related diseases. In addition, screening technologies for larger numbers of proteins and antibodies may also be used to discover novel antibody associations with MS, disease progression and response to therapy.<br><br>Analyses will include but may not be limited to Complement Factor H (CFH) and the B-cell activating factor (BAFF).<br><br>6 mL sample of whole blood will be collected in a plain tube without EDTA for serum isolation. Blood specimens for protein biomarker discovery and validation will be collected from all patients as per Schedule of Assessments. These specimens will be stored for 5 years after the end of the study and then destroyed. |
| - EXPLORATORY BIOMARKERS (non-DNA) | Roche Clinical Repository (RCR) non-DNA, (dynamic, non-inherited) RNA specimen and plasma for biomarker discovery and validation will be collected only from patients consenting to RCR as per Schedule of Assessments. RCR samples (2 x approximately 2.5 mL of blood collected in PAXgene vacutainers and 6 ml of blood collected in a tube with EDTA for plasma isolation) will be collected to promote, facilitate and improve individualized healthcare by better understanding/predicting ocrelizumab efficacy, dose responses, safety, ocrelizumab mode of action, progression of multiple sclerosis and associated diseases. These specimens may be stored for up to 15 years after the end of the study. |
| - EXPLORATORY BIOMARKERS (DNA) | All patients who have been enrolled in the study will be asked to donate an optional DNA specimen (by written informed consent) for pharmacogenetic and genetic research.<br><br>RCR DNA sampling will involve taking one sample of 6 mL of blood taken as per Schedule of Assessments. The study protocol which includes RCR sampling is submitted to the concerned Ethics Committee and is available for Competent Authority review upon request. These specimens will be stored for up to 15 years after the end of the study. |

PROCEDURES (summary):

**Figure 1: Overview of Study Design**

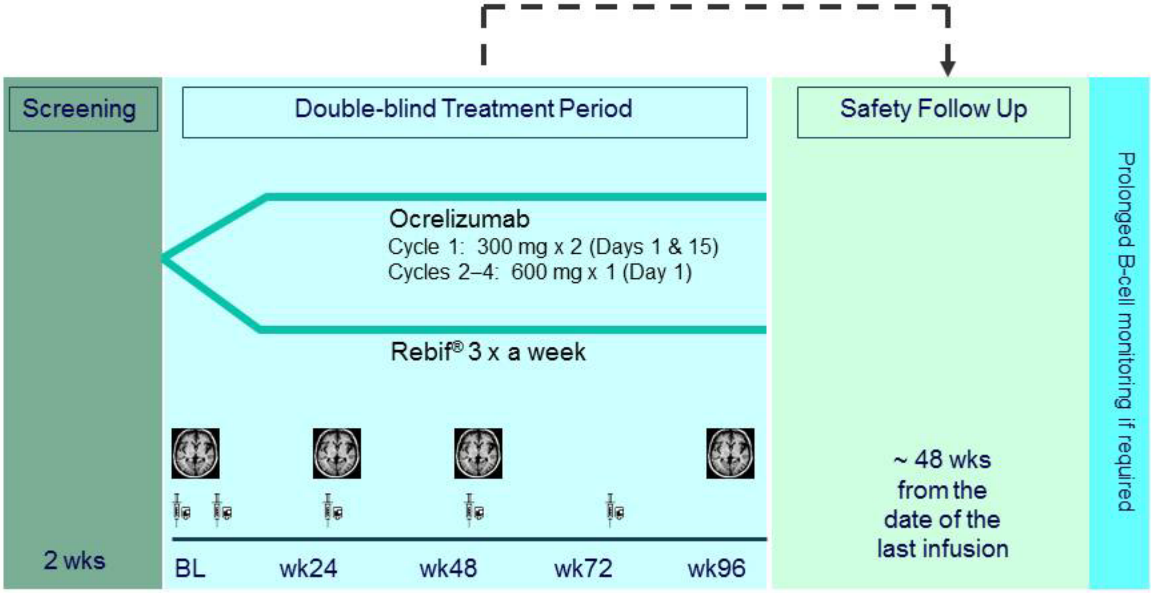


Please note: patients who complete the 96 week treatment period may become eligible for a separate, open-label extension study, under a separate protocol.

## Screening:

Consenting patients will enter the 2-week screening period to be evaluated for eligibility. Procedures at screening will include collecting medical history, medical examination including thorough neurological exam, EDSS score, MSFCS score, ECG, blood and urine sampling. Please see Table 3 - "Schedule of Assessments: Screening Through the End of Double-Blind Treatment Period" for further details.

*Please note that based on local Ethics Committees or National Competent Authority requirements, additional diagnostic testing may be required for selected patients or selected centers to exclude tuberculosis, Lyme disease, HTLV-1 associated myelopathy (HAM), acquired immune deficiency syndrome (AIDS), hereditary disorders, connective tissue disorders, or sarcoidosis.*

## Treatment Period:

Eligible patients will be randomized via IxRS into one of two treatment groups: ocrelizumab 600 mg regimen (group A) or interferon beta-1a (Rebif®) (group B) – please refer to Table 1 and Table 2 for more details.

To prevent potential unblinding as a result of adverse events or changes to laboratory results, the following, additional measures have been implemented:

- **The Examining Investigator/EDSS assessor** will perform the neurological examination, document the FSS scores and assess EDSS scores. The examining investigator will be also responsible for performing and documenting results from: MSFCS, the Karnofsky Performance Status Scale, low contrast visual acuity testing and the Symbol Digit Modalities Test. He or she will have access only to data from assessements listed above.  The examining physician/EDSS assessor will not be involved with any aspect of medical management of the patient and will not have access to patient data. Every effort will be made to ensure that there is no change in the examining physician/EDSS assessor throughout the course of the study for any individual patient. The examining physician/EDSS assessors will be trained and instructed not to discuss what adverse effects (if any) the patient is experiencing from their medication. Examining physician/EDSS assessors will receive training in performing EDSS assessments prior to the beginning of the study and must have successfully passed an examination on performance of the *Neurostatus EDSS examination* within 24 months of participation. All examining physician/EDSS assessors will receive ongoing training on performance of the *Neurostatus EDSS examination* throughout the course of the study.

- **Patient education:** prior to being examined by the Examining Investigator/EDSS assessor, patients will be instructed not to discuss what (if any) adverse effects they may be experiencing. Treating physicians and/or study coordinators should remind patients of these instructions prior to EDSS assessments and this should be documented in the source documents.

- **Blinded, central MRI assessments:** a blinded, central MRI reader will assess all on-study MRI scans. These assessments will provide independent confirmation of the relative changes in immune-mediated, CNS damage.

**Blinding of laboratory parameters:** laboratory parameters which may lead to unblinding to treatment assignment, such as FACS cell counts including $CD19^{+}$ cells, lymphocyte count, IgM and IgG levels and type I interferon neutralizing antibody levels will be blinded in all patients. In order to ensure patients' safety in the study and to allow for assessments of the re-treatment criteria, a central laboratory will provide study investigators and Medical Monitors with reflex messages triggered by critical blinded laboratory results. Investigators notified of their patient's critical laboratory test results will be instructed to suspend further treatment with study drug until the patient becomes eligible for re-treatment. During the treatment period, patients will be assessed at clinical visits as per Schedule of Assessments: Screening Through the End of Treatment Period – please refer to Table 3 for further details.

Prior to the next infusion of study drug, patients will be evaluated for pre-specified conditions and laboratory abnormalities to allow for re-treatment.

**Patients who complete the 96 week treatment period may become eligible for a separate open-label extension study, under a separate protocol.**

**Please note: patients who discontinue from study medication within the 96-week double-blind, comparative phase (treatment period) of the study will enter the Safety Follow up Period (see below); they will not be eligible for the open-label extension study, even if they complete the 96-week treatment period.**

## Safety Follow up Period

Patients who discontinue treatment with study drug will enter the Safety Follow up Period for at least 48 weeks counting from the date of the last infusion of the ocrelizumab/placebo. However, if after this time the peripheral blood B-cells remain depleted, patient should continue to be monitored at 24-week intervals until B-cell count has returned to the baseline value or to the lower limit of the normal range (whichever is the lower). Please refer to Figure 2 for more details.

**Figure 2: Safety Follow up – Prolonged B-cell Monitoring Period**

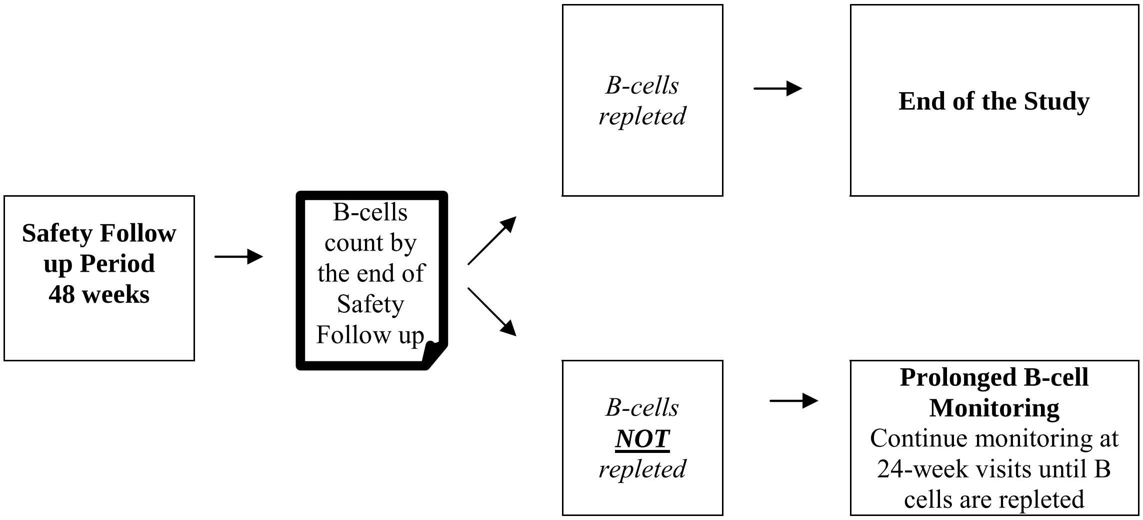


*Please note: patients in Safety Follow up who receive other B-cell targeted therapies will only be followed for 48 weeks from the date of the last infusion of the study drug regardless of their B-cell count.*

During Safety Follow up patients will be assessed at clinical visits every 12 weeks as per Schedule of Assessments. Telephone interviews will be performed every 4 weeks. If prolonged B-cell monitoring is required, patients will be assessed at clinical visits every 24 weeks (as per Schedule of Assessments) and telephone interviews will be performed every 12 weeks. Please refer to Table 4 for further details.

Please note: It is important to distinguish between "withdrawal from treatment" and "withdrawal from study". Patients who withdraw from treatment should be encouraged to remain in the study for the full duration of the Safety Follow Up Period (minimum of 48 weeks following the last infusion).

**Every effort should be made to have patients, who withdraw from the study treatment, complete the Safety Follow up Period and all related assessments, <u>regardless of whether or not they receive alternative treatment for MS.</u>**

## Table 1: Overview of Dosing Regimen

| **Group** | **Treatment Period**[1,2] | | | | |
|---|---|---|---|---|---|
| | **1st Cycle**[3] (Weeks 1-24) | | **2nd Cycle**[3] (Weeks 24-48) | **3rd Cycle**[3] (Weeks 48-72) | **4th Cycle**[3] (Weeks 72-96) |
| | **Day 1 Infusion** | **Day 15 Infusion** | **Week 24 Infusion** | **Week 48 Infusion** | **Week 72 Infusion** |
| **A Ocrelizumab 600 mg regimen** | **Ocrelizumab** 300 mg i.v. | **Ocrelizumab** 300 mg i.v. | **Ocrelizumab** 600 mg i.v. | **Ocrelizumab** 600 mg i.v. | **Ocrelizumab** 600 mg i.v. |
| **B Rebif® regimen**[4] | **Rebif®** s.c. three times per week | → | → | → | → |

1. The treatment period consists of 96 weeks of treatment; patients will receive a maximum of 4 treatment cycles.
2. Each treatment cycle has a duration of 24 weeks. The first cycle consists of two 300 mg ocrelizumab i.v. infusions separated by 14 days. Cycles 2 – 4 consist of a single i.v. infusion of 600 mg ocrelizumab.
3. Prior to each infusion, a clinical evaluation will be performed to ensure that the patient remains eligible for treatment.
4. Please refer to Table 2 for detailed Rebif® dosing regimen.

Please note: 100 mg of methylprednisolone i.v. will be administered in both treatment arms prior to each infusions of ocrelizumab/ocrelizumab placebo.

**Table 2: Overview of Rebif® Dosing Regimen**

| | Treatment Initiation | | Treatment Continuation | Dose modification (if required) |
|---|---|---|---|---|
| **Week** | **Weeks 1- 2** | **Weeks 3-4** | **Week 5 onwards** | – |
| *Study Day* | *1-14* | *15-28* | *29+* | At any time >29 |
| **Dose of Rebif®** | **Rebif® 8.8µg** (one pre-filled syringe [0.2 mL] containing 2.4 MIU of interferon beta-1a) s.c. **3x per week** | **Rebif® 22 µg** (one pre-filled syringe [0.5 mL] containing 6 MIU of interferon beta-1a) s.c. **3x per week** | **Rebif® 44 µg** (one pre-filled syringe [0.5 mL]) containing 12 MIU of interferon beta-1a) s.c. **3x per week** | **Rebif® 22 µg** (one pre-filled syringe [0.5 mL] containing 6 MIU of interferon beta-1a) s.c. **3x per week** |

Please note: if Rebif® dose modification is required due to laboratory abnormalities possibly related to the treatment with Rebif®, the investigator (the treating physician) will need to notify IxRS and the blinded study medication (Rebif® placebo or Rebif® verum) will be dispensed accordingly. In addition, to ensure patient safety in the study, unscheduled visits may be required for additional assessments, monitoring and for dispensing study medication.

**In case of elevation of liver function tests the following rules will apply:**

| | |
|---|---|
| ⇨ **ALT ≥ 10 x ULN**, jaundice or other clinical symptoms of liver dysfunction | In case of detection of elevated **ALT ≥ 10 x ULN**, jaundice or other clinical symptoms of liver dysfunction the injections of Rebif®/Rebif® placebo must be discontinued permanently. The monitoring of liver function tests should be continued on a monthly basis until return to normal baseline levels or CTCAE v.4.0 grade 1 toxicity (ALT: >ULN - 3.0 x ULN). A consultation with hepatologist is recommended. Patients should move to Safety Follow up Period. |

| | |
|---|---|
| ⇨ **ALT ≥ 5 x ULN** | In case of detection of elevated **ALT ≥ 5 x ULN** (but below 10 xULN) the injections of Rebif®/Rebif® placebo must be discontinued temporarily. Additional blood chemistry panel including AST, ALP, GGT and bilirubin should be performed biweekly until no further increase is observed. Subsequently, ALT analysis has to be performed every month until return to normal baseline levels or CTCAE v.4.0 grade 1 toxicity (ALT >ULN - 3.0 x ULN). A consultation with hepatologist should be considered as per investigator judgment.<br>If causes of toxicity other than possible treatment with Rebif® are excluded, the patient may then be cautiously re-challenged with Rebif®/Rebif® placebo 22µg provided in a blinded fashion upon request to IxRS. The monitoring of liver function tests should continue on a monthly basis. If there is no further recurrence of toxicity, patient may continue treatment with Rebif®/Rebif® placebo 44 µg provided in a blinded fashion upon investigator's request to IxRS. **In case of recurrence of toxicity (ALT > 3 x ULN, or other clinical symptoms of liver dysfunction) the injections of Rebif®/Rebif® placebo should be discontinued permanently.** Patients should move to Safety Follow up Period.<br><br>Please note: Re-initiation of therapy with Rebif® following elevation of liver function tests can only be considered once. |
| ⇨ **ALT > 3 x ULN** | In case of detection of elevated **ALT > 3 x ULN** (but below 5x ULN) additional blood chemistry panel including AST, ALP, GGT and bilirubin should be performed biweekly until no further increase is observed. Subsequently, ALT analysis has to be performed every month until return to normal baseline levels or CTCAE v.4.0 grade 1 toxicity (ALT>ULN - 3.0 x ULN). |

## Table 3: Schedule of Assessments: Screening Through the End of Double-Blind Treatment Period

| | **Screen** | **Treatment Period** | | | | | | | | | | | | |
|---|---|---|---|---|---|---|---|---|---|---|---|---|---|---|
| **Visit** | **1** | **2 BL** | **3** | **4** | **5** | **6** | **7** | **8** | **9** | **10** | **11** | **Delayed Dosing Visit[22]** | **Unscheduled Visit[23]** | **Withdrawal from Treatment Visit** |
| **Week** | **-2** | **-** | **w2** | **w12** | **w24** | **w36** | **w48** | **w60** | **w72** | **w84** | **w96** | | | |
| *Study Day (window in days)* | *-14* | *1* | *15 (± 1)* | *85 (±4)* | *169 (±1)* | *253 (±4)* | *337 (±1)* | *421 (±4)* | *505 (±1)* | *589 (±4)* | *673 (±1)* | | | |
| | | | | | | | | | | | | | | |
| Informed consent[1] | x | | | | | | | | | | | | | |
| Medical history | x | | | | | | | | | | | | | |
| Review of eligibility criteria | x | x | | | | | | | | | | | | |
| CES-D, MFIS, EQ-5D, SF-36 | | x | | | | | x | | | | x | | | x |
| Patient's Assessment of Treatment Benefit | | | | | | | x | | | | x | | | x |
| C-SSRS | | x | | x | x | x | x | x | x | x | x | | x | x |
| Physical examination | x | x | x | | x | | x | | x | | x | x | | x |
| Vital signs[2] | x | x | x | x | x | x | x | x | x | x | x | x | x | x |
| 12 lead ECG (pre- and post-dose)[3] | x | x | | | | | | | x | | | | | x |
| Height | x | | | | | | | | | | | | | |
| Weight | x | | | | | | | | x | | x | | | x |
| Neurological exam and EDSS | x | x | | x | x | x | x | x | x | x | x | | x | x |
| MSFCS, LCVA, SDMT | | x | | x | x | x | x | x | x | x | x | | x | x |
| Karnofsky Performance Status Scale | | x | | | x | | x | | x | | x | | | x |
| MRI[4] | | x | | | x | | x | | | | x | | | x |
| Concomitant Treatment | | x | x | x | x | x | x | x | x | x | x | x | x | x |
| Adverse Events | *Only SAEs* | x | x | x | x | x | x | x | x | x | x | x | x | x |
| Potential relapses recorded | | x | x | x | x | x | x | x | x | x | x | x | x | x |
| Telephone interview (every 4 wks)[5] | x | | | → | → | → | → | → | → | → | → | | | x |

## Table 3: Schedule of Assessments: Screening Through the End of Double-Blind Treatment Period (Cont.)

| | Screen | Treatment Period | | | | | | | | | | | | |
|---|---|---|---|---|---|---|---|---|---|---|---|---|---|---|
| **Visit** | 1 | 2 BL | 3 | 4 | 5 | 6 | 7 | 8 | 9 | 10 | 11 | Delayed Dosing Visit [22] | Unscheduled Visit [23] | Withdrawal from Treatment Visit |
| **Week** | -2 | - | w2 | w12 | w24 | w36 | w48 | w60 | w72 | w84 | w96 | | | |
| *Study Day (window in days)* | *-14* | *1* | *15 (± 1)* | *85 (±4)* | *169 (±1)* | *253 (±4)* | *337 (±1)* | *421 (±4)* | *505 (±1)* | *589 (±4)* | *673 (±1)* | | | |
| Pregnancy test [6] | x | x | x | x | x | x | x | x | x | x | x | x | | x |
| Antibody Titers [7] | | x | | x | x | | x | | x | | x | | | x |
| RCR (non-DNA) [8] | | x | | x | x | | x | | x | | x | | | x |
| RCR (DNA) [9] | | x | | | | | | | | | | | | |
| Protein biomarker sampling [10] | | x | | x | x | | x | | x | | x | | | x |
| HAHA [11] | | x | | | x | | x | | x | | x | | | x |
| Plasma/ urine banking for JCV[12] | | x | | x | x | x | x | x | x | x | x | | | x |
| PK Samples [13] | | x | | | x | | x | | x[13] | x | x | | | x |
| Thyroid function tests [14] | x | | | | x | | x | | x | | x | | | x |
| FSH [15] | x | | | | | | | | | | | | | |
| Hepatitis Screening [16] | x | | | | | | | | | | | | | |
| Hepatitis B virus DNA[16] | x | (x) | | (x) | (x) | (x) | (x) | (x) | (x) | (x) | (x) | | | (x) |
| RPR | x | | | | | | | | | | | | | |
| CD4 count | x | | | x | | x | | x | | x | | | | |
| IgG | | | | x | | x | | x | | x | | | | |
| Total Ig, IgA, IgG, IgM | x | | | | x | | x | | x | | x | | | x |
| FACS [17] | | x | x | x | x | | x | | x | | x | | | x |
| Routine safety lab [18] | x | x | x | x | x | x | x | x | x | x | x | | | x |
| Type I interferon neutralizing antibody assay [19] | | x | | | x | | x | | x | | x | | | x |

## Table 3: Schedule of Assessments: Screening Through the End of Double-Blind Treatment Period (Cont.)

| | Screen | Treatment Period | | | | | | | | | | | |
|---|---|---|---|---|---|---|---|---|---|---|---|---|---|
| **Visit** | **1** | **2 BL** | **3** | **4** | **5** | **6** | **7** | **8** | **9** | **10** | **11** | **Delayed Dosing Visit [22]** | **Unscheduled Visit [23]** | **Withdrawal from Treatment Visit** |
| **Week** | **-2** | **-** | **w2** | **w12** | **w24** | **w36** | **w48** | **w60** | **w72** | **w84** | **w96** | | | |
| *Study Day (window in days)* | *-14* | *1* | *15 (± 1)* | *85 (±4)* | *169 (±1)* | *253 (±4)* | *337 (±1)* | *421 (±4)* | *505 (±1)* | *589 (±4)* | *673 (±1)* | | | |
| Pre-treatment with i.v. methylprednisolone [20] | | x | x | | x | | x | | x | | | x | | |
| Administration of i.v. ocrelizumab / ocrelizumab placebo [21] | | x | x | | x | | x | | x | | | x | | |
| Assessment of s.c. Rebif®/ Rebif® placebo compliance | | x | x | x | x | x | x | x | x | x | x | | x | x |
| Administration of s.c. Rebif® / Rebif® placebo 3x/wk | | x | x | x | x | x | x | x | x | x | | | | |

1. **Informed Consent** must be obtained in written form from all patients at screening (prior to any study–related procedure) in order to meet eligibility for the study.
2. **Vital signs** (i.e., pulse rate, systolic and diastolic blood pressure, respiration rate and temperature) will be obtained while the patient is in the semi supine position (after 5 minutes). On infusion visits, the vital signs should be taken within 45 minutes prior to the methylprednisolone infusion in all patients. In addition, vital signs should be obtained prior to ocrelizumab/ocrelizumab placebo infusion, then every 15 minutes (± 5 minutes) for the first hour; then every 30 minutes (± 10 minutes) until 1 hour after the end of the infusion. On non-infusion days, the vital signs may be taken at any time during the visit.
3. **ECG (pre- and post-dose):** on infusion visits ECG should be taken within 45 minutes prior to the methylprednisolone infusion in all patients, and within 60 minutes after completion of ocrelizumab/ocrelizumab placebo infusion. On non-infusion days, the ECG may be taken at any time during the visit.
4. **MRI**: brain MRI scans will be obtained in patients withdrawn from the treatment period (at a withdrawal visit) if not performed during last 4 weeks.
5. **A structured telephone interview** will be conducted by site personnel every 4 weeks (± 3 days) from Week 8 through the study to identify any new or worsening neurological symptoms that warrant an unscheduled visit and collect data on possible events of infections.

6. **Serum β-hCG** must be performed at screening in women of childbearing potential. Subsequently, urine β-hCG [sensitivity of at least 25 mIU/mL] will be performed. On infusion visits, the urine pregnancy test should be performed prior to methylprednisolone infusion in all women of child-bearing potential. If positive, the patient will not receive the scheduled dose and confirmation, a serum pregnancy test, will be performed.
7. **Antibody Titers**: measurement of antibody titers against common antigens (mumps, rubella, varicella and Streptococcus pneumoniae) will be performed.
8. **RCR - Roche Clinical Repository non-DNA (RNA – and protein):** for RNA 2x 2.5 ml whole blood samples to be taken from consenting patients only for expression profiling analysis. For protein: 6 ml blood samples in EDTA tube for plasma samples will be taken from consenting patients only for analysis of protein biomarkers. On infusion visits, ALL samples should be taken 5-30 minutes prior to methylprednisolone infusion.
9. **RCR - Roche Clinical Repository (DNA):** 6 ml whole blood sample to be taken from only from patients consenting to RCR for pharmacogenetic and genetic analysis. If not done at Baseline (Visit 2), sample may be collected at next visit.
10. **Protein biomarker sampling:** one serum sample (6 ml) will be taken from all patients for analysis of protein biomarkers. On infusion visits, samples should be taken 5-30 minutes prior to methylprednisolone infusion.
11. **HAHA**: On infusion visits, serum samples are collected 5-30 minutes prior to the methylprednisolone infusion.
12. **Plasma and urine samples for JCV will be collected** at specified time points and analyzed in batches.
13. **PK samples:** on the infusion day at week 72,, two serum samples should be collected, one 5-30 minutes prior to the methylprednisolone infusion and the second one 30 minutes (±10 minutes) following the completion of the ocrelizumab/ocrelizumab placebo infusion. For all other infusion visits, a blood sample should be taken 5 – 30 minutes before the methylprednisolone infusion. At other times (non-infusion visits) samples may be taken at any time during the visit.
14. **sTSH** will be tested at screening and during double-blind treatment period. Thyroid autoantibodies will be assayed only at screening.
15. **FSH**: <u>only applicable to women</u> to confirm the post-menopausal status.
16. **Hepatitis** screening & monitoring: all patients must have negative HBsAg result and negative HepCAb screening tests prior to enrollment. If total HBcAb is positive at screening, HB virus DNA measured by PCR must be negative to be eligible. For those patients enrolled with negative HBsAg and positive total HBcAb, HB virus DNA (PCR) must be repeated every 12 weeks during the treatment period.
17. **FACS**: including CD19 and other circulating B-cell subsets, T-cells, natural killer cells and other leukocytes. On infusion visits, blood samples should be collected prior to the infusion of methylprednisolone.
18. **Routine safety lab:** hematology, chemistry and urinalysis: on infusion visits, all urine and blood samples should be collected prior to the infusion of methylprednisolone. At other times, samples may be taken at any time during the visit.
19. **Type I interferon neutralizing antibody assay:** samples should be taken at least 36 hours following last injection of Rebif®/Rebif® placebo.
20. All patients receive **prophylactic treatment** with 100 mg of methylprednisolone i.v. prior to infusion of ocrelizumab /ocrelizumab placebo. It is also recommended that patients receive an analgesic/antipyretic such as acetaminophen/paracetamol (1 g) and an i.v. or oral antihistamine such as diphenhydramine 50 mg 30-60 minutes prior to ocrelizumab/ ocrelizumab placebo.

21. **Administration (infusion) of i.v. ocrelizumab/ocrelizumab placebo**: the Treating Investigator must review the clinical and laboratory re-treatment criteria prior to subsequent infusion of ocrelizumab/ocrelizumab placebo.
22. **A delayed dosing visit** will be performed and recorded in the Delayed Dosing Visit eCRF form when dosing cannot be administered at the scheduled dosing visit. Other tests or assessments may be done as appropriate.
23. **Unscheduled Visit**: assessments performed at unscheduled (non-dosing) visits will depend on the clinical needs of the patient. All patients with new neurological symptoms suggestive of relapse should have EDSS performed by examining investigator. Other tests/assessments may be done as appropriate. Please note: in case of ALT elevations dose modification should be necessary, additional visits may be required for dispensing of study medication.

Please note: based on local Ethics Committees or National Competent Authority requirements, additional diagnostic testing may be required for selected patients or selected centers to exclude tuberculosis, Lyme disease, HTLV-1 associated myelopathy (HAM), acquired immune deficiency syndrome (AIDS), hereditary disorders, connective tissue disorders, or sarcoidosis. Other specific diagnostic tests may be requested when deemed necessary by the investigator**.**

## Table 4: Schedule of Assessments: Safety Follow up (including prolonged B-cell monitoring if required)

| | **Safety Follow up** | ***Prolonged B-cell Monitoring*[1]** | ***End of observation or withdrawal from Safety Follow up*** |
|---|---|---|---|
| **Assessments** | *Visits every 12 weeks (±7 days)* [2] | *Visits every 24 weeks (±7 days)* | |
| Urine pregnancy test | x | x | x |
| Routine Safety Labs[3] | x | x | x |
| FACS [4] | x | x | x |
| Total Ig, IgA, IgG, IgM | x[10] | x | x |
| HAHA[5] | x[10] | x | x |
| Plasma/urine banking for JCV | x | x | x |
| Antibody titers | x[10] | x | x |
| Hepatitis B viral DNA[6] | (x) | (x) | (x) |
| RCR non-DNA [7] | x[10] | x | x |
| Protein biomarker sampling [8] | x[10] | x | x |
| Vital Signs | x | x | x |
| EDSS | x | | x |
| Neurological examination | x | x | x |
| Physical examination | x[10] | x | x |
| Potential relapses recorded | x | x | x |
| Adverse events | x | x | x |
| Concomitant Medication | x | x | x |
| Telephone interview [9] | x | x | |

1. **Prolonged B-cell monitoring:** patients whose B-cells have not been repleted after 48 weeks of Safety Follow up period will continue with visits every 24 weeks (± 7 days) until B-cell repletion.
2. Visits will be performed at 12-week intervals counting from the date of last infusion of ocrelizumab.
3. **Routine safety lab**: hematology, chemistry and urinalysis.
4. **FACS** including CD19 and other circulating B-cell subsets, T cells, natural killer cells and other leukocytes.
5. **HAHA**: two serum samples are required.
6. **Hepatitis monitoring:** hepatitis to be monitored only in patients with screening results of HbsAg negative, HBcAb positive and HBV DNA negative, inclusive.
7. **RCR (Roche Clinical Repository) non-DNA (RNA and protein):** for RNA 2x 2.5 ml whole blood samples to be taken from consenting patients only for expression profiling analysis. For protein 6 ml blood samples in EDTA tube for plasma samples to be taken from consenting patients only for analysis of protein biomarkers.
8. **Protein biomarker sampling**: 6 ml blood sample in a plain tube without EDTA for serum isolation will be taken from all patients for analysis of protein biomarkers.
9. **A structured telephone interview** will be performed by site personnel every 4 weeks (± 3 days) between visits until 48 weeks after the last infusion to identify any new or worsening neurological symptoms that warrant an unscheduled visit and collect data on possible events of infections. If prolonged B-cell monitoring is required beyond 48 weeks after the last infusion, telephone interviews will be done every 12 weeks (± 7 days) between visits.
10. Needs to be assessed only every 24 weeks.

<u>Please note:</u> patients in Safety Follow up who receive other B-cell targeted therapies will only be followed for 48 weeks from the date of the last infusion of the study drug regardless of their B-cell count.

## SAMPLE SIZE AND STATISTICAL ANALYSES

The sample size for this study has been estimated based on data from previous RRMS trials, with the use of two-sided tests with an experiment-wise alpha of 0.05. The annualized rate of relapse among patients receiving ocrelizumab at 96 weeks is predicted to be 0.165 (standard deviation of approximately 0.60), as compared with 0.33 (standard deviation of approximately 0.80) among patients receiving the control treatment, Rebif® (this represents a relative reduction of 50% on ocrelizumab compared to the active comparator). For the annualized relapse rate, a t-test has been used to determine the sample size between ocrelizumab and the control arm. The sample size of 400 patients per arm provides 84 percent power, maintaining the type I error rate of 0.05, and assuming a drop out rate of 20 percent approximately (assuming relative reduction among patients drop out is 25%). For sustained disease progression, a two group test of equal exponential survival with exponential dropout is used to determine the sample size. Assuming the 2 year sustained disability progression rate is 18% for the Rebif® arm and 12.6% for the ocrelizumab arm (this represents a relative reduction of 30% on ocrelizumab compared to the active comparator), and assuming a drop out rate of 20 percent over 2 years approximately, the sample size of 400 per arm will provide 80 percent power, maintaining the type I error rate of 0.05 based on the pooled analysis of two identical RMS trials (800 patients treated with ocrelizumab 600 mg and 800 patients treated with Rebif®).

All eligible patients will be randomized to treatment (with 1:1 ratio to ocrelizumab 600 mg regimen or the control arm) stratified by region (US versus ROW) and baseline EDSS (< 4.0 versus ≥ 4.0).

The primary efficacy endpoint is the annualized relapse rate by 96 weeks. The annualized relapse rates at 96 weeks will be calculated using negative binomial model, adjusting for region (United States versus ROW) and baseline EDSS (< 4.0 versus ≥ 4.0). The adjusted annualized relapse rates and the 95% confidence intervals for the relapse rates will be presented along with the p-value.

Summaries of safety data will be produced using data from all patients who have received any study treatment and provided at least one assessment of safety.

## Table of Contents

1. Background and Rationale . . . . . 39
1.1 Background . . . . . 39
1.1.1 Multiple Sclerosis . . . . . 39
1.1.2 Ocrelizumab . . . . . 40
1.1.3 Rationale for Targeting B-cells in MS . . . . . 41
1.1.4 Sponsor Experience with Anti-CD20 Compounds in MS . . . . . 42
1.1.4.1 Ocrelizumab in RRMS . . . . . 42
1.1.4.2 Rituximab in RRMS . . . . . 44
1.1.4.3 Rituximab in PPMS . . . . . 45
1.1.5 Rebif® . . . . . 46
1.2 Rationale for the Study . . . . . 48
2. Objectives . . . . . 49
2.1 Primary Objective . . . . . 49
2.2 Secondary Objectives . . . . . 49
2.3 Exploratory Objectives . . . . . 50
2.4 Roche Clinical Repository (RCR) Exploratory Objectives . . . . . 50
3. Study Design . . . . . 51
3.1 Overview of Study Design and Dosing Regimen . . . . . 51
3.1.1 Rationale for Study Design . . . . . 54
3.1.2 Rationale for Dose Selection . . . . . 57
3.1.3 End of Study . . . . . 58
3.2 Number of Subjects / Assignment to Treatment Groups . . . . . 58
3.3 Centers . . . . . 58
4. Study Population . . . . . 58
4.1 Overview . . . . . 58
4.1.1 Recruitment Procedures . . . . . 58
4.2 Inclusion Criteria . . . . . 59
4.3 Exclusion Criteria . . . . . 59
4.4 Concomitant Medication and Treatment . . . . . 62
4.4.1 Definition of Concomitant Treatment . . . . . 62
4.4.2 Treatment for Symptoms of MS . . . . . 62

4.4.2.1 Prohibited Concomitant Treatments . . . . . . . . 63
4.4.3 Immunization . . . . . . . . 63
4.5 Criteria for Premature Withdrawal . . . . . . . . 64
4.5.1 Withdrawal of Subjects from the Roche Clinical Repository (RCR) . 65
4.5.2 Patient Agreement for Continuation in the Study (in case of sustained disability progression) . . . . . . . . 66
4.6 Replacement Policy (Ensuring Adequate Numbers of Evaluable Subjects) . . . . . . . . 66
4.6.1 For Subjects . . . . . . . . 66
4.6.2 For Centers . . . . . . . . 66

5. Schedule of Assessment and Procedures . . . . . . . . 67

5.1 Screening Examination and Eligibility Screening Form . . . . . . . . 74
5.2 Procedures for Enrollment of Eligible Subjects . . . . . . . . 74
5.3 Clinical Assessments and Procedures . . . . . . . . 75
5.3.1 Overview of Clinical Visits . . . . . . . . 75
5.3.1.1 Delayed Dosing Visit . . . . . . . . 76
5.3.1.2 Unscheduled Visits . . . . . . . . 76
5.3.1.3 Withdrawal Visits . . . . . . . . 76
5.3.2 Assessment of Efficacy . . . . . . . . 77
5.3.2.1 Assessment of Relapse . . . . . . . . 77
5.3.2.2 Assessment of Disability . . . . . . . . 77
5.3.2.3 Kurtzke Expanded Disability Status Scale (EDSS) . . . . . . . . 77
5.3.2.4 The Multiple Sclerosis Functional Composite Scale (MSFCS) . . . 78
5.3.2.5 Low-Contrast Visual Acuity (LCVA) Testing . . . . . . . . 78
5.3.2.6 The Symbol Digit Modalities Test (SDMT) . . . . . . . . 78
5.3.3 Brain MRI Imaging . . . . . . . . 78
5.3.4 Safety . . . . . . . . 79
5.3.4.1 Electrocardiogram (ECG) . . . . . . . . 80
5.3.4.2 Physical Examination . . . . . . . . 80
5.3.4.3 Neurological Examination . . . . . . . . 80
5.3.4.4 Telephone Interviews . . . . . . . . 80
5.3.4.5 Columbia-Suicide Severity Rating Scale C-SSRS . . . . . . . . 81
5.3.5 The Karnofsky Performance Scale (clinician-reported version) . . . . 81
5.4 Laboratory Assessments . . . . . . . . 81

5.4.1 Standard Laboratory Assessments . . . . . . . . . . . . . . . . . . . . . . . . . . . 82
5.4.2 Hepatitis Screening and Liver Function Monitoring . . . . . . . . . . . . . 83
5.4.3 Plasma and Urine Banking for JC Virus . . . . . . . . . . . . . . . . . . . . . . 84
5.4.4 Pharmacokinetic (PK)/Pharmacodynamic (PD) Assessments . . . . . . 84
5.4.5 Type I Interferon Neutralizing Antibody Assay . . . . . . . . . . . . . . . . . 84
5.5 Roche Clinical Repository Specimen(s) . . . . . . . . . . . . . . . . . . . . . . . 84
5.5.1 Specimen Types . . . . . . . . . . . . . . . . . . . . . . . . . . . . . . . . . . . . . . 85
5.6 Protein Biomarker Samples . . . . . . . . . . . . . . . . . . . . . . . . . . . . . . . . 85
5.7 Patient Reported Outcome(s) . . . . . . . . . . . . . . . . . . . . . . . . . . . . . . . 86
5.7.1 Modified Fatigue Impact Scale (MFIS) . . . . . . . . . . . . . . . . . . . . . . 86
5.7.2 The Center for Epidemiologic Studies Depression Scale (CES-D) . . 86
5.7.3 The Short Form (SF-36) Health Survey . . . . . . . . . . . . . . . . . . . . . . 87
5.7.4 Patient's Assessment of Treatment Benefit . . . . . . . . . . . . . . . . . . . . 87
5.8 Pharmacoeconomic Assessments/ EQ-5D . . . . . . . . . . . . . . . . . . . . . . 87
5.9 Post Study Provisional Care . . . . . . . . . . . . . . . . . . . . . . . . . . . . . . . 87

6. Investigational Medicinal Product . . . . . . . . . . . . . . . . . . . . . . . . . . . . 88

6.1 Ocrelizumab . . . . . . . . . . . . . . . . . . . . . . . . . . . . . . . . . . . . . . . . . . 88
6.1.1 Preparation and Administration of Ocrelizumab Infusions . . . . . . . . 88
6.1.2 Prevention and Treatment of Infusion Related Reactions . . . . . . . . . 90
6.1.3 Ocrelizumab Dose Modifications, Interruptions and Delays . . . . . . . 91
6.1.4 Criteria for Re-Treatment with Ocrelizumab . . . . . . . . . . . . . . . . . . 92
6.2 Rebif® . . . . . . . . . . . . . . . . . . . . . . . . . . . . . . . . . . . . . . . . . . . . . . 92
6.2.1 Dose and Schedule of Rebif® . . . . . . . . . . . . . . . . . . . . . . . . . . . . . 92
6.2.2 Rebif® Dose Modifications, Interruptions and Delays . . . . . . . . . . . 93
6.3 Formulation, Packaging and Labeling . . . . . . . . . . . . . . . . . . . . . . . . . 94
6.3.1 Ocrelizumab . . . . . . . . . . . . . . . . . . . . . . . . . . . . . . . . . . . . . . . . . 95
6.3.2 Rebif® . . . . . . . . . . . . . . . . . . . . . . . . . . . . . . . . . . . . . . . . . . . . . 96
6.4 Blinding and Unblinding . . . . . . . . . . . . . . . . . . . . . . . . . . . . . . . . . . . 96
6.5 Accountability of IMP and Assessment of Compliance . . . . . . . . . . . . 97
6.5.1 Accountability of IMP . . . . . . . . . . . . . . . . . . . . . . . . . . . . . . . . . . 97
6.5.2 Assessment of Compliance . . . . . . . . . . . . . . . . . . . . . . . . . . . . . . . 97
6.6 Destruction of the IMP/Comparator . . . . . . . . . . . . . . . . . . . . . . . . . . 97

7. Safety Instructions and Guidance . . . . . . . . . . . . . . . . . . . . . . . . . . . . . 98

7.1 Adverse Events (AEs) and Laboratory Abnormalities . . . . . . . . . . . . . 98
7.1.1 Clinical AEs . . . . . . . . . . . . . . . . . . . . . . . . . . . . . . . . . . . . . . . . 98
7.1.1.1 Intensity . . . . . . . . . . . . . . . . . . . . . . . . . . . . . . . . . . . . . . . . . 99
7.1.1.2 Drug - Adverse Event Relationship . . . . . . . . . . . . . . . . . . . . . . . . 99
7.1.1.3 Serious Adverse Events (Immediately Reportable to Sponsor) . . . 99
7.1.2 Treatment and Follow-up of AEs . . . . . . . . . . . . . . . . . . . . . . . . . . 100
7.1.3 Laboratory Test Abnormalities . . . . . . . . . . . . . . . . . . . . . . . . . . . 100
7.1.3.1 Follow-up of Abnormal Laboratory Test Values . . . . . . . . . . . . . . 100
7.2 Handling of Safety Parameters . . . . . . . . . . . . . . . . . . . . . . . . . . . 101
7.2.1 Reporting of AEs . . . . . . . . . . . . . . . . . . . . . . . . . . . . . . . . . . . . 101
7.2.2 Reporting of Serious Adverse Events . . . . . . . . . . . . . . . . . . . . . . . 101
7.2.2.1 Immediate Reporting to the Sponsor . . . . . . . . . . . . . . . . . . . . . . 101
7.2.2.2 Expedited Reporting to Health Authorities, Investigators, Institutional Review Boards, and Ethics Committees . . . . . . . . . . . . . . 102
7.2.3 Pregnancy and Lactation . . . . . . . . . . . . . . . . . . . . . . . . . . . . . . . 103
7.3 Warnings and Precautions . . . . . . . . . . . . . . . . . . . . . . . . . . . . . . 104
7.3.1 Ocrelizumab . . . . . . . . . . . . . . . . . . . . . . . . . . . . . . . . . . . . . . . 104
7.3.2 Rebif® . . . . . . . . . . . . . . . . . . . . . . . . . . . . . . . . . . . . . . . . . . . 106
7.3.3 Corticosteroids . . . . . . . . . . . . . . . . . . . . . . . . . . . . . . . . . . . . . 107
7.3.4 Progressive Multifocal Leukoencephalopathy . . . . . . . . . . . . . . . . . 107
7.3.4.1 Guidance for Diagnosis of PML . . . . . . . . . . . . . . . . . . . . . . . . . 108

8. Statistical Considerations and Analytical Plan . . . . . . . . . . . . . . . . . . . 112

8.1 Study Endpoints . . . . . . . . . . . . . . . . . . . . . . . . . . . . . . . . . . . . . . 112
8.1.1 Primary Efficacy Endpoint . . . . . . . . . . . . . . . . . . . . . . . . . . . . . . 112
8.1.2 Secondary Efficacy Endpoints . . . . . . . . . . . . . . . . . . . . . . . . . . . . 112
8.1.3 Exploratory Efficacy Endpoints . . . . . . . . . . . . . . . . . . . . . . . . . . . 112
8.1.4 Safety . . . . . . . . . . . . . . . . . . . . . . . . . . . . . . . . . . . . . . . . . . . . 113
8.2 Statistical and Analytical Methods . . . . . . . . . . . . . . . . . . . . . . . . . . 113
8.2.1 Primary Efficacy Analysis . . . . . . . . . . . . . . . . . . . . . . . . . . . . . . . 114
8.2.2 Secondary Efficacy Analyses . . . . . . . . . . . . . . . . . . . . . . . . . . . . . 114
8.2.2.1 The Time to Onset of Sustained Disability Progression for At Least 12 Weeks During the 96-Week Comparative Treatment Period . 115
8.2.2.2 The Time to Onset of Sustained Disability Progression for At Least 24 Weeks During the 96-Week Comparative Treatment Period . 116

8.2.2.3 The Proportion of Relapse-Free Patients by 96 Weeks . . . . . . . . . . 116
8.2.2.4 The Change in Total T2 Lesion Volume as Detected by Brain MRI from Baseline to Week 96 . . . . . . . . . . . . . . . . . . . . . . . . . . . . . 116
8.2.2.5 The Total Number of New, and/or Enlarging T2 Hyperintense Lesions as Detected by Brain MRI at Week 24, Week 48 and Week 96 . . . . . . . . . . . . . . . . . . . . . . . . . . . . . . . . . . . . . . . . . . . . 117
8.2.2.6 The Change in Multiple Sclerosis Functional Composite Scale (MSFCS) Score from Baseline to Weeks 96 . . . . . . . . . . . . . . . . . . . . . 117
8.2.2.7 The Change in Brain Volume as Detected by Brain MRI Scan from Week 24 to Week 96 . . . . . . . . . . . . . . . . . . . . . . . . . . . . . . . . . 117
8.2.3 Exploratory Analyses . . . . . . . . . . . . . . . . . . . . . . . . . . . . . . . . . . 117
8.2.4 Sample Size . . . . . . . . . . . . . . . . . . . . . . . . . . . . . . . . . . . . . . . . . 117
8.2.5 Hypothesis Testing . . . . . . . . . . . . . . . . . . . . . . . . . . . . . . . . . . . . 118
8.2.6 Analysis Populations . . . . . . . . . . . . . . . . . . . . . . . . . . . . . . . . . . 118
8.2.6.1 Safety Population . . . . . . . . . . . . . . . . . . . . . . . . . . . . . . . . . . . . 118
8.2.6.2 Intent-to-Treat Population . . . . . . . . . . . . . . . . . . . . . . . . . . . . . . 118
8.2.6.3 Per Protocol Population . . . . . . . . . . . . . . . . . . . . . . . . . . . . . . . . 119
8.2.7 Interim Analysis . . . . . . . . . . . . . . . . . . . . . . . . . . . . . . . . . . . . . . 119
8.2.8 Safety Data Analysis . . . . . . . . . . . . . . . . . . . . . . . . . . . . . . . . . . . 119
8.2.9 Safety Follow-up Period . . . . . . . . . . . . . . . . . . . . . . . . . . . . . . . . . 120
8.2.10 Other Analyses . . . . . . . . . . . . . . . . . . . . . . . . . . . . . . . . . . . . . . . 120
8.2.10.1 Pharmacokinetic Analysis . . . . . . . . . . . . . . . . . . . . . . . . . . . . . . 120
8.2.10.2 Pharmacodynamic Analysis . . . . . . . . . . . . . . . . . . . . . . . . . . . . . 120
8.2.10.3 Roche Clinical Repository / Protein Biomarker Samples . . . . . . . 121

9. Data Collection, Management and Quality Assurance . . . . . . . . . . . . . . . . . . 121
9.1 Assignment of Preferred Terms and Original Terminology . . . . . . . . . 121

10. Study Committees . . . . . . . . . . . . . . . . . . . . . . . . . . . . . . . . . . . . . . . . . . 121

11. References . . . . . . . . . . . . . . . . . . . . . . . . . . . . . . . . . . . . . . . . . . . . . . 123

12. Ethical Aspects . . . . . . . . . . . . . . . . . . . . . . . . . . . . . . . . . . . . . . . . . . . . 128
12.1 Local Regulations/Declaration of Helsinki . . . . . . . . . . . . . . . . . . . . 128
12.2 Informed Consent . . . . . . . . . . . . . . . . . . . . . . . . . . . . . . . . . . . . . . . 128
12.2.1 Main Study Informed Consent . . . . . . . . . . . . . . . . . . . . . . . . . . . . 128
12.2.2 RCR Informed Consent . . . . . . . . . . . . . . . . . . . . . . . . . . . . . . . . . 129

12.2.3 Death or Loss of Competence of Participant who has donated a specimen(s) that is stored in the RCR . . . . . . . . . . . . . . . . . . . . . . . . . 129
12.3 Independent Ethics Committees (IEC) and Institutional Review Board (IRB) . . . . . . . . . . . . . . . . . . . . . . . . . . . . . . . . . . . . . . . . . . 130
12.4 Role of the Science and Ethics Advisory Group (SEAG) . . . . . . . . . . 131

13. Conditions for Modifying the Protocol. . . . . . . . . . . . . . . . . . . . . . . . . . 131

14. Conditions for Terminating the Study . . . . . . . . . . . . . . . . . . . . . . . . . . 131

15. Study Documentation, CRFs and Record Keeping . . . . . . . . . . . . . . . . . . . 131
15.1 Investigator's Files / Retention of Documents . . . . . . . . . . . . . . . . . . . 131
15.2 Source Documents and Background Data . . . . . . . . . . . . . . . . . . . . . 132
15.3 Audits and Inspections. . . . . . . . . . . . . . . . . . . . . . . . . . . . . . . . . . . 132
15.4 Electronic Case Report Forms . . . . . . . . . . . . . . . . . . . . . . . . . . . . . 132
15.5 Financial Disclosure . . . . . . . . . . . . . . . . . . . . . . . . . . . . . . . . . . . 133

16. Monitoring the Study . . . . . . . . . . . . . . . . . . . . . . . . . . . . . . . . . . . . . 133

17. Confidentiality of Trial Documents and Subject Records . . . . . . . . . . . . . 133

18. Clinical Study Report (CSR) . . . . . . . . . . . . . . . . . . . . . . . . . . . . . . . . 134

19. Publication of Data and Protection of Trade Secrets . . . . . . . . . . . . . . . . . 134

## List of Tables


Table 1: Summary of the Most Frequent Rebif® Adverse Reactions by MedDRA System Organ Class . . . . . . . . . . . . . . . . . . . . . . . . . . 48

Table 2: Overview of Dosing Regimen . . . . . . . . . . . . . . . . . . . . . . . . . . . . . . . 53

Table 3: Schedule of Assessments: Screening Through the End of Double-Blind Treatment Period . . . . . . . . . . . . . . . . . . . . . . . . . 67

Table 4: Schedule of Assessments: Safety Follow up (including prolonged B-cell monitoring if required). . . . . . . . . . . . . . . . . . . . . . . . . . 72

Table 5: Treatment Groups and Schedule of Study Medication. . . . . . . . . . . . . 88

Table 6: Infusions of Ocrelizumab 300 mg . . . . . . . . . . . . . . . . . . . . . . . . . . . 89

Table 7: Subsequent Infusions of Ocrelizumab 600 mg. . . . . . . . . . . . . . . . . . . 90

Table 8: Overview of Rebif® Dosing Regimen * . . . . . . . . . . . . . . . . . . . . . . . 93

Table 9: Clinical features to distinguish between MS relapse and PML* . . . . . 110

Table 10: MRI Lesion Characteristics Typical of PML and MS . . . . . . . . . . . . 111

## List of Figures

Figure 1: Overview of Study Design . . . . . . . . . . . . . . . . . . . . . . . . . . . . . . . . . . 51

Figure 2: Safety Follow up - Variable B-cell Monitoring. . . . . . . . . . . . . . . . . . . 52

Figure 3: Diagnostic Algorithm for PML . . . . . . . . . . . . . . . . . . . . . . . . . . . . . . 110

Figure 4: Hierarchal Order of Key Efficacy Endpoints . . . . . . . . . . . . . . . . . . . 115

## List of Appendices

Appendix 1: AEs Categories for Determining Relationship to Test Drug . . . . . 135

Appendix 2: ICH Guidelines for Clinical Safety Data Management, Definitions and Standards for Expedited Reporting, Topic E2 . 136

Appendix 3: Common Terminology Criteria for Adverse Events (CTCAE) . . . 138

Appendix 4: Telephone Interviews . . . . . . . . . . . . . . . . . . . . . . . . . . . . . . . . . . 139

Appendix 5: Modified Fatigue Impact Scale (MFIS) . . . . . . . . . . . . . . . . . . . . . 141

Appendix 6: The Center for Epidemiologic Studies Depression Scale (CES-D) . . . . . . . . . . . . . . . . . . . . . . . . . . . . . . . . . . . . . . . . . . . 144

Appendix 7: The Short Form (SF-36) Health Survey . . . . . . . . . . . . . . . . . . . . 146

# GLOSSARY OF ABBREVIATIONS

| | |
|---|---|
| ADCC | Antibody dependent cellular cytotoxicity |
| AE | Adverse Event |
| AIDS | Acquired Immune Deficiency Syndrome |
| ALT (SGPT) | Alanine aminotransferase |
| ALP | Alkaline Phosphatase |
| ARR | Annualized Relapse Rate |
| AST (SGOT) | Aspartate aminotransferase |
| AUC | Area Under Curve |
| BAFF | B-cell activating factor |
| BCG | Bacille Calmette-Guérin – TB vaccine<br>(Fr. Bacille billié de Calmette-Guérin) |
| β hCG | Beta human Chorionic Gonadotropin |
| CD | Cluster of Differentiation |
| CDC | Complement-dependent cytotoxicity |
| CES-D | Center for Epidemiologic Studies Depression Scale |
| CFH | Complement Factor H |
| CSF | Cerebrospinal Fluid |
| C-SSRS | Columbia - Suicide Severity Rating Scale |
| CTCAE | Common Terminology Criteria for Adverse Events |
| DMARD | Disease-modifying anti-rheumatic drugs |
| DMC | Data Monitoring Committee |
| DNA | Deoxyribonucleic Acid |
| DAP | Data Analysis Plan |
| EBV | Epstein-Barr Virus |
| ECG | Electrocardiogram |
| eCRF | Electronic Case Report Form(s) |
| EDC | Electronic Data Capture |
| EDSS | Expanded Disability Status Scale |

## GLOSSARY OF ABBREVIATIONS

| | |
|---|---|
| eform | Electronic form |
| ELISA | Enzyme-Linked Immunosorbent Assay |
| ESF | Eligibility Screening Form |
| EQ-5D | EuroQoL |
| FDA | Food and Drug Administration |
| FLAIR | Fluid-attenuated Inversion Recovery |
| FSH | Follicle Stimulating Hormone |
| FSS | Functional Systems Scores |
| GGT | Gamma Glutamyl Transferase |
| HAHA | Human Anti-human Antibodies |
| HAM | Human T-lymphotropic virus (HTLV)-1 Associated Myelopathy |
| HBsAg | Hepatitis B Surface Antigen |
| HBcAb | Hepatitis B Core Antibody |
| HepCAb | Hepatitis C Antibody |
| HDHF | High Dose High Frequency |
| HIV | Human Immunodeficiency Virus |
| HTLV | Human T-lymphotropic Virus |
| IB | Investigator Brochure |
| ICH | International Conference on Harmonisation |
| ICMJE | International Committee of Medical Journal Editors |
| IFN | Interferon |
| Ig | Immunoglobulin |
| i.m. | Intramuscular |
| IMP | Investigational Medicinal Product |
| IND | Investigational New Drug |
| INN | International Non-proprietary Name |
| IRB/IEC | Institutional Review Board/Independent Ethics Committee |

## GLOSSARY OF ABBREVIATIONS

| | |
|---|---|
| IRR | Infusion Related Reaction |
| ITT | Intent-To-Treat |
| i.v. | Intravenous |
| IxRS | Interactive Voice and Web Response System |
| JCV | JC Virus |
| KLH | Keyhole Limpet Haemocyanin |
| LCVA | Low Contrast Visual Acuity |
| LLN | Lower Limit of Normal |
| MBP | Myelin Basic Protein |
| MedDRA | Medical Dictionary for Regulatory Activities |
| MFIS | Modified Fatigue Impact Scale |
| MOG | Myelin Oligodendrocyte Glycoprotein |
| MRI | Magnetic Resonance Imaging |
| MS | Multiple Sclerosis |
| MSFCS | Multiple Sclerosis Functional Composite Scale |
| MTX | Methotrexate |
| NAb | Neutralizing Antibody |
| NHL | Non Hodgkin Lymphoma |
| NK | Natural killer |
| NYHA | New York Heart Association |
| OCB | Oligoclonal Band |
| OCR | Ocrelizumab |
| OCT | Optical Coherence Tomography |
| PASAT | Paced Auditory Serial Addition Test |
| PCR | Polymerase Chain Reaction |
| PD | Pharmacodynamics |
| PK | Pharmacokinetics |
| PML | Progressive Multifocal Leukoencephalopathy |

## GLOSSARY OF ABBREVIATIONS

| | |
|---|---|
| PP | Per protocol (population) |
| PPMS | Primary Progressive Multiple Sclerosis |
| PRMS | Progressive Relapsing Multiple Sclerosis |
| PRO | Patient-Reported Outcome |
| RA | Rheumatoid Arthritis |
| RBC | Red Blood Cells |
| RCR | Roche Clinical Repository |
| RMS | Relapsing Multiple Sclerosis |
| RNA | Ribonucleic Acid |
| ROW | Rest of the World |
| RMS | Relapsing Multiple Sclerosis |
| RNFL | Retinal Nerve Fiber Layer |
| RPR | Rapid Plasma Reagin |
| RRMS | Relapsing-Remitting Multiple Sclerosis |
| SAE | Serious Adverse Event |
| s.c. | Subcutaneous |
| SDMT | The Symbol Digit Modalities Test |
| SEAG | Science and Ethics Advisory Group |
| SF-36 | SF-36 Health Survey |
| SMT | Study Management Team |
| SPMS | Secondary Progressive Multiple Sclerosis |
| TNF | Tumor Necrosis Factor |
| sTSH | sensitive Thyroid Stimulating Hormone |
| TB | Tuberculosis |
| $T_{CTL}$ | Cytotoxic Lymphocyte T |
| ULN | Upper Limit of Normal |
| WBC | White Blood Cells |

# PART I: STUDY DESIGN AND CONDUCT

## 1. BACKGROUND AND RATIONALE

### 1.1 Background

#### 1.1.1 Multiple Sclerosis

Multiple sclerosis (MS) is an inflammatory and degenerative demyelinating disease of the human central nervous system (CNS). Multiple sclerosis affects around 2.5 million people worldwide: it is one of the most common neurological disorders and causes of disability of young adults, especially in Europe and North America [1]. The condition manifests as neurological deficits referable to damage to the spinal cord, brainstem, optic nerves, cerebellum, and cerebrum. Resulting symptoms may include weakness, pain, visual loss, bowel/bladder dysfunction, and cognitive dysfunction. Diagnosis of MS typically occurs through the application of highly structured diagnostic criteria that rely on clinical observation, neurological examination, brain and spinal cord Magnetic Resonance Imaging (MRI) scans, evoked potentials, and examination of cerebrospinal fluid (CSF) [2, 3].

MS is clinically subcategorized into four phenotypic disease patterns distinguished by the occurrence and timing of relapses relative to disease onset and disability progression [4]. These include relapsing remitting MS (RRMS), primary progressive MS (PPMS), progressive relapsing MS (PRMS); and secondary progressive MS (SPMS).

Approximately 80% of MS patients present with RRMS. If left untreated, the majority of RRMS patients will transition into SPMS (with progressive loss of neurologic function, in the absence of relapses) within 20 years. The term relapsing MS (RMS) applies to those patients either RRMS or SPMS, who continue to suffer relapses. Patients with RMS, whether or not they suffer from neurologic progression in the absence of relapses, have a common, inflammatory pathophysiology and therefore, constitute a common target for treatment.

Currently available first-line therapies for the treatment of either relapsing MS or relapsing-remitting MS include interferon (IFN)- β-1a (Rebif® and Avonex®), IFN-β-1b (Betaferon®/Extavia®) and glatiramer acetate (Copaxone®). The currently approved first-line treatments are only modestly effective in reducing the frequency of relapses and preventing disability in patients with RMS. The magnitude of these disease modifying effects are an approximately 30% relative improvement versus placebo [5]. The first-line disease modifying agents reduce the frequency of new episodes but do not reverse fixed deficits and have questionable effects on long-term disease progression [6].

Fingolimod (FTY720) is an oral modulator of sphingosine-1 phosphate (S1P) receptors, a unbiquitous group of transmembrane receptors involved with cellular growth and differentiation. Fingolimod's immunomodulatory effects are believed to be due to binding to and internalization of the S1P1 receptor on lymphocytes, thereby rendering them insensitive to S1P gradients in lymph and inhibiting egress from lymph nodes and other secondary lymphoid organs. Fingolimod is known to readily cross the blood-brain barrier and there are S1P receptors on glial cells and neurons, however the implications

of any possible direct CNS S1P receptor modulation effects are currently unknown. Fingolimod was shown to reduce ARR by approximately 50% versus both placebo and interferon beta-1a, 30 mcg im weekly (Avonex®), in confirmatory Phase III clinical trials. Due to the presence of S1P receptors on many different cell types, the adverse event profile of fingolimod is complex, with potential effects on cardiac, ophthalmic, hepatic and pulmonary function, as well as an increased risk of infection, due to inhibition of lymphocyte trafficking. Fingolimod was approved in 2010 by the FDA for patients with relapsing forms of MS and has recently gained a favorable ruling by the CHMP, for use in patients who have previously failed first-line disease-modifying therapy or who have highly active disease.

Natalizumab (Tysabri®) is a monoclonal antibody directed against alpha-4 beta-1 integrin (VLA-4), an adhesion molecule expressed on activated lymphocytes. Natalizumab binds to VLA-4, inhibiting trafficking of activated lymphocytes into the CNS and other extravascular tissues. Natalizumab was shown to have a 66% relative reduction in ARR versus placebo in a Phase III clinical trial. Natalizumab use is generally limited to RRMS patients who have failed to respond to first-line disease modifying therapy or to highly active RRMS patients due to a risk of Progressive Multifocal Leukoencephalopathy (PML).

Mitoxantrone (Novantrone®), a chemotherapeutic agent, is also approved for treatment of relapsing MS in the United States of America, but is generally reserved for secondary progressive and severe relapsing remitting forms of disease. Other drugs have been used with varying degrees of success, including corticosteroids, methotrexate, cyclophosphamide, azathioprine, and intravenous immunoglobulin.

Despite significant advances in MS therapy many patients continue to experience disease activity; thus there remains a need to develop more effective and better tolerated therapies for the treatment of RMS.

### 1.1.2 Ocrelizumab

Ocrelizumab is a humanized, glycosylated, monoclonal antibody directed against the CD20 antigen present on select B-cells. Ocrelizumab binds to the CD20 antigen thereby resulting in B-cell depletion via antibody-dependant cellular cytotoxicity (ADCC), complement-dependent cytotoxicity (CDC) and enhanced apoptosis.

Ocrelizumab was constructed using a recombinant DNA technique. This antibody shares an overlapping epitope on CD20 with rituximab (chimeric monoclonal antibody, Mabthera®/Rituxan®), as determined by direct competition and epitope-mapping experiments. In-vitro, ocrelizumab was shown to be approximately 5 times more potent than rituximab in ADCC activity on a B-cell tumor line over-expressing CD20, approximately 3 times less potent via CDC, and approximately equal in inducing apoptosis in a B-cell lymphoma cell line.

There is substantial proof-of-concept clinical data to support the use of B-cell depleting therapies in patients with relapsing MS. Ocrelizumab shares the same basic mechanism of action as rituximab. In a proof of concept study, rituximab treatment resulted in a robust reduction in MRI based measures of CNS inflammation and clinical benefit vs.

placebo, in patients with RRMS [7]. WA21493/ACT4422g, an ongoing Phase II study of ocrelizumab in RRMS patients provides proof-of-concept support for ocrelizumab efficacy and safety in patients with relapsing remitting MS; please refer to Section 1.1.4.1 for more details.

Ocrelizumab is also known as Ro 496-4913, PRO70769 and rhuMAb 2H7 (refer to Investigator's Brochure for further information).

### 1.1.3 Rationale for Targeting B-cells in MS

Humoral immunity has been implicated in MS for decades, as evidenced by inclusion of cerebrospinal fluid (CSF) oligoclonal bands (OCB) and increased intrathecal IgG synthesis in diagnostic criteria for MS [2, 3, 8]. Although, until very recently, the prevailing view of MS pathophysiology held that the CNS inflammation seen in MS is principally mediated by $CD4^+$ proinflammatory (Th1, Th17) T cells, rapidly expanding evidence suggests that B-cells may contribute to MS pathogenesis much more fundamentally than was previously believed, potentially through either antibody-dependent or independent mechanisms [9, 10, 11]. B lymphocytes have been detected within MS lesions and in the CSF of MS patients. Molecular analysis of both lesional and CSF B-cell repertoires reveals dominant, clonally expanded B-cell populations exhibiting somatic hypermutation in the antigen-recognizing CDR3 regions of immunoglobulin (Ig) heavy chains, predominately within the VH4 gene family [12, 13, 1, 15, 16, 17, 18].

Detection of these affinity-matured, clonally expanded repertoires in the CSF but not peripheral blood of MS patients suggests that a localized, antigen driven B-cell response is present in the CNS compartment. CSF clonal B-cell expansion has been reported in patients with both RRMS and PPMS shortly after diagnosis, implying a role for B-cells early in MS pathogenesis rather than as a late response to longstanding tissue damage [19]. More recently, cDNA transcriptomes of clonally expanded affinity-matured B-cells isolated from the CSF of MS patients have been sequence-matched to specific IgG OCBs from the same CSF samples, indicating that this longstanding hallmark of MS diagnosis derives from identifiable B-cell clones present in the CNS compartment [20].

Both antibody-dependent and independent hypotheses for the role of B-cells in MS pathophysiology have been postulated and are currently the subject of intensive research. B-cells may differentiate into plasma cells and produce CNS-directed auto-antibodies, potentially triggering cellular and complement-dependent cytotoxicity. Although a pathogenic role of anti-myelin antibodies in MS has not been established, they have been detected in the CSF of MS patients [21, 22, 23] and in active MS lesions [24] and remain potential candidates as effectors of myelin sheath damage. B-cells may also function as antigen presenting cells and thereby modulate effector T-cell responses, as they exhibit regulated secretion of both pro-inflammatory and anti-inflammatory cytokines, a function that appears to be abnormal in patients with MS [9]. Finally, B-cells may be a site of latent viral infections such as Epstein Barr Virus (EBV), which may drive CNS autoimmune responses through molecular mimicry or other pro-inflammatory mechanisms [10].

Postmortem pathological studies have identified the presence of ectopic follicular lymphoid structures in the meninges anatomically proximal to sites of grey matter demyelination in a subset of SPMS patients [25, 26, 14]. Similar tertiary lymphoid structures form *de novo* in various tissues of many autoimmune disorders and represent potential *de novo* sites of chronic autoantigenic B-cell activation, maturation and clonal expansion [28]. SPMS patients exhibiting these lymphoid structures have been found to have worse progression rates, when compared to controls without such follicular structures [29] and a pathomechanistic link to grey matter demyelination typical for SPMS has been suggested. Whether or not an anti-CD20 therapeutic antibody can affect the formation or persistence of meningeal lymphoid follicles or the grey matter demyelination prominent in progressive forms of MS is unknown.

In summary, B lymphocytes are believed to contribute to the pathogenesis of all subtypes of MS. Removing select peripheral B-cells from circulation may beneficially disrupt inflammatory processes that potentially involve chronic antigenic stimulation or other regulatory functions promoting chronic autoimmunity. Ocrelizumab specifically depletes CD20+ B-cells, making it a potentially attractive pharmacological agent to test for therapeutic potential in patients with multiple sclerosis.

### 1.1.4 Sponsor Experience with Anti-CD20 Compounds in MS

#### *1.1.4.1 Ocrelizumab in RRMS*

Study WA21493/ACT4422G is a 220-patient Phase II, multicenter, randomized, parallel-group, placebo-controlled, proof-of-concept study to evaluate the safety and efficacy of two dose regimens of ocrelizumab (1000 mg x 2 [administered on Day 1 and Day 15, followed by single infusions of 1000 mg for subsequent cycles] and 300 mg x 2 [administered on Day 1 and Day 15 followed by single infusions of 600 mg for subsequent cycles]), with an additional randomized open label arm of interferon β1-a 30 μg i.m. every week (Avonex®) arm. The primary objective was to evaluate the efficacy of two dose regimens of ocrelizumab compared with placebo, in reducing brain inflammation, as measured by the total number of gadolinium-enhancing T1 lesions observed on serial MRI scans of the brain at Weeks 12, 16, 20, and 24. Key secondary objectives were to evaluate the efficacy of both dose regimens of ocrelizumab compared with placebo in reducing annualized relapse rates at 24 weeks and to evaluate the safety and tolerability of both dose regimens of ocrelizumab in patients with RRMS. Exploratory outcomes included analysis of both dose regimens of ocrelizumab compared to interferon β-1a 30 μg i.m. weekly (Avonex®) along various study measures. Treatment with ocrelizumab is planned for 72 to 96 weeks total, depending on study arm (patients from both placebo and Avonex group switched to ocrelizumab 300 mg x 2 after Week 24). Additional MRI scans of the brain will be obtained at weeks 96 and 144 for a subgroup of patients.

This study is currently ongoing. Week 24 results demonstrated that both doses of ocrelizumab achieved the primary endpoint by significantly reducing the number of gadolinium-enhancing lesions compared with placebo ($p < 0.0001$). Both OCR dose groups showed statistically significant reductions in ARR compared with the placebo group (ARR = 0.125 for the OCR 300 mg x 2 group [$p = 0.0005$] and ARR = 0.169 for the OCR 1000 mg x 2 group [$p = 0.0014$] compared with ARR = 0.637 for the placebo

group, representing a relative reduction (RR) of 80% and 73% in ARR versus placebo group for the low and high OCR groups, respectively. In exploratory analyses, both ocrelizumab groups were superior to the Avonex group for the primary endpoint ($p < 0.0001$) and the 300 mg x 2 group for ARR (ARR = 0.364 for the Avonex group, representing a RR of 66% in ARR with $p = 0.03$ for the OCR 300 mg x 2 group versus Avonex group and a RR of 53.6% in the ARR with $p = 0.086$ for the OCR 1000 mg x 2 group versus Avonex group).

Patients from both placebo and Avonex groups switched to ocrelizumab 300 mg x 2 after Week 24. By 48 weeks, the level of benefit of ocrelizumab in reduction of ARR was maintained, where the patients in the ocrelizumab 300 mg x 2 group continued to have a suppressed ARR of 0.086 from Week 24 to 48, and patients switched to ocrelizumab from either placebo or Avonex®, derive a similar degree of efficacy to those randomized to ocrelizumab from onset (ARR for placebo-to-ocrelizumab = 0.161 and for Avonex-to-ocrelizumab = 0.137 after the switch, representing a RR of 74% and 62.4% compared with ARR before the switch respectively). From week 0 to 72, patients originally randomized to ocrelizumab 300 mg x 2 maintained clinical efficacy with an ARR of 0.186.

The most commonly reported adverse events in ocrelizumab-treated patients were infusion related reactions (IRRs). IRRs were reported during/after the first infusion (Day 1) for 30–43.6% of patients treated with ocrelizumab. Fewer patients (2.1–9.4%) experienced IRRs during/after the second infusion (Day 15). The most common symptoms were rash, pruritus, flushing, tachycardia, headache, pyrexia, and throat irritation.

No unanticipated, clinically significant abnormalities in vital signs, electrocardiograms (ECGs), or laboratory parameters were observed in association with ocrelizumab treatment.

On review of the placebo-controlled, double-blinded 24-week safety data, no imbalance in adverse events (or infection adverse events) or serious adverse events (or infection serious adverse events) between the placebo and active ocrelizumab arms was observed. The rate of adverse events (or infection adverse events) and serious adverse events (or infection serious adverse events) did not increase in ocrelizumab-treated patients at 48 weeks compared with 24 weeks. There is no trend toward an increased risk of adverse events (or infection adverse events) or serious adverse events (or infection serious adverse events) for ocrelizumab-treated patients with previous IFN treatment (Avonex for 6 months).

By the time all patients finished the Week 48 of treatment period, the incidence of infections and serious infections was 92.41/100 PY (95% CI: 76.59, 111.5) and 3.39/100 PY (95% CI: 1.27, 9.04) in patients exposed to low-dose ocrelizumab, including patients who switched from placebo or Avonex®. The incidence of infections and serious infections was 97.38/100 PY (95% CI: 74.76, 126.84) and 5.31/100 PY (95% CI: 1.71, 16.47) in those exposed to the high dose of ocrelizumab. The most common infections in ocrelizumab-treated patients included urinary tract infections, upper respiratory infections, and nasopharyngitis.

To date, in study WA21493, after over 250 patient years exposure to ocrelizumab, there have been no reports of opportunistic or fatal infections. There was 1 death during the 24-week treatment period: a patient in the ocrelizumab 1000 mg x 2 group was hospitalized with acute onset of encephalopathy and status epilepticus due to systemic inflammatory response syndrome with disseminated intravascular coagulation of unknown cause, following infusion of gadolinium. The patient's course rapidly progressed to multi-organ failure. While hospitalized, the patient developed nosocomial pneumonia in the setting of severe renal and hepatic insufficiency. After 2 weeks of intensive care the patient died of transforaminal herniation of the brain, due to massive cerebral edema.

Study patients have now completed Week 72, and preliminary analysis shows that the rates of serious infections remain stable over time compared to 24 or 48 week experience. A comprehensive analysis of the safety and efficacy data through this time point is currently ongoing. The Data Monitoring Committee, based on analysis of safety and efficacy outcomes from the 72-week analysis recommended continuation of Phase II WA21493 study; the DMC also agreed that these results support further development of ocrelizumab in Phase III program.

#### *1.1.4.2 Rituximab in RRMS*

Two clinical trials have been conducted in RRMS patients with rituximab, a chimeric mouse/human monoclonal antibody that binds shares the same basic mechanism of action as ocrelizumab. Findings briefly highlighted below, offer additional support for the therapeutic potential of the anti-CD20 mechanism in MS.

Study U3264g (HERMES Jr.) was a Phase I, open-label, multicenter study in 26 adults with RRMS to evaluate the safety and tolerability of two treatment cycles of rituximab administered at baseline and after 24 weeks. Re-treatment with rituximab (1000 mg × 2) at 24 weeks was safe and well tolerated, with an observed decrease in relapses and gadolinium-enhancing lesions through 72 weeks [30].

Study U2787g (HERMES) was a Phase II, proof-of-concept, randomized, double-blind, parallel-group, placebo-controlled, multicenter study to evaluate the safety and efficacy of rituximab in 104 adults with RRMS. The primary objectives were to investigate the efficacy of rituximab compared with placebo, as measured by the total number of gadolinium-enhancing T1 lesions observed on serial MRI scans of the brain at Weeks 12, 16, 20, and 24, and to evaluate the safety and tolerability of rituximab in patients with RRMS. Secondary objectives were to evaluate additional MRI parameters and the proportion of patients relapsing. The trial met its primary efficacy endpoint and all secondary endpoints. Rituximab was safe and generally well tolerated in this study through 48 weeks though the rate of infusion-associated adverse events, particularly after the first infusion, was higher in rituximab-treated patients (78%) than in placebo patients (40%); corticosteroid pre-medication was not administered before or at the time of infusion. Study U2787g provides proof of principle that an anti-CD20 therapeutic approach can reduce both MRI and clinical evidence of inflammatory activity in adults with RRMS [7].

#### 1.1.4.3 Rituximab in PPMS

A single Phase II/III, randomized, double blinded, placebo-controlled trial was conducted with rituximab in PPMS. The findings, summarized below, represent the largest and longest duration trial experience to date evaluating the safety and efficacy of anti-CD20 therapy in individuals with multiple sclerosis.

Study U2786g (OLYMPUS) was a Phase II/III randomized, double-blind, parallel-group, placebo-controlled, multicenter study evaluating the safety and efficacy of rituximab in patients with PPMS over a 96 week treatment period consisting of 4 treatment cycles with dual infusions of 1000 mg (2000 mg/cycle). Although the trial did not demonstrate significant primary efficacy on time to confirmed disease progression as measured by Expanded Disability Status Scale (EDSS), a difference was observed with 38.5% of patients in the placebo group experiencing confirmed disease progression vs. 30.2% in the rituximab group. Biological activity was evidenced by significantly lower T2 lesion volume accumulation on brain MRI, a secondary efficacy endpoint, in rituximab-treated patients compared with placebo (p=.0008). Subgroup analyses suggest that PPMS patients with evidence of active disease may have shown significant clinical treatment response as measured by time to confirmed disease progression over a 96-week timeframe. Factors that appeared prognostic for disease progression and potentially predictive of treatment response in the rituximab group included younger age, presence of contrast enhancing lesions at baseline on brain MRI, and higher MS severity score.

Rituximab was generally safe and well tolerated in Study U2786g. The proportions of patients with at least one adverse event (100% placebo vs. 99% rituximab) and one serious adverse event (13.6% placebo vs. 16.1% rituximab) were comparable between treatment groups. Three adverse events that occurred during the study led to death: one in the rituximab group following recurrent aspiration pneumonias and two in the placebo group due to pneumonia and cardiopulmonary failure. More infusion-associated adverse events were observed in rituximab-treated patients (73.6% vs. 40.3% for placebo), particularly after the first infusion, but rates declined in both groups to similar levels upon successive infusions. Patients were not premedicated with glucocorticoids before rituximab infusions in Study U2786g. The vast majorities (92%) of infusion associated events in rituximab treated patients were mild to moderate in severity; no Grade 4 or 5 infusion-associated events were observed. The proportion of patients with at least one infection was comparable between groups (68.2% rituximab vs. 65.3% placebo), but a higher proportion of patients with at least one serious infection was observed in the rituximab-treated group (4.5%) compared with placebo (<1%). No opportunistic infections occurred.

Treatment with rituximab was associated with rapid and near-complete depletion of circulating CD19 positive B lymphocytes beginning 2 weeks post-treatment through 96 weeks. Approximately 35% of rituximab-treated patients had recovered peripheral CD19 B-cell counts to 80 cells/μL (laboratory defined lower limit of normal (LLN) in healthy volunteers) within 48 weeks after the last dose. Median circulating CD3 T-lymphocyte counts were not appreciably altered by rituximab. At any time in the trial, IgM levels were below the LLN in 31.7% of rituximab-treated patients and 5.9% of placebo-treated patients. The proportion of patients with IgG and IgA levels below LLN

were not different between groups. The incidence of infectious adverse events and infectious SAEs did not appear higher in patients with immunoglobulin levels (all isotypes) below LLN in either treatment group compared with patients with immunoglobulin levels in the normal range or above upper limit of normal (ULN) [31].

### 1.1.5 Rebif®

The active comparator for this study is Rebif® (interferon β-1a), which has been approved for treatment of relapsing multiple sclerosis.

The efficacy and safety of Rebif® was demonstrated in the PRISMS study (The Prevention of Relapses and Disability by Interferon β-1a Subcutaneously in Multiple Sclerosis) which led to the approval of Rebif® in RMS. This was a multicentre controlled trial of 560 patients with an EDSS score between 1.0 and 5.0 and at least two relapses in the preceding 2 years. Patients were randomized to 2-year treatment with placebo or IFN β-1a (22 or 44 µg subcutaneously three times weekly). Following the 2 years of treatment, both doses of Rebif® showed significant benefits compared with placebo on major efficacy outcome measures. There was a non-significant trend towards greater efficacy with the higher dose on most clinical measures, and a statistically significant dose-effect favoring the higher dose in terms of impact on the number of T2-active lesions. In a subgroup of patients with more severe disease (baseline EDSS >3.5), the 44-µg dose delayed progression of disability significantly better than either the placebo or the 22 µg dose. Neutralizing antibodies were significantly less frequent in the 44 µg group than in the low-dose group [33].

After 2 years, patients who had initially received placebo in the PRISMS study were re-randomized to receive Rebif® (22 or 44 µg subcutaneously three times weekly) and were followed for an additional 2 years. By the end of the 4 year period, patients who had switched from placebo to Rebif® experienced an approximate 50% reduction in ARR compared with the end of year 2. Also, after 4 years, the higher dose approached significance for annual relapse rates (0.8 for 22 µg vs. 0.72 for 44 µg; p=0.069). The mean ARR was significantly lower in patients who had received Rebif® for the full 4 years compared with those who had received placebo for the first 2 years. During Years 3 and 4, relapse rates were significantly lower for the 44 µg group with relapse rates decreasing progressively with each year of treatment – 0.92, 0.82, 0.57, and 0.44 relapses/year for each year of the study. Patients who received the highest cumulative dose of active therapy had the lowest rate of disability progression. The time to first confirmed EDSS progression was 42.1 months for the 44 µg group compared with 24.2 months for the crossover group. The time to first confirmed progression did not differ significantly between the 22 µg group (35.9 months) and the crossover group [34].

Rebif® showed superiority versus Avonex® in the EVIDENCE trial. This was a randomized, controlled, multicenter trial which compared the efficacy and safety of Rebif® 44 µg subcutaneously three times weekly and Avonex® 30 µg i.m. once weekly in 677 patients with RRMS. The primary endpoint was the proportion of patients who were relapse free at 24 weeks; the principal MRI endpoint was the number of active lesions per patient per scan at 24 weeks. After 24 weeks, 74.9% of patients receiving Rebif® 44 µg three times a week remained relapse free compared with 63.3% of those given Avonex® 30 µg once a week. Patients receiving Rebif® 44 µg three times a week had fewer active

MRI lesions (p = 0.001 at 24 and 48 weeks) compared with those receiving Avonex® 30 µg once a week. Injection-site reactions were more frequent with Rebif® 44 µg three times a week (83% vs. 28%, p = 0.001), and there were asymptomatic abnormalities of liver enzymes (18% vs. 9%, p= 0.002) and altered leukocyte counts (11% vs. 5%, p=0.003) compared with the Avonex® 30 µg once a week dosage. Neutralizing antibodies developed in 25% of Rebif® 44 µg three times a week patients and in 2% of patients receiving Avonex® 30 µg once a week [32].

Rebif® has also been studied in the SPMS population. The SPECTRIMS study was a multicenter, randomized, parallel-group, placebo-controlled study which tested two doses of Rebif® in patients with SPMS. Patients had to have clinically definite SPMS which was defined as progressive deterioration of disability for at least 6 months with an increase of at least 1 EDSS point over the previous 2 years (or 0.5 point between EDSS score of 6.0 and 6.5), with or without relapses, following an initial course of RRMS. Baseline EDSS scores had to be from 3.0 to 6.5 and the pyramidal functional score of at least 2. Patients were randomized to 3-year treatment with placebo or IFN β-1a (22 or 44 µg subcutaneously three times weekly). The primary outcome was time of confirmed progression, defined as increase from baseline by at least 1 EDSS point (or 0.5 point if baseline EDSS ≥ 5.5), confirmed 3 months later with no intervening score lower than the minimum required level. The primary outcome was not significantly influenced by treatment with Rebif® as compared to placebo (p = 0.146). A significant benefit was seen on relapse rate for both doses of Rebif®. These findings suggest that treatment with Rebif® has clinical benefit in SPMS, predominantly affecting relapses, but only modest effect on disability [35].

In controlled clinical trials, the most commonly observed adverse reactions were: injection-site reactions, influenza-like symptoms (headache, fatigue, fever, rigors, chest pain, back pain, myalgia), elevated liver enzymes, hematological abnormalities, abdominal pain, and depression. Most of these adverse reactions are unique to treatment with interferon beta, presenting potential difficulties in maintaining blinding in controlled clinical trials. Summary of the most frequent Rebif® adverse reactions by MedDRA system organ class have been summarized in Table 1.

**Table 1: Summary of the Most Frequent Rebif® Adverse Reactions by MedDRA System Organ Class**

| System Organ Class | Very Common ADR *frequency of occurrence ≥ 1/10* | Common ADR *frequency of occurrence ≥ 1/100 to 1/10* |
|---|---|---|
| **General disorders and administration site conditions** | Injection site inflammation, injection site reaction, influenza-like symptoms | Injection site pain, fatigue, rigors, fever |
| **Investigations** | Asymptomatic transaminase increase | Severe elevation of transaminase |
| **Blood and lymphatic system disorders** | Neutropenia, lymphopenia, leucopenia, thrombocytopenia, anaemia | |
| **Psychiatric disorders** | | Depression, insomnia |
| **Nervous system disorders** | Headache | |
| **Gastrointestinal disorders** | | Diarrhoea, vomiting, nausea |
| **Skin and subcutaneous tissue disorders** | | Pruritus, rash, erythematous rash, macula-papular rash |
| **Musculoskeletal and connective tissue disorders** | | Myalgia, arthralgia |

*Based on Rebif® Summary of Product Characteristic 2010 [36]. Please refer to local label for more details.*

Severe liver injury, including some cases of hepatic failure requiring liver transplantation, has been reported rarely in patients taking Rebif®. Treatment with Rebif® should be stopped immediately if jaundice or other symptoms of liver dysfunction appear [36, 37]. Please refer to Section 6.2.2 for further details.

Neutralizing antibodies to IFN β-1a can develop in some patients, usually following the first year of therapy. Long-term consequences of these antibodies are still not known, however, current evidence shows that they may reduce the efficacy of the drug. The antibodies tend to cross-react with different IFN β formulations. For this reason, switching to another IFN β drug is unlikely to be effective [38, 39].

## 1.2 Rationale for the Study

This study serves as a pivotal Phase III clinical trial designed to demonstrate the efficacy and safety of ocrelizumab in relapsing multiple sclerosis in comparison to high dose high frequency IFN (Rebif®). This study is part of a broader, confirmatory clinical development program investigating the safety and efficacy of ocrelizumab in patients

with both primary progressive and relapsing multiple sclerosis. A Phase II study WA21493/ACT4422G is ongoing in RRMS patients and three Phase III pivotal trials are planned, (including the one presented in this protocol), two in RMS and one in PPMS. Please refer to Section 3.1.1 for further details on study design and choice of comparator.

# 2. OBJECTIVES

## 2.1 Primary Objective

The primary objective of this study is to assess whether the efficacy of ocrelizumab 600 mg (given as dual infusions of 300 mg on Days 1 and 15 of the first 24-week treatment cycle and as a single infusion of 600 mg on Day 1 of each 24-week treatment cycle thereafter) intravenously every 24 weeks is superior to Rebif® as measured by the annualized protocol-defined* relapse rate by two years (96 weeks) in patients with relapsing multiple sclerosis.

**See Section 5.3.2.1 for the definition of protocol-defined relapse.*

## 2.2 Secondary Objectives

The secondary objectives of this study are to evaluate whether the efficacy of ocrelizumab is superior to Rebif®, as reflected by the following measures:

- The time to onset of sustained disability progression for at least 12 weeks during the 96-week comparative treatment period. **
- The time to onset of sustained disability progression for at least 24 weeks during the 96-week comparative treatment period. **
- The proportion of relapse-free patients by 96 weeks.
- The change in total T2 lesion volume as detected by brain MRI from baseline to Week 96.
- The total number of new, and/or enlarging T2 hyperintense lesions as detected by brain MRI at week 24, week 48 and week 96.
- The change in Multiple Sclerosis Functional Composite Scale (MSFCS) score from baseline to Week 96.
- The change in brain volume as detected by brain MRI from Week 24 to Week 96.

***See Section 5.3.2.2 for the definition of sustained disability progression.*

**Safety:**

To evaluate the safety and tolerability of ocrelizumab 600 mg (given as dual infusions of 300 mg on Days 1 and 15 of the first 24-week treatment cycle and as a single infusion of 600 mg on Day 1 of each 24-week treatment cycle thereafter) intravenously every 24 weeks in patients with relapsing MS.

**Pharmacokinetics/Pharmacodynamics:**

To explore the pharmacokinetics, immunogenicity and pharmacodynamics of ocrelizumab in patients with relapsing MS.

## 2.3 Exploratory Objectives

- The change in low-contrast visual acuity from baseline to Weeks 48 and 96.
- The change in the Symbol Digit Modalities Test from baseline to Weeks 48 and 96.
- The annualized relapse rate, based on clinical and protocol-defined relapses at the end of the 96-week comparative treatment period.
- The total number of T1 gadolinium-enhanced lesions as detected by brain MRI at Weeks 24, 48, and 96.
- The change in brain volume as detected by brain MRI from baseline to Week 96.
- The change in brain volume as detected by brain MRI from Week 48 to Week 96.
- The change in Multiple Sclerosis Functional Composite Scale (MSFCS) score from baseline to Week 48.
- The cumulative change in EDSS scores, measured in area under the curve (AUC) by Week 96.
- The change in EDSS from baseline to Weeks 48 and 96.
- The change in timed 25 foot walk from baseline to Weeks 48 and 96.
- The change in 9-hole peg test from baseline to Weeks 48 and 96.
- The change in paced auditory serial addition test (PASAT) from baseline to Weeks 48 and 96.
- The time to onset of sustained 20% increase in 9-hole peg test for at least 12 weeks.
- The time to onset of sustained 20% increase in timed 25 foot walk for at least 12 weeks.
- Patient Reported Outcomes (PROs): Modified Fatigue Impact Scale (MFIS), EuroQol instrument (EQ-5D), Center for Epidemiology Studies Depression Scale (CES-D) and Medical Outcomes Study 36-Item Short Form Survey Instrument (SF-36) at baseline, Week 48 and Week 96.
- The change in Karnofsky Performance Status Scale (clinician-reported version) score from baseline to Weeks 48 and 96.

## 2.4 Roche Clinical Repository (RCR) Exploratory Objectives

The Roche Clinical Repository (RCR) is a centrally administered facility for the long term storage of human biological specimens including body fluids, solid tissues and derivatives thereof (e.g. DNA, RNA proteins/ peptides). Specimens stored in the RCR will be used to:

- Study the association of biomarkers with efficacy and/ or adverse events associated with ocrelizumab
- Increase the knowledge and the understanding of biology of multiple sclerosis and mode of action of ocrelizumab.

# 3. STUDY DESIGN

## 3.1 Overview of Study Design and Dosing Regimen

**Figure 1: Overview of Study Design**

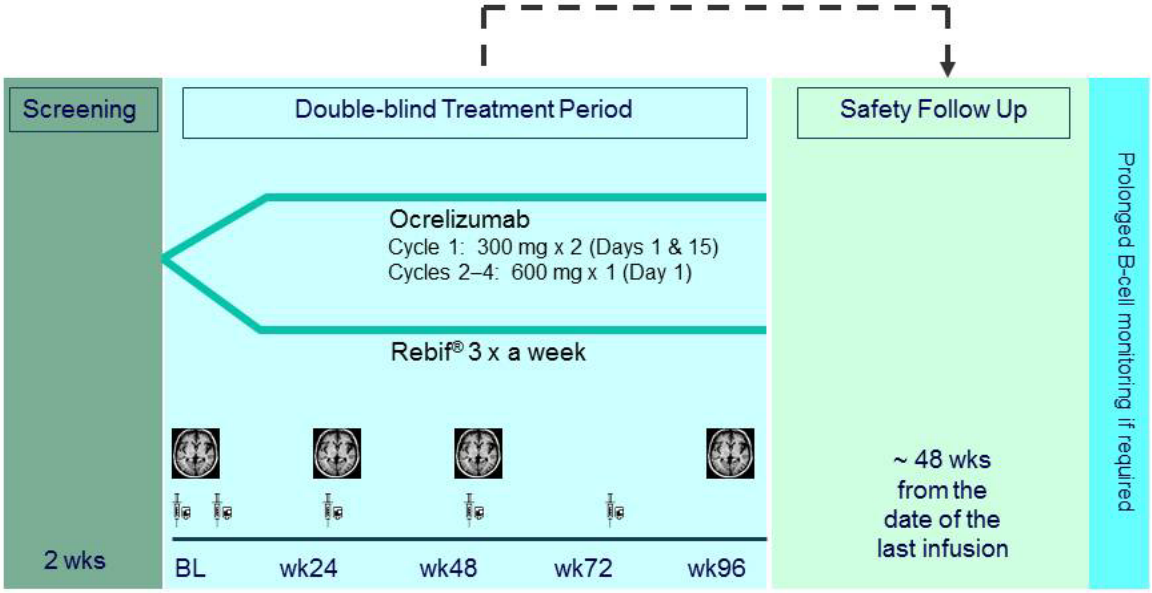


The study will consist of the following periods:

**Screening:**

Consenting patients will enter the 2-week screening period to be evaluated for eligibility. Procedures at screening will include collecting medical history, medical examination including thorough neurological exam, EDSS score, LCVA score, SDMT score, MSFCS score, ECG, blood and urine sampling. Please see Table 3 - "Schedule of Assessments: Screening Through the End of Double-Blind Treatment Period" for further details.

*Please note that based on local Ethics Committees or National Competent Authority requirements, additional diagnostic testing may be required for selected patients or selected centers to exclude tuberculosis, Lyme disease, HTLV-1 associated myelopathy (HAM), acquired immune deficiency syndrome (AIDS), hereditary disorders, connective tissue disorders, or sarcoidosis.*

**Treatment Period:**

**- Double-blind, comparative treatment period**

Eligible patients will be randomized via IxRS into one of two treatment groups: ocrelizumab 600 mg regimen (group A) or interferon β-1a - Rebif® (group B) – please refer to Table 5 and Table 8 for more details.

During the double-blind comparative treatment period, patients will be assessed at clinical visits as per Schedule of Assessments: Screening Through the End of Double-Blind Treatment Period – please refer to Table 3 for further details.

Prior to the next cycle of study drug, patients will be evaluated for pre-specified conditions and laboratory abnormalities to allow for re-treatment please refer to Section 6.1.4 for more details.

**Patients who complete the 96-week treatment period may become eligible for a separate open-label extension study, under a separate protocol.**

**Please note: Patients who discontinue from study medication within the 96-week double-blind, comparative phase (treatment period) of the study will enter the Safety Follow up Period (see below); they will not be eligible for the open-label extension study, even if they complete the 96-week treatment period.**

**Safety Follow up Period**

Patients who discontinue treatment with study drug will enter the Safety Follow up Period for at least 48 weeks counting from the date of the last infusion of the ocrelizumab/ocrelizumab placebo. However, if after this time the peripheral blood B-cells remain depleted, patient should continue to be monitored at 24-week intervals until B-cell count has returned to the baseline value or to the lower limit of the normal range (whichever is the lower). Please refer to Figure 2 for more details.

**Figure 2: Safety Follow up - Variable B-cell Monitoring.**

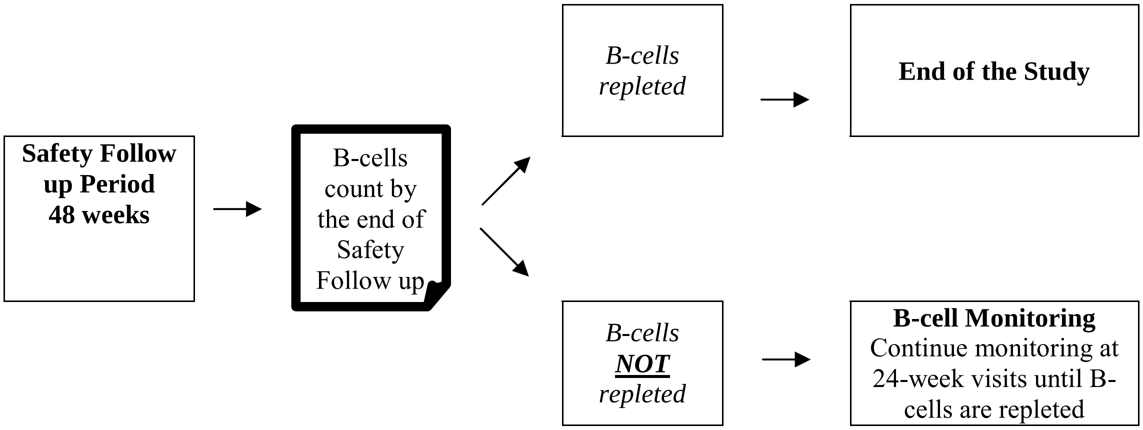


*Please note: patients in Safety Follow up who receive other B-cell targeted therapies will only be followed for 48 weeks from the date of the last infusion of the study drug regardless of their B-cell count.*

During Safety Follow up patients will be assessed at clinical visits every 12 weeks. Telephone interviews will be performed every 4 weeks. If prolonged B-cell monitoring is required, patients will be assessed at clinical visits every 24 weeks and telephone interviews will be performed every 12 weeks. Please refer to Schedule of Assessments: Safety Follow up (including prolonged B-cell monitoring if required)—Table 4—for further details.

**Every effort should be made to have patients who withdraw from Study Medication complete the Safety Follow up Period and all related assessments, regardless of whether or not they receive alternative treatment for MS.**

**Table 2: Overview of Dosing Regimen**

| **Group** | **Treatment Period**[1,2] | | | | |
|---|---|---|---|---|---|
| | **1st Cycle**[3] (Weeks 1-24) | | **2nd Cycle**[3] (Weeks 24-48) | **3rd Cycle**[3] (Weeks 48-72) | **4th Cycle**[3] (Weeks 72-96) |
| | **Day 1 Infusion** | **Day 15 Infusion** | **Week 24 Infusion** | **Week 48 Infusion** | **Week 72 Infusion** |
| **A Ocrelizumab 600 mg regimen** | **Ocrelizumab** 300 mg i.v. | **Ocrelizumab** 300 mg i.v. | **Ocrelizumab** 600 mg i.v. | **Ocrelizumab** 600 mg i.v. | **Ocrelizumab** 600 mg i.v. |
| **B Rebif® regimen**[4] | **Rebif®** s.c. three times per week | → | → | → | → |

1. The treatment period consists of 96 weeks of treatment; patients will receive a maximum of 4 treatment cycles.
2. Each treatment cycle has a duration of 24 weeks. The first cycle consists of two 300 mg ocrelizumab i.v. infusions separated by 14 days. Cycles 2 – 4 consist of a single i.v. infusion of 600 mg ocrelizumab.
3. Prior to each infusion, a clinical evaluation will be performed to ensure that the patient remains eligible for treatment.
4. Please refer to Table 8 for detailed Rebif® dosing regimen.

Please note: 100 mg of methylprednisolone i.v. will be administered in both treatment arms prior to each infusions of ocrelizumab/ocrelizumab placebo.

### 3.1.1 Rationale for Study Design

**Rationale for the use of an active comparator**

There is consensus in the MS community, that the use of placebo in Phase III studies of patients with RMS is (except in exceptional circumstances) ethically indefensible, due to the availability of established, effective therapies [40].

**Rationale for choice of active comparator**

Interferon β-1a 44 μg s.c. 3 times weekly (Rebif®) has been chosen as the active comparator for the ocrelizumab RMS, Phase III clinical program, based on its consistent evidence of efficacy on reducing MRI activity, relapses and disease progression in patients with relapsing forms of MS see Section 1.1.5.

**Rationale for double-blind, double-dummy study design**

The use of a double-blind, double-dummy study design minimizes the potential for bias and safeguards the integrity of the clinical data generated from this study. It is acknowledged that this approach increases patient's burden. However, this design reduces the risk of concluding that superiority to the active comparator was driven by patient and assessor bias. Regulatory agencies have mandated this study design be implemented throughout the Phase III clinical program. For additional measures intended to minimize bias, please see below.

**Rationale for choice of study population**

This study plans to enroll RMS patients with an EDSS score of 0 to 5.5 at screening who had two documented clinical attacks within the previous 2 years or one clinical attack that occurred within the last year prior to screening. These criteria have been implemented to further characterize the benefits of treatment with ocrelizumab in a wide range of RMS patients with varying degrees of disease activity and severity.

The age range will be limited to $\leq$ 55 years to avoid confounding by neurological conditions prevalent in older individuals, including but not limited to microvascular disease.

Exclusion of patients who have failed or cannot tolerate Rebif® prevents these patients from being randomized to further Rebif® therapy and reduces the potential for unbalanced dropout rates. Additional exclusion criteria, relating to concomitant diseases, laboratory parameters, and previous medications help to ensure patients safety in the study – please refer to Section 4.3 for more details.

**Rationale for study endpoints**

The proposed study endpoints are widely accepted as clinically relevant and have been used in numerous pivotal clinical trials, in relapsing MS. The primary endpoint for the study will be annualized relapse rate (ARR) over 96 weeks, based on protocol-defined relapses. Key secondary endpoints will include the time to onset of sustained disability progression, confirmed at scheduled clinic visits, for at least 12 and 24 weeks. Prevention of relapses as well as the prevention or delay of accumulation of sustained neurological disability are meaningful goals in the treatment of patients with MS.

**Rationale for the treatment duration**

The 96-week treatment duration has been chosen to allow for the assessment of clinical efficacy and safety over 2 years of treatment, consistent with current regulatory guidelines.

Patients who complete the double-blind treatment period may become eligible for participation in the extension study under a separate protocol. This will allow for collection of safety information with long-term exposure.

Please note: Patients who discontinue from study medication within the 96-week double-blind, comparative phase (treatment period) of the study will enter the Safety Follow up Period (see below); they will not be eligible for the open-label extension study, even if they complete the 96 week treatment period.

**Rationale for the Safety Follow up Period (including Prolonged B-cell Monitoring)**

Data collected during this period will allow evaluation of B-cell repletion after stopping anti-CD20 treatment and collection of safety and efficacy data to document maintenance of the effect and/or the potential for a withdrawal effect. Based on results obtained from Study U2786g with rituximab [7] up to 60-65% of the ocrelizumab-treated patients are anticipated to enter the B-cell Monitoring portion of the safety follow up period with targeted assessment every 24 weeks until their B-cell counts recover.

**Rationale for the use of methylprednisolone**

In order to mitigate the risk and severity of infusion-related reactions, 100 mg of i.v. methylprednisolone will be administered to all patients, approximately 30 minutes prior to administration of ocrelizumab/ocrelizumab placebo. In order to mitigate the risk, that even a low dose of methylprednisolone may have a small effect on the efficacy outcomes of the study, methylprednisolone will be administered to patients in all treatment groups. It is of note that the dose of methylprednisolone used for premedication will be up to 50 times smaller than that used for the symptomatic treatment of relapses in MS.

**Additional measures to mitigate bias**

The use of high dose high frequency (HDHF) interferon β, as the active comparator for this study, presents some difficulties for maintaining patient and physician blinding – please see Section 1.1.5.

To prevent potential unblinding as a result of adverse events or changes to laboratory results, the following, additional measures have been implemented:

- **The Examining Investigator/EDSS assessor** will perform the neurological examination, document the Kurtzke Functional Systems (KFS) scores and assess EDSS scores. The examining physician/EDSS assessor will not be involved with any aspect of medical management of the patient and will not have access to patient data. Every effort will be made to ensure that there is no change in the examining physician/EDSS assessor throughout the course of the study for any individual patient. The examining physician/EDSS assessors will be trained and instructed not to discuss what adverse effects (if any) the patient is experiencing from their medication. Examining physician/EDSS assessors will receive training in performing EDSS assessments prior to the beginning of the study and must have successfully passed an examination on performance of the *Neurostatus EDSS examination* within 24 months of participation. All examining physician/EDSS assessors will receive ongoing training on performance of the *Neurostatus EDSS examination* throughout the course of the study.

- **Patient education:** Prior to being examined by the Examining Investigator/EDSS assessor, patients will be instructed not to discuss what (if any) adverse effects they may be experiencing. Treating physicians and/or study coordinators should remind patients of these instructions prior to EDSS assessments and this should be documented in the source documents.

- **Blinded, central MRI assessments:** A blinded, central MRI reader will assess all on-study MRI scans. These assessments will provide independent confirmation of the relative changes in immune-mediated, CNS damage.

- **Blinding of laboratory parameters:** Laboratory parameters that may lead to unblinding to treatment assignment, such as FACS cell counts including $CD19^{+}$ cells, lymphocyte count, IgM and IgG levels, and type I interferon neutralizing antibody levels will be blinded in all patients, except those meeting unblinding criteria for safety reasons.

### 3.1.2 Rationale for Dose Selection

The dose for the ocrelizumab Phase III clinical program was chosen to bring the MS community significant improvement in clinical efficacy versus current standard of care, with acceptable safety. The dose of ocrelizumab in the Phase III clinical program is 600 mg ocrelizumab every 24 weeks (administered as dual infusions of ocrelizumab 300 mg on Days 1 and 15 of the first 24-week treatment cycle, and 600 mg on Day 1 of each 24-week treatment cycle thereafter). This dose has been established as the lowest, maximally effective dose, based on the results from study WA21493/ACT4422g. The safety of this dose has substantial support from the Phase III clinical program in rheumatoid arthritis (RA), an analogous, although systemic autoimmune disease, in a population at greater risk.

ACT2847g was a Phase I/II, dose escalation study in patients with RA, examining 5 dose regimens. In ACT2847g, the two lowest dose groups (receiving less than 200 mg x 2) demonstrated reduced clinical benefits on some endpoints, earlier return of peripheral B-cell counts and higher rates of immunogenicity. In the RA Phase III program, with the exception of patients recruited from Asia, the dose of 200 mg x 2 established a safety profile comparable to placebo. The higher dose of 500 mg x 2 demonstrated apparently superior efficacy, especially in "high hurdle" clinical endpoints and joint preservation, based on X-ray imaging.

In the MS population (study WA21493/ACT4422g), two doses of ocrelizumab were studied, 2000 mg (administered as dual 1000 mg infusions on Days 1 and 15 of the first, 24-week treatment cycle) and 600 mg (administered as dual 300 mg infusions on Days 1 and 15 of the first treatment cycle). Pre-specified primary and secondary efficacy analyses for Study WA21493/ACT4422g indicate that 300 mg x 2 of ocrelizumab is highly effective in suppressing MRI lesion activity and reducing the risk of clinical relapses in RRMS patients over 24 weeks. No difference in efficacy was seen between the ocrelizumab 1000 mg x 2 and 300 mg x 2 doses, on either MRI or clinical endpoints, in the ITT study population. However, exploratory analyses, stratifying groups according to baseline MRI activity, suggest superior efficacy with 1000 mg x 2 versus 300 mg x 2, at 24 weeks, in patients with MRI activity at baseline (≥ 4 enhancing lesions). Similarly, in these patients, the 1000 mg x 2 dose was apparently more effective than the 300 mg x 2 dose at Week 24 and (to a lesser extent) at Week 48, in reducing the absolute number of clinical relapses. Neither the MRI nor the clinical efficacy differences are statistically significant; however, these results suggest reduction of clinical efficacy at lower doses, in active MS patients. This apparent dose effect was seen despite the fact that linear kinetics (so that complete receptor occupancy can reasonably be assumed) and near complete peripheral CD19 suppression were observed for both doses. Preclinical studies in primates have shown differential susceptibility of tissue resident versus circulating B-cell populations in response to anti-CD20 antibodies. [41, 42, 43]. As tissue resident B-cell populations are beyond our ability to measure directly, it is likely that peripheral CD19 count is a sensitive but non-specific pharmacodynamic marker for anti-CD20 efficacy.

Conclusion

Based on available data, the dose of 600 mg of ocrelizumab i.v. (given as dual infusions of 300 mg 14 days apart for the first 24 weeks and a single infusion of 600 mg every 24 weeks thereafter) is the most likely dose to be able to demonstrate robust clinical efficacy, an acceptable safety profile and a low risk of immunogenicity, maximizing the likelihood of significant benefit versus standard of care in patients with relapsing MS.

#### 3.1.3 End of Study

The end of the study has been defined as the date at which the last data point from the last patient, which was required for statistical analysis as defined in Data Analysis Plan (DAP), was received.

### 3.2 Number of Subjects / Assignment to Treatment Groups

Approximately 800 patients (400 per treatment arm) will be recruited over a planned recruitment period of 16 months.

Patients will be randomized in 2 groups in a 1:1 ratio. An independent Interactive Voice and Web Response System (IxRS) provider will conduct randomization and hold the treatment assignment code. Patients will be stratified by region (US vs. ROW) and baseline EDSS ($< 4$ and $\geq 4$).

### 3.3 Centers

This will be a multicenter, international study. It is anticipated that approximately 220 centers worldwide will participate.

## 4. STUDY POPULATION

Under no circumstances are patients who enroll in this study permitted to be re-randomized to this study and enrolled for a second course of treatment.

### 4.1 Overview

Adult patients with relapsing MS who fulfill the eligibility criteria specified in Sections 4.2 and 4.3 are eligible for enrollment into the study.

#### 4.1.1 Recruitment Procedures

Patients will be identified for potential recruitment using pre-screening enrollment logs and pre-ID website.

Patients who are candidates for enrollment into the study will be evaluated for eligibility by the investigator to ensure they fulfill eligibility criteria (please refer to Sections 4.2 and 4.3).

All patients must sign the informed consent form prior to screening and prior to any changes to their existing medication for the purposes of enrollment into the trial.

No patient may begin treatment prior to randomization and assignment of a medication number. Under no circumstances are patients who enroll in this study and who have completed treatment as specified, permitted to be re-randomized to this study.

The investigators will be notified by the Sponsor if the study is placed on clinical hold and when the study is completed or closed to further patient enrollment.

No replacement for patients who withdraw from the study after randomization is planned.

## 4.2 Inclusion Criteria

1. Ability to provide written, informed consent and to be compliant with the schedule of protocol assessments.
2. Ages 18-55 years at screening, inclusive.
3. Diagnosis of MS, in accordance with the revised McDonald criteria (2010).
4. At least 2 documented clinical attacks within the last 2 years prior to screening, or one clinical attack in the year prior to screening (but not within 30 days prior to screening).
5. Neurological stability for ≥ 30 days prior to both screening and baseline.
6. EDSS, at screening, from 0 to 5.5 inclusive.
7. Documented MRI of brain with abnormalities consistent with MS prior to screening.
8. Patients of reproductive potential must use reliable means of contraception as described below as a minimum (adherence to local requirements, if more stringent, is required*):
   - Two methods of contraception throughout the trial, including the active treatment phase AND for 48 weeks after the last dose of ocrelizumab, or until their B-cells have repleted, whichever is longer. Acceptable methods of contraception include one primary (e.g., systemic hormonal contraception or tubal ligation of the female partner, vasectomy of the male partner) AND one secondary barrier method (e.g., latex condoms, spermicide) OR a double barrier method (e.g., latex condom, intrauterine device, vaginal ring or pessary plus spermicide [e.g,. foam, vaginal suppository, gel, cream]).

9. For patients of non reproductive potential (adherence to local requirements, if more stringent, is required*):
   - Women may be enrolled if postmenopausal (i.e., spontaneous amenorrhea for the past year confirmed by an FSH level greater than 40 mIU/mL) unless the patient is receiving a hormonal therapy for their menopause or surgically sterile (i.e., hysterectomy, complete bilateral oophorectomy);
   - Men may be enrolled if they are surgically sterile (castration).

* Based on local Ethics Committees or National Competent Authority feedback, additional requirements to assure contraception or to confirm menopause may be required (e.g. serum estradiol compatible with post-menopause status, longer duration of amenorrhea, higher level of FSH).

## 4.3 Exclusion Criteria

Patients who meet the following criteria must be excluded from study entry:

1. Diagnosis of primary progressive MS.
2. Disease duration of more than 10 years in patients with an EDSS ≤ 2.0 at screening.

3. Inability to complete an MRI (contraindications for MRI include but are not restricted to claustrophobia, weight ≥ 140 kg, pacemaker, cochlear implants, presence of foreign substances in the eye, intracranial vascular clips, surgery within 6 weeks of entry into the study, coronary stent implanted within 8 weeks prior to the time of the intended MRI, etc).
4. Known presence of other neurological disorders which may mimic MS including but not limited to: neuromeylitis optica, Lyme disease, untreated vitamin B12 deficiency, neurosarcoidosis and cerebrovascular disorders.

**<u>Exclusions Related to General Health</u>**

5. Pregnancy or lactation.
6. Any concomitant disease that may require chronic treatment with systemic corticosteroids or immunosuppressants during the course of the study.
7. History or currently active primary or secondary immunodeficiency.
8. Lack of peripheral venous access.
9. History of severe allergic or anaphylactic reactions to humanized or murine monoclonal antibodies.
10. Significant or uncontrolled somatic disease or any other significant disease that may preclude patient from participating in the study.
11. Congestive heart failure (NYHA III or IV functional severity).
12. Known active bacterial, viral, fungal, mycobacterial infection or other infection, excluding fungal infection of nail beds.
13. Infection requiring hospitalization or treatment with i.v. antibiotics within 4 weeks prior to baseline visit or oral antibiotics within 2 weeks prior to baseline visit.
14. History or known presence of recurrent or chronic infection (e.g., hepatitis B or C, HIV, syphilis, tuberculosis).
15. History of progressive multifocal leukoencephalopathy (PML)
16. History of malignancy, including solid tumors and hematological malignancies, except basal cell carcinoma, *in situ* squamous cell carcinoma of the skin, and *in situ* carcinoma of the cervix of the uterus that have been previously completely excised with documented, clear margins.
17. History of alcohol or drug abuse within 24 weeks prior to baseline.
18. History or laboratory evidence of coagulation disorders.

**<u>Exclusions Related to Medications</u>***

19. Receipt of a live vaccine within 6 weeks prior to baseline.
    *In rare cases when patient requires vaccination with a live vaccine, the screening period may be extended but cannot exceed 8 weeks.*
20. Treatment with any investigational agent within 24 weeks of screening (Visit 1) or five half-lives of the investigational drug (whichever is longer).
21. Contraindications to or intolerance of oral or i.v. corticosteroids, including methylprednisolone administered i.v., according to the country label, including:
    a) Psychosis not yet controlled by a treatment;
    b) Hypersensitivity to any of the constituents.

22. Contraindication to Rebif® or incompatibility with Rebif® use, including:
    a) Current severe depression and/or suicidal ideation;
    b) Hypersensitivity to natural or recombinant interferon-β, or to any excipients;
    c) Previous suboptimal response to High Dose High Frequency (HDHF) interferon or cessation of HDHF interferon therapy due to poor tolerability;
    d) Prior cessation of Rebif® therapy due to toxicity, which is likely to recur.

23. Treatment with β interferons (with exemptions for HDHF interferon as listed above), glatiramer acetate, plasmapheresis, or other immunomodulatory therapies within 4 weeks prior to baseline.
24. Treatment with dalfamipridine (Ampyra®) unless on stable dose for ≥ 30 days prior to screening. Patients should remain on stable doses throughout the 96 week treatment period.
25. Previous treatment with B-cell targeted therapies (i.e. rituximab, ocrelizumab, atacicept, belimumab or ofatumumab).
26. Systemic corticosteroid therapy within 4 weeks prior to screening.**
27. Any previous treatment with alemtuzumab (Campath), anti-CD4, cladribine, mitoxantrone, daclizumab, BG12, teriflunomide, laquinimod, total body irradiation or bone marrow transplantation.
28. Treatment with cyclophosphamide, azathioprine, mycophenolate mofetil (MMF), cyclosporine, methotrexate, or natalizumab within 24 months prior to screening. NB P*atients previously treated with natalizumab will be eligible for this study only if duration of treatment with natalizumab was < 1 year.*
29. Treatment with fingolimod (FTY720, Gilenya®) or other S1P receptor modulator (i.e., BAF312) within 24 weeks prior to screening. NB *Only patients with T lymphocyte count ≥ LLN will be eligible for this study.*
30. Treatment with i.v. immunoglobulin within 12 weeks prior to baseline.

** Patients screened for this study should not be withdrawn from therapies for the sole purpose of meeting eligibility for the trial. Patients, who discontinue their current therapy for non-medical reasons, should specifically be informed before deciding to enter the study of their treatment options.*

*** The screening period may be extended (but cannot exceed 8 weeks) for patients who have used systemic corticosteroids for their MS before screening. For a patient to be eligible, systemic corticosteroids should not have been administered also between screening and baseline.*

**Exclusions Related to Laboratory Findings***

31. Positive serum β hCG measured at screening.
32. Positive screening tests for hepatitis B (hepatitis B surface antigen [HBsAg] positive, or positive hepatitis B core antibody [total HBcAb] confirmed by a positive viral deoxyribonucleic acid [DNA] polymerase chain reaction [PCR]) or hepatitis C (HepCAb).
33. Positive rapid plasma reagin (RPR).
34. CD4 count < 300/µL.

35. AST/SGOT or ALT/SGPT ≥ 2.0 Upper Limit of Normal (ULN).
36. Platelet count <100,000/µL (<100 x $10^9$/L).
37. Levels of serum IgG <5.65 g/L.
38. Levels of serum IgM < 0.55 g/L.
39. Total neutrophil count <1.5 x $10^3$/µL.

*<u>*Re-testing before baseline</u>: in rare cases in which the screening laboratory samples are rejected by the central laboratory (example: hemolyzed sample) or the results are not assessable (example: indeterminate) or abnormal, the tests need to be repeated within 4 weeks. The last value before randomization must meet study criteria. In such circumstances, the screening period may need to be prolonged but should not exceed 8 weeks.*

<u>Please note:</u> based on local Ethics Committees or National Competent Authority requirements, additional diagnostic testing may be required for selected patients or selected centers to exclude tuberculosis, Lyme disease, HTLV-1 associated myelopathy (HAM), acquired immune deficiency syndrome (AIDS), hereditary disorders, connective tissue disorders, or sarcoidosis. Other specific diagnostic tests may be requested when deemed necessary by the investigator.

## 4.4 Concomitant Medication and Treatment

### 4.4.1 Definition of Concomitant Treatment

A <u>concomitant medication</u> is any drug or substance taken during the study, including the screening period. Over the counter medications and preventative vaccines received during the study are considered concomitant medications.

A <u>concomitant procedure</u> is any therapeutic or elective intervention (e.g. surgery, biopsy) or diagnostic evaluation (e.g. blood gas measurements, bacterial cultures) performed during the study, including the screening period.

Concomitant medications and procedures will be reported at each visit in the relevant form of eCRFs starting from the baseline visit (including medication and procedures taken between screening and baseline). Medications taken for the treatment of multiple sclerosis in the 2-year period prior to the baseline visit and medications taken for the symptoms of multiple sclerosis in the 3 month period prior to the baseline visit will be recorded at the baseline visit. Additionally, medications and medical/surgical procedures administered for any non-MS condition within 12 months prior to the baseline visit will also be recorded at the baseline visit.

### 4.4.2 Treatment for Symptoms of MS

The Treating Investigator should attempt to maintain therapies or treatments for symptoms related to MS (e.g., walking ability, spasticity, incontinence, pain, fatigue) reasonably constant throughout the study.

**Treatment of relapses:** patients who experience a relapse during the treatment period may receive treatment with i.v. (methylprednisolone) or oral corticosteroids, if judged to be clinically appropriate by the investigator. The following standardized treatment regimen should be used as warranted, 1 g i.v. methylprednisolone per day for a maximum

of 5 consecutive days. In addition, at the discretion of the investigator, corticosteroids may be stopped abruptly or tapered over a maximum of 10 days. Such patients should not discontinue the treatment period solely based on the occurrence of a relapse, unless the patient or investigator feels he or she has met the criteria for withdrawal (See Section 4.5 for further details).

#### 4.4.2.1 Prohibited Concomitant Treatments

Therapies for MS noted in the exclusion criteria under "Exclusions Related to Medications" (Section 4.3) are not permitted during the study treatment period with the exception of systemic corticosteroids for the treatment of a relapse.

After patients have finished the treatment with ocrelizumab, they may receive alternative treatment for their MS as judged clinically appropriate by the Treating Investigator. However treatment with immunosuppressants, lymphocyte depleting agents, or lymphocyte trafficking blockers is not allowed while patients remain B-cell depleted due to the potential for increased risk of infection.

### 4.4.3 Immunization

No formal vaccination study has been conducted in ocrelizumab-treated patients. Results from studies done with a similar monoclonal antibody, rituximab, which provide additional information on the impact of anti-CD20 antibodies on the response to vaccinations, are presented below.

A small study comparing responses to influenza vaccination among RA patients receiving rituximab or tumor necrosis factor (TNF) inhibitors vs. normal controls found significantly lower post vaccination titres and protection rates (the proportion of a group with a titre $\geq 40$) in rituximab-treated patients compared to both control groups [44]. Results from another study are in line with the ones previously stated. This study showed that RA patients treated with rituximab compared with RA patients receiving methotrexate (MTX) and healthy adults had a severely hampered humoral immune response to influenza vaccine. This response remained reduced 6-10 months after rituximab treatment. In the rituximab group, patients who had been previously vaccinated achieved higher anti-influenza titers following influenza vaccination [45].

Another study assessed vaccine responses to influenza vaccine (containing 15 µg hemagglutinin/dose of B/Shanghai/361/02 (SHAN), A/New Caledonia 20/99 (NC) (H1N1) and A/California/7/04 (CAL) (H3N2)) among RA patients treated with disease-modifying anti-rheumatic drugs (DMARDs) with or without rituximab and normal control. After 4 weeks post vaccination geometric mean titers increased for New Caledonia and California antigens in all subjects, but not for the Shanghai antigen in the rituximab group. In addition, in rituximab treated patients, the percentage of responders was low for all three antigens tested, achieving statistical significance for California antigen [46].

In a randomized study with rituximab, patients with RA had comparable responses to tetanus recall antigen (39% vs. 42%), reduced responses to pneumococcal polysaccharide vaccine (43% vs. 82% to at least two pneumococcal antibody serotypes) and to Keyhole Limpet Haemocyanin (KLH) neoantigen (47% vs. 93%), when given 6 months after

rituximab as compared with patients only receiving methotrexate (MTX) [47]. Because of the mechanism of action, it is expected that similar findings would apply to ocrelizumab and patients treated with ocrelizumab may experience lower response rates to non-live vaccines than the general population.

Physicians are advised to review the immunization status of patients being considered for treatment with ocrelizumab and follow local/national guidance for adult vaccination against infectious disease. Known dates of immunizations will be recorded on specific eCRF pages. **Immunizations should be completed at least 6 weeks prior to first administration of ocrelizumab.**

Patients requiring de novo hepatitis B vaccination (which involves three separate doses of vaccine) should also have completed the course at least 6 weeks prior to the first infusion of study drug.

The safety of immunization with live viral vaccines following ocrelizumab or rituximab therapy has not been studied. Immunization with any live or live-attenuated vaccine (i.e. measles, mumps, rubella, oral polio vaccine, Bacille Calmette-Guerin (BCG), typhoid, yellow fever, vaccinia, cold adapted live influenza strain vaccine or any other vaccines not yet licensed but belonging to this category) is not recommended within 6 weeks of first dosing (see exclusion criteria), during ocrelizumab treatment and for as long as the patient is B-cell depleted.

## 4.5 Criteria for Premature Withdrawal

Patients have the right to withdraw from the study at any time for any reason.

**Patients must be withdrawn from treatment under the following circumstances:**

– Patients with Grade 4 infusion reaction, or severe allergic or anaphylactic reaction to an ocrelizumab infusion;

– If a woman becomes pregnant and chooses to carry her pregnancy during the study;

– Patients who demonstrate active hepatitis B or C infection, either new onset or reactivation in the case of hepatitis B;

– Patients who demonstrate active tuberculosis, either new onset or reactivation;

– Patients with PML;

– Patients with elevation of ALT ≥ 10 x ULN, jaundice or other clinical symptoms of liver dysfunction;

– Patients with persisting elevation of ALT > 3x ULN, or other clinical symptoms of liver dysfunction that did not resolve with Rebif®/Rebif® placebo dose modification (please refer to Section 6.2.2 for more details;

– Patients who decide to discontinue the treatment;

– The patient's Treating Investigator decides that discontinuation of treatment is in the best clinical interest of the patient.

Patients who withdraw from study treatment for any reason should complete the Safety Follow up Period. If the patient insists on discontinuing the study, he/she should be asked if he/she can still be contacted for further information. The outcome of that discussion should be documented in both the medical records and in the eCRF. If lost to follow-up, the investigator should contact the patient or a responsible relative by telephone followed by registered mail or through a personal visit to establish as completely as possible the reason for the withdrawal. A complete final evaluation at the time of the patient's withdrawal should be made with an explanation of why the patient is withdrawing from the study.

When applicable, patients should be informed of circumstances under which their participation may be terminated by the investigator without the patient's consent. The investigator may withdraw patients from the study in the event of intercurrent illness, adverse events, treatment failure, after a prescribed procedure, lack of compliance with the study and/or study procedures (e.g., dosing instructions, study visits), cure or any reason where it is felt by the investigator that it is in the best interest of the patient to be terminated from the study. Any administrative or other reasons for withdrawal must be documented and explained to the patient. If the reason for removal of a patient from the study is an adverse event, the principal specific event will be recorded on the eCRF. If possible, the patient should be followed until the adverse event has resolved.

An excessive rate of withdrawals can render the study non-interpretable; therefore, unnecessary withdrawal of patients should be avoided. Should a patient decide to withdraw, all efforts will be made to complete and report the observations prior to withdrawal as thoroughly as possible.

Please note: It is important to distinguish between "withdrawal from treatment" and "withdrawal from study". Patients who withdraw from treatment should be encouraged to remain in the study for the full duration of the Safety Follow Up Period (minimum of 48 weeks following the last infusion).

It should be noted that upon withdrawal from the study, any untested routine samples will be destroyed. However, information already obtained from samples up until the time of withdrawal will be used.

### 4.5.1 Withdrawal of Subjects from the Roche Clinical Repository (RCR)

Subjects who gave consent to provide RCR specimens have the right to withdraw their specimen from the RCR at any time for any reason. If a patient wishes to withdraw his/her consent to the testing of his/her specimen(s), the investigator must inform Roche in writing of the patient's wishes using the RCR Subject Withdrawal Form and enter the date of withdrawal in the patient's electronic Case Report Form (eCRF). A patient's withdrawal from the main trial does not, by itself, constitute withdrawal of the specimen from the RCR; likewise, a patient's withdrawal from the RCR does not constitute a withdrawal from the main trial.

### 4.5.2 Patient Agreement for Continuation in the Study (in case of sustained disability progression)

During the treatment period, in the event of sustained disability progression on EDSS confirmed for 24-weeks, the benefits and risks of study treatment should be reassessed with the patient prior to any further dosing, including a discussion of alternative treatment options available for that patient. The result of this discussion must be included in the patient's file, prior to any further dosing of study medication.

If, after the discussion, the patient decides not to continue with the study treatment, they should be discontinued from any further treatment, complete applicable Withdrawal from Treatment Visit procedures and be entered into the Safety Follow Up Period.

For definition of sustained disease progression, please refer to Section 5.3.2.2.

## 4.6 Replacement Policy (Ensuring Adequate Numbers of Evaluable Subjects)

### 4.6.1 For Subjects

Patients prematurely discontinued from the study for any reason will not be replaced.

### 4.6.2 For Centers

A center may be replaced for the following administrative reasons:

- Excessively slow recruitment.
- Poor protocol adherence.
- Sponsor's discretion (Sponsor refers to F. Hoffmann-La Roche Ltd and Genentech, Inc.)

# 5. SCHEDULE OF ASSESSMENT AND PROCEDURES

## Table 3: Schedule of Assessments: Screening Through the End of Double-Blind Treatment Period

| | Screen | Treatment Period | | | | | | | | | | | | |
|---|---|---|---|---|---|---|---|---|---|---|---|---|---|---|
| **Visit** | 1 | 2 BL | 3 | 4 | 5 | 6 | 7 | 8 | 9 | 10 | 11 | Delayed Dosing Visit [22] | Unscheduled Visit [23] | Withdrawal from Treatment Visit |
| **Week** | -2 | - | w2 | w12 | w24 | w36 | w48 | w60 | w72 | w84 | w96 | | | |
| *Study Day (window in days)* | *-14* | *1* | *15 (± 1)* | *85 (±4)* | *169 (±1)* | *253 (±4)* | *337 (±1)* | *421 (±4)* | *505 (±1)* | *589 (±4)* | *673 (±1)* | | | |
| | | | | | | | | | | | | | | |
| Informed consent [1] | x | | | | | | | | | | | | | |
| Medical history | x | | | | | | | | | | | | | |
| Review of eligibility criteria | x | x | | | | | | | | | | | | |
| CES-D, MFIS, EQ-5D, SF-36 | | x | | | | | x | | | | x | | | x |
| Patient's Assessment of Treatment Benefit | | | | | | | x | | | | x | | | x |
| C-SSRS | | x | | x | x | x | x | x | x | x | x | | x | x |
| Physical examination | x | x | x | | x | | x | | x | | x | x | | x |
| Vital signs [2] | x | x | x | x | x | x | x | x | x | x | x | x | x | x |
| 12 lead ECG (pre- and post-dose) [3] | x | x | | | | | | | x | | | | | x |
| Height | x | | | | | | | | | | | | | |
| Weight | x | | | | | | | | x | | x | | | x |
| Neurological exam and EDSS | x | x | | x | x | x | x | x | x | x | x | | x | x |
| MSFCS, LCVA, SDMT | | x | | x | x | x | x | x | x | x | x | | x | x |
| Karnofsky Performance Status Scale | | x | | | x | | x | | x | | x | | | x |
| MRI[4] | | x | | | x | | x | | | | x | | | x |
| Concomitant Treatment | | x | x | x | x | x | x | x | x | x | x | x | x | x |
| Adverse Events | *Only SAEs* | x | x | x | x | x | x | x | x | x | x | x | x | x |
| Potential relapses recorded | | x | x | x | x | x | x | x | x | x | x | x | x | x |
| Telephone interview (every 4 wks)[5] | x | | | – – – | – – – | – – – | – – – | – – – | – – – | – – – | – – → | | | x |

CONFIDENTIAL *Roche Protocol WA21092B (RO4964913) - Page 67*

## Table 3: Schedule of Assessments: Screening Through the End of Double-Blind Treatment Period (Cont.)

| | Screen | Treatment Period | | | | | | | | | | | | |
|---|---|---|---|---|---|---|---|---|---|---|---|---|---|---|
| **Visit** | **1** | **2 BL** | **3** | **4** | **5** | **6** | **7** | **8** | **9** | **10** | **11** | **Delayed Dosing Visit** [22] | **Unscheduled Visit** [23] | **Withdrawal from Treatment Visit** |
| **Week** | **-2** | **-** | **w2** | **w12** | **w24** | **w36** | **w48** | **w60** | **w72** | **w84** | **w96** | | | |
| *Study Day (window in days)* | *-14* | *1* | *15 (± 1)* | *85 (±4)* | *169 (±1)* | *253 (±4)* | *337 (±1)* | *421 (±4)* | *505 (±1)* | *589 (±4)* | *673 (±1)* | | | |
| | | | | | | | | | | | | | | |
| Pregnancy test [6] | x | x | x | x | x | x | x | x | x | x | x | x | | x |
| Antibody Titers [7] | | x | | x | x | | x | | x | | x | | | x |
| RCR (non-DNA) [8] | | x | | x | x | | x | | x | | x | | | x |
| RCR (DNA) [9] | | x | | | | | | | | | | | | |
| Protein biomarker sampling [10] | | x | | x | x | | x | | x | | x | | | x |
| HAHA [11] | | x | | | x | | x | | x | | x | | | x |
| Plasma/ urine banking for JCV[12] | | x | | x | x | x | x | x | x | x | x | | | x |
| PK Samples [13] | | x | | | x | | x | | x[13] | x | x | | | x |
| Thyroid function tests [14] | x | | | | x | | x | | x | | x | | | x |
| FSH [15] | x | | | | | | | | | | | | | |
| Hepatitis Screening [16] | x | | | | | | | | | | | | | |
| Hepatitis B virus DNA[16] | x | (x) | | (x) | (x) | (x) | (x) | (x) | (x) | (x) | (x) | | | (x) |
| RPR | x | | | | | | | | | | | | | |
| CD4 count | x | | | x | | x | | x | | x | | | | |
| IgG | | | | x | | x | | x | | x | | | | |
| Total Ig, IgA, IgG, IgM | x | | | | x | | x | | x | | x | | | x |
| FACS [17] | | x | x | x | x | | x | | x | | x | | | x |
| Routine safety lab [18] | x | x | x | x | x | x | x | x | x | x | x | | | x |
| Type I interferon neutralizing antibody assay [19] | | x | | | x | | x | | x | | x | | | x |

## Table 3: Schedule of Assessments: Screening Through the End of Double-Blind Treatment Period (Cont.)

| | Screen | Treatment Period | | | | | | | | | | | | |
|---|---|---|---|---|---|---|---|---|---|---|---|---|---|---|
| **Visit** | 1 | 2 BL | 3 | 4 | 5 | 6 | 7 | 8 | 9 | 10 | 11 | Delayed Dosing Visit [22] | Unscheduled Visit [23] | Withdrawal from Treatment Visit |
| **Week** | -2 | - | w2 | w12 | w24 | w36 | w48 | w60 | w72 | w84 | w96 | | | |
| *Study Day (window in days)* | -14 | 1 | 15 (± 1) | 85 (±4) | 169 (±1) | 253 (±4) | 337 (±1) | 421 (±4) | 505 (±1) | 589 (±4) | 673 (±1) | | | |
| Pre-treatment with i.v. methylprednisolone [20] | | x | x | | x | | x | | x | | | x | | |
| Administration of i.v. ocrelizumab / ocrelizumab placebo [21] | | x | x | | x | | x | | x | | | x | | |
| Assessment of s.c. Rebif®/ Rebif® placebo compliance | | x | x | x | x | x | x | x | x | x | x | | x | x |
| Administration of s.c. Rebif® / Rebif® placebo 3x/wk | | x | x | x | x | x | x | x | x | x | | | | |

1. **Informed Consent** must be obtained in written form from all patients at screening (prior to any study–related procedure) in order to meet eligibility for the study.
2. **Vital signs** (i.e., pulse rate, systolic and diastolic blood pressure, respiration rate and temperature) will be obtained while the patient is in the semi supine position (after 5 minutes). On infusion visits, the vital signs should be taken within 45 minutes prior to the methylprednisolone infusion in all patients. In addition, vital signs should be obtained prior to ocrelizumab/ocrelizumab placebo infusion, then every 15 minutes (± 5 minutes) for the first hour; then every 30 minutes (± 10 minutes) until 1 hour after the end of the infusion. On non-infusion days, the vital signs may be taken at any time during the visit.
3. **ECG (pre- and post-dose):** on infusion visits ECG should be taken within 45 minutes prior to the methylprednisolone infusion in all patients, and within 60 minutes after completion of the ocrelizumab/ocrelizumab placebo infusion. On non-infusion days, the ECG may be taken at any time during the visit.
4. **MRI**: brain MRI scans will be obtained in patients withdrawn from the treatment period (at a withdrawal visit) if not performed during last 4 weeks.

**Table 3: Schedule of Assessments: Screening Through the End of Double-Blind Treatment Period (Cont.)**

5. **A structured telephone interview** will be conducted by site personnel every 4 weeks (± 3 days) from Week 8 through the study to identify any new or worsening neurological symptoms that warrant an unscheduled visit and collect data on possible events of infections.
6. **Serum β-hCG** must be performed at screening in women of childbearing potential. Subsequently, urine β-hCG [sensitivity of at least 25 mIU/mL] will be performed. On infusion visits, the urine pregnancy test should be performed prior to methylprednisolone infusion in all women of child-bearing potential. If positive, the patient will not receive the scheduled dose and confirmation, a serum pregnancy test, will be performed.
7. **Antibody Titers**: measurement of antibody titers against common antigens (mumps, rubella, varicella and Streptococcus pneumoniae) will be performed.
8. **RCR - Roche Clinical Repository non-DNA (RNA – and protein):** for RNA 2x 2.5 ml whole blood samples to be taken from consenting patients only for expression profiling analysis. For protein: 6 ml blood samples in EDTA tube for plasma samples will be taken from consenting patients only for analysis of protein biomarkers. On infusion visits, ALL samples should be taken 5-30 minutes prior to methylprednisolone infusion.
9. **RCR - Roche Clinical Repository (DNA):** 6 ml whole blood sample to be taken from only from patients consenting to RCR for pharmacogenetic and genetic analysis. If not done at Baseline (Visit 2), sample may be collected at next visit.
10. **Protein biomarker sampling:** one serum sample (6 ml) will be taken from all patients for analysis of protein biomarkers. On infusion visits, samples should be taken 5-30 minutes prior to methylprednisolone infusion.
11. **HAHA**: On infusion visits, serum samples are collected 5-30 minutes prior to the methylprednisolone infusion.
12. **Plasma and urine samples for JCV will be collected** at specified time points and analyzed in batches.
13. **PK samples:** on the infusion day at week 72, two serum samples should be collected, one 5-30 minutes prior to the methylprednisolone infusion and the second one 30 minutes (±10 minutes) following the completion of the ocrelizumab/ocrelizumab placebo infusion. For all other infusion visits, a blood sample should be taken 5 – 30 minutes before the methylprednisolone infusion. At other times (non-infusion visits) samples may be taken at any time during the visit.
14. **sTSH** will be tested at screening and during double-blind treatment period. Thyroid autoantibodies will be assayed only at screening.
15. **FSH**: only applicable to women to confirm the post-menopausal status.
16. **Hepatitis** screening & monitoring: all patients must have negative HBsAg result and negative HepCAb screening tests prior to enrollment. If total HBcAb is positive at screening, HB virus DNA measured by PCR must be negative to be eligible. For those patients enrolled with negative HBsAg and positive total HBcAb, HB virus DNA (PCR) must be repeated every 12 weeks during the treatment period.

## Table 3: Schedule of Assessments: Screening Through the End of Double-Blind Treatment Period (Cont.)

17. **FACS**: including CD19 and other circulating B-cell subsets, T-cells, natural killer cells and other leukocytes. On infusion visits, blood samples should be collected prior to the infusion of methylprednisolone.
18. **Routine safety lab:** hematology, chemistry and urinalysis: on infusion visits, all urine and blood samples should be collected prior to the infusion of methylprednisolone. At other times, samples may be taken at any time during the visit.
19. **Type I interferon neutralizing antibody assay:** samples should be taken at least 36 hours following last injection of Rebif®/Rebif® placebo.
20. All patients receive **prophylactic treatment** with 100 mg of methylprednisolone i.v. prior to infusion of ocrelizumab /ocrelizumab placebo. It is also recommended that patients receive an analgesic/antipyretic such as acetaminophen/paracetamol (1 g) and an i.v. or oral antihistaminic such as diphenhydramine 50 mg 30-60 minutes prior to ocrelizumab/ ocrelizumab placebo.
21. **Administration (infusion) of i.v. ocrelizumab/ocrelizumab placebo**: the Treating Investigator must review the clinical and laboratory re-treatment criteria prior to subsequent infusion of ocrelizumab/ocrelizumab placebo.
22. **A delayed dosing visit** will be performed and recorded in the Delayed Dosing Visit eCRF form when dosing cannot be administered at the scheduled dosing visit. Other tests or assessments may be done as appropriate.
23. **Unscheduled Visit**: assessments performed at unscheduled (non-dosing) visits will depend on the clinical needs of the patient. All patients with new neurological symptoms suggestive of relapse should have EDSS performed by examining investigator. Other tests/assessments may be done as appropriate. Please note: in case of ALT elevations dose modification should be necessary, additional visits may be required for dispensing of study medication.

Please note: based on local Ethics Committees or National Competent Authority requirements, additional diagnostic testing may be required for selected patients or selected centers to exclude tuberculosis, Lyme disease, HTLV-1 associated myelopathy (HAM), acquired immune deficiency syndrome (AIDS), hereditary disorders, connective tissue disorders, or sarcoidosis. Other specific diagnostic tests may be requested when deemed necessary by the investigator.

**Table 4: Schedule of Assessments: Safety Follow up (including prolonged B-cell monitoring if required)**

| | **Safety Follow up** | ***Prolonged B-cell Monitoring[1]*** | ***End of observation or withdrawal from Safety Follow up*** |
|---|---|---|---|
| **Assessments** | *Visits every 12 weeks (±7 days) [2]* | *Visits every 24 weeks (±7 days)* | |
| Urine pregnancy test | x | x | x |
| Routine Safety Labs[3] | x | x | x |
| FACS [4] | x | x | x |
| Total Ig, IgA, IgG, IgM | x[10] | x | x |
| HAHA[5] | x[10] | x | x |
| Plasma/urine banking for JCV | x | x | x |
| Antibody titers | x[10] | x | x |
| Hepatitis B viral DNA[6] | (x) | (x) | (x) |
| RCR non-DNA [7] | x[10] | x | x |
| Protein biomarker sampling [8] | x[10] | x | x |
| Vital Signs | x | x | x |
| EDSS | x | | x |
| Neurological examination | x | x | x |
| Physical examination | x[10] | x | x |
| Potential relapses recorded | x | x | x |
| Adverse events | x | x | x |
| Concomitant Medication | x | x | x |
| Telephone interview [9] | x | x | |

**Table 4: Schedule of Assessments: Safety Follow up (including prolonged B-cell monitoring if required) (Cont.)**

1. **Prolonged B-cell monitoring:** patients whose B-cells have not been repleted after 48 weeks of Safety Follow up period will continue with visits every 24 weeks (± 7 days) until B-cell repletion.
2. Visits will be performed at 12-week intervals counting from the date of last infusion of ocrelizumab.
3. **Routine safety lab**: hematology, chemistry and urinalysis.
4. **FACS** including CD19 and other circulating B-cell subsets, T cells, natural killer cells and other leukocytes.
5. **HAHA**: two serum samples are required.
6. **Hepatitis monitoring:** hepatitis to be monitored only in patients with screening results of HbsAg negative, HBcAb positive and HBV DNA negative, inclusive.
7. **RCR (Roche Clinical Repository) non-DNA (RNA and protein):** for RNA 2x 2.5 ml whole blood samples to be taken from consenting patients only for expression profiling analysis. For protein 6 ml blood samples in EDTA tube for plasma samples to be taken from consenting patients only for analysis of protein biomarkers.
8. **Protein biomarker sampling**: 6 ml blood sample in a plain tube without EDTA for serum isolation will be taken from all patients for analysis of protein biomarkers.
9. **A structured telephone interview** will be performed by site personnel every 4 weeks (± 3 days) between visits until 48 weeks after the last infusion to identify any new or worsening neurological symptoms that warrant an unscheduled visit and collect data on possible events of infections. If prolonged B-cell monitoring is required beyond 48 weeks after the last infusion, telephone interviews will be done every 12 weeks (± 7 days) between visits.
10. Needs to be assessed only every 24 weeks.

<u>Please note:</u> patients in Safety Follow up who receive other B-cell targeted therapies will only be followed for 48 weeks from the date of the last infusion of the study drug regardless of their B-cell count.

## 5.1 Screening Examination and Eligibility Screening Form

All patients must sign and date the most current Institutional Review Board/Institutional Ethics Committee's (IRB/IEC) approved written informed consent before any study specific assessments or procedures are performed.

Consenting patients will enter the 2-week screening period to be evaluated for eligibility. Please refer to "Schedule of Assessments: Screening through the End of Double-Blind Treatment Period" - Table 3 for details. Patient must fulfill all entry criteria for participation in the study.

*Please note that based on local Ethics Committees or National Competent Authority requirements, additional diagnostic testing may be required for selected patients or selected centers to exclude tuberculosis, Lyme disease, HTLV-1 associated myelopathy (HAM), acquired immune deficiency syndrome (AIDS), hereditary disorders, connective tissue disorders, or sarcoidosis.*

An Eligibility Screening Form [ESF] documenting the investigator's assessment of each screened patient with regard to the protocol's inclusion and exclusion criteria is to be completed by the investigator.

Each patient screened must be registered in the IxRS by the investigator or the investigator's research staff at screening. A screen failure record must be maintained by the investigator, and reasons must be captured in the IxRS.

It should be stated in the medical record that the patient is participating in this clinical study.

## 5.2 Procedures for Enrollment of Eligible Subjects

Once a patient has fulfilled all eligibility criteria, he or she will be randomized via IxRS to one of two treatment groups: ocrelizumab 600 mg (given as 300 mg x 2 14 days apart for the first 24 weeks and 600 mg x 1 every 24 weeks thereafter) or Rebif®.

Patient eligibility information will be provided to the IxRS by the investigator or the investigator's research staff at randomization. The patient will be randomized and assigned a unique treatment box number (medication number) and randomization number. As confirmation, the site will be provided with a verification of each patient's randomization.

The patient randomization numbers will be generated by Roche or its designee and incorporated into the double-blind labeling.

The patient randomization numbers are to be allocated sequentially in the order in which the patients are enrolled according to the specification document agreed with the external randomization company/center.

Treatment with the first study drug infusion should occur within 24 hours of randomization. In exceptional cases where all baseline assessments cannot be completed within 24 hours, the first study drug infusion can be administered within 48 hours of randomization provided that the investigator assures that all inclusion and exclusion

criteria are still met on the day of dosing. In particular, there should be no evidence of an ongoing infection at the time of dosing.

No patient may begin treatment prior to randomization and assignment of a medication number.

## 5.3 Clinical Assessments and Procedures

This is an assessor blinded study. Each site will have two investigators: a principal or Treating Investigator and an Examining Investigator or rater.

- **The Treating Investigator** is the physician responsible for the patient care and should be a neurologist experienced in the care of MS patients. The Treating Investigator will have access to safety and blinded efficacy data and will make treatment decisions based on the patient's clinical response and laboratory findings.
- **The Examining Investigator** should be a neurologist or other health care practitioner and must be trained and certified in administering the *Neurostatus* Functional System Scores (FSS) and Expanded Disability Status Scale (EDSS) examination prior to study start.

The Examining Investigator will perform the neurological examination, document the FSS scores, and assess EDSS scores. The examining investigator will also be responsible for performing and documenting results from the following: MSFCS, the Karnofsky Performance Status Scale, low-contrast visual acuity testing, and the Symbol Digit Modalities Test. They will only have access to data from the assessments listed above. Every effort will be made to ensure that there is no change in the EDSS rater throughout the course of the study for any individual patient. Whenever possible, the same person should perform the examination for the full study duration.

All efforts should be made to keep the Examining Investigator blinded to the treatment assignment. Patients will be instructed not to discuss any symptoms related to the study treatment with the Examining Investigator; the Examining Investigator should remind the patient at the start of the examination. In view of the extended duration of this study, each site will identify a primary and back-up for Treating and Examining Investigator. **The Treating Investigator and the Examining Investigator will not be allowed to switch roles**.

### 5.3.1 Overview of Clinical Visits

After the screening visit, patients fulfilling the entry criteria will be scheduled for the baseline assessments. Randomization will occur only after the patient meets all inclusion and exclusion criteria on Day 1. Visits will take place as described in the Schedule of Assessments.

Visits should be scheduled in relation to the baseline visit (Day 1) A minimum of 22 weeks should occur between infusions. Patients should not receive their infusions within a shorter interval. Patients who cannot receive their infusion at the visit, should be re-scheduled for a delayed dosing visit – see Section 5.3.1.1.

At infusion visits patients treated with ocrelizumab should remain in observation for at least 1 hour after the completion of the infusion.

Patients who cannot receive their infusion at the visit, should be re-scheduled for a delayed dosing visit. Additional unscheduled visits for the assessment of potential relapses, new neurological symptoms, safety events or for dispensing Rebif®/Rebif® placebo if down titration is needed may occur at any time.

#### *5.3.1.1 Delayed Dosing Visit*

Delayed dosing visits may be scheduled only if the infusion cannot be administered at the time points defined in Schedule of Assessments - Table 3. Thus, a patient who had all assessments of a dosing visit performed, but could not receive his/her infusion, should be re-scheduled for the infusion.

**If the delayed infusion is the first infusion of the first treatment cycle (Day 1), then the visit for the second infusion should be scheduled 14 days after the delayed first infusion (± 1 day). In the event any subsequent infusion needs to be delayed, at least a 22-week period still must be maintained between one infusion and the next.**

At the delayed dosing visit, additional tests or assessments, such as routine safety laboratory tests, may be performed when the investigator judges that these are warranted.

#### *5.3.1.2 Unscheduled Visits*

Patients developing new or worsening neurological symptoms should be seen at the investigational site as soon as possible regardless of the treatment group to which they were randomized, regardless of the dates of their pre-planned, scheduled study visits, and regardless of the study period. Assessments performed at unscheduled (non-dosing) visits will depend on the clinical needs of the patient.

Patients with new neurological symptoms suggestive of relapse should have an EDSS performed by Examining Investigator. Other tests/assessments may be done as appropriate. Please note: should the Rebif®/Rebif® placebo dose modification be necessary in case of ALT elevations, unscheduled visits may be required for dispensing of study medication.

Please refer also to Section 7.3.4.1 for guidance on the diagnosis of progressive multifocal leukoencephalopathy (PML).

#### *5.3.1.3 Withdrawal Visits*

At the moment a patient meets one or more of the withdrawal criteria (Section 4.5), this patient is regarded withdrawn from treatment. Patient who withdraw from ocrelizumab treatment will need to complete all assessments as shown in Schedule of Assessments and will enter the Safety Follow-up.

Please note: at the Withdrawal from Double-blind Treatment Period Visit, an MRI scan will be required only if not performed in the prior 4 weeks.

### 5.3.2 Assessment of Efficacy

#### *5.3.2.1 Assessment of Relapse*

All new or worsening neurological events consistent with MS representing a clinical relapse are to be reported on the dedicated page of eCRF. Patients with clinical relapses should be referred to the Examining Investigator who will assess the FSS/EDSS independently to allow confirmation as to whether or not the clinical relapse(s) meet the criteria for protocol-defined relapse(s).

**Protocol-defined relapse** is the occurrence of new or worsening neurological symptoms attributable to MS. Symptoms must persist for >24 hours and should not be attributable to confounding clinical factors (e.g. fever, infection, injury, adverse reactions to medications) and immediately preceded by a stable or improving neurological state for at least 30 days. The new or worsening neurological symptoms must be accompanied by objective neurological worsening consistent with an increase of at least half a step on the EDSS scale, or 2 points on one of the appropriate FSS, or 1 point on two or more of the appropriate FSS. The change must affect the selected FSS (i.e., pyramidal, ambulation, cerebellar, brainstem, sensory, or visual). Episodic spasms, sexual dysfunction, fatigue, mood change, or bladder or bowel urgency or incontinence will not suffice to establish a relapse. NB: Sexual dysfunction and Fatigue will not be scored. Please note: adjudication of protocol-defined relapses will be performed by the Sponsor based on pre-specified criteria, applied to data collected by investigator, in a blinded fashion.

**All patients with new neurological symptoms suggestive of a relapse should be referred to the Examining Investigator for EDSS assessment. Any patient, complaining of a neurological symptom, defined at a visit or over the phone, should be referred to the Examining Investigator unless the Treating Investigator determines that the symptom is due to mitigating circumstances (such as an intensification of neurological symptoms from a transient systemic infection).**

Please note: clinical relapses (i.e., regardless of whether they meet criteria for a protocol-defined relapse) will be recorded on a pre-specified eCRF “MS relapse” eform. MS relapses should not be reported on Adverse Event eform of eCRF.

#### *5.3.2.2 Assessment of Disability*

**Disability progression** has been defined as an increase of ≥ 1.0 point from the baseline EDSS score that is not attributable to another etiology (e.g. fever, concurrent illness, or concomitant medication) when the baseline score is 5.5 or less, and ≥ 0.5 when the baseline score is above 5.5. Disability progression is considered sustained when the increase in the EDSS is confirmed at a regularly scheduled visit at least 12 weeks or 24 weeks, after the initial documentation of neurological worsening.

Sustained disability progression, confirmed for both 12 and 24 weeks, after the initial documentation of neurological worsening, will be analyzed as key secondary endpoints.

#### *5.3.2.3 Kurtzke Expanded Disability Status Scale (EDSS)*

The EDSS is based on a standard neurological examination, incorporating the following functional systems (pyramidal, cerebellar, brainstem, sensory, bowel and bladder, visual,

and cerebral [or mental]) and ambulation rated and scored as functional system scores (FSS). Each FSS is an ordinal clinical rating scale ranging from 0 to 5 or 6. These ratings are then used in conjunction with observations and information concerning ambulation and use of assistive devices to determine the EDSS score. The EDSS is a disability scale that ranges in 0.5-point steps from 0 (normal) to 10 (death) [48].

The EDSS will be assessed by the Examining Investigator. **All patients with new neurological symptoms suggestive of relapse should have EDSS performed during an unscheduled visit.**

#### *5.3.2.4 The Multiple Sclerosis Functional Composite Scale (MSFCS)*

The Multiple Sclerosis Functional Composite Scale (MSFCS) consists of three subscales, including the 9-Hole Peg Test, Paced Auditory Serial Addition Test (PASAT), and Timed 25-Foot Walk (25-TW), which provide a global quantitative estimate of MS disease progression [49].

The MFSCS will be performed by the Examining Investigator who must remain blinded to the treatment assignment.

#### *5.3.2.5 Low-Contrast Visual Acuity (LCVA) Testing*

Low-contrast letter acuity charts (Sloan charts) have gained validity in the assessment of visual dysfunction in patients with MS not readily apparent on commonly used high-contrast acuity tests. Reductions in low-contrast letter acuity are associated with MS and correlate with increasing disability, MRI abnormalities, and reduced retinal nerve fiber layer (RNFL) thickness as measured by optical coherence tomography (OCT).

LCVA testing will be performed using low contrast letter acuity charts (low contrast Sloan letter charts) by the examining investigator at the timepoints indicated in the Schedule of Assessments - Table 3.

#### *5.3.2.6 The Symbol Digit Modalities Test (SDMT)*

The SDMT has demonstrated sensitivity in detecting not only the presence of cognitive impairment, but also changes in cognitive functioning over time and in response to treatment. The SDMT is brief, easy to administer, and involves a simple substitution task that normal children and adults can easily perform. Using a reference key, the examinee has 90 seconds to pair specific numbers with given geometric figures. Responses can be written or oral, and for either response mode, administration time is just 5 minutes.

SDMT will be administered by the examining investigator at the timepoints indicated in the Schedule of Assessments - Table 3.

### 5.3.3 Brain MRI Imaging

Magnetic resonance imaging (MRI) is a useful tool for monitoring CNS lesions in MS. Different MRI derived parameters have been related to clinical activity and T1 weighted gadolinium-enhancing lesions or new and/or enlarging hyperintense T2 lesions have been related to relapses. It is hypothesized that changes in brain volume may reflect brain atrophy as a result of MS-related tissue loss and may thereby correlate with long-term clinical outcome in these patients.

Brain MRI scans will be obtained in all patients as detailed in the Schedule of Assessments - Table 3. In addition, brain MRI scans will be obtained in patients withdrawn from the double-blind phase of treatment period (at the withdrawal visit) if not performed during the previous 4 weeks.

Scans will be performed by trained and certified MRI technicians. **The following time windows apply:**

- **"Baseline" MRI should be performed after screening visit, but at least 10 days prior to the baseline visit.**
- **MRI at visits scheduled at Weeks: 24, 48, 96 or at withdrawal visit (if applicable) should be performed within 4 weeks of the scheduled visit.**

If patients receive corticosteroids for a relapse, every effort should be made to obtain the scan prior to the first steroid dose if the pre-steroid scan is within 1 week of the scheduled visit. In patients receiving corticosteroids, there should be an interval of 3 weeks between the last dose of corticosteroids and the scan.

The MRI will include the acquisition of scans at each time point with and without intravenously administered gadolinium contrast enhancement.

MRI scans will be read by a centralized reading center for both efficacy and safety endpoints. The centralized reading center is blinded to the treatment assignment and the reading is performed in the absence of clinical information. Further details on scanning acquisition sequences, methods, handling and transmission of the scans, certification of site MRI radiologist/technicians, and the procedures for the blinded analysis of the scans at the central reading center are described in a separate MRI Acquisition Procedures Manual.

All MRI scans will also be reviewed locally by a radiologist for safety and the MRI scan report containing only non-MS pathology will be provided to the Treating Investigator (see Section 5.3 for definition). At the investigational site, only the local radiologist/technician assigned to this study may have access to the MRI scans; the Treating Investigator should not review the MRI scans unless a safety concern arises. In the event that the Treating Investigator does become aware of the MRI results, this should be documented in the eCRF, indicating the reason.

#### 5.3.4 Safety

Adverse events, vital signs, weight, physical and neurological examination, clinical laboratory tests (including pregnancy tests), 12 lead ECG, locally reviewed MRI for safety (non MS CNS pathology), and data on concomitant medications and diseases will be collected throughout the study.

Please note: On the infusion days, the vital signs should be taken within 45 minutes prior to the methylprednisolone infusion in all patients. In addition, the vital signs should be obtained prior to the study drug infusion, then every 15 minutes (± 5 minutes) for the first hour; then every 30 minutes (± 10 minutes) until 1 hour after the end of the infusion. On non-infusion days, the vital signs may be taken at any time during the visit. Additional vital signs readings may be taken at the discretion of the investigator in the event of an

infusion related reaction or if clinically indicated and should be recorded on the unscheduled vital signs eCRF.

Please refer to relevant sections of protocol for more details.

#### 5.3.4.1 Electrocardiogram (ECG)

A 12-lead ECG should be taken at the visits indicated in the Schedule of Assessments - Table 3. Comments generated automatically by the ECG machine should not be recorded in the eCRF unless confirmed by a physician. An ECG is also required if the patient prematurely withdraws from the study.

#### 5.3.4.2 Physical Examination

The physical examination will be performed as per Schedule of Assessments. Diagnosis of new abnormalities or clinically significant worsening of pre-existing abnormalities should be recorded as adverse events if appropriate.

#### 5.3.4.3 Neurological Examination

A neurological examination will be performed at every planned visit and at unscheduled visit if applicable.

- In the presence of newly identified or worsening neurological symptoms, a neurological evaluation should be scheduled promptly. In case of events suggestive of relapse the Treating Investigator should request EDSS to be performed by the Examining Investigator.

Study investigators will screen patients for signs and symptoms of PML by evaluating neurological deficits localized to the cerebral cortex, such as cortical symptoms/signs, behavioral and neuropsychological alteration, retrochiasmal visual defects, hemiparesis, cerebellar symptoms/signs (e.g., gait abnormalities, limb incoordination). A brain MRI scan and CSF analysis may be warranted to assist in the diagnosis of PML. See Section 7.3.4.1 for guidance on the diagnosis of PML.

Patients with suspected PML, defined as a new or worsening neurological symptom which necessitates MRI and or lumbar puncture and CSF analyses to rule out PML, should be withheld from study treatment until PML is ruled out by complete clinical evaluation and appropriate diagnostic testing (see Section 7.3.4.1). The Sponsor's Medical Responsible and Medical Monitor should be contacted by email. In addition Sponsor medical responsible person should be immediately contacted by phone.

**A patient with confirmed PML should be withdrawn from the treatment.** PML should be reported as an SAE (with all available information) with immediate notification of the Medical Monitor (see also Section 7.1.1.3).

#### 5.3.4.4 Telephone Interviews

The purpose of this semi-structured interview is to identify new or worsening neurological symptoms that warrant an unscheduled visit and collect information on possible events of infections. The telephone interview will be conducted by site personnel familiar with the patient(s) every 4 weeks (± 3 days) between the study visits during the study treatment and Safety Follow up period starting from Week 8, until 48 weeks after

the last infusion. Thereafter, for those patients who require prolonged B-cell monitoring, telephone interviews will continue every 12 weeks (± 7 days) between regular visits.

The site will record in the eCRF the telephone interview as “Done” or “Not Done” and documentation of the interview will be maintained in the patient’s study file.

Please refer to Appendix 4 for detailed information.

##### 5.3.4.5 Columbia-Suicide Severity Rating Scale C-SSRS

The Columbia-Suicide Severity Rating Scale (C-SSRS) will be used for prospective suicidality assessment. C-SSRS is a tool used to assess the lifetime suicidality of a patient and to track suicidal events through the treatment. The structured interview prompts recollection of suicidal ideation, including the intensity of the ideation, behavior and attempts with actual/potential lethality.

The scale will be administered by the Treating Physician at the timepoints indicated in the Schedule of Assessments. The C-SSRS “*baseline*” will be collected at baseline and the C-SSRS “*since last visit*” will be collected at subsequent visits.

Please note: assessing the risk of suicide is a difficult and complex task when applied to the individual patient. Certainly, no single clinical scale can replace a thorough medical examination and suicide risk assessment. Ultimately, the determination of the presence of suicidality depends on clinical judgment.

### 5.3.5 The Karnofsky Performance Scale (clinician-reported version)

The Karnofsky Performance Scale score allows patients to be classified as to their functional impairment. This scale is usually used to compare effectiveness of different therapies and to assess the prognosis in individual patients. The lower the Karnofsky score, the worse the survival for most serious illnesses.

The scale will be administered by Examining Investigator at the time points indicated in the Schedule of Assessments - Table 3.

## 5.4 Laboratory Assessments

Roche Clinical Repository biomarker samples will be shipped directly to Roche Clinical Sample Operations unit. All other lab samples collected during the study will be shipped to Central Laboratory.

The procedures for the collection, handling and shipping of laboratory samples are specified in the Laboratory Manual.

The samples for this study should be classified, packed and shipped as UN3373 Biological Substance, Category B.

Full details of the central laboratory sample handling, shipment and reporting of results will be described in the Laboratory Manual.

During the double-blind treatment period of the study, the total volume of blood loss for laboratory assessments will be approximately 345 mL over 2 years. The amount of blood taken at each visit will vary, but will be no more than 56 mL. In the safety follow-up

period, the amount of blood taken at each visit will be no more than 41 mL. Patients consenting for RCR project may have additional blood samples taken – please refer to Section 5.5.1 for more details.

### 5.4.1 Standard Laboratory Assessments

Please note: Some laboratory parameters that could reveal patient's allocation to study treatment, such as FACS cell counts, absolute neutrophil counts, Ig levels, and type I interferon neutralizing antibody levels, will be blinded. In order to ensure patients' safety in the study and to allow for assessments of the re-treatment criteria, a central laboratory will provide study investigators and Medical Monitors with reflex messages triggered by critical blinded laboratory results. Investigators notified of their patient's critical laboratory test results will be instructed to suspend further treatment with study drug until the patient becomes eligible for re-treatment. The reflex messages from a central laboratory, together with non-blinded laboratory results, should be carefully reviewed at every visit before continuing with study treatment. Further details will be provided in Laboratory Manual.

**Hematology:** Hemoglobin, hematocrit, red blood cells (RBC), white blood cells (WBC) (absolute and differential), absolute neutrophil count, and quantitative platelet count.

**Blood chemistry:** AST/SGOT, ALT/SGPT, GGT, alkaline phosphatase, amylase, lipase, total protein, albumin, cholesterol, total bilirubin, urea, uric acid, creatinine, random glucose, potassium, sodium, calcium, phosphorus, lactic dehydrogenase, creatine phosphokinase, and triglycerides.

**Thyroid function test:** sTSH will be tested at screening, and yearly during the Double-blind Treatment Period. Thyroid autoantibodies will be assayed only at screening.

**FACS** will include (but is not limited to) the following cells:

- Total B cells ($CD19^{pos}$)
- Total T cell ($CD3^{pos}$)
- T helper cells ($CD3^{pos}$, $CD4^{pos}$)
- $T_{CTL}$ ($CD3^{pos}$, $CD8^{pos}$)
- NK Cells ($CD3^{neg}$, $CD16/56^{pos}$)
- B-cell subsets:
  - memory B-cells ($CD19^{pos}$, $CD27^{pos}$, $CD38^{neg}$)
  - naïve B-cells ($CD19^{pos}$, $CD27^{neg}$, $IgD^{pos}$)
  - plasmablasts ($CD19^{lo}$, $CD27^{pos}$, $CD38^{hi}$,)

**Quantitative Immunoglobulin:** Ig levels (including Total Ig, IgG, IgM, and IgA isotypes).

**Antibody titers:** Measurement of antibody titers to common antigens (mumps, rubella, varicella, S. pneumoniae) will be performed. This information is used to assess the effect of ocrelizumab on specific humoral immunity to bacterial and viral antigens.

**HAHA:** Serum samples will be collected for determination of antibodies against ocrelizumab (HAHA). Since ocrelizumab concentrations affect the HAHA assay, the concentration of ocrelizumab will be measured as well at all timepoints with HAHA assessment to enable interpretation of the results (PK sample). For details please refer to Schedule of Assessments.

**Pregnancy Test**: All women of childbearing potential must have regular pregnancy tests. At screening, a serum pregnancy test will be performed in central laboratory. During the study treatment period and Safety Follow up, a urine pregnancy test (sensitivity of at least 25 mIU/mL β-hCG) will be performed locally at the time points shown in Schedule of Assessments. On infusion visits, the urine pregnancy test should be performed prior to the methylprednisolone infusion. A positive urine pregnancy test should be confirmed with a serum test through the central laboratory prior to any further dosing with ocrelizumab.

Please note: additional laboratory tests will be performed at screening in order to verify eligibility criteria. Please refer to Table 3 for further details.

### 5.4.2 Hepatitis Screening and Liver Function Monitoring

Patients with a history or known presence of recurrent or chronic hepatitis B or C infection must be excluded from enrollment into the study (see Section 4.3). In addition, hepatitis B and C serology will be performed at screening. A positive result to either hepatitis surface antigen (HBsAg), or hepatitis B core antibody (total HBcAb) associated with positive viral DNA titres as measured by PCR, or a positive result for hepatitis C antibody (HepCAb) should result in the patient's exclusion. Patients with evidence of past resolved hepatitis B infection (i.e. positive total hepatitis B core antibody associated with a negative viral DNA) can be enrolled, and will have the hepatitis B viral DNA checked every 12 weeks as per Schedule of Assessment. Patients in whom the viral DNA becomes positive but in whom the quantity is at the lower limit of detection of the assay should have the test repeated as soon as possible. These patients may be referred to a hepatologist and treated, as clinically indicated. Patients found to have a viral DNA positive test with a copy number **exceeding $10^4$ copies/mL** should be referred to a hepatologist for assessment immediately. Patients in whom viral copy numbers increase beyond **$10^4$ copies/mL** during the cycle of the study will not receive further infusions of ocrelizumab and will enter the Safety Follow up Period.

Liver function, i.e. ALT/SGPT, AST/SGOT, gamma glutamyl transferase (GGT), alkaline phosphatase, total bilirubin, should be reviewed throughout the study. Patients developing evidence of liver dysfunction should be assessed for viral hepatitis and, if necessary, referred to a hepatologist or other appropriately qualified expert. Study drug should be withheld until the diagnosis of viral hepatitis has been excluded. Patients developing hepatitis B or C should be withdrawn from the study and should enter the Safety Follow up period. Should treatment be prescribed, this will be recorded in the

eCRF. Patients with viral hepatitis due to other agents, such as hepatitis A, may resume treatment after the patient's recovery.

Please refer also to Section 6.2.2 for further guidelines on liver function monitoring.

### 5.4.3 Plasma and Urine Banking for JC Virus

Long-term storage of plasma samples and urine is planned for JC virus DNA and/or other relevant tests for JC virus. Plasma samples (5 mL) and urine samples (10 ml) will be collected as per Schedule of Assessments. As the assay of the DNA virus has not been standardized, and a correlation between viremia and onset of PML has not been established, the JC virus assessments in plasma and urine will be performed if deemed necessary in the future and not on an ongoing basis.

### 5.4.4 Pharmacokinetic (PK)/Pharmacodynamic (PD) Assessments

Blood samples will be collected to evaluate the pharmacokinetics and pharmacodynamics of ocrelizumab as described in the Schedule of Assessments. The blood volume collected for pharmacokinetic assessments will be approximately 2 mL per sample. These samples will be assayed for ocrelizumab concentration using an enzyme-linked immunosorbent assay (ELISA).

Serum samples for determination of ocrelizumab concentrations will be collected at the time points detailed in the Schedule of Assessments. On the infusion visit at week 72, two serum samples should be collected, one 5-30 minutes prior to the methylprednisolone infusion and the second one 30 minutes (±10 minutes) following the completion of the ocrelizumab infusion. For all other infusion visits, a blood sample should be taken 5 - 30 minutes before the methylprednisolone infusion. At other times (non-infusion visits), samples may be taken at any time during the visit.

For sampling procedures, storage conditions, and shipment instructions, see the Sample Handling and Logistics Manual, which will be provided to each site.

### 5.4.5 Type I Interferon Neutralizing Antibody Assay

Type I interferon neutralizing antibody assay will be performed during double-blind phase of treatment period - please see Table 3 - Schedule of Assessments.

## 5.5 Roche Clinical Repository Specimen(s)

<u>Please note:</u> the Roche Clinical Repository research is contingent on review and approval for the exploratory biomarker assessments by an appropriate regulatory body (depending on the country where the study is performed) and a site's Institutional Review Board/ Ethics Committee. Written patient's informed consent to RCR project is also required. If a regulatory body or site's Institutional Review Board / Ethics Committee does not approve the extended analysis and long term storage of the biomarker samples, this section of protocol will not be applicable.

Specimens for dynamic (non-inherited) biomarker discovery and validation will be collected only from patients consenting to RCR.

These specimens will be used for research purposes to identify dynamic biomarkers that are predictive of response to ocrelizumab treatment (in terms of dose, safety and

tolerability) and will help to better understand the pathogenesis, course and outcome of multiple sclerosis and related diseases. Specimens for dynamic biomarker discovery will be single coded like any other clinical sample (labeled and tracked using the patient's study identification number (see Section 17).

The results of specimen analysis from the RCR will facilitate the rational design of new pharmaceutical agents and the development of diagnostic tests, which may allow for individualized drug therapy for patients in the future.

All RCR specimens will be destroyed no later than 15 years after the final freeze of the respective clinical database unless regulatory authorities require that specimens be maintained for a longer period. The specimens in the RCR will be made available for future biomarker research towards further understanding of MS treatment with ocrelizumab, related diseases and adverse events and for the development of potential associated diagnostic assays. The implementation and use of the RCR specimens is governed by the Roche Clinical Repository policy to ensure the appropriate use of the RCR specimens.

### 5.5.1 Specimen Types

Exploratory Biomarkers (non-DNA):

- **Plasma assays**

Blood (one, approximately 6 mL sample in EDTA) for plasma isolation will be obtained at various time points as shown in Schedule of Assessments. These samples will be used for biomarker assays which may include chemokines and other candidate biomarkers in multiple sclerosis. For sampling procedures, storage conditions and shipment instructions see study Sample Handling and Logistics Manual.

- **Blood for RNA expression profiling**

Blood (2 x approximately 2.5 mL collected in PAXgene vacutainers) for RNA isolation will be obtained at various time points as shown in Schedule of Assessments. The samples may be tested using techniques such as a micro array profiling system and/or RT PCR to study the expression profile of genes known to be involved with multiple sclerosis, and any other differentially expressed genes relative to treatment response or re-treatment. For sampling procedures, storage conditions and shipment instructions see study Sample Handling and Logistics Manual.

Exploratory Biomarkers (DNA): **one sample of 6 mL of blood** will be taken as per Schedule of Assessments. 6 ml whole blood sample to be taken only from patients consenting to RCR for pharmacogenetic and genetic analysis. If not done at Baseline (Visit 2), sample may be collected at next visit.

For all samples, dates of specimen collection should be recorded on the associated RCR page of the eCRF and/or in the clinical database.

## 5.6 Protein Biomarker Samples

Specimens for protein biomarker discovery and validation will be collected from all patients. These specimens will be used for research purposes to identify and/or verify

protein biomarkers that are predictive of response to ocrelizumab treatment (in terms of dose, safety and tolerability) and will help to understand the pathogenesis, course and outcome of relapsing MS and related diseases. Identification of patient subgroups with increased response to therapy or increased progression rates would provide information of significant clinical value to guide treatment decisions and aid in the appropriate use of the therapy. Analyses will include but are not limited to the B-cell activating factor (BAFF) and Complement Factor H (CFH) [50].

A 6 ml sample of whole blood will be collected in a plain tube without EDTA for serum isolation. For sampling procedures, storage conditions and shipment instructions see study Sample Handling and Logistics Manual, which will be provided to each study site.

Blood specimens for protein biomarker discovery and validation will be collected from all patients as per Schedule of Assessments. These specimens will be stored for 5 years after the end of the study and then destroyed.

## 5.7 Patient Reported Outcome(s)

PRO data will be collected at the study visit with an electronic tablet device. The tablet with the PRO instruments will be distributed by the investigator staff and completed in their entirely by the patient.

Please note: all PROs are required to be administered prior to administration of study drug and prior to any other study assessment(s) to ensure the validity of the instruments is not compromised, and data quality meet requirements of regulatory authorities [51] and best practices [52, 53].

PRO data will be elicited from patients in this study to better characterize the clinical profile of ocrelizumab. These PRO measurements are described in Sections 5.71, 5.7.2, and 5.7.3. Please note that the methods for collecting and analyzing PRO data are different from those for the ascertainment of observed or volunteered adverse events. Due to these differences, PRO data will not be reported as adverse events and no attempt will be made to resolve any noticeable discrepancies between PRO data and observed or volunteered adverse events.

### 5.7.1 Modified Fatigue Impact Scale (MFIS)

The Modified Fatigue Impact Scale (MFIS) will assess change in the level of fatigue. The MFIS is a 21-item instrument that asks patients to rate their fatigue over the past four weeks on a 5-point Likert scale, indicating “Never” to “Almost always.” Four scores can be derived from the MFIS, including a total score as well as scores for three subscales: physical, cognitive, and psychosocial functioning. Changes from baseline will be calculated for the total scale scores as well as for the subscale scores.

English version of MFIS is provided in Appendix 5.

### 5.7.2 The Center for Epidemiologic Studies Depression Scale (CES-D)

The Center for Epidemiologic Studies Depression Scale (CES-D) will be used to evaluate patients for depressive symptoms. The CES-D is a 20-item self-report instrument that asks patients to rate their feelings and behaviors over the past week on a 4-point Likert

scale, from “Rarely or none of the time (less than one day)” to “Most or all of the time (5-7 days).” Only a total scale score is calculated for the CES-D.

English version of CES-D is provided in Appendix 6.

#### 5.7.3 The Short Form (SF-36) Health Survey

The Short Form (SF-36) Health Survey is a generic quality of life instrument that has been widely tested for its psychometric properties and is widely used in clinical and epidemiological studies. The SF-36 contains 36 items and measures eight health domains: vitality, physical functioning, bodily pain, general health perceptions, physical role functioning, emotional role functioning, social role functioning, and mental health. The SF-36 yields a score for each domain, as well as summary scores for the physical and mental dimensions, and a single health utility index. It can be completed in 5-10 minutes.

English version of the Short Form (SF-36) Health Survey is provided in Appendix 7.

#### 5.7.4 Patient’s Assessment of Treatment Benefit

The Patient Perception of Treatment Questionnaire will be assessed before administration of PRO instruments at each visit in which PRO assessments are made, except for baseline. Specifically, patients will be asked whether they think their MS has become better, become worse or been stable since baseline. This question will serve as a global assessment from the patient perspective and will provide a useful anchor to help interpret the clinical meaningfulness of PRO results.

### 5.8 Pharmacoeconomic Assessments/ EQ-5D

Pharmacoeconomic assessments will be included for purposes of deriving health utilities for economic modeling. The EQ-5D will be used to derive utilities for health states included in MS economic models and will be administered as per Schedule of Assessments.

The EQ-5D (formerly known as EuroQOL) is a generic, preference-based health-related quality of life instrument. It has five dimensions assessing mobility, self-care, usual activities, pain/discomfort and anxiety /depression. Each dimension has 3 possible levels. Different combinations of responses are utility-weighted to produce a single health utility index. The Visual Analog Scale (VAS) measures self-reported health on a scale between “worst imaginable“ and “best imaginable“ health states.

EQ-5D is a patient reported outcome and should be performed before any other study assessments and before administration of study drug in order to minimize bias.

### 5.9 Post Study Provisional Care

Patients who complete the 96-week double-blind treatment period, and who in the opinion of the investigator may benefit from the further treatment with ocrelizumab, may become eligible for a separate open label extension study, under a separate protocol.

For patients who have withdrawn from treatment or who are not otherwise eligible for treatment with ocrelizumab, it is at the discretion of the investigator to decide on further treatment of the underlying disease However, immunosuppressants, lymphocyte depleting therapies, and lymphocyte tracking blockers may increase the risk of infections while patients remain B-cell depleted and therefore are not allowed during the Safety Follow up Period.

# 6. INVESTIGATIONAL MEDICINAL PRODUCT

Patients will be randomly assigned into one of two treatment groups:

– **Group A** – Ocrelizumab 600 mg regimen (given as dual infusions of 300 mg of ocrelizumab 14 days apart for the first 24 weeks and single infusions of 600 mg every 24 weeks thereafter)

– **Group B** – Rebif® subcutaneous injections, 3x weekly

**Table 5: Treatment Groups and Schedule of Study Medication**

| Treatment Group | Schedule of Study Medication |
|---|---|
| **Group A**<br>Ocrelizumab 600 mg regimen | Two i.v. infusions of ocrelizumab 300 mg separated by 14 days for the first 24 weeks, followed by single i.v. infusions of ocrelizumab 600 mg every 24 weeks thereafter<br>AND<br>placebo Rebif® subcutaneous injections, 3x weekly. |
| **Group B**<br>Rebif® | Rebif® subcutaneous injections, 3x weekly<br>AND<br>Two i.v. infusions of placebo ocrelizumab 300 mg separated by 14 days for the first 24 weeks, followed by single i.v. infusions of placebo ocrelizumab 600 mg every 24 weeks thereafter. |

The first i.v. infusion of ocrelizumab or placebo, or the first subcutaneous injection of Rebif® or placebo will be administered on study Day 1.

Please note: 100 mg of methylprednisolone i.v. will be administered prior to each intravenous infusion of ocrelizumab/ocrelizumab placebo.

## 6.1 Ocrelizumab

### 6.1.1 Preparation and Administration of Ocrelizumab Infusions

**Detailed instructions for the preparation of the infusion bags containing the study drug will be provided separately in the Dose Preparation Guidelines.**

Although ocrelizumab may be administered on an outpatient basis, patients may be hospitalized for observation at the discretion of the investigator (in some countries this is the standard procedure). The study drug infusions should always be administered in a hospital or clinic environment under close supervision of the

investigator or a medically qualified staff member with immediate availability of full resuscitation facilities.

### Preparation of infusion

Ocrelizumab drug product must be diluted before administration. Solutions of ocrelizumab for i.v. administration are prepared by dilution of the drug product or ocrelizumab-matching placebo into an infusion bag containing 0.9% sodium chloride, to a final drug concentration of approximately 0.7 to 2 mg/mL. It is important not to use evacuated glass containers (to prepare the infusion), which require vented administration sets because this causes foaming as air bubbles pass through the solution.

Prior to the start of the infusion, please ensure that the content of the bags is at room temperature to avoid an infusion reaction due to the administration of the solution at low temperatures.

### Infusion procedures

Ocrelizumab should be given as a slow i.v. infusion. It must not be administered as an i.v. push or bolus. Well adjusted infusion pumps should be used to control the infusion rate and the study drug should be infused through a dedicated line.

All patients should receive pre-treatment before the infusion (see Section 6.1.2).

### Dual infusion cycle (Cycle 1)

The first cycle will consist of 2 infusions of 300 mg ocrelizumab administered 14 days apart. For each infusion it is necessary to prepare a single infusion bag containing 300 mg ocrelizumab. Specific instructions will be provided separately in the Dose Preparation Guidelines and must be followed exactly. The infusion should be started at a rate of 25 mL/h. This should be escalated at the rates shown in Table 6.

**Table 6: Infusions of Ocrelizumab 300 mg**

| Time (Minutes) | Infusion Rate (mL/hr) | Maximum Dose per Interval (mg) | Cumulative Dose (mg) |
|---|---|---|---|
| 0-30 | 25 | 18.75 | 18.75 |
| 31-60 | 50 | 37.5 | 56.25 |
| 61-90 | 75 | 56.25 | 112.5 |
| 91-120 | 100 | 75 | 187.5 |
| 121-150* | 150 | 112.5 | 300* |

*Infusion of 300 mg of ocrelizumab should be completed at approximately 150 minutes (~2.5 hours)

### Single infusion cycles (Cycles 2 - 4)

Cycles 2 through 4 will consist of one infusion of 600 mg ocrelizumab administered on Day 1 of each cycle. For each cycle it is necessary to prepare **two infusion bags** containing a total of 600 mg ocrelizumab. Specific instructions will be provided separately in the Dose Preparation Guidelines and must be followed exactly. The infusion should be started at a rate of 25 mL/h. This should be escalated at the rates shown in Table 7.

**Table 7: Subsequent Infusions of Ocrelizumab 600 mg**

| Infusion Bag | Time (Minutes) | Infusion Rate (mL/hr) | Maximum Dose per Interval (mg) | Cumulative Dose (mg) |
|---|---|---|---|---|
| Bag 1 | 0-30<br>31-60<br>61-90<br>91-120<br>121-150 | 25<br>50<br>75<br>100<br>150 | 25<br>50<br>75<br>100<br>150 | 25<br>75<br>150<br>250<br>400* |
| Bag 2 | 151-180<br>181-210<br>210-240** | 200<br>200<br>200 | 66.7<br>66.7<br>66.7 | 467<br>533<br>600 |

*Change to Bag 2
**Infusions of Bag 1 and Bag 2 should be completed at approximately 240 minutes (≈4 hours)

**Please note: The contents of Bag 1 and Bag 2 will not be identical with respect to volume and drug concentration. Therefore, it is essential that the specific instructions provided in the Dose Preparation Guidelines be followed exactly. In addition, since the contents of Bags 1 and 2 vary, the regular increments in the infusion rates also vary. Thus, the bags must be administered strictly in the correct order according to the instructions outlined in Table 7.**

Because of the varying volumes in the infusion bags and the possible need to vary infusion rates depending on tolerance of the infusion, the total infusion time may exceed the time stated. UNLESS AN INFUSION REACTION OCCURS NECESSITATING DISCONTINUATION, THE ENTIRE CONTENTS OF BOTH INFUSION BAGS MUST BE ADMINISTERED TO THE PATIENT.

After completion of the infusion, the i.v. cannula should remain in situ for at least 1 hour in order to be able to administer drugs intravenously, if necessary in the event of a delayed reaction. If no adverse events occur during this period of time, the i.v. cannula may be removed and the patient may be discharged.

Because ocrelizumab solutions for infusion do not contain a preservative, the i.v. bags containing ocrelizumab diluted solutions should be stored refrigerated at 2-8°C. They may be stored under refrigerated conditions for up to 24 hours prior to use. Bags containing ocrelizumab diluted solutions for i.v. infusion need to be used within 48 hours of preparation (i.e., stable 24 hours at 2-8°C plus 24 hours at room temperature). As noted above, the diluted infusion bags should be at room temperature prior to administration to the patient.

### 6.1.2 Prevention and Treatment of Infusion Related Reactions

Methylprednisolone has been shown to decrease the incidence and the severity of infusion reactions. In RA patients treated with a similar agent, rituximab, the rate and severity of infusion reactions markedly decreased with i.v. corticosteroid pre-medication [54]. To reduce potential infusion reactions, all patients will receive prophylactic

treatment with 100 mg of methylprednisolone, administered by slow i.v. infusion, to be completed approximately 30 minutes before the start of each ocrelizumab infusion.

It is also recommended that the infusion is accompanied by prophylactic treatment with an analgesic/antipyretic such as acetaminophen/paracetamol (1 g) and an i.v. or oral antihistaminic (such as i.v. diphenhydramine 50 mg; or equivalent dose of alternative) 30 to 60 minutes prior to the start of an infusion to reduce potential infusion reactions. Patients administered a sedating antihistaminic for the treatment or prevention of infusion reactions should be given appropriate warnings concerning drowsiness and potential impairment of ability to drive or operate machinery.

Since transient hypotension may occur during ocrelizumab infusion, the investigator may wish to withhold anti-hypertensive medications 12 hours prior to ocrelizumab infusion.

For the treatment of fever, discomfort or allergic events, the use of oral acetaminophen/paracetamol (1 g), and intramuscular or slow i.v. antihistaminic administration, such as diphenhydramine (25 mg to 100 mg i.v.), and/or a bronchodilator, is recommended. The acetaminophen/paracetamol and diphenhydramine doses should be repeated as clinically indicated. Non allergic events should be treated symptomatically as judged clinically relevant by the investigator.

**In patients with CTCAE Grade 3 or higher (severe) infusion reactions with associated respiratory symptoms (stridor, wheeze or bronchospasm), additional treatment with bronchodilators may be indicated.**

One patient with well-controlled asthma at baseline experienced an acute asthma attack following their first rituximab infusion. Physicians should therefore monitor patients with a history of asthma carefully and institute an appropriate treatment if signs and symptoms of asthma are noticed.

Section 6.1.3 details the reduction, interruption or discontinuation of the infusion in the event of an infusion reaction.

### 6.1.3 Ocrelizumab Dose Modifications, Interruptions and Delays

No ocrelizumab dose modifications are foreseen.

Slowing of the infusion rate or interruption of the infusion may be necessary in the event of an infusion reaction. In rare patients, ocrelizumab treatment may need to be discontinued. Guidance is provided below.

**Handling infusion reactions:**

In the event that a patient experiences a mild to moderate (CTCAE Grade 1 or 2 – Appendix 3) infusion-related event, the infusion rate should be reduced to half the rate being given at the time of onset of the event (e.g. from 50 mL/hr to 25 mL/hr or from 100 mL/hr to 50 mL/hr). Once the event has resolved, the investigator should wait for 30 minutes while delivering the infusion at the reduced rate. If tolerated, the infusion rate may then be increased to the next closest rate on the patient's infusion schedule and the rate increments resumed.

Patients who experience a severe infusion-related event (CTCAE Grade 3) or flushing, fever and throat pain cluster should have their infusion interrupted immediately and should receive aggressive symptomatic treatment. The infusion should be re-started only after all the symptoms have disappeared. The initial infusion rate at restart should be half of the infusion rate that was in progress at the time of onset of the reaction.

**Please note: patients who experience a life threatening or disabling infusion-related event (CTCAE Grade 4), such as anaphylaxis, during an infusion should have their infusion immediately stopped and should receive appropriate treatment (including use of resuscitation medications and equipment that must be available and used as clinically indicated). These patients should be withdrawn from treatment and should enter the Safety Follow-up Period.**

#### 6.1.4 Criteria for Re-Treatment with Ocrelizumab

Prior to re-treatment with ocrelizumab, patients will be evaluated for the following conditions and laboratory abnormalities. If any of these are present prior to re-dosing, further administration of ocrelizumab should be suspended until resolved or held indefinitely:

- Severe allergic or anaphylactic reaction to a previous ocrelizumab infusion
- Any significant or uncontrolled medical condition or treatment-emergent, clinically significant laboratory abnormality
- Active infection
- Absolute neutrophil count < 1.5 x $10^3$/µL
- CD4 cell count < 250/µL
- Hypogammaglobulinemia IgG < 4.0 g/L

Please note: any critical blinded laboratory values for IgG, absolute neutrophil count and CD4 will be provided to the Treating Investigator and the Medical Monitor. Investigators notified of their patient's critical laboratory test result will be instructed to suspend further treatment with study drug until the patient can be further evaluated. A repeat laboratory test may be necessary to confirm the results. Patients with values below these critical values should not be retreated until the re-treatment criteria are met and these laboratory values have normalized.

### 6.2 Rebif®

#### 6.2.1 Dose and Schedule of Rebif®

Please refer to Table 8 for overview of Rebif® regimen.

The first subcutaneous injection of Rebif®/placebo will be administered on Study Day 1. Patients will be instructed by a nurse or investigator how to self-administer the injections; the first dose of Rebif®/placebo will be self-administered under the supervision of a nurse or physician. Thereafter, patients will self-administer their Rebif®/placebo treatment three times weekly. Rebif®/placebo must be administered, if possible, at the same time (preferably in the late afternoon or evening) on the same three days (e.g., Monday, Wednesday, and Friday) at least 48 hours apart. Patients must be instructed in the use of

aseptic techniques when administering Rebif®/placebo injections. Patient understanding and use of aseptic self-injection techniques and procedures must be periodically re-evaluated.

Since Rebif® needs to be stored at 2-8°C, it is recommended to remove the syringe from refrigerator at least 30 minutes prior to use. Patient should be reminded not to heat or microwave a syringe.

When starting treatment with Rebif®, the dose will be gradually escalated (please refer to Table 8). The Rebif®/placebo initiation package corresponds to the patient needs for the first month of treatment.

**Table 8: Overview of Rebif® Dosing Regimen ***

| | **Treatment Initiation** | | **Treatment Continuation** | **Dose modification (if required)** |
|---|---|---|---|---|
| **Week** | **Weeks 1- 2** | **Weeks 3-4** | **Week 5 onwards** | **–** |
| *Study Day* | *1-14* | *15-28* | *29+* | *At any time >Day 29* |
| **Dose of Rebif®** | **Rebif® 8.8 µg** (1 pre-filled syringe [0.2 ml] containing 2.4 MIU of interferon β-1a) s.c. **3x per week** | **Rebif® 22 µg** (1 pre-filled syringe [0.5 ml] containing 6 MIU of interferon β-1a) s.c. **3x per week** | **Rebif® 44 µg** (1 pre-filled syringe [0.5 m] containing 12 MIU of interferon β-1a) s.c. **3x per week** | **Rebif® 22 µg** (1 pre-filled syringe [0.5 ml] containing 6 MIU of interferon β-1a) s.c. **3x per week** |

** provided in blinded fashion*

Non-steroid anti-inflammatory drugs (ibuprofen) or acetaminophen are recommended in case of injection site reaction; investigator should follow local label for further information.

### 6.2.2 Rebif® Dose Modifications, Interruptions and Delays

Rebif®/ Rebif® placebo should be taken three times a week. Rebif®/ Rebif® placebo should never be taken on two consecutive days. If a patient misses a dose, then the next dose must be taken as soon as possible. The patient should avoid taking Rebif®/ Rebif® placebo on the following day. The patient should return to their regular schedule the following week. If a patient takes more than the prescribed dose or takes it on 2 consecutive days, they should inform the investigator immediately.

Asymptomatic increases in laboratory parameters of hepatic function have been associated with Rebif®.

In case of elevation of liver function tests the following rules will apply:

| | |
|---|---|
| – **ALT ≥ 10 x ULN** OR jaundice or other clinical symptoms of liver dysfunction | In case of detection of elevated **ALT ≥ 10 x ULN**, jaundice or other clinical symptoms of liver dysfunction the injections of Rebif®/Rebif® placebo must be discontinued permanently. The monitoring of liver function tests should be continued on a monthly basis until return to normal baseline levels or CTCAE v.4.0 Grade 1 toxicity (ALT: >ULN - 3.0 x ULN). A consultation with hepatologist is recommended. Patients should move to Safety Follow up Period. |
| – **ALT ≥ 5 x ULN** | In case of detection of elevated **ALT ≥ 5 x ULN** (but below 10 xULN) the injections of Rebif®/Rebif® placebo must be discontinued temporarily. Additional blood chemistry panel including AST, ALP, GGT and bilirubin should be performed biweekly until no further increase is observed. Subsequently, ALT analysis has to be performed every month until return to normal baseline levels or CTCAE v.4.0 Grade 1 toxicity (ALT >ULN - 3.0 x ULN). A consultation with hepatologist should be considered as per investigator judgment.<br>If causes of toxicity other than possible treatment with Rebif® are excluded, the patient may then be cautiously re-challenged with Rebif®/Rebif® placebo 22µg provided in a blinded fashion upon request to IxRS. The monitoring of liver function tests should continue on a monthly basis. If there is no further recurrence of toxicity, patient may continue treatment with Rebif®/Rebif® placebo 44 µg provided in a blinded fashion upon investigator's request to IxRS. **In case of recurrence of toxicity (ALT > 3 xULN, or other clinical symptoms of liver dysfunction) the injections of Rebif®/Rebif® placebo should be discontinued permanently.** Patients should move to Safety Follow up Period.<br><br>Please note: Re-initiation of therapy with Rebif® following elevation of liver function tests can only be considered once. |
| – **ALT > 3 x ULN** | In case of detection of elevated **ALT > 3 x ULN** (but below 5x ULN) additional blood chemistry panel including AST, ALP, GGT and bilirubin should be performed biweekly until no further increase is observed. Subsequently, ALT analysis has to be performed every month until return to normal baseline levels or CTCAE v.4.0 Grade 1 toxicity (ALT>ULN - 3.0 x ULN). |

## 6.3 Formulation, Packaging and Labeling

Study drug packaging will be overseen by the Roche clinical trial supplies department and bear a label with the identification required by local law, the protocol number, drug identification and dosage.

The packaging and labeling of the study medication will be in accordance with Roche standards and local regulations.

Upon arrival of investigational products at the site, site personnel should check them for damage and verify proper identity, quantity, integrity of seals and temperature conditions, and report any deviations or product complaints to the monitor upon discovery.

### 6.3.1 Ocrelizumab

**Formulation**

Ocrelizumab is manufactured as a sterile, clear, colorless, preservative free liquid intended for dilution for i.v. administration.

Ocrelizumab is supplied as a liquid formulation containing 30 mg/mL ocrelizumab in 20 mM sodium acetate at pH 5.3, with 4% (106 mM) trehalose dihydrate and 0.02% polysorbate 20. The drug product is provided as a single-use liquid formulation in a 15 cc Type I USP glass vial, fitted with a 20 mm fluoro-resin laminated stopper and an aluminum seal with a flip-off plastic cap and contains a nominal 200 mg ocrelizumab. No preservative is used as each vial is designed for single use.

Ocrelizumab-matching placebo is also supplied in 15 cc single-use vials. Placebo has the same composition and configuration as the drug product, but does not contain ocrelizumab.

**Packaging**

The hospital units/pharmacy will receive study medication kits for each patient.

For the double-blind treatment in Cycle 1, consisting of two 300 mg infusions 14 days apart, the study medication kit will contain 2 single-use liquid vials with ocrelizumab (or ocrelizumab placebo).

For each of the subsequent Cycles 2-4 consisting of a single 600 mg infusion, two kits will be dispensed. Each kit will contain 2 single-use liquid vials with ocrelizumab (or ocrelizumab placebo), from which only 3 vials should be used.

**Storage of Ocrelizumab and Placebo Vials for Infusion:**

Ocrelizumab and placebo vials are stable at 2-8°C (refrigerated storage). They should not be used beyond the expiration date stamped on the carton. Expiration dating may be extended during the trial; the Sponsor will provide documentation. Ocrelizumab vials should not be frozen or shaken and should be protected from direct sunlight.

The study medication labels will be produced in accordance with the local requirements.

### 6.3.2 Rebif®

**Formulation and packaging**

Rebif® (IFN β-1a) will be supplied as a liquid formulation for injection in pre-filled syringes.

The liquid formulation is supplied in syringes containing 0.2 mL or 0.5 mL of solution. These commercially available syringes will be provided to the sites by the Sponsor and re-labeled as investigational medicinal product.

The placebo to Rebif® is provided as a liquid formulation in a pre-filled syringe containing 0.2 mL or 0.5 mL of 0.9% sodium chloride solution without any active substance.

The study medication kits, which will be used for the initial 4 weeks of treatment, will contain 12 pre-filled syringes, either 6x 8.8 µg and 6x 22 µg OR placebo. The study medication kits that will be used for treatment continuation will contain 12 pre-filled syringes 12x44 µg of IFN β-1a or 12x22 µg of IFN β-1a or placebo

The Rebif® and Rebif® placebo pre-filled syringes are for subcutaneous use only.

Please note: if Rebif® dose modification is required due to lab abnormalities possibly related to the treatment with Rebif®, the investigator (the treating physician) will need to notify IxRS and the blinded study medication (Rebif® placebo or Rebif® verum) will be dispensed accordingly. In addition, to ensure patient safety in the study, unscheduled visits may be required for additional assessments, monitoring and for dispensing study medication.

The study medication labels will be produced in accordance with the local requirements. The strength will be presented as follows: 44 µg / 22 µg / placebo.

**Storage of Rebif®**

Rebif®/Rebif® placebo pre-filled syringes need to be stored in a refrigerator at 2-8°C, in the original package in order to protect from light. The patient may remove Rebif® from the refrigerator and store it not above 25°C for one single period of up to 14 days. Rebif® must then be returned to the refrigerator and used before the expiry date.

## 6.4 Blinding and Unblinding

The Patient Randomization List will be generated by IxRS using a pre-defined randomization specification. The Randomization List will not be available at the study center, to the Roche monitors, project Statisticians or to the Sponsor's project team. Unblinding of treatment assignment should not occur except in the case of emergency situations, where the knowledge of what study medication the patient is receiving is critical for clinical management. Treating Investigators are asked to contact the Roche Medical Monitor, prior to unblinding any patient, in order to discuss the medical necessity for unblinding. Any request from the investigator for information about the treatment administered to study patients for another purpose must be discussed with Roche. Unblinding will be performed by means of an Interactive Web Response System (IxRS). As per regulatory reporting requirement, Roche will unblind the identity of the

study medication for serious adverse events that are considered by the investigator or the Sponsor to be related to study drug, that are unexpected as per safety reference document(s), e.g., IB, CDS, and SPC, and that are not exempted from unblinding as per Section 7.2.2.2. Details of patients who are unblinded during the study will be included in the Clinical Study Report.

Unblinding for analysis of biological samples, or ongoing safety monitoring by a Data Monitoring Committee [DMC], will be performed according to procedures in place to ensure integrity of the data.

## 6.5 Accountability of IMP and Assessment of Compliance

### 6.5.1 Accountability of IMP

The investigator is responsible for the control of drugs under investigation. Adequate records for the receipt and disposition of the study drug must be maintained. Accountability will be assessed by maintaining adequate drug dispensing and return records.

Accurate records must be kept for each study drug provided by the Sponsor. These records must contain the following:

- Documentation of drug shipments received from the Sponsor (date received and quantity)
- Disposition of unused study drug not dispensed to patient.

A Drug Dispensing Log must be kept current and should contain the following information:

- the identification of the patient to whom the study medication was dispensed
- the date[s] and quantity of the study medication dispensed *to* the patient
- the date[s] and quantity of the study medication returned *by* the patient.

All records and drug supplies must be available for inspection/accountability by the Monitor at every monitoring visit.

### 6.5.2 Assessment of Compliance

Patient compliance will be assessed by maintaining adequate study drug dispensing records. The investigator is responsible for ensuring that dosing is administered in compliance with the protocol. Delegation of this task must be clearly documented and approved by the investigator.

The study pharmacist should keep all ocrelizumab/ocrelizumab placebo vials to measure compliance. All patients will be asked to return on regular intervals all used and unused Rebif®/Rebif® placebo containers to the site as a measure of compliance.

## 6.6 Destruction of the IMP/Comparator

Local or institutional regulations may require immediate destruction of used IMP for safety reasons. In these cases, it may be acceptable for investigational site staff to destroy dispensed IMP before a monitoring inspection provided that source document verification is performed on the remaining inventory and reconciled against the documentation of

quantity shipped, dispensed, returned and destroyed. Written authorization must be obtained from the Sponsor at study start up before destruction.

Written documentation of destruction must contain the following:

– Identity (batch numbers or medication numbers) of IMP and comparators destroyed

– Quantity of IMP destroyed

– Date of destruction

– Method of destruction

– Name and signature of responsible person who destroyed the IMP.

Wherever possible, preferably drug should be destroyed locally on site according to their local policies and procedures once drug accountability has been completed by the monitor.

# 7. SAFETY INSTRUCTIONS AND GUIDANCE

## 7.1 Adverse Events (AEs) and Laboratory Abnormalities

### 7.1.1 Clinical AEs

According to the International Conference of Harmonisation (ICH), an AE is any untoward medical occurrence in a patient or clinical investigation patient administered a pharmaceutical product and which does not necessarily have a causal relationship with this treatment. An AE can therefore be any unfavorable and unintended sign, including an abnormal laboratory finding, symptom, or disease temporally associated with the use of a (investigational) medicinal product, whether or not considered related to the medicinal (investigational) product. Pre-existing conditions which worsen during a study are to be reported as AEs.

In the eCRF, adverse events will be reported at each visit.

**Clinical relapses** will be recorded only on a pre-specified eCRF "MS relapse" eform.

Infusion-related reactions will be recorded only on a pre-specified eCRF "Infusion Related Reaction" eform.

B-cell depletion is the expected outcome of ocrelizumab treatment and is not an adverse event. However, patients may be at risk for infections and particular attention should be directed toward early identification and treatment of infections. During the study, investigators are requested to promptly investigate patients reporting signs or symptoms of infection, to take appropriate specimens for identification of the pathogen and to treat infections aggressively (see Section 7.3.1). Prior to enrollment into the study, it is recommended that the investigators review and, if warranted, update patient's immunizations in accordance with country medical immunization guidelines (see also Section 4.4.3).

#### *7.1.1.1 Intensity*

Adverse events will be graded according to Common Terminology Criteria for Adverse Events (CTCAE), version 4 and is provided to the investigator in a separate handout entitled "Common Terminology Criteria for Adverse Events v4.0"- see Appendix 3.

Adverse events not listed by the CTCAE will be graded using the following criteria:

Grade 1: Discomfort noticed but no disruption of normal daily activity

Grade 2: Discomfort sufficient to reduce or affect normal daily activity

Grade 3: Inability to work or perform normal daily activity

Grade 4: Represents an immediate threat to life.

Any Grade 4 or higher AE, either by CTCAE criteria or the additional criteria listed below, should be reported as an SAE (see Section 7.1.1.3).

#### *7.1.1.2 Drug - Adverse Event Relationship*

**Relationship** of the AE to the treatment should always be assessed by the investigator. The causality relationship of study drug to the adverse event will be assessed by the investigator as either: Yes or No. Please refer to Appendix 1 for more details.

#### *7.1.1.3 Serious Adverse Events (Immediately Reportable to Sponsor)*

A Serious Adverse Event is any experience that suggests a significant hazard, contraindication, side effect or precaution. It is any AE that, at any dose, fulfils at least one of the following criteria:

- is fatal; (results in **death***; please note: death is an outcome, not an event)
- is Life-Threatening (please note: the term "Life-Threatening" refers to an event in which the patient was at immediate risk of death at the time of the event; it does not refer to an event which could hypothetically have caused a death had it been more severe).
- required in-patient hospitalization or prolongation of existing hospitalization;
- results in persistent or significant disability/incapacity;
- is a congenital anomaly/birth defect;
- is medically significant or requires intervention to prevent one or other of the outcomes listed above.

***The term sudden death should only be used when the cause is of a cardiac origin as per standard definition. The terms death and sudden death are clearly distinct and must not be used interchangeably**.

The exception to this definition of an SAE is in the rare event that a patient is hospitalized following an MS relapse, as long as the reason for hospitalization is to receive standard treatment with i.v. methylprednisolone. The rationale for this exception is that some countries and/or clinical sites routinely hospitalize patients who require

administration of methylprednisolone in the event of an MS relapse. Thus, the SAE criteria for "hospitalization" would be met on the basis of local practice and would not reflect the seriousness of the event.

When the MS relapse results in hospitalization for any reason other than for routine treatment of the relapse (such as for a treatment course beyond the standard treatment described in (see Section 4.4.2) or when hospitalization is prolonged, the MS relapse should be considered a SAE.

The study will comply with all local regulatory requirements and will adhere to the full requirements of the ICH Guideline for Clinical Safety Data Management, Definitions and Standards for Expedited Reporting, Topic E2 (see Appendix 2).

### 7.1.2 Treatment and Follow-up of AEs

Adverse events (AEs) should be followed up until they have stabilized or have returned to baseline status (in the event of an exacerbation of a pre-existing condition). This is especially important for those events where the reported causal relationship to study medication(s) is "related". If a clear explanation is established, it should be recorded on the eCRF.

If after study completion or withdrawal, return to baseline status or stabilization cannot be established an explanation should be recorded on the eCRF.

### 7.1.3 Laboratory Test Abnormalities

Laboratory test results will be recorded on the laboratory results eform of the eCRF, or appear on electronically produced laboratory reports submitted directly from the central laboratory, if applicable.

Any treatment-emergent abnormal laboratory result which is clinically significant, i.e., meeting one or more of the following conditions, should be recorded as a single diagnosis on the AE eform in the eCRF:

- Accompanied by clinical symptoms,
- Leading to a change in study medication (e.g. dose modification, interruption or permanent discontinuation),
- Requiring a change in concomitant therapy (e.g. addition of, interruption of, discontinuation of, or any other change in a concomitant medication, therapy or treatment).

Any laboratory result abnormality fulfilling the criteria for an SAE should be reported as such, in addition to being recorded as an AE in the eCRF.

#### *7.1.3.1 Follow-up of Abnormal Laboratory Test Values*

In the event of medically significant unexplained abnormal laboratory test values, the tests should be repeated and followed up until they have returned to the normal range and/or an adequate explanation of the abnormality is found. If a clear explanation is established it should be recorded on the eCRF.

B-cell depletion is a pharmacodynamic effect and is not an adverse event.

**Blinded laboratory values for IgG, absolute neutrophil count and CD4 which are critical will be provided to the investigator and the Medical Monitor. Investigators notified of their patient's critical laboratory test result will be instructed to suspend further treatment with study drug until the patient can be further evaluated. A repeat laboratory test may be necessary to confirm the results. Patients with values below these critical values should not be re-treated until the re-treatment criteria are met (see Section 6.1.4) and these laboratory values have normalized.**

## 7.2 Handling of Safety Parameters

### 7.2.1 Reporting of AEs

All adverse events will be documented in the eCRF.

New or worsening neurological symptoms not considered MS-related should be recorded on an AE page and the monitor should be informed.

### 7.2.2 Reporting of Serious Adverse Events

#### *7.2.2.1 Immediate Reporting to the Sponsor*

Any clinical AE or abnormal laboratory test value that is *serious* (as defined in Section 7.1.1.3), which occurs during the course of the study, regardless of the treatment group, must be reported to the Sponsor **within *one* working day** of the investigator becoming aware of the event (expedited reporting). In addition, for fatal and life-threatening events, the Medical Monitor should be contacted immediately. Contact numbers for the Medical Monitor (including after hours cover) will be provided to the site before any patients are screened.

After the patient signs the Informed Consent, but prior to initiation of study medication, only SAEs caused by a protocol-mandated intervention will be collected (e.g., SAEs related to MRI exam). After first dose of study medication, all SAEs must be reported.

Related SAEs ***MUST*** be collected and reported regardless of the time elapsed from the last study drug administration, even if the study has been closed.

Unrelated SAEs must be collected and reported during the study through the end of the Safety Follow-up Period, which is at least 48 weeks after the last infusion but may be extended in patients whose B-cells take longer to replete.

The investigator must complete the SAE reporting form in the eCRF. Relevant follow-up information should be submitted as soon as it becomes available. Only if a technical failure prevents the ability to report an SAE in the eCRF, then the paper *SAE Reporting Form* provided by the Sponsor must be completed and faxed to the number provided.

A death occurring during the study or information related to such occurrence that comes to the attention of the investigator during the study must be reported immediately to the Sponsor, whether considered treatment-related or not.

The following are not considered as an SAE:

- Elective hospitalizations or surgical procedures that are a result of a patient's pre-existing condition(s) that have not worsened since receiving trial medication. Examples may include, but are not limited to, cholecystectomy for gallstones, and diagnostic testing. Such events should still be recorded as medical procedures in the eCRF.
- Hospitalization to receive trial medication such as infusions of ocrelizumab unless this is prolonged (more than 24 hours).
  Hospitalization following an MS relapse as long as the reason for hospitalization is to receive standard treatment with i.v. methylprednisolone

Of specific importance is the prompt reporting of serious infections. In particular, PML should be reported as an SAE (with all available information) with immediate notification of the Medical Monitor.

This study adheres to the definition and reporting requirements of ICH Guideline for Clinical Safety Data Management, Definitions, and Standards for Expedited Reporting, Topic E2 (see Appendix 2).

#### *7.2.2.2 Expedited Reporting to Health Authorities, Investigators, Institutional Review Boards, and Ethics Committees*

The Sponsor will promptly evaluate all reported SAEs against cumulative product experience to identify and expeditiously communicate possible new safety findings to investigators, IRBs, ECs, and relevant health authorities based on applicable legislation.

Reporting requirements will be based on the investigator's assessment of causality and seriousness, with allowance for upgrading by the Sponsor as needed. To determine reporting requirements for single adverse event cases, the Sponsor will also assess the expectedness of the event on the basis of the Investigator's Brochure.

In principle, adverse events which are serious, related and unexpected will be reported in an expedited manner within 15 days (non-fatal/non-life-threatening) or 7 days (fatal or life-threatening).

Only those adverse events qualifying for expedited reporting occurring in patients on active treatment will be sent in an expedited timeframe to Health Authorities, Investigators, Institutional Review Boards, and Ethics Committees. This requires unblinding of patient treatment allocation.

For certain types of adverse events, the relation to study medication cannot be assessed based on single case evaluation. Therefore, in order to prevent unnecessary unblinding of study participants, the following events are exempted from expedited reporting:

- neoplasms benign, malignant, and unspecified (including cysts and polyps),

- infections and infestations with the exception of opportunistic infections (including PML and reactivation of viral infections).

The DMC will review adverse events at each quarterly meeting and assess their relation to study medication based on review of aggregate unblinded safety information.

### 7.2.3 Pregnancy and Lactation

Female patients should take all appropriate precautions to avoid becoming pregnant during this study and for the entire duration of B-cell depletion. As such, women of childbearing potential should use adequate contraception for the duration of the trial and for 48 weeks after receiving their last infusion of ocrelizumab, or until their B-cells have repleted whichever is the longer. Regular pregnancy tests will be performed during the study. If a female patient becomes pregnant during the study and chooses to carry her pregnancy, no further infusions of ocrelizumab should be administered.

Effects on pregnancies from the female partners of B-cell depleted males have not been studied. Therefore, it is required that male patients also use reliable contraception while receiving ocrelizumab treatment in this study for 48 weeks after receiving their last infusion of ocrelizumab, or until their B-cells have repleted whichever is the longer.

Reproductive toxicology studies of ocrelizumab conducted in cynomolgus monkeys are described in the IB. Studies of the effect of ocrelizumab on human reproduction have not been performed. It is not known whether ocrelizumab can cause fetal harm when administered to pregnant women or whether it can affect reproductive capacity. However, since IgG molecules such as ocrelizumab are known to cross the placenta, ocrelizumab may cause fetal CD20 B-cell depletion. It is not known whether ocrelizumab is excreted in breast milk, and what effect this might have on the breast feeding infant. However, since immunoglobulins are found in breast milk, breast feeding mothers are excluded from participation in the study.

Well-controlled reproductive studies with corticosteroids have not been performed in humans but high doses of corticosteroids given during pregnancy have caused hypoadrenalism in newborns.

Regardless of the treatment assignment, a female patient must be instructed to immediately inform the investigator if she becomes pregnant during the study (including the Safety Follow up Period). If she chooses to carry her pregnancy, she must be withdrawn from treatment, and she should enter the Safety Follow up Period. The investigator should report all pregnancies within 24 hours to the Sponsor by means of an eCRF Pregnancy Reporting Form.

As ocrelizumab may cross the placenta and cause B-cell depletion in the neonate, babies born to mothers participating in this study should have an assessment of their lymphocyte counts and be carefully followed until these are within the normal range for the age of the infant. The investigator should counsel the patient as to the risks of continuing with the pregnancy and the possible effects on the fetus. Monitoring of the patient should continue until conclusion of the pregnancy. Informed consent will be sought for the Sponsor to collect information on the health and well being of the baby.

Whether the drug is excreted in the semen is unknown. Therefore, pregnancy occurring in the partner of a male patient participating in the study should also be reported to the investigator, and the investigator should inform the Sponsor. If appropriate, an additional consent form will be provided (subject to ethics committee review) to solicit information about the pregnancy.

## 7.3 Warnings and Precautions

### 7.3.1 Ocrelizumab

Patients should be informed of the risks associated with taking ocrelizumab. Below are listed specific major risks of which the patients should be made aware. Further information on ocrelizumab is given in the current version of IB.

**Infusion-Related Reactions**

All CD20 depleting agents including ocrelizumab have been associated with acute infusion-related reactions (fever, urticaria/rash, chills, rigors, sneezing, angioneurotic edema, throat irritation, nausea, fatigue, headache, dyspnea, rhinitis, vomiting, or flushing cough and bronchospasm, with or without associated hypotension or hypertension). Some of these events have been severe enough to warrant interruption or discontinuation of the infusion. Symptoms are often reversible if the infusion is interrupted and/or patients receive additional treatment with an antihistaminic, acetaminophen, epinephrine or an i.v. corticosteroid. Please refer to Sections 6.1.2 and 6.1.3 for further information.

**Infection Risks**

Prolonged peripheral B-cell depletion is the expected outcome of ocrelizumab treatment. Infection is a potentially serious complication of B-cell depleting therapy and thus requires vigilant attention and prompt investigation and treatment in patients that exhibit signs of infection at any time following anti-CD20 antibody therapy.

Data on the long term risk of infection in MS patients treated with ocrelizumab are not available at this time however in review of the Phase II data in patients with RRMS no imbalance in the overall number of infections or serious infections between placebo and active ocrelizumab arms was observed at week 24. The rate of infections did not increase in ocrelizumab-treated patients at 48 weeks compared with 24 weeks. There was no trend of increase of risk of infection or serious infection for ocrelizumab treated patients with previous interferon treatment (Avonex® for 6 months). There was no trend of increase risk of infections or serious infections with high dose.

The incidence of infections and serious infections was 92.41/100 PY (95% CI 76.59, 111.5) and 3.39 /100 PY (95% CI 1.27, 9.04) in patients exposed to 600 mg dose of ocrelizumab including also patients switching from placebo or Avonex. The incidence of infections and serious infections was 95.61/100 PY (95%CI 73.23, 124.83) and 3.54/100 PY (95% CI 0.89, 14.16) in those exposed to the ocrelizumab 2 x 1000 mg dose. The most common infections in the ocrelizumab treated patients included urinary tract infections, upper respiratory infections, and nasopharyngitis.

Following an analysis of interim data through Week 72, no increase in the rate of serious or non-serious infections has occurred, from Week 24, in patients treated with ocrelizumab. To date, in study WA21493, after over 250 patient years exposure to ocrelizumab, there have been no reports of opportunistic or fatal infections.

Rarely, cases of hepatitis B reactivation, including fulminant hepatitis which have occasionally been fatal, have been reported in NHL patients receiving rituximab. A case of hepatitis B reactivation in a Hep B core Ab positive patient with RA treated with

ocrelizumab has been reported (see Section 5.4.2 for hepatitis screening and monitoring of liver function).

**Other serious, opportunistic and fatal infections have occurred in patients with lupus and RA treated with ocrelizumab in Phase III clinical trials. Data from completed studies regarding infection risks with ocrelizumab treatment in these patient populations are provided in the Investigator Brochure (IB).**

Ocrelizumab should not be administered to patients with an active infection. Physicians should exercise caution when considering the use of ocrelizumab in patients with underlying conditions that may predispose patients to serious infection. Patients who develop signs/symptoms of infection while participating in this trial should be seen immediately, samples taken for appropriate microbiological analysis and appropriate treatment instituted promptly.

Patients should be screened for tuberculosis according to national guidelines. As with other infections, patients with active tuberculosis should not be enrolled, patients with latent tuberculosis should be treated prior to enrollment.

Patients should be warned that the risk of serious infection may be increased by exposure to the medications to be used in this study and should be asked to contact the clinic staff if they start to develop signs of infection. Patients will be provided with a warning card which specifically delineates this risk, which is to be carried on their person at all times in case they are admitted to a hospital which is not participating in the study.

Please refer to the ocrelizumab IB for further information on infection risks.

**Prolonged B-cell Depletion**

In patients with RA that were treated with rituximab, prolonged peripheral B-cell depletion has been reported up to 4 years following a single course of therapy. It is not known whether this will occur following use of ocrelizumab. Patients with prolonged B-cell depletion should be monitored until their B-cells have repleted. (see Section 3.1).

**Progressive multifocal leukoencephalopathy (PML)**

To date there have been no confirmed cases of PML in any MS patient treated with either rituximab or ocrelizumab. No confirmed cases of PML have been observed in any patient receiving ocrelizumab, for any indication.

Among patients treated with rituximab, cases of PML have been observed, in oncology and other autoimmune diseases. The vast majority of these cases have occurred in patients being treated for hematological malignancy and many of these patients were also HIV positive.

There is no currently accepted screening test for PML, neither known interventions that can reliably prevent PML or adequately treat PML. See also Section 7.3.4 for more details. Guidance for diagnosis is given in Section 7.3.4.1.

**Cardiovascular Disorders**

Rarely, cardiac arrhythmias, cardiac ischemia and death due to myocardial dysfunction have been associated with rituximab administration in patients with oncologic disorders. In these cases, the presumed cause was decompensated cardiac disease as a result of cytokine release and/or infusion associated reactions. Patients with a history of cardiac disease (i.e. angina pectoris, cardiac arrhythmias, or congestive heart failure) should be monitored closely during and following infusions. It should be noted that the exclusion criteria exclude enrollment of patients with significant cardiac diseases and congestive heart failure (NYHA III or IV) – see Section 4.3.

**Immunogenicity**

Positive HAHA responses were observed and were most frequent in the lower dose groups in both RA Phase I/II studies; no HAHA responses were observed in the NHL study. In Study ACT2847g, which included doses of 10 mg × 2, 50 mg × 2, 200 mg × 2, 500 mg×2, and 1000 mg × 2, HAHA was observed in 19% and 10% of patients receiving 10 mg × 2 and 50 mg × 2, respectively, vs. 0−5% of patients receiving 200−1000 mg × 2. In Study WA18230, which included doses of 400, 1000, 1500, and 2000 mg, HAHA was observed in 10% and 5% of patients receiving 400 mg and 1000 mg, respectively, and in none of the patients receiving 1500 mg and 2000 mg.

In the RRMS Phase II study (WA21093), no new HAHA seropositivity occurred after initiation of ocrelizumab treatment (300 mg x 2 or 1000 mg x 2).

The clinical significance of positive HAHA is unknown at this time.

**Immunization**

The effect of ocrelizumab on the response to immunization is not known – please refer to Section 4.4.3 for more details; patients receiving ocrelizumab may not mount a humoral response to recall antigens during B-cell depletion. Physicians should review the patient's vaccine history, and be aware that immune response to vaccination could be reduced. Current administration of live vaccines during the Treatment Period and thereafter when B-cells remain depleted, is not allowed.

### 7.3.2 Rebif®

Patients should be informed of the risks associated with taking Rebif®. The most frequent Rebif® adverse reactions of which the patients should be made aware have been summarized in Section 1.1.5.

Depression and suicide ideation are known to occur in increased frequency in the MS and in association with interferon use. Therefore all patients should be advised to immediately report any symptoms of depression and/or suicidal ideation to investigator. Patients exhibiting depression should be monitored closely and treated appropriately. Cessation of double-blind treatment should be considered.

Injection site necrosis has been reported in patients using Rebif®. To minimize the risk of injection site necrosis patients should be advised to:

- use an aseptic injection technique,
- rotate the injection sites with each dose.

If the patient experiences any break in the skin, which may be associated with swelling or drainage of fluid from the injection site, the patient should be advised to consult with their physician before continuing injections with Rebif®/ Rebif® placebo. If the patient has multiple lesions, injections should be discontinued until healing has occurred. Patients with single lesions may continue provided that the necrosis is not too extensive.

Rebif®, like other interferons β, has a potential for causing severe liver injury including acute hepatic failure. The mechanism for the rare symptomatic hepatic dysfunction is not known. No specific risk factors have been identified. Please refer to Section 6.2.2 for additional guidelines.

### 7.3.3 Corticosteroids

Systemic corticosteroids, such as methylprednisolone, can cause immunosuppression, hypertension, diabetes mellitus, cataract, glaucoma, bruising, thinning of the skin, weight gain, psychological changes including psychosis, osteoporosis, accelerated atherosclerosis, increased risk of gastrointestinal bleeding, aseptic necrosis of bone and adrenal insufficiency. Although rare, corticosteroid induced hypersensitivity reactions may occur. They range from minor rashes to the more serious cardiovascular collapse. For additional safety data, refer to the local prescribing information.

### 7.3.4 Progressive Multifocal Leukoencephalopathy

Progressive multifocal leukoencephalopathy (PML) is a potentially fatal neurological condition linked to reactivation of a polyomavirus (JC virus) and active viral replication in the brain. Polyomavirus infection is acquired in childhood and up to 80% of adults demonstrate serological evidence of past infection. Reactivation of JC virus replication with transient viremia or viruria unassociated with clinical symptoms may occur spontaneously in healthy persons. Less frequently, central nervous system symptoms associated with active viral replication in brain tissue is observed. The clinical syndrome is significantly more frequent among immune suppressed patients.

To date there have been no confirmed cases of PML in any MS patient treated with either rituximab or ocrelizumab and no confirmed cases in any patient taking ocrelizumab for any indication. Cases of PML have been reported in patients receiving rituximab in oncology and other autoimmune indications. The vast majority of these cases have occurred in patients being treated for hematological malignancy and many of these patients were also HIV positive. The majority of these patients received rituximab in combination with chemotherapy or as part of a haematopoietic stem cell transplant.

Physicians should consider the diagnosis of PML in any patient presenting with new and/or progressive neurological deficits localized to the cerebral cortex, such as cortical symptoms/signs, behavioral and neuropsychological alteration, retrochiasmal visual

defects, hemiparesis, cerebellar symptoms/signs (e.g., gait abnormalities, limb incoordination), at each visit.

If PML is considered, a neurological consultation should be obtained and treatment suspended until PML has been ruled out. If PML is confirmed in a patient receiving ocrelizumab, no further infusions should be administered and the patient will be withdrawn from treatment (see Section 4.5). No known interventions can reliably prevent PML or adequately treat PML, if it occurs.

It is not known whether the risk of PML is altered by anti-CD20 treatment given as monotherapy. Please refer to Section 7.3.4.1 for guidance on the diagnosis of PML.

PML should be reported as an SAE (with all available information) with immediate notification of the Medical Monitor. Study drug should be withheld and patients with confirmed PML should be withdrawn from the study.

There is no known treatment or cure for PML. Treatment considerations are discussed in the medical literature [55].

#### 7.3.4.1 Guidance for Diagnosis of PML

The following safety monitoring algorithm (Figure 3) will be implemented in this study. This algorithm was implemented in the Phase I/II studies with rituximab in patients with MS and is consistent with the algorithm used in natalizumab studies.

Comprehensive neurological assessments will be performed every 12 weeks at the regular study visits. Patients will be required to undergo a neurological exam for calculation of an Expanded Disability Status Scale (EDSS) score every 12 weeks. This requires that Functional System Score (FSS) also be determined. The examination to calculate the FSS includes cognitive, visual and motor assessments, the neurological systems most often affected by PML, as well as assessments of other neurological systems.

In the eCRF, the investigator will record the presence or absence of neurological deficits localized to the cerebral cortex (e.g., cortical symptoms/signs, behavioral and neuropsychological alteration, retrochiasmal visual defects, hemiparesis), cerebellar symptoms/signs (e.g., gait abnormalities, limb incoordination), at each visit. Presence of such neurological findings will be recorded as adverse events. *If a diagnosis for the deficits is identified, the symptoms should be replaced by the diagnosis in the adverse event eCRF.*

In addition to the neurological evaluation at regular visits, patients will undergo a telephone interview between the study visits by site personnel familiar with the patient(s). The purpose of this interview is to identify new or worsening neurological symptoms that warrant an unscheduled visit (Appendix 4). Partners or caregivers of study patients, if applicable, will be informed on symptoms and signs that may be suggestive of PML and should be instructed to contact the site, should any such signs or symptoms appear.

In the event that new or worsening neurological symptoms are considered during the telephone interview, a neurological evaluation will be conducted. Should a non MS

etiology, such as PML, be considered, further assessments should be done. The evaluation of PML may include a brain MRI scan and CSF analysis per the proposed treatment algorithm (see Figure 3).

**The following clinical guidance is provided:**

**Treatment of Relapse and Other Neurological Symptoms**

- As in all MS studies, new or recurrent neurological symptoms occurring in study patients should prompt careful clinical evaluation.
- Given the occurrence of PML in immunocompromised patients who had received rituximab, PML should be considered in patients who develop worsening neurological signs or symptoms.
- There are no pathognomonic signs or symptoms that distinguish MS from PML, but there are certain clinical features that may help differentiate between the two conditions (see Table 10).
- In addition to PML and MS, other CNS conditions (e.g., stroke, migraine, etc.) should be considered when evaluating a patient with new neurological changes.
- Relapses should be managed according to the study protocol.
- Corticosteroid treatment should only be considered for cases in which PML is unlikely on clinical grounds and when the severity of the relapse warrants such treatment. Lack of response to corticosteroids should trigger further investigation.

**Action Steps if PML is Suspected**

- If the clinical presentation is suggestive of PML, further investigations should include brain MRI evaluation as soon as possible. If MRI evaluation reveals lesions suspicious for PML (see Figure 3) a lumbar puncture with evaluation of the cerebrospinal fluid (CSF) for the detection of JCV DNA should be undertaken. A diagnosis of PML can potentially be made by evaluating clinical and MRI findings plus the identification of JCV in the CSF.

Please note: In the event that PML is suspected, an additional plasma, urine, as well as a CSF sample should be obtained for JCV analysis. Samples will be analyzed upon receipt and the results will be provided directly to the investigational site and to the Sponsor. Storage conditions and shipment instructions will be provided.

**MRI Assessment**

- Although there are no pathognomonic findings that differentiate PML from MS, a brain MRI scan that includes fluid-attenuated inversion recovery (FLAIR) and T2-weighted and T1-weighted sequences, with and without gadolinium, should be performed to assess patients with neurological changes suggestive of PML – see Figure 3.
- Comparison with a baseline scan may assist with interpretation of the findings on the newly acquired MRI (see Table 10 for differences in lesion characteristics that may help differentiate between PML and MS).

### CSF Assessment

- The detection of JCV DNA in the CSF of a patient with clinical and MRI features suggestive of PML establishes the diagnosis of PML.
- If JCV DNA is not detected in CSF and if clinical suspicion of PML remains high, a repeat lumbar puncture should be performed.
- If diagnosis remains uncertain and suspicion of PML remains high, a brain biopsy may be considered to establish a definitive diagnosis.

**Figure 3: Diagnostic Algorithm for PML**

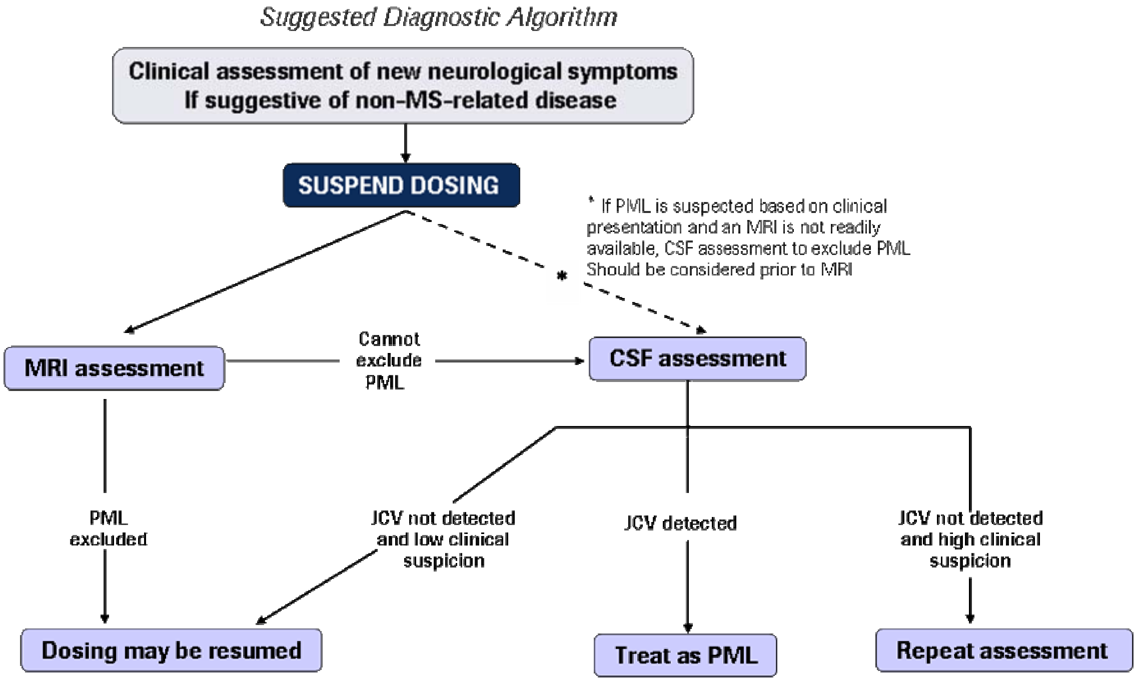


**Table 9: Clinical features to distinguish between MS relapse and PML***

| | MS relapse | PML |
|---|---|---|
| ***Onset*** | Acute | Subacute |
| ***Evolution*** | - Over hours to days<br>- Normally stabilizes<br>- Resolves spontaneously or with treatment | - Over weeks<br>- Progressive |
| ***Clinical presentation*** | - Optic neuritis<br>- Incomplete myelopathy or partial myelitis | - Cortical signs and symptoms<br>- Behavioral and neuropsychological alterations<br>- Retrochiasmal visual deficits<br>- Hemiparesis |

**Adapted from Kappos L et al [56].*

## Table 10: MRI Lesion Characteristics Typical of PML and MS

| Feature | MS (relapse) | PML |
|---|---|---|
| **Location of new lesions** | Mostly focal; affect entire brain and spinal chord, in white and possibly gray matter | Diffuse lesions, mainly subcortical and rarely periventricular, located almost exclusively in white matter, although occasional extension to gray matter has been seen; posterior fossa frequently involved (cerebellum) |
| **Borders** | Sharp edges; mostly round or finger-like in shape (especially periventricular lesions), confluent with other lesions; U-fibers may be involved | Ill-defined edges; irregular in shape; confined to white matter; sparing gray matter; pushing against the cerebral cortex; U-fibers destroyed |
| **Mode of extension** | Initially focal; lesions enlarge within days or weeks and later decrease in size within months | Lesions are diffuse and asymmetric, extending homogeneously; no confluence with other lesions; confined to white-matter tracks, sparing the cortex; continuous progression |
| **Mass effect** | Acute lesions show some mass effect | No mass effect even in large lesions (but lesion slightly abuts cerebral cortex) |
| **On T2-weighted sequence** | - Acute lesions: hyperintense center, isointense ring, discrete hyperintensity outside the ring structure<br>- Subacute and chronic lesions: hyperintense with no ring structure | Diffuse hyperintensity, slightly increased intensity of newly involved areas compared with old areas, little irregular signal intensity of lesions |
| **On T1-weighted sequence** | Acute lesions: densely hypointense (large lesions) or isointense (small lesions); increasing signal intensity over time in 80%; decreasing signal intensity (axonal loss) in about 20% | Slightly hypointense at onset, with signal intensity decreasing over time and along the affected area; no reversion of signal intensity |
| **On FLAIR sequence** | Hyperintense, sharply delineated | Hyperintensity more obvious; true extension of abnormality more clearly visible than in T2-weighted images |
| **With enhancement** | - Acute lesions: dense homogeneous enhancement, sharp edges<br>- Subacute lesions: ring enhancement<br>- Chronic lesions: no enhancement | Usually no enhancement, even in large lesions; in patients with HIV, some peripheral enhancement is possible, especially under therapy. |
| **Atrophy** | Focal atrophy possible due to focal white-matter degeneration; no progression | No focal atrophy |

*Adapted from Yousry TA et al [57].*

# 8. STATISTICAL CONSIDERATIONS AND ANALYTICAL PLAN

Full details of all statistical issues and planned statistical analyses will be specified in a separate Data Analysis Plan (DAP), which will be finalized prior to the locking and unblinding of the study database.

## 8.1 Study Endpoints

### 8.1.1 Primary Efficacy Endpoint

The primary efficacy endpoint is annualized protocol-defined relapse rate by two years (96 weeks).

Protocol-defined relapse, is defined as the occurrence of new or worsening neurological symptoms attributable to MS. Symptoms must persist for >24 hours and should not be attributable to confounding clinical factors (e.g. fever, infection, injury, adverse reactions to medications) and immediately preceded by a stable or improving neurological state for least 30 days. The new or worsening neurological symptoms must be accompanied by objective neurological worsening consistent with an increase of at least half a step on the EDSS scale, or 2 points on one of the appropriate FSS, or 1 point on two or more of the appropriate FSS. The change must affect the selected FSS (i.e., pyramidal, ambulation, cerebellar, brainstem, sensory, or visual). Episodic spasms, sexual dysfunction, fatigue, mood change or bladder or bowel urgency or incontinence will not suffice to establish a relapse. Please note: *Sexual dysfunction* and *Fatigue* will not be scored.

Adjudication of protocol-defined relapses will be performed by the Sponsor based on pre-specified criteria, applied to data collected by investigator, in a blinded fashion.

### 8.1.2 Secondary Efficacy Endpoints

The secondary efficacy endpoints are:

- The time to onset of sustained disability progression for at least 12 weeks during the 96-week comparative treatment period.
- The time to onset of sustained disability progression for at least 24 weeks during the 96-week comparative treatment period.
- The proportion of relapse-free patients by 96 weeks.
- The change in total T2 lesion volume as detected by brain MRI from baseline to Week 96.
- The total number of new, and/or enlarging T2 hyperintense lesions as detected by brain MRI at week 24, week 48 and week 96.
- The change in Multiple Sclerosis Functional Composite Scale (MSFCS) score from baseline to Week 96.
- The change in brain volume as detected by brain MRI from Week 24 to Week 96.

### 8.1.3 Exploratory Efficacy Endpoints

The exploratory efficacy endpoints in this study will include, but may not be limited to:

- The change in low contrast visual acuity from baseline to Weeks 48 and 96.
- The change in the Symbol Digit Modalities Test from baseline to Weeks 48 and 96.

- The annualized relapse rate, based on clinical and protocol-defined relapses at the end of the 96-week comparative treatment period.
- The total number of T1 gadolinium-enhanced lesions as detected by brain MRI at weeks 24, 48 and 96.
- The change in brain volume as detected by brain MRI from baseline to Week 96.
- The change in brain volume as detected by brain MRI from week 48 to Week 96.
- The change in Multiple Sclerosis Functional Composite Scale (MSFCS) score from baseline to Week 48.
- The cumulative change in EDSS scores, measured in area under the curve (AUC) by Week 96.
- The change in EDSS from baseline to Weeks 48 and 96.
- The change in timed 25 foot walk from baseline to Weeks 48 and 96.
- The change in 9-hole peg test from baseline to Weeks 48 and 96.
- The change in paced auditory serial addition test (PASAT) from baseline to Weeks 48 and 96.
- The time to onset of sustained 20% increase in 9-hole peg test for at least 12 weeks.
- The time to onset of sustained 20% increase in timed 25 foot walk for at least 12 weeks.
- Patient Reported Outcomes (PROs): Modified Fatigue Impact Scale (MFIS), EuroQol instrument (EQ-5D), Center for Epidemiology Studies Depression Scale (CES-D) and Medical Outcomes Study 36-Item Short Form Survey Instrument (SF-36) at baseline, Week 48 and Week 96.
- The change in Karnofsky Performance Status Scale (clinician-reported version) from baseline to Weeks 24, 48, 72 and 96.

### 8.1.4 Safety

Safety will be assessed through regular neurological and physical examinations, vital signs, ECG, and the occurrence of adverse events. In addition, the following will be examined:

- Non-MS pathology at all available MRI scans.
- Columbia-suicide severity rating scale (C-SSRS).
- Complete routine hematology, chemistry and urinalyses;
- Circulating B-cell subsets, T cells, natural killer cells and other leukocytes;
- Plasma immunoglobulins;
- HAHA;
- Antibody titers for mumps, rubella, varicella, and Streptococcus pneumoniae;
- Serial pregnancy tests [serum/urine β subunit human chorionic gonadotropin (β hCG)] will be performed in women of child bearing potential.
- JC virus (JCV) plasma/urine sampling – only if deemed necessary.

## 8.2 Statistical and Analytical Methods

Prior to unblinding the treatment groups, a DAP will be produced that will contain full details of all planned analyses. An outline of the planned analyses is described below. A database lock will occur when the last patient has completed his Week 96 assessment,

i.e. completed his $4^{th}$ treatment cycle, for the purposes of the primary efficacy and safety analysis. The treatment assignments will be unblinded to the Sponsor at this point for the purposes of data analysis.

All analyses comparing ocrelizumab group with Rebif® group will use data collected for each patient up to and including Week 96.

The time to onset of sustained disability progression for at least 12 wks during the 96-week comparative treatment period and the time to onset of sustained disability progression for at least 24 wks during the 96-week comparative treatment period will be analyzed using pooled data across the two identical studies Sponsor plan to run as a part of Phase III program, with respect to ocrelizumab group vs. Rebif® group.

All eligible patients will be randomized to treatment stratified by region (United States versus ROW) and baseline EDSS (< 4.0 versus ≥ 4.0). All analyses will also be stratified by region (United States versus ROW) and baseline EDSS (< 4.0 versus ≥ 4.0).

All analyses, summaries and listings will be performed using SAS® software (Version 8.2 or higher in a UNIX environment).

### 8.2.1 Primary Efficacy Analysis

The primary efficacy analysis for this trial will compare annualized protocol-defined relapse rate by 96 weeks between ocrelizumab group and Rebif® group. The annualized relapse rates by 96 weeks will be calculated using negative binomial model, adjusting for region (United States versus ROW) and baseline EDSS (< 4.0 versus ≥ 4.0). The adjusted annualized relapse rates and the two-sided 95% confidence intervals for the relapse rates will be presented along with the p-value.

Other sensitivity analyses may also be performed for the primary efficacy endpoint (and documented in the DAP).

### 8.2.2 Secondary Efficacy Analyses

Secondary efficacy endpoints will be tested in hierarchal order (listed as following), all at alpha = 0.05 level. The first secondary efficacy endpoint will be tested if and only if the primary endpoint has reached the significant level at 0.05 (e.g., P-value ≤ 0.05). The rest of the secondary endpoints will be tested if and only if the secondary endpoint list ahead of it has reached the significant level at 0.05.

**Figure 4: Hierarchal Order of Key Efficacy Endpoints**

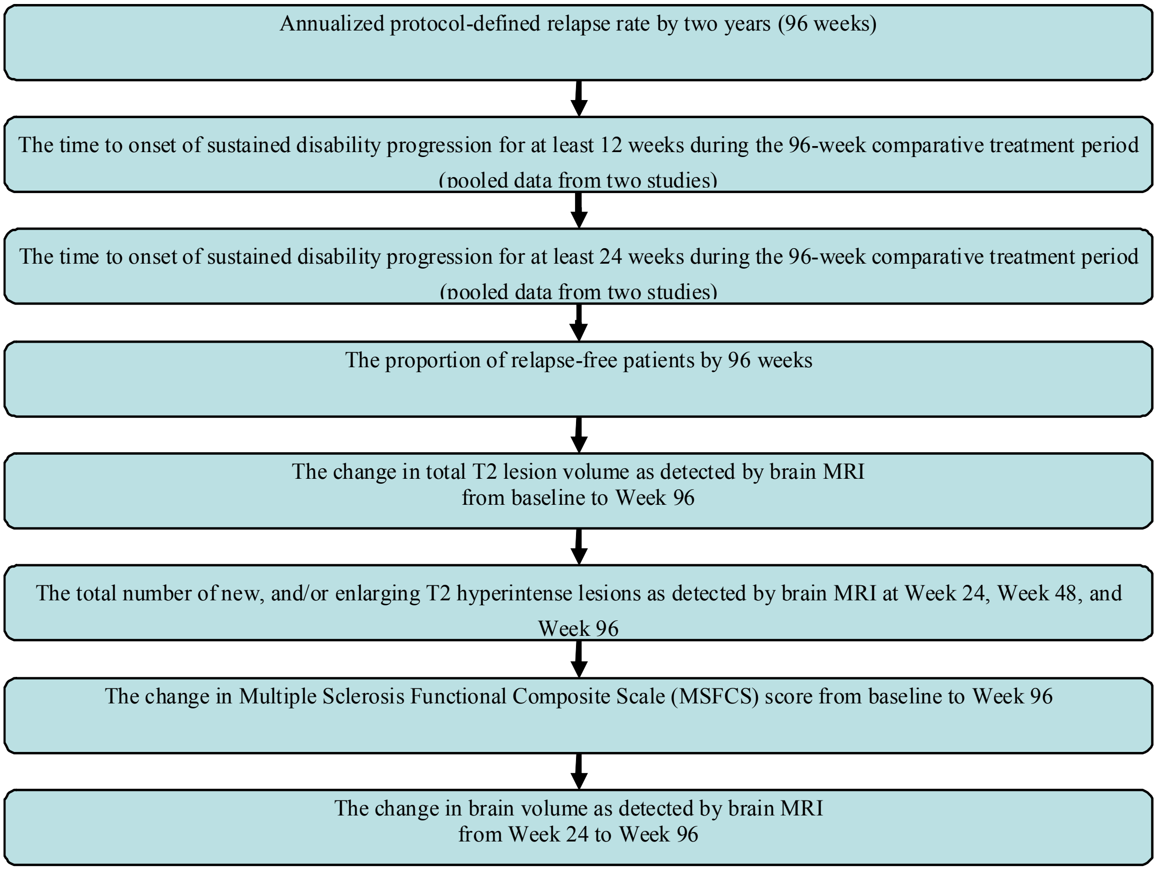


#### *8.2.2.1 The Time to Onset of Sustained Disability Progression for At Least 12 Weeks During the 96-Week Comparative Treatment Period*

Time to sustained disability progression (12 week confirmation) is defined as the time from Baseline to the first disability progression, which is confirmed at the next regularly scheduled visit ≥ 84 days after the initial disability progression. Disability progression is defined as an increase of ≥ 1.0 point from baseline EDSS, if the baseline EDSS is between 0 and 5.5 points (inclusive), or an increase of ≥ 0.5 points, if the baseline EDSS is > 5.5 points. Please note that the inclusion criteria of EDSS (0–5.5) only applies to screening EDSS. It is still possible that a patient's baseline EDSS (derived based on both screening and day 1 EDSS results) is >5.5. The non-confirmatory EDSS assessments (if any) between the initial and confirmation of disability progression should be at least as high as the minimum change required for progression. Patients who did not have sustained disability progression by Week 96 visit, time of early discontinuation of treatment, or loss to follow up will be censored at the date of their last EDSS assessment.

Data from the two studies with respect to ocrelizumab group vs Rebif® group will be pooled for analysis of this endpoint. To assess the validity of pooling data across the two RMS studies, demographic and baseline characteristics will be compared by trials. The treatment effect (hazard ratio and CI) for sustained disability progression within each trial

will be compared between the two trials. In interpreting the trial comparisons with a view toward assessing the validity of the pooled dataset, the primary interest is in confirming that the treatment effect is qualitatively similar across the two studies – positive treatment effects (the estimated hazard to have sustained disease progression in patients treated with OCR is numerically smaller than that in patients treated with Rebif) are shown in both studies. If the results from the two studies are not qualitatively similar (e.g., positive treatment effect is only shown in one study), data will not be pooled.

Time to sustained disability progression for ocrelizumab group and Rebif® group (across the studies) will be compared using a two-sided log-rank test stratifying by region (United States versus ROW), baseline EDSS (< 4.0 versus ≥ 4.0), and study. The proportion of patients with sustained disability progression will be estimated using Kaplan-Meier methodology. The overall hazard ratio will be estimated using a stratified Cox regression model with the same stratification factors used in the stratified log-rank test above.

#### 8.2.2.2 The Time to Onset of Sustained Disability Progression for At Least 24 Weeks During the 96-Week Comparative Treatment Period

Time to sustained disability progression between ocrelizumab group and Rebif® group using a 24 week confirmation window for disability progression will be compared using the same analysis method for time to sustained disability progression using a 12 week confirmation window. Time to sustained disability progression (24 week confirmation) is defined as the time from Baseline (Day 1) to the first disability progression, which is confirmed at the next regularly scheduled visit ≥ 161 days after the initial disability progression.

#### 8.2.2.3 The Proportion of Relapse-Free Patients by 96 Weeks

The proportion of patients who remain relapse-free by Week 96 will be analyzed using a Cochran-Mantel-Haenszel $\chi 2$ test stratified by region (United States versus ROW) and baseline EDSS (< 4.0 versus ≥ 4.0) to compare ocrelizumab group to Rebif®. The difference in the proportions, along with the 95% confidence interval for the difference will be presented. Relative risk (of having relapse) will also be produced for ocrelizumab group compared to Rebif®, along with corresponding confidence intervals.

#### 8.2.2.4 The Change in Total T2 Lesion Volume as Detected by Brain MRI from Baseline to Week 96

The change in total volume of T2 lesions on MRI scans of the brain from baseline to Week 96 will be compared between ocrelizumab group and Rebif® group using the ranked ANCOVA[1] adjusting baseline T2 lesion volume, baseline Gd lesion (present or not), region (United States versus ROW) and baseline EDSS (< 4.0 versus ≥ 4.0). The median change in total volume of T2 lesions for each treatment group and the corresponding 95% confidence interval for the median will be presented.

[1] Categorical Data Analysis using the SAS system. Stokes, Davis, and Koch

#### *8.2.2.5 The Total Number of New, and/or Enlarging T2 Hyperintense Lesions as Detected by Brain MRI at Week 24, Week 48 and Week 96*

Negative binomial model adjusting baseline Gd lesion (present or not), region (United States versus ROW) and baseline EDSS (< 4.0 versus ≥ 4.0) will be applied to compare the difference between ocrelizumab and Rebif® groups. The median change in the total number of new, and/or enlaring T2 hyperintense lesions for each treatment group and the corresponding 95% confidence interval for the median will be presented.

#### *8.2.2.6 The Change in Multiple Sclerosis Functional Composite Scale (MSFCS) Score from Baseline to Weeks 96*

The change in MSFCS from baseline to Week 96 will be compared between ocrelizumab group and Rebif® group using ranked ANCOVA adjusting baseline MSFCS, region (United States versus ROW) and baseline EDSS (< 4.0 versus ≥ 4.0). The median change in MSFCS for each treatment group and the corresponding 95% confidence interval for the median will be presented.

#### *8.2.2.7 The Change in Brain Volume as Detected by Brain MRI Scan from Week 24 to Week 96*

The change in brain volume as detected by brain MRI from week 24 to Week 96 will be compared between ocrelizumab group and Rebif® group using the ranked ANCOVA adjusting baseline brain volume, baseline Gd lesion (present or not), region (United States versus ROW) and baseline EDSS (< 4.0 versus ≥ 4.0). The median change in brain volume for each treatment group and the corresponding 95% confidence interval for the median will be presented.

### 8.2.3 Exploratory Analyses

The exploratory endpoints will be summarized using tables, listings and graphs, where appropriate. Full details of the derivations and analyses of all exploratory endpoints will be provided in the DAP.

### 8.2.4 Sample Size

The sample size for this study has been estimated based on data from previous RRMS trials, with the use of two-sided tests with an experiment-wise alpha of 0.05. The annualized rate of relapse among patients receiving ocrelizumab at 96 weeks is predicted to be 0.165 (standard deviation of approximately 0.60), as compared with 0.33 (standard deviation of approximately 0.80) among patients receiving the control treatment, Rebif® (this represents a relative reduction of 50% on ocrelizumab compared to the active comparator). For the annualized relapse rate, a t-test has been used to determine the sample size between ocrelizumab and the control arm. The sample size of 400 patients per arm provides 84 percent power, maintaining the type I error rate of 0.05, and assuming a drop out rate of 20 percent approximately (assuming relative reduction among patients drop out is 25%).

For sustained disease progression, a two group test of equal exponential survival with exponential dropout is used to determine the sample size. Assuming the 2 year sustained disability progression rate is 18% for the Rebif® arm and 12.6% for the ocrelizumab arm

(this represents a relative reduction of 30% on ocrelizumab compared to the active comparator), and assuming a drop out rate of 20 percent over 2 years approximately, the sample size of 400 per arm will provide 80 percent power, maintaining the type I error rate of 0.05 based on the pooled analysis of two RMS trials (800 patients treated with ocrelizumab 600 mg and 800 patients treated with Rebif®).

### 8.2.5 Hypothesis Testing

The hypotheses to be tested are:

$H_0$ (null hypothesis): There is no statistically significant difference in annualized protocol-defined relapse rate at two years between ocrelizumab group and Rebif® group.

$H_1$ (alternative hypothesis): There is a statistically significant difference in annualized protocol-defined relapse rate at two years between ocrelizumab group and Rebif® group.

Annualized protocol-defined relapse rate at two years between the ocrelizumab group and Rebif® group will be compared using negative binomial model adjusting region (United States versus ROW) and baseline EDSS (< 4.0 versus ≥ 4.0). If the test result for comparing 600 mg ocrelizumab and Rebif® groups is statistically significant at $\alpha < 0.05$ level (two-sided test), we will conclude that the 600 mg ocrelizumab group demonstrated a superior effect of reducing Annualized protocol defined relapse rate, when compared to Rebif® group.

Similar hypotheses will also be tested for the secondary efficacy parameters. Methods for handling multiplicity issues related to secondary endpoints will be described in the DAP.

### 8.2.6 Analysis Populations

One patient population will be defined for the purpose of the safety analysis and two for the efficacy analysis. All efficacy analyses will be performed using the intent-to-treat (ITT) population. The per-protocol (PP) population will be used for all primary and secondary efficacy analyses in order to evaluate the influence of major protocol violators and as a sensitivity check to the ITT analysis.

#### *8.2.6.1 Safety Population*

This population will be used for all summaries of safety data. The safety population will include all patients who received any study drug and provided at least one assessment of safety. Randomized patients that receive incorrect therapy from that intended will be summarized in the group according to the therapy actually received. Patients who are not randomized, but who receive study drug will be included in the safety population and summarized according to the therapy actually received.

#### *8.2.6.2 Intent-to-Treat Population*

All randomized patients who have received any study drug will be included in the intent–to-treat population. Patients who prematurely withdraw from the study for any reason and for whom an assessment is not performed for whatever reason will still be included in the ITT analysis. Patients who receive an incorrect therapy from that which is intended will be summarized according to their randomized treatment.

#### 8.2.6.3 Per Protocol Population

The per protocol population will include all patients in the ITT population adhering to the protocol. Patients may be excluded if they significantly violate the inclusion/exclusion criteria or deviate from the study plan. Specific reasons for warranting exclusion will be agreed and documented in the DAP prior to unblinding of the treatment groups. Only those patients with violations that are deemed to potentially affect the efficacy of study treatment will be excluded from the per protocol population. Patients who receive an incorrect therapy from that intended will be excluded from the per protocol population.

### 8.2.7 Interim Analysis

No formal efficacy interim analyses are planned.

### 8.2.8 Safety Data Analysis

The safety population will include all patients who received any study drug and provided at least one post-dose safety assessment. All safety parameters will be summarized and presented in tables based on this safety population. Randomized patients that receive incorrect therapy from that intended will be summarized in the group according to the therapy actually received. Patients who are not randomized, but who receive study drug will be included in the safety population and summarized according to the therapy actually received.

The safety data will be listed and summarized at determined cut off points, e.g. using data for each patient up to Week 96, using all available data at the Week 96 database lock for the primary analysis.

All adverse events will be coded and tabulated by system organ class and preferred term for individual events within each system organ class, and will be presented in descending frequency. Adverse events will also be tabulated by severity and relationship to the study medication. Serious adverse events will be summarized separately. Results of C-SSRS will be listed for each visit by patient.

Non-MS pathology reported by local safety radiologist will be summarized by treatment group.

Associated laboratory parameters such as hepatic function, renal function and hematology values will be grouped and presented together. Correlation between low IgG and IgM and infections will be presented separately. Marked abnormalities will also be flagged. Marked abnormalities will be tabulated for each laboratory test by treatment group.

Analysis of HAHA to ocrelizumab will be summarized graphically and descriptively. Correlation between presence of HAHA and IRR/B-cell depletion will be presented descriptively.

The results of vital sign, physical examination and ECG will be included in individual patient listings. Change from baseline in vital signs will be summarized by groups.

An external, independent DMC will review safety data throughout the study and will convene at least three times per year. Analyses required for the DMC data review will be performed as described in the DMC Charter and DMC data handling plan.

### 8.2.9 Safety Follow-up Period

Data from this period will be analyzed to provide information on the maintenance effect and the potential withdrawal effect of ocrelizumab. In addition, data will be analyzed to provide information concerning the long-term safety of ocrelizumab. Data will be summarized and tables and listings will be produced.

### 8.2.10 Other Analyses

#### *8.2.10.1 Pharmacokinetic Analysis*

**Pharmacokinetic Parameters**

Ocrelizumab serum concentration-time data will be modeled using a population approach. The primary population PK parameters (Clearances and Volumes) for ocrelizumab will be estimated by means of NONMEM analysis of the sparse PK data. Clearances with associated inter-patient variability may be characterized by a saturable and non-saturable clearance as well as an intercompartmental clearance depending on the final structural model. Volumes with associated inter-patient variability may be characterized by central and peripheral volumes depending on the final structural model. Exposure (AUC) to ocrelizumab will be estimated. The selection of other parameters will depend on the final PK model used for this analysis.

**Pharmacokinetic Analysis**

Nonlinear mixed effects modeling (with software NONMEM [58]) will be used to analyze the sparse sampling dose-concentration-time data of ocrelizumab. Patients who have measurable concentrations of ocrelizumab will be included in the PK analysis unless major protocol deviations or unavailability of information (e.g. exact blood sampling time) occurred which may interfere with PK evaluation. The PK data of this study may be pooled with more extensive data from other studies. Population PK parameters (Clearances and Volumes) will be estimated and the influence of covariates, such as age, gender, weight, HAHA, and baseline CD19 lymphocytes, on these parameters will be investigated.

Details of the mixed-effects modeling analyses will be described in a Modeling and Simulation Analysis Plan and results will be reported separately.

#### *8.2.10.2 Pharmacodynamic Analysis*

The relationship between individual ocrelizumab exposure and selected safety and efficacy parameters will be analyzed and explored, in order to characterize the exposure/dose response curve of ocrelizumab. This may include but is not limited to annualized relapse rate, T1 and T2 lesions at week 96, IRRs, infections, and other AEs or safety parameters of interest. Other exploratory analyses may be performed to assess the possible relationship between PD markers e.g. CD19 count, PK, and clinical response.

#### 8.2.10.3 Roche Clinical Repository / Protein Biomarker Samples

Additional blood samples for serum and/or plasma analyses will be taken for research purposes subject to discretionary approval from each center's IRB/IEC and the patient's specific written consent. These samples will be used to identify dynamic biomarkers to help us better understand the pathogenesis of RMS and response to treatment with ocrelizumab. Such future biomarkers have yet to be determined but may include circulating biochemical markers in blood including cytokines as well as peripheral blood gene expression patterns. Exploratory statistical data analyses may include assessments for possible relationships between these biomarker levels, PK and clinical response.

# 9. DATA COLLECTION, MANAGEMENT AND QUALITY ASSURANCE

The overall procedures for quality assurance of clinical study data are described in the Sponsor's (or designee) Standard Operational Procedures.

Data for this study will be recorded via an Electronic Data Capture (EDC) system using electronic Case Report Forms. It will be transcribed by the site from the paper source documents onto the eCRF. In addition, EDSS, MSFC, C-SSRS, Karnofsky Performance Status Scale and patient reported outcomes will be collected via an electronic interface. The data will be transmitted from the electronic interface to a central database that will later be transferred to the Sponsor (or designee). Only if a technical failure prevents the ability to collect data electronically, the paper forms may be used.

Accurate and reliable data collection will be assured by verification and cross–check of the eCRFs against the investigator's records by the study monitor (source document verification), and the maintenance of a drug–dispensing log by the investigator.

A comprehensive validation check program utilizing front-end checks in the eCRF/electronic interface and back-end checks in the data base will verify the data and discrepancies will be generated accordingly. These are transferred electronically to the site for resolution by the investigator.

Throughout the study the Study Management Team (SMT) will review data according to the EDC Cleaning Process as described in the Data Management Plan.

## 9.1 Assignment of Preferred Terms and Original Terminology

For classification purposes, preferred terms will be assigned by the Sponsor to the original terms entered on the eCRF, using the most up-to-date version of the Medical Dictionary for Regulatory Activities (MedDRA) terminology for adverse events and diseases and the International Non-proprietary Name (INN) Drug Terms and Procedures Dictionary for treatments and surgical and medical procedures.

# 10. STUDY COMMITTEES

**Steering Committee**

An external Steering Committee will provide general guidance, assist with liaison to investigators and oversee any external communication of the results of the study.

**Data Monitoring Committee (DMC)**

An external independent Data Monitoring Committee (DMC) will be chartered to review safety data throughout the study and make recommendations regarding continuation, termination, or modification of the study. Regularly scheduled safety data reviews will occur at least three times per year after the first patient is enrolled.

Any safety event that requires unblinding of study treatment allocation will be immediately reported to the DMC and to the health authorities in an expedited safety report. The DMC may request and review any additional reports outside of the planned analyses at any time if deemed necessary to ensure the safety of patients. The safety evaluations will be conducted on parameters specified within the DMC charter and may vary depending on the requirements and requests of the DMC.

The details of the DMC roles and responsibilities, scope of work and the logistics of the DMC activities will be outlined in a DMC Charter. The purpose of the DMC interim analyses is primarily safety evaluation, and the study may be stopped or amended because of significant safety concerns.

## 11. REFERENCES

1. Neurological Disorders: Public health Challenges WHO, WHO Press, 1211 Geneva 27, Switzerland, assessed on line on July 6, 2010. http://www.who.int/mental_health/neurology/chapter_3_a_neuro_disorders_public_h_challenges.pdf

2. McDonald WI, Compston A, Edan G, et al. Recommended diagnostic criteria for multiple sclerosis: guidelines from the International Panel on the Diagnosis of Multiple Sclerosis. Ann Neurol 2001;50:121–7.

3. Polman CH, Reingold SC, Banwell B, et al. Diagnostic criteria for multiple sclerosis: 2010 revisions to the "McDonald Criteria". Ann Neurol 2011;69:292–302.

4. Lublin FD and Reingold SC. Defining the clinical course of multiple sclerosis: results of an international survey. National Multiple Sclerosis Society (USA) Advisory Committee on Clinical Trials of New Agents in Multiple Sclerosis. Neurology 1996;46:907–11.

5. Filippini G, Munari L, Incorvaia B, Ebers GC, Polman C, D’Amico R, et al. Interferons in relapsing remitting multiple sclerosis: a systematic review. Lancet 2003;361:545–52.

6. Compston A, Coles A. Multiple sclerosis. Lancet 2008; 372:1502-17.

7. Hauser SL, Waubant E, Arnold DL, et al; HERMES Trial Group. B-cell depletion with rituximab in relapsing-remitting multiple sclerosis. N Engl J Med 2008;358:676–88.

8. Sidén A. Isoelectric focusing and crossed immunoelectrofocusing of CSF immunoglobulins in MS. J Neurol 1979;221:39–51.

9. Meinl E, Krumbholz M, Hohlfeld R. B lineage cells in the inflammatory central nervous system environment: migration, maintenance, local antibody production, and therapeutic modulation. Ann Neurol 2006;59:880–92.

10. Franciotta D, Salvetti M, Lolli F, Serafini B, Aloisi F. B cells and multiple sclerosis. Lancet Neurol. 2008;7:852-8.

11. McFarland HF. The B cell--old player, new position on the team. N Engl J Med 2008;358:664–5.

12. Owens GP, Kraus H, Burgoon MP, et al. Restricted use of VH4 germline segments in an acute multiple sclerosis brain. Ann Neurol 1998;43:236–43.

13. Baranzini SE, Jeong MC, Butunoi C, Murray RS, Bernard CC, Oksenberg JR. B cell repertoire diversity and clonal expansion in multiple sclerosis brain lesions. J Immunol. 1999;163:5133-44.

14. Qin Y, Duquette P, Zhang Y, et al. Clonal expansion and somatic hypermutation of V(H) genes of B cells from cerebrospinal fluid in multiple sclerosis. J Clin Invest 1998;102:1045–50.

15. Colombo M, Dono M, Gazzola P, et al. Accumulation of clonally related B lymphocytes in the cerebrospinal fluid of multiple sclerosis patients. J Immunol. 2000;164:2782-9.

16. Ritchie AM, Gilden DH, Williamson RA, et al. Comparative analysis of the CD19+ and CD138+ cell antibody repertoires in the cerebrospinal fluid of patients with multiple sclerosis. J Immunol 2004;173:649–56.

17. Lambracht-Washington D, O'Connor KC, et al. Antigen specificity of clonally expanded and receptor edited cerebrospinal fluid B cells from patients with relapsing remitting MS. J Neuroimmunol 2007;186:164–76.

18. Owens GP, Winges KM, Ritchie AM, et al. VH4 gene segments dominate the intrathecal humoral immune response in multiple sclerosis. J Immunol 2007;179:6343–51.

19. Monson NL, Brezinschek HP, Brezinschek RI, et al. Receptor revision and atypical mutational characteristics in clonally expanded B cells from the cerebrospinal fluid of recently diagnosed multiple sclerosis patients. J Neuroimmunol 2005;158:170–81.

20. Obermeier B, Mentele R, Malotka J, et al. Matching of oligoclonal immunoglobulin transcriptomes and proteomes of cerebrospinal fluid in multiple sclerosis. Nat Med 2008;14:688–93.

21. Reindl M, Linington C, Brehm U, et al. Antibodies against the myelin oligodendrocyte glycoprotein and the myelin basic protein in multiple sclerosis and other neurological diseases: a comparative study. Brain 1999;122:2047–56.

22. Egg R, Reindl M, Deisenhammer F, et al. Anti-MOG and anti-MBP antibody subclasses in multiple sclerosis. Mult Scler 2001;7:285–9.

23. Andersson M, Yu M, Söderström M, et al. Multiple MAG peptides are recognized by circulating T and B lymphocytes in polyneuropathy and multiple sclerosis. Eur J Neurol 2002;9:243–51.

24. Genain CP, Cannella B, Hauser SL, et al. Identification of autoantibodies associated with myelin damage in multiple sclerosis. Nat Med 1999;5:170–5.

25. Serafini B, Rosicarelli B, Magliozzi R, et al. Detection of ectopic B-cell follicles with germinal centers in the meninges of patients with secondary progressive multiple sclerosis. Brain Pathol 2004;14:164–74.

26. Magliozzi R, Howell O, Vora A, et al. Meningeal B-cell follicles in secondary progressive multiple sclerosis associate with early onset of disease and severe cortical pathology. Brain 2007;130:1089–104.

27. Kutzelnigg A, Faber-Rod JC, Bauer J, et al. Widespread demyelination in the cerebellar cortex in multiple sclerosis. Brain Pathol 2007;17:38–44.

28. Aloisi F and Pujol-Borrell R. Lymphoid neogenesis in chronic inflammatory diseases. Nat Rev Immunol 2006;6:205–17.

29. Howell OW, Reeves C, Magliozzi R, et al. The incidence of meningeal B-cell follicles in secondary progressive multiple sclerosis: a neuropathological study of 96 cases (abstract). Mult Scler 2009;15:S5.

30. Bar-Or A, Calabresi PA, Arnold D, et al. Rituximab in relapsing-remitting multiple sclerosis: a 72-week, open-label, phase I trial. Ann Neurol 2008;63:395400.

31. Hawker K, O'Connor P, Freedman M, et al. Rituximab in patients with primary progressive multiple sclerosis: results of a randomized double-blind placebo-controlled multicenter trial. Ann Neurol. 2009;66:460-71.

32. Panitch H, Goodin DS, Francis G, Chang P, Coyle PK, O'Connor P, Monaghan E, Li D, Weinshenker B; EVIDENCE Study Group. Evidence of Interferon Dose-response: European North American Comparative Efficacy; University of British Columbia MS/MRI Research Group. Randomized, comparative study of interferon beta-1a treatment regimens in MS: The EVIDENCE Trial. Neurology. 2002 Nov 26;59(10):1496-506.

33. PRISMS Study Group. Randomized double-blind placebo controlled study of interferon- 1a in relapsing/remitting multiple sclerosis. Lancet 1998;352:1498–1504.

34. PRISMS Study Group, University of British Columbia MS/MRI Analysis Group. PRISMS-4: long-term efficacy of interferon- -1a in relapsing MS. Neurology 2001;56:1628–1636.

35. Secondary Progressive Efficacy Clinical Trial of Recombinant Interferon-Beta-1a in MS (SPECTRIMS) Study Group. Randomized controlled trial of interferon- beta-1a in secondary progressive MS: Clinical results. Neurology. 2001 Jun 12;56(11):1496-504.

36. European Public Assessments reports, Rebif Summary of product characteristic, January 27, 2010.

37. REBIF U.S. Physician Prescribing Information, Revised: July 2009.

38. Lim SY, Constantinescu CS. Current and future disease-modifying therapies in multiple sclerosis. Int J Clin Pract. 2010 Apr;64(5):637-50.

39. Polman C et al; Recommendations for clinical use of data on neutralizing antibodies to interferon-beta therapy in multiple sclerosis. Lancet Neurol 2010; 9: 740–50.

40. Polman CH, Reingold SC, Barkhof F, et al. Ethics of placebo-controlled clinical trials in multiple sclerosis: a reassessment. Neurology 2008;70:1134–40.

41. Kap YS, van Driel N, Blezer E, et al. Late B Cell Depletion with a Human Anti-Human CD20 IgG1 κ Monoclonal Antibody halts the Development of Experimental Autoimmune Encephalomyelitis in Marmosets. J Immunol 2010; 185; 3990-4003.

42. Gong Q, Ou Q, Ye S, et al. Importance of Cellular Microenvironment and Circulatory Dynamics in B Cell Immunotherapy. J. Immunol. 2005;174;817-826.

43. Ahuja A, Shupe J, Dunn R, et al. Depletion of B Cells in Murine Lupus: Efficacy and Resistance. The Journal of Immunology, 2007, 179: 3351–3361.

44. Gelinck LBS, et al. Poor serological responses upon influenza vaccination in patients with rheumatoid arthritis treated with rituximab. Ann Rheum Dis 2007; 66:1402-1403.

45. van Assen S, Holvast A, Benne CA, et al. Humoral responses after influenza vaccination are severely reduced in patients with rheumatoid arthritis treated with Rituximab. Arthritis & Rheumatism 2010; 62:75-81.

46. Oren S. Vaccination against influenza in rheumatoid arthritis patients: the effect of rituximab on the humoral response. Ann Rheum Dis published online November 2, 2007.

47. Bingham C, Looney R, Deodhar A, et al. Results from a controlled clinical trial (SIERRA) to evaluate primary and recall responses to immunizations in RA patients treated with rituximab. Arthritis Rheum 2008; 58:900-901[abstract#1999]. http://acr.confex.com/acr/2008/webprogram/Paper3941.html assessed on December 17, 2009

48. Cohen JA, Fischer JS, Bolibrush DM, et al. Intrarater and interrater reliability of the MS functional composite outcome measure. Neurology. 2000;54:802-6.

49. Fischer JS, Rudick RA, Cutter GR, Reingold SC. The Multiple Sclerosis Functional Composite Measure (MSFC): an integrated approach to MS clinical outcome assessment. National MS Society Clinical Outcomes Assessment Task Force. Mult Scler. 1999;5:244-50.

50. Ingram G, Hakobyan S, Hirst CL, et al. Complement regulator factor H as a serum biomarker of multiple sclerosis disease state. Brain 2010;133:1602-11.

51. Guidance for Industry: Patient-reported outcome measures: Use in medical product development to support labeling claims, FDA 2009, accessed at http://www.fda.gov/downloads/Drugs/GuidanceComplianceRegulatoryInformation/Guidances/UCM193282.pdf

52. Ware JE et al., SF-36 Health Survey: Manual & Interpretation Guide, Lincoln, RI, QualityMetric Incorporated.

53. Fairclough DL, Design and analysis of quality of life studies in clinical trials, 2010, Boca Raton, FL, CRC Press, 2010.

54. Emery P, Fleischmann R, Filipowicz-Sosnowska A, et al. The efficacy and safety of rituximab in patients with active rheumatoid arthritis despite methotrexate treatment: results of a phase IIB randomized, double-blind, placebo-controlled, dose-ranging trial. Arthritis Rheum. 2006;54:1390-400.

55. Calabrese LH, Molloy ES, Huang D, Ransohoff RM. Progressive multifocal leukoencephalopathy in rheumatic diseases: evolving clinical and pathologic patterns of disease. Arthritis Rheum. 2007;56:2116-28.

56. Kappos L, Bates D, Hartung HP, et al. Natalizumab treatment for multiple sclerosis: recommendations for patient selection and monitoring. Lancet Neurol. 2007;6:431–441.

57. Yousry TA, Major EO, Ryschkewitsch C, et al. Evaluation of patients treated with natalizumab for progressive multifocal leukoencephalopathy. N Engl J Med. 2006;354:924-33.

58. Beal SL., Boeckman AJ., Sheiner LB. NONMEM User's Guide, Parts I-VIII San Francisco: Division of Clinical Pharmacology- University of California, 1992.

# PART II: ETHICS AND GENERAL STUDY ADMINISTRATION

## 12. ETHICAL ASPECTS

### 12.1 Local Regulations/Declaration of Helsinki

The investigator will ensure that this study is conducted in full conformance with the principles of the "Declaration of Helsinki" or with the laws and regulations of the country in which the research is conducted, whichever affords the greater protection to the individual. The study must fully adhere to the principles outlined in "Guideline for Good Clinical Practice" ICH Tripartite Guideline or with local law if it affords greater protection to the patient. For studies conducted in the EU/EEA countries, the investigator will ensure compliance with the EU Clinical Trial Directive [2001/20/EC]. For studies conducted in the USA or under US IND, the investigator will additionally ensure adherence to the basic principles of "Good Clinical Practice" as outlined in the current version of 21 CFR, subchapter D, part 312, "Responsibilities of Sponsors and Investigators", part 50, "Protection of Human Subjects", and part 56, "Institutional Review Boards".

In other countries where a "Guideline for Good Clinical Practice" exists, Roche and the investigators will strictly ensure adherence to the stated provisions.

### 12.2 Informed Consent

#### 12.2.1 Main Study Informed Consent

**It is the responsibility of the investigator, or a person designated by the investigator [if acceptable by local regulations], to obtain signed informed consent from each patient prior to participating in this study after adequate explanation of the aims, methods, anticipated benefits, and potential hazards of the study**.

The investigator or designee must also explain that the patients are completely free to refuse to enter the study or to withdraw from it at any time, for any reason.

The electronic Case Report Forms for this study contain a section for documenting patient informed consent, and this must be completed appropriately. If new safety information results in significant changes in the risk/benefit assessment, the consent form should be reviewed and updated if necessary. All patients (including those already being treated) should be informed of the new information, given a copy of the revised form and give their consent to continue in the study.

For the patient not qualified or incapable of giving legal consent, written consent must be obtained from the legally acceptable representative. In the case where both the patient and his/her legally acceptable representative are unable to read, an impartial witness should be present during the entire informed consent discussion. After the patient and representative have orally consented to participation in the trial, the witness' signature on the form will attest that the information in the consent form was accurately explained and understood.

**For U.S.-IND studies:** In a life-threatening situation where a patient is unconscious or otherwise unable to communicate, the emergency is such that there is not enough time to obtain consent from the patient's legally acceptable representative, and there is no other or better treatment available, it is permissible to treat the patient under protocol with consent of both the investigator and another physician not involved in the study, with appropriate documentation submitted to the IRB within 5 days. If this collaboration is not immediately possible, there must be a written evaluation by a physician independent of the study and the appropriate documentation be submitted to the IRB within 5 days of treating the patient. In addition, the patient or his/her legally acceptable representative should be informed about the trial as soon as possible and consent to continue, giving written consent as described above.

**For non-U.S.-IND studies:** In a life-threatening situation where a patient is unconscious or otherwise unable to communicate, the emergency is such that there is not enough time to obtain consent from the patient's legally acceptable representative, and there is no other or better treatment available, it is permissible to treat the patient under protocol with consent of the investigator, with appropriate documentation that the IEC had approved the procedures used to enroll patients in such situations. In addition, the patient or his/her legally acceptable representative should be informed about the trial as soon as possible and consent to continue, giving written consent as described above.

### 12.2.2 RCR Informed Consent

It is the responsibility of the investigator, or a person designated by the investigator (if acceptable under local regulations), to obtain written informed consent from each individual who has consented to RCR sampling after adequate explanation of the aims, methods, objectives and potential hazards. Subjects must receive an explanation that they are completely free to refuse to provide the RCR specimen(s) and may withdraw his/ her sample at any time and for any reason during the study or 15 year storage period of the specimen(s). The Informed Consent for an **optional** specimen donation will be incorporated as a specific section into the main Clinical Trial Informed Consent Form (ICF). A second, separate, specific signature consenting to specimen donation will be required to document the study participant’s agreement to provide an **optional** specimen; if the participant declines, he/ she will check a “no” box in the appropriate section and not provide a second signature.

The patient does not have to provide a separate consent for protein biomarker RCR sampling.

The eCRF for the associated clinical study contains a page for documenting patient informed consent to the RCR, and this must be completed appropriately.

### 12.2.3 Death or Loss of Competence of Participant who has donated a specimen(s) that is stored in the RCR

In case the Informed Consent Form and/or the Study Protocol do not provide any specific provisions for death or loss of competence, specimen and data will continue to be used as part of RCR research.

In the event of the death of a participant of a Roche Clinical Trial or Experimental Medicine Research study or if a participant is legally incompetent at the time of the specimen and data procurement, or becomes legally incompetent thereafter, applicable provisions as stated for such situations in the respective Informed Consent Form and/or the Study Protocol shall become effective and be followed accordingly.

Additional procurement of assent from legally incompetent persons and minors shall take place according to local laws and international best practice, as it applies to the specific case.

## 12.3 Independent Ethics Committees (IEC) and Institutional Review Board (IRB)

The protocol, informed consent and any accompanying material provided to the patient in the U.S. will be submitted by the investigator to an IRB for review. For EEA member states, the Sponsor will submit to the Competent Authority and IEC, the protocol and any accompanying material provided to the patient. In both the US and EEA member states, the accompanying material may include patient information sheets, descriptions of the study used to obtain informed consent and terms of any compensation given to the patient as well as advertisements for the trial.

An approval letter or certificate (specifying the protocol number and title) from the IEC/IRB must be obtained before study initiation by the investigator specifying the date on which the committee met and granted the approval. This applies whenever subsequent amendments/modifications are made to the protocol.

Any modifications made to the protocol, informed consent or material provided to the patient after receipt of the IEC/IRB approval must also be submitted by the investigator in the U.S. and by the Sponsor in the EEA member states in accordance with local procedures and regulatory requirements.

When no local review board exists, the investigator is expected to submit the protocol to a regional committee. If no regional committee exists, Roche will assist the investigator in submitting the protocol to the European Ethics Review Committee.

Sampling for the RCR is contingent on review and approval for the exploratory biomarker assessments and written informed consent by an appropriate regulatory body (depending on the country where the study is performed) and a site's Institutional Review Board (IRB) / Ethics Committee (EC). If a regulatory or site's IRB/EC does not approve the sampling for the exploratory assessments the section on biomarker sampling will not be applicable.

Roche shall also submit an Annual Safety Report once a year to the IEC and Competent Authorities (CAs) according to local regulatory requirements and timelines of each country participating in the study. In the U.S. Roche submits an IND Annual Report to the FDA according to local regulatory requirements and timelines.

## 12.4 Role of the Science and Ethics Advisory Group (SEAG)

A Science and Ethics Advisory Group consisting of experts in the fields of biology, ethics, sociology and law will advise Roche regarding the use of specimens stored in the RCR and on the scientific and ethical aspects of handling genetic information. The SEAG is independent of Roche.

# 13. CONDITIONS FOR MODIFYING THE PROTOCOL

Requests from investigators to modify the protocol to ongoing studies will be considered only by consultation between an appropriate representative of the Sponsor and the investigator [investigator representative(s) in the case of a multicenter trial]. Protocol modifications must be prepared by a representative of the Sponsor and initially reviewed and approved by the Clinical Science Leader and Biostatistician.

All protocol modifications must be submitted to the appropriate Independent Ethics Committee or Institutional Review Board for information and approval in accordance with local requirements, and to Regulatory Agencies if required. Approval must be obtained before any changes can be implemented, except for changes necessary to eliminate an immediate hazard to trial patients, or when the change(s) involves only logistical or administrative aspects of the trial (e.g., change in monitor[s], change of telephone number[s]).

# 14. CONDITIONS FOR TERMINATING THE STUDY

Both the Sponsor and the investigator reserve the right to terminate the study at any time. Should this be necessary, both parties will arrange the procedures on an individual study basis after review and consultation. In terminating the study, Roche and the investigator will assure that adequate consideration is given to the protection of the patients' interests. The appropriate IRB/EC and Regulatory Agencies should be informed accordingly.

# 15. STUDY DOCUMENTATION, CRFS AND RECORD KEEPING

## 15.1 Investigator's Files / Retention of Documents

The Investigator must maintain adequate and accurate records to enable the conduct of the study to be fully documented and the study data to be subsequently verified. These documents should be classified into two different separate categories [1] Investigator's Study File, and [2] patient clinical source documents.

The Investigator's Study File will contain the protocol/amendments, eCRF and schedule of assessments, Independent Ethics Committee/Institutional Review Board and governmental approval with correspondence, sample informed consent, drug records, staff curriculum vitae and authorization forms and other appropriate documents/correspondence, etc. In addition at the end of the study the investigator will receive the patient data, which includes an audit trail containing a complete record of all changes to data, query resolution correspondence and reasons for changes, in human readable format on CD which also has to be kept with the Investigator's Study File.

Subject clinical source documents (usually defined by the project in advance to record key efficacy/safety parameters independent of the eCRFs) would include patient hospital/clinic records, physician's and nurse's notes, appointment book, original

laboratory reports, ECG, EEG, X-ray, pathology and special assessment reports, signed informed consent forms, consultant letters, and patient screening and enrollment logs. The Investigator must keep the two categories of documents as described above (including the archival CD) on file for at least 15 years after completion or discontinuation of the study. After that period of time the documents may be destroyed, patient to local regulations.

Should the Investigator wish to assign the study records to another party or move them to another location, Roche must be notified in advance.

If the Investigator can not guarantee this archiving requirement at the investigational site for any or all of the documents, special arrangements must be made between the Investigator and Roche to store these in a sealed container(s) outside of the site so that they can be returned sealed to the Investigator in case of a regulatory audit. Where source documents are required for the continued care of the patient, appropriate copies should be made for storing outside of the site.

ICH GCP guidelines require that Investigators maintain information in the study patient's records which corroborate data collected on the eCRF(s). Completed eCRF will be transferred to Sponsor.

## 15.2 Source Documents and Background Data

The investigator shall supply the Sponsor on request with any required background data from the study documentation or clinic records. This is particularly important when errors in data transcription are suspected. In case of special problems and/or governmental queries or requests for audit inspections, it is also necessary to have access to the complete study records, provided that patient confidentiality is protected.

## 15.3 Audits and Inspections

The investigator should understand that source documents for this trial should be made available to appropriately qualified personnel from the Roche Pharma Development Quality Assurance Unit or its designees, or to health authority inspectors after appropriate notification. The verification of the eCRF data must be by direct inspection of source documents.

## 15.4 Electronic Case Report Forms

Data for this study will be captured via an Electronic Data Capture (EDC) system by using eCRFs. An audit trail will maintain a record of initial entries and changes made; reasons for change; time and date of entry; and user name of person authorizing entry or change. The investigator must update eCRF and connect on a regular basis.

For each patient enrolled, an eCRF must be completed and electronically signed by the principal investigator or authorized delegate from the study staff. This also applies to records for those patients who fail to complete the study (even during a pre-randomization screening period if an eCRF was initiated). If a patient withdraws from the study, the reason must be noted on the eCRF. If a patient is withdrawn from the study because of a treatment-limiting AE, thorough efforts should be made to clearly document the outcome.

The investigator should ensure the accuracy, completeness and timeliness of the data reported to the Sponsor in the eCRFs and in all required reports.

### 15.5 Financial Disclosure

The investigator(s) will provide the Sponsor with sufficient accurate financial information (PD35) to allow the Sponsor to submit complete and accurate financial certification or disclosure statements to the appropriate regulatory authorities. The investigator is responsible to promptly update any information provided to the Sponsor if relevant changes occur in the course of the investigation and for 1 year following the completion of the study (last patient, last visit).

## 16. MONITORING THE STUDY

It is understood that the responsible Roche monitor [or designee] will contact and visit the investigator regularly and will be allowed, on request, to inspect the various records of the trial (eCRFs and other pertinent data) provided that patient confidentiality is maintained in accord with local requirements.

It will be the monitor's responsibility to inspect the eCRFs at regular intervals throughout the study, to verify the adherence to the protocol and the completeness, consistency and accuracy of the data being entered on them. The monitor must verify that the patient received the study drug assigned by the randomization center (by controlling the written confirmation of the randomization by IxRS). The monitor should have access to laboratory test reports and other patient records needed to verify the entries in the eCRF. The investigator (or deputy) agrees to cooperate with the monitor to ensure that any problems detected in the course of these monitoring visits are resolved.

Roche Clinical Repository specimens will at all times be tracked in a manner consistent with Good Clinical Practice, by a quality controlled, auditable and validated Laboratory Information Management System, to ensure compliance with data confidentiality as well as adherence to authorized use of specimens as specified in the study protocol and ICF, respectively. Roche monitors and auditors will have direct access to appropriate parts of records relating to patients participating in this study for the purposes of verifying the data provided to Roche. The site will permit monitoring, audits, Institutional Review Board/Independent Ethics Committee (IRB/IEC) review, and regulatory inspections by providing direct access to source data and documents related to the RCR Research Project.

## 17. CONFIDENTIALITY OF TRIAL DOCUMENTS AND SUBJECT RECORDS

The investigator must assure that patients' anonymity will be maintained and that their identities are protected from unauthorized parties. On CRFs or other documents submitted to the Sponsor, patients should not be identified by their names, but by an identification code. The investigator should keep a patient enrollment log showing codes, names and addresses. The investigator should maintain documents not for submission to Roche, e.g., patients' written consent forms, in strict confidence.

Roche already maintains rigorous confidentiality standards for clinical studies by "coding" (i.e. assigning a unique patient ID number at the investigator site) all patients enrolled in Roche clinical studies. This means that patient names are not included in

data sets that are transmitted to any Roche location. Given the sensitive nature of genetic data, Roche has implemented a number of additional processes to assure patient confidentiality. All specimens taken for inherited genetic research that will be stored in the RCR (see Section 5.5) undergo a second level of "coding". At Roche, the specimen is transferred to a new tube and labeled with a new random number. This is referred to as "Double Coding (De-Identification)". Data generated following the use of these specimens and all clinical data transferred from the clinical study database and considered relevant, will also be labeled with this same code. The "linking key" between the participant's identification number and this new independent code will be stored in a secure database system. Access to the table linking the participant identification number to the specimen code will be strictly limited and monitored by audit trail. Legitimate operational reasons for accessing the "linking key" will be documented in a standard operating procedure. Access to the "linking key" for any other reason will require written approval from the Governance Committee responsible for the specimen(s).

# 18. CLINICAL STUDY REPORT (CSR)

A clinical study report will be written and distributed to Health Authorities as required by applicable regulatory requirements.

# 19. PUBLICATION OF DATA AND PROTECTION OF TRADE SECRETS

Roche will comply with the requirements for publication of study results.

The results of this study may be published or presented at scientific meetings. If this is foreseen, the investigator agrees to submit all manuscripts or abstracts to Roche prior to submission. This allows the Sponsor to protect proprietary information and to provide comments based on information from other studies that may not yet be available to the investigator.

In accordance with standard editorial and ethical practice, Roche will generally support publication of multicenter trials only in their entirety and not as individual center data. In this case, a coordinating investigator will be designated by mutual agreement.

Authorship will be determined by mutual agreement and in line with International Committee of Medical Journal Editors (ICMJE) authorship requirements. Any formal publication of the study in which contribution of Roche personnel exceeded that of conventional monitoring will be considered as a joint publication by the investigator and the appropriate Roche personnel.

Data derived from RCR specimen analysis on individual patients will not be provided to study investigators, except where explicitly stipulated in a study protocol (e.g. if the result is an enrollment criterion). Exceptions may be granted (e.g. if biomarker data would be linked to safety issues). The aggregate results of any research conducted using RCR specimens will be available in accordance with the effective Roche policy on study data publication.

Any inventions and resulting patents, improvements and / or know- how originating from the use of the RCR will become and remain the exclusive and unburdened property of Roche, except where agreed otherwise.

## Appendix 1: AEs Categories for Determining Relationship to Test Drug

The causality relationship of study drug to the adverse event will be assessed by the investigator as either: Yes or No.

If there is a reasonable suspected causal relationship to the study medication, i.e., there are facts (evidence) or arguments to suggest a causal relationship, drug-event relationship should be assessed as Yes.

**The following criteria should be considered in order to assess the relationship as Yes:**

- Reasonable temporal association with drug administration
- It may or may not have been produced by the subject's clinical state, environmental or toxic factors, or other modes of therapy administered to the subject.
- Known response pattern to suspected drug
- Disappears or decreases on cessation or reduction in dose
- Reappears on rechallenge

**The following criteria should be considered in order to assess the relationship as No:**

- It does not follow a reasonable temporal sequence from administration of the drug.
- It may readily have been produced by the subject's clinical state, environmental or toxic factors, or other modes of therapy administered to the subject.
- It does not follow a known pattern of response to the suspected drug.
- It does not reappear or worsen when the drug is readministered.

## Appendix 2: ICH Guidelines for Clinical Safety Data Management, Definitions and Standards for Expedited Reporting, Topic E2

A serious adverse event is any experience that suggests a significant hazard, contraindication, side effect or precaution. It is any AE that at any dose fulfills at least one of the following criteria:

– is fatal; [results in death] [**NOTE**: death is an outcome, not an event]

– is Life-Threatening [**NOTE**: the term "Life-Threatening" refers to an event in which the patient was at immediate risk of death at the time of the event; it does not refer to an event which could hypothetically have caused a death had it been more severe]

– requires in-patient hospitalization or prolongation of existing hospitalization

– results in persistent or significant disability/incapacity

– is a congenital anomaly/birth defect

– is medically significant or requires intervention to prevent one or other of the outcomes listed above.

Medical and scientific judgment should be exercised in deciding whether expedited reporting to the Sponsor is appropriate in other situations, such as important medical events that may not be immediately life-threatening or result in death or hospitalization but may jeopardize the patient or may require intervention to prevent one of the outcomes listed in the definitions above. These situations should also usually be considered serious.

Examples of such events are intensive treatment in an emergency room or at home for allergic bronchospasm; blood dyscrasias or convulsions that do not result in hospitalization; or development of drug dependency or drug abuse.

An unexpected AE is one in which the nature or severity is not consistent with the applicable product information.

Causality is initially assessed by the investigator. For Serious Adverse Events, possible causes of the event are indicated by selecting one or more options. (Check all that apply)

– Pre-existing/Underlying disease - specify

– Study treatment - specify the drug(s) related to the event

– Other treatment (concomitant or previous) - specify

– Protocol-related procedure

– Other (e.g. accident, new or intercurrent illness) - specify

The term severe is a measure of intensity, thus a severe AE is not necessarily serious. For example, nausea of several hours' duration may be rated as severe, but may not be clinically serious.

A serious adverse event occurring during the study or which comes to the attention of the investigator within 15 days after stopping the treatment or during the protocol-defined follow-up period, if this is longer, whether considered treatment-related or not, must be reported. In addition, a serious adverse event that occurs after this time, if considered related to test “drug”, should be reported.

Such preliminary reports will be followed by detailed descriptions later which will include copies of hospital case reports, autopsy reports and other documents when requested and applicable.

For serious adverse events, the following must be assessed and recorded on the AEs eform of the eCRF: intensity, relationship to test substance, action taken, and outcome to date.

The investigator must notify the Ethics Review Committee/Institutional Review Board of a serious adverse event in writing as soon as is practical and in accordance with international and local laws and regulations.

ROCHE LOCAL COUNTRY CONTACT for SAEs: Local Monitor:

See attached *Protocol Administrative and Contact Information & List of Investigators Form,[ gcp_for000227]*, for details of administrative and contact information.

ROCHE HEADQUARTERS CONTACT for SAEs and other medical emergencies: Clinical Operations/Clinical Science:

See attached *Protocol Administrative and Contact Information & List of Investigators form,[gcp_for000227]*, for details of administrative and contact information.

24 HOUR MEDICAL COVERAGE:

Identification of a contact for 24 Hour Medical Coverage is mandatory to be compliant with worldwide regulatory agencies and to ensure the safety of study patients.

An Emergency Medical Call Center Help Desk will access the Roche Medical Emergency List, escalate emergency medical calls, provide medical translation service (if necessary), connect the investigator with the Roche medical contact for this study and track all calls. The Emergency Medical Call Center Help Desk will be manned 24 hours 7 days a week. Toll free numbers will be distributed to all investigators running Roche Pharma Development clinical trials. The Help Desk will be used for medical emergencies outside regular business hours, or when the regular Clinical Science Leader cannot be reached.

See the attached *Protocol Administrative and Contact Information & List of Investigators form [gcp_for000227]*, for details of administrative, contact information, and Emergency Medical Call Center Help Desk toll-free numbers.

## Appendix 3: Common Terminology Criteria for Adverse Events (CTCAE)

In the present study, toxicities will be graded according to the Common Terminology Criteria for Adverse Events (CTCAE), version 4.0.

The Common Terminology Criteria for Adverse Events v4.0 (CTCAE) can be found in the Roche hand-out entitled: " Common Terminology Criteria for Adverse Events v4.0" or via the following web-site: http://ctep.cancer.gov

## Appendix 4: Telephone Interviews

The purpose of this interview is to identify any new or worsening neurological symptoms that warrant an unscheduled visit and to collect data on possible events of infections. Telephone interviews should be performed by study personnel every 4 weeks between clinic visits (with exemption of prolonged B-cell monitoring period when telephone interviews need to be performed every 12 weeks) – see also Section 5.3.4.4.

**Please ask the following questions and record patient's answers during the Telephone Interview:**

| Questions | No | Yes |
|---|---|---|
| **1. Since your last visit or telephone interview, have you had any new or worsening medical problems (such as sudden changes in your thinking, alterations in your behavior, visual disturbances, extremity weakness, limb coordination problems, or gait abnormalities) that have persisted over several days?** | | |
| **2. Since your last visit or telephone interview, have you taken any new medicines to treat cancer or MS or any other new medicines that weaken your immune system?** | | |
| **3. Since your last visit or telephone interview, other than for the treatment of a recent relapse, have you taken any of the following medicines: methylprednisone, (e.g. Depo-Medrol®, Solu-Medrol®), dexamethasone (e.g. Decadron®), prednisolone, or other steroid medicine?** | | |
| **4. Since your last visit or telephone interview, have you had any signs or symptoms of infection?** | | |

If the patient answered YES to any question, contact the Treating Investigator and review the patient's answers. The Investigator can determine if an unscheduled visit is required.

**Record any pertinent comments made by the patient during the interview:**

______________________________________________________________________

______________________________________________________________________

______________________________________________________________________

NAME:_______________________________ Date:_______________________________

*Name of person completing the telephone interview*

## Appendix 4: Telephone Interviews (Cont.)

**Below is a sample list of medications that can weaken the immune system. This list does not include all drugs that can suppress the immune system.**

**Approved MS Therapies**:

Glatiramer acetate (Copaxone®)

Interferon β-1a (Rebif®, AVONEX®)

Interferon β-1b (Betaseron®)

Mitoxantrone (Novantrone®)

Natalizumab (Tysabri®)

Fingolimod (Gilenya®) – if relevant

**Immunosuppressants/Antineoplastics:**

Azathioprine (Imuran®, Azasan®)

Cladribine (Leustatin®)

Cyclophosphamide (Cytoxan®, Neosar®)

Cyclosporine (Sandimmune®, Neoral®)

Fludarabine phosphate (Fludara®)

Leflunomide (Arava®)

Mercaptopurine (Purinethol®)

Methotrexate (Methotrex®, Rheumatrex®, Trexall®)

Mycophenolate mofetil (CellCept®)

Pemetrexed (Alimta®)

**Additional Immunomodulators and Immunosuppressants:**

Other interferons (Actimmune®, Infergen®, Intron® A,
Pegasys®, PEG-Intron®, Rebetron®, Roferon®-A)

Adalimumab (Humira®)

Alefacept (Amevive®)

Alemtuzumab (Campath®)

Anakinra (Kineret®)

Daclizumab (Zenapax®)

Etanercept (Enbrel®)

Infliximab (Remicade®)

Intravenous immunoglobulin (IVIG)

Ofatumumab (Arzerra®)

Rituximab (Rituxan/MabThera®)

Trastuzumab (Herceptin®)

## Appendix 5: Modified Fatigue Impact Scale (MFIS)

MFIS

### MODIFIED FATIGUE IMPACT SCALE (MFIS)

Following is a list of statements that describe how fatigue may affect a person. Fatigue is a feeling of physical tiredness and lack of energy that many people experience from time to time. In medical conditions like MS, feelings of fatigue can occur more often and have a greater impact than usual. Please read each statement carefully, and then SELECT THE ONE NUMBER that best indicates how often fatigue has affected you in this way during the PAST 4 WEEKS. (If you need help in marking your responses, TELL THE INTERVIEWER THE NUMBER of the best response.) PLEASE ANSWER EVERY QUESTION. If you are not sure which answer to select, please choose the one answer that comes closest to describing you. The interviewer can explain any words or phrases that you do not understand.

| **Because of my fatigue during the PAST 4 WEEKS....** | Never | Rarely | Sometimes | Often | Almost always |
|---|---|---|---|---|---|
| * 1. I have been less alert. | ○ 0 | ○ 1 | ○ 2 | ○ 3 | ○ 4 |
| * 2. I have had difficulty paying attention for long periods of time. | ○ 0 | ○ 1 | ○ 2 | ○ 3 | ○ 4 |
| * 3. I have been unable to think clearly. | ○ 0 | ○ 1 | ○ 2 | ○ 3 | ○ 4 |
| * 4. I have been clumsy and uncoordinated. | ○ 0 | ○ 1 | ○ 2 | ○ 3 | ○ 4 |
| * 5. I have been forgetful. | ○ 0 | ○ 1 | ○ 2 | ○ 3 | ○ 4 |
| * 6. I have had to pace myself in my physical activities. | ○ 0 | ○ 1 | ○ 2 | ○ 3 | ○ 4 |

Back | Next

## Appendix 5: Modified Fatigue Impact Scale (MFIS) (Cont.)

MFIS

### MODIFIED FATIGUE IMPACT SCALE (MFIS)

| Because of my fatigue during the PAST 4 WEEKS.... | Never | Rarely | Sometimes | Often | Almost always |
|---|---|---|---|---|---|
| * 7. I have been less motivated to do anything that requires physical effort. | ◯ 0 | ◯ 1 | ◯ 2 | ◯ 3 | ◯ 4 |
| * 8. I have been less motivated to participate in social activities. | ◯ 0 | ◯ 1 | ◯ 2 | ◯ 3 | ◯ 4 |
| * 9. I have been limited in my ability to do things away from home. | ◯ 0 | ◯ 1 | ◯ 2 | ◯ 3 | ◯ 4 |
| * 10. I have had trouble maintaining physical effort for long periods. | ◯ 0 | ◯ 1 | ◯ 2 | ◯ 3 | ◯ 4 |
| * 11. I have had difficulty making decisions. | ◯ 0 | ◯ 1 | ◯ 2 | ◯ 3 | ◯ 4 |
| * 12. I have been less motivated to do anything that requires thinking. | ◯ 0 | ◯ 1 | ◯ 2 | ◯ 3 | ◯ 4 |
| * 13. my muscles have felt weak. | ◯ 0 | ◯ 1 | ◯ 2 | ◯ 3 | ◯ 4 |
| * 14. I have been physically uncomfortable. | ◯ 0 | ◯ 1 | ◯ 2 | ◯ 3 | ◯ 4 |

Back | Next

## Appendix 5: Modified Fatigue Impact Scale (MFIS) (Cont.)

MFIS

### MODIFIED FATIGUE IMPACT SCALE (MFIS)

| Because of my fatigue during the PAST 4 WEEKS.... | Never | Rarely | Sometimes | Often | Almost always |
|---|---|---|---|---|---|
| * 15. I have had trouble finishing tasks that require thinking. | 0 | 1 | 2 | 3 | 4 |
| * 16. I have had difficulty organizing my thoughts when doing things at home or at work. | 0 | 1 | 2 | 3 | 4 |
| * 17. I have been less able to complete tasks that require physical effort. | 0 | 1 | 2 | 3 | 4 |
| * 18. my thinking has been slowed down. | 0 | 1 | 2 | 3 | 4 |
| * 19. I have had trouble concentrating. | 0 | 1 | 2 | 3 | 4 |
| * 20. I have limited my physical activities. | 0 | 1 | 2 | 3 | 4 |
| * 21. I have needed to rest more often or for longer periods. | 0 | 1 | 2 | 3 | 4 |

Back Next

## Appendix 6: The Center for Epidemiologic Studies Depression Scale (CES-D)

**CES-D Scale**

### Center for Epidemiologic Studies Depression Scale (CES-D, NIMH)

Below is a list of the ways you might have felt or behaved. Please tell me how often you have felt this way in the last week.

| | During the Past Week | | | |
|---|---|---|---|---|
| | Rarely or none of the time (less than 1 Day) | Some or a little of the time (1-2 days) | Occasionally or a moderate amount of time (3-4 days) | Most or all of the time (5-7 days) |
| * 1. I was bothered by things that usually don't bother me. | ○ | ○ | ○ | ○ |
| * 2. I did not feel like eating: my appetite was poor. | ○ | ○ | ○ | ○ |
| * 3. I felt that I could not shake off the blues even with help from my family or friends. | ○ | ○ | ○ | ○ |
| * 4. I felt that I was just as good as other people. | ○ | ○ | ○ | ○ |
| * 5. I had trouble keeping my mind on what I was doing. | ○ | ○ | ○ | ○ |
| * 6. I felt depressed. | ○ | ○ | ○ | ○ |
| * 7. I felt that everything I did was an effort. | ○ | ○ | ○ | ○ |
| * 8. I felt hopeful about the future. | ○ | ○ | ○ | ○ |
| * 9. I thought my life had been a failure. | ○ | ○ | ○ | ○ |
| * 10. I felt fearful. | ○ | ○ | ○ | ○ |

**Back** **Next**

## Appendix 6: The Center for Epidemiologic Studies Depression Scale (CES-D) (Cont.)

**CES-D Scale**

### Center for Epidemiologic Studies Depression Scale (CES-D, NIMH)

Below is a list of the ways you might have felt or behaved. Please tell me how often you have felt this way in the last week.

| | During the Past Week | | | |
|---|---|---|---|---|
| | Rarely or none of the time (less than 1 Day) | Some or a little of the time (1-2 days) | Occasionally or a moderate amount of time (3-4 days) | Most or all of the time (5-7 days) |
| * 11. My sleep was restless. | ○ | ○ | ○ | ○ |
| * 12. I was happy. | ○ | ○ | ○ | ○ |
| * 13. I talked less than usual. | ○ | ○ | ○ | ○ |
| * 14. I felt lonely. | ○ | ○ | ○ | ○ |
| * 15. People were unfriendly. | ○ | ○ | ○ | ○ |
| * 16. I enjoyed life. | ○ | ○ | ○ | ○ |
| * 17. I had crying spells. | ○ | ○ | ○ | ○ |
| * 18. I felt sad. | ○ | ○ | ○ | ○ |
| * 19. I felt that people dislike me. | ○ | ○ | ○ | ○ |
| * 20. I could not get "going". | ○ | ○ | ○ | ○ |

SCORING: zero for answers in the first column, 1 for answers in the second column, 2 for answers in the third column, 3 for answers in the fourth column. The scoring of positive items is reversed. Possible range of scores is zero to 60, with the higher scores indicating the presence of more symptomatology.

Back | Next

## Appendix 7: The Short Form (SF-36) Health Survey

**SF-36**

### Your Health and Well-Being

This survey asks for your views about your health. This information will help keep track of how you feel and how well you are able to do your usual activities. Thank you for completing this survey!

For each of the following questions, please SELECT the response that best describes your answer.

*** 1. In general, would you say your health is:**

| Excellent | Very good | Good | Fair | Poor |
|---|---|---|---|---|
| ○ 1 | ○ 2 | ○ 3 | ○ 4 | ○ 5 |

*** 2. COMPARED TO ONE YEAR AGO, how would you rate your health in general NOW?**

| Much better now than one year ago | Somewhat better now than one year ago | About the same as one year ago | Somewhat worse now than one year ago | Much worse now than one year ago |
|---|---|---|---|---|
| ○ 1 | ○ 2 | ○ 3 | ○ 4 | ○ 5 |

SF-36v2® Health Survey © 1992, 2002, 2009 Medical Outcomes Trust and QualityMetric Incorporated. All rights reserved.
SF-36® is a registered trademark of Medical Outcomes Trust.

Back | Next

# Appendix 7: The Short Form (SF-36) Health Survey (Cont.)

**SF-36**

3. The following questions are about activities you might do during a typical day. Does YOUR HEALTH NOW LIMIT YOU in these activities? If so, how much?

| | Yes, limited a lot | Yes, limited a little | No, not limited at all |
|---|---|---|---|
| * a. VIGOROUS ACTIVITIES, such as running, lifting heavy objects, participating in strenuous sports. | ○ 1 | ○ 2 | ○ 3 |
| * b. MODERATE ACTIVITIES, such as moving a table, pushing a vacuum cleaner, bowling, or playing golf. | ○ 1 | ○ 2 | ○ 3 |
| * c. Lifting or carrying groceries. | ○ 1 | ○ 2 | ○ 3 |
| * d. Climbing SEVERAL flights of stairs. | ○ 1 | ○ 2 | ○ 3 |
| * e. Climbing ONE flight of stairs. | ○ 1 | ○ 2 | ○ 3 |
| * f. Bending, kneeling, or stooping. | ○ 1 | ○ 2 | ○ 3 |
| * g. Walking MORE THAN A MILE. | ○ 1 | ○ 2 | ○ 3 |
| * h. Walking SEVERAL HUNDRED YARDS. | ○ 1 | ○ 2 | ○ 3 |
| * i. Walking ONE HUNDRED YARDS. | ○ 1 | ○ 2 | ○ 3 |
| * j. Bathing or dressing yourself. | ○ 1 | ○ 2 | ○ 3 |

SF-36v2® Health Survey © 1992, 2002, 2009 Medical Outcomes Trust and QualityMetric Incorporated. All rights reserved.
SF-36® is a registered trademark of Medical Outcomes Trust.

Back | Next

**SF-36**

**4. During the PAST 4 WEEKS, how much of the time have you had any of the following problems with your work or other regular daily activities AS A RESULT OF YOUR PHYSICAL HEALTH?**

| | All of the time | Most of the time | Some of the time | A little of the time | None of the time |
|---|---|---|---|---|---|
| * a. Cut down on the AMOUNT OF TIME you spent on work or other activities. | 1 | 2 | 3 | 4 | 5 |
| * b. ACCOMPLISHED LESS than you would like. | 1 | 2 | 3 | 4 | 5 |
| * c. Were limited in the KIND of work or other activities. | 1 | 2 | 3 | 4 | 5 |
| * d. Had DIFFICULTY performing the work or other activities (for example, it took extra effort). | 1 | 2 | 3 | 4 | 5 |

**5. During the PAST 4 WEEKS, how much of the time have you had any of the following problems with your work or other regular daily activities AS A RESULT OF ANY EMOTIONAL PROBLEMS (such as feeling depressed or anxious)?**

| | All of the time | Most of the time | Some of the time | A little of the time | None of the time |
|---|---|---|---|---|---|
| * a. Cut down on the AMOUNT OF TIME you spent on work or other activities. | 1 | 2 | 3 | 4 | 5 |
| * b. ACCOMPLISHED LESS than you would like. | 1 | 2 | 3 | 4 | 5 |
| * c. Did work or other activities LESS CAREFULLY THAN USUAL. | 1 | 2 | 3 | 4 | 5 |

SF-36v2® Health Survey © 1992, 2002, 2009 Medical Outcomes Trust and QualityMetric Incorporated. All rights reserved.
SF-36® is a registered trademark of Medical Outcomes Trust.

Back | Next

**SF-36**

* 6. During the PAST 4 WEEKS, to what extent has your PHYSICAL HEALTH OR EMOTIONAL PROBLEMS interfered with your normal social activities with family, friends, neighbors, or groups?

| Not at all | Slightly | Moderately | Quite a bit | Extremely |
|---|---|---|---|---|
| ○ 1 | ○ 2 | ○ 3 | ○ 4 | ○ 5 |

* 7. How much BODILY pain have you had during the PAST 4 WEEKS?

| None | Very mild | Mild | Moderate | Severe | Very severe |
|---|---|---|---|---|---|
| ○ 1 | ○ 2 | ○ 3 | ○ 4 | ○ 5 | ○ 6 |

* 8. During the PAST 4 WEEKS, how much did PAIN interfere with your normal work (including both work outside the home and housework)?

| Not at all | A little bit | Moderately | Quite a bit | Extremely |
|---|---|---|---|---|
| ○ 1 | ○ 2 | ○ 3 | ○ 4 | ○ 5 |

SF-36v2® Health Survey © 1992, 2002, 2009 Medical Outcomes Trust and QualityMetric Incorporated. All rights reserved.
SF-36® is a registered trademark of Medical Outcomes Trust.

Back | Next

**SF-36**

9. These questions are about how you feel and how things have been with you DURING THE PAST 4 WEEKS. For each question, please give the one answer that comes closest to the way you have been feeling. How much of the time during the PAST 4 WEEKS...

| | All of the time | Most of the time | Some of the time | A little of the time | None of the time |
|---|---|---|---|---|---|
| * a. Did you feel full of life? | ○ 1 | ○ 2 | ○ 3 | ○ 4 | ○ 5 |
| * b. Have you been very nervous? | ○ 1 | ○ 2 | ○ 3 | ○ 4 | ○ 5 |
| * c. Have you felt so down in the dumps that nothing could cheer you up? | ○ 1 | ○ 2 | ○ 3 | ○ 4 | ○ 5 |
| * d. Have you felt calm and peaceful? | ○ 1 | ○ 2 | ○ 3 | ○ 4 | ○ 5 |
| * e. Did you have a lot of energy? | ○ 1 | ○ 2 | ○ 3 | ○ 4 | ○ 5 |
| * f. Have you felt downhearted and depressed? | ○ 1 | ○ 2 | ○ 3 | ○ 4 | ○ 5 |
| * g. Did you feel worn out? | ○ 1 | ○ 2 | ○ 3 | ○ 4 | ○ 5 |
| * h. Have you been happy? | ○ 1 | ○ 2 | ○ 3 | ○ 4 | ○ 5 |
| * i. Did you feel tired? | ○ 1 | ○ 2 | ○ 3 | ○ 4 | ○ 5 |

SF-36v2® Health Survey © 1992, 2002, 2009 Medical Outcomes Trust and QualityMetric Incorporated. All rights reserved.
SF-36® is a registered trademark of Medical Outcomes Trust.

Back | Next

**SF-36**

* 10. During the PAST 4 WEEKS, how much of the time has your PHYSICAL HEALTH OR EMOTIONAL PROBLEMS interfered with your social activities (like visiting with friends, relatives, etc.)?

| All of the time | Most of the time | Some of the time | A little of the time | None of the time |
|---|---|---|---|---|
| ○ 1 | ○ 2 | ○ 3 | ○ 4 | ○ 5 |

11. How TRUE or FALSE is EACH of the following statements for you?

| | Definitely true | Mostly true | Don't know | Mostly false | Definitely false |
|---|---|---|---|---|---|
| * a. I seem to get sick a little easier than other people. | ○ 1 | ○ 2 | ○ 3 | ○ 4 | ○ 5 |
| * b. I am as healthy as anybody I know. | ○ 1 | ○ 2 | ○ 3 | ○ 4 | ○ 5 |
| * c. I expect my health to get worse. | ○ 1 | ○ 2 | ○ 3 | ○ 4 | ○ 5 |
| * d. My health is excellent. | ○ 1 | ○ 2 | ○ 3 | ○ 4 | ○ 5 |

SF-36v2® Health Survey © 1992, 2002, 2009 Medical Outcomes Trust and QualityMetric Incorporated. All rights reserved.
SF-36® is a registered trademark of Medical Outcomes Trust.

Back | Next

**PROTOCOL**

| | |
|---|---|
| **TITLE:** | **A RANDOMIZED, DOUBLE-BLIND, DOUBLE-DUMMY, PARALLEL-GROUP STUDY TO EVALUATE THE EFFICACY AND SAFETY OF OCRELIZUMAB IN COMPARISON TO INTERFERON BETA-1A (REBIF®) IN PATIENTS WITH RELAPSING MULTIPLE SCLEROSIS** |
| **PROTOCOL NUMBER:** | WA21092 |
| **VERSION NUMBER:** | *E* |
| **EUDRACT NUMBER:** | 2010-020337-99 |
| **IND NUMBER:** | 100,593 |
| **TEST PRODUCT:** | Ocrelizumab (RO4964913) |
| **MEDICAL MONITOR:** | Dr. Algirdas Kakarieka |
| **SPONSOR:** | F. Hoffmann-La Roche, Ltd |
| **DATE FINAL:** | 25 August 2010 |
| **DATES AMENDED:** | Version B: 01 June 2011<br>Version C: 15 June 2012<br>Version D: 14 March 2013<br>Version E: See electronic date stamp below |

**PROTOCOL AMENDMENT APPROVAL**

| **Approver's Name** | **Title** | **Date and Time (UTC)** |
|---|---|---|
| Hope,Mark | Company Signatory | 04-Sep-2014 10:21:29 |

**CONFIDENTIAL**
The information contained in this document, especially any unpublished data, is the property of F. Hoffmann-La Roche, Ltd (or under its control) and therefore is provided to you in confidence as an investigator, potential investigator, or consultant, for review by you, your staff, and an applicable Ethics Committee or Institutional Review Board. It is understood that this information will not be disclosed to others without written authorization from Roche except to the extent necessary to obtain informed consent from persons to whom the drug may be administered.

**PROTOCOL WA21092 COORDINATING INVESTIGATOR:**

**Prof. Dr. Ludwig Kappos**
Chair Neurology
Research Group Leader Clinical Neuroimmunology and Neurobiology
Department of Biomedicine
University Hospital
Petersgraben 4
CH-4031 Basel
Switzerland
Phone: +41 61 265 44 64

# SYNOPSIS OF PROTOCOL NUMBER WA21092*E*

| | |
|---|---|
| TITLE | **A Randomized, Double-Blind, Double-Dummy, Parallel-Group Study To Evaluate The Efficacy And Safety Of Ocrelizumab In Comparison To Interferon Beta-1a (Rebif®) In Patients With Relapsing Multiple Sclerosis** |
| SPONSOR | F. Hoffmann-La Roche Ltd<br>Genentech Inc    CLINICAL PHASE    III |
| INDICATION | Relapsing Multiple Sclerosis |
| OBJECTIVES | **Primary:**<br>The primary objective of this study is to assess whether the efficacy of ocrelizumab 600 mg (given as dual infusions of 300 mg on Days 1 and 15 of the first 24-week treatment cycle and as a single infusion of 600 mg on Day 1 of each 24-week treatment cycle thereafter) intravenously every 24 weeks is superior to Rebif® as measured by the annualized protocol-defined* relapse rate by 2 years (96 weeks) in patients with relapsing multiple sclerosis (MS).<br><br>**Secondary:**<br>The *key* secondary objectives of this study are to evaluate whether the efficacy of ocrelizumab is superior to Rebif®, as reflected by the following measures:<br>• The time to onset of *confirmed* disability progression for at least 12 weeks *with the* initial event of neurological worsening occur*ring* during the 96-week, double-blind, double-dummy, treatment period.<br>• *The total number of T1 Gd-enhancing lesions as detected by brain MRI at Weeks 24, 48, and 96*<br>• The total number of new, and/or enlarging T2 hyperintense lesions as detected by brain MRI at Weeks 24, 48, and 96.<br>• *The proportion of patients who have confirmed disability improvement for at least 12 weeks with the initial event of neurological improvement occurring during the 96-week double-blind, double-dummy treatment period.*<br>• The time to onset of *confirmed* disability progression for at least 24 weeks, *with the* initial event of neurological worsening occur*ring* during the 96-week, double-blind, double-dummy, treatment period.<br>• *The total number of T1-hypo-intense lesions (Chronic Black Holes) at Weeks 24, 48, and 96*<br>• The change in Multiple Sclerosis Functional Composite Scale (MSFCS) score from baseline to Week 96.<br>• The *percentage* change in brain volume as detected by brain MRI from Week 24 to Week 96.<br>• *The change in SF-36 Physical Component Summary (PCS) Score from baseline to Week 96*<br>• *The proportion of patients who have no evidence of disease activity (NEDA) by Week 96* |

**Safety:**

To evaluate the safety and tolerability of ocrelizumab 600 mg (given as dual infusions of 300 mg on Days 1 and 15 of the first 24-week treatment cycle and as a single infusion of 600 mg on Day 1 of each 24-week treatment cycle thereafter) intravenously every 24 weeks in patients with relapsing MS (including exploratory, long-term safety and tolerability in those patients entering the Open-Label Extension [OLE] Phase).

**Pharmacokinetics/Pharmacodynamics:**

To explore the pharmacokinetics, immunogenicity and pharmacodynamics of ocrelizumab in patients with relapsing MS.

**Exploratory objectives:**

- The change in low contrast visual acuity from baseline to Weeks 48 and 96.
- The change in the Symbol Digit Modalities Test from baseline to Weeks 48 and 96.
- *The proportion of relapse free patients by Week 96.*
- *The change in total T2 hyperintense lesion volume as detected by brain MRI from baseline to Week 96.*
- The annualized relapse rate, based on *all* clinical relapses at the end of the 96-week comparative treatment period *(protocol-defined relapses are a subset of all clinical relapses).*
- *The ARR of relapses requiring IV steroid therapy.*
- *The ARR of severe relapses.*
- The *percentage* change in brain volume as detected by brain MRI from baseline to Week 96.
- The change in Multiple Sclerosis Functional Composite Scale (MSFCS) score from baseline to Week 48.
- The cumulative change in EDSS scores, measured in area under the curve (AUC) by Week 96.
- The change in EDSS from baseline to Week 96.
- The change in timed 25-foot walk from baseline to Week 96.
- The change in 9-hole peg test from baseline to Week 96.
- The change in paced auditory serial addition test (PASAT) from baseline to Weeks 48 and 96.
- The time to onset of sustained 20% increase in 9-hole peg test for at least 12 weeks.
- The time to onset of sustained 20% increase in timed 25 foot walk for at least 12 weeks.
- *The change in fatigue, as measured by the Modified Fatigue Impact Scale (MFIS) total score from baseline to Week 96.*
- *The change from baseline in patient-reported depressive symptoms, as measured by the Center for Epidemiologic Studies Depression Scale (CES-D), from baseline to Week 96.*
- *Analyses of EQ-5D, collected at baseline, Week 48, and Week 96.*
- The change in Karnofsky Performance Status Scale from baseline to Weeks 96.
- *The percentage change in cortical grey matter volume from baseline to Week 96.*
- *The percentage change in white matter volume from baseline to*

| | |
|---|---|
| | *Week 96.*<br>• *The proportion of patients who have confirmed disability improvement sustained for at least 24 weeks, with the initial event of neurological improvement occurring during the 96-week double-blind double-dummy treatment period.*<br>• *The proportion of patients who have disability improvement sustained for at least 12 weeks and sustained until the end of the 96-week, double-blind, double-dummy treatment period, with the initial event of neurological improvement occurring during the 96-week, double-blind, double-dummy treatment period.*<br>• *The duration of the confirmed disability improvement.*<br>• *The proportion of patients who, at Week 96, have improved, stable, or worsened disability, compared to baseline.*<br>• *The change in Quality of Life, as measured by the Short Form 36 version 2 Mental Component Summary (MCS) Score from baseline to Week 96.*<br>• To evaluate the long-term safety, tolerability, and efficacy of ocrelizumab in patients with the relapsing form of MS who are enrolled in the OLE Phase. |
| TRIAL DESIGN | Multicenter, randomized, double-blind, double-dummy, parallel-group study |
| NUMBER OF SUBJECTS | 800 patients in total, 400 patients per group using a 1:1 randomization ratio. Please refer to Sample Size and Statistical Analyses section of the synopsis for more details. |
| TARGET POPULATION | **Inclusion criteria:**<br>1. Ability to provide written, informed consent and to be able to follow the schedule of protocol assessments *.<br>2. Ages 18-55 years at screening, inclusive.<br>3. Diagnosis of MS, in accordance with the revised McDonald criteria (2010).<br>4. At least 2 documented clinical attacks within the last 2 years prior to screening or one clinical attack in the year prior to screening (but not within 30 days prior to screening).<br>5. Neurological stability for ≥30 days prior to both screening and baseline.<br>6. EDSS, at screening, from 0 to 5.5 inclusive.<br>7. Documented MRI of brain with abnormalities consistent with MS prior to screening.<br>8. Patients of reproductive potential must use reliable means of contraception as described below as a minimum (adherence to local requirements, if more stringent, is required**):<br>• For female patients: Two methods of contraception throughout the trial, including the active treatment phase AND for 48 weeks after the last dose of ocrelizumab, or until their B-cells have repleted, whichever is longer.<br>• For male patients: Two methods of contraception throughout the trial, including the active treatment phase AND for 24 weeks after the last dose of ocrelizumab. Acceptable methods of contraception include one primary (e.g., systemic hormonal contraception or tubal ligation of the female partner, vasectomy |

of the male partner) AND one secondary barrier method (e.g., latex condoms, spermicide) OR a double barrier method (e.g., latex condom, intrauterine device, vaginal ring or pessary plus spermicide [e.g., foam, vaginal suppository, gel, cream]).

9. For patients of <u>non reproductive potential</u> (adherence to local requirements, if more stringent, is required**):
   - Women may be enrolled if postmenopausal (i.e., spontaneous amenorrhea for the past year confirmed by an FSH level greater than 40 mIU/mL) unless the patient is receiving a hormonal therapy for their menopause or surgically sterile (i.e., hysterectomy, complete bilateral oophorectomy);
   - Men may be enrolled if they are surgically sterile (castration).

* Patients who are unable to complete exploratory assessments (e.g., electronic patient reported outcomes [ePROs]) due to physical/disease limitations will not be excluded from the study.

** Based on local Ethics Committees or National Competent Authority feedback additional requirements to assure contraception or to confirm menopause may be required (e.g., serum estradiol compatible with post-menopause status, longer duration of amenorrhea, higher level of FSH).

**<u>Exclusion Criteria</u>**

Patients who meet the following criteria must be excluded from study entry:

1. Diagnosis of primary progressive MS.
2. Disease duration of more than 10 years in patients with an EDSS ≤2.0 at screening.
3. Inability to complete an MRI (contraindications for MRI include but are not restricted to weight ≥140 kg, pacemaker, cochlear implants, presence of foreign substances in the eye, intracranial vascular clips, surgery within 6 weeks of entry into the study, coronary stent implanted within 8 weeks prior to the time of the intended MRI, etc).
   (Patients with contraindication to Gd can be enrolled into the study but cannot receive Gd contrast dyes during their MRI scans.)
4. Known presence of other neurological disorders which may mimic MS including but not limited to: neuromeylitis optica, Lyme disease, untreated vitamin B12 deficiency, neurosarcoidosis and cerebrovascular disorders.

**<u>Exclusions Related to General Health</u>**

5. Pregnancy or lactation.
6. Any concomitant disease that may require chronic treatment with systemic corticosteroids or immunosuppressants during the course of the study.
7. History or currently active primary or secondary immunodeficiency.
8. Lack of peripheral venous access.
9. History of severe allergic or anaphylactic reactions to humanized or murine monoclonal antibodies.
10. Significant or uncontrolled somatic disease or any other significant disease that may preclude patient from participating in the study.
11. Congestive heart failure (NYHA III or IV functional severity).
12. Known active bacterial, viral, fungal, mycobacterial infection or other infection, excluding fungal infection of nail beds.

13. Infection requiring hospitalization or treatment with intravenous (i.v.) antibiotics within 4 weeks prior to baseline visit or oral antibiotics within 2 weeks prior to baseline visit.

14. History or known presence of recurrent or chronic infection (e.g., HIV, syphilis, tuberculosis).
    *Please note:* in Germany the following additional exclusion criteria apply:
    - *Positive anti – HIV I at screening*
    - *Positive anti – HIV II at screening*
    - *Positive QuantiFERON®-TB Gold test at screening*

    *Patients in Germany with an indeterminate result are not eligible for the study unless additional testing demonstrating a negative result is provided. Thus, these patients should have either a tuberculin skin test or have the QuantiFERON® TB Gold test repeated prior to enrollment into the study. If a tuberculin skin test is performed, an induration of ≥ 6 mm is "positive" for a patient with history of BCG vaccine, while an induration of ≥ 10 mm is "positive" for a patient without history of BCG vaccine.*
    *If necessary a QuantiFERON®-TB Gold test might be complemented by additional specific diagnostic tests as per standard procedures in Germany.*
15. History of progressive multifocal leukoencephalopathy (PML)
16. History of malignancy, including solid tumors and hematological malignancies, except basal cell carcinoma, *in situ* squamous cell carcinoma of the skin, and *in situ* carcinoma of the cervix of the uterus that have been previously completely excised with documented, clear margins.
17. History of alcohol or drug abuse within 24 weeks prior to baseline.
18. History or laboratory evidence of coagulation disorders.

**Exclusions Related to Medications***

19. Receipt of a live vaccine within 6 weeks prior to the baseline visit. In rare cases when patient requires vaccination with a live vaccine, the screening period may be extended but cannot exceed 8 weeks.
20. Treatment with any investigational agent within 24 weeks of screening (Visit 1) or five half-lives of the investigational drug (whichever is longer; or treatment with any experimental procedures for MS [e.g., treatment for chronic cerebrospinal venous insufficiency]).
21. Contraindications to or intolerance of oral or i.v. corticosteroids, according to the country label, including:
    a) Psychosis not yet controlled by a treatment;
    b) Hypersensitivity to any of the constituents.
22. Contraindication to Rebif® or incompatibility with Rebif® use, including:
    a) Current severe depression and/or suicidal ideation;
    b) Hypersensitivity to natural or recombinant interferon-β, or to any excipients;
    c) Previous suboptimal response to High Dose High Frequency (HDHF) interferon or cessation of HDHF interferon therapy due to poor tolerability;
    d) Prior cessation of Rebif® therapy due to toxicity, which is likely to recur.
23. Treatment with dalfamipridine (Ampyra®) unless on stable dose for ≥ 30 days prior to screening. Wherever possible, patients should

remain on stable doses throughout the 96 week treatment period.

24. Previous treatment with B-cell targeted therapies (i.e., rituximab, ocrelizumab, atacicept, belimumab or ofatumumab).
25. Systemic corticosteroid therapy within 4 weeks prior to screening.**
26. Any previous treatment with alemtuzumab (Campath), anti-CD4, cladribine, mitoxantrone, daclizumab, teriflunomide, laquinimod, total body irradiation or bone marrow transplantation.
27. Treatment with cyclophosphamide, azathioprine, mycophenolate mofetil (MMF), cyclosporine, methotrexate or natalizumab within 24 months prior to screening. NB. Patients previously treated with natalizumab will be eligible for this study only if duration of treatment with natalizumab was < 1 year.
28. Treatment with fingolimod (FTY720, Gilenya®) or other S1P receptor modulator (i.e., BAF312), or with BG12, within 24 weeks prior to screening.
    (NB. Only patients with T lymphocyte count ≥ LLN will be eligible for this study.)
29. Treatment with i.v. immunoglobulin within 12 weeks prior to baseline.

* Patients screened for this study should not be withdrawn from therapies for the sole purpose of meeting eligibility for the trial. Patients, who discontinue their current therapy for non-medical reasons, should specifically be informed before deciding to enter the study of their treatment options.

** The screening period may be extended (but cannot exceed 8 weeks) for patients who have used systemic corticosteroids for their MS before screening. For a patient to be eligible, systemic corticosteroids should not have been administered also between screening and baseline.

**<u>Exclusions Related to Laboratory Findings*</u>**

30. Positive serum β hCG measured at screening.
31. Positive screening tests for hepatitis B (hepatitis B surface antigen [HBsAg] positive, or positive hepatitis B core antibody [total HBcAb] confirmed by a positive viral deoxyribonucleic acid [DNA] polymerase chain reaction [PCR]) or hepatitis C (HepCAb).
32. Positive rapid plasma reagin (RPR).
33. CD4 count < 300/µL.
34. AST/SGOT or ALT/SGPT ≥2.0 Upper Limit of Normal (ULN).
35. Platelet count <100,000/µL (<100 x $10^9$/L).
36. Levels of serum IgG 18% below the LLN (for central lab IgG <4.6 g/L).
37. Levels of serum IgM 8% below the LLN (for central lab IgM <0.37 g/L).
38. Total neutrophil count <1.5 × $10^3$/µL.

<u>*Re-testing before baseline</u>: in rare cases in which the screening laboratory samples are rejected by the central laboratory (example: hemolyzed sample) or the results are not assessable (example: indeterminate) or abnormal, the tests need to be repeated within 4 weeks. Any abnormal screening laboratory value that is clinically relevant should be retested in order to rule out any progressive or uncontrolled underlying condition. The last value before randomization must meet study criteria. In such circumstances, the screening period may need to be prolonged but should not exceed

| | |
|---|---|
| | 8 weeks.<br><br><u>Please note:</u> based on local Ethics Committees or National Competent Authority requirements, additional diagnostic testing may be required for selected patients or selected centers to exclude tuberculosis, Lyme disease, HTLV-1 associated myelopathy (HAM), acquired immune deficiency syndrome (AIDS), hereditary disorders, connective tissue disorders, or sarcoidosis. Other specific diagnostic tests may be requested when deemed necessary by the investigator.<br><br>**Eligibility criteria for Open-Label Extension Phase:**<br><br>Patients who meet the following entry criteria may participate in the OLE Phase:<br><br>1. Complete the 96-week, double-blind, double-dummy treatment period, and who in the opinion of the Investigator may benefit from treatment with ocrelizumab;<br>2. Are able and willing to provide written informed consent for the OLE Phase (e.g., before the first infusion at Cycle 5) and to comply with the study protocol;<br>3. Are willing to continue to use at least two contraceptive methods;<br>4. Meet re-treatment criteria with ocrelizumab. |
| LENGTH OF STUDY | The study will consist of the following periods:<br>- **Screening period**: approximately 2 weeks. The screening period may be prolonged for up to 8 weeks for relevant clinical, administrative or operational reasons.<br><br>**- Double-blind, double-dummy treatment period:** 96 weeks;<br><br>The double-blind, double-dummy comparative treatment period will consist of 4 treatment cycles 24 weeks apart.<br><br>Patients who complete the 96-week treatment period may become eligible for a separate, open-label extension study.<br><br>**- Safety Follow-Up Period**: at least 48 weeks;<br><br>Patients who complete or withdraw from study treatment will be observed for a period of at least 48 weeks counting from the date of the last infusion of study drug. If at this time the peripheral blood B-cells remain depleted patient should continue to be monitored at 24-week intervals until B-cell count has returned to the baseline value or to the lower limit of the normal range (whichever is the lower).<br><br>**- OLE Phase;**<br><br>The OLE Phase will continue until ocrelizumab is commercially available in the patient's country, or as per local regulation, or should the Sponsor decide to terminate the ocrelizumab program for MS, but will not exceed 4 years after the last patient to reach the Week 96 Visit in the double-blind, double-dummy treatment period.<br><br>During the OLE Phase, all patients will receive the ocrelizumab 600 mg regimen every 24 weeks. Patients who withdraw from the OLE Phase will be entered into the Safety Follow-Up Period.<br><br>See section "Procedures" below for more details. |

| | |
|---|---|
| END OF DOUBLE-BLIND, DOUBLE-DUMMY TREATMENT PERIOD OF THE STUDY | The end of the double-blind, double-dummy treatment period of the study is defined as the date at which the last data point during double-blind, double-dummy treatment from the last patient is received, as required for statistical analysis defined in the *Statistical* Analysis Plan (*S*AP). |
| END OF STUDY | The end of study is defined as either the last patient last visit of the OLE Phase or the last patient last visit in B-cell monitoring of Safety Follow-Up Period, whichever is later.<br><br>The OLE Phase will continue until ocrelizumab is commercially available in the patient's country, or as per local regulation, or should the Sponsor decide to terminate the ocrelizumab program for MS, but will not exceed 4 years after the last patient to reach the Week 96 Visit in the double-blind, double-dummy treatment period.<br><br>The B-cell monitoring of the Safety Follow-Up Period of each patient will last until the B-cell count has returned to the baseline value or to the lower limit of the normal range (whichever is lower). |
| INVESTIGATIONAL MEDICAL PRODUCT(S) DOSE/ ROUTE/ REGIMEN | **For the double-blind, double-dummy treatment period:**<br>– Group A (Ocrelizumab): Ocrelizumab 600 mg (given as dual infusions of ocrelizumab 300 mg on Days 1 and 15 of the first 24-week treatment cycle and as single infusions of 600 mg on Day 1 for each 24-week treatment cycle, thereafter) every 24 weeks.<br>– Group B (Interferon beta-1a (Rebif®))<br>Rebif® will be administered as follows:<br>▪ Treatment initiation:<br>⇒ during Weeks 1 and 2, Rebif® 8.8 µg (one pre-filled syringe [0.2 mL] containing 8.8 µg [2.4 MIU]) of interferon beta-1a given via subcutaneous (s.c.) injection 3 times per week<br>⇒ during Weeks 3 and 4, Rebif® 22 µg (one pre-filled syringe [0.5 mL] containing 22 µg [6 MIU]) of interferon beta-1a) given via s.c. injection 3 times per week<br><br>▪ Treatment continuation:<br>⇒ From the fifth week onwards, Rebif® 44 µg (one pre-filled syringe (0.5 mL) containing 44 µg (12 MIU) of interferon beta-1a) given via s.c. injection 3 times per week<br>⇒ A lower dose of 22 µg, given via s.c. injection 3 times per week, will be available for patients who cannot tolerate the 44 µg dose of Rebif®. *Please refer to detailed guidelines in the study protocol.*<br><br>Patients randomized to active ocrelizumab group will also receive dummy placebo of Rebif® (administered via s.c. injection three times per week).<br>Patients randomized to active Rebif® group will also receive dummy placebo of ocrelizumab (administered as i.v. infusions at similar time points to those of the ocrelizumab group).<br>Dummy placebos of ocrelizumab and of Rebif® will be similar in appearance and administration as the investigational product.<br><br>**For the Open-Label Extension Phase Screening Period:**<br>– Group A: Rebif® placebo (one pre-filled syringe [0.5 mL]) given via s.c. injection 3 times per week<br>– Group B: Rebif® 44 µg (one pre-filled syringe [0.5 mL] containing 44 µg [12 MIU] of interferon beta-1a) given via s.c. injection 3 times per week |

| | |
|---|---|
| | A lower dose of 22 µg, given via s.c. injection three times per week, will be available for patients who cannot tolerate the 44-µg dose of Rebif®. Please refer to detailed guidelines in the study protocol.<br><br>**For the Open-Label Extension Phase:**<br>Ocrelizumab 600 mg (given as dual infusions of ocrelizumab 300 mg on Days 1 and 15 of the first 24-week treatment cycle of the OLE Phase and as single infusions of 600 mg on Day 1 for each 24-week treatment cycle, thereafter) every 24 weeks. |
| NON-INVESTIGATIONAL MEDICAL PRODUCT(S) | **<u>During the double-blind, double-dummy treatment period:</u>**<br><u>Ocrelizumab/ocrelizumab dummy placebo:</u> Thirty minutes prior to the start of each infusion, patients will receive a methylprednisolone 100 mg i.v. infusion. Pre-infusion treatment with an oral analgesic/antipyretic (e.g., acetaminophen) and an oral antihistamine (e.g., diphenhydramine) is also recommended.<br><u>Rebif®/ Rebif® dummy placebo:</u> Pre-treatment with non-steroid anti-inflammatory drugs (ibuprofen) or acetaminophen is recommended; investigators should follow their local label for further information.<br><br>**<u>During the Open-Label Extension Phase Screening Period:</u>**<br><u>Rebif®/ Rebif® dummy placebo:</u> Pre-treatment with non-steroid anti-inflammatory drugs (ibuprofen) or acetaminophen is recommended; investigators should follow their local label for further information.<br><br>**<u>During the Open-Label Extension Phase:</u>**<br><u>Ocrelizumab:</u> Thirty minutes prior to the start of each infusion, patients will receive a methylprednisolone 100 mg i.v. infusion. Pre-infusion treatment with an oral analgesic/antipyretic (e.g., acetaminophen) and an oral antihistamine (e.g., diphenhydramine) recommended. |
| ASSESSMENTS OF:<br>- EFFICACY | – **Assessment of clinical and protocol defined relapses**<br>▪ <u>Protocol-defined relapse</u> is the occurrence of new or worsening neurological symptoms attributable to MS. Symptoms must persist for >24 hours and should not be attributable to confounding clinical factors (e.g., fever, infection, injury, adverse reactions to medications) and immediately preceded by a stable or improving neurological state for least 30 days. The new or worsening neurological symptoms must be accompanied by objective neurological worsening consistent with an increase of at least half a step on the EDSS scale, or 2 points on one of the appropriate FSS, or 1 point on two or more of the appropriate FSS. The change must affect the selected FSS (i.e., pyramidal, ambulation, cerebellar, brainstem, sensory, or visual). Episodic spasms, sexual dysfunction, fatigue, mood change or bladder or bowel urgency or incontinence will not suffice to establish a relapse. NB: Sexual dysfunction and Fatigue will not be scored.<br>⇨ Please note: adjudication of protocol-defined relapses will be performed by the Sponsor based on pre-specified criteria, applied to data collected by investigator, in a blinded fashion.<br><br>– **Brain MRI acquisition sequences**<br>▪ T1-weighted MRI (without Gd-enhancement)<br>▪ T1-weighted MRI (with Gd-enhancement) |

| | |
|---|---|
| | ▪ Fluid-attenuated, inversion recovery (FLAIR), Proton-density–weighted and/or T2-weighted MRI<br>– **Assessment of *confirmed* disability progression**<br>▪ Disability progression is defined as an increase of ≥1.0 point from the baseline EDSS score that is not attributable to another etiology (e.g., fever, concurrent illness, or concomitant medication) when the baseline score is 5.5 or less, and ≥0.5 when the baseline score is above 5.5. Disability progression is considered *confirmed* when the increase in the EDSS is confirmed at regularly scheduled visits at least 12 and 24 weeks after the initial documentation of neurological worsening.<br>▪ *Confirmed* disability progression, confirmed for both 12 and 24 weeks after the initial documentation of neurological worsening, will be analyzed.<br><br>The following instruments will be used to assess the patient's functional ability: Low Contrast Letter Acuity Charts (LCVA/Sloan charts), Symbol Digit Modalities Test (SDMT), Kurtzke Expanded Disability Status Scale (EDSS), Multiple Sclerosis Functional Composite Scale (MSFCS) and Karnofsky Performance Status Scale (clinician-reported version). |
| - SAFETY | Adverse events, vital signs, weight, physical and neurological examination, clinical laboratory tests, 12 lead ECG, locally reviewed MRI for safety (non-MS CNS pathology), concomitant medications.<br><br>Pregnancy tests [serum/urine beta subunit human chorionic gonadotropin (beta hCG)] will be performed in women of childbearing potential. Plasma and urine samples will be collected for JCV assessments.<br><br>Columbia-Suicide Severity Rating Scale (C-SSRS) will be used for prospective suicidality assessment. |
| - PHARMACOKINETICS/ PHARMACODYNAMICS | Blood samples will be taken for measurement of ocrelizumab serum concentration at the time points detailed in the Schedule of Assessments. Nonlinear mixed effects modeling will be used to analyze the sparse sampling dose-concentration-time data of ocrelizumab in order to assess the pharmacokinetics. Population PK parameters will be estimated and the influence of covariates, such as age, gender, weight, Human Anti-human Antibodies (HAHA), and CD19 lymphocyte count, investigated. The relationship between ocrelizumab exposure and selected safety and efficacy parameters will be analyzed and explored in order to characterize the exposure-response curve of ocrelizumab. |
| –IMMUNOGENICITY | Serum samples for measurement of HAHA to ocrelizumab are collected according to the Schedule of Assessments. |
| – PATIENT REPORTED OUTCOMES (PRO) | ▪ Modified Fatigue Impact Scale (MFIS Standard)<br>▪ Center for Epidemiology Studies Depression Scale (CES-D3)<br>▪ Medical Outcomes Study 36-Item Short Form Survey Instrument (SF-36v2 Standard)<br>▪ EuroQol instrument (EQ-5D-3L-Tablet) |

| | | |
|---|---|---|
| – | PROTEIN BIOMARKER SAMPLES | Specimens for protein biomarker discovery and validation will be collected from all patients. These specimens will be used for research purposes to identify and/or verify protein biomarkers that are predictive of response to ocrelizumab treatment (in terms of dose, safety and tolerability) and will help understand the pathogenesis, course and outcome of multiple sclerosis and related diseases. In addition, screening technologies for larger numbers of proteins and antibodies may also be used to discover novel antibody associations with MS, *disability* progression and response to therapy.<br><br>Analyses will include but may not be limited to *interleukin-6*.<br><br>6 mL sample of whole blood will be collected in a plain tube without EDTA for serum isolation. Blood specimens for protein biomarker discovery and validation will be collected from all patients as per Schedule of Assessments. These specimens will be stored for 5 years after the end of the study and then destroyed unless a different regulation for storage time is in place at a given site. |
| - | EXPLORATORY BIOMARKERS (non-DNA) | Roche Clinical Repository (RCR) non-DNA, (dynamic, non-inherited) RNA specimen and plasma for biomarker discovery and validation will be collected only from patients consenting to RCR as per Schedule of Assessments. RCR samples (2 x approximately 2.5 mL of blood collected in PAXgene vacutainers and 6 mL of blood collected in a tube with EDTA for plasma isolation) will be collected to promote, facilitate and improve individualized healthcare by better understanding/predicting ocrelizumab efficacy, dose responses, safety, ocrelizumab mode of action, progression of multiple sclerosis and associated diseases. These specimens may be stored for up to 15 years after the end of the study. |
| - | EXPLORATORY BIOMARKERS (DNA) | All patients who have been enrolled in the study will be asked to donate an optional DNA specimen (by written informed consent) for pharmacogenetic and genetic research.<br><br>RCR DNA sampling will involve taking one sample of 6 mL of blood *in the double-blind, double-dummy treatment phase of the study* taken as per Schedule of Assessments. The study protocol which includes RCR sampling is submitted to the concerned Ethics Committee and is available for Competent Authority review upon request. These specimens will be stored for up to 15 years after the end of the study. |
| - | OPTICAL COHERENCE TOMOGRAPHY EXPLORATORY SUBSTUDY | Optical Coherence Tomography (OCT) scans will be performed only for patients consenting to OCT substudy at certain selected centers. OCT will be performed as described in Appendix 8 of the protocol. |

**PROCEDURES (summary):**

## Figure 1: Overview of Study Design

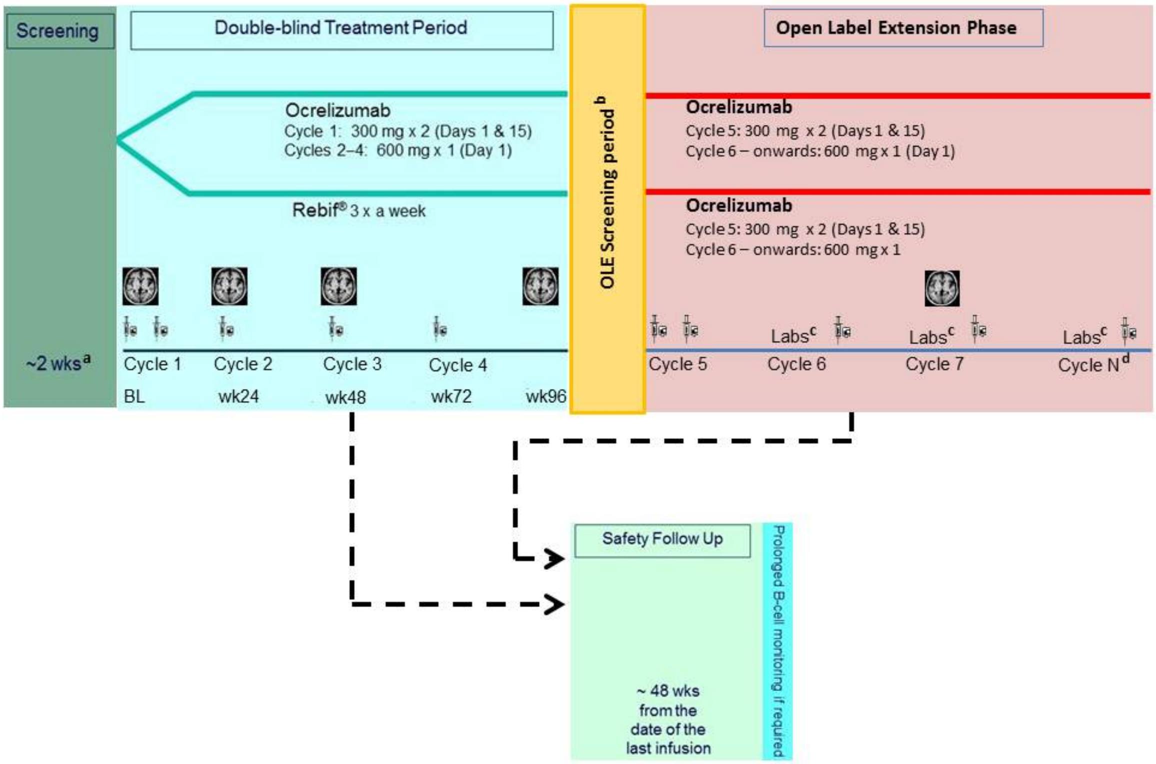


a. Screening for the study may be prolonged for up to 8 weeks for relevant clinical, administrative, or operational reasons.
b. The OLE Phase Screening Period will start after all assessments at the Week 96 Visit have been done. It will last up to 4 weeks. It is possible that for particular reasons, the OLE Phase Screening Period could be longer than 4 weeks. If a prolongation of the OLE Phase Screening Period is needed, it should be discussed with the Sponsor on a case-by-case basis.
Note: During the OLE Phase Screening Period, patients should receive Rebif®/Rebif® placebo until the first infusion of Cycle 5.
c. In order to verify if patients meet re-treatment criteria, patients in the Open-Label Extension Phase of the study should come to the clinic approximately 2 weeks prior to infusions of Cycle 6, 7, etc.
d. The OLE Phase of the study can be terminated at any time (please refer to Sections 3.1.4 and 5.10). Cycle N represents a typical cycle that occurs every 24 weeks.

## Screening:

Consenting patients will enter a screening period to be evaluated for eligibility. The Screening period will last approximately 2 weeks, but it may be prolonged for up to 8 weeks for relevant clinical, administrative, or operational reasons. Procedures at screening will include collecting medical history, medical examination including thorough neurological exam, EDSS score, ECG, blood and urine sampling. Please see *Table 4* - “Schedule of Assessments: Screening through the End of Double-Blind, Double-Dummy Treatment Period” for further details.

*Please note that based on local Ethics Committees or National Competent Authority requirements, additional diagnostic testing may be required for selected patients or*

*selected centers to exclude tuberculosis, Lyme disease, HTLV-1 associated myelopathy (HAM), acquired immune deficiency syndrome (AIDS), hereditary disorders, connective tissue disorders, or sarcoidosis.*

## Treatment Period:

### Double-blind, double-dummy, comparative treatment period

Eligible patients will be randomized via IxRS into one of two treatment groups: ocrelizumab 600 mg regimen (group A) or interferon beta-1a (Rebif®) (group B) – please refer to Table 1 and Table 2 for more details.

To prevent potential unblinding as a result of adverse events or changes to laboratory results, the following, additional measures have been implemented:

– **The Examining Investigator/EDSS assessor** will perform the neurological examination, document the FSS scores and assess EDSS scores and the Karnofsky Performance Status Scale. The examining investigator or a qualified designee will be also responsible for performing and documenting results from: MSFCS, low contrast visual acuity testing, and the Symbol Digit Modalities Test. He or she will have access only to data from assessments listed above. The examining Investigator/EDSS assessor will not be involved with any aspect of medical management of the patient and will not have access to patient data. Every effort will be made to ensure that there is no change in the examining Investigator/EDSS assessor throughout the course of the study for any individual patient. The examining Investigator/EDSS assessors will be trained and instructed not to discuss what adverse effects (if any) the patient is experiencing from their medication. Examining Investigator/EDSS assessors will receive training in performing EDSS assessments prior to the beginning of the study and must have successfully passed an examination on performance of the Neurostatus EDSS examination within 24 months of participation. All examining Investigator/EDSS assessors will receive ongoing training on performance of the Neurostatus EDSS examination throughout the course of the study.

– **Patient education:** prior to being examined by the Examining Investigator/EDSS assessor, patients will be instructed not to discuss what (if any) adverse effects they may be experiencing. Treating Investigators and/or study coordinators should remind patients of these instructions prior to EDSS assessments and this should be documented in the source documents.

– **Blinded, central MRI assessments:** a blinded, central MRI reader will assess all on-study MRI scans. These assessments will provide independent confirmation of the relative changes in immune-mediated, CNS damage.

**Blinding of laboratory parameters:** laboratory parameters which may lead to unblinding to treatment assignment, such as FACS cell counts including $CD19^+$ cells, lymphocyte count, IgM and IgG levels and type I interferon neutralizing antibody levels will be blinded in all patients. In order to ensure patients' safety in the study and to allow for assessments of the re-treatment criteria, a central laboratory will provide study

investigators and Medical Monitors with reflex messages triggered by critical blinded laboratory results. Investigators notified of their patient's critical laboratory test results will be instructed to suspend further treatment with study drug until the patient becomes eligible for re-treatment. The reflex messages from a central laboratory, together with non-blinded laboratory results, should be carefully reviewed at every visit before continuing with study treatment. The reflex messages will occur during the double-blind, double-dummy treatment period until the fifth cycle (first cycle of OLE Phase). The reflex messages will not be in effect from the sixth cycle onward. During the treatment period, patients will be assessed at clinical visits as per Schedule of Assessments: Screening through the End of Double-Blind, Double-Dummy Treatment Period – please refer to *Table 4* for further details.

Prior to the next infusion of study drug, patients will be evaluated for pre-specified conditions and laboratory abnormalities to allow for re-treatment.

**Please note: patients who discontinue from study medication within the 96-week double-blind, double-dummy comparative phase (treatment period) of the study will enter the Safety Follow-Up Period (see below); they will not be eligible for the OLE Phase.**

**Open-Label Extension Phase Screening Period**

Patients who complete the 96-week treatment period may become eligible for the OLE Phase of the study. Patients will be consented for participation in the OLE Phase if, in the opinion of the Investigator, they may benefit from treatment with ocrelizumab. Patients who are not willing to participate in the OLE Phase of the study will be entered into the Safety Follow-Up Period (see below). Patient treatment allocation during the double-blind, double-dummy treatment period should not be unblinded regardless of participation in the OLE Phase.

In the case of a patient who initially declines participation in the OLE Phase and subsequently reconsiders the decision, the patient will have up to 24 weeks after the Week 96 Visit to enter the OLE Phase. In this instance, he or she should not have taken any prohibited medication. Patients who decline participation in the OLE Phase should enter the Safety Follow-Up Period.

Patients who have consented to participate in the OLE Phase will enter an OLE Phase Screening Period to be evaluated for eligibility. The OLE Phase Screening Period will start after all assessments at the Week 96 Visit have been performed. This screening period will last up to 4 weeks. It is possible that the OLE Phase Screening Period could be longer than 4 weeks. If a prolongation of the OLE Phase Screening Period is needed, it should be discussed with the Sponsor on a case-by-case basis.

Information from assessments performed during the Week 96 Visit will be utilized to verify the eligibility of the patient for the OLE Phase of the study.

During the OLE Phase Screening Period, all patients should receive Rebif®/Rebif® placebo (depending on initial arm assigned to) until the first infusion of Cycle 5.

Patients who withdraw from the OLE Phase Screening Period will be entered into the Safety Follow-Up Period (see below).

**Open-Label Extension Phase**

Duration: The OLE Phase will continue until ocrelizumab is commercially available in the patient's country, or as per local regulation, or should the Sponsor decide to terminate the ocrelizumab program for MS, but will not exceed 4 years after the last patient to reach the Week 96 Visit in the double-blind, double-dummy treatment period.

*In the United Kingdom, the OLE Phase will last for 4 years. The 4-year duration of the OLE Phase serves to evaluate long-term safety, tolerability, and efficacy of ocrelizumab treatment in patients with relapsing forms of MS.*

Treatment: During the OLE Phase, all patients will receive the ocrelizumab 600 mg regimen every 24 weeks.

Withdrawal: Patients who withdraw from the OLE Phase will be entered into the Safety Follow-Up Period (see below).

**Safety Follow-Up Period**

Patients who discontinue treatment *prematurely* for any reason during the following periods will be entered into the Safety Follow-Up Period:

- Prior to completion of the 96-week double-blind, double-dummy treatment period;
- During the OLE Phase Screening Period;
- During the OLE Phase;
- Patients who choose not to enter the OLE Phase or are not eligible for the OLE Phase after completing the 96-week, double-blind, double-dummy treatment period.

As noted previously in the OLE Phase Screening Period, in the case of a patient who initially declines participation in the OLE Phase and subsequently reconsiders the decision, the patient will have up to 24 weeks after the Week 96 Visit to enter the OLE Phase. In this instance, he or she should not have taken any prohibited medication.

The Safety Follow-Up Period will last for at least 48 weeks counting from the date of the last infusion of the ocrelizumab/placebo. Safety Follow-Up visits will be performed at 12-week intervals starting from the date of patient's latest visit (Withdrawal from Treatment Visit). However, if after this time the peripheral blood B-cells remain depleted, patient should continue to be monitored at 24-week intervals until B-cell count has returned to the baseline value or to the lower limit of the normal range (whichever is the lower). Please refer to Figure 2 and to Section 4.4.2.1 for more details.

**Figure 2: Safety Follow-Up – Prolonged B-cell Monitoring Period**

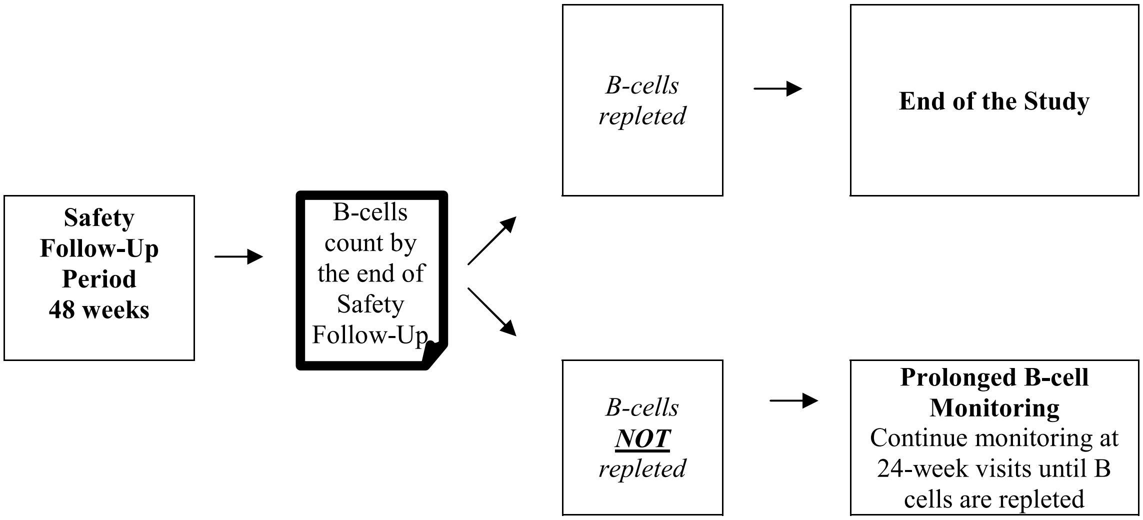


*Please note: patients in Safety Follow-Up who receive other therapies that may decrease B-cell level will only be followed for 48 weeks from the date of the last infusion of the study drug regardless of their B-cell count.*

During Safety Follow-Up patients will be assessed at clinical visits every 12 weeks as per Schedule of Assessments. Telephone interviews will be performed every 4 weeks. If prolonged B-cell monitoring is required, patients will be assessed at clinical visits every 24 weeks (as per Schedule of Assessments) and telephone interviews will be performed every 12 weeks. Please refer to Table 4 for further details.

Please note: It is important to distinguish between "withdrawal from treatment" and "withdrawal from study". Patients who withdraw from treatment should be encouraged to remain in the study for the full duration of the Safety Follow-Up Period (minimum of 48 weeks following the last infusion).

**Every effort should be made to have patients, who withdraw from the study treatment, complete the Safety Follow-Up Period and all related assessments, regardless of whether or not they receive alternative treatment for MS.**

**Table 1: Overview of Dosing Regimen During the Double-Blind, Double-Dummy Treatment Period**

| **Study Medication** | **Double-Blind, Double-Dummy Treatment Period**[1,2] | | | | |
|---|---|---|---|---|---|
| | **1st Cycle**[3] (Weeks 1-24) | | **2nd Cycle**[3] (Weeks 24-48) | **3rd Cycle**[3] (Weeks 48-72) | **4th Cycle**[3] (Weeks 72-96) |
| | **Day 1 Infusion** | **Day 15 Infusion** | **Week 24 Infusion** | **Week 48 Infusion** | **Week 72 Infusion** |
| **A Ocrelizumab 600 mg regimen** | **Ocrelizumab** 300 mg i.v. | **Ocrelizumab** 300 mg i.v. | **Ocrelizumab** 600 mg i.v. | **Ocrelizumab** 600 mg i.v. | **Ocrelizumab** 600 mg i.v. |
| **B Rebif® regimen**[4] | **Rebif®** s.c. 3 times per week | → | → | → | → |

i.v.=intravenous; s.c.=subcutaneous.

1. The double-blind, double-dummy treatment period consists of 96 weeks of treatment (4 treatment cycles).
2. Each treatment cycle has a duration of 24 weeks. The first cycle consists of two 300 mg ocrelizumab i.v. infusions separated by 14 days. Cycles 2 – 4 consist of a single i.v. infusion of 600 mg ocrelizumab.
3. Prior to each infusion, a clinical evaluation will be performed to ensure that the patient remains eligible for treatment.
4. Please refer to Table 2 for detailed Rebif® dosing regimen.

Please note: 100 mg of methylprednisolone i.v. will be administered in both treatment arms prior to each infusions of ocrelizumab/ocrelizumab placebo.

**Table 2: Overview of Rebif® Dosing Regimen During the Double-Blind, Double-Dummy Treatment Period**

| | **Treatment Initiation** | | **Treatment Continuation** | **Dose modification (if required)** |
|---|---|---|---|---|
| **Week** | **Weeks 1- 2** | **Weeks 3-4** | **Week 5 onwards** | – |
| *Study Day* | *1-14* | *15-28* | *29+* | At any time >29 |
| **Dose of Rebif®** | **Rebif® 8.8µg** (one pre-filled syringe [0.2 mL] containing 2.4 MIU of interferon beta-1a) s.c. **3x per week** | **Rebif® 22 µg** (one pre-filled syringe [0.5 mL] containing 6 MIU of interferon beta-1a) s.c. **3x per week** | **Rebif® 44 µg** (one pre-filled syringe [0.5 mL]) containing 12 MIU of interferon beta-1a) s.c. **3x per week** | **Rebif® 22 µg** (one pre-filled syringe [0.5 mL] containing 6 MIU of interferon beta-1a) s.c. **3x per week** |

IxRS=interactive voice and web response system; s.c.=subcutaneous.

Please note: As per discretion of the Treating Investigator, Rebif®/Rebif® placebo dosage can be modified due to safety reasons at any time; investigator should follow local label for further information. if Rebif® dose modification is required due to laboratory abnormalities possibly related to the treatment with Rebif®, the investigator (the treating investigator) will need to notify IxRS and the blinded study medication (Rebif® placebo or Rebif® verum) will be dispensed accordingly. In addition, to ensure patient safety in the study, unscheduled visits may be required for additional assessments, monitoring and for dispensing study medication.

**Table 3: Overview of Dosing Regimen During the OLE Phase Screening Period and the OLE Phase**

| Study Medication | OLE Phase Screening Period | OLE Phase[1] | | | | |
|---|---|---|---|---|---|---|
| | | 5th Cycle[2,3] | | 6th Cycle[2,3] | 7th Cycle[2,3] | Nth Cycle[2,3,4] |
| | | Day 1 Infusion | Day 15 Infusion | | | |
| **Ocrelizumab 600 mg regimen** | -.-[5] | **Ocrelizumab** 300 mg i.v. | **Ocrelizumab** 300 mg i.v. | **Ocrelizumab** 600 mg i.v. | **Ocrelizumab** 600 mg i.v. | **Ocrelizumab 600 mg i.v.** |
| **Rebif® regimen** | **Rebif® s.c.** 3 times per week[6] | -.-[7] | -.-[7] | -.-[7] | -.-[7] | -.-[7] |

i.v.=intravenous; OLE=Open-Label Extension; s.c.=subcutaneous.

1. The OLE Phase can terminate at any moment or cycle (please refer to End of Study Section 3.1.4).
2. The assessments requested for N represents the typical schedule of assessments during a cycle.
3. Prior to each infusion, a clinical evaluation will be performed to ensure that the patient remains eligible for treatment.
4. Each treatment cycle has a duration of 24 weeks. The first cycle of the OLE Phase consists of two 300 mg ocrelizumab i.v. infusions separated by 14 days. Cycle 6 onwards consists of a single i.v. infusion of 600 mg ocrelizumab.
5. During the OLE Phase Screening Period there will be no administration of ocrelizumab.
6. Please refer to Table 2 for the detailed Rebif® dosing regimen.
7. During the OLE Phase, there will be no administration of Rebif® verum or placebo.

Please note: 100 mg of methylprednisolone i.v. will be administered in both treatment arms prior to each infusion of ocrelizumab/ocrelizumab placebo.

**In case of elevation of liver function tests the following rules will apply:**

| | |
|---|---|
| ⇨ **ALT ≥ 10 x ULN**, jaundice or other clinical symptoms of liver dysfunction | In case of detection of elevated **ALT ≥ 10 x ULN**, jaundice or other clinical symptoms of liver dysfunction the injections of Rebif®/Rebif® placebo must be discontinued permanently. The monitoring of liver function tests should be continued on a monthly basis until return to normal baseline levels or CTCAE v.4.0 grade 1 toxicity (ALT: >ULN - 3.0 x ULN). A consultation with hepatologist is recommended. Patients should move to Safety Follow-Up Period. |
| ⇨ **ALT ≥ 5 x ULN** | In case of detection of elevated **ALT ≥ 5 x ULN** (but below 10 xULN) the injections of Rebif®/Rebif® placebo must be discontinued temporarily. Additional blood chemistry panel including AST, ALP, GGT and bilirubin should be performed biweekly until no further increase is observed. Subsequently, ALT analysis has to be performed every month until return to normal baseline levels or CTCAE v.4.0 grade 1 toxicity (ALT >ULN - 3.0 x ULN). A consultation with hepatologist should be considered as per investigator judgment.<br>If causes of toxicity other than possible treatment with Rebif® are excluded, the patient may then be cautiously re-challenged with Rebif®/Rebif® placebo 22µg provided in a blinded fashion upon request to IxRS. The monitoring of liver function tests should continue on a monthly basis. If there is no further recurrence of toxicity, patient may continue treatment with Rebif®/Rebif® placebo 44 µg provided in a blinded fashion upon investigator's request to IxRS. **In case of recurrence of toxicity (ALT > 3 x ULN, or other clinical symptoms of liver dysfunction) the injections of Rebif®/Rebif® placebo should be discontinued permanently.** Patients should move to Safety Follow-Up Period.<br><br><u>Please note:</u> Re-initiation of therapy with Rebif® following elevation of liver function tests can only be considered once. |
| ⇨ **ALT > 3 x ULN** | In case of detection of elevated **ALT > 3 x ULN** (but below 5x ULN) additional blood chemistry panel including AST, ALP, GGT and bilirubin should be performed biweekly until no further increase is observed. Subsequently, ALT analysis has to be performed every month until return to normal baseline levels or CTCAE v.4.0 grade 1 toxicity (ALT>ULN - 3.0 x ULN). |

# Table 4: Schedule of Assessments: Screening through the End of Double-Blind, Double-Dummy Treatment Period

| | **Screen** | **Double-blind, Double-Dummy Treatment Period** | | | | | | | | | | | | |
|---|---|---|---|---|---|---|---|---|---|---|---|---|---|---|
| **Cycle** | | **1** | | | **2** | | **3** | | **4** | | | **Delayed Dosing Visit** [22] | **Unscheduled Visit** [23] | **Withdrawal from Treatment Visit** |
| **Visit** | **1** | **2 BL** | **3** | **4** | **5** | **6** | **7** | **8** | **9** | **10** | **11** | | | |
| **Week** | **-2** | **-** | **w2** | **w12** | **w24** | **w36** | **w48** | **w60** | **w72** | **w84** | **w96** | | | |
| *Study Day (window in days)* | *-14* | *1* | *15 (± 2)* | *85 (±4)* | *169 (± 2)* | *253 (±4)* | *337 (± 2)* | *421 (±4)* | *505 (± 2)* | *589 (±4)* | *673 (± 2)* | | | |
| Informed consent [1] | x | | | | | | | | | x[24] | | | | |
| Medical history | x | | | | | | | | | | | | | |
| Review of eligibility criteria | x | x | | | | | | | | | x[24] | | | |
| CES-D, MFIS, EQ-5D, SF-36 | | x | | | | | x | | | | x | | | x |
| Patient's Assessment of Treatment Benefit | | | | | | | x | | | | x | | | x |
| C-SSRS | | x | | x | x | x | x | x | x | x | x | | x | x |
| Physical examination | x | x | x | | x | | x | | x | | x | x | | x |
| Vital signs [2] | x | x | x | x | x | x | x | x | x | x | x | x | x | x |
| 12 lead ECG (pre- and post-dose) [3] | x | x | | | | | | | x | | | | | x |
| Height | x | | | | | | | | | | | | | |
| Weight | x | | | | | | | | x | | x | | | x |
| Neurological exam and EDSS | x | x | | x | x | x | x | x | x | x | x | | x | x |
| MSFCS, LCVA, SDMT | | x | | x | x | x | x | x | x | x | x | | x | x |
| Karnofsky Performance Status Scale | | x | | | x | | x | | x | | x | | | x |
| MRI[4] | | x | | | x | | x | | | | x | | | x |
| Concomitant Treatment | | x | x | x | x | x | x | x | x | x | x | x | x | x |
| Adverse Events | *Only SAEs* | x | x | x | x | x | x | x | x | x | x | x | x | x |
| Potential relapses recorded | | x | x | x | x | x | x | x | x | x | x | x | x | x |
| Telephone interview (every 4 wks)[5] | | | | – – – – | – – – – | – – – – | – – – – | – – – – | – – – – | – – – – | – – → | | | |

## Table 4: Schedule of Assessments: Screening through the End of Double-Blind, Double-Dummy Treatment Period (Cont.)

| | Screen | Double-Blind, Double-Dummy Treatment Period | | | | | | | | | | | | |
|---|---|---|---|---|---|---|---|---|---|---|---|---|---|---|
| **Cycle** | | 1 | | | 2 | | 3 | | 4 | | | Delayed Dosing Visit [22] | Unscheduled Visit [23] | Withdrawal from Treatment Visit |
| **Visit** | 1 | 2 BL | 3 | 4 | 5 | 6 | 7 | 8 | 9 | 10 | 11 | | | |
| **Week** | -2 | - | w2 | w12 | w24 | w36 | w48 | w60 | w72 | w84 | w96 | | | |
| *Study Day (window in days)* | *-14* | *1* | *15 (±2)* | *85 (±4)* | *169 (± 2)* | *253 (±4)* | *337 (± 2)* | *421 (±4)* | *505 (± 2)* | *589 (±4)* | *673 (± 2)* | | | |
| Pregnancy test [6] | x | x | x | x | x | x | x | x | x | x | x | x | | x |
| Antibody Titers [7] | | x | | x | x | | x | | x | | x | | | x |
| RCR (non-DNA) [8] | | x | | x | x | | x | | x | | x | | | x |
| RCR (DNA) [9] | | x | | | | | | | | | | | | |
| Protein biomarker sampling [10] | | x | | x | x | | x | | x | | x | | | x |
| HAHA [11] | | x | | | x | | x | | x | | x | | | x |
| Plasma/ urine banking for JCV[12] | | x | | x | x | x | x | x | x | x | x | | | x |
| PK Samples [13] | | x | | | x | | x | | x[13] | x | x | | | x |
| Thyroid function tests [14] | x | | | | x | | x | | x | | x | | | x |
| FSH [15] | x | | | | | | | | | | | | | |
| Hepatitis Screening [16] | x | | | | | | | | | | | | | |
| Hepatitis B virus DNA[16] | x | (x) | | (x) | (x) | (x) | (x) | (x) | (x) | (x) | (x) | | | (x) |
| RPR | x | | | | | | | | | | | | | |
| CD4 count | x | | | x | | x | | x | | x | | | | |
| IgG | | | | x | | x | | x | | x | | | | |
| Total Ig, IgA, IgG, IgM | x | | | | x | | x | | x | | x | | | x |
| FACS [17] | | x | x | x | x | | x | | x | | x | | | x |

## Table 4: Schedule of Assessments: Screening through the End of Double-Blind, Double-Dummy Treatment Period (Cont.)

| | Screen | Double-Blind, Double-Dummy Treatment Period | | | | | | | | | | | | |
|---|---|---|---|---|---|---|---|---|---|---|---|---|---|---|
| **Cycle** | | 1 | | | 2 | | 3 | | 4 | | | Delayed Dosing Visit [22] | Unscheduled Visit [23] | Withdrawal from Treatment Visit |
| **Visit** | 1 | 2 BL | 3 | 4 | 5 | 6 | 7 | 8 | 9 | 10 | 11 | | | |
| **Week** | -2 | - | w2 | w12 | w24 | w36 | w48 | w60 | w72 | w84 | w96 | | | |
| *Study Day (window in days)* | *-14* | *1* | *15 (± 2)* | *85 (±4)* | *169 (± 2)* | *253 (±4)* | *337 (± 2)* | *421 (±4)* | *505 (± 2)* | *589 (±4)* | *673 (± 2)* | | | |
| | | | | | | | | | | | | | | |
| Routine safety lab [18] | x | x | x | x | x | x | x | x | x | x | x | | | x |
| Type I interferon neutralizing antibody assay [19] | | x | | | x | | x | | x | | x | | | x |
| Pre-treatment with i.v. methylprednisolone [20] | | x | x | | x | | x | | x | | | x | | |
| Administration of i.v. ocrelizumab / ocrelizumab placebo [21] | | x | x | | x | | x | | x | | | x | | |
| Assessment of s.c. Rebif®/ Rebif® placebo compliance | | x | x | x | x | x | x | x | x | x | x | | x | x |
| Administration of s.c. Rebif® / Rebif® placebo 3x/wk | | x | x | x | x | x | x | x | x | x | x[25] | | | |

β-hCG=beta human chorionic gonadotropin; BL=baseline; CD4=cluster of differentiation; CES-D=Center for Epidemiologic Studies Depression Scale; C-SSRS=Columbia-Suicide Severity Rating Scale; DNA=deoxyribonucleic acid; ECG=electrocardiogram; eCRF=electronic case report form; EDSS=Expanded Disability Status Scale; EDTA=ethylenediaminetetraacetic acid; EQ-5D=EuroQoL; FACS=fluorescence activated cell sorting; FSH=follicle-stimulating hormone; HAHA=human anti-human antibodies; Ig=immunoglobulin; IgA=immunoglobulin A; IgG=immunoglobulin G; IgM=immunoglobulin M; i.v.=intravenous; JCV=JC virus; LCVA=Low Contrast Visual Acuity; MFIS=Modified Fatigue Impact Scale; MRI=magnetic resonance imaging; MSFCS=Multiple Sclerosis Functional Composite Scale; n=number; OLE=Open-Label Extension; PK=pharmacokinetic; RCR=Roche Clinical Repository; RNA=ribonucleic acid; RPR=rapid plasma reagin; SAE=serious adverse event; s.c.=subcutaneous; SDMT=Symbol Digit Modalities Test; SF-36=Short-Form 36; w=week.

## Table 4: Schedule of Assessments: Screening through the End of Double-Blind, Double-Dummy Treatment Period (Cont.)

1. **Informed Consent** must be obtained in written form from all patients at screening (prior to any study–related procedure) in order to meet eligibility for the study.
2. **Vital signs** (i.e., pulse rate, systolic and diastolic blood pressure, respiration rate and temperature) will be obtained while the patient is in the semi supine position (after 5 minutes). On infusion visits, the vital signs should be taken within 45 minutes prior to the methylprednisolone infusion in all patients. In addition, vital signs should be obtained prior to ocrelizumab/ocrelizumab placebo infusion, then every 15 minutes (± 5 minutes) for the first hour; then every 30 minutes (± 10 minutes) until 1 hour after the end of the infusion. On non-infusion days, the vital signs may be taken at any time during the visit.
3. **ECG (pre- and post-dose):** on infusion visits ECG should be taken within 45 minutes prior to the methylprednisolone infusion in all patients, and within 60 minutes after completion of the ocrelizumab/ocrelizumab placebo infusion. On non-infusion days, the ECG may be taken at any time during the visit.
4. **MRI**: brain MRI scans *should occur within a window or ± 4 weeks of the scheduled visit. Also, brain MRI scans* will be obtained in patients withdrawn from the treatment period (at a withdrawal visit) if not performed during last 4 weeks.
5. **A structured telephone interview** will be conducted by site personnel every 4 weeks (± 3 days) from Week 8 through the study to identify any new or worsening neurological symptoms that warrant an unscheduled visit and collect data on possible events of infections.
6. **Serum β-hCG** must be performed at screening in women of childbearing potential. Subsequently, urine β-hCG [sensitivity of at least 25 mIU/mL] will be performed. On infusion visits, the urine pregnancy test should be performed prior to methylprednisolone infusion in all women of child-bearing potential. If positive, the patient will not receive the scheduled dose and confirmation, a serum pregnancy test, will be performed.
7. **Antibody Titers**: measurement of antibody titers against common antigens (mumps, rubella, varicella and *Streptococcus pneumoniae*) will be performed.
8. **RCR - Roche Clinical Repository non-DNA (RNA – and protein):** for RNA 2x 2.5 mL whole blood samples to be collected from consenting patients only for expression profiling analysis. For protein: 6 mL blood samples in EDTA tube for plasma samples will be collected from consenting patients only for analysis of protein biomarkers. On infusion visits, ALL samples should be collected 5-30 minutes prior to methylprednisolone infusion.
9. **RCR - Roche Clinical Repository (DNA):** 6 mL whole blood sample to be collected from only from patients consenting to RCR for pharmacogenetic and genetic analysis. If not done at Baseline (Visit 2), sample may be collected at next visit.
10. **Protein biomarker sampling:** one serum sample (6 mL) will be collected from all patients for analysis of protein biomarkers. On infusion visits, samples should be collected 5-30 minutes prior to methylprednisolone infusion.
11. **HAHA**: On infusion visits, serum samples are collected 5-30 minutes prior to the methylprednisolone infusion.
12. **Plasma and urine samples for JCV will be collected** at specified time points and analyzed in batches, if decided by the Sponsor.

## Table 4: Schedule of Assessments: Screening through the End of Double-Blind, Double-Dummy Treatment Period (Cont.)

13. **PK samples:** on the infusion day at week 72, two serum samples should be collected, one 5-30 minutes prior to the methylprednisolone infusion and the second one 30 minutes (±10 minutes) following the completion of the ocrelizumab/ocrelizumab placebo infusion. For all other infusion visits, a blood sample should be collected 5 – 30 minutes before the methylprednisolone infusion. At other times (non-infusion visits) samples may be collected at any time during the visit.
14. **Sensitive thyroid-stimulating hormone (sTSH)** will be tested at screening and during the double-blind, double-dummy treatment period. Thyroid autoantibodies will be assayed only at screening.
15. **FSH**: only applicable to women to confirm the postmenopausal status.
16. **Hepatitis** screening & monitoring: all patients must have negative hepatitis B surface antigen (HbsAg) result and negative hepatitis C antibody (HepCAb) screening tests prior to enrollment. If total hepatitis B core antibody (HbcAb) is positive at screening, HB virus DNA measured by polymerase chain reaction (PCR) must be negative to be eligible. For those patients enrolled with negative HBsAg and positive total HBcAb, HB virus DNA (PCR) must be repeated every 12 weeks during the treatment period.
17. **FACS**: including CD19 and other circulating B-cell subsets, T-cells, natural killer cells, and other leukocytes. On infusion visits, blood samples should be collected prior to the infusion of methylprednisolone.
18. **Routine safety lab:** hematology, chemistry and urinalysis: on infusion visits, all urine and blood samples should be collected prior to the infusion of methylprednisolone. At other times, samples may be collected at any time during the visit.
19. **Type I interferon neutralizing antibody assay:** At baseline (visit 2), sample should be collected before first Rebif®/Rebif® placebo injection. At subsequent visits, samples should be collected at least 36 hours following last injection of Rebif®/Rebif® placebo.
20. All patients receive **prophylactic treatment** with 100 mg of methylprednisolone i.v. prior to infusion of ocrelizumab /ocrelizumab placebo. In the rare case when the use of methylprednisolone is contraindicated for the patient, use of an equivalent dose of an alternative steroid should be used as premedication prior to the infusion. It is also recommended that patients receive an analgesic/antipyretic such as acetaminophen/paracetamol (1 g) and an i.v. or oral antihistaminic such as diphenhydramine 50 mg 30-60 minutes prior to ocrelizumab/ ocrelizumab placebo.
21. **Administration (infusion) of i.v. ocrelizumab/ocrelizumab placebo**: the Treating Investigator must review the clinical and laboratory re-treatment criteria prior to subsequent infusion of ocrelizumab/ocrelizumab placebo.
22. **A delayed dosing visit** will be performed and recorded in the Delayed Dosing Visit eCRF form when dosing cannot be administered at the scheduled dosing visit. Other tests or assessments may be done as appropriate.

## Table 4: Schedule of Assessments: Screening through the End of Double-Blind, Double-Dummy Treatment Period (Cont.)

23. **Unscheduled Visit:** assessments performed at unscheduled (non-dosing) visits will depend on the clinical needs of the patient. All patients with new neurological symptoms suggestive of relapse should have EDSS performed by examining Investigator, *whenever possible within 7 days of the onset of the relapse*. Other tests/assessments may be done as appropriate. Please note: in case of alanine aminotransferase (ALT) elevations dose modification should be necessary, additional visits may be required for dispensing of study medication.
24. At the Week 84 Visit, a discussion with the patient regarding participation in the Open-Label Extension Phase should occur if the Treating Investigator is of the opinion that the patient may benefit from treatment with ocrelizumab. An informed consent for the OLE Phase should be provided to the patient. It is recommended that the Informed Consent Form of the Open-Label Extension Phase be signed at the Week 96 Visit.
25. If the patient enters OLE Phase Screening Period, Rebif® / Rebif® placebo should be provided to the patient at the Week 96 Visit (please see Section 5.10.1).

Please note: based on local Ethics Committees or National Competent Authority requirements, additional diagnostic testing may be required for selected patients or selected centers to exclude tuberculosis, Lyme disease, HTLV-1 associated myelopathy (HAM), acquired immune deficiency syndrome (AIDS), hereditary disorders, connective tissue disorders, or sarcoidosis. Other specific diagnostic tests may be requested when deemed necessary by the Investigator.

## Table 5: Schedule of Assessments: Safety Follow-Up (including prolonged B-cell monitoring if required)

| | **Safety Follow-Up** | **Prolonged B-cell Monitoring**[1] | **End of observation or withdrawal from Safety Follow up** |
|---|---|---|---|
| **Assessments** | *Visits every 12 weeks (±7 days)* [2] | *Visits every 24 weeks (±7 days)* | |
| Urine pregnancy test | x | x | x |
| Routine Safety Labs[3] | x | x | x |
| FACS [4] | x | x | x |
| Total Ig, IgA, IgG, IgM | x[10] | x | x |
| HAHA [5] | x[10] | x | x |
| Plasma/urine banking for JCV | x | x | x |
| Antibody titers | x[10] | x | x |
| Hepatitis B viral DNA[6] | (x) | (x) | (x) |
| Protein biomarker sampling [8] | x[10] | x | x |
| Vital Signs | x | x | x |
| EDSS | x | | x |
| Neurological examination | x | x | x |
| Physical examination | x[10] | x | x |
| Potential relapses recorded | x | x | x |
| Adverse events | x | x | x |
| Concomitant Medication | x | x | x |
| Telephone interview [9] | x | x | |

CD=cluster of differentiation; DNA=deoxyribonucleic acid; EDSS=Expanded Disability Status Scale; EDTA=ethylenediaminetetraacetic acid; FACS=fluorescence activated cell sorting; HAHA=human anti-human antibodies; HbcAb=hepatitis C antibody; HbsAg=hepatitis B surface antigen; HBV=hepatitis B virus; Ig=immunoglobulin; IgA=immunoglobulin A; IgG=immunoglobulin G; IgM=immunoglobulin M; i.v.=intravenous; JCV=JC virus; RNA=ribonucleic acid.

## Table 5: Schedule of Assessments: Safety Follow-Up (including prolonged B-cell monitoring if required) (Cont.)

1. **Prolonged B-cell monitoring**: patients whose B-cells have not been repleted after 48 weeks of Safety Follow-Up Period will continue with visits every 24 weeks (± 7 days) until B-cell repletion.
2. **Safety Follow-Up** will be carried out for at least 48 weeks starting from the date of last infusion of ocrelizumab. Visits will be performed at 12-week intervals starting from the date of the patient's Withdrawal From Treatment Visit. *Safety Follow-Up applies to study patients who have completed the blinded treatment period (or open label treatment period, if applicable) and to patients who withdraw early from treatment. If B-cells have returned to normal levels at this visit, then the 48 week Safety Follow-up visit will become the End of Observation visit and the patient will have completed the study. An End of observation visit will be performed only in case of occurrence of new adverse event and/or if the investigator considers the prior safety assessment (laboratory, other) as abnormal and clinically significant.*
3. **Routine safety lab**: hematology, chemistry and urinalysis.
4. **FACS** including CD19 and other circulating B-cell subsets, T cells, natural killer cells and other leukocytes.
5. **HAHA**: two serum samples are required.
6. **Hepatitis monitoring:** hepatitis to be monitored only in patients with screening results of HbsAg negative, HBcAb positive and HBV DNA negative, inclusive.
7. **Protein biomarker sampling**: 6 mL blood sample in a plain tube without EDTA for serum isolation will be collected from all patients for analysis of protein biomarkers.
8. **A structured telephone interview** will be performed by site personnel every 4 weeks (± 3 days) between visits until 48 weeks after the last infusion to identify any new or worsening neurological symptoms that warrant an unscheduled visit and collect data on possible events of infections. If prolonged B-cell monitoring is required beyond 48 weeks after the last infusion, telephone interviews will be done every 12 weeks (± 7 days) between visits.
9. Needs to be assessed only every 24 weeks.

<u>Please note:</u> patients in Safety Follow-Up who receive other B-cell targeted therapies will only be followed for 48 weeks from the date of the last infusion of the study drug regardless of their B-cell count.

## Table 6: Schedule of Assessments: Open Label Extension Phase

| | OLE Screen | Open-Label Extension Phase[1] | | | | | | | | | | | Delayed dosing Visit[20] | Unscheduled Visit[21] | Withdrawal from Treatment Visit |
|---|---|---|---|---|---|---|---|---|---|---|---|---|---|---|---|
| **Cycle[1]** | | 5 | | | 6[1] | | 7[1] | | 8[1] | | N[1,3] | | | | |
| **Visit** | | 12 | 13 | 14 | 15 | 16 | 17 | 18 | 19 | 20 | … | … | | | |
| **Week in OLE Phase (window in days)** | | *0*[2] | *2 (± 2)* | *12 (± 7)* | *22 (± 7)* | *24 (±5)* | *46 (± 7)* | *48 (± 5)* | *70 (± 7)* | *72 (± 5)* | *n-2 wk (± 7)* | *n (± 7)* | | | |
| Informed consent[4] | x | | | | | | | | | | | | | | |
| Review of eligibility criteria | x | x | | | | | | | | | | | | | |
| Review of retreatment criteria | | x | x | | | x | | x | | x | | x | x | | |
| Administration of s.c. Rebif® / Rebif® placebo 3x/wk [5] | x | | | | | | | | | | | | | | |
| Assessment of s.c. Rebif®/ Rebif® placebo compliance | | x | | | | | | | | | | | | | |
| Pre-treatment with i.v. methylprednisolone[6] | | x | x | | | x | | x | | x | | x | x | | |
| Administration of i.v. ocrelizumab[7] | | x | x | | | x | | x | | x | | x | x | | |
| Physical examination | | x | x | | | x | | x | | x | | x | x | | x |
| Vital signs, weight[8] | | x | x | x | | x | | x | | x | | x | x | x | x |
| 12-lead ECG (pre- and post-dose, once early)[9] | | x | x | | | | | | | x | | (x) | | | x |
| Neurological exam and EDSS | | x | | x | | x | | x | | x | | x | | x | x |
| Routine safety labs[10] | | x | | x | x | | x | | x | | x | | | x | x |
| Adverse events | | x | x | x | x | x | x | x | x | x | x | x | x | x | x |
| Concomitant treatments | | x | x | x | x | x | x | x | x | x | x | x | x | x | x |
| Potential relapses recorded | | x | x | x | x | x | x | x | x | x | x | x | x | x | x |
| Pregnancy test[11] | | x | x | x | | x | | x | | x | | x | x | | x |
| Antibody titers[12] | | x | | x | x | | x | | x | | x | | | | x |
| Total Ig, IgA, IgG, IgM | | x | | | x | | x | | x | | x | | | | |
| CD4 count | | x | x | | x | | x | | x | | x | | | | |

## Table 6: Schedule of Assessments: Open Label Extension Phase (Cont.)

| | OLE Screen | Open-Label Extension Phase[1] | | | | | | | | | | | Delayed dosing Visit[20] | Unscheduled Visit[21] | Withdrawal from Treatment Visit |
|---|---|---|---|---|---|---|---|---|---|---|---|---|---|---|---|
| **Cycle[1]** | | **5** | | | **6[1]** | | **7[1]** | | **8[1]** | | **N[1,3]** | | | | |
| **Visit** | | **12** | **13** | **14** | **15** | **16** | **17** | **18** | **19** | **20** | **…** | **…** | | | |
| **Week in OLE Phase (window in days)** | | *0[2]* | *2* (± 2) | *12* (± 7) | *22* (± 7) | *24* (±5) | *46* (± 7) | *48* (± 5) | *70* (± 7) | *72* (± 5) | *n-2 wk* (± 7) | *n* (± 7) | | | |
| HAHA[22] | | x | | | x | | x | | x | | x | | | | *x* |
| *Ocrelizumab concentration sample[22]* | | *x* | | | *x* | | *x* | | *x* | | *x* | | | | *x* |
| Telephone interview (every 4 weeks)[13] | | | | – – – – – – – – – – – – – – – – – – → | | | | | | | | | | | |
| EQ-5D (once yearly) | | x | | | | | x | | | | (x) | | | | |
| Protein biomarker sampling[15] | | x | | x | x | | x | | x | | x | | | | |
| FACS[16] | | | x | x | x | | x | | x | | x | | | | |
| Hepatitis B virus DNA[17] | | | | (x) | (x) | | (x) | | (x) | | (x) | | | | |
| MRI (once yearly)[18] | | | | | | | x | | | | (x) | | | | |
| Patient's Assessment of Treatment Benefit (once yearly) | | | | | | | x | | | | (x) | | | | |
| Plasma/urine banking for JCV[19] | | x | | x | x | | x | | x | | x | | | | x |

CD4 = cluster of differentiation; DNA = deoxyribonucleic acid; ECG = electrocardiogram; eCRF = electronic case report form; EDSS = Expanded Disability Status Scale; EQ-5D = EuroQoL; FACS = fluorescence activated cell sorting; HAHA = human anti-human antibodies; Ig = immunoglobulin; IgA = immunoglobulin A; IgG = immunoglobulin G; IgM = immunoglobulin M; i.v. = intravenous; JCV = JC virus; MRI = magnetic resonance imaging; n = number; OLE = Open-Label Extension; RCR = Roche Clinical Repository; s.c. = subcutaneous.

1. **The OLE Phase** can terminate at any moment or cycle (please refer to End of Study Section 3.1.4). In case the study is ended, a Withdrawal from Treatment Visit should occur.
2. **The OLE Phase** starts at the first infusion of Cycle 5. The OLE Phase Screening Period will start after all assessments at the Week 96 Visit have been performed, and it should last approximately 4 weeks. It is possible that the OLE Phase Screening Period could be longer than 4 weeks. If a prolongation of the OLE Phase Screening Period is needed, it should be discussed with the Sponsor on a case-by-case basis.
3. The assessments requested for N represent the typical schedule of assessments during a cycle.
4. The **informed consent** should have been provided to the patient at the Week 84 Visit and signed by the patient at the Week 96 Visit.

## Table 6: Schedule of Assessments: Open Label Extension Phase (Cont.)

5. During the OLE Phase Screening Period, s.c. administration Rebif® / Rebif® placebo 3 times per week should occur until one day prior to the first infusion of ocrelizumab of Cycle 5 (beginning of OLE Phase). If during this period, the patient decides not to participate in the OLE Phase, then administration of Rebif® / Rebif® placebo 3 times per week should stop immediately, and the patient will be entered in the Safety Follow-Up Period.
6. All patients receive prophylactic treatment with 100 mg of methylprednisolone i.v. prior to infusion of ocrelizumab. In the rare case when the use of methylprednisolone is contraindicated for the patient, use of an equivalent dose of an alternative steroid should be used as premedication prior to the infusion. It is also recommended that patients receive an analgesic/antipyretic such as acetaminophen/paracetamol (1 g) and an i.v. or oral antihistaminic such as diphenhydramine 50 mg 30-60 minutes prior to ocrelizumab.
7. **Administration (infusion) of i.v. ocrelizumab**: the Investigator must review the clinical and laboratory re-treatment criteria prior to subsequent infusion of ocrelizumab.
8. **Vital signs** (i.e., pulse rate, systolic and diastolic blood pressure, respiration rate, and temperature) will be obtained while the patient is in the semi-supine position (after 5 minutes). Vital signs should be collected within 45 minutes prior to the methylprednisolone infusion in all patients. In addition, vital signs should be obtained prior to ocrelizumab infusion, then every 15 minutes (± 5 minutes) for the first hour; then every 30 minutes (± 10 minutes) until 1 hour after the end of the infusion.
9. **ECGs** (pre- and post-dose): ECG should be performed within 45 minutes prior to the methylprednisolone infusion in all patients, and within 60 minutes after completion of the ocrelizumab infusion.
10. **Routine safety lab**: hematology, chemistry, and urinalysis. On infusion visits at Cycle 5, all urine and blood samples should be collected prior to the infusion of methylprednisolone. At other times, samples may be collected at any time during the visit.
11. **Urine β-hCG** [sensitivity of at least 25 mIU/mL] will be performed. On infusion visits, the urine pregnancy test should be performed prior to methylprednisolone infusion in all women of childbearing potential. If positive, the patient will not receive the scheduled dose and for confirmation a serum pregnancy test will be performed.
12. **Antibody Titers**: measurement of antibody titers against common antigens (mumps, rubella, varicella, and Streptococcus pneumoniae) will be performed.
13. A structured **telephone interview** will be conducted by site personnel every 4 weeks (± 3 days) from Cycle 5 *(Week 8 of OLE Phase)* through the study to identify any new or worsening neurological symptoms that warrant an unscheduled visit and collect data on possible events of infections.
14. **Protein biomarker sampling**: one serum sample (6 mL) will be collected from all patients for analysis of protein biomarkers. On infusion visits, samples should be collected 5-30 minutes prior to methylprednisolone infusion.
15. **FACS**: including CD19 and other circulating B-cell subsets, T-cells, natural killer cells, and other leukocytes.
16. **Hepatitis monitoring**: For those patients enrolled with negative HBsAg and positive total HBcAb, HB virus DNA (PCR) must be repeated every 24 weeks during the treatment period.
17. **MRI**: brain MRI scans *should occur within a window of ± 4 weeks of the scheduled visit. Also, brain MRI scans* will be obtained in patients withdrawn from the OLE Phase (at a withdrawal visit) if not performed during last 4 weeks.
18. **Plasma and urine samples for JCV** will be collected at specified time points
19. A **delayed dosing visit** will be performed and recorded in the Delayed Dosing Visit eCRF form when dosing cannot be administered at the scheduled dosing visit. Other tests or assessments may be done as appropriate.

### Table 6: Schedule of Assessments: Open Label Extension Phase (Cont.)

*20.* **Unscheduled Visit**: assessments performed at unscheduled (non-dosing) visits will depend on the clinical needs of the patient. All patients with new neurological symptoms suggestive of relapse should have EDSS performed*, whenever possible within 7 days of the onset of the relapse*. Other tests/assessments may be done as appropriate.

*21.* ***HAHA and Ocrelizumab drug concentration samples**: At the infusion Visit 12 (Cycle 5), a blood sample should be collected before the methylprednisolone infusion. At non-infusion visits, samples may be collected at any time during the visit.*

## SAMPLE SIZE AND STATISTICAL ANALYSES

The sample size for this study has been estimated based on data from previous RRMS trials, with the use of two-sided tests with an experiment-wise alpha of 0.05. The annualized rate of relapse among patients receiving ocrelizumab at 96 weeks is predicted to be 0.165 (standard deviation of approximately 0.60), as compared with 0.33 (standard deviation of approximately 0.80) among patients receiving the control treatment, Rebif® (this represents a relative reduction of 50% on ocrelizumab compared to the active comparator). For the annualized relapse rate, a t-test has been used to determine the sample size between ocrelizumab and the control arm. The sample size of 400 patients per arm provides 84 percent power, maintaining the type I error rate of 0.05, and assuming a dropout rate of 20 percent approximately (assuming relative reduction among patients drop out is 25%).

For *confirmed disability* progression, a two group test of equal exponential survival with exponential dropout is used to determine the sample size. Assuming the 2 year *confirmed* disability progression rate is 18% for the Rebif® arm and 12.6% for the ocrelizumab arm (this represents a relative reduction of 30% on ocrelizumab compared to the active comparator), and assuming a dropout rate of 20 percent over 2 years approximately, the sample size of 400 per arm will provide 80 percent power, maintaining the type I error rate of 0.05 based on the pooled analysis of two identical RMS trials (800 patients treated with ocrelizumab 600 mg and 800 patients treated with Rebif®).

All eligible patients will be randomized to treatment (with 1:1 ratio to ocrelizumab 600 mg regimen or the control arm) stratified by region (US versus ROW) and baseline EDSS (<4.0 versus ≥4.0).

The primary efficacy endpoint is the annualized relapse rate by 96 weeks. The annualized relapse rates at 96 weeks will be *analyzed* using negative binomial model, adjusting for region (United States versus ROW) and baseline EDSS (<4.0 versus ≥4.0). The adjusted annualized relapse rates and the 95% confidence intervals for the relapse rates will be presented along with the p-value.

Summaries of safety data will be produced using data from all patients who have received any study treatment.

## Table of Contents

1. Background and Rationale . . . . . . . . . . . . . . . . . . . . . . . . . . . . . . . . . . . . . . 51
1.1 Background . . . . . . . . . . . . . . . . . . . . . . . . . . . . . . . . . . . . . . . . . . 51
1.1.1 Multiple Sclerosis . . . . . . . . . . . . . . . . . . . . . . . . . . . . . . . . . . . . . 51
1.1.2 Ocrelizumab . . . . . . . . . . . . . . . . . . . . . . . . . . . . . . . . . . . . . . . . 52
1.1.3 Rationale for Targeting B-cells in MS . . . . . . . . . . . . . . . . . . . . . . . 53
1.1.4 Sponsor Experience with Anti-CD20 Compounds in MS . . . . . . . . . 54
1.1.4.1 Ocrelizumab in RRMS . . . . . . . . . . . . . . . . . . . . . . . . . . . . . . . . 54
1.1.4.2 Long-Term Results of Phase II Study WA21493/ACT4422G . . . . 56
1.1.4.3 Rituximab in RRMS . . . . . . . . . . . . . . . . . . . . . . . . . . . . . . . . . 57
1.1.4.4 Rituximab in PPMS . . . . . . . . . . . . . . . . . . . . . . . . . . . . . . . . . 58
1.1.5 Rebif® . . . . . . . . . . . . . . . . . . . . . . . . . . . . . . . . . . . . . . . . . . . . 59
1.2 Rationale for the Study . . . . . . . . . . . . . . . . . . . . . . . . . . . . . . . . . . 61

2. Objectives . . . . . . . . . . . . . . . . . . . . . . . . . . . . . . . . . . . . . . . . . . . . 62
2.1 Primary Objective . . . . . . . . . . . . . . . . . . . . . . . . . . . . . . . . . . . . . 62
2.2 Secondary Objectives . . . . . . . . . . . . . . . . . . . . . . . . . . . . . . . . . . . 62
2.3 Exploratory Objectives . . . . . . . . . . . . . . . . . . . . . . . . . . . . . . . . . . 63
2.4 Roche Clinical Repository Exploratory Objectives . . . . . . . . . . . . . . . . 64
2.5 Optional Exploratory Substudies . . . . . . . . . . . . . . . . . . . . . . . . . . . . 64
2.5.1 Optical Coherence Tomography Exploratory Substudy . . . . . . . . . . 64

3. Study Design . . . . . . . . . . . . . . . . . . . . . . . . . . . . . . . . . . . . . . . . . . 65
3.1 Overview of Study Design and Dosing Regimen . . . . . . . . . . . . . . . . . 65
3.1.1 Rationale for Study Design . . . . . . . . . . . . . . . . . . . . . . . . . . . . . . 71
3.1.2 Rationale for Dose Selection . . . . . . . . . . . . . . . . . . . . . . . . . . . . . 74
3.1.3 End of Double-Blind, Double-Dummy Treatment Period of the Study . . . . . . . . . . . . . . . . . . . . . . . . . . . . . . . . . . . . . . . . . 75
3.1.4 End of Study . . . . . . . . . . . . . . . . . . . . . . . . . . . . . . . . . . . . . . . . 75
3.2 Number of Subjects / Assignment to Treatment Groups . . . . . . . . . . . 75
3.3 Centers . . . . . . . . . . . . . . . . . . . . . . . . . . . . . . . . . . . . . . . . . . . . . 76

4. Study Population . . . . . . . . . . . . . . . . . . . . . . . . . . . . . . . . . . . . . . . 76
4.1 Overview . . . . . . . . . . . . . . . . . . . . . . . . . . . . . . . . . . . . . . . . . . . . 76
4.1.1 Recruitment Procedures . . . . . . . . . . . . . . . . . . . . . . . . . . . . . . . . 76

4.2 Inclusion Criteria . . . . . . . . . . 76
4.3 Exclusion Criteria . . . . . . . . . . 77
4.4 Eligibility Criteria for Open-Label Extension Phase . . . . . . . . . . 80
4.5 Concomitant Medication and Treatment . . . . . . . . . . 80
4.5.1 Definition of Concomitant Treatment . . . . . . . . . . 80
4.5.2 Treatment for Symptoms of Multiple Sclerosis . . . . . . . . . . 80
4.5.2.1 Prohibited Concomitant Treatments . . . . . . . . . . 81
4.5.3 Immunization . . . . . . . . . . 81
4.6 Criteria for Premature Withdrawal . . . . . . . . . . 82
4.6.1 Withdrawal of Subjects from the Roche Clinical Repository . . . . . . . . . . 84
4.6.2 Patient Agreement for Continuation in the Study (in Case of Confirmed Disability Progression) . . . . . . . . . . 84
4.7 Replacement Policy (Ensuring Adequate Numbers of Evaluable Subjects) . . . . . . . . . . 84
4.7.1 For Subjects . . . . . . . . . . 84
4.7.2 For Centers . . . . . . . . . . 84

5. Schedule of Assessment and Procedures . . . . . . . . . . 85

5.1 Screening Examination and Eligibility Screening Form . . . . . . . . . . 97
5.2 Procedures for Enrollment of Eligible Subjects . . . . . . . . . . 97
5.3 Clinical Assessments and Procedures During the Double Blind, Double-Dummy Treatment Period . . . . . . . . . . 98
5.3.1 Overview of Clinical Visits During the Double-Blind, Double-Dummy Treatment Period . . . . . . . . . . 98
5.3.1.1 Delayed Dosing Visit . . . . . . . . . . 99
5.3.1.2 Unscheduled Visits . . . . . . . . . . 99
5.3.1.3 Withdrawal Visits . . . . . . . . . . 100
5.3.2 Assessment of Efficacy . . . . . . . . . . 100
5.3.2.1 Assessment of Relapse . . . . . . . . . . 100
5.3.2.2 Assessment of Disability . . . . . . . . . . 101
5.3.2.3 Kurtzke Expanded Disability Status Scale . . . . . . . . . . 101
5.3.2.4 The Multiple Sclerosis Functional Composite Scale . . . . . . . . . . 102
5.3.2.5 Low-Contrast Visual Acuity Testing . . . . . . . . . . 102
5.3.2.6 The Symbol Digit Modalities Test . . . . . . . . . . 102
5.3.3 Brain Magnetic Resonance Imaging . . . . . . . . . . 102

5.3.4 Safety . . . . . 103
5.3.4.1 Electrocardiogram . . . . . 104
5.3.4.2 Physical Examination . . . . . 104
5.3.4.3 Neurological Examination . . . . . 104
5.3.4.4 Telephone Interviews . . . . . 104
5.3.4.5 Columbia-Suicide Severity Rating Scale . . . . . 105
5.3.5 The Karnofsky Performance Scale (Clinician-Reported Version) . . . 105
5.4 Laboratory Assessments . . . . . 105
5.4.1 Standard Laboratory Assessments . . . . . 106
5.4.2 Hepatitis Screening and Liver Function Monitoring . . . . . 107
5.4.3 Plasma and Urine Banking for JC Virus . . . . . 108
5.4.4 Pharmacokinetic/Pharmacodynamic Assessments . . . . . 108
5.4.5 Type I Interferon Neutralizing Antibody Assay . . . . . 108
5.5 Roche Clinical Repository Specimen(s) . . . . . 108
5.5.1 Specimen Types . . . . . 109
5.6 Protein Biomarker Samples . . . . . 110
5.7 Patient Reported Outcome(s) . . . . . 110
5.7.1 Modified Fatigue Impact Scale . . . . . 110
5.7.2 The Center for Epidemiologic Studies Depression Scale . . . . . 111
5.7.3 The Short Form Health Survey . . . . . 111
5.7.4 Patient’s Assessment of Treatment Benefit . . . . . 111
5.8 Pharmacoeconomic Assessments/EQ-5D . . . . . 111
5.9 Optional Exploratory Substudies . . . . . 112
5.9.1 Optical Coherence Tomography Exploratory Substudy . . . . . 112
5.10 Open-Label Extension Phase . . . . . 112
5.10.1 Open-Label Extension Phase Screening Period . . . . . 112
5.10.2 Open-Label Extension Phase . . . . . 113
5.10.3 Overview of Schedule of Assessments in the Open-Label Extension Phase . . . . . 114
5.10.3.1 Delayed Dosing Visit in the Open-Label Extension Phase . . . . . 114
5.10.3.2 Unscheduled Visits in the Open-Label Extension Phase . . . . . 114
5.10.3.3 Withdrawal Visits in the Open-Label Extension Phase . . . . . 115

6. Investigational Medicinal Product . . . . . 115
6.1 Ocrelizumab . . . . . 116

6.1.1 Preparation and Administration of Ocrelizumab Infusions . . . . . . . . 116
6.1.2 Prevention and Treatment of Infusion Related Reactions . . . . . . . . . 118
6.1.3 Ocrelizumab Dose Modifications, Interruptions and Delays . . . . . . . 119
6.1.4 Criteria for Re-Treatment with Ocrelizumab . . . . . . . . . . . . . . . . . . 119
6.2 Rebif® . . . . . . . . . . . . . . . . . . . . . . . . . . . . . . . . . . . . . . . . . . . . . 120
6.2.1 Dose and Schedule of Rebif® . . . . . . . . . . . . . . . . . . . . . . . . . . . . 120
6.2.2 Rebif® Dose Modifications, Interruptions and Delays . . . . . . . . . . . 121
6.3 Formulation, Packaging and Labeling . . . . . . . . . . . . . . . . . . . . . . . . 123
6.3.1 Ocrelizumab . . . . . . . . . . . . . . . . . . . . . . . . . . . . . . . . . . . . . . . . . 123
6.3.2 Rebif® . . . . . . . . . . . . . . . . . . . . . . . . . . . . . . . . . . . . . . . . . . . . 124
6.4 Blinding and Unblinding . . . . . . . . . . . . . . . . . . . . . . . . . . . . . . . . . 124
6.5 Accountability of Investigational Medicinal Product and Assessment of Compliance . . . . . . . . . . . . . . . . . . . . . . . . . . . . . . . . . . . . . . . . . 125
6.5.1 Accountability of Investigational Medicinal Product . . . . . . . . . . . . . 125
6.5.2 Assessment of Compliance . . . . . . . . . . . . . . . . . . . . . . . . . . . . . . . 125
6.6 Destruction of the Investigational Medicinal Product/Comparator . . . . 126

7. Safety Instructions and Guidance . . . . . . . . . . . . . . . . . . . . . . . . . . . . 126

7.1 Adverse Events and Laboratory Abnormalities . . . . . . . . . . . . . . . . . . 126
7.1.1 Clinical Adverse Events . . . . . . . . . . . . . . . . . . . . . . . . . . . . . . . . . 126
7.1.1.1 Intensity of Clinical Adverse Events . . . . . . . . . . . . . . . . . . . . . . . 127
7.1.1.2 Drug-Adverse Event Relationship . . . . . . . . . . . . . . . . . . . . . . . . 127
7.1.1.3 Serious Adverse Events (Immediately Reportable to Sponsor) . . . 127
7.1.2 Treatment and Follow-up of Adverse Events . . . . . . . . . . . . . . . . . . 128
7.1.3 Laboratory Test Abnormalities . . . . . . . . . . . . . . . . . . . . . . . . . . . . 128
7.1.3.1 Follow-up of Abnormal Laboratory Test Values . . . . . . . . . . . . . . 129
7.2 Handling of Safety Parameters . . . . . . . . . . . . . . . . . . . . . . . . . . . . . 129
7.2.1 Reporting of Adverse Events . . . . . . . . . . . . . . . . . . . . . . . . . . . . . 129
7.2.2 Reporting of Serious Adverse Events . . . . . . . . . . . . . . . . . . . . . . . 129
7.2.2.1 Immediate Reporting to the Sponsor . . . . . . . . . . . . . . . . . . . . . . 129
7.2.2.2 Emergency Medical Contacts . . . . . . . . . . . . . . . . . . . . . . . . . . . 130
7.2.2.3 Expedited Reporting to Health Authorities, Investigators, Institutional Review Boards, and Ethics Committees . . . . . . . . . . . . . . 130
7.2.3 Pregnancy and Lactation . . . . . . . . . . . . . . . . . . . . . . . . . . . . . . . . 131
7.3 Warnings and Precautions . . . . . . . . . . . . . . . . . . . . . . . . . . . . . . . . 132

7.3.1 Ocrelizumab . . . . . . . . . . 132
7.3.2 Rebif® . . . . . . . . . . 134
7.3.3 Corticosteroids . . . . . . . . . . 135
7.3.4 Progressive Multifocal Leukoencephalopathy . . . . . . . . . . 135
7.3.4.1 Guidance for Diagnosis of Progressive Multifocal Leukoencephalopathy . . . . . . . . . . 136

8. Statistical Considerations and Analytical Plan . . . . . . . . . . 140

8.1 Study Endpoints . . . . . . . . . . 140
8.1.1 Primary Efficacy Endpoint . . . . . . . . . . 140
8.1.2 Secondary Efficacy Endpoints . . . . . . . . . . 140
8.1.3 Exploratory Efficacy Endpoints . . . . . . . . . . 141
8.1.4 Safety . . . . . . . . . . 142
8.2 Statistical and Analytical Methods . . . . . . . . . . 143
8.2.1 Primary Efficacy Analysis . . . . . . . . . . 143
8.2.2 Secondary Efficacy Analyses . . . . . . . . . . 143
8.2.2.1 The Time to Onset of Confirmed Disability Progression for At Least 12 Weeks During the 96-Week Comparative Treatment Period . 144
8.2.2.2 Total Number of T1 Gadolinium-Enhanced Lesions as Detected by Brain MRI at Weeks 24, 48, and 96 . . . . . . . . . . 145
8.2.2.3 The Total Number of New, and/or Enlarging T2 Hyperintense Lesions as Detected by Brain Magnetic Resonance Imaging at Week 24, Week 48 and Week 96 . . . . . . . . . . 145
8.2.2.4 Proportion of Patients who have Disability Improvement Confirmed for At Least 12 Weeks . . . . . . . . . . 145
8.2.2.5 The Time to Onset of Confirmed Disability Progression for At Least 24 Weeks During the 96-Week Comparative Treatment Period . 146
8.2.2.6 Total Number of T1-Hypo-Intense Lesions (Chronic Black Holes) at Weeks 24, 48, and 96 . . . . . . . . . . 146
8.2.2.7 The Change in Multiple Sclerosis Functional Composite Scale (MSFCS) Score from Baseline to Weeks 96 . . . . . . . . . . 146
8.2.2.8 The Percentage Change in Brain Volume as Detected by Brain Magnetic Resonance Imaging Scan from Week 24 to Week 96 . . . . . . 146
8.2.2.9 Change in Quality of Life, as Measured by the Short Form 36 version 2 Physical Component Summary (PCS) Score from Baseline to Week 96 . . . . . . . . . . 146

8.2.2.10 Proportion of Patients Who Have No Evidence of Disease Activity (NEDA) by Week 96 . . . . . 147
8.2.3 Exploratory Analyses . . . . . 147
8.2.4 Sample Size . . . . . 147
8.2.5 Hypothesis Testing . . . . . 148
8.2.6 Analysis Populations . . . . . 148
8.2.6.1 Safety Population . . . . . 148
8.2.6.2 Intent-to-Treat Population . . . . . 148
8.2.6.3 Per Protocol Population . . . . . 148
8.2.7 Interim Analysis . . . . . 149
8.2.8 Safety Data Analysis . . . . . 149
8.2.9 Safety Follow-up Period . . . . . 149
8.2.10 Open-Label Extension Phase . . . . . 150
8.2.11 Other Analyses . . . . . 150
8.2.11.1 Pharmacokinetic Analysis . . . . . 150
8.2.11.2 Pharmacodynamic Analysis . . . . . 150
8.2.11.3 Roche Clinical Repository / Protein Biomarker Samples . . . . . 150

9. Data Collection, Management and Quality Assurance . . . . . 151
9.1 Assignment of Preferred Terms and Original Terminology . . . . . 151

10. Study Committees . . . . . 151

11. References . . . . . 153

12. Ethical Aspects . . . . . 159
12.1 Local Regulations/Declaration of Helsinki . . . . . 159
12.2 Informed Consent . . . . . 159
12.2.1 Study Informed Consent . . . . . 159
12.2.2 RCR Informed Consent . . . . . 160
12.2.3 Death or Loss of Competence of Participant who has Donated a Specimen(s) that is Stored in the RCR . . . . . 160
12.3 Independent Ethics Committees (IEC) and Institutional Review Board (IRB) . . . . . 161
12.4 Role of the Science and Ethics Advisory Group (SEAG) . . . . . 162

13. Conditions for Modifying the Protocol . . . . . 162

14. Conditions for Terminating the Study . . . . . . . . . . . . . . . . . . . . . . . . . . . . . 162

15. Study Documentation, CRFs and Record Keeping . . . . . . . . . . . . . . . . . . . 162

15.1 Investigator's Files / Retention of Documents . . . . . . . . . . . . . . . . . . . 162

15.2 Source Documents and Background Data . . . . . . . . . . . . . . . . . . . . . . 163

15.3 Audits and Inspections. . . . . . . . . . . . . . . . . . . . . . . . . . . . . . . . . . . . 163

15.4 Electronic Case Report Forms . . . . . . . . . . . . . . . . . . . . . . . . . . . . . 163

15.5 Financial Disclosure . . . . . . . . . . . . . . . . . . . . . . . . . . . . . . . . . . . . 164

16. Monitoring the Study . . . . . . . . . . . . . . . . . . . . . . . . . . . . . . . . . . . . . . . . 164

17. Confidentiality of Trial Documents and Subject Records . . . . . . . . . . . . . 164

18. Clinical Study Report (CSR) . . . . . . . . . . . . . . . . . . . . . . . . . . . . . . . . . . 165

19. Publication of Data and Protection of Trade Secrets . . . . . . . . . . . . . . . . . 165

## List of Tables


Table 1: Summary of the Most Frequent Rebif® Adverse Reactions by MedDRA System Organ Class . . . 61

Table 2: Overview of Dosing Regimen in the Double-Blind, Double-Dummy Treatment Period. . . . 69

Table 3: Overview of Dosing Regimen in the OLE Phase Screening Period and the OLE Phase . . . 70

Table 6: Schedule of Assessments: Open Label Extension Phase . . . 93

Table 7: Treatment Groups and Schedule of Study Medication During the Double-Blind, Double-Dummy Treatment Period . . . 115

Table 8: Infusions of Ocrelizumab 300 mg . . . 117

Table 9: Subsequent Infusions of Ocrelizumab 600 mg. . . . 117

Table 10: Overview of Rebif® Dosing Regimen . . . 121

Table 11: Clinical Features to Distinguish between MS Relapse and PML*. . . 139

Table 12: MRI Lesion Characteristics Typical of PML and MS . . . 139

## List of Figures

Figure 1: Overview of Study Design . . . . . . . . . . . . . . . . . . . . . . . . . . . . . . . . . . 65

Figure 2: Safety Follow-Up - Variable B-cell Monitoring . . . . . . . . . . . . . . . . . 68

Figure 3: Diagnostic Algorithm for PML . . . . . . . . . . . . . . . . . . . . . . . . . . . . . 138

## List of Appendices


Appendix 1: AEs Categories for Determining Relationship to Test Drug . . . . . 167

Appendix 2: ICH Guidelines for Clinical Safety Data Management, Definitions and Standards for Expedited Reporting, Topic E2 . 168

Appendix 3: Common Terminology Criteria (CTCAE). . . . . . . . . . . . . . . . . . . 170

Appendix 4: Telephone Interviews. . . . . . . . . . . . . . . . . . . . . . . . . . . . . . . . . . . 171

Appendix 5: Modified Fatigue Impact Scale (MFIS). . . . . . . . . . . . . . . . . . . . . 174

Appendix 6: The Center for Epidemiologic Studies Depression Scale (CES-D) . . . . . . . . . . . . . . . . . . . . . . . . . . . . . . . . . . . . . . . . . . . 177

Appendix 7: The Short Form (SF-36) Health Survey . . . . . . . . . . . . . . . . . . . . 179

Appendix 8: Optical Coherence Tomography Exploratory Substudy . . . . . . . . 185

# GLOSSARY OF ABBREVIATIONS

| | |
|---|---|
| *ADA* | *Anti-drug antibody (also known as Human Anti-human Antibody [HAHA])* |
| ADCC | Antibody dependent cellular cytotoxicity |
| AE | Adverse Event |
| AIDS | Acquired Immune Deficiency Syndrome |
| ALT (SGPT) | Alanine aminotransferase |
| ALP | Alkaline Phosphatase |
| ARR | Annualized Relapse Rate |
| AST (SGOT) | Aspartate aminotransferase |
| AUC | Area Under the Curve |
| BAFF | B-cell activating factor |
| BCG | Bacille Calmette-Guérin – TB vaccine |
| | (Fr.  Bacille billié de Calmette-Guérin) |
| β hCG | Beta human Chorionic Gonadotropin |
| CD | Cluster of Differentiation |
| CDC | Complement-dependent cytotoxicity |
| CES-D | Center for Epidemiologic Studies Depression Scale |
| CFH | Complement Factor H |
| CSF | Cerebrospinal Fluid |
| C-SSRS | Columbia - Suicide Severity Rating Scale |
| CTCAE | Common Terminology Criteria for Adverse Events |
| DMARD | Disease-modifying anti-rheumatic drug |
| DMC | Data Monitoring Committee |
| DMT | Disease-modifying therapy |
| DNA | Deoxyribonucleic Acid |
| EBV | Epstein-Barr Virus |
| ECG | Electrocardiogram |
| eCRF | Electronic Case Report Form(s) |

## GLOSSARY OF ABBREVIATIONS

| | |
|---|---|
| EDC | Electronic Data Capture |
| EDSS | Expanded Disability Status Scale |
| EEG | Electroencephalogram |
| eform | Electronic form |
| ELISA | Enzyme-Linked Immunosorbent Assay |
| EMA | European Medicines Agency |
| EQ-5D | EuroQoL |
| ESF | Eligibility Screening Form |
| ETDRS | Early Treatment Diabetic Retinopathy Study |
| FACS | Fluorescence-activated Cell Sorting |
| FDA | Food and Drug Administration |
| FLAIR | Fluid-attenuated Inversion Recovery |
| FSH | Follicle Stimulating Hormone |
| FSS | Functional Systems Scores |
| Gd | Gadolinium |
| GGT | Gamma Glutamyl Transferase |
| HAHA | Human Anti-human Antibody *(also known as anti-drug antibody [ADA])* |
| HAM | Human T-lymphotropic virus (HTLV)-1 Associated Myelopathy |
| HBsAg | Hepatitis B Surface Antigen |
| HBcAb | Hepatitis B Core Antibody |
| HepCAb | Hepatitis C Antibody |
| HDHF | High Dose High Frequency |
| HIV | Human Immunodeficiency Virus |
| HTLV | Human T-lymphotropic Virus |
| IB | Investigator Brochure |
| ICH | International Conference on Harmonisation |

## GLOSSARY OF ABBREVIATIONS

| | |
|---|---|
| ICMJE | International Committee of Medical Journal Editors |
| IFN | Interferon |
| Ig | Immunoglobulin |
| i.m. | Intramuscular |
| IMP | Investigational Medicinal Product |
| IND | Investigational New Drug |
| INN | International Non-proprietary Name |
| IRB/IEC | Institutional Review Board/Independent Ethics Committee |
| IRR | Infusion Related Reaction |
| ITT | Intent-To-Treat |
| i.v. | Intravenous |
| IxRS | Interactive Voice and Web Response System |
| JCV | JC Virus |
| KLH | Keyhole Limpet Haemocyanin |
| LCVA | Low Contrast Visual Acuity |
| LLN | Lower Limit of Normal |
| MBP | Myelin Basic Protein |
| *MCS* | *Mental Component Summary* |
| MedDRA | Medical Dictionary for Regulatory Activities |
| mEP | Multimodal Evoked Potentials |
| MFIS | Modified Fatigue Impact Scale |
| *MMRM* | *Mixed-Effect Model Repeated Measures* |
| MOG | Myelin Oligodendrocyte Glycoprotein |
| MRI | Magnetic Resonance Imaging |
| MS | Multiple Sclerosis |
| MSFCS | Multiple Sclerosis Functional Composite Scale |
| MTX | Methotrexate |

# GLOSSARY OF ABBREVIATIONS

| | |
|---|---|
| NAb | Neutralizing Antibody |
| NHL | Non Hodgkin Lymphoma |
| NK | Natural killer |
| NYHA | New York Heart Association |
| OCB | Oligoclonal Band |
| OCR | Ocrelizumab |
| OCT | Optical Coherence Tomography |
| OLE | Open-Label Extension |
| PASAT | Paced Auditory Serial Addition Test |
| PCR | Polymerase Chain Reaction |
| *PCS* | *Physical Component Summary* |
| PD | Pharmacodynamics |
| PK | Pharmacokinetics |
| PML | Progressive Multifocal Leukoencephalopathy |
| PP | Per protocol (population) |
| PPMS | Primary Progressive Multiple Sclerosis |
| PRMS | Progressive Relapsing Multiple Sclerosis |
| PRO | Patient-Reported Outcome |
| RA | Rheumatoid Arthritis |
| RBC | Red Blood Cells |
| RCR | Roche Clinical Repository |
| RMS | Relapsing Multiple Sclerosis |
| RNA | Ribonucleic Acid |
| RNFL | Retinal Nerve Fiber Layer |
| ROW | Rest of the World |
| RMS | Relapsing Multiple Sclerosis |
| RNFL | Retinal Nerve Fiber Layer |
| RPR | Rapid Plasma Reagin |

# GLOSSARY OF ABBREVIATIONS

| | |
|---|---|
| RRMS | Relapsing-Remitting Multiple Sclerosis |
| SAE | Serious Adverse Event |
| *SAP* | *Statistical Analysis Plan* |
| s.c. | Subcutaneous |
| SDMT | The Symbol Digit Modalities Test |
| SEAG | Science and Ethics Advisory Group |
| SF-36 | SF-36 Health Survey |
| SMT | Study Management Team |
| SPMS | Secondary Progressive Multiple Sclerosis |
| TNF | Tumor Necrosis Factor |
| sTSH | sensitive Thyroid Stimulating Hormone |
| TB | Tuberculosis |
| $T_{CTL}$ | Cytotoxic Lymphocyte T |
| ULN | Upper Limit of Normal |
| VEP | Visual Evoked Potential |
| WBC | White Blood Cells |

# PART I: STUDY DESIGN AND CONDUCT

## 1. BACKGROUND AND RATIONALE

### 1.1 Background

#### 1.1.1 Multiple Sclerosis

Multiple sclerosis (MS) is an inflammatory and degenerative demyelinating disease of the human central nervous system (CNS). Multiple sclerosis affects around 2.5 million people worldwide: it is one of the most common neurological disorders and causes of disability of young adults, especially in Europe and North America [1]. The condition manifests as neurological deficits referable to damage to the spinal cord, brainstem, optic nerves, cerebellum, and cerebrum. Resulting symptoms may include weakness, pain, visual loss, bowel/bladder dysfunction, and cognitive dysfunction. Diagnosis of MS typically occurs through the application of highly structured diagnostic criteria that rely on clinical observation, neurological examination, brain and spinal cord Magnetic Resonance Imaging (MRI) scans, evoked potentials, and examination of cerebrospinal fluid (CSF) [2, 3].

MS is clinically subcategorized into four phenotypic disease patterns distinguished by the occurrence and timing of relapses relative to disease onset and disability progression [4]. These include relapsing remitting MS (RRMS), primary progressive MS (PPMS), progressive relapsing MS (PRMS); and secondary progressive MS (SPMS).

Approximately 80% of MS patients present with RRMS. If left untreated, the majority of RRMS patients will transition into SPMS (with progressive loss of neurologic function, in the absence of relapses) within 20 years. The term relapsing MS (RMS) applies to those patients either RRMS or SPMS, who continue to suffer relapses. Patients with RMS, whether or not they suffer from neurologic progression in the absence of relapses, have a common, inflammatory pathophysiology and therefore, constitute a common target for treatment.

Currently available first-line therapies for the treatment of either relapsing MS or relapsing-remitting MS include interferon (IFN)-β-1a (Rebif® and Avonex®), IFN-β-1b (Betaferon®/Extavia®) and glatiramer acetate (Copaxone®). The currently approved first-line treatments are only modestly effective in reducing the frequency of relapses and preventing disability in patients with RMS. The magnitude of these disease modifying effects are an approximately 30% relative improvement versus placebo [5]. The first-line disease modifying agents reduce the frequency of new episodes but do not reverse fixed deficits and have questionable effects on long-term *disability* progression [6].

Fingolimod (*Gilenya®*) is an oral modulator of sphingosine-1 phosphate (S1P) receptors, a ubiquitous group of transmembrane receptors involved with cellular growth and differentiation. Fingolimod's immunomodulatory effects are believed to be due to binding to and internalization of the S1P1 receptor on lymphocytes, thereby rendering them insensitive to S1P gradients in lymph and inhibiting egress from lymph nodes and other secondary lymphoid organs. Fingolimod is known to readily cross the blood-brain barrier and there are S1P receptors on glial cells and neurons, however the implications

of any possible direct CNS S1P receptor modulation effects are currently unknown. Fingolimod was shown to reduce the annualized relapse rate (ARR) by approximately 50% versus both placebo and interferon β-1a, intramuscular (i.m.) 30 mcg weekly (Avonex®), in confirmatory Phase III clinical trials. Due to the presence of S1P receptors on many different cell types, the adverse event profile of fingolimod is complex, with potential effects on cardiac, ophthalmic, hepatic and pulmonary function, as well as an increased risk of infection, due to inhibition of lymphocyte trafficking. Fingolimod was approved in 2010 by the US Food and Drug Administration (FDA) for patients with relapsing forms of MS and by the European Medicines Agency (EMA) in 2011 for use in patients who have previously failed first-line disease-modifying therapy (DMT) or who have highly active disease.

Natalizumab (Tysabri®) is a monoclonal antibody directed against alpha-4 beta-1 integrin (VLA-4), an adhesion molecule expressed on activated lymphocytes. Natalizumab binds to VLA-4, inhibiting trafficking of activated lymphocytes into the CNS and other extravascular tissues. Natalizumab was shown to have a 66% relative reduction in ARR versus placebo in a Phase III clinical trial. Natalizumab use is generally limited to RRMS patients who have failed to respond to first-line disease modifying therapy or to highly active RRMS patients due to a risk of Progressive Multifocal Leukoencephalopathy (PML).

Teriflunomide (Aubagio®) is a once-daily oral immunomodulator that inhibits dihydroorotate dehydrogenase, a key mitochondrial enzyme involved in de novo pyrimidine synthesis for deoxyribonucleic acid (DNA) replication, and reduces T-cell and B-cell proliferation and function in response to autoantigens. Teriflunomide has been approved in 2012 for treatment of relapsing forms of MS by the FDA. Two Phase III trials showed a significant reduction in ARR of teriflunomide against placebo [7, 8]. A third Phase III trial, TENERE, showed that teriflunomide was not statistically different than interferon β-1a on risk of treatment failure [9]. Risk of treatment failure was defined as the occurrence of a confirmed relapse or permanent treatment discontinuation for any cause, whichever came first.

Mitoxantrone (Novantrone®), a chemotherapeutic agent, is also approved for treatment of relapsing MS in the United States of America, but is generally reserved for secondary progressive and severe relapsing remitting forms of disease. Other drugs have been used with varying degrees of success, including corticosteroids, methotrexate (MTX), cyclophosphamide, azathioprine, and intravenous (i.v.) immunoglobulin.

Despite significant advances in MS therapy many patients continue to experience disease activity; thus there remains a need to develop more effective and better tolerated therapies for the treatment of RMS.

### 1.1.2 Ocrelizumab

Ocrelizumab is a humanized, glycosylated, monoclonal antibody directed against the cluster of differentiation (CD)20 antigen present on select B-cells. Ocrelizumab binds to the CD20 antigen thereby resulting in B-cell depletion via antibody-dependant cellular cytotoxicity (ADCC), complement-dependent cytotoxicity (CDC) and enhanced apoptosis.

Ocrelizumab was constructed using a recombinant DNA technique. This antibody shares an overlapping epitope on CD20 with rituximab (chimeric monoclonal antibody, Mabthera®/Rituxan®), as determined by direct competition and epitope-mapping experiments. In-vitro, ocrelizumab was shown to be approximately 5 times more potent than rituximab in ADCC activity on a B-cell tumor line over-expressing CD20, approximately 3 times less potent via CDC, and approximately equal in inducing apoptosis in a B-cell lymphoma cell line.

There is substantial proof-of-concept clinical data to support the use of B-cell depleting therapies in patients with relapsing MS. Ocrelizumab shares the same basic mechanism of action as rituximab. In a proof of concept study, rituximab treatment resulted in a robust reduction in MRI based measures of CNS inflammation and clinical benefit versus placebo, in patients with RRMS [10]. WA21493/ACT4422g, a Phase II study of ocrelizumab in RRMS patients provides proof-of-concept support for ocrelizumab efficacy and safety in patients with relapsing remitting MS; please refer to Section 1.1.4.1 for more details.

Ocrelizumab is also known as Ro 496-4913, PRO70769, and rhuMAb 2H7 (refer to the ocrelizumab Investigator's Brochure [IB] for further information).

### 1.1.3 Rationale for Targeting B-cells in MS

Humoral immunity has been implicated in MS for decades, as evidenced by inclusion of CSF oligoclonal bands (OCBs) and increased intrathecal immunoglobulin (Ig)G synthesis in diagnostic criteria for MS [2, 3, 11]. Although, until very recently, the prevailing view of MS pathophysiology held that the CNS inflammation seen in MS is principally mediated by $CD4^+$ proinflammatory (Th1, Th17) T cells, rapidly expanding evidence suggests that B-cells may contribute to MS pathogenesis much more fundamentally than was previously believed, potentially through either antibody-dependent or independent mechanisms [12, 13, 14]. B lymphocytes have been detected within MS lesions and in the CSF of MS patients. Molecular analysis of both lesional and CSF B-cell repertoires reveals dominant, clonally expanded B-cell populations exhibiting somatic hypermutation in the antigen-recognizing CDR3 regions of Ig heavy chains, predominately within the VH4 gene family [15, 16, 17, 18, 19, 20].

Detection of these affinity-matured, clonally expanded repertoires in the CSF but not peripheral blood of MS patients suggests that a localized, antigen driven B-cell response is present in the CNS compartment. CSF clonal B-cell expansion has been reported in patients with both RRMS and PPMS shortly after diagnosis, implying a role for B-cells early in MS pathogenesis rather than as a late response to longstanding tissue damage [21]. More recently, cDNA transcriptomes of clonally expanded affinity-matured B-cells isolated from the CSF of MS patients have been sequence-matched to specific IgG OCBs from the same CSF samples, indicating that this longstanding hallmark of MS diagnosis derives from identifiable B-cell clones present in the CNS compartment [22].

Both antibody-dependent and independent hypotheses for the role of B-cells in MS pathophysiology have been postulated and are currently the subject of intensive research. B-cells may differentiate into plasma cells and produce CNS-directed auto-antibodies,

potentially triggering cellular and complement-dependent cytotoxicity. Although a pathogenic role of anti-myelin antibodies in MS has not been established, they have been detected in the CSF of MS patients [23, 24, 25] and in active MS lesions [26] and remain potential candidates as effectors of myelin sheath damage. B-cells may also function as antigen presenting cells and thereby modulate effector T-cell responses, as they exhibit regulated secretion of both pro-inflammatory and anti-inflammatory cytokines, a function that appears to be abnormal in patients with MS [12]. Finally, B-cells may be a site of latent viral infections such as Epstein Barr Virus (EBV), which may drive CNS autoimmune responses through molecular mimicry or other pro-inflammatory mechanisms [13].

Postmortem pathological studies have identified the presence of ectopic follicular lymphoid structures in the meninges anatomically proximal to sites of grey matter demyelination in a subset of SPMS patients [27, 28, 29]. Similar tertiary lymphoid structures form *de novo* in various tissues of many autoimmune disorders and represent potential *de novo* sites of chronic autoantigenic B-cell activation, maturation and clonal expansion [30]. SPMS patients exhibiting these lymphoid structures have been found to have worse progression rates, when compared to controls without such follicular structures [31] and a pathomechanistic link to grey matter demyelination typical for SPMS has been suggested. Whether or not an anti-CD20 therapeutic antibody can affect the formation or persistence of meningeal lymphoid follicles or the grey matter demyelination prominent in progressive forms of MS is unknown.

In summary, B lymphocytes are believed to contribute to the pathogenesis of all subtypes of MS. Removing select peripheral B-cells from circulation may beneficially disrupt inflammatory processes that potentially involve chronic antigenic stimulation or other regulatory functions promoting chronic autoimmunity. Ocrelizumab specifically depletes CD20+ B-cells, making it a potentially attractive pharmacological agent to test for therapeutic potential in patients with MS.

### 1.1.4 Sponsor Experience with Anti-CD20 Compounds in MS

#### *1.1.4.1 Ocrelizumab in RRMS*

Study WA21493/ACT4422G was a 220-patient Phase II, multicenter, randomized, parallel-group, placebo-controlled, proof-of-concept study to evaluate the safety and efficacy of two dose regimens of ocrelizumab (1000 mg × 2 [administered on Day 1 and Day 15, followed by single infusions of 1000 mg for subsequent cycles] and 300 mg × 2 [administered on Day 1 and Day 15 followed by single infusions of 600 mg for subsequent cycles]), with an additional randomized open label arm of interferon β1-a 30 µg i.m. every week (Avonex®) arm. The primary objective was to evaluate the efficacy of two dose regimens of ocrelizumab compared with placebo, in reducing brain inflammation, as measured by the total number of gadolinium (Gd)-enhancing T1 lesions observed on serial MRI scans of the brain at Weeks 12, 16, 20, and 24. Key secondary objectives were to evaluate the efficacy of both dose regimens of ocrelizumab compared with placebo in reducing ARR at 24 weeks and to evaluate the safety and tolerability of both dose regimens of ocrelizumab in patients with RRMS. Exploratory outcomes included analysis of both dose regimens of ocrelizumab compared to interferon β-1a 30 µg i.m. weekly (Avonex®) along various study measures. Treatment with ocrelizumab

was planned for 72 to 96 weeks total, depending on study arm (patients from both placebo and Avonex group switched to ocrelizumab 300 mg × 2 after Week 24). Additional MRI scans of the brain will be obtained at Weeks 96 and 144 for a subgroup of patients.

Week 24 results demonstrated that both doses of ocrelizumab achieved the primary endpoint by significantly reducing the number of Gd-enhancing lesions compared with placebo ($p < 0.0001$). Both ocrelizumab (OCR) dose groups showed statistically significant reductions in ARR compared with the placebo group (ARR = 0.125 for the OCR 300 mg × 2 group [p = 0.0005] and ARR = 0.169 for the OCR 1000 mg × 2 group [p = 0.0014] compared with ARR = 0.637 for the placebo group, representing a relative reduction (RR) of 80% and 73% in ARR versus placebo group for the low and high OCR groups, respectively. In exploratory analyses, both ocrelizumab groups were superior to the Avonex group for the primary endpoint ($p < 0.0001$) and the 300 mg × 2 group for ARR (ARR = 0.364 for the Avonex group, representing a RR of 66% in ARR with p = 0.03 for the OCR 300 mg × 2 group versus Avonex group and a RR of 53.6% in the ARR with p = 0.086 for the OCR 1000 mg × 2 group versus Avonex group) [32].

Patients from both placebo and Avonex groups switched to ocrelizumab 300 mg × 2 after Week 24. By 48 weeks, the level of benefit of ocrelizumab in reduction of ARR was maintained, where the patients in the ocrelizumab 300 mg × 2 group continued to have a suppressed ARR of 0.086 from Week 24 to 48, and patients switched to ocrelizumab from either placebo or Avonex®, derive a similar degree of efficacy to those randomized to ocrelizumab from onset (ARR for placebo-to-ocrelizumab = 0.161 and for Avonex-to-ocrelizumab = 0.137 after the switch, representing a RR of 74% and 62.4% compared with ARR before the switch, respectively). From Weeks 0 to 72, patients originally randomized to ocrelizumab 300 mg × 2 maintained clinical efficacy with an ARR of 0.186.

The most commonly reported adverse events in ocrelizumab-treated patients were infusion related reactions (IRRs). IRRs were reported during/after the first infusion (Day 1) for 30–43.6% of patients treated with ocrelizumab. Fewer patients (2.1–9.4%) experienced IRRs during/after the second infusion (Day 15). The most common symptoms were rash, pruritus, flushing, tachycardia, headache, pyrexia, and throat irritation. No unanticipated, clinically significant abnormalities in vital signs, electrocardiograms (ECGs), or laboratory parameters were observed in association with ocrelizumab treatment.

On review of the placebo-controlled, double-blinded, 24-week safety data, no imbalance in adverse events (or infection adverse events) or serious adverse events (or infection serious adverse events) between the placebo and active ocrelizumab arms was observed. The rate of adverse events (or infection adverse events) and serious adverse events (or infection serious adverse events) did not increase in ocrelizumab-treated patients at 48 weeks compared with 24 weeks. There is no trend toward an increased risk of adverse events (or infection adverse events) or serious adverse events (or infection serious adverse events) for ocrelizumab-treated patients with previous IFN treatment (Avonex for 6 months).

By the time all patients finished the Week 48 of treatment period, the incidence of infections and serious infections was 92.41/100 PY (95% CI: 76.59, 111.5) and 3.39/100 PY (95% CI: 1.27, 9.04) in patients exposed to low-dose ocrelizumab, including patients who switched from placebo or Avonex®. The incidence of infections and serious infections was 97.38/100 PY (95% CI: 74.76, 126.84) and 5.31/100 PY (95% CI: 1.71, 16.47) in those exposed to the high dose of ocrelizumab. The most common infections in ocrelizumab-treated patients included urinary tract infections, upper respiratory infections, and nasopharyngitis.

To date, in study WA21493, after over 250 patient-years exposure to ocrelizumab, there have been no reports of opportunistic or fatal infections.

One 41-year-old female patient receiving ocrelizumab 1000 mg × 2 died during the Phase II RRMS study at Week 14. This patient was hospitalized with acute onset of encephalopathy and status epilepticus due to systemic inflammatory response syndrome with disseminated intravascular coagulation of unknown cause, following infusion of Gd. The patient's course rapidly progressed to multi-organ failure. While hospitalized, the patient developed nosocomial pneumonia in the setting of severe renal and hepatic insufficiency. After 2 weeks of intensive care, the patient died of transforaminal herniation of the brain, due to massive cerebral edema. Despite a thorough clinical pathological review, the exact cause of death could not be determined. The Investigator assessed the event as related to ocrelizumab.

In a separate trial, another patient receiving ocrelizumab 300 mg x 2 died during the PPMS Phase III study (Study WA25046) 10 days after onset of community-acquired pneumonia. The Investigator reported the event as unrelated to study medication. The initial event appears to be of infectious origin; to determine causal association with ocrelizumab will require assessment of aggregate data. Pneumonia was confirmed at autopsy. It is unclear to what extent ocrelizumab may have contributed to the fatal outcome of the event as there is currently no indication that the patient benefited from any medical attention during the 2 weeks after onset of dyspnea and general weakness until death. The causal relationship to ocrelizumab remains possible as assessed by the Sponsor.

Two other patients died during the Safety Follow-Up Period of the Phase II RRMS study at a time when B cells had recovered. Details regarding these cases are provided in the ocrelizumab IB.

#### *1.1.4.2 Long-Term Results of Phase II Study WA21493/ACT4422G*

To further understand the long-term effect of ocrelizumab therapy, the Phase II Study WA21493/ACT4422G was designed with a 48-week treatment-free period after the 96-week treatment period with ocrelizumab. Across all treatment groups, 86-91% of the 220 initially randomized patients entered the treatment-free period. This included patients who had withdrawn from treatment. Subsequently, 73-82% of patients completed Week 120; 69-80% completed Week 144; and 36-56% entered a 24-week observation drug-free period after B-cell repletion at Week 144. During the treatment-free period, ARR remained at similar low levels as during the treatment period (0.04–0.29 across

treatment groups), with no indication of increase or rebound. The mean number of Gd-enhancing T1 lesions remained at 0 at Week 144 in the ocrelizumab 600 mg group and increased from 0 at Week 96 to 0.3 at Week 144 in the ocrelizumab 1000 mg group. Furthermore, there was no imbalance in the rates of adverse events or serious adverse events across all treatment groups over 144 weeks. No new serious infections and no opportunistic infections were reported since the last ocrelizumab administration. Infection rates did not increase over time: at Week 144, infection rates were 6.5% and 11.1% with the ocrelizumab 600 mg and 1000 mg regimens, respectively. The most common types of infections were upper respiratory tract infections, nasopharyngitis, and urinary tract infections [33].

The long-term efficacy data from the Phase II Study WA21493/ACT4422G show that the low-level of disease activity observed after ocrelizumab treatment is sustained through Week 144. This includes the observation that no Gd-enhancing T1 lesions were observed in the ocrelizumab 600 mg group at Week 144, indicating a CNS anti-inflammatory long-term effect of ocrelizumab. Up to Week 144, no new safety issues were observed with ocrelizumab. In particular, there were no increases in infections and no new serious adverse events, indicating a positive benefit/risk profile. Currently, eligible patients *continue ocrelizumab treatment in* the Open-Label Extension (OLE) Phase of this study.

#### *1.1.4.3 Rituximab in RRMS*

Two clinical trials have been conducted in RRMS patients with rituximab, a chimeric mouse/human monoclonal antibody that shares the same basic mechanism of action as ocrelizumab. Findings briefly highlighted below, offer additional support for the therapeutic potential of the anti-CD20 mechanism in MS.

Study U3264g (HERMES Jr.) was a Phase I, open-label, multicenter study in 26 adults with RRMS to evaluate the safety and tolerability of two treatment cycles of rituximab administered at baseline and after 24 weeks. Re-treatment with rituximab (1000 mg × 2) at 24 weeks was safe and well tolerated, with an observed decrease in relapses and Gd-enhancing lesions through 72 weeks [34].

Study U2787g (HERMES) was a Phase II, proof-of-concept, randomized, double-blind, parallel-group, placebo-controlled, multicenter study to evaluate the safety and efficacy of rituximab in 104 adults with RRMS. The primary objectives were to investigate the efficacy of rituximab compared with placebo, as measured by the total number of Gd-enhancing T1 lesions observed on serial MRI scans of the brain at Weeks 12, 16, 20, and 24, and to evaluate the safety and tolerability of rituximab in patients with RRMS. Secondary objectives were to evaluate additional MRI parameters and the proportion of patients relapsing. The trial met its primary efficacy endpoint and all secondary endpoints. Rituximab was safe and generally well tolerated in this study through 48 weeks though the rate of infusion-associated adverse events, particularly after the first infusion, was higher in rituximab-treated patients (78%) than in placebo patients (40%); corticosteroid premedication was not administered before or at the time of infusion. Study U2787g provides proof of principle that an anti-CD20 therapeutic approach can reduce both MRI and clinical evidence of inflammatory activity in adults with RRMS [10].

#### 1.1.4.4 *Rituximab in PPMS*

A single Phase II/III, randomized, double-blinded, placebo-controlled trial was conducted with rituximab in PPMS. The findings, summarized below, represent the largest and longest duration trial experience to date evaluating the safety and efficacy of anti-CD20 therapy in individuals with multiple sclerosis.

Study U2786g (OLYMPUS) was a Phase II/III randomized, double-blind, parallel-group, placebo-controlled, multicenter study evaluating the safety and efficacy of rituximab in patients with PPMS over a 96-week treatment period consisting of 4 treatment cycles with dual infusions of 1000 mg (2000 mg/cycle). Although the trial did not demonstrate significant primary efficacy on time to confirmed disease progression as measured by Expanded Disability Status Scale (EDSS), a difference was observed with 38.5% of patients in the placebo group experiencing confirmed disease progression versus 30.2% in the rituximab group. Biological activity was evidenced by significantly lower T2 lesion volume accumulation on brain MRI, a secondary efficacy endpoint, in rituximab-treated patients compared with placebo (p=0.0008). Subgroup analyses suggest that PPMS patients with evidence of active disease may have shown significant clinical treatment response as measured by time to confirmed disease progression over a 96-week timeframe. Factors that appeared prognostic for disease progression and potentially predictive of treatment response in the rituximab group included younger age, presence of contrast enhancing lesions at baseline on brain MRI, and higher MS severity score.

Rituximab was generally safe and well tolerated in Study U2786g. The proportions of patients with at least one adverse event (100% placebo versus 99% rituximab) and one serious adverse event (13.6% placebo versus 16.1% rituximab) were comparable between treatment groups. Three adverse events that occurred during the study led to death: one in the rituximab group following recurrent aspiration pneumonias and two in the placebo group due to pneumonia and cardiopulmonary failure. More infusion-associated adverse events were observed in rituximab-treated patients (73.6% versus 40.3% for placebo), particularly after the first infusion, but rates declined in both groups to similar levels upon successive infusions. Patients were not premedicated with glucocorticoids before rituximab infusions in Study U2786g. The vast majorities (92%) of infusion associated events in rituximab treated patients were mild to moderate in severity; no Grade 4 or 5 infusion-associated events were observed. The proportion of patients with at least one infection was comparable between groups (68.2% rituximab versus 65.3% placebo), but a higher proportion of patients with at least one serious infection was observed in the rituximab-treated group (4.5%) compared with placebo (<1%). No opportunistic infections occurred.

Treatment with rituximab was associated with rapid and near-complete depletion of circulating CD19 positive B lymphocytes beginning 2 weeks post-treatment through 96 weeks. Approximately 35% of rituximab-treated patients had recovered peripheral CD19 B-cell counts to 80 cells/μL (laboratory defined lower limit of normal (LLN) in healthy volunteers) within 48 weeks after the last dose. Median circulating CD3 T-lymphocyte counts were not appreciably altered by rituximab. At any time in the trial, IgM levels were below the LLN in 31.7% of rituximab-treated patients and 5.9% of placebo-treated patients. The proportion of patients with IgG and IgA levels below LLN

were not different between groups. The incidence of infectious adverse events and infectious serious adverse events did not appear higher in patients with Ig levels (all isotypes) below LLN in either treatment group compared with patients with Ig levels in the normal range or above upper limit of normal (ULN) [35].

### 1.1.5 Rebif®

The active comparator for this study is Rebif® (interferon β-1a), which has been approved for treatment of RMS.

The efficacy and safety of Rebif® was demonstrated in the PRISMS study (The Prevention of Relapses and Disability by Interferon β-1a Subcutaneously in Multiple Sclerosis) which led to the approval of Rebif® in RMS. This was a multicentre controlled trial of 560 patients with an EDSS score between 1.0 and 5.0 and at least two relapses in the preceding 2 years. Patients were randomized to 2-year treatment with placebo or IFN β-1a (22 or 44 µg subcutaneously three times weekly). Following the 2 years of treatment, both doses of Rebif® showed significant benefits compared with placebo on major efficacy outcome measures. There was a non-significant trend towards greater efficacy with the higher dose on most clinical measures, and a statistically significant dose-effect favoring the higher dose in terms of impact on the number of T2-active lesions. In a subgroup of patients with more severe disease (baseline EDSS >3.5), the 44-µg dose delayed progression of disability significantly better than either the placebo or the 22 µg dose. Neutralizing antibodies were significantly less frequent in the 44 µg group than in the low-dose group [36].

After 2 years, patients who had initially received placebo in the PRISMS study were re-randomized to receive Rebif® (22 or 44 µg subcutaneously three times weekly) and were followed for an additional 2 years. By the end of the 4 year period, patients who had switched from placebo to Rebif® experienced an approximate 50% reduction in ARR compared with the end of year 2. Also, after 4 years, the higher dose approached significance for annual relapse rates (0.8 for 22 µg versus 0.72 for 44 µg; $p=0.069$). The mean ARR was significantly lower in patients who had received Rebif® for the full 4 years compared with those who had received placebo for the first 2 years. During Years 3 and 4, relapse rates were significantly lower for the 44 µg group with relapse rates decreasing progressively with each year of treatment – 0.92, 0.82, 0.57, and 0.44 relapses/year for each year of the study. Patients who received the highest cumulative dose of active therapy had the lowest rate of disability progression. The time to first confirmed EDSS progression was 42.1 months for the 44 µg group compared with 24.2 months for the crossover group. The time to first confirmed progression did not differ significantly between the 22 µg group (35.9 months) and the crossover group [37].

Rebif® showed superiority versus Avonex® in the EVIDENCE trial. This was a randomized, controlled, multicenter trial which compared the efficacy and safety of Rebif® 44 µg subcutaneously three times weekly and Avonex® 30 µg i.m. once weekly in 677 patients with RRMS. The primary endpoint was the proportion of patients who were relapse free at 24 weeks; the principal MRI endpoint was the number of active lesions per patient per scan at 24 weeks. After 24 weeks, 74.9% of patients receiving Rebif® 44 µg three times a week remained relapse free compared with 63.3% of those given Avonex® 30 µg once a week. Patients receiving Rebif® 44 µg three times a week

had fewer active MRI lesions ($p = 0.001$ at 24 and 48 weeks) compared with those receiving Avonex® 30 µg once a week. Injection-site reactions were more frequent with Rebif® 44 µg three times a week (83% versus 28%, $p = 0.001$), and there were asymptomatic abnormalities of liver enzymes (18% versus 9%, $p = 0.002$) and altered leukocyte counts (11% versus 5%, $p = 0.003$) compared with the Avonex® 30 µg once a week dosage. Neutralizing antibodies developed in 25% of Rebif® 44 µg three times a week patients and in 2% of patients receiving Avonex® 30 µg once a week [38].

Rebif® has also been studied in the SPMS population. The SPECTRIMS study was a multicenter, randomized, parallel-group, placebo-controlled study which tested two doses of Rebif® in patients with SPMS. Patients had to have clinically definite SPMS which was defined as progressive deterioration of disability for at least 6 months with an increase of at least 1 EDSS point over the previous 2 years (or 0.5 point between EDSS score of 6.0 and 6.5), with or without relapses, following an initial course of RRMS. Baseline EDSS scores had to be from 3.0 to 6.5 and the pyramidal functional score of at least 2. Patients were randomized to 3-year treatment with placebo or IFN β-1a (22 or 44 µg subcutaneously three times weekly). The primary outcome was time of confirmed progression, defined as increase from baseline by at least 1 EDSS point (or 0.5 point if baseline EDSS ≥ 5.5), confirmed 3 months later with no intervening score lower than the minimum required level. The primary outcome was not significantly influenced by treatment with Rebif® as compared to placebo ($p = 0.146$). A significant benefit was seen on relapse rate for both doses of Rebif®. These findings suggest that treatment with Rebif® has clinical benefit in SPMS, predominantly affecting relapses, but only modest effect on disability [39].

In controlled clinical trials, the most commonly observed adverse reactions were: injection-site reactions, influenza-like symptoms (headache, fatigue, fever, rigors, chest pain, back pain, myalgia), elevated liver enzymes, hematological abnormalities, abdominal pain, and depression. Most of these adverse reactions are unique to treatment with interferon beta, presenting potential difficulties in maintaining blinding in controlled clinical trials. Summary of the most frequent Rebif® adverse reactions by Medical Dictionary for Regulatory Activities (MedDRA) system organ class have been summarized in Table 1.

**Table 1: Summary of the Most Frequent Rebif® Adverse Reactions by MedDRA System Organ Class**

| System Organ Class | Very Common ADR<br>frequency of occurrence<br>≥ 1/10 | Common ADR<br>frequency of occurrence<br>≥ 1/100 to 1/10 |
|---|---|---|
| **General disorders and administration site conditions** | Injection site inflammation, injection site reaction, influenza-like symptoms | Injection site pain, fatigue, rigors, fever |
| **Investigations** | Asymptomatic transaminase increase | Severe elevation of transaminase |
| **Blood and lymphatic system disorders** | Neutropenia, lymphopenia, leucopenia, thrombocytopenia, anaemia | |
| **Psychiatric disorders** | | Depression, insomnia |
| **Nervous system disorders** | Headache | |
| **Gastrointestinal disorders** | | Diarrhoea, vomiting, nausea |
| **Skin and subcutaneous tissue disorders** | | Pruritus, rash, erythematous rash, macula-papular rash |
| **Musculoskeletal and connective tissue disorders** | | Myalgia, arthralgia |

ADR=adverse drug reaction; MedDRA=Medical Dictionary for Regulatory Activities.
Based on Rebif® Summary of Product Characteristic 2010 [40]. Please refer to local label for more details.

Severe liver injury, including some cases of hepatic failure requiring liver transplantation, has been reported rarely in patients taking Rebif®. Treatment with Rebif® should be stopped immediately if jaundice or other symptoms of liver dysfunction appear [40, 41]. Please refer to Section 6.2.2 for further details.

Neutralizing antibodies to IFN β-1a can develop in some patients, usually following the first year of therapy. Long-term consequences of these antibodies are still not known, however, current evidence shows that they may reduce the efficacy of the drug. The antibodies tend to cross-react with different IFN β formulations. For this reason, switching to another IFN β drug is unlikely to be effective [42, 43].

## 1.2 Rationale for the Study

This study is a pivotal Phase III clinical trial, and is composed of the following periods: a double-blind, double-dummy treatment period, a Safety Follow-Up Period, and an Open-Label Extension Phase. The double-blind, double-dummy treatment period is designed to demonstrate the efficacy and safety of ocrelizumab in relapsing MS in comparison to high-dose, high-frequency (HDHF) IFN (Rebif®). The Open-Label Extension Phase

serves to evaluate long-term safety, tolerability, and efficacy of ocrelizumab treatment in patients with relapsing forms of MS.

This study is part of a broader, confirmatory clinical development program investigating the safety and efficacy of ocrelizumab in patients with both primary progressive and relapsing MS. An Open-Label Extension Phase of the Phase II study WA21493/ACT4422G is ongoing for eligible RRMS patients. There are three ongoing Phase III pivotal trials (including the one presented in this protocol), two in RMS and one in PPMS. Please refer to Section 3.1.1 for further details on study design and choice of comparator.

# 2. OBJECTIVES

## 2.1 Primary Objective

The primary objective of this study is to assess whether the efficacy of ocrelizumab 600 mg (given as dual infusions of 300 mg on Days 1 and 15 of the first 24-week treatment cycle and as a single infusion of 600 mg on Day 1 of each 24-week treatment cycle thereafter) intravenously every 24 weeks is superior to Rebif® as measured by the annualized protocol-defined relapse rate (see Section 5.3.2.1 for the definition of protocol-defined relapse) by two years (96 weeks) in patients with relapsing multiple sclerosis.

## 2.2 Secondary Objectives

The *key* secondary objectives of this study are to evaluate whether the efficacy of ocrelizumab is superior to Rebif®, as reflected by the following measures:

- The time to onset of *confirmed* disability progression for at least 12 weeks *with the* initial event of neurological worsening occur*ring* during the 96-week, double-blind, double-dummy, treatment period (see Section 5.3.2.2 for the definition of *confirmed* disability progression).
- *The total number of T1 Gd-enhancing lesions as detected by brain MRI at Weeks 24, 48, and 96*
- The total number of new, and/or enlarging T2 hyperintense lesions as detected by brain MRI at Weeks 24, 48, and 96.
- *The proportion of patients who have confirmed disability improvement for at least 12 weeks with the initial event of neurological improvement occurring during the 96-week double-blind, double-dummy treatment period.*
- The time to onset of *confirmed* disability progression for at least 24 weeks, *with the* initial event of neurological worsening occur*ring* during the 96-week, double-blind, double-dummy, treatment period (see Section 5.3.2.2 for the definition of *confirmed* disability progression).
- *The total number of T1-hypo-intense lesions (Chronic Black Holes) at Weeks 24, 48, and 96*
- The change in Multiple Sclerosis Functional Composite Scale (MSFCS) score from baseline to Week 96.
- The *percentage* change in brain volume as detected by brain MRI from Week 24 to Week 96.

- *The change in SF-36 Physical Component Summary (PCS) Score from baseline to Week 96*
- *The proportion of patients who have no evidence of disease activity (NEDA) by Week 96*

**Safety:**

To evaluate the safety and tolerability of ocrelizumab 600 mg (given as dual infusions of 300 mg on Days 1 and 15 of the first 24-week treatment cycle and as a single infusion of 600 mg on Day 1 of each 24-week treatment cycle thereafter) intravenously every 24 weeks in patients with relapsing MS (including exploratory, long-term safety and tolerability in those patients entering the OLE Phase).

**Pharmacokinetics/Pharmacodynamics:**

To explore the pharmacokinetics (PK), immunogenicity and pharmacodynamics (PD) of ocrelizumab in patients with relapsing MS.

## 2.3 Exploratory Objectives

- The change in low contrast visual acuity from baseline to Weeks 48 and 96.
- The change in the Symbol Digit Modalities Test from baseline to Weeks 48 and 96.
- *The proportion of relapse free patients by Week 96.*
- *The change in total T2 hyperintense lesion volume as detected by brain MRI from baseline to Week 96.*
- The annualized relapse rate, based on *all* clinical relapses at the end of the 96-week comparative treatment period *(protocol-defined relapses are a subset of all clinical relapses).*
- *The ARR of relapses requiring IV steroid therapy.*
- *The ARR of severe relapses.*
- The *percentage* change in brain volume as detected by brain MRI from baseline to Week 96.
- The change in Multiple Sclerosis Functional Composite Scale (MSFCS) score from baseline to Week 48.
- The cumulative change in EDSS scores, measured in area under the curve (AUC) by Week 96.
- The change in EDSS from baseline to Week 96.
- The change in timed 25-foot walk from baseline to Week 96.
- The change in 9-hole peg test from baseline to Week 96.
- The change in paced auditory serial addition test (PASAT) from baseline to Weeks 48 and 96.
- The time to onset of sustained 20% increase in 9-hole peg test for at least 12 weeks.
- The time to onset of sustained 20% increase in timed 25 foot walk for at least 12 weeks.
- *The change in fatigue, as measured by the Modified Fatigue Impact Scale (MFIS) total score from baseline to Week 96.*

- *The change in patient-reported depressive symptoms, as measured by the Center for Epidemiologic Studies Depression Scale (CES-D), from baseline to Week 96.*
- *Analyses of EQ-5D, collected at baseline, Week 48, and Week 96.*
- The change in Karnofsky Performance Status Scale from baseline to Week 96.
- *The percentage change in cortical grey matter volume from baseline to Week 96.*
- *The percentage change in white matter volume from baseline to Week 96.*
- *The proportion of patients who have confirmed disability improvement sustained for at least 24 weeks, with the initial event of neurological improvement occurring during the 96-week double-blind double-dummy treatment period.*
- *The proportion of patients who have disability improvement sustained for at least 12 weeks and sustained until the end of the 96-week, double-blind, double-dummy treatment period, with the initial event of neurological improvement occurring during the 96-week, double-blind, double-dummy treatment period.*
- *The duration of the confirmed disability improvement.*
- *The proportion of patients who, at Week 96, have improved, stable, or worsened disability, compared to baseline.*
- *The change in Quality of Life, as measured by the Short Form 36 version 2 Mental Component Summary (MCS) Score from baseline to Week 96.*
- To evaluate the long-term safety, tolerability, and efficacy of ocrelizumab in patients with relapsing form of MS who are enrolled in the OLE Phase.

## 2.4 Roche Clinical Repository Exploratory Objectives

The Roche Clinical Repository (RCR) is a centrally administered facility for the long-term storage of human biological specimens including body fluids, solid tissues and derivatives thereof (e.g., DNA, ribonucleic acid [RNA] proteins/ peptides). Specimens stored in the RCR will be used to:

- Study the association of biomarkers with efficacy and/ or adverse events associated with ocrelizumab.
- Increase the knowledge and the understanding of biology of MS and mode of action of ocrelizumab.

## 2.5 Optional Exploratory Substudies

Consenting patients who enrolled in the main study WA21092 and who are eligible will be offered the opportunity to participate in optional substudies. Please refer to Section 5.9 for details.

### 2.5.1 Optical Coherence Tomography Exploratory Substudy

This substudy will be conducted at certain selected centers and will be used to evaluate the neuroprotective effect of ocrelizumab as measured by retinal nerve fiber layer (RNFL) thickness and macular volume in both eyes (see Section 5.9.1 or Appendix 8).

# 3. STUDY DESIGN

## 3.1 Overview of Study Design and Dosing Regimen

### Figure 1: Overview of Study Design

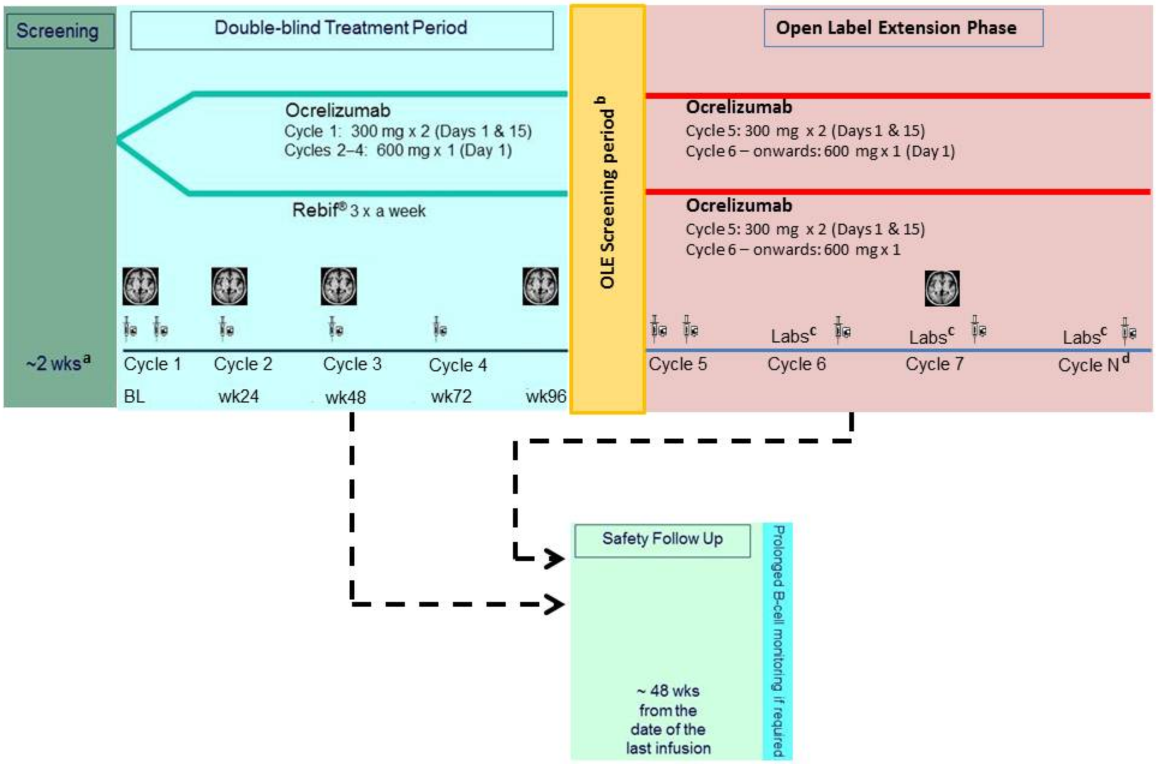


a.Screening for the study may be prolonged for up to 8 weeks for relevant clinical, administrative, or operational reasons.
b. The OLE Phase Screening Period will start after all assessments at the Week 96 Visit have been done. It will last up to 4 weeks. It is possible that for particular reasons, the OLE Phase Screening Period could be longer than 4 weeks. If a prolongation of the OLE Phase Screening Period is needed, it should be discussed with the Sponsor on a case-by-case basis. Note that during the OLE Phase Screening Period, patients should receive Rebif®/Rebif® placebo until the first infusion of Cycle 5.
c. In order to verify if patients meet re-treatment criteria, patients in the Open-Label Extension Phase of the study should come to the clinic approximately 2 weeks prior to infusions of Cycle 6, 7, etc.
d. The OLE Phase of the study can be terminated at any time (please refer to Section 3.1.4 and Section 5.10). Cycle N represents a typical cycle that occurs every 24 weeks.

The study will consist of the following periods:

**Screening:**

Consenting patients will enter a Screening period to be evaluated for eligibility. The Screening period will last approximately 2 weeks, but it may be prolonged for up to 8 weeks for relevant clinical, administrative, or operational reasons. Procedures at screening will include collecting medical history, medical examination including thorough neurological exam, EDSS score, ECG, blood and urine sampling. Please see Table 4 - "Schedule of Assessments: Screening Through the End of Double-Blind, Double-Dummy Treatment Period" for further details.

*Please note* that based on local Ethics Committees or National Competent Authority requirements, additional diagnostic testing may be required for selected patients or selected centers to exclude tuberculosis, Lyme disease, HTLV-1 associated myelopathy (HAM), acquired immune deficiency syndrome (AIDS), hereditary disorders, connective tissue disorders, or sarcoidosis.

**Treatment Period:**

**Double-blind, double-dummy, comparative treatment period**

Eligible patients will be randomized via an Interactive Voice and Web Response System (IxRS) into one of two treatment groups: ocrelizumab 600 mg regimen (group A) or interferon β-1a - Rebif® (group B) – please refer to Table 7 and Table 10 for more details.

During the double-blind, double-dummy comparative treatment period, patients will be assessed at clinical visits as per the Schedule of Assessments: Screening Through the End of Double-Blind, Double-Dummy Treatment Period – please refer to Table 4 for further details.

Prior to the next cycle of study drug, patients will be evaluated for pre-specified conditions and laboratory abnormalities to allow for re-treatment (please refer to Section 6.1.4 for more details).

Patients who discontinue from study medication within the 96-week, double-blind, double-dummy, comparative phase (treatment period) of the study will enter the Safety Follow-Up Period (see below); they will not be eligible for the Open-Label Extension Phase.

**Open-Label Extension Phase Screening Period**

Patients who complete the 96-week treatment period may become eligible for the OLE Phase of the study. Patients will be consented for participation in the OLE Phase if, in the opinion of the Investigator, they may benefit from treatment with ocrelizumab. Patients who are not willing to participate in the OLE Phase of the study will be entered into the Safety Follow-Up Period (see below). Patient treatment allocation during the double-blind, double-dummy treatment period should not be unblinded regardless of participation in the OLE Phase.

In the case of a patient who initially declines participation in the OLE Phase and subsequently reconsiders the decision, the patient will have up to 24 weeks after the Week 96 Visit to enter the OLE Phase. In this instance, he or she should not have taken any prohibited medication as specified in Section 4.5.2.1. Patients who decline participation in the OLE Phase should enter the Safety Follow-Up Period.

Patients who have consented to participate in the OLE Phase will enter an OLE Phase Screening Period to be evaluated for eligibility. The OLE Phase Screening Period will start after all assessments at the Week 96 Visit have been performed. This screening period will last up to 4 weeks. It is possible that the OLE Phase Screening Period could be longer than 4 weeks. If a prolongation of the OLE Phase Screening Period is needed, it should be discussed with the Sponsor on a case-by-case basis.

Information from assessments performed during the Week 96 Visit will be utilized to verify the eligibility of the patient for the OLE Phase of the study. Please refer to Section 4.4.

During the OLE Phase Screening Period, all patients should receive Rebif®/Rebif® placebo (depending on initial arm assigned to) until the first infusion of Cycle 5. Please refer to Table 7 and Table 10 for more details regarding the Rebif®/Rebif® placebo regimen.

Patients who withdraw from the OLE Phase Screening Period will be entered into the Safety Follow-Up Period (see below).

**Open-Label Extension Phase**

Duration: The OLE Phase will continue until ocrelizumab is commercially available in the patient's country, or as per local regulation, or should the Sponsor decide to terminate the ocrelizumab program for MS, but will not exceed 4 years after the last patient to reach the Week 96 Visit in the double-blind, double-dummy treatment period.

*In the United Kindgom, the OLE Phase will last for 4 years. The 4-year duration of the OLE Phase serves to evaluate long-term safety, tolerability, and efficacy of ocrelizumab treatment in patients with relapsing forms of MS (please refer to Section 1.2).*

Treatment: During the OLE Phase, all patients will receive the ocrelizumab 600 mg regimen every 24 weeks (please refer to Table 3 and Section 5.10 for more details).

Withdrawal: Patients who withdraw from the OLE Phase will be entered into the Safety Follow-Up Period (see below).

**Safety Follow-Up Period**

Patients who discontinue treatment *prematurely* for any reason during the following periods will be entered into the Safety Follow-Up Period:

- Prior to completion of the 96-week double-blind, double-dummy treatment period;
- During the OLE Phase Screening Period;
- During the OLE Phase;
- Patients who choose not to enter the OLE Phase or are not eligible for the OLE Phase after completing the 96-week, double-blind, double-dummy treatment period.

In the OLE Phase Screening Period, in the case of a patient who initially declines participation in the OLE Phase and subsequently reconsiders the decision, the patient will have up to 24 weeks after the Week 96 Visit to enter the OLE Phase. In this instance, he or she should not have taken any prohibited medication as specified in Section 4.5.2.1.

The Safety Follow-Up Period will last for at least 48 weeks starting from the date of the last infusion of the ocrelizumab/ocrelizumab placebo. Safety Follow-Up visits will be performed at 12-week intervals starting from the date of patient's last visit (Withdrawal from Treatment Visit). However, if after the Safety Follow-Up Period the peripheral

blood B-cells remain depleted, the patient should continue to be monitored at 24-week intervals until the B-cell count has returned to the baseline value or to the lower limit of the normal range (whichever is the lower). Please refer to Figure 2 and to Section 4.5.2.1 for more details.

**Figure 2: Safety Follow-Up - Variable B-cell Monitoring**

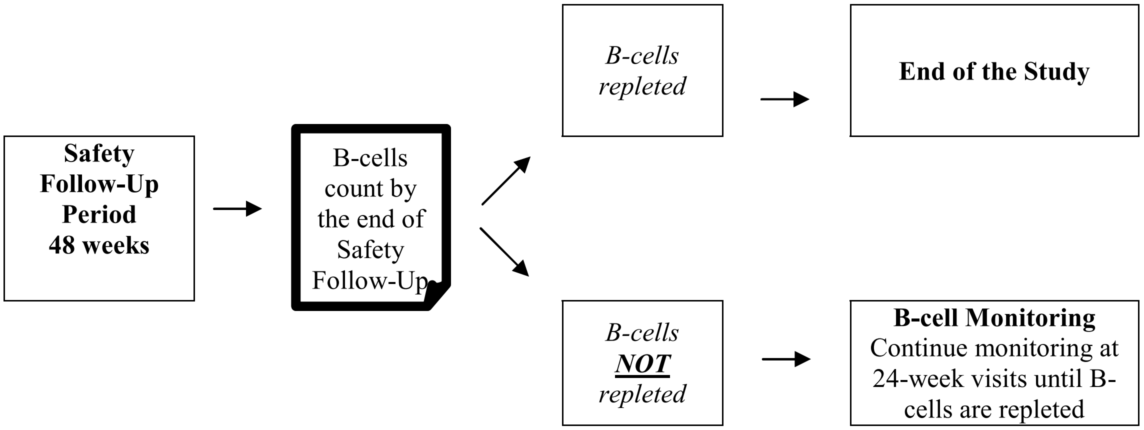


Please note: patients in Safety Follow-up who receive other therapies that may decrease B-cell level will only be followed for 48 weeks from the date of the last infusion of the study drug regardless of their B-cell count.

During Safety Follow-up patients will be assessed at clinical visits every 12 weeks. Telephone interviews will be performed every 4 weeks. If prolonged B-cell monitoring is required, patients will be assessed at clinical visits every 24 weeks and telephone interviews will be performed every 12 weeks. Please refer to Schedule of Assessments: Safety Follow-Up Period (including prolonged B-cell monitoring if required)—Table 5—for further details.

**Every effort should be made to have patients who withdraw from Study Medication complete the Safety Follow-Up Period and all related assessments, <u>regardless of whether or not they receive alternative treatment for MS.</u>**

**Table 2: Overview of Dosing Regimen in the Double-Blind, Double-Dummy Treatment Period**

| Study Medication | Double-Blind, Double-Dummy Treatment Period[1,2] | | | | |
|---|---|---|---|---|---|
| | 1st Cycle[3] (Weeks 1-24) | | 2nd Cycle[3] (Weeks 24-48) | 3rd Cycle[3] (Weeks 48-72) | 4th Cycle[3] (Weeks 72-96) |
| | **Day 1 Infusion** | **Day 15 Infusion** | **Week 24 Infusion** | **Week 48 Infusion** | **Week 72 Infusion** |
| **A Ocrelizumab 600 mg regimen** | **Ocrelizumab** 300 mg i.v. | **Ocrelizumab** 300 mg i.v. | **Ocrelizumab** 600 mg i.v. | **Ocrelizumab** 600 mg i.v. | **Ocrelizumab** 600 mg i.v. |
| **B Rebif® regimen[4]** | **Rebif®** s.c. 3 times per week | → | → | → | → |

i.v.= intravenous; s.c.= subcutaneous.

1. The double-blind, double-dummy treatment period consists of 96 weeks of treatment (4 treatment cycles).
2. Each treatment cycle has a duration of 24 weeks. The first cycle consists of two 300 mg ocrelizumab i.v. infusions separated by 14 days. Cycles 2 – 4 consist of a single i.v. infusion of 600 mg ocrelizumab.
3. Prior to each infusion, a clinical evaluation will be performed to ensure that the patient remains eligible for treatment.
4. Please refer to Table 10 for detailed Rebif® dosing regimen.

Please note: 100 mg of methylprednisolone i.v. will be administered in both treatment arms prior to each infusions of ocrelizumab/ocrelizumab placebo.

**Table 3: Overview of Dosing Regimen in the OLE Phase Screening Period and the OLE Phase**

| Study Medication | OLE Phase Screening Period | OLE Phase[1] | | | | |
|---|---|---|---|---|---|---|
| | | 5th Cycle[2,3] | | 6th Cycle[2,3] | 7th Cycle[2,3] | Nth Cycle[2,3,4] |
| | | Day 1 Infusion | Day 15 Infusion | | | |
| **Ocrelizumab 600 mg regimen** | -.-[5] | **Ocrelizumab** 300 mg i.v. | **Ocrelizumab** 300 mg i.v. | **Ocrelizumab** 600 mg i.v. | **Ocrelizumab** 600 mg i.v. | **Ocrelizumab 600 mg i.v.** |
| **Rebif® regimen** | **Rebif® s.c.** 3 times per week[6] | -.-[7] | -.-[7] | -.-[7] | -.-[7] | -.-[7] |

i.v.=intravenous; OLE=Open-Label Extension; s.c.=subcutaneous.

1. The OLE Phase can terminate at any moment or cycle (please refer to End of Study Section 3.1.4).
2. The assessments requested for N represents the typical schedule of assessments during a cycle.
3. Prior to each infusion, a clinical evaluation will be performed to ensure that the patient remains eligible for treatment.
4. Each treatment cycle has a duration of 24 weeks. The first cycle of the OLE Phase consists of two 300 mg ocrelizumab i.v. infusions separated by 14 days. Cycle 6 onwards consists of a single i.v. infusion of 600 mg ocrelizumab.
5. During the OLE Phase Screening Period there will be no administration of ocrelizumab.
6. Please refer to Table 10 for the detailed Rebif® dosing regimen.
7. During the OLE Phase, there will be no administration of Rebif® verum or placebo.

Please note: 100 mg of methylprednisolone i.v. will be administered in both treatment arms prior to each infusion of ocrelizumab/ocrelizumab placebo.

### 3.1.1 Rationale for Study Design

**Rationale for the use of an active comparator**

There is consensus in the MS community, that the use of placebo in Phase III studies of patients with RMS is (except in exceptional circumstances) ethically indefensible, due to the availability of established, effective therapies [44].

**Rationale for choice of active comparator**

Interferon β-1a 44 μg s.c. 3 times weekly (Rebif®) has been chosen as the active comparator for the ocrelizumab RMS, Phase III clinical program, based on its consistent evidence of efficacy on reducing MRI activity, relapses, and *disability* progression in patients with relapsing forms of MS (see Section 1.1.5).

**Rationale for double-blind, double-dummy study design**

The use of a double-blind, double-dummy study design minimizes the potential for bias and safeguards the integrity of the clinical data generated from this study. It is acknowledged that this approach increases patient's burden. However, this design reduces the risk of concluding that superiority to the active comparator was driven by patient and assessor bias. Regulatory agencies have mandated this study design be implemented throughout the Phase III clinical program. For additional measures intended to minimize bias, please see below.

**Rationale for choice of study population**

This study plans to enroll RMS patients with an EDSS score of 0 to 5.5 at screening who had two documented clinical attacks within the previous 2 years or one clinical attack that occurred within the last year prior to screening. These criteria have been implemented to further characterize the benefits of treatment with ocrelizumab in a wide range of RMS patients with varying degrees of disease activity and severity.

The age range will be limited to ≤ 55 years to avoid confounding by neurological conditions prevalent in older individuals, including but not limited to microvascular disease.

Exclusion of patients who have failed or cannot tolerate Rebif® prevents these patients from being randomized to further Rebif® therapy and reduces the potential for unbalanced dropout rates. Additional exclusion criteria, relating to concomitant diseases, laboratory parameters, and previous medications help to ensure patient safety in the study – please refer to Section 4.3 for more details.

**Rationale for study endpoints**

The proposed study endpoints are widely accepted as clinically relevant and have been used in numerous pivotal clinical trials, in relapsing MS. The primary endpoint for the study will be ARR over 96 weeks, based on protocol-defined relapses. Key secondary endpoints will include the time to onset of *confirmed* disability progression, confirmed at scheduled clinic visits, for at least 12 weeks. *Confirmed disability progression at 24 weeks will also be examined.* Prevention of relapses as well as the prevention or delay of

accumulation of sustained neurological disability are meaningful goals in the treatment of patients with MS.

**Rationale for the duration of the double-blind, double-dummy treatment period**

The 96-week, double-blind, double-dummy treatment duration has been chosen to allow for the assessment of clinical efficacy and safety over 2 years of treatment, consistent with current regulatory guidelines.

**Rationale for the Open-Label Extension Phase**

Multiple sclerosis is a chronic disease that requires lifelong treatment in order to reduce frequency of relapse and accumulation of disability. Once a patient is diagnosed with definite MS, accepted treatment guidelines propose that he/she be offered lifelong treatment, as there is no evidence to suggest that MS spontaneously remits [45].

Results of long-term, follow-up exploratory studies suggest that exposure to DMT for more than 2 years improves outcomes by delaying the time to *disability* progression. Furthermore, there is accumulating evidence that, in MS, inflammatory damage is a continuous process leading to demyelination and axonal transection, and is the substrate of permanent disability in MS [46, 47, 48].

Based on the long-term efficacy and safety data of the Phase II study WA21493/ACT4422G (for details, please refer to Section 1.1.4.1), it is justified to offer ocrelizumab to patients who would otherwise receive treatment that, in the majority of cases, is modestly effective in reducing the frequency of relapses and in preventing sustained disability [33, 49].

Thus, patients who complete the 96-week, double-blind, double-dummy treatment period will be offered participation in an OLE Phase of the study. Providing patients with the opportunity to prolong treatment with ocrelizumab beyond 2 years will provide more information on the long-term safety of ocrelizumab in RMS, (e.g., the risk of infections/serious infections/opportunistic infections or potential loss of previously acquired immunity [e.g., hypogammaglobulinemia, specific serological titers]), as well as further collection of tolerability and efficacy information from patients with long-term exposure. Furthermore, the OLE Phase will increase the overall number of patients exposed and patient-year exposure, thus increasing the likelihood of detecting rare events prior to launch, and understanding the safety/efficacy profile. Analyzing the long-term safety, tolerability, and efficacy of ocrelizumab is of critical importance to eventually help clinicians make informed decisions on therapy for patients [50].

**Rationale for the Safety Follow-Up Period (including Prolonged B-cell Monitoring)**

Data collected during this period will allow evaluation of B-cell repletion after stopping anti-CD20 treatment and collection of safety and efficacy data to document maintenance of the effect and/or the potential for a withdrawal effect. Based on results obtained from the Phase II Study WA21493 [10] with ocrelizumab up to 80% of the ocrelizumab-treated patients are anticipated to enter the B-cell monitoring portion of the Safety Follow-Up Period with targeted assessment every 24 weeks until their B-cell counts recover.

**Rationale for the use of methylprednisolone**

In order to mitigate the risk and severity of infusion-related reactions, 100 mg of i.v. methylprednisolone will be administered to all patients, approximately 30 minutes prior to administration of ocrelizumab/ocrelizumab placebo. In order to mitigate the risk, that even a low dose of methylprednisolone may have a small effect on the efficacy outcomes of the study, methylprednisolone will be administered to patients in both treatment groups. It is of note that the dose of methylprednisolone used for premedication will be up to 50 times smaller than that used for the symptomatic treatment of relapses in MS. In the event that methylprednisolone is contraindicated for the patient, use of an equivalent dose of an alternative steroid (e.g., dexamethasone) should be used as premedication prior to the infusion.

**Additional measures to mitigate bias**

The use of HDHF IFN β, as the active comparator for this study during the double-blind, double-dummy treatment period, presents some difficulties for maintaining patient and physician blinding – please see Section 1.1.5.

To prevent potential unblinding of the assigned arm in the double-blind, double-dummy treatment period as a result of adverse events or changes to laboratory results, the following, additional measures have been implemented:

– **The Examining Investigator/EDSS assessor** will perform the neurological examination, document the Kurtzke Functional Systems (KFS) scores and assess EDSS scores. During the double-blind, double-dummy treatment period, the Examining Investigator/EDSS assessor and their qualified designees (if applicable) will not be involved with any aspect of medical management of the patient and will not have access to patient data. Every effort will be made to ensure that there is no change in the Examining Investigator/EDSS assessor throughout the course of the study for any individual patient. The Examining Investigator/EDSS assessors will be trained and instructed not to discuss what adverse effects (if any) the patient is experiencing from their medication. Examining Investigator/EDSS assessors will receive training in performing EDSS assessments prior to the beginning of the study and must have successfully passed an examination on performance of the Neurostatus EDSS examination within 24 months of participation. All examining physician/EDSS assessors will receive ongoing training on performance of the Neurostatus EDSS examination throughout the course of the study.

– **Patient education:** During the double-blind, double-dummy treatment period, prior to being examined by the Examining Investigator/EDSS assessor, patients will be instructed not to discuss what (if any) adverse effects they may be experiencing. Treating physicians and/or study coordinators should remind patients of these instructions prior to EDSS assessments and this should be documented in the source documents.

– **Blinded, central MRI assessments:** During the double-blind, double-dummy treatment period, a blinded, central MRI reader will assess all on-study MRI scans.

These assessments will provide independent confirmation of the relative changes in immune-mediated, CNS damage.

– **Blinding of laboratory parameters:** Laboratory parameters that may lead to unblinding to treatment assignment, such as fluorescence-activated cell sorting (FACS) cell counts including $CD19^+$ cells, lymphocyte count, IgM and IgG levels, and type I interferon neutralizing antibody levels will be blinded in all patients, except those meeting unblinding criteria for safety reasons. These laboratory parameters will remain blinded during the double-blind, double-dummy treatment period, Safety Follow-Up Period, OLE Phase Screening Period, and during the first cycle of the OLE Phase (Cycle 5).

### 3.1.2 Rationale for Dose Selection

The dose for the ocrelizumab Phase III clinical program was chosen to bring the MS community significant improvement in clinical efficacy versus current standard of care, with acceptable safety. The dose of ocrelizumab in the Phase III clinical program is 600 mg ocrelizumab every 24 weeks (administered as dual infusions of ocrelizumab 300 mg on Days 1 and 15 of the first 24-week treatment cycle, and 600 mg on Day 1 of each 24-week treatment cycle thereafter). This dose has been established as the lowest, maximally effective dose, based on the results from study WA21493/ACT4422g. The safety of this dose has substantial support from the Phase III clinical program in rheumatoid arthritis (RA), an analogous, although systemic autoimmune disease, in a population at greater risk.

ACT2847g was a Phase I/II, dose escalation study in patients with RA, examining 5 dose regimens. In ACT2847g, the two lowest dose groups (receiving less than 200 mg × 2) demonstrated reduced clinical benefits on some endpoints, earlier return of peripheral B-cell counts and higher rates of immunogenicity. In the RA Phase III program, with the exception of patients recruited from Asia, the dose of 200 mg × 2 established a safety profile comparable to placebo. The higher dose of 500 mg × 2 demonstrated apparently superior efficacy, especially in "high hurdle" clinical endpoints and joint preservation, based on X-ray imaging.

In the MS population (study WA21493/ACT4422g), two doses of ocrelizumab were studied, 2000 mg (administered as dual 1000 mg infusions on Days 1 and 15 of the first, 24-week treatment cycle) and 600 mg (administered as dual 300 mg infusions on Days 1 and 15 of the first treatment cycle). Pre-specified primary and secondary efficacy analyses for Study WA21493/ACT4422g indicate that 300 mg × 2 of ocrelizumab is highly effective in suppressing MRI lesion activity and reducing the risk of clinical relapses in RRMS patients over 24 weeks. No difference in efficacy was seen between the ocrelizumab 1000 mg × 2 and 300 mg × 2 doses, on either MRI or clinical endpoints, in the intent-to-treat (ITT) study population. However, exploratory analyses, stratifying groups according to baseline MRI activity, suggest superior efficacy with 1000 mg × 2 versus 300 mg × 2, at 24 weeks, in patients with MRI activity at baseline (≥ 4 enhancing lesions). Similarly, in these patients, the 1000 mg × 2 dose was apparently more effective than the 300 mg × 2 dose at Week 24 and (to a lesser extent) at Week 48, in reducing the absolute number of clinical relapses. Neither the MRI nor the clinical efficacy

differences are statistically significant; however, these results suggest reduction of clinical efficacy at lower doses, in active MS patients. This apparent dose effect was seen despite the fact that linear kinetics (so that complete receptor occupancy can reasonably be assumed) and near complete peripheral CD19 suppression were observed for both doses. Preclinical studies in primates have shown differential susceptibility of tissue resident versus circulating B-cell populations in response to anti-CD20 antibodies [51, 52, 53]. As tissue resident B-cell populations are beyond our ability to measure directly, it is likely that peripheral CD19 count is a sensitive but non-specific PD marker for anti-CD20 efficacy.

**Conclusion**

Based on available data, the dose of 600 mg of ocrelizumab i.v. (given as dual infusions of 300 mg 14 days apart for the first 24 weeks and a single infusion of 600 mg every 24 weeks thereafter) is the most likely dose to be able to demonstrate robust clinical efficacy, an acceptable safety profile and a low risk of immunogenicity, maximizing the likelihood of significant benefit versus standard of care in patients with relapsing MS.

### 3.1.3 End of Double-Blind, Double-Dummy Treatment Period of the Study

The end of the double-blind, double-dummy treatment period of the study is defined as the date at which the last data point during double-blind, double-dummy treatment from the last patient is received, as required for statistical analysis defined in the *Statistical* Analysis Plan (*S*AP).

*In order to maintain integrity of the study data, all study sites and all EDSS assessors will remain blinded to patient treatment allocation until approximately 24 weeks after the Week 96 visit of the last patient randomised, to allow the confirmation of the last 24 week-confirmed disability progression event (see Section 8.2).*

### 3.1.4 End of Study

The end of study is defined as either the last patient last visit of the OLE Phase or the last patient last visit in B-cell monitoring of Safety Follow-Up Period, whichever is later.

The OLE Phase will continue until ocrelizumab is commercially available in the patient's country, or as per local regulation, or should the Sponsor decide to terminate the ocrelizumab program for MS, but will not exceed 4 years after the last patient to reach the Week 96 Visit in the double-blind, double-dummy treatment period.

The B-cell monitoring of the Safety Follow-Up Period of each patient will last until the B-cell count has returned to the baseline value or to the lower limit of the normal range (whichever is lower).

## 3.2 Number of Subjects / Assignment to Treatment Groups

Approximately 800 patients (400 per treatment arm) will be recruited over a planned recruitment period of 16 months.

Patients will be randomized in 2 groups in a 1:1 ratio. An independent IxRS provider will conduct randomization and hold the treatment assignment code. Patients will be stratified by region (US versus ROW) and baseline EDSS (< 4 and ≥ 4).

## 3.3 Centers

This will be a multicenter, international study. It is anticipated that approximately 220 centers worldwide will participate.

# 4. STUDY POPULATION

Under no circumstances are patients who enroll in this study permitted to be re-randomized to this study and enrolled for a second course of treatment.

## 4.1 Overview

Adult patients with relapsing MS who fulfill the eligibility criteria specified in Sections 4.2 and 4.3 are eligible for enrollment into the study. For the eligibility criteria for the OLE Phase, please see Section 4.4.

### 4.1.1 Recruitment Procedures

Sites are encouraged to identify patients for potential recruitment using pre-screening enrollment logs or a pre-ID website.

Patients who are candidates for enrollment into the study will be evaluated for eligibility by the Investigator to ensure they fulfill eligibility criteria (please refer to Sections 4.2 and 4.3).

All patients must sign the informed consent form prior to screening and prior to any changes to their existing medication for the purposes of enrollment into the trial.

No patient may begin treatment prior to randomization and assignment of a medication number. Under no circumstances are patients who enroll in this study permitted to be re-randomized to this study.

The Investigators will be notified by the Sponsor if the study is placed on clinical hold and when the study is completed or closed to further patient enrollment.

No replacement for patients who withdraw from the study after randomization is planned.

## 4.2 Inclusion Criteria

1. Ability to provide written, informed consent and be able to follow the schedule of protocol assessments *.
2. Ages 18-55 years at screening, inclusive.
3. Diagnosis of MS, in accordance with the revised McDonald criteria (2010).
4. At least 2 documented clinical attacks within the last 2 years prior to screening, or one clinical attack in the year prior to screening (but not within 30 days prior to screening).
5. Neurological stability for ≥ 30 days prior to both screening and baseline.
6. EDSS, at screening, from 0 to 5.5 inclusive.
7. Documented MRI of brain with abnormalities consistent with MS prior to screening.

8. Patients of reproductive potential must use reliable means of contraception as described below as a minimum (adherence to local requirements, if more stringent, is required**):
   - For female patients: Two methods of contraception throughout the trial, including the active treatment phase AND for 48 weeks after the last dose of ocrelizumab, or until their B-cells have repleted, whichever is longer.
   - For male patients: Two methods of contraception throughout the trial, including the active treatment phase AND for 24 weeks after the last dose of ocrelizumab. Acceptable methods of contraception include one primary (e.g., systemic hormonal contraception or tubal ligation of the female partner, vasectomy of the male partner) AND one secondary barrier method (e.g., latex condoms, spermicide) OR a double barrier method (e.g., latex condom, intrauterine device, vaginal ring or pessary plus spermicide [e.g., foam, vaginal suppository, gel, cream]).
9. For patients of non reproductive potential (adherence to local requirements, if more stringent, is required**):
   - Women may be enrolled if postmenopausal (i.e., spontaneous amenorrhea for the past year confirmed by a follicle-stimulating hormone [FSH] level greater than 40 mIU/mL, unless the patient is receiving a hormonal therapy for their menopause or surgically sterile (i.e., hysterectomy, complete bilateral oophorectomy);
   - Men may be enrolled if they are surgically sterile (castration).

* Patients who are unable to complete exploratory assessments (e.g., electronic patient reported outcomes [ePROs]) due to physical/disease limitations will not be excluded from the study.

** Based on local Ethics Committees or National Competent Authority feedback, additional requirements to assure contraception or to confirm menopause may be required (e.g., serum estradiol compatible with post-menopause status, longer duration of amenorrhea, higher level of FSH).

## 4.3 Exclusion Criteria

Patients who meet the following criteria must be excluded from study entry:

1. Diagnosis of primary progressive MS.
2. Disease duration of more than 10 years in patients with an EDSS ≤ 2.0 at screening.
3. Inability to complete an MRI (contraindications for MRI include but are not restricted to weight ≥ 140 kg, pacemaker, cochlear implants, presence of foreign substances in the eye, intracranial vascular clips, surgery within 6 weeks of entry into the study, coronary stent implanted within 8 weeks prior to the time of the intended MRI, etc)
   (Patients with contraindication to Gd can be enrolled into the study but cannot receive Gd contrast dyes during their MRI scans.)
4. Known presence of other neurological disorders that may mimic MS, including but not limited to: neuromeylitis optica, Lyme disease, untreated vitamin B12 deficiency, neurosarcoidosis, and cerebrovascular disorders.

**Exclusions Related to General Health**

5. Pregnancy or lactation.

6. Any concomitant disease that may require chronic treatment with systemic corticosteroids or immunosuppressants during the course of the study.
7. History or currently active primary or secondary immunodeficiency.
8. Lack of peripheral venous access.
9. History of severe allergic or anaphylactic reactions to humanized or murine monoclonal antibodies.
10. Significant or uncontrolled somatic disease or any other significant disease that may preclude patient from participating in the study.
11. Congestive heart failure (NYHA III or IV functional severity).
12. Known active bacterial, viral, fungal, mycobacterial infection or other infection, excluding fungal infection of nail beds.
13. Infection requiring hospitalization or treatment with i.v. antibiotics within 4 weeks prior to baseline visit or oral antibiotics within 2 weeks prior to baseline visit.
14. History or known presence of recurrent or chronic infection (e.g., HIV, syphilis, tuberculosis [TB]).
    *Please note: in Germany the following additional exclusion criteria apply:*
    - *Positive anti – HIV I at screening*
    - *Positive anti – HIV II at screening*
    - *Positive QuantiFERON®-TB Gold test at screening*

    *Patients in Germany with an indeterminate result are not eligible for the study unless additional testing demonstrating a negative result is provided. Thus, these patients should have either a tuberculin skin test or have the QuantiFERON® TB Gold test repeated prior to enrollment into the study. If a tuberculin skin test is performed, an induration of ≥ 6 mm is "positive" for a patient with history of BCG vaccine, while an induration of ≥ 10 mm is "positive" for a patient without history of BCG vaccine.*
    *If necessary a QuantiFERON®-TB Gold test might be complemented by additional specific diagnostic tests as per standard procedures in Germany.*
    History of PML
15. History of malignancy, including solid tumors and hematological malignancies, except basal cell carcinoma, *in situ* squamous cell carcinoma of the skin, and *in situ* carcinoma of the cervix of the uterus that have been previously completely excised with documented, clear margins.
16. History of alcohol or drug abuse within 24 weeks prior to baseline.
17. History or laboratory evidence of coagulation disorders.

**Exclusions Related to Medications***

18. Receipt of a live vaccine within 6 weeks prior to the baseline visit.
    In rare cases when patient requires vaccination with a live vaccine, the screening period may be extended but cannot exceed 8 weeks.
19. Treatment with any investigational agent within 24 weeks of screening (Visit 1) or five half-lives of the investigational drug (whichever is longer; or treatment with any experimental procedures for MS [e.g., treatment for chronic cerebrospinal venous insufficiency]).

20. Contraindications to or intolerance of oral or i.v. corticosteroids, according to the country label, including:
    a) Psychosis not yet controlled by a treatment;
    b) Hypersensitivity to any of the constituents.
21. Contraindication to Rebif® or incompatibility with Rebif® use, including:
    a) Current severe depression and/or suicidal ideation;
    b) Hypersensitivity to natural or recombinant interferon-β, or to any excipients;
    c) Previous suboptimal response to HDHF IFN or cessation of HDHF IFN therapy due to poor tolerability;
    d) Prior cessation of Rebif® therapy due to toxicity, which is likely to recur.
22. Treatment with dalfamipridine (Ampyra®) unless on stable dose for ≥ 30 days prior to screening. Wherever possible, patients should remain on stable doses throughout the 96-week treatment period.
23. Previous treatment with B-cell targeted therapies (i.e., rituximab, ocrelizumab, atacicept, belimumab, or ofatumumab).
24. Systemic corticosteroid therapy within 4 weeks prior to screening.**
25. Any previous treatment with alemtuzumab (Campath), anti-CD4, cladribine, mitoxantrone, daclizumab, teriflunomide, laquinimod, total body irradiation, or bone marrow transplantation.
26. Treatment with cyclophosphamide, azathioprine, mycophenolate mofetil (MMF), cyclosporine, MTX, or natalizumab within 24 months prior to screening. NB Patients previously treated with natalizumab will be eligible for this study only if duration of treatment with natalizumab was < 1 year.
27. Treatment with fingolimod (FTY720, Gilenya®) or other S1P receptor modulator (i.e., BAF312), or with BG12, within 24 weeks prior to screening. (NB. Only patients with T lymphocyte count ≥ LLN will be eligible for this study.)
28. Treatment with i.v. immunoglobulin within 12 weeks prior to baseline.

* Patients screened for this study should not be withdrawn from therapies for the sole purpose of meeting eligibility for the trial. Patients who discontinue their current therapy for non-medical reasons should specifically be informed before deciding to enter the study of their treatment options.

** The screening period may be extended (but cannot exceed 8 weeks) for patients who have used systemic corticosteroids for their MS before screening. For a patient to be eligible, systemic corticosteroids should not have been administered also between screening and baseline.

**Exclusions Related to Laboratory Findings***

29. Positive serum β hCG measured at screening.
30. Positive screening tests for hepatitis B (hepatitis B surface antigen [HBsAg] positive, or positive hepatitis B core antibody [total HBcAb] confirmed by a positive viral DNA polymerase chain reaction [PCR]) or hepatitis C (HepCAb).
31. Positive rapid plasma reagin (RPR).
32. CD4 count<300/μL.
33. AST/SGOT or ALT/SGPT ≥ 2.0 × ULN.
34. Platelet count <100,000/μL (<100 × $10^9$/L).
35. Levels of serum IgG 18% below the LLN (for central lab IgG<4.6 g/L).
36. Levels of serum IgM 8% below the LLN (for central lab IgM<0.37 g/L).
37. Total neutrophil count <1.5 × $10^3$/μL.

*Re-testing before baseline: in rare cases in which the screening laboratory samples are rejected by the central laboratory (example: hemolyzed sample) or the results are not assessable (example: indeterminate) or abnormal, the tests need to be repeated within 4 weeks. Any abnormal screening laboratory value that is clinically relevant should be retested in order to rule out any progressive or uncontrolled underlying condition. The last value before randomization must meet study criteria. In such circumstances, the screening period may need to be prolonged but should not exceed 8 weeks.

Please note: based on local Ethics Committees or National Competent Authority requirements, additional diagnostic testing may be required for selected patients or selected centers to exclude tuberculosis, Lyme disease, HAM, AIDS, hereditary disorders, connective tissue disorders, or sarcoidosis. Other specific diagnostic tests may be requested when deemed necessary by the Investigator.

## 4.4 Eligibility Criteria for Open-Label Extension Phase

Patients who meet the following entry criteria may participate in the OLE Phase:

1. Complete the 96-week, double-blind, double-dummy treatment period, and who, in the opinion of the Investigator, may benefit from treatment with ocrelizumab;
2. Are able and willing to provide written informed consent for the OLE Phase (e.g., before the first infusion at Cycle 5) and to comply with the study protocol;
3. Are willing to continue to use at least two contraceptive methods as described in Section 4.2 (items 8 and 9);
4. Meet re-treatment criteria with ocrelizumab (see Section 6.1.4.)

## 4.5 Concomitant Medication and Treatment

### 4.5.1 Definition of Concomitant Treatment

A concomitant medication is any drug or substance taken during the study, including the screening period. Over the counter medications and preventative vaccines received during the study are considered concomitant medications.

A concomitant procedure is any therapeutic or elective intervention (e.g., surgery, biopsy) or diagnostic evaluation (e.g., blood gas measurements, bacterial cultures) performed during the study, including the screening period.

Concomitant medications and procedures will be reported at each visit in the relevant form of electronic Case Report Forms (eCRFs) starting from the baseline visit (including medication and procedures taken between screening and baseline). Medications taken for the treatment of MS in the 2-year period prior to the baseline visit and medications taken for the symptoms of MS in the 3-month period prior to the baseline visit will be recorded at the baseline visit. Additionally, medications and medical/surgical procedures administered for any non-MS condition within 12 months prior to the baseline visit will also be recorded at the baseline visit.

### 4.5.2 Treatment for Symptoms of Multiple Sclerosis

The Treating Investigator should attempt to maintain therapies or treatments for symptoms related to MS (e.g., walking ability, spasticity, incontinence, pain, fatigue) reasonably constant throughout the study. *During the OLE Phase of the study, initiation*

*of therapy with dalfampridine (Ampyra®) is allowed, if indicated by the treating physician.*

**Treatment of relapses:** patients who experience a relapse during the double-blind, double-dummy treatment period, OLE Phase Screening Period, or the OLE Phase may receive treatment with i.v. or oral corticosteroids, if judged to be clinically appropriate by the Investigator. The following standardized treatment regimen may be used as warranted, 1 g i.v. methylprednisolone per day for a maximum of 5 consecutive days. In addition, at the discretion of the Investigator, corticosteroids may be stopped abruptly or tapered over a maximum of 10 days. Such patients should not discontinue the treatment period solely based on the occurrence of a relapse, unless the patient or Investigator feels he or she has met the criteria for withdrawal (See Section 4.6 for further details).

#### *4.5.2.1 Prohibited Concomitant Treatments*

Therapies for MS noted in the exclusion criteria under "Exclusions Related to Medications" (Section 4.3) are not permitted during the double-blind, double-dummy treatment period, OLE Phase Screening Period, or the OLE Phase with the exception of systemic corticosteroids for the treatment of a relapse.

After patients have finished the treatment with ocrelizumab, they may receive alternative treatment for their MS as judged clinically appropriate by the Treating Investigator. However, treatment with immunosuppressants, lymphocyte depleting agents, or lymphocyte trafficking blockers is not allowed while patients remain B-cell depleted due to the potential for increased risk of infection.

### 4.5.3 Immunization

No formal vaccination study has been conducted in ocrelizumab-treated patients. Results from studies done with a similar monoclonal antibody, rituximab, which provide additional information on the impact of anti-CD20 antibodies on the response to vaccinations, are presented below.

A small study comparing responses to influenza vaccination among RA patients receiving rituximab or tumor necrosis factor (TNF) inhibitors versus normal controls found significantly lower post vaccination titres and protection rates (the proportion of a group with a titre $\geq 40$) in rituximab-treated patients compared to both control groups [54]. Results from another study are in line with the ones previously stated. This study showed that RA patients treated with rituximab compared with RA patients receiving MTX and healthy adults had a severely hampered humoral immune response to influenza vaccine. This response remained reduced 6-10 months after rituximab treatment. In the rituximab group, patients who had been previously vaccinated achieved higher anti-influenza titers following influenza vaccination [55].

Another study assessed vaccine responses to influenza vaccine (containing 15 µg hemagglutinin/dose of B/Shanghai/361/02 (SHAN), A/New Caledonia 20/99 (NC) (H1N1) and A/California/7/04 (CAL) (H3N2)) among RA patients treated with disease-modifying anti-rheumatic drugs (DMARDs) with or without rituximab and normal control. After 4 weeks post vaccination geometric mean titers increased for New Caledonia and California antigens in all subjects, but not for the Shanghai antigen in

the rituximab group. In addition, in rituximab treated patients, the percentage of responders was low for all three antigens tested, achieving statistical significance for California antigen [56].

In a randomized study with rituximab, patients with RA had comparable responses to tetanus recall antigen (39% versus 42%), reduced responses to pneumococcal polysaccharide vaccine (43% versus 82% to at least two pneumococcal antibody serotypes) and to Keyhole Limpet Haemocyanin (KLH) neoantigen (47% versus 93%), when given 6 months after rituximab as compared with patients only receiving MTX [57]. Because of the mechanism of action, it is expected that similar findings would apply to ocrelizumab and patients treated with ocrelizumab may experience lower response rates to non-live vaccines than the general population.

Physicians are advised to review the immunization status of patients being considered for treatment with ocrelizumab and follow local/national guidance for adult vaccination against infectious disease. Known dates of immunizations will be recorded on specific eCRF pages. **Immunizations should be completed at least 6 weeks prior to first administration of ocrelizumab.**

Patients requiring *de novo* hepatitis B vaccination (which involves three separate doses of vaccine) should also have completed the course at least 6 weeks prior to the first infusion of study drug.

The safety of immunization with live viral vaccines following ocrelizumab or rituximab therapy has not been studied. Immunization with any live or live-attenuated vaccine (i.e., measles, mumps, rubella, oral polio vaccine, Bacille Calmette-Guerin (BCG), typhoid, yellow fever, vaccinia, cold adapted live influenza strain vaccine, or any other vaccines not yet licensed but belonging to this category) is not recommended within 6 weeks of first dosing (see exclusion criteria, Section 4.3), during ocrelizumab treatment and for as long as the patient is B-cell depleted.

## 4.6 Criteria for Premature Withdrawal

Patients have the right to withdraw from the study at any time for any reason.

**Patients must be withdrawn from treatment (regardless of whether they are in the double-blind, double-dummy treatment period or in the OLE Phase) under the following circumstances:**

- Life threatening (Common Terminology Criteria for Adverse Events [CTCAE] Grade 4) infusion-related event that occurred during a previous ocrelizumab infusion;
- Ongoing pregnancy; Please note that the pregnancy should be followed up to determine outcome, including spontaneous or voluntary termination, details of birth, and the presence or absence of any birth defects, congenital abnormalities, or maternal and newborn complications.
- Patients who demonstrate active hepatitis B or C infection, either new onset or reactivation in the case of hepatitis B;
- Patients who demonstrate active TB, either new onset or reactivation;
- Patients with PML;

– Patients with elevation of ALT ≥ 10 × ULN, jaundice, or other clinical symptoms of liver dysfunction (please refer to Section 6.2.2 for more details);*

– Patients with persisting elevation of ALT > 3 × ULN, or other clinical symptoms of liver dysfunction that did not resolve with Rebif®/Rebif® placebo dose modification (please refer to Section 6.2.2 for more details);*

*Only applicable during the double-blind, double-dummy treatment period.

– Patients who decide to discontinue the treatment;

– The patient's Treating Investigator decides that discontinuation of treatment is in the best clinical interest of the patient.

Patients who withdraw during the double-blind, double-dummy treatment period, OLE Phase Screening Period, or the OLE Phase, for any reason, should complete the Safety Follow-Up Period. If the patient insists on discontinuing from the study, he/she should be asked if he/she can still be contacted for further information. The outcome of that discussion should be documented in both the medical records and in the eCRF. If lost to follow-up, the Investigator should contact the patient or a responsible relative by telephone followed by registered mail or through a personal visit to establish as completely as possible the reason for the withdrawal. A complete final evaluation at the time of the patient's withdrawal should be made with an explanation of why the patient is withdrawing from the study.

When applicable, patients should be informed of circumstances under which their participation may be terminated by the Investigator without the patient's consent. The Investigator may withdraw patients from the study in the event of intercurrent illness, adverse events, treatment failure, after a prescribed procedure, lack of compliance with the study and/or study procedures (e.g., dosing instructions, study visits), cure or any reason where it is felt by the Investigator that it is in the best interest of the patient to be terminated from the study. Any administrative or other reasons for withdrawal must be documented and explained to the patient. If the reason for removal of a patient from the study is an adverse event, the principal specific event will be recorded on the eCRF. If possible, the patient should be followed until the adverse event has resolved.

An excessive rate of withdrawals can render the study non-interpretable; therefore, unnecessary withdrawal of patients should be avoided. Should a patient decide to withdraw, all efforts will be made to complete and report the observations prior to withdrawal as thoroughly as possible.

Please note: It is important to distinguish between "withdrawal from treatment" and "withdrawal from study". Patients who withdraw from treatment should be encouraged to remain in the study for the full duration of the Safety Follow-Up Period (minimum of 48 weeks following the last infusion).

It should be noted that upon withdrawal from the study, any untested routine samples will be destroyed. However, information already obtained from samples up until the time of withdrawal will be used.

### 4.6.1 Withdrawal of Subjects from the Roche Clinical Repository

Subjects who gave consent to provide RCR specimens have the right to withdraw their specimen from the RCR at any time for any reason. If a patient wishes to withdraw his/her consent to the testing of his/her specimen(s), the Investigator must inform Roche in writing of the patient's wishes using the RCR Subject Withdrawal Form and enter the date of withdrawal in the patient's eCRF. A patient's withdrawal from the main trial does not, by itself, constitute withdrawal of the specimen from the RCR; likewise, a patient's withdrawal from the RCR does not constitute a withdrawal from the main trial.

### 4.6.2 Patient Agreement for Continuation in the Study (in Case of *Confirmed* Disability Progression)

During the treatment period, in the event of *confirmed* disability progression on EDSS confirmed for 24 weeks, the benefits and risks of study treatment should be reassessed with the patient prior to any further dosing, including a discussion of alternative treatment options available for that patient. The result of this discussion must be included in the patient's file, prior to any further dosing of study medication.

If, after the discussion, the patient decides not to continue with the study treatment, they should be discontinued from any further treatment, complete applicable Withdrawal from Treatment Visit procedures and be entered into the Safety Follow-Up Period.

For definition of *confirmed disability* progression, please refer to Section 5.3.2.2.

## 4.7 Replacement Policy (Ensuring Adequate Numbers of Evaluable Subjects)

### 4.7.1 For Subjects

Patients prematurely discontinued from the study for any reason will not be replaced.

### 4.7.2 For Centers

A center may be replaced for the following administrative reasons:

- Excessively slow recruitment.
- Poor protocol adherence.
- Sponsor's discretion (Sponsor refers to F. Hoffmann-La Roche, Ltd and Genentech, Inc.)

# 5. SCHEDULE OF ASSESSMENT AND PROCEDURES

## Table 4: Schedule of Assessments: Screening through the End of Double-Blind, Double-Dummy Treatment Period

| | **Screen** | **Double-blind, Double-Dummy Treatment Period** | | | | | | | | | | | | |
|---|---|---|---|---|---|---|---|---|---|---|---|---|---|---|
| **Cycle** | | **1** | | | **2** | | **3** | | **4** | | | **Delayed Dosing Visit** [22] | **Unscheduled Visit** [23] | **Withdrawal from Treatment Visit** |
| **Visit** | **1** | **2 BL** | **3** | **4** | **5** | **6** | **7** | **8** | **9** | **10** | **11** | | | |
| **Week** | **-2** | **-** | **w2** | **w12** | **w24** | **w36** | **w48** | **w60** | **w72** | **w84** | **w96** | | | |
| *Study Day (window in days)* | *-14* | *1* | *15 (± 2)* | *85 (±4)* | *169 (± 2)* | *253 (±4)* | *337 (± 2)* | *421 (±4)* | *505 (± 2)* | *589 (±4)* | *673 (± 2)* | | | |
| | | | | | | | | | | | | | | |
| Informed consent [1] | x | | | | | | | | | x[24] | | | | |
| Medical history | x | | | | | | | | | | | | | |
| Review of eligibility criteria | x | x | | | | | | | | | x[24] | | | |
| CES-D, MFIS, EQ-5D, SF-36 | | x | | | | | x | | | | x | | | x |
| Patient's Assessment of Treatment Benefit | | | | | | | x | | | | x | | | x |
| C-SSRS | | x | | x | x | x | x | x | x | x | x | | x | x |
| Physical examination | x | x | x | | x | | x | | x | | x | x | | x |
| Vital signs [2] | x | x | x | x | x | x | x | x | x | x | x | x | x | x |
| 12 lead ECG (pre- and post-dose) [3] | x | x | | | | | | | x | | | | | x |
| Height | x | | | | | | | | | | | | | |
| Weight | x | | | | | | | | x | | x | | | x |
| Neurological exam and EDSS | x | x | | x | x | x | x | x | x | x | x | | x | x |
| MSFCS, LCVA, SDMT | | x | | x | x | x | x | x | x | x | x | | x | x |
| Karnofsky Performance Status Scale | | x | | | x | | x | | x | | x | | | x |
| MRI[4] | | x | | | x | | x | | | | x | | | x |
| Concomitant Treatment | | x | x | x | x | x | x | x | x | x | x | x | x | x |
| Adverse Events | *Only SAEs* | x | x | x | x | x | x | x | x | x | x | x | x | x |
| Potential relapses recorded | | x | x | x | x | x | x | x | x | x | x | x | x | x |
| Telephone interview (every 4 wks)[5] | | | | – – – – – – – – – – – – – – – → | | | | | | | | | | |

## Table 4: Schedule of Assessments: Screening through the End of Double-Blind, Double-Dummy Treatment Period (Cont.)

| | Screen | Double-Blind, Double-Dummy Treatment Period | | | | | | | | | | | | |
|---|---|---|---|---|---|---|---|---|---|---|---|---|---|---|
| **Cycle** | | 1 | | | 2 | | 3 | | 4 | | | Delayed Dosing Visit [22] | Unscheduled Visit [23] | Withdrawal from Treatment Visit |
| **Visit** | 1 | 2 BL | 3 | 4 | 5 | 6 | 7 | 8 | 9 | 10 | 11 | | | |
| **Week** | -2 | - | w2 | w12 | w24 | w36 | w48 | w60 | w72 | w84 | w96 | | | |
| *Study Day (window in days)* | -14 | 1 | 15 (±2) | 85 (±4) | 169 (± 2) | 253 (±4) | 337 (± 2) | 421 (±4) | 505 (± 2) | 589 (±4) | 673 (± 2) | | | |
| Pregnancy test [6] | x | x | x | x | x | x | x | x | x | x | x | x | | x |
| Antibody Titers [7] | | x | | x | x | | x | | x | | x | | | x |
| RCR (non-DNA) [8] | | x | | x | x | | x | | x | | x | | | x |
| RCR (DNA) [9] | | x | | | | | | | | | | | | |
| Protein biomarker sampling [10] | | x | | x | x | | x | | x | | x | | | x |
| HAHA [11] | | x | | | x | | x | | x | | x | | | x |
| Plasma/ urine banking for JCV [12] | | x | | x | x | x | x | x | x | x | x | | | x |
| PK Samples [13] | | x | | | x | | x | | x [13] | x | x | | | x |
| Thyroid function tests [14] | x | | | | x | | x | | x | | x | | | x |
| FSH [15] | x | | | | | | | | | | | | | |
| Hepatitis Screening [16] | x | | | | | | | | | | | | | |
| Hepatitis B virus DNA [16] | x | (x) | | (x) | (x) | (x) | (x) | (x) | (x) | (x) | (x) | | | (x) |
| RPR | x | | | | | | | | | | | | | |
| CD4 count | x | | | x | | x | | x | | x | | | | |
| IgG | | | | x | | x | | x | | x | | | | |
| Total Ig, IgA, IgG, IgM | x | | | | x | | x | | x | | x | | | x |
| FACS [17] | | x | x | x | x | | x | | x | | x | | | x |

## Table 4: Schedule of Assessments: Screening through the End of Double-Blind, Double-Dummy Treatment Period (Cont.)

| | Screen | Double-Blind, Double-Dummy Treatment Period | | | | | | | | | | | | |
|---|---|---|---|---|---|---|---|---|---|---|---|---|---|---|
| **Cycle** | | 1 | | | 2 | | 3 | | 4 | | | Delayed Dosing Visit [22] | Unscheduled Visit [23] | Withdrawal from Treatment Visit |
| **Visit** | 1 | 2 BL | 3 | 4 | 5 | 6 | 7 | 8 | 9 | 10 | 11 | | | |
| **Week** | -2 | - | w2 | w12 | w24 | w36 | w48 | w60 | w72 | w84 | w96 | | | |
| *Study Day (window in days)* | *-14* | *1* | *15 (± 2)* | *85 (±4)* | *169 (± 2)* | *253 (±4)* | *337 (± 2)* | *421 (±4)* | *505 (± 2)* | *589 (±4)* | *673 (± 2)* | | | |
| Routine safety lab [18] | x | x | x | x | x | x | x | x | x | x | x | | | x |
| Type I interferon neutralizing antibody assay [19] | | x | | | x | | x | | x | | x | | | x |
| Pre-treatment with i.v. methylprednisolone [20] | | x | x | | x | | x | | x | | | x | | |
| Administration of i.v. ocrelizumab / ocrelizumab placebo [21] | | x | x | | x | | x | | x | | | x | | |
| Assessment of s.c. Rebif®/ Rebif® placebo compliance | | x | x | x | x | x | x | x | x | x | x | | x | x |
| Administration of s.c. Rebif® / Rebif® placebo 3x/wk | | x | x | x | x | x | x | x | x | x | x[25] | | | |

1. β-hCG=beta human chorionic gonadotropin; BL=baseline; CD4=cluster of differentiation; CES-D=Center for Epidemiologic Studies Depression Scale; C-SSRS=Columbia-Suicide Severity Rating Scale; DNA=deoxyribonucleic acid; ECG=electrocardiogram; eCRF=electronic case report form; EDSS=Expanded Disability Status Scale; EDTA=ethylenediaminetetraacetic acid; EQ-5D=EuroQoL; FACS=fluorescence activated cell sorting; FSH=follicle-stimulating hormone; HAHA=human anti-human antibodies; Ig=immunoglobulin; IgA=immunoglobulin A; IgG=immunoglobulin G; IgM=immunoglobulin M; i.v.=intravenous; JCV=JC virus; LCVA=Low Contrast Visual Acuity; MFIS=Modified Fatigue Impact Scale; MRI=magnetic resonance imaging; MSFCS=Multiple Sclerosis Functional Composite Scale; n=number; OLE=Open-Label Extension; PK=pharmacokinetic; RCR=Roche Clinical Repository; RNA=ribonucleic acid; RPR=rapid plasma reagin; SAE=serious adverse event; s.c.=subcutaneous; SDMT=Symbol Digit Modalities Test; SF-36=Short-Form 36; w=week.

## Table 4: Schedule of Assessments: Screening through the End of Double-Blind, Double-Dummy Treatment Period (Cont.)

1. **Informed Consent** must be obtained in written form from all patients at screening (prior to any study–related procedure) in order to meet eligibility for the study.
2. **Vital signs** (i.e., pulse rate, systolic and diastolic blood pressure, respiration rate and temperature) will be obtained while the patient is in the semi supine position (after 5 minutes). On infusion visits, the vital signs should be taken within 45 minutes prior to the methylprednisolone infusion in all patients. In addition, vital signs should be obtained prior to ocrelizumab/ocrelizumab placebo infusion, then every 15 minutes (± 5 minutes) for the first hour; then every 30 minutes (± 10 minutes) until 1 hour after the end of the infusion. On non-infusion days, the vital signs may be taken at any time during the visit.
3. **ECG (pre- and post-dose):** on infusion visits ECG should be taken within 45 minutes prior to the methylprednisolone infusion in all patients, and within 60 minutes after completion of the ocrelizumab/ocrelizumab placebo infusion. On non-infusion days, the ECG may be taken at any time during the visit.
4. **MRI**: brain MRI scans *should occur within a window or* ± *4 weeks of the scheduled visit. Also, brain MRI scans* will be obtained in patients withdrawn from the treatment period (at a withdrawal visit) if not performed during last 4 weeks.
5. **A structured telephone interview** will be conducted by site personnel every 4 weeks (± 3 days) from Week 8 through the study to identify any new or worsening neurological symptoms that warrant an unscheduled visit and collect data on possible events of infections.
6. **Serum β-hCG** must be performed at screening in women of childbearing potential. Subsequently, urine β-hCG [sensitivity of at least 25 mIU/mL] will be performed. On infusion visits, the urine pregnancy test should be performed prior to methylprednisolone infusion in all women of child-bearing potential. If positive, the patient will not receive the scheduled dose and confirmation, a serum pregnancy test, will be performed.
7. **Antibody Titers**: measurement of antibody titers against common antigens (mumps, rubella, varicella and *Streptococcus pneumoniae*) will be performed.
8. **RCR - Roche Clinical Repository non-DNA (RNA – and protein):** for RNA 2x 2.5 mL whole blood samples to be collected from consenting patients only for expression profiling analysis. For protein: 6 mL blood samples in EDTA tube for plasma samples will be collected from consenting patients only for analysis of protein biomarkers. On infusion visits, ALL samples should be collected 5-30 minutes prior to methylprednisolone infusion.
9. **RCR - Roche Clinical Repository (DNA):** 6 mL whole blood sample to be collected from only from patients consenting to RCR for pharmacogenetic and genetic analysis. If not done at Baseline (Visit 2), sample may be collected at next visit.
10. **Protein biomarker sampling:** one serum sample (6 mL) will be collected from all patients for analysis of protein biomarkers. On infusion visits, samples should be collected 5-30 minutes prior to methylprednisolone infusion.
11. **HAHA**: On infusion visits, serum samples are collected 5-30 minutes prior to the methylprednisolone infusion.
12. **Plasma and urine samples for JCV will be collected** at specified time points and analyzed in batches, if decided by the Sponsor.

## Table 4: Schedule of Assessments: Screening through the End of Double-Blind, Double-Dummy Treatment Period (Cont.)

13. **PK samples:** on the infusion day at week 72, two serum samples should be collected, one 5-30 minutes prior to the methylprednisolone infusion and the second one 30 minutes (±10 minutes) following the completion of the ocrelizumab/ocrelizumab placebo infusion. For all other infusion visits, a blood sample should be collected 5 – 30 minutes before the methylprednisolone infusion. At other times (non-infusion visits) samples may be collected at any time during the visit.
14. **Sensitive thyroid-stimulating hormone (sTSH)** will be tested at screening and during the double-blind, double-dummy treatment period. Thyroid autoantibodies will be assayed only at screening.
15. **FSH**: only applicable to women to confirm the postmenopausal status.
16. **Hepatitis** screening & monitoring: all patients must have negative hepatitis B surface antigen (HbsAg) result and negative hepatitis C antibody (HepCAb) screening tests prior to enrollment. If total hepatitis B core antibody (HbcAb) is positive at screening, HB virus DNA measured bypolymerase chain reaction (PCR) must be negative to be eligible. For those patients enrolled with negative HBsAg and positive total HBcAb, HB virus DNA (PCR) must be repeated every 12 weeks during the treatment period.
17. **FACS**: including CD19 and other circulating B-cell subsets, T-cells, natural killer cells, and other leukocytes. On infusion visits, blood samples should be collected prior to the infusion of methylprednisolone.
18. **Routine safety lab:** hematology, chemistry and urinalysis: on infusion visits, all urine and blood samples should be collected prior to the infusion of methylprednisolone. At other times, samples may be collected at any time during the visit.
19. **Type I interferon neutralizing antibody assay:** At baseline (visit 2), sample should be collected before first Rebif®/Rebif® placebo injection. At subsequent visits, samples should be collected at least 36 hours following last injection of Rebif®/Rebif® placebo.
20. All patients receive **prophylactic treatment** with 100 mg of methylprednisolone i.v. prior to infusion of ocrelizumab /ocrelizumab placebo. In the rare case when the use of methylprednisolone is contraindicated for the patient, use of an equivalent dose of an alternative steroid should be used as premedicationprior to the infusion. It is also recommended that patients receive an analgesic/antipyretic such as acetaminophen/paracetamol (1 g) and an i.v. or oral antihistaminic such as diphenhydramine 50 mg 30-60 minutes prior to ocrelizumab/ ocrelizumab placebo.
21. **Administration (infusion) of i.v. ocrelizumab/ocrelizumab placebo**: the Treating Investigator must review the clinical and laboratory re-treatment criteria prior to subsequent infusion of ocrelizumab/ocrelizumab placebo.
22. **A delayed dosing visit** will be performed and recorded in the Delayed Dosing Visit eCRF form when dosing cannot be administered at the scheduled dosing visit. Other tests or assessments may be done as appropriate.

## Table 4: Schedule of Assessments: Screening through the End of Double-Blind, Double-Dummy Treatment Period (Cont.)

23. **Unscheduled Visit:** assessments performed at unscheduled (non-dosing) visits will depend on the clinical needs of the patient. All patients with new neurological symptoms suggestive of relapse should have EDSS performed by examining Investigator, *whenever possible within 7 days of the onset of the relapse*. Other tests/assessments may be done as appropriate. Please note: in case of alanine aminotransferase (ALT) elevations dose modification should be necessary, additional visits may be required for dispensing of study medication.
24. At the Week 84 Visit, a discussion with the patient regarding participation in the Open-Label Extension Phase should occur if the Treating Investigator is of the opinion that the patient may benefit from treatment with ocrelizumab. An informed consent for the OLE Phase should be provided to the patient. It is recommended that the Informed Consent Form of the Open-Label Extension Phase be signed at the Week 96 Visit.
25. If the patient enters OLE Phase Screening Period, Rebif® / Rebif® placebo should be provided to the patient at the Week 96 Visit (please see Section 5.10.1).

Please note: based on local Ethics Committees or National Competent Authority requirements, additional diagnostic testing may be required for selected patients or selected centers to exclude tuberculosis, Lyme disease, HTLV-1 associated myelopathy (HAM), acquired immune deficiency syndrome (AIDS), hereditary disorders, connective tissue disorders, or sarcoidosis. Other specific diagnostic tests may be requested when deemed necessary by the Investigator.

## Table 5: Schedule of Assessments: Safety Follow-Up (including prolonged B-cell monitoring if required)

| | Safety Follow-Up | Prolonged B-cell Monitoring[1] | End of observation or withdrawal from Safety Follow up |
|---|---|---|---|
| **Assessments** | *Visits every 12 weeks (±7 days)* [2] | *Visits every 24 weeks (±7 days)* | |
| Urine pregnancy test | x | x | x |
| Routine Safety Labs[3] | x | x | x |
| FACS [4] | x | x | x |
| Total Ig, IgA, IgG, IgM | x[10] | x | x |
| HAHA [5] | x[10] | x | x |
| Plasma/urine banking for JCV | x | x | x |
| Antibody titers | x[10] | x | x |
| Hepatitis B viral DNA[6] | (x) | (x) | (x) |
| Protein biomarker sampling [8] | x[10] | x | x |
| Vital Signs | x | x | x |
| EDSS | x | | x |
| Neurological examination | x | x | x |
| Physical examination | x[10] | x | x |
| Potential relapses recorded | x | x | x |
| Adverse events | x | x | x |
| Concomitant Medication | x | x | x |
| Telephone interview [9] | x | x | |

CD=cluster of differentiation; DNA=deoxyribonucleic acid; EDSS=Expanded Disability Status Scale; EDTA=ethylenediaminetetraacetic acid; FACS=fluorescence activated cell sorting; HAHA=human anti-human antibodies; HbcAb=hepatitis C antibody; HbsAg=hepatitis B surface antigen; HBV=hepatitis B virus; Ig=immunoglobulin; IgA=immunoglobulin A; IgG=immunoglobulin G; IgM=immunoglobulin M; i.v.=intravenous; JCV=JC virus; RNA=ribonucleic acid.

**Table 5: Schedule of Assessments: Safety Follow-Up (including prolonged B-cell monitoring if required) (Cont.)**

1. **Prolonged B-cell monitoring**: patients whose B-cells have not been repleted after 48 weeks of Safety Follow-Up Period will continue with visits every 24 weeks (± 7 days) until B-cell repletion.
2. **Safety Follow-Up** will be carried out for at least 48 weeks starting from the date of last infusion of ocrelizumab. Visits will be performed at 12-week intervals starting from the date of the patient's Withdrawal From Treatment Visit. *Safety Follow-Up applies to study patients who have completed the blinded treatment period (or open label treatment period, if applicable) and to patients who withdraw early from treatment. If B-cells have returned to normal levels at this visit, then the 48 week Safety Follow-up visit will become the End of Observation visit and the patient will have completed the study. An End of observation visit will be performed only in case of occurrence of new adverse event and/or if the investigator considers the prior safety assessment (laboratory, other) as abnormal and clinically significant.*
3. **Routine safety lab**: hematology, chemistry and urinalysis.
4. **FACS** including CD19 and other circulating B-cell subsets, T cells, natural killer cells and other leukocytes.
5. **HAHA**: two serum samples are required.
6. **Hepatitis monitoring:** hepatitis to be monitored only in patients with screening results of HbsAg negative, HBcAb positive and HBV DNA negative, inclusive.
7. **Protein biomarker sampling**: 6 mL blood sample in a plain tube without EDTA for serum isolation will be collected from all patients for analysis of protein biomarkers.
8. **A structured telephone interview** will be performed by site personnel every 4 weeks (± 3 days) between visits until 48 weeks after the last infusion to identify any new or worsening neurological symptoms that warrant an unscheduled visit and collect data on possible events of infections. If prolonged B-cell monitoring is required beyond 48 weeks after the last infusion, telephone interviews will be done every 12 weeks (± 7 days) between visits.
9. Needs to be assessed only every 24 weeks.

Please note: patients in Safety Follow-Up who receive other B-cell targeted therapies will only be followed for 48 weeks from the date of the last infusion of the study drug regardless of their B-cell count.

## Table 6: Schedule of Assessments: Open Label Extension Phase

| | OLE Screen | Open-Label Extension Phase[1] | | | | | | | | | | | Delayed dosing Visit[20] | Unscheduled Visit[21] | Withdrawal from Treatment Visit |
|---|---|---|---|---|---|---|---|---|---|---|---|---|---|---|---|
| Cycle[1] | | 5 | | | 6[1] | | 7[1] | | 8[1] | | N[1,3] | | | | |
| Visit | | 12 | 13 | 14 | 15 | 16 | 17 | 18 | 19 | 20 | … | … | | | |
| Week in OLE Phase (window in days) | | *0*[2] | *2* (± 2) | *12* (± 7) | *22* (± 7) | *24* (±5) | *46* (± 7) | *48* (± 5) | *70* (± 7) | *72* (± 5) | *n-2 wk* (± 7) | *n* (± 7) | | | |
| Informed consent[4] | x | | | | | | | | | | | | | | |
| Review of eligibility criteria | x | x | | | | | | | | | | | | | |
| Review of retreatment criteria | | x | x | | | x | | x | | x | | x | x | | |
| Administration of s.c. Rebif® / Rebif® placebo 3x/wk [5] | x | | | | | | | | | | | | | | |
| Assessment of s.c. Rebif®/ Rebif® placebo compliance | | x | | | | | | | | | | | | | |
| Pre-treatment with i.v. methylprednisolone[6] | | x | x | | | x | | x | | x | | x | x | | |
| Administration of i.v. ocrelizumab[7] | | x | x | | | x | | x | | x | | x | x | | |
| Physical examination | | x | x | | | x | | x | | x | | x | x | | x |
| Vital signs, weight[8] | | x | x | x | | x | | x | | x | | x | x | x | x |
| 12-lead ECG (pre- and post-dose, once early)[9] | | x | x | | | | | | | x | | (x) | | | x |
| Neurological exam and EDSS | | x | | x | | x | | x | | x | | x | | x | x |
| Routine safety labs[10] | | x | | x | x | | x | | x | | x | | | x | x |
| Adverse events | | x | x | x | x | x | x | x | x | x | x | x | x | x | x |
| Concomitant treatments | | x | x | x | x | x | x | x | x | x | x | x | x | x | x |
| Potential relapses recorded | | x | x | x | x | x | x | x | x | x | x | x | x | x | x |
| Pregnancy test[11] | | x | x | x | | x | | x | | x | | x | x | | x |
| Antibody titers[12] | | x | | x | x | | x | | x | | x | | | | x |
| Total Ig, IgA, IgG, IgM | | x | | | x | | x | | x | | x | | | | |
| CD4 count | | x | x | | x | | x | | x | | x | | | | |

## Table 6: Schedule of Assessments: Open Label Extension Phase (Cont.)

| | | Open-Label Extension Phase[1] | | | | | | | | | | | | | |
|---|---|---|---|---|---|---|---|---|---|---|---|---|---|---|---|
| Cycle[1] | OLE Screen | 5 | | | 6[1] | | 7[1] | | 8[1] | | N[1,3] | | Delayed dosing Visit[20] | Unscheduled Visit[21] | Withdrawal from Treatment Visit |
| Visit | | 12 | 13 | 14 | 15 | 16 | 17 | 18 | 19 | 20 | … | … | | | |
| Week in OLE Phase (window in days) | | *0[2]* | *2 (± 2)* | *12 (± 7)* | *22 (± 7)* | *24 (±5)* | *46 (± 7)* | *48 (± 5)* | *70 (± 7)* | *72 (± 5)* | *n-2 wk (± 7)* | *n (± 7)* | | | |
| HAHA[22] | | x | | | x | | x | | x | | x | | | | *x* |
| *Ocrelizumab concentration sample[22]* | | *x* | | | *x* | | *x* | | *x* | | *x* | | | | *x* |
| Telephone interview (every 4 weeks)[13] | | | | ‑ ‑ ‑ ‑ ‑ ‑ | ‑ ‑ ‑ ‑ ‑ ‑ | ‑ ‑ ‑ ‑ ‑ ‑ | ‑ ‑ ‑ ‑ ‑ ‑ | ‑ ‑ ‑ ‑ ‑ ‑ | ‑ ‑ ‑ ‑ ‑ ‑ | ‑ ‑ ‑ ‑ ‑ ‑ | ‑ ‑ ‑ ‑ ‑ ‑ | ‑ ‑ ► | | | |
| EQ-5D (once yearly) | | x | | | | | x | | | | (x) | | | | |
| Protein biomarker sampling[15] | | x | | x | x | | x | | x | | x | | | | |
| FACS[16] | | | x | x | x | | x | | x | | x | | | | |
| Hepatitis B virus DNA[17] | | | | (x) | (x) | | (x) | | (x) | | (x) | | | | |
| MRI (once yearly)[18] | | | | | | | x | | | | (x) | | | | |
| Patient's Assessment of Treatment Benefit (once yearly) | | | | | | | x | | | | (x) | | | | |
| Plasma/urine banking for JCV[19] | | x | | x | x | | x | | x | | x | | | | x |

CD4 = cluster of differentiation; DNA = deoxyribonucleic acid; ECG = electrocardiogram; eCRF = electronic case report form; EDSS = Expanded Disability Status Scale; EQ-5D = EuroQoL; FACS = fluorescence activated cell sorting; HAHA = human anti-human antibodies; Ig = immunoglobulin; IgA = immunoglobulin A; IgG = immunoglobulin G; IgM = immunoglobulin M; i.v. = intravenous; JCV = JC virus; MRI = magnetic resonance imaging; n = number; OLE = Open-Label Extension; RCR = Roche Clinical Repository; s.c. = subcutaneous.

1. **The OLE Phase** can terminate at any moment or cycle (please refer to End of Study Section 3.1.4). In case the study is ended, a Withdrawal from Treatment Visit should occur.
2. **The OLE Phase** starts at the first infusion of Cycle 5. The OLE Phase Screening Period will start after all assessments at the Week 96 Visit have been performed, and it should last approximately 4 weeks. It is possible that the OLE Phase Screening Period could be longer than 4 weeks. If a prolongation of the OLE Phase Screening Period is needed, it should be discussed with the Sponsor on a case-by-case basis.
3. The assessments requested for N represent the typical schedule of assessments during a cycle.
4. The **informed consent** should have been provided to the patient at the Week 84 Visit and signed by the patient at the Week 96 Visit.

## Table 6: Schedule of Assessments: Open Label Extension Phase (Cont.)

5. During the OLE Phase Screening Period, s.c. administration Rebif® / Rebif® placebo 3 times per week should occur until one day prior to the first infusion of ocrelizumab of Cycle 5 (beginning of OLE Phase). If during this period, the patient decides not to participate in the OLE Phase, then administration of Rebif® / Rebif® placebo 3 times per week should stop immediately, and the patient will be entered in the Safety Follow-Up Period.
6. All patients receive prophylactic treatment with 100 mg of methylprednisolone i.v. prior to infusion of ocrelizumab. In the rare case when the use of methylprednisolone is contraindicated for the patient, use of an equivalent dose of an alternative steroid should be used as premedication prior to the infusion. It is also recommended that patients receive an analgesic/antipyretic such as acetaminophen/paracetamol (1 g) and an i.v. or oral antihistaminic such as diphenhydramine 50 mg 30-60 minutes prior to ocrelizumab.
7. **Administration (infusion) of i.v. ocrelizumab**: the Investigator must review the clinical and laboratory re-treatment criteria prior to subsequent infusion of ocrelizumab.
8. **Vital signs** (i.e., pulse rate, systolic and diastolic blood pressure, respiration rate, and temperature) will be obtained while the patient is in the semi-supine position (after 5 minutes). Vital signs should be collected within 45 minutes prior to the methylprednisolone infusion in all patients. In addition, vital signs should be obtained prior to ocrelizumab infusion, then every 15 minutes (± 5 minutes) for the first hour; then every 30 minutes (± 10 minutes) until 1 hour after the end of the infusion.
9. **ECGs** (pre- and post-dose): ECG should be performed within 45 minutes prior to the methylprednisolone infusion in all patients, and within 60 minutes after completion of the ocrelizumab infusion.
10. **Routine safety lab**: hematology, chemistry, and urinalysis. On infusion visits at Cycle 5, all urine and blood samples should be collected prior to the infusion of methylprednisolone. At other times, samples may be collected at any time during the visit.
11. **Urine β-hCG** [sensitivity of at least 25 mIU/mL] will be performed. On infusion visits, the urine pregnancy test should be performed prior to methylprednisolone infusion in all women of childbearing potential. If positive, the patient will not receive the scheduled dose and for confirmation a serum pregnancy test will be performed.
12. **Antibody Titers**: measurement of antibody titers against common antigens (mumps, rubella, varicella, and Streptococcus pneumoniae) will be performed.
13. A structured **telephone interview** will be conducted by site personnel every 4 weeks (± 3 days) from Cycle 5 *(Week 8 of OLE Phase)* through the study to identify any new or worsening neurological symptoms that warrant an unscheduled visit and collect data on possible events of infections.
14. **Protein biomarker sampling**: one serum sample (6 mL) will be collected from all patients for analysis of protein biomarkers. On infusion visits, samples should be collected 5-30 minutes prior to methylprednisolone infusion.
15. **FACS**: including CD19 and other circulating B-cell subsets, T-cells, natural killer cells, and other leukocytes.
16. **Hepatitis monitoring**: For those patients enrolled with negative HBsAg and positive total HBcAb, HB virus DNA (PCR) must be repeated every 24 weeks during the treatment period.
17. **MRI**: brain MRI scans *should occur within a window of* ± *4 weeks of the scheduled visit. Also, brain MRI scans* will be obtained in patients withdrawn from the OLE Phase (at a withdrawal visit) if not performed during last 4 weeks.
18. **Plasma and urine samples for JCV** will be collected at specified time points
19. A **delayed dosing visit** will be performed and recorded in the Delayed Dosing Visit eCRF form when dosing cannot be administered at the scheduled dosing visit. Other tests or assessments may be done as appropriate.

## Table 6: Schedule of Assessments: Open Label Extension Phase (Cont.)

20. **Unscheduled Visit**: assessments performed at unscheduled (non-dosing) visits will depend on the clinical needs of the patient. All patients with new neurological symptoms suggestive of relapse should have EDSS performed*, whenever possible within 7 days of the onset of the relapse*. Other tests/assessments may be done as appropriate.
21. ***HAHA and Ocrelizumab drug concentration samples**: At the infusion Visit 12 (Cycle 5), a blood sample should be collected before the methylprednisolone infusion. At non-infusion visits, samples may be collected at any time during the visit.*

## 5.1 Screening Examination and Eligibility Screening Form

All patients must sign and date the most current Institutional Review Board/Institutional Ethics Committee's (IRB/IEC) approved written informed consent before any study specific assessments or procedures are performed.

Consenting patients will enter the 2-week screening period to be evaluated for eligibility. Please refer to "Schedule of Assessments: Screening through the End of Double-Blind, Double-Dummy Treatment Period" - Table 4 for details. Patient must fulfill all entry criteria for participation in the study.

The screening period can be extended to a total period of 8 weeks in cases when a laboratory blood test or MRI scan need to be repeated for confirmation during the screening interval, or for other relevant clinical, administrative, or operational reasons.

Please note that based on local Ethics Committees or National Competent Authority requirements, additional diagnostic testing may be required for selected patients or selected centers to exclude tuberculosis, Lyme disease, HAM, AIDS, hereditary disorders, connective tissue disorders, or sarcoidosis.

An Eligibility Screening Form [ESF] documenting the Investigator's assessment of each screened patient with regard to the protocol's inclusion and exclusion criteria is to be completed by the Investigator.

Each patient screened must be registered in the IxRS by the Investigator or the Investigator's research staff at screening. A screen failure record must be maintained by the Investigator, and reasons must be captured in the IxRS.

It should be stated in the medical record that the patient is participating in this clinical study.

## 5.2 Procedures for Enrollment of Eligible Subjects

Once a patient has fulfilled all eligibility criteria, he or she will be randomized via IxRS to one of two treatment groups: ocrelizumab 600 mg (given as 300 mg × 2 14 days apart for the first 24 weeks and 600 mg × 1 every 24 weeks thereafter) or Rebif®.

Patient eligibility information will be provided to the IxRS by the Investigator or the Investigator's research staff at randomization. The patient will be randomized and assigned a unique treatment box number (medication number) and randomization number. As confirmation, the site will be provided with a verification of each patient's randomization.

The patient randomization numbers will be generated by Roche or its designee and incorporated into the double-blind labeling.

The patient randomization numbers are to be allocated sequentially in the order in which the patients are enrolled according to the specification document agreed with the external randomization company/center.

Treatment with the first study drug infusion should occur within 24 hours of randomization. In exceptional cases where all baseline assessments cannot be completed

within 24 hours, the first study drug infusion can be administered within 48 hours of randomization provided that the Investigator assures that all inclusion and exclusion criteria are still met on the day of dosing. In particular, there should be no evidence of an ongoing infection at the time of dosing.

No patient may begin treatment prior to randomization and assignment of a medication number.

## 5.3 Clinical Assessments and Procedures During the Double Blind, Double-Dummy Treatment Period

This is an assessor blinded study. During the double-blind, double-dummy treatment period, each site will have two Investigators: a principal or Treating Investigator and an Examining Investigator or rater.

- **The Treating Investigator** is the physician responsible for the patient care and should be a neurologist experienced in the care of MS patients. The Treating Investigator will have access to safety and blinded efficacy data and will make treatment decisions based on the patient's clinical response and laboratory findings.
- **The Examining Investigator** should be a neurologist or other health care practitioner and must be trained and certified in administering the Neurostatus Functional System Scores (FSS) and Expanded Disability Status Scale (EDSS) examination prior to study start.

The Examining Investigator will perform the neurological examination, document the FSS scores, and assess EDSS scores and the Karnofsky Performance Status Scale. The Examining Investigator or a qualified designee will also be responsible for performing and documenting results from the following: MSFCS, low-contrast visual acuity testing, and the Symbol Digit Modalities Test. They will only have access to data from the assessments listed above. Every effort will be made to ensure that there is no change in the EDSS rater throughout the course of the study for any individual patient. Whenever possible, the same person should perform the examination for the full study duration.

All efforts should be made to keep the Examining Investigator blinded to the treatment assignment during the double-blind, double-dummy treatment period. Patients will be instructed not to discuss any symptoms related to the study treatment with the Examining Investigator; the Examining Investigator should remind the patient at the start of the examination. In view of the extended duration of this study, each site will identify a primary and back-up for Treating and Examining Investigator. **The Treating Investigator and the Examining Investigator will not be allowed to switch roles**.

### 5.3.1 Overview of Clinical Visits During the Double-Blind, Double-Dummy Treatment Period

After the screening visit, patients fulfilling the entry criteria will be scheduled for the baseline assessments. Randomization will occur only after the patient meets all inclusion and exclusion criteria on Day 1. Visits will take place as described in the Schedule of Assessments (Table 4).

Visits should be scheduled with reference to the date of the baseline visit (Day 1). A minimum interval of 20 weeks kept between the ocrelizumab/ocrelizumab placebo second infusion of Cycle 1 (i.e., infusion Week 2) and the next infusion on Cycle 2 (Week 24). A minimum of 22 weeks must occur between ocrelizumab/ ocrelizumab placebo single infusions administered during Weeks 24, 48, and 72.

At infusion visits, patients treated with ocrelizumab /ocelizumab placebo should remain in observation for at least 1 hour after the completion of the infusion.

If for logistical reasons the ocrelizumab/ocrelizumab placebo infusion at Week 24, 48, or 72 cannot be administered on the same study visit day, the infusion should be given within the next 24 hours provided that the patient still meets re-treatment criteria (refer to Section 6.1.4).

Patients who cannot receive their infusion at the scheduled visit or within 24 hours of the visit should be re-scheduled for a delayed dosing visit– see Section 5.3.1.1._Additional unscheduled visits for the assessment of potential relapses, new neurological symptoms, safety events or for dispensing Rebif®/Rebif® placebo if down titration is needed may occur at any time.

#### 5.3.1.1 Delayed Dosing Visit

Delayed dosing visits may be scheduled only if the infusion cannot be administered at the time points defined in Schedule of Assessments -Table 4. Thus, a patient who had all assessments of a dosing visit performed, but could not receive his/her infusion, should be re-scheduled for the infusion. Delayed dosing visit should not be scheduled for the first infusion of the first treatment cycle (Day 1), as treatment with the first study drug infusion should occur within 24 hours of randomization (in exceptional cases within 48 hours of randomization provided that the Investigator assures that all inclusion and exclusion criteria are still met on the day of dosing), see Section 5.2.

In unforeseen situations, if the infusion of the first treatment cycle (Day 1) is delayed, then the visit for the second infusion should be scheduled 14 days after the delayed first infusion (± 2 days). In the event any subsequent infusion needs to be delayed, a minimum interval of 20 weeks between the second infusion of cycle 1 (Week 2) and the next infusion on cycle 2 (Week 24) is required; a minimum of 22 weeks must occur between infusions administered during Weeks 24, 48, and 72.

At the delayed dosing visit, additional tests or assessments, such as routine safety laboratory tests, may be performed when the Investigator judges that these are warranted.

#### 5.3.1.2 Unscheduled Visits

Patients developing new or worsening neurological symptoms should be seen at the investigational site as soon as possible regardless of the treatment group to which they were randomized, regardless of the dates of their pre-planned, scheduled study visits, and regardless of the study period. Assessments performed at unscheduled (non-dosing) visits will depend on the clinical needs of the patient.

Patients with new neurological symptoms suggestive of relapse should have an EDSS performed by Examining Investigator*, whenever possible within 7 days of the onset of the relapse*. Other tests/assessments may be done as appropriate. Please note: if, during the double-blind, double-dummy treatment period, Rebif®/Rebif® placebo dose modification is necessary in case of ALT elevations, then unscheduled visits may be required for dispensing of study medication (see Section 6.2).

Please refer also to Section 7.3.4.1 for guidance on the diagnosis of progressive multifocal leukoencephalopathy (PML).

#### *5.3.1.3 Withdrawal Visits*

At the moment a patient meets one or more of the withdrawal criteria (Section 4.6), this patient is regarded withdrawn from treatment. Patient who withdraw from ocrelizumab treatment will need to complete all assessments as shown in Schedule of Assessments and will enter the Safety Follow-up.

*At the termination of the study (see also Section 3.1.4 for definition of end of the study), the patients will receive either commercial ocrelizumab (if available in that country) or will enter the Safety Follow-up. All patients will undergo a complete final evaluation according to the 'Withdrawal from Treatment Visit' in the Schedule of Assessments, Table 6. Thereafter, all patients will be treated according to individual center practice.*

For patients who have withdrawn from the double-blind, double-dummy treatment period, OLE Phase, or the OLE Phase Screening Period or who are not eligible for treatment with ocrelizumab, it is at the discretion of the Investigator to decide on further treatment of the underlying disease. However, immunosuppressants, lymphocyte depleting therapies, and lymphocyte trafficking blockers may increase the risk of infections while patients remain B-cell depleted and therefore are not allowed during the Safety Follow-Up Period.

Please note: at the Withdrawal from Double-Blind, Double-Dummy Treatment Period Visit *or OLE Phase*, an MRI scan will be required only if not performed in the prior 4 weeks.

### 5.3.2 Assessment of Efficacy

#### *5.3.2.1 Assessment of Relapse*

All new or worsening neurological events consistent with MS representing a clinical relapse are to be reported on the dedicated page of eCRF. Patients with clinical relapses should be referred to the Examining Investigator who will assess the FSS/EDSS independently to allow confirmation as to whether or not the clinical relapse(s) meet the criteria for protocol-defined relapse(s).

**Protocol-defined relapse** is the occurrence of new or worsening neurological symptoms attributable to MS. Symptoms must persist for >24 hours and should not be attributable to confounding clinical factors (e.g., fever, infection, injury, adverse reactions to medications) and immediately preceded by a stable or improving neurological state for at least 30 days. The new or worsening neurological symptoms must be accompanied by objective neurological worsening consistent with an increase of at least half a step on the

EDSS scale, or 2 points on one of the appropriate FSS, or 1 point on two or more of the appropriate FSS. The change must affect the selected FSS (i.e., pyramidal, ambulation, cerebellar, brainstem, sensory, or visual). Episodic spasms, sexual dysfunction, fatigue, mood change, or bladder or bowel urgency or incontinence will not suffice to establish a relapse. NB: Sexual dysfunction and Fatigue will not be scored. Please note: adjudication of protocol-defined relapses will be performed by the Sponsor based on pre-specified criteria, applied to data collected by Investigator, in a blinded fashion.

**All patients with new neurological symptoms suggestive of a relapse should be referred to the Examining Investigator for EDSS assessment*, whenever possible within 7 days of the onset of the relapse*. Any patient, complaining of a neurological symptom, defined at a visit or over the phone, should be referred to the Examining Investigator unless the Treating Investigator determines that the symptom is due to mitigating circumstances (such as an intensification of neurological symptoms from a transient systemic infection).**

Please note: clinical relapses (i.e., regardless of whether they meet criteria for a protocol-defined relapse) will be recorded on a pre-specified eCRF "MS relapse" eform. MS relapses should not be reported on Adverse Event eform of eCRF.

#### 5.3.2.2 Assessment of Disability

**Disability progression** has been defined as an increase of ≥ 1.0 point from the baseline EDSS score that is not attributable to another etiology (e.g., fever, concurrent illness, or concomitant medication) when the baseline score is 5.5 or less, and ≥ 0.5 when the baseline score is above 5.5. Disability progression is considered *confirmed* when the increase in the EDSS is confirmed at a regularly scheduled visit at least 12 weeks or 24 weeks, after the initial documentation of neurological worsening.

*Confirmed* disability progression, confirmed for 12 weeks, after the initial documentation of neurological worsening, will be analyzed as key secondary endpoint. *Additionally, confirmed disability progression for 24 weeks will be assessed.* The initial event of neurological worsening must occur during the 96-week, double-blind, double-dummy, treatment period.

#### 5.3.2.3 Kurtzke Expanded Disability Status Scale

The EDSS is based on a standard neurological examination, incorporating the following functional systems (pyramidal, cerebellar, brainstem, sensory, bowel and bladder, visual, and cerebral [or mental]) and ambulation rated and scored as functional system scores (FSS). Each FSS is an ordinal clinical rating scale ranging from 0 to 5 or 6. These ratings are then used in conjunction with observations and information concerning ambulation and use of assistive devices to determine the EDSS score. The EDSS is a disability scale that ranges in 0.5-point steps from 0 (normal) to 10 (death) [58].

The EDSS will be assessed by the Examining Investigator. **All patients with new neurological symptoms suggestive of relapse should have EDSS performed during an unscheduled visit.**

#### 5.3.2.4 *The Multiple Sclerosis Functional Composite Scale*

The Multiple Sclerosis Functional Composite Scale (MSFCS) consists of three subscales, including the 9-Hole Peg Test, Paced Auditory Serial Addition Test (PASAT), and Timed 25-Foot Walk (25-TW), which provide a global quantitative estimate of MS *disability* progression [59].

The MFSCS will be performed by the Examining Investigator or a qualified designee who must remain blinded to the treatment assignment.

#### 5.3.2.5 *Low-Contrast Visual Acuity Testing*

Low-contrast letter acuity charts (Sloan charts) have gained validity in the assessment of visual dysfunction in patients with MS not readily apparent on commonly used high-contrast acuity tests. Reductions in low-contrast letter acuity are associated with MS and correlate with increasing disability, MRI abnormalities, and reduced retinal nerve fiber layer (RNFL) thickness as measured by OCT.

LCVA testing will be performed using low contrast letter acuity charts (low contrast Sloan letter charts) by the Examining Investigator or a qualified designee at the timepoints indicated in the Schedule of Assessments - Table 4.

#### 5.3.2.6 *The Symbol Digit Modalities Test*

The SDMT has demonstrated sensitivity in detecting not only the presence of cognitive impairment, but also changes in cognitive functioning over time and in response to treatment. The SDMT is brief, easy to administer, and involves a simple substitution task that normal children and adults can easily perform. Using a reference key, the examinee has 90 seconds to pair specific numbers with given geometric figures. Responses can be written or oral, and for either response mode, administration time is just 5 minutes.

SDMT will be administered by the Examining Investigator or a qualified designee at the timepoints indicated in the Schedule of Assessments - Table 4.

### 5.3.3 Brain Magnetic Resonance Imaging

MRI is a useful tool for monitoring CNS lesions in MS. Different MRI derived parameters have been related to clinical activity and T1 weighted Gd-enhancing lesions or new and/or enlarging hyperintense T2 lesions have been related to relapses. It is hypothesized that changes in brain volume may reflect brain atrophy as a result of MS-related tissue loss and may thereby correlate with long-term clinical outcome in these patients.

Brain MRI scans will be obtained in all patients as detailed in the Schedule of Assessments - Table 4. In addition, brain MRI scans will be obtained in patients withdrawn from the double-blind *double-dummy* phase of treatment period (at the withdrawal visit) if not performed during the previous 4 weeks.

Scans will be performed by trained and certified MRI technicians. The following time windows apply:

- "Baseline" MRI should be performed after screening visit, but at least 10 days prior to the baseline visit.

- MRI at visits scheduled at Weeks: 24, 48, 96 or at withdrawal visit (if applicable) should be performed within a window of ± 4 weeks of the scheduled visit.
- In the OLE Phase, MRI should be performed yearly (e.g., Cycles 7, 9, etc.) *within a window of ± 4 weeks of the scheduled visit.*

If patients receive corticosteroids for an MS relapse, every effort should be made to obtain the scan prior to the first steroid dose if the pre-steroid scan is within 1 week of the scheduled visit. In patients receiving corticosteroids for an MS relapse, there should be an interval of 3 weeks between the last dose of corticosteroids and the scan.

The MRI will include the acquisition of scans at each time point with and/or without intravenously administered Gd contrast enhancement.

MRI scans will be read by a centralized reading center for efficacy endpoints. The centralized reading center is blinded to the treatment assignment and the reading is performed in the absence of clinical information. Further details on scanning acquisition sequences, methods, handling and transmission of the scans, certification of site MRI radiologist/technicians, and the procedures for the blinded analysis of the scans at the central reading center are described in a separate MRI Acquisition Procedures Manual.

All MRI scans will also be reviewed locally by a radiologist for safety. During the double-blind, double-dummy treatment period, the MRI scan report containing only non-MS pathology will be provided to the Treating Investigator (see Section 5.3 for definition). At the investigational site, only the local radiologist/technician assigned to this study may have access to the MRI scans post-randomization; the Treating Investigator should not review the MRI scans obtained after randomization unless a safety concern arises. In the event that the Treating Investigator does become aware of these MRI results, this should be documented in the eCRF, indicating the reason.

*Note that d*uring the OLE Phase, it is possible for the Treating Investigator to have access to MRI scans performed during the OLE Phase.

#### 5.3.4 Safety

Adverse events, vital signs, weight, physical and neurological examination, clinical laboratory tests (including pregnancy tests), 12 lead ECG, locally reviewed MRI for safety (non MS CNS pathology), and data on concomitant medications and diseases will be collected throughout the study.

Please note: On the infusion days, the vital signs should be taken within 45 minutes prior to the methylprednisolone infusion in all patients. In addition, the vital signs should be obtained prior to the study drug infusion, then every 15 minutes (± 5 minutes) for the first hour; then every 30 minutes (± 10 minutes) until 1 hour after the end of the infusion. On non-infusion days, the vital signs may be taken at any time during the visit. Additional vital signs readings may be taken at the discretion of the Investigator in the event of an infusion related reaction or if clinically indicated and should be recorded on the unscheduled vital signs eCRF.

Please refer to relevant sections of protocol for more details.

#### 5.3.4.1 Electrocardiogram

A 12-lead ECG should be taken at the visits indicated in the Schedule of Assessments - Table 4. Comments generated automatically by the ECG machine should not be recorded in the eCRF unless confirmed by a physician. An ECG is also required if the patient prematurely withdraws from the study.

#### 5.3.4.2 Physical Examination

The physical examination will be performed as per Schedule of Assessments. Diagnosis of new abnormalities or clinically significant worsening of pre-existing abnormalities should be recorded as adverse events if appropriate.

#### 5.3.4.3 Neurological Examination

A neurological examination will be performed at every planned visit and at unscheduled visit if applicable.

- In the presence of newly identified or worsening neurological symptoms *at any given time in the study (double-blind treatment, safety follow-up, or open-label extension period)*, a neurological evaluation should be scheduled promptly. In case of events suggestive of relapse the Treating Investigator should request EDSS to be performed by the Examining Investigator*, whenever possible within 7 days of the onset of the relapse*.

Study Investigators will screen patients for signs and symptoms of PML by evaluating neurological deficits localized to the cerebral cortex, such as cortical symptoms/signs, behavioral and neuropsychological alteration, retrochiasmal visual defects, hemiparesis, cerebellar symptoms/signs (e.g., gait abnormalities, limb incoordination). A brain MRI scan and CSF analysis may be warranted to assist in the diagnosis of PML. See Section 7.3.4.1 for guidance on the diagnosis of PML.

Patients with suspected PML, defined as a new or worsening neurological symptom which necessitates MRI and or lumbar puncture and CSF analyses to rule out PML, should be withheld from study treatment until PML is ruled out by complete clinical evaluation and appropriate diagnostic testing (see Section 7.3.4.1) The Sponsor's Medical Responsible and Medical Monitor should be contacted by email. In addition Sponsor medical responsible person should be immediately contacted by phone.

A patient with confirmed PML should be withdrawn from treatment. PML should be reported as an SAE (with all available information) with immediate notification of the Medical Monitor (see also Section 7.1.1.3).

#### 5.3.4.4 Telephone Interviews

The purpose of this semi-structured interview is to identify new or worsening neurological symptoms that warrant an unscheduled visit and collect information on possible events of infections. The telephone interview will be conducted by site personnel familiar with the patient(s) every 4 weeks (± 3 days) between the study visits during the double-blind, double-dummy treatment period, OLE Phase Screening Period, OLE Phase, and Safety Follow-Up Period starting from Week 8, until 48 weeks after the

last infusion. Thereafter, for those patients who require prolonged B-cell monitoring, telephone interviews will continue every 12 weeks (±7 days) between regular visits.

The site will record in the eCRF the telephone interview as "Done" or "Not Done" and documentation of the interview will be maintained in the patient's study file.

Please refer to Appendix 4 for detailed information.

##### *5.3.4.5 Columbia-Suicide Severity Rating Scale*

The Columbia-Suicide Severity Rating Scale (C-SSRS) will be used during the double-blind, double-dummy treatment period and the OLE Phase Screening Period for prospective suicidality assessment. C-SSRS is a tool used to assess the lifetime suicidality of a patient and to track suicidal events through the treatment. The structured interview prompts recollection of suicidal ideation, including the intensity of the ideation, behavior and attempts with actual/potential lethality.

The scale will be administered by the Treating Physician or a qualified designee at the timepoints indicated in the Schedule of Assessments. The C-SSRS "*baseline*" will be collected at baseline and the C-SSRS "*since last visit*" will be collected at subsequent visits.

Please note: assessing the risk of suicide is a difficult and complex task when applied to the individual patient. Certainly, no single clinical scale can replace a thorough medical examination and suicide risk assessment. Ultimately, the determination of the presence of suicidality depends on clinical judgment.

### 5.3.5 The Karnofsky Performance Scale (Clinician-Reported Version)

The Karnofsky Performance Scale score allows patients to be classified as to their functional impairment. This scale is usually used to compare effectiveness of different therapies and to assess the prognosis in individual patients. The lower the Karnofsky score, the worse the survival for most serious illnesses.

The scale will be administered by Examining Investigator at the time points indicated in the Schedule of Assessments - Table 4.

## 5.4 Laboratory Assessments

Roche Clinical Repository biomarker samples will be shipped directly to Roche Clinical Sample Operations unit. All other lab samples collected during the study will be shipped to Central Laboratory.

The procedures for the collection, handling and shipping of laboratory samples are specified in the Laboratory Manual.

The samples for this study should be classified, packed and shipped as UN3373 Biological Substance, Category B.

Full details of the central laboratory sample handling, shipment and reporting of results will be described in the Laboratory Manual.

During the double-blind treatment period of the study, the total volume of blood loss for laboratory assessments will be approximately 341 mL over 2 years. The amount of blood taken at each visit will vary, but will be no more than 56 mL. During the OLE Phase and in the Safety Follow-Up Period, the amount of blood taken at each visit will be no more than 41 mL. Patients consenting for RCR project may have additional blood samples taken – please refer to Section 5.5.1 for more details.

### 5.4.1 Standard Laboratory Assessments

Please note: Some laboratory parameters that could reveal patient's allocation to study treatment, such as FACS cell counts, absolute neutrophil counts, Ig levels, and type I interferon neutralizing antibody levels, will be blinded. In order to ensure patients' safety in the study and to allow for assessments of the re-treatment criteria, a central laboratory will provide study Investigators and Medical Monitors with reflex messages triggered by critical blinded laboratory results. Investigators notified of their patient's critical laboratory test results will be instructed to suspend further treatment with study drug until the patient becomes eligible for re-treatment. The reflex messages from a central laboratory, together with non-blinded laboratory results, should be carefully reviewed at every visit before continuing with study treatment. The reflex messages will occur during the double-blind, double-dummy treatment period until the fifth cycle (first cycle of OLE Phase). The reflex messages will not be in effect from the sixth cycle onward. Further details will be provided in Laboratory Manual.

**Hematology:** Hemoglobin, hematocrit, red blood cells (RBC), white blood cells (WBC) (absolute and differential), absolute neutrophil count, and quantitative platelet count.

**Blood chemistry:** AST/SGOT, ALT/SGPT, GGT, alkaline phosphatase, amylase, lipase, total protein, albumin, cholesterol, total bilirubin, urea, uric acid, creatinine, random glucose, potassium, sodium, calcium, phosphorus, lactic dehydrogenase, creatine phosphokinase, and triglycerides.

**Thyroid function test:** sTSH will be tested at screening, and yearly during the double-blind, double–dummy Treatment Period. Thyroid autoantibodies will be assayed only at screening.

**FACS** will include (but is not limited to) the following cells:

- Total B cells ($CD19^{pos}$)
- Total T cell ($CD3^{pos}$)
- T helper cells ($CD3^{pos}$, $CD4^{pos}$)
- $T_{CTL}$ ($CD3^{pos}$, $CD8^{pos}$)
- NK Cells ($CD3^{neg}$, $CD16/56^{pos}$)
- B-cell subsets:
  - memory B-cells ($CD19^{pos}$, $CD27^{pos}$, $CD38^{neg}$)
  - naïve B-cells ($CD19^{pos}$, $CD27^{neg}$, $IgD^{pos}$)

– plasmablasts ($CD19^{lo}$, $CD27^{pos}$, $CD38^{hi}$,)

**Quantitative Immunoglobulin:** Ig levels (including Total Ig, IgG, IgM, and IgA isotypes).

**Antibody titers:** Measurement of antibody titers to common antigens (mumps, rubella, varicella, *S. pneumoniae*) will be performed. This information is used to assess the effect of ocrelizumab on specific humoral immunity to bacterial and viral antigens.

**HAHA:** Serum samples will be collected for determination of antibodies against ocrelizumab (HAHA; *also known as ADA*). Since ocrelizumab concentrations affect the HAHA assay, the concentration of ocrelizumab will be measured as well at all timepoints with HAHA assessment to enable interpretation of the results (PK sample). For details please refer to Schedule of Assessments.

**Pregnancy Test**: All women of childbearing potential must have regular pregnancy tests. At screening, a serum pregnancy test will be performed in central laboratory. During the *double-blind, double-dummy* study treatment period and Safety Follow-Up, a urine pregnancy test (sensitivity of at least 25 mIU/mL β-hCG) will be performed locally at the time points shown in Schedule of Assessments - Table 4. On infusion visits, the urine pregnancy test should be performed prior to the methylprednisolone infusion. A positive urine pregnancy test should be confirmed with a serum test through the central laboratory prior to any further dosing with ocrelizumab.

Please note: additional laboratory tests will be performed at screening in order to verify eligibility criteria. Please refer to Table 4 for further details.

### 5.4.2 Hepatitis Screening and Liver Function Monitoring

Patients with recurrent or chronic hepatitis B or history/presence of hepatitis C infection must be excluded from enrollment into the study (see Section 4.3). In addition, hepatitis B and C serology will be performed at screening. A positive result to either hepatitis surface antigen (HBsAg), or hepatitis B core antibody (total HBcAb) associated with positive viral DNA titres as measured by PCR, or a positive result for hepatitis C antibody (HepCAb) should result in the patient's exclusion. Patients with evidence of past resolved hepatitis B infection (i.e., positive total hepatitis B core antibody associated with a negative viral DNA) can be enrolled, and will have the hepatitis B viral DNA checked *regularly* as per Schedule of Assessment. Patients in whom the viral DNA becomes positive but in whom the quantity is at the lower limit of detection of the assay should have the test repeated as soon as possible. Patients found to have a confirmed viral DNA-positive test should be referred to a hepatologist for immediate assessment. These patients will not receive further infusions of ocrelizumab and will enter the Safety Follow-Up Period.

Liver function, i.e., ALT/SGPT, AST/SGOT, gamma glutamyl transferase (GGT), alkaline phosphatase, total bilirubin, should be reviewed throughout the study. Patients developing evidence of liver dysfunction should be assessed for viral hepatitis and, if necessary, referred to a hepatologist or other appropriately qualified expert. Study drug should be withheld until the diagnosis of viral hepatitis has been excluded. Patients developing hepatitis B or C should be withdrawn from the study and should enter

the Safety Follow-Up period. Should treatment be prescribed, this will be recorded in the eCRF. Patients with viral hepatitis due to other agents, such as hepatitis A, may resume treatment after the patient's recovery.

Please refer also to Section 6.2.2 for further guidelines on liver function monitoring.

#### 5.4.3 Plasma and Urine Banking for JC Virus

Long-term storage of plasma samples and urine is planned for JC virus DNA and/or other relevant tests for JC virus, independent of an occurrence of suspected PML case (see Section 7.3.4.1). Plasma samples (5 mL) and urine samples (10 mL) will be collected as per Schedule of Assessments. As the assay of the virus DNA has not been standardized, and a correlation between viremia and onset of PML has not been established, the JC virus assessments in plasma and urine will be performed if deemed necessary in the future and not on an ongoing basis.

#### 5.4.4 Pharmacokinetic/Pharmacodynamic Assessments

Blood samples will be collected to evaluate the pharmacokinetics and pharmacodynamics of ocrelizumab as described in the Schedule of Assessments (Table 4). The blood volume collected for pharmacokinetic assessments will be approximately 2 mL per sample. These samples will be assayed for ocrelizumab concentration using an enzyme-linked immunosorbent assay (ELISA).

Serum samples for determination of ocrelizumab concentrations will be collected at the time points detailed in the Schedule of Assessments (Table 4). On the infusion visit at week 72, two serum samples should be collected, one 5-30 minutes prior to the methylprednisolone infusion and the second one 30 minutes (±10 minutes) following the completion of the ocrelizumab infusion. For all other infusion visits, a blood sample should be taken 5 - 30 minutes before the methylprednisolone infusion. At other times (non-infusion visits), samples may be taken at any time during the visit.

For sampling procedures, storage conditions, and shipment instructions, see the Sample Handling and Logistics Manual, which will be provided to each site.

#### 5.4.5 Type I Interferon Neutralizing Antibody Assay

Type I interferon neutralizing antibody assay will be performed during the Double-Blind, Double-Dummy Treatment Period - please see Table 4 - Schedule of Assessments.

### 5.5 Roche Clinical Repository Specimen(s)

Please note: the Roche Clinical Repository research is contingent on review and approval for the exploratory biomarker assessments by an appropriate regulatory body (depending on the country where the study is performed) and a site's Institutional Review Board/ Ethics Committee. Written patient's informed consent to RCR project is also required. If a regulatory body or site's Institutional Review Board / Ethics Committee does not approve the extended analysis and long term storage of the biomarker samples, this section of protocol will not be applicable.

Specimens for dynamic (non-inherited) biomarker discovery and validation will be collected only from patients consenting to RCR.

These specimens will be used for research purposes to identify dynamic biomarkers that are predictive of response to ocrelizumab treatment (in terms of dose, safety and tolerability) and will help to better understand the pathogenesis, course and outcome of multiple sclerosis and related diseases. Specimens for dynamic biomarker discovery will be single coded like any other clinical sample (labeled and tracked using the patient's study identification number (see Section 17).

The results of specimen analysis from the RCR will facilitate the rational design of new pharmaceutical agents and the development of diagnostic tests, which may allow for individualized drug therapy for patients in the future.

All RCR specimens will be destroyed no later than 15 years after the final freeze of the respective clinical database unless regulatory authorities require that specimens be maintained for a longer period. The specimens in the RCR will be made available for future biomarker research towards further understanding of MS treatment with ocrelizumab, related diseases and adverse events and for the development of potential associated diagnostic assays. The implementation and use of the RCR specimens is governed by the Roche Clinical Repository policy to ensure the appropriate use of the RCR specimens.

#### 5.5.1 Specimen Types

Exploratory Biomarkers (non-DNA):

– **Plasma assays**

Blood (one, approximately 6 mL sample in EDTA) for plasma isolation will be obtained at various time points as shown in Schedule of Assessments (Table 4). These samples will be used for biomarker assays which may include chemokines and other candidate biomarkers in multiple sclerosis. For sampling procedures, storage conditions and shipment instructions see study Sample Handling and Logistics Manual.

– **Blood for RNA expression profiling**

Blood (2 × approximately 2.5 mL collected in PAXgene vacutainers) for RNA isolation will be obtained at various time points as shown in Schedule of Assessments (Table 4). The samples may be tested using techniques such as a micro array profiling system and/or RT PCR to study the expression profile of genes known to be involved with multiple sclerosis, and any other differentially expressed genes relative to treatment response or re-treatment. For sampling procedures, storage conditions and shipment instructions see study Sample Handling and Logistics Manual.

Exploratory Biomarkers (DNA): one sample of 6 mL of blood will be taken *in the double-blind, double-dummy treatment phase of the study* as per Schedule of Assessments (Table 4). A 6 mL whole blood sample will be obtained only from patients consenting to RCR for pharmacogenetic and genetic analysis. If not done at Baseline (Visit 2), the sample may be collected at the next visit.

For all samples, dates of specimen collection should be recorded on the associated RCR page of the eCRF and/or in the clinical database.

## 5.6 Protein Biomarker Samples

Specimens for protein biomarker discovery and validation will be collected from all patients. These specimens will be used for research purposes to identify and/or verify protein biomarkers that are predictive of response to ocrelizumab treatment (in terms of dose, safety and tolerability) and will help to understand the pathogenesis, course and outcome of relapsing MS and related diseases. Identification of patient subgroups with increased response to therapy or increased progression rates would provide information of significant clinical value to guide treatment decisions and aid in the appropriate use of the therapy. Analyses will include but are not limited to *interleukin-6*.

A 6 mL sample of whole blood will be collected in a plain tube without EDTA for serum isolation. For sampling procedures, storage conditions and shipment instructions see study Sample Handling and Logistics Manual, which will be provided to each study site.

Blood specimens for protein biomarker discovery and validation will be collected from all patients as per Schedule of Assessments. These specimens will be stored for 5 years after the end of the study and then destroyed, unless a different regulation for storage time is in place at a given site.

## 5.7 Patient Reported Outcome(s)

PRO data will be collected at the study visit with an electronic tablet device during the double-blind, double-dummy treatment period. The tablet with the PRO instruments will be distributed by the Investigator staff and completed in their entirely by the patient.

Please note: all PROs are required to be administered prior to administration of study drug and prior to any other study assessment(s) to ensure the validity of the instruments is not compromised, and data quality meet requirements of regulatory authorities and best practices [60, 61].

PRO data will be elicited from patients in this study to better characterize the clinical profile of ocrelizumab. These PRO measurements are described in Sections 5.7.1, 5.7.2, and 5.7.3. Please note that the methods for collecting and analyzing PRO data are different from those for the ascertainment of observed or volunteered adverse events. Due to these differences, PRO data will not be reported as adverse events and no attempt will be made to resolve any noticeable discrepancies between PRO data and observed or volunteered adverse events.

Patients who are unable to complete exploratory assessments (e.g., ePROs) due to physical/disease limitations will not be excluded from the study.

### 5.7.1 Modified Fatigue Impact Scale

The Modified Fatigue Impact Scale (MFIS) will assess change in the level of fatigue during the double-blind, double-dummy treatment period. The MFIS is a 21-item instrument that asks patients to rate their fatigue over the past four weeks on a 5-point Likert scale, indicating “Never” to “Almost always.” Four scores can be derived from the MFIS, including a total score as well as scores for three subscales: physical, cognitive,

and psychosocial functioning. Changes from baseline will be calculated for the total scale scores as well as for the subscale scores.

English version of MFIS is provided in Appendix 5.

### 5.7.2 The Center for Epidemiologic Studies Depression Scale

The Center for Epidemiologic Studies Depression Scale (CES-D) will be used to evaluate patients for depressive symptoms during the double-blind, double-dummy treatment period. The CES-D is a 20-item self-report instrument that asks patients to rate their feelings and behaviors over the past week on a 4-point Likert scale, from "Rarely or none of the time (less than one day)" to "Most or all of the time (5-7 days)." Only a total scale score is calculated for the CES-D.

English version of CES-D is provided in Appendix 6.

### 5.7.3 The Short Form Health Survey

The Short Form (SF-36*v2*) Health Survey is a generic quality of life instrument that has been widely tested for its psychometric properties and is widely used in clinical and epidemiological studies. The SF-36*v2* contains 36 items and measures eight health domains: vitality, physical functioning, bodily pain, general health perceptions, physical role functioning, emotional role functioning, social role functioning, and mental health. The SF-36*v2* yields a score for each domain, as well as summary scores for the physical and mental dimensions. It can be completed in 5-10 minutes.

English version of the Short Form (SF-36*v2*) Health Survey is provided in Appendix 7.

### 5.7.4 Patient's Assessment of Treatment Benefit

The Patient Perception of Treatment Questionnaire will be assessed *after* administration of PRO instruments at each visit in which PRO assessments are made, except for baseline. Specifically, patients will be asked whether they think their MS has become better, become worse or been stable since baseline. This question will serve as a global assessment from the patient perspective and will provide a useful anchor to help interpret the clinical meaningfulness of PRO results.

## 5.8 Pharmacoeconomic Assessments/EQ-5D

Pharmacoeconomic assessments will be included for purposes of deriving health utilities for economic modeling. The EQ-5D will be used to derive utilities for health states included in MS economic models and will be administered as per Schedule of Assessments.

The EQ-5D (formerly known as EuroQOL) is a generic, preference-based health-related quality of life instrument. It has five dimensions assessing mobility, self-care, usual activities, pain/discomfort and anxiety /depression. Each dimension has 3 possible levels. Different combinations of responses are utility-weighted to produce a single health utility index. The Visual Analog Scale (VAS) measures self-reported health on a scale between "worst imaginable" and "best imaginable" health states.

EQ-5D is a patient reported outcome and should be performed before any other study assessments and before administration of study drug in order to minimize bias.

## 5.9 Optional Exploratory Substudies

Patients who are randomized to the main study protocol WA21092 have the option to participate in exploratory substudies upon separate consent and fulfillment of additional exploratory substudy protocol criteria. *The Optical Coherence Tomography Exploratory Substudy (OCT) is included in the main study protocol (see Section 5.9.1 and Appendix 8). Other* exploratory substudies are *run under separate study protocols: Substudy of brain and spinal cord MRI in patients with MS participating in the OPERA clinical trial (BE29352); B cell and T cell repertoires in ocrelizumab-treated MS patients (BE29353); brain myelin mapping to quantify demyelination and repair in MS in a Phase 3 trial of ocrelizumab (BE29340); and Assessment of ocrelizumab treatment effects on disability of MS patients enrolled in the Phase 3 Orchestra program using multimodal evoked potentials (mEP) and high-resolution electroencephalogram (EEG; BE29354). Substudies will be run only at the specific assigned sites that are referred to in the substudy protocols.*

### 5.9.1 Optical Coherence Tomography Exploratory Substudy

A Roche-sponsored, multi-center, Optical Coherence Tomography (OCT) Exploratory Substudy is being conducted. OCT is a noninvasive imaging tool capable of measuring changes in structural architecture of the retina and retinal nerve fiber sensitively and rapidly [62] OCT can be of particular interest in MS, because optic neuritis is often the pivotal event in establishing the diagnosis of MS. Optic nerve dysfunction is characterized by optic disc pallor, loss of contrast sensitivity, and visual field defects and may occur subclinically in many other patients. It is estimated that nearly 20% of all patients with MS present initially with optical neuritis, and an additional 30%–100% will have optical neuritis at some point in their disease course [63]. OCT outcome measures such as RNFL thickness and macular volumes have been shown to correlate with clinical measures of vision loss and may facilitate visualization of any process of neurodegeneration or repair as part of natural history of MS or as a consequence of neuroprotective interventions [64].

The procedures and schedule of assessment are specified in the substudy protocol (Appendix 8) and in the Independent Review Committee Charter.

## 5.10 Open-Label Extension Phase

### 5.10.1 Open-Label Extension Phase Screening Period

Patients who have completed the 96-week double-blind, double-dummy treatment period, and who, in the opinion of the Treating Investigator, may benefit from treatment with ocrelizumab, will be offered the opportunity to participate in the OLE Phase of the study. Eligible patients who are not willing to participate in the OLE Phase of the study will be entered into the Safety Follow-Up Period (see below).

It should be noted that in the case of a patient who initially declines participation in the OLE Phase and subsequently reconsiders the decision, the patient will have up to 24 weeks after the Week 96 Visit to enter the OLE Phase. In this instance, he or she should not have taken any prohibited medication as specified in Section 4.5.2.1.

Patients who have consented to participate in the OLE Phase will enter an OLE Phase Screening Period to be evaluated for eligibility. The OLE Phase Screening Period will start after all assessments at the Week 96 Visit have been performed, and it should last *up to* 4 weeks. It is possible that the OLE Phase Screening Period could be longer than 4 weeks. If a prolongation of the OLE Phase Screening Period is needed, it should be discussed with the Sponsor on a case-by-case basis.

Information from assessments performed during the Week 96 Visit will be used to verify the eligibility of the patient for the OLE Phase of the study. Please refer to Section 4.4.

During the OLE Phase Screening Period, all patients should receive Rebif®/Rebif® placebo (depending on initial arm assigned to) until the first infusion of Cycle 5. Please refer to Table 7 and Table 10 for more details regarding the Rebif®/Rebif® placebo regimen.

Patients who withdraw from the OLE Phase Screening Period will also be entered into the Safety Follow-Up Period (see below).

### 5.10.2 Open-Label Extension Phase

The OLE Phase starts on the first infusion of Cycle 5 with continued dosing cycles every 24 weeks and up to the end of study (see Section 3.1.4). Patient treatment allocation during the double-blind, double-dummy treatment period will not be unblinded *until the the last data point from the last patient is received, as required for statistical analysis defined in Section 8.2 and the SAP* (see *also* Sections 3.1.3 and 6.4).

Study procedures at this visit will also include the following, but are not limited to:

- Physical examination;
- Neurological exam;
- Routine safety lab (blood and urine sampling), Hep B virus DNA (if applicable), FACS analysis, immunoglobulin levels.

The first cycle of the OLE Phase (Cycle 5) will consist of two i.v. infusions of 300 mg ocrelizumab separated by 14 days. Subsequent cycles (Cycles 6, 7, 8, etc.) will be with single i.v. infusions of 600 mg ocrelizumab to all patients enrolled in OLE Phase of the study.

Note: This initial dual infusion is designed to both optimize the initial treatment effect and to minimize any infusion-related reactions for patients initially assigned to the ocrelizumab placebo/Rebif® verum arm. In order to avoid unblinding of the assigned arm in the double-blind, double-dummy treatment period, both arms should receive dual infusion as described above.

### 5.10.3 Overview of Schedule of Assessments in the Open-Label Extension Phase

Patients participating in OLE Phase will be assessed at clinical visits every 24 weeks as per the Schedule of Assessments (see Table 4). For the description of the assessments, please refer to Section 5.3.2.

The mechanisms necessary to guarantee assessor blindness are not necessary during the OLE Phase. All required assessments during the OLE Phase should occur as described in Section 5. It is recommended that the same EDSS assessor perform the test throughout the OLE Phase.

Visits should be scheduled with reference to the date of first infusion during the OLE Phase (Cycle 5). The visit for the second infusion should be scheduled 14 days after the first infusion of Cycle 5. A minimum interval of 20 weeks should be kept between the ocrelizumab second infusion of Cycle 5 and the next infusion at Cycle 6. A minimum of 22 weeks should occur between ocrelizumab single infusions administered during Cycle 6 onward. In the unforeseen cases that an infusion is delayed, additional tests or assessments, such as routine safety laboratory tests, may be performed when the Investigator judges that these are warranted. At infusion visits, patients should remain in observation for at least 1 hour after the completion of the infusion.

In order to verify re-treatment criteria for infusions in Cycle 6 onwards, patients should attend a scheduled visit 2 weeks prior to the infusion visit.

Additional unscheduled visits for the assessment of potential MS relapses, new neurological symptoms, or safety events may occur at any time. Assessments performed at unscheduled (non-dosing) visits will depend on the clinical needs of the patient.

Patients with new neurological symptoms suggestive of MS relapse should have an EDSS performed by the Examining Investigator*, whenever possible within 7 days of the relapse onset date*. Other tests/assessments may be done as appropriate.

Please refer also to Section 7.3.4.1 for guidance on the diagnosis of PML.

#### *5.10.3.1 Delayed Dosing Visit in the Open-Label Extension Phase*

Delayed dosing visits may be scheduled if the infusion cannot be administered at the time points defined in the Schedule of Assessments - Table 6. Thus, a patient who had all assessments of a dosing visit performed, but could not receive his/her infusion, should be re-scheduled for the infusion.

At the delayed dosing visit, additional tests or assessments, such as routine safety laboratory tests, may be performed when the Investigator judges that these are warranted.

#### *5.10.3.2 Unscheduled Visits in the Open-Label Extension Phase*

Patients who develop new or worsening neurological symptoms should be seen at the investigational site as soon as possible regardless of the dates of their pre-planned, scheduled study visits. Assessments performed at unscheduled (non-dosing) visits will depend on the clinical needs of the patient.

Patients with new neurological symptoms suggestive of relapse should have an EDSS performed by the Examining Investigator, *whenever possible within 7 days of the relapse onset date*. Other tests/assessments may be done as appropriate. It should be noted if, during the *OLE screening* period, Rebif®/Rebif® Placebo dose modification is necessary in the case of ALT elevations, then unscheduled visits may be required for dispensing of study medication (please refer to Section 6.2).

Please refer also to Section 7.3.4.1 for guidance on the diagnosis of PML.

#### 5.10.3.3 Withdrawal Visits in the Open-Label Extension Phase

At the moment a patient meets one or more of the withdrawal criteria (Section 4.6), this patient is regarded as withdrawn from treatment. Patients who withdraw from ocrelizumab treatment will need to complete all assessments as shown in Schedule of Assessments - Table 6 and will be entered into the Safety Follow-Up Period.

For patients who have withdrawn from the OLE Phase or who are not eligible for treatment with ocrelizumab, it is at the discretion of the Investigator to decide on further treatment of the underlying disease. However, immunosuppressants, lymphocyte depleting therapies, and lymphocyte trafficking blockers may increase the risk of infections while patients remain B-cell depleted and therefore are not allowed during the Safety Follow-Up Period.

# 6. INVESTIGATIONAL MEDICINAL PRODUCT

During the double-blind, double-dummy treatment period, patients will be randomly assigned into one of two treatment groups:

– **Group A** – Ocrelizumab 600 mg regimen (given as dual infusions of 300 mg of ocrelizumab 14 days apart for the first 24 weeks and single infusions of 600 mg every 24 weeks thereafter)

– **Group B** – Rebif® subcutaneous injections, 3x weekly

**Table 7: Treatment Groups and Schedule of Study Medication During the Double-Blind, Double-Dummy Treatment Period**

| Treatment Group | Schedule of Study Medication |
|---|---|
| **Group A**<br>Ocrelizumab 600 mg regimen | Two i.v. infusions of ocrelizumab 300 mg separated by 14 days for the first 24 weeks, followed by single i.v. infusions of ocrelizumab 600 mg every 24 weeks thereafter<br>AND<br>placebo Rebif® subcutaneous injections, 3x weekly. |
| **Group B**<br>Rebif® | Rebif® subcutaneous injections, 3x weekly<br>AND<br>Two i.v. infusions of placebo ocrelizumab 300 mg separated by 14 days for the first 24 weeks, followed by single i.v. infusions of placebo ocrelizumab 600 mg every 24 weeks thereafter. |

The first i.v. infusion of ocrelizumab or placebo, or the first subcutaneous injection of Rebif® or placebo will be administered on study Day 1.

During the OLE Phase Screening Period, patients will continue to receive blinded treatment according to their initial assignment:

- **Group A** – Rebif® placebo s.c. injections, 3x weekly
- **Group B** – Rebif® s.c. injections, 3x weekly

During the OLE Phase, all patients will receive ocrelizumab i.v. infusions every 24 weeks. At Cycle 5, patients will receive 2 i.v. infusions of 300 mg ocrelizumab separated by 14 days during the 24-week cycle. Beginning with Cycle 6, patients will receive ocrelizumab 600 mg every 24 weeks.

Please note: 100 mg of methylprednisolone i.v. will be administered prior to each i.v. infusion of ocrelizumab/ocrelizumab placebo during the double-blind, double-dummy treatment period and prior to each i.v. infusion of ocrelizumab during the OLE Phase. In the event that the use of methylprednisolone is contraindicated for the patient, use of an equivalent dose of an alternative steroid is allowed as premedication.

## 6.1 Ocrelizumab

### 6.1.1 Preparation and Administration of Ocrelizumab Infusions

**Detailed instructions for the preparation of the infusion bags containing the study drug will be provided separately in the Dose Preparation Guidelines.**

Although ocrelizumab may be administered on an outpatient basis, patients may be hospitalized for observation at the discretion of the Investigator (in some countries this is the standard procedure). The study drug infusions should always be administered in a hospital or clinic environment under close supervision of the Investigator or a medically qualified staff member with immediate availability of full resuscitation facilities.

**Preparation of infusion**

Ocrelizumab drug product must be diluted before administration. Solutions of ocrelizumab for i.v. administration are prepared by dilution of the drug product or ocrelizumab-matching placebo into an infusion bag containing 0.9% sodium chloride, to a final drug concentration of approximately 1.16 mg/mL. Specific instructions are provided separately in the Dose Preparation Guidelines. It is important not to use evacuated glass containers (to prepare the infusion), which require vented administration sets because this causes foaming as air bubbles pass through the solution.

Prior to the start of the infusion, please ensure that the content of the bags is at room temperature to avoid an infusion reaction due to the administration of the solution at low temperatures.

**Infusion procedures**

Ocrelizumab should be given as a slow i.v. infusion. It must not be administered as an i.v. push or bolus. Well adjusted infusion pumps should be used to control the infusion rate and the study drug should be infused through a dedicated line.

All patients should receive pre-treatment before the infusion (see Section 6.1.2).

**Dual infusion cycle (Cycle 1 *and OLE Cycle* 5)**

The first cycle *of the double-blind double dummy treatment period (Cycle 1) and OLE Phase (Cycle 5)* will consist of 2 infusions of 300 mg ocrelizumab administered 14 days apart. For each infusion it is necessary to prepare a single infusion bag containing 300 mg ocrelizumab. Specific instructions will be provided separately in the Dose Preparation Guidelines and must be followed exactly. The infusion should be started at a rate of 32 mL/h. This should be escalated at the rates shown in Table 8.

**Table 8: Infusions of Ocrelizumab 300 mg**

| Time (Minutes) | Infusion Rate (mL/hr) | Maximum Dose per Interval (mg) | Cumulative Dose (mg) |
|---|---|---|---|
| 0-30 | 32 | 18.75 | 18.75 |
| 31-60 | 65 | 37.5 | 56.25 |
| 61-90 | 97 | 56.25 | 112.5 |
| 91-120 | 129 | 75 | 187.5 |
| 121-150* | 194 | 112.5 | 300* |

*Infusion of 300 mg of ocrelizumab should be completed at approximately 150 minutes (~2.5 hours).

**Single infusion cycles (Cycles 2 - 4*, and OLE Cycle 6 onwards*)**

Cycles 2 through 4 *of the double-blind, double dummy treatment period and Cycle 6 onwards of OLE Phase* will consist of one infusion of 600 mg ocrelizumab administered on Day 1 of each cycle. For each cycle it is necessary to prepare a single infusion bag containing a total of 600 mg ocrelizumab/ocrelizumab placebo. Specific instructions will be provided separately in the Dose Preparation Guidelines and must be followed exactly. The infusion should be started at a rate of 40 mL/h. This should be escalated at the rates shown in Table 9.

**Table 9: Subsequent Infusions of Ocrelizumab 600 mg**

| Time (Minutes) | Infusion Rate (mL/hr) | Maximum Dose per Interval (mg) | Cumulative Dose (mg) |
|---|---|---|---|
| 0-30 | 40 | 23.18 | 23.18 |
| 31-60 | 85 | 49.27 | 72.45 |
| 61-90 | 130 | 75.36 | 147.81 |
| 91-120 | 169 | 98.05 | 245.86 |
| 121-215* | 200 | 354.14** | 600.00 |

*The infusion of 600 mg ocrelizumab should be completed at approximately 215 minutes (~3.6 hours).
**This last interval is approximately 95 minutes, delivering a max dose of 115.94 mg per 0.5 hour.

Because of possible need to vary infusion rates depending on tolerance of the infusion, the total infusion time may exceed the time stated. UNLESS AN INFUSION REACTION OCCURS NECESSITATING DISCONTINUATION, THE ENTIRE CONTENT OF INFUSION BAG MUST BE ADMINISTERED TO THE PATIENT.

After completion of the infusion, the i.v. cannula should remain in situ for at least 1 hour in order to be able to administer drugs intravenously, if necessary in the event of a delayed reaction. If no adverse events occur during this period of time, the i.v. cannula may be removed and the patient may be discharged.

Because ocrelizumab solutions for infusion do not contain a preservative, the i.v. bags containing ocrelizumab diluted solutions should be stored refrigerated at 2-8°C. They may be stored under refrigerated conditions for up to 24 hours prior to use. Bags containing ocrelizumab diluted solutions for i.v. infusion need to be used within 48 hours of preparation (i.e., stable 24 hours at 2-8°C plus 24 hours at room temperature). As noted above, the diluted infusion bags should be at room temperature prior to administration to the patient.

#### 6.1.2 Prevention and Treatment of Infusion Related Reactions

Methylprednisolone has been shown to decrease the incidence and the severity of infusion reactions. In RA patients treated with a similar agent, rituximab, the rate and severity of infusion reactions markedly decreased with i.v. corticosteroid pre-medication [65]. To reduce potential infusion reactions, all patients will receive prophylactic treatment with 100 mg of methylprednisolone, administered by slow i.v. infusion, to be completed approximately 30 minutes before the start of each ocrelizumab infusion. In the event that the use of methylprednisolone is contraindicated, use of an equivalent dose of alternative steroid should be used as premedication prior to the infusion.

It is also recommended that the infusion is accompanied by prophylactic treatment with an analgesic/antipyretic such as acetaminophen/paracetamol (1 g) and an i.v. or oral antihistaminic (such as i.v. diphenhydramine 50 mg; or equivalent dose of alternative) 30 to 60 minutes prior to the start of an infusion to reduce potential infusion reactions. Patients administered a sedating antihistaminic for the treatment or prevention of infusion reactions should be given appropriate warnings concerning drowsiness and potential impairment of ability to drive or operate machinery.

Since transient hypotension may occur during ocrelizumab infusion, the Investigator may wish to withhold anti-hypertensive medications 12 hours prior to ocrelizumab infusion.

IRRs should be treated symptomatically with oral acetaminophen/paracetamol (1 g), and intramuscular or slow i.v. antihistamine administration, such as diphenhydramine (25 mg to 100 mg). Acetaminophen/paracetamol and diphenhydramine dosing should be repeated as clinically indicated. Non-allergic events should be treated symptomatically as judged clinically relevant by the Investigator.

**In patients with associated respiratory symptoms (stridor, wheeze or bronchospasm), additional treatment with bronchodilators may be indicated.**

One patient with well-controlled asthma at baseline experienced an acute asthma attack following their first rituximab infusion. Physicians should therefore monitor patients with a history of asthma carefully and institute an appropriate treatment if signs and symptoms of asthma are noticed.

Section 6.1.3 details the reduction, interruption or discontinuation of the infusion in the event of an infusion reaction.

### 6.1.3 Ocrelizumab Dose Modifications, Interruptions and Delays

No ocrelizumab dose modifications are foreseen.

Slowing of the infusion rate or interruption of the infusion may be necessary in the event of an infusion reaction. In rare patients, ocrelizumab treatment may need to be discontinued. Guidance is provided below.

**Handling infusion reactions:**

Handling of IRRs will depend on the intensity of symptoms (see also Section 7.1.1.1 for grading of intensity of IRRs).

In the event that a patient experiences a (CTCAE Grade 1 or 2 – Appendix 3) infusion-related event, the infusion rate should be reduced to half the rate being given at the time of onset of the event (e.g., from 50 mL/hr to 25 mL/hr or from 100 mL/hr to 50 mL/hr). Once the event has resolved, the Investigator should wait for 30 minutes while delivering the infusion at the reduced rate. If tolerated, the infusion rate may then be increased to the next closest rate on the patient’s infusion schedule and the rate increments resumed.

Patients who experience an infusion-related event (CTCAE Grade 3) or flushing, fever and throat pain cluster should have their infusion interrupted immediately and should receive aggressive symptomatic treatment. The infusion should be re-started only after all the symptoms have disappeared. The initial infusion rate at restart should be half of the infusion rate that was in progress at the time of onset of the reaction.

**Please note: patients who experience a life threatening infusion-related event** (CTCAE Grade 4) **during an infusion should have their infusion immediately stopped and should receive appropriate treatment (including use of resuscitation medications and equipment that must be available and used as clinically indicated). These patients should be withdrawn from treatment and should enter the Safety Follow-up Period.**

### 6.1.4 Criteria for Re-Treatment with Ocrelizumab

Prior to re-treatment with ocrelizumab, patients will be evaluated for the following conditions and laboratory abnormalities. If any of these are present prior to re-dosing, further administration of ocrelizumab should be suspended until resolved or held indefinitely:

- Life threatening (CTCAE Grade 4) infusion-related event that occurred during a previous ocrelizumab infusion

- Any significant or uncontrolled medical condition or treatment-emergent, clinically significant laboratory abnormality
- Active infection
- Absolute neutrophil count < $1.5 \times 10^3/\mu L$
- CD4 cell count < 250/μL
- Hypogammaglobulinemia IgG < 3.3 g/L
- Ongoing pregnancy (for female patients)

Please note: any critical blinded laboratory values for IgG, absolute neutrophil count and CD4 will be provided to the Treating Investigator and the Medical Monitor. Investigators notified of their patient's critical laboratory test result will be instructed to suspend further treatment with study drug until the patient can be further evaluated. A repeat laboratory test may be necessary to confirm the results. Patients with values below these critical values should not be retreated until the re-treatment criteria are met and these laboratory values have normalized.

## 6.2 Rebif®

### 6.2.1 Dose and Schedule of Rebif®

Please refer to Table 10 for overview of Rebif® regimen.

The first subcutaneous injection of Rebif®/placebo will be administered on Study Day 1. Patients will be instructed by a nurse or Investigator how to self-administer the injections; the first dose of Rebif®/placebo will be self-administered under the supervision of a nurse or physician. Thereafter, patients will self-administer their Rebif®/placebo treatment three times weekly. Rebif®/placebo must be administered, if possible, at the same time (preferably in the late afternoon or evening) on the same 3 days (e.g., Monday, Wednesday, and Friday) at least 48 hours apart. Patients must be instructed in the use of aseptic techniques when administering Rebif®/placebo injections. Patient understanding and use of aseptic self-injection techniques and procedures must be periodically re-evaluated.

Since Rebif® needs to be stored at 2-8°C, it is recommended to remove the syringe from refrigerator at least 30 minutes prior to use. Patient should be reminded not to heat or microwave a syringe.

When starting treatment with Rebif®, the dose will be gradually escalated (please refer to Table 10). The Rebif®/placebo initiation package corresponds to the patient needs for the first month of treatment.

**Table 10: Overview of Rebif® Dosing Regimen ***

| | Treatment Initiation | | Treatment Continuation | Dose modification (if required) |
|---|---|---|---|---|
| **Week** | **Weeks 1- 2** | **Weeks 3-4** | **Week 5 onwards** | — |
| Study Day | 1-14 | 15-28 | 29+ | At any time >Day 29 |
| **Dose of Rebif®** | **Rebif® 8.8 µg** (1 pre-filled syringe [0.2 mL] containing 2.4 MIU of interferon β-1a) s.c. **3x per week** | **Rebif® 22 µg** (1 pre-filled syringe [0.5 mL] containing 6 MIU of interferon β-1a) s.c. **3x per week** | **Rebif® 44 µg** (1 pre-filled syringe [0.5 mL] containing 12 MIU of interferon β-1a) s.c. **3x per week** | **Rebif® 22 µg** (1 pre-filled syringe [0.5 mL] containing 6 MIU of interferon β-1a) s.c. **3x per week** |

* provided in blinded fashion

Non-steroid anti-inflammatory drugs (ibuprofen) or acetaminophen are recommended in case of injection site reaction; Investigator should follow local label for further information.

Note: During the OLE Phase Screening Period, administration of Rebif® / Rebif® placebo subcutanousely 3 times per week should occur. If during this period, the patient decides not to participate in the OLE Phase then administration of Rebif® / Rebif® placebo 3 times per week should be terminated.

### 6.2.2 Rebif® Dose Modifications, Interruptions and Delays

Rebif®/ Rebif® placebo should be taken three times a week. Rebif®/ Rebif® placebo should never be taken on two consecutive days. If a patient misses a dose, then the next dose must be taken as soon as possible. The patient should avoid taking Rebif®/ Rebif® placebo on the following day. The patient should return to their regular schedule the following week. If a patient takes more than the prescribed dose or takes it on 2 consecutive days, they should inform the Investigator immediately.

The Rebif®/Rebif® placebo dosage can be modified at the discretion of the Treating Investigator due to safety reasons at any time; the Treating Investigator should follow information on the local label for further information.

Asymptomatic increases in laboratory parameters of hepatic function have been associated with Rebif®.

In case of elevation of liver function tests the following rules will apply:

| | |
|---|---|
| – **ALT ≥ 10 × ULN** OR jaundice or other clinical symptoms of liver dysfunction | In case of detection of elevated **ALT ≥ 10 × ULN**, jaundice or other clinical symptoms of liver dysfunction the injections of Rebif®/Rebif® placebo must be discontinued permanently. The monitoring of liver function tests should be continued on a monthly basis until return to normal baseline levels or CTCAE v.4.0 Grade 1 toxicity (ALT: >ULN - 3.0 × ULN). A consultation with hepatologist is recommended. Patients should move to Safety Follow-Up Period. |
| – **ALT ≥ 5 × ULN** | In case of detection of elevated **ALT ≥ 5 × ULN** (but below 10 xULN) the injections of Rebif®/Rebif® placebo must be discontinued temporarily. Additional blood chemistry panel including AST, ALP, GGT, and bilirubin should be performed biweekly until no further increase is observed. Subsequently, ALT analysis has to be performed every month until return to normal baseline levels or CTCAE v.4.0 Grade 1 toxicity (ALT >ULN - 3.0 × ULN). A consultation with hepatologist should be considered as per Investigator judgment.<br>If causes of toxicity other than possible treatment with Rebif® are excluded, the patient may then be cautiously re-challenged with Rebif®/Rebif® placebo 22µg provided in a blinded fashion upon request to IxRS. The monitoring of liver function tests should continue on a monthly basis. If there is no further recurrence of toxicity, patient may continue treatment with Rebif®/Rebif® placebo 44 µg provided in a blinded fashion upon Investigator's request to IxRS. **In case of recurrence of toxicity (ALT > 3 xULN, or other clinical symptoms of liver dysfunction) the injections of Rebif®/Rebif® placebo should be discontinued permanently.** Patients should move to Safety Follow-Up Period.<br><br><u>Please note:</u> Re-initiation of therapy with Rebif® following elevation of liver function tests can only be considered once. |
| – **ALT > 3 × ULN** | In case of detection of elevated **ALT > 3 × ULN** (but below 5 × ULN) additional blood chemistry panel including AST, ALP, GGT and bilirubin should be performed biweekly until no further increase is observed. Subsequently, ALT analysis has to be performed every month until return to normal baseline levels or CTCAE v.4.0 Grade 1 toxicity (ALT > ULN - 3.0 × ULN). |

## 6.3 Formulation, Packaging and Labeling

Study drug packaging will be overseen by the Roche clinical trial supplies department and bear a label with the identification required by local law, the protocol number, drug identification and dosage.

The packaging and labeling of the study medication will be in accordance with Roche standards and local regulations.

Upon arrival of investigational products at the site, site personnel should check them for damage and verify proper identity, quantity, integrity of seals and temperature conditions, and report any deviations or product complaints to the monitor upon discovery.

### 6.3.1 Ocrelizumab

**Formulation**

Ocrelizumab is manufactured as a sterile, clear, colorless, preservative free liquid intended for dilution for i.v. administration.

Ocrelizumab is supplied as a liquid formulation containing 30 mg/mL ocrelizumab in 20 mM sodium acetate at pH 5.3, with 4% (106 mM) trehalose dihydrate and 0.02% polysorbate 20. The drug product is provided as a single-use liquid formulation in a 15 cc Type I USP glass vial, fitted with a 20 mm fluoro-resin laminated stopper and an aluminum seal with a flip-off plastic cap and contains a nominal 200 mg ocrelizumab. No preservative is used as each vial is designed for single use.

Ocrelizumab-matching placebo is also supplied in 15 cc single-use vials. Placebo has the same composition and configuration as the drug product, but does not contain ocrelizumab.

**Packaging**

The hospital units/pharmacy will receive study medication kits for each patient.

For the double-blind treatment in Cycle 1, consisting of two 300 mg infusions 14 days apart, the study medication kit will contain 2 single-use liquid vials with ocrelizumab (or ocrelizumab placebo).

For each of the subsequent Cycles 2 to 4, consisting of a single 600 mg infusion, two kits will be dispensed. Each kit will contain 2 single-use liquid vials with ocrelizumab (or ocrelizumab placebo), from which only 3 vials should be used.

*For the OLE Phase, each study medication kit will contain 1 single-use vial. For OLE Phase Cycle 5, consisting of two 300 mg infusion 14 days apart, 2 study medication kits will be supplied per infusion. For OLE Phase Cycle 6 and each of the subsequent cycles consisting of 600 mg infusions, three study medication kits will be dispensed. Detailed instructions are provided separately in the Dose Preparation Guidelines.*

**Storage of Ocrelizumab and Placebo Vials for Infusion:**

Ocrelizumab and placebo vials are stable at 2-8°C (refrigerated storage). They should not be used beyond the expiration date stamped on the carton. Expiration dating may be

extended during the trial; the Sponsor will provide documentation. Ocrelizumab vials should not be frozen or shaken and should be protected from direct sunlight.

The study medication labels will be produced in accordance with the local requirements.

### 6.3.2 Rebif®

**Formulation and packaging**

Rebif® (IFN β-1a) will be supplied as a liquid formulation for injection in pre-filled syringes.

The liquid formulation is supplied in syringes containing 0.2 mL or 0.5 mL of solution. These commercially available syringes will be provided to the sites by the Sponsor and re-labeled as investigational medicinal product.

The placebo to Rebif® is provided as a liquid formulation in a pre-filled syringe containing 0.2 mL or 0.5 mL of 0.9% sodium chloride solution without any active substance.

The study medication kits, which will be used for the initial 4 weeks of treatment, will contain 12 pre-filled syringes, either 6x 8.8 μg and 6x 22 μg OR placebo. The study medication kits that will be used for treatment continuation will contain 12 pre-filled syringes 12x44 μg of IFN β-1a or 12x22 μg of IFN β-1a or placebo

The Rebif® and Rebif® placebo pre-filled syringes are for subcutaneous use only.

Please note: if Rebif® dose modification is required due to lab abnormalities possibly related to the treatment with Rebif®, the Investigator (the treating physician) will need to notify IxRS and the blinded study medication (Rebif® placebo or Rebif® verum) will be dispensed accordingly. In addition, to ensure patient safety in the study, unscheduled visits may be required for additional assessments, monitoring and for dispensing study medication.

The study medication labels will be produced in accordance with the local requirements. The strength will be presented as follows: 44 μg / 22 μg / placebo.

**Storage of Rebif®**

Rebif®/Rebif® placebo pre-filled syringes need to be stored in a refrigerator at 2-8°C, in the original package in order to protect from light. The patient may remove Rebif® from the refrigerator and store it not above 25°C for one single period of up to 14 days. Rebif® must then be returned to the refrigerator and used before the expiry date.

## 6.4 Blinding and Unblinding

The Patient Randomization List will be generated by IxRS using a pre-defined randomization specification. The Randomization List will not be available at the study center, to the Roche monitors, project Statisticians or to the Sponsor's project team. Unblinding of treatment assignment should not occur except in the case of emergency situations, where the knowledge of what study medication the patient is receiving is critical for clinical management. Treating Investigators are asked to contact the Roche

Medical Monitor, prior to unblinding any patient, in order to discuss the medical necessity for unblinding. Any request from the Investigator for information about the treatment administered to study patients for another purpose must be discussed with Roche. Unblinding will be performed by means of an Interactive Web Response System (IxRS). As per regulatory reporting requirement, Roche will unblind the identity of the study medication for serious adverse events that are considered by the Investigator or the Sponsor to be related to study drug, that are unexpected as per safety reference document(s), e.g., IB, CDS, and SPC, and that are not exempted from unblinding as per Section 7.2.2.2. Details of patients who are unblinded during the study will be included in the Clinical Study Report.

Unblinding for analysis of biological samples, *pharmacokinetic data analysis,* or ongoing safety monitoring by a Data Monitoring Committee [DMC], will be performed according to procedures in place to ensure integrity of the data.

*Patient treatment allocation during the double-blind, double-dummy treatment period will not be unblinded until the the last data point from the last patient is received, as required for statistical analysis defined in Section 8.2 and the SAP* (see *also* Section 3.1.3).

## 6.5 Accountability of Investigational Medicinal Product and Assessment of Compliance

### 6.5.1 Accountability of Investigational Medicinal Product

The Investigator is responsible for the control of drugs under investigation. Adequate records for the receipt and disposition of the study drug must be maintained. Accountability will be assessed by maintaining adequate drug dispensing and return records.

Accurate records must be kept for each study drug provided by the Sponsor. These records must contain the following:

- Documentation of drug shipments received from the Sponsor (date received and quantity)
- Disposition of unused study drug not dispensed to patient.

A Drug Dispensing Log must be kept current and should contain the following information:

- the identification of the patient to whom the study medication was dispensed
- the date[s] and quantity of the study medication dispensed *to* the patient
- the date[s] and quantity of the study medication returned *by* the patient.

All records and drug supplies must be available for inspection/accountability by the Monitor at every monitoring visit.

### 6.5.2 Assessment of Compliance

Patient compliance will be assessed by maintaining adequate study drug dispensing records. The Investigator is responsible for ensuring that dosing is administered in

compliance with the protocol. Delegation of this task must be clearly documented and approved by the Investigator.

The study pharmacist should keep all ocrelizumab/ocrelizumab placebo vials to measure compliance. All patients will be asked to return on regular intervals all used and unused Rebif®/Rebif® placebo containers to the site as a measure of compliance.

### 6.6 Destruction of the Investigational Medicinal Product/Comparator

Local or institutional regulations may require immediate destruction of used IMP for safety reasons. In these cases, it may be acceptable for investigational site staff to destroy dispensed IMP before a monitoring inspection provided that source document verification is performed on the remaining inventory and reconciled against the documentation of quantity shipped, dispensed, returned and destroyed. Written authorization must be obtained from the Sponsor at study start up before destruction.

Written documentation of destruction must contain the following:

- Identity (batch numbers or medication numbers) of IMP and comparators destroyed
- Quantity of IMP destroyed
- Date of destruction
- Method of destruction
- Name and signature of responsible person who destroyed the IMP.

Wherever possible, preferably drug should be destroyed locally on site according to their local policies and procedures once drug accountability has been completed by the monitor.

# 7. SAFETY INSTRUCTIONS AND GUIDANCE

## 7.1 Adverse Events and Laboratory Abnormalities

### 7.1.1 Clinical Adverse Events

According to the International Conference of Harmonisation (ICH), an AE is any untoward medical occurrence in a patient or clinical investigation patient administered a pharmaceutical product and which does not necessarily have a causal relationship with this treatment. An AE can therefore be any unfavorable and unintended sign, including an abnormal laboratory finding, symptom, or disease temporally associated with the use of a (investigational) medicinal product, whether or not considered related to the medicinal (investigational) product. Pre-existing conditions which worsen during a study are to be reported as AEs.

In the eCRF, adverse events will be reported at each visit.

**Clinical relapses** will be recorded only on a pre-specified eCRF "MS relapse" eform.

Infusion-related reactions will be recorded only on a pre-specified eCRF "Infusion Related Reaction" eform.

B-cell depletion is the expected outcome of ocrelizumab treatment and is not an adverse event. However, patients may be at risk for infections and particular attention should be directed toward early identification and treatment of infections. During the study, Investigators are requested to promptly investigate patients reporting signs or symptoms of infection, to take appropriate specimens for identification of the pathogen and to treat infections aggressively (see Section 7.3.1). Prior to enrollment into the study, it is recommended that the Investigators review and, if warranted, update patient's immunizations in accordance with country medical immunization guidelines (see also Section 4.5.3).

#### 7.1.1.1 Intensity of Clinical Adverse Events

Adverse events will be graded according to CTCAE, version 4 and is provided to the investigator in a separate handout entitled "Common Terminology Criteria for Adverse Events v4.0"- see Appendix 3.

Adverse events not listed by the CTCAE *and IRRs as a comprehensive entity* will be graded using the following criteria:

Grade 1: Discomfort noticed but no disruption of normal daily activity

Grade 2: Discomfort sufficient to reduce or affect normal daily activity

Grade 3: Inability to work or perform normal daily activity

Grade 4: Represents an immediate threat to life.

Any Grade 4 adverse event, either by CTCAE criteria or the additional criteria listed *in Section 7.1.1.3*, should be reported as an SAE.

#### 7.1.1.2 Drug - Adverse Event Relationship

**Relationship** of the adverse event to the treatment should always be assessed by the Investigator. The causality relationship of study drug to the adverse event will be assessed by the Investigator as either: Yes or No. Please refer to Appendix 1 for more details.

#### 7.1.1.3 Serious Adverse Events (Immediately Reportable to Sponsor)

A Serious Adverse Event is any experience that suggests a significant hazard, contraindication, side effect or precaution. It is any adverse event that, at any dose, fulfils at least one of the following criteria:

– is fatal; (results in **death***; please note: death is an outcome, not an event)

– is Life-Threatening (please note: the term "Life-Threatening" refers to an event in which the patient was at immediate risk of death at the time of the event; it does not refer to an event which could hypothetically have caused a death had it been more severe).

– required in-patient hospitalization or prolongation of existing hospitalization;

– results in persistent or significant disability/incapacity;

– is a congenital anomaly/birth defect;

– is medically significant or requires intervention to prevent one or other of the outcomes listed above.

***The term sudden death should only be used when the cause is of a cardiac origin as per standard definition. The terms death and sudden death are clearly distinct and must not be used interchangeably**.

The exception to this definition of a serious adverse eventis in the rare event that a patient is hospitalized following an MS relapse, as long as the reason for hospitalization is to receive standard treatment with i.v. methylprednisolone. The rationale for this exception is that some countries and/or clinical sites routinely hospitalize patients who require administration of methylprednisolone in the event of an MS relapse. Thus, the serious adverse event criteria for "hospitalization" would be met on the basis of local practice and would not reflect the seriousness of the event.

When the MS relapse results in hospitalization for any reason other than for routine treatment of the relapse (such as for a treatment course beyond the standard treatment described in (see Section 4.5.2) or when hospitalization is prolonged, the MS relapse should be considered a serious adverse event.

The study will comply with all local regulatory requirements and will adhere to the full requirements of the ICH Guideline for Clinical Safety Data Management, Definitions and Standards for Expedited Reporting, Topic E2 (see Appendix 2).

### 7.1.2 Treatment and Follow-up of Adverse Events

Adverse events should be followed up until they have stabilized or have returned to baseline status (in the event of an exacerbation of a pre-existing condition). This is especially important for those events where the reported causal relationship to study medication(s) is "related". If a clear explanation is established, it should be recorded on the eCRF.

If after study completion or withdrawal, return to baseline status or stabilization cannot be established an explanation should be recorded on the eCRF.

### 7.1.3 Laboratory Test Abnormalities

Laboratory test results will appear on electronically produced laboratory reports submitted directly from the central laboratory.

Any treatment-emergent abnormal laboratory result which is clinically significant, i.e., meeting one or more of the following conditions, should be recorded as a single diagnosis on the adverse event eform in the eCRF:

- Accompanied by clinical symptoms,
- Leading to a change in study medication (e.g., dose modification, interruption or permanent discontinuation),
- Requiring a change in concomitant therapy (e.g., addition of, interruption of, discontinuation of, or any other change in a concomitant medication, therapy or treatment).

Any laboratory result abnormality fulfilling the criteria for an SAE should be reported as such, in addition to being recorded as an AE in the eCRF.

#### *7.1.3.1 Follow-up of Abnormal Laboratory Test Values*

In the event of medically significant unexplained abnormal laboratory test values, the tests should be repeated and followed up until they have returned to the normal range and/or an adequate explanation of the abnormality is found. If a clear explanation is established it should be recorded on the eCRF.

B-cell depletion is a pharmacodynamic effect and is not an adverse event.

During the double-blind, dummy-dummy treatment period, blinded laboratory values for IgG, absolute neutrophil count and CD4 will be provided to the Investigator and the Medical Monitor. Investigators notified of their patient's critical laboratory test result will be instructed to suspend further treatment with study drug until the patient can be further evaluated. A repeat laboratory test may be necessary to confirm the results. Patients with values below these critical values should not be re-treated until the re-treatment criteria are met (see Section 6.1.4) and these laboratory values have normalized. During the OLE Phase from Cycle 6 onward, these laboratory values will not be blinded.

## 7.2 Handling of Safety Parameters

### 7.2.1 Reporting of Adverse Events

All adverse events will be documented in the eCRF.

New or worsening neurological symptoms not considered MS-related should be recorded on an AE page and the monitor should be informed.

### 7.2.2 Reporting of Serious Adverse Events

#### *7.2.2.1 Immediate Reporting to the Sponsor*

Any clinical adverse event or abnormal laboratory test value that is *serious* (as defined in Section 7.1.1.3), which occurs during the course of the study, regardless of the treatment group, must be reported to the Sponsor **within** 24 hours of the Investigator becoming aware of the event (expedited reporting). In addition, for fatal and life-threatening events, the Roche Medical Monitor should be contacted immediately. Contact numbers for the Roche Medical Monitor (including after hours cover) will be provided to the site before any patients are screened.

After the patient signs the Informed Consent, but prior to initiation of study medication, only serious adverse events caused by a protocol-mandated intervention will be collected (e.g., serious adverse events related to MRI exam). After first dose of study medication, all serious adverse events must be reported.

Related serious adverse events **MUST** be collected and reported regardless of the time elapsed from the last study drug administration, even if the study has been closed.

Unrelated serious adverse events must be collected and reported during the study through the end of the Safety Follow-up Period, which is at least 48 weeks after the last infusion but may be extended in patients whose B-cells take longer to replete.

The Investigator must complete the serious adverse event reporting form in the eCRF. Relevant follow-up information should be submitted as soon as it becomes available. Only if a technical failure prevents the ability to report a serious adverse event in the eCRF, then the paper serious adverse event Reporting Form provided by the Sponsor must be completed and faxed to the number provided.

A death occurring during the study or information related to such occurrence that comes to the attention of the Investigator during the study must be reported immediately to the Sponsor, whether considered treatment-related or not.

The following are not considered as a serious adverse event:

- Elective hospitalizations or surgical procedures that are a result of a patient's pre-existing condition(s) that have not worsened since receiving trial medication. Examples may include, but are not limited to, cholecystectomy for gallstones, and diagnostic testing. Such events should still be recorded as medical procedures in the eCRF.
- Hospitalization to receive trial medication such as infusions of ocrelizumab unless this is prolonged (more than 24 hours).
- Hospitalization following an MS relapse as long as the reason for hospitalization is to receive standard treatment with i.v. methylprednisolone

Of specific importance is the prompt reporting of serious infections. In particular, PML should be reported as a serious adverse event (with all available information) with immediate notification of the Medical Monitor.

This study adheres to the definition and reporting requirements of ICH Guideline for Clinical Safety Data Management, Definitions, and Standards for Expedited Reporting, Topic E2 (see Appendix 2).

#### 7.2.2.2 Emergency Medical Contacts

To ensure the safety of the study patients, an Emergency Medical Call Center Help Desk will access the Roche Medical Emergency List, escalate emergency medical calls, provide medical translation services (if necessary), connect the Investigator with a Roche Medical Monitor, and track all calls. The Emergency Medical Call Center Help Desk will be available 24 hours per day, 7 days per week. Toll-free numbers for the Help Desk and Medical Monitor contact information will be distributed to all Investigators (see "Protocol Administrative and Contact Information and List of Investigators").

#### 7.2.2.3 Expedited Reporting to Health Authorities, Investigators, Institutional Review Boards, and Ethics Committees

The Sponsor will promptly evaluate all reported serious adverse events against cumulative product experience to identify and expeditiously communicate possible new safety findings to Investigators, IRBs, ECs, and relevant health authorities based on applicable legislation.

Reporting requirements will be based on the Investigator's assessment of causality and seriousness, with allowance for upgrading by the Sponsor as needed. To determine

reporting requirements for single adverse event cases, the Sponsor will also assess the expectedness of the event on the basis of the Investigator's Brochure.

In principle, adverse events which are serious, related and unexpected will be reported in an expedited manner within 15 days (non-fatal/non-life-threatening) or 7 days (fatal or life-threatening).

Only those adverse events qualifying for expedited reporting occurring in patients on active treatment will be sent in an expedited timeframe to Health Authorities. This requires unblinding of patient treatment allocation. Investigators, Institutional Review Boards, and Ethics Committees will receive blinded reports unless local regulations require that unblinded expedited reports are sent.

For certain types of adverse events, the relation to study medication cannot be assessed based on single case evaluation. Therefore, in order to prevent unnecessary unblinding of study participants, the following events are exempted from expedited reporting:

- Neoplasms benign, malignant, and unspecified (including cysts and polyps),
- Infections and infestations with the exception of opportunistic infections (including PML and reactivation of viral infections).

The DMC will review adverse events at *periodic* meetings and assess their relation to study medication based on review of aggregate unblinded safety information. *The DMC will continue safety monitoring during the OLE phase.*

### 7.2.3 Pregnancy and Lactation

Female patients should take all appropriate precautions to avoid becoming pregnant during this study and for the entire duration of B-cell depletion. As such, women of childbearing potential should use adequate contraception for the duration of the trial and for 48 weeks after receiving their last infusion of ocrelizumab, or until their B-cells have repleted whichever is the longer. Regular pregnancy tests will be performed during the study. In case of an ongoing pregnancy during the study, no further infusions of ocrelizumab should be administered.

Effects on pregnancies from the female partners of B-cell depleted males have not been studied. Therefore, it is required that male patients also use reliable contraception while receiving ocrelizumab treatment in this study for 24 weeks after receiving their last infusion of ocrelizumab.

Reproductive toxicology studies of ocrelizumab conducted in cynomolgus monkeys are described in the IB. Studies of the effect of ocrelizumab on human reproduction have not been performed. It is not known whether ocrelizumab can cause fetal harm when administered to pregnant women or whether it can affect reproductive capacity. However, since IgG molecules such as ocrelizumab are known to cross the placenta, ocrelizumab may cause fetal CD20 B-cell depletion. It is not known whether ocrelizumab is excreted in breast milk, and what effect this might have on the breast feeding infant. However, since immunoglobulins are found in breast milk, breast feeding mothers are excluded from participation in the study.

Regardless of the treatment assignment, a female patient must be instructed to immediately inform the Investigator if she becomes pregnant during the study (including the Safety Follow-Up Period). In case of pregnancy, patient must be withdrawn from treatment and she should enter the Safety Follow-Up Period. The Investigator should report all pregnancies within 24 hours to the Sponsor by means of an eCRF Pregnancy Reporting Form.

As ocrelizumab may cross the placenta and cause B-cell depletion in the neonate, babies born to mothers participating in this study should have an assessment of their lymphocyte counts and be carefully followed until these are within the normal range for the age of the infant. The Investigator should counsel the patient as to the risks of continuing with the pregnancy and the possible effects on the fetus. Monitoring of the patient should continue until conclusion of the pregnancy. Informed consent will be sought for the Sponsor to collect information on the health and well being of the baby.

Whether the drug is excreted in the semen is unknown. Therefore, pregnancy occurring in the partner of a male patient participating in the study should also be reported to the Investigator, and the Investigator should inform the Sponsor. If appropriate, an additional consent form will be provided (subject to ethics committee review) to solicit information about the pregnancy.

## 7.3 Warnings and Precautions

### 7.3.1 Ocrelizumab

Patients should be informed of the risks associated with taking ocrelizumab. Below are listed specific major risks of which the patients should be made aware. Further information on ocrelizumab is given in the current version of IB.

**Infusion-Related Reactions**

All CD20 depleting agents including ocrelizumab have been associated with acute infusion-related reactions (fever, urticaria/rash, chills, rigors, sneezing, angioneurotic edema, throat irritation, nausea, fatigue, headache, dyspnea, rhinitis, vomiting, or flushing cough and bronchospasm, with or without associated hypotension or hypertension). Some of these events have been severe enough to warrant interruption or discontinuation of the infusion. Symptoms are often reversible if the infusion is interrupted and/or patients receive additional treatment with an antihistaminic, acetaminophen, epinephrine or an i.v. corticosteroid. Please refer to Sections 6.1.2 and 6.1.3 for further information.

**Infection Risks**

Prolonged peripheral B-cell depletion is the expected outcome of ocrelizumab treatment. Infection is a potentially serious complication of B-cell depleting therapy and thus requires vigilant attention and prompt investigation and treatment in patients that exhibit signs of infection at any time following anti-CD20 antibody therapy.

Data on the long term risk of infection in MS patients treated with ocrelizumab are not available at this time however in review of the Phase II data in patients with RRMS no imbalance in the overall number of infections or serious infections between placebo and active ocrelizumab arms was observed at week 24. The rate of infections did not increase

in ocrelizumab-treated patients at 96 weeks compared with 24 weeks. There was no trend of increase of risk of infection or serious infection for ocrelizumab treated patients with previous interferon treatment (Avonex® for 6 months). There was no trend of increase risk of infections or serious infections with high dose. Please see Section 1.1.4 and the ocrelizumab IB for details.

Rarely, cases of hepatitis B reactivation, including fulminant hepatitis which have occasionally been fatal, have been reported in NHL patients receiving rituximab. A case of hepatitis B reactivation in a Hep B core Ab positive patient with RA treated with ocrelizumab has been reported (see Section 5.4.2 for hepatitis screening and monitoring of liver function).

Other serious, opportunistic and fatal infections have occurred in patients with lupus and RA treated with ocrelizumab in Phase III clinical trials. Data from completed studies regarding infection risks with ocrelizumab treatment in these patient populations are provided in the Investigator Brochure (IB).

Ocrelizumab should not be administered to patients with an active infection. Physicians should exercise caution when considering the use of ocrelizumab in patients with underlying conditions that may predispose patients to serious infection. Patients who develop signs/symptoms of infection while participating in this trial should be seen immediately, samples taken for appropriate microbiological analysis and appropriate treatment instituted promptly.

Patients should be screened for tuberculosis according to national guidelines. As with other infections, patients with active tuberculosis should not be enrolled, patients with latent tuberculosis should be treated prior to enrollment.

Patients should be warned that the risk of serious infection may be increased by exposure to the medications to be used in this study and should be asked to contact the clinic staff if they start to develop signs of infection. Patients will be provided with a warning card which specifically delineates this risk, which is to be carried on their person at all times in case they are admitted to a hospital which is not participating in the study.

Please refer to the ocrelizumab IB for further information on infection risks.

**Prolonged B-cell Depletion**

In patients with RA that were treated with rituximab, prolonged peripheral B-cell depletion has been reported up to 4 years following a single course of therapy. It is not known whether this will occur following use of ocrelizumab. Patients with prolonged B-cell depletion should be monitored until their B-cells have repleted (see Section 3.1).

**Progressive multifocal leukoencephalopathy**

To date there have been no confirmed cases of PML in any MS patient treated with either rituximab or ocrelizumab. No confirmed cases of PML have been observed in any patient receiving ocrelizumab, for any indication.

Among patients treated with rituximab, cases of PML have been observed, in oncology and other autoimmune diseases. The vast majority of these cases have occurred in patients being treated for hematological malignancy.

There is no currently accepted screening test for PML, nor are there known interventions that can reliably prevent PML or adequately treat PML. See also section 7.3.4 for more details. Guidance for diagnosis is given in Section 7.3.4.1.

**Cardiovascular Disorders**

Rarely, cardiac arrhythmias, cardiac ischemia and death due to myocardial dysfunction have been associated with rituximab administration in patients with oncologic disorders. In these cases, the presumed cause was decompensated cardiac disease as a result of cytokine release and/or infusion associated reactions. Patients with a history of cardiac disease (i.e., angina pectoris, cardiac arrhythmias, or congestive heart failure) should be monitored closely during and following infusions. It should be noted that the exclusion criteria exclude enrollment of patients with significant cardiac diseases and congestive heart failure (NYHA III or IV) – see Section 4.3.

**Immunogenicity**

Positive HAHA responses were observed and were most frequent in the lower dose groups in both RA Phase I/II studies; no HAHA responses were observed in the NHL study. In Study ACT2847g, which included doses of 10 mg × 2, 50 mg × 2, 200 mg × 2, 500 mg×2, and 1000 mg × 2, HAHA was observed in 19% and 10% of patients receiving 10 mg × 2 and 50 mg × 2, respectively, versus 0% to 5% of patients receiving 200−1000 mg × 2. In Study WA18230, which included doses of 400, 1000, 1500, and 2000 mg, HAHA was observed in 10% and 5% of patients receiving 400 mg and 1000 mg, respectively, and in none of the patients receiving 1500 mg and 2000 mg.

In the RRMS Phase II study (WA21493), no new HAHA seropositivity occurred after initiation of ocrelizumab treatment (300 mg × 2 or 1000 mg × 2).

The clinical significance of positive HAHA is unknown at this time.

**Immunization**

The effect of ocrelizumab on the response to immunization is not known – please refer to Section 4.5.3 for more details; patients receiving ocrelizumab may not mount a humoral response to recall antigens during B-cell depletion. Physicians should review the patient's vaccine history, and be aware that immune response to vaccination could be reduced. Current administration of live vaccines during the Treatment Period and thereafter when B-cells remain depleted is not allowed.

### 7.3.2 Rebif®

Patients should be informed of the risks associated with taking Rebif®. The most frequent Rebif® adverse reactions of which the patients should be made aware have been summarized in Section 1.1.5.

Depression and suicide ideation are known to occur in increased frequency in the MS and in association with interferon use. Therefore all patients should be advised to

immediately report any symptoms of depression and/or suicidal ideation to Investigator. Patients exhibiting depression should be monitored closely and treated appropriately. Cessation of double-blind treatment should be considered.

Injection site necrosis has been reported in patients using Rebif®. To minimize the risk of injection site necrosis patients should be advised to:

- use an aseptic injection technique,
- rotate the injection sites with each dose.

If the patient experiences any break in the skin, which may be associated with swelling or drainage of fluid from the injection site, the patient should be advised to consult with their physician before continuing injections with Rebif®/ Rebif® placebo. If the patient has multiple lesions, injections should be discontinued until healing has occurred. Patients with single lesions may continue provided that the necrosis is not too extensive.

Rebif®, like other interferons β, has a potential for causing severe liver injury including acute hepatic failure. The mechanism for the rare symptomatic hepatic dysfunction is not known. No specific risk factors have been identified. Please refer to Section 6.2.2 for additional guidelines.

#### 7.3.3 Corticosteroids

Systemic corticosteroids, such as methylprednisolone, can cause immunosuppression, hypertension, diabetes mellitus, cataract, glaucoma, bruising, thinning of the skin, weight gain, psychological changes including psychosis, osteoporosis, accelerated atherosclerosis, increased risk of gastrointestinal bleeding, aseptic necrosis of bone and adrenal insufficiency. Although rare, corticosteroid induced hypersensitivity reactions may occur. They range from minor rashes to the more serious cardiovascular collapse. For additional safety data, refer to the local prescribing information.

#### 7.3.4 Progressive Multifocal Leukoencephalopathy

Progressive multifocal leukoencephalopathy (PML) is a potentially fatal neurological condition linked to reactivation of a polyomavirus (JC virus) and active viral replication in the brain. Polyomavirus infection is acquired in childhood and up to 80% of adults demonstrate serological evidence of past infection. Reactivation of JC virus replication with transient viremia or viruria unassociated with clinical symptoms may occur spontaneously in healthy persons. Less frequently, central nervous system symptoms associated with active viral replication in brain tissue is observed. The clinical syndrome is significantly more frequent among immune suppressed patients.

To date there have been no confirmed cases of PML in any MS patient treated with either rituximab or ocrelizumab and no confirmed cases in any patient taking ocrelizumab for any indication. Cases of PML have been reported in patients receiving rituximab in oncology and other autoimmune indications. The vast majority of these cases have occurred in patients being treated for hematological malignancy and many of these patients were also HIV positive. The majority of these patients received rituximab in combination with chemotherapy or as part of a haematopoietic stem cell transplant.

Physicians should consider the diagnosis of PML in any patient presenting with new and/or progressive neurological deficits localized to the cerebral cortex, such as cortical symptoms/signs, behavioral and neuropsychological alteration, retrochiasmal visual defects, hemiparesis, cerebellar symptoms/signs (e.g., gait abnormalities, limb incoordination), at each visit.

If PML is considered, a neurological consultation should be obtained and treatment suspended until PML has been ruled out. If PML is confirmed in a patient receiving ocrelizumab, no further infusions should be administered and the patient will be withdrawn from treatment (see Section 4.6). No known interventions can reliably prevent PML or adequately treat PML, if it occurs.

It is not known whether the risk of PML is altered by anti-CD20 treatment given as monotherapy. Please refer to Section 7.3.4.1 for guidance on the diagnosis of PML.

PML should be reported as an SAE (with all available information) with immediate notification of the Medical Monitor. Study drug should be withheld and patients with confirmed PML should *enter the safety follow-up phase of* the study.

There is no known treatment or cure for PML. Treatment considerations are discussed in the medical literature [66].

#### *7.3.4.1 Guidance for Diagnosis of Progressive Multifocal Leukoencephalopathy*

The following safety monitoring algorithm (Figure 3) will be implemented in this study. This algorithm was implemented in the Phase I/II studies with rituximab in patients with MS and is consistent with the algorithm used in natalizumab studies.

Comprehensive neurological assessments will be performed every 12 weeks at the regular study visits. Patients will be required to undergo a neurological exam for calculation of an Expanded Disability Status Scale (EDSS) score every 12 weeks. This requires that Functional System Score (FSS) also be determined. The examination to calculate the FSS includes cognitive, visual and motor assessments, the neurological systems most often affected by PML, as well as assessments of other neurological systems.

In the eCRF, the Investigator will record the presence or absence of neurological deficits localized to the cerebral cortex (e.g., cortical symptoms/signs, behavioral and neuropsychological alteration, retrochiasmal visual defects, hemiparesis), cerebellar symptoms/signs (e.g., gait abnormalities, limb incoordination), at each visit. Presence of such neurological findings will be recorded as adverse events. If a diagnosis for the deficits is identified, the symptoms should be replaced by the diagnosis in the adverse event eCRF.

In addition to the neurological evaluation at regular visits, patients will undergo a telephone interview between the study visits by site personnel familiar with the patient(s). The purpose of this interview is to identify new or worsening neurological symptoms that warrant an unscheduled visit (Appendix 4). Partners or caregivers of study patients, if

applicable, will be informed on symptoms and signs that may be suggestive of PML and should be instructed to contact the site, should any such signs or symptoms appear.

In the event that new or worsening neurological symptoms are considered during the telephone interview, a neurological evaluation will be conducted. Should a non MS etiology, such as PML, be considered, further assessments should be done. The evaluation of PML may include a brain MRI scan and CSF analysis per the proposed treatment algorithm (see Figure 3).

**The following clinical guidance is provided:**

**Treatment of Relapse and Other Neurological Symptoms**

- As in all MS studies, new or recurrent neurological symptoms occurring in study patients should prompt careful clinical evaluation.
- Given the occurrence of PML in immunocompromised patients who had received rituximab, PML should be considered in patients who develop worsening neurological signs or symptoms.
- There are no pathognomonic signs or symptoms that distinguish MS from PML, but there are certain clinical features that may help differentiate between the two conditions (see Table 12).
- In addition to PML and MS, other CNS conditions (e.g., stroke, migraine, etc.) should be considered when evaluating a patient with new neurological changes.
- Relapses should be managed according to the study protocol.
- Corticosteroid treatment should only be considered for cases in which PML is unlikely on clinical grounds and when the severity of the relapse warrants such treatment. Lack of response to corticosteroids should trigger further investigation.

**Action Steps if PML is Suspected**

- If the clinical presentation is suggestive of PML, further investigations should include brain MRI evaluation as soon as possible. If MRI evaluation reveals lesions suspicious for PML (see Figure 3) a lumbar puncture with evaluation of the cerebrospinal fluid (CSF) for the detection of JCV DNA should be undertaken. A diagnosis of PML can potentially be made by evaluating clinical and MRI findings plus the identification of JCV in the CSF.

Please note: In the event that PML is suspected, an additional plasma, urine, as well as a CSF sample should be obtained for JCV analysis. CSF samples will be analyzed upon receipt and the results will be provided directly to the investigational site and to the Sponsor. The additional plasma and urine samples will be stored together with the routine JCV samples. Storage conditions and shipment instructions will be provided.

**MRI Assessment**

- Although there are no pathognomonic findings that differentiate PML from MS, a brain MRI scan that includes fluid-attenuated inversion recovery (FLAIR) and T2-weighted and T1-weighted sequences, with and without Gd, should be performed to assess patients with neurological changes suggestive of PML – see Figure 3.

- Comparison with a baseline scan may assist with interpretation of the findings on the newly acquired MRI (see Table 12 for differences in lesion characteristics that may help differentiate between PML and MS).

### CSF Assessment

- The detection of JCV DNA in the CSF of a patient with clinical and MRI features suggestive of PML establishes the diagnosis of PML.
- If JCV DNA is not detected in CSF and if clinical suspicion of PML remains high, a repeat lumbar puncture should be performed.
- If diagnosis remains uncertain and suspicion of PML remains high, a brain biopsy may be considered to establish a definitive diagnosis.

**Figure 3: Diagnostic Algorithm for PML**

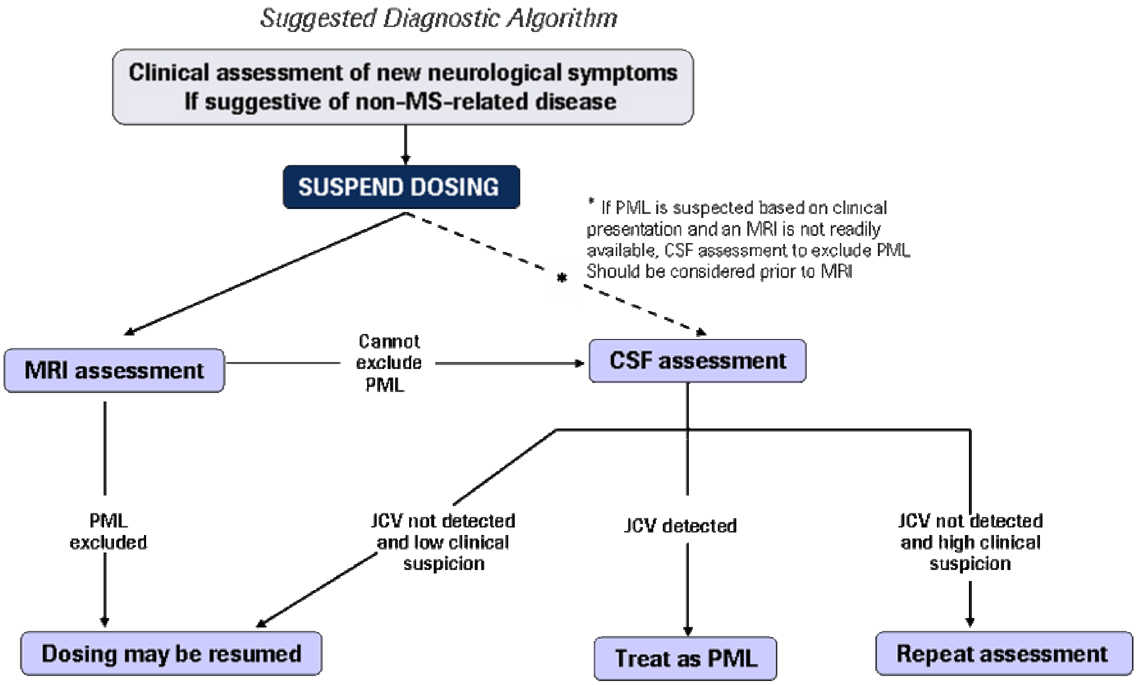

## Table 11: Clinical features to distinguish between MS relapse and PML*

| | MS relapse | PML |
|---|---|---|
| **Onset** | Acute | Subacute |
| **Evolution** | - Over hours to days<br>- Normally stabilizes<br>- Resolves spontaneously or with treatment | - Over weeks<br>- Progressive |
| **Clinical presentation** | - Optic neuritis<br>- Incomplete myelopathy or partial myelitis | - Cortical signs and symptoms<br>- Behavioral and neuropsychological alterations<br>- Retrochiasmal visual deficits<br>- Hemiparesis |

*Adapted from Kappos L et al [67]

## Table 12: MRI Lesion Characteristics Typical of PML and MS

| Feature | MS (relapse) | PML |
|---|---|---|
| **Location of new lesions** | Mostly focal; affect entire brain and spinal chord, in white and possibly gray matter | Diffuse lesions, mainly subcortical and rarely periventricular, located almost exclusively in white matter, although occasional extension to gray matter has been seen; posterior fossa frequently involved (cerebellum) |
| **Borders** | Sharp edges; mostly round or finger-like in shape (especially periventricular lesions), confluent with other lesions; U-fibers may be involved | Ill-defined edges; irregular in shape; confined to white matter; sparing gray matter; pushing against the cerebral cortex; U-fibers destroyed |
| **Mode of extension** | Initially focal; lesions enlarge within days or weeks and later decrease in size within months | Lesions are diffuse and asymmetric, extending homogeneously; no confluence with other lesions; confined to white-matter tracks, sparing the cortex; continuous progression |
| **Mass effect** | Acute lesions show some mass effect | No mass effect even in large lesions (but lesion slightly abuts cerebral cortex) |
| **On T2-weighted sequence** | - Acute lesions: hyperintense center, isointense ring, discrete hyperintensity outside the ring structure<br>- Subacute and chronic lesions: hyperintense with no ring structure | Diffuse hyperintensity, slightly increased intensity of newly involved areas compared with old areas, little irregular signal intensity of lesions |
| **On T1-weighted sequence** | Acute lesions: densely hypointense (large lesions) or isointense (small lesions); increasing signal intensity over time in 80%; decreasing signal intensity (axonal loss) in about 20% | Slightly hypointense at onset, with signal intensity decreasing over time and along the affected area; no reversion of signal intensity |

| Feature | MS (relapse) | PML |
|---|---|---|
| **On FLAIR sequence** | Hyperintense, sharply delineated | Hyperintensity more obvious; true extension of abnormality more clearly visible than in T2-weighted images |
| **With enhancement** | - Acute lesions: dense homogeneous enhancement, sharp edges<br>- Subacute lesions: ring enhancement<br>- Chronic lesions: no enhancement | Usually no enhancement, even in large lesions; in patients with HIV, some peripheral enhancement is possible, especially under therapy. |
| **Atrophy** | Focal atrophy possible due to focal white-matter degeneration; no progression | No focal atrophy |

Adapted from Yousry TA et al [68]

# 8. STATISTICAL CONSIDERATIONS AND ANALYTICAL PLAN

Full details of all statistical issues and planned statistical analyses will be specified in a separate *Statistical* Analysis Plan (*S*AP), which will be finalized prior to the locking and unblinding of the study database.

## 8.1 Study Endpoints

### 8.1.1 Primary Efficacy Endpoint

The primary efficacy endpoint is annualized protocol-defined relapse rate by two years (96 weeks).

Protocol-defined relapse is defined as the occurrence of new or worsening neurological symptoms attributable to MS. Symptoms must persist for >24 hours and should not be attributable to confounding clinical factors (e.g., fever, infection, injury, adverse reactions to medications) and immediately preceded by a stable or improving neurological state for least 30 days. The new or worsening neurological symptoms must be accompanied by objective neurological worsening consistent with an increase of at least half a step on the EDSS scale, or 2 points on one of the appropriate FSS, or 1 point on two or more of the appropriate FSS. The change must affect the selected FSS (i.e., pyramidal, ambulation, cerebellar, brainstem, sensory, or visual). Episodic spasms, sexual dysfunction, fatigue, mood change or bladder or bowel urgency or incontinence will not suffice to establish a relapse. Please note: Sexual dysfunction and Fatigue will not be scored.

Adjudication of protocol-defined relapses will be performed by the Sponsor based on pre-specified criteria, applied to data collected by Investigator, in a blinded fashion.

### 8.1.2 Secondary Efficacy Endpoints

The *key* secondary efficacy endpoints are:

- The time to onset of *confirmed* disability progression for at least 12 weeks *with the* initial event of neurological worsening occur*ring* during the 96-week, double-blind, double-dummy, treatment period (see Section 5.3.2.2 for the definition of *confirmed* disability progression).

- *The total number of T1 Gd-enhancing lesions as detected by brain MRI at Weeks 24, 48, and 96*
- The total number of new, and/or enlarging T2 hyperintense lesions as detected by brain MRI at Weeks 24, 48, and 96.
- *The proportion of patients who have confirmed disability improvement for at least 12 weeks, with the initial event of neurological improvement occurring during the 96-week double-blind, double-dummy treatment period.*
- The time to onset of *confirmed* disability progression for at least 24 weeks *with the.* initial event of neurological worsening occur*ring* during the 96-week, double-blind, double-dummy, treatment period (see Section 5.3.2.2 for the definition of *confirmed* disability progression).
- *The total number of T1-hypo-intense lesions (chronic black holes) at Weeks 24, 48, and 96*
- The change in MSFCS score from baseline to Week 96.
- The *percentage* change in brain volume as detected by brain MRI from Week 24 to Week 96.
- *The change in SF-36 PCS Score from baseline to Week 96*
- *The proportion of patients who have NEDA by Week 96*

### 8.1.3 Exploratory Efficacy Endpoints

The exploratory efficacy endpoints in this study *may* include, but may not be limited to:

- The change in low contrast visual acuity from baseline to Weeks 48 and 96.
- The change in the Symbol Digit Modalities Test from baseline to Weeks 48 and 96.
- *The proportion of relapse free patients by 96 weeks*
- *The change in total T2 hyperintense lesion volume as detected by brain MRI from baseline to Week 96*
- The annualized relapse rate, based on *all* clinical at the end of the 96-week comparative treatment period *(protocol-defined relapses are a subset of all clinical relapses).*
- *The ARR of relapses requiring IV steroid therapy*
- *The ARR of severe relapses*
- The *percentage* change in brain volume as detected by brain MRI from baseline to Week 96.
- The change in Multiple Sclerosis Functional Composite Scale (MSFCS) score from baseline to Week 48.
- The cumulative change in EDSS scores, measured in area under the curve (AUC) by Week 96.
- The change in EDSS from baseline to Week 96.
- The change in timed 25-foot walk from baseline to Week 96.
- The change in 9-hole peg test from baseline to Week 96.
- The change in paced auditory serial addition test (PASAT) from baseline to Weeks 48 and 96.
- The time to onset of sustained 20% increase in 9-hole peg test for at least 12 weeks.

- The time to onset of sustained 20% increase in timed 25 foot walk for at least 12 weeks.
- *The change in fatigue, as measured by the MFIS total score from baseline to Week 96*
- *The change from baseline in patient-reported depressive symptoms, as measured by the CES-D, from baseline to Week 96.*
- *Analyses of EQ-5D, collected at baseline, Week 48, and Week 96.*
- The change in Karnofsky Performance Status Scale from baseline to Week 96.
- *The percentage change in cortical grey matter volume from baseline to Week 96.*
- *The percentage change in white matter volume from baseline to Week 96.*
- *The proportion of patients who have disability improvement confirmed for at least 24 weeks, with the initial event of neurological improvement occurring during the 96-week double-blind double-dummy treatment period.*
- *The proportion of patients who have disability improvement sustained for at least 12 weeks and sustained until the end of the 96-week, double-blind, double-dummy treatment period, with the initial event of neurological improvement occurring during the 96-week, double-blind, double-dummy treatment period.*
- *The duration of the confirmed disability improvement.*
- *The proportion of patients who at Week 96 have improved, stable or worsened disability, compared to baseline.*
- *The change in Quality of Life, as measured by the Short Form 36 version 2 Mental Component Summary (MCS) Score from baseline to Week 96.*
- To evaluate the long-term safety, tolerability, and efficacy of ocrelizumab in patients with relapsing form of MS who are enrolled in the OLE Phase.

#### 8.1.4 Safety

Safety will be assessed through regular neurological and physical examinations, vital signs, ECG, and the occurrence of adverse events. In addition, the following will be examined:

- Non-MS pathology in all available MRI scans.
- Columbia-suicide severity rating scale (C-SSRS).
- *Standard* hematology, chemistry, and urinalysis *assessments*;
- Circulating B-cell *total and* subsets, T cells, natural killer cells and other leukocytes;
- Plasma immunoglobulins;
- HAHA;
- Antibody titers for mumps, rubella, varicella, and Streptococcus pneumoniae;
- *MS relapses classified as serious*
- Serial pregnancy tests [serum/urine β subunit human chorionic gonadotropin (β hCG)] will be performed in women of child bearing potential.
- JC virus (JCV) plasma/urine sampling only if deemed necessary.

## 8.2 Statistical and Analytical Methods

Prior to unblinding the treatment groups, a *S*AP will be produced that will contain full details of all planned analyses. An outline of the planned analyses is described below.

*After the Week 96 visit of the last patient randomised, approximately 12 weeks may be needed to allow the confirmation of the last event of the 12 week-confirmed disability progression. Therefore the clinical cut-off date will occur approximately 12 weeks after the last patient's Week 96 visit when the status is clarified for each patient. Database lock and unblinding of the Sponsor will occur several weeks after the clinical cut-off in order to clarify all outstanding queries. The sites and EDSS raters will remain blinded until approximately 24 weeks after the Week 96 visit of the last patient randomised to allow the confirmation of the last 24 week-confirmed disability progression event, in case an updated analysis of this endpoint is requested at a later point.*

The time to onset of *confirmed* disability progression for at least 12 weeks during the 96-week comparative treatment period*, the proportion of patients who have confirmed disability improvement for at least 12 weeks with the initial event of neurological improvement occurring during the 96-week double-blind, double-dummy treatment period,* and the time to onset of *confirmed* disability progression for at least 24 weeks during the 96-week comparative treatment period will be analyzed using pooled data across the two identical studies *running* as a part of the Phase III program, with respect to ocrelizumab group versus Rebif® group.

All eligible patients will be randomized to treatment stratified by region (United States versus ROW) and baseline EDSS (<4.0 versus ≥4.0). All analyses will also be stratified by region (United States versus ROW) and baseline EDSS (<4.0 versus ≥4.0).

*Some efficacy analyses will be undertaken for some subgroups, as agreed with regulatory authorities. Details of these subgroup analyses are presented in the SAP.*

All analyses, summaries and listings will be performed using SAS® software (Version *9*.2 or higher in a UNIX environment).

### 8.2.1 Primary Efficacy Analysis

The primary efficacy analysis for this trial will compare annualized protocol-defined relapse rate by 96 weeks between ocrelizumab group and Rebif® group. The annualized relapse rates by 96 weeks will be *analyzed* using negative binomial model, adjusting for region (United States versus ROW) and baseline EDSS (<4.0 versus ≥4.0). The adjusted annualized relapse rates and the two-sided 95% confidence intervals for the relapse rates will be presented along with the p-value.

Other sensitivity analyses may also be performed for the primary efficacy endpoint (and documented in the *S*AP).

### 8.2.2 Secondary Efficacy Analyses

*The statistical testing strategy for all secondary efficacy endpoints, the testing hierarchy and the rationale for this hierarchical order of secondary endpoints is fully explained in the SAP.*

Secondary efficacy endpoints will be tested in hierarchal order, all at alpha = 0.05 level. The first secondary efficacy endpoint will be tested if and only if the primary endpoint has reached the significant level at 0.05 (e.g., P-value ≤0.05). *With the exception of the three secondary efficacy endpoints which will be analyzed at the pooled level (see Section 8.2), all* secondary *efficacy* endpoints will be tested if and only if the secondary endpoint list*ed* ahead of it has reached the significance level at 0.05.

*Additional pooled analyses are also described in a separate SAP (called the pooled SAP). This document describes solely those analyses that will be performed on the two studies combined.*

#### 8.2.2.1 The Time to Onset of *Confirmed* Disability Progression for At Least 12 Weeks During the 96-Week Comparative Treatment Period

Time to *confirmed* disability progression (12 week confirmation) is defined as the time from Baseline to the first disability progression, which is confirmed at the next regularly scheduled visit ≥84 days after the initial disability progression. Disability progression is defined as an increase of ≥1.0 point from baseline EDSS, if the baseline EDSS is between 0 and 5.5 points (inclusive), or an increase of ≥ 0.5 points, if the baseline EDSS is >5.5 points. Please note that the inclusion criteria of EDSS (0−5.5) only applies to screening EDSS. It is still possible that a patient's baseline EDSS (derived based on both screening and day 1 EDSS results) is > 5.5. The non-confirmatory EDSS assessments (if any) between the initial and confirmation of disability progression should be at least as high as the minimum change required for progression. All initial disability progression events up to Week 96 with corresponding confirmation visits at the next scheduled visit (see Schedule of Assessments in Section 5) will be taken into account for the statistical analysis irrespective of whether or not the confirmation visit occurred during the treatment phase or after study drug discontinuation*, or during the OLE phase*. Thus, patients who prematurely discontinue study drug treatment should be kept in the study, and every effort should be made to follow up their EDSS status at the next scheduled visit. Patients who according to the above definition did not have *confirmed* disability progression by Week 96 visit, time of early discontinuation of treatment, or loss to follow up will be censored at the date of their last EDSS assessment.

Data from the two studies with respect to ocrelizumab group vs Rebif® group will be pooled for analysis of this endpoint. To assess the validity of pooling data across the two RMS studies, demographic and baseline characteristics will be compared by trials. The treatment effect (hazard ratio and CI) for *confirmed* disability progression within each trial will be compared between the two trials. In interpreting the trial comparisons with a view toward assessing the validity of the pooled dataset, the primary interest is in confirming that the treatment effect is qualitatively similar across the two studies – positive treatment effects (the estimated hazard to have *confirmed disability* progression in patients treated with OCR is numerically smaller than that in patients treated with Rebif) are shown in both studies. If the results from the two studies are not qualitatively similar (e.g., positive treatment effect is only shown in one study), data will not be pooled. *Further details regarding the assessment of poolability are provided in the SAP.*

Time to *confirmed* disability progression for ocrelizumab group and Rebif® group (across the studies) will be compared using a two-sided log-rank test stratifying by region (United States versus ROW), baseline EDSS (<4.0 versus ≥4.0). The proportion of patients with *confirmed* disability progression will be estimated using Kaplan-Meier methodology. The overall hazard ratio will be estimated using a stratified Cox regression model with the same stratification factors used in the stratified log-rank test above. *More details are provided in the SAP for the study.*

#### 8.2.2.2 Total Number of T1 Gadolinium-Enhanced Lesions as Detected by Brain MRI at Weeks 24, 48, and 96

*The total number of T1 gadolinium (Gd)-enhanced lesions will be calculated as the sum of the individual number of T1 Gd-enhanced lesions at Weeks 24, 48 and 96. Data from other unscheduled assessments will not be included in this summary or analysis.*

*A negative binomial model will be used to compare the difference between ocrelizumab and Rebif groups.*

#### 8.2.2.3 The Total Number of New, and/or Enlarging T2 Hyperintense Lesions as Detected by Brain Magnetic Resonance Imaging at Week 24, Week 48 and Week 96

*The same approach will be used for the statistical analysis as for the total number of T1 Gadolinium-enhanced lesions.*

#### 8.2.2.4 Proportion of Patients who have Disability Improvement Confirmed for At Least 12 Weeks

*This endpoint will be analyzed only for the subgroup of patients with a baseline EDSS score ≥2.0. Exactly the same approach to data derivation will be used for disability improvement as for disability progression (refer to Section 8.2.2.1, although note that, here, the endpoint is a binary improved/not improved variable, rather than a time-to-event endpoint). In particular, the same approach to the timing of the confirmation of disability improvement will be applied as for disability progression. The baseline EDSS score is the average of the EDSS score at the Screening Period and the score at the baseline visit, without rounding. For patients with a baseline EDSS score ≥2 and ≤5.5, disability improvement is defined as a reduction in EDSS score ≥1.0 compared to baseline EDSS score. For patients with a baseline EDSS score >5.5, disability improvement is defined as a reduction in EDSS score of 0.5. All patients without disability improvement will be counted as not improved, independent of follow-up time.*

*Data from the two studies with respect to ocrelizumab group vs Rebif® group will be pooled for analysis of this endpoint.*

*The proportions in treatment groups will be compared using the Cochran-Mantel-Haenszel (CMH) $\chi^2$ test stratified by geographical region (United States vs ROW) and baseline EDSS score (<4.0 vs ≥4.0).*

#### 8.2.2.5 The Time to Onset of *Confirmed* Disability Progression for At Least 24 Weeks During the 96-Week Comparative Treatment Period

Time to *confirmed* disability progression between ocrelizumab group and Rebif® group using a 24-week confirmation window for disability progression will be compared using the same analysis method for time to *confirmed* disability progression using a 12-week confirmation window. Time to *confirmed* disability progression (24-week confirmation) is defined as the time from Baseline (Day 1) to the first disability progression, which is confirmed at the next regularly scheduled visit ≥ 161 days after the initial disability progression. All initial disability progression events up to Week 96 with corresponding confirmation visits at the next schedule visit (see Schedule of Assessments in Section 5) will be taken into account for the statistical analysis. The same analysis principles as described in Section 8.2.2.1 will be applied to the 24-week disability endpoint.

*Data from the two studies with respect to ocrelizumab group versus Rebif® group will be pooled for analysis of this endpoint.*

#### *8.2.2.6 Total Number of T1-Hypo-Intense Lesions (Chronic Black Holes) at Weeks 24, 48, and 96*

*The same approach will be used for the statistical analysis as for the totoal number of T1 Gadolinium-enhanced lesions.*

#### 8.2.2.7 The Change in Multiple Sclerosis Functional Composite Scale (MSFCS) Score from Baseline to Weeks 96

The change in MSFCS from baseline to Week 96 will be compared between ocrelizumab group and Rebif® group *using a Mixed-Effect Model Repeated Measures (MMRM) analysis, adjusting for baseline MSFCS, region (United States versus ROW), and baseline EDSS (<4.0 versus ≥4.0).* .

#### 8.2.2.8 The *Percentage* Change in Brain Volume as Detected by Brain Magnetic Resonance Imaging Scan from Week 24 to Week 96

The change in brain volume as detected by brain MRI from week 24 to Week 96 will be compared between ocrelizumab group and Rebif® group using *an MMRM analysis. Baseline covariates here will be as follows: brain volume at Week 24, baseline Gd lesion (present or not), region (United States versus ROW), and baseline EDSS score (< 4.0 vs ≥ 4.0).*

#### *8.2.2.9 Change in Quality of Life, as Measured by the Short Form 36 version 2 Physical Component Summary (PCS) Score from Baseline to Week 96*

*The change in quality of life, as measured by the SF-36 PCS score from baseline to week 96 will be compared between ocrelizumab group and Rebif group using a MMRM analysis. Baseline covariates here will be as follows: baseline PCS score, region (United States versus ROW), and baseline EDSS (< 4.0 vs ≥ 4.0).*

#### 8.2.2.10 *Proportion of Patients Who Have No Evidence of Disease Activity (NEDA) by Week 96*

*This endpoint will be defined only for those patients with a baseline EDSS score ≥ 2.0. All available data during the 96-week treatment period will be used for the analysis. Patients who complete the 96-week treatment period will be considered as having evidence of disease activity if at least one protocol defined relapse, a CDP event having occurred or at least one MRI scan showing MRI activity (defined as Gd-enhancing T1 lesions, or new or enlarging T2 lesions) was reported during the 96-week treatment period, otherwise the patient will be considered as having no evidence of disease activity (NEDA). Patients who discontinue treatment early with at least one event before early discontinuation will be considered as having evidence of disease activity.*

*Even if an event was not reported before early discontinuation, the patient will be considered as having evidence of disease activity if the reason for early discontinuation is lack of efficacy or death; otherwise, it will be considered a missing observation.*

*The proportions within treatment groups will be compared using the Cochran-Mantel-Haenszel (CMH)* $\chi^2$ *test stratified by region (United States versus ROW) and baseline EDSS (<4.0 versus ≥4.0).*

### 8.2.3 Exploratory Analyses

The exploratory endpoints will be summarized using tables, listings and graphs, where appropriate. Full details of the derivations and analyses of exploratory endpoints will be provided in the *S*AP.

### 8.2.4 Sample Size

The sample size for this study has been estimated based on data from previous RRMS trials, with the use of two-sided tests with an experiment-wise alpha of 0.05. The annualized rate of relapse among patients receiving ocrelizumab at 96 weeks is predicted to be 0.165 (standard deviation of approximately 0.60), as compared with 0.33 (standard deviation of approximately 0.80) among patients receiving the control treatment, Rebif® (this represents a relative reduction of 50% on ocrelizumab compared to the active comparator). For the annualized relapse rate, a t-test has been used to determine the sample size between ocrelizumab and the control arm. The sample size of 400 patients per arm provides 84 percent power, maintaining the type I error rate of 0.05, and assuming a drop out rate of 20 percent approximately (assuming relative reduction among patients drop out is 25%).

For *confirmed disability* progression, a two group test of equal exponential survival with exponential dropout is used to determine the sample size. Assuming the 2-year *confirmed* disability progression rate is 18% for the Rebif® arm and 12.6% for the ocrelizumab arm (this represents a relative reduction of 30% on ocrelizumab compared to the active comparator), and assuming a drop out rate of 20 percent over 2 years approximately, the sample size of 400 per arm will provide 80 percent power, maintaining the type I error rate of 0.05 based on the pooled analysis of two RMS trials (800 patients treated with ocrelizumab 600 mg and 800 patients treated with Rebif®).

### 8.2.5 Hypothesis Testing

The hypotheses to be tested are:

$H_0$ (null hypothesis): There is no statistically significant difference in annualized protocol-defined relapse rate at 2 years between ocrelizumab group and Rebif® group.

$H_1$ (alternative hypothesis): There is a statistically significant difference in annualized protocol-defined relapse rate at 2 years between ocrelizumab group and Rebif® group.

Annualized protocol-defined relapse rate at 2 years between the ocrelizumab group and Rebif® group will be compared using negative binomial model adjusting region (United States versus ROW) and baseline EDSS (<4.0 versus ≥4.0). If the test result for comparing 600 mg ocrelizumab and Rebif® groups is statistically significant at $\alpha<0.05$ level (two-sided test), we will conclude that the 600 mg ocrelizumab group demonstrated a superior effect of reducing Annualized protocol defined relapse rate, when compared to Rebif· group.

Similar hypotheses will also be tested for the secondary efficacy parameters. Methods for handling multiplicity issues related to secondary endpoints will be described in the SAP.

### 8.2.6 Analysis Populations

One patient population will be defined for the purpose of the safety analysis and two for the efficacy analysis. All efficacy analyses will be performed using the intent-to-treat (ITT) population. The per-protocol (PP) population will be used for all primary efficacy analyses in order to evaluate the influence of major protocol violators and as a sensitivity check to the ITT analysis.

#### *8.2.6.1 Safety Population*

This population will be used for all summaries of safety data. The safety population will include all patients who received any study drug. Randomized patients that receive incorrect therapy from that intended will be summarized in the group according to the therapy actually received. Patients who are not randomized, but who receive study drug will be included in the safety population and summarized according to the therapy actually received.

#### *8.2.6.2 Intent-to-Treat Population*

All randomized patients will be included in the intent–to-treat population. Patients who prematurely withdraw from the study for any reason and for whom an assessment is not performed for whatever reason will still be included in the ITT analysis. Patients who receive an incorrect therapy from that which is intended will be summarized according to their randomized treatment.

#### *8.2.6.3 Per Protocol Population*

The per protocol population will include all patients in the ITT population adhering to the protocol. Patients may be excluded if they significantly violate the inclusion/exclusion criteria or deviate from the study plan. Specific reasons for warranting exclusion will be agreed and documented in the SAP prior to unblinding of the treatment groups. Only

those patients with violations that are deemed to potentially affect the efficacy of study treatment will be excluded from the per protocol population. Patients who receive an incorrect therapy from that intended will be excluded from the per protocol population.

#### 8.2.7 Interim Analysis

No formal efficacy interim analyses are planned.

#### 8.2.8 Safety Data Analysis

All safety parameters will be summarized and presented in tables based on *the* safety population. The safety data will be listed and summarized at determined cut off points, e.g., using data for each patient up to Week 96, using all available data at the Week 96 database lock for the primary analysis.

All adverse events will be coded and tabulated by system organ class and preferred term for individual events within each system organ class, and will be presented in descending frequency. Adverse events will also be tabulated by severity and relationship to the study medication. Serious adverse events will be summarized separately. Results of C-SSRS will be *summarized by treatment group*.

Non-MS pathology reported by local safety radiologist will be summarized by treatment group.

Associated laboratory parameters such as hepatic function, renal function and hematology values will be grouped and presented together. Correlation between low IgG and IgM and infections will be presented separately. Marked abnormalities will also be flagged. Marked abnormalities will be tabulated for each laboratory test by treatment group.

Analysis of HAHA to ocrelizumab will be summarized graphically and descriptively. Correlation between presence of HAHA and IRR/B-cell depletion will be presented descriptively.

The results of vital sign, physical examination and ECG will be included in individual patient listings. Change from baseline in vital signs will be summarized by groups.

*Type I interferon neutralizing antibody data will be summarized in listings and additional analysis will be performed as appropriate.*

An external, independent DMC will *periodically* review safety data throughout the study. *The DMC will continue safety monitoring during the OLE phase.* Analyses required for the DMC data review will be performed as described in the DMC Charter and DMC data handling plan.

#### 8.2.9 Safety Follow-up Period

Data from this period will be analyzed to provide information on the maintenance effect and the potential withdrawal effect of ocrelizumab. In addition, data will be analyzed to provide information concerning the long-term safety of ocrelizumab. Data will be summarized and tables and listings will be produced.

### 8.2.10 Open-Label Extension Phase

Data from this period of the study will be analyzed in order to characterize the long-term safety and efficacy of patients treated with ocrelizumab beyond the double-blind, double-dummy treatment period of the study. The data will be summarized according to the randomized treatment groups of the double-blind, double-dummy treatment period of the study. Details of the statistical analyses will be provided in a separate SAP.

### 8.2.11 Other Analyses

#### *8.2.11.1 Pharmacokinetic Analysis*

**Pharmacokinetic Parameters**

Ocrelizumab serum concentration-time data will be modeled using a population approach. The primary population PK parameters (Clearances and Volumes) for ocrelizumab will be estimated by means of NONMEM analysis of the sparse PK data. Clearances with associated inter-patient variability may be characterized by a saturable and non-saturable clearance as well as an intercompartmental clearance depending on the final structural model. Volumes with associated inter-patient variability may be characterized by central and peripheral volumes depending on the final structural model. Exposure (AUC) to ocrelizumab will be estimated. The selection of other parameters will depend on the final PK model used for this analysis.

**Pharmacokinetic Analysis**

Nonlinear mixed effects modeling (with software NONMEM [69]) will be used to analyze the sparse sampling dose-concentration-time data of ocrelizumab. Patients who have measurable concentrations of ocrelizumab will be included in the PK analysis unless major protocol deviations or unavailability of information (e.g., exact blood sampling time) occurred which may interfere with PK evaluation. The PK data of this study may be pooled with more extensive data from other studies. Population PK parameters (Clearances and Volumes) will be estimated and the influence of covariates, such as age, gender, weight, HAHA, and baseline CD19 lymphocytes, on these parameters will be investigated.

Details of the mixed-effects modeling analyses will be described in a Modeling and Simulation Analysis Plan and results will be reported separately.

#### *8.2.11.2 Pharmacodynamic Analysis*

The relationship between individual ocrelizumab exposure and selected safety and efficacy parameters will be analyzed and explored, in order to characterize the exposure/dose response curve of ocrelizumab. This may include but is not limited to annualized relapse rate, T1 and T2 lesions at week 96, IRRs, infections, and other AEs or safety parameters of interest. Other exploratory analyses may be performed to assess the possible relationship between PD markers e.g., CD19 count, PK, and clinical response.

#### *8.2.11.3 Roche Clinical Repository / Protein Biomarker Samples*

Additional blood samples for serum and/or plasma analyses will be taken for research purposes subject to discretionary approval from each center's IRB/IEC and the patient's specific written consent. These samples will be used to identify dynamic biomarkers to

help us better understand the pathogenesis of RMS and response to treatment with ocrelizumab. Such future biomarkers have yet to be determined but may include circulating biochemical markers in blood including cytokines as well as peripheral blood gene expression patterns. Exploratory statistical data analyses may include assessments for possible relationships between these biomarker levels, PK and clinical response.

# 9. DATA COLLECTION, MANAGEMENT AND QUALITY ASSURANCE

The overall procedures for quality assurance of clinical study data are described in the Sponsor's (or designee) Standard Operational Procedures.

Data for this study will be recorded via an Electronic Data Capture (EDC) system using electronic Case Report Forms. It will be transcribed by the site from the paper source documents onto the eCRF. In addition, EDSS, MSFC, C-SSRS, Karnofsky Performance Status Scale and patient reported outcomes will be collected via an electronic interface. The data will be transmitted from the electronic interface to a central database that will later be transferred to the Sponsor (or designee).

Accurate and reliable data collection will be assured by verification and cross–check of the eCRFs against the Investigator's records by the study monitor (source document verification), and the maintenance of a drug–dispensing log by the Investigator.

A comprehensive validation check program utilizing front-end checks in the eCRF/electronic interface and back-end checks in the data base will verify the data and discrepancies will be generated accordingly. These are transferred electronically to the site for resolution by the Investigator.

Throughout the study the Study Management Team (SMT) will review data according to the EDC Cleaning Process as described in the Data Management Plan.

## 9.1 Assignment of Preferred Terms and Original Terminology

For classification purposes, preferred terms will be assigned by the Sponsor to the original terms entered on the eCRF, using the most up-to-date version of the Medical Dictionary for Regulatory Activities (MedDRA) terminology for adverse events and diseases and the International Non-proprietary Name (INN) Drug Terms and Procedures Dictionary for treatments and surgical and medical procedures.

# 10. STUDY COMMITTEES

**Steering Committee**

An external Steering Committee will provide general guidance, assist with liaison to Investigators and oversee any external communication of the results of the study.

**Data Monitoring Committee (DMC)**

An external independent Data Monitoring Committee (DMC) will be chartered to review safety data throughout the study and make recommendations regarding continuation, termination, or modification of the study. *During the double-blind, double-dummy phase of the study,* regularly scheduled safety data reviews will occur at least three times per year after the first patient is enrolled. *The DMC will continue safety monitoring during the OLE phase.*

Any safety event that requires unblinding of study treatment allocation will be immediately reported to the DMC and to the health authorities in an expedited safety report. The DMC may request and review any additional reports outside of the planned analyses at any time if deemed necessary to ensure the safety of patients. The safety evaluations will be conducted on parameters specified within the DMC charter and may vary depending on the requirements and requests of the DMC.

The details of the DMC roles and responsibilities, scope of work and the logistics of the DMC activities will be outlined in a DMC Charter. The purpose of the DMC interim analyses is primarily safety evaluation, and the study may be stopped or amended because of significant safety concerns.

## 11. REFERENCES


1. Neurological Disorders: Public health Challenges WHO, WHO Press, 1211 Geneva 27, Switzerland, assessed on line on July 6, 2010. http://www.who.int/mental_health/neurology/chapter_3_a_neuro_disorders_public_h_challenges.pdf

2. McDonald WI, Compston A, Edan G, et al. Recommended diagnostic criteria for multiple sclerosis: guidelines from the International Panel on the Diagnosis of Multiple Sclerosis. Ann Neurol 2001;50:121–7.

3. Polman CH, Reingold SC, Banwell B, et al. Diagnostic criteria for multiple sclerosis: 2010 revisions to the "McDonald Criteria". Ann Neurol 2011;69:292–302.

4. Lublin FD and Reingold SC. Defining the clinical course of multiple sclerosis: results of an international survey. National Multiple Sclerosis Society (USA) Advisory Committee on Clinical Trials of New Agents in Multiple Sclerosis. Neurology 1996;46:907–11.

5. Filippini G, Munari L, Incorvaia B, Ebers GC, Polman C, D'Amico R, et al. Interferons in relapsing remitting multiple sclerosis: a systematic review. Lancet 2003;361:545–52.

6. Compston A, Coles A. Multiple sclerosis. Lancet 2008; 372:1502-17.

7. O'Connor P, Wolinsky JS, Confavreux C, et al. Randomized trial of oral teriflunomide for relapsing multiple sclerosis. N Engl J Med 2011;365:1293-1303.

8. Kappos, et al. The efficacy and safety of teriflunomide in patients with relapsing MS: results from TOWER, a phase III, placebo-controlled study [abstract]. ECTRIMS 2012. 2012:Abstract 153.

9. A multicenter, randomized, parallel-group, rater-blinded study comparing the effectiveness and safety of teriflunomide and interferon beta-1a in patients with relapsing multiple sclerosis. [TENERE]; Date of access 04 Mar 2013. http://clinicaltrials.gov/ct2/show/results/NCT00883337.

10. Hauser SL, Waubant E, Arnold DL, et al; HERMES Trial Group. B-cell depletion with rituximab in relapsing-remitting multiple sclerosis. N Engl J Med 2008;358:676–88.

11. Sidén A. Isoelectric focusing and crossed immunoelectrofocusing of CSF immunoglobulins in MS. J Neurol 1979;221:39–51.

12. Meinl E, Krumbholz M, Hohlfeld R. B lineage cells in the inflammatory central nervous system environment: migration, maintenance, local antibody production, and therapeutic modulation. Ann Neurol 2006;59:880–92.

13. Franciotta D, Salvetti M, Lolli F, Serafini B, Aloisi F. B cells and multiple sclerosis. Lancet Neurol 2008;7:852-8.

14. McFarland HF. The B cell--old player, new position on the team. N Engl J Med 2008;358:664–5.

15. Owens GP, Kraus H, Burgoon MP, et al. Restricted use of VH4 germline segments in an acute multiple sclerosis brain. Ann Neurol 1998;43:236–43.

16. Baranzini SE, Jeong MC, Butunoi C, Murray RS, Bernard CC, Oksenberg JR. B cell repertoire diversity and clonal expansion in multiple sclerosis brain lesions. J Immunol 1999;163:5133-44.

17. Colombo M, Dono M, Gazzola P, et al. Accumulation of clonally related B lymphocytes in the cerebrospinal fluid of multiple sclerosis patients. J Immunol 2000;164:2782-9.

18. Ritchie AM, Gilden DH, Williamson RA, et al. Comparative analysis of the CD19+ and CD138+ cell antibody repertoires in the cerebrospinal fluid of patients with multiple sclerosis. J Immunol 2004;173:649–56.

19. Lambracht-Washington D, O'Connor KC, et al. Antigen specificity of clonally expanded and receptor edited cerebrospinal fluid B cells from patients with relapsing remitting MS. J Neuroimmunol 2007;186:164–76.

20. Owens GP, Winges KM, Ritchie AM, et al. VH4 gene segments dominate the intrathecal humoral immune response in multiple sclerosis. J Immunol 2007;179:6343–51.

21. Monson NL, Brezinschek HP, Brezinschek RI, et al. Receptor revision and atypical mutational characteristics in clonally expanded B cells from the cerebrospinal fluid of recently diagnosed multiple sclerosis patients. J Neuroimmunol 2005;158:170–81.

22. Obermeier B, Mentele R, Malotka J, et al. Matching of oligoclonal immunoglobulin transcriptomes and proteomes of cerebrospinal fluid in multiple sclerosis. Nat Med 2008;14:688–93.

23. Reindl M, Linington C, Brehm U, et al. Antibodies against the myelin oligodendrocyte glycoprotein and the myelin basic protein in multiple sclerosis and other neurological diseases: a comparative study. Brain 1999;122:2047–56.

24. Egg R, Reindl M, Deisenhammer F, et al. Anti-MOG and anti-MBP antibody subclasses in multiple sclerosis. Mult Scler 2001;7:285–9.

25. Andersson M, Yu M, Söderström M, et al. Multiple MAG peptides are recognized by circulating T and B lymphocytes in polyneuropathy and multiple sclerosis. Eur J Neurol 2002;9:243–51.

26. Genain CP, Cannella B, Hauser SL, et al. Identification of autoantibodies associated with myelin damage in multiple sclerosis. Nat Med 1999;5:170–5.

27. Serafini B, Rosicarelli B, Magliozzi R, et al. Detection of ectopic B-cell follicles with germinal centers in the meninges of patients with secondary progressive multiple sclerosis. Brain Pathol 2004;14:164–74.

28. Magliozzi R, Howell O, Vora A, et al. Meningeal B-cell follicles in secondary progressive multiple sclerosis associate with early onset of disease and severe cortical pathology. Brain 2007;130:1089–104.

29. Qin Y, Duquette P, Zhang Y, et al. Clonal expansion and somatic hypermutation of V(H) genes of B cells from cerebrospinal fluid in multiple sclerosis. J Clin Invest 1998;102:1045–50.

30. Aloisi F and Pujol-Borrell R. Lymphoid neogenesis in chronic inflammatory diseases. Nat Rev Immunol 2006;6:205–17.

31. Howell OW, Reeves C, Magliozzi R, et al. The incidence of meningeal B-cell follicles in secondary progressive multiple sclerosis: a neuropathological study of 96 cases (abstract). Mult Scler 2009;15:S5.

32. Kappos L, Li D, Calabresi PA, et al. Ocrelizumab in relapsing-remitting multiple sclerosis: a phase 2, randomised, placebo-controlled, multicentre trial. Lancet 2011;378(9805):1779-87.

33. Kappos L, et al. Long-term safety and efficacy of ocrelizumab in patients with relapsing-remitting multiple sclerosis: Week 144 results of a phase II, randomised, multicentre trial [abstract]. ECTRIMS 2012. 2012:abstract 362.

34. Bar-Or A, Calabresi PA, Arnold D, et al. Rituximab in relapsing-remitting multiple sclerosis: a 72-week, open-label, phase I trial. Ann Neurol 2008;63:395-400.

35. Hawker K, O’Connor P, Freedman M, et al. Rituximab in patients with primary progressive multiple sclerosis: results of a randomized double-blind placebo-controlled multicenter trial. Ann Neurol 2009;66:460-71.

36. PRISMS Study Group. Randomized double-blind placebo controlled study of interferon-beta-1a in relapsing/remitting multiple sclerosis. Lancet 1998;352:1498–504.

37. PRISMS Study Group, University of British Columbia MS/MRI Analysis Group. PRISMS-4: long-term efficacy of interferon-beta-1a in relapsing MS. Neurology 2001;56:1628–36.

38. Panitch H, Goodin DS, Francis G, Chang P, Coyle PK, O'Connor P, Monaghan E, Li D, Weinshenker B; EVIDENCE Study Group. Evidence of Interferon Dose-response: European North American Comparative Efficacy; University of British Columbia MSMRI Research Group. Randomized, comparative study of interferon beta-1a treatment regimens in MS: The EVIDENCE Trial. Neurology 2002;59(10):1496-506.

39. Secondary Progressive Efficacy Clinical Trial of Recombinant Interferon-Beta-1a in MS (SPECTRIMS) Study Group. Randomized controlled trial of interferon- beta-1a in secondary progressive MS: Clinical results. Neurology 2001;56(11):1496-504.

40. European Public Assessments reports, Rebif Summary of product characteristic, January 27, 2010.

41. REBIF U.S. Physician Prescribing Information, Revised: July 2009.

42. Lim SY, Constantinescu CS. Current and future disease-modifying therapies in multiple sclerosis. Int J Clin Pract. 2010;64(5):637-50.

43. Polman C et al; Recommendations for clinical use of data on neutralizing antibodies to interferon-beta therapy in multiple sclerosis. Lancet Neurol 2010;9:740–50.

44. Polman CH, Reingold SC, Barkhof F, et al. Ethics of placebo-controlled clinical trials in multiple sclerosis: a reassessment. Neurology 2008;70:1134–40.

45. Rio J, Comabella M, Montalban X. Multiple sclerosis: current treatment algorithms. Curr Opin Neurol 2011:24;230-7.

46. Lindberg RL, De Groot CJ, Certa U, et al. Multiple sclerosis as a generalized CNS disease – comparative microarray analysis of normal appearing white matter and lesions in secondary progressive MS. J Neuroimmunol 2004;152:154-67.

47. Trapp, BD, Peterson J, Ransohoff RM, et al. Axonal transection in the lesions of multiple sclerosis. N Engl J Med 1998;338:278-85.

48. Freedman MS. Long-term follow-up of clinical trials of multiple sclerosis therapies. Neurology 2011:76; S26-34.

49. Scalfari A, Neuhaus A, Degenhardt A, et al. The natural history of multiple sclerosis: a geographically based study 10: relapses and long-term disability. Brain 2010: 133;1914-29.

50. Day RO, Williams KM. Open-label extension studies: do they provide meaningful information on the safety of new drugs? Drug Saf 2007;30:93-105.

51. Kap YS, van Driel N, Blezer E, et al. Late B Cell Depletion with a Human Anti-Human CD20 IgG1 κ Monoclonal Antibody halts the Development of Experimental Autoimmune Encephalomyelitis in Marmosets. J Immunol 2010: 185; 3990-4003.

52. Gong Q, Ou Q, Ye S, et al. Importance of Cellular Microenvironment and Circulatory Dynamics in B Cell Immunotherapy. J Immunol 2005:174;817-26.

53. Ahuja A, Shupe J, Dunn R, et al. Depletion of B Cells in Murine Lupus: Efficacy and Resistance. J Immunol 2007:179;3351–61.

54. Gelinck LBS, et al. Poor serological responses upon influenza vaccination in patients with rheumatoid arthritis treated with rituximab. Ann Rheum Dis 2007; 66:1402-3.

55. van Assen S, Holvast A, Benne CA, et al. Humoral responses after influenza vaccination are severely reduced in patients with rheumatoid arthritis treated with Rituximab. Arthritis & Rheumatism 2010; 62:75-81.

56. Oren S. Vaccination against influenza in rheumatoid arthritis patients: the effect of rituximab on the humoral response. Ann Rheum Dis published online November 2, 2007.

57. Bingham C, Looney R, Deodhar A, et al. Results from a controlled clinical trial (SIERRA) to evaluate primary and recall responses to immunizations in RA patients treated with rituximab. Arthritis Rheum 2008; 58:900-901[abstract#1999]. http://acr.confex.com/acr/2008/webprogram/Paper3941.html assessed on December 17, 2009

58. Cohen JA, Fischer JS, Bolibrush DM, et al. Intrarater and interrater reliability of the MS functional composite outcome measure. Neurology 2000;54:802-6.

59. Fischer JS, Rudick RA, Cutter GR, Reingold SC. The Multiple Sclerosis Functional Composite Measure (MSFC): an integrated approach to MS clinical outcome assessment. National MS Society Clinical Outcomes Assessment Task Force. Mult Scler 1999;5:244-50.

60. Guidance for Industry: Patient-reported outcome measures: Use in medical product development to support labeling claims, FDA 2009, accessed at http://www.fda.gov/downloads/Drugs/GuidanceComplianceRegulatoryInformation/Guidances/UCM193282.pdf

61. Fairclough DL, Design and analysis of quality of life studies in clinical trials. Boca Raton, FL: CRC Press; 2010.

62. Frohman EM, Fujimoto JG, Frohman TC, et al. Optical coherence tomography: a window into the mechanisms of multiple sclerosis. Nature Clinical Practice 2008;4:664-75.

63. Sergott RC, Frohman E, Glanzman R, et al. The role of optical coherence tomography in multiple sclerosis: expert panel consensus. J Neurol Sci 2007;263:3-14.

64. Costello F, Hodge W, Pan YI, et al. Tracking retinal nerve fiber layer loss after optic neuritis: a prospective study using optical coherence tomography. Multiple Sclerosis 2008;14:893-905.

65. Emery P, Fleischmann R, Filipowicz-Sosnowska A, et al. The efficacy and safety of rituximab in patients with active rheumatoid arthritis despite methotrexate treatment: results of a phase IIB randomized, double-blind, placebo-controlled, dose-ranging trial. Arthritis Rheum 2006;54:1390-400.

66. Calabrese LH, Molloy ES, Huang D, Ransohoff RM. Progressive multifocal leukoencephalopathy in rheumatic diseases: evolving clinical and pathologic patterns of disease. Arthritis Rheum 2007;56:2116-28.

67. Kappos L, Bates D, Hartung HP, et al. Natalizumab treatment for multiple sclerosis: recommendations for patient selection and monitoring. Lancet Neurol 2007;6:431-41.

68. Yousry TA, Major EO, Ryschkewitsch C, et al. Evaluation of patients treated with natalizumab for progressive multifocal leukoencephalopathy. N Engl J Med 2006;354:924-33.

69. Beal SL, Boeckman AJ, Sheiner LB. NONMEM User's Guide, Parts I-VIII San Francisco: Division of Clinical Pharmacology- University of California, 1992.

# PART II: ETHICS AND GENERAL STUDY ADMINISTRATION

## 12. ETHICAL ASPECTS

### 12.1 Local Regulations/Declaration of Helsinki

The Investigator will ensure that this study is conducted in full conformance with the principles of the "Declaration of Helsinki" or with the laws and regulations of the country in which the research is conducted, whichever affords the greater protection to the individual. The study must fully adhere to the principles outlined in "Guideline for Good Clinical Practice" ICH Tripartite Guideline or with local law if it affords greater protection to the patient. For studies conducted in the EU/EEA countries, the Investigator will ensure compliance with the EU Clinical Trial Directive [2001/20/EC]. For studies conducted in the USA or under US IND, the Investigator will additionally ensure adherence to the basic principles of "Good Clinical Practice" as outlined in the current version of 21 CFR, subchapter D, part 312, "Responsibilities of Sponsors and Investigators", part 50, "Protection of Human Subjects", and part 56, "Institutional Review Boards".

In other countries where a "Guideline for Good Clinical Practice" exists, Roche and the Investigators will strictly ensure adherence to the stated provisions.

### 12.2 Informed Consent

#### 12.2.1 Study Informed Consent

**It is the responsibility of the Investigator, or a person designated by the Investigator [if acceptable by local regulations], to obtain signed informed consent for the double-blind, double-dummy treatment period and the OLE Phase of the study from each patient prior to participating in either the double-blind, double-dummy treatment period or the OLE Phase of this study after adequate explanation of the aims, methods, anticipated benefits, and potential hazards of the study**.

The Investigator or designee must also explain that the patients are completely free to refuse to enter any period of the study or to withdraw from it at any time, for any reason.

The electronic Case Report Forms for this study contain a section for documenting patient informed consent, and this must be completed appropriately. If new safety information results in significant changes in the risk/benefit assessment, the consent form should be reviewed and updated if necessary. All patients (including those already being treated) should be informed of the new information, given a copy of the revised form and give their consent to continue in the study.

For the patient not qualified or incapable of giving legal consent, written consent must be obtained from the legally acceptable representative. In the case where both the patient and his/her legally acceptable representative are unable to read, an impartial witness should be present during the entire informed consent discussion. After the patient and representative have orally consented to participation in the trial, the witness' signature on the form will attest that the information in the consent form was accurately explained and understood.

**For U.S.-IND studies:** In a life-threatening situation where a patient is unconscious or otherwise unable to communicate, the emergency is such that there is not enough time to obtain consent from the patient's legally acceptable representative, and there is no other or better treatment available, it is permissible to treat the patient under protocol with consent of both the Investigator and another physician not involved in the study, with appropriate documentation submitted to the IRB within 5 days. If this collaboration is not immediately possible, there must be a written evaluation by a physician independent of the study and the appropriate documentation be submitted to the IRB within 5 days of treating the patient. In addition, the patient or his/her legally acceptable representative should be informed about the trial as soon as possible and consent to continue, giving written consent as described above.

**For non-U.S.-IND studies:** In a life-threatening situation where a patient is unconscious or otherwise unable to communicate, the emergency is such that there is not enough time to obtain consent from the patient's legally acceptable representative, and there is no other or better treatment available, it is permissible to treat the patient under protocol with consent of the Investigator, with appropriate documentation that the IEC had approved the procedures used to enroll patients in such situations. In addition, the patient or his/her legally acceptable representative should be informed about the trial as soon as possible and consent to continue, giving written consent as described above.

### 12.2.2 RCR Informed Consent

It is the responsibility of the Investigator, or a person designated by the Investigator (if acceptable under local regulations), to obtain written informed consent from each individual who has consented to RCR sampling after adequate explanation of the aims, methods, objectives and potential hazards. Subjects must receive an explanation that they are completely free to refuse to provide the RCR specimen(s) and may withdraw his/ her sample at any time and for any reason during the study or 15 year storage period of the specimen(s). The Informed Consent for an **optional** specimen donation will be incorporated as a specific section into the main Clinical Trial Informed Consent Form (ICF). A second, separate, specific signature consenting to specimen donation will be required to document the study participant’s agreement to provide an **optional** specimen; if the participant declines, he/ she will check a “no” box in the appropriate section and not provide a second signature.

The patient does not have to provide a separate consent for protein biomarker RCR sampling.

The eCRF for the associated clinical study contains a page for documenting patient informed consent to the RCR, and this must be completed appropriately.

### 12.2.3 Death or Loss of Competence of Participant who has donated a specimen(s) that is stored in the RCR

In case the Informed Consent Form and/or the Study Protocol do not provide any specific provisions for death or loss of competence, specimen and data will continue to be used as part of RCR research.

In the event of the death of a participant of a Roche Clinical Trial or Experimental Medicine Research study or if a participant is legally incompetent at the time of the specimen and data procurement, or becomes legally incompetent thereafter, applicable provisions as stated for such situations in the respective Informed Consent Form and/or the Study Protocol shall become effective and be followed accordingly.

Additional procurement of assent from legally incompetent persons and minors shall take place according to local laws and international best practice, as it applies to the specific case.

## 12.3 Independent Ethics Committees (IEC) and Institutional Review Board (IRB)

The protocol, informed consent and any accompanying material provided to the patient in the U.S. will be submitted by the Investigator to an IRB for review. For EEA member states, the Sponsor will submit to the Competent Authority and IEC, the protocol and any accompanying material provided to the patient. In both the US and EEA member states, the accompanying material may include patient information sheets, descriptions of the study used to obtain informed consent and terms of any compensation given to the patient as well as advertisements for the trial.

An approval letter or certificate (specifying the protocol number and title) from the IEC/IRB must be obtained before study initiation by the Investigator specifying the date on which the committee met and granted the approval. This applies whenever subsequent amendments/modifications are made to the protocol.

Any modifications made to the protocol, informed consent or material provided to the patient after receipt of the IEC/IRB approval must also be submitted by the Investigator in the U.S. and by the Sponsor in the EEA member states in accordance with local procedures and regulatory requirements.

When no local review board exists, the Investigator is expected to submit the protocol to a regional committee. If no regional committee exists, Roche will assist the Investigator in submitting the protocol to the European Ethics Review Committee.

Sampling for the RCR is contingent on review and approval for the exploratory biomarker assessments and written informed consent by an appropriate regulatory body (depending on the country where the study is performed) and a site's Institutional Review Board (IRB) / Ethics Committee (EC). If a regulatory or site's IRB/EC does not approve the sampling for the exploratory assessments the section on biomarker sampling will not be applicable.

Roche shall also submit an Annual Safety Report once a year to the IEC and Competent Authorities (CAs) according to local regulatory requirements and timelines of each country participating in the study. In the U.S. Roche submits an IND Annual Report to the FDA according to local regulatory requirements and timelines.

## 12.4 Role of the Science and Ethics Advisory Group (SEAG)

A Science and Ethics Advisory Group consisting of experts in the fields of biology, ethics, sociology and law will advise Roche regarding the use of specimens stored in the RCR and on the scientific and ethical aspects of handling genetic information. The SEAG is independent of Roche.

# 13. CONDITIONS FOR MODIFYING THE PROTOCOL

Requests from Investigators to modify the protocol to ongoing studies will be considered only by consultation between an appropriate representative of the Sponsor and the Investigator [Investigator representative(s) in the case of a multicenter trial]. Protocol modifications must be prepared by a representative of the Sponsor and initially reviewed and approved by the Clinical Science Leader and Biostatistician.

All protocol modifications must be submitted to the appropriate Independent Ethics Committee or Institutional Review Board for information and approval in accordance with local requirements, and to Regulatory Agencies if required. Approval must be obtained before any changes can be implemented, except for changes necessary to eliminate an immediate hazard to trial patients, or when the change(s) involves only logistical or administrative aspects of the trial (e.g., change in monitor[s], change of telephone number[s]).

# 14. CONDITIONS FOR TERMINATING THE STUDY

Both the Sponsor and the Investigator reserve the right to terminate the study at any time. Should this be necessary, both parties will arrange the procedures on an individual study basis after review and consultation. In terminating the study, Roche and the Investigator will assure that adequate consideration is given to the protection of the patients' interests. The appropriate IRB/EC and Regulatory Agencies should be informed accordingly.

# 15. STUDY DOCUMENTATION, CRFS AND RECORD KEEPING

## 15.1 Investigator's Files / Retention of Documents

The Investigator must maintain adequate and accurate records to enable the conduct of the study to be fully documented and the study data to be subsequently verified. These documents should be classified into two different separate categories [1] Investigator's Study File, and [2] patient clinical source documents.

The Investigator's Study File will contain the protocol/amendments, eCRF and schedule of assessments, Independent Ethics Committee/Institutional Review Board and governmental approval with correspondence, sample informed consent, drug records, staff curriculum vitae and authorization forms and other appropriate documents/correspondence, etc. In addition at the end of the study the Investigator will receive the patient data, which includes an audit trail containing a complete record of all changes to data, query resolution correspondence and reasons for changes, in human readable format on CD which also has to be kept with the Investigator's Study File.

Subject clinical source documents (usually defined by the project in advance to record key efficacy/safety parameters independent of the eCRFs) would include patient hospital/clinic records, physician's and nurse's notes, appointment book, original

laboratory reports, ECG, EEG, X-ray, pathology and special assessment reports, signed informed consent forms, consultant letters, and patient screening and enrollment logs. The Investigator must keep the two categories of documents as described above (including the archival CD) on file for at least 15 years after completion or discontinuation of the study. After that period of time the documents may be destroyed, subject to local regulations.

Should the Investigator wish to assign the study records to another party or move them to another location, Roche must be notified in advance.

If the Investigator can not guarantee this archiving requirement at the investigational site for any or all of the documents, special arrangements must be made between the Investigator and Roche to store these in a sealed container(s) outside of the site so that they can be returned sealed to the Investigator in case of a regulatory audit. Where source documents are required for the continued care of the patient, appropriate copies should be made for storing outside of the site.

ICH GCP guidelines require that Investigators maintain information in the study patient's records which corroborate data collected on the eCRF(s). Completed eCRF will be transferred to Sponsor.

## 15.2 Source Documents and Background Data

The Investigator shall supply the Sponsor on request with any required background data from the study documentation or clinic records. This is particularly important when errors in data transcription are suspected. In case of special problems and/or governmental queries or requests for audit inspections, it is also necessary to have access to the complete study records, provided that patient confidentiality is protected.

## 15.3 Audits and Inspections

The Investigator should understand that source documents for this trial should be made available to appropriately qualified personnel from the Roche Pharma Development Quality Assurance Unit or its designees, or to health authority inspectors after appropriate notification. The verification of the eCRF data must be by direct inspection of source documents.

## 15.4 Electronic Case Report Forms

Data for this study will be captured via an Electronic Data Capture (EDC) system by using eCRFs. An audit trail will maintain a record of initial entries and changes made; reasons for change; time and date of entry; and user name of person authorizing entry or change. The Investigator must update eCRF and connect on a regular basis.

For each patient enrolled, an eCRF must be completed and electronically signed by the principal Investigator or authorized delegate from the study staff. This also applies to records for those patients who fail to complete the study (even during a pre-randomization screening period if an eCRF was initiated). If a patient withdraws from the study, the reason must be noted on the eCRF. If a patient is withdrawn from the study because of a treatment-limiting AE, thorough efforts should be made to clearly document the outcome.

The Investigator should ensure the accuracy, completeness and timeliness of the data reported to the Sponsor in the eCRFs and in all required reports.

## 15.5 Financial Disclosure

The Investigator(s) will provide the Sponsor with sufficient accurate financial information (PD35) to allow the Sponsor to submit complete and accurate financial certification or disclosure statements to the appropriate regulatory authorities. The Investigator is responsible to promptly update any information provided to the Sponsor if relevant changes occur in the course of the investigation and for 1 year following the completion of the study (last patient, last visit).

# 16. MONITORING THE STUDY

It is understood that the responsible Roche monitor [or designee] will contact and visit the Investigator regularly and will be allowed, on request, to inspect the various records of the trial (eCRFs and other pertinent data) provided that patient confidentiality is maintained in accord with local requirements.

It will be the monitor's responsibility to inspect the eCRFs at regular intervals throughout the study, to verify the adherence to the protocol and the completeness, consistency and accuracy of the data being entered on them. The monitor must verify that the patient received the study drug assigned by the randomization center (by controlling the written confirmation of the randomization by IxRS). The monitor should have access to laboratory test reports and other patient records needed to verify the entries in the eCRF. The Investigator (or deputy) agrees to cooperate with the monitor to ensure that any problems detected in the course of these monitoring visits are resolved.

Roche Clinical Repository specimens will at all times be tracked in a manner consistent with Good Clinical Practice, by a quality controlled, auditable and validated Laboratory Information Management System, to ensure compliance with data confidentiality as well as adherence to authorized use of specimens as specified in the study protocol and ICF, respectively. Roche monitors and auditors will have direct access to appropriate parts of records relating to patients participating in this study for the purposes of verifying the data provided to Roche. The site will permit monitoring, audits, Institutional Review Board/Independent Ethics Committee (IRB/IEC) review, and regulatory inspections by providing direct access to source data and documents related to the RCR Research Project.

# 17. CONFIDENTIALITY OF TRIAL DOCUMENTS AND SUBJECT RECORDS

The Investigator must assure that patients' anonymity will be maintained and that their identities are protected from unauthorized parties. On CRFs or other documents submitted to the Sponsor, patients should not be identified by their names, but by an identification code. The Investigator should keep a patient enrollment log showing codes, names and addresses. The Investigator should maintain documents not for submission to Roche, e.g., patients' written consent forms, in strict confidence.

Roche already maintains rigorous confidentiality standards for clinical studies by "coding" (i.e., assigning a unique patient ID number at the Investigator site) all patients enrolled in Roche clinical studies. This means that patient names are not included in data sets that are transmitted to any Roche location. Given the sensitive nature of genetic data, Roche has implemented a number of additional processes to assure patient confidentiality. All specimens taken for inherited genetic research that will be stored in the RCR (see Section 5.5) undergo a second level of "coding". At Roche, the specimen is transferred to a new tube and labeled with a new random number. This is referred to as "Double Coding (De-Identification)". Data generated following the use of these specimens and all clinical data transferred from the clinical study database and considered relevant, will also be labeled with this same code. The "linking key" between the participant's identification number and this new independent code will be stored in a secure database system. Access to the table linking the participant identification number to the specimen code will be strictly limited and monitored by audit trail. Legitimate operational reasons for accessing the "linking key" will be documented in a standard operating procedure. Access to the "linking key" for any other reason will require written approval from the Governance Committee responsible for the specimen(s).

# 18. CLINICAL STUDY REPORT (CSR)

A clinical study report will be written and distributed to Health Authorities as required by applicable regulatory requirements.

# 19. PUBLICATION OF DATA AND PROTECTION OF TRADE SECRETS

Roche will comply with the requirements for publication of study results.

The results of this study may be published or presented at scientific meetings. If this is foreseen, the Investigator agrees to submit all manuscripts or abstracts to Roche prior to submission. This allows the Sponsor to protect proprietary information and to provide comments based on information from other studies that may not yet be available to the Investigator.

In accordance with standard editorial and ethical practice, Roche will generally support publication of multicenter trials only in their entirety and not as individual center data. In this case, a coordinating Investigator will be designated by mutual agreement.

Authorship will be determined by mutual agreement and in line with International Committee of Medical Journal Editors (ICMJE) authorship requirements. Any formal publication of the study in which contribution of Roche personnel exceeded that of conventional monitoring will be considered as a joint publication by the Investigator and the appropriate Roche personnel.

Data derived from RCR specimen analysis on individual patients will not be provided to study Investigators, except where explicitly stipulated in a study protocol (e.g., if the result is an enrollment criterion). Exceptions may be granted (e.g., if biomarker data would be linked to safety issues). The aggregate results of any research conducted using RCR specimens will be available in accordance with the effective Roche policy on study data publication.

Any inventions and resulting patents, improvements and / or know- how originating from the use of the RCR will become and remain the exclusive and unburdened property of Roche, except where agreed otherwise.

## Appendix 1: AEs Categories for Determining Relationship to Test Drug

The causality relationship of study drug to the adverse event will be assessed by the Investigator as either: Yes or No.

If there is a reasonable suspected causal relationship to the study medication, i.e., there are facts (evidence) or arguments to suggest a causal relationship, drug-event relationship should be assessed as Yes.

**The following criteria should be considered in order to assess the relationship as Yes:**

– Reasonable temporal association with drug administration

– It may or may not have been produced by the subject's clinical state, environmental or toxic factors, or other modes of therapy administered to the subject.

– Known response pattern to suspected drug

– Disappears or decreases on cessation or reduction in dose

– Reappears on rechallenge

**The following criteria should be considered in order to assess the relationship as No:**

– It does not follow a reasonable temporal sequence from administration of the drug.

– It may readily have been produced by the subject's clinical state, environmentalor toxic factors, or other modes of therapy administered to the subject.

– It does not follow a known pattern of response to the suspected drug.

– It does not reappear or worsen when the drug is readministered.

## Appendix 2: ICH Guidelines for Clinical Safety Data Management, Definitions and Standards for Expedited Reporting, Topic E2

A serious adverse event is any experience that suggests a significant hazard, contraindication, side effect or precaution. It is any AE that at any dose fulfills at least one of the following criteria:

- is fatal; [results in death] [**NOTE**: death is an outcome, not an event]
- is Life-Threatening [**NOTE**: the term "Life-Threatening" refers to an event in which the patient was at immediate risk of death at the time of the event; it does not refer to an event which could hypothetically have caused a death had it been more severe]
- requires in-patient hospitalization or prolongation of existing hospitalization
- results in persistent or significant disability/incapacity
- is a congenital anomaly/birth defect
- is medically significant or requires intervention to prevent one or other of the outcomes listed above.

Medical and scientific judgment should be exercised in deciding whether expedited reporting to the Sponsor is appropriate in other situations, such as important medical events that may not be immediately life-threatening or result in death or hospitalization but may jeopardize the patient or may require intervention to prevent one of the outcomes listed in the definitions above. These situations should also usually be considered serious.

Examples of such events are intensive treatment in an emergency room or at home for allergic bronchospasm; blood dyscrasias or convulsions that do not result in hospitalization; or development of drug dependency or drug abuse.

An unexpected AE is one in which the nature or severity is not consistent with the applicable product information.

Causality is initially assessed by the Investigator. For Serious Adverse Events, possible causes of the event are indicated by selecting one or more options. (Check all that apply)

- Pre-existing/Underlying disease - specify
- Study treatment - specify the drug(s) related to the event
- Other treatment (concomitant or previous) - specify
- Protocol-related procedure
- Other (e.g., accident, new or intercurrent illness) - specify

The term severe is a measure of intensity, thus a severe AE is not necessarily serious. For example, nausea of several hours' duration may be rated as severe, but may not be clinically serious.

A serious adverse event occurring during the study or which comes to the attention of the Investigator within 15 days after stopping the treatment or during the protocol-defined follow-up period, if this is longer, whether considered treatment-related or not, must be reported. In addition, a serious adverse event that occurs after this time, if considered related to test "drug", should be reported.

Such preliminary reports will be followed by detailed descriptions later which will include copies of hospital case reports, autopsy reports and other documents when requested and applicable.

For serious adverse events, the following must be assessed and recorded on the AEs eform of the eCRF: intensity, relationship to test substance, action taken, and outcome to date.

The Investigator must notify the Ethics Review Committee/Institutional Review Board of a serious adverse event in writing as soon as is practical and in accordance with international and local laws and regulations.

ROCHE LOCAL COUNTRY CONTACT for SAEs: Local Monitor:

See attached *Protocol Administrative and Contact Information & List of Investigators Form,[ gcp_for000227]*, for details of administrative and contact information.

ROCHE HEADQUARTERS CONTACT for SAEs and other medical emergencies: Clinical Operations/Clinical Science:

See attached *Protocol Administrative and Contact Information & List of Investigators form,[gcp_for000227]*, for details of administrative and contact information.

24 HOUR MEDICAL COVERAGE:

Identification of a contact for 24 Hour Medical Coverage is mandatory to be compliant with worldwide regulatory agencies and to ensure the safety of study patients.

An Emergency Medical Call Center Help Desk will access the Roche Medical Emergency List, escalate emergency medical calls, provide medical translation service (if necessary), connect the Investigator with the Roche medical contact for this study and track all calls. The Emergency Medical Call Center Help Desk will be manned 24 hours 7 days a week. Toll free numbers will be distributed to all Investigators running Roche Pharma Development clinical trials. The Help Desk will be used for medical emergencies outside regular business hours, or when the regular Clinical Science Leader cannot be reached.

See the attached *Protocol Administrative and Contact Information & List of Investigators form [gcp_for000227]*, for details of administrative, contact information, and Emergency Medical Call Center Help Desk toll-free numbers.

## Appendix 3: Common Terminology Criteria (CTCAE)

In the present study, toxicities will be graded according to the Common Terminology Criteria for Adverse Events (CTCAE), version 4.0.

The Common Terminology Criteria for Adverse Events v4.0 (CTCAE) can be found in the Roche hand-out entitled: " Common Terminology Criteria for Adverse Events v4.0" or via the following web-site: http://ctep.cancer.gov

## Appendix 4: Telephone Interviews

The purpose of this interview is to identify any new or worsening neurological symptoms that warrant an unscheduled visit and to collect data on possible events of infections. Telephone interviews should be performed by study personnel every 4 weeks between clinic visits (with exemption of prolonged B-cell monitoring period when telephone interviews need to be performed every 12 weeks) – see also Section 5.3.4.4.

**Please ask the following questions and record patient's answers during the Telephone Interview:**

| Questions | No | Yes |
|---|---|---|
| **1. Since your last visit or telephone interview, have you had any new or worsening medical problems (such as sudden changes in your thinking, alterations in your behavior, visual disturbances, extremity weakness, limb coordination problems, or gait abnormalities) that have persisted over several days?** | | |
| **2. Since your last visit or telephone interview, have you taken any new medicines to treat cancer or MS or any other new medicines that weaken your immune system?** | | |
| **3. Since your last visit or telephone interview, other than for the treatment of a recent relapse, have you taken any of the following medicines: methylprednisone, (e.g., Depo-Medrol®, Solu-Medrol®), dexamethasone (e.g., Decadron®), prednisolone, or other steroid medicine?** | | |
| **4. Since your last visit or telephone interview, have you had any signs or symptoms of infection?** | | |

If the patient answered YES to any question, contact the Treating Investigator and review the patient's answers. The Investigator can determine if an unscheduled visit is required.

**Record any pertinent comments made by the patient during the interview:**

________________________________________________________________

________________________________________________________________

________________________________________________________________

NAME:__________________________________ Date:__________________________________
*Name of person completing the telephone interview*

## Appendix 4: Telephone Interviews (Cont.)

**Below is a sample list of medications that can weaken the immune system. This list does not include all drugs that can suppress the immune system.**

**Approved MS Therapies:**

Glatiramer acetate (Copaxone®)

Interferon β-1a (Rebif®, AVONEX®)

Interferon β-1b (Betaseron®)

Mitoxantrone (Novantrone®)

Natalizumab (Tysabri®)

Fingolimod (Gilenya®) – if relevant

**Immunosuppressants/Antineoplastics:**

Azathioprine (Imuran®, Azasan®)

Cladribine (Leustatin®)

Cyclophosphamide (Cytoxan®, Neosar®)

Cyclosporine (Sandimmune®, Neoral®)

Fludarabine phosphate (Fludara®)

Leflunomide (Arava®)

Mercaptopurine (Purinethol®)

Methotrexate (Methotrex®, Rheumatrex®, Trexall®)

Mycophenolate mofetil (CellCept®)

Pemetrexed (Alimta®)

**Additional Immunomodulators and Immunosuppressants:**

Other interferons (Actimmune®, Infergen®, Intron® A,

Pegasys®, PEG-Intron®, Rebetron®, Roferon®-A)

Adalimumab (Humira®)

Alefacept (Amevive®)

Alemtuzumab (Campath®)

Anakinra (Kineret®)

Daclizumab (Zenapax®)

Etanercept (Enbrel®)

Infliximab (Remicade®)

Intravenous immunoglobulin (IVIG)

Ofatumumab (Arzerra®)

Rituximab (Rituxan/MabThera®)

Trastuzumab (Herceptin®)

## Appendix 5: Modified Fatigue Impact Scale (MFIS)

MFIS

### MODIFIED FATIGUE IMPACT SCALE (MFIS)

Following is a list of statements that describe how fatigue may affect a person. Fatigue is a feeling of physical tiredness and lack of energy that many people experience from time to time. In medical conditions like MS, feelings of fatigue can occur more often and have a greater impact than usual. Please read each statement carefully, and then SELECT THE ONE NUMBER that best indicates how often fatigue has affected you in this way during the PAST 4 WEEKS. (If you need help in marking your responses, TELL THE INTERVIEWER THE NUMBER of the best response.) PLEASE ANSWER EVERY QUESTION. If you are not sure which answer to select, please choose the one answer that comes closest to describing you. The interviewer can explain any words or phrases that you do not understand.

| **Because of my fatigue during the PAST 4 WEEKS....** | Never | Rarely | Sometimes | Often | Almost always |
|---|---|---|---|---|---|
| * 1. I have been less alert. | ◯ 0 | ◯ 1 | ◯ 2 | ◯ 3 | ◯ 4 |
| * 2. I have had difficulty paying attention for long periods of time. | ◯ 0 | ◯ 1 | ◯ 2 | ◯ 3 | ◯ 4 |
| * 3. I have been unable to think clearly. | ◯ 0 | ◯ 1 | ◯ 2 | ◯ 3 | ◯ 4 |
| * 4. I have been clumsy and uncoordinated. | ◯ 0 | ◯ 1 | ◯ 2 | ◯ 3 | ◯ 4 |
| * 5. I have been forgetful. | ◯ 0 | ◯ 1 | ◯ 2 | ◯ 3 | ◯ 4 |
| * 6. I have had to pace myself in my physical activities. | ◯ 0 | ◯ 1 | ◯ 2 | ◯ 3 | ◯ 4 |

Back | Next

## Appendix 5: Modified Fatigue Impact Scale (MFIS) (Cont.)

MFIS

### MODIFIED FATIGUE IMPACT SCALE (MFIS)

| Because of my fatigue during the PAST 4 WEEKS.... | Never | Rarely | Sometimes | Often | Almost always |
|---|---|---|---|---|---|
| * 7. I have been less motivated to do anything that requires physical effort. | 0 | 1 | 2 | 3 | 4 |
| * 8. I have been less motivated to participate in social activities. | 0 | 1 | 2 | 3 | 4 |
| * 9. I have been limited in my ability to do things away from home. | 0 | 1 | 2 | 3 | 4 |
| * 10. I have had trouble maintaining physical effort for long periods. | 0 | 1 | 2 | 3 | 4 |
| * 11. I have had difficulty making decisions. | 0 | 1 | 2 | 3 | 4 |
| * 12. I have been less motivated to do anything that requires thinking. | 0 | 1 | 2 | 3 | 4 |
| * 13. my muscles have felt weak. | 0 | 1 | 2 | 3 | 4 |
| * 14. I have been physically uncomfortable. | 0 | 1 | 2 | 3 | 4 |

Back | Next

## Appendix 5: Modified Fatigue Impact Scale (MFIS) (Cont.)

MFIS

### MODIFIED FATIGUE IMPACT SCALE (MFIS)

| Because of my fatigue during the PAST 4 WEEKS.... | Never | Rarely | Sometimes | Often | Almost always |
|---|---|---|---|---|---|
| * 15. I have had trouble finishing tasks that require thinking. | ◯ 0 | ◯ 1 | ◯ 2 | ◯ 3 | ◯ 4 |
| * 16. I have had difficulty organizing my thoughts when doing things at home or at work. | ◯ 0 | ◯ 1 | ◯ 2 | ◯ 3 | ◯ 4 |
| * 17. I have been less able to complete tasks that require physical effort. | ◯ 0 | ◯ 1 | ◯ 2 | ◯ 3 | ◯ 4 |
| * 18. my thinking has been slowed down. | ◯ 0 | ◯ 1 | ◯ 2 | ◯ 3 | ◯ 4 |
| * 19. I have had trouble concentrating. | ◯ 0 | ◯ 1 | ◯ 2 | ◯ 3 | ◯ 4 |
| * 20. I have limited my physical activities. | ◯ 0 | ◯ 1 | ◯ 2 | ◯ 3 | ◯ 4 |
| * 21. I have needed to rest more often or for longer periods. | ◯ 0 | ◯ 1 | ◯ 2 | ◯ 3 | ◯ 4 |

Back | Next

## Appendix 6: The Center for Epidemiologic Studies Depression Scale (CES-D)

**CES-D Scale**

### Center for Epidemiologic Studies Depression Scale (CES-D, NIMH)

Below is a list of the ways you might have felt or behaved. Please tell me how often you have felt this way in the last week.

| | During the Past Week | | | |
|---|---|---|---|---|
| | Rarely or none of the time (less than 1 Day) | Some or a little of the time (1-2 days) | Occasionally or a moderate amount of time (3-4 days) | Most or all of the time (5-7 days) |
| * 1. I was bothered by things that usually don't bother me. | ○ | ○ | ○ | ○ |
| * 2. I did not feel like eating: my appetite was poor. | ○ | ○ | ○ | ○ |
| * 3. I felt that I could not shake off the blues even with help from my family or friends. | ○ | ○ | ○ | ○ |
| * 4. I felt that I was just as good as other people. | ○ | ○ | ○ | ○ |
| * 5. I had trouble keeping my mind on what I was doing. | ○ | ○ | ○ | ○ |
| * 6. I felt depressed. | ○ | ○ | ○ | ○ |
| * 7. I felt that everything I did was an effort. | ○ | ○ | ○ | ○ |
| * 8. I felt hopeful about the future. | ○ | ○ | ○ | ○ |
| * 9. I thought my life had been a failure. | ○ | ○ | ○ | ○ |
| * 10. I felt fearful. | ○ | ○ | ○ | ○ |

Back | Next

CES-D Scale

## Center for Epidemiologic Studies Depression Scale (CES-D, NIMH)

Below is a list of the ways you might have felt or behaved. Please tell me how often you have felt this way in the last week.

| | During the Past Week | | | |
|---|---|---|---|---|
| | Rarely or none of the time (less than 1 Day) | Some or a little of the time (1-2 days) | Occasionally or a moderate amount of time (3-4 days) | Most or all of the time (5-7 days) |
| * 11. My sleep was restless. | ○ | ○ | ○ | ○ |
| * 12. I was happy. | ○ | ○ | ○ | ○ |
| * 13. I talked less than usual. | ○ | ○ | ○ | ○ |
| * 14. I felt lonely. | ○ | ○ | ○ | ○ |
| * 15. People were unfriendly. | ○ | ○ | ○ | ○ |
| * 16. I enjoyed life. | ○ | ○ | ○ | ○ |
| * 17. I had crying spells. | ○ | ○ | ○ | ○ |
| * 18. I felt sad. | ○ | ○ | ○ | ○ |
| * 19. I felt that people dislike me. | ○ | ○ | ○ | ○ |
| * 20. I could not get "going". | ○ | ○ | ○ | ○ |

SCORING: zero for answers in the first column, 1 for answers in the second column, 2 for answers in the third column, 3 for answers in the fourth column. The scoring of positive items is reversed. Possible range of scores is zero to 60, with the higher scores indicating the presence of more symptomatology.

Back | Next

## Appendix 7: The Short Form (SF-36) Health Survey

**SF-36**

### Your Health and Well-Being

This survey asks for your views about your health. This information will help keep track of how you feel and how well you are able to do your usual activities. Thank you for completing this survey!

For each of the following questions, please SELECT the response that best describes your answer.

*** 1. In general, would you say your health is:**

| Excellent | Very good | Good | Fair | Poor |
|---|---|---|---|---|
| ○ 1 | ○ 2 | ○ 3 | ○ 4 | ○ 5 |

*** 2. COMPARED TO ONE YEAR AGO, how would you rate your health in general NOW?**

| Much better now than one year ago | Somewhat better now than one year ago | About the same as one year ago | Somewhat worse now than one year ago | Much worse now than one year ago |
|---|---|---|---|---|
| ○ 1 | ○ 2 | ○ 3 | ○ 4 | ○ 5 |

SF-36v2® Health Survey © 1992, 2002, 2009 Medical Outcomes Trust and QualityMetric Incorporated. All rights reserved.
SF-36® is a registered trademark of Medical Outcomes Trust.

Back | Next

# Appendix 7: The Short Form (SF-36) Health Survey (Cont.)

**SF-36**

**3. The following questions are about activities you might do during a typical day. Does YOUR HEALTH NOW LIMIT YOU in these activities? If so, how much?**

| | Yes, limited a lot | Yes, limited a little | No, not limited at all |
|---|---|---|---|
| * a. VIGOROUS ACTIVITIES, such as running, lifting heavy objects, participating in strenuous sports. | ○ 1 | ○ 2 | ○ 3 |
| * b. MODERATE ACTIVITIES, such as moving a table, pushing a vacuum cleaner, bowling, or playing golf. | ○ 1 | ○ 2 | ○ 3 |
| * c. Lifting or carrying groceries. | ○ 1 | ○ 2 | ○ 3 |
| * d. Climbing SEVERAL flights of stairs. | ○ 1 | ○ 2 | ○ 3 |
| * e. Climbing ONE flight of stairs. | ○ 1 | ○ 2 | ○ 3 |
| * f. Bending, kneeling, or stooping. | ○ 1 | ○ 2 | ○ 3 |
| * g. Walking MORE THAN A MILE. | ○ 1 | ○ 2 | ○ 3 |
| * h. Walking SEVERAL HUNDRED YARDS. | ○ 1 | ○ 2 | ○ 3 |
| * i. Walking ONE HUNDRED YARDS. | ○ 1 | ○ 2 | ○ 3 |
| * j. Bathing or dressing yourself. | ○ 1 | ○ 2 | ○ 3 |

SF-36v2® Health Survey © 1992, 2002, 2009 Medical Outcomes Trust and QualityMetric Incorporated. All rights reserved.
SF-36® is a registered trademark of Medical Outcomes Trust.

Back | Next

## SF-36

4. During the PAST 4 WEEKS, how much of the time have you had any of the following problems with your work or other regular daily activities AS A RESULT OF YOUR PHYSICAL HEALTH?

| | All of the time | Most of the time | Some of the time | A little of the time | None of the time |
|---|---|---|---|---|---|
| * a. Cut down on the AMOUNT OF TIME you spent on work or other activities. | ◯ 1 | ◯ 2 | ◯ 3 | ◯ 4 | ◯ 5 |
| * b. ACCOMPLISHED LESS than you would like. | ◯ 1 | ◯ 2 | ◯ 3 | ◯ 4 | ◯ 5 |
| * c. Were limited in the KIND of work or other activities. | ◯ 1 | ◯ 2 | ◯ 3 | ◯ 4 | ◯ 5 |
| * d. Had DIFFICULTY performing the work or other activities (for example, it took extra effort). | ◯ 1 | ◯ 2 | ◯ 3 | ◯ 4 | ◯ 5 |

5. During the PAST 4 WEEKS, how much of the time have you had any of the following problems with your work or other regular daily activities AS A RESULT OF ANY EMOTIONAL PROBLEMS (such as feeling depressed or anxious)?

| | All of the time | Most of the time | Some of the time | A little of the time | None of the time |
|---|---|---|---|---|---|
| * a. Cut down on the AMOUNT OF TIME you spent on work or other activities. | ◯ 1 | ◯ 2 | ◯ 3 | ◯ 4 | ◯ 5 |
| * b. ACCOMPLISHED LESS than you would like. | ◯ 1 | ◯ 2 | ◯ 3 | ◯ 4 | ◯ 5 |
| * c. Did work or other activities LESS CAREFULLY THAN USUAL. | ◯ 1 | ◯ 2 | ◯ 3 | ◯ 4 | ◯ 5 |

SF-36v2® Health Survey © 1992, 2002, 2009 Medical Outcomes Trust and QualityMetric Incorporated. All rights reserved.
SF-36® is a registered trademark of Medical Outcomes Trust.

Back | Next

## SF-36

* 6. During the PAST 4 WEEKS, to what extent has your PHYSICAL HEALTH OR EMOTIONAL PROBLEMS interfered with your normal social activities with family, friends, neighbors, or groups?

| Not at all | Slightly | Moderately | Quite a bit | Extremely |
|---|---|---|---|---|
| ○ 1 | ○ 2 | ○ 3 | ○ 4 | ○ 5 |

* 7. How much BODILY pain have you had during the PAST 4 WEEKS?

| None | Very mild | Mild | Moderate | Severe | Very severe |
|---|---|---|---|---|---|
| ○ 1 | ○ 2 | ○ 3 | ○ 4 | ○ 5 | ○ 6 |

* 8. During the PAST 4 WEEKS, how much did PAIN interfere with your normal work (including both work outside the home and housework)?

| Not at all | A little bit | Moderately | Quite a bit | Extremely |
|---|---|---|---|---|
| ○ 1 | ○ 2 | ○ 3 | ○ 4 | ○ 5 |

SF-36v2® Health Survey © 1992, 2002, 2009 Medical Outcomes Trust and QualityMetric Incorporated. All rights reserved.
SF-36® is a registered trademark of Medical Outcomes Trust.

Back | Next

**SF-36**

9. These questions are about how you feel and how things have been with you DURING THE PAST 4 WEEKS. For each question, please give the one answer that comes closest to the way you have been feeling. How much of the time during the PAST 4 WEEKS...

| | All of the time | Most of the time | Some of the time | A little of the time | None of the time |
|---|---|---|---|---|---|
| * a. Did you feel full of life? | ○ 1 | ○ 2 | ○ 3 | ○ 4 | ○ 5 |
| * b. Have you been very nervous? | ○ 1 | ○ 2 | ○ 3 | ○ 4 | ○ 5 |
| * c. Have you felt so down in the dumps that nothing could cheer you up? | ○ 1 | ○ 2 | ○ 3 | ○ 4 | ○ 5 |
| * d. Have you felt calm and peaceful? | ○ 1 | ○ 2 | ○ 3 | ○ 4 | ○ 5 |
| * e. Did you have a lot of energy? | ○ 1 | ○ 2 | ○ 3 | ○ 4 | ○ 5 |
| * f. Have you felt downhearted and depressed? | ○ 1 | ○ 2 | ○ 3 | ○ 4 | ○ 5 |
| * g. Did you feel worn out? | ○ 1 | ○ 2 | ○ 3 | ○ 4 | ○ 5 |
| * h. Have you been happy? | ○ 1 | ○ 2 | ○ 3 | ○ 4 | ○ 5 |
| * i. Did you feel tired? | ○ 1 | ○ 2 | ○ 3 | ○ 4 | ○ 5 |

SF-36v2® Health Survey © 1992, 2002, 2009 Medical Outcomes Trust and QualityMetric Incorporated. All rights reserved.
SF-36® is a registered trademark of Medical Outcomes Trust.

Back | Next

# Appendix 7: The Short Form (SF-36) Health Survey (Cont.)

**SF-36**

* 10. During the PAST 4 WEEKS, how much of the time has your PHYSICAL HEALTH OR EMOTIONAL PROBLEMS interfered with your social activities (like visiting with friends, relatives, etc.)?

| All of the time | Most of the time | Some of the time | A little of the time | None of the time |
|---|---|---|---|---|
| ○ 1 | ○ 2 | ○ 3 | ○ 4 | ○ 5 |

11. How TRUE or FALSE is EACH of the following statements for you?

| | Definitely true | Mostly true | Don't know | Mostly false | Definitely false |
|---|---|---|---|---|---|
| * a. I seem to get sick a little easier than other people. | ○ 1 | ○ 2 | ○ 3 | ○ 4 | ○ 5 |
| * b. I am as healthy as anybody I know. | ○ 1 | ○ 2 | ○ 3 | ○ 4 | ○ 5 |
| * c. I expect my health to get worse. | ○ 1 | ○ 2 | ○ 3 | ○ 4 | ○ 5 |
| * d. My health is excellent. | ○ 1 | ○ 2 | ○ 3 | ○ 4 | ○ 5 |

SF-36v2® Health Survey © 1992, 2002, 2009 Medical Outcomes Trust and QualityMetric Incorporated. All rights reserved.
SF-36® is a registered trademark of Medical Outcomes Trust.

Back | Next

## Appendix 8: Optical Coherence Tomography Exploratory Substudy

*Introduction*

Optical coherence tomography (OCT) is a noninvasive imaging tool capable of sensitive, reproducible and rapid measuruments of structural architecture of the retina and retinal nerve fiber layer [1]. OCT can be of particular interest in multiple sclerosis (MS), because optic neuritis is often the pivotal event in establishing the diagnosis of MS. Optic nerve dysfunction is characterized by optic disc pallor and loss of contrast sensitivity, and visual field defects, which may occur subclinically in many patients. It is estimated that nearly 20% of all patients with MS present initially with optic neuritis, and an additional 30% – 100% will have optic neuritis at some point in their disease course [2]. OCT outcome measures such as retinal nerve fiber layer (RNFL) thickness and macular volumes have been shown to correlate with clinical measures of vision loss and may facilitate visualization of any process of neurodegeneration or repair as part of natural history of MS or as a consequence of neuroprotective interventions [3].

This substudy is part of three ongoing Phase III studies that serve to evaluate the neuroprotective effect of ocrelizumab in MS as measured by RNFL thickness and macular volume in both eyes of patients who participate in the confirmatory pivotal studies in patients with relapsing multiple sclerosis (RMS; Studies WA21092 and WA21093) or patients with primary progressive multiple sclerosis (PPMS; Study WA25046).

***Objectives***

***Efficacy Objectives***

The primary efficacy objectives for this substudy are as follows:

- To evaluate the neuroprotective effect of ocrelizumab therapy as measured by macular volume and RNFL over time
- To characterize the time-course of changes in RNFL that imply axonal loss in patients with both RMS and PPMS with or without ocrelizumab treatment

The secondary efficacy objectives for this study are as follows:

- In the case of RMS patients (Studies WA21092 and WA21093), the study will assess whether ocrelizumab 600 mg has superior neuroprotective effect compared with Rebif® as measured by RNFL thickness.
- In the case of PPMS patients (Study WA25046), the study will assess whether ocrelizumab 600 mg has superior neuroprotective effect compared with placebo as measured by RNFL thickness.

### *Safety Objectives*

The safety objectives for this study are as follows:

- To evaluate the ophthalmological safety of ocrelizumab therapy in patients with MS, focusing on serious adverse events (SAEs)

### *Exploratory Objectives*

The exploratory objectives for this study are as follows:

- To evaluate if OCT outcomes can serve as a reliable and predictive measure of response to ocrelizumab therapy in MS patients or progression to a more severe disease state
- To evaluate the relationship of OCT outcomes with outcomes from ocrelizumab Phase III pivotal studies, such as
  - Change in brain volume as measured by brain magnetic resonance imaging (MRI)
  - *Confirmed* disability progression
  - T2 lesion volume
  - Number of T1 Gd-enhanced lesions
  - Change in Multiple Sclerosis Functional Composite Scale score

## ***Study Design***

### *Description of Study*

#### *Overview*

The current substudy is an add-on, multicenter, longitudinal study to the ongoing Phase III ocrelizumab Studies WA21092, WA21093, and WA25046 to evaluate the neuroprotective effects of ocrelizumab treatment as measured by OCT. OCT will be performed in parallel to the ocrelizumab pivotal Phase III studies.

Patients can be enrolled at any time during the first 48 weeks after enrollment in the main pivotal studies. However, all attempts should be made to enroll the patient at the time of screening of the main pivotal study. Patients will undergo an ophthalmological examination prior to first OCT scan and at the end of the study. Patients will also undergo at least three OCT scans at 24-week intervals (see schedule of assessments of this substudy, Appendix A).

For patients participating in Studies WA21092 and WA21093:

- The patient should undergo OCT measurement every 24 weeks after the first OCT visit (Visit 1 of the OCT substudy). If the patient is enrolled into the OCT substudy at baseline of the main study, the following visits should occur: Visit 1 (Week 0; occurring at the baseline visit of the main study), Visit 2 (Week 24; occurring at Visit 5 of the main study), Visit 3 (Week 48; occurring at Visit 7 of the main study), Visit 4 (Week 72; occurring at Visit 9 of the main study) and Visit 5 (Week 96; occurring at Visit 11 of the main study). If the patient is enrolled after baseline of the main study, OCT visits should occur every 24 weeks after the first OCT visit (Visit 1, Week 0).

For patients participating in Study WA25046:

- The patient should undergo OCT measurement every 24 weeks after the first OCT visit (Visit 1 of the OCT substudy). If the patient is enrolled into the OCT substudy at baseline of the main study, the following visits should occur: Visit 1 (Week 0; occurring at the baseline visit of the main study), Visit 2 (Week 24, occurring at Visit 5 of the main study), Visit 3 (Week 48, occurring at Visit 8 of the main study), Visit 4 (Week 72, occurring at Visit 11 of the main study), Visit 5 (Week 96, occurring at Visit 14 of the main study), and Visit 6 (Week 120, occurring at Visit 17 of the main study). If the patient is enrolled after baseline of the main study, OCT visits should occur every 24 weeks after the first OCT visit (Visit 1, Week 0).

If the patient is withdrawn from study treatment in the main protocol, an OCT visit should occur if it has not been performed during the previous 4 weeks. Patients should then have an OCT measurement at the end of the safety follow-up of the main protocol.

If a patient decides to participate in the Open-Label Extension Phase of the main protocol, OCT measurements should continue to occur every 24 weeks during the OLE Phase.

A schedule of assessments is provided in Appendix A of this substudy.

*Independent Review Committee (IRC)*

A masked, central OCT reading center will review and analyze OCT endpoints. The Independent Review Committee (IRC) membership and procedures will be detailed in an IRC charter.

***End of Study***

The end of the study is defined as the date when the last patient, last visit (LPLV) of this substudy occurs. LPLV of this substudy is expected to occur at the same time of LPLV of the last ongoing Phase III pivotal ocrelizumab studies (i.e., either WA21092, WA21093, or WA25046), or earlier.

***Outcome Measures***

*Efficacy Outcome Measures*

The efficacy outcome measures for this study are as follows:

- Overall and quadrant RNFL thickness measured by OCT
- Macular volume maps as measured by OCT

*Safety Outcome Measures*

The safety outcome measures for this study are as follows:

- Incidence, nature, and severity of ophthalmological adverse events (AEs)

***Materials and Methods***

***Patients***

Adult patients who fulfill eligibility criteria for one of the main Phase III ocrelizumab pivotal studies (i.e., WA21092, WA21093, or WA25046) and the eligibility criteria outlined in the following inclusion and exclusion sections of this substudy can be enrolled into the study.

*Inclusion Criteria*

Patients must meet the following criteria for study entry:

- Able and willing to provide written informed consent and comply with the study protocol
- Be a participant in one of the following studies: WA21092, WA21093, or WA25046

*Exclusion Criteria*

Patients who meet any of the following criteria will be excluded from study entry:

- Medical history of macular degeneration, retinopathy, glaucoma, amblyopia, diabetes, or any other documented cause of vision loss
- Inability to undergo reliable OCT testing
- More than 48 weeks have lapsed since randomization

***Study Assessments***

*Screening and Baseline Examination and Eligibility Screening Form*

All patients must sign and date the most current Institutional Review Board/Institutional Ethics Committee's approved written informed consent before any study specific assessments or procedures are performed.

Consenting patients must also have signed the informed consent and be eligible for the main pivotal Phase III study. Patients will receive an ophthalmological and eye examination to be evaluated for eligibility to participate in this substudy. If the patient is eligible, this ophthalmological examination will be considered the baseline ophthalmological measure (OCT Visit 1).

An Eligibility Screening Form documenting the Investigator's assessment of each screened patient with regard to the protocol's inclusion and exclusion criteria is to be completed by the Investigator.

It should be stated in the medical record that the patient is participating in this clinical study.

Once a patient has fulfilled all eligibility criteria, he or she will undergo OCT according to schedule of assessments (Appendix A of this substudy).

*Procedure in Case of Delayed Dosing Visit, Relapse or Unscheduled Visits, and Withdrawal Visits in the Main Phase III Ocrelizumab Pivotal Studies*

The main ocrelizumab pivotal studies have mechanisms set up regarding delayed dosing visits, unscheduled visits due to relapse, and withdrawal visits.

OCT measurements should occur at initially scheduled times and should not be rescheduled due to delayed dosing schedule or unscheduled visit due to relapse. In case there is a relapse during an OCT scheduled visit, the Investigator should document all ocular symptoms, including ocular neuritis, in the electronic Case Report Form as well as communicate them to the Principal Investigator of the main study. If a patient suffers a relapse with symptoms acute optic neuritis during the month before OCT study, a second OCT must be performed 1 month later to account for the effect of papilledema.

If the patient is withdrawn from study treatment in the main protocol, an OCT visit should occur if it has not been performed during the previous 4 weeks. Patients should then have an OCT measurement every 24 weeks until the end of the safety follow-up of the main protocol.

It is possible that the patient withdraws from only the OCT Exploratory substudy. In this case, an OCT visit should occur if it has not been performed during the previous 4 weeks.

*Description of Study Assessments*

*Medical History and Demographic Data*

Medical history and demographic data will be captured in main study in which the patient is enrolled. Further medical history includes clinically significant diseases that may cause vision loss of the affected eye that have been documented by the ophthalmologist.

*Ophthalmological and Eye Examination.*

The presence of any visual abnormalities will be established in a full eye exam. This will include an examination performed by an ophthalmologist during Visit 1 (Week 0) and completion/withdrawal visit. This will include best corrected visual acuity evaluation (with ETDRS standardized eye chart); color vision test (Hardy Rand Rittler pseudoisochormatic plate); visual field measurement; intraocular pressure; slitlamp examination to examine the anterior parts (cornea, lens, and sclera); and a dilated ophthalmoscopic examination of retina, optic nerve, retinal blood vessels, and macula. The eye chart chosen for a patient must be consistently used throughout the study as well as the use of corrective lenses during the ophthalmological exam.

*Visual Evoked Potential*

Visual Evoked Potential (VEP) is a sensitive test of visual pathway function and a marker of optic nerve involvement in MS, and it has been used as a diagnostic tool for RMS and PPMS [4]. It can be used to supplement information provided by a clinical examination to provide objective evidence of a second lesion provided that the only clinically expressed lesion did not affect the visual pathways. VEP measurement should occur during OCT Visit 1 (Week 0). If available at the center, multifocal VEP will be performed instead of full-field VEP.

Note: In the rare cases that the selected site cannot deliver VEP data for any reason, VEP measurements will not be necessary after discussion with the Sponsor. However, all efforts should be made to collect VEP data during Visit 1 (Week 0).

*Optical Coherence Tomography*

OCT images will be acquired in all patients as detailed in the schedule of assessments (Appendix A of this substudy). In addition, an OCT scan will be performed in patients withdrawn from the main study treatment if it has not been performed within the previous 4 weeks.

All OCT images will be performed by certified personnel. The following time windows apply:

- OCT Visit 1 (Week 0) should occur prior to baseline visit of the primary study and at Visit 2 (Week 24), Visit 3 (Week 48), Visit 4 (Week 72), and Visit 5 (Week 96).
- In case the patient is enrolled after the baseline visit of main study, OCT Visit 1 should occur at time of enrollment into the substudy. All further OCT scans should occur every 24 weeks thereafter.
- *If a patient enrolls into the OLE Phase of the main study, OCT assessments should continue to occur every 24 weeks.*

During OCT Visit 1 (Week 0), the OCT scan should be performed twice in order to control for test-retest reliability.

OCT images will be read by a centralized reading center. The reading will be performed in a masked fashion in the absence of clinical information. Further details on the OCT protocols and standardization of machines are described in the separate Independent Review Committee Charter.

*Procedure in Case of Ocular Neuritis or Relapse*

If the patient presents with optic neuritis, the affected eye should be documented. The patient should also be referred to the Principal Investigator of the main study for examination. If the patient suffers a relapse with symptoms of acute optic neuritis during the month before an OCT scan, a second OCT scan must be performed 1 month later to account for the effect of papilledema.

***Assessment of Safety***

***Safety Plan***

Any AE regarding ocular findings should be reported to the Principal Investigator of the main study in which the subject is participating, as well as the Sponsor. Please refer to main protocol Section 7 regarding the procedures related to reporting of AEs and SAEs.

***Statistical Considerations and Analysis Plan***

Full details of all statistical issues and planned statistical analyses will be specified in the IRC charter.

***Sample Size***

A total of approximately 300 patients will be enrolled in this substudy, and the total study duration will be approximately 96 weeks, depending on the time of enrollment.

***Efficacy Analyses***

The purpose of this study is to estimate the neuroprotective effect of ocrelizumab treatment in patients with MS relative to Rebif in the case of Studies WA21092 and WA21093 and relative to placebo in the case of Study WA25046. Point and interval estimates of the decrease of RNFL thickness and macular volume will be obtained.

*Primary Efficacy Endpoint*

The primary efficacy endpoint is the decrease in RNFL thickness over time during the duration of the main study.

*Secondary and Exploratory Efficacy Endpoints*

Secondary and exploratory endpoints are:

- Determination of macular volume over time during the duration of the study
- Exploratory correlation analyses to determine the predictive value of OCT measures (RNFL thickness and macular volume) with outcomes measured in the main study, such as (but not restricted to):
  - Change in brain volume as measured by brain MRI
  - *Confirmed* disability progression
  - T2 lesion volume
  - Number of T1 Gd-enhanced lesions
  - Change in Multiple Sclerosis Functional Composite Scale score

***Safety Analyses***

No safety analyses are planned for this substudy. Any safety event noted during this substudy will be forward to the principal Investigator of the main study. Please refer to Section 7 of the main protocol.

References

1. Frohman, EM, Fujimoto, JG, Frohman, TC, et al. Optical coherence tomography: a window into the mechanisms of multiple sclerosis. Nature Clinical Practice 2008;4:664-675.

2. Sergott RC, Frohman E, Glanzman R, et al. The role of optical coherence tomography in multiple sclerosis: expert panel consensus. J Neurol Sci 2007;263:3-14.

3. Costello F, Hodge W, Pan YI et al. Tracking retinal nerve fiber layer loss after optic neuritis: a prospective study using optical coherence tomography. Multiple Sclerosis 2008;14:893-905.

4. Polman CH, Reingold SC, Edan G, et al. Diagnostic criteria for multiple sclerosis: 2005 revisions to the "McDonald Criteria". Ann Neurol 2005;58:8406.

## Appendix 8: Optical Coherence Tomography Exploratory Substudy (Cont.)

## Appendix A OPTICAL COHERENCE TOMOGRAPHY EXPLORATORY SUBSTUDY SCHEDULE OF ASSESSMENTS

Schedule of Assessments

| | Treatment Period [a] | | | | | Open-Label Extension Phase (for OPERA study)[e] | Completion Visit | Safety Follow-Up | Withdrawal Visit a |
|---|---|---|---|---|---|---|---|---|---|
| Visit | 1 | 2 | 3 | 4 | 5 | | | | |
| Week (window in days) | 0 | 24 (±14) | 48 (±14) | 72 (±14) | 96 (±14) | | | | |
| Informed consent | x | | | | | | | | |
| Ophthalmological and Eye Examination | x | | | | | | x | | x |
| Visual Evoked Potential | x | | | | | | x | | x |
| Medical History | x | | | | | | x | | x |
| Baseline Conditions | x | | | | | | x | | x |
| OCT[b] | x | x | x | x | x | x | x | xc | |
| Adverse events [d] | x | x | x | x | x | x | x | x | x |

OCT = optical coherence tomography

a) Treatment period is variable, as it depends on the time of enrollment within the main study.

b) In cases where the initially submitted OCT visit scan is inadequate/not evaluable by Bern Photographic Reading Centre, the site should make all efforts to request the patient to come back to site to perform a new OCT scan.

c) Safety Follow-Up: OCT assessment should continue every 48 weeks counting after last OCT assessment visit.

d) Any adverse events should be reported to the principal investigator of the study.

*e) If a patient enrolls in the OLE Phase of the main study, OCT assessments should occur every 24 weeks.*

## WA21092: Changes in Conduct of Study or Planned Analyses

There were four protocol amendments to the original Protocol WA21092 Version A released on 25 August 2010.

### Protocol WA21092 Version B

Study protocol WA21092 Version B was released on 1 June 2011 *prior to any patients being enrolled.*

### Protocol WA21092 Version C

Study protocol WA21092 Version C was released on 15 June 2012. The main changes to the protocol were as follows:

- Dosing preparation and infusion guidance were revised to simplify the preparation of infusion bags and dosing procedures
- Specific eligibility cutoff values for immunoglobulin M (IgM) and immunoglobulin G (IgG) and the re-treatment criteria for IgG were modified to reflect the central lab reference ranges
- Inclusion/exclusion criteria were revised to:
  - Exclude enrollment of patients who had undergone experimental procedures for treating MS
  - Allow patients who were unable to receive gadolinium contrast for magnetic resonance imagining (MRI) to participate in the study (such patients could participate in the study, but not receive gadolinium during an MRI scan
  - Allow patients to continue previous MS therapies with β-interferons, glatiramer acetate and other permitted immunomodulatory therapies until randomization
  - Allow patients previously treated with dimethyl fumarate (Tecfidera)to enter the study following an adequate washout period (24 weeks)
  - Allow patients with contraindication to methylprednisolone to enter the study; these patients thus received an equivalent dose of an alternative corticosteroid as premedication prior to ocrelizumab infusion
- Sites were informed of additional, optional sub-studies conducted at select centers in which patients could be eligible to participate.

### Protocol WA21092 Version D

Study protocol WA21092 Version D was released on 14 March 2013. The main changes to the protocol were as follows:

- Inclusion of an OLE phase under the same protocol
- Addition of the following exploratory objectives: proportion of disease activity free patients, defined as absence of both relapses and sustained accumulation of disability, and absence of MRI activity by Week 96; evaluation of long term safety and efficacy of ocrelizumab in the OLE study phase
- Clarification on how sustained disability progression is calculated.

**Protocol WA21092 Version E**

Study Protocol WA21092 Version E was released on 4 September 2014. The main changes to the protocol were as follows:

- Update to the Statistical Considerations and Analytical Plan section of the protocol in line with the SAP amendment to implement European Medicines Agency (EMA) Scientific Advice and to increase statistical rigor. This included:
    - Modifications to the secondary efficacy endpoints, their definitions and hierarchical order
    - Modifications to the exploratory efficacy endpoints and their definitions
    - Modifications to statistical methodology (replacement of analysis of covariance ANCOVA with Mixed-Effect Model Repeated Measures [MMRM] method)
    - Clarifications to various aspects of the safety analysis, including change to the definition of safety population
    - Change to the timing of database lock to allow for 12-week confirmation of all initial disability progression events during the 96-week comparative treatment period
    - Clarification of the calculation of the baseline EDSS value, the timing of unblinding of the sites and EDSS examining investigators
- Conversion of the optional investigator-sponsored Roche-supported exploratory substudies to Roche-sponsored studies as per current policy of the sponsor
- Revision of the pre-specified mandatory biomarker

# F. HOFFMANN-LA ROCHE LTD
# CLINICAL STUDY PROTOCOL
# PROTOCOL NUMBER WA21093
# RO 4964913

# EUDRACT NUMBER 2010-020315-36

## PROTOCOL APPROVAL

Protocol Number / Version: WA21093 /A

Date: See last date in electronic signature manifestation below.

Protocol approved by: See electronic signature manifestation below.

| Name | Reason for Signing | Date and Time (UTC) |
|---|---|---|
| Glanzman, Robert | Clinical Science Leader | 27-May-2011 20:12:17 |


**Confidentiality Statement**

The information contained in this document, especially unpublished data, is the property of F. Hoffmann-La Roche Ltd (or under its control), and therefore provided to you in confidence as an investigator, potential investigator or consultant, for review by you, your staff and an applicable Independent Ethics Committee/Institutional Review Board. It is understood that this information will not be disclosed to others without written authorization from Roche except to the extent necessary to obtain informed consent from those persons to whom the drug may be administered.

# SYNOPSIS OF PROTOCOL NUMBER WA21093

| | | | |
|---|---|---|---|
| TITLE | **A Randomized, Double-Blind, Double-Dummy, Parallel-Group Study To Evaluate The Efficacy And Safety Of Ocrelizumab In Comparison To Interferon Beta-1a (Rebif®) In Patients With Relapsing Multiple Sclerosis** | | |
| SPONSOR | F.Hoffmann-La Roche Ltd<br>Genentech Inc | CLINICAL PHASE | III |
| INDICATION | Relapsing Multiple Sclerosis | | |
| OBJECTIVES | **Primary:**<br>The primary objective of this study is to assess whether the efficacy of ocrelizumab 600 mg (given as dual infusions of 300 mg on Days 1 and 15 of the first 24-week treatment cycle and as a single infusion of 600 mg on Day 1 of each 24-week treatment cycle thereafter) intravenously every 24 weeks is superior to Rebif® as measured by the annualized protocol-defined* relapse rate by two years (96 weeks) in patients with relapsing multiple sclerosis.<br>**Secondary:**<br>To evaluate whether the efficacy of ocrelizumab is superior to Rebif®, as reflected by the following measures:<br>• The time to onset of sustained disability progression for at least 12 weeks during the 96-week comparative treatment period.*<br>• The time to onset of sustained disability progression for at least 24 weeks during the 96-week comparative treatment period.*<br>• The proportion of relapse-free patients by 96 weeks.<br>• The change in total T2 lesion volume as detected by brain MRI from baseline to Week 96.<br>• The total number of new, and/or enlarging T2 hyperintense lesions as detected by brain MRI at weeks 24, 48 and 96.<br>• The change in Multiple Sclerosis Functional Composite Scale (MSFCS) score from baseline to Week 96.<br>• The change in brain volume as detected by brain MRI from Week 24 to Week 96.<br>**See section: "Assessments of efficacy" for the definition*<br>**Safety:**<br>To evaluate the safety and tolerability of ocrelizumab 600 mg (given as dual infusions of 300 mg on Days 1 and 15 of the first 24-week treatment cycle and as a single infusion of 600 mg on Day 1 of each 24-week treatment cycle thereafter) intravenously every 24 weeks in patients with relapsing MS.<br>**Pharmacokinetics/Pharmacodynamics:**<br>To explore the pharmacokinetics, immunogenicity and pharmacodynamics of ocrelizumab in patients with relapsing MS.<br>**Exploratory objectives:**<br>• The change in low contrast visual acuity from baseline to Weeks 48 and 96.<br>• The change in the Symbol Digit Modalities Test from baseline to Weeks 48 and 96.<br>• The annualized relapse rate, based on clinical and protocol-defined relapses at the end of the 96-week comparative treatment period.<br>• The total number of T1 gadolinium-enhanced lesions as detected by brain MRI at weeks 24, 48 and 96. | | |

| | |
|---|---|
| | • The change in brain volume as detected by brain MRI from baseline to Week 96.<br>• The change in brain volume as detected by brain MRI from week 48 to Week 96.<br>• The change in Multiple Sclerosis Functional Composite Scale (MSFCS) score from baseline to Week 48.<br>• The cumulative change in EDSS scores, measured in area under the curve (AUC) by Week 96.<br>• The change in EDSS from baseline to Weeks 48 and 96.<br>• The change in timed 25 foot walk from baseline to Weeks 48 and 96.<br>• The change in 9-hole peg test from baseline to Weeks 48 and 96.<br>• The change in paced auditory serial addition test (PASAT) from baseline to Weeks 48 and 96.<br>• The time to onset of sustained 20% increase in 9-hole peg test for at least 12 weeks.<br>• The time to onset of sustained 20% increase in timed 25 foot walk for at least 12 weeks.<br>• Patient Reported Outcomes (PROs): Modified Fatigue Impact Scale (MFIS), EuroQol instrument (EQ-5D), Center for Epidemiology Studies Depression Scale (CES-D) and Medical Outcomes Study 36-Item Short Form Survey Instrument (SF-36) at baseline, Week 48 and Week 96.<br>• The change in Karnofsky Performance Status Scale (clinician-reported version) from baseline to Weeks 48 and 96. |
| TRIAL DESIGN | Multicentre, randomized, double-blind, double-dummy, parallel-group study |
| NUMBER OF SUBJECTS | 800 patients in total, 400 patients per group using a 1:1 randomization ratio. Please refer to Sample Size and Statistical Analyses section of the synopsis for more details. |
| TARGET POPULATION | **Inclusion criteria:**<br>1. Ability to provide written, informed consent and to be compliant with the schedule of protocol assessments.<br>2. Ages 18-55 years at screening, inclusive.<br>3. Diagnosis of MS, in accordance with the revised McDonald criteria (2010).<br>4. At least 2 documented clinical attacks within the last 2 years prior to screening or one clinical attack in the year prior to screening (but not within 30 days prior to screening).<br>5. Neurological stability for ≥ 30 days prior to both screening and baseline.<br>6. EDSS, at screening, from 0 to 5.5 inclusive.<br>7. Documented MRI of brain with abnormalities consistent with MS prior to screening.<br>8. Patients of reproductive potential must use reliable means of contraception as described below as a minimum (adherence to local requirements, if more stringent, is required*):<br>• Two methods of contraception throughout the trial, including the active treatment phase AND for 48 weeks after the last dose of ocrelizumab, or until their B-cells have repleted, whichever is longer. Acceptable methods of contraception include one primary (e.g. systemic hormonal contraception or tubal ligation of the female partner, vasectomy of the male partner) AND one secondary barrier method (e.g. latex condoms, spermicide) OR a double barrier method (e.g. latex condom, intrauterine device, vaginal ring or pessary plus spermicide [e.g. foam, vaginal suppository, gel, cream]). |

9. For patients of non reproductive potential (adherence to local requirements, if more stringent, is required*):
   - Women may be enrolled if postmenopausal (i.e. spontaneous amenorrhea for the past year confirmed by an FSH level greater than 40 mIU/mL) unless the patient is receiving a hormonal therapy for their menopause or surgically sterile (i.e. hysterectomy, complete bilateral oophorectomy);
   - Men may be enrolled if they are surgically sterile (castration).

* Based on local Ethics Committees or National Competent Authority feedback additional requirements to assure contraception or to confirm menopause may be required (e.g. serum estradiol compatible with post-menopause status, longer duration of amenorrhea, higher level of FSH).

**Exclusion Criteria**

Patients who meet the following criteria must be excluded from study entry:

1. Diagnosis of primary progressive MS.
2. Disease duration of more than 10 years in patients with an EDSS $\leq 2.0$ at screening.
3. Inability to complete an MRI (contraindications for MRI include but are not restricted to claustrophobia, weight $\geq$ 140 kg, pacemaker, cochlear implants, presence of foreign substances in the eye, intracranial vascular clips, surgery within 6 weeks of entry into the study, coronary stent implanted within 8 weeks prior to the time of the intended MRI, etc).
4. Known presence of other neurological disorders which may mimic MS including but not limited to: neuromeylitis optica, Lyme disease, untreated vitamin B12 deficiency, neurosarcoidosis and cerebrovascular disorders.

**Exclusions Related to General Health**

5. Pregnancy or lactation.
6. Any concomitant disease that may require chronic treatment with systemic corticosteroids or immunosuppressants during the course of the study.
7. History or currently active primary or secondary immunodeficiency.
8. Lack of peripheral venous access.
9. History of severe allergic or anaphylactic reactions to humanized or murine monoclonal antibodies.
10. Significant or uncontrolled somatic disease or any other significant disease that may preclude patient from participating in the study.
11. Congestive heart failure (NYHA III or IV functional severity).
12. Known active bacterial, viral, fungal, mycobacterial infection or other infection, excluding fungal infection of nail beds.
13. Infection requiring hospitalization or treatment with i.v. antibiotics within 4 weeks prior to baseline visit or oral antibiotics within 2 weeks prior to baseline visit.
14. History or known presence of recurrent or chronic infection (e.g., hepatitis B or C, HIV, syphilis, tuberculosis).
15. History of progressive multifocal leukoencephalopathy (PML)
16. History of malignancy, including solid tumors and hematological malignancies, except basal cell carcinoma, *in situ* squamous cell carcinoma of the skin, and *in situ* carcinoma of the cervix of the uterus that have been previously completely excised with documented, clear margins.
17. History of alcohol or drug abuse within 24 weeks prior to baseline.
18. History or laboratory evidence of coagulation disorders.

**Exclusions Related to Medications***

19. Receipt of a live vaccine within 6 weeks prior to baseline.
    *In rare cases when patient requires vaccination with a live vaccine, the screening period may be extended but cannot exceed 8 weeks.*
20. Treatment with any investigational agent within 24 weeks of screening (Visit 1) or five half-lives of the investigational drug (whichever is longer).
21. Contraindications to or intolerance of oral or i.v. corticosteroids, including methylprednisolone administered i.v., according to the country label, including:
    a) Psychosis not yet controlled by a treatment;
    b) Hypersensitivity to any of the constituents.
22. Contraindication to Rebif® or incompatibility with Rebif® use, including:
    a) Current severe depression and/or suicidal ideation;
    b) Hypersensitivity to natural or recombinant interferon-β, or to any excipients;
    c) Previous suboptimal response to High Dose High Frequency (HDHF) interferon or cessation of HDHF interferon therapy due to poor tolerability;
    d) Prior cessation of Rebif® therapy due to toxicity, which is likely to recur.
23. Treatment with β interferons (with exemptions for HDHF interferon as listed above), glatiramer acetate, plasmapheresis, or other immunomodulatory therapies within 4 weeks prior to baseline.
24. Treatment with dalfampridine (Ampyra®) unless on stable dose for ≥ 30 days prior to screening. Patients should remain on stable doses throughout the 96 week treatment period.
25. Previous treatment with B-cell targeted therapies (i.e. rituximab, ocrelizumab, atacicept, belimumab or ofatumumab).
26. Systemic corticosteroid therapy within 4 weeks prior to screening.**
27. Any previous treatment with alemtuzumab (Campath), anti-CD4, cladribine, mitoxantrone, daclizumab, BG12, teriflunomide, laquinimod, total body irradiation or bone marrow transplantation.
28. Treatment with cyclophosphamide, azathioprine, mycophenolate mofetil (MMF), cyclosporine, methotrexate or natalizumab within 24 months prior to screening. NB. P*atients previously treated with natalizumab will be eligible for this study only if duration of treatment with natalizumab was < 1 year.*
29. Treatment with fingolimod (FTY720, Gilenya®) or other S1P receptor modulator (i.e. BAF312) within 24 weeks prior to screening. NB. *Only patients with T lymphocyte count ≥ LLN will be eligible for this study.*
30. Treatment with i.v. immunoglobulin within 12 weeks prior to baseline.

* *Patients screened for this study should not be withdrawn from therapies for the sole purpose of meeting eligibility for the trial. Patients, who discontinue their current therapy for non-medical reasons, should specifically be informed before deciding to enter the study of their treatment options.*

** *The screening period may be extended (but cannot exceed 8 weeks) for patients who have used systemic corticosteroids for their MS before screening. For a patient to be eligible, systemic corticosteroids should not have been administered also between screening and baseline.*

| | |
|---|---|
| | **Exclusions Related to Laboratory Findings*** |

31. Positive serum β hCG measured at screening.
32. Positive screening tests for hepatitis B (hepatitis B surface antigen [HBsAg] positive, or positive hepatitis B core antibody [total HBcAb] confirmed by a positive viral deoxyribonucleic acid [DNA] polymerase chain reaction [PCR]) or hepatitis C (HepCAb).
33. Positive rapid plasma reagin (RPR).
34. CD4 count < 300/µL.
35. AST/SGOT or ALT/SGPT ≥ 2.0 Upper Limit of Normal (ULN).
36. Platelet count <100,000/µL (<100 x $10^9$/L).
37. Levels of serum IgG <5.65 g/L.
38. Levels of serum IgM < 0.55 g/L.
39. Total neutrophil count <1.5 x $10^3$/µL.

*Re-testing before baseline: in rare cases in which the screening laboratory samples are rejected by the central laboratory (example: hemolyzed sample) or the results are not assessable (example: indeterminate) or abnormal, the tests need to be repeated within 4 weeks. The last value before randomization must meet study criteria. In such circumstances, the screening period may need to be prolonged but should not exceed 8 weeks.*

Please note: based on local Ethics Committees or National Competent Authority requirements, additional diagnostic testing may be required for selected patients or selected centers to exclude tuberculosis, Lyme disease, HTLV-1 associated myelopathy (HAM), acquired immune deficiency syndrome (AIDS), hereditary disorders, connective tissue disorders, or sarcoidosis. Other specific diagnostic tests may be requested when deemed necessary by the investigator**.**

---

LENGTH OF STUDY

The study will consist of the following periods:
- **Screening period**: 2 weeks.

*In rare cases (i.e. if the screening laboratory samples are rejected by the central laboratory) the screening period may be extended but cannot exceed 8 weeks.*

- **Treatment period:** 96 weeks

The double-blind comparative treatment period will consist of 4 treatment cycles 24 weeks apart.

*Patients who complete the 96 week treatment period may become eligible for a separate, open-label extension study, under a separate protocol.*

-**Safety Follow up Period**: at least 48 weeks;

Patients who withdraw from study treatment will be observed for a period of at least 48 weeks counting from the date of the last infusion of study drug. If at this time the peripheral blood B-cells remain depleted patient should continue to be monitored at 24-week intervals until B-cell count has returned to the baseline value or to the lower limit of the normal range (whichever is the lower).

See section “Procedures” below for more details.

| | |
|---|---|
| END OF STUDY | The end of the study has been defined as the date at which the last data point from the last patient, which was required for statistical analysis as defined in Data Analysis Plan (DAP), was received. |
| INVESTIGATIONAL MEDICAL PRODUCT(S) DOSE/ ROUTE/ REGIMEN | – Group A (Ocrelizumab): Ocrelizumab 600 mg (given as dual infusions of ocrelizumab 300 mg on Days 1 and 15 of the first 24-week treatment cycle and as single infusions of 600 mg on Day 1 for each 24-week treatment cycle, thereafter) every 24 weeks.<br>– Group B (Interferon beta-1a (Rebif®))<br>Rebif® will be administered as follows:<br>▪ Treatment initiation:<br>⇒ during weeks one and two, Rebif® 8.8 µg (one pre-filled syringe (0.2 mL) containing 8.8 µg (2.4 MIU) of interferon beta-1a given via subcutaneous injection three times per week<br>⇒ during weeks three and four, Rebif® 22 µg (one pre-filled syringe (0.5 mL) containing 22 µg (6 MIU) of interferon beta-1a) given via subcutaneous injection three times per week<br>▪ Treatment continuation:<br>⇒ From the fifth week onwards, Rebif® 44 µg (one pre-filled syringe (0.5 mL) containing 44 µg (12 MIU) of interferon beta-1a) given via subcutaneous injection three times per week<br>⇒ A lower dose of 22 µg, given via subcutaneous injection three times per week, will be available for patients who cannot tolerate the 44 µg dose of Rebif®. *Please refer to detailed guidelines in the study protocol.*<br><br>Patients randomized to active ocrelizumab group will also receive dummy placebo of Rebif® (administered via subcutaneous injection three times per week).<br>Patients randomized to active Rebif® group will also receive dummy placebo of ocrelizumab (administered as intravenous infusions at similar time points to those of the ocrelizumab group).<br>Dummy placebos of ocrelizumab and of Rebif® will be similar in appearance and administration as the investigational product. |
| NON-INVESTIGATIONAL MEDICAL PRODUCT(S) | Ocrelizumab/ocrelizumab dummy placebo: Thirty minutes prior to the start of each infusion, patients will receive a methylprednisolone 100 mg i.v. infusion. Pre-infusion treatment with an oral analgesic/antipyretic (e.g.: acetaminophen), and an oral antihistamine (e.g. diphenhydramine) is also recommended.<br>Rebif®/ Rebif® dummy placebo: Pre-treatment with non-steroid anti-inflammatory drugs (ibuprofen) or acetaminophen are recommended; investigators should follow their local label for further information. |
| ASSESSMENTS OF:<br>- EFFICACY | – **Assessment of clinical and protocol defined relapses**<br>▪ Protocol-defined relapse is the occurrence of new or worsening neurological symptoms attributable to MS. Symptoms must persist for >24 hours and should not be attributable to confounding clinical factors (e.g. fever, infection, injury, adverse reactions to medications) and immediately preceded by a stable or improving neurological state for least 30 days. The new or worsening neurological symptoms must be accompanied by objective neurological worsening consistent with an increase of at least half a step on the EDSS scale, or 2 points on one of the appropriate FSS, or 1 point on two or more of the appropriate FSS. The change must affect the selected FSS (i.e., pyramidal, ambulation, cerebellar, brainstem, sensory, or visual). Episodic spasms, sexual |

dysfunction, fatigue, mood change or bladder or bowel urgency or incontinence will not suffice to establish a relapse. NB: Sexual dysfunction and Fatigue will not be scored.

⇨ *Please note: adjudication of protocol-defined relapses will be performed by the Sponsor based on pre-specified criteria, applied to data collected by investigator, in a blinded fashion.*

– **Brain MRI acquisition sequences**
  - T1-weighted MRI (without gadolinium-enhancement)
  - T1-weighted MRI (with gadolinium-enhancement)
  - Fluid-attenuated, inversion recovery (FLAIR), Proton-density–weighted and/or T2-weighted MRI

– **Assessment of sustained disability progression**
  - Disability progression is defined as an increase of ≥ 1.0 point from the baseline EDSS score that is not attributable to another etiology (e.g. fever, concurrent illness, or concomitant medication) when the baseline score is 5.5 or less, and ≥ 0.5 when the baseline score is above 5.5. Disability progression is considered sustained when the increase in the EDSS is confirmed at regularly scheduled visits at least 12 and 24 weeks after the initial documentation of neurological worsening.
  - Sustained disability progression, confirmed for both 12 and 24 weeks after the initial documentation of neurological worsening, will be analyzed.

The following instruments will be used to assess the patient's functional ability: Low Contrast Letter Acuity Charts (LCVA/Sloan charts), Symbol Digit Modalities Test (SDMT), Kurtzke Expanded Disability Status Scale (EDSS), Multiple Sclerosis Functional Composite Scale (MSFCS) and Karnofsky Performance Status Scale (clinician-reported version).

- SAFETY

Adverse events, vital signs, weight, physical and neurological examination, clinical laboratory tests, 12 lead ECG, locally reviewed MRI for safety (non-MS CNS pathology), concomitant medications.

Pregnancy tests [serum/urine beta subunit human chorionic gonadotropin (beta hCG)] will be performed in women of childbearing potential. Plasma and urine samples will be collected for JCV assessments.

Columbia-Suicide Severity Rating Scale (C-SSRS) will be used for prospective suicidality assessment.

- PHARMACOKINETICS/ PHARMACODYNAMICS

Blood samples will be taken for measurement of ocrelizumab serum concentration at the time points detailed in the Schedule of Assessments. Nonlinear mixed effects modeling will be used to analyze the sparse sampling dose-concentration-time data of ocrelizumab in order to assess the pharmacokinetics. Population PK parameters will be estimated and the influence of covariates, such as age, gender, weight, HAHA, and CD19 lymphocyte count, investigated. The relationship between ocrelizumab exposure and selected safety and efficacy parameters will be analyzed and explored in order to characterize the exposure-response curve of ocrelizumab.

–IMMUNOGENICITY

Serum samples for measurement of HAHA (Human Anti-human Antibodies) to ocrelizumab are collected according to the Schedule of Assessments.

| | |
|---|---|
| – PATIENT REPORTED OUTCOMES (PRO) | ▪ Modified Fatigue Impact Scale (MFIS Standard)<br>▪ Center for Epidemiology Studies Depression Scale (CES-D3)<br>▪ Medical Outcomes Study 36-Item Short Form Survey Instrument (SF-36: SF-36v2 Standard)<br>▪ EuroQol instrument (EQ-5D-3L-Tablet) |
| – PROTEIN BIOMARKER SAMPLES | Specimens for protein biomarker discovery and validation will be collected from all patients. These specimens will be used for research purposes to identify and/or verify protein biomarkers that are predictive of response to ocrelizumab treatment (in terms of dose, safety and tolerability) and will help understand the pathogenesis, course and outcome of multiple sclerosis and related diseases. In addition, screening technologies for larger numbers of proteins and antibodies may also be used to discover novel antibody associations with MS, disease progression and response to therapy.<br><br>Analyses will include but may not be limited to Complement Factor H (CFH) and the B-cell activating factor (BAFF).<br><br>6 mL sample of whole blood will be collected in a plain tube without EDTA for serum isolation. Blood specimens for protein biomarker discovery and validation will be collected from all patients as per Schedule of Assessments. These specimens will be stored for 5 years after the end of the study and then destroyed. |
| - EXPLORATORY BIOMARKERS (non-DNA) | Roche Clinical Repository (RCR) non-DNA, (dynamic, non-inherited) RNA specimen and plasma for biomarker discovery and validation will be collected only from patients consenting to RCR as per Schedule of Assessments. RCR samples (2 x approximately 2.5 mL of blood collected in PAXgene vacutainers and 6 ml of blood collected in a tube with EDTA for plasma isolation) will be collected to promote, facilitate and improve individualized healthcare by better understanding/predicting ocrelizumab efficacy, dose responses, safety, ocrelizumab mode of action, progression of multiple sclerosis and associated diseases. These specimens may be stored for up to 15 years after the end of the study. |
| - EXPLORATORY BIOMARKERS (DNA) | All patients who have been enrolled in the study will be asked to donate an optional DNA specimen (by written informed consent) for pharmacogenetic and genetic research.<br><br>RCR DNA sampling will involve taking one sample of 6 mL of blood taken as per Schedule of Assessments. The study protocol which includes RCR sampling is submitted to the concerned Ethics Committee and is available for Competent Authority review upon request. These specimens will be stored for up to 15 years after the end of the study. |

PROCEDURES (summary):

**Figure 1 Overview of Study Design**

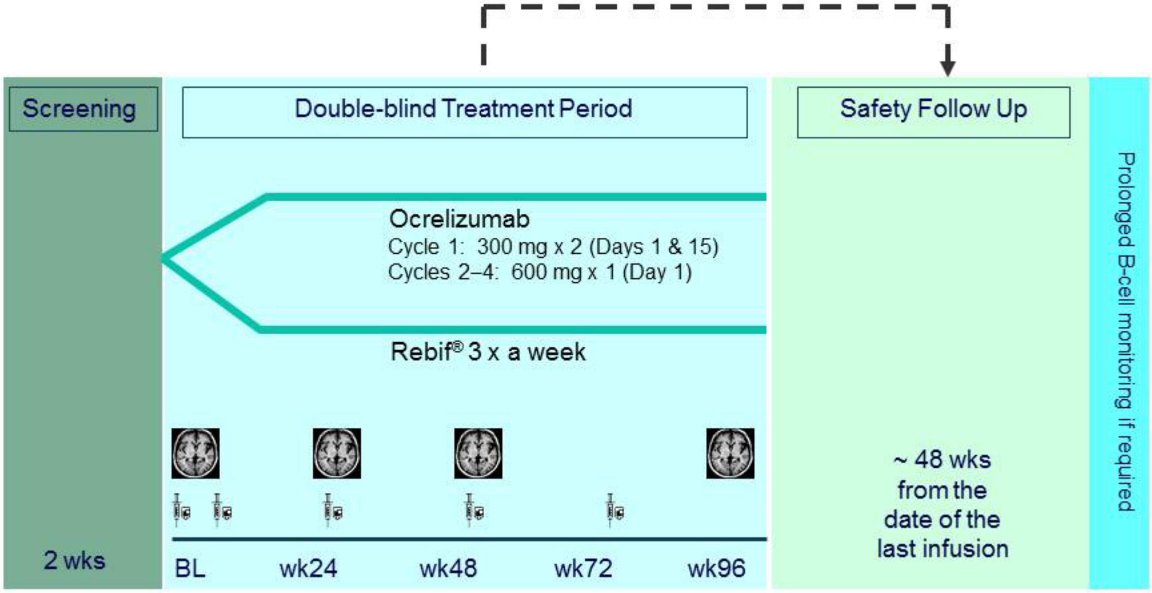


Please note: patients who complete the 96 week treatment period may become eligible for a separate, open-label extension study, under a separate protocol.

## Screening:

Consenting patients will enter the 2-week screening period to be evaluated for eligibility. Procedures at screening will include collecting medical history, medical examination including thorough neurological exam, EDSS score, MSFCS score, ECG, blood and urine sampling. Please see Table 3 - "Schedule of Assessments: Screening Through the End of Double-Blind Treatment Period" for further details.

*Please note that based on local Ethics Committees or National Competent Authority requirements, additional diagnostic testing may be required for selected patients or selected centers to exclude tuberculosis, Lyme disease, HTLV-1 associated myelopathy (HAM), acquired immune deficiency syndrome (AIDS), hereditary disorders, connective tissue disorders, or sarcoidosis.*

## Treatment Period:

Eligible patients will be randomized via IxRS into one of two treatment groups: ocrelizumab 600 mg regimen (group A) or interferon beta-1a (Rebif®) (group B) – please refer to Table 1 and Table 2 for more details.

To prevent potential unblinding as a result of adverse events or changes to laboratory results, the following, additional measures have been implemented:

– **The Examining Investigator/EDSS assessor** will perform the neurological examination, document the FSS scores and assess EDSS scores. The examining investigator will be also responsible for performing and documenting results from: MSFCS, the Karnofsky Performance Status Scale, low contrast visual acuity testing and the Symbol Digit Modalities Test. He or she will have access only to data from assessements listed above. The examining physician/EDSS assessor will not be involved with any aspect of medical management of the patient and will not have access to patient data. Every effort will be made to ensure that there is no change in the examining physician/EDSS assessor throughout the course of the study for any individual patient. The examining physician/EDSS assessors will be trained and instructed not to discuss what adverse effects (if any) the patient is experiencing from their medication. Examining physician/EDSS assessors will receive training in performing EDSS assessments prior to the beginning of the study and must have successfully passed an examination on performance of the *Neurostatus EDSS examination* within 24 months of participation. All examining physician/EDSS assessors will receive ongoing training on performance of the *Neurostatus EDSS examination* throughout the course of the study.

– **Patient education:** prior to being examined by the Examining Investigator/EDSS assessor, patients will be instructed not to discuss what (if any) adverse effects they may be experiencing. Treating physicians and/or study coordinators should remind patients of these instructions prior to EDSS assessments and this should be documented in the source documents.

– **Blinded, central MRI assessments:** a blinded, central MRI reader will assess all on-study MRI scans. These assessments will provide independent confirmation of the relative changes in immune-mediated, CNS damage.

**Blinding of laboratory parameters:** laboratory parameters which may lead to unblinding to treatment assignment, such as FACS cell counts including $CD19^+$ cells, lymphocyte count, IgM and IgG levels and type I interferon neutralizing antibody levels will be blinded in all patients. In order to ensure patients' safety in the study and to allow for assessments of the re-treatment criteria, a central laboratory will provide study investigators and Medical Monitors with reflex messages triggered by critical blinded laboratory results. Investigators notified of their patient's critical laboratory test results will be instructed to suspend further treatment with study drug until the patient becomes eligible for re-treatment. During the treatment period, patients will be assessed at clinical visits as per Schedule of Assessments: Screening Through the End of Treatment Period – please refer to Table 3 for further details.

Prior to the next infusion of study drug, patients will be evaluated for pre-specified conditions and laboratory abnormalities to allow for re-treatment.

**Patients who complete the 96 week treatment period may become eligible for a separate open-label extension study, under a separate protocol.**

**<u>Please note:</u> patients who discontinue from study medication within the 96-week double-blind, comparative phase (treatment period) of the study will enter the Safety Follow up Period (see below); they will not be eligible for the open-label extension study, even if they complete the 96-week treatment period.**

## <u>Safety Follow up Period</u>

Patients who discontinue treatment with study drug will enter the Safety Follow up Period for at least 48 weeks counting from the date of the last infusion of the ocrelizumab/placebo. However, if after this time the peripheral blood B-cells remain depleted, patient should continue to be monitored at 24-week intervals until B-cell count has returned to the baseline value or to the lower limit of the normal range (whichever is the lower). Please refer to Figure 2 for more details.

**Figure 2 Safety Follow up – Prolonged B-cell Monitoring Period**

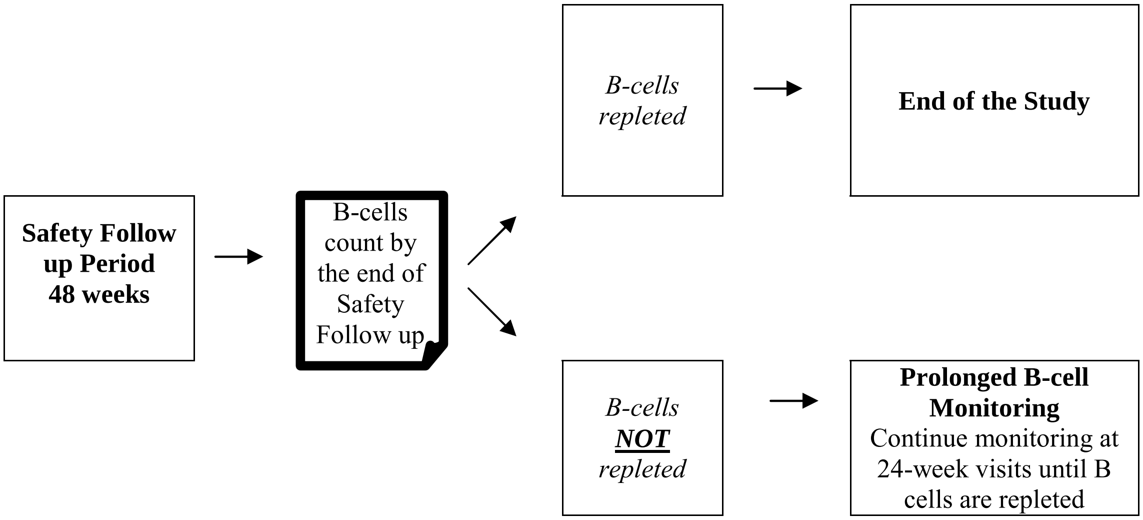


*Please note: patients in Safety Follow up who receive other B-cell targeted therapies will only be followed for 48 weeks from the date of the last infusion of the study drug regardless of their B-cell count.*

During Safety Follow up patients will be assessed at clinical visits every 12 weeks as per Schedule of Assessments. Telephone interviews will be performed every 4 weeks. If prolonged B-cell monitoring is required, patients will be assessed at clinical visits every 24 weeks (as per Schedule of Assessments) and telephone interviews will be performed every 12 weeks. Please refer to Table 4 for further details.

Please note: It is important to distinguish between <u>"withdrawal from treatment"</u> and <u>"withdrawal from study"</u>. Patients who withdraw from treatment should be encouraged to remain in the study for the full duration of the Safety Follow Up Period (minimum of 48 weeks following the last infusion).

**Every effort should be made to have patients, who withdraw from the study treatment, complete the Safety Follow up Period and all related assessments, regardless of whether or not they receive alternative treatment for MS.**

### Table 1: Overview of Dosing Regimen

| **Group** | **Treatment Period**[1,2] | | | | |
|---|---|---|---|---|---|
| | **1st Cycle**[3] (Weeks 1-24) | | **2nd Cycle**[3] (Weeks 24-48) | **3rd Cycle**[3] (Weeks 48-72) | **4th Cycle**[3] (Weeks 72-96) |
| | **Day 1 Infusion** | **Day 15 Infusion** | **Week 24 Infusion** | **Week 48 Infusion** | **Week 72 Infusion** |
| **A Ocrelizumab 600 mg regimen** | **Ocrelizumab** 300 mg i.v. | **Ocrelizumab** 300 mg i.v. | **Ocrelizumab** 600 mg i.v. | **Ocrelizumab** 600 mg i.v. | **Ocrelizumab** 600 mg i.v. |
| **B Rebif® regimen**[4] | **Rebif®** s.c. three times per week | → | → | → | → |

1. The treatment period consists of 96 weeks of treatment; patients will receive a maximum of 4 treatment cycles.
2. Each treatment cycle has a duration of 24 weeks. The first cycle consists of two 300 mg ocrelizumab i.v. infusions separated by 14 days. Cycles 2 – 4 consist of a single i.v. infusion of 600 mg ocrelizumab.
3. Prior to each infusion, a clinical evaluation will be performed to ensure that the patient remains eligible for treatment.
4. Please refer to Table 2 for detailed Rebif® dosing regimen.

Please note: 100 mg of methylprednisolone i.v. will be administered in both treatment arms prior to each infusions of ocrelizumab/ocrelizumab placebo.

**Table 2: Overview of Rebif® Dosing Regimen**

| | **Treatment Initiation** | | **Treatment Continuation** | **Dose modification (if required)** |
|---|---|---|---|---|
| **Week** | **Weeks 1- 2** | **Weeks 3-4** | **Week 5 onwards** | – |
| *Study Day* | *1-14* | *15-28* | *29+* | At any time >29 |
| **Dose of Rebif®** | **Rebif® 8.8µg** (one pre-filled syringe [0.2 mL] containing 2.4 MIU of interferon beta-1a) s.c. **3x per week** | **Rebif® 22 µg** (one pre-filled syringe [0.5 mL] containing 6 MIU of interferon beta-1a) s.c. **3x per week** | **Rebif® 44 µg** (one pre-filled syringe [0.5 mL]) containing 12 MIU of interferon beta-1a) s.c. **3x per week** | **Rebif® 22 µg** (one pre-filled syringe [0.5 mL] containing 6 MIU of interferon beta-1a) s.c. **3x per week** |

Please note: if Rebif® dose modification is required due to laboratory abnormalities possibly related to the treatment with Rebif®, the investigator (the treating physician) will need to notify IxRS and the blinded study medication (Rebif® placebo or Rebif® verum) will be dispensed accordingly. In addition, to ensure patient safety in the study, unscheduled visits may be required for additional assessments, monitoring and for dispensing study medication.

**In case of elevation of liver function tests the following rules will apply:**

| | |
|---|---|
| ⇨ **ALT ≥ 10 x ULN**, jaundice or other clinical symptoms of liver dysfunction | In case of detection of elevated **ALT ≥ 10 x ULN**, jaundice or other clinical symptoms of liver dysfunction the injections of Rebif®/Rebif® placebo must be discontinued permanently. The monitoring of liver function tests should be continued on a monthly basis until return to normal baseline levels or CTCAE v.4.0 grade 1 toxicity (ALT: >ULN - 3.0 x ULN). A consultation with hepatologist is recommended. Patients should move to Safety Follow up Period. |

| | |
|---|---|
| ⇨ **ALT ≥ 5 x ULN** | In case of detection of elevated **ALT ≥ 5 x ULN** (but below 10 xULN) the injections of Rebif®/Rebif® placebo must be discontinued temporarily. Additional blood chemistry panel including AST, ALP, GGT and bilirubin should be performed biweekly until no further increase is observed. Subsequently, ALT analysis has to be performed every month until return to normal baseline levels or CTCAE v.4.0 grade 1 toxicity (ALT >ULN - 3.0 x ULN). A consultation with hepatologist should be considered as per investigator judgment.<br>If causes of toxicity other than possible treatment with Rebif® are excluded, the patient may then be cautiously re-challenged with Rebif®/Rebif® placebo 22µg provided in a blinded fashion upon request to IxRS. The monitoring of liver function tests should continue on a monthly basis. If there is no further recurrence of toxicity, patient may continue treatment with Rebif®/Rebif® placebo 44 µg provided in a blinded fashion upon investigator's request to IxRS. **In case of recurrence of toxicity (ALT > 3 x ULN, or other clinical symptoms of liver dysfunction) the injections of Rebif®/Rebif® placebo should be discontinued permanently.** Patients should move to Safety Follow up Period.<br><br>Please note: Re-initiation of therapy with Rebif® following elevation of liver function tests can only be considered once. |
| ⇨ **ALT > 3 x ULN** | In case of detection of elevated **ALT > 3 x ULN** (but below 5x ULN) additional blood chemistry panel including AST, ALP, GGT and bilirubin should be performed biweekly until no further increase is observed. Subsequently, ALT analysis has to be performed every month until return to normal baseline levels or CTCAE v.4.0 grade 1 toxicity (ALT>ULN - 3.0 x ULN). |

## Table 3: Schedule of Assessments: Screening Through the End of Double-Blind Treatment Period

| | Screen | Treatment Period | | | | | | | | | | | | |
|---|---|---|---|---|---|---|---|---|---|---|---|---|---|---|
| **Visit** | 1 | 2 BL | 3 | 4 | 5 | 6 | 7 | 8 | 9 | 10 | 11 | Delayed Dosing Visit 22 | Unscheduled Visit 23 | Withdrawal from Treatment Visit |
| **Week** | -2 | - | w2 | w12 | w24 | w36 | w48 | w60 | w72 | w84 | w96 | | | |
| *Study Day (window in days)* | *-14* | *1* | *15 (± 1)* | *85 (±4)* | *169 (±1)* | *253 (±4)* | *337 (±1)* | *421 (±4)* | *505 (±1)* | *589 (±4)* | *673 (±1)* | | | |
| | | | | | | | | | | | | | | |
| Informed consent [1] | x | | | | | | | | | | | | | |
| Medical history | x | | | | | | | | | | | | | |
| Review of eligibility criteria | x | x | | | | | | | | | | | | |
| CES-D, MFIS, EQ-5D, SF-36 | | x | | | | | x | | | | x | | | x |
| Patient's Assessment of Treatment Benefit | | | | | | | x | | | | x | | | x |
| C-SSRS | | x | | x | x | x | x | x | x | x | x | | x | x |
| Physical examination | x | x | x | | x | | x | | x | | x | x | | x |
| Vital signs [2] | x | x | x | x | x | x | x | x | x | x | x | x | x | x |
| 12 lead ECG (pre- and post-dose) [3] | x | x | | | | | | | x | | | | | x |
| Height | x | | | | | | | | | | | | | |
| Weight | x | | | | | | | | x | | x | | | x |
| Neurological exam and EDSS | x | x | | x | x | x | x | x | x | x | x | | x | x |
| MSFCS, LCVA, SDMT | | x | | x | x | x | x | x | x | x | x | | x | x |
| Karnofsky Performance Status Scale | | x | | | x | | x | | x | | x | | | x |
| MRI[4] | | x | | | x | | x | | | | x | | | x |
| Concomitant Treatment | | x | x | x | x | x | x | x | x | x | x | x | x | x |
| Adverse Events | *Only SAEs* | x | x | x | x | x | x | x | x | x | x | x | x | x |
| Potential relapses recorded | | x | x | x | x | x | x | x | x | x | x | x | x | x |
| Telephone interview (every 4 wks)[5] | x | | | – – – – | – – – – | – – – – | – – – – | – – – – | – – – – | – – – – | – – → | | | x |

## Table 3: Schedule of Assessments: Screening Through the End of Double-Blind Treatment Period (Cont.)

| | Screen | Treatment Period | | | | | | | | | | | | |
|---|---|---|---|---|---|---|---|---|---|---|---|---|---|---|
| **Visit** | 1 | 2 BL | 3 | 4 | 5 | 6 | 7 | 8 | 9 | 10 | 11 | Delayed Dosing Visit [22] | Unscheduled Visit [23] | Withdrawal from Treatment Visit |
| **Week** | -2 | - | w2 | w12 | w24 | w36 | w48 | w60 | w72 | w84 | w96 | | | |
| *Study Day (window in days)* | *-14* | *1* | *15 (± 1)* | *85 (±4)* | *169 (±1)* | *253 (±4)* | *337 (±1)* | *421 (±4)* | *505 (±1)* | *589 (±4)* | *673 (±1)* | | | |
| Pregnancy test [6] | x | x | x | x | x | x | x | x | x | x | x | x | | x |
| Antibody Titers [7] | | x | | x | x | | x | | x | | x | | | x |
| RCR (non-DNA) [8] | | x | | x | x | | x | | x | | x | | | x |
| RCR (DNA) [9] | | x | | | | | | | | | | | | |
| Protein biomarker sampling [10] | | x | | x | x | | x | | x | | x | | | x |
| HAHA [11] | | x | | | x | | x | | x | | x | | | x |
| Plasma/ urine banking for JCV[12] | | x | | x | x | x | x | x | x | x | x | | | x |
| PK Samples [13] | | x | | | x | | x | | x[13] | x | x | | | x |
| Thyroid function tests [14] | x | | | | x | | x | | x | | x | | | x |
| FSH [15] | x | | | | | | | | | | | | | |
| Hepatitis Screening [16] | x | | | | | | | | | | | | | |
| Hepatitis B virus DNA[16] | x | (x) | | (x) | (x) | (x) | (x) | (x) | (x) | (x) | (x) | | | (x) |
| RPR | x | | | | | | | | | | | | | |
| CD4 count | x | | | x | | x | | x | | x | | | | |
| IgG | | | | x | | x | | x | | x | | | | |
| Total Ig, IgA, IgG, IgM | x | | | | x | | x | | x | | x | | | x |
| FACS [17] | | x | x | x | x | | x | | x | | x | | | x |
| Routine safety lab [18] | x | x | x | x | x | x | x | x | x | x | x | | | x |
| Type I interferon neutralizing antibody assay [19] | | x | | | x | | x | | x | | x | | | x |

## Table 3: Schedule of Assessments: Screening Through the End of Double-Blind Treatment Period (Cont.)

| | Screen | Treatment Period | | | | | | | | | | | | |
|---|---|---|---|---|---|---|---|---|---|---|---|---|---|---|
| **Visit** | **1** | **2 BL** | **3** | **4** | **5** | **6** | **7** | **8** | **9** | **10** | **11** | **Delayed Dosing Visit** [22] | **Unscheduled Visit** [23] | **Withdrawal from Treatment Visit** |
| **Week** | **-2** | **-** | **w2** | **w12** | **w24** | **w36** | **w48** | **w60** | **w72** | **w84** | **w96** | | | |
| *Study Day (window in days)* | *-14* | *1* | *15 (± 1)* | *85 (±4)* | *169 (±1)* | *253 (±4)* | *337 (±1)* | *421 (±4)* | *505 (±1)* | *589 (±4)* | *673 (±1)* | | | |
| | | | | | | | | | | | | | | |
| Pre-treatment with i.v. methylprednisolone [20] | | x | x | | x | | x | | x | | | x | | |
| Administration of i.v. ocrelizumab / ocrelizumab placebo [21] | | x | x | | x | | x | | x | | | x | | |
| Assessment of s.c. Rebif®/ Rebif® placebo compliance | | x | x | x | x | x | x | x | x | x | x | | x | x |
| Administration of s.c. Rebif® / Rebif® placebo 3x/wk | | x | x | x | x | x | x | x | x | x | | | | |

1. **Informed Consent** must be obtained in written form from all patients at screening (prior to any study–related procedure) in order to meet eligibility for the study.
2. **Vital signs** (i.e., pulse rate, systolic and diastolic blood pressure, respiration rate and temperature) will be obtained while the patient is in the semi supine position (after 5 minutes). On infusion visits, the vital signs should be taken within 45 minutes prior to the methylprednisolone infusion in all patients. In addition, vital signs should be obtained prior to ocrelizumab/ocrelizumab placebo infusion, then every 15 minutes (± 5 minutes) for the first hour; then every 30 minutes (± 10 minutes) until 1 hour after the end of the infusion. On non-infusion days, the vital signs may be taken at any time during the visit.
3. **ECG (pre- and post-dose):** on infusion visits ECG should be taken within 45 minutes prior to the methylprednisolone infusion in all patients, and within 60 minutes after completion of ocrelizumab/ocrelizumab placebo infusion. On non-infusion days, the ECG may be taken at any time during the visit.
4. **MRI**: brain MRI scans will be obtained in patients withdrawn from the treatment period (at a withdrawal visit) if not performed during last 4 weeks.
5. **A structured telephone interview** will be conducted by site personnel every 4 weeks (± 3 days) from Week 8 through the study to identify any new or worsening neurological symptoms that warrant an unscheduled visit and collect data on possible events of infections.

6. **Serum β-hCG** must be performed at screening in women of childbearing potential. Subsequently, urine β-hCG [sensitivity of at least 25 mIU/mL] will be performed. On infusion visits, the urine pregnancy test should be performed prior to methylprednisolone infusion in all women of child-bearing potential. If positive, the patient will not receive the scheduled dose and confirmation, a serum pregnancy test, will be performed.
7. **Antibody Titers**: measurement of antibody titers against common antigens (mumps, rubella, varicella and Streptococcus pneumoniae) will be performed.
8. **RCR - Roche Clinical Repository non-DNA (RNA – and protein):** for RNA 2x 2.5 ml whole blood samples to be taken from consenting patients only for expression profiling analysis. For protein: 6 ml blood samples in EDTA tube for plasma samples will be taken from consenting patients only for analysis of protein biomarkers. On infusion visits, ALL samples should be taken 5-30 minutes prior to methylprednisolone infusion.
9. **RCR - Roche Clinical Repository (DNA):** 6 ml whole blood sample to be taken from only from patients consenting to RCR for pharmacogenetic and genetic analysis. If not done at Baseline (Visit 2), sample may be collected at next visit.
10. **Protein biomarker sampling:** one serum sample (6 ml) will be taken from all patients for analysis of protein biomarkers. On infusion visits, samples should be taken 5-30 minutes prior to methylprednisolone infusion.
11. **HAHA**: On infusion visits, serum samples are collected 5-30 minutes prior to the methylprednisolone infusion.
12. **Plasma and urine samples for JCV will be collected** at specified time points and analyzed in batches.
13. **PK samples:** on the infusion day at week 72,, two serum samples should be collected, one 5-30 minutes prior to the methylprednisolone infusion and the second one 30 minutes (±10 minutes) following the completion of the ocrelizumab/ocrelizumab placebo infusion. For all other infusion visits, a blood sample should be taken 5 – 30 minutes before the methylprednisolone infusion. At other times (non-infusion visits) samples may be taken at any time during the visit.
14. **sTSH** will be tested at screening and during double-blind treatment period. Thyroid autoantibodies will be assayed only at screening.
15. **FSH**: only applicable to women to confirm the post-menopausal status.
16. **Hepatitis** screening & monitoring: all patients must have negative HBsAg result and negative HepCAb screening tests prior to enrollment. If total HBcAb is positive at screening, HB virus DNA measured by PCR must be negative to be eligible. For those patients enrolled with negative HBsAg and positive total HBcAb, HB virus DNA (PCR) must be repeated every 12 weeks during the treatment period.
17. **FACS**: including CD19 and other circulating B-cell subsets, T-cells, natural killer cells and other leukocytes. On infusion visits, blood samples should be collected prior to the infusion of methylprednisolone.
18. **Routine safety lab:** hematology, chemistry and urinalysis: on infusion visits, all urine and blood samples should be collected prior to the infusion of methylprednisolone. At other times, samples may be taken at any time during the visit.
19. **Type I interferon neutralizing antibody assay:** samples should be taken at least 36 hours following last injection of Rebif®/Rebif® placebo.
20. All patients receive **prophylactic treatment** with 100 mg of methylprednisolone i.v. prior to infusion of ocrelizumab /ocrelizumab placebo. It is also recommended that patients receive an analgesic/antipyretic such as acetaminophen/paracetamol (1 g) and an i.v. or oral antihistamine such as diphenhydramine 50 mg 30-60 minutes prior to ocrelizumab/ ocrelizumab placebo.

21. **Administration (infusion) of i.v. ocrelizumab/ocrelizumab placebo**: the Treating Investigator must review the clinical and laboratory re-treatment criteria prior to subsequent infusion of ocrelizumab/ocrelizumab placebo.
22. **A delayed dosing visit** will be performed and recorded in the Delayed Dosing Visit eCRF form when dosing cannot be administered at the scheduled dosing visit. Other tests or assessments may be done as appropriate.
23. **Unscheduled Visit**: assessments performed at unscheduled (non-dosing) visits will depend on the clinical needs of the patient. All patients with new neurological symptoms suggestive of relapse should have EDSS performed by examining investigator. Other tests/assessments may be done as appropriate. Please note: in case of ALT elevations dose modification should be necessary, additional visits may be required for dispensing of study medication.

Please note: based on local Ethics Committees or National Competent Authority requirements, additional diagnostic testing may be required for selected patients or selected centers to exclude tuberculosis, Lyme disease, HTLV-1 associated myelopathy (HAM), acquired immune deficiency syndrome (AIDS), hereditary disorders, connective tissue disorders, or sarcoidosis. Other specific diagnostic tests may be requested when deemed necessary by the investigator**.**

## Table 4: Schedule of Assessments: Safety Follow up (including prolonged B-cell monitoring if required)

| | **Safety Follow up** | ***Prolonged B-cell Monitoring*[1]** | ***End of observation or withdrawal from Safety Follow up*** |
|---|---|---|---|
| **Assessments** | *Visits every 12 weeks (±7 days)* [2] | *Visits every 24 weeks (±7 days)* | |
| Urine pregnancy test | x | x | x |
| Routine Safety Labs[3] | x | x | x |
| FACS [4] | x | x | x |
| Total Ig, IgA, IgG, IgM | x[10] | x | x |
| HAHA[5] | x[10] | x | x |
| Plasma/urine banking for JCV | x | x | x |
| Antibody titers | x[10] | x | x |
| Hepatitis B viral DNA[6] | (x) | (x) | (x) |
| RCR non-DNA [7] | x[10] | x | x |
| Protein biomarker sampling [8] | x[10] | x | x |
| Vital Signs | x | x | x |
| EDSS | x | | x |
| Neurological examination | x | x | x |
| Physical examination | x[10] | x | x |
| Potential relapses recorded | x | x | x |
| Adverse events | x | x | x |
| Concomitant Medication | x | x | x |
| Telephone interview [9] | x | x | |

1. **Prolonged B-cell monitoring:** patients whose B-cells have not been repleted after 48 weeks of Safety Follow up period will continue with visits every 24 weeks (± 7 days) until B-cell repletion.
2. Visits will be performed at 12-week intervals counting from the date of last infusion of ocrelizumab.
3. **Routine safety lab**: hematology, chemistry and urinalysis.
4. **FACS** including CD19 and other circulating B-cell subsets, T cells, natural killer cells and other leukocytes.
5. **HAHA**: two serum samples are required.
6. **Hepatitis monitoring:** hepatitis to be monitored only in patients with screening results of HbsAg negative, HBcAb positive and HBV DNA negative, inclusive.
7. **RCR (Roche Clinical Repository) non-DNA (RNA and protein):** for RNA 2x 2.5 ml whole blood samples to be taken from consenting patients only for expression profiling analysis. For protein 6 ml blood samples in EDTA tube for plasma samples to be taken from consenting patients only for analysis of protein biomarkers.
8. **Protein biomarker sampling**: 6 ml blood sample in a plain tube without EDTA for serum isolation will be taken from all patients for analysis of protein biomarkers.
9. **A structured telephone interview** will be performed by site personnel every 4 weeks (± 3 days) between visits until 48 weeks after the last infusion to identify any new or worsening neurological symptoms that warrant an unscheduled visit and collect data on possible events of infections. If prolonged B-cell monitoring is required beyond 48 weeks after the last infusion, telephone interviews will be done every 12 weeks (± 7 days) between visits.
10. Needs to be assessed only every 24 weeks.

<u>Please note:</u> patients in Safety Follow up who receive other B-cell targeted therapies will only be followed for 48 weeks from the date of the last infusion of the study drug regardless of their B-cell count.

## SAMPLE SIZE AND STATISTICAL ANALYSES

The sample size for this study has been estimated based on data from previous RRMS trials, with the use of two-sided tests with an experiment-wise alpha of 0.05. The annualized rate of relapse among patients receiving ocrelizumab at 96 weeks is predicted to be 0.165 (standard deviation of approximately 0.60), as compared with 0.33 (standard deviation of approximately 0.80) among patients receiving the control treatment, Rebif® (this represents a relative reduction of 50% on ocrelizumab compared to the active comparator). For the annualized relapse rate, a t-test has been used to determine the sample size between ocrelizumab and the control arm. The sample size of 400 patients per arm provides 84 percent power, maintaining the type I error rate of 0.05, and assuming a drop out rate of 20 percent approximately (assuming relative reduction among patients drop out is 25%). For sustained disease progression, a two group test of equal exponential survival with exponential dropout is used to determine the sample size. Assuming the 2 year sustained disability progression rate is 18% for the Rebif® arm and 12.6% for the ocrelizumab arm (this represents a relative reduction of 30% on ocrelizumab compared to the active comparator), and assuming a drop out rate of 20 percent over 2 years approximately, the sample size of 400 per arm will provide 80 percent power, maintaining the type I error rate of 0.05 based on the pooled analysis of two identical RMS trials (800 patients treated with ocrelizumab 600 mg and 800 patients treated with Rebif®).

All eligible patients will be randomized to treatment (with 1:1 ratio to ocrelizumab 600 mg regimen or the control arm) stratified by region (US versus ROW) and baseline EDSS (< 4.0 versus ≥ 4.0).

The primary efficacy endpoint is the annualized relapse rate by 96 weeks. The annualized relapse rates at 96 weeks will be calculated using negative binomial model, adjusting for region (United States versus ROW) and baseline EDSS (< 4.0 versus ≥ 4.0). The adjusted annualized relapse rates and the 95% confidence intervals for the relapse rates will be presented along with the p-value.

Summaries of safety data will be produced using data from all patients who have received any study treatment and provided at least one assessment of safety.

## Table of Contents


1. Background and Rationale . . . . . . . . . . 38

1.1 Background . . . . . . . . . . 38

1.1.1 Multiple Sclerosis . . . . . . . . . . 38

1.1.2 Ocrelizumab . . . . . . . . . . 39

1.1.3 Rationale for Targeting B-cells in MS . . . . . . . . . . 40

1.1.4 Sponsor Experience with Anti-CD20 Compounds in MS . . . . . . . . . . 41

1.1.4.1 Ocrelizumab in RRMS . . . . . . . . . . 41

1.1.4.2 Rituximab in RRMS . . . . . . . . . . 43

1.1.4.3 Rituximab in PPMS . . . . . . . . . . 44

1.1.5 Rebif® . . . . . . . . . . 45

1.2 Rationale for the Study . . . . . . . . . . 47

2. Objectives . . . . . . . . . . 48

2.1 Primary Objective . . . . . . . . . . 48

2.2 Secondary Objectives . . . . . . . . . . 48

2.3 Exploratory Objectives . . . . . . . . . . 49

2.4 Roche Clinical Repository (RCR) Exploratory Objectives . . . . . . . . . . 49

3. Study Design . . . . . . . . . . 50

3.1 Overview of Study Design and Dosing Regimen . . . . . . . . . . 50

3.1.1 Rationale for Study Design . . . . . . . . . . 53

3.1.2 Rationale for Dose Selection . . . . . . . . . . 56

3.1.3 End of Study . . . . . . . . . . 57

3.2 Number of Subjects / Assignment to Treatment Groups . . . . . . . . . . 57

3.3 Centers . . . . . . . . . . 57

4. Study Population . . . . . . . . . . 57

4.1 Overview . . . . . . . . . . 57

4.1.1 Recruitment Procedures . . . . . . . . . . 57

4.2 Inclusion Criteria . . . . . . . . . . 58

4.3 Exclusion Criteria . . . . . . . . . . 58

4.4 Concomitant Medication and Treatment . . . . . . . . . . 61

4.4.1 Definition of Concomitant Treatment . . . . . . . . . . 61

4.4.2 Treatment for Symptoms of MS . . . . . . . . . . 61

4.4.2.1 Prohibited Concomitant Treatments . . . . . . . . . . . . . . . . . . . . . . . 62
4.4.3 Immunization . . . . . . . . . . . . . . . . . . . . . . . . . . . . . . . . . . . . . . . 62
4.5 Criteria for Premature Withdrawal . . . . . . . . . . . . . . . . . . . . . . . . . . 63
4.5.1 Withdrawal of Subjects from the Roche Clinical Repository (RCR) . 64
4.5.2 Patient Agreement for Continuation in the Study (in case of sustained disability progression) . . . . . . . . . . . . . . . . . . . . . . . . . . . . . 65
4.6 Replacement Policy (Ensuring Adequate Numbers of Evaluable Subjects) . . . . . . . . . . . . . . . . . . . . . . . . . . . . . . . . . . . . . . . . . . 65
4.6.1 For Subjects . . . . . . . . . . . . . . . . . . . . . . . . . . . . . . . . . . . . . . 65
4.6.2 For Centers . . . . . . . . . . . . . . . . . . . . . . . . . . . . . . . . . . . . . . . 65

5. Schedule of Assessment and Procedures . . . . . . . . . . . . . . . . . . . . . . . . . . 66

5.1 Screening Examination and Eligibility Screening Form . . . . . . . . . . . 73
5.2 Procedures for Enrollment of Eligible Subjects . . . . . . . . . . . . . . . . . 73
5.3 Clinical Assessments and Procedures . . . . . . . . . . . . . . . . . . . . . . . . 74
5.3.1 Overview of Clinical Visits . . . . . . . . . . . . . . . . . . . . . . . . . . . . . 74
5.3.1.1 Delayed Dosing Visit . . . . . . . . . . . . . . . . . . . . . . . . . . . . . . . . 75
5.3.1.2 Unscheduled Visits . . . . . . . . . . . . . . . . . . . . . . . . . . . . . . . . . 75
5.3.1.3 Withdrawal Visits . . . . . . . . . . . . . . . . . . . . . . . . . . . . . . . . . . 75
5.3.2 Assessment of Efficacy . . . . . . . . . . . . . . . . . . . . . . . . . . . . . . . . 76
5.3.2.1 Assessment of Relapse . . . . . . . . . . . . . . . . . . . . . . . . . . . . . . . 76
5.3.2.2 Assessment of Disability . . . . . . . . . . . . . . . . . . . . . . . . . . . . . . 76
5.3.2.3 Kurtzke Expanded Disability Status Scale (EDSS) . . . . . . . . . . . . 76
5.3.2.4 The Multiple Sclerosis Functional Composite Scale (MSFCS) . . . 77
5.3.2.5 Low-Contrast Visual Acuity (LCVA) Testing . . . . . . . . . . . . . . . 77
5.3.2.6 The Symbol Digit Modalities Test (SDMT) . . . . . . . . . . . . . . . . 77
5.3.3 Brain MRI Imaging . . . . . . . . . . . . . . . . . . . . . . . . . . . . . . . . . . 77
5.3.4 Safety . . . . . . . . . . . . . . . . . . . . . . . . . . . . . . . . . . . . . . . . . . . 78
5.3.4.1 Electrocardiogram (ECG) . . . . . . . . . . . . . . . . . . . . . . . . . . . . . 79
5.3.4.2 Physical Examination . . . . . . . . . . . . . . . . . . . . . . . . . . . . . . . . 79
5.3.4.3 Neurological Examination . . . . . . . . . . . . . . . . . . . . . . . . . . . . . 79
5.3.4.4 Telephone Interviews . . . . . . . . . . . . . . . . . . . . . . . . . . . . . . . . 79
5.3.4.5 Columbia-Suicide Severity Rating Scale C-SSRS . . . . . . . . . . . . 80
5.3.5 The Karnofsky Performance Scale (clinician-reported version) . . . . 80
5.4 Laboratory Assessments . . . . . . . . . . . . . . . . . . . . . . . . . . . . . . . . 80

5.4.1 Standard Laboratory Assessments . . . . . . . . . . . . . . . . . . . . . . . . . . . 81
5.4.2 Hepatitis Screening and Liver Function Monitoring . . . . . . . . . . . . . 82
5.4.3 Plasma and Urine Banking for JC Virus . . . . . . . . . . . . . . . . . . . . . . 83
5.4.4 Pharmacokinetic (PK)/Pharmacodynamic (PD) Assessments . . . . . . 83
5.4.5 Type I Interferon Neutralizing Antibody Assay . . . . . . . . . . . . . . . . . 83
5.5 Roche Clinical Repository Specimen(s) . . . . . . . . . . . . . . . . . . . . . . . 83
5.5.1 Specimen Types . . . . . . . . . . . . . . . . . . . . . . . . . . . . . . . . . . . . . . 84
5.6 Protein Biomarker Samples . . . . . . . . . . . . . . . . . . . . . . . . . . . . . . . 84
5.7 Patient Reported Outcome(s) . . . . . . . . . . . . . . . . . . . . . . . . . . . . . . 85
5.7.1 Modified Fatigue Impact Scale (MFIS) . . . . . . . . . . . . . . . . . . . . . . 85
5.7.2 The Center for Epidemiologic Studies Depression Scale (CES-D) . . 85
5.7.3 The Short Form (SF-36) Health Survey . . . . . . . . . . . . . . . . . . . . . . 86
5.7.4 Patient's Assessment of Treatment Benefit . . . . . . . . . . . . . . . . . . . 86
5.8 Pharmacoeconomic Assessments/ EQ-5D . . . . . . . . . . . . . . . . . . . . . . 86
5.9 Post Study Provisional Care . . . . . . . . . . . . . . . . . . . . . . . . . . . . . . . 86

6. Investigational Medicinal Product . . . . . . . . . . . . . . . . . . . . . . . . . . . . 87

6.1 Ocrelizumab . . . . . . . . . . . . . . . . . . . . . . . . . . . . . . . . . . . . . . . . . . 87
6.1.1 Preparation and Administration of Ocrelizumab Infusions . . . . . . . . 87
6.1.2 Prevention and Treatment of Infusion Related Reactions . . . . . . . . . 89
6.1.3 Ocrelizumab Dose Modifications, Interruptions and Delays . . . . . . . 90
6.1.4 Criteria for Re-Treatment with Ocrelizumab . . . . . . . . . . . . . . . . . . 91
6.2 Rebif® . . . . . . . . . . . . . . . . . . . . . . . . . . . . . . . . . . . . . . . . . . . . . . 91
6.2.1 Dose and Schedule of Rebif® . . . . . . . . . . . . . . . . . . . . . . . . . . . . 91
6.2.2 Rebif® Dose Modifications, Interruptions and Delays . . . . . . . . . . . 92
6.3 Formulation, Packaging and Labeling . . . . . . . . . . . . . . . . . . . . . . . . . 93
6.3.1 Ocrelizumab . . . . . . . . . . . . . . . . . . . . . . . . . . . . . . . . . . . . . . . . . 94
6.3.2 Rebif® . . . . . . . . . . . . . . . . . . . . . . . . . . . . . . . . . . . . . . . . . . . . . 95
6.4 Blinding and Unblinding . . . . . . . . . . . . . . . . . . . . . . . . . . . . . . . . . . 95
6.5 Accountability of IMP and Assessment of Compliance . . . . . . . . . . . . 96
6.5.1 Accountability of IMP . . . . . . . . . . . . . . . . . . . . . . . . . . . . . . . . . . 96
6.5.2 Assessment of Compliance . . . . . . . . . . . . . . . . . . . . . . . . . . . . . . . 96
6.6 Destruction of the IMP/Comparator . . . . . . . . . . . . . . . . . . . . . . . . . . 96

7. Safety Instructions and Guidance . . . . . . . . . . . . . . . . . . . . . . . . . . . . 97

7.1 Adverse Events (AEs) and Laboratory Abnormalities . . . . . . . . . . . . . 97
7.1.1 Clinical AEs . . . . . . . . . . . . . . . . . . . . . . . . . . . . . . . . . . . . . . . . . 97
7.1.1.1 Intensity . . . . . . . . . . . . . . . . . . . . . . . . . . . . . . . . . . . . . . . . . . 98
7.1.1.2 Drug - Adverse Event Relationship . . . . . . . . . . . . . . . . . . . . . . . . 98
7.1.1.3 Serious Adverse Events (Immediately Reportable to Sponsor) . . . 98
7.1.2 Treatment and Follow-up of AEs . . . . . . . . . . . . . . . . . . . . . . . . . . 99
7.1.3 Laboratory Test Abnormalities . . . . . . . . . . . . . . . . . . . . . . . . . . . . 99
7.1.3.1 Follow-up of Abnormal Laboratory Test Values . . . . . . . . . . . . . . 99
7.2 Handling of Safety Parameters . . . . . . . . . . . . . . . . . . . . . . . . . . . . 100
7.2.1 Reporting of AEs . . . . . . . . . . . . . . . . . . . . . . . . . . . . . . . . . . . . . 100
7.2.2 Reporting of Serious Adverse Events . . . . . . . . . . . . . . . . . . . . . . . 100
7.2.2.1 Immediate Reporting to the Sponsor . . . . . . . . . . . . . . . . . . . . . . 100
7.2.2.2 Expedited Reporting to Health Authorities, Investigators, Institutional Review Boards, and Ethics Committees . . . . . . . . . . . . . . 101
7.2.3 Pregnancy and Lactation . . . . . . . . . . . . . . . . . . . . . . . . . . . . . . . . 102
7.3 Warnings and Precautions . . . . . . . . . . . . . . . . . . . . . . . . . . . . . . . 103
7.3.1 Ocrelizumab . . . . . . . . . . . . . . . . . . . . . . . . . . . . . . . . . . . . . . . . . 103
7.3.2 Rebif® . . . . . . . . . . . . . . . . . . . . . . . . . . . . . . . . . . . . . . . . . . . . . 105
7.3.3 Corticosteroids . . . . . . . . . . . . . . . . . . . . . . . . . . . . . . . . . . . . . . . 106
7.3.4 Progressive Multifocal Leukoencephalopathy . . . . . . . . . . . . . . . . . 106
7.3.4.1 Guidance for Diagnosis of PML . . . . . . . . . . . . . . . . . . . . . . . . . . 107

8. Statistical Considerations and Analytical Plan . . . . . . . . . . . . . . . . . . . . . 111

8.1 Study Endpoints . . . . . . . . . . . . . . . . . . . . . . . . . . . . . . . . . . . . . . . 111
8.1.1 Primary Efficacy Endpoint . . . . . . . . . . . . . . . . . . . . . . . . . . . . . . . 111
8.1.2 Secondary Efficacy Endpoints . . . . . . . . . . . . . . . . . . . . . . . . . . . . 111
8.1.3 Exploratory Efficacy Endpoints . . . . . . . . . . . . . . . . . . . . . . . . . . . 111
8.1.4 Safety . . . . . . . . . . . . . . . . . . . . . . . . . . . . . . . . . . . . . . . . . . . . . . 112
8.2 Statistical and Analytical Methods . . . . . . . . . . . . . . . . . . . . . . . . . . 112
8.2.1 Primary Efficacy Analysis . . . . . . . . . . . . . . . . . . . . . . . . . . . . . . . 113
8.2.2 Secondary Efficacy Analyses . . . . . . . . . . . . . . . . . . . . . . . . . . . . . 113
8.2.2.1 The Time to Onset of Sustained Disability Progression for At Least 12 Weeks During the 96-Week Comparative Treatment Period . 114
8.2.2.2 The Time to Onset of Sustained Disability Progression for At Least 24 Weeks During the 96-Week Comparative Treatment Period . 115

8.2.2.3 The Proportion of Relapse-Free Patients by 96 Weeks . . . . . . . . . . 115
8.2.2.4 The Change in Total T2 Lesion Volume as Detected by Brain MRI from Baseline to Week 96 . . . . . . . . . . . . . . . . . . . . . . . . . . . . . . 115
8.2.2.5 The Total Number of New, and/or Enlarging T2 Hyperintense Lesions as Detected by Brain MRI at Week 24, Week 48 and Week 96. . . . . . . . . . . . . . . . . . . . . . . . . . . . . . . . . . . . . . . . . . . . . . . 116
8.2.2.6 The Change in Multiple Sclerosis Functional Composite Scale (MSFCS) Score from Baseline to Weeks 96 . . . . . . . . . . . . . . . . . . . . . 116
8.2.2.7 The Change in Brain Volume as Detected by Brain MRI Scan from Week 24 to Week 96 . . . . . . . . . . . . . . . . . . . . . . . . . . . . . . . . . . 116
8.2.3 Exploratory Analyses . . . . . . . . . . . . . . . . . . . . . . . . . . . . . . . . . . 116
8.2.4 Sample Size . . . . . . . . . . . . . . . . . . . . . . . . . . . . . . . . . . . . . . . . . . 116
8.2.5 Hypothesis Testing . . . . . . . . . . . . . . . . . . . . . . . . . . . . . . . . . . . . 117
8.2.6 Analysis Populations . . . . . . . . . . . . . . . . . . . . . . . . . . . . . . . . . . 117
8.2.6.1 Safety Population . . . . . . . . . . . . . . . . . . . . . . . . . . . . . . . . . . . . 117
8.2.6.2 Intent-to-Treat Population . . . . . . . . . . . . . . . . . . . . . . . . . . . . . 117
8.2.6.3 Per Protocol Population . . . . . . . . . . . . . . . . . . . . . . . . . . . . . . . 118
8.2.7 Interim Analysis . . . . . . . . . . . . . . . . . . . . . . . . . . . . . . . . . . . . . . 118
8.2.8 Safety Data Analysis . . . . . . . . . . . . . . . . . . . . . . . . . . . . . . . . . . 118
8.2.9 Safety Follow-up Period . . . . . . . . . . . . . . . . . . . . . . . . . . . . . . . . 119
8.2.10 Other Analyses . . . . . . . . . . . . . . . . . . . . . . . . . . . . . . . . . . . . . . 119
8.2.10.1 Pharmacokinetic Analysis . . . . . . . . . . . . . . . . . . . . . . . . . . . . . 119
8.2.10.2 Pharmacodynamic Analysis . . . . . . . . . . . . . . . . . . . . . . . . . . . 119
8.2.10.3 Roche Clinical Repository / Protein Biomarker Samples . . . . . . . 120

9. Data Collection, Management and Quality Assurance . . . . . . . . . . . . . . . . . . 120
9.1 Assignment of Preferred Terms and Original Terminology . . . . . . . . . 120

10. Study Committees . . . . . . . . . . . . . . . . . . . . . . . . . . . . . . . . . . . . . . . . 120

11. References . . . . . . . . . . . . . . . . . . . . . . . . . . . . . . . . . . . . . . . . . . . . 122

12. Ethical Aspects . . . . . . . . . . . . . . . . . . . . . . . . . . . . . . . . . . . . . . . . . 127
12.1 Local Regulations/Declaration of Helsinki . . . . . . . . . . . . . . . . . . . . . 127
12.2 Informed Consent . . . . . . . . . . . . . . . . . . . . . . . . . . . . . . . . . . . . . . . 127
12.2.1 Main Study Informed Consent . . . . . . . . . . . . . . . . . . . . . . . . . . . . 127
12.2.2 RCR Informed Consent . . . . . . . . . . . . . . . . . . . . . . . . . . . . . . . . . 128

12.2.3 Death or Loss of Competence of Participant who has donated a specimen(s) that is stored in the RCR . . . . . . . . . . . . . . . . . . . . . . . . . 128
12.3 Independent Ethics Committees (IEC) and Institutional Review Board (IRB) . . . . . . . . . . . . . . . . . . . . . . . . . . . . . . . . . . . . . . . . . . . . 129
12.4 Role of the Science and Ethics Advisory Group (SEAG) . . . . . . . . . . 130

13. Conditions for Modifying the Protocol . . . . . . . . . . . . . . . . . . . . . . . . . . . 130

14. Conditions for Terminating the Study . . . . . . . . . . . . . . . . . . . . . . . . . . . 130

15. Study Documentation, CRFs and Record Keeping . . . . . . . . . . . . . . . . . . . 130
15.1 Investigator's Files / Retention of Documents . . . . . . . . . . . . . . . . . . . 130
15.2 Source Documents and Background Data . . . . . . . . . . . . . . . . . . . . . . 131
15.3 Audits and Inspections . . . . . . . . . . . . . . . . . . . . . . . . . . . . . . . . . . . . 131
15.4 Electronic Case Report Forms . . . . . . . . . . . . . . . . . . . . . . . . . . . . . . . 131
15.5 Financial Disclosure . . . . . . . . . . . . . . . . . . . . . . . . . . . . . . . . . . . . . . 132

16. Monitoring the Study . . . . . . . . . . . . . . . . . . . . . . . . . . . . . . . . . . . . . . . 132

17. Confidentiality of Trial Documents and Subject Records . . . . . . . . . . . . . 132

18. Clinical Study Report (CSR) . . . . . . . . . . . . . . . . . . . . . . . . . . . . . . . . . . 133

19. Publication of Data and Protection of Trade Secrets . . . . . . . . . . . . . . . . . . 133

## List of Tables


Table 1: Summary of the Most Frequent Rebif® Adverse Reactions by MedDRA System Organ Class . . . . . . . . . . . . . . . . . . . . . . . . . . 47

Table 2: Overview of Dosing Regimen . . . . . . . . . . . . . . . . . . . . . . . . . . . . . . . . 52

Table 3: Schedule of Assessments: Screening Through the End of Double-Blind Treatment Period . . . . . . . . . . . . . . . . . . . . . . . . . . 66

Table 4: Schedule of Assessments: Safety Follow up (including prolonged B-cell monitoring if required). . . . . . . . . . . . . . . . . . . . . . . . . . . 71

Table 5: Treatment Groups and Schedule of Study Medication. . . . . . . . . . . . . . 87

Table 6: Infusions of Ocrelizumab 300 mg . . . . . . . . . . . . . . . . . . . . . . . . . . . . 88

Table 7: Subsequent Infusions of Ocrelizumab 600 mg. . . . . . . . . . . . . . . . . . . 89

Table 8: Overview of Rebif® Dosing Regimen * . . . . . . . . . . . . . . . . . . . . . . . 92

Table 9: Clinical features to distinguish between MS relapse and PML* . . . . . 109

Table 10: MRI Lesion Characteristics Typical of PML and MS . . . . . . . . . . . . 110

## List of Figures

Figure 1: Overview of Study Design . . . . . . . . . . . . . . . . . . . . . . . . . . . . . . . . . . 50
Figure 2: Safety Follow up - Variable B-cell Monitoring. . . . . . . . . . . . . . . . . . . 51
Figure 3: Diagnostic Algorithm for PML . . . . . . . . . . . . . . . . . . . . . . . . . . . . . 109
Figure 4: Hierarchal Order of Key Efficacy Endpoints . . . . . . . . . . . . . . . . . . . 114

## List of Appendices

Appendix 1: AEs Categories for Determining Relationship to Test Drug . . . . . 134

Appendix 2: ICH Guidelines for Clinical Safety Data Management, Definitions and Standards for Expedited Reporting, Topic E2 . 135

Appendix 3: Common Terminology Criteria for Adverse Events (CTCAE) . . . 137

Appendix 4: Telephone Interviews . . . . . . . . . . . . . . . . . . . . . . . . . . . . . . . . . . 138

Appendix 5: Modified Fatigue Impact Scale (MFIS) . . . . . . . . . . . . . . . . . . . . 140

Appendix 6: The Center for Epidemiologic Studies Depression Scale (CES-D) . . . . . . . . . . . . . . . . . . . . . . . . . . . . . . . . . . . . . . . . . . . . 143

Appendix 7: The Short Form (SF-36) Health Survey . . . . . . . . . . . . . . . . . . . . 145

# GLOSSARY OF ABBREVIATIONS

| | |
|---|---|
| ADCC | Antibody dependent cellular cytotoxicity |
| AE | Adverse Event |
| AIDS | Acquired Immune Deficiency Syndrome |
| ALT (SGPT) | Alanine aminotransferase |
| ALP | Alkaline Phosphatase |
| ARR | Annualized Relapse Rate |
| AST (SGOT) | Aspartate aminotransferase |
| AUC | Area Under Curve |
| BAFF | B-cell activating factor |
| BCG | Bacille Calmette-Guérin – TB vaccine (Fr. Bacille billié de Calmette-Guérin) |
| β hCG | Beta human Chorionic Gonadotropin |
| CD | Cluster of Differentiation |
| CDC | Complement-dependent cytotoxicity |
| CES-D | Center for Epidemiologic Studies Depression Scale |
| CFH | Complement Factor H |
| CSF | Cerebrospinal Fluid |
| C-SSRS | Columbia - Suicide Severity Rating Scale |
| CTCAE | Common Terminology Criteria for Adverse Events |
| DMARD | Disease-modifying anti-rheumatic drugs |
| DMC | Data Monitoring Committee |
| DNA | Deoxyribonucleic Acid |
| DAP | Data Analysis Plan |
| EBV | Epstein-Barr Virus |
| ECG | Electrocardiogram |
| eCRF | Electronic Case Report Form(s) |
| EDC | Electronic Data Capture |
| EDSS | Expanded Disability Status Scale |

## GLOSSARY OF ABBREVIATIONS

| | |
|---|---|
| eform | Electronic form |
| ELISA | Enzyme-Linked Immunosorbent Assay |
| ESF | Eligibility Screening Form |
| EQ-5D | EuroQoL |
| FDA | Food and Drug Administration |
| FLAIR | Fluid-attenuated Inversion Recovery |
| FSH | Follicle Stimulating Hormone |
| FSS | Functional Systems Scores |
| GGT | Gamma Glutamyl Transferase |
| HAHA | Human Anti-human Antibodies |
| HAM | Human T-lymphotropic virus (HTLV)-1 Associated Myelopathy |
| HBsAg | Hepatitis B Surface Antigen |
| HBcAb | Hepatitis B Core Antibody |
| HepCAb | Hepatitis C Antibody |
| HDHF | High Dose High Frequency |
| HIV | Human Immunodeficiency Virus |
| HTLV | Human T-lymphotropic Virus |
| IB | Investigator Brochure |
| ICH | International Conference on Harmonisation |
| ICMJE | International Committee of Medical Journal Editors |
| IFN | Interferon |
| Ig | Immunoglobulin |
| i.m. | Intramuscular |
| IMP | Investigational Medicinal Product |
| IND | Investigational New Drug |
| INN | International Non-proprietary Name |
| IRB/IEC | Institutional Review Board/Independent Ethics Committee |

## GLOSSARY OF ABBREVIATIONS

| | |
|---|---|
| IRR | Infusion Related Reaction |
| ITT | Intent-To-Treat |
| i.v. | Intravenous |
| IxRS | Interactive Voice and Web Response System |
| JCV | JC Virus |
| KLH | Keyhole Limpet Haemocyanin |
| LCVA | Low Contrast Visual Acuity |
| LLN | Lower Limit of Normal |
| MBP | Myelin Basic Protein |
| MedDRA | Medical Dictionary for Regulatory Activities |
| MFIS | Modified Fatigue Impact Scale |
| MOG | Myelin Oligodendrocyte Glycoprotein |
| MRI | Magnetic Resonance Imaging |
| MS | Multiple Sclerosis |
| MSFCS | Multiple Sclerosis Functional Composite Scale |
| MTX | Methotrexate |
| NAb | Neutralizing Antibody |
| NHL | Non Hodgkin Lymphoma |
| NK | Natural killer |
| NYHA | New York Heart Association |
| OCB | Oligoclonal Band |
| OCR | Ocrelizumab |
| OCT | Optical Coherence Tomography |
| PASAT | Paced Auditory Serial Addition Test |
| PCR | Polymerase Chain Reaction |
| PD | Pharmacodynamics |
| PK | Pharmacokinetics |
| PML | Progressive Multifocal Leukoencephalopathy |

## GLOSSARY OF ABBREVIATIONS

| | |
|---|---|
| PP | Per protocol (population) |
| PPMS | Primary Progressive Multiple Sclerosis |
| PRMS | Progressive Relapsing Multiple Sclerosis |
| PRO | Patient-Reported Outcome |
| RA | Rheumatoid Arthritis |
| RBC | Red Blood Cells |
| RCR | Roche Clinical Repository |
| RMS | Relapsing Multiple Sclerosis |
| RNA | Ribonucleic Acid |
| ROW | Rest of the World |
| RMS | Relapsing Multiple Sclerosis |
| RNFL | Retinal Nerve Fiber Layer |
| RPR | Rapid Plasma Reagin |
| RRMS | Relapsing-Remitting Multiple Sclerosis |
| SAE | Serious Adverse Event |
| s.c. | Subcutaneous |
| SDMT | The Symbol Digit Modalities Test |
| SEAG | Science and Ethics Advisory Group |
| SF-36 | SF-36 Health Survey |
| SMT | Study Management Team |
| SPMS | Secondary Progressive Multiple Sclerosis |
| TNF | Tumor Necrosis Factor |
| sTSH | sensitive Thyroid Stimulating Hormone |
| TB | Tuberculosis |
| $T_{CTL}$ | Cytotoxic Lymphocyte T |
| ULN | Upper Limit of Normal |
| WBC | White Blood Cells |

# PART I: STUDY DESIGN AND CONDUCT

## 1. BACKGROUND AND RATIONALE

## 1.1 Background

### 1.1.1 Multiple Sclerosis

Multiple sclerosis (MS) is an inflammatory and degenerative demyelinating disease of the human central nervous system (CNS). Multiple sclerosis affects around 2.5 million people worldwide: it is one of the most common neurological disorders and causes of disability of young adults, especially in Europe and North America [1]. The condition manifests as neurological deficits referable to damage to the spinal cord, brainstem, optic nerves, cerebellum, and cerebrum. Resulting symptoms may include weakness, pain, visual loss, bowel/bladder dysfunction, and cognitive dysfunction. Diagnosis of MS typically occurs through the application of highly structured diagnostic criteria that rely on clinical observation, neurological examination, brain and spinal cord Magnetic Resonance Imaging (MRI) scans, evoked potentials, and examination of cerebrospinal fluid (CSF) [2, 3].

MS is clinically subcategorized into four phenotypic disease patterns distinguished by the occurrence and timing of relapses relative to disease onset and disability progression [4]. These include relapsing remitting MS (RRMS), primary progressive MS (PPMS), progressive relapsing MS (PRMS); and secondary progressive MS (SPMS).

Approximately 80% of MS patients present with RRMS. If left untreated, the majority of RRMS patients will transition into SPMS (with progressive loss of neurologic function, in the absence of relapses) within 20 years. The term relapsing MS (RMS) applies to those patients either RRMS or SPMS, who continue to suffer relapses. Patients with RMS, whether or not they suffer from neurologic progression in the absence of relapses, have a common, inflammatory pathophysiology and therefore, constitute a common target for treatment.

Currently available first-line therapies for the treatment of either relapsing MS or relapsing-remitting MS include interferon (IFN)- β-1a (Rebif® and Avonex®), IFN-β-1b (Betaferon®/Extavia®) and glatiramer acetate (Copaxone®). The currently approved first-line treatments are only modestly effective in reducing the frequency of relapses and preventing disability in patients with RMS. The magnitude of these disease modifying effects are an approximately 30% relative improvement versus placebo [5]. The first-line disease modifying agents reduce the frequency of new episodes but do not reverse fixed deficits and have questionable effects on long-term disease progression [6].

Fingolimod (FTY720) is an oral modulator of sphingosine-1 phosphate (S1P) receptors, a unbiquitous group of transmembrane receptors involved with cellular growth and differentiation. Fingolimod's immunomodulatory effects are believed to be due to binding to and internalization of the S1P1 receptor on lymphocytes, thereby rendering them insensitive to S1P gradients in lymph and inhibiting egress from lymph nodes and other secondary lymphoid organs. Fingolimod is known to readily cross the blood-brain barrier and there are S1P receptors on glial cells and neurons, however the implications

of any possible direct CNS S1P receptor modulation effects are currently unknown. Fingolimod was shown to reduce ARR by approximately 50% versus both placebo and interferon beta-1a, 30 mcg im weekly (Avonex®), in confirmatory Phase III clinical trials. Due to the presence of S1P receptors on many different cell types, the adverse event profile of fingolimod is complex, with potential effects on cardiac, ophthalmic, hepatic and pulmonary function, as well as an increased risk of infection, due to inhibition of lymphocyte trafficking. Fingolimod was approved in 2010 by the FDA for patients with relapsing forms of MS and has recently gained a favorable ruling by the CHMP, for use in patients who have previously failed first-line disease-modifying therapy or who have highly active disease.

Natalizumab (Tysabri®) is a monoclonal antibody directed against alpha-4 beta-1 integrin (VLA-4), an adhesion molecule expressed on activated lymphocytes. Natalizumab binds to VLA-4, inhibiting trafficking of activated lymphocytes into the CNS and other extravascular tissues. Natalizumab was shown to have a 66% relative reduction in ARR versus placebo in a Phase III clinical trial. Natalizumab use is generally limited to RRMS patients who have failed to respond to first-line disease modifying therapy or to highly active RRMS patients due to a risk of Progressive Multifocal Leukoencephalopathy (PML).

Mitoxantrone (Novantrone®), a chemotherapeutic agent, is also approved for treatment of relapsing MS in the United States of America, but is generally reserved for secondary progressive and severe relapsing remitting forms of disease. Other drugs have been used with varying degrees of success, including corticosteroids, methotrexate, cyclophosphamide, azathioprine, and intravenous immunoglobulin.

Despite significant advances in MS therapy many patients continue to experience disease activity; thus there remains a need to develop more effective and better tolerated therapies for the treatment of RMS.

### 1.1.2 Ocrelizumab

Ocrelizumab is a humanized, glycosylated, monoclonal antibody directed against the CD20 antigen present on select B-cells. Ocrelizumab binds to the CD20 antigen thereby resulting in B-cell depletion via antibody-dependant cellular cytotoxicity (ADCC), complement-dependent cytotoxicity (CDC) and enhanced apoptosis.

Ocrelizumab was constructed using a recombinant DNA technique. This antibody shares an overlapping epitope on CD20 with rituximab (chimeric monoclonal antibody, Mabthera®/Rituxan®), as determined by direct competition and epitope-mapping experiments. In-vitro, ocrelizumab was shown to be approximately 5 times more potent than rituximab in ADCC activity on a B-cell tumor line over-expressing CD20, approximately 3 times less potent via CDC, and approximately equal in inducing apoptosis in a B-cell lymphoma cell line.

There is substantial proof-of-concept clinical data to support the use of B-cell depleting therapies in patients with relapsing MS. Ocrelizumab shares the same basic mechanism of action as rituximab. In a proof of concept study, rituximab treatment resulted in a robust reduction in MRI based measures of CNS inflammation and clinical benefit vs.

placebo, in patients with RRMS [7]. WA21493/ACT4422g, an ongoing Phase II study of ocrelizumab in RRMS patients provides proof-of-concept support for ocrelizumab efficacy and safety in patients with relapsing remitting MS; please refer to Section 1.1.4.1 for more details.

Ocrelizumab is also known as Ro 496-4913, PRO70769 and rhuMAb 2H7 (refer to Investigator's Brochure for further information).

### 1.1.3 Rationale for Targeting B-cells in MS

Humoral immunity has been implicated in MS for decades, as evidenced by inclusion of cerebrospinal fluid (CSF) oligoclonal bands (OCB) and increased intrathecal IgG synthesis in diagnostic criteria for MS [2, 3, 8]. Although, until very recently, the prevailing view of MS pathophysiology held that the CNS inflammation seen in MS is principally mediated by $CD4^+$ proinflammatory (Th1, Th17) T cells, rapidly expanding evidence suggests that B-cells may contribute to MS pathogenesis much more fundamentally than was previously believed, potentially through either antibody-dependent or independent mechanisms [9, 10, 11]. B lymphocytes have been detected within MS lesions and in the CSF of MS patients. Molecular analysis of both lesional and CSF B-cell repertoires reveals dominant, clonally expanded B-cell populations exhibiting somatic hypermutation in the antigen-recognizing CDR3 regions of immunoglobulin (Ig) heavy chains, predominately within the VH4 gene family [12, 13, 1, 15, 16, 17, 18].

Detection of these affinity-matured, clonally expanded repertoires in the CSF but not peripheral blood of MS patients suggests that a localized, antigen driven B-cell response is present in the CNS compartment. CSF clonal B-cell expansion has been reported in patients with both RRMS and PPMS shortly after diagnosis, implying a role for B-cells early in MS pathogenesis rather than as a late response to longstanding tissue damage [19]. More recently, cDNA transcriptomes of clonally expanded affinity-matured B-cells isolated from the CSF of MS patients have been sequence-matched to specific IgG OCBs from the same CSF samples, indicating that this longstanding hallmark of MS diagnosis derives from identifiable B-cell clones present in the CNS compartment [20].

Both antibody-dependent and independent hypotheses for the role of B-cells in MS pathophysiology have been postulated and are currently the subject of intensive research. B-cells may differentiate into plasma cells and produce CNS-directed auto-antibodies, potentially triggering cellular and complement-dependent cytotoxicity. Although a pathogenic role of anti-myelin antibodies in MS has not been established, they have been detected in the CSF of MS patients [21, 22, 23] and in active MS lesions [24] and remain potential candidates as effectors of myelin sheath damage. B-cells may also function as antigen presenting cells and thereby modulate effector T-cell responses, as they exhibit regulated secretion of both pro-inflammatory and anti-inflammatory cytokines, a function that appears to be abnormal in patients with MS [9]. Finally, B-cells may be a site of latent viral infections such as Epstein Barr Virus (EBV), which may drive CNS autoimmune responses through molecular mimicry or other pro-inflammatory mechanisms [10].

Postmortem pathological studies have identified the presence of ectopic follicular lymphoid structures in the meninges anatomically proximal to sites of grey matter demyelination in a subset of SPMS patients [25, 26, 14]. Similar tertiary lymphoid structures form *de novo* in various tissues of many autoimmune disorders and represent potential *de novo* sites of chronic autoantigenic B-cell activation, maturation and clonal expansion [28]. SPMS patients exhibiting these lymphoid structures have been found to have worse progression rates, when compared to controls without such follicular structures [29] and a pathomechanistic link to grey matter demyelination typical for SPMS has been suggested. Whether or not an anti-CD20 therapeutic antibody can affect the formation or persistence of meningeal lymphoid follicles or the grey matter demyelination prominent in progressive forms of MS is unknown.

In summary, B lymphocytes are believed to contribute to the pathogenesis of all subtypes of MS. Removing select peripheral B-cells from circulation may beneficially disrupt inflammatory processes that potentially involve chronic antigenic stimulation or other regulatory functions promoting chronic autoimmunity. Ocrelizumab specifically depletes CD20+ B-cells, making it a potentially attractive pharmacological agent to test for therapeutic potential in patients with multiple sclerosis.

### 1.1.4 Sponsor Experience with Anti-CD20 Compounds in MS

#### *1.1.4.1 Ocrelizumab in RRMS*

Study WA21493/ACT4422G is a 220-patient Phase II, multicenter, randomized, parallel-group, placebo-controlled, proof-of-concept study to evaluate the safety and efficacy of two dose regimens of ocrelizumab (1000 mg x 2 [administered on Day 1 and Day 15, followed by single infusions of 1000 mg for subsequent cycles] and 300 mg x 2 [administered on Day 1 and Day 15 followed by single infusions of 600 mg for subsequent cycles]), with an additional randomized open label arm of interferon β1-a 30 μg i.m. every week (Avonex®) arm. The primary objective was to evaluate the efficacy of two dose regimens of ocrelizumab compared with placebo, in reducing brain inflammation, as measured by the total number of gadolinium-enhancing T1 lesions observed on serial MRI scans of the brain at Weeks 12, 16, 20, and 24. Key secondary objectives were to evaluate the efficacy of both dose regimens of ocrelizumab compared with placebo in reducing annualized relapse rates at 24 weeks and to evaluate the safety and tolerability of both dose regimens of ocrelizumab in patients with RRMS. Exploratory outcomes included analysis of both dose regimens of ocrelizumab compared to interferon β-1a 30 μg i.m. weekly (Avonex®) along various study measures. Treatment with ocrelizumab is planned for 72 to 96 weeks total, depending on study arm (patients from both placebo and Avonex group switched to ocrelizumab 300 mg x 2 after Week 24). Additional MRI scans of the brain will be obtained at weeks 96 and 144 for a subgroup of patients.

This study is currently ongoing. Week 24 results demonstrated that both doses of ocrelizumab achieved the primary endpoint by significantly reducing the number of gadolinium-enhancing lesions compared with placebo ($p < 0.0001$). Both OCR dose groups showed statistically significant reductions in ARR compared with the placebo group (ARR = 0.125 for the OCR 300 mg x 2 group [$p = 0.0005$] and ARR = 0.169 for the OCR 1000 mg x 2 group [$p = 0.0014$] compared with ARR = 0.637 for the placebo

group, representing a relative reduction (RR) of 80% and 73% in ARR versus placebo group for the low and high OCR groups, respectively. In exploratory analyses, both ocrelizumab groups were superior to the Avonex group for the primary endpoint ($p < 0.0001$) and the 300 mg x 2 group for ARR (ARR = 0.364 for the Avonex group, representing a RR of 66% in ARR with $p = 0.03$ for the OCR 300 mg x 2 group versus Avonex group and a RR of 53.6% in the ARR with $p = 0.086$ for the OCR 1000 mg x 2 group versus Avonex group).

Patients from both placebo and Avonex groups switched to ocrelizumab 300 mg x 2 after Week 24. By 48 weeks, the level of benefit of ocrelizumab in reduction of ARR was maintained, where the patients in the ocrelizumab 300 mg x 2 group continued to have a suppressed ARR of 0.086 from Week 24 to 48, and patients switched to ocrelizumab from either placebo or Avonex®, derive a similar degree of efficacy to those randomized to ocrelizumab from onset (ARR for placebo-to-ocrelizumab = 0.161 and for Avonex-to-ocrelizumab = 0.137 after the switch, representing a RR of 74% and 62.4% compared with ARR before the switch respectively). From week 0 to 72, patients originally randomized to ocrelizumab 300 mg x 2 maintained clinical efficacy with an ARR of 0.186.

The most commonly reported adverse events in ocrelizumab-treated patients were infusion related reactions (IRRs). IRRs were reported during/after the first infusion (Day 1) for 30–43.6% of patients treated with ocrelizumab. Fewer patients (2.1–9.4%) experienced IRRs during/after the second infusion (Day 15). The most common symptoms were rash, pruritus, flushing, tachycardia, headache, pyrexia, and throat irritation.

No unanticipated, clinically significant abnormalities in vital signs, electrocardiograms (ECGs), or laboratory parameters were observed in association with ocrelizumab treatment.

On review of the placebo-controlled, double-blinded 24-week safety data, no imbalance in adverse events (or infection adverse events) or serious adverse events (or infection serious adverse events) between the placebo and active ocrelizumab arms was observed. The rate of adverse events (or infection adverse events) and serious adverse events (or infection serious adverse events) did not increase in ocrelizumab-treated patients at 48 weeks compared with 24 weeks. There is no trend toward an increased risk of adverse events (or infection adverse events) or serious adverse events (or infection serious adverse events) for ocrelizumab-treated patients with previous IFN treatment (Avonex for 6 months).

By the time all patients finished the Week 48 of treatment period, the incidence of infections and serious infections was 92.41/100 PY (95% CI: 76.59, 111.5) and 3.39/100 PY (95% CI: 1.27, 9.04) in patients exposed to low-dose ocrelizumab, including patients who switched from placebo or Avonex®. The incidence of infections and serious infections was 97.38/100 PY (95% CI: 74.76, 126.84) and 5.31/100 PY (95% CI: 1.71, 16.47) in those exposed to the high dose of ocrelizumab. The most common infections in ocrelizumab-treated patients included urinary tract infections, upper respiratory infections, and nasopharyngitis.

To date, in study WA21493, after over 250 patient years exposure to ocrelizumab, there have been no reports of opportunistic or fatal infections. There was 1 death during the 24-week treatment period: a patient in the ocrelizumab 1000 mg x 2 group was hospitalized with acute onset of encephalopathy and status epilepticus due to systemic inflammatory response syndrome with disseminated intravascular coagulation of unknown cause, following infusion of gadolinium. The patient's course rapidly progressed to multi-organ failure. While hospitalized, the patient developed nosocomial pneumonia in the setting of severe renal and hepatic insufficiency. After 2 weeks of intensive care the patient died of transforaminal herniation of the brain, due to massive cerebral edema.

Study patients have now completed Week 72, and preliminary analysis shows that the rates of serious infections remain stable over time compared to 24 or 48 week experience. A comprehensive analysis of the safety and efficacy data through this time point is currently ongoing. The Data Monitoring Committee, based on analysis of safety and efficacy outcomes from the 72-week analysis recommended continuation of Phase II WA21493 study; the DMC also agreed that these results support further development of ocrelizumab in Phase III program.

#### *1.1.4.2 Rituximab in RRMS*

Two clinical trials have been conducted in RRMS patients with rituximab, a chimeric mouse/human monoclonal antibody that binds shares the same basic mechanism of action as ocrelizumab. Findings briefly highlighted below, offer additional support for the therapeutic potential of the anti-CD20 mechanism in MS.

Study U3264g (HERMES Jr.) was a Phase I, open-label, multicenter study in 26 adults with RRMS to evaluate the safety and tolerability of two treatment cycles of rituximab administered at baseline and after 24 weeks. Re-treatment with rituximab (1000 mg × 2) at 24 weeks was safe and well tolerated, with an observed decrease in relapses and gadolinium-enhancing lesions through 72 weeks [30].

Study U2787g (HERMES) was a Phase II, proof-of-concept, randomized, double-blind, parallel-group, placebo-controlled, multicenter study to evaluate the safety and efficacy of rituximab in 104 adults with RRMS. The primary objectives were to investigate the efficacy of rituximab compared with placebo, as measured by the total number of gadolinium-enhancing T1 lesions observed on serial MRI scans of the brain at Weeks 12, 16, 20, and 24, and to evaluate the safety and tolerability of rituximab in patients with RRMS. Secondary objectives were to evaluate additional MRI parameters and the proportion of patients relapsing. The trial met its primary efficacy endpoint and all secondary endpoints. Rituximab was safe and generally well tolerated in this study through 48 weeks though the rate of infusion-associated adverse events, particularly after the first infusion, was higher in rituximab-treated patients (78%) than in placebo patients (40%); corticosteroid pre-medication was not administered before or at the time of infusion. Study U2787g provides proof of principle that an anti-CD20 therapeutic approach can reduce both MRI and clinical evidence of inflammatory activity in adults with RRMS [7].

#### 1.1.4.3 Rituximab in PPMS

A single Phase II/III, randomized, double blinded, placebo-controlled trial was conducted with rituximab in PPMS. The findings, summarized below, represent the largest and longest duration trial experience to date evaluating the safety and efficacy of anti-CD20 therapy in individuals with multiple sclerosis.

Study U2786g (OLYMPUS) was a Phase II/III randomized, double-blind, parallel-group, placebo-controlled, multicenter study evaluating the safety and efficacy of rituximab in patients with PPMS over a 96 week treatment period consisting of 4 treatment cycles with dual infusions of 1000 mg (2000 mg/cycle). Although the trial did not demonstrate significant primary efficacy on time to confirmed disease progression as measured by Expanded Disability Status Scale (EDSS), a difference was observed with 38.5% of patients in the placebo group experiencing confirmed disease progression vs. 30.2% in the rituximab group. Biological activity was evidenced by significantly lower T2 lesion volume accumulation on brain MRI, a secondary efficacy endpoint, in rituximab-treated patients compared with placebo (p=.0008). Subgroup analyses suggest that PPMS patients with evidence of active disease may have shown significant clinical treatment response as measured by time to confirmed disease progression over a 96-week timeframe. Factors that appeared prognostic for disease progression and potentially predictive of treatment response in the rituximab group included younger age, presence of contrast enhancing lesions at baseline on brain MRI, and higher MS severity score.

Rituximab was generally safe and well tolerated in Study U2786g. The proportions of patients with at least one adverse event (100% placebo vs. 99% rituximab) and one serious adverse event (13.6% placebo vs. 16.1% rituximab) were comparable between treatment groups. Three adverse events that occurred during the study led to death: one in the rituximab group following recurrent aspiration pneumonias and two in the placebo group due to pneumonia and cardiopulmonary failure. More infusion-associated adverse events were observed in rituximab-treated patients (73.6% vs. 40.3% for placebo), particularly after the first infusion, but rates declined in both groups to similar levels upon successive infusions. Patients were not premedicated with glucocorticoids before rituximab infusions in Study U2786g. The vast majorities (92%) of infusion associated events in rituximab treated patients were mild to moderate in severity; no Grade 4 or 5 infusion-associated events were observed. The proportion of patients with at least one infection was comparable between groups (68.2% rituximab vs. 65.3% placebo), but a higher proportion of patients with at least one serious infection was observed in the rituximab-treated group (4.5%) compared with placebo (<1%). No opportunistic infections occurred.

Treatment with rituximab was associated with rapid and near-complete depletion of circulating CD19 positive B lymphocytes beginning 2 weeks post-treatment through 96 weeks. Approximately 35% of rituximab-treated patients had recovered peripheral CD19 B-cell counts to 80 cells/μL (laboratory defined lower limit of normal (LLN) in healthy volunteers) within 48 weeks after the last dose. Median circulating CD3 T-lymphocyte counts were not appreciably altered by rituximab. At any time in the trial, IgM levels were below the LLN in 31.7% of rituximab-treated patients and 5.9% of placebo-treated patients. The proportion of patients with IgG and IgA levels below LLN

were not different between groups. The incidence of infectious adverse events and infectious SAEs did not appear higher in patients with immunoglobulin levels (all isotypes) below LLN in either treatment group compared with patients with immunoglobulin levels in the normal range or above upper limit of normal (ULN) [31].

### 1.1.5 Rebif®

The active comparator for this study is Rebif® (interferon β-1a), which has been approved for treatment of relapsing multiple sclerosis.

The efficacy and safety of Rebif® was demonstrated in the PRISMS study (The Prevention of Relapses and Disability by Interferon β-1a Subcutaneously in Multiple Sclerosis) which led to the approval of Rebif® in RMS. This was a multicentre controlled trial of 560 patients with an EDSS score between 1.0 and 5.0 and at least two relapses in the preceding 2 years. Patients were randomized to 2-year treatment with placebo or IFN β-1a (22 or 44 μg subcutaneously three times weekly). Following the 2 years of treatment, both doses of Rebif® showed significant benefits compared with placebo on major efficacy outcome measures. There was a non-significant trend towards greater efficacy with the higher dose on most clinical measures, and a statistically significant dose-effect favoring the higher dose in terms of impact on the number of T2-active lesions. In a subgroup of patients with more severe disease (baseline EDSS >3.5), the 44-μg dose delayed progression of disability significantly better than either the placebo or the 22 μg dose. Neutralizing antibodies were significantly less frequent in the 44 μg group than in the low-dose group [33].

After 2 years, patients who had initially received placebo in the PRISMS study were re-randomized to receive Rebif® (22 or 44 μg subcutaneously three times weekly) and were followed for an additional 2 years. By the end of the 4 year period, patients who had switched from placebo to Rebif® experienced an approximate 50% reduction in ARR compared with the end of year 2. Also, after 4 years, the higher dose approached significance for annual relapse rates (0.8 for 22 μg vs. 0.72 for 44 μg; $p=0.069$). The mean ARR was significantly lower in patients who had received Rebif® for the full 4 years compared with those who had received placebo for the first 2 years. During Years 3 and 4, relapse rates were significantly lower for the 44 μg group with relapse rates decreasing progressively with each year of treatment – 0.92, 0.82, 0.57, and 0.44 relapses/year for each year of the study. Patients who received the highest cumulative dose of active therapy had the lowest rate of disability progression. The time to first confirmed EDSS progression was 42.1 months for the 44 μg group compared with 24.2 months for the crossover group. The time to first confirmed progression did not differ significantly between the 22 μg group (35.9 months) and the crossover group [34].

Rebif® showed superiority versus Avonex® in the EVIDENCE trial. This was a randomized, controlled, multicenter trial which compared the efficacy and safety of Rebif® 44 μg subcutaneously three times weekly and Avonex® 30 μg i.m. once weekly in 677 patients with RRMS. The primary endpoint was the proportion of patients who were relapse free at 24 weeks; the principal MRI endpoint was the number of active lesions per patient per scan at 24 weeks. After 24 weeks, 74.9% of patients receiving Rebif® 44 μg three times a week remained relapse free compared with 63.3% of those given Avonex® 30 μg once a week. Patients receiving Rebif® 44 μg three times a week had fewer active

MRI lesions (p = 0.001 at 24 and 48 weeks) compared with those receiving Avonex® 30 µg once a week. Injection-site reactions were more frequent with Rebif® 44 µg three times a week (83% vs. 28%, p = 0.001), and there were asymptomatic abnormalities of liver enzymes (18% vs. 9%, p= 0.002) and altered leukocyte counts (11% vs. 5%, p=0.003) compared with the Avonex® 30 µg once a week dosage. Neutralizing antibodies developed in 25% of Rebif® 44 µg three times a week patients and in 2% of patients receiving Avonex® 30 µg once a week [32].

Rebif® has also been studied in the SPMS population. The SPECTRIMS study was a multicenter, randomized, parallel-group, placebo-controlled study which tested two doses of Rebif® in patients with SPMS. Patients had to have clinically definite SPMS which was defined as progressive deterioration of disability for at least 6 months with an increase of at least 1 EDSS point over the previous 2 years (or 0.5 point between EDSS score of 6.0 and 6.5), with or without relapses, following an initial course of RRMS. Baseline EDSS scores had to be from 3.0 to 6.5 and the pyramidal functional score of at least 2. Patients were randomized to 3-year treatment with placebo or IFN β-1a (22 or 44 µg subcutaneously three times weekly). The primary outcome was time of confirmed progression, defined as increase from baseline by at least 1 EDSS point (or 0.5 point if baseline EDSS ≥ 5.5), confirmed 3 months later with no intervening score lower than the minimum required level. The primary outcome was not significantly influenced by treatment with Rebif® as compared to placebo (p = 0.146). A significant benefit was seen on relapse rate for both doses of Rebif®. These findings suggest that treatment with Rebif® has clinical benefit in SPMS, predominantly affecting relapses, but only modest effect on disability [35].

In controlled clinical trials, the most commonly observed adverse reactions were: injection-site reactions, influenza-like symptoms (headache, fatigue, fever, rigors, chest pain, back pain, myalgia), elevated liver enzymes, hematological abnormalities, abdominal pain, and depression. Most of these adverse reactions are unique to treatment with interferon beta, presenting potential difficulties in maintaining blinding in controlled clinical trials. Summary of the most frequent Rebif® adverse reactions by MedDRA system organ class have been summarized in Table 1.

**Table 1: Summary of the Most Frequent Rebif® Adverse Reactions by MedDRA System Organ Class**

| System Organ Class | Very Common ADR *frequency of occurrence ≥ 1/10* | Common ADR *frequency of occurrence ≥ 1/100 to 1/10* |
|---|---|---|
| **General disorders and administration site conditions** | Injection site inflammation, injection site reaction, influenza-like symptoms | Injection site pain, fatigue, rigors, fever |
| **Investigations** | Asymptomatic transaminase increase | Severe elevation of transaminase |
| **Blood and lymphatic system disorders** | Neutropenia, lymphopenia, leucopenia, thrombocytopenia, anaemia | |
| **Psychiatric disorders** | | Depression, insomnia |
| **Nervous system disorders** | Headache | |
| **Gastrointestinal disorders** | | Diarrhoea, vomiting, nausea |
| **Skin and subcutaneous tissue disorders** | | Pruritus, rash, erythematous rash, macula-papular rash |
| **Musculoskeletal and connective tissue disorders** | | Myalgia, arthralgia |

*Based on Rebif® Summary of Product Characteristic 2010 [36]. Please refer to local label for more details.*

Severe liver injury, including some cases of hepatic failure requiring liver transplantation, has been reported rarely in patients taking Rebif®. Treatment with Rebif® should be stopped immediately if jaundice or other symptoms of liver dysfunction appear [36, 37]. Please refer to Section 6.2.2 for further details.

Neutralizing antibodies to IFN β-1a can develop in some patients, usually following the first year of therapy. Long-term consequences of these antibodies are still not known, however, current evidence shows that they may reduce the efficacy of the drug. The antibodies tend to cross-react with different IFN β formulations. For this reason, switching to another IFN β drug is unlikely to be effective [38, 39].

## 1.2 Rationale for the Study

This study serves as a pivotal Phase III clinical trial designed to demonstrate the efficacy and safety of ocrelizumab in relapsing multiple sclerosis in comparison to high dose high frequency IFN (Rebif®). This study is part of a broader, confirmatory clinical development program investigating the safety and efficacy of ocrelizumab in patients

with both primary progressive and relapsing multiple sclerosis. A Phase II study WA21493/ACT4422G is ongoing in RRMS patients and three Phase III pivotal trials are planned, (including the one presented in this protocol), two in RMS and one in PPMS. Please refer to Section 3.1.1 for further details on study design and choice of comparator.

# 2. OBJECTIVES

## 2.1 Primary Objective

The primary objective of this study is to assess whether the efficacy of ocrelizumab 600 mg (given as dual infusions of 300 mg on Days 1 and 15 of the first 24-week treatment cycle and as a single infusion of 600 mg on Day 1 of each 24-week treatment cycle thereafter) intravenously every 24 weeks is superior to Rebif® as measured by the annualized protocol-defined* relapse rate by two years (96 weeks) in patients with relapsing multiple sclerosis.

**See Section 5.3.2.1 for the definition of protocol-defined relapse.*

## 2.2 Secondary Objectives

The secondary objectives of this study are to evaluate whether the efficacy of ocrelizumab is superior to Rebif®, as reflected by the following measures:

- The time to onset of sustained disability progression for at least 12 weeks during the 96-week comparative treatment period. **
- The time to onset of sustained disability progression for at least 24 weeks during the 96-week comparative treatment period. **
- The proportion of relapse-free patients by 96 weeks.
- The change in total T2 lesion volume as detected by brain MRI from baseline to Week 96.
- The total number of new, and/or enlarging T2 hyperintense lesions as detected by brain MRI at week 24, week 48 and week 96.
- The change in Multiple Sclerosis Functional Composite Scale (MSFCS) score from baseline to Week 96.
- The change in brain volume as detected by brain MRI from Week 24 to Week 96.

***See Section 5.3.2.2 for the definition of sustained disability progression.*

**Safety:**

To evaluate the safety and tolerability of ocrelizumab 600 mg (given as dual infusions of 300 mg on Days 1 and 15 of the first 24-week treatment cycle and as a single infusion of 600 mg on Day 1 of each 24-week treatment cycle thereafter) intravenously every 24 weeks in patients with relapsing MS.

**Pharmacokinetics/Pharmacodynamics:**

To explore the pharmacokinetics, immunogenicity and pharmacodynamics of ocrelizumab in patients with relapsing MS.

## 2.3 Exploratory Objectives

- The change in low-contrast visual acuity from baseline to Weeks 48 and 96.
- The change in the Symbol Digit Modalities Test from baseline to Weeks 48 and 96.
- The annualized relapse rate, based on clinical and protocol-defined relapses at the end of the 96-week comparative treatment period.
- The total number of T1 gadolinium-enhanced lesions as detected by brain MRI at Weeks 24, 48, and 96.
- The change in brain volume as detected by brain MRI from baseline to Week 96.
- The change in brain volume as detected by brain MRI from Week 48 to Week 96.
- The change in Multiple Sclerosis Functional Composite Scale (MSFCS) score from baseline to Week 48.
- The cumulative change in EDSS scores, measured in area under the curve (AUC) by Week 96.
- The change in EDSS from baseline to Weeks 48 and 96.
- The change in timed 25 foot walk from baseline to Weeks 48 and 96.
- The change in 9-hole peg test from baseline to Weeks 48 and 96.
- The change in paced auditory serial addition test (PASAT) from baseline to Weeks 48 and 96.
- The time to onset of sustained 20% increase in 9-hole peg test for at least 12 weeks.
- The time to onset of sustained 20% increase in timed 25 foot walk for at least 12 weeks.
- Patient Reported Outcomes (PROs): Modified Fatigue Impact Scale (MFIS), EuroQol instrument (EQ-5D), Center for Epidemiology Studies Depression Scale (CES-D) and Medical Outcomes Study 36-Item Short Form Survey Instrument (SF-36) at baseline, Week 48 and Week 96.
- The change in Karnofsky Performance Status Scale (clinician-reported version) score from baseline to Weeks 48 and 96.

## 2.4 Roche Clinical Repository (RCR) Exploratory Objectives

The Roche Clinical Repository (RCR) is a centrally administered facility for the long term storage of human biological specimens including body fluids, solid tissues and derivatives thereof (e.g. DNA, RNA proteins/ peptides). Specimens stored in the RCR will be used to:

- Study the association of biomarkers with efficacy and/ or adverse events associated with ocrelizumab
- Increase the knowledge and the understanding of biology of multiple sclerosis and mode of action of ocrelizumab.

# 3. STUDY DESIGN

## 3.1 Overview of Study Design and Dosing Regimen

**Figure 1: Overview of Study Design**

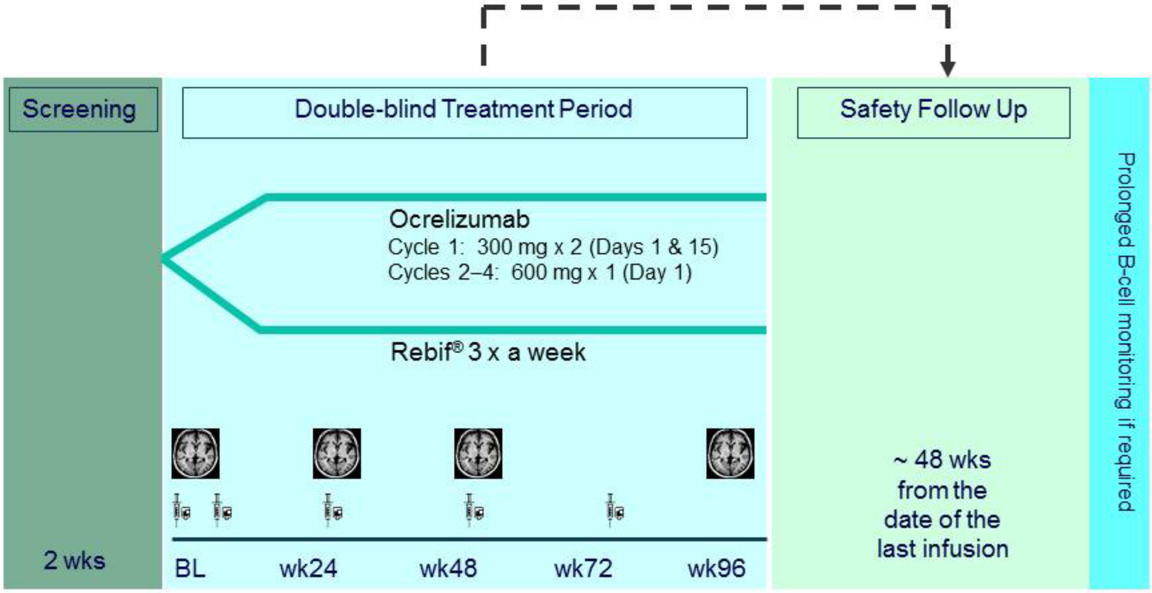


The study will consist of the following periods:

**<u>Screening:</u>**

Consenting patients will enter the 2-week screening period to be evaluated for eligibility. Procedures at screening will include collecting medical history, medical examination including thorough neurological exam, EDSS score, LCVA score, SDMT score, MSFCS score, ECG, blood and urine sampling. Please see Table 3 - "Schedule of Assessments: Screening Through the End of Double-Blind Treatment Period" for further details.

*<u>Please note</u> that based on local Ethics Committees or National Competent Authority requirements, additional diagnostic testing may be required for selected patients or selected centers to exclude tuberculosis, Lyme disease, HTLV-1 associated myelopathy (HAM), acquired immune deficiency syndrome (AIDS), hereditary disorders, connective tissue disorders, or sarcoidosis.*

**<u>Treatment Period:</u>**

**- Double-blind, comparative treatment period**

Eligible patients will be randomized via IxRS into one of two treatment groups: ocrelizumab 600 mg regimen (group A) or interferon β-1a - Rebif® (group B) – please refer to Table 5 and Table 8 for more details.

During the double-blind comparative treatment period, patients will be assessed at clinical visits as per Schedule of Assessments: Screening Through the End of Double-Blind Treatment Period – please refer to Table 3 for further details.

Prior to the next cycle of study drug, patients will be evaluated for pre-specified conditions and laboratory abnormalities to allow for re-treatment please refer to Section 6.1.4 for more details.

**Patients who complete the 96-week treatment period may become eligible for a separate open-label extension study, under a separate protocol.**

**<u>Please note:</u> Patients who discontinue from study medication within the 96-week double-blind, comparative phase (treatment period) of the study will enter the Safety Follow up Period (see below); they will not be eligible for the open-label extension study, even if they complete the 96-week treatment period.**

**<u>Safety Follow up Period</u>**

Patients who discontinue treatment with study drug will enter the Safety Follow up Period for at least 48 weeks counting from the date of the last infusion of the ocrelizumab/ocrelizumab placebo. However, if after this time the peripheral blood B-cells remain depleted, patient should continue to be monitored at 24-week intervals until B-cell count has returned to the baseline value or to the lower limit of the normal range (whichever is the lower). Please refer to Figure 2 for more details.

**Figure 2: Safety Follow up - Variable B-cell Monitoring.**

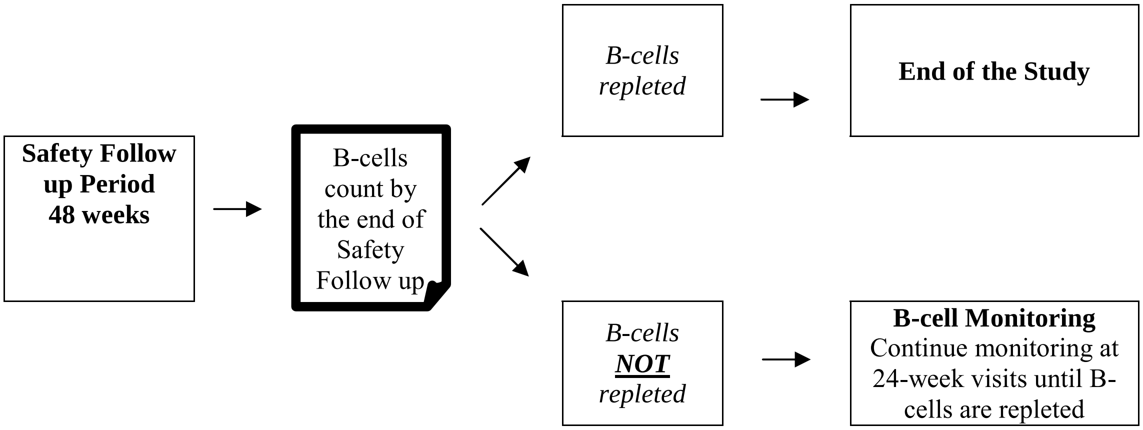


*Please note: patients in Safety Follow up who receive other B-cell targeted therapies will only be followed for 48 weeks from the date of the last infusion of the study drug regardless of their B-cell count.*

During Safety Follow up patients will be assessed at clinical visits every 12 weeks. Telephone interviews will be performed every 4 weeks. If prolonged B-cell monitoring is required, patients will be assessed at clinical visits every 24 weeks and telephone interviews will be performed every 12 weeks. Please refer to Schedule of Assessments: Safety Follow up (including prolonged B-cell monitoring if required)—Table 4—for further details.

**Every effort should be made to have patients who withdraw from Study Medication complete the Safety Follow up Period and all related assessments, <u>regardless of whether or not they receive alternative treatment for MS.</u>**

**Table 2: Overview of Dosing Regimen**

| Group | Treatment Period[1,2] | | | | |
|---|---|---|---|---|---|
| | 1st Cycle[3] (Weeks 1-24) | | 2nd Cycle[3] (Weeks 24-48) | 3rd Cycle[3] (Weeks 48-72) | 4th Cycle[3] (Weeks 72-96) |
| | **Day 1 Infusion** | **Day 15 Infusion** | **Week 24 Infusion** | **Week 48 Infusion** | **Week 72 Infusion** |
| **A Ocrelizumab 600 mg regimen** | **Ocrelizumab** 300 mg i.v. | **Ocrelizumab** 300 mg i.v. | **Ocrelizumab** 600 mg i.v. | **Ocrelizumab** 600 mg i.v. | **Ocrelizumab** 600 mg i.v. |
| **B Rebif® regimen[4]** | **Rebif®** s.c. three times per week | → | → | → | → |

1. The treatment period consists of 96 weeks of treatment; patients will receive a maximum of 4 treatment cycles.
2. Each treatment cycle has a duration of 24 weeks. The first cycle consists of two 300 mg ocrelizumab i.v. infusions separated by 14 days. Cycles 2 – 4 consist of a single i.v. infusion of 600 mg ocrelizumab.
3. Prior to each infusion, a clinical evaluation will be performed to ensure that the patient remains eligible for treatment.
4. Please refer to Table 8 for detailed Rebif® dosing regimen.

Please note: 100 mg of methylprednisolone i.v. will be administered in both treatment arms prior to each infusions of ocrelizumab/ocrelizumab placebo.

### 3.1.1 Rationale for Study Design

**Rationale for the use of an active comparator**

There is consensus in the MS community, that the use of placebo in Phase III studies of patients with RMS is (except in exceptional circumstances) ethically indefensible, due to the availability of established, effective therapies [40].

**Rationale for choice of active comparator**

Interferon β-1a 44 µg s.c. 3 times weekly (Rebif®) has been chosen as the active comparator for the ocrelizumab RMS, Phase III clinical program, based on its consistent evidence of efficacy on reducing MRI activity, relapses and disease progression in patients with relapsing forms of MS see Section 1.1.5.

**Rationale for double-blind, double-dummy study design**

The use of a double-blind, double-dummy study design minimizes the potential for bias and safeguards the integrity of the clinical data generated from this study. It is acknowledged that this approach increases patient's burden. However, this design reduces the risk of concluding that superiority to the active comparator was driven by patient and assessor bias. Regulatory agencies have mandated this study design be implemented throughout the Phase III clinical program. For additional measures intended to minimize bias, please see below.

**Rationale for choice of study population**

This study plans to enroll RMS patients with an EDSS score of 0 to 5.5 at screening who had two documented clinical attacks within the previous 2 years or one clinical attack that occurred within the last year prior to screening. These criteria have been implemented to further characterize the benefits of treatment with ocrelizumab in a wide range of RMS patients with varying degrees of disease activity and severity.

The age range will be limited to ≤ 55 years to avoid confounding by neurological conditions prevalent in older individuals, including but not limited to microvascular disease.

Exclusion of patients who have failed or cannot tolerate Rebif® prevents these patients from being randomized to further Rebif® therapy and reduces the potential for unbalanced dropout rates. Additional exclusion criteria, relating to concomitant diseases, laboratory parameters, and previous medications help to ensure patients safety in the study – please refer to Section 4.3 for more details.

**Rationale for study endpoints**

The proposed study endpoints are widely accepted as clinically relevant and have been used in numerous pivotal clinical trials, in relapsing MS. The primary endpoint for the study will be annualized relapse rate (ARR) over 96 weeks, based on protocol-defined relapses. Key secondary endpoints will include the time to onset of sustained disability progression, confirmed at scheduled clinic visits, for at least 12 and 24 weeks. Prevention of relapses as well as the prevention or delay of accumulation of sustained neurological disability are meaningful goals in the treatment of patients with MS.

**Rationale for the treatment duration**

The 96-week treatment duration has been chosen to allow for the assessment of clinical efficacy and safety over 2 years of treatment, consistent with current regulatory guidelines.

Patients who complete the double-blind treatment period may become eligible for participation in the extension study under a separate protocol. This will allow for collection of safety information with long-term exposure.

Please note: Patients who discontinue from study medication within the 96-week double-blind, comparative phase (treatment period) of the study will enter the Safety Follow up Period (see below); they will not be eligible for the open-label extension study, even if they complete the 96 week treatment period.

**Rationale for the Safety Follow up Period (including Prolonged B-cell Monitoring)**

Data collected during this period will allow evaluation of B-cell repletion after stopping anti-CD20 treatment and collection of safety and efficacy data to document maintenance of the effect and/or the potential for a withdrawal effect. Based on results obtained from Study U2786g with rituximab [7] up to 60-65% of the ocrelizumab-treated patients are anticipated to enter the B-cell Monitoring portion of the safety follow up period with targeted assessment every 24 weeks until their B-cell counts recover.

**Rationale for the use of methylprednisolone**

In order to mitigate the risk and severity of infusion-related reactions, 100 mg of i.v. methylprednisolone will be administered to all patients, approximately 30 minutes prior to administration of ocrelizumab/ocrelizumab placebo. In order to mitigate the risk, that even a low dose of methylprednisolone may have a small effect on the efficacy outcomes of the study, methylprednisolone will be administered to patients in all treatment groups. It is of note that the dose of methylprednisolone used for premedication will be up to 50 times smaller than that used for the symptomatic treatment of relapses in MS.

**Additional measures to mitigate bias**

The use of high dose high frequency (HDHF) interferon β, as the active comparator for this study, presents some difficulties for maintaining patient and physician blinding – please see Section 1.1.5.

To prevent potential unblinding as a result of adverse events or changes to laboratory results, the following, additional measures have been implemented:

– **The Examining Investigator/EDSS assessor** will perform the neurological examination, document the Kurtzke Functional Systems (KFS) scores and assess EDSS scores. The examining physician/EDSS assessor will not be involved with any aspect of medical management of the patient and will not have access to patient data. Every effort will be made to ensure that there is no change in the examining physician/EDSS assessor throughout the course of the study for any individual patient. The examining physician/EDSS assessors will be trained and instructed not to discuss what adverse effects (if any) the patient is experiencing from their medication. Examining physician/EDSS assessors will receive training in performing EDSS assessments prior to the beginning of the study and must have successfully passed an examination on performance of the *Neurostatus EDSS examination* within 24 months of participation. All examining physician/EDSS assessors will receive ongoing training on performance of the *Neurostatus EDSS examination* throughout the course of the study.

– **Patient education:** Prior to being examined by the Examining Investigator/EDSS assessor, patients will be instructed not to discuss what (if any) adverse effects they may be experiencing. Treating physicians and/or study coordinators should remind patients of these instructions prior to EDSS assessments and this should be documented in the source documents.

– **Blinded, central MRI assessments:** A blinded, central MRI reader will assess all on-study MRI scans. These assessments will provide independent confirmation of the relative changes in immune-mediated, CNS damage.

– **Blinding of laboratory parameters:** Laboratory parameters that may lead to unblinding to treatment assignment, such as FACS cell counts including $CD19^+$ cells, lymphocyte count, IgM and IgG levels, and type I interferon neutralizing antibody levels will be blinded in all patients, except those meeting unblinding criteria for safety reasons.

### 3.1.2 Rationale for Dose Selection

The dose for the ocrelizumab Phase III clinical program was chosen to bring the MS community significant improvement in clinical efficacy versus current standard of care, with acceptable safety. The dose of ocrelizumab in the Phase III clinical program is 600 mg ocrelizumab every 24 weeks (administered as dual infusions of ocrelizumab 300 mg on Days 1 and 15 of the first 24-week treatment cycle, and 600 mg on Day 1 of each 24-week treatment cycle thereafter). This dose has been established as the lowest, maximally effective dose, based on the results from study WA21493/ACT4422g. The safety of this dose has substantial support from the Phase III clinical program in rheumatoid arthritis (RA), an analogous, although systemic autoimmune disease, in a population at greater risk.

ACT2847g was a Phase I/II, dose escalation study in patients with RA, examining 5 dose regimens. In ACT2847g, the two lowest dose groups (receiving less than 200 mg x 2) demonstrated reduced clinical benefits on some endpoints, earlier return of peripheral B-cell counts and higher rates of immunogenicity. In the RA Phase III program, with the exception of patients recruited from Asia, the dose of 200 mg x 2 established a safety profile comparable to placebo. The higher dose of 500 mg x 2 demonstrated apparently superior efficacy, especially in “high hurdle” clinical endpoints and joint preservation, based on X-ray imaging.

In the MS population (study WA21493/ACT4422g), two doses of ocrelizumab were studied, 2000 mg (administered as dual 1000 mg infusions on Days 1 and 15 of the first, 24-week treatment cycle) and 600 mg (administered as dual 300 mg infusions on Days 1 and 15 of the first treatment cycle). Pre-specified primary and secondary efficacy analyses for Study WA21493/ACT4422g indicate that 300 mg x 2 of ocrelizumab is highly effective in suppressing MRI lesion activity and reducing the risk of clinical relapses in RRMS patients over 24 weeks. No difference in efficacy was seen between the ocrelizumab 1000 mg x 2 and 300 mg x 2 doses, on either MRI or clinical endpoints, in the ITT study population. However, exploratory analyses, stratifying groups according to baseline MRI activity, suggest superior efficacy with 1000 mg x 2 versus 300 mg x 2, at 24 weeks, in patients with MRI activity at baseline (≥ 4 enhancing lesions). Similarly, in these patients, the 1000 mg x 2 dose was apparently more effective than the 300 mg x 2 dose at Week 24 and (to a lesser extent) at Week 48, in reducing the absolute number of clinical relapses. Neither the MRI nor the clinical efficacy differences are statistically significant; however, these results suggest reduction of clinical efficacy at lower doses, in active MS patients. This apparent dose effect was seen despite the fact that linear kinetics (so that complete receptor occupancy can reasonably be assumed) and near complete peripheral CD19 suppression were observed for both doses. Preclinical studies in primates have shown differential susceptibility of tissue resident versus circulating B-cell populations in response to anti-CD20 antibodies. [41, 42, 43]. As tissue resident B-cell populations are beyond our ability to measure directly, it is likely that peripheral CD19 count is a sensitive but non-specific pharmacodynamic marker for anti-CD20 efficacy.

Conclusion

Based on available data, the dose of 600 mg of ocrelizumab i.v. (given as dual infusions of 300 mg 14 days apart for the first 24 weeks and a single infusion of 600 mg every 24 weeks thereafter) is the most likely dose to be able to demonstrate robust clinical efficacy, an acceptable safety profile and a low risk of immunogenicity, maximizing the likelihood of significant benefit versus standard of care in patients with relapsing MS.

#### 3.1.3 End of Study

The end of the study has been defined as the date at which the last data point from the last patient, which was required for statistical analysis as defined in Data Analysis Plan (DAP), was received.

### 3.2 Number of Subjects / Assignment to Treatment Groups

Approximately 800 patients (400 per treatment arm) will be recruited over a planned recruitment period of 16 months.

Patients will be randomized in 2 groups in a 1:1 ratio. An independent Interactive Voice and Web Response System (IxRS) provider will conduct randomization and hold the treatment assignment code. Patients will be stratified by region (US vs. ROW) and baseline EDSS ($< 4$ and $\geq 4$).

### 3.3 Centers

This will be a multicenter, international study. It is anticipated that approximately 220 centers worldwide will participate.

## 4. STUDY POPULATION

Under no circumstances are patients who enroll in this study permitted to be re-randomized to this study and enrolled for a second course of treatment.

### 4.1 Overview

Adult patients with relapsing MS who fulfill the eligibility criteria specified in Sections 4.2 and 4.3 are eligible for enrollment into the study.

#### 4.1.1 Recruitment Procedures

Patients will be identified for potential recruitment using pre-screening enrollment logs and pre-ID website.

Patients who are candidates for enrollment into the study will be evaluated for eligibility by the investigator to ensure they fulfill eligibility criteria (please refer to Sections 4.2 and 4.3).

All patients must sign the informed consent form prior to screening and prior to any changes to their existing medication for the purposes of enrollment into the trial.

No patient may begin treatment prior to randomization and assignment of a medication number. Under no circumstances are patients who enroll in this study and who have completed treatment as specified, permitted to be re-randomized to this study.

The investigators will be notified by the Sponsor if the study is placed on clinical hold and when the study is completed or closed to further patient enrollment.

No replacement for patients who withdraw from the study after randomization is planned.

## 4.2 Inclusion Criteria

1. Ability to provide written, informed consent and to be compliant with the schedule of protocol assessments.
2. Ages 18-55 years at screening, inclusive.
3. Diagnosis of MS, in accordance with the revised McDonald criteria (2010).
4. At least 2 documented clinical attacks within the last 2 years prior to screening, or one clinical attack in the year prior to screening (but not within 30 days prior to screening).
5. Neurological stability for ≥ 30 days prior to both screening and baseline.
6. EDSS, at screening, from 0 to 5.5 inclusive.
7. Documented MRI of brain with abnormalities consistent with MS prior to screening.
8. Patients of reproductive potential must use reliable means of contraception as described below as a minimum (adherence to local requirements, if more stringent, is required*):
    - Two methods of contraception throughout the trial, including the active treatment phase AND for 48 weeks after the last dose of ocrelizumab, or until their B-cells have repleted, whichever is longer. Acceptable methods of contraception include one primary (e.g., systemic hormonal contraception or tubal ligation of the female partner, vasectomy of the male partner) AND one secondary barrier method (e.g., latex condoms, spermicide) OR a double barrier method (e.g., latex condom, intrauterine device, vaginal ring or pessary plus spermicide [e.g,. foam, vaginal suppository, gel, cream]).
9. For patients of non reproductive potential (adherence to local requirements, if more stringent, is required*):
    - Women may be enrolled if postmenopausal (i.e., spontaneous amenorrhea for the past year confirmed by an FSH level greater than 40 mIU/mL) unless the patient is receiving a hormonal therapy for their menopause or surgically sterile (i.e., hysterectomy, complete bilateral oophorectomy);
    - Men may be enrolled if they are surgically sterile (castration).

* Based on local Ethics Committees or National Competent Authority feedback, additional requirements to assure contraception or to confirm menopause may be required (e.g. serum estradiol compatible with post-menopause status, longer duration of amenorrhea, higher level of FSH).

## 4.3 Exclusion Criteria

Patients who meet the following criteria must be excluded from study entry:

1. Diagnosis of primary progressive MS.
2. Disease duration of more than 10 years in patients with an EDSS ≤ 2.0 at screening.

3. Inability to complete an MRI (contraindications for MRI include but are not restricted to claustrophobia, weight ≥ 140 kg, pacemaker, cochlear implants, presence of foreign substances in the eye, intracranial vascular clips, surgery within 6 weeks of entry into the study, coronary stent implanted within 8 weeks prior to the time of the intended MRI, etc).
4. Known presence of other neurological disorders which may mimic MS including but not limited to: neuromeylitis optica, Lyme disease, untreated vitamin B12 deficiency, neurosarcoidosis and cerebrovascular disorders.

**<u>Exclusions Related to General Health</u>**

5. Pregnancy or lactation.
6. Any concomitant disease that may require chronic treatment with systemic corticosteroids or immunosuppressants during the course of the study.
7. History or currently active primary or secondary immunodeficiency.
8. Lack of peripheral venous access.
9. History of severe allergic or anaphylactic reactions to humanized or murine monoclonal antibodies.
10. Significant or uncontrolled somatic disease or any other significant disease that may preclude patient from participating in the study.
11. Congestive heart failure (NYHA III or IV functional severity).
12. Known active bacterial, viral, fungal, mycobacterial infection or other infection, excluding fungal infection of nail beds.
13. Infection requiring hospitalization or treatment with i.v. antibiotics within 4 weeks prior to baseline visit or oral antibiotics within 2 weeks prior to baseline visit.
14. History or known presence of recurrent or chronic infection (e.g., hepatitis B or C, HIV, syphilis, tuberculosis).
15. History of progressive multifocal leukoencephalopathy (PML)
16. History of malignancy, including solid tumors and hematological malignancies, except basal cell carcinoma, *in situ* squamous cell carcinoma of the skin, and *in situ* carcinoma of the cervix of the uterus that have been previously completely excised with documented, clear margins.
17. History of alcohol or drug abuse within 24 weeks prior to baseline.
18. History or laboratory evidence of coagulation disorders.

**<u>Exclusions Related to Medications</u>***

19. Receipt of a live vaccine within 6 weeks prior to baseline.
    *In rare cases when patient requires vaccination with a live vaccine, the screening period may be extended but cannot exceed 8 weeks.*
20. Treatment with any investigational agent within 24 weeks of screening (Visit 1) or five half-lives of the investigational drug (whichever is longer).
21. Contraindications to or intolerance of oral or i.v. corticosteroids, including methylprednisolone administered i.v., according to the country label, including:
    a) Psychosis not yet controlled by a treatment;
    b) Hypersensitivity to any of the constituents.

22. Contraindication to Rebif® or incompatibility with Rebif® use, including:
    a) Current severe depression and/or suicidal ideation;
    b) Hypersensitivity to natural or recombinant interferon-β, or to any excipients;
    c) Previous suboptimal response to High Dose High Frequency (HDHF) interferon or cessation of HDHF interferon therapy due to poor tolerability;
    d) Prior cessation of Rebif® therapy due to toxicity, which is likely to recur.
23. Treatment with β interferons (with exemptions for HDHF interferon as listed above), glatiramer acetate, plasmapheresis, or other immunomodulatory therapies within 4 weeks prior to baseline.
24. Treatment with dalfamipridine (Ampyra®) unless on stable dose for ≥ 30 days prior to screening. Patients should remain on stable doses throughout the 96 week treatment period.
25. Previous treatment with B-cell targeted therapies (i.e. rituximab, ocrelizumab, atacicept, belimumab or ofatumumab).
26. Systemic corticosteroid therapy within 4 weeks prior to screening.**
27. Any previous treatment with alemtuzumab (Campath), anti-CD4, cladribine, mitoxantrone, daclizumab, BG12, teriflunomide, laquinimod, total body irradiation or bone marrow transplantation.
28. Treatment with cyclophosphamide, azathioprine, mycophenolate mofetil (MMF), cyclosporine, methotrexate, or natalizumab within 24 months prior to screening. NB P*atients previously treated with natalizumab will be eligible for this study only if duration of treatment with natalizumab was < 1 year.*
29. Treatment with fingolimod (FTY720, Gilenya®) or other S1P receptor modulator (i.e., BAF312) within 24 weeks prior to screening. NB *Only patients with T lymphocyte count ≥ LLN will be eligible for this study.*
30. Treatment with i.v. immunoglobulin within 12 weeks prior to baseline.

** Patients screened for this study should not be withdrawn from therapies for the sole purpose of meeting eligibility for the trial. Patients, who discontinue their current therapy for non-medical reasons, should specifically be informed before deciding to enter the study of their treatment options.*

*** The screening period may be extended (but cannot exceed 8 weeks) for patients who have used systemic corticosteroids for their MS before screening. For a patient to be eligible, systemic corticosteroids should not have been administered also between screening and baseline.*

**Exclusions Related to Laboratory Findings***

31. Positive serum β hCG measured at screening.
32. Positive screening tests for hepatitis B (hepatitis B surface antigen [HBsAg] positive, or positive hepatitis B core antibody [total HBcAb] confirmed by a positive viral deoxyribonucleic acid [DNA] polymerase chain reaction [PCR]) or hepatitis C (HepCAb).
33. Positive rapid plasma reagin (RPR).
34. CD4 count < 300/µL.

35. AST/SGOT or ALT/SGPT ≥ 2.0 Upper Limit of Normal (ULN).
36. Platelet count <100,000/µL (<100 x $10^9$/L).
37. Levels of serum IgG <5.65 g/L.
38. Levels of serum IgM < 0.55 g/L.
39. Total neutrophil count <1.5 x $10^3$/µL.

**Re-testing before baseline: in rare cases in which the screening laboratory samples are rejected by the central laboratory (example: hemolyzed sample) or the results are not assessable (example: indeterminate) or abnormal, the tests need to be repeated within 4 weeks. The last value before randomization must meet study criteria. In such circumstances, the screening period may need to be prolonged but should not exceed 8 weeks.*

Please note: based on local Ethics Committees or National Competent Authority requirements, additional diagnostic testing may be required for selected patients or selected centers to exclude tuberculosis, Lyme disease, HTLV-1 associated myelopathy (HAM), acquired immune deficiency syndrome (AIDS), hereditary disorders, connective tissue disorders, or sarcoidosis. Other specific diagnostic tests may be requested when deemed necessary by the investigator.

## 4.4 Concomitant Medication and Treatment

### 4.4.1 Definition of Concomitant Treatment

A concomitant medication is any drug or substance taken during the study, including the screening period. Over the counter medications and preventative vaccines received during the study are considered concomitant medications.

A concomitant procedure is any therapeutic or elective intervention (e.g. surgery, biopsy) or diagnostic evaluation (e.g. blood gas measurements, bacterial cultures) performed during the study, including the screening period.

Concomitant medications and procedures will be reported at each visit in the relevant form of eCRFs starting from the baseline visit (including medication and procedures taken between screening and baseline). Medications taken for the treatment of multiple sclerosis in the 2-year period prior to the baseline visit and medications taken for the symptoms of multiple sclerosis in the 3 month period prior to the baseline visit will be recorded at the baseline visit. Additionally, medications and medical/surgical procedures administered for any non-MS condition within 12 months prior to the baseline visit will also be recorded at the baseline visit.

### 4.4.2 Treatment for Symptoms of MS

The Treating Investigator should attempt to maintain therapies or treatments for symptoms related to MS (e.g., walking ability, spasticity, incontinence, pain, fatigue) reasonably constant throughout the study.

**Treatment of relapses:** patients who experience a relapse during the treatment period may receive treatment with i.v. (methylprednisolone) or oral corticosteroids, if judged to be clinically appropriate by the investigator. The following standardized treatment regimen should be used as warranted, 1 g i.v. methylprednisolone per day for a maximum

of 5 consecutive days. In addition, at the discretion of the investigator, corticosteroids may be stopped abruptly or tapered over a maximum of 10 days. Such patients should not discontinue the treatment period solely based on the occurrence of a relapse, unless the patient or investigator feels he or she has met the criteria for withdrawal (See Section 4.5 for further details).

#### *4.4.2.1 Prohibited Concomitant Treatments*

Therapies for MS noted in the exclusion criteria under "Exclusions Related to Medications" (Section 4.3) are not permitted during the study treatment period with the exception of systemic corticosteroids for the treatment of a relapse.

After patients have finished the treatment with ocrelizumab, they may receive alternative treatment for their MS as judged clinically appropriate by the Treating Investigator. However treatment with immunosuppressants, lymphocyte depleting agents, or lymphocyte trafficking blockers is not allowed while patients remain B-cell depleted due to the potential for increased risk of infection.

### 4.4.3 Immunization

No formal vaccination study has been conducted in ocrelizumab-treated patients. Results from studies done with a similar monoclonal antibody, rituximab, which provide additional information on the impact of anti-CD20 antibodies on the response to vaccinations, are presented below.

A small study comparing responses to influenza vaccination among RA patients receiving rituximab or tumor necrosis factor (TNF) inhibitors vs. normal controls found significantly lower post vaccination titres and protection rates (the proportion of a group with a titre $\geq 40$) in rituximab-treated patients compared to both control groups [44]. Results from another study are in line with the ones previously stated. This study showed that RA patients treated with rituximab compared with RA patients receiving methotrexate (MTX) and healthy adults had a severely hampered humoral immune response to influenza vaccine. This response remained reduced 6-10 months after rituximab treatment. In the rituximab group, patients who had been previously vaccinated achieved higher anti-influenza titers following influenza vaccination [45].

Another study assessed vaccine responses to influenza vaccine (containing 15 µg hemagglutinin/dose of B/Shanghai/361/02 (SHAN), A/New Caledonia 20/99 (NC) (H1N1) and A/California/7/04 (CAL) (H3N2)) among RA patients treated with disease-modifying anti-rheumatic drugs (DMARDs) with or without rituximab and normal control. After 4 weeks post vaccination geometric mean titers increased for New Caledonia and California antigens in all subjects, but not for the Shanghai antigen in the rituximab group. In addition, in rituximab treated patients, the percentage of responders was low for all three antigens tested, achieving statistical significance for California antigen [46].

In a randomized study with rituximab, patients with RA had comparable responses to tetanus recall antigen (39% vs. 42%), reduced responses to pneumococcal polysaccharide vaccine (43% vs. 82% to at least two pneumococcal antibody serotypes) and to Keyhole Limpet Haemocyanin (KLH) neoantigen (47% vs. 93%), when given 6 months after

rituximab as compared with patients only receiving methotrexate (MTX) [47]. Because of the mechanism of action, it is expected that similar findings would apply to ocrelizumab and patients treated with ocrelizumab may experience lower response rates to non-live vaccines than the general population.

Physicians are advised to review the immunization status of patients being considered for treatment with ocrelizumab and follow local/national guidance for adult vaccination against infectious disease. Known dates of immunizations will be recorded on specific eCRF pages. **Immunizations should be completed at least 6 weeks prior to first administration of ocrelizumab.**

Patients requiring de novo hepatitis B vaccination (which involves three separate doses of vaccine) should also have completed the course at least 6 weeks prior to the first infusion of study drug.

The safety of immunization with live viral vaccines following ocrelizumab or rituximab therapy has not been studied. Immunization with any live or live-attenuated vaccine (i.e. measles, mumps, rubella, oral polio vaccine, Bacille Calmette-Guerin (BCG), typhoid, yellow fever, vaccinia, cold adapted live influenza strain vaccine or any other vaccines not yet licensed but belonging to this category) is not recommended within 6 weeks of first dosing (see exclusion criteria), during ocrelizumab treatment and for as long as the patient is B-cell depleted.

## 4.5 Criteria for Premature Withdrawal

Patients have the right to withdraw from the study at any time for any reason.

**Patients must be withdrawn from treatment under the following circumstances:**

– Patients with Grade 4 infusion reaction, or severe allergic or anaphylactic reaction to an ocrelizumab infusion;

– If a woman becomes pregnant and chooses to carry her pregnancy during the study;

– Patients who demonstrate active hepatitis B or C infection, either new onset or reactivation in the case of hepatitis B;

– Patients who demonstrate active tuberculosis, either new onset or reactivation;

– Patients with PML;

– Patients with elevation of ALT ≥ 10 x ULN, jaundice or other clinical symptoms of liver dysfunction;

– Patients with persisting elevation of ALT > 3x ULN, or other clinical symptoms of liver dysfunction that did not resolve with Rebif®/Rebif® placebo dose modification (please refer to Section 6.2.2 for more details;

– Patients who decide to discontinue the treatment;

– The patient's Treating Investigator decides that discontinuation of treatment is in the best clinical interest of the patient.

Patients who withdraw from study treatment for any reason should complete the Safety Follow up Period. If the patient insists on discontinuing the study, he/she should be asked if he/she can still be contacted for further information. The outcome of that discussion should be documented in both the medical records and in the eCRF. If lost to follow-up, the investigator should contact the patient or a responsible relative by telephone followed by registered mail or through a personal visit to establish as completely as possible the reason for the withdrawal. A complete final evaluation at the time of the patient's withdrawal should be made with an explanation of why the patient is withdrawing from the study.

When applicable, patients should be informed of circumstances under which their participation may be terminated by the investigator without the patient's consent. The investigator may withdraw patients from the study in the event of intercurrent illness, adverse events, treatment failure, after a prescribed procedure, lack of compliance with the study and/or study procedures (e.g., dosing instructions, study visits), cure or any reason where it is felt by the investigator that it is in the best interest of the patient to be terminated from the study. Any administrative or other reasons for withdrawal must be documented and explained to the patient. If the reason for removal of a patient from the study is an adverse event, the principal specific event will be recorded on the eCRF. If possible, the patient should be followed until the adverse event has resolved.

An excessive rate of withdrawals can render the study non-interpretable; therefore, unnecessary withdrawal of patients should be avoided. Should a patient decide to withdraw, all efforts will be made to complete and report the observations prior to withdrawal as thoroughly as possible.

Please note: It is important to distinguish between "withdrawal from treatment" and "withdrawal from study". Patients who withdraw from treatment should be encouraged to remain in the study for the full duration of the Safety Follow Up Period (minimum of 48 weeks following the last infusion).

It should be noted that upon withdrawal from the study, any untested routine samples will be destroyed. However, information already obtained from samples up until the time of withdrawal will be used.

### 4.5.1 Withdrawal of Subjects from the Roche Clinical Repository (RCR)

Subjects who gave consent to provide RCR specimens have the right to withdraw their specimen from the RCR at any time for any reason. If a patient wishes to withdraw his/her consent to the testing of his/her specimen(s), the investigator must inform Roche in writing of the patient's wishes using the RCR Subject Withdrawal Form and enter the date of withdrawal in the patient's electronic Case Report Form (eCRF). A patient's withdrawal from the main trial does not, by itself, constitute withdrawal of the specimen from the RCR; likewise, a patient's withdrawal from the RCR does not constitute a withdrawal from the main trial**.**

### 4.5.2 Patient Agreement for Continuation in the Study (in case of sustained disability progression)

During the treatment period, in the event of sustained disability progression on EDSS confirmed for 24-weeks, the benefits and risks of study treatment should be reassessed with the patient prior to any further dosing, including a discussion of alternative treatment options available for that patient. The result of this discussion must be included in the patient's file, prior to any further dosing of study medication.

If, after the discussion, the patient decides not to continue with the study treatment, they should be discontinued from any further treatment, complete applicable Withdrawal from Treatment Visit procedures and be entered into the Safety Follow Up Period.

For definition of sustained disease progression, please refer to Section 5.3.2.2.

## 4.6 Replacement Policy (Ensuring Adequate Numbers of Evaluable Subjects)

### 4.6.1 For Subjects

Patients prematurely discontinued from the study for any reason will not be replaced.

### 4.6.2 For Centers

A center may be replaced for the following administrative reasons:

- Excessively slow recruitment.
- Poor protocol adherence.
- Sponsor's discretion (Sponsor refers to F. Hoffmann-La Roche Ltd and Genentech, Inc.)

# 5. SCHEDULE OF ASSESSMENT AND PROCEDURES

## Table 3: Schedule of Assessments: Screening Through the End of Double-Blind Treatment Period

| | Screen | Treatment Period | | | | | | | | | | | | |
|---|---|---|---|---|---|---|---|---|---|---|---|---|---|---|
| **Visit** | **1** | **2 BL** | **3** | **4** | **5** | **6** | **7** | **8** | **9** | **10** | **11** | **Delayed Dosing Visit [22]** | **Unscheduled Visit [23]** | **Withdrawal from Treatment Visit** |
| **Week** | **-2** | **-** | **w2** | **w12** | **w24** | **w36** | **w48** | **w60** | **w72** | **w84** | **w96** | | | |
| *Study Day (window in days)* | *-14* | *1* | *15 (± 1)* | *85 (±4)* | *169 (±1)* | *253 (±4)* | *337 (±1)* | *421 (±4)* | *505 (±1)* | *589 (±4)* | *673 (±1)* | | | |
| Informed consent [1] | x | | | | | | | | | | | | | |
| Medical history | x | | | | | | | | | | | | | |
| Review of eligibility criteria | x | x | | | | | | | | | | | | |
| CES-D, MFIS, EQ-5D, SF-36 | | x | | | | | x | | | | x | | | x |
| Patient's Assessment of Treatment Benefit | | | | | | | x | | | | x | | | x |
| C-SSRS | | x | | x | x | x | x | x | x | x | x | | x | x |
| Physical examination | x | x | x | | x | | x | | x | | x | x | | x |
| Vital signs [2] | x | x | x | x | x | x | x | x | x | x | x | x | x | x |
| 12 lead ECG (pre- and post-dose) [3] | x | x | | | | | | | x | | | | | x |
| Height | x | | | | | | | | | | | | | |
| Weight | x | | | | | | | | x | | x | | | x |
| Neurological exam and EDSS | x | x | | x | x | x | x | x | x | x | x | | x | x |
| MSFCS, LCVA, SDMT | | x | | x | x | x | x | x | x | x | x | | x | x |
| Karnofsky Performance Status Scale | | x | | | x | | x | | x | | x | | | x |
| MRI [4] | | x | | | x | | x | | | | x | | | x |
| Concomitant Treatment | | x | x | x | x | x | x | x | x | x | x | x | x | x |
| Adverse Events | *Only SAEs* | x | x | x | x | x | x | x | x | x | x | x | x | x |
| Potential relapses recorded | | x | x | x | x | x | x | x | x | x | x | x | x | x |
| Telephone interview (every 4 wks) [5] | x | | | – – – – | – – – – | – – – – | – – – – | – – – – | – – – – | – – – – | – – – → | | | x |

## Table 3: Schedule of Assessments: Screening Through the End of Double-Blind Treatment Period (Cont.)

| | Screen | Treatment Period | | | | | | | | | | | | |
|---|---|---|---|---|---|---|---|---|---|---|---|---|---|---|
| **Visit** | **1** | **2 BL** | **3** | **4** | **5** | **6** | **7** | **8** | **9** | **10** | **11** | **Delayed Dosing Visit** [22] | **Unscheduled Visit** [23] | **Withdrawal from Treatment Visit** |
| **Week** | **-2** | **-** | **w2** | **w12** | **w24** | **w36** | **w48** | **w60** | **w72** | **w84** | **w96** | | | |
| *Study Day (window in days)* | *-14* | *1* | *15 (± 1)* | *85 (±4)* | *169 (±1)* | *253 (±4)* | *337 (±1)* | *421 (±4)* | *505 (±1)* | *589 (±4)* | *673 (±1)* | | | |
| Pregnancy test [6] | x | x | x | x | x | x | x | x | x | x | x | x | | x |
| Antibody Titers [7] | | x | | x | x | | x | | x | | x | | | x |
| RCR (non-DNA) [8] | | x | | x | x | | x | | x | | x | | | x |
| RCR (DNA) [9] | | x | | | | | | | | | | | | |
| Protein biomarker sampling [10] | | x | | x | x | | x | | x | | x | | | x |
| HAHA [11] | | x | | | x | | x | | x | | x | | | x |
| Plasma/ urine banking for JCV[12] | | x | | x | x | x | x | x | x | x | x | | | x |
| PK Samples [13] | | x | | | x | | x | | x[13] | x | x | | | x |
| Thyroid function tests [14] | x | | | | x | | x | | x | | x | | | x |
| FSH [15] | x | | | | | | | | | | | | | |
| Hepatitis Screening [16] | x | | | | | | | | | | | | | |
| Hepatitis B virus DNA[16] | x | (x) | | (x) | (x) | (x) | (x) | (x) | (x) | (x) | (x) | | | (x) |
| RPR | x | | | | | | | | | | | | | |
| CD4 count | x | | | x | | x | | x | | x | | | | |
| IgG | | | | x | | x | | x | | x | | | | |
| Total Ig, IgA, IgG, IgM | x | | | | x | | x | | x | | x | | | x |
| FACS [17] | | x | x | x | x | | x | | x | | x | | | x |
| Routine safety lab [18] | x | x | x | x | x | x | x | x | x | x | x | | | x |
| Type I interferon neutralizing antibody assay [19] | | x | | | x | | x | | x | | x | | | x |

## Table 3: Schedule of Assessments: Screening Through the End of Double-Blind Treatment Period (Cont.)

| | Screen | Treatment Period | | | | | | | | | | | |
|---|---|---|---|---|---|---|---|---|---|---|---|---|---|
| **Visit** | 1 | 2 BL | 3 | 4 | 5 | 6 | 7 | 8 | 9 | 10 | 11 | Delayed Dosing Visit [22] | Unscheduled Visit [23] | Withdrawal from Treatment Visit |
| **Week** | -2 | - | w2 | w12 | w24 | w36 | w48 | w60 | w72 | w84 | w96 | | | |
| *Study Day (window in days)* | -14 | 1 | 15 (± 1) | 85 (±4) | 169 (±1) | 253 (±4) | 337 (±1) | 421 (±4) | 505 (±1) | 589 (±4) | 673 (±1) | | | |
| Pre-treatment with i.v. methylprednisolone [20] | | x | x | | x | | x | | x | | | x | | |
| Administration of i.v. ocrelizumab / ocrelizumab placebo [21] | | x | x | | x | | x | | x | | | x | | |
| Assessment of s.c. Rebif®/ Rebif® placebo compliance | | x | x | x | x | x | x | x | x | x | x | | x | x |
| Administration of s.c. Rebif® / Rebif® placebo 3x/wk | | x | x | x | x | x | x | x | x | x | | | | |

1. **Informed Consent** must be obtained in written form from all patients at screening (prior to any study–related procedure) in order to meet eligibility for the study.
2. **Vital signs** (i.e., pulse rate, systolic and diastolic blood pressure, respiration rate and temperature) will be obtained while the patient is in the semi supine position (after 5 minutes). On infusion visits, the vital signs should be taken within 45 minutes prior to the methylprednisolone infusion in all patients. In addition, vital signs should be obtained prior to ocrelizumab/ocrelizumab placebo infusion, then every 15 minutes (± 5 minutes) for the first hour; then every 30 minutes (± 10 minutes) until 1 hour after the end of the infusion. On non-infusion days, the vital signs may be taken at any time during the visit.
3. **ECG (pre- and post-dose):** on infusion visits ECG should be taken within 45 minutes prior to the methylprednisolone infusion in all patients, and within 60 minutes after completion of the ocrelizumab/ocrelizumab placebo infusion. On non-infusion days, the ECG may be taken at any time during the visit.
4. **MRI**: brain MRI scans will be obtained in patients withdrawn from the treatment period (at a withdrawal visit) if not performed during last 4 weeks.

## Table 3: Schedule of Assessments: Screening Through the End of Double-Blind Treatment Period (Cont.)

5. **A structured telephone interview** will be conducted by site personnel every 4 weeks (± 3 days) from Week 8 through the study to identify any new or worsening neurological symptoms that warrant an unscheduled visit and collect data on possible events of infections.
6. **Serum β-hCG** must be performed at screening in women of childbearing potential. Subsequently, urine β-hCG [sensitivity of at least 25 mIU/mL] will be performed. On infusion visits, the urine pregnancy test should be performed prior to methylprednisolone infusion in all women of child-bearing potential. If positive, the patient will not receive the scheduled dose and confirmation, a serum pregnancy test, will be performed.
7. **Antibody Titers**: measurement of antibody titers against common antigens (mumps, rubella, varicella and Streptococcus pneumoniae) will be performed.
8. **RCR - Roche Clinical Repository non-DNA (RNA – and protein):** for RNA 2x 2.5 ml whole blood samples to be taken from consenting patients only for expression profiling analysis. For protein: 6 ml blood samples in EDTA tube for plasma samples will be taken from consenting patients only for analysis of protein biomarkers. On infusion visits, ALL samples should be taken 5-30 minutes prior to methylprednisolone infusion.
9. **RCR - Roche Clinical Repository (DNA):** 6 ml whole blood sample to be taken from only from patients consenting to RCR for pharmacogenetic and genetic analysis. If not done at Baseline (Visit 2), sample may be collected at next visit.
10. **Protein biomarker sampling:** one serum sample (6 ml) will be taken from all patients for analysis of protein biomarkers. On infusion visits, samples should be taken 5-30 minutes prior to methylprednisolone infusion.
11. **HAHA**: On infusion visits, serum samples are collected 5-30 minutes prior to the methylprednisolone infusion.
12. **Plasma and urine samples for JCV will be collected** at specified time points and analyzed in batches.
13. **PK samples:** on the infusion day at week 72, two serum samples should be collected, one 5-30 minutes prior to the methylprednisolone infusion and the second one 30 minutes (±10 minutes) following the completion of the ocrelizumab/ocrelizumab placebo infusion. For all other infusion visits, a blood sample should be taken 5 – 30 minutes before the methylprednisolone infusion. At other times (non-infusion visits) samples may be taken at any time during the visit.
14. **sTSH** will be tested at screening and during double-blind treatment period. Thyroid autoantibodies will be assayed only at screening.
15. **FSH**: only applicable to women to confirm the post-menopausal status.
16. **Hepatitis** screening & monitoring: all patients must have negative HBsAg result and negative HepCAb screening tests prior to enrollment. If total HBcAb is positive at screening, HB virus DNA measured by PCR must be negative to be eligible. For those patients enrolled with negative HBsAg and positive total HBcAb, HB virus DNA (PCR) must be repeated every 12 weeks during the treatment period.

## Table 3: Schedule of Assessments: Screening Through the End of Double-Blind Treatment Period (Cont.)

17. **FACS**: including CD19 and other circulating B-cell subsets, T-cells, natural killer cells and other leukocytes. On infusion visits, blood samples should be collected prior to the infusion of methylprednisolone.
18. **Routine safety lab:** hematology, chemistry and urinalysis: on infusion visits, all urine and blood samples should be collected prior to the infusion of methylprednisolone. At other times, samples may be taken at any time during the visit.
19. **Type I interferon neutralizing antibody assay:** samples should be taken at least 36 hours following last injection of Rebif®/Rebif® placebo.
20. All patients receive **prophylactic treatment** with 100 mg of methylprednisolone i.v. prior to infusion of ocrelizumab /ocrelizumab placebo. It is also recommended that patients receive an analgesic/antipyretic such as acetaminophen/paracetamol (1 g) and an i.v. or oral antihistaminic such as diphenhydramine 50 mg 30-60 minutes prior to ocrelizumab/ ocrelizumab placebo.
21. **Administration (infusion) of i.v. ocrelizumab/ocrelizumab placebo**: the Treating Investigator must review the clinical and laboratory re-treatment criteria prior to subsequent infusion of ocrelizumab/ocrelizumab placebo.
22. **A delayed dosing visit** will be performed and recorded in the Delayed Dosing Visit eCRF form when dosing cannot be administered at the scheduled dosing visit. Other tests or assessments may be done as appropriate.
23. **Unscheduled Visit**: assessments performed at unscheduled (non-dosing) visits will depend on the clinical needs of the patient. All patients with new neurological symptoms suggestive of relapse should have EDSS performed by examining investigator. Other tests/assessments may be done as appropriate. Please note: in case of ALT elevations dose modification should be necessary, additional visits may be required for dispensing of study medication.

Please note: based on local Ethics Committees or National Competent Authority requirements, additional diagnostic testing may be required for selected patients or selected centers to exclude tuberculosis, Lyme disease, HTLV-1 associated myelopathy (HAM), acquired immune deficiency syndrome (AIDS), hereditary disorders, connective tissue disorders, or sarcoidosis. Other specific diagnostic tests may be requested when deemed necessary by the investigator**.**

**Table 4: Schedule of Assessments: Safety Follow up (including prolonged B-cell monitoring if required)**

| | **Safety Follow up** | ***Prolonged B-cell Monitoring*[1]** | ***End of observation or withdrawal from Safety Follow up*** |
|---|---|---|---|
| **Assessments** | *Visits every 12 weeks (±7 days)* [2] | *Visits every 24 weeks (±7 days)* | |
| Urine pregnancy test | x | x | x |
| Routine Safety Labs[3] | x | x | x |
| FACS [4] | x | x | x |
| Total Ig, IgA, IgG, IgM | x[10] | x | x |
| HAHA[5] | x[10] | x | x |
| Plasma/urine banking for JCV | x | x | x |
| Antibody titers | x[10] | x | x |
| Hepatitis B viral DNA[6] | (x) | (x) | (x) |
| RCR non-DNA [7] | x[10] | x | x |
| Protein biomarker sampling [8] | x[10] | x | x |
| Vital Signs | x | x | x |
| EDSS | x | | x |
| Neurological examination | x | x | x |
| Physical examination | x[10] | x | x |
| Potential relapses recorded | x | x | x |
| Adverse events | x | x | x |
| Concomitant Medication | x | x | x |
| Telephone interview [9] | x | x | |

## Table 4: Schedule of Assessments: Safety Follow up (including prolonged B-cell monitoring if required) (Cont.)

1. **Prolonged B-cell monitoring:** patients whose B-cells have not been repleted after 48 weeks of Safety Follow up period will continue with visits every 24 weeks (± 7 days) until B-cell repletion.
2. Visits will be performed at 12-week intervals counting from the date of last infusion of ocrelizumab.
3. **Routine safety lab**: hematology, chemistry and urinalysis.
4. **FACS** including CD19 and other circulating B-cell subsets, T cells, natural killer cells and other leukocytes.
5. **HAHA**: two serum samples are required.
6. **Hepatitis monitoring:** hepatitis to be monitored only in patients with screening results of HbsAg negative, HBcAb positive and HBV DNA negative, inclusive.
7. **RCR (Roche Clinical Repository) non-DNA (RNA and protein):** for RNA 2x 2.5 ml whole blood samples to be taken from consenting patients only for expression profiling analysis. For protein 6 ml blood samples in EDTA tube for plasma samples to be taken from consenting patients only for analysis of protein biomarkers.
8. **Protein biomarker sampling**: 6 ml blood sample in a plain tube without EDTA for serum isolation will be taken from all patients for analysis of protein biomarkers.
9. **A structured telephone interview** will be performed by site personnel every 4 weeks (± 3 days) between visits until 48 weeks after the last infusion to identify any new or worsening neurological symptoms that warrant an unscheduled visit and collect data on possible events of infections. If prolonged B-cell monitoring is required beyond 48 weeks after the last infusion, telephone interviews will be done every 12 weeks (± 7 days) between visits.
10. Needs to be assessed only every 24 weeks.

<u>Please note:</u> patients in Safety Follow up who receive other B-cell targeted therapies will only be followed for 48 weeks from the date of the last infusion of the study drug regardless of their B-cell count.

## 5.1 Screening Examination and Eligibility Screening Form

All patients must sign and date the most current Institutional Review Board/Institutional Ethics Committee's (IRB/IEC) approved written informed consent before any study specific assessments or procedures are performed.

Consenting patients will enter the 2-week screening period to be evaluated for eligibility. Please refer to "Schedule of Assessments: Screening through the End of Double-Blind Treatment Period" - Table 3 for details. Patient must fulfill all entry criteria for participation in the study.

*Please note that based on local Ethics Committees or National Competent Authority requirements, additional diagnostic testing may be required for selected patients or selected centers to exclude tuberculosis, Lyme disease, HTLV-1 associated myelopathy (HAM), acquired immune deficiency syndrome (AIDS), hereditary disorders, connective tissue disorders, or sarcoidosis.*

An Eligibility Screening Form [ESF] documenting the investigator's assessment of each screened patient with regard to the protocol's inclusion and exclusion criteria is to be completed by the investigator.

Each patient screened must be registered in the IxRS by the investigator or the investigator's research staff at screening. A screen failure record must be maintained by the investigator, and reasons must be captured in the IxRS.

It should be stated in the medical record that the patient is participating in this clinical study.

## 5.2 Procedures for Enrollment of Eligible Subjects

Once a patient has fulfilled all eligibility criteria, he or she will be randomized via IxRS to one of two treatment groups: ocrelizumab 600 mg (given as 300 mg x 2 14 days apart for the first 24 weeks and 600 mg x 1 every 24 weeks thereafter) or Rebif®.

Patient eligibility information will be provided to the IxRS by the investigator or the investigator's research staff at randomization. The patient will be randomized and assigned a unique treatment box number (medication number) and randomization number. As confirmation, the site will be provided with a verification of each patient's randomization.

The patient randomization numbers will be generated by Roche or its designee and incorporated into the double-blind labeling.

The patient randomization numbers are to be allocated sequentially in the order in which the patients are enrolled according to the specification document agreed with the external randomization company/center.

Treatment with the first study drug infusion should occur within 24 hours of randomization. In exceptional cases where all baseline assessments cannot be completed within 24 hours, the first study drug infusion can be administered within 48 hours of randomization provided that the investigator assures that all inclusion and exclusion

criteria are still met on the day of dosing. In particular, there should be no evidence of an ongoing infection at the time of dosing.

No patient may begin treatment prior to randomization and assignment of a medication number.

## 5.3 Clinical Assessments and Procedures

This is an assessor blinded study. Each site will have two investigators: a principal or Treating Investigator and an Examining Investigator or rater.

- **The Treating Investigator** is the physician responsible for the patient care and should be a neurologist experienced in the care of MS patients. The Treating Investigator will have access to safety and blinded efficacy data and will make treatment decisions based on the patient's clinical response and laboratory findings.
- **The Examining Investigator** should be a neurologist or other health care practitioner and must be trained and certified in administering the *Neurostatus* Functional System Scores (FSS) and Expanded Disability Status Scale (EDSS) examination prior to study start.

The Examining Investigator will perform the neurological examination, document the FSS scores, and assess EDSS scores. The examining investigator will also be responsible for performing and documenting results from the following: MSFCS, the Karnofsky Performance Status Scale, low-contrast visual acuity testing, and the Symbol Digit Modalities Test. They will only have access to data from the assessments listed above. Every effort will be made to ensure that there is no change in the EDSS rater throughout the course of the study for any individual patient. Whenever possible, the same person should perform the examination for the full study duration.

All efforts should be made to keep the Examining Investigator blinded to the treatment assignment. Patients will be instructed not to discuss any symptoms related to the study treatment with the Examining Investigator; the Examining Investigator should remind the patient at the start of the examination. In view of the extended duration of this study, each site will identify a primary and back-up for Treating and Examining Investigator. **The Treating Investigator and the Examining Investigator will not be allowed to switch roles**.

### 5.3.1 Overview of Clinical Visits

After the screening visit, patients fulfilling the entry criteria will be scheduled for the baseline assessments. Randomization will occur only after the patient meets all inclusion and exclusion criteria on Day 1. Visits will take place as described in the Schedule of Assessments.

Visits should be scheduled in relation to the baseline visit (Day 1) A minimum of 22 weeks should occur between infusions. Patients should not receive their infusions within a shorter interval. Patients who cannot receive their infusion at the visit, should be re-scheduled for a delayed dosing visit – see Section 5.3.1.1.

At infusion visits patients treated with ocrelizumab should remain in observation for at least 1 hour after the completion of the infusion.

Patients who cannot receive their infusion at the visit, should be re-scheduled for a delayed dosing visit. Additional unscheduled visits for the assessment of potential relapses, new neurological symptoms, safety events or for dispensing Rebif®/Rebif® placebo if down titration is needed may occur at any time.

#### *5.3.1.1 Delayed Dosing Visit*

Delayed dosing visits may be scheduled only if the infusion cannot be administered at the time points defined in Schedule of Assessments - Table 3. Thus, a patient who had all assessments of a dosing visit performed, but could not receive his/her infusion, should be re-scheduled for the infusion.

**If the delayed infusion is the first infusion of the first treatment cycle (Day 1), then the visit for the second infusion should be scheduled 14 days after the delayed first infusion (± 1 day). In the event any subsequent infusion needs to be delayed, at least a 22-week period still must be maintained between one infusion and the next.**

At the delayed dosing visit, additional tests or assessments, such as routine safety laboratory tests, may be performed when the investigator judges that these are warranted.

#### *5.3.1.2 Unscheduled Visits*

Patients developing new or worsening neurological symptoms should be seen at the investigational site as soon as possible regardless of the treatment group to which they were randomized, regardless of the dates of their pre-planned, scheduled study visits, and regardless of the study period. Assessments performed at unscheduled (non-dosing) visits will depend on the clinical needs of the patient.

Patients with new neurological symptoms suggestive of relapse should have an EDSS performed by Examining Investigator. Other tests/assessments may be done as appropriate. Please note: should the Rebif®/Rebif® placebo dose modification be necessary in case of ALT elevations, unscheduled visits may be required for dispensing of study medication.

Please refer also to Section 7.3.4.1 for guidance on the diagnosis of progressive multifocal leukoencephalopathy (PML).

#### *5.3.1.3 Withdrawal Visits*

At the moment a patient meets one or more of the withdrawal criteria (Section 4.5), this patient is regarded withdrawn from treatment. Patient who withdraw from ocrelizumab treatment will need to complete all assessments as shown in Schedule of Assessments and will enter the Safety Follow-up.

Please note: at the Withdrawal from Double-blind Treatment Period Visit, an MRI scan will be required only if not performed in the prior 4 weeks.

### 5.3.2 Assessment of Efficacy

#### *5.3.2.1 Assessment of Relapse*

All new or worsening neurological events consistent with MS representing a clinical relapse are to be reported on the dedicated page of eCRF. Patients with clinical relapses should be referred to the Examining Investigator who will assess the FSS/EDSS independently to allow confirmation as to whether or not the clinical relapse(s) meet the criteria for protocol-defined relapse(s).

**Protocol-defined relapse** is the occurrence of new or worsening neurological symptoms attributable to MS. Symptoms must persist for >24 hours and should not be attributable to confounding clinical factors (e.g. fever, infection, injury, adverse reactions to medications) and immediately preceded by a stable or improving neurological state for at least 30 days. The new or worsening neurological symptoms must be accompanied by objective neurological worsening consistent with an increase of at least half a step on the EDSS scale, or 2 points on one of the appropriate FSS, or 1 point on two or more of the appropriate FSS. The change must affect the selected FSS (i.e., pyramidal, ambulation, cerebellar, brainstem, sensory, or visual). Episodic spasms, sexual dysfunction, fatigue, mood change, or bladder or bowel urgency or incontinence will not suffice to establish a relapse. NB: Sexual dysfunction and Fatigue will not be scored. Please note: adjudication of protocol-defined relapses will be performed by the Sponsor based on pre-specified criteria, applied to data collected by investigator, in a blinded fashion.

**All patients with new neurological symptoms suggestive of a relapse should be referred to the Examining Investigator for EDSS assessment. Any patient, complaining of a neurological symptom, defined at a visit or over the phone, should be referred to the Examining Investigator unless the Treating Investigator determines that the symptom is due to mitigating circumstances (such as an intensification of neurological symptoms from a transient systemic infection).**

Please note: clinical relapses (i.e., regardless of whether they meet criteria for a protocol-defined relapse) will be recorded on a pre-specified eCRF “MS relapse” eform. MS relapses should not be reported on Adverse Event eform of eCRF.

#### *5.3.2.2 Assessment of Disability*

**Disability progression** has been defined as an increase of ≥ 1.0 point from the baseline EDSS score that is not attributable to another etiology (e.g. fever, concurrent illness, or concomitant medication) when the baseline score is 5.5 or less, and ≥ 0.5 when the baseline score is above 5.5. Disability progression is considered sustained when the increase in the EDSS is confirmed at a regularly scheduled visit at least 12 weeks or 24 weeks, after the initial documentation of neurological worsening.

Sustained disability progression, confirmed for both 12 and 24 weeks, after the initial documentation of neurological worsening, will be analyzed as key secondary endpoints.

#### *5.3.2.3 Kurtzke Expanded Disability Status Scale (EDSS)*

The EDSS is based on a standard neurological examination, incorporating the following functional systems (pyramidal, cerebellar, brainstem, sensory, bowel and bladder, visual,

and cerebral [or mental]) and ambulation rated and scored as functional system scores (FSS). Each FSS is an ordinal clinical rating scale ranging from 0 to 5 or 6. These ratings are then used in conjunction with observations and information concerning ambulation and use of assistive devices to determine the EDSS score. The EDSS is a disability scale that ranges in 0.5-point steps from 0 (normal) to 10 (death) [48].

The EDSS will be assessed by the Examining Investigator. **All patients with new neurological symptoms suggestive of relapse should have EDSS performed during an unscheduled visit.**

#### *5.3.2.4 The Multiple Sclerosis Functional Composite Scale (MSFCS)*

The Multiple Sclerosis Functional Composite Scale (MSFCS) consists of three subscales, including the 9-Hole Peg Test, Paced Auditory Serial Addition Test (PASAT), and Timed 25-Foot Walk (25-TW), which provide a global quantitative estimate of MS disease progression [49].

The MFSCS will be performed by the Examining Investigator who must remain blinded to the treatment assignment.

#### *5.3.2.5 Low-Contrast Visual Acuity (LCVA) Testing*

Low-contrast letter acuity charts (Sloan charts) have gained validity in the assessment of visual dysfunction in patients with MS not readily apparent on commonly used high-contrast acuity tests. Reductions in low-contrast letter acuity are associated with MS and correlate with increasing disability, MRI abnormalities, and reduced retinal nerve fiber layer (RNFL) thickness as measured by optical coherence tomography (OCT).

LCVA testing will be performed using low contrast letter acuity charts (low contrast Sloan letter charts) by the examining investigator at the timepoints indicated in the Schedule of Assessments - Table 3.

#### *5.3.2.6 The Symbol Digit Modalities Test (SDMT)*

The SDMT has demonstrated sensitivity in detecting not only the presence of cognitive impairment, but also changes in cognitive functioning over time and in response to treatment. The SDMT is brief, easy to administer, and involves a simple substitution task that normal children and adults can easily perform. Using a reference key, the examinee has 90 seconds to pair specific numbers with given geometric figures. Responses can be written or oral, and for either response mode, administration time is just 5 minutes.

SDMT will be administered by the examining investigator at the timepoints indicated in the Schedule of Assessments - Table 3.

### 5.3.3 Brain MRI Imaging

Magnetic resonance imaging (MRI) is a useful tool for monitoring CNS lesions in MS. Different MRI derived parameters have been related to clinical activity and T1 weighted gadolinium-enhancing lesions or new and/or enlarging hyperintense T2 lesions have been related to relapses. It is hypothesized that changes in brain volume may reflect brain atrophy as a result of MS-related tissue loss and may thereby correlate with long-term clinical outcome in these patients.

Brain MRI scans will be obtained in all patients as detailed in the Schedule of Assessments - Table 3. In addition, brain MRI scans will be obtained in patients withdrawn from the double-blind phase of treatment period (at the withdrawal visit) if not performed during the previous 4 weeks.

Scans will be performed by trained and certified MRI technicians. **The following time windows apply:**

- **"Baseline" MRI should be performed after screening visit, but at least 10 days prior to the baseline visit.**
- **MRI at visits scheduled at Weeks: 24, 48, 96 or at withdrawal visit (if applicable) should be performed within 4 weeks of the scheduled visit.**

If patients receive corticosteroids for a relapse, every effort should be made to obtain the scan prior to the first steroid dose if the pre-steroid scan is within 1 week of the scheduled visit. In patients receiving corticosteroids, there should be an interval of 3 weeks between the last dose of corticosteroids and the scan.

The MRI will include the acquisition of scans at each time point with and without intravenously administered gadolinium contrast enhancement.

MRI scans will be read by a centralized reading center for both efficacy and safety endpoints. The centralized reading center is blinded to the treatment assignment and the reading is performed in the absence of clinical information. Further details on scanning acquisition sequences, methods, handling and transmission of the scans, certification of site MRI radiologist/technicians, and the procedures for the blinded analysis of the scans at the central reading center are described in a separate MRI Acquisition Procedures Manual.

All MRI scans will also be reviewed locally by a radiologist for safety and the MRI scan report containing only non-MS pathology will be provided to the Treating Investigator (see Section 5.3 for definition). At the investigational site, only the local radiologist/technician assigned to this study may have access to the MRI scans; the Treating Investigator should not review the MRI scans unless a safety concern arises. In the event that the Treating Investigator does become aware of the MRI results, this should be documented in the eCRF, indicating the reason.

### 5.3.4 Safety

Adverse events, vital signs, weight, physical and neurological examination, clinical laboratory tests (including pregnancy tests), 12 lead ECG, locally reviewed MRI for safety (non MS CNS pathology), and data on concomitant medications and diseases will be collected throughout the study.

Please note: On the infusion days, the vital signs should be taken within 45 minutes prior to the methylprednisolone infusion in all patients. In addition, the vital signs should be obtained prior to the study drug infusion, then every 15 minutes (± 5 minutes) for the first hour; then every 30 minutes (± 10 minutes) until 1 hour after the end of the infusion. On non-infusion days, the vital signs may be taken at any time during the visit. Additional vital signs readings may be taken at the discretion of the investigator in the event of an

infusion related reaction or if clinically indicated and should be recorded on the unscheduled vital signs eCRF.

Please refer to relevant sections of protocol for more details.

#### 5.3.4.1 Electrocardiogram (ECG)

A 12-lead ECG should be taken at the visits indicated in the Schedule of Assessments - Table 3. Comments generated automatically by the ECG machine should not be recorded in the eCRF unless confirmed by a physician. An ECG is also required if the patient prematurely withdraws from the study.

#### 5.3.4.2 Physical Examination

The physical examination will be performed as per Schedule of Assessments. Diagnosis of new abnormalities or clinically significant worsening of pre-existing abnormalities should be recorded as adverse events if appropriate.

#### 5.3.4.3 Neurological Examination

A neurological examination will be performed at every planned visit and at unscheduled visit if applicable.

- In the presence of newly identified or worsening neurological symptoms, a neurological evaluation should be scheduled promptly. In case of events suggestive of relapse the Treating Investigator should request EDSS to be performed by the Examining Investigator.

Study investigators will screen patients for signs and symptoms of PML by evaluating neurological deficits localized to the cerebral cortex, such as cortical symptoms/signs, behavioral and neuropsychological alteration, retrochiasmal visual defects, hemiparesis, cerebellar symptoms/signs (e.g., gait abnormalities, limb incoordination). A brain MRI scan and CSF analysis may be warranted to assist in the diagnosis of PML. See Section 7.3.4.1 for guidance on the diagnosis of PML.

Patients with suspected PML, defined as a new or worsening neurological symptom which necessitates MRI and or lumbar puncture and CSF analyses to rule out PML, should be withheld from study treatment until PML is ruled out by complete clinical evaluation and appropriate diagnostic testing (see Section 7.3.4.1). The Sponsor's Medical Responsible and Medical Monitor should be contacted by email. In addition Sponsor medical responsible person should be immediately contacted by phone.

**A patient with confirmed PML should be withdrawn from the treatment.** PML should be reported as an SAE (with all available information) with immediate notification of the Medical Monitor (see also Section 7.1.1.3).

#### 5.3.4.4 Telephone Interviews

The purpose of this semi-structured interview is to identify new or worsening neurological symptoms that warrant an unscheduled visit and collect information on possible events of infections. The telephone interview will be conducted by site personnel familiar with the patient(s) every 4 weeks (± 3 days) between the study visits during the study treatment and Safety Follow up period starting from Week 8, until 48 weeks after

the last infusion. Thereafter, for those patients who require prolonged B-cell monitoring, telephone interviews will continue every 12 weeks (± 7 days) between regular visits.

The site will record in the eCRF the telephone interview as "Done" or "Not Done" and documentation of the interview will be maintained in the patient's study file.

Please refer to Appendix 4 for detailed information.

#### 5.3.4.5 Columbia-Suicide Severity Rating Scale C-SSRS

The Columbia-Suicide Severity Rating Scale (C-SSRS) will be used for prospective suicidality assessment. C-SSRS is a tool used to assess the lifetime suicidality of a patient and to track suicidal events through the treatment. The structured interview prompts recollection of suicidal ideation, including the intensity of the ideation, behavior and attempts with actual/potential lethality.

The scale will be administered by the Treating Physician at the timepoints indicated in the Schedule of Assessments. The C-SSRS "*baseline*" will be collected at baseline and the C-SSRS "*since last visit*" will be collected at subsequent visits.

Please note: assessing the risk of suicide is a difficult and complex task when applied to the individual patient. Certainly, no single clinical scale can replace a thorough medical examination and suicide risk assessment. Ultimately, the determination of the presence of suicidality depends on clinical judgment.

### 5.3.5 The Karnofsky Performance Scale (clinician-reported version)

The Karnofsky Performance Scale score allows patients to be classified as to their functional impairment. This scale is usually used to compare effectiveness of different therapies and to assess the prognosis in individual patients. The lower the Karnofsky score, the worse the survival for most serious illnesses.

The scale will be administered by Examining Investigator at the time points indicated in the Schedule of Assessments - Table 3.

## 5.4 Laboratory Assessments

Roche Clinical Repository biomarker samples will be shipped directly to Roche Clinical Sample Operations unit. All other lab samples collected during the study will be shipped to Central Laboratory.

The procedures for the collection, handling and shipping of laboratory samples are specified in the Laboratory Manual.

The samples for this study should be classified, packed and shipped as UN3373 Biological Substance, Category B.

Full details of the central laboratory sample handling, shipment and reporting of results will be described in the Laboratory Manual.

During the double-blind treatment period of the study, the total volume of blood loss for laboratory assessments will be approximately 345 mL over 2 years. The amount of blood taken at each visit will vary, but will be no more than 56 mL. In the safety follow-up

period, the amount of blood taken at each visit will be no more than 41 mL. Patients consenting for RCR project may have additional blood samples taken – please refer to Section 5.5.1 for more details.

### 5.4.1 Standard Laboratory Assessments

Please note: Some laboratory parameters that could reveal patient's allocation to study treatment, such as FACS cell counts, absolute neutrophil counts, Ig levels, and type I interferon neutralizing antibody levels, will be blinded. In order to ensure patients' safety in the study and to allow for assessments of the re-treatment criteria, a central laboratory will provide study investigators and Medical Monitors with reflex messages triggered by critical blinded laboratory results. Investigators notified of their patient's critical laboratory test results will be instructed to suspend further treatment with study drug until the patient becomes eligible for re-treatment. The reflex messages from a central laboratory, together with non-blinded laboratory results, should be carefully reviewed at every visit before continuing with study treatment. Further details will be provided in Laboratory Manual.

**Hematology:** Hemoglobin, hematocrit, red blood cells (RBC), white blood cells (WBC) (absolute and differential), absolute neutrophil count, and quantitative platelet count.

**Blood chemistry:** AST/SGOT, ALT/SGPT, GGT, alkaline phosphatase, amylase, lipase, total protein, albumin, cholesterol, total bilirubin, urea, uric acid, creatinine, random glucose, potassium, sodium, calcium, phosphorus, lactic dehydrogenase, creatine phosphokinase, and triglycerides.

**Thyroid function test:** sTSH will be tested at screening, and yearly during the Double-blind Treatment Period. Thyroid autoantibodies will be assayed only at screening.

**FACS** will include (but is not limited to) the following cells:

- Total B cells ($CD19^{pos}$)
- Total T cell ($CD3^{pos}$)
- T helper cells ($CD3^{pos}$, $CD4^{pos}$)
- $T_{CTL}$ ($CD3^{pos}$, $CD8^{pos}$)
- NK Cells ($CD3^{neg}$, $CD16/56^{pos}$)
- B-cell subsets:
  - memory B-cells ($CD19^{pos}$, $CD27^{pos}$, $CD38^{neg}$)
  - naïve B-cells ($CD19^{pos}$, $CD27^{neg}$, $IgD^{pos}$)
  - plasmablasts ($CD19^{lo}$, $CD27^{pos}$, $CD38^{hi}$,)

**Quantitative Immunoglobulin:** Ig levels (including Total Ig, IgG, IgM, and IgA isotypes).

**Antibody titers:** Measurement of antibody titers to common antigens (mumps, rubella, varicella, S. pneumoniae) will be performed. This information is used to assess the effect of ocrelizumab on specific humoral immunity to bacterial and viral antigens.

**HAHA:** Serum samples will be collected for determination of antibodies against ocrelizumab (HAHA). Since ocrelizumab concentrations affect the HAHA assay, the concentration of ocrelizumab will be measured as well at all timepoints with HAHA assessment to enable interpretation of the results (PK sample). For details please refer to Schedule of Assessments.

**Pregnancy Test**: All women of childbearing potential must have regular pregnancy tests. At screening, a serum pregnancy test will be performed in central laboratory. During the study treatment period and Safety Follow up, a urine pregnancy test (sensitivity of at least 25 mIU/mL β-hCG) will be performed locally at the time points shown in Schedule of Assessments. On infusion visits, the urine pregnancy test should be performed prior to the methylprednisolone infusion. A positive urine pregnancy test should be confirmed with a serum test through the central laboratory prior to any further dosing with ocrelizumab.

Please note: additional laboratory tests will be performed at screening in order to verify eligibility criteria. Please refer to Table 3 for further details.

### 5.4.2 Hepatitis Screening and Liver Function Monitoring

Patients with a history or known presence of recurrent or chronic hepatitis B or C infection must be excluded from enrollment into the study (see Section 4.3). In addition, hepatitis B and C serology will be performed at screening. A positive result to either hepatitis surface antigen (HBsAg), or hepatitis B core antibody (total HBcAb) associated with positive viral DNA titres as measured by PCR, or a positive result for hepatitis C antibody (HepCAb) should result in the patient's exclusion. Patients with evidence of past resolved hepatitis B infection (i.e. positive total hepatitis B core antibody associated with a negative viral DNA) can be enrolled, and will have the hepatitis B viral DNA checked every 12 weeks as per Schedule of Assessment. Patients in whom the viral DNA becomes positive but in whom the quantity is at the lower limit of detection of the assay should have the test repeated as soon as possible. These patients may be referred to a hepatologist and treated, as clinically indicated. Patients found to have a viral DNA positive test with a copy number **exceeding $10^4$ copies/mL** should be referred to a hepatologist for assessment immediately. Patients in whom viral copy numbers increase beyond **$10^4$ copies/mL** during the cycle of the study will not receive further infusions of ocrelizumab and will enter the Safety Follow up Period.

Liver function, i.e. ALT/SGPT, AST/SGOT, gamma glutamyl transferase (GGT), alkaline phosphatase, total bilirubin, should be reviewed throughout the study. Patients developing evidence of liver dysfunction should be assessed for viral hepatitis and, if necessary, referred to a hepatologist or other appropriately qualified expert. Study drug should be withheld until the diagnosis of viral hepatitis has been excluded. Patients developing hepatitis B or C should be withdrawn from the study and should enter the Safety Follow up period. Should treatment be prescribed, this will be recorded in the

eCRF. Patients with viral hepatitis due to other agents, such as hepatitis A, may resume treatment after the patient's recovery.

Please refer also to Section 6.2.2 for further guidelines on liver function monitoring.

### 5.4.3 Plasma and Urine Banking for JC Virus

Long-term storage of plasma samples and urine is planned for JC virus DNA and/or other relevant tests for JC virus. Plasma samples (5 mL) and urine samples (10 ml) will be collected as per Schedule of Assessments. As the assay of the DNA virus has not been standardized, and a correlation between viremia and onset of PML has not been established, the JC virus assessments in plasma and urine will be performed if deemed necessary in the future and not on an ongoing basis.

### 5.4.4 Pharmacokinetic (PK)/Pharmacodynamic (PD) Assessments

Blood samples will be collected to evaluate the pharmacokinetics and pharmacodynamics of ocrelizumab as described in the Schedule of Assessments. The blood volume collected for pharmacokinetic assessments will be approximately 2 mL per sample. These samples will be assayed for ocrelizumab concentration using an enzyme-linked immunosorbent assay (ELISA).

Serum samples for determination of ocrelizumab concentrations will be collected at the time points detailed in the Schedule of Assessments. On the infusion visit at week 72, two serum samples should be collected, one 5-30 minutes prior to the methylprednisolone infusion and the second one 30 minutes (±10 minutes) following the completion of the ocrelizumab infusion. For all other infusion visits, a blood sample should be taken 5 - 30 minutes before the methylprednisolone infusion. At other times (non-infusion visits), samples may be taken at any time during the visit.

For sampling procedures, storage conditions, and shipment instructions, see the Sample Handling and Logistics Manual, which will be provided to each site.

### 5.4.5 Type I Interferon Neutralizing Antibody Assay

Type I interferon neutralizing antibody assay will be performed during double-blind phase of treatment period - please see Table 3 - Schedule of Assessments.

## 5.5 Roche Clinical Repository Specimen(s)

Please note: the Roche Clinical Repository research is contingent on review and approval for the exploratory biomarker assessments by an appropriate regulatory body (depending on the country where the study is performed) and a site's Institutional Review Board/ Ethics Committee. Written patient's informed consent to RCR project is also required. If a regulatory body or site's Institutional Review Board / Ethics Committee does not approve the extended analysis and long term storage of the biomarker samples, this section of protocol will not be applicable.

Specimens for dynamic (non-inherited) biomarker discovery and validation will be collected only from patients consenting to RCR.

These specimens will be used for research purposes to identify dynamic biomarkers that are predictive of response to ocrelizumab treatment (in terms of dose, safety and

tolerability) and will help to better understand the pathogenesis, course and outcome of multiple sclerosis and related diseases. Specimens for dynamic biomarker discovery will be single coded like any other clinical sample (labeled and tracked using the patient's study identification number (see Section 17).

The results of specimen analysis from the RCR will facilitate the rational design of new pharmaceutical agents and the development of diagnostic tests, which may allow for individualized drug therapy for patients in the future.

All RCR specimens will be destroyed no later than 15 years after the final freeze of the respective clinical database unless regulatory authorities require that specimens be maintained for a longer period. The specimens in the RCR will be made available for future biomarker research towards further understanding of MS treatment with ocrelizumab, related diseases and adverse events and for the development of potential associated diagnostic assays. The implementation and use of the RCR specimens is governed by the Roche Clinical Repository policy to ensure the appropriate use of the RCR specimens.

### 5.5.1 Specimen Types

Exploratory Biomarkers (non-DNA):

- **Plasma assays**

Blood (one, approximately 6 mL sample in EDTA) for plasma isolation will be obtained at various time points as shown in Schedule of Assessments. These samples will be used for biomarker assays which may include chemokines and other candidate biomarkers in multiple sclerosis. For sampling procedures, storage conditions and shipment instructions see study Sample Handling and Logistics Manual.

- **Blood for RNA expression profiling**

Blood (2 x approximately 2.5 mL collected in PAXgene vacutainers) for RNA isolation will be obtained at various time points as shown in Schedule of Assessments. The samples may be tested using techniques such as a micro array profiling system and/or RT PCR to study the expression profile of genes known to be involved with multiple sclerosis, and any other differentially expressed genes relative to treatment response or re-treatment. For sampling procedures, storage conditions and shipment instructions see study Sample Handling and Logistics Manual.

Exploratory Biomarkers (DNA): **one sample of 6 mL of blood** will be taken as per Schedule of Assessments. 6 ml whole blood sample to be taken only from patients consenting to RCR for pharmacogenetic and genetic analysis. If not done at Baseline (Visit 2), sample may be collected at next visit.

For all samples, dates of specimen collection should be recorded on the associated RCR page of the eCRF and/or in the clinical database.

## 5.6 Protein Biomarker Samples

Specimens for protein biomarker discovery and validation will be collected from all patients. These specimens will be used for research purposes to identify and/or verify

protein biomarkers that are predictive of response to ocrelizumab treatment (in terms of dose, safety and tolerability) and will help to understand the pathogenesis, course and outcome of relapsing MS and related diseases. Identification of patient subgroups with increased response to therapy or increased progression rates would provide information of significant clinical value to guide treatment decisions and aid in the appropriate use of the therapy. Analyses will include but are not limited to the B-cell activating factor (BAFF) and Complement Factor H (CFH) [50].

A 6 ml sample of whole blood will be collected in a plain tube without EDTA for serum isolation. For sampling procedures, storage conditions and shipment instructions see study Sample Handling and Logistics Manual, which will be provided to each study site.

Blood specimens for protein biomarker discovery and validation will be collected from all patients as per Schedule of Assessments. These specimens will be stored for 5 years after the end of the study and then destroyed.

## 5.7 Patient Reported Outcome(s)

PRO data will be collected at the study visit with an electronic tablet device. The tablet with the PRO instruments will be distributed by the investigator staff and completed in their entirely by the patient.

Please note: all PROs are required to be administered prior to administration of study drug and prior to any other study assessment(s) to ensure the validity of the instruments is not compromised, and data quality meet requirements of regulatory authorities [51] and best practices [52, 53].

PRO data will be elicited from patients in this study to better characterize the clinical profile of ocrelizumab. These PRO measurements are described in Sections 5.71, 5.7.2, and 5.7.3. Please note that the methods for collecting and analyzing PRO data are different from those for the ascertainment of observed or volunteered adverse events. Due to these differences, PRO data will not be reported as adverse events and no attempt will be made to resolve any noticeable discrepancies between PRO data and observed or volunteered adverse events.

### 5.7.1 Modified Fatigue Impact Scale (MFIS)

The Modified Fatigue Impact Scale (MFIS) will assess change in the level of fatigue. The MFIS is a 21-item instrument that asks patients to rate their fatigue over the past four weeks on a 5-point Likert scale, indicating "Never" to "Almost always." Four scores can be derived from the MFIS, including a total score as well as scores for three subscales: physical, cognitive, and psychosocial functioning. Changes from baseline will be calculated for the total scale scores as well as for the subscale scores.

English version of MFIS is provided in Appendix 5.

### 5.7.2 The Center for Epidemiologic Studies Depression Scale (CES-D)

The Center for Epidemiologic Studies Depression Scale (CES-D) will be used to evaluate patients for depressive symptoms. The CES-D is a 20-item self-report instrument that asks patients to rate their feelings and behaviors over the past week on a 4-point Likert

scale, from “Rarely or none of the time (less than one day)” to “Most or all of the time (5-7 days).” Only a total scale score is calculated for the CES-D.

English version of CES-D is provided in Appendix 6.

#### 5.7.3 The Short Form (SF-36) Health Survey

The Short Form (SF-36) Health Survey is a generic quality of life instrument that has been widely tested for its psychometric properties and is widely used in clinical and epidemiological studies. The SF-36 contains 36 items and measures eight health domains: vitality, physical functioning, bodily pain, general health perceptions, physical role functioning, emotional role functioning, social role functioning, and mental health. The SF-36 yields a score for each domain, as well as summary scores for the physical and mental dimensions, and a single health utility index. It can be completed in 5-10 minutes.

English version of the Short Form (SF-36) Health Survey is provided in Appendix 7.

#### 5.7.4 Patient’s Assessment of Treatment Benefit

The Patient Perception of Treatment Questionnaire will be assessed before administration of PRO instruments at each visit in which PRO assessments are made, except for baseline. Specifically, patients will be asked whether they think their MS has become better, become worse or been stable since baseline. This question will serve as a global assessment from the patient perspective and will provide a useful anchor to help interpret the clinical meaningfulness of PRO results.

### 5.8 Pharmacoeconomic Assessments/ EQ-5D

Pharmacoeconomic assessments will be included for purposes of deriving health utilities for economic modeling. The EQ-5D will be used to derive utilities for health states included in MS economic models and will be administered as per Schedule of Assessments.

The EQ-5D (formerly known as EuroQOL) is a generic, preference-based health-related quality of life instrument. It has five dimensions assessing mobility, self-care, usual activities, pain/discomfort and anxiety /depression. Each dimension has 3 possible levels. Different combinations of responses are utility-weighted to produce a single health utility index. The Visual Analog Scale (VAS) measures self-reported health on a scale between “worst imaginable“ and “best imaginable“ health states.

EQ-5D is a patient reported outcome and should be performed before any other study assessments and before administration of study drug in order to minimize bias.

### 5.9 Post Study Provisional Care

Patients who complete the 96-week double-blind treatment period, and who in the opinion of the investigator may benefit from the further treatment with ocrelizumab, may become eligible for a separate open label extension study, under a separate protocol.

For patients who have withdrawn from treatment or who are not otherwise eligible for treatment with ocrelizumab, it is at the discretion of the investigator to decide on further treatment of the underlying disease However, immunosuppressants, lymphocyte depleting therapies, and lymphocyte tracking blockers may increase the risk of infections while patients remain B-cell depleted and therefore are not allowed during the Safety Follow up Period.

# 6. INVESTIGATIONAL MEDICINAL PRODUCT

Patients will be randomly assigned into one of two treatment groups:

- **Group A** – Ocrelizumab 600 mg regimen (given as dual infusions of 300 mg of ocrelizumab 14 days apart for the first 24 weeks and single infusions of 600 mg every 24 weeks thereafter)
- **Group B** – Rebif® subcutaneous injections, 3x weekly

**Table 5: Treatment Groups and Schedule of Study Medication**

| Treatment Group | Schedule of Study Medication |
|---|---|
| **Group A**<br>Ocrelizumab 600 mg regimen | Two i.v. infusions of ocrelizumab 300 mg separated by 14 days for the first 24 weeks, followed by single i.v. infusions of ocrelizumab 600 mg every 24 weeks thereafter<br>AND<br>placebo Rebif® subcutaneous injections, 3x weekly. |
| **Group B**<br>Rebif® | Rebif® subcutaneous injections, 3x weekly<br>AND<br>Two i.v. infusions of placebo ocrelizumab 300 mg separated by 14 days for the first 24 weeks, followed by single i.v. infusions of placebo ocrelizumab 600 mg every 24 weeks thereafter. |

The first i.v. infusion of ocrelizumab or placebo, or the first subcutaneous injection of Rebif® or placebo will be administered on study Day 1.

Please note: 100 mg of methylprednisolone i.v. will be administered prior to each intravenous infusion of ocrelizumab/ocrelizumab placebo.

## 6.1 Ocrelizumab

### 6.1.1 Preparation and Administration of Ocrelizumab Infusions

**Detailed instructions for the preparation of the infusion bags containing the study drug will be provided separately in the Dose Preparation Guidelines.**

Although ocrelizumab may be administered on an outpatient basis, patients may be hospitalized for observation at the discretion of the investigator (in some countries this is the standard procedure). The study drug infusions should always be administered in a hospital or clinic environment under close supervision of the

investigator or a medically qualified staff member with immediate availability of full resuscitation facilities.

**Preparation of infusion**

Ocrelizumab drug product must be diluted before administration. Solutions of ocrelizumab for i.v. administration are prepared by dilution of the drug product or ocrelizumab-matching placebo into an infusion bag containing 0.9% sodium chloride, to a final drug concentration of approximately 0.7 to 2 mg/mL. It is important not to use evacuated glass containers (to prepare the infusion), which require vented administration sets because this causes foaming as air bubbles pass through the solution.

Prior to the start of the infusion, please ensure that the content of the bags is at room temperature to avoid an infusion reaction due to the administration of the solution at low temperatures.

**Infusion procedures**

Ocrelizumab should be given as a slow i.v. infusion. It must not be administered as an i.v. push or bolus. Well adjusted infusion pumps should be used to control the infusion rate and the study drug should be infused through a dedicated line.

All patients should receive pre-treatment before the infusion (see Section 6.1.2).

**Dual infusion cycle (Cycle 1)**

The first cycle will consist of 2 infusions of 300 mg ocrelizumab administered 14 days apart. For each infusion it is necessary to prepare a single infusion bag containing 300 mg ocrelizumab. Specific instructions will be provided separately in the Dose Preparation Guidelines and must be followed exactly. The infusion should be started at a rate of 25 mL/h. This should be escalated at the rates shown in Table 6.

**Table 6: Infusions of Ocrelizumab 300 mg**

| Time (Minutes) | Infusion Rate (mL/hr) | Maximum Dose per Interval (mg) | Cumulative Dose (mg) |
|---|---|---|---|
| 0-30 | 25 | 18.75 | 18.75 |
| 31-60 | 50 | 37.5 | 56.25 |
| 61-90 | 75 | 56.25 | 112.5 |
| 91-120 | 100 | 75 | 187.5 |
| 121-150* | 150 | 112.5 | 300* |

*Infusion of 300 mg of ocrelizumab should be completed at approximately 150 minutes (~2.5 hours)

**Single infusion cycles (Cycles 2 - 4)**

Cycles 2 through 4 will consist of one infusion of 600 mg ocrelizumab administered on Day 1 of each cycle. For each cycle it is necessary to prepare **two infusion bags** containing a total of 600 mg ocrelizumab. Specific instructions will be provided separately in the Dose Preparation Guidelines and must be followed exactly. The infusion should be started at a rate of 25 mL/h. This should be escalated at the rates shown in Table 7.

**Table 7: Subsequent Infusions of Ocrelizumab 600 mg**

| Infusion Bag | Time (Minutes) | Infusion Rate (mL/hr) | Maximum Dose per Interval (mg) | Cumulative Dose (mg) |
|---|---|---|---|---|
| Bag 1 | 0-30<br>31-60<br>61-90<br>91-120<br>121-150 | 25<br>50<br>75<br>100<br>150 | 25<br>50<br>75<br>100<br>150 | 25<br>75<br>150<br>250<br>400* |
| Bag 2 | 151-180<br>181-210<br>210-240** | 200<br>200<br>200 | 66.7<br>66.7<br>66.7 | 467<br>533<br>600 |

*Change to Bag 2
**Infusions of Bag 1 and Bag 2 should be completed at approximately 240 minutes (≈4 hours)

**Please note: The contents of Bag 1 and Bag 2 will not be identical with respect to volume and drug concentration. Therefore, it is essential that the specific instructions provided in the Dose Preparation Guidelines be followed exactly. In addition, since the contents of Bags 1 and 2 vary, the regular increments in the infusion rates also vary. Thus, the bags must be administered strictly in the correct order according to the instructions outlined in Table 7.**

Because of the varying volumes in the infusion bags and the possible need to vary infusion rates depending on tolerance of the infusion, the total infusion time may exceed the time stated. UNLESS AN INFUSION REACTION OCCURS NECESSITATING DISCONTINUATION, THE ENTIRE CONTENTS OF BOTH INFUSION BAGS MUST BE ADMINISTERED TO THE PATIENT.

After completion of the infusion, the i.v. cannula should remain in situ for at least 1 hour in order to be able to administer drugs intravenously, if necessary in the event of a delayed reaction. If no adverse events occur during this period of time, the i.v. cannula may be removed and the patient may be discharged.

Because ocrelizumab solutions for infusion do not contain a preservative, the i.v. bags containing ocrelizumab diluted solutions should be stored refrigerated at 2-8°C. They may be stored under refrigerated conditions for up to 24 hours prior to use. Bags containing ocrelizumab diluted solutions for i.v. infusion need to be used within 48 hours of preparation (i.e., stable 24 hours at 2-8°C plus 24 hours at room temperature). As noted above, the diluted infusion bags should be at room temperature prior to administration to the patient.

### 6.1.2 Prevention and Treatment of Infusion Related Reactions

Methylprednisolone has been shown to decrease the incidence and the severity of infusion reactions. In RA patients treated with a similar agent, rituximab, the rate and severity of infusion reactions markedly decreased with i.v. corticosteroid pre-medication [54]. To reduce potential infusion reactions, all patients will receive prophylactic

treatment with 100 mg of methylprednisolone, administered by slow i.v. infusion, to be completed approximately 30 minutes before the start of each ocrelizumab infusion.

It is also recommended that the infusion is accompanied by prophylactic treatment with an analgesic/antipyretic such as acetaminophen/paracetamol (1 g) and an i.v. or oral antihistaminic (such as i.v. diphenhydramine 50 mg; or equivalent dose of alternative) 30 to 60 minutes prior to the start of an infusion to reduce potential infusion reactions. Patients administered a sedating antihistaminic for the treatment or prevention of infusion reactions should be given appropriate warnings concerning drowsiness and potential impairment of ability to drive or operate machinery.

Since transient hypotension may occur during ocrelizumab infusion, the investigator may wish to withhold anti-hypertensive medications 12 hours prior to ocrelizumab infusion.

For the treatment of fever, discomfort or allergic events, the use of oral acetaminophen/paracetamol (1 g), and intramuscular or slow i.v. antihistaminic administration, such as diphenhydramine (25 mg to 100 mg i.v.), and/or a bronchodilator, is recommended. The acetaminophen/paracetamol and diphenhydramine doses should be repeated as clinically indicated. Non allergic events should be treated symptomatically as judged clinically relevant by the investigator.

**In patients with CTCAE Grade 3 or higher (severe) infusion reactions with associated respiratory symptoms (stridor, wheeze or bronchospasm), additional treatment with bronchodilators may be indicated.**

One patient with well-controlled asthma at baseline experienced an acute asthma attack following their first rituximab infusion. Physicians should therefore monitor patients with a history of asthma carefully and institute an appropriate treatment if signs and symptoms of asthma are noticed.

Section 6.1.3 details the reduction, interruption or discontinuation of the infusion in the event of an infusion reaction.

### 6.1.3 Ocrelizumab Dose Modifications, Interruptions and Delays

No ocrelizumab dose modifications are foreseen.

Slowing of the infusion rate or interruption of the infusion may be necessary in the event of an infusion reaction. In rare patients, ocrelizumab treatment may need to be discontinued. Guidance is provided below.

**Handling infusion reactions:**

In the event that a patient experiences a mild to moderate (CTCAE Grade 1 or 2 – Appendix 3) infusion-related event, the infusion rate should be reduced to half the rate being given at the time of onset of the event (e.g. from 50 mL/hr to 25 mL/hr or from 100 mL/hr to 50 mL/hr). Once the event has resolved, the investigator should wait for 30 minutes while delivering the infusion at the reduced rate. If tolerated, the infusion rate may then be increased to the next closest rate on the patient’s infusion schedule and the rate increments resumed.

Patients who experience a severe infusion-related event (CTCAE Grade 3) or flushing, fever and throat pain cluster should have their infusion interrupted immediately and should receive aggressive symptomatic treatment. The infusion should be re-started only after all the symptoms have disappeared. The initial infusion rate at restart should be half of the infusion rate that was in progress at the time of onset of the reaction.

**Please note: patients who experience a life threatening or disabling infusion-related event (CTCAE Grade 4), such as anaphylaxis, during an infusion should have their infusion immediately stopped and should receive appropriate treatment (including use of resuscitation medications and equipment that must be available and used as clinically indicated). These patients should be withdrawn from treatment and should enter the Safety Follow-up Period.**

### 6.1.4 Criteria for Re-Treatment with Ocrelizumab

Prior to re-treatment with ocrelizumab, patients will be evaluated for the following conditions and laboratory abnormalities. If any of these are present prior to re-dosing, further administration of ocrelizumab should be suspended until resolved or held indefinitely:

- Severe allergic or anaphylactic reaction to a previous ocrelizumab infusion
- Any significant or uncontrolled medical condition or treatment-emergent, clinically significant laboratory abnormality
- Active infection
- Absolute neutrophil count < 1.5 x $10^3$/µL
- CD4 cell count < 250/µL
- Hypogammaglobulinemia IgG < 4.0 g/L

Please note: any critical blinded laboratory values for IgG, absolute neutrophil count and CD4 will be provided to the Treating Investigator and the Medical Monitor. Investigators notified of their patient’s critical laboratory test result will be instructed to suspend further treatment with study drug until the patient can be further evaluated. A repeat laboratory test may be necessary to confirm the results. Patients with values below these critical values should not be retreated until the re-treatment criteria are met and these laboratory values have normalized.

## 6.2 Rebif®

### 6.2.1 Dose and Schedule of Rebif®

Please refer to Table 8 for overview of Rebif® regimen.

The first subcutaneous injection of Rebif®/placebo will be administered on Study Day 1. Patients will be instructed by a nurse or investigator how to self-administer the injections; the first dose of Rebif®/placebo will be self-administered under the supervision of a nurse or physician. Thereafter, patients will self-administer their Rebif®/placebo treatment three times weekly. Rebif®/placebo must be administered, if possible, at the same time (preferably in the late afternoon or evening) on the same three days (e.g., Monday, Wednesday, and Friday) at least 48 hours apart. Patients must be instructed in the use of

aseptic techniques when administering Rebif®/placebo injections. Patient understanding and use of aseptic self-injection techniques and procedures must be periodically re-evaluated.

Since Rebif® needs to be stored at 2-8°C, it is recommended to remove the syringe from refrigerator at least 30 minutes prior to use. Patient should be reminded not to heat or microwave a syringe.

When starting treatment with Rebif®, the dose will be gradually escalated (please refer to Table 8). The Rebif®/placebo initiation package corresponds to the patient needs for the first month of treatment.

**Table 8: Overview of Rebif® Dosing Regimen ***

| | Treatment Initiation | | Treatment Continuation | Dose modification (if required) |
|---|---|---|---|---|
| **Week** | **Weeks 1- 2** | **Weeks 3-4** | **Week 5 onwards** | – |
| *Study Day* | *1-14* | *15-28* | *29+* | *At any time >Day 29* |
| **Dose of Rebif®** | **Rebif® 8.8 µg** (1 pre-filled syringe [0.2 ml] containing 2.4 MIU of interferon β-1a) s.c. **3x per week** | **Rebif® 22 µg** (1 pre-filled syringe [0.5 ml] containing 6 MIU of interferon β-1a) s.c. **3x per week** | **Rebif® 44 µg** (1 pre-filled syringe [0.5 m] containing 12 MIU of interferon β-1a) s.c. **3x per week** | **Rebif® 22 µg** (1 pre-filled syringe [0.5 ml] containing 6 MIU of interferon β-1a) s.c. **3x per week** |

** provided in blinded fashion*

Non-steroid anti-inflammatory drugs (ibuprofen) or acetaminophen are recommended in case of injection site reaction; investigator should follow local label for further information.

### 6.2.2 Rebif® Dose Modifications, Interruptions and Delays

Rebif®/ Rebif® placebo should be taken three times a week. Rebif®/ Rebif® placebo should never be taken on two consecutive days. If a patient misses a dose, then the next dose must be taken as soon as possible. The patient should avoid taking Rebif®/ Rebif® placebo on the following day. The patient should return to their regular schedule the following week. If a patient takes more than the prescribed dose or takes it on 2 consecutive days, they should inform the investigator immediately.

Asymptomatic increases in laboratory parameters of hepatic function have been associated with Rebif®.

In case of elevation of liver function tests the following rules will apply:

| | |
|---|---|
| – **ALT ≥ 10 x ULN** OR jaundice or other clinical symptoms of liver dysfunction | In case of detection of elevated **ALT ≥ 10 x ULN**, jaundice or other clinical symptoms of liver dysfunction the injections of Rebif®/Rebif® placebo must be discontinued permanently. The monitoring of liver function tests should be continued on a monthly basis until return to normal baseline levels or CTCAE v.4.0 Grade 1 toxicity (ALT: >ULN - 3.0 x ULN). A consultation with hepatologist is recommended. Patients should move to Safety Follow up Period. |
| – **ALT ≥ 5 x ULN** | In case of detection of elevated **ALT ≥ 5 x ULN** (but below 10 xULN) the injections of Rebif®/Rebif® placebo must be discontinued temporarily. Additional blood chemistry panel including AST, ALP, GGT and bilirubin should be performed biweekly until no further increase is observed. Subsequently, ALT analysis has to be performed every month until return to normal baseline levels or CTCAE v.4.0 Grade 1 toxicity (ALT >ULN - 3.0 x ULN). A consultation with hepatologist should be considered as per investigator judgment.<br>If causes of toxicity other than possible treatment with Rebif® are excluded, the patient may then be cautiously re-challenged with Rebif®/Rebif® placebo 22µg provided in a blinded fashion upon request to IxRS. The monitoring of liver function tests should continue on a monthly basis. If there is no further recurrence of toxicity, patient may continue treatment with Rebif®/Rebif® placebo 44 µg provided in a blinded fashion upon investigator's request to IxRS. **In case of recurrence of toxicity (ALT > 3 xULN, or other clinical symptoms of liver dysfunction) the injections of Rebif®/Rebif® placebo should be discontinued permanently.** Patients should move to Safety Follow up Period.<br><br>Please note: Re-initiation of therapy with Rebif® following elevation of liver function tests can only be considered once. |
| – **ALT > 3 x ULN** | In case of detection of elevated **ALT > 3 x ULN** (but below 5x ULN) additional blood chemistry panel including AST, ALP, GGT and bilirubin should be performed biweekly until no further increase is observed. Subsequently, ALT analysis has to be performed every month until return to normal baseline levels or CTCAE v.4.0 Grade 1 toxicity (ALT>ULN - 3.0 x ULN). |

## 6.3 Formulation, Packaging and Labeling

Study drug packaging will be overseen by the Roche clinical trial supplies department and bear a label with the identification required by local law, the protocol number, drug identification and dosage.

The packaging and labeling of the study medication will be in accordance with Roche standards and local regulations.

Upon arrival of investigational products at the site, site personnel should check them for damage and verify proper identity, quantity, integrity of seals and temperature conditions, and report any deviations or product complaints to the monitor upon discovery.

### 6.3.1 Ocrelizumab

**Formulation**

Ocrelizumab is manufactured as a sterile, clear, colorless, preservative free liquid intended for dilution for i.v. administration.

Ocrelizumab is supplied as a liquid formulation containing 30 mg/mL ocrelizumab in 20 mM sodium acetate at pH 5.3, with 4% (106 mM) trehalose dihydrate and 0.02% polysorbate 20. The drug product is provided as a single-use liquid formulation in a 15 cc Type I USP glass vial, fitted with a 20 mm fluoro-resin laminated stopper and an aluminum seal with a flip-off plastic cap and contains a nominal 200 mg ocrelizumab. No preservative is used as each vial is designed for single use.

Ocrelizumab-matching placebo is also supplied in 15 cc single-use vials. Placebo has the same composition and configuration as the drug product, but does not contain ocrelizumab.

**Packaging**

The hospital units/pharmacy will receive study medication kits for each patient.

For the double-blind treatment in Cycle 1, consisting of two 300 mg infusions 14 days apart, the study medication kit will contain 2 single-use liquid vials with ocrelizumab (or ocrelizumab placebo).

For each of the subsequent Cycles 2-4 consisting of a single 600 mg infusion, two kits will be dispensed. Each kit will contain 2 single-use liquid vials with ocrelizumab (or ocrelizumab placebo), from which only 3 vials should be used.

**Storage of Ocrelizumab and Placebo Vials for Infusion:**

Ocrelizumab and placebo vials are stable at 2-8°C (refrigerated storage). They should not be used beyond the expiration date stamped on the carton. Expiration dating may be extended during the trial; the Sponsor will provide documentation. Ocrelizumab vials should not be frozen or shaken and should be protected from direct sunlight.

The study medication labels will be produced in accordance with the local requirements.

### 6.3.2 Rebif®

**Formulation and packaging**

Rebif® (IFN β-1a) will be supplied as a liquid formulation for injection in pre-filled syringes.

The liquid formulation is supplied in syringes containing 0.2 mL or 0.5 mL of solution. These commercially available syringes will be provided to the sites by the Sponsor and re-labeled as investigational medicinal product.

The placebo to Rebif® is provided as a liquid formulation in a pre-filled syringe containing 0.2 mL or 0.5 mL of 0.9% sodium chloride solution without any active substance.

The study medication kits, which will be used for the initial 4 weeks of treatment, will contain 12 pre-filled syringes, either 6x 8.8 µg and 6x 22 µg OR placebo. The study medication kits that will be used for treatment continuation will contain 12 pre-filled syringes 12x44 µg of IFN β-1a or 12x22 µg of IFN β-1a or placebo

The Rebif® and Rebif® placebo pre-filled syringes are for subcutaneous use only.

Please note: if Rebif® dose modification is required due to lab abnormalities possibly related to the treatment with Rebif®, the investigator (the treating physician) will need to notify IxRS and the blinded study medication (Rebif® placebo or Rebif® verum) will be dispensed accordingly. In addition, to ensure patient safety in the study, unscheduled visits may be required for additional assessments, monitoring and for dispensing study medication.

The study medication labels will be produced in accordance with the local requirements. The strength will be presented as follows: 44 µg / 22 µg / placebo.

**Storage of Rebif®**

Rebif®/Rebif® placebo pre-filled syringes need to be stored in a refrigerator at 2-8°C, in the original package in order to protect from light. The patient may remove Rebif® from the refrigerator and store it not above 25°C for one single period of up to 14 days. Rebif® must then be returned to the refrigerator and used before the expiry date.

## 6.4 Blinding and Unblinding

The Patient Randomization List will be generated by IxRS using a pre-defined randomization specification. The Randomization List will not be available at the study center, to the Roche monitors, project Statisticians or to the Sponsor's project team. Unblinding of treatment assignment should not occur except in the case of emergency situations, where the knowledge of what study medication the patient is receiving is critical for clinical management. Treating Investigators are asked to contact the Roche Medical Monitor, prior to unblinding any patient, in order to discuss the medical necessity for unblinding. Any request from the investigator for information about the treatment administered to study patients for another purpose must be discussed with Roche. Unblinding will be performed by means of an Interactive Web Response System (IxRS). As per regulatory reporting requirement, Roche will unblind the identity of the

study medication for serious adverse events that are considered by the investigator or the Sponsor to be related to study drug, that are unexpected as per safety reference document(s), e.g., IB, CDS, and SPC, and that are not exempted from unblinding as per Section 7.2.2.2. Details of patients who are unblinded during the study will be included in the Clinical Study Report.

Unblinding for analysis of biological samples, or ongoing safety monitoring by a Data Monitoring Committee [DMC], will be performed according to procedures in place to ensure integrity of the data.

## 6.5 Accountability of IMP and Assessment of Compliance

### 6.5.1 Accountability of IMP

The investigator is responsible for the control of drugs under investigation. Adequate records for the receipt and disposition of the study drug must be maintained. Accountability will be assessed by maintaining adequate drug dispensing and return records.

Accurate records must be kept for each study drug provided by the Sponsor. These records must contain the following:

- Documentation of drug shipments received from the Sponsor (date received and quantity)
- Disposition of unused study drug not dispensed to patient.

A Drug Dispensing Log must be kept current and should contain the following information:

- the identification of the patient to whom the study medication was dispensed
- the date[s] and quantity of the study medication dispensed *to* the patient
- the date[s] and quantity of the study medication returned *by* the patient.

All records and drug supplies must be available for inspection/accountability by the Monitor at every monitoring visit.

### 6.5.2 Assessment of Compliance

Patient compliance will be assessed by maintaining adequate study drug dispensing records. The investigator is responsible for ensuring that dosing is administered in compliance with the protocol. Delegation of this task must be clearly documented and approved by the investigator.

The study pharmacist should keep all ocrelizumab/ocrelizumab placebo vials to measure compliance. All patients will be asked to return on regular intervals all used and unused Rebif®/Rebif® placebo containers to the site as a measure of compliance.

## 6.6 Destruction of the IMP/Comparator

Local or institutional regulations may require immediate destruction of used IMP for safety reasons. In these cases, it may be acceptable for investigational site staff to destroy dispensed IMP before a monitoring inspection provided that source document verification is performed on the remaining inventory and reconciled against the documentation of

quantity shipped, dispensed, returned and destroyed. Written authorization must be obtained from the Sponsor at study start up before destruction.

Written documentation of destruction must contain the following:

– Identity (batch numbers or medication numbers) of IMP and comparators destroyed

– Quantity of IMP destroyed

– Date of destruction

– Method of destruction

– Name and signature of responsible person who destroyed the IMP.

Wherever possible, preferably drug should be destroyed locally on site according to their local policies and procedures once drug accountability has been completed by the monitor.

# 7. SAFETY INSTRUCTIONS AND GUIDANCE

## 7.1 Adverse Events (AEs) and Laboratory Abnormalities

### 7.1.1 Clinical AEs

According to the International Conference of Harmonisation (ICH), an AE is any untoward medical occurrence in a patient or clinical investigation patient administered a pharmaceutical product and which does not necessarily have a causal relationship with this treatment. An AE can therefore be any unfavorable and unintended sign, including an abnormal laboratory finding, symptom, or disease temporally associated with the use of a (investigational) medicinal product, whether or not considered related to the medicinal (investigational) product. Pre-existing conditions which worsen during a study are to be reported as AEs.

In the eCRF, adverse events will be reported at each visit.

**Clinical relapses** will be recorded only on a pre-specified eCRF “MS relapse” eform.

Infusion-related reactions will be recorded only on a pre-specified eCRF “Infusion Related Reaction” eform.

B-cell depletion is the expected outcome of ocrelizumab treatment and is not an adverse event. However, patients may be at risk for infections and particular attention should be directed toward early identification and treatment of infections. During the study, investigators are requested to promptly investigate patients reporting signs or symptoms of infection, to take appropriate specimens for identification of the pathogen and to treat infections aggressively (see Section 7.3.1). Prior to enrollment into the study, it is recommended that the investigators review and, if warranted, update patient’s immunizations in accordance with country medical immunization guidelines (see also Section 4.4.3).

#### 7.1.1.1 Intensity

Adverse events will be graded according to Common Terminology Criteria for Adverse Events (CTCAE), version 4 and is provided to the investigator in a separate handout entitled "Common Terminology Criteria for Adverse Events v4.0"- see Appendix 3.

Adverse events not listed by the CTCAE will be graded using the following criteria:

Grade 1: Discomfort noticed but no disruption of normal daily activity

Grade 2: Discomfort sufficient to reduce or affect normal daily activity

Grade 3: Inability to work or perform normal daily activity

Grade 4: Represents an immediate threat to life.

Any Grade 4 or higher AE, either by CTCAE criteria or the additional criteria listed below, should be reported as an SAE (see Section 7.1.1.3).

#### 7.1.1.2 Drug - Adverse Event Relationship

**Relationship** of the AE to the treatment should always be assessed by the investigator. The causality relationship of study drug to the adverse event will be assessed by the investigator as either: Yes or No. Please refer to Appendix 1 for more details.

#### 7.1.1.3 Serious Adverse Events (Immediately Reportable to Sponsor)

A Serious Adverse Event is any experience that suggests a significant hazard, contraindication, side effect or precaution. It is any AE that, at any dose, fulfils at least one of the following criteria:

– is fatal; (results in **death***; please note: death is an outcome, not an event)

– is Life-Threatening (please note: the term "Life-Threatening" refers to an event in which the patient was at immediate risk of death at the time of the event; it does not refer to an event which could hypothetically have caused a death had it been more severe).

– required in-patient hospitalization or prolongation of existing hospitalization;

– results in persistent or significant disability/incapacity;

– is a congenital anomaly/birth defect;

– is medically significant or requires intervention to prevent one or other of the outcomes listed above.

***The term sudden death should only be used when the cause is of a cardiac origin as per standard definition. The terms death and sudden death are clearly distinct and must not be used interchangeably**.

The exception to this definition of an SAE is in the rare event that a patient is hospitalized following an MS relapse, as long as the reason for hospitalization is to receive standard treatment with i.v. methylprednisolone. The rationale for this exception is that some countries and/or clinical sites routinely hospitalize patients who require

administration of methylprednisolone in the event of an MS relapse. Thus, the SAE criteria for "hospitalization" would be met on the basis of local practice and would not reflect the seriousness of the event.

When the MS relapse results in hospitalization for any reason other than for routine treatment of the relapse (such as for a treatment course beyond the standard treatment described in (see Section 4.4.2) or when hospitalization is prolonged, the MS relapse should be considered a SAE.

The study will comply with all local regulatory requirements and will adhere to the full requirements of the ICH Guideline for Clinical Safety Data Management, Definitions and Standards for Expedited Reporting, Topic E2 (see Appendix 2).

### 7.1.2 Treatment and Follow-up of AEs

Adverse events (AEs) should be followed up until they have stabilized or have returned to baseline status (in the event of an exacerbation of a pre-existing condition). This is especially important for those events where the reported causal relationship to study medication(s) is "related". If a clear explanation is established, it should be recorded on the eCRF.

If after study completion or withdrawal, return to baseline status or stabilization cannot be established an explanation should be recorded on the eCRF.

### 7.1.3 Laboratory Test Abnormalities

Laboratory test results will be recorded on the laboratory results eform of the eCRF, or appear on electronically produced laboratory reports submitted directly from the central laboratory, if applicable.

Any treatment-emergent abnormal laboratory result which is clinically significant, i.e., meeting one or more of the following conditions, should be recorded as a single diagnosis on the AE eform in the eCRF:

- Accompanied by clinical symptoms,
- Leading to a change in study medication (e.g. dose modification, interruption or permanent discontinuation),
- Requiring a change in concomitant therapy (e.g. addition of, interruption of, discontinuation of, or any other change in a concomitant medication, therapy or treatment).

Any laboratory result abnormality fulfilling the criteria for an SAE should be reported as such, in addition to being recorded as an AE in the eCRF.

#### *7.1.3.1 Follow-up of Abnormal Laboratory Test Values*

In the event of medically significant unexplained abnormal laboratory test values, the tests should be repeated and followed up until they have returned to the normal range and/or an adequate explanation of the abnormality is found. If a clear explanation is established it should be recorded on the eCRF.

B-cell depletion is a pharmacodynamic effect and is not an adverse event.

**Blinded laboratory values for IgG, absolute neutrophil count and CD4 which are critical will be provided to the investigator and the Medical Monitor. Investigators notified of their patient's critical laboratory test result will be instructed to suspend further treatment with study drug until the patient can be further evaluated. A repeat laboratory test may be necessary to confirm the results. Patients with values below these critical values should not be re-treated until the re-treatment criteria are met (see Section 6.1.4) and these laboratory values have normalized.**

## 7.2 Handling of Safety Parameters

### 7.2.1 Reporting of AEs

All adverse events will be documented in the eCRF.

New or worsening neurological symptoms not considered MS-related should be recorded on an AE page and the monitor should be informed.

### 7.2.2 Reporting of Serious Adverse Events

#### *7.2.2.1 Immediate Reporting to the Sponsor*

Any clinical AE or abnormal laboratory test value that is *serious* (as defined in Section 7.1.1.3), which occurs during the course of the study, regardless of the treatment group, must be reported to the Sponsor **within *one* working day** of the investigator becoming aware of the event (expedited reporting). In addition, for fatal and life-threatening events, the Medical Monitor should be contacted immediately. Contact numbers for the Medical Monitor (including after hours cover) will be provided to the site before any patients are screened.

After the patient signs the Informed Consent, but prior to initiation of study medication, only SAEs caused by a protocol-mandated intervention will be collected (e.g., SAEs related to MRI exam). After first dose of study medication, all SAEs must be reported.

Related SAEs ***MUST*** be collected and reported regardless of the time elapsed from the last study drug administration, even if the study has been closed.

Unrelated SAEs must be collected and reported during the study through the end of the Safety Follow-up Period, which is at least 48 weeks after the last infusion but may be extended in patients whose B-cells take longer to replete.

The investigator must complete the SAE reporting form in the eCRF. Relevant follow-up information should be submitted as soon as it becomes available. Only if a technical failure prevents the ability to report an SAE in the eCRF, then the paper *SAE Reporting Form* provided by the Sponsor must be completed and faxed to the number provided.

A death occurring during the study or information related to such occurrence that comes to the attention of the investigator during the study must be reported immediately to the Sponsor, whether considered treatment-related or not.

The following are not considered as an SAE:

- Elective hospitalizations or surgical procedures that are a result of a patient's pre-existing condition(s) that have not worsened since receiving trial medication. Examples may include, but are not limited to, cholecystectomy for gallstones, and diagnostic testing. Such events should still be recorded as medical procedures in the eCRF.
- Hospitalization to receive trial medication such as infusions of ocrelizumab unless this is prolonged (more than 24 hours).
  Hospitalization following an MS relapse as long as the reason for hospitalization is to receive standard treatment with i.v. methylprednisolone

Of specific importance is the prompt reporting of serious infections. In particular, PML should be reported as an SAE (with all available information) with immediate notification of the Medical Monitor.

This study adheres to the definition and reporting requirements of ICH Guideline for Clinical Safety Data Management, Definitions, and Standards for Expedited Reporting, Topic E2 (see Appendix 2).

#### 7.2.2.2 Expedited Reporting to Health Authorities, Investigators, Institutional Review Boards, and Ethics Committees

The Sponsor will promptly evaluate all reported SAEs against cumulative product experience to identify and expeditiously communicate possible new safety findings to investigators, IRBs, ECs, and relevant health authorities based on applicable legislation.

Reporting requirements will be based on the investigator's assessment of causality and seriousness, with allowance for upgrading by the Sponsor as needed. To determine reporting requirements for single adverse event cases, the Sponsor will also assess the expectedness of the event on the basis of the Investigator's Brochure.

In principle, adverse events which are serious, related and unexpected will be reported in an expedited manner within 15 days (non-fatal/non-life-threatening) or 7 days (fatal or life-threatening).

Only those adverse events qualifying for expedited reporting occurring in patients on active treatment will be sent in an expedited timeframe to Health Authorities, Investigators, Institutional Review Boards, and Ethics Committees. This requires unblinding of patient treatment allocation.

For certain types of adverse events, the relation to study medication cannot be assessed based on single case evaluation. Therefore, in order to prevent unnecessary unblinding of study participants, the following events are exempted from expedited reporting:

- neoplasms benign, malignant, and unspecified (including cysts and polyps),

- infections and infestations with the exception of opportunistic infections (including PML and reactivation of viral infections).

The DMC will review adverse events at each quarterly meeting and assess their relation to study medication based on review of aggregate unblinded safety information.

### 7.2.3 Pregnancy and Lactation

Female patients should take all appropriate precautions to avoid becoming pregnant during this study and for the entire duration of B-cell depletion. As such, women of childbearing potential should use adequate contraception for the duration of the trial and for 48 weeks after receiving their last infusion of ocrelizumab, or until their B-cells have repleted whichever is the longer. Regular pregnancy tests will be performed during the study. If a female patient becomes pregnant during the study and chooses to carry her pregnancy, no further infusions of ocrelizumab should be administered.

Effects on pregnancies from the female partners of B-cell depleted males have not been studied. Therefore, it is required that male patients also use reliable contraception while receiving ocrelizumab treatment in this study for 48 weeks after receiving their last infusion of ocrelizumab, or until their B-cells have repleted whichever is the longer.

Reproductive toxicology studies of ocrelizumab conducted in cynomolgus monkeys are described in the IB. Studies of the effect of ocrelizumab on human reproduction have not been performed. It is not known whether ocrelizumab can cause fetal harm when administered to pregnant women or whether it can affect reproductive capacity. However, since IgG molecules such as ocrelizumab are known to cross the placenta, ocrelizumab may cause fetal CD20 B-cell depletion. It is not known whether ocrelizumab is excreted in breast milk, and what effect this might have on the breast feeding infant. However, since immunoglobulins are found in breast milk, breast feeding mothers are excluded from participation in the study.

Well-controlled reproductive studies with corticosteroids have not been performed in humans but high doses of corticosteroids given during pregnancy have caused hypoadrenalism in newborns.

Regardless of the treatment assignment, a female patient must be instructed to immediately inform the investigator if she becomes pregnant during the study (including the Safety Follow up Period). If she chooses to carry her pregnancy, she must be withdrawn from treatment, and she should enter the Safety Follow up Period. The investigator should report all pregnancies within 24 hours to the Sponsor by means of an eCRF Pregnancy Reporting Form.

As ocrelizumab may cross the placenta and cause B-cell depletion in the neonate, babies born to mothers participating in this study should have an assessment of their lymphocyte counts and be carefully followed until these are within the normal range for the age of the infant. The investigator should counsel the patient as to the risks of continuing with the pregnancy and the possible effects on the fetus. Monitoring of the patient should continue until conclusion of the pregnancy. Informed consent will be sought for the Sponsor to collect information on the health and well being of the baby.

Whether the drug is excreted in the semen is unknown. Therefore, pregnancy occurring in the partner of a male patient participating in the study should also be reported to the investigator, and the investigator should inform the Sponsor. If appropriate, an additional consent form will be provided (subject to ethics committee review) to solicit information about the pregnancy.

## 7.3 Warnings and Precautions

### 7.3.1 Ocrelizumab

Patients should be informed of the risks associated with taking ocrelizumab. Below are listed specific major risks of which the patients should be made aware. Further information on ocrelizumab is given in the current version of IB.

**Infusion-Related Reactions**

All CD20 depleting agents including ocrelizumab have been associated with acute infusion-related reactions (fever, urticaria/rash, chills, rigors, sneezing, angioneurotic edema, throat irritation, nausea, fatigue, headache, dyspnea, rhinitis, vomiting, or flushing cough and bronchospasm, with or without associated hypotension or hypertension). Some of these events have been severe enough to warrant interruption or discontinuation of the infusion. Symptoms are often reversible if the infusion is interrupted and/or patients receive additional treatment with an antihistaminic, acetaminophen, epinephrine or an i.v. corticosteroid. Please refer to Sections 6.1.2 and 6.1.3 for further information.

**Infection Risks**

Prolonged peripheral B-cell depletion is the expected outcome of ocrelizumab treatment. Infection is a potentially serious complication of B-cell depleting therapy and thus requires vigilant attention and prompt investigation and treatment in patients that exhibit signs of infection at any time following anti-CD20 antibody therapy.

Data on the long term risk of infection in MS patients treated with ocrelizumab are not available at this time however in review of the Phase II data in patients with RRMS no imbalance in the overall number of infections or serious infections between placebo and active ocrelizumab arms was observed at week 24. The rate of infections did not increase in ocrelizumab-treated patients at 48 weeks compared with 24 weeks. There was no trend of increase of risk of infection or serious infection for ocrelizumab treated patients with previous interferon treatment (Avonex® for 6 months). There was no trend of increase risk of infections or serious infections with high dose.

The incidence of infections and serious infections was 92.41/100 PY (95% CI 76.59, 111.5) and 3.39 /100 PY (95% CI 1.27, 9.04) in patients exposed to 600 mg dose of ocrelizumab including also patients switching from placebo or Avonex. The incidence of infections and serious infections was 95.61/100 PY (95%CI 73.23, 124.83) and 3.54/100 PY (95% CI 0.89, 14.16) in those exposed to the ocrelizumab 2 x 1000 mg dose. The most common infections in the ocrelizumab treated patients included urinary tract infections, upper respiratory infections, and nasopharyngitis.

Following an analysis of interim data through Week 72, no increase in the rate of serious or non-serious infections has occurred, from Week 24, in patients treated with ocrelizumab. To date, in study WA21493, after over 250 patient years exposure to ocrelizumab, there have been no reports of opportunistic or fatal infections.

Rarely, cases of hepatitis B reactivation, including fulminant hepatitis which have occasionally been fatal, have been reported in NHL patients receiving rituximab. A case of hepatitis B reactivation in a Hep B core Ab positive patient with RA treated with

ocrelizumab has been reported (see Section 5.4.2 for hepatitis screening and monitoring of liver function).

**Other serious, opportunistic and fatal infections have occurred in patients with lupus and RA treated with ocrelizumab in Phase III clinical trials. Data from completed studies regarding infection risks with ocrelizumab treatment in these patient populations are provided in the Investigator Brochure (IB).**

Ocrelizumab should not be administered to patients with an active infection. Physicians should exercise caution when considering the use of ocrelizumab in patients with underlying conditions that may predispose patients to serious infection. Patients who develop signs/symptoms of infection while participating in this trial should be seen immediately, samples taken for appropriate microbiological analysis and appropriate treatment instituted promptly.

Patients should be screened for tuberculosis according to national guidelines. As with other infections, patients with active tuberculosis should not be enrolled, patients with latent tuberculosis should be treated prior to enrollment.

Patients should be warned that the risk of serious infection may be increased by exposure to the medications to be used in this study and should be asked to contact the clinic staff if they start to develop signs of infection. Patients will be provided with a warning card which specifically delineates this risk, which is to be carried on their person at all times in case they are admitted to a hospital which is not participating in the study.

Please refer to the ocrelizumab IB for further information on infection risks.

**Prolonged B-cell Depletion**

In patients with RA that were treated with rituximab, prolonged peripheral B-cell depletion has been reported up to 4 years following a single course of therapy. It is not known whether this will occur following use of ocrelizumab. Patients with prolonged B-cell depletion should be monitored until their B-cells have repleted. (see Section 3.1).

**Progressive multifocal leukoencephalopathy (PML)**

To date there have been no confirmed cases of PML in any MS patient treated with either rituximab or ocrelizumab. No confirmed cases of PML have been observed in any patient receiving ocrelizumab, for any indication.

Among patients treated with rituximab, cases of PML have been observed, in oncology and other autoimmune diseases. The vast majority of these cases have occurred in patients being treated for hematological malignancy and many of these patients were also HIV positive.

There is no currently accepted screening test for PML, neither known interventions that can reliably prevent PML or adequately treat PML. See also Section 7.3.4 for more details. Guidance for diagnosis is given in Section 7.3.4.1.

**Cardiovascular Disorders**

Rarely, cardiac arrhythmias, cardiac ischemia and death due to myocardial dysfunction have been associated with rituximab administration in patients with oncologic disorders. In these cases, the presumed cause was decompensated cardiac disease as a result of cytokine release and/or infusion associated reactions. Patients with a history of cardiac disease (i.e. angina pectoris, cardiac arrhythmias, or congestive heart failure) should be monitored closely during and following infusions. It should be noted that the exclusion criteria exclude enrollment of patients with significant cardiac diseases and congestive heart failure (NYHA III or IV) – see Section 4.3.

**Immunogenicity**

Positive HAHA responses were observed and were most frequent in the lower dose groups in both RA Phase I/II studies; no HAHA responses were observed in the NHL study. In Study ACT2847g, which included doses of 10 mg × 2, 50 mg × 2, 200 mg × 2, 500 mg×2, and 1000 mg × 2, HAHA was observed in 19% and 10% of patients receiving 10 mg × 2 and 50 mg × 2, respectively, vs. 0−5% of patients receiving 200−1000 mg × 2. In Study WA18230, which included doses of 400, 1000, 1500, and 2000 mg, HAHA was observed in 10% and 5% of patients receiving 400 mg and 1000 mg, respectively, and in none of the patients receiving 1500 mg and 2000 mg.

In the RRMS Phase II study (WA21093), no new HAHA seropositivity occurred after initiation of ocrelizumab treatment (300 mg x 2 or 1000 mg x 2).

The clinical significance of positive HAHA is unknown at this time.

**Immunization**

The effect of ocrelizumab on the response to immunization is not known – please refer to Section 4.4.3 for more details; patients receiving ocrelizumab may not mount a humoral response to recall antigens during B-cell depletion. Physicians should review the patient's vaccine history, and be aware that immune response to vaccination could be reduced. Current administration of live vaccines during the Treatment Period and thereafter when B-cells remain depleted, is not allowed.

### 7.3.2 Rebif®

Patients should be informed of the risks associated with taking Rebif®. The most frequent Rebif® adverse reactions of which the patients should be made aware have been summarized in Section 1.1.5.

Depression and suicide ideation are known to occur in increased frequency in the MS and in association with interferon use. Therefore all patients should be advised to immediately report any symptoms of depression and/or suicidal ideation to investigator. Patients exhibiting depression should be monitored closely and treated appropriately. Cessation of double-blind treatment should be considered.

Injection site necrosis has been reported in patients using Rebif®. To minimize the risk of injection site necrosis patients should be advised to:

- use an aseptic injection technique,
- rotate the injection sites with each dose.

If the patient experiences any break in the skin, which may be associated with swelling or drainage of fluid from the injection site, the patient should be advised to consult with their physician before continuing injections with Rebif®/ Rebif® placebo. If the patient has multiple lesions, injections should be discontinued until healing has occurred. Patients with single lesions may continue provided that the necrosis is not too extensive.

Rebif®, like other interferons β, has a potential for causing severe liver injury including acute hepatic failure. The mechanism for the rare symptomatic hepatic dysfunction is not known. No specific risk factors have been identified. Please refer to Section 6.2.2 for additional guidelines.

### 7.3.3 Corticosteroids

Systemic corticosteroids, such as methylprednisolone, can cause immunosuppression, hypertension, diabetes mellitus, cataract, glaucoma, bruising, thinning of the skin, weight gain, psychological changes including psychosis, osteoporosis, accelerated atherosclerosis, increased risk of gastrointestinal bleeding, aseptic necrosis of bone and adrenal insufficiency. Although rare, corticosteroid induced hypersensitivity reactions may occur. They range from minor rashes to the more serious cardiovascular collapse. For additional safety data, refer to the local prescribing information.

### 7.3.4 Progressive Multifocal Leukoencephalopathy

Progressive multifocal leukoencephalopathy (PML) is a potentially fatal neurological condition linked to reactivation of a polyomavirus (JC virus) and active viral replication in the brain. Polyomavirus infection is acquired in childhood and up to 80% of adults demonstrate serological evidence of past infection. Reactivation of JC virus replication with transient viremia or viruria unassociated with clinical symptoms may occur spontaneously in healthy persons. Less frequently, central nervous system symptoms associated with active viral replication in brain tissue is observed. The clinical syndrome is significantly more frequent among immune suppressed patients.

To date there have been no confirmed cases of PML in any MS patient treated with either rituximab or ocrelizumab and no confirmed cases in any patient taking ocrelizumab for any indication. Cases of PML have been reported in patients receiving rituximab in oncology and other autoimmune indications. The vast majority of these cases have occurred in patients being treated for hematological malignancy and many of these patients were also HIV positive. The majority of these patients received rituximab in combination with chemotherapy or as part of a haematopoietic stem cell transplant.

Physicians should consider the diagnosis of PML in any patient presenting with new and/or progressive neurological deficits localized to the cerebral cortex, such as cortical symptoms/signs, behavioral and neuropsychological alteration, retrochiasmal visual

defects, hemiparesis, cerebellar symptoms/signs (e.g., gait abnormalities, limb incoordination), at each visit.

If PML is considered, a neurological consultation should be obtained and treatment suspended until PML has been ruled out. If PML is confirmed in a patient receiving ocrelizumab, no further infusions should be administered and the patient will be withdrawn from treatment (see Section 4.5). No known interventions can reliably prevent PML or adequately treat PML, if it occurs.

It is not known whether the risk of PML is altered by anti-CD20 treatment given as monotherapy. Please refer to Section 7.3.4.1 for guidance on the diagnosis of PML.

PML should be reported as an SAE (with all available information) with immediate notification of the Medical Monitor. Study drug should be withheld and patients with confirmed PML should be withdrawn from the study.

There is no known treatment or cure for PML. Treatment considerations are discussed in the medical literature [55].

#### *7.3.4.1 Guidance for Diagnosis of PML*

The following safety monitoring algorithm (Figure 3) will be implemented in this study. This algorithm was implemented in the Phase I/II studies with rituximab in patients with MS and is consistent with the algorithm used in natalizumab studies.

Comprehensive neurological assessments will be performed every 12 weeks at the regular study visits. Patients will be required to undergo a neurological exam for calculation of an Expanded Disability Status Scale (EDSS) score every 12 weeks. This requires that Functional System Score (FSS) also be determined. The examination to calculate the FSS includes cognitive, visual and motor assessments, the neurological systems most often affected by PML, as well as assessments of other neurological systems.

In the eCRF, the investigator will record the presence or absence of neurological deficits localized to the cerebral cortex (e.g., cortical symptoms/signs, behavioral and neuropsychological alteration, retrochiasmal visual defects, hemiparesis), cerebellar symptoms/signs (e.g., gait abnormalities, limb incoordination), at each visit. Presence of such neurological findings will be recorded as adverse events. *If a diagnosis for the deficits is identified, the symptoms should be replaced by the diagnosis in the adverse event eCRF.*

In addition to the neurological evaluation at regular visits, patients will undergo a telephone interview between the study visits by site personnel familiar with the patient(s). The purpose of this interview is to identify new or worsening neurological symptoms that warrant an unscheduled visit (Appendix 4). Partners or caregivers of study patients, if applicable, will be informed on symptoms and signs that may be suggestive of PML and should be instructed to contact the site, should any such signs or symptoms appear.

In the event that new or worsening neurological symptoms are considered during the telephone interview, a neurological evaluation will be conducted. Should a non MS

etiology, such as PML, be considered, further assessments should be done. The evaluation of PML may include a brain MRI scan and CSF analysis per the proposed treatment algorithm (see Figure 3).

**The following clinical guidance is provided:**

### Treatment of Relapse and Other Neurological Symptoms

- As in all MS studies, new or recurrent neurological symptoms occurring in study patients should prompt careful clinical evaluation.
- Given the occurrence of PML in immunocompromised patients who had received rituximab, PML should be considered in patients who develop worsening neurological signs or symptoms.
- There are no pathognomonic signs or symptoms that distinguish MS from PML, but there are certain clinical features that may help differentiate between the two conditions (see Table 10).
- In addition to PML and MS, other CNS conditions (e.g., stroke, migraine, etc.) should be considered when evaluating a patient with new neurological changes.
- Relapses should be managed according to the study protocol.
- Corticosteroid treatment should only be considered for cases in which PML is unlikely on clinical grounds and when the severity of the relapse warrants such treatment. Lack of response to corticosteroids should trigger further investigation.

### Action Steps if PML is Suspected

- If the clinical presentation is suggestive of PML, further investigations should include brain MRI evaluation as soon as possible. If MRI evaluation reveals lesions suspicious for PML (see Figure 3) a lumbar puncture with evaluation of the cerebrospinal fluid (CSF) for the detection of JCV DNA should be undertaken. A diagnosis of PML can potentially be made by evaluating clinical and MRI findings plus the identification of JCV in the CSF.

Please note: In the event that PML is suspected, an additional plasma, urine, as well as a CSF sample should be obtained for JCV analysis. Samples will be analyzed upon receipt and the results will be provided directly to the investigational site and to the Sponsor. Storage conditions and shipment instructions will be provided.

### MRI Assessment

- Although there are no pathognomonic findings that differentiate PML from MS, a brain MRI scan that includes fluid-attenuated inversion recovery (FLAIR) and T2-weighted and T1-weighted sequences, with and without gadolinium, should be performed to assess patients with neurological changes suggestive of PML – see Figure 3.
- Comparison with a baseline scan may assist with interpretation of the findings on the newly acquired MRI (see Table 10 for differences in lesion characteristics that may help differentiate between PML and MS).

**CSF Assessment**

- The detection of JCV DNA in the CSF of a patient with clinical and MRI features suggestive of PML establishes the diagnosis of PML.
- If JCV DNA is not detected in CSF and if clinical suspicion of PML remains high, a repeat lumbar puncture should be performed.
- If diagnosis remains uncertain and suspicion of PML remains high, a brain biopsy may be considered to establish a definitive diagnosis.

**Figure 3: Diagnostic Algorithm for PML**

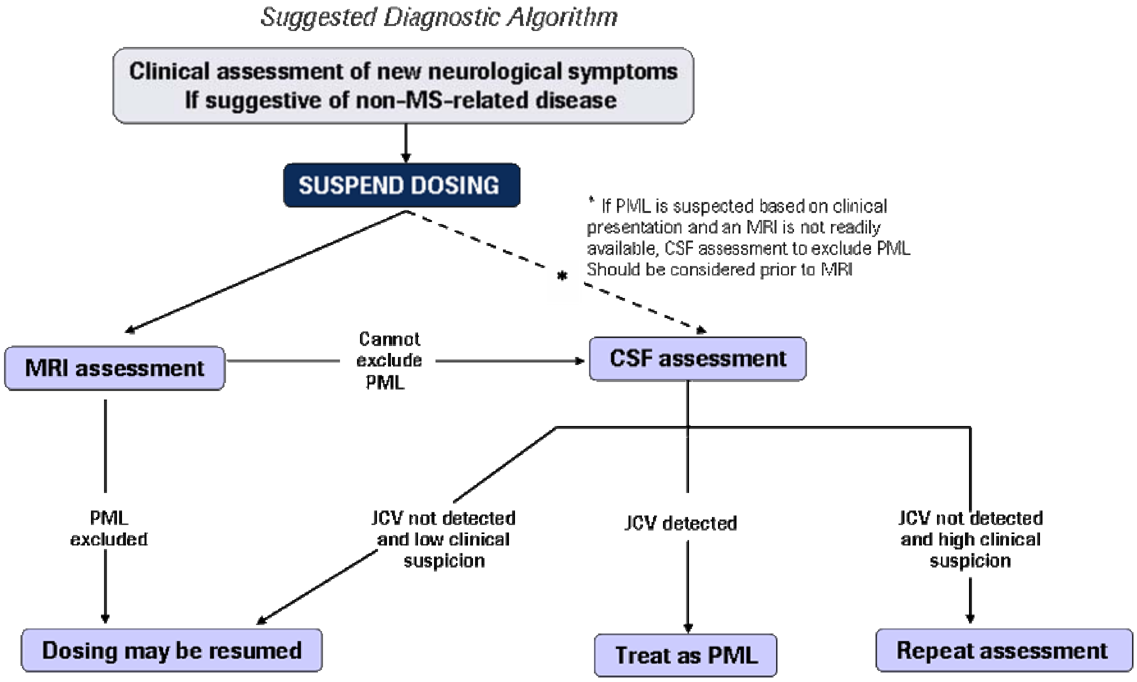


**Table 9: Clinical features to distinguish between MS relapse and PML***

| | MS relapse | PML |
|---|---|---|
| ***Onset*** | Acute | Subacute |
| ***Evolution*** | - Over hours to days<br>- Normally stabilizes<br>- Resolves spontaneously or with treatment | - Over weeks<br>- Progressive |
| ***Clinical presentation*** | - Optic neuritis<br>- Incomplete myelopathy or partial myelitis | - Cortical signs and symptoms<br>- Behavioral and neuropsychological alterations<br>- Retrochiasmal visual deficits<br>- Hemiparesis |

**Adapted from Kappos L et al [56].*

## Table 10: MRI Lesion Characteristics Typical of PML and MS

| Feature | MS (relapse) | PML |
|---|---|---|
| **Location of new lesions** | Mostly focal; affect entire brain and spinal chord, in white and possibly gray matter | Diffuse lesions, mainly subcortical and rarely periventricular, located almost exclusively in white matter, although occasional extension to gray matter has been seen; posterior fossa frequently involved (cerebellum) |
| **Borders** | Sharp edges; mostly round or finger-like in shape (especially periventricular lesions), confluent with other lesions; U-fibers may be involved | Ill-defined edges; irregular in shape; confined to white matter; sparing gray matter; pushing against the cerebral cortex; U-fibers destroyed |
| **Mode of extension** | Initially focal; lesions enlarge within days or weeks and later decrease in size within months | Lesions are diffuse and asymmetric, extending homogeneously; no confluence with other lesions; confined to white-matter tracks, sparing the cortex; continuous progression |
| **Mass effect** | Acute lesions show some mass effect | No mass effect even in large lesions (but lesion slightly abuts cerebral cortex) |
| **On T2-weighted sequence** | - Acute lesions: hyperintense center, isointense ring, discrete hyperintensity outside the ring structure<br>- Subacute and chronic lesions: hyperintense with no ring structure | Diffuse hyperintensity, slightly increased intensity of newly involved areas compared with old areas, little irregular signal intensity of lesions |
| **On T1-weighted sequence** | Acute lesions: densely hypointense (large lesions) or isointense (small lesions); increasing signal intensity over time in 80%; decreasing signal intensity (axonal loss) in about 20% | Slightly hypointense at onset, with signal intensity decreasing over time and along the affected area; no reversion of signal intensity |
| **On FLAIR sequence** | Hyperintense, sharply delineated | Hyperintensity more obvious; true extension of abnormality more clearly visible than in T2-weighted images |
| **With enhancement** | - Acute lesions: dense homogeneous enhancement, sharp edges<br>- Subacute lesions: ring enhancement<br>- Chronic lesions: no enhancement | Usually no enhancement, even in large lesions; in patients with HIV, some peripheral enhancement is possible, especially under therapy. |
| **Atrophy** | Focal atrophy possible due to focal white-matter degeneration; no progression | No focal atrophy |

*Adapted from Yousry TA et al [57].*

# 8. STATISTICAL CONSIDERATIONS AND ANALYTICAL PLAN

Full details of all statistical issues and planned statistical analyses will be specified in a separate Data Analysis Plan (DAP), which will be finalized prior to the locking and unblinding of the study database.

## 8.1 Study Endpoints

### 8.1.1 Primary Efficacy Endpoint

The primary efficacy endpoint is annualized protocol-defined relapse rate by two years (96 weeks).

Protocol-defined relapse, is defined as the occurrence of new or worsening neurological symptoms attributable to MS. Symptoms must persist for >24 hours and should not be attributable to confounding clinical factors (e.g. fever, infection, injury, adverse reactions to medications) and immediately preceded by a stable or improving neurological state for least 30 days. The new or worsening neurological symptoms must be accompanied by objective neurological worsening consistent with an increase of at least half a step on the EDSS scale, or 2 points on one of the appropriate FSS, or 1 point on two or more of the appropriate FSS. The change must affect the selected FSS (i.e., pyramidal, ambulation, cerebellar, brainstem, sensory, or visual). Episodic spasms, sexual dysfunction, fatigue, mood change or bladder or bowel urgency or incontinence will not suffice to establish a relapse. Please note: *Sexual dysfunction* and *Fatigue* will not be scored.

Adjudication of protocol-defined relapses will be performed by the Sponsor based on pre-specified criteria, applied to data collected by investigator, in a blinded fashion.

### 8.1.2 Secondary Efficacy Endpoints

The secondary efficacy endpoints are:

- The time to onset of sustained disability progression for at least 12 weeks during the 96-week comparative treatment period.
- The time to onset of sustained disability progression for at least 24 weeks during the 96-week comparative treatment period.
- The proportion of relapse-free patients by 96 weeks.
- The change in total T2 lesion volume as detected by brain MRI from baseline to Week 96.
- The total number of new, and/or enlarging T2 hyperintense lesions as detected by brain MRI at week 24, week 48 and week 96.
- The change in Multiple Sclerosis Functional Composite Scale (MSFCS) score from baseline to Week 96.
- The change in brain volume as detected by brain MRI from Week 24 to Week 96.

### 8.1.3 Exploratory Efficacy Endpoints

The exploratory efficacy endpoints in this study will include, but may not be limited to:

- The change in low contrast visual acuity from baseline to Weeks 48 and 96.
- The change in the Symbol Digit Modalities Test from baseline to Weeks 48 and 96.

- The annualized relapse rate, based on clinical and protocol-defined relapses at the end of the 96-week comparative treatment period.
- The total number of T1 gadolinium-enhanced lesions as detected by brain MRI at weeks 24, 48 and 96.
- The change in brain volume as detected by brain MRI from baseline to Week 96.
- The change in brain volume as detected by brain MRI from week 48 to Week 96.
- The change in Multiple Sclerosis Functional Composite Scale (MSFCS) score from baseline to Week 48.
- The cumulative change in EDSS scores, measured in area under the curve (AUC) by Week 96.
- The change in EDSS from baseline to Weeks 48 and 96.
- The change in timed 25 foot walk from baseline to Weeks 48 and 96.
- The change in 9-hole peg test from baseline to Weeks 48 and 96.
- The change in paced auditory serial addition test (PASAT) from baseline to Weeks 48 and 96.
- The time to onset of sustained 20% increase in 9-hole peg test for at least 12 weeks.
- The time to onset of sustained 20% increase in timed 25 foot walk for at least 12 weeks.
- Patient Reported Outcomes (PROs): Modified Fatigue Impact Scale (MFIS), EuroQol instrument (EQ-5D), Center for Epidemiology Studies Depression Scale (CES-D) and Medical Outcomes Study 36-Item Short Form Survey Instrument (SF-36) at baseline, Week 48 and Week 96.
- The change in Karnofsky Performance Status Scale (clinician-reported version) from baseline to Weeks 24, 48, 72 and 96.

### 8.1.4 Safety

Safety will be assessed through regular neurological and physical examinations, vital signs, ECG, and the occurrence of adverse events. In addition, the following will be examined:

- Non-MS pathology at all available MRI scans.
- Columbia-suicide severity rating scale (C-SSRS).
- Complete routine hematology, chemistry and urinalyses;
- Circulating B-cell subsets, T cells, natural killer cells and other leukocytes;
- Plasma immunoglobulins;
- HAHA;
- Antibody titers for mumps, rubella, varicella, and Streptococcus pneumoniae;
- Serial pregnancy tests [serum/urine β subunit human chorionic gonadotropin (β hCG)] will be performed in women of child bearing potential.
- JC virus (JCV) plasma/urine sampling – only if deemed necessary.

## 8.2 Statistical and Analytical Methods

Prior to unblinding the treatment groups, a DAP will be produced that will contain full details of all planned analyses. An outline of the planned analyses is described below. A database lock will occur when the last patient has completed his Week 96 assessment,

i.e. completed his 4th treatment cycle, for the purposes of the primary efficacy and safety analysis. The treatment assignments will be unblinded to the Sponsor at this point for the purposes of data analysis.

All analyses comparing ocrelizumab group with Rebif® group will use data collected for each patient up to and including Week 96.

The time to onset of sustained disability progression for at least 12 wks during the 96-week comparative treatment period and the time to onset of sustained disability progression for at least 24 wks during the 96-week comparative treatment period will be analyzed using pooled data across the two identical studies Sponsor plan to run as a part of Phase III program, with respect to ocrelizumab group vs. Rebif® group.

All eligible patients will be randomized to treatment stratified by region (United States versus ROW) and baseline EDSS (< 4.0 versus ≥ 4.0). All analyses will also be stratified by region (United States versus ROW) and baseline EDSS (< 4.0 versus ≥ 4.0).

All analyses, summaries and listings will be performed using SAS® software (Version 8.2 or higher in a UNIX environment).

### 8.2.1 Primary Efficacy Analysis

The primary efficacy analysis for this trial will compare annualized protocol-defined relapse rate by 96 weeks between ocrelizumab group and Rebif® group. The annualized relapse rates by 96 weeks will be calculated using negative binomial model, adjusting for region (United States versus ROW) and baseline EDSS (< 4.0 versus ≥ 4.0). The adjusted annualized relapse rates and the two-sided 95% confidence intervals for the relapse rates will be presented along with the p-value.

Other sensitivity analyses may also be performed for the primary efficacy endpoint (and documented in the DAP).

### 8.2.2 Secondary Efficacy Analyses

Secondary efficacy endpoints will be tested in hierarchal order (listed as following), all at alpha = 0.05 level. The first secondary efficacy endpoint will be tested if and only if the primary endpoint has reached the significant level at 0.05 (e.g., P-value ≤ 0.05). The rest of the secondary endpoints will be tested if and only if the secondary endpoint list ahead of it has reached the significant level at 0.05.

## Figure 4: Hierarchal Order of Key Efficacy Endpoints

1. Annualized protocol-defined relapse rate by two years (96 weeks)
2. The time to onset of sustained disability progression for at least 12 weeks during the 96-week comparative treatment period (pooled data from two studies)
3. The time to onset of sustained disability progression for at least 24 weeks during the 96-week comparative treatment period (pooled data from two studies)
4. The proportion of relapse-free patients by 96 weeks
5. The change in total T2 lesion volume as detected by brain MRI from baseline to Week 96
6. The total number of new, and/or enlarging T2 hyperintense lesions as detected by brain MRI at Week 24, Week 48, and Week 96
7. The change in Multiple Sclerosis Functional Composite Scale (MSFCS) score from baseline to Week 96
8. The change in brain volume as detected by brain MRI from Week 24 to Week 96

### *8.2.2.1 The Time to Onset of Sustained Disability Progression for At Least 12 Weeks During the 96-Week Comparative Treatment Period*

Time to sustained disability progression (12 week confirmation) is defined as the time from Baseline to the first disability progression, which is confirmed at the next regularly scheduled visit ≥ 84 days after the initial disability progression. Disability progression is defined as an increase of ≥ 1.0 point from baseline EDSS, if the baseline EDSS is between 0 and 5.5 points (inclusive), or an increase of ≥ 0.5 points, if the baseline EDSS is > 5.5 points. Please note that the inclusion criteria of EDSS (0–5.5) only applies to screening EDSS. It is still possible that a patient's baseline EDSS (derived based on both screening and day 1 EDSS results) is >5.5. The non-confirmatory EDSS assessments (if any) between the initial and confirmation of disability progression should be at least as high as the minimum change required for progression. Patients who did not have sustained disability progression by Week 96 visit, time of early discontinuation of treatment, or loss to follow up will be censored at the date of their last EDSS assessment.

Data from the two studies with respect to ocrelizumab group vs Rebif® group will be pooled for analysis of this endpoint. To assess the validity of pooling data across the two RMS studies, demographic and baseline characteristics will be compared by trials. The treatment effect (hazard ratio and CI) for sustained disability progression within each trial

will be compared between the two trials. In interpreting the trial comparisons with a view toward assessing the validity of the pooled dataset, the primary interest is in confirming that the treatment effect is qualitatively similar across the two studies – positive treatment effects (the estimated hazard to have sustained disease progression in patients treated with OCR is numerically smaller than that in patients treated with Rebif) are shown in both studies. If the results from the two studies are not qualitatively similar (e.g., positive treatment effect is only shown in one study), data will not be pooled.

Time to sustained disability progression for ocrelizumab group and Rebif® group (across the studies) will be compared using a two-sided log-rank test stratifying by region (United States versus ROW), baseline EDSS (< 4.0 versus ≥ 4.0), and study. The proportion of patients with sustained disability progression will be estimated using Kaplan-Meier methodology. The overall hazard ratio will be estimated using a stratified Cox regression model with the same stratification factors used in the stratified log-rank test above.

#### 8.2.2.2 The Time to Onset of Sustained Disability Progression for At Least 24 Weeks During the 96-Week Comparative Treatment Period

Time to sustained disability progression between ocrelizumab group and Rebif® group using a 24 week confirmation window for disability progression will be compared using the same analysis method for time to sustained disability progression using a 12 week confirmation window. Time to sustained disability progression (24 week confirmation) is defined as the time from Baseline (Day 1) to the first disability progression, which is confirmed at the next regularly scheduled visit ≥ 161 days after the initial disability progression.

#### 8.2.2.3 The Proportion of Relapse-Free Patients by 96 Weeks

The proportion of patients who remain relapse-free by Week 96 will be analyzed using a Cochran-Mantel-Haenszel $\chi 2$ test stratified by region (United States versus ROW) and baseline EDSS (< 4.0 versus ≥ 4.0) to compare ocrelizumab group to Rebif®. The difference in the proportions, along with the 95% confidence interval for the difference will be presented. Relative risk (of having relapse) will also be produced for ocrelizumab group compared to Rebif®, along with corresponding confidence intervals.

#### 8.2.2.4 The Change in Total T2 Lesion Volume as Detected by Brain MRI from Baseline to Week 96

The change in total volume of T2 lesions on MRI scans of the brain from baseline to Week 96 will be compared between ocrelizumab group and Rebif® group using the ranked ANCOVA[1] adjusting baseline T2 lesion volume, baseline Gd lesion (present or not), region (United States versus ROW) and baseline EDSS (< 4.0 versus ≥ 4.0). The median change in total volume of T2 lesions for each treatment group and the corresponding 95% confidence interval for the median will be presented.

---

[1] Categorical Data Analysis using the SAS system. Stokes, Davis, and Koch

#### *8.2.2.5 The Total Number of New, and/or Enlarging T2 Hyperintense Lesions as Detected by Brain MRI at Week 24, Week 48 and Week 96.*

Negative binomial model adjusting baseline Gd lesion (present or not), region (United States versus ROW) and baseline EDSS (< 4.0 versus ≥ 4.0) will be applied to compare the difference between ocrelizumab and Rebif® groups. The median change in the total number of new, and/or enlaring T2 hyperintense lesions for each treatment group and the corresponding 95% confidence interval for the median will be presented.

#### *8.2.2.6 The Change in Multiple Sclerosis Functional Composite Scale (MSFCS) Score from Baseline to Weeks 96*

The change in MSFCS from baseline to Week 96 will be compared between ocrelizumab group and Rebif® group using ranked ANCOVA adjusting baseline MSFCS, region (United States versus ROW) and baseline EDSS (< 4.0 versus ≥ 4.0). The median change in MSFCS for each treatment group and the corresponding 95% confidence interval for the median will be presented.

#### *8.2.2.7 The Change in Brain Volume as Detected by Brain MRI Scan from Week 24 to Week 96*

The change in brain volume as detected by brain MRI from week 24 to Week 96 will be compared between ocrelizumab group and Rebif® group using the ranked ANCOVA adjusting baseline brain volume, baseline Gd lesion (present or not), region (United States versus ROW) and baseline EDSS (< 4.0 versus ≥ 4.0). The median change in brain volume for each treatment group and the corresponding 95% confidence interval for the median will be presented.

### 8.2.3 Exploratory Analyses

The exploratory endpoints will be summarized using tables, listings and graphs, where appropriate. Full details of the derivations and analyses of all exploratory endpoints will be provided in the DAP.

### 8.2.4 Sample Size

The sample size for this study has been estimated based on data from previous RRMS trials, with the use of two-sided tests with an experiment-wise alpha of 0.05. The annualized rate of relapse among patients receiving ocrelizumab at 96 weeks is predicted to be 0.165 (standard deviation of approximately 0.60), as compared with 0.33 (standard deviation of approximately 0.80) among patients receiving the control treatment, Rebif® (this represents a relative reduction of 50% on ocrelizumab compared to the active comparator). For the annualized relapse rate, a t-test has been used to determine the sample size between ocrelizumab and the control arm. The sample size of 400 patients per arm provides 84 percent power, maintaining the type I error rate of 0.05, and assuming a drop out rate of 20 percent approximately (assuming relative reduction among patients drop out is 25%).

For sustained disease progression, a two group test of equal exponential survival with exponential dropout is used to determine the sample size. Assuming the 2 year sustained disability progression rate is 18% for the Rebif® arm and 12.6% for the ocrelizumab arm

(this represents a relative reduction of 30% on ocrelizumab compared to the active comparator), and assuming a drop out rate of 20 percent over 2 years approximately, the sample size of 400 per arm will provide 80 percent power, maintaining the type I error rate of 0.05 based on the pooled analysis of two RMS trials (800 patients treated with ocrelizumab 600 mg and 800 patients treated with Rebif®).

### 8.2.5 Hypothesis Testing

The hypotheses to be tested are:

$H_0$ (null hypothesis): There is no statistically significant difference in annualized protocol-defined relapse rate at two years between ocrelizumab group and Rebif® group.

$H_1$ (alternative hypothesis): There is a statistically significant difference in annualized protocol-defined relapse rate at two years between ocrelizumab group and Rebif® group.

Annualized protocol-defined relapse rate at two years between the ocrelizumab group and Rebif® group will be compared using negative binomial model adjusting region (United States versus ROW) and baseline EDSS (< 4.0 versus ≥ 4.0). If the test result for comparing 600 mg ocrelizumab and Rebif® groups is statistically significant at $\alpha < 0.05$ level (two-sided test), we will conclude that the 600 mg ocrelizumab group demonstrated a superior effect of reducing Annualized protocol defined relapse rate, when compared to Rebif® group.

Similar hypotheses will also be tested for the secondary efficacy parameters. Methods for handling multiplicity issues related to secondary endpoints will be described in the DAP.

### 8.2.6 Analysis Populations

One patient population will be defined for the purpose of the safety analysis and two for the efficacy analysis. All efficacy analyses will be performed using the intent-to-treat (ITT) population. The per-protocol (PP) population will be used for all primary and secondary efficacy analyses in order to evaluate the influence of major protocol violators and as a sensitivity check to the ITT analysis.

#### *8.2.6.1 Safety Population*

This population will be used for all summaries of safety data. The safety population will include all patients who received any study drug and provided at least one assessment of safety. Randomized patients that receive incorrect therapy from that intended will be summarized in the group according to the therapy actually received. Patients who are not randomized, but who receive study drug will be included in the safety population and summarized according to the therapy actually received.

#### *8.2.6.2 Intent-to-Treat Population*

All randomized patients who have received any study drug will be included in the intent–to-treat population. Patients who prematurely withdraw from the study for any reason and for whom an assessment is not performed for whatever reason will still be included in the ITT analysis. Patients who receive an incorrect therapy from that which is intended will be summarized according to their randomized treatment.

#### *8.2.6.3 Per Protocol Population*

The per protocol population will include all patients in the ITT population adhering to the protocol. Patients may be excluded if they significantly violate the inclusion/exclusion criteria or deviate from the study plan. Specific reasons for warranting exclusion will be agreed and documented in the DAP prior to unblinding of the treatment groups. Only those patients with violations that are deemed to potentially affect the efficacy of study treatment will be excluded from the per protocol population. Patients who receive an incorrect therapy from that intended will be excluded from the per protocol population.

### 8.2.7 Interim Analysis

No formal efficacy interim analyses are planned.

### 8.2.8 Safety Data Analysis

The safety population will include all patients who received any study drug and provided at least one post-dose safety assessment. All safety parameters will be summarized and presented in tables based on this safety population. Randomized patients that receive incorrect therapy from that intended will be summarized in the group according to the therapy actually received. Patients who are not randomized, but who receive study drug will be included in the safety population and summarized according to the therapy actually received.

The safety data will be listed and summarized at determined cut off points, e.g. using data for each patient up to Week 96, using all available data at the Week 96 database lock for the primary analysis.

All adverse events will be coded and tabulated by system organ class and preferred term for individual events within each system organ class, and will be presented in descending frequency. Adverse events will also be tabulated by severity and relationship to the study medication. Serious adverse events will be summarized separately. Results of C-SSRS will be listed for each visit by patient.

Non-MS pathology reported by local safety radiologist will be summarized by treatment group.

Associated laboratory parameters such as hepatic function, renal function and hematology values will be grouped and presented together. Correlation between low IgG and IgM and infections will be presented separately. Marked abnormalities will also be flagged. Marked abnormalities will be tabulated for each laboratory test by treatment group.

Analysis of HAHA to ocrelizumab will be summarized graphically and descriptively. Correlation between presence of HAHA and IRR/B-cell depletion will be presented descriptively.

The results of vital sign, physical examination and ECG will be included in individual patient listings. Change from baseline in vital signs will be summarized by groups.

An external, independent DMC will review safety data throughout the study and will convene at least three times per year. Analyses required for the DMC data review will be performed as described in the DMC Charter and DMC data handling plan.

#### 8.2.9 Safety Follow-up Period

Data from this period will be analyzed to provide information on the maintenance effect and the potential withdrawal effect of ocrelizumab. In addition, data will be analyzed to provide information concerning the long-term safety of ocrelizumab. Data will be summarized and tables and listings will be produced.

#### 8.2.10 Other Analyses

##### *8.2.10.1 Pharmacokinetic Analysis*

**Pharmacokinetic Parameters**

Ocrelizumab serum concentration-time data will be modeled using a population approach. The primary population PK parameters (Clearances and Volumes) for ocrelizumab will be estimated by means of NONMEM analysis of the sparse PK data. Clearances with associated inter-patient variability may be characterized by a saturable and non-saturable clearance as well as an intercompartmental clearance depending on the final structural model. Volumes with associated inter-patient variability may be characterized by central and peripheral volumes depending on the final structural model. Exposure (AUC) to ocrelizumab will be estimated. The selection of other parameters will depend on the final PK model used for this analysis.

**Pharmacokinetic Analysis**

Nonlinear mixed effects modeling (with software NONMEM [58]) will be used to analyze the sparse sampling dose-concentration-time data of ocrelizumab. Patients who have measurable concentrations of ocrelizumab will be included in the PK analysis unless major protocol deviations or unavailability of information (e.g. exact blood sampling time) occurred which may interfere with PK evaluation. The PK data of this study may be pooled with more extensive data from other studies. Population PK parameters (Clearances and Volumes) will be estimated and the influence of covariates, such as age, gender, weight, HAHA, and baseline CD19 lymphocytes, on these parameters will be investigated.

Details of the mixed-effects modeling analyses will be described in a Modeling and Simulation Analysis Plan and results will be reported separately.

##### *8.2.10.2 Pharmacodynamic Analysis*

The relationship between individual ocrelizumab exposure and selected safety and efficacy parameters will be analyzed and explored, in order to characterize the exposure/dose response curve of ocrelizumab. This may include but is not limited to annualized relapse rate, T1 and T2 lesions at week 96, IRRs, infections, and other AEs or safety parameters of interest. Other exploratory analyses may be performed to assess the possible relationship between PD markers e.g. CD19 count, PK, and clinical response.

#### *8.2.10.3 Roche Clinical Repository / Protein Biomarker Samples*

Additional blood samples for serum and/or plasma analyses will be taken for research purposes subject to discretionary approval from each center's IRB/IEC and the patient's specific written consent. These samples will be used to identify dynamic biomarkers to help us better understand the pathogenesis of RMS and response to treatment with ocrelizumab. Such future biomarkers have yet to be determined but may include circulating biochemical markers in blood including cytokines as well as peripheral blood gene expression patterns. Exploratory statistical data analyses may include assessments for possible relationships between these biomarker levels, PK and clinical response.

# 9. DATA COLLECTION, MANAGEMENT AND QUALITY ASSURANCE

The overall procedures for quality assurance of clinical study data are described in the Sponsor's (or designee) Standard Operational Procedures.

Data for this study will be recorded via an Electronic Data Capture (EDC) system using electronic Case Report Forms. It will be transcribed by the site from the paper source documents onto the eCRF. In addition, EDSS, MSFC, C-SSRS, Karnofsky Performance Status Scale and patient reported outcomes will be collected via an electronic interface. The data will be transmitted from the electronic interface to a central database that will later be transferred to the Sponsor (or designee). Only if a technical failure prevents the ability to collect data electronically, the paper forms may be used.

Accurate and reliable data collection will be assured by verification and cross–check of the eCRFs against the investigator's records by the study monitor (source document verification), and the maintenance of a drug–dispensing log by the investigator.

A comprehensive validation check program utilizing front-end checks in the eCRF/electronic interface and back-end checks in the data base will verify the data and discrepancies will be generated accordingly. These are transferred electronically to the site for resolution by the investigator.

Throughout the study the Study Management Team (SMT) will review data according to the EDC Cleaning Process as described in the Data Management Plan.

## 9.1 Assignment of Preferred Terms and Original Terminology

For classification purposes, preferred terms will be assigned by the Sponsor to the original terms entered on the eCRF, using the most up-to-date version of the Medical Dictionary for Regulatory Activities (MedDRA) terminology for adverse events and diseases and the International Non-proprietary Name (INN) Drug Terms and Procedures Dictionary for treatments and surgical and medical procedures.

# 10. STUDY COMMITTEES

**Steering Committee**

An external Steering Committee will provide general guidance, assist with liaison to investigators and oversee any external communication of the results of the study.

**Data Monitoring Committee (DMC)**

An external independent Data Monitoring Committee (DMC) will be chartered to review safety data throughout the study and make recommendations regarding continuation, termination, or modification of the study. Regularly scheduled safety data reviews will occur at least three times per year after the first patient is enrolled.

Any safety event that requires unblinding of study treatment allocation will be immediately reported to the DMC and to the health authorities in an expedited safety report. The DMC may request and review any additional reports outside of the planned analyses at any time if deemed necessary to ensure the safety of patients. The safety evaluations will be conducted on parameters specified within the DMC charter and may vary depending on the requirements and requests of the DMC.

The details of the DMC roles and responsibilities, scope of work and the logistics of the DMC activities will be outlined in a DMC Charter. The purpose of the DMC interim analyses is primarily safety evaluation, and the study may be stopped or amended because of significant safety concerns.

# 11. REFERENCES

1. Neurological Disorders: Public health Challenges WHO, WHO Press, 1211 Geneva 27, Switzerland, assessed on line on July 6, 2010. http://www.who.int/mental_health/neurology/chapter_3_a_neuro_disorders_public_h_challenges.pdf

2. McDonald WI, Compston A, Edan G, et al. Recommended diagnostic criteria for multiple sclerosis: guidelines from the International Panel on the Diagnosis of Multiple Sclerosis. Ann Neurol 2001;50:121–7.

3. Polman CH, Reingold SC, Banwell B, et al. Diagnostic criteria for multiple sclerosis: 2010 revisions to the "McDonald Criteria". Ann Neurol 2011;69:292–302.

4. Lublin FD and Reingold SC. Defining the clinical course of multiple sclerosis: results of an international survey. National Multiple Sclerosis Society (USA) Advisory Committee on Clinical Trials of New Agents in Multiple Sclerosis. Neurology 1996;46:907–11.

5. Filippini G, Munari L, Incorvaia B, Ebers GC, Polman C, D'Amico R, et al. Interferons in relapsing remitting multiple sclerosis: a systematic review. Lancet 2003;361:545–52.

6. Compston A, Coles A. Multiple sclerosis. Lancet 2008; 372:1502-17.

7. Hauser SL, Waubant E, Arnold DL, et al; HERMES Trial Group. B-cell depletion with rituximab in relapsing-remitting multiple sclerosis. N Engl J Med 2008;358:676–88.

8. Sidén A. Isoelectric focusing and crossed immunoelectrofocusing of CSF immunoglobulins in MS. J Neurol 1979;221:39–51.

9. Meinl E, Krumbholz M, Hohlfeld R. B lineage cells in the inflammatory central nervous system environment: migration, maintenance, local antibody production, and therapeutic modulation. Ann Neurol 2006;59:880–92.

10. Franciotta D, Salvetti M, Lolli F, Serafini B, Aloisi F. B cells and multiple sclerosis. Lancet Neurol. 2008;7:852-8.

11. McFarland HF. The B cell--old player, new position on the team. N Engl J Med 2008;358:664–5.

12. Owens GP, Kraus H, Burgoon MP, et al. Restricted use of VH4 germline segments in an acute multiple sclerosis brain. Ann Neurol 1998;43:236–43.

13. Baranzini SE, Jeong MC, Butunoi C, Murray RS, Bernard CC, Oksenberg JR. B cell repertoire diversity and clonal expansion in multiple sclerosis brain lesions. J Immunol. 1999;163:5133-44.

14. Qin Y, Duquette P, Zhang Y, et al. Clonal expansion and somatic hypermutation of V(H) genes of B cells from cerebrospinal fluid in multiple sclerosis. J Clin Invest 1998;102:1045–50.

15. Colombo M, Dono M, Gazzola P, et al. Accumulation of clonally related B lymphocytes in the cerebrospinal fluid of multiple sclerosis patients. J Immunol. 2000;164:2782-9.

16. Ritchie AM, Gilden DH, Williamson RA, et al. Comparative analysis of the CD19+ and CD138+ cell antibody repertoires in the cerebrospinal fluid of patients with multiple sclerosis. J Immunol 2004;173:649–56.

17. Lambracht-Washington D, O'Connor KC, et al. Antigen specificity of clonally expanded and receptor edited cerebrospinal fluid B cells from patients with relapsing remitting MS. J Neuroimmunol 2007;186:164–76.

18. Owens GP, Winges KM, Ritchie AM, et al. VH4 gene segments dominate the intrathecal humoral immune response in multiple sclerosis. J Immunol 2007;179:6343–51.

19. Monson NL, Brezinschek HP, Brezinschek RI, et al. Receptor revision and atypical mutational characteristics in clonally expanded B cells from the cerebrospinal fluid of recently diagnosed multiple sclerosis patients. J Neuroimmunol 2005;158:170–81.

20. Obermeier B, Mentele R, Malotka J, et al. Matching of oligoclonal immunoglobulin transcriptomes and proteomes of cerebrospinal fluid in multiple sclerosis. Nat Med 2008;14:688–93.

21. Reindl M, Linington C, Brehm U, et al. Antibodies against the myelin oligodendrocyte glycoprotein and the myelin basic protein in multiple sclerosis and other neurological diseases: a comparative study. Brain 1999;122:2047–56.

22. Egg R, Reindl M, Deisenhammer F, et al. Anti-MOG and anti-MBP antibody subclasses in multiple sclerosis. Mult Scler 2001;7:285–9.

23. Andersson M, Yu M, Söderström M, et al. Multiple MAG peptides are recognized by circulating T and B lymphocytes in polyneuropathy and multiple sclerosis. Eur J Neurol 2002;9:243–51.

24. Genain CP, Cannella B, Hauser SL, et al. Identification of autoantibodies associated with myelin damage in multiple sclerosis. Nat Med 1999;5:170–5.

25. Serafini B, Rosicarelli B, Magliozzi R, et al. Detection of ectopic B-cell follicles with germinal centers in the meninges of patients with secondary progressive multiple sclerosis. Brain Pathol 2004;14:164–74.

26. Magliozzi R, Howell O, Vora A, et al. Meningeal B-cell follicles in secondary progressive multiple sclerosis associate with early onset of disease and severe cortical pathology. Brain 2007;130:1089−104.

27. Kutzelnigg A, Faber-Rod JC, Bauer J, et al. Widespread demyelination in the cerebellar cortex in multiple sclerosis. Brain Pathol 2007;17:38–44.

28. Aloisi F and Pujol-Borrell R. Lymphoid neogenesis in chronic inflammatory diseases. Nat Rev Immunol 2006;6:205−17.

29. Howell OW, Reeves C, Magliozzi R, et al. The incidence of meningeal B-cell follicles in secondary progressive multiple sclerosis: a neuropathological study of 96 cases (abstract). Mult Scler 2009;15:S5.

30. Bar-Or A, Calabresi PA, Arnold D, et al. Rituximab in relapsing-remitting multiple sclerosis: a 72-week, open-label, phase I trial. Ann Neurol 2008;63:395400.

31. Hawker K, O'Connor P, Freedman M, et al. Rituximab in patients with primary progressive multiple sclerosis: results of a randomized double-blind placebo-controlled multicenter trial. Ann Neurol. 2009;66:460-71.

32. Panitch H, Goodin DS, Francis G, Chang P, Coyle PK, O'Connor P, Monaghan E, Li D, Weinshenker B; EVIDENCE Study Group. Evidence of Interferon Dose-response: European North American Comparative Efficacy; University of British Columbia MS/MRI Research Group. Randomized, comparative study of interferon beta-1a treatment regimens in MS: The EVIDENCE Trial. Neurology. 2002 Nov 26;59(10):1496-506.

33. PRISMS Study Group. Randomized double-blind placebo controlled study of interferon- 1a in relapsing/remitting multiple sclerosis. Lancet 1998;352:1498–1504.

34. PRISMS Study Group, University of British Columbia MS/MRI Analysis Group. PRISMS-4: long-term efficacy of interferon- -1a in relapsing MS. Neurology 2001;56:1628–1636.

35. Secondary Progressive Efficacy Clinical Trial of Recombinant Interferon-Beta-1a in MS (SPECTRIMS) Study Group. Randomized controlled trial of interferon- beta-1a in secondary progressive MS: Clinical results. Neurology. 2001 Jun 12;56(11):1496-504.

36. European Public Assessments reports, Rebif Summary of product characteristic, January 27, 2010.

37. REBIF U.S. Physician Prescribing Information, Revised: July 2009.

38. Lim SY, Constantinescu CS. Current and future disease-modifying therapies in multiple sclerosis. Int J Clin Pract. 2010 Apr;64(5):637-50.

39. Polman C et al; Recommendations for clinical use of data on neutralizing antibodies to interferon-beta therapy in multiple sclerosis. Lancet Neurol 2010; 9: 740–50.

40. Polman CH, Reingold SC, Barkhof F, et al. Ethics of placebo-controlled clinical trials in multiple sclerosis: a reassessment. Neurology 2008;70:1134–40.

41. Kap YS, van Driel N, Blezer E, et al. Late B Cell Depletion with a Human Anti-Human CD20 IgG1 κ Monoclonal Antibody halts the Development of Experimental Autoimmune Encephalomyelitis in Marmosets. J Immunol 2010; 185; 3990-4003.

42. Gong Q, Ou Q, Ye S, et al. Importance of Cellular Microenvironment and Circulatory Dynamics in B Cell Immunotherapy. J. Immunol. 2005;174;817-826.

43. Ahuja A, Shupe J, Dunn R, et al. Depletion of B Cells in Murine Lupus: Efficacy and Resistance. The Journal of Immunology, 2007, 179: 3351–3361.

44. Gelinck LBS, et al. Poor serological responses upon influenza vaccination in patients with rheumatoid arthritis treated with rituximab. Ann Rheum Dis 2007; 66:1402-1403.

45. van Assen S, Holvast A, Benne CA, et al. Humoral responses after influenza vaccination are severely reduced in patients with rheumatoid arthritis treated with Rituximab. Arthritis & Rheumatism 2010; 62:75-81.

46. Oren S. Vaccination against influenza in rheumatoid arthritis patients: the effect of rituximab on the humoral response. Ann Rheum Dis published online November 2, 2007.

47. Bingham C, Looney R, Deodhar A, et al. Results from a controlled clinical trial (SIERRA) to evaluate primary and recall responses to immunizations in RA patients treated with rituximab. Arthritis Rheum 2008; 58:900-901[abstract#1999]. http://acr.confex.com/acr/2008/webprogram/Paper3941.html assessed on December 17, 2009

48. Cohen JA, Fischer JS, Bolibrush DM, et al. Intrarater and interrater reliability of the MS functional composite outcome measure. Neurology. 2000;54:802-6.

49. Fischer JS, Rudick RA, Cutter GR, Reingold SC. The Multiple Sclerosis Functional Composite Measure (MSFC): an integrated approach to MS clinical outcome assessment. National MS Society Clinical Outcomes Assessment Task Force. Mult Scler. 1999;5:244-50.

50. Ingram G, Hakobyan S, Hirst CL, et al. Complement regulator factor H as a serum biomarker of multiple sclerosis disease state. Brain 2010;133:1602-11.

51. Guidance for Industry: Patient-reported outcome measures: Use in medical product development to support labeling claims, FDA 2009, accessed at http://www.fda.gov/downloads/Drugs/GuidanceComplianceRegulatoryInformation/Guidances/UCM193282.pdf

52. Ware JE et al., SF-36 Health Survey: Manual & Interpretation Guide, Lincoln, RI, QualityMetric Incorporated.

53. Fairclough DL, Design and analysis of quality of life studies in clinical trials, 2010, Boca Raton, FL, CRC Press, 2010.

54. Emery P, Fleischmann R, Filipowicz-Sosnowska A, et al. The efficacy and safety of rituximab in patients with active rheumatoid arthritis despite methotrexate treatment: results of a phase IIB randomized, double-blind, placebo-controlled, dose-ranging trial. Arthritis Rheum. 2006;54:1390-400.

55. Calabrese LH, Molloy ES, Huang D, Ransohoff RM. Progressive multifocal leukoencephalopathy in rheumatic diseases: evolving clinical and pathologic patterns of disease. Arthritis Rheum. 2007;56:2116-28.

56. Kappos L, Bates D, Hartung HP, et al. Natalizumab treatment for multiple sclerosis: recommendations for patient selection and monitoring. Lancet Neurol. 2007;6:431–441.

57. Yousry TA, Major EO, Ryschkewitsch C, et al. Evaluation of patients treated with natalizumab for progressive multifocal leukoencephalopathy. N Engl J Med. 2006;354:924-33.

58. Beal SL., Boeckman AJ., Sheiner LB. NONMEM User's Guide, Parts I-VIII San Francisco: Division of Clinical Pharmacology- University of California, 1992.

# PART II: ETHICS AND GENERAL STUDY ADMINISTRATION

## 12. ETHICAL ASPECTS

### 12.1 Local Regulations/Declaration of Helsinki

The investigator will ensure that this study is conducted in full conformance with the principles of the "Declaration of Helsinki" or with the laws and regulations of the country in which the research is conducted, whichever affords the greater protection to the individual. The study must fully adhere to the principles outlined in "Guideline for Good Clinical Practice" ICH Tripartite Guideline or with local law if it affords greater protection to the patient. For studies conducted in the EU/EEA countries, the investigator will ensure compliance with the EU Clinical Trial Directive [2001/20/EC]. For studies conducted in the USA or under US IND, the investigator will additionally ensure adherence to the basic principles of "Good Clinical Practice" as outlined in the current version of 21 CFR, subchapter D, part 312, "Responsibilities of Sponsors and Investigators", part 50, "Protection of Human Subjects", and part 56, "Institutional Review Boards".

In other countries where a "Guideline for Good Clinical Practice" exists, Roche and the investigators will strictly ensure adherence to the stated provisions.

### 12.2 Informed Consent

#### 12.2.1 Main Study Informed Consent

**It is the responsibility of the investigator, or a person designated by the investigator [if acceptable by local regulations], to obtain signed informed consent from each patient prior to participating in this study after adequate explanation of the aims, methods, anticipated benefits, and potential hazards of the study**.

The investigator or designee must also explain that the patients are completely free to refuse to enter the study or to withdraw from it at any time, for any reason.

The electronic Case Report Forms for this study contain a section for documenting patient informed consent, and this must be completed appropriately. If new safety information results in significant changes in the risk/benefit assessment, the consent form should be reviewed and updated if necessary. All patients (including those already being treated) should be informed of the new information, given a copy of the revised form and give their consent to continue in the study.

For the patient not qualified or incapable of giving legal consent, written consent must be obtained from the legally acceptable representative. In the case where both the patient and his/her legally acceptable representative are unable to read, an impartial witness should be present during the entire informed consent discussion. After the patient and representative have orally consented to participation in the trial, the witness' signature on the form will attest that the information in the consent form was accurately explained and understood.

**For U.S.-IND studies:** In a life-threatening situation where a patient is unconscious or otherwise unable to communicate, the emergency is such that there is not enough time to obtain consent from the patient's legally acceptable representative, and there is no other or better treatment available, it is permissible to treat the patient under protocol with consent of both the investigator and another physician not involved in the study, with appropriate documentation submitted to the IRB within 5 days. If this collaboration is not immediately possible, there must be a written evaluation by a physician independent of the study and the appropriate documentation be submitted to the IRB within 5 days of treating the patient. In addition, the patient or his/her legally acceptable representative should be informed about the trial as soon as possible and consent to continue, giving written consent as described above.

**For non-U.S.-IND studies:** In a life-threatening situation where a patient is unconscious or otherwise unable to communicate, the emergency is such that there is not enough time to obtain consent from the patient's legally acceptable representative, and there is no other or better treatment available, it is permissible to treat the patient under protocol with consent of the investigator, with appropriate documentation that the IEC had approved the procedures used to enroll patients in such situations. In addition, the patient or his/her legally acceptable representative should be informed about the trial as soon as possible and consent to continue, giving written consent as described above.

### 12.2.2 RCR Informed Consent

It is the responsibility of the investigator, or a person designated by the investigator (if acceptable under local regulations), to obtain written informed consent from each individual who has consented to RCR sampling after adequate explanation of the aims, methods, objectives and potential hazards. Subjects must receive an explanation that they are completely free to refuse to provide the RCR specimen(s) and may withdraw his/ her sample at any time and for any reason during the study or 15 year storage period of the specimen(s). The Informed Consent for an **optional** specimen donation will be incorporated as a specific section into the main Clinical Trial Informed Consent Form (ICF). A second, separate, specific signature consenting to specimen donation will be required to document the study participant’s agreement to provide an **optional** specimen; if the participant declines, he/ she will check a “no” box in the appropriate section and not provide a second signature.

The patient does not have to provide a separate consent for protein biomarker RCR sampling.

The eCRF for the associated clinical study contains a page for documenting patient informed consent to the RCR, and this must be completed appropriately.

### 12.2.3 Death or Loss of Competence of Participant who has donated a specimen(s) that is stored in the RCR

In case the Informed Consent Form and/or the Study Protocol do not provide any specific provisions for death or loss of competence, specimen and data will continue to be used as part of RCR research.

In the event of the death of a participant of a Roche Clinical Trial or Experimental Medicine Research study or if a participant is legally incompetent at the time of the specimen and data procurement, or becomes legally incompetent thereafter, applicable provisions as stated for such situations in the respective Informed Consent Form and/or the Study Protocol shall become effective and be followed accordingly.

Additional procurement of assent from legally incompetent persons and minors shall take place according to local laws and international best practice, as it applies to the specific case.

## 12.3 Independent Ethics Committees (IEC) and Institutional Review Board (IRB)

The protocol, informed consent and any accompanying material provided to the patient in the U.S. will be submitted by the investigator to an IRB for review. For EEA member states, the Sponsor will submit to the Competent Authority and IEC, the protocol and any accompanying material provided to the patient. In both the US and EEA member states, the accompanying material may include patient information sheets, descriptions of the study used to obtain informed consent and terms of any compensation given to the patient as well as advertisements for the trial.

An approval letter or certificate (specifying the protocol number and title) from the IEC/IRB must be obtained before study initiation by the investigator specifying the date on which the committee met and granted the approval. This applies whenever subsequent amendments/modifications are made to the protocol.

Any modifications made to the protocol, informed consent or material provided to the patient after receipt of the IEC/IRB approval must also be submitted by the investigator in the U.S. and by the Sponsor in the EEA member states in accordance with local procedures and regulatory requirements.

When no local review board exists, the investigator is expected to submit the protocol to a regional committee. If no regional committee exists, Roche will assist the investigator in submitting the protocol to the European Ethics Review Committee.

Sampling for the RCR is contingent on review and approval for the exploratory biomarker assessments and written informed consent by an appropriate regulatory body (depending on the country where the study is performed) and a site's Institutional Review Board (IRB) / Ethics Committee (EC). If a regulatory or site's IRB/EC does not approve the sampling for the exploratory assessments the section on biomarker sampling will not be applicable.

Roche shall also submit an Annual Safety Report once a year to the IEC and Competent Authorities (CAs) according to local regulatory requirements and timelines of each country participating in the study. In the U.S. Roche submits an IND Annual Report to the FDA according to local regulatory requirements and timelines.

## 12.4 Role of the Science and Ethics Advisory Group (SEAG)

A Science and Ethics Advisory Group consisting of experts in the fields of biology, ethics, sociology and law will advise Roche regarding the use of specimens stored in the RCR and on the scientific and ethical aspects of handling genetic information. The SEAG is independent of Roche.

# 13. CONDITIONS FOR MODIFYING THE PROTOCOL

Requests from investigators to modify the protocol to ongoing studies will be considered only by consultation between an appropriate representative of the Sponsor and the investigator [investigator representative(s) in the case of a multicenter trial]. Protocol modifications must be prepared by a representative of the Sponsor and initially reviewed and approved by the Clinical Science Leader and Biostatistician.

All protocol modifications must be submitted to the appropriate Independent Ethics Committee or Institutional Review Board for information and approval in accordance with local requirements, and to Regulatory Agencies if required. Approval must be obtained before any changes can be implemented, except for changes necessary to eliminate an immediate hazard to trial patients, or when the change(s) involves only logistical or administrative aspects of the trial (e.g., change in monitor[s], change of telephone number[s]).

# 14. CONDITIONS FOR TERMINATING THE STUDY

Both the Sponsor and the investigator reserve the right to terminate the study at any time. Should this be necessary, both parties will arrange the procedures on an individual study basis after review and consultation. In terminating the study, Roche and the investigator will assure that adequate consideration is given to the protection of the patients' interests. The appropriate IRB/EC and Regulatory Agencies should be informed accordingly.

# 15. STUDY DOCUMENTATION, CRFS AND RECORD KEEPING

## 15.1 Investigator's Files / Retention of Documents

The Investigator must maintain adequate and accurate records to enable the conduct of the study to be fully documented and the study data to be subsequently verified. These documents should be classified into two different separate categories [1] Investigator's Study File, and [2] patient clinical source documents.

The Investigator's Study File will contain the protocol/amendments, eCRF and schedule of assessments, Independent Ethics Committee/Institutional Review Board and governmental approval with correspondence, sample informed consent, drug records, staff curriculum vitae and authorization forms and other appropriate documents/correspondence, etc. In addition at the end of the study the investigator will receive the patient data, which includes an audit trail containing a complete record of all changes to data, query resolution correspondence and reasons for changes, in human readable format on CD which also has to be kept with the Investigator's Study File.

Subject clinical source documents (usually defined by the project in advance to record key efficacy/safety parameters independent of the eCRFs) would include patient hospital/clinic records, physician's and nurse's notes, appointment book, original

laboratory reports, ECG, EEG, X-ray, pathology and special assessment reports, signed informed consent forms, consultant letters, and patient screening and enrollment logs. The Investigator must keep the two categories of documents as described above (including the archival CD) on file for at least 15 years after completion or discontinuation of the study. After that period of time the documents may be destroyed, patient to local regulations.

Should the Investigator wish to assign the study records to another party or move them to another location, Roche must be notified in advance.

If the Investigator can not guarantee this archiving requirement at the investigational site for any or all of the documents, special arrangements must be made between the Investigator and Roche to store these in a sealed container(s) outside of the site so that they can be returned sealed to the Investigator in case of a regulatory audit. Where source documents are required for the continued care of the patient, appropriate copies should be made for storing outside of the site.

ICH GCP guidelines require that Investigators maintain information in the study patient's records which corroborate data collected on the eCRF(s). Completed eCRF will be transferred to Sponsor.

## 15.2 Source Documents and Background Data

The investigator shall supply the Sponsor on request with any required background data from the study documentation or clinic records. This is particularly important when errors in data transcription are suspected. In case of special problems and/or governmental queries or requests for audit inspections, it is also necessary to have access to the complete study records, provided that patient confidentiality is protected.

## 15.3 Audits and Inspections

The investigator should understand that source documents for this trial should be made available to appropriately qualified personnel from the Roche Pharma Development Quality Assurance Unit or its designees, or to health authority inspectors after appropriate notification. The verification of the eCRF data must be by direct inspection of source documents.

## 15.4 Electronic Case Report Forms

Data for this study will be captured via an Electronic Data Capture (EDC) system by using eCRFs. An audit trail will maintain a record of initial entries and changes made; reasons for change; time and date of entry; and user name of person authorizing entry or change. The investigator must update eCRF and connect on a regular basis.

For each patient enrolled, an eCRF must be completed and electronically signed by the principal investigator or authorized delegate from the study staff. This also applies to records for those patients who fail to complete the study (even during a pre-randomization screening period if an eCRF was initiated). If a patient withdraws from the study, the reason must be noted on the eCRF. If a patient is withdrawn from the study because of a treatment-limiting AE, thorough efforts should be made to clearly document the outcome.

The investigator should ensure the accuracy, completeness and timeliness of the data reported to the Sponsor in the eCRFs and in all required reports.

### 15.5 Financial Disclosure

The investigator(s) will provide the Sponsor with sufficient accurate financial information (PD35) to allow the Sponsor to submit complete and accurate financial certification or disclosure statements to the appropriate regulatory authorities. The investigator is responsible to promptly update any information provided to the Sponsor if relevant changes occur in the course of the investigation and for 1 year following the completion of the study (last patient, last visit).

## 16. MONITORING THE STUDY

It is understood that the responsible Roche monitor [or designee] will contact and visit the investigator regularly and will be allowed, on request, to inspect the various records of the trial (eCRFs and other pertinent data) provided that patient confidentiality is maintained in accord with local requirements.

It will be the monitor's responsibility to inspect the eCRFs at regular intervals throughout the study, to verify the adherence to the protocol and the completeness, consistency and accuracy of the data being entered on them. The monitor must verify that the patient received the study drug assigned by the randomization center (by controlling the written confirmation of the randomization by IxRS). The monitor should have access to laboratory test reports and other patient records needed to verify the entries in the eCRF. The investigator (or deputy) agrees to cooperate with the monitor to ensure that any problems detected in the course of these monitoring visits are resolved.

Roche Clinical Repository specimens will at all times be tracked in a manner consistent with Good Clinical Practice, by a quality controlled, auditable and validated Laboratory Information Management System, to ensure compliance with data confidentiality as well as adherence to authorized use of specimens as specified in the study protocol and ICF, respectively. Roche monitors and auditors will have direct access to appropriate parts of records relating to patients participating in this study for the purposes of verifying the data provided to Roche. The site will permit monitoring, audits, Institutional Review Board/Independent Ethics Committee (IRB/IEC) review, and regulatory inspections by providing direct access to source data and documents related to the RCR Research Project.

## 17. CONFIDENTIALITY OF TRIAL DOCUMENTS AND SUBJECT RECORDS

The investigator must assure that patients' anonymity will be maintained and that their identities are protected from unauthorized parties. On CRFs or other documents submitted to the Sponsor, patients should not be identified by their names, but by an identification code. The investigator should keep a patient enrollment log showing codes, names and addresses. The investigator should maintain documents not for submission to Roche, e.g., patients' written consent forms, in strict confidence.

Roche already maintains rigorous confidentiality standards for clinical studies by "coding" (i.e. assigning a unique patient ID number at the investigator site) all patients enrolled in Roche clinical studies. This means that patient names are not included in

data sets that are transmitted to any Roche location. Given the sensitive nature of genetic data, Roche has implemented a number of additional processes to assure patient confidentiality. All specimens taken for inherited genetic research that will be stored in the RCR (see Section 5.5) undergo a second level of "coding". At Roche, the specimen is transferred to a new tube and labeled with a new random number. This is referred to as "Double Coding (De-Identification)". Data generated following the use of these specimens and all clinical data transferred from the clinical study database and considered relevant, will also be labeled with this same code. The "linking key" between the participant's identification number and this new independent code will be stored in a secure database system. Access to the table linking the participant identification number to the specimen code will be strictly limited and monitored by audit trail. Legitimate operational reasons for accessing the "linking key" will be documented in a standard operating procedure. Access to the "linking key" for any other reason will require written approval from the Governance Committee responsible for the specimen(s).

# 18. CLINICAL STUDY REPORT (CSR)

A clinical study report will be written and distributed to Health Authorities as required by applicable regulatory requirements.

# 19. PUBLICATION OF DATA AND PROTECTION OF TRADE SECRETS

Roche will comply with the requirements for publication of study results.

The results of this study may be published or presented at scientific meetings. If this is foreseen, the investigator agrees to submit all manuscripts or abstracts to Roche prior to submission. This allows the Sponsor to protect proprietary information and to provide comments based on information from other studies that may not yet be available to the investigator.

In accordance with standard editorial and ethical practice, Roche will generally support publication of multicenter trials only in their entirety and not as individual center data. In this case, a coordinating investigator will be designated by mutual agreement.

Authorship will be determined by mutual agreement and in line with International Committee of Medical Journal Editors (ICMJE) authorship requirements. Any formal publication of the study in which contribution of Roche personnel exceeded that of conventional monitoring will be considered as a joint publication by the investigator and the appropriate Roche personnel.

Data derived from RCR specimen analysis on individual patients will not be provided to study investigators, except where explicitly stipulated in a study protocol (e.g. if the result is an enrollment criterion). Exceptions may be granted (e.g. if biomarker data would be linked to safety issues). The aggregate results of any research conducted using RCR specimens will be available in accordance with the effective Roche policy on study data publication.

Any inventions and resulting patents, improvements and / or know- how originating from the use of the RCR will become and remain the exclusive and unburdened property of Roche, except where agreed otherwise.

## Appendix 1: AEs Categories for Determining Relationship to Test Drug

The causality relationship of study drug to the adverse event will be assessed by the investigator as either: Yes or No.

If there is a reasonable suspected causal relationship to the study medication, i.e., there are facts (evidence) or arguments to suggest a causal relationship, drug-event relationship should be assessed as Yes.

**The following criteria should be considered in order to assess the relationship as Yes:**

– Reasonable temporal association with drug administration

– It may or may not have been produced by the subject's clinical state, environmental or toxic factors, or other modes of therapy administered to the subject.

– Known response pattern to suspected drug

– Disappears or decreases on cessation or reduction in dose

– Reappears on rechallenge

**The following criteria should be considered in order to assess the relationship as No:**

– It does not follow a reasonable temporal sequence from administration of the drug.

– It may readily have been produced by the subject's clinical state, environmental or toxic factors, or other modes of therapy administered to the subject.

– It does not follow a known pattern of response to the suspected drug.

– It does not reappear or worsen when the drug is readministered.

## Appendix 2: ICH Guidelines for Clinical Safety Data Management, Definitions and Standards for Expedited Reporting, Topic E2

A serious adverse event is any experience that suggests a significant hazard, contraindication, side effect or precaution. It is any AE that at any dose fulfills at least one of the following criteria:

– is fatal; [results in death] [**NOTE**: death is an outcome, not an event]

– is Life-Threatening [**NOTE**: the term "Life-Threatening" refers to an event in which the patient was at immediate risk of death at the time of the event; it does not refer to an event which could hypothetically have caused a death had it been more severe]

– requires in-patient hospitalization or prolongation of existing hospitalization

– results in persistent or significant disability/incapacity

– is a congenital anomaly/birth defect

– is medically significant or requires intervention to prevent one or other of the outcomes listed above.

Medical and scientific judgment should be exercised in deciding whether expedited reporting to the Sponsor is appropriate in other situations, such as important medical events that may not be immediately life-threatening or result in death or hospitalization but may jeopardize the patient or may require intervention to prevent one of the outcomes listed in the definitions above. These situations should also usually be considered serious.

Examples of such events are intensive treatment in an emergency room or at home for allergic bronchospasm; blood dyscrasias or convulsions that do not result in hospitalization; or development of drug dependency or drug abuse.

An unexpected AE is one in which the nature or severity is not consistent with the applicable product information.

Causality is initially assessed by the investigator. For Serious Adverse Events, possible causes of the event are indicated by selecting one or more options. (Check all that apply)

– Pre-existing/Underlying disease - specify

– Study treatment - specify the drug(s) related to the event

– Other treatment (concomitant or previous) - specify

– Protocol-related procedure

– Other (e.g. accident, new or intercurrent illness) - specify

The term severe is a measure of intensity, thus a severe AE is not necessarily serious. For example, nausea of several hours' duration may be rated as severe, but may not be clinically serious.

A serious adverse event occurring during the study or which comes to the attention of the investigator within 15 days after stopping the treatment or during the protocol-defined follow-up period, if this is longer, whether considered treatment-related or not, must be reported. In addition, a serious adverse event that occurs after this time, if considered related to test "drug", should be reported.

Such preliminary reports will be followed by detailed descriptions later which will include copies of hospital case reports, autopsy reports and other documents when requested and applicable.

For serious adverse events, the following must be assessed and recorded on the AEs eform of the eCRF: intensity, relationship to test substance, action taken, and outcome to date.

The investigator must notify the Ethics Review Committee/Institutional Review Board of a serious adverse event in writing as soon as is practical and in accordance with international and local laws and regulations.

ROCHE LOCAL COUNTRY CONTACT for SAEs: Local Monitor:

See attached *Protocol Administrative and Contact Information & List of Investigators Form,[ gcp_for000227]*, for details of administrative and contact information.

ROCHE HEADQUARTERS CONTACT for SAEs and other medical emergencies: Clinical Operations/Clinical Science:

See attached *Protocol Administrative and Contact Information & List of Investigators form,[gcp_for000227]*, for details of administrative and contact information.

24 HOUR MEDICAL COVERAGE:

Identification of a contact for 24 Hour Medical Coverage is mandatory to be compliant with worldwide regulatory agencies and to ensure the safety of study patients.

An Emergency Medical Call Center Help Desk will access the Roche Medical Emergency List, escalate emergency medical calls, provide medical translation service (if necessary), connect the investigator with the Roche medical contact for this study and track all calls. The Emergency Medical Call Center Help Desk will be manned 24 hours 7 days a week. Toll free numbers will be distributed to all investigators running Roche Pharma Development clinical trials. The Help Desk will be used for medical emergencies outside regular business hours, or when the regular Clinical Science Leader cannot be reached.

See the attached *Protocol Administrative and Contact Information & List of Investigators form [gcp_for000227]*, for details of administrative, contact information, and Emergency Medical Call Center Help Desk toll-free numbers.

## Appendix 3: Common Terminology Criteria for Adverse Events (CTCAE)

In the present study, toxicities will be graded according to the Common Terminology Criteria for Adverse Events (CTCAE), version 4.0.

The Common Terminology Criteria for Adverse Events v4.0 (CTCAE) can be found in the Roche hand-out entitled: " Common Terminology Criteria for Adverse Events v4.0" or via the following web-site: http://ctep.cancer.gov

## Appendix 4: Telephone Interviews

The purpose of this interview is to identify any new or worsening neurological symptoms that warrant an unscheduled visit and to collect data on possible events of infections. Telephone interviews should be performed by study personnel every 4 weeks between clinic visits (with exemption of prolonged B-cell monitoring period when telephone interviews need to be performed every 12 weeks) – see also Section 5.3.4.4.

**Please ask the following questions and record patient's answers during the Telephone Interview:**

| Questions | No | Yes |
|---|---|---|
| **1. Since your last visit or telephone interview, have you had any new or worsening medical problems (such as sudden changes in your thinking, alterations in your behavior, visual disturbances, extremity weakness, limb coordination problems, or gait abnormalities) that have persisted over several days?** | | |
| **2. Since your last visit or telephone interview, have you taken any new medicines to treat cancer or MS or any other new medicines that weaken your immune system?** | | |
| **3. Since your last visit or telephone interview, other than for the treatment of a recent relapse, have you taken any of the following medicines: methylprednisone, (e.g. Depo-Medrol®, Solu-Medrol®), dexamethasone (e.g. Decadron®), prednisolone, or other steroid medicine?** | | |
| **4. Since your last visit or telephone interview, have you had any signs or symptoms of infection?** | | |

If the patient answered YES to any question, contact the Treating Investigator and review the patient's answers. The Investigator can determine if an unscheduled visit is required.

**Record any pertinent comments made by the patient during the interview:**

______________________________________________________________

______________________________________________________________

______________________________________________________________

NAME:________________________________ Date:________________________________
*Name of person completing the telephone interview*

## Appendix 4: Telephone Interviews (Cont.)

**Below is a sample list of medications that can weaken the immune system. This list does not include all drugs that can suppress the immune system.**

**Approved MS Therapies**:

Glatiramer acetate (Copaxone®)

Interferon β-1a (Rebif®, AVONEX®)

Interferon β-1b (Betaseron®)

Mitoxantrone (Novantrone®)

Natalizumab (Tysabri®)

Fingolimod (Gilenya®) – if relevant

**Immunosuppressants/Antineoplastics:**

Azathioprine (Imuran®, Azasan®)

Cladribine (Leustatin®)

Cyclophosphamide (Cytoxan®, Neosar®)

Cyclosporine (Sandimmune®, Neoral®)

Fludarabine phosphate (Fludara®)

Leflunomide (Arava®)

Mercaptopurine (Purinethol®)

Methotrexate (Methotrex®, Rheumatrex®, Trexall®)

Mycophenolate mofetil (CellCept®)

Pemetrexed (Alimta®)

**Additional Immunomodulators and Immunosuppressants:**

Other interferons (Actimmune®, Infergen®, Intron® A,
Pegasys®, PEG-Intron®, Rebetron®, Roferon®-A)

Adalimumab (Humira®)

Alefacept (Amevive®)

Alemtuzumab (Campath®)

Anakinra (Kineret®)

Daclizumab (Zenapax®)

Etanercept (Enbrel®)

Infliximab (Remicade®)

Intravenous immunoglobulin (IVIG)

Ofatumumab (Arzerra®)

Rituximab (Rituxan/MabThera®)

Trastuzumab (Herceptin®)

## Appendix 5: Modified Fatigue Impact Scale (MFIS)

**MFIS**

### MODIFIED FATIGUE IMPACT SCALE (MFIS)

Following is a list of statements that describe how fatigue may affect a person. Fatigue is a feeling of physical tiredness and lack of energy that many people experience from time to time. In medical conditions like MS, feelings of fatigue can occur more often and have a greater impact than usual. Please read each statement carefully, and then SELECT THE ONE NUMBER that best indicates how often fatigue has affected you in this way during the PAST 4 WEEKS. (If you need help in marking your responses, TELL THE INTERVIEWER THE NUMBER of the best response.) PLEASE ANSWER EVERY QUESTION. If you are not sure which answer to select, please choose the one answer that comes closest to describing you. The interviewer can explain any words or phrases that you do not understand.

| Because of my fatigue during the PAST 4 WEEKS.... | Never | Rarely | Sometimes | Often | Almost always |
|---|---|---|---|---|---|
| * 1. I have been less alert. | ○ 0 | ○ 1 | ○ 2 | ○ 3 | ○ 4 |
| * 2. I have had difficulty paying attention for long periods of time. | ○ 0 | ○ 1 | ○ 2 | ○ 3 | ○ 4 |
| * 3. I have been unable to think clearly. | ○ 0 | ○ 1 | ○ 2 | ○ 3 | ○ 4 |
| * 4. I have been clumsy and uncoordinated. | ○ 0 | ○ 1 | ○ 2 | ○ 3 | ○ 4 |
| * 5. I have been forgetful. | ○ 0 | ○ 1 | ○ 2 | ○ 3 | ○ 4 |
| * 6. I have had to pace myself in my physical activities. | ○ 0 | ○ 1 | ○ 2 | ○ 3 | ○ 4 |

Back | Next

MFIS

## MODIFIED FATIGUE IMPACT SCALE (MFIS)

| Because of my fatigue during the PAST 4 WEEKS.... | Never | Rarely | Sometimes | Often | Almost always |
|---|---|---|---|---|---|
| * 7. I have been less motivated to do anything that requires physical effort. | ◯ 0 | ◯ 1 | ◯ 2 | ◯ 3 | ◯ 4 |
| * 8. I have been less motivated to participate in social activities. | ◯ 0 | ◯ 1 | ◯ 2 | ◯ 3 | ◯ 4 |
| * 9. I have been limited in my ability to do things away from home. | ◯ 0 | ◯ 1 | ◯ 2 | ◯ 3 | ◯ 4 |
| * 10. I have had trouble maintaining physical effort for long periods. | ◯ 0 | ◯ 1 | ◯ 2 | ◯ 3 | ◯ 4 |
| * 11. I have had difficulty making decisions. | ◯ 0 | ◯ 1 | ◯ 2 | ◯ 3 | ◯ 4 |
| * 12. I have been less motivated to do anything that requires thinking. | ◯ 0 | ◯ 1 | ◯ 2 | ◯ 3 | ◯ 4 |
| * 13. my muscles have felt weak. | ◯ 0 | ◯ 1 | ◯ 2 | ◯ 3 | ◯ 4 |
| * 14. I have been physically uncomfortable. | ◯ 0 | ◯ 1 | ◯ 2 | ◯ 3 | ◯ 4 |

Back | Next

## Appendix 5: Modified Fatigue Impact Scale (MFIS) (Cont.)

MFIS

### MODIFIED FATIGUE IMPACT SCALE (MFIS)

| Because of my fatigue during the PAST 4 WEEKS.... | Never | Rarely | Sometimes | Often | Almost always |
|---|---|---|---|---|---|
| * 15. I have had trouble finishing tasks that require thinking. | ◯ 0 | ◯ 1 | ◯ 2 | ◯ 3 | ◯ 4 |
| * 16. I have had difficulty organizing my thoughts when doing things at home or at work. | ◯ 0 | ◯ 1 | ◯ 2 | ◯ 3 | ◯ 4 |
| * 17. I have been less able to complete tasks that require physical effort. | ◯ 0 | ◯ 1 | ◯ 2 | ◯ 3 | ◯ 4 |
| * 18. my thinking has been slowed down. | ◯ 0 | ◯ 1 | ◯ 2 | ◯ 3 | ◯ 4 |
| * 19. I have had trouble concentrating. | ◯ 0 | ◯ 1 | ◯ 2 | ◯ 3 | ◯ 4 |
| * 20. I have limited my physical activities. | ◯ 0 | ◯ 1 | ◯ 2 | ◯ 3 | ◯ 4 |
| * 21. I have needed to rest more often or for longer periods. | ◯ 0 | ◯ 1 | ◯ 2 | ◯ 3 | ◯ 4 |

Back | Next

## Appendix 6: The Center for Epidemiologic Studies Depression Scale (CES-D)

**CES-D Scale**

### Center for Epidemiologic Studies Depression Scale (CES-D, NIMH)

Below is a list of the ways you might have felt or behaved. Please tell me how often you have felt this way in the last week.

| | During the Past Week | | | |
|---|---|---|---|---|
| | Rarely or none of the time (less than 1 Day) | Some or a little of the time (1-2 days) | Occasionally or a moderate amount of time (3-4 days) | Most or all of the time (5-7 days) |
| * 1. I was bothered by things that usually don't bother me. | ○ | ○ | ○ | ○ |
| * 2. I did not feel like eating: my appetite was poor. | ○ | ○ | ○ | ○ |
| * 3. I felt that I could not shake off the blues even with help from my family or friends. | ○ | ○ | ○ | ○ |
| * 4. I felt that I was just as good as other people. | ○ | ○ | ○ | ○ |
| * 5. I had trouble keeping my mind on what I was doing. | ○ | ○ | ○ | ○ |
| * 6. I felt depressed. | ○ | ○ | ○ | ○ |
| * 7. I felt that everything I did was an effort. | ○ | ○ | ○ | ○ |
| * 8. I felt hopeful about the future. | ○ | ○ | ○ | ○ |
| * 9. I thought my life had been a failure. | ○ | ○ | ○ | ○ |
| * 10. I felt fearful. | ○ | ○ | ○ | ○ |

Back | Next

## Appendix 6: The Center for Epidemiologic Studies Depression Scale (CES-D) (Cont.)

**CES-D Scale**

### Center for Epidemiologic Studies Depression Scale (CES-D, NIMH)

Below is a list of the ways you might have felt or behaved. Please tell me how often you have felt this way in the last week.

| | During the Past Week | | | |
|---|---|---|---|---|
| | Rarely or none of the time (less than 1 Day) | Some or a little of the time (1-2 days) | Occasionally or a moderate amount of time (3-4 days) | Most or all of the time (5-7 days) |
| * 11. My sleep was restless. | ○ | ○ | ○ | ○ |
| * 12. I was happy. | ○ | ○ | ○ | ○ |
| * 13. I talked less than usual. | ○ | ○ | ○ | ○ |
| * 14. I felt lonely. | ○ | ○ | ○ | ○ |
| * 15. People were unfriendly. | ○ | ○ | ○ | ○ |
| * 16. I enjoyed life. | ○ | ○ | ○ | ○ |
| * 17. I had crying spells. | ○ | ○ | ○ | ○ |
| * 18. I felt sad. | ○ | ○ | ○ | ○ |
| * 19. I felt that people dislike me. | ○ | ○ | ○ | ○ |
| * 20. I could not get "going". | ○ | ○ | ○ | ○ |

SCORING: zero for answers in the first column, 1 for answers in the second column, 2 for answers in the third column, 3 for answers in the fourth column. The scoring of positive items is reversed. Possible range of scores is zero to 60, with the higher scores indicating the presence of more symptomatology.

Back | Next

## Appendix 7: The Short Form (SF-36) Health Survey

**SF-36**

### Your Health and Well-Being

This survey asks for your views about your health. This information will help keep track of how you feel and how well you are able to do your usual activities. Thank you for completing this survey!

For each of the following questions, please SELECT the response that best describes your answer.

*** 1. In general, would you say your health is:**

| Excellent | Very good | Good | Fair | Poor |
|---|---|---|---|---|
| ○ 1 | ○ 2 | ○ 3 | ○ 4 | ○ 5 |

*** 2. COMPARED TO ONE YEAR AGO, how would you rate your health in general NOW?**

| Much better now than one year ago | Somewhat better now than one year ago | About the same as one year ago | Somewhat worse now than one year ago | Much worse now than one year ago |
|---|---|---|---|---|
| ○ 1 | ○ 2 | ○ 3 | ○ 4 | ○ 5 |

SF-36v2® Health Survey © 1992, 2002, 2009 Medical Outcomes Trust and QualityMetric Incorporated. All rights reserved.
SF-36® is a registered trademark of Medical Outcomes Trust.

Back | Next

## SF-36

3. The following questions are about activities you might do during a typical day. Does YOUR HEALTH NOW LIMIT YOU in these activities? If so, how much?

| | Yes, limited a lot | Yes, limited a little | No, not limited at all |
|---|---|---|---|
| * a. VIGOROUS ACTIVITIES, such as running, lifting heavy objects, participating in strenuous sports. | ○ 1 | ○ 2 | ○ 3 |
| * b. MODERATE ACTIVITIES, such as moving a table, pushing a vacuum cleaner, bowling, or playing golf. | ○ 1 | ○ 2 | ○ 3 |
| * c. Lifting or carrying groceries. | ○ 1 | ○ 2 | ○ 3 |
| * d. Climbing SEVERAL flights of stairs. | ○ 1 | ○ 2 | ○ 3 |
| * e. Climbing ONE flight of stairs. | ○ 1 | ○ 2 | ○ 3 |
| * f. Bending, kneeling, or stooping. | ○ 1 | ○ 2 | ○ 3 |
| * g. Walking MORE THAN A MILE. | ○ 1 | ○ 2 | ○ 3 |
| * h. Walking SEVERAL HUNDRED YARDS. | ○ 1 | ○ 2 | ○ 3 |
| * i. Walking ONE HUNDRED YARDS. | ○ 1 | ○ 2 | ○ 3 |
| * j. Bathing or dressing yourself. | ○ 1 | ○ 2 | ○ 3 |

SF-36v2® Health Survey © 1992, 2002, 2009 Medical Outcomes Trust and QualityMetric Incorporated. All rights reserved.
SF-36® is a registered trademark of Medical Outcomes Trust.

Back | Next

# Appendix 7: The Short Form (SF-36) Health Survey (Cont.)

**SF-36**

**4. During the PAST 4 WEEKS, how much of the time have you had any of the following problems with your work or other regular daily activities AS A RESULT OF YOUR PHYSICAL HEALTH?**

| | All of the time | Most of the time | Some of the time | A little of the time | None of the time |
|---|---|---|---|---|---|
| * a. Cut down on the AMOUNT OF TIME you spent on work or other activities. | ○ 1 | ○ 2 | ○ 3 | ○ 4 | ○ 5 |
| * b. ACCOMPLISHED LESS than you would like. | ○ 1 | ○ 2 | ○ 3 | ○ 4 | ○ 5 |
| * c. Were limited in the KIND of work or other activities. | ○ 1 | ○ 2 | ○ 3 | ○ 4 | ○ 5 |
| * d. Had DIFFICULTY performing the work or other activities (for example, it took extra effort). | ○ 1 | ○ 2 | ○ 3 | ○ 4 | ○ 5 |

**5. During the PAST 4 WEEKS, how much of the time have you had any of the following problems with your work or other regular daily activities AS A RESULT OF ANY EMOTIONAL PROBLEMS (such as feeling depressed or anxious)?**

| | All of the time | Most of the time | Some of the time | A little of the time | None of the time |
|---|---|---|---|---|---|
| * a. Cut down on the AMOUNT OF TIME you spent on work or other activities. | ○ 1 | ○ 2 | ○ 3 | ○ 4 | ○ 5 |
| * b. ACCOMPLISHED LESS than you would like. | ○ 1 | ○ 2 | ○ 3 | ○ 4 | ○ 5 |
| * c. Did work or other activities LESS CAREFULLY THAN USUAL. | ○ 1 | ○ 2 | ○ 3 | ○ 4 | ○ 5 |

SF-36v2® Health Survey © 1992, 2002, 2009 Medical Outcomes Trust and QualityMetric Incorporated. All rights reserved.
SF-36® is a registered trademark of Medical Outcomes Trust.

Back | Next

## SF-36

* 6. During the PAST 4 WEEKS, to what extent has your PHYSICAL HEALTH OR EMOTIONAL PROBLEMS interfered with your normal social activities with family, friends, neighbors, or groups?

| Not at all | Slightly | Moderately | Quite a bit | Extremely |
|---|---|---|---|---|
| ○ 1 | ○ 2 | ○ 3 | ○ 4 | ○ 5 |

* 7. How much BODILY pain have you had during the PAST 4 WEEKS?

| None | Very mild | Mild | Moderate | Severe | Very severe |
|---|---|---|---|---|---|
| ○ 1 | ○ 2 | ○ 3 | ○ 4 | ○ 5 | ○ 6 |

* 8. During the PAST 4 WEEKS, how much did PAIN interfere with your normal work (including both work outside the home and housework)?

| Not at all | A little bit | Moderately | Quite a bit | Extremely |
|---|---|---|---|---|
| ○ 1 | ○ 2 | ○ 3 | ○ 4 | ○ 5 |

SF-36v2® Health Survey © 1992, 2002, 2009 Medical Outcomes Trust and QualityMetric Incorporated. All rights reserved.
SF-36® is a registered trademark of Medical Outcomes Trust.

Back | Next

## SF-36

9. These questions are about how you feel and how things have been with you DURING THE PAST 4 WEEKS. For each question, please give the one answer that comes closest to the way you have been feeling. How much of the time during the PAST 4 WEEKS...

| | All of the time | Most of the time | Some of the time | A little of the time | None of the time |
|---|---|---|---|---|---|
| * a. Did you feel full of life? | ○ 1 | ○ 2 | ○ 3 | ○ 4 | ○ 5 |
| * b. Have you been very nervous? | ○ 1 | ○ 2 | ○ 3 | ○ 4 | ○ 5 |
| * c. Have you felt so down in the dumps that nothing could cheer you up? | ○ 1 | ○ 2 | ○ 3 | ○ 4 | ○ 5 |
| * d. Have you felt calm and peaceful? | ○ 1 | ○ 2 | ○ 3 | ○ 4 | ○ 5 |
| * e. Did you have a lot of energy? | ○ 1 | ○ 2 | ○ 3 | ○ 4 | ○ 5 |
| * f. Have you felt downhearted and depressed? | ○ 1 | ○ 2 | ○ 3 | ○ 4 | ○ 5 |
| * g. Did you feel worn out? | ○ 1 | ○ 2 | ○ 3 | ○ 4 | ○ 5 |
| * h. Have you been happy? | ○ 1 | ○ 2 | ○ 3 | ○ 4 | ○ 5 |
| * i. Did you feel tired? | ○ 1 | ○ 2 | ○ 3 | ○ 4 | ○ 5 |

SF-36v2® Health Survey © 1992, 2002, 2009 Medical Outcomes Trust and QualityMetric Incorporated. All rights reserved.
SF-36® is a registered trademark of Medical Outcomes Trust.

Back | Next

**SF-36**

* 10. During the PAST 4 WEEKS, how much of the time has your PHYSICAL HEALTH OR EMOTIONAL PROBLEMS interfered with your social activities (like visiting with friends, relatives, etc.)?

| All of the time | Most of the time | Some of the time | A little of the time | None of the time |
|---|---|---|---|---|
| ○ 1 | ○ 2 | ○ 3 | ○ 4 | ○ 5 |

11. How TRUE or FALSE is EACH of the following statements for you?

| | Definitely true | Mostly true | Don't know | Mostly false | Definitely false |
|---|---|---|---|---|---|
| * a. I seem to get sick a little easier than other people. | ○ 1 | ○ 2 | ○ 3 | ○ 4 | ○ 5 |
| * b. I am as healthy as anybody I know. | ○ 1 | ○ 2 | ○ 3 | ○ 4 | ○ 5 |
| * c. I expect my health to get worse. | ○ 1 | ○ 2 | ○ 3 | ○ 4 | ○ 5 |
| * d. My health is excellent. | ○ 1 | ○ 2 | ○ 3 | ○ 4 | ○ 5 |

SF-36v2® Health Survey © 1992, 2002, 2009 Medical Outcomes Trust and QualityMetric Incorporated. All rights reserved.
SF-36® is a registered trademark of Medical Outcomes Trust.

Back | Next

# PROTOCOL

| | |
|---|---|
| **TITLE:** | **A RANDOMIZED, DOUBLE-BLIND, DOUBLE-DUMMY, PARALLEL-GROUP STUDY TO EVALUATE THE EFFICACY AND SAFETY OF OCRELIZUMAB IN COMPARISON TO INTERFERON BETA-1A (REBIF®) IN PATIENTS WITH RELAPSING MULTIPLE SCLEROSIS** |
| **PROTOCOL NUMBER:** | WA21093 |
| **VERSION NUMBER:** | *D* |
| **EUDRACT NUMBER:** | 2010-020315-36 |
| **IND NUMBER:** | 100,593 |
| **TEST PRODUCT:** | Ocrelizumab (RO4964913) |
| **MEDICAL MONITOR:** | Dr. Algirdas Kakarieka |
| **SPONSOR:** | F. Hoffmann-La Roche, Ltd |
| **DATE FINAL:** | 27 May 2011 |
| **DATES AMENDED:** | Version B: 15 June 2012<br>Version C: 28 March 2013<br>Version D: See electronic date stamp below |

**PROTOCOL AMENDMENT APPROVAL**

| Approver's Name | Title | Date and Time (UTC) |
|---|---|---|
| Hope,Mark | Company Signatory | 04-Sep-2014 12:16:04 |

**CONFIDENTIAL**
The information contained in this document, especially any unpublished data, is the property of F. Hoffmann-La Roche, Ltd (or under its control) and therefore is provided to you in confidence as an investigator, potential investigator, or consultant, for review by you, your staff, and an applicable Ethics Committee or Institutional Review Board. It is understood that this information will not be disclosed to others without written authorization from Roche except to the extent necessary to obtain informed consent from persons to whom the drug may be administered.

**PROTOCOL WA21093 COORDINATING INVESTIGATOR:**

**Prof. Dr. Anthony Traboulsee**
Professor of Neurology
Director of MS Clinical Research Group
Koerner Pavilion
2211 Wesbrook Mall
Vancouver BC V6T 2B5
Canada
Phone: (+1) 604 822-0788

## SYNOPSIS OF PROTOCOL NUMBER WA21093*D*

| | | | |
|---|---|---|---|
| TITLE | **A Randomized, Double-Blind, Double-Dummy, Parallel-Group Study To Evaluate The Efficacy And Safety Of Ocrelizumab In Comparison To Interferon Beta-1a (Rebif®) In Patients With Relapsing Multiple Sclerosis** | | |
| SPONSOR | F. Hoffmann-La Roche Ltd<br>Genentech Inc | CLINICAL PHASE | III |
| INDICATION | Relapsing Multiple Sclerosis | | |
| OBJECTIVES | **Primary:**<br>The primary objective of this study is to assess whether the efficacy of ocrelizumab 600 mg (given as dual infusions of 300 mg on Days 1 and 15 of the first 24-week treatment cycle and as a single infusion of 600 mg on Day 1 of each 24-week treatment cycle thereafter) intravenously every 24 weeks is superior to Rebif® as measured by the annualized protocol-defined* relapse rate by 2 years (96 weeks) in patients with relapsing multiple sclerosis (MS).<br>**Secondary:**<br>The *key* secondary objectives of this study are to evaluate whether the efficacy of ocrelizumab is superior to Rebif®, as reflected by the following measures:<br>• The time to onset of *confirmed* disability progression for at least 12 weeks *with the* initial event of neurological worsening occur*ring* during the 96-week, double-blind, double-dummy, treatment period.<br>• *The total number of T1 Gd-enhancing lesions as detected by brain MRI at Weeks 24, 48, and 96*<br>• The total number of new, and/or enlarging T2 hyperintense lesions as detected by brain MRI at Weeks 24, 48, and 96.<br>• *The proportion of patients who have confirmed disability improvement for at least 12 weeks with the initial event of neurological improvement occurring during the 96-week double-blind, double-dummy treatment period.*<br>• The time to onset of *confirmed* disability progression for at least 24 weeks, *with the* initial event of neurological worsening occur*ring* during the 96-week, double-blind, double-dummy, treatment period.<br>• *The total number of T1-hypo-intense lesions (Chronic Black Holes) at Weeks 24, 48, and 96*<br>• The change in Multiple Sclerosis Functional Composite Scale (MSFCS) score from baseline to Week 96.<br>• The *percentage* change in brain volume as detected by brain MRI from Week 24 to Week 96.<br>• *The change in SF-36 Physical Component Summary (PCS) Score from baseline to Week 96*<br>• *The proportion of patients who have no evidence of disease activity (NEDA) by Week 96*<br>**Safety:**<br>To evaluate the safety and tolerability of ocrelizumab 600 mg (given as | | |

dual infusions of 300 mg on Days 1 and 15 of the first 24-week treatment cycle and as a single infusion of 600 mg on Day 1 of each 24-week treatment cycle thereafter) intravenously every 24 weeks in patients with relapsing MS (including exploratory, long-term safety and tolerability in those patients entering the Open-Label Extension [OLE] Phase).

**Pharmacokinetics/Pharmacodynamics:**

To explore the pharmacokinetics, immunogenicity and pharmacodynamics of ocrelizumab in patients with relapsing MS.

**Exploratory objectives:**

- The change in low contrast visual acuity from baseline to Weeks 48 and 96.
- The change in the Symbol Digit Modalities Test from baseline to Weeks 48 and 96.
- *The proportion of relapse free patients by Week 96.*
- *The change in total T2 hyperintense lesion volume as detected by brain MRI from baseline to Week 96.*
- The annualized relapse rate, based on *all* clinical relapses at the end of the 96-week comparative treatment period *(protocol-defined relapses are a subset of all clinical relapses).*
- *The ARR of relapses requiring IV steroid therapy.*
- *The ARR of severe relapses.*
- The *percentage* change in brain volume as detected by brain MRI from baseline to Week 96.
- The change in Multiple Sclerosis Functional Composite Scale (MSFCS) score from baseline to Week 48.
- The cumulative change in EDSS scores, measured in area under the curve (AUC) by Week 96.
- The change in EDSS from baseline to Week 96.
- The change in timed 25-foot walk from baseline to Week 96.
- The change in 9-hole peg test from baseline to Week 96.
- The change in paced auditory serial addition test (PASAT) from baseline to Weeks 48 and 96.
- The time to onset of sustained 20% increase in 9-hole peg test for at least 12 weeks.
- The time to onset of sustained 20% increase in timed 25 foot walk for at least 12 weeks.
- *The change in fatigue, as measured by the Modified Fatigue Impact Scale (MFIS) total score from baseline to Week 96.*
- *The change from baseline in patient-reported depressive symptoms, as measured by the Center for Epidemiologic Studies Depression Scale (CES-D), from baseline to Week 96.*
- *Analyses of EQ-5D, collected at baseline, Week 48, and Week 96.*
- The change in Karnofsky Performance Status Scale from baseline to Weeks 96.
- *The percentage change in cortical grey matter volume from baseline to Week 96.*
- *The percentage change in white matter volume from baseline to Week 96.*
- *The proportion of patients who have confirmed disability*

| | |
|---|---|
| | *improvement sustained for at least 24 weeks, with the initial event of neurological improvement occurring during the 96-week double-blind double-dummy treatment period.*<br>• *The proportion of patients who have disability improvement sustained for at least 12 weeks and sustained until the end of the 96-week, double-blind, double-dummy treatment period, with the initial event of neurological improvement occurring during the 96-week, double-blind, double-dummy treatment period.*<br>• *The duration of the confirmed disability improvement.*<br>• *The proportion of patients who, at Week 96, have improved, stable, or worsened disability, compared to baseline.*<br>• *The change in Quality of Life, as measured by the Short Form 36 version 2 Mental Component Summary (MCS) Score from baseline to Week 96.*<br>• To evaluate the long-term safety, tolerability, and efficacy of ocrelizumab in patients with the relapsing form of MS who are enrolled in the OLE Phase. |
| TRIAL DESIGN | Multicenter, randomized, double-blind, double-dummy, parallel-group study |
| NUMBER OF SUBJECTS | 800 patients in total, 400 patients per group using a 1:1 randomization ratio. Please refer to Sample Size and Statistical Analyses section of the synopsis for more details. |
| TARGET POPULATION | **<u>Inclusion criteria:</u>**<br>1. Ability to provide written, informed consent and to be able to follow the schedule of protocol assessments *.<br>2. Ages 18-55 years at screening, inclusive.<br>3. Diagnosis of MS, in accordance with the revised McDonald criteria (2010).<br>4. At least 2 documented clinical attacks within the last 2 years prior to screening or one clinical attack in the year prior to screening (but not within 30 days prior to screening).<br>5. Neurological stability for ≥30 days prior to both screening and baseline.<br>6. EDSS, at screening, from 0 to 5.5 inclusive.<br>7. Documented MRI of brain with abnormalities consistent with MS prior to screening.<br>8. Patients of <u>reproductive potential</u> must use reliable means of contraception as described below as a minimum (adherence to local requirements, if more stringent, is required**):<br>• For female patients: Two methods of contraception throughout the trial, including the active treatment phase AND for 48 weeks after the last dose of ocrelizumab, or until their B-cells have repleted, whichever is longer.<br>• For male patients: Two methods of contraception throughout the trial, including the active treatment phase AND for 24 weeks after the last dose of ocrelizumab. Acceptable methods of contraception include one primary (e.g., systemic hormonal contraception or tubal ligation of the female partner, vasectomy of the male partner) AND one secondary barrier method (e.g., latex condoms, spermicide) OR a double barrier method (e.g., |

latex condom, intrauterine device, vaginal ring or pessary plus spermicide [e.g., foam, vaginal suppository, gel, cream]).

9. For patients of non reproductive potential (adherence to local requirements, if more stringent, is required**):
   - Women may be enrolled if postmenopausal (i.e., spontaneous amenorrhea for the past year confirmed by an FSH level greater than 40 mIU/mL) unless the patient is receiving a hormonal therapy for their menopause or surgically sterile (i.e., hysterectomy, complete bilateral oophorectomy);
   - Men may be enrolled if they are surgically sterile (castration).

* Patients who are unable to complete exploratory assessments (e.g., electronic patient reported outcomes [ePROs]) due to physical/disease limitations will not be excluded from the study.

** Based on local Ethics Committees or National Competent Authority feedback additional requirements to assure contraception or to confirm menopause may be required (e.g., serum estradiol compatible with post-menopause status, longer duration of amenorrhea, higher level of FSH).

**Exclusion Criteria**

Patients who meet the following criteria must be excluded from study entry:

1. Diagnosis of primary progressive MS.
2. Disease duration of more than 10 years in patients with an EDSS ≤2.0 at screening.
3. Inability to complete an MRI (contraindications for MRI include but are not restricted to weight ≥140 kg, pacemaker, cochlear implants, presence of foreign substances in the eye, intracranial vascular clips, surgery within 6 weeks of entry into the study, coronary stent implanted within 8 weeks prior to the time of the intended MRI, etc).
   (Patients with contraindication to Gd can be enrolled into the study but cannot receive Gd contrast dyes during their MRI scans.)
4. Known presence of other neurological disorders which may mimic MS including but not limited to: neuromeylitis optica, Lyme disease, untreated vitamin B12 deficiency, neurosarcoidosis and cerebrovascular disorders.

**Exclusions Related to General Health**

5. Pregnancy or lactation.
6. Any concomitant disease that may require chronic treatment with systemic corticosteroids or immunosuppressants during the course of the study.
7. History or currently active primary or secondary immunodeficiency.
8. Lack of peripheral venous access.
9. History of severe allergic or anaphylactic reactions to humanized or murine monoclonal antibodies.
10. Significant or uncontrolled somatic disease or any other significant disease that may preclude patient from participating in the study.
11. Congestive heart failure (NYHA III or IV functional severity).
12. Known active bacterial, viral, fungal, mycobacterial infection or other infection, excluding fungal infection of nail beds.
13. Infection requiring hospitalization or treatment with intravenous

(i.v.) antibiotics within 4 weeks prior to baseline visit or oral antibiotics within 2 weeks prior to baseline visit.

14. History or known presence of recurrent or chronic infection (e.g., HIV, syphilis, tuberculosis).

    *Please note:* in Germany the following additional exclusion criteria apply:

    - *Positive anti – HIV I at screening*
    - *Positive anti – HIV II at screening*
    - *Positive QuantiFERON®-TB Gold test at screening*

    *Patients in Germany with an indeterminate result are not eligible for the study unless additional testing demonstrating a negative result is provided. Thus, these patients should have either a tuberculin skin test or have the QuantiFERON® TB Gold test repeated prior to enrollment into the study. If a tuberculin skin test is performed, an induration of ≥ 6 mm is "positive" for a patient with history of BCG vaccine, while an induration of ≥ 10 mm is "positive" for a patient without history of BCG vaccine.*

    *If necessary a QuantiFERON®-TB Gold test might be complemented by additional specific diagnostic tests as per standard procedures in Germany.*

15. History of progressive multifocal leukoencephalopathy (PML)
16. History of malignancy, including solid tumors and hematological malignancies, except basal cell carcinoma, *in situ* squamous cell carcinoma of the skin, and *in situ* carcinoma of the cervix of the uterus that have been previously completely excised with documented, clear margins.
17. History of alcohol or drug abuse within 24 weeks prior to baseline.
18. History or laboratory evidence of coagulation disorders.

**Exclusions Related to Medications***

19. Receipt of a live vaccine within 6 weeks prior to the baseline visit. In rare cases when patient requires vaccination with a live vaccine, the screening period may be extended but cannot exceed 8 weeks.
20. Treatment with any investigational agent within 24 weeks of screening (Visit 1) or five half-lives of the investigational drug (whichever is longer; or treatment with any experimental procedures for MS [e.g., treatment for chronic cerebrospinal venous insufficiency]).
21. Contraindications to or intolerance of oral or i.v. corticosteroids, according to the country label, including:
    a) Psychosis not yet controlled by a treatment;
    b) Hypersensitivity to any of the constituents.
22. Contraindication to Rebif® or incompatibility with Rebif® use, including:
    a) Current severe depression and/or suicidal ideation;
    b) Hypersensitivity to natural or recombinant interferon-β, or to any excipients;
    c) Previous suboptimal response to High Dose High Frequency (HDHF) interferon or cessation of HDHF interferon therapy due to poor tolerability;
    d) Prior cessation of Rebif® therapy due to toxicity, which is likely to recur.
23. Treatment with dalfamipridine (Ampyra®) unless on stable dose for ≥ 30 days prior to screening. Wherever possible, patients should remain on stable doses throughout the 96 week treatment period.
24. Previous treatment with B-cell targeted therapies (i.e., rituximab,

ocrelizumab, atacicept, belimumab or ofatumumab).

25. Systemic corticosteroid therapy within 4 weeks prior to screening.**
26. Any previous treatment with alemtuzumab (Campath), anti-CD4, cladribine, mitoxantrone, daclizumab, teriflunomide, laquinimod, total body irradiation or bone marrow transplantation.
27. Treatment with cyclophosphamide, azathioprine, mycophenolate mofetil (MMF), cyclosporine, methotrexate or natalizumab within 24 months prior to screening. NB. Patients previously treated with natalizumab will be eligible for this study only if duration of treatment with natalizumab was < 1 year.
28. Treatment with fingolimod (FTY720, Gilenya®) or other S1P receptor modulator (i.e., BAF312), or with BG12, within 24 weeks prior to screening.
    (NB. Only patients with T lymphocyte count ≥ LLN will be eligible for this study.)
29. Treatment with i.v. immunoglobulin within 12 weeks prior to baseline.

* Patients screened for this study should not be withdrawn from therapies for the sole purpose of meeting eligibility for the trial. Patients, who discontinue their current therapy for non-medical reasons, should specifically be informed before deciding to enter the study of their treatment options.

** The screening period may be extended (but cannot exceed 8 weeks) for patients who have used systemic corticosteroids for their MS before screening. For a patient to be eligible, systemic corticosteroids should not have been administered also between screening and baseline.

**Exclusions Related to Laboratory Findings***

30. Positive serum β hCG measured at screening.
31. Positive screening tests for hepatitis B (hepatitis B surface antigen [HBsAg] positive, or positive hepatitis B core antibody [total HBcAb] confirmed by a positive viral deoxyribonucleic acid [DNA] polymerase chain reaction [PCR]) or hepatitis C (HepCAb).
32. Positive rapid plasma reagin (RPR).
33. CD4 count < 300/μL.
34. AST/SGOT or ALT/SGPT ≥2.0 Upper Limit of Normal (ULN).
35. Platelet count <100,000/μL (<100 x $10^9$/L).
36. Levels of serum IgG 18% below the LLN (for central lab IgG <4.6 g/L).
37. Levels of serum IgM 8% below the LLN (for central lab IgM <0.37 g/L).
38. Total neutrophil count <1.5 × $10^3$/μL.

*Re-testing before baseline*: in rare cases in which the screening laboratory samples are rejected by the central laboratory (example: hemolyzed sample) or the results are not assessable (example: indeterminate) or abnormal, the tests need to be repeated within 4 weeks. Any abnormal screening laboratory value that is clinically relevant should be retested in order to rule out any progressive or uncontrolled underlying condition. The last value before randomization must meet study criteria. In such circumstances, the screening period may need to be prolonged but should not exceed 8 weeks.

Please note: based on local Ethics Committees or National Competent

| | |
|---|---|
| | Authority requirements, additional diagnostic testing may be required for selected patients or selected centers to exclude tuberculosis, Lyme disease, HTLV-1 associated myelopathy (HAM), acquired immune deficiency syndrome (AIDS), hereditary disorders, connective tissue disorders, or sarcoidosis. Other specific diagnostic tests may be requested when deemed necessary by the investigator.<br><br>**Eligibility criteria for Open-Label Extension Phase:**<br>Patients who meet the following entry criteria may participate in the OLE Phase:<br><br>1. Complete the 96-week, double-blind, double-dummy treatment period, and who in the opinion of the Investigator may benefit from treatment with ocrelizumab;<br>2. Are able and willing to provide written informed consent for the OLE Phase (e.g., before the first infusion at Cycle 5) and to comply with the study protocol;<br>3. Are willing to continue to use at least two contraceptive methods;<br>4. Meet re-treatment criteria with ocrelizumab (see Protocol Section 6.1.4). |
| LENGTH OF STUDY | The study will consist of the following periods:<br>- **Screening period**: approximately 2 weeks. The screening period may be prolonged for up to 8 weeks for relevant clinical, administrative or operational reasons.<br><br>- **Double-blind, double-dummy treatment period:** 96 weeks;<br><br>The double-blind, double-dummy comparative treatment period will consist of 4 treatment cycles 24 weeks apart.<br><br>**- OLE Phase;**<br><br>The OLE Phase will continue until ocrelizumab is commercially available in the patient's country, or as per local regulation, or should the Sponsor decide to terminate the ocrelizumab program for MS, but will not exceed 4 years after the last patient to reach the Week 96 Visit in the double-blind, double-dummy treatment period.<br><br>During the OLE Phase, all patients will receive the ocrelizumab 600 mg regimen every 24 weeks. Patients who withdraw from the OLE Phase will be entered into the Safety Follow-Up Period.<br><br>- **Safety Follow-Up Period**: at least 48 weeks;<br><br>Patients who complete or withdraw from study treatment will be observed for a period of at least 48 weeks counting from the date of the last infusion of study drug. If at this time the peripheral blood B-cells remain depleted patient should continue to be monitored at 24-week intervals until B-cell count has returned to the baseline value or to the lower limit of the normal range (whichever is the lower).<br><br>See section "Procedures" below for more details. |
| END OF DOUBLE-BLIND, DOUBLE-DUMMY TREATMENT PERIOD OF THE STUDY | The end of the double-blind, double-dummy treatment period of the study is defined as the date at which the last data point during double-blind, double-dummy treatment from the last patient is received, as required for statistical analysis defined in the *Statistical* Analysis Plan (SAP). |

| | |
|---|---|
| END OF STUDY | The end of study is defined as either the last patient last visit of the OLE Phase or the last patient last visit in B-cell monitoring of Safety Follow-Up Period, whichever is later.<br><br>The OLE Phase will continue until ocrelizumab is commercially available in the patient's country, or as per local regulation, or should the Sponsor decide to terminate the ocrelizumab program for MS, but will not exceed 4 years after the last patient to reach the Week 96 Visit in the double-blind, double-dummy treatment period.<br><br>The B-cell monitoring of the Safety Follow-Up Period of each patient will last until the B-cell count has returned to the baseline value or to the lower limit of the normal range (whichever is lower). |
| INVESTIGATIONAL MEDICAL PRODUCT(S) DOSE/ ROUTE/ REGIMEN | **For the double-blind, double-dummy treatment period:**<br>– Group A (Ocrelizumab): Ocrelizumab 600 mg (given as dual infusions of ocrelizumab 300 mg on Days 1 and 15 of the first 24-week treatment cycle and as single infusions of 600 mg on Day 1 for each 24-week treatment cycle, thereafter) every 24 weeks.<br>– Group B (Interferon beta-1a (Rebif®))<br>Rebif® will be administered as follows:<br>▪ Treatment initiation:<br>⇒ during Weeks 1 and 2, Rebif® 8.8 µg (one pre-filled syringe [0.2 mL] containing 8.8 µg [2.4 MIU]) of interferon beta-1a given via subcutaneous (s.c.) injection 3 times per week<br>⇒ during Weeks 3 and 4, Rebif® 22 µg (one pre-filled syringe [0.5 mL] containing 22 µg [6 MIU] of interferon beta-1a) given via s.c. injection 3 times per week<br>▪ Treatment continuation:<br>⇒ From the fifth week onwards, Rebif® 44 µg (one pre-filled syringe [0.5 mL] containing 44 µg [12 MIU] of interferon beta-1a) given via s.c. injection 3 times per week<br>⇒ A lower dose of 22 µg, given via s.c. injection 3 times per week, will be available for patients who cannot tolerate the 44 µg dose of Rebif®. Please refer to detailed guidelines in the study protocol.<br><br>Patients randomized to active ocrelizumab group will also receive dummy placebo of Rebif® (administered via s.c. injection three times per week).<br>Patients randomized to active Rebif® group will also receive dummy placebo of ocrelizumab (administered as i.v. infusions at similar time points to those of the ocrelizumab group).<br>Dummy placebos of ocrelizumab and of Rebif® will be similar in appearance and administration as the investigational product.<br><br>**For the Open-Label Extension Phase Screening Period:**<br>– Group A: Rebif® placebo (one pre-filled syringe [0.5 mL]) given via s.c. injection 3 times per week<br>– Group B: Rebif® 44 µg (one pre-filled syringe [0.5 mL] containing 44 µg [12 MIU] of interferon beta-1a) given via s.c. injection 3 times per week<br>A lower dose of 22 µg, given via s.c. injection three times per week, will be available for patients who cannot tolerate the 44-µg dose of Rebif®. Please refer to detailed guidelines in the study protocol. |

| | |
|---|---|
| | **For the Open-Label Extension Phase:**<br>Ocrelizumab 600 mg (given as dual infusions of ocrelizumab 300 mg on Days 1 and 15 of the first 24-week treatment cycle of the OLE Phase and as single infusions of 600 mg on Day 1 for each 24-week treatment cycle, thereafter) every 24 weeks. |
| NON-INVESTIGATIONAL MEDICAL PRODUCT(S) | **During the double-blind, double-dummy treatment period:**<br>Ocrelizumab/ocrelizumab dummy placebo: Thirty minutes prior to the start of each infusion, patients will receive a methylprednisolone 100 mg i.v. infusion. Pre-infusion treatment with an oral analgesic/antipyretic (e.g., acetaminophen) and an oral antihistamine (e.g., diphenhydramine) is also recommended.<br>Rebif®/ Rebif® dummy placebo: Pre-treatment with non-steroid anti-inflammatory drugs (ibuprofen) or acetaminophen is recommended; investigators should follow their local label for further information.<br><br>**During the Open-Label Extension Phase Screening Period:**<br>Rebif®/ Rebif® dummy placebo: Pre-treatment with non-steroid anti-inflammatory drugs (ibuprofen) or acetaminophen is recommended; investigators should follow their local label for further information.<br><br>**During the Open-Label Extension Phase:**<br>Ocrelizumab: Thirty minutes prior to the start of each infusion, patients will receive a methylprednisolone 100 mg i.v. infusion. Pre-infusion treatment with an oral analgesic/antipyretic (e.g., acetaminophen) and an oral antihistamine (e.g., diphenhydramine) recommended. |

| ASSESSMENTS OF: | |
|---|---|
| - EFFICACY | – **Assessment of clinical and protocol defined relapses**<br>▪ Protocol-defined relapse is the occurrence of new or worsening neurological symptoms attributable to MS. Symptoms must persist for >24 hours and should not be attributable to confounding clinical factors (e.g., fever, infection, injury, adverse reactions to medications) and immediately preceded by a stable or improving neurological state for least 30 days. The new or worsening neurological symptoms must be accompanied by objective neurological worsening consistent with an increase of at least half a step on the EDSS scale, or 2 points on one of the appropriate FSS, or 1 point on two or more of the appropriate FSS. The change must affect the selected FSS (i.e., pyramidal, ambulation, cerebellar, brainstem, sensory, or visual). Episodic spasms, sexual dysfunction, fatigue, mood change or bladder or bowel urgency or incontinence will not suffice to establish a relapse. NB: Sexual dysfunction and Fatigue will not be scored.<br>⇨ Please note: adjudication of protocol-defined relapses will be performed by the Sponsor based on pre-specified criteria, applied to data collected by investigator, in a blinded fashion.<br>– **Brain MRI acquisition sequences**<br>▪ T1-weighted MRI (without Gd-enhancement)<br>▪ T1-weighted MRI (with Gd-enhancement)<br>▪ Fluid-attenuated, inversion recovery (FLAIR), Proton-density–weighted and/or T2-weighted MRI<br>– **Assessment of *confirmed* disability progression**<br>▪ Disability progression is defined as an increase of ≥1.0 point from the baseline EDSS score that is not attributable to another etiology (e.g., fever, concurrent illness, or concomitant medication) when the baseline score is 5.5 or less, and ≥0.5 when the baseline score is above 5.5. Disability progression is considered *confirmed* when the increase in the EDSS is confirmed at regularly scheduled visits at least 12 and 24 weeks after the initial documentation of neurological worsening.<br>▪ *Confirmed* disability progression, confirmed for both 12 and 24 weeks after the initial documentation of neurological worsening, will be analyzed.<br><br>The following instruments will be used to assess the patient's functional ability: Low Contrast Letter Acuity Charts (LCVA/Sloan charts), Symbol Digit Modalities Test (SDMT), Kurtzke Expanded Disability Status Scale (EDSS), Multiple Sclerosis Functional Composite Scale (MSFCS) and Karnofsky Performance Status Scale (clinician-reported version). |

| | |
|---|---|
| - SAFETY | Adverse events, vital signs, weight, physical and neurological examination, clinical laboratory tests, 12 lead ECG, locally reviewed MRI for safety (non-MS CNS pathology), concomitant medications.<br><br>Pregnancy tests [serum/urine beta subunit human chorionic gonadotropin (beta hCG)] will be performed in women of childbearing potential. Plasma and urine samples will be collected for JCV assessments.<br><br>Columbia-Suicide Severity Rating Scale (C-SSRS) will be used for prospective suicidality assessment. |
| - PHARMACOKINETICS/ PHARMACODYNAMICS | Blood samples will be taken for measurement of ocrelizumab serum concentration at the time points detailed in the Schedule of Assessments. Nonlinear mixed effects modeling will be used to analyze the sparse sampling dose-concentration-time data of ocrelizumab in order to assess the pharmacokinetics. Population PK parameters will be estimated and the influence of covariates, such as age, gender, weight, Human Anti-human Antibodies (HAHA), and CD19 lymphocyte count, investigated. The relationship between ocrelizumab exposure and selected safety and efficacy parameters will be analyzed and explored in order to characterize the exposure-response curve of ocrelizumab. |
| –IMMUNOGENICITY | Serum samples for measurement of of HAHA to ocrelizumab are collected according to the Schedule of Assessments. |
| – PATIENT REPORTED OUTCOMES (PRO) | ▪ Modified Fatigue Impact Scale (MFIS Standard)<br>▪ Center for Epidemiology Studies Depression Scale (CES-D3)<br>▪ Medical Outcomes Study 36-Item Short Form Survey Instrument (SF-36v2 Standard)<br>▪ EuroQol instrument (EQ-5D-3L-Tablet) |
| – PROTEIN BIOMARKER SAMPLES | Specimens for protein biomarker discovery and validation will be collected from all patients. These specimens will be used for research purposes to identify and/or verify protein biomarkers that are predictive of response to ocrelizumab treatment (in terms of dose, safety and tolerability) and will help understand the pathogenesis, course and outcome of multiple sclerosis and related diseases. In addition, screening technologies for larger numbers of proteins and antibodies may also be used to discover novel antibody associations with MS, *disability* progression and response to therapy.<br><br>Analyses will include but may not be limited to *interleukin-6*.<br><br>A 6 mL sample of whole blood will be collected in a plain tube without EDTA for serum isolation. Blood specimens for protein biomarker discovery and validation will be collected from all patients as per Schedule of Assessments. These specimens will be stored for 5 years after the end of the study and then destroyed unless a different regulation for storage time is in place at a given site. |

| | | |
|---|---|---|
| - | EXPLORATORY BIOMARKERS (non-DNA) | Roche Clinical Repository (RCR) non-DNA, (dynamic, non-inherited) RNA specimen and plasma for biomarker discovery and validation will be collected only from patients consenting to RCR as per Schedule of Assessments. RCR samples (2 x approximately 2.5 mL of blood collected in PAXgene vacutainers and 6 mL of blood collected in a tube with EDTA for plasma isolation) will be collected to promote, facilitate and improve individualized healthcare by better understanding/predicting ocrelizumab efficacy, dose responses, safety, ocrelizumab mode of action, progression of multiple sclerosis and associated diseases. These specimens may be stored for up to 15 years after the end of the study. |
| - | EXPLORATORY BIOMARKERS (DNA) | All patients who have been enrolled in the study will be asked to donate an optional DNA specimen (by written informed consent) for pharmacogenetic and genetic research.<br><br>RCR DNA sampling will involve taking one sample of 6 mL of blood *in the double-blind, double-dummy treatment phase of the study* taken as per Schedule of Assessments. The study protocol which includes RCR sampling is submitted to the concerned Ethics Committee and is available for Competent Authority review upon request. These specimens will be stored for up to 15 years after the end of the study. |
| - | OPTICAL COHERENCE TOMOGRAPHY EXPLORATORY SUBSTUDY | Optical Coherence Tomography (OCT) scans will be performed only for patients consenting to OCT substudy at certain selected centers. OCT will be performed as described in Appendix 8 of the protocol. |

**PROCEDURES (summary):**

## Figure 1: Overview of Study Design

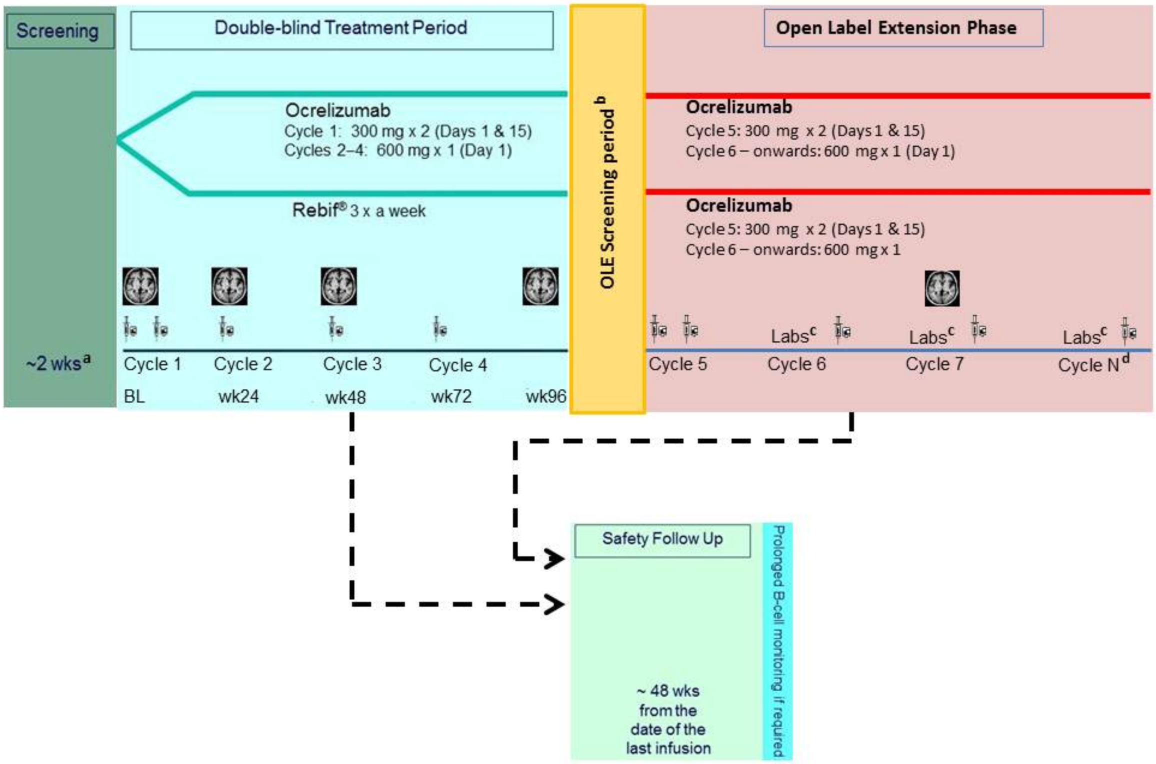


a. Screening for the study may be prolonged for up to 8 weeks for relevant clinical, administrative, or operational reasons.
b. The OLE Phase Screening Period will start after all assessments at the Week 96 Visit have been done. It will last up to 4 weeks. It is possible that for particular reasons, the OLE Phase Screening Period could be longer than 4 weeks. If a prolongation of the OLE Phase Screening Period is needed, it should be discussed with the Sponsor on a case-by-case basis. Note that during the OLE Phase Screening Period, patients should receive Rebif®/Rebif® placebo until the first infusion of Cycle 5.
c. In order to verify if patients meet re-treatment criteria, patients in the Open-Label Extension Phase of the study should come to the clinic approximately 2 weeks prior to infusions of Cycle 6, 7, etc.
d. The OLE Phase of the study can be terminated at any time (please refer to Sections 3.1.4 and 5.10). Cycle N represents a typical cycle that occurs every 24 weeks.

## <u>Screening:</u>

Consenting patients will enter a screening period to be evaluated for eligibility. The Screening period will last approximately 2 weeks, but it may be prolonged for up to 8 weeks for relevant clinical, administrative, or operational reasons. Procedures at screening will include collecting medical history, medical examination including thorough neurological exam, EDSS score, ECG, blood and urine sampling. Please see Table 4 - “Schedule of Assessments: Screening through the End of Double-Blind, Double-Dummy Treatment Period” for further details.

<u>Please note</u> that based on local Ethics Committees or National Competent Authority requirements, additional diagnostic testing may be required for selected patients or selected centers to exclude tuberculosis, Lyme disease, HTLV-1 associated myelopathy

(HAM), acquired immune deficiency syndrome (AIDS), hereditary disorders, connective tissue disorders, or sarcoidosis.

## Treatment Period:

– **Double-blind, double-dummy, comparative treatment period**

Eligible patients will be randomized via IxRS into one of two treatment groups: ocrelizumab 600 mg regimen (group A) or interferon beta-1a (Rebif®) (group B) – please refer to Table 1 and Table 2 for more details.

To prevent potential unblinding as a result of adverse events or changes to laboratory results, the following, additional measures have been implemented:

– **The Examining Investigator/EDSS assessor** will perform the neurological examination, document the FSS scores and assess EDSS scores and the Karnofsky Performance Status Scale. The examining Investigator or a qualified designee will be also responsible for performing and documenting results from: MSFCS, low contrast visual acuity testing, and the Symbol Digit Modalities Test. He or she will have access only to data from assessments listed above. The examining Investigator/EDSS assessor will not be involved with any aspect of medical management of the patient and will not have access to patient data. Every effort will be made to ensure that there is no change in the examining Investigator/EDSS assessor throughout the course of the study for any individual patient. The examining Investigator/EDSS assessors will be trained and instructed not to discuss what adverse effects (if any) the patient is experiencing from their medication. Examining Investigator/EDSS assessors will receive training in performing EDSS assessments prior to the beginning of the study and must have successfully passed an examination on performance of the Neurostatus EDSS examination within 24 months of participation. All examining Investigator/EDSS assessors will receive ongoing training on performance of the Neurostatus EDSS examination throughout the course of the study.

– **Patient education:** prior to being examined by the examining Investigator/EDSS assessor, patients will be instructed not to discuss what (if any) adverse effects they may be experiencing. Treating Investigators and/or study coordinators should remind patients of these instructions prior to EDSS assessments and this should be documented in the source documents.

– **Blinded, central MRI assessments:** a blinded, central MRI reader will assess all on-study MRI scans. These assessments will provide independent confirmation of the relative changes in immune-mediated, CNS damage.

**Blinding of laboratory parameters:** laboratory parameters which may lead to unblinding to treatment assignment, such as FACS cell counts including $CD19^+$ cells, lymphocyte count, IgM and IgG levels and type I interferon neutralizing antibody levels will be blinded in all patients. In order to ensure patients' safety in the study and to allow for assessments of the re-treatment criteria, a central laboratory will provide study investigators and Medical Monitors with reflex messages triggered by critical blinded

laboratory results. Investigators notified of their patient's critical laboratory test results will be instructed to suspend further treatment with study drug until the patient becomes eligible for re-treatment. The reflex messages from a central laboratory, together with non-blinded laboratory results, should be carefully reviewed at every visit before continuing with study treatment. The reflex messages will occur during the double-blind, double-dummy treatment period until the fifth cycle (first cycle of OLE Phase). The reflex messages will not be in effect from the sixth cycle onward. During the treatment period, patients will be assessed at clinical visits as per Schedule of Assessments: Screening through the End of Double-Blind, Double-Dummy Treatment Period – please refer to Table 4 for further details.

Prior to the next infusion of study drug, patients will be evaluated for pre-specified conditions and laboratory abnormalities to allow for re-treatment.

**Please note: patients who discontinue from study medication within the 96-week double-blind, double-dummy comparative phase (treatment period) of the study will enter the Safety Follow-Up Period (see below); they will not be eligible for the OLE Phase.**

**Open-Label Extension Phase Screening Period**

Patients who complete the 96-week treatment period may become eligible for the OLE Phase of the study. Patients will be consented for participation in the OLE Phase if, in the opinion of the Investigator, they may benefit from treatment with ocrelizumab. Patients who are not willing to participate in the OLE Phase of the study will be entered into the Safety Follow-Up Period (see below). Patient treatment allocation during the double-blind, double-dummy treatment period should not be unblinded regardless of participation in the OLE Phase.

In the case of a patient who initially declines participation in the OLE Phase and subsequently reconsiders the decision, the patient will have up to 24 weeks after the Week 96 Visit to enter the OLE Phase. In this instance, he or she should not have taken any prohibited medication. Patients who decline participation in the OLE Phase should enter the Safety Follow-Up Period.

Patients who have consented to participate in the OLE Phase will enter an OLE Phase Screening Period to be evaluated for eligibility. The OLE Phase Screening Period will start after all assessments at the Week 96 Visit have been performed. This screening period will last up to 4 weeks. It is possible that the OLE Phase Screening Period could be longer than 4 weeks. If a prolongation of the OLE Phase Screening Period is needed, it should be discussed with the Sponsor on a case-by-case basis.

Information from assessments performed during the Week 96 Visit will be utilized to verify the eligibility of the patient for the OLE Phase of the study.

During the OLE Phase Screening Period, all patients should receive Rebif®/Rebif® placebo (depending on initial arm assigned to) until the first infusion of Cycle 5.

Patients who withdraw from the OLE Phase Screening Period will be entered into the Safety Follow-Up Period (see below).

**Open-Label Extension Phase**

Duration: The OLE Phase will continue until ocrelizumab is commercially available in the patient's country, or as per local regulation, or should the Sponsor decide to terminate the ocrelizumab program for MS, but will not exceed 4 years after the last patient to reach the Week 96 Visit in the double-blind, double-dummy treatment period.

*In the United Kingdom, the OLE Phase will last for 4 years. The 4-year duration of the OLE Phase serves to evaluate long-term safety, tolerability, and efficacy of ocrelizumab treatment in patients with relapsing forms of MS.*

Treatment: During the OLE Phase, all patients will receive the ocrelizumab 600 mg regimen every 24 weeks.

Withdrawal: Patients who withdraw from the OLE Phase will be entered into the Safety Follow-Up Period (see below).

**Safety Follow-Up Period**

Patients who discontinue *prematurely* treatment for any reason during the following periods will be entered into the Safety Follow-Up Period:

- Prior to completion of the 96-week double-blind, double-dummy treatment period;
- During the OLE Phase Screening Period;
- During the OLE Phase;
- Patients who choose not to enter the OLE Phase or are not eligible for the OLE Phase after completing the 96-week, double-blind, double-dummy treatment period.

In the OLE Phase Screening Period, in the case of a patient who initially declines participation in the OLE Phase and subsequently reconsiders the decision, the patient will have up to 24 weeks after the Week 96 Visit to enter the OLE Phase. In this instance, he or she should not have taken any prohibited medication.

The Safety Follow-Up Period will last for at least 48 weeks counting from the date of the last infusion of the ocrelizumab/placebo. Safety Follow up visits will be performed at 12-week intervals starting from the date of patient's latest visit (Withdrawal from Treatment Visit). However, if after this time the peripheral blood B-cells remain depleted, patient should continue to be monitored at 24-week intervals until B-cell count has returned to the baseline value or to the lower limit of the normal range (whichever is the lower). Please refer to Figure 2 and to Section 4.4.2.1 for more details.

**Figure 2: Safety Follow-Up – Prolonged B-cell Monitoring Period**

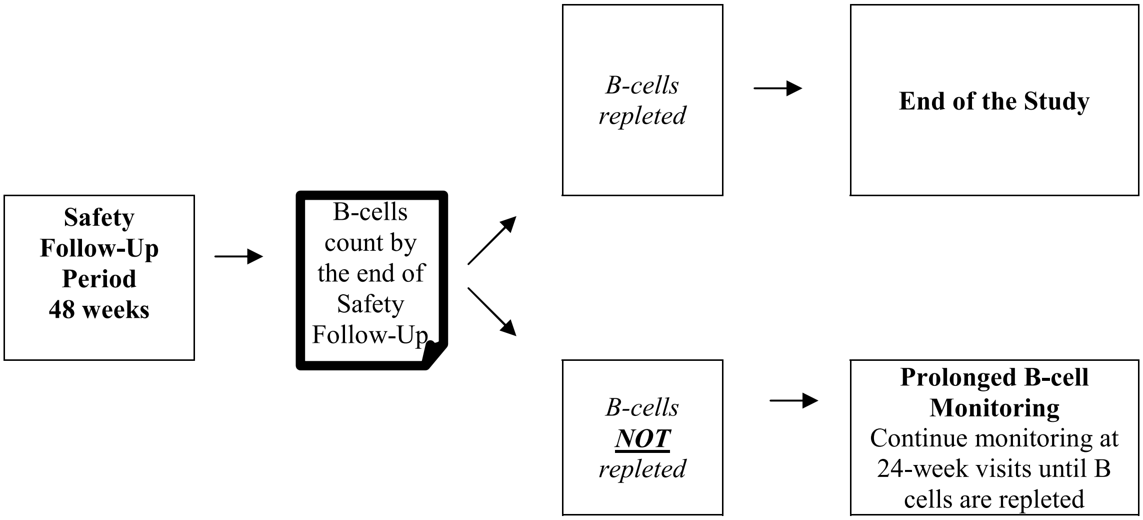


Please note: patients in Safety Follow-Up who receive other therapies that may decrease B-cell level will only be followed for 48 weeks from the date of the last infusion of the study drug regardless of their B-cell count.

During Safety Follow-Up patients will be assessed at clinical visits every 12 weeks as per Schedule of Assessments. Telephone interviews will be performed every 4 weeks. If prolonged B-cell monitoring is required, patients will be assessed at clinical visits every 24 weeks (as per Schedule of Assessments) and telephone interviews will be performed every 12 weeks. Please refer to Table 4 for further details.

Please note: It is important to distinguish between "withdrawal from treatment" and "withdrawal from study". Patients who withdraw from treatment should be encouraged to remain in the study for the full duration of the Safety Follow-Up Period (minimum of 48 weeks following the last infusion).

**Every effort should be made to have patients who withdraw from the study treatment complete the Safety Follow-Up Period and all related assessments, regardless of whether or not they receive alternative treatment for MS.**

**Table 1: Overview of Dosing Regimen *in* the Double-Blind, Double-Dummy Treatment Period**

| Study Medication | Double-Blind, Double-Dummy Treatment Period[1,2] | | | | |
|---|---|---|---|---|---|
| | 1st Cycle[3] (Weeks 1-24) | | 2nd Cycle[3] (Weeks 24-48) | 3rd Cycle[3] (Weeks 48-72) | 4th Cycle[3] (Weeks 72-96) |
| | **Day 1 Infusion** | **Day 15 Infusion** | **Week 24 Infusion** | **Week 48 Infusion** | **Week 72 Infusion** |
| **A Ocrelizumab 600 mg regimen** | **Ocrelizumab** 300 mg i.v. | **Ocrelizumab** 300 mg i.v. | **Ocrelizumab** 600 mg i.v. | **Ocrelizumab** 600 mg i.v. | **Ocrelizumab** 600 mg i.v. |
| **B Rebif® regimen[4]** | **Rebif®** s.c. 3 times per week | → | → | → | → |

i.v.=intravenous; s.c.=subcutaneous.

1. The double-blind, double-dummy treatment period consists of 96 weeks of treatment (4 treatment cycles).
2. Each treatment cycle has a duration of 24 weeks. The first cycle consists of two 300 mg ocrelizumab i.v. infusions separated by 14 days. Cycles 2 – 4 consist of a single i.v. infusion of 600 mg ocrelizumab.
3. Prior to each infusion, a clinical evaluation will be performed to ensure that the patient remains eligible for treatment.
4. Please refer to Table 2 for detailed Rebif® dosing regimen.

Please note: 100 mg of methylprednisolone i.v. will be administered in both treatment arms prior to each infusions of ocrelizumab/ocrelizumab placebo.

**Table 2: Overview of Rebif® Dosing Regimen During the Double-Blind, Double-Dummy Treatment Period**

| | **Treatment Initiation** | | **Treatment Continuation** | **Dose modification (if required)** |
|---|---|---|---|---|
| **Week** | **Weeks 1- 2** | **Weeks 3-4** | **Week 5 onwards** | – |
| Study Day | 1-14 | 15-28 | 29+ | At any time >29 |
| **Dose of Rebif®** | **Rebif® 8.8µg**<br>(one pre-filled syringe [0.2 mL] containing 2.4 MIU of interferon beta-1a) s.c.<br>**3x per week** | **Rebif® 22 µg**<br>(one pre-filled syringe [0.5 mL] containing 6 MIU of interferon beta-1a) s.c.<br>**3x per week** | **Rebif® 44 µg**<br>(one pre-filled syringe [0.5 mL]) containing 12 MIU of interferon beta-1a) s.c.<br>**3x per week** | **Rebif® 22 µg**<br>(one pre-filled syringe [0.5 mL] containing 6 MIU of interferon beta-1a) s.c.<br>**3x per week** |

IxRS=interactive voice and web response system; s.c.=subcutaneous.
Please note: As per discretion of the Treating Investigator, Rebif®/Rebif® placebo dosage can be modified due to safety reasons at any time; Investigator should follow local label for further information. If Rebif® dose modification is required due to laboratory abnormalities possibly related to the treatment with Rebif®, the Investigator (the Treating Investigator) will need to notify IxRS and the blinded study medication (Rebif® placebo or Rebif® verum) will be dispensed accordingly. In addition, to ensure patient safety in the study, unscheduled visits may be required for additional assessments, monitoring and for dispensing study medication.

**Table 3: Overview of Dosing Regimen *in* the OLE Phase Screening Period and the OLE Phase**

| Study Medication | OLE Phase Screening Period | OLE Phase[1] | | | | |
|---|---|---|---|---|---|---|
| | | 5th Cycle[2,3] | | 6th Cycle[2,3] | 7th Cycle[2,3] | Nth Cycle[2,3,4] |
| | | Day 1 Infusion | Day 15 Infusion | | | |
| **Ocrelizumab 600 mg regimen** | -.-[5] | **Ocrelizumab** 300 mg i.v. | **Ocrelizumab** 300 mg i.v. | **Ocrelizumab** 600 mg i.v. | **Ocrelizumab** 600 mg i.v. | **Ocrelizumab** 600 mg i.v. |
| **Rebif® regimen** | **Rebif®** s.c. 3 times per week[6] | -.-[7] | -.-[7] | -.-[7] | -.-[7] | -.-[7] |

i.v.=intravenous; OLE=Open-Label Extension; s.c.=subcutaneous.

1. The OLE Phase can terminate at any moment or cycle (please refer to End of Study Section 3.1.4).
2. The assessments requested for N represents the typical schedule of assessments during a cycle.
3. Prior to each infusion, a clinical evaluation will be performed to ensure that the patient remains eligible for treatment.
4. Each treatment cycle has a duration of 24 weeks. The first cycle of the OLE Phase consists of two 300 mg ocrelizumab i.v. infusions separated by 14 days. Cycle 6 onwards consists of a single i.v. infusion of 600 mg ocrelizumab.
5. During the OLE Phase Screening Period there will be no administration of ocrelizumab.
6. Please refer to Table 2 for the detailed Rebif® dosing regimen.
7. During the OLE Phase, there will be no administration of Rebif® verum or placebo.

Please note: 100 mg of methylprednisolone i.v. will be administered in both treatment arms prior to each infusion of ocrelizumab/ocrelizumab placebo.

**In case of elevation of liver function tests the following rules will apply:**

| | |
|---|---|
| ⇨ **ALT ≥ 10 x ULN**, jaundice or other clinical symptoms of liver dysfunction | In case of detection of elevated **ALT ≥ 10 x ULN**, jaundice or other clinical symptoms of liver dysfunction the injections of Rebif®/Rebif® placebo must be discontinued permanently. The monitoring of liver function tests should be continued on a monthly basis until return to normal baseline levels or CTCAE v.4.0 grade 1 toxicity (ALT: >ULN - 3.0 x ULN). A consultation with hepatologist is recommended. Patients should move to Safety Follow-Up Period. |
| ⇨ **ALT ≥ 5 x ULN** | In case of detection of elevated **ALT ≥ 5 x ULN** (but below 10 xULN) the injections of Rebif®/Rebif® placebo must be discontinued temporarily. Additional blood chemistry panel including AST, ALP, GGT and bilirubin should be performed biweekly until no further increase is observed. Subsequently, ALT analysis has to be performed every month until return to normal baseline levels or CTCAE v.4.0 grade 1 toxicity (ALT >ULN - 3.0 x ULN). A consultation with hepatologist should be considered as per investigator judgment.<br>If causes of toxicity other than possible treatment with Rebif® are excluded, the patient may then be cautiously re-challenged with Rebif®/Rebif® placebo 22µg provided in a blinded fashion upon request to IxRS. The monitoring of liver function tests should continue on a monthly basis. If there is no further recurrence of toxicity, patient may continue treatment with Rebif®/Rebif® placebo 44 µg provided in a blinded fashion upon investigator's request to IxRS. **In case of recurrence of toxicity (ALT > 3 x ULN, or other clinical symptoms of liver dysfunction) the injections of Rebif®/Rebif® placebo should be discontinued permanently.** Patients should move to Safety Follow-Up Period.<br><br><u>Please note:</u> Re-initiation of therapy with Rebif® following elevation of liver function tests can only be considered once. |
| ⇨ **ALT > 3 x ULN** | In case of detection of elevated **ALT > 3 x ULN** (but below 5x ULN) additional blood chemistry panel including AST, ALP, GGT and bilirubin should be performed biweekly until no further increase is observed. Subsequently, ALT analysis has to be performed every month until return to normal baseline levels or CTCAE v.4.0 grade 1 toxicity (ALT>ULN - 3.0 x ULN). |

## Table 4: Schedule of Assessments: Screening through the End of Double-Blind, Double-Dummy Treatment Period

| | Screen | Double-blind, Double-Dummy Treatment Period | | | | | | | | | | | | |
|---|---|---|---|---|---|---|---|---|---|---|---|---|---|---|
| **Cycle** | | 1 | | | 2 | | 3 | | 4 | | | Delayed Dosing Visit [22] | Unscheduled Visit [23] | Withdrawal from Treatment Visit |
| **Visit** | 1 | 2 BL | 3 | 4 | 5 | 6 | 7 | 8 | 9 | 10 | 11 | | | |
| **Week** | -2 | - | w2 | w12 | w24 | w36 | w48 | w60 | w72 | w84 | w96 | | | |
| *Study Day (window in days)* | *-14* | *1* | *15 (± 2)* | *85 (±4)* | *169 (± 2)* | *253 (±4)* | *337 (± 2)* | *421 (±4)* | *505 (± 2)* | *589 (±4)* | *673 (± 2)* | | | |
| Informed consent [1] | x | | | | | | | | | x[24] | | | | |
| Medical history | x | | | | | | | | | | | | | |
| Review of eligibility criteria | x | x | | | | | | | | | x[24] | | | |
| CES-D, MFIS, EQ-5D, SF-36 | | x | | | | | x | | | | x | | | x |
| Patient's Assessment of Treatment Benefit | | | | | | | x | | | | x | | | x |
| C-SSRS | | x | | x | x | x | x | x | x | x | x | | x | x |
| Physical examination | x | x | x | | x | | x | | x | | x | x | | x |
| Vital signs [2] | x | x | x | x | x | x | x | x | x | x | x | x | x | x |
| 12 lead ECG (pre- and post-dose) [3] | x | x | | | | | | | x | | | | | x |
| Height | x | | | | | | | | | | | | | |
| Weight | x | | | | | | | | x | | x | | | x |
| Neurological exam and EDSS | x | x | | x | x | x | x | x | x | x | x | | x | x |
| MSFCS, LCVA, SDMT | | x | | x | x | x | x | x | x | x | x | | x | x |
| Karnofsky Performance Status Scale | | x | | | x | | x | | x | | x | | | x |
| MRI[4] | | x | | | x | | x | | | | x | | | x |
| Concomitant Treatment | | x | x | x | x | x | x | x | x | x | x | x | x | x |
| Adverse Events | *Only SAEs* | x | x | x | x | x | x | x | x | x | x | x | x | x |
| Potential relapses recorded | | x | x | x | x | x | x | x | x | x | x | x | x | x |
| Telephone interview (every 4 wks)[5] | | | | → | → | → | → | → | → | → | → | | | |

## Table 4: Schedule of Assessments: Screening through the End of Double-Blind, Double-Dummy Treatment Period (Cont.)

| | Screen | Double-Blind, Double-Dummy Treatment Period | | | | | | | | | | | | |
|---|---|---|---|---|---|---|---|---|---|---|---|---|---|---|
| **Cycle** | | 1 | | | 2 | | 3 | | 4 | | | Delayed Dosing Visit [22] | Unscheduled Visit [23] | Withdrawal from Treatment Visit |
| **Visit** | 1 | 2 BL | 3 | 4 | 5 | 6 | 7 | 8 | 9 | 10 | 11 | | | |
| **Week** | -2 | - | w2 | w12 | w24 | w36 | w48 | w60 | w72 | w84 | w96 | | | |
| *Study Day (window in days)* | *-14* | *1* | *15 (±2)* | *85 (±4)* | *169 (± 2)* | *253 (±4)* | *337 (± 2)* | *421 (±4)* | *505 (± 2)* | *589 (±4)* | *673 (± 2)* | | | |
| Pregnancy test [6] | x | x | x | x | x | x | x | x | x | x | x | x | | x |
| Antibody Titers [7] | | x | | x | x | | x | | x | | x | | | x |
| RCR (non-DNA) [8] | | x | | x | x | | x | | x | | x | | | x |
| RCR (DNA) [9] | | x | | | | | | | | | | | | |
| Protein biomarker sampling [10] | | x | | x | x | | x | | x | | x | | | x |
| HAHA [11] | | x | | | x | | x | | x | | x | | | x |
| Plasma/ urine banking for JCV[12] | | x | | x | x | x | x | x | x | x | x | | | x |
| PK Samples [13] | | x | | | x | | x | | x[13] | x | x | | | x |
| Thyroid function tests [14] | x | | | | x | | x | | x | | x | | | x |
| FSH [15] | x | | | | | | | | | | | | | |
| Hepatitis Screening [16] | x | | | | | | | | | | | | | |
| Hepatitis B virus DNA[16] | x | (x) | | (x) | (x) | (x) | (x) | (x) | (x) | (x) | (x) | | | (x) |
| RPR | x | | | | | | | | | | | | | |
| CD4 count | x | | | x | | x | | x | | x | | | | |
| IgG | | | | x | | x | | x | | x | | | | |
| Total Ig, IgA, IgG, IgM | x | | | | x | | x | | x | | x | | | x |
| FACS [17] | | x | x | x | x | | x | | x | | x | | | x |

## Table 4: Schedule of Assessments: Screening through the End of Double-Blind, Double-Dummy Treatment Period (Cont.)

| | Screen | Double-Blind, Double-Dummy Treatment Period | | | | | | | | | | | | |
|---|---|---|---|---|---|---|---|---|---|---|---|---|---|---|
| **Cycle** | | 1 | | | 2 | | 3 | | 4 | | | | | |
| **Visit** | 1 | 2 BL | 3 | 4 | 5 | 6 | 7 | 8 | 9 | 10 | 11 | Delayed Dosing Visit [22] | Unscheduled Visit [23] | Withdrawal from Treatment Visit |
| **Week** | -2 | - | w2 | w12 | w24 | w36 | w48 | w60 | w72 | w84 | w96 | | | |
| *Study Day (window in days)* | *-14* | *1* | *15 (± 2)* | *85 (±4)* | *169 (± 2)* | *253 (±4)* | *337 (± 2)* | *421 (±4)* | *505 (± 2)* | *589 (±4)* | *673 (± 2)* | | | |
| Routine safety lab [18] | x | x | x | x | x | x | x | x | x | x | x | | | x |
| Type I interferon neutralizing antibody assay [19] | | x | | | x | | x | | x | | x | | | x |
| Pre-treatment with i.v. methylprednisolone [20] | | x | x | | x | | x | | x | | | x | | |
| Administration of i.v. ocrelizumab / ocrelizumab placebo [21] | | x | x | | x | | x | | x | | | x | | |
| Assessment of s.c. Rebif®/ Rebif® placebo compliance | | x | x | x | x | x | x | x | x | x | x | | x | x |
| Administration of s.c. Rebif® / Rebif® placebo 3x/wk | | x | x | x | x | x | x | x | x | x | x[25] | | | |

1. β-hCG=beta human chorionic gonadotropin; BL=baseline; CD4=cluster of differentiation; CES-D=Center for Epidemiologic Studies Depression Scale; C-SSRS=Columbia-Suicide Severity Rating Scale; DNA=deoxyribonucleic acid; ECG=electrocardiogram; eCRF=electronic case report form; EDSS=Expanded Disability Status Scale; EDTA=ethylenediaminetetraacetic acid; EQ-5D=EuroQoL; FACS=fluorescence activated cell sorting; FSH=follicle-stimulating hormone; HAHA=human anti-human antibodies; Ig=immunoglobulin; IgA=immunoglobulin A; IgG=immunoglobulin G; IgM=immunoglobulin M; i.v.=intravenous; JCV=JC virus; LCVA=Low Contrast Visual Acuity; MFIS=Modified Fatigue Impact Scale; MRI=magnetic resonance imaging; MSFCS=Multiple Sclerosis Functional Composite Scale; n=number; OLE=Open-Label Extension; PK=pharmacokinetic; RCR=Roche Clinical Repository; RNA=ribonucleic acid; RPR=rapid plasma reagin; SAE=serious adverse event; s.c.=subcutaneous; SDMT=Symbol Digit Modalities Test; SF-36=Short-Form 36; w=week.

**Table 4: Schedule of Assessments: Screening through the End of Double-Blind, Double-Dummy Treatment Period (Cont.)**

1. **Informed Consent** must be obtained in written form from all patients at screening (prior to any study–related procedure) in order to meet eligibility for the study.
2. **Vital signs** (i.e., pulse rate, systolic and diastolic blood pressure, respiration rate and temperature) will be obtained while the patient is in the semi supine position (after 5 minutes). On infusion visits, the vital signs should be taken within 45 minutes prior to the methylprednisolone infusion in all patients. In addition, vital signs should be obtained prior to ocrelizumab/ocrelizumab placebo infusion, then every 15 minutes (± 5 minutes) for the first hour; then every 30 minutes (± 10 minutes) until 1 hour after the end of the infusion. On non-infusion days, the vital signs may be taken at any time during the visit.
3. **ECG (pre- and post-dose):** on infusion visits ECG should be taken within 45 minutes prior to the methylprednisolone infusion in all patients, and within 60 minutes after completion of the ocrelizumab/ocrelizumab placebo infusion. On non-infusion days, the ECG may be taken at any time during the visit.
4. **MRI**: brain MRI scans *should occur within a window or ± 4 weeks of the scheduled visit. Also, brain MRI scans* will be obtained in patients withdrawn from the treatment period (at a withdrawal visit) if not performed during last 4 weeks.
5. **A structured telephone interview** will be conducted by site personnel every 4 weeks (± 3 days) from Week 8 through the study to identify any new or worsening neurological symptoms that warrant an unscheduled visit and collect data on possible events of infections.
6. **Serum β-hCG** must be performed at screening in women of childbearing potential. Subsequently, urine β-hCG [sensitivity of at least 25 mIU/mL] will be performed. On infusion visits, the urine pregnancy test should be performed prior to methylprednisolone infusion in all women of child-bearing potential. If positive, the patient will not receive the scheduled dose and confirmation, a serum pregnancy test, will be performed.
7. **Antibody Titers**: measurement of antibody titers against common antigens (mumps, rubella, varicella and *Streptococcus pneumoniae*) will be performed.
8. **RCR - Roche Clinical Repository non-DNA (RNA – and protein):** for RNA 2x 2.5 mL whole blood samples to be collected from consenting patients only for expression profiling analysis. For protein: 6 mL blood samples in EDTA tube for plasma samples will be collected from consenting patients only for analysis of protein biomarkers. On infusion visits, ALL samples should be collected 5-30 minutes prior to methylprednisolone infusion.
9. **RCR - Roche Clinical Repository (DNA):** 6 mL whole blood sample to be collected from only from patients consenting to RCR for pharmacogenetic and genetic analysis. If not done at Baseline (Visit 2), sample may be collected at next visit.
10. **Protein biomarker sampling:** one serum sample (6 mL) will be collected from all patients for analysis of protein biomarkers. On infusion visits, samples should be collected 5-30 minutes prior to methylprednisolone infusion.
11. **HAHA**: On infusion visits, serum samples are collected 5-30 minutes prior to the methylprednisolone infusion.
12. **Plasma and urine samples for JCV will be collected** at specified time points and analyzed in batches, if decided by the Sponsor.

## Table 4: Schedule of Assessments: Screening through the End of Double-Blind, Double-Dummy Treatment Period (Cont.)

13. **PK samples:** on the infusion day at week 72, two serum samples should be collected, one 5-30 minutes prior to the methylprednisolone infusion and the second one 30 minutes (±10 minutes) following the completion of the ocrelizumab/ocrelizumab placebo infusion. For all other infusion visits, a blood sample should be collected 5 – 30 minutes before the methylprednisolone infusion. At other times (non-infusion visits) samples may be collected at any time during the visit.
14. **Sensitive thyroid-stimulating hormone (sTSH)** will be tested at screening and during the double-blind, double-dummy treatment period. Thyroid autoantibodies will be assayed only at screening.
15. **FSH**: only applicable to women to confirm the postmenopausal status.
16. **Hepatitis** screening & monitoring: all patients must have negative hepatitis B surface antigen (HbsAg) result and negative hepatitis C antibody (HepCAb) screening tests prior to enrollment. If total hepatitis B core antibody (HbcAb) is positive at screening, HB virus DNA measured bypolymerase chain reaction (PCR) must be negative to be eligible. For those patients enrolled with negative HBsAg and positive total HBcAb, HB virus DNA (PCR) must be repeated every 12 weeks during the treatment period.
17. **FACS**: including CD19 and other circulating B-cell subsets, T-cells, natural killer cells, and other leukocytes. On infusion visits, blood samples should be collected prior to the infusion of methylprednisolone.
18. **Routine safety lab:** hematology, chemistry and urinalysis: on infusion visits, all urine and blood samples should be collected prior to the infusion of methylprednisolone. At other times, samples may be collected at any time during the visit.
19. **Type I interferon neutralizing antibody assay:** At baseline (visit 2), sample should be collected before first Rebif®/Rebif® placebo injection. At subsequent visits, samples should be collected at least 36 hours following last injection of Rebif®/Rebif® placebo.
20. All patients receive **prophylactic treatment** with 100 mg of methylprednisolone i.v. prior to infusion of ocrelizumab /ocrelizumab placebo. In the rare case when the use of methylprednisolone is contraindicated for the patient, use of an equivalent dose of an alternative steroid should be used as premedicationprior to the infusion. It is also recommended that patients receive an analgesic/antipyretic such as acetaminophen/paracetamol (1 g) and an i.v. or oral antihistaminic such as diphenhydramine 50 mg 30-60 minutes prior to ocrelizumab/ ocrelizumab placebo.
21. **Administration (infusion) of i.v. ocrelizumab/ocrelizumab placebo**: the Treating Investigator must review the clinical and laboratory re-treatment criteria prior to subsequent infusion of ocrelizumab/ocrelizumab placebo.
22. **A delayed dosing visit** will be performed and recorded in the Delayed Dosing Visit eCRF form when dosing cannot be administered at the scheduled dosing visit. Other tests or assessments may be done as appropriate.

## Table 4: Schedule of Assessments: Screening through the End of Double-Blind, Double-Dummy Treatment Period (Cont.)

23. **Unscheduled Visit:** assessments performed at unscheduled (non-dosing) visits will depend on the clinical needs of the patient. All patients with new neurological symptoms suggestive of relapse should have EDSS performed by examining Investigator, *whenever possible within 7 days of the onset of the relapse*. Other tests/assessments may be done as appropriate. Please note: in case of alanine aminotransferase (ALT) elevations dose modification should be necessary, additional visits may be required for dispensing of study medication.
24. At the Week 84 Visit, a discussion with the patient regarding participation in the Open-Label Extension Phase should occur if the Treating Investigator is of the opinion that the patient may benefit from treatment with ocrelizumab. An informed consent for the OLE Phase should be provided to the patient. It is recommended that the Informed Consent Form of the Open-Label Extension Phase be signed at the Week 96 Visit.
25. If the patient enters OLE Phase Screening Period, Rebif® / Rebif® placebo should be provided to the patient at the Week 96 Visit (please see Section 5.10.1).

Please note: based on local Ethics Committees or National Competent Authority requirements, additional diagnostic testing may be required for selected patients or selected centers to exclude tuberculosis, Lyme disease, HTLV-1 associated myelopathy (HAM), acquired immune deficiency syndrome (AIDS), hereditary disorders, connective tissue disorders, or sarcoidosis. Other specific diagnostic tests may be requested when deemed necessary by the Investigator**.**

## Table 5: Schedule of Assessments: Safety Follow-Up (including prolonged B-cell monitoring if required)

| | **Safety Follow-Up** | **Prolonged B-cell Monitoring[1]** | **End of observation or withdrawal from Safety Follow up** |
|---|---|---|---|
| **Assessments** | *Visits every 12 weeks (±7 days)* [2] | *Visits every 24 weeks (±7 days)* | |
| Urine pregnancy test | x | x | x |
| Routine Safety Labs[3] | x | x | x |
| FACS [4] | x | x | x |
| Total Ig, IgA, IgG, IgM | x[10] | x | x |
| HAHA [5] | x[10] | x | x |
| Plasma/urine banking for JCV | x | x | x |
| Antibody titers | x[10] | x | x |
| Hepatitis B viral DNA[6] | (x) | (x) | (x) |
| Protein biomarker sampling [8] | x[10] | x | x |
| Vital Signs | x | x | x |
| EDSS | x | | x |
| Neurological examination | x | x | x |
| Physical examination | x[10] | x | x |
| Potential relapses recorded | x | x | x |
| Adverse events | x | x | x |
| Concomitant Medication | x | x | x |
| Telephone interview [9] | x | x | |

CD=cluster of differentiation; DNA=deoxyribonucleic acid; EDSS=Expanded Disability Status Scale; EDTA=ethylenediaminetetraacetic acid; FACS=fluorescence activated cell sorting; HAHA=human anti-human antibodies; HbcAb=hepatitis C antibody; HbsAg=hepatitis B surface antigen; HBV=hepatitis B virus; Ig=immunoglobulin; IgA=immunoglobulin A; IgG=immunoglobulin G; IgM=immunoglobulin M; i.v.=intravenous; JCV=JC virus; RNA=ribonucleic acid.

## Table 5: Schedule of Assessments: Safety Follow-Up (including prolonged B-cell monitoring if required) (Cont.)

1. **Prolonged B-cell monitoring**: patients whose B-cells have not been repleted after 48 weeks of Safety Follow-Up Period will continue with visits every 24 weeks (± 7 days) until B-cell repletion.
2. **Safety Follow-Up** will be carried out for at least 48 weeks starting from the date of last infusion of ocrelizumab. Visits will be performed at 12-week intervals starting from the date of the patient's Withdrawal From Treatment Visit. *Safety Follow-Up applies to study patients who have completed the blinded treatment period (or open label treatment period, if applicable) and to patients who withdraw early from treatment. If B-cells have returned to normal levels at this visit, then the 48 week Safety Follow-up visit will become the End of Observation visit and the patient will have completed the study. An End of observation visit will be performed only in case of occurrence of new adverse event and/or if the investigator considers the prior safety assessment (laboratory, other) as abnormal and clinically significant.*
3. **Routine safety lab**: hematology, chemistry and urinalysis.
4. **FACS** including CD19 and other circulating B-cell subsets, T cells, natural killer cells and other leukocytes.
5. **HAHA**: two serum samples are required.
6. **Hepatitis monitoring:** hepatitis to be monitored only in patients with screening results of HbsAg negative, HBcAb positive and HBV DNA negative, inclusive.
7. **Protein biomarker sampling**: 6 mL blood sample in a plain tube without EDTA for serum isolation will be collected from all patients for analysis of protein biomarkers.
8. **A structured telephone interview** will be performed by site personnel every 4 weeks (± 3 days) between visits until 48 weeks after the last infusion to identify any new or worsening neurological symptoms that warrant an unscheduled visit and collect data on possible events of infections. If prolonged B-cell monitoring is required beyond 48 weeks after the last infusion, telephone interviews will be done every 12 weeks (± 7 days) between visits.
9. Needs to be assessed only every 24 weeks.

Please note: patients in Safety Follow-Up who receive other B-cell targeted therapies will only be followed for 48 weeks from the date of the last infusion of the study drug regardless of their B-cell count.

## Table 6: Schedule of Assessments: Open Label Extension Phase

| | OLE Screen | Open-Label Extension Phase[1] | | | | | | | | | | | Delayed dosing Visit[20] | Unscheduled Visit[21] | Withdrawal from Treatment Visit |
|---|---|---|---|---|---|---|---|---|---|---|---|---|---|---|---|
| Cycle[1] | | 5 | | | 6[1] | | 7[1] | | 8[1] | | N[1,3] | | | | |
| Visit | | 12 | 13 | 14 | 15 | 16 | 17 | 18 | 19 | 20 | … | … | | | |
| Week in OLE Phase (window in days) | | *0*[2] | *2* (± 2) | *12* (± 7) | *22* (± 7) | *24* (±5) | *46* (± 7) | *48* (± 5) | *70* (± 7) | *72* (± 5) | *n-2 wk* (± 7) | *n* (± 7) | | | |
| Informed consent[4] | x | | | | | | | | | | | | | | |
| Review of eligibility criteria | x | x | | | | | | | | | | | | | |
| Review of retreatment criteria | | x | x | | | x | | x | | x | | x | x | | |
| Administration of s.c. Rebif® / Rebif® placebo 3x/wk [5] | x | | | | | | | | | | | | | | |
| Assessment of s.c. Rebif®/ Rebif® placebo compliance | | x | | | | | | | | | | | | | |
| Pre-treatment with i.v. methylprednisolone[6] | | x | x | | | x | | x | | x | | x | x | | |
| Administration of i.v. ocrelizumab[7] | | x | x | | | x | | x | | x | | x | x | | |
| Physical examination | | x | x | | | x | | x | | x | | x | x | | x |
| Vital signs, weight[8] | | x | x | x | | x | | x | | x | | x | x | x | x |
| 12-lead ECG (pre- and post-dose, once early)[9] | | x | x | | | | | | | x | | (x) | | | x |
| Neurological exam and EDSS | | x | | x | | x | | x | | x | | x | | x | x |
| Routine safety labs[10] | | x | | x | x | | x | | x | | x | | | x | x |
| Adverse events | | x | x | x | x | x | x | x | x | x | x | x | x | x | x |
| Concomitant treatments | | x | x | x | x | x | x | x | x | x | x | x | x | x | x |
| Potential relapses recorded | | x | x | x | x | x | x | x | x | x | x | x | x | x | x |
| Pregnancy test[11] | | x | x | x | | x | | x | | x | | x | x | | x |
| Antibody titers[12] | | x | | x | x | | x | | x | | x | | | | x |
| Total Ig, IgA, IgG, IgM | | x | | | x | | x | | x | | x | | | | |
| CD4 count | | x | x | | x | | x | | x | | x | | | | |

## Table 6: Schedule of Assessments: Open Label Extension Phase (Cont.)

| Cycle[1] / Visit / Week in OLE Phase (window in days) | OLE Screen | Open-Label Extension Phase[1] | | | | | | | | | | | Delayed dosing Visit[20] | Unscheduled Visit[21] | Withdrawal from Treatment Visit |
|---|---|---|---|---|---|---|---|---|---|---|---|---|---|---|---|
| Cycle[1] | | 5 | | | 6[1] | | 7[1] | | 8[1] | | N[1,3] | | | | |
| Visit | | 12 | 13 | 14 | 15 | 16 | 17 | 18 | 19 | 20 | … | … | | | |
| Week in OLE Phase (window in days) | | *0*[2] | *2* (± 2) | *12* (± 7) | *22* (± 7) | *24* (±5) | *46* (± 7) | *48* (± 5) | *70* (± 7) | *72* (± 5) | *n-2 wk* (± 7) | *n* (± 7) | | | |
| HAHA[22] | | x | | | x | | x | | x | | x | | | | *x* |
| *Ocrelizumab concentration sample*[22] | | *x* | | | *x* | | *x* | | *x* | | *x* | | | | *x* |
| Telephone interview (every 4 weeks)[13] | | | | - - - | - - - | - - - | - - - | - - - | - - - | - - - | - - - | → | | | |
| EQ-5D (once yearly) | | x | | | | | x | | | | (x) | | | | |
| Protein biomarker sampling[15] | | x | | x | x | | x | | x | | x | | | | |
| FACS[16] | | | x | x | x | | x | | x | | x | | | | |
| Hepatitis B virus DNA[17] | | | | (x) | (x) | | (x) | | (x) | | (x) | | | | |
| MRI (once yearly)[18] | | | | | | | x | | | | (x) | | | | |
| Patient's Assessment of Treatment Benefit (once yearly) | | | | | | | x | | | | (x) | | | | |
| Plasma/urine banking for JCV[19] | | x | | x | x | | x | | x | | x | | | | x |

CD4 = cluster of differentiation; DNA = deoxyribonucleic acid; ECG = electrocardiogram; eCRF = electronic case report form; EDSS = Expanded Disability Status Scale; EQ-5D = EuroQoL; FACS = fluorescence activated cell sorting; HAHA = human anti-human antibodies; Ig = immunoglobulin; IgA = immunoglobulin A; IgG = immunoglobulin G; IgM = immunoglobulin M; i.v. = intravenous; JCV = JC virus; MRI = magnetic resonance imaging; n = number; OLE = Open-Label Extension; RCR = Roche Clinical Repository; s.c. = subcutaneous.

1. **The OLE Phase** can terminate at any moment or cycle (please refer to End of Study Section 3.1.4). In case the study is ended, a Withdrawal from Treatment Visit should occur.
2. **The OLE Phase** starts at the first infusion of Cycle 5. The OLE Phase Screening Period will start after all assessments at the Week 96 Visit have been performed, and it should last approximately 4 weeks. It is possible that the OLE Phase Screening Period could be longer than 4 weeks. If a prolongation of the OLE Phase Screening Period is needed, it should be discussed with the Sponsor on a case-by-case basis.
3. The assessments requested for N represent the typical schedule of assessments during a cycle.
4. The **informed consent** should have been provided to the patient at the Week 84 Visit and signed by the patient at the Week 96 Visit.

## Table 6: Schedule of Assessments: Open Label Extension Phase (Cont.)

5. During the OLE Phase Screening Period, s.c. administration Rebif® / Rebif® placebo 3 times per week should occur until one day prior to the first infusion of ocrelizumab of Cycle 5 (beginning of OLE Phase). If during this period, the patient decides not to participate in the OLE Phase, then administration of Rebif® / Rebif® placebo 3 times per week should stop immediately, and the patient will be entered in the Safety Follow-Up Period.
6. All patients receive prophylactic treatment with 100 mg of methylprednisolone i.v. prior to infusion of ocrelizumab. In the rare case when the use of methylprednisolone is contraindicated for the patient, use of an equivalent dose of an alternative steroid should be used as premedication prior to the infusion. It is also recommended that patients receive an analgesic/antipyretic such as acetaminophen/paracetamol (1 g) and an i.v. or oral antihistaminic such as diphenhydramine 50 mg 30-60 minutes prior to ocrelizumab.
7. **Administration (infusion) of i.v. ocrelizumab**: the Investigator must review the clinical and laboratory re-treatment criteria prior to subsequent infusion of ocrelizumab.
8. **Vital signs** (i.e., pulse rate, systolic and diastolic blood pressure, respiration rate, and temperature) will be obtained while the patient is in the semi-supine position (after 5 minutes). Vital signs should be collected within 45 minutes prior to the methylprednisolone infusion in all patients. In addition, vital signs should be obtained prior to ocrelizumab infusion, then every 15 minutes (± 5 minutes) for the first hour; then every 30 minutes (± 10 minutes) until 1 hour after the end of the infusion.
9. **ECGs** (pre- and post-dose): ECG should be performed within 45 minutes prior to the methylprednisolone infusion in all patients, and within 60 minutes after completion of the ocrelizumab infusion.
10. **Routine safety lab**: hematology, chemistry, and urinalysis. On infusion visits at Cycle 5, all urine and blood samples should be collected prior to the infusion of methylprednisolone. At other times, samples may be collected at any time during the visit.
11. **Urine β-hCG** [sensitivity of at least 25 mIU/mL] will be performed. On infusion visits, the urine pregnancy test should be performed prior to methylprednisolone infusion in all women of childbearing potential. If positive, the patient will not receive the scheduled dose and for confirmation a serum pregnancy test will be performed.
12. **Antibody Titers**: measurement of antibody titers against common antigens (mumps, rubella, varicella, and Streptococcus pneumoniae) will be performed.
13. A structured **telephone interview** will be conducted by site personnel every 4 weeks (± 3 days) from Cycle 5 *(Week 8 of OLE Phase)* through the study to identify any new or worsening neurological symptoms that warrant an unscheduled visit and collect data on possible events of infections.
14. **Protein biomarker sampling**: one serum sample (6 mL) will be collected from all patients for analysis of protein biomarkers. On infusion visits, samples should be collected 5-30 minutes prior to methylprednisolone infusion.
15. **FACS**: including CD19 and other circulating B-cell subsets, T-cells, natural killer cells, and other leukocytes.
16. **Hepatitis monitoring**: For those patients enrolled with negative HBsAg and positive total HBcAb, HB virus DNA (PCR) must be repeated every 24 weeks during the treatment period.
17. **MRI**: brain MRI scans *should occur within a window of ± 4 weeks of the scheduled visit. Also, brain MRI scans* will be obtained in patients withdrawn from the OLE Phase (at a withdrawal visit) if not performed during last 4 weeks.
18. **Plasma and urine samples for JCV** will be collected at specified time points
19. A **delayed dosing visit** will be performed and recorded in the Delayed Dosing Visit eCRF form when dosing cannot be administered at the scheduled dosing visit. Other tests or assessments may be done as appropriate.

### Table 6: Schedule of Assessments: Open Label Extension Phase (Cont.)

20. **Unscheduled Visit**: assessments performed at unscheduled (non-dosing) visits will depend on the clinical needs of the patient. All patients with new neurological symptoms suggestive of relapse should have EDSS performed*, whenever possible within 7 days of the onset of the relapse*. Other tests/assessments may be done as appropriate.
21. ***HAHA and Ocrelizumab drug concentration samples**: At the infusion Visit 12 (Cycle 5), a blood sample should be collected before the methylprednisolone infusion. At non-infusion visits, samples may be collected at any time during the visit.*

## SAMPLE SIZE AND STATISTICAL ANALYSES

The sample size for this study has been estimated based on data from previous RRMS trials, with the use of two-sided tests with an experiment-wise alpha of 0.05. The annualized rate of relapse among patients receiving ocrelizumab at 96 weeks is predicted to be 0.165 (standard deviation of approximately 0.60), as compared with 0.33 (standard deviation of approximately 0.80) among patients receiving the control treatment, Rebif® (this represents a relative reduction of 50% on ocrelizumab compared to the active comparator). For the annualized relapse rate, a t-test has been used to determine the sample size between ocrelizumab and the control arm. The sample size of 400 patients per arm provides 84 percent power, maintaining the type I error rate of 0.05, and assuming a dropout rate of 20 percent approximately (assuming relative reduction among patients drop out is 25%). For *confirmed disability* progression, a two group test of equal exponential survival with exponential dropout is used to determine the sample size. Assuming the 2 year *confirmed* disability progression rate is 18% for the Rebif® arm and 12.6% for the ocrelizumab arm (this represents a relative reduction of 30% on ocrelizumab compared to the active comparator), and assuming a dropout rate of 20 percent over 2 years approximately, the sample size of 400 per arm will provide 80 percent power, maintaining the type I error rate of 0.05 based on the pooled analysis of two identical RMS trials (800 patients treated with ocrelizumab 600 mg and 800 patients treated with Rebif®).

All eligible patients will be randomized to treatment (with 1:1 ratio to ocrelizumab 600 mg regimen or the control arm) stratified by region (US versus ROW) and baseline EDSS (<4.0 versus ≥4.0).

The primary efficacy endpoint is the annualized relapse rate by 96 weeks. The annualized relapse rates at 96 weeks will be *analyzed* using negative binomial model, adjusting for region (United States versus ROW) and baseline EDSS (<4.0 versus ≥4.0). The adjusted annualized relapse rates and the 95% confidence intervals for the relapse rates will be presented along with the p-value.

Summaries of safety data will be produced using data from all patients who have received any study treatment.

## Table of Contents

1. Background and Rationale . . . . . . . . . . 52
1.1 Background . . . . . . . . . . 52
1.1.1 Multiple Sclerosis . . . . . . . . . . 52
1.1.2 Ocrelizumab . . . . . . . . . . 53
1.1.3 Rationale for Targeting B-cells in MS . . . . . . . . . . 54
1.1.4 Sponsor Experience with Anti-CD20 Compounds in MS . . . . . . . . . . 55
1.1.4.1 Ocrelizumab in RRMS . . . . . . . . . . 55
1.1.4.2 Long-Term Results of Phase II Study WA21493/ACT4422G . . . . 57
1.1.4.3 Rituximab in RRMS . . . . . . . . . . 58
1.1.4.4 Rituximab in PPMS . . . . . . . . . . 59
1.1.5 Rebif® . . . . . . . . . . 60
1.2 Rationale for the Study . . . . . . . . . . 62

2. Objectives . . . . . . . . . . 63
2.1 Primary Objective . . . . . . . . . . 63
2.2 Secondary Objectives . . . . . . . . . . 63
2.3 Exploratory Objectives . . . . . . . . . . 64
2.4 Roche Clinical Repository Exploratory Objectives . . . . . . . . . . 65
2.5 Optional Exploratory Substudies . . . . . . . . . . 65
2.5.1 Optical Coherence Tomography Exploratory Substudy . . . . . . . . . . 65

3. Study Design . . . . . . . . . . 66
3.1 Overview of Study Design and Dosing Regimen . . . . . . . . . . 66
3.1.1 Rationale for Study Design . . . . . . . . . . 72
3.1.2 Rationale for Dose Selection . . . . . . . . . . 75
3.1.3 End of Double-Blind, Double-Dummy Treatment Period of the Study . . . . . . . . . . 76
3.1.4 End of Study . . . . . . . . . . 76
3.2 Number of Subjects / Assignment to Treatment Groups . . . . . . . . . . 76
3.3 Centers . . . . . . . . . . 77

4. Study Population . . . . . . . . . . 77
4.1 Overview . . . . . . . . . . 77
4.1.1 Recruitment Procedures . . . . . . . . . . 77

4.2 Inclusion Criteria . . . . . . . . . . 77
4.3 Exclusion Criteria . . . . . . . . . . 78
4.4 Eligibility Criteria for Open-Label Extension Phase . . . . . . . . . . 81
4.5 Concomitant Medication and Treatment . . . . . . . . . . 81
4.5.1 Definition of Concomitant Treatment . . . . . . . . . . 81
4.5.2 Treatment for Symptoms of Multiple Sclerosis . . . . . . . . . . 82
4.5.2.1 Prohibited Concomitant Treatments . . . . . . . . . . 82
4.5.3 Immunization . . . . . . . . . . 82
4.6 Criteria for Premature Withdrawal . . . . . . . . . . 83
4.6.1 Withdrawal of Subjects from the Roche Clinical Repository . . . . . . 85
4.6.2 Patient Agreement for Continuation in the Study (in Case of Confirmed Disability Progression) . . . . . . . . . . 85
4.7 Replacement Policy (Ensuring Adequate Numbers of Evaluable Subjects) . . . . . . . . . . 85
4.7.1 For Subjects . . . . . . . . . . 85
4.7.2 For Centers . . . . . . . . . . 85

5. Schedule of Assessment and Procedures . . . . . . . . . . 86

5.1 Screening Examination and Eligibility Screening Form . . . . . . . . . . 98
5.2 Procedures for Enrollment of Eligible Subjects . . . . . . . . . . 98
5.3 Clinical Assessments and Procedures During the Double-Blind, Double-Dummy Treatment Period . . . . . . . . . . 99
5.3.1 Overview of Clinical Visits During the Double-Blind, Double-Dummy Treatment Period . . . . . . . . . . 99
5.3.1.1 Delayed Dosing Visit . . . . . . . . . . 100
5.3.1.2 Unscheduled Visits . . . . . . . . . . 100
5.3.1.3 Withdrawal Visits . . . . . . . . . . 101
5.3.2 Assessment of Efficacy . . . . . . . . . . 101
5.3.2.1 Assessment of Relapse . . . . . . . . . . 101
5.3.2.2 Assessment of Disability . . . . . . . . . . 102
5.3.2.3 Kurtzke Expanded Disability Status Scale . . . . . . . . . . 102
5.3.2.4 The Multiple Sclerosis Functional Composite Scale . . . . . . . . . . 102
5.3.2.5 Low-Contrast Visual Acuity Testing . . . . . . . . . . 103
5.3.2.6 The Symbol Digit Modalities Test . . . . . . . . . . 103
5.3.3 Brain Magnetic Resonance Imaging . . . . . . . . . . 103

5.3.4 Safety . . . . . 104
5.3.4.1 Electrocardiogram . . . . . 105
5.3.4.2 Physical Examination . . . . . 105
5.3.4.3 Neurological Examination . . . . . 105
5.3.4.4 Telephone Interviews . . . . . 105
5.3.4.5 Columbia-Suicide Severity Rating Scale . . . . . 106
5.3.5 The Karnofsky Performance Scale (Clinician-Reported Version) . . . 106
5.4 Laboratory Assessments . . . . . 106
5.4.1 Standard Laboratory Assessments . . . . . 107
5.4.2 Hepatitis Screening and Liver Function Monitoring . . . . . 108
5.4.3 Plasma and Urine Banking for JC Virus . . . . . 109
5.4.4 Pharmacokinetic/Pharmacodynamic Assessments . . . . . 109
5.4.5 Type I Interferon Neutralizing Antibody Assay . . . . . 109
5.5 Roche Clinical Repository Specimen(s) . . . . . 109
5.5.1 Specimen Types . . . . . 110
5.6 Protein Biomarker Samples . . . . . 111
5.7 Patient Reported Outcome(s) . . . . . 111
5.7.1 Modified Fatigue Impact Scale . . . . . 111
5.7.2 The Center for Epidemiologic Studies Depression Scale . . . . . 112
5.7.3 The Short Form Health Survey . . . . . 112
5.7.4 Patient's Assessment of Treatment Benefit . . . . . 112
5.8 Pharmacoeconomic Assessments/EQ-5D . . . . . 112
5.9 Optional Exploratory Substudies . . . . . 113
5.9.1 Optical Coherence Tomography Exploratory Substudy . . . . . 113
5.10 Open-Label Extension Phase . . . . . 113
5.10.1 Open-Label Extension Phase Screening Period . . . . . 113
5.10.2 Open-Label Extension Phase . . . . . 114
5.10.3 Overview of Schedule of Assessments in the Open-Label Extension Phase . . . . . 114
5.10.3.1 Delayed Dosing Visit in the Open-Label Extension Phase . . . . . 115
5.10.3.2 Unscheduled Visits in the Open-Label Extension Phase . . . . . 115
5.10.3.3 Withdrawal Visits in the Open-Label Extension Phase . . . . . 116

6. Investigational Medicinal Product . . . . . 116

6.1 Ocrelizumab . . . . . 117

6.1.1 Preparation and Administration of Ocrelizumab Infusions . . . . . . . . 117
6.1.2 Prevention and Treatment of Infusion Related Reactions . . . . . . . . . 119
6.1.3 Ocrelizumab Dose Modifications, Interruptions and Delays . . . . . . . 120
6.1.4 Criteria for Re-Treatment with Ocrelizumab . . . . . . . . . . . . . . . . . . 120
6.2 Rebif® . . . . . . . . . . . . . . . . . . . . . . . . . . . . . . . . . . . . . . . . . . . . . 121
6.2.1 Dose and Schedule of Rebif® . . . . . . . . . . . . . . . . . . . . . . . . . . . . . 121
6.2.2 Rebif® Dose Modifications, Interruptions and Delays . . . . . . . . . . . 122
6.3 Formulation, Packaging and Labeling . . . . . . . . . . . . . . . . . . . . . . . . . 123
6.3.1 Ocrelizumab . . . . . . . . . . . . . . . . . . . . . . . . . . . . . . . . . . . . . . . . . 123
6.3.2 Rebif® . . . . . . . . . . . . . . . . . . . . . . . . . . . . . . . . . . . . . . . . . . . . 124
6.4 Blinding and Unblinding . . . . . . . . . . . . . . . . . . . . . . . . . . . . . . . . . 125
6.5 Accountability of Investigational Medicinal Product and Assessment of Compliance . . . . . . . . . . . . . . . . . . . . . . . . . . . . . . . . . . . . . . . . . 125
6.5.1 Accountability of Investigational Medicinal Product . . . . . . . . . . . . . 125
6.5.2 Assessment of Compliance . . . . . . . . . . . . . . . . . . . . . . . . . . . . . . . 126
6.6 Destruction of the Investigational Medicinal Product/Comparator . . . . 126

7. Safety Instructions and Guidance . . . . . . . . . . . . . . . . . . . . . . . . . . . . 127

7.1 Adverse Events and Laboratory Abnormalities . . . . . . . . . . . . . . . . . . 127
7.1.1 Clinical Adverse Events . . . . . . . . . . . . . . . . . . . . . . . . . . . . . . . . . 127
7.1.1.1 Intensity of Clinical Adverse Events . . . . . . . . . . . . . . . . . . . . . . . 127
7.1.1.2 Drug - Adverse Event Relationship . . . . . . . . . . . . . . . . . . . . . . . . 128
7.1.1.3 Serious Adverse Events (Immediately Reportable to Sponsor) . . . 128
7.1.2 Treatment and Follow-up of Adverse Events . . . . . . . . . . . . . . . . . . 128
7.1.3 Laboratory Test Abnormalities . . . . . . . . . . . . . . . . . . . . . . . . . . . . 129
7.1.3.1 Follow-up of Abnormal Laboratory Test Values . . . . . . . . . . . . . . 129
7.2 Handling of Safety Parameters . . . . . . . . . . . . . . . . . . . . . . . . . . . . . 129
7.2.1 Reporting of Adverse Events . . . . . . . . . . . . . . . . . . . . . . . . . . . . . 129
7.2.2 Reporting of Serious Adverse Events . . . . . . . . . . . . . . . . . . . . . . . . 130
7.2.2.1 Immediate Reporting to the Sponsor . . . . . . . . . . . . . . . . . . . . . . . 130
7.2.2.2 Emergency Medical Contacts . . . . . . . . . . . . . . . . . . . . . . . . . . . . 131
7.2.2.3 Expedited Reporting to Health Authorities, Investigators, Institutional Review Boards, and Ethics Committees . . . . . . . . . . . . . . 131
7.2.3 Pregnancy and Lactation . . . . . . . . . . . . . . . . . . . . . . . . . . . . . . . . 132
7.3 Warnings and Precautions . . . . . . . . . . . . . . . . . . . . . . . . . . . . . . . . 133

7.3.1 Ocrelizumab . . . 133
7.3.2 Rebif® . . . 135
7.3.3 Corticosteroids . . . 136
7.3.4 Progressive Multifocal Leukoencephalopathy . . . 136
7.3.4.1 Guidance for Diagnosis of Progressive Multifocal Leukoencephalopathy . . . 137

8. Statistical Considerations and Analytical Plan . . . 141

8.1 Study Endpoints . . . 141
8.1.1 Primary Efficacy Endpoint . . . 141
8.1.2 Secondary Efficacy Endpoints . . . 141
8.1.3 Exploratory Efficacy Endpoints . . . 142
8.1.4 Safety . . . 143
8.2 Statistical and Analytical Methods . . . 143
8.2.1 Primary Efficacy Analysis . . . 144
8.2.2 Secondary Efficacy Analyses . . . 144
8.2.2.1 The Time to Onset of Confirmed Disability Progression for At Least 12 Weeks During the 96-Week Comparative Treatment Period . 144
8.2.2.2 Total Number of T1 Gadolinium-Enhanced Lesions as Detected by Brain MRI at Weeks 24, 48, and 96 . . . 145
8.2.2.3 The Total Number of New, and/or Enlarging T2 Hyperintense Lesions as Detected by Brain Magnetic Resonance Imaging at Week 24, Week 48 and Week 96 . . . 146
8.2.2.4 Proportion of Patients who have Disability Improvement Confirmed for At Least 12 Weeks . . . 146
8.2.2.5 The Time to Onset of Confirmed Disability Progression for At Least 24 Weeks During the 96-Week Comparative Treatment Period . 146
8.2.2.6 Total Number of T1-Hypo-Intense Lesions (Chronic Black Holes) at Weeks 24, 48, and 96 . . . 147
8.2.2.7 The Change in Multiple Sclerosis Functional Composite Scale (MSFCS) Score from Baseline to Weeks 96 . . . 147
8.2.2.8 The Percentage Change in Brain Volume as Detected by Brain Magnetic Resonance Imaging Scan from Week 24 to Week 96 . . . 147
8.2.2.9 Change in Quality of Life, as Measured by the Short Form 36 version 2 Physical Component Summary (PCS) Score from Baseline to Week 96 . . . 147

8.2.2.10 Proportion of Patients Who Have No Evidence of Disease Activity (NEDA) by Week 96 . . . . 147
8.2.3 Exploratory Analyses . . . . 148
8.2.4 Sample Size . . . . 149
8.2.5 Hypothesis Testing . . . . 149
8.2.6 Analysis Populations . . . . 149
8.2.6.1 Safety Population . . . . 150
8.2.6.2 Intent-to-Treat Population . . . . 150
8.2.6.3 Per Protocol Population . . . . 150
8.2.7 Interim Analysis . . . . 150
8.2.8 Safety Data Analysis . . . . 150
8.2.9 Safety Follow-up Period . . . . 151
8.2.10 Open-Label Extension Phase . . . . 151
8.2.11 Other Analyses . . . . 151
8.2.11.1 Pharmacokinetic Analysis . . . . 151
8.2.11.2 Pharmacodynamic Analysis . . . . 152
8.2.11.3 Roche Clinical Repository / Protein Biomarker Samples . . . . 152

9. Data Collection, Management and Quality Assurance . . . . 152
9.1 Assignment of Preferred Terms and Original Terminology . . . . 153

10. Study Committees . . . . 153

11. References . . . . 154

12. Ethical Aspects . . . . 160
12.1 Local Regulations/Declaration of Helsinki . . . . 160
12.2 Informed Consent . . . . 160
12.2.1 Study Informed Consent . . . . 160
12.2.2 RCR Informed Consent . . . . 161
12.2.3 Death or Loss of Competence of Participant who has donated a specimen(s) that is stored in the RCR . . . . 161
12.3 Independent Ethics Committees (IEC) and Institutional Review Board (IRB) . . . . 162
12.4 Role of the Science and Ethics Advisory Group (SEAG) . . . . 163

13. Conditions for Modifying the Protocol . . . . 163

14. Conditions for Terminating the Study . . . . . . . . . . . . . . . . . . . . . . . . . . . . . 163

15. Study Documentation, CRFs and Record Keeping . . . . . . . . . . . . . . . . . . . 163

15.1 Investigator's Files / Retention of Documents . . . . . . . . . . . . . . . . . . . 163

15.2 Source Documents and Background Data . . . . . . . . . . . . . . . . . . . . . . 164

15.3 Audits and Inspections . . . . . . . . . . . . . . . . . . . . . . . . . . . . . . . . . . . 164

15.4 Electronic Case Report Forms . . . . . . . . . . . . . . . . . . . . . . . . . . . . . 164

15.5 Financial Disclosure . . . . . . . . . . . . . . . . . . . . . . . . . . . . . . . . . . . . 165

16. Monitoring the Study . . . . . . . . . . . . . . . . . . . . . . . . . . . . . . . . . . . . . 165

17. Confidentiality of Trial Documents and Subject Records . . . . . . . . . . . . . . 165

18. Clinical Study Report (CSR) . . . . . . . . . . . . . . . . . . . . . . . . . . . . . . . . 166

19. Publication of Data and Protection of Trade Secrets . . . . . . . . . . . . . . . . . 166

## List of Tables


Table 1: Summary of the Most Frequent Rebif® Adverse Reactions by MedDRA System Organ Class . . . . . . . . . . . . . . . . . . . . . . . . . . 62

Table 2: Overview of Dosing Regimen in the Double-Blind, Double-Dummy Treatment Period. . . . . . . . . . . . . . . . . . . . . . . . . . . . . . . . . . . . . 70

Table 6: Schedule of Assessments: Open Label Extension Phase . . . . . . . . . . . 94

Table 7: Treatment Groups and Schedule of Study Medication During the Double-Blind, Double-Dummy Treatment Period . . . . . . . . . . . 116

Table 8: Infusions of Ocrelizumab 300 mg . . . . . . . . . . . . . . . . . . . . . . . . . . . 118

Table 9: Subsequent Infusions of Ocrelizumab 600 mg. . . . . . . . . . . . . . . . . . . 118

Table 10: Overview of Rebif® Dosing Regimen . . . . . . . . . . . . . . . . . . . . . . . . 121

Table 11: Clinical features to distinguish between MS relapse and PML* . . . . 139

Table 12: MRI Lesion Characteristics Typical of PML and MS . . . . . . . . . . . . 140

## List of Figures


Figure 1: Overview of Study Design . . . . . . . . . . . . . . . . . . . . . . . . . . . . . . . . . 66

Figure 2: Safety Follow-Up - Variable B-cell Monitoring . . . . . . . . . . . . . . . . . 69

Figure 3: Diagnostic Algorithm for PML . . . . . . . . . . . . . . . . . . . . . . . . . . . . . 139

## List of Appendices


Appendix 1: AEs Categories for Determining Relationship to Test Drug ..... 167

Appendix 2: ICH Guidelines for Clinical Safety Data Management, Definitions and Standards for Expedited Reporting, Topic E2 . 168

Appendix 3: Common Terminology Criteria (CTCAE)................... 170

Appendix 4: Telephone Interviews.................................... 171

Appendix 5: Modified Fatigue Impact Scale (MFIS)..................... 174

Appendix 6: The Center for Epidemiologic Studies Depression Scale (CES-D) ........................................... 177

Appendix 7: The Short Form (SF-36) Health Survey .................... 179

Appendix 8: Optical Coherence Tomography Exploratory Substudy ........ 185

# GLOSSARY OF ABBREVIATIONS

| | |
|---|---|
| *ADA* | *Anti-drug antibody (also known as Human Anti-human Antibodies [HAHA])* |
| ADCC | Antibody dependent cellular cytotoxicity |
| AE | Adverse Event |
| AIDS | Acquired Immune Deficiency Syndrome |
| ALT (SGPT) | Alanine aminotransferase |
| ALP | Alkaline Phosphatase |
| ARR | Annualized Relapse Rate |
| AST (SGOT) | Aspartate aminotransferase |
| AUC | Area Under the Curve |
| BAFF | B-cell activating factor |
| BCG | Bacille Calmette-Guérin – TB vaccine |
| | (Fr. Bacille billié de Calmette-Guérin) |
| $\beta$ hCG | Beta human Chorionic Gonadotropin |
| CD | Cluster of Differentiation |
| CDC | Complement-dependent cytotoxicity |
| CES-D | Center for Epidemiologic Studies Depression Scale |
| CFH | Complement Factor H |
| CSF | Cerebrospinal Fluid |
| C-SSRS | Columbia - Suicide Severity Rating Scale |
| CTCAE | Common Terminology Criteria for Adverse Events |
| DMARD | Disease-modifying anti-rheumatic drug |
| DMC | Data Monitoring Committee |
| DMT | Disease-modifying therapy |
| DNA | Deoxyribonucleic Acid |
| EBV | Epstein-Barr Virus |
| ECG | Electrocardiogram |
| eCRF | Electronic Case Report Form(s) |

## GLOSSARY OF ABBREVIATIONS

| | |
|---|---|
| EDC | Electronic Data Capture |
| EDSS | Expanded Disability Status Scale |
| EEG | Electroencephalogram |
| eform | Electronic form |
| ELISA | Enzyme-Linked Immunosorbent Assay |
| EMA | European Medicines Agency |
| EQ-5D | EuroQoL |
| ESF | Eligibility Screening Form |
| ETDRS | Early Treatment Diabetic Retinopathy Study |
| FACS | Fluorescence-activated Cell Sorting |
| FDA | Food and Drug Administration |
| FLAIR | Fluid-attenuated Inversion Recovery |
| FSH | Follicle Stimulating Hormone |
| FSS | Functional Systems Scores |
| Gd | Gadolinium |
| GGT | Gamma Glutamyl Transferase |
| HAHA | Human Anti-human Antibodies *(also known as anti-drug antibody [ADA])* |
| HAM | Human T-lymphotropic virus (HTLV)-1 Associated Myelopathy |
| HBsAg | Hepatitis B Surface Antigen |
| HBcAb | Hepatitis B Core Antibody |
| HepCAb | Hepatitis C Antibody |
| HDHF | High Dose High Frequency |
| HIV | Human Immunodeficiency Virus |
| HTLV | Human T-lymphotropic Virus |
| IB | Investigator Brochure |
| ICH | International Conference on Harmonisation |

## GLOSSARY OF ABBREVIATIONS

| | |
|---|---|
| ICMJE | International Committee of Medical Journal Editors |
| IFN | Interferon |
| Ig | Immunoglobulin |
| i.m. | Intramuscular |
| IMP | Investigational Medicinal Product |
| IND | Investigational New Drug |
| INN | International Non-proprietary Name |
| IRB/IEC | Institutional Review Board/Independent Ethics Committee |
| IRR | Infusion Related Reaction |
| ITT | Intent-To-Treat |
| i.v. | Intravenous |
| IxRS | Interactive Voice and Web Response System |
| JCV | JC Virus |
| KLH | Keyhole Limpet Haemocyanin |
| LCVA | Low Contrast Visual Acuity |
| LLN | Lower Limit of Normal |
| MBP | Myelin Basic Protein |
| *MCS* | *Mental Component Summary* |
| MedDRA | Medical Dictionary for Regulatory Activities |
| mEP | Multimodal Evoked Potentials |
| MFIS | Modified Fatigue Impact Scale |
| MOG | Myelin Oligodendrocyte Glycoprotein |
| MRI | Magnetic Resonance Imaging |
| MS | Multiple Sclerosis |
| MSFCS | Multiple Sclerosis Functional Composite Scale |
| *MMRM* | *Mixed-Effect Model Repeated Measures* |
| MTX | Methotrexate |

## GLOSSARY OF ABBREVIATIONS

| | |
|---|---|
| NAb | Neutralizing Antibody |
| NHL | Non Hodgkin Lymphoma |
| NK | Natural killer |
| NYHA | New York Heart Association |
| OCB | Oligoclonal Band |
| OCR | Ocrelizumab |
| OCT | Optical Coherence Tomography |
| OLE | Open-Label Extension |
| PASAT | Paced Auditory Serial Addition Test |
| PCR | Polymerase Chain Reaction |
| *PCS* | *Physical Component Summary* |
| PD | Pharmacodynamics |
| PK | Pharmacokinetics |
| PML | Progressive Multifocal Leukoencephalopathy |
| PP | Per protocol (population) |
| PPMS | Primary Progressive Multiple Sclerosis |
| PRMS | Progressive Relapsing Multiple Sclerosis |
| PRO | Patient-Reported Outcome |
| RA | Rheumatoid Arthritis |
| RBC | Red Blood Cells |
| RCR | Roche Clinical Repository |
| RMS | Relapsing Multiple Sclerosis |
| RNA | Ribonucleic Acid |
| RNFL | Retinal Nerve Fiber Layer |
| ROW | Rest of the World |
| RMS | Relapsing Multiple Sclerosis |
| RNFL | Retinal Nerve Fiber Layer |
| RPR | Rapid Plasma Reagin |

## GLOSSARY OF ABBREVIATIONS

| | |
|---|---|
| RRMS | Relapsing-Remitting Multiple Sclerosis |
| SAE | Serious Adverse Event |
| *SAP* | *Statistical Analysis Plan* |
| s.c. | Subcutaneous |
| SDMT | The Symbol Digit Modalities Test |
| SEAG | Science and Ethics Advisory Group |
| SF-36 | SF-36 Health Survey |
| SMT | Study Management Team |
| SPMS | Secondary Progressive Multiple Sclerosis |
| TNF | Tumor Necrosis Factor |
| sTSH | sensitive Thyroid Stimulating Hormone |
| TB | Tuberculosis |
| $T_{CTL}$ | Cytotoxic Lymphocyte T |
| ULN | Upper Limit of Normal |
| VEP | Visual Evoked Potential |
| WBC | White Blood Cells |

# PART I: STUDY DESIGN AND CONDUCT

## 1. BACKGROUND AND RATIONALE

## 1.1 Background

### 1.1.1 Multiple Sclerosis

Multiple sclerosis (MS) is an inflammatory and degenerative demyelinating disease of the human central nervous system (CNS). Multiple sclerosis affects around 2.5 million people worldwide: it is one of the most common neurological disorders and causes of disability of young adults, especially in Europe and North America [1]. The condition manifests as neurological deficits referable to damage to the spinal cord, brainstem, optic nerves, cerebellum, and cerebrum. Resulting symptoms may include weakness, pain, visual loss, bowel/bladder dysfunction, and cognitive dysfunction. Diagnosis of MS typically occurs through the application of highly structured diagnostic criteria that rely on clinical observation, neurological examination, brain and spinal cord Magnetic Resonance Imaging (MRI) scans, evoked potentials, and examination of cerebrospinal fluid (CSF) [2, 3].

MS is clinically subcategorized into four phenotypic disease patterns distinguished by the occurrence and timing of relapses relative to disease onset and disability progression [4]. These include relapsing remitting MS (RRMS), primary progressive MS (PPMS), progressive relapsing MS (PRMS); and secondary progressive MS (SPMS).

Approximately 80% of MS patients present with RRMS. If left untreated, the majority of RRMS patients will transition into SPMS (with progressive loss of neurologic function, in the absence of relapses) within 20 years. The term relapsing MS (RMS) applies to those patients either RRMS or SPMS, who continue to suffer relapses. Patients with RMS, whether or not they suffer from neurologic progression in the absence of relapses, have a common, inflammatory pathophysiology and therefore, constitute a common target for treatment.

Currently available first-line therapies for the treatment of either relapsing MS or relapsing-remitting MS include interferon (IFN)-β-1a (Rebif® and Avonex®), IFN-β-1b (Betaferon®/Extavia®) and glatiramer acetate (Copaxone®). The currently approved first-line treatments are only modestly effective in reducing the frequency of relapses and preventing disability in patients with RMS. The magnitude of these disease modifying effects are an approximately 30% relative improvement versus placebo [5]. The first-line disease modifying agents reduce the frequency of new episodes but do not reverse fixed deficits and have questionable effects on long-term *disability* progression [6].

Fingolimod (*Gilenya®*) is an oral modulator of sphingosine-1 phosphate (S1P) receptors, a ubiquitous group of transmembrane receptors involved with cellular growth and differentiation. Fingolimod's immunomodulatory effects are believed to be due to binding to and internalization of the S1P1 receptor on lymphocytes, thereby rendering them insensitive to S1P gradients in lymph and inhibiting egress from lymph nodes and other secondary lymphoid organs. Fingolimod is known to readily cross the blood-brain barrier and there are S1P receptors on glial cells and neurons, however the implications

of any possible direct CNS S1P receptor modulation effects are currently unknown. Fingolimod was shown to reduce the annualized relapse rate (ARR) by approximately 50% versus both placebo and interferon β-1a, intramuscular (i.m.) 30 mcg weekly (Avonex®), in confirmatory Phase III clinical trials. Due to the presence of S1P receptors on many different cell types, the adverse event profile of fingolimod is complex, with potential effects on cardiac, ophthalmic, hepatic and pulmonary function, as well as an increased risk of infection, due to inhibition of lymphocyte trafficking. Fingolimod was approved in 2010 by the US Food and Drug Administration (FDA) for patients with relapsing forms of MS and by the European Medicines Agency (EMA) in 2011 for use in patients who have previously failed first-line disease-modifying therapy (DMT) or who have highly active disease.

Natalizumab (Tysabri®) is a monoclonal antibody directed against alpha-4 beta-1 integrin (VLA-4), an adhesion molecule expressed on activated lymphocytes. Natalizumab binds to VLA-4, inhibiting trafficking of activated lymphocytes into the CNS and other extravascular tissues. Natalizumab was shown to have a 66% relative reduction in ARR versus placebo in a Phase III clinical trial. Natalizumab use is generally limited to RRMS patients who have failed to respond to first-line disease modifying therapy or to highly active RRMS patients due to a risk of Progressive Multifocal Leukoencephalopathy (PML).

Teriflunomide (Aubagio®) is a once-daily oral immunomodulator that inhibits dihydroorotate dehydrogenase, a key mitochondrial enzyme involved in de novo pyrimidine synthesis for deoxyribonucleic acid (DNA) replication, and reduces T-cell and B-cell proliferation and function in response to autoantigens. Teriflunomide has been approved in 2012 for treatment of relapsing forms of MS by the FDA. Two Phase III trials showed a significant reduction in ARR of teriflunomide against placebo [7, 8]. A third Phase III trial, TENERE, showed that teriflunomide was not statistically different than interferon β-1a on risk of treatment failure [9]. Risk of treatment failure was defined as the occurrence of a confirmed relapse or permanent treatment discontinuation for any cause, whichever came first.

Mitoxantrone (Novantrone®), a chemotherapeutic agent, is also approved for treatment of relapsing MS in the United States of America, but is generally reserved for secondary progressive and severe relapsing remitting forms of disease. Other drugs have been used with varying degrees of success, including corticosteroids, methotrexate (MTX), cyclophosphamide, azathioprine, and intravenous (i.v.) immunoglobulin.

Despite significant advances in MS therapy many patients continue to experience disease activity; thus there remains a need to develop more effective and better tolerated therapies for the treatment of RMS.

#### 1.1.2 Ocrelizumab

Ocrelizumab is a humanized, glycosylated, monoclonal antibody directed against the cluster of differentiation (CD)20 antigen present on select B-cells. Ocrelizumab binds to the CD20 antigen thereby resulting in B-cell depletion via antibody-dependant cellular cytotoxicity (ADCC), complement-dependent cytotoxicity (CDC) and enhanced apoptosis.

Ocrelizumab was constructed using a recombinant DNA technique. This antibody shares an overlapping epitope on CD20 with rituximab (chimeric monoclonal antibody, Mabthera®/Rituxan®), as determined by direct competition and epitope-mapping experiments. In-vitro, ocrelizumab was shown to be approximately 5 times more potent than rituximab in ADCC activity on a B-cell tumor line over-expressing CD20, approximately 3 times less potent via CDC, and approximately equal in inducing apoptosis in a B-cell lymphoma cell line.

There is substantial proof-of-concept clinical data to support the use of B-cell depleting therapies in patients with relapsing MS. Ocrelizumab shares the same basic mechanism of action as rituximab. In a proof of concept study, rituximab treatment resulted in a robust reduction in MRI based measures of CNS inflammation and clinical benefit versus placebo, in patients with RRMS [10]. WA21493/ACT4422g, a Phase II study of ocrelizumab in RRMS patients provides proof-of-concept support for ocrelizumab efficacy and safety in patients with relapsing remitting MS; please refer to Section 1.1.4.1 for more details.

Ocrelizumab is also known as Ro 496-4913, PRO70769, and rhuMAb 2H7 (refer to the ocrelizumab Investigator's Brochure [IB] for further information).

### 1.1.3 Rationale for Targeting B-cells in MS

Humoral immunity has been implicated in MS for decades, as evidenced by inclusion of CSF oligoclonal bands (OCBs) and increased intrathecal immunoglobulin (Ig)G synthesis in diagnostic criteria for MS [2, 3, 11]. Although, until very recently, the prevailing view of MS pathophysiology held that the CNS inflammation seen in MS is principally mediated by $CD4^+$ proinflammatory (Th1, Th17) T cells, rapidly expanding evidence suggests that B-cells may contribute to MS pathogenesis much more fundamentally than was previously believed, potentially through either antibody-dependent or independent mechanisms [12, 13, 14]. B lymphocytes have been detected within MS lesions and in the CSF of MS patients. Molecular analysis of both lesional and CSF B-cell repertoires reveals dominant, clonally expanded B-cell populations exhibiting somatic hypermutation in the antigen-recognizing CDR3 regions of Ig heavy chains, predominately within the VH4 gene family [15, 16, 17, 18, 19, 20].

Detection of these affinity-matured, clonally expanded repertoires in the CSF but not peripheral blood of MS patients suggests that a localized, antigen driven B-cell response is present in the CNS compartment. CSF clonal B-cell expansion has been reported in patients with both RRMS and PPMS shortly after diagnosis, implying a role for B-cells early in MS pathogenesis rather than as a late response to longstanding tissue damage [21]. More recently, cDNA transcriptomes of clonally expanded affinity-matured B-cells isolated from the CSF of MS patients have been sequence-matched to specific IgG OCBs from the same CSF samples, indicating that this longstanding hallmark of MS diagnosis derives from identifiable B-cell clones present in the CNS compartment [22].

Both antibody-dependent and independent hypotheses for the role of B-cells in MS pathophysiology have been postulated and are currently the subject of intensive research. B-cells may differentiate into plasma cells and produce CNS-directed auto-antibodies,

potentially triggering cellular and complement-dependent cytotoxicity. Although a pathogenic role of anti-myelin antibodies in MS has not been established, they have been detected in the CSF of MS patients [23, 24, 25] and in active MS lesions [26] and remain potential candidates as effectors of myelin sheath damage. B-cells may also function as antigen presenting cells and thereby modulate effector T-cell responses, as they exhibit regulated secretion of both pro-inflammatory and anti-inflammatory cytokines, a function that appears to be abnormal in patients with MS [12]. Finally, B-cells may be a site of latent viral infections such as Epstein Barr Virus (EBV), which may drive CNS autoimmune responses through molecular mimicry or other pro-inflammatory mechanisms [13].

Postmortem pathological studies have identified the presence of ectopic follicular lymphoid structures in the meninges anatomically proximal to sites of grey matter demyelination in a subset of SPMS patients [27, 28, 29]. Similar tertiary lymphoid structures form *de novo* in various tissues of many autoimmune disorders and represent potential *de novo* sites of chronic autoantigenic B-cell activation, maturation and clonal expansion [30]. SPMS patients exhibiting these lymphoid structures have been found to have worse progression rates, when compared to controls without such follicular structures [31] and a pathomechanistic link to grey matter demyelination typical for SPMS has been suggested. Whether or not an anti-CD20 therapeutic antibody can affect the formation or persistence of meningeal lymphoid follicles or the grey matter demyelination prominent in progressive forms of MS is unknown.

In summary, B lymphocytes are believed to contribute to the pathogenesis of all subtypes of MS. Removing select peripheral B-cells from circulation may beneficially disrupt inflammatory processes that potentially involve chronic antigenic stimulation or other regulatory functions promoting chronic autoimmunity. Ocrelizumab specifically depletes CD20+ B-cells, making it a potentially attractive pharmacological agent to test for therapeutic potential in patients with MS.

### 1.1.4 Sponsor Experience with Anti-CD20 Compounds in MS

#### *1.1.4.1 Ocrelizumab in RRMS*

Study WA21493/ACT4422G was a 220-patient Phase II, multicenter, randomized, parallel-group, placebo-controlled, proof-of-concept study to evaluate the safety and efficacy of two dose regimens of ocrelizumab (1000 mg × 2 [administered on Day 1 and Day 15, followed by single infusions of 1000 mg for subsequent cycles] and 300 mg × 2 [administered on Day 1 and Day 15 followed by single infusions of 600 mg for subsequent cycles]), with an additional randomized open label arm of interferon β1-a 30 µg i.m. every week (Avonex®) arm. The primary objective was to evaluate the efficacy of two dose regimens of ocrelizumab compared with placebo, in reducing brain inflammation, as measured by the total number of gadolinium (Gd)-enhancing T1 lesions observed on serial MRI scans of the brain at Weeks 12, 16, 20, and 24. Key secondary objectives were to evaluate the efficacy of both dose regimens of ocrelizumab compared with placebo in reducing ARR at 24 weeks and to evaluate the safety and tolerability of both dose regimens of ocrelizumab in patients with RRMS. Exploratory outcomes included analysis of both dose regimens of ocrelizumab compared to interferon β-1a 30 µg i.m. weekly (Avonex®) along various study measures. Treatment with ocrelizumab

was planned for 72 to 96 weeks total, depending on study arm (patients from both placebo and Avonex group switched to ocrelizumab 300 mg × 2 after Week 24). Additional MRI scans of the brain will be obtained at Weeks 96 and 144 for a subgroup of patients.

Week 24 results demonstrated that both doses of ocrelizumab achieved the primary endpoint by significantly reducing the number of Gd-enhancing lesions compared with placebo ($p< 0.0001$). Both ocrelizumab (OCR) dose groups showed statistically significant reductions in ARR compared with the placebo group (ARR = 0.125 for the OCR 300 mg × 2 group [$p = 0.0005$] and ARR = 0.169 for the OCR 1000 mg × 2 group [$p = 0.0014$] compared with ARR = 0.637 for the placebo group, representing a relative reduction (RR) of 80% and 73% in ARR versus placebo group for the low and high OCR groups, respectively. In exploratory analyses, both ocrelizumab groups were superior to the Avonex group for the primary endpoint ($p < 0.0001$) and the 300 mg × 2 group for ARR (ARR = 0.364 for the Avonex group, representing a RR of 66% in ARR with $p = 0.03$ for the OCR 300 mg × 2 group versus Avonex group and a RR of 53.6% in the ARR with $p = 0.086$ for the OCR 1000 mg × 2 group versus Avonex group) [32].

Patients from both placebo and Avonex groups switched to ocrelizumab 300 mg × 2 after Week 24. By 48 weeks, the level of benefit of ocrelizumab in reduction of ARR was maintained, where the patients in the ocrelizumab 300 mg × 2 group continued to have a suppressed ARR of 0.086 from Week 24 to 48, and patients switched to ocrelizumab from either placebo or Avonex®, derive a similar degree of efficacy to those randomized to ocrelizumab from onset (ARR for placebo-to-ocrelizumab = 0.161 and for Avonex-to-ocrelizumab = 0.137 after the switch, representing a RR of 74% and 62.4% compared with ARR before the switch, respectively). From Weeks 0 to 72, patients originally randomized to ocrelizumab 300 mg × 2 maintained clinical efficacy with an ARR of 0.186.

The most commonly reported adverse events in ocrelizumab-treated patients were infusion related reactions (IRRs). IRRs were reported during/after the first infusion (Day 1) for 30–43.6% of patients treated with ocrelizumab. Fewer patients (2.1–9.4%) experienced IRRs during/after the second infusion (Day 15). The most common symptoms were rash, pruritus, flushing, tachycardia, headache, pyrexia, and throat irritation. No unanticipated, clinically significant abnormalities in vital signs, electrocardiograms (ECGs), or laboratory parameters were observed in association with ocrelizumab treatment.

On review of the placebo-controlled, double-blinded, 24-week safety data, no imbalance in adverse events (or infection adverse events) or serious adverse events (or infection serious adverse events) between the placebo and active ocrelizumab arms was observed. The rate of adverse events (or infection adverse events) and serious adverse events (or infection serious adverse events) did not increase in ocrelizumab-treated patients at 48 weeks compared with 24 weeks. There is no trend toward an increased risk of adverse events (or infection adverse events) or serious adverse events (or infection serious adverse events) for ocrelizumab-treated patients with previous IFN treatment (Avonex for 6 months).

By the time all patients finished the Week 48 of treatment period, the incidence of infections and serious infections was 92.41/100 PY (95% CI: 76.59, 111.5) and 3.39/100 PY (95% CI: 1.27, 9.04) in patients exposed to low-dose ocrelizumab, including patients who switched from placebo or Avonex®. The incidence of infections and serious infections was 97.38/100 PY (95% CI: 74.76, 126.84) and 5.31/100 PY (95% CI: 1.71, 16.47) in those exposed to the high dose of ocrelizumab. The most common infections in ocrelizumab-treated patients included urinary tract infections, upper respiratory infections, and nasopharyngitis.

To date, in study WA21493, after over 250 patient-years exposure to ocrelizumab, there have been no reports of opportunistic or fatal infections.

One 41-year-old female patient receiving ocrelizumab 1000 mg × 2 died during the Phase II RRMS study at Week 14. This patient was hospitalized with acute onset of encephalopathy and status epilepticus due to systemic inflammatory response syndrome with disseminated intravascular coagulation of unknown cause, following infusion of Gd. The patient's course rapidly progressed to multi-organ failure. While hospitalized, the patient developed nosocomial pneumonia in the setting of severe renal and hepatic insufficiency. After 2 weeks of intensive care, the patient died of transforaminal herniation of the brain, due to massive cerebral edema. Despite a thorough clinical pathological review, the exact cause of death could not be determined. The Investigator assessed the event as related to ocrelizumab.

In a separate trial, another patient receiving ocrelizumab 300 mg x 2 died during the PPMS Phase III study (Study WA25046) 10 days after onset of community-acquired pneumonia. The Investigator reported the event as unrelated to study medication. The initial event appears to be of infectious origin; to determine causal association with ocrelizumab will require assessment of aggregate data. Pneumonia was confirmed at autopsy. It is unclear to what extent ocrelizumab may have contributed to the fatal outcome of the event as there is currently no indication that the patient benefited from any medical attention during the 2 weeks after onset of dyspnea and general weakness until death. The causal relationship to ocrelizumab remains possible as assessed by the Sponsor.

Two other patients died during the Safety Follow-Up Period of the Phase II RRMS study at a time when B cells had recovered. Details regarding these cases are provided in the ocrelizumab IB.

#### 1.1.4.2 Long-Term Results of Phase II Study WA21493/ACT4422G

To further understand the long-term effect of ocrelizumab therapy, the Phase II Study WA21493/ACT4422G was designed with a 48-week treatment-free period after the 96-week treatment period with ocrelizumab. Across all treatment groups, 86-91% of the 220 initially randomized patients entered the treatment-free period. This included patients who had withdrawn from treatment. Subsequently, 73-82% of patients completed Week 120; 69-80% completed Week 144; and 36-56% entered a 24-week observation drug-free period after B-cell repletion at Week 144. During the treatment-free period, ARR remained at similar low levels as during the treatment period (0.04–0.29 across treatment groups), with no indication of increase or rebound. The mean number of

Gd-enhancing T1 lesions remained at 0 at Week 144 in the ocrelizumab 600 mg group and increased from 0 at Week 96 to 0.3 at Week 144 in the ocrelizumab 1000 mg group. Furthermore, there was no imbalance in the rates of adverse events or serious adverse events across all treatment groups over 144 weeks. No new serious infections and no opportunistic infections were reported since the last ocrelizumab administration. Infection rates did not increase over time: at Week 144, infection rates were 6.5% and 11.1% with the ocrelizumab 600 mg and 1000 mg regimens, respectively. The most common types of infections were upper respiratory tract infections, nasopharyngitis, and urinary tract infections [33].

The long-term efficacy data from the Phase II Study WA21493/ACT4422G show that the low-level of disease activity observed after ocrelizumab treatment is sustained through Week 144. This includes the observation that no Gd-enhancing T1 lesions were observed in the ocrelizumab 600 mg group at Week 144, indicating a CNS anti-inflammatory long-term effect of ocrelizumab. Up to Week 144, no new safety issues were observed with ocrelizumab. In particular, there were no increases in infections and no new serious adverse events, indicating a positive benefit/risk profile. Currently, eligible patients *continue ocrelizumab treatment in the* Open-Label Extension (OLE) Phase of this study.

#### *1.1.4.3 Rituximab in RRMS*

Two clinical trials have been conducted in RRMS patients with rituximab, a chimeric mouse/human monoclonal antibody that shares the same basic mechanism of action as ocrelizumab. Findings briefly highlighted below, offer additional support for the therapeutic potential of the anti-CD20 mechanism in MS.

Study U3264g (HERMES Jr.) was a Phase I, open-label, multicenter study in 26 adults with RRMS to evaluate the safety and tolerability of two treatment cycles of rituximab administered at baseline and after 24 weeks. Re-treatment with rituximab (1000 mg × 2) at 24 weeks was safe and well tolerated, with an observed decrease in relapses and Gd-enhancing lesions through 72 weeks [34]

Study U2787g (HERMES) was a Phase II, proof-of-concept, randomized, double-blind, parallel-group, placebo-controlled, multicenter study to evaluate the safety and efficacy of rituximab in 104 adults with RRMS. The primary objectives were to investigate the efficacy of rituximab compared with placebo, as measured by the total number of Gd-enhancing T1 lesions observed on serial MRI scans of the brain at Weeks 12, 16, 20, and 24, and to evaluate the safety and tolerability of rituximab in patients with RRMS. Secondary objectives were to evaluate additional MRI parameters and the proportion of patients relapsing. The trial met its primary efficacy endpoint and all secondary endpoints. Rituximab was safe and generally well tolerated in this study through 48 weeks though the rate of infusion-associated adverse events, particularly after the first infusion, was higher in rituximab-treated patients (78%) than in placebo patients (40%); corticosteroid premedication was not administered before or at the time of infusion. Study U2787g provides proof of principle that an anti-CD20 therapeutic approach can reduce both MRI and clinical evidence of inflammatory activity in adults with RRMS [10].

#### *1.1.4.4 Rituximab in PPMS*

A single Phase II/III, randomized, double-blinded, placebo-controlled trial was conducted with rituximab in PPMS. The findings, summarized below, represent the largest and longest duration trial experience to date evaluating the safety and efficacy of anti-CD20 therapy in individuals with multiple sclerosis.

Study U2786g (OLYMPUS) was a Phase II/III randomized, double-blind, parallel-group, placebo-controlled, multicenter study evaluating the safety and efficacy of rituximab in patients with PPMS over a 96-week treatment period consisting of 4 treatment cycles with dual infusions of 1000 mg (2000 mg/cycle). Although the trial did not demonstrate significant primary efficacy on time to confirmed disease progression as measured by Expanded Disability Status Scale (EDSS), a difference was observed with 38.5% of patients in the placebo group experiencing confirmed disease progression versus 30.2% in the rituximab group. Biological activity was evidenced by significantly lower T2 lesion volume accumulation on brain MRI, a secondary efficacy endpoint, in rituximab-treated patients compared with placebo (p=0.0008). Subgroup analyses suggest that PPMS patients with evidence of active disease may have shown significant clinical treatment response as measured by time to confirmed disease progression over a 96-week timeframe. Factors that appeared prognostic for disease progression and potentially predictive of treatment response in the rituximab group included younger age, presence of contrast enhancing lesions at baseline on brain MRI, and higher MS severity score.

Rituximab was generally safe and well tolerated in Study U2786g. The proportions of patients with at least one adverse event (100% placebo versus 99% rituximab) and one serious adverse event (13.6% placebo versus 16.1% rituximab) were comparable between treatment groups. Three adverse events that occurred during the study led to death: one in the rituximab group following recurrent aspiration pneumonias and two in the placebo group due to pneumonia and cardiopulmonary failure. More infusion-associated adverse events were observed in rituximab-treated patients (73.6% versus 40.3% for placebo), particularly after the first infusion, but rates declined in both groups to similar levels upon successive infusions. Patients were not premedicated with glucocorticoids before rituximab infusions in Study U2786g. The vast majorities (92%) of infusion associated events in rituximab treated patients were mild to moderate in severity; no Grade 4 or 5 infusion-associated events were observed. The proportion of patients with at least one infection was comparable between groups (68.2% rituximab versus 65.3% placebo), but a higher proportion of patients with at least one serious infection was observed in the rituximab-treated group (4.5%) compared with placebo (< 1%). No opportunistic infections occurred.

Treatment with rituximab was associated with rapid and near-complete depletion of circulating CD19 positive B lymphocytes beginning 2 weeks post-treatment through 96 weeks. Approximately 35% of rituximab-treated patients had recovered peripheral CD19 B-cell counts to 80 cells/µL (laboratory defined lower limit of normal (LLN) in healthy volunteers) within 48 weeks after the last dose. Median circulating CD3 T-lymphocyte counts were not appreciably altered by rituximab. At any time in the trial, IgM levels were below the LLN in 31.7% of rituximab-treated patients and 5.9% of placebo-treated patients. The proportion of patients with IgG and IgA levels below LLN

were not different between groups. The incidence of infectious adverse events and infectious serious adverse events did not appear higher in patients with Ig levels (all isotypes) below LLN in either treatment group compared with patients with Ig levels in the normal range or above upper limit of normal (ULN) [35].

### 1.1.5 Rebif®

The active comparator for this study is Rebif® (interferon β-1a), which has been approved for treatment of RMS.

The efficacy and safety of Rebif® was demonstrated in the PRISMS study (The Prevention of Relapses and Disability by Interferon β-1a Subcutaneously in Multiple Sclerosis) which led to the approval of Rebif® in RMS. This was a multicentre controlled trial of 560 patients with an EDSS score between 1.0 and 5.0 and at least two relapses in the preceding 2 years. Patients were randomized to 2-year treatment with placebo or IFN β-1a (22 or 44 µg subcutaneously three times weekly). Following the 2 years of treatment, both doses of Rebif® showed significant benefits compared with placebo on major efficacy outcome measures. There was a non-significant trend towards greater efficacy with the higher dose on most clinical measures, and a statistically significant dose-effect favoring the higher dose in terms of impact on the number of T2-active lesions. In a subgroup of patients with more severe disease (baseline EDSS >3.5), the 44-µg dose delayed progression of disability significantly better than either the placebo or the 22 µg dose. Neutralizing antibodies were significantly less frequent in the 44 µg group than in the low-dose group [36].

After 2 years, patients who had initially received placebo in the PRISMS study were re-randomized to receive Rebif® (22 or 44 µg subcutaneously three times weekly) and were followed for an additional 2 years. By the end of the 4 year period, patients who had switched from placebo to Rebif® experienced an approximate 50% reduction in ARR compared with the end of year 2. Also, after 4 years, the higher dose approached significance for annual relapse rates (0.8 for 22 µg versus 0.72 for 44 µg; p=0.069). The mean ARR was significantly lower in patients who had received Rebif® for the full 4 years compared with those who had received placebo for the first 2 years. During Years 3 and 4, relapse rates were significantly lower for the 44 µg group with relapse rates decreasing progressively with each year of treatment – 0.92, 0.82, 0.57, and 0.44 relapses/year for each year of the study. Patients who received the highest cumulative dose of active therapy had the lowest rate of disability progression. The time to first confirmed EDSS progression was 42.1 months for the 44 µg group compared with 24.2 months for the crossover group. The time to first confirmed progression did not differ significantly between the 22 µg group (35.9 months) and the crossover group [37].

Rebif® showed superiority versus Avonex® in the EVIDENCE trial. This was a randomized, controlled, multicenter trial which compared the efficacy and safety of Rebif® 44 µg subcutaneously three times weekly and Avonex® 30 µg i.m. once weekly in 677 patients with RRMS. The primary endpoint was the proportion of patients who were relapse free at 24 weeks; the principal MRI endpoint was the number of active lesions per patient per scan at 24 weeks. After 24 weeks, 74.9% of patients receiving Rebif® 44 µg three times a week remained relapse free compared with 63.3% of those given Avonex® 30 µg once a week. Patients receiving Rebif® 44 µg three times a week

had fewer active MRI lesions ($p = 0.001$ at 24 and 48 weeks) compared with those receiving Avonex® 30 µg once a week. Injection-site reactions were more frequent with Rebif® 44 µg three times a week (83% versus 28%, $p = 0.001$), and there were asymptomatic abnormalities of liver enzymes (18% versus 9%, $p = 0.002$) and altered leukocyte counts (11% versus 5%, $p = 0.003$) compared with the Avonex® 30 µg once a week dosage. Neutralizing antibodies developed in 25% of Rebif® 44 µg three times a week patients and in 2% of patients receiving Avonex® 30 µg once a week [38].

Rebif® has also been studied in the SPMS population. The SPECTRIMS study was a multicenter, randomized, parallel-group, placebo-controlled study which tested two doses of Rebif® in patients with SPMS. Patients had to have clinically definite SPMS which was defined as progressive deterioration of disability for at least 6 months with an increase of at least 1 EDSS point over the previous 2 years (or 0.5 point between EDSS score of 6.0 and 6.5), with or without relapses, following an initial course of RRMS. Baseline EDSS scores had to be from 3.0 to 6.5 and the pyramidal functional score of at least 2. Patients were randomized to 3-year treatment with placebo or IFN β-1a (22 or 44 µg subcutaneously three times weekly). The primary outcome was time of confirmed progression, defined as increase from baseline by at least 1 EDSS point (or 0.5 point if baseline EDSS ≥ 5.5), confirmed 3 months later with no intervening score lower than the minimum required level. The primary outcome was not significantly influenced by treatment with Rebif® as compared to placebo ($p = 0.146$). A significant benefit was seen on relapse rate for both doses of Rebif®. These findings suggest that treatment with Rebif® has clinical benefit in SPMS, predominantly affecting relapses, but only modest effect on disability [39].

In controlled clinical trials, the most commonly observed adverse reactions were: injection-site reactions, influenza-like symptoms (headache, fatigue, fever, rigors, chest pain, back pain, myalgia), elevated liver enzymes, hematological abnormalities, abdominal pain, and depression. Most of these adverse reactions are unique to treatment with interferon beta, presenting potential difficulties in maintaining blinding in controlled clinical trials. Summary of the most frequent Rebif® adverse reactions by Medical Dictionary for Regulatory Activities (MedDRA) system organ class have been summarized in Table 1.

**Table 1: Summary of the Most Frequent Rebif® Adverse Reactions by MedDRA System Organ Class**

| System Organ Class | Very Common ADR frequency of occurrence ≥ 1/10 | Common ADR frequency of occurrence ≥ 1/100 to 1/10 |
|---|---|---|
| **General disorders and administration site conditions** | Injection site inflammation, injection site reaction, influenza-like symptoms | Injection site pain, fatigue, rigors, fever |
| **Investigations** | Asymptomatic transaminase increase | Severe elevation of transaminase |
| **Blood and lymphatic system disorders** | Neutropenia, lymphopenia, leucopenia, thrombocytopenia, anaemia | |
| **Psychiatric disorders** | | Depression, insomnia |
| **Nervous system disorders** | Headache | |
| **Gastrointestinal disorders** | | Diarrhoea, vomiting, nausea |
| **Skin and subcutaneous tissue disorders** | | Pruritus, rash, erythematous rash, macula-papular rash |
| **Musculoskeletal and connective tissue disorders** | | Myalgia, arthralgia |

ADR=adverse drug reaction; MedDRA=Medical Dictionary for Regulatory Activities.
Based on Rebif® Summary of Product Characteristic 2010 [40]. Please refer to local label for more details.

Severe liver injury, including some cases of hepatic failure requiring liver transplantation, has been reported rarely in patients taking Rebif®. Treatment with Rebif® should be stopped immediately if jaundice or other symptoms of liver dysfunction appear [40, 41]. Please refer to Section 6.2.2 for further details.

Neutralizing antibodies to IFN β-1a can develop in some patients, usually following the first year of therapy. Long-term consequences of these antibodies are still not known, however, current evidence shows that they may reduce the efficacy of the drug. The antibodies tend to cross-react with different IFN β formulations. For this reason, switching to another IFN β drug is unlikely to be effective [42, 43].

## 1.2 Rationale for the Study

This study is a pivotal Phase III clinical trial, and is composed of the following periods: a double-blind, double-dummy treatment period, a Safety Follow-Up Period, and an Open-Label Extension Phase. The double-blind, double-dummy treatment period is designed to demonstrate the efficacy and safety of ocrelizumab in relapsing MS in comparison to high-dose, high-frequency (HDHF) IFN (Rebif®). The Open-Label Extension Phase

serves to evaluate long-term safety, tolerability, and efficacy of ocrelizumab treatment in patients with relapsing forms of MS.

This study is part of a broader, confirmatory clinical development program investigating the safety and efficacy of ocrelizumab in patients with both primary progressive and relapsing MS. An Open-Label Extension Phase of the Phase II study WA21493/ACT4422G is ongoing for eligible RRMS patients. There are three ongoing Phase III pivotal trials (including the one presented in this protocol), two in RMS and one in PPMS. Please refer to Section 3.1.1 for further details on study design and choice of comparator.

# 2. OBJECTIVES

## 2.1 Primary Objective

The primary objective of this study is to assess whether the efficacy of ocrelizumab 600 mg (given as dual infusions of 300 mg on Days 1 and 15 of the first 24-week treatment cycle and as a single infusion of 600 mg on Day 1 of each 24-week treatment cycle thereafter) intravenously every 24 weeks is superior to Rebif® as measured by the annualized protocol-defined relapse rate (see Section 5.3.2.1 for the definition of protocol-defined relapse) by two years (96 weeks) in patients with relapsing multiple sclerosis.

## 2.2 Secondary Objectives

The *key* secondary objectives of this study are to evaluate whether the efficacy of ocrelizumab is superior to Rebif®, as reflected by the following measures:

- The time to onset of *confirmed* disability progression for at least 12 weeks *with the* initial event of neurological worsening occur*ring* during the 96-week, double-blind, double-dummy, treatment period (see Section 5.3.2.2 for the definition of *confirmed* disability progression).
- *The total number of T1 Gd-enhancing lesions as detected by brain MRI at Weeks 24, 48, and 96*
- The total number of new, and/or enlarging T2 hyperintense lesions as detected by brain MRI at Weeks 24, 48, and 96.
- *The proportion of patients who have confirmed disability improvement for at least 12 weeks with the initial event of neurological improvement occurring during the 96-week double-blind, double-dummy treatment period.*
- The time to onset of *confirmed* disability progression for at least 24 weeks, *with the* initial event of neurological worsening occur*ring* during the 96-week, double-blind, double-dummy, treatment period (see Section 5.3.2.2 for the definition of *confirmed* disability progression).
- *The total number of T1-hypo-intense lesions (Chronic Black Holes) at Weeks 24, 48, and 96*
- The change in Multiple Sclerosis Functional Composite Scale (MSFCS) score from baseline to Week 96.
- The *percentage* change in brain volume as detected by brain MRI from Week 24 to Week 96.

- *The change in SF-36 Physical Component Summary (PCS) Score from baseline to Week 96*
- *The proportion of patients who have no evidence of disease activity (NEDA) by Week 96*

**Safety:**

To evaluate the safety and tolerability of ocrelizumab 600 mg (given as dual infusions of 300 mg on Days 1 and 15 of the first 24-week treatment cycle and as a single infusion of 600 mg on Day 1 of each 24-week treatment cycle thereafter) intravenously every 24 weeks in patients with relapsing MS (including exploratory, long-term safety and tolerability in those patients entering the OLE Phase).

**Pharmacokinetics/Pharmacodynamics:**

To explore the pharmacokinetics (PK), immunogenicity, and pharmacodynamics (PD) of ocrelizumab in patients with relapsing MS.

## 2.3 Exploratory Objectives

- The change in low contrast visual acuity from baseline to Weeks 48 and 96.
- The change in the Symbol Digit Modalities Test from baseline to Weeks 48 and 96.
- *The proportion of relapse free patients by Week 96.*
- *The change in total T2 hyperintense lesion volume as detected by brain MRI from baseline to Week 96.*
- The annualized relapse rate, based on *all* clinical relapses at the end of the 96-week comparative treatment period *(protocol-defined relapses are a subset of all clinical relapses)*.
- *The ARR of relapses requiring IV steroid therapy.*
- *The ARR of severe relapses.*
- The *percentage* change in brain volume as detected by brain MRI from baseline to Week 96.
- The change in Multiple Sclerosis Functional Composite Scale (MSFCS) score from baseline to Week 48.
- The cumulative change in EDSS scores, measured in area under the curve (AUC) by Week 96.
- The change in EDSS from baseline to Week 96.
- The change in timed 25-foot walk from baseline to Week 96.
- The change in 9-hole peg test from baseline to Week 96.
- The change in paced auditory serial addition test (PASAT) from baseline to Weeks 48 and 96.
- The time to onset of sustained 20% increase in 9-hole peg test for at least 12 weeks.
- The time to onset of sustained 20% increase in timed 25 foot walk for at least 12 weeks.
- *The change in fatigue, as measured by the Modified Fatigue Impact Scale (MFIS) total score from baseline to Week 96.*

- *The change in patient-reported depressive symptoms, as measured by the Center for Epidemiologic Studies Depression Scale (CES-D), from baseline to Week 96.*
- *Analyses of EQ-5D, collected at baseline, Week 48, and Week 96.*
- The change in Karnofsky Performance Status Scale from baseline to Week 96.
- *The percentage change in cortical grey matter volume from baseline to Week 96.*
- *The percentage change in white matter volume from baseline to Week 96.*
- *The proportion of patients who have confirmed disability improvement sustained for at least 24 weeks, with the initial event of neurological improvement occurring during the 96-week double-blind double-dummy treatment period.*
- *The proportion of patients who have disability improvement sustained for at least 12 weeks and sustained until the end of the 96-week, double-blind, double-dummy treatment period, with the initial event of neurological improvement occurring during the 96-week, double-blind, double-dummy treatment period.*
- *The duration of the confirmed disability improvement.*
- *The proportion of patients who, at Week 96, have improved, stable, or worsened disability, compared to baseline.*
- *The change in Quality of Life, as measured by the Short Form 36 version 2 Mental Component Summary (MCS) Score from baseline to Week 96.*
- To evaluate the long-term safety, tolerability, and efficacy of ocrelizumab in patients with relapsing form of MS who are enrolled in the OLE Phase.

## 2.4 Roche Clinical Repository Exploratory Objectives

The Roche Clinical Repository (RCR) is a centrally administered facility for the long-term storage of human biological specimens including body fluids, solid tissues and derivatives thereof (e.g., DNA, ribonucleic acid [RNA] proteins/ peptides). Specimens stored in the RCR will be used to:

- Study the association of biomarkers with efficacy and/ or adverse events associated with ocrelizumab.
- Increase the knowledge and the understanding of biology of MS and mode of action of ocrelizumab.

## 2.5 Optional Exploratory Substudies

Consenting patients who enrolled in the main study WA21093 and who are eligible will be offered the opportunity to participate in optional substudies. Please refer to Section 5.9 for details.

### 2.5.1 Optical Coherence Tomography Exploratory Substudy

This substudy will be conducted at certain selected centers and will be used to evaluate the neuroprotective effect of ocrelizumab as measured by retinal nerve fiber layer (RNFL) thickness and macular volume in both eyes (see Section 5.9.1 or Appendix 8).

## 3. STUDY DESIGN

### 3.1 Overview of Study Design and Dosing Regimen

**Figure 1: Overview of Study Design**

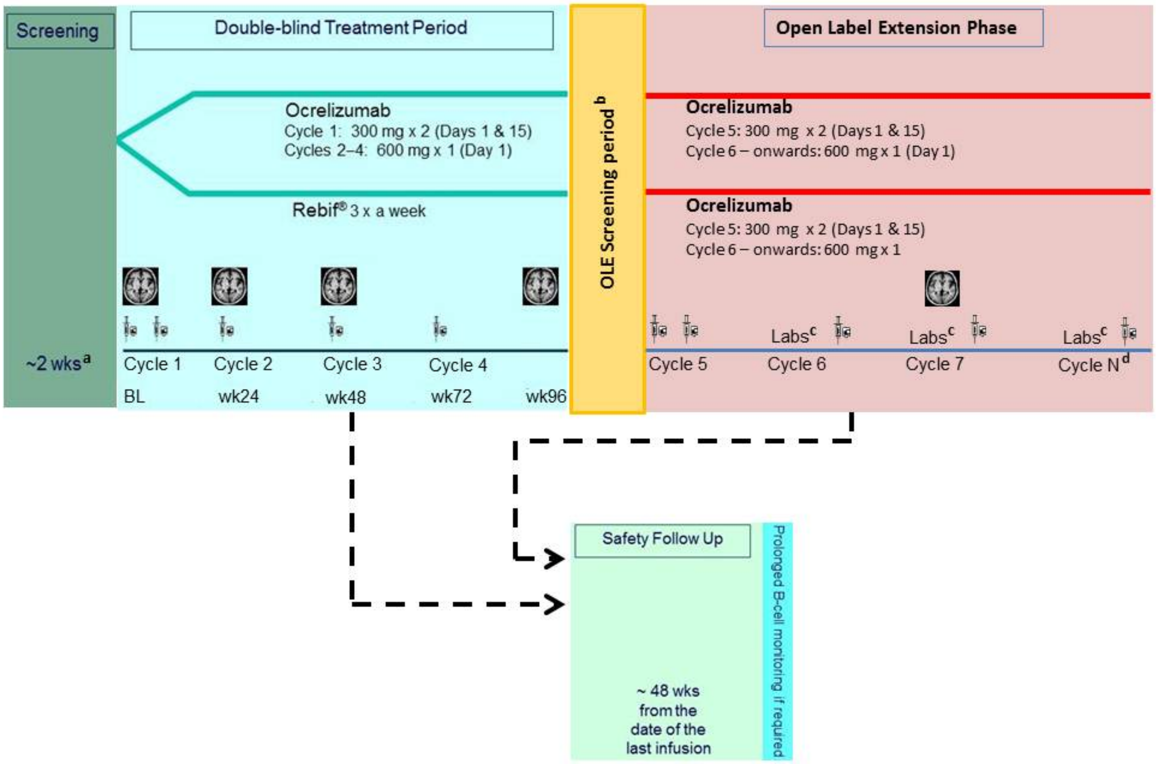


a. Screening for the study may be prolonged for up to 8 weeks for relevant clinical, administrative, or operational reasons.
b. The OLE Phase Screening Period will start after all assessments at the Week 96 Visit have been done. It will last up to 4 weeks. It is possible that for particular reasons, the OLE Phase Screening Period could be longer than 4 weeks. If a prolongation of the OLE Phase Screening Period is needed, it should be discussed with the Sponsor on a case-by-case basis. Note that during the OLE Phase Screening Period, patients should receive Rebif®/Rebif® placebo until the first infusion of Cycle 5.
c. In order to verify if patients meet re-treatment criteria, patients in the Open-Label Extension Phase of the study should come to the clinic approximately 2 weeks prior to infusions of Cycle 6, 7, etc.
d. The OLE Phase of the study can be terminated at any time (please refer to Section 3.1.4 and Section 5.10). Cycle N represents a typical cycle that occurs every 24 weeks.

The study will consist of the following periods:

**Screening:**

Consenting patients will enter a Screening period to be evaluated for eligibility. The Screening period will last approximately 2 weeks, but it may be prolonged for up to 8 weeks for relevant clinical, administrative, or operational reasons. Procedures at screening will include collecting medical history, medical examination including thorough neurological exam, EDSS score, ECG, blood and urine sampling. Please see Table 4 - "Schedule of Assessments: Screening Through the End of Double-Blind, Double-Dummy Treatment Period" for further details.

Please note that based on local Ethics Committees or National Competent Authority requirements, additional diagnostic testing may be required for selected patients or selected centers to exclude tuberculosis, Lyme disease, HTLV-1 associated myelopathy (HAM), acquired immune deficiency syndrome (AIDS), hereditary disorders, connective tissue disorders, or sarcoidosis.

**Treatment Period:**

**Double-blind, double-dummy, comparative treatment period**

Eligible patients will be randomized via an Interactive Voice and Web Response System (IxRS) into one of two treatment groups: ocrelizumab 600 mg regimen (group A) or interferon β-1a - Rebif® (group B) – please refer to Table 7 and Table 10 for more details.

During the double-blind, double-dummy comparative treatment period, patients will be assessed at clinical visits as per the Schedule of Assessments: Screening Through the End of Double-Blind, Double-Dummy Treatment Period – please refer to Table 4 for further details.

Prior to the next cycle of study drug, patients will be evaluated for pre-specified conditions and laboratory abnormalities to allow for re-treatment (please refer to Section 6.1.4 for more details).

Patients who discontinue from study medication within the 96-week, double-blind, double-dummy, comparative phase (treatment period) of the study will enter the Safety Follow-Up Period (see below); they will not be eligible for the Open-Label Extension Phase.

**Open-Label Extension Phase Screening Period**

Patients who complete the 96-week treatment period may become eligible for the OLE Phase of the study. Patients will be consented for participation in the OLE Phase if, in the opinion of the Investigator, they may benefit from treatment with ocrelizumab. Patients who are not willing to participate in the OLE Phase of the study will be entered into the Safety Follow-Up Period (see below). Patient treatment allocation during the double-blind, double-dummy treatment period should not be unblinded regardless of participation in the OLE Phase.

In the case of a patient who initially declines participation in the OLE Phase and subsequently reconsiders the decision, the patient will have up to 24 weeks after the Week 96 Visit to enter the OLE Phase. In this instance, he or she should not have taken any prohibited medication as specified in Section 4.5.2.1. Patients who decline participation in the OLE Phase should enter the Safety Follow-Up Period.

Patients who have consented to participate in the OLE Phase will enter an OLE Phase Screening Period to be evaluated for eligibility. The OLE Phase Screening Period will start after all assessments at the Week 96 Visit have been performed. This screening period will last up to 4 weeks. It is possible that the OLE Phase Screening Period could be longer than 4 weeks. If a prolongation of the OLE Phase Screening Period is needed, it should be discussed with the Sponsor on a case-by-case basis.

Information from assessments performed during the Week 96 Visit will be utilized to verify the eligibility of the patient for the OLE Phase of the study. Please refer to Section 4.4.

During the OLE Phase Screening Period, all patients should receive Rebif®/Rebif® placebo (depending on initial arm assigned to) until the first infusion of Cycle 5. Please refer to Table 7 and Table 10 for more details regarding the Rebif®/Rebif® placebo regimen.

Patients who withdraw from the OLE Phase Screening Period will be entered into the Safety Follow-Up Period (see below).

**Open-Label Extension Phase**

Duration: The OLE Phase will continue until ocrelizumab is commercially available in the patient's country, or as per local regulation, or should the Sponsor decide to terminate the ocrelizumab program for MS, but will not exceed 4 years after the last patient to reach the Week 96 Visit in the double-blind, double-dummy treatment period.

*In the United Kindgom, the OLE Phase will last for 4 years. The 4-year duration of the OLE Phase serves to evaluate long-term safety, tolerability, and efficacy of ocrelizumab treatment in patients with relapsing forms of MS (please refer to Section1.2).*

Treatment: During the OLE Phase, all patients will receive the ocrelizumab 600 mg regimen every 24 weeks (please refer to Table 3 and Section 5.10 for more details).

Withdrawal: Patients who withdraw from the OLE Phase will be entered into the Safety Follow-Up Period (see below).

**Safety Follow-Up Period**

Patients who discontinue treatment *prematurely* for any reason during the following periods will be entered into the Safety Follow-Up Period:

- Prior to completion of the 96-week, double-blind, double-dummy treatment period;
- During the OLE Phase Screening Period;
- During the OLE Phase;
- Patients who choose not to enter the OLE Phase or are not eligible for the OLE Phase after completing the 96-week, double-blind, double-dummy treatment period.

In the OLE Phase Screening Period, in the case of a patient who initially declines participation in the OLE Phase and subsequently reconsiders the decision, the patient will have up to 24 weeks after the Week 96 Visit to enter the OLE Phase. In this instance, he or she should not have taken any prohibited medication as specified in Section 4.5.2.1.

The Safety Follow-Up Period will last for at least 48 weeks starting from the date of the last infusion of the ocrelizumab/ocrelizumab placebo. Safety follow-up visits will be

performed at 12-week intervals starting from the date of patient's last visit (Withdrawal from Treatment Visit). However, if after the Safety Follow-Up Period the peripheral blood B-cells remain depleted, the patient should continue to be monitored at 24-week intervals until the B-cell count has returned to the baseline value or to the lower limit of the normal range (whichever is the lower). Please refer to Figure 2 and to Section 4.5.2.1 for more details.

**Figure 2: Safety Follow-Up - Variable B-cell Monitoring**

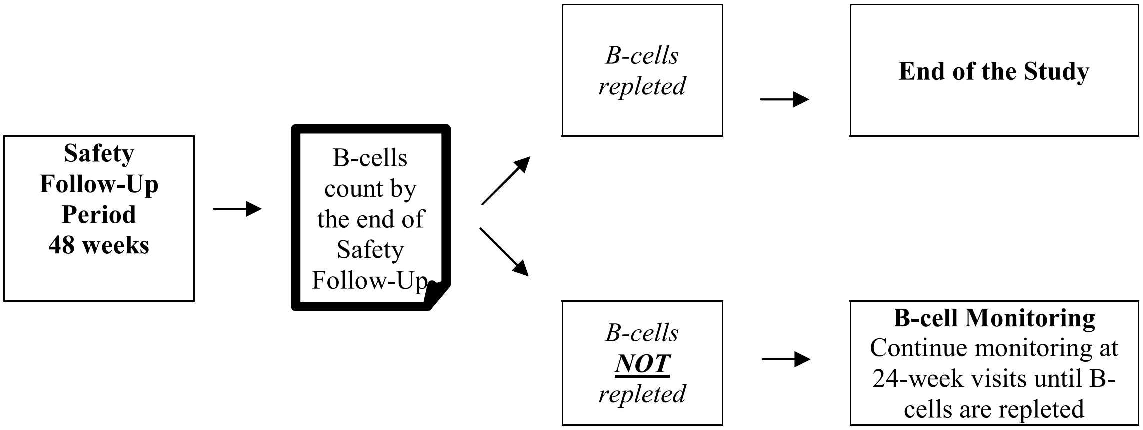


Please note: patients in Safety Follow-Up who receive other therapies that may decrease B-cell level will only be followed for 48 weeks from the date of the last infusion of the study drug regardless of their B-cell count.

During Safety Follow-up patients will be assessed at clinical visits every 12 weeks. Telephone interviews will be performed every 4 weeks. If prolonged B-cell monitoring is required, patients will be assessed at clinical visits every 24 weeks and telephone interviews will be performed every 12 weeks. Please refer to Schedule of Assessments: Safety Follow-Up Period (including prolonged B-cell monitoring if required)—Table 5**Error! Reference source not found.**—for further details.

**Every effort should be made to have patients who withdraw from Study Medication complete the Safety Follow-Up Period and all related assessments, regardless of whether or not they receive alternative treatment for MS.**

**Table 2: Overview of Dosing Regimen in the Double-Blind, Double-Dummy Treatment Period**

| Study Medication | **Double-Blind, Double-Dummy Treatment Period[1,2]** | | | | |
|---|---|---|---|---|---|
| | **1st Cycle[3]** (Weeks 1-24) | | **2nd Cycle[3]** (Weeks 24-48) | **3rd Cycle[3]** (Weeks 48-72) | **4th Cycle[3]** (Weeks 72-96) |
| | **Day 1 Infusion** | **Day 15 Infusion** | **Week 24 Infusion** | **Week 48 Infusion** | **Week 72 Infusion** |
| **A Ocrelizumab 600 mg regimen** | **Ocrelizumab** 300 mg i.v. | **Ocrelizumab** 300 mg i.v. | **Ocrelizumab** 600 mg i.v. | **Ocrelizumab** 600 mg i.v. | **Ocrelizumab** 600 mg i.v. |
| **B Rebif® regimen[4]** | **Rebif®** s.c. 3 times per week | → | → | → | → |

i.v.= intravenous; s.c.= subcutaneous.

1. The double-blind, double-dummy treatment period consists of 96 weeks of treatment (4 treatment cycles).
2. Each treatment cycle has a duration of 24 weeks. The first cycle consists of two 300 mg ocrelizumab i.v. infusions separated by 14 days. Cycles 2 – 4 consist of a single i.v. infusion of 600 mg ocrelizumab.
3. Prior to each infusion, a clinical evaluation will be performed to ensure that the patient remains eligible for treatment.
4. Please refer to Table 10 for detailed Rebif® dosing regimen.

Please note: 100 mg of methylprednisolone i.v. will be administered in both treatment arms prior to each infusions of ocrelizumab/ocrelizumab placebo.

**Table 3: Overview of Dosing Regimen *in* the OLE Phase Screening Period and the OLE Phase**

| Study Medication | OLE Phase Screening Period | OLE Phase[1] | | | | |
|---|---|---|---|---|---|---|
| | | 5th Cycle[2,3] | | 6th Cycle[2,3] | 7th Cycle[2,3] | Nth Cycle[2,3,4] |
| | | Day 1 Infusion | Day 15 Infusion | | | |
| **Ocrelizumab 600 mg regimen** | -.-[5] | **Ocrelizumab** 300 mg i.v. | **Ocrelizumab** 300 mg i.v. | **Ocrelizumab** 600 mg i.v. | **Ocrelizumab** 600 mg i.v. | **Ocrelizumab** 600 mg i.v. |
| **Rebif® regimen** | **Rebif®** s.c. 3 times per week[6] | -.-[7] | -.-[7] | -.-[7] | -.-[7] | -.-[7] |

i.v.=intravenous; OLE=Open-Label Extension; s.c.=subcutaneous.

1. The OLE Phase can terminate at any moment or cycle (please refer to End of Study Section 3.1.4).
2. The assessments requested for N represents the typical schedule of assessments during a cycle.
3. Prior to each infusion, a clinical evaluation will be performed to ensure that the patient remains eligible for treatment.
4. Each treatment cycle has a duration of 24 weeks. The first cycle of the OLE Phase consists of two 300 mg ocrelizumab i.v. infusions separated by 14 days. Cycle 6 onwards consists of a single i.v. infusion of 600 mg ocrelizumab.
5. During the OLE Phase Screening Period there will be no administration of ocrelizumab.
6. Please refer to *Table 10* for the detailed Rebif® dosing regimen.
7. During the OLE Phase, there will be no administration of Rebif® verum or placebo.

Please note: 100 mg of methylprednisolone i.v. will be administered in both treatment arms prior to each infusion of ocrelizumab/ocrelizumab placebo.

### 3.1.1 Rationale for Study Design

**Rationale for the use of an active comparator**

There is consensus in the MS community, that the use of placebo in Phase III studies of patients with RMS is (except in exceptional circumstances) ethically indefensible, due to the availability of established, effective therapies [44].

**Rationale for choice of active comparator**

Interferon β-1a 44 µg s.c. 3 times weekly (Rebif®) has been chosen as the active comparator for the ocrelizumab RMS, Phase III clinical program, based on its consistent evidence of efficacy on reducing MRI activity, relapses, and *disability* progression in patients with relapsing forms of MS (see Section 1.1.5).

**Rationale for double-blind, double-dummy study design**

The use of a double-blind, double-dummy study design minimizes the potential for bias and safeguards the integrity of the clinical data generated from this study. It is acknowledged that this approach increases patient's burden. However, this design reduces the risk of concluding that superiority to the active comparator was driven by patient and assessor bias. Regulatory agencies have mandated this study design be implemented throughout the Phase III clinical program. For additional measures intended to minimize bias, please see below.

**Rationale for choice of study population**

This study plans to enroll RMS patients with an EDSS score of 0 to 5.5 at screening who had two documented clinical attacks within the previous 2 years or one clinical attack that occurred within the last year prior to screening. These criteria have been implemented to further characterize the benefits of treatment with ocrelizumab in a wide range of RMS patients with varying degrees of disease activity and severity.

The age range will be limited to ≤55 years to avoid confounding by neurological conditions prevalent in older individuals, including but not limited to microvascular disease.

Exclusion of patients who have failed or cannot tolerate Rebif® prevents these patients from being randomized to further Rebif® therapy and reduces the potential for unbalanced dropout rates. Additional exclusion criteria, relating to concomitant diseases, laboratory parameters, and previous medications help to ensure patient safety in the study – please refer to Section 4.3 for more details.

**Rationale for study endpoints**

The proposed study endpoints are widely accepted as clinically relevant and have been used in numerous pivotal clinical trials, in relapsing MS. The primary endpoint for the study will be ARR over 96 weeks, based on protocol-defined relapses. Key secondary endpoints will include the time to onset of *confirmed* disability progression, confirmed at scheduled clinic visits, for at least 12 weeks. *Confirmed disability progression at 24 weeks will also be examined.* Prevention of relapses as well as the prevention or delay of

accumulation of sustained neurological disability are meaningful goals in the treatment of patients with MS.

**Rationale for the duration of the double-blind, double-dummy treatment period**

The 96-week, double-blind, double-dummy treatment duration has been chosen to allow for the assessment of clinical efficacy and safety over 2 years of treatment, consistent with current regulatory guidelines.

**Rationale for the Open-Label Extension Phase**

Multiple sclerosis is a chronic disease that requires lifelong treatment in order to reduce frequency of relapse and accumulation of disability. Once a patient is diagnosed with definite MS, accepted treatment guidelines propose that he/she be offered lifelong treatment, as there is no evidence to suggest that MS spontaneously remits [45].

Results of long-term, follow-up exploratory studies suggest that exposure to DMT for more than 2 years improves outcomes by delaying the time to *disability* progression. Furthermore, there is accumulating evidence that, in MS, inflammatory damage is a continuous process leading to demyelination and axonal transection, and is the substrate of permanent disability in MS [46, 47, 48].

Based on the long-term efficacy and safety data of the Phase II study WA21493/ACT4422G (for details, please refer to Section 1.1.4.1), it is justified to offer ocrelizumab to patients who would otherwise receive treatment that, in the majority of cases, is modestly effective in reducing the frequency of relapses and in preventing sustained disability [33, 49].

Thus, patients who complete the 96-week, double-blind, double-dummy treatment period will be offered participation in an OLE Phase of the study. Providing patients with the opportunity to prolong treatment with ocrelizumab beyond 2 years will provide more information on the long-term safety of ocrelizumab in RMS, (e.g., the risk of infections/serious infections/opportunistic infections or potential loss of previously acquired immunity [e.g., hypogammaglobulinemia, specific serological titers]), as well as further collection of tolerability and efficacy information from patients with long-term exposure. Furthermore, the OLE Phase will increase the overall number of patients exposed and patient-year exposure, thus increasing the likelihood of detecting rare events prior to launch, and understanding the safety/efficacy profile. Analyzing the long-term safety, tolerability, and efficacy of ocrelizumab is of critical importance to eventually help clinicians make informed decisions on therapy for patients [50].

**Rationale for the Safety Follow-Up Period (including Prolonged B-cell Monitoring)**

Data collected during this period will allow evaluation of B-cell repletion after stopping anti-CD20 treatment and collection of safety and efficacy data to document maintenance of the effect and/or the potential for a withdrawal effect. Based on results obtained from the Phase II Study WA21493 [10] with ocrelizumab up to 80% of the ocrelizumab-treated patients are anticipated to enter the B-cell monitoring portion of the Safety Follow-Up Period with targeted assessment every 24 weeks until their B-cell counts recover.

**Rationale for the use of methylprednisolone**

In order to mitigate the risk and severity of infusion-related reactions, 100 mg of i.v. methylprednisolone will be administered to all patients, approximately 30 minutes prior to administration of ocrelizumab/ocrelizumab placebo. In order to mitigate the risk, that even a low dose of methylprednisolone may have a small effect on the efficacy outcomes of the study, methylprednisolone will be administered to patients in both treatment groups. It is of note that the dose of methylprednisolone used for premedication will be up to 50 times smaller than that used for the symptomatic treatment of relapses in MS. In the event that methylprednisolone is contraindicated for the patient, use of an equivalent dose of an alternative steroid (e.g., dexamethasone) should be used as premedication prior to the infusion.

**Additional measures to mitigate bias**

The use of HDHF IFN β, as the active comparator for this study during the double-blind, double-dummy treatment period, presents some difficulties for maintaining patient and physician blinding – please see Section 1.1.5.

To prevent potential unblinding of the assigned arm in the double-blind, double-dummy treatment period as a result of adverse events or changes to laboratory results, the following, additional measures have been implemented:

- **The Examining Investigator/EDSS assessor** will perform the neurological examination, document the Kurtzke Functional Systems (KFS) scores and assess EDSS scores. During the double-blind, double-dummy treatment period, the Examining Investigator/EDSS assessor and their qualified designees (if applicable) will not be involved with any aspect of medical management of the patient and will not have access to patient data. Every effort will be made to ensure that there is no change in the Examining Investigator/EDSS assessor throughout the course of the study for any individual patient. The Examining Investigator/EDSS assessors will be trained and instructed not to discuss what adverse effects (if any) the patient is experiencing from their medication. Examining Investigator/EDSS assessors will receive training in performing EDSS assessments prior to the beginning of the study and must have successfully passed an examination on performance of the Neurostatus EDSS examination within 24 months of participation. All examining physician/EDSS assessors will receive ongoing training on performance of the Neurostatus EDSS examination throughout the course of the study.
- **Patient education**: During the double-blind, double-dummy treatment period, prior to being examined by the Examining Investigator/EDSS assessor, patients will be instructed not to discuss what (if any) adverse effects they may be experiencing. Treating physicians and/or study coordinators should remind patients of these instructions prior to EDSS assessments and this should be documented in the source documents.
- **Blinded, central MRI assessments**: During the double-blind, double-dummy treatment period, a blinded, central MRI reader will assess all on-study MRI scans.

These assessments will provide independent confirmation of the relative changes in immune-mediated, CNS damage.

– **Blinding of laboratory parameters:** Laboratory parameters that may lead to unblinding to treatment assignment, such as fluorescence-activated cell sorting (FACS) cell counts including $CD19^+$ cells, lymphocyte count, IgM and IgG levels, and type I interferon neutralizing antibody levels will be blinded in all patients, except those meeting unblinding criteria for safety reasons. These laboratory parameters will remain blinded during the double-blind, double-dummy treatment period, Safety Follow-Up Period, OLE Phase Screening Period, and during the first cycle of the OLE Phase (Cycle 5).

### 3.1.2 Rationale for Dose Selection

The dose for the ocrelizumab Phase III clinical program was chosen to bring the MS community significant improvement in clinical efficacy versus current standard of care, with acceptable safety. The dose of ocrelizumab in the Phase III clinical program is 600 mg ocrelizumab every 24 weeks (administered as dual infusions of ocrelizumab 300 mg on Days 1 and 15 of the first 24-week treatment cycle, and 600 mg on Day 1 of each 24-week treatment cycle thereafter). This dose has been established as the lowest, maximally effective dose, based on the results from study WA21493/ACT4422g. The safety of this dose has substantial support from the Phase III clinical program in rheumatoid arthritis (RA), an analogous, although systemic autoimmune disease, in a population at greater risk.

ACT2847g was a Phase I/II, dose escalation study in patients with RA, examining 5 dose regimens. In ACT2847g, the two lowest dose groups (receiving less than 200 mg × 2) demonstrated reduced clinical benefits on some endpoints, earlier return of peripheral B-cell counts and higher rates of immunogenicity. In the RA Phase III program, with the exception of patients recruited from Asia, the dose of 200 mg × 2 established a safety profile comparable to placebo. The higher dose of 500 mg × 2 demonstrated apparently superior efficacy, especially in "high hurdle" clinical endpoints and joint preservation, based on X-ray imaging.

In the MS population (study WA21493/ACT4422g), two doses of ocrelizumab were studied, 2000 mg (administered as dual 1000 mg infusions on Days 1 and 15 of the first, 24-week treatment cycle) and 600 mg (administered as dual 300 mg infusions on Days 1 and 15 of the first treatment cycle). Pre-specified primary and secondary efficacy analyses for Study WA21493/ACT4422g indicate that 300 mg × 2 of ocrelizumab is highly effective in suppressing MRI lesion activity and reducing the risk of clinical relapses in RRMS patients over 24 weeks. No difference in efficacy was seen between the ocrelizumab 1000 mg × 2 and 300 mg × 2 doses, on either MRI or clinical endpoints, in the intent-to-treat (ITT) study population. However, exploratory analyses, stratifying groups according to baseline MRI activity, suggest superior efficacy with 1000 mg × 2 versus 300 mg × 2, at 24 weeks, in patients with MRI activity at baseline (≥ 4 enhancing lesions). Similarly, in these patients, the 1000 mg × 2 dose was apparently more effective than the 300 mg × 2 dose at Week 24 and (to a lesser extent) at Week 48, in reducing the

absolute number of clinical relapses. Neither the MRI nor the clinical efficacy differences are statistically significant; however, these results suggest reduction of clinical efficacy at lower doses, in active MS patients. This apparent dose effect was seen despite the fact that linear kinetics (so that complete receptor occupancy can reasonably be assumed) and near complete peripheral CD19 suppression were observed for both doses. Preclinical studies in primates have shown differential susceptibility of tissue resident versus circulating B-cell populations in response to anti-CD20 antibodies [51, 52, 53]. As tissue resident B-cell populations are beyond our ability to measure directly, it is likely that peripheral CD19 count is a sensitive but non-specific PD marker for anti-CD20 efficacy.

**Conclusion**

Based on available data, the dose of 600 mg of ocrelizumab i.v. (given as dual infusions of 300 mg 14 days apart for the first 24 weeks and a single infusion of 600 mg every 24 weeks thereafter) is the most likely dose to be able to demonstrate robust clinical efficacy, an acceptable safety profile and a low risk of immunogenicity, maximizing the likelihood of significant benefit versus standard of care in patients with relapsing MS.

### 3.1.3 End of Double-Blind, Double-Dummy Treatment Period of the Study

The end of the double-blind, double-dummy treatment period of the study is defined as the date at which the last data point during double-blind, double-dummy treatment from the last patient is received, as required for statistical analysis defined in the *Statistical* Analysis Plan (*S*AP).

*In order to maintain integrity of the study data, all study sites and all EDSS assessors will remain blinded to patient treatment allocation until approximately 24 weeks after the Week 96 visit of the last patient randomised, to allow the confirmation of the last 24 week-confirmed disability progression event (see Section8.2).*

### 3.1.4 End of Study

The end of study is defined as either the last patient last visit of the OLE Phase or the last patient last visit in the B-cell monitoring of the Safety Follow-Up Period, whichever is later.

The OLE Phase will continue until ocrelizumab is commercially available in the patient's country, or as per local regulation, or should the Sponsor decide to terminate the ocrelizumab program for MS, but will not exceed 4 years after the last patient to reach the Week 96 Visit in the double-blind, double-dummy treatment period.

The B-cell monitoring of the Safety Follow-Up Period of each patient will last until the B-cell count has returned to the baseline value or to the lower limit of the normal range (whichever is lower).

## 3.2 Number of Subjects / Assignment to Treatment Groups

Approximately 800 patients (400 per treatment arm) will be recruited over a planned recruitment period of 16 months.

Patients will be randomized in 2 groups in a 1:1 ratio. An independent IxRS provider will conduct randomization and hold the treatment assignment code. Patients will be stratified by region (US versus ROW) and baseline EDSS (< 4 and ≥ 4).

### 3.3 Centers

This will be a multicenter, international study. It is anticipated that approximately 220 centers worldwide will participate.

## 4. STUDY POPULATION

Under no circumstances are patients who enroll in this study permitted to be re-randomized to this study and enrolled for a second course of treatment.

### 4.1 Overview

Adult patients with relapsing MS who fulfill the eligibility criteria specified in Sections 4.2 and 4.3 are eligible for enrollment into the study. For the eligibility criteria for the OLE Phase, please see Section 4.4.

#### 4.1.1 Recruitment Procedures

Sites are encouraged to identify patients for potential recruitment using pre-screening enrollment logs or a pre-ID website.

Patients who are candidates for enrollment into the study will be evaluated for eligibility by the Investigator to ensure they fulfill eligibility criteria (please refer to Sections 4.2 and 4.3).

All patients must sign the informed consent form prior to screening and prior to any changes to their existing medication for the purposes of enrollment into the trial.

No patient may begin treatment prior to randomization and assignment of a medication number. Under no circumstances are patients who enroll in this study permitted to be re-randomized to this study.

The Investigators will be notified by the Sponsor if the study is placed on clinical hold and when the study is completed or closed to further patient enrollment.

No replacement for patients who withdraw from the study after randomization is planned.

### 4.2 Inclusion Criteria

1. Ability to provide written, informed consent and be able to follow the schedule of protocol assessments *.
2. Ages 18-55 years at screening, inclusive.
3. Diagnosis of MS, in accordance with the revised McDonald criteria (2010).
4. At least 2 documented clinical attacks within the last 2 years prior to screening, or one clinical attack in the year prior to screening (but not within 30 days prior to screening).
5. Neurological stability for ≥ 30 days prior to both screening and baseline.
6. EDSS, at screening, from 0 to 5.5 inclusive.
7. Documented MRI of brain with abnormalities consistent with MS prior to screening.

8. Patients of reproductive potential must use reliable means of contraception as described below as a minimum (adherence to local requirements, if more stringent, is required**):
   - For female patients: Two methods of contraception throughout the trial, including the active treatment phase AND for 48 weeks after the last dose of ocrelizumab, or until their B-cells have repleted, whichever is longer.
   - For male patients: Two methods of contraception throughout the trial, including the active treatment phase AND for 24 weeks after the last dose of ocrelizumab. Acceptable methods of contraception include one primary (e.g., systemic hormonal contraception or tubal ligation of the female partner, vasectomy of the male partner) AND one secondary barrier method (e.g., latex condoms, spermicide) OR a double barrier method (e.g., latex condom, intrauterine device, vaginal ring or pessary plus spermicide [e.g., foam, vaginal suppository, gel, cream]).
9. For patients of non reproductive potential (adherence to local requirements, if more stringent, is required**):
   - Women may be enrolled if postmenopausal (i.e., spontaneous amenorrhea for the past year confirmed by a follicle-stimulating hormone [FSH] level greater than 40 mIU/mL, unless the patient is receiving a hormonal therapy for their menopause or surgically sterile (i.e., hysterectomy, complete bilateral oophorectomy);
   - Men may be enrolled if they are surgically sterile (castration).

* Patients who are unable to complete exploratory assessments (e.g., electronic patient reported outcomes [ePROs]) due to physical/disease limitations will not be excluded from the study.

** Based on local Ethics Committees or National Competent Authority feedback, additional requirements to assure contraception or to confirm menopause may be required (e.g., serum estradiol compatible with post-menopause status, longer duration of amenorrhea, higher level of FSH).

## 4.3 Exclusion Criteria

Patients who meet the following criteria must be excluded from study entry:

1. Diagnosis of primary progressive MS.
2. Disease duration of more than 10 years in patients with an EDSS ≤2.0 at screening.
3. Inability to complete an MRI (contraindications for MRI include but are not restricted to weight ≥140 kg, pacemaker, cochlear implants, presence of foreign substances in the eye, intracranial vascular clips, surgery within 6 weeks of entry into the study, coronary stent implanted within 8 weeks prior to the time of the intended MRI, etc)

   (Patients with contraindication to Gd can be enrolled into the study but cannot receive Gd contrast dyes during their MRI scans.)
4. Known presence of other neurological disorders that may mimic MS, including but not limited to: neuromeylitis optica, Lyme disease, untreated vitamin B12 deficiency, neurosarcoidosis, and cerebrovascular disorders.

**Exclusions Related to General Health**

5. Pregnancy or lactation.
6. Any concomitant disease that may require chronic treatment with systemic corticosteroids or immunosuppressants during the course of the study.
7. History or currently active primary or secondary immunodeficiency.
8. Lack of peripheral venous access.
9. History of severe allergic or anaphylactic reactions to humanized or murine monoclonal antibodies.
10. Significant or uncontrolled somatic disease or any other significant disease that may preclude patient from participating in the study.
11. Congestive heart failure (NYHA III or IV functional severity).
12. Known active bacterial, viral, fungal, mycobacterial infection or other infection, excluding fungal infection of nail beds.
13. Infection requiring hospitalization or treatment with i.v. antibiotics within 4 weeks prior to baseline visit or oral antibiotics within 2 weeks prior to baseline visit.
14. History or known presence of recurrent or chronic infection (e.g., HIV, syphilis, tuberculosis [TB]).
    *Please note: in Germany the following additional exclusion criteria apply:*
    - *Positive anti – HIV I at screening*
    - *Positive anti – HIV II at screening*
    - *Positive QuantiFERON®-TB Gold test at screening*

    *Patients in Germany with an indeterminate result are not eligible for the study unless additional testing demonstrating a negative result is provided. Thus, these patients should have either a tuberculin skin test or have the QuantiFERON® TB Gold test repeated prior to enrollment into the study. If a tuberculin skin test is performed, an induration of ≥ 6 mm is "positive" for a patient with history of BCG vaccine, while an induration of ≥ 10 mm is "positive" for a patient without history of BCG vaccine.*
    *If necessary a QuantiFERON®-TB Gold test might be complemented by additional specific diagnostic tests as per standard procedures in Germany.*
15. History of PML
16. History of malignancy, including solid tumors and hematological malignancies, except basal cell carcinoma, *in situ* squamous cell carcinoma of the skin, and *in situ* carcinoma of the cervix of the uterus that have been previously completely excised with documented, clear margins.
17. History of alcohol or drug abuse within 24 weeks prior to baseline.
18. History or laboratory evidence of coagulation disorders.

**Exclusions Related to Medications***

19. Receipt of a live vaccine within 6 weeks prior to the baseline visit.
    In rare cases when patient requires vaccination with a live vaccine, the screening period may be extended but cannot exceed 8 weeks.
20. Treatment with any investigational agent within 24 weeks of screening (Visit 1) or five half-lives of the investigational drug (whichever is longer; or treatment with any

experimental procedures for MS [e.g., treatment for chronic cerebrospinal venous insufficiency]).

21. Contraindications to or intolerance of oral or i.v. corticosteroids, according to the country label, including:
    a) Psychosis not yet controlled by a treatment;
    b) Hypersensitivity to any of the constituents.
22. Contraindication to Rebif® or incompatibility with Rebif® use, including:
    a) Current severe depression and/or suicidal ideation;
    b) Hypersensitivity to natural or recombinant interferon-β, or to any excipients;
    c) Previous suboptimal response to HDHF IFN or cessation of HDHF IFN therapy due to poor tolerability;
    d) Prior cessation of Rebif® therapy due to toxicity, which is likely to recur.
23. Treatment with dalfamipridine (Ampyra®) unless on stable dose for ≥30 days prior to screening. Wherever possible, patients should remain on stable doses throughout the 96-week treatment period.
24. Previous treatment with B-cell targeted therapies (i.e., rituximab, ocrelizumab, atacicept, belimumab, or ofatumumab).
25. Systemic corticosteroid therapy within 4 weeks prior to screening.**
26. Any previous treatment with alemtuzumab (Campath), anti-CD4, cladribine, mitoxantrone, daclizumab, teriflunomide, laquinimod, total body irradiation, or bone marrow transplantation.
27. Treatment with cyclophosphamide, azathioprine, mycophenolate mofetil (MMF), cyclosporine, MTX, or natalizumab within 24 months prior to screening. NB Patients previously treated with natalizumab will be eligible for this study only if duration of treatment with natalizumab was < 1 year.
28. Treatment with fingolimod (FTY720, Gilenya®) or other S1P receptor modulator (i.e., BAF312), or with BG12, within 24 weeks prior to screening. (NB. Only patients with T lymphocyte count ≥ LLN will be eligible for this study.)
29. Treatment with i.v. immunoglobulin within 12 weeks prior to baseline.

* Patients screened for this study should not be withdrawn from therapies for the sole purpose of meeting eligibility for the trial. Patients who discontinue their current therapy for non-medical reasons should specifically be informed before deciding to enter the study of their treatment options.

** The screening period may be extended (but cannot exceed 8 weeks) for patients who have used systemic corticosteroids for their MS before screening. For a patient to be eligible, systemic corticosteroids should not have been administered also between screening and baseline.

**<u>Exclusions Related to Laboratory Findings</u>***

30. Positive serum β hCG measured at screening.
31. Positive screening tests for hepatitis B (hepatitis B surface antigen [HBsAg] positive, or positive hepatitis B core antibody [total HBcAb] confirmed by a positive viral DNA polymerase chain reaction [PCR]) or hepatitis C (HepCAb).
32. Positive rapid plasma reagin (RPR).
33. CD4 count<300/μL.
34. AST/SGOT or ALT/SGPT ≥ 2.0 × ULN.
35. Platelet count <100,000/μL (<100 × $10^9$/L).

36. Levels of serum IgG 18% below the LLN (for central lab IgG<4.6 g/L).
37. Levels of serum IgM 8% below the LLN (for central lab IgM<0.37 g/L).
38. Total neutrophil count $<1.5 \times 10^3/\mu L$.

*Re-testing before baseline: in rare cases in which the screening laboratory samples are rejected by the central laboratory (example: hemolyzed sample) or the results are not assessable (example: indeterminate) or abnormal, the tests need to be repeated within 4 weeks. Any abnormal screening laboratory value that is clinically relevant should be retested in order to rule out any progressive or uncontrolled underlying condition. The last value before randomization must meet study criteria. In such circumstances, the screening period may need to be prolonged but should not exceed 8 weeks.

Please note: based on local Ethics Committees or National Competent Authority requirements, additional diagnostic testing may be required for selected patients or selected centers to exclude tuberculosis, Lyme disease, HAM, AIDS, hereditary disorders, connective tissue disorders, or sarcoidosis. Other specific diagnostic tests may be requested when deemed necessary by the Investigator.

## 4.4 Eligibility Criteria for Open-Label Extension Phase

Patients who meet the following entry criteria may participate in the OLE Phase:

1. Complete the 96-week, double-blind, double-dummy treatment period, and who, in the opinion of the Investigator, may benefit from treatment with ocrelizumab;
2. Are able and willing to provide written informed consent for the OLE Phase (e.g., before the first infusion at Cycle 5) and to comply with the study protocol;
3. Are willing to continue to use at least two contraceptive methods as described in Section 4.2 (items 8 and 9);
4. Meet re-treatment criteria with ocrelizumab (see Section 6.1.4.)

## 4.5 Concomitant Medication and Treatment

### 4.5.1 Definition of Concomitant Treatment

A concomitant medication is any drug or substance taken during the study, including the screening period. Over the counter medications and preventative vaccines received during the study are considered concomitant medications.

A concomitant procedure is any therapeutic or elective intervention (e.g., surgery, biopsy) or diagnostic evaluation (e.g., blood gas measurements, bacterial cultures) performed during the study, including the screening period.

Concomitant medications and procedures will be reported at each visit in the relevant form of electronic Case Report Forms (eCRFs) starting from the baseline visit (including medication and procedures taken between screening and baseline). Medications taken for the treatment of MS in the 2-year period prior to the baseline visit and medications taken for the symptoms of MS in the 3-month period prior to the baseline visit will be recorded at the baseline visit. Additionally, medications and medical/surgical procedures administered for any non-MS condition within 12 months prior to the baseline visit will also be recorded at the baseline visit.

### 4.5.2 Treatment for Symptoms of Multiple Sclerosis

The Treating Investigator should attempt to maintain therapies or treatments for symptoms related to MS (e.g., walking ability, spasticity, incontinence, pain, fatigue) reasonably constant throughout the study. *During the OLE Phase of the study, initiation of therapy with dalfampridine (Ampyra®) is allowed, if indicated by the treating physician.*

**Treatment of relapses:** patients who experience a relapse during the double-blind, double-dummy treatment period, OLE Phase Screening Period, or the OLE Phase may receive treatment with i.v. or oral corticosteroids, if judged to be clinically appropriate by the Investigator. The following standardized treatment regimen may be used as warranted, 1 g i.v. methylprednisolone per day for a maximum of 5 consecutive days. In addition, at the discretion of the Investigator, corticosteroids may be stopped abruptly or tapered over a maximum of 10 days. Such patients should not discontinue the treatment period solely based on the occurrence of a relapse, unless the patient or Investigator feels he or she has met the criteria for withdrawal (See Section 4.6 for further details).

#### *4.5.2.1 Prohibited Concomitant Treatments*

Therapies for MS noted in the exclusion criteria under "Exclusions Related to Medications" (Section 4.3) are not permitted during the double-blind, double-dummy treatment period, OLE Phase Screening Period, or the OLE Phase with the exception of systemic corticosteroids for the treatment of a relapse.

After patients have finished the treatment with ocrelizumab, they may receive alternative treatment for their MS as judged clinically appropriate by the Treating Investigator. However, treatment with immunosuppressants, lymphocyte depleting agents, or lymphocyte trafficking blockers is not allowed while patients remain B-cell depleted due to the potential for increased risk of infection.

### 4.5.3 Immunization

No formal vaccination study has been conducted in ocrelizumab-treated patients. Results from studies done with a similar monoclonal antibody, rituximab, which provide additional information on the impact of anti-CD20 antibodies on the response to vaccinations, are presented below.

A small study comparing responses to influenza vaccination among RA patients receiving rituximab or tumor necrosis factor (TNF) inhibitors versus normal controls found significantly lower post vaccination titres and protection rates (the proportion of a group with a titre $\geq 40$) in rituximab-treated patients compared to both control groups [54]. Results from another study are in line with the ones previously stated. This study showed that RA patients treated with rituximab compared with RA patients receiving MTX and healthy adults had a severely hampered humoral immune response to influenza vaccine. This response remained reduced 6-10 months after rituximab treatment. In the rituximab group, patients who had been previously vaccinated achieved higher anti-influenza titers following influenza vaccination [55].

Another study assessed vaccine responses to influenza vaccine (containing 15 µg hemagglutinin/dose of B/Shanghai/361/02 (SHAN), A/New Caledonia 20/99 (NC)

(H1N1) and A/California/7/04 (CAL) (H3N2)) among RA patients treated with disease-modifying anti-rheumatic drugs (DMARDs) with or without rituximab and normal control. After 4 weeks post vaccination geometric mean titers increased for New Caledonia and California antigens in all subjects, but not for the Shanghai antigen in the rituximab group. In addition, in rituximab treated patients, the percentage of responders was low for all three antigens tested, achieving statistical significance for California antigen [56].

In a randomized study with rituximab, patients with RA had comparable responses to tetanus recall antigen (39% versus 42%), reduced responses to pneumococcal polysaccharide vaccine (43% versus 82% to at least two pneumococcal antibody serotypes) and to Keyhole Limpet Haemocyanin (KLH) neoantigen (47% versus 93%), when given 6 months after rituximab as compared with patients only receiving MTX [57]. Because of the mechanism of action, it is expected that similar findings would apply to ocrelizumab and patients treated with ocrelizumab may experience lower response rates to non-live vaccines than the general population.

Physicians are advised to review the immunization status of patients being considered for treatment with ocrelizumab and follow local/national guidance for adult vaccination against infectious disease. Known dates of immunizations will be recorded on specific eCRF pages. **Immunizations should be completed at least 6 weeks prior to first administration of ocrelizumab.**

Patients requiring *de novo* hepatitis B vaccination (which involves three separate doses of vaccine) should also have completed the course at least 6 weeks prior to the first infusion of study drug.

The safety of immunization with live viral vaccines following ocrelizumab or rituximab therapy has not been studied. Immunization with any live or live-attenuated vaccine (i.e., measles, mumps, rubella, oral polio vaccine, Bacille Calmette-Guerin (BCG), typhoid, yellow fever, vaccinia, cold adapted live influenza strain vaccine, or any other vaccines not yet licensed but belonging to this category) is not recommended within 6 weeks of first dosing (see exclusion criteria, Section 4.3), during ocrelizumab treatment and for as long as the patient is B-cell depleted.

## 4.6 Criteria for Premature Withdrawal

Patients have the right to withdraw from the study at any time for any reason.

**Patients must be withdrawn from treatment (regardless of whether they are in the double-blind, double-dummy treatment period or in the OLE Phase) under the following circumstances:**

– Life threatening (Common Terminology Criteria for Adverse Events [CTCAE] Grade 4) infusion-related event that occurred during a previous ocrelizumab infusion;

– Ongoing pregnancy; Please note that the pregnancy should be followed up to determine outcome, including spontaneous or voluntary termination, details of birth, and the presence or absence of any birth defects, congenital abnormalities, or maternal and newborn complications.

- Patients who demonstrate active hepatitis B or C infection, either new onset or reactivation in the case of hepatitis B;
- Patients who demonstrate active TB, either new onset or reactivation;
- Patients with PML;
- Patients with elevation of ALT ≥10 × ULN, jaundice, or other clinical symptoms of liver dysfunction (please refer to Section 6.2.2 for more details);*
- Patients with persisting elevation of ALT >3 × ULN, or other clinical symptoms of liver dysfunction that did not resolve with Rebif®/Rebif® placebo dose modification (please refer to Section 6.2.2 for more details*);**

*Only applicable during the double-blind, double-dummy treatment period.

- Patients who decide to discontinue the treatment;
- The patient's Treating Investigator decides that discontinuation of treatment is in the best clinical interest of the patient.

Patients who withdraw during the double-blind, double-dummy treatment period, OLE Phase Screening Period, or the OLE Phase, for any reason, should complete the Safety Follow-Up Period. If the patient insists on discontinuing *from* the study, he/she should be asked if he/she can still be contacted for further information. The outcome of that discussion should be documented in both the medical records and in the eCRF. If lost to follow-up, the Investigator should contact the patient or a responsible relative by telephone followed by registered mail or through a personal visit to establish as completely as possible the reason for the withdrawal. A complete final evaluation at the time of the patient's withdrawal should be made with an explanation of why the patient is withdrawing from the study.

When applicable, patients should be informed of circumstances under which their participation may be terminated by the Investigator without the patient's consent. The Investigator may withdraw patients from the study in the event of intercurrent illness, adverse events, treatment failure, after a prescribed procedure, lack of compliance with the study and/or study procedures (e.g., dosing instructions, study visits), cure or any reason where it is felt by the Investigator that it is in the best interest of the patient to be terminated from the study. Any administrative or other reasons for withdrawal must be documented and explained to the patient. If the reason for removal of a patient from the study is an adverse event, the principal specific event will be recorded on the eCRF. If possible, the patient should be followed until the adverse event has resolved.

An excessive rate of withdrawals can render the study non-interpretable; therefore, unnecessary withdrawal of patients should be avoided. Should a patient decide to withdraw, all efforts will be made to complete and report the observations prior to withdrawal as thoroughly as possible.

Please note: It is important to distinguish between "withdrawal from treatment" and "withdrawal from study". Patients who withdraw from treatment should be encouraged

to remain in the study for the full duration of the Safety Follow-Up Period (minimum of 48 weeks following the last infusion).

It should be noted that upon withdrawal from the study, any untested routine samples will be destroyed. However, information already obtained from samples up until the time of withdrawal will be used.

#### 4.6.1 Withdrawal of Subjects from the Roche Clinical Repository

Subjects who gave consent to provide RCR specimens have the right to withdraw their specimen from the RCR at any time for any reason. If a patient wishes to withdraw his/her consent to the testing of his/her specimen(s), the Investigator must inform Roche in writing of the patient's wishes using the RCR Subject Withdrawal Form and enter the date of withdrawal in the patient's eCRF. A patient's withdrawal from the main trial does not, by itself, constitute withdrawal of the specimen from the RCR; likewise, a patient's withdrawal from the RCR does not constitute a withdrawal from the main trial.

#### 4.6.2 Patient Agreement for Continuation in the Study (in Case of *Confirmed* Disability Progression)

During the treatment period, in the event of *confirmed* disability progression on EDSS confirmed for 24 weeks, the benefits and risks of study treatment should be reassessed with the patient prior to any further dosing, including a discussion of alternative treatment options available for that patient. The result of this discussion must be included in the patient's file, prior to any further dosing of study medication.

If, after the discussion, the patient decides not to continue with the study treatment, they should be discontinued from any further treatment, complete applicable Withdrawal from Treatment Visit procedures and be entered into the Safety Follow-Up Period.

For definition of *confirmed disability* progression, please refer to Section 5.3.2.2.

### 4.7 Replacement Policy (Ensuring Adequate Numbers of Evaluable Subjects)

#### 4.7.1 For Subjects

Patients prematurely discontinued from the study for any reason will not be replaced.

#### 4.7.2 For Centers

A center may be replaced for the following administrative reasons:

- Excessively slow recruitment.
- Poor protocol adherence.
- Sponsor's discretion (Sponsor refers to F. Hoffmann-La Roche, Ltd and Genentech, Inc.)

## 5. SCHEDULE OF ASSESSMENT AND PROCEDURES

### Table 4: Schedule of Assessments: Screening through the End of Double-Blind, Double-Dummy Treatment Period

| | Screen | Double-blind, Double-Dummy Treatment Period | | | | | | | | | | | | |
|---|---|---|---|---|---|---|---|---|---|---|---|---|---|---|
| **Cycle** | | 1 | | | 2 | | 3 | | 4 | | | Delayed Dosing Visit [22] | Unscheduled Visit [23] | Withdrawal from Treatment Visit |
| **Visit** | 1 | 2 BL | 3 | 4 | 5 | 6 | 7 | 8 | 9 | 10 | 11 | | | |
| **Week** | -2 | - | w2 | w12 | w24 | w36 | w48 | w60 | w72 | w84 | w96 | | | |
| *Study Day (window in days)* | *-14* | *1* | *15 (± 2)* | *85 (±4)* | *169 (± 2)* | *253 (±4)* | *337 (± 2)* | *421 (±4)* | *505 (± 2)* | *589 (±4)* | *673 (± 2)* | | | |
| Informed consent [1] | x | | | | | | | | | x[24] | | | | |
| Medical history | x | | | | | | | | | | | | | |
| Review of eligibility criteria | x | x | | | | | | | | | x[24] | | | |
| CES-D, MFIS, EQ-5D, SF-36 | | x | | | | | x | | | | x | | | x |
| Patient's Assessment of Treatment Benefit | | | | | | | x | | | | x | | | x |
| C-SSRS | | x | | x | x | x | x | x | x | x | x | | x | x |
| Physical examination | x | x | x | | x | | x | | x | | x | x | | x |
| Vital signs [2] | x | x | x | x | x | x | x | x | x | x | x | x | x | x |
| 12 lead ECG (pre- and post-dose) [3] | x | x | | | | | | | x | | | | | x |
| Height | x | | | | | | | | | | | | | |
| Weight | x | | | | | | | | x | | x | | | x |
| Neurological exam and EDSS | x | x | | x | x | x | x | x | x | x | x | | x | x |
| MSFCS, LCVA, SDMT | | x | | x | x | x | x | x | x | x | x | | x | x |
| Karnofsky Performance Status Scale | | x | | | x | | x | | x | | x | | | x |
| MRI[4] | | x | | | x | | x | | | | x | | | x |
| Concomitant Treatment | | x | x | x | x | x | x | x | x | x | x | x | x | x |
| Adverse Events | *Only SAEs* | x | x | x | x | x | x | x | x | x | x | x | x | x |
| Potential relapses recorded | | x | x | x | x | x | x | x | x | x | x | x | x | x |
| Telephone interview (every 4 wks)[5] | | | | → | → | → | → | → | → | → | → | | | |

## Table 4: Schedule of Assessments: Screening through the End of Double-Blind, Double-Dummy Treatment Period (Cont.)

| | Screen | Double-Blind, Double-Dummy Treatment Period | | | | | | | | | | | | |
|---|---|---|---|---|---|---|---|---|---|---|---|---|---|---|
| **Cycle** | | 1 | | | 2 | | 3 | | 4 | | | Delayed Dosing Visit [22] | Unscheduled Visit [23] | Withdrawal from Treatment Visit |
| **Visit** | 1 | 2 BL | 3 | 4 | 5 | 6 | 7 | 8 | 9 | 10 | 11 | | | |
| **Week** | -2 | - | w2 | w12 | w24 | w36 | w48 | w60 | w72 | w84 | w96 | | | |
| *Study Day (window in days)* | *-14* | *1* | *15 (±2)* | *85 (±4)* | *169 (± 2)* | *253 (±4)* | *337 (± 2)* | *421 (±4)* | *505 (± 2)* | *589 (±4)* | *673 (± 2)* | | | |
| Pregnancy test [6] | x | x | x | x | x | x | x | x | x | x | x | x | | x |
| Antibody Titers [7] | | x | | x | x | | x | | x | | x | | | x |
| RCR (non-DNA) [8] | | x | | x | x | | x | | x | | x | | | x |
| RCR (DNA) [9] | | x | | | | | | | | | | | | |
| Protein biomarker sampling [10] | | x | | x | x | | x | | x | | x | | | x |
| HAHA [11] | | x | | | x | | x | | x | | x | | | x |
| Plasma/ urine banking for JCV[12] | | x | | x | x | x | x | x | x | x | x | | | x |
| PK Samples [13] | | x | | | x | | x | | x[13] | x | x | | | x |
| Thyroid function tests [14] | x | | | | x | | x | | x | | x | | | x |
| FSH [15] | x | | | | | | | | | | | | | |
| Hepatitis Screening [16] | x | | | | | | | | | | | | | |
| Hepatitis B virus DNA[16] | x | (x) | | (x) | (x) | (x) | (x) | (x) | (x) | (x) | (x) | | | (x) |
| RPR | x | | | | | | | | | | | | | |
| CD4 count | x | | | x | | x | | x | | x | | | | |
| IgG | | | | x | | x | | x | | x | | | | |
| Total Ig, IgA, IgG, IgM | x | | | | x | | x | | x | | x | | | x |
| FACS [17] | | x | x | x | x | | x | | x | | x | | | x |

## Table 4: Schedule of Assessments: Screening through the End of Double-Blind, Double-Dummy Treatment Period (Cont.)

| | Screen | Double-Blind, Double-Dummy Treatment Period | | | | | | | | | | | | |
|---|---|---|---|---|---|---|---|---|---|---|---|---|---|---|
| **Cycle** | | 1 | | | 2 | | 3 | | 4 | | | Delayed Dosing Visit [22] | Unscheduled Visit [23] | Withdrawal from Treatment Visit |
| **Visit** | 1 | 2 BL | 3 | 4 | 5 | 6 | 7 | 8 | 9 | 10 | 11 | | | |
| **Week** | -2 | - | w2 | w12 | w24 | w36 | w48 | w60 | w72 | w84 | w96 | | | |
| *Study Day (window in days)* | *-14* | *1* | *15 (± 2)* | *85 (±4)* | *169 (± 2)* | *253 (±4)* | *337 (± 2)* | *421 (±4)* | *505 (± 2)* | *589 (±4)* | *673 (± 2)* | | | |
| Routine safety lab [18] | x | x | x | x | x | x | x | x | x | x | x | | | x |
| Type I interferon neutralizing antibody assay [19] | | x | | | x | | x | | x | | x | | | x |
| Pre-treatment with i.v. methylprednisolone [20] | | x | x | | x | | x | | x | | | x | | |
| Administration of i.v. ocrelizumab / ocrelizumab placebo [21] | | x | x | | x | | x | | x | | | x | | |
| Assessment of s.c. Rebif®/ Rebif® placebo compliance | | x | x | x | x | x | x | x | x | x | x | | x | x |
| Administration of s.c. Rebif® / Rebif® placebo 3x/wk | | x | x | x | x | x | x | x | x | x | x[25] | | | |

1. β-hCG=beta human chorionic gonadotropin; BL=baseline; CD4=cluster of differentiation; CES-D=Center for Epidemiologic Studies Depression Scale; C-SSRS=Columbia-Suicide Severity Rating Scale; DNA=deoxyribonucleic acid; ECG=electrocardiogram; eCRF=electronic case report form; EDSS=Expanded Disability Status Scale; EDTA=ethylenediaminetetraacetic acid; EQ-5D=EuroQoL; FACS=fluorescence activated cell sorting; FSH=follicle-stimulating hormone; HAHA=human anti-human antibodies; Ig=immunoglobulin; IgA=immunoglobulin A; IgG=immunoglobulin G; IgM=immunoglobulin M; i.v.=intravenous; JCV=JC virus; LCVA=Low Contrast Visual Acuity; MFIS=Modified Fatigue Impact Scale; MRI=magnetic resonance imaging; MSFCS=Multiple Sclerosis Functional Composite Scale; n=number; OLE=Open-Label Extension; PK=pharmacokinetic; RCR=Roche Clinical Repository; RNA=ribonucleic acid; RPR=rapid plasma reagin; SAE=serious adverse event; s.c.=subcutaneous; SDMT=Symbol Digit Modalities Test; SF-36=Short-Form 36; w=week.

## Table 4: Schedule of Assessments: Screening through the End of Double-Blind, Double-Dummy Treatment Period (Cont.)

1. **Informed Consent** must be obtained in written form from all patients at screening (prior to any study–related procedure) in order to meet eligibility for the study.
2. **Vital signs** (i.e., pulse rate, systolic and diastolic blood pressure, respiration rate and temperature) will be obtained while the patient is in the semi supine position (after 5 minutes). On infusion visits, the vital signs should be taken within 45 minutes prior to the methylprednisolone infusion in all patients. In addition, vital signs should be obtained prior to ocrelizumab/ocrelizumab placebo infusion, then every 15 minutes (± 5 minutes) for the first hour; then every 30 minutes (± 10 minutes) until 1 hour after the end of the infusion. On non-infusion days, the vital signs may be taken at any time during the visit.
3. **ECG (pre- and post-dose):** on infusion visits ECG should be taken within 45 minutes prior to the methylprednisolone infusion in all patients, and within 60 minutes after completion of the ocrelizumab/ocrelizumab placebo infusion. On non-infusion days, the ECG may be taken at any time during the visit.
4. **MRI**: brain MRI scans *should occur within a window or* ± *4 weeks of the scheduled visit. Also, brain MRI scans* will be obtained in patients withdrawn from the treatment period (at a withdrawal visit) if not performed during last 4 weeks.
5. **A structured telephone interview** will be conducted by site personnel every 4 weeks (± 3 days) from Week 8 through the study to identify any new or worsening neurological symptoms that warrant an unscheduled visit and collect data on possible events of infections.
6. **Serum β-hCG** must be performed at screening in women of childbearing potential. Subsequently, urine β-hCG [sensitivity of at least 25 mIU/mL] will be performed. On infusion visits, the urine pregnancy test should be performed prior to methylprednisolone infusion in all women of child-bearing potential. If positive, the patient will not receive the scheduled dose and confirmation, a serum pregnancy test, will be performed.
7. **Antibody Titers**: measurement of antibody titers against common antigens (mumps, rubella, varicella and *Streptococcus pneumoniae*) will be performed.
8. **RCR - Roche Clinical Repository non-DNA (RNA – and protein):** for RNA 2x 2.5 mL whole blood samples to be collected from consenting patients only for expression profiling analysis. For protein: 6 mL blood samples in EDTA tube for plasma samples will be collected from consenting patients only for analysis of protein biomarkers. On infusion visits, ALL samples should be collected 5-30 minutes prior to methylprednisolone infusion.
9. **RCR - Roche Clinical Repository (DNA):** 6 mL whole blood sample to be collected from only from patients consenting to RCR for pharmacogenetic and genetic analysis. If not done at Baseline (Visit 2), sample may be collected at next visit.
10. **Protein biomarker sampling:** one serum sample (6 mL) will be collected from all patients for analysis of protein biomarkers. On infusion visits, samples should be collected 5-30 minutes prior to methylprednisolone infusion.
11. **HAHA**: On infusion visits, serum samples are collected 5-30 minutes prior to the methylprednisolone infusion.
12. **Plasma and urine samples for JCV will be collected** at specified time points and analyzed in batches, if decided by the Sponsor.

## Table 4: Schedule of Assessments: Screening through the End of Double-Blind, Double-Dummy Treatment Period (Cont.)

13. **PK samples:** on the infusion day at week 72, two serum samples should be collected, one 5-30 minutes prior to the methylprednisolone infusion and the second one 30 minutes (±10 minutes) following the completion of the ocrelizumab/ocrelizumab placebo infusion. For all other infusion visits, a blood sample should be collected 5 – 30 minutes before the methylprednisolone infusion. At other times (non-infusion visits) samples may be collected at any time during the visit.
14. **Sensitive thyroid-stimulating hormone (sTSH)** will be tested at screening and during the double-blind, double-dummy treatment period. Thyroid autoantibodies will be assayed only at screening.
15. **FSH**: only applicable to women to confirm the postmenopausal status.
16. **Hepatitis** screening & monitoring: all patients must have negative hepatitis B surface antigen (HbsAg) result and negative hepatitis C antibody (HepCAb) screening tests prior to enrollment. If total hepatitis B core antibody (HbcAb) is positive at screening, HB virus DNA measured bypolymerase chain reaction (PCR) must be negative to be eligible. For those patients enrolled with negative HBsAg and positive total HBcAb, HB virus DNA (PCR) must be repeated every 12 weeks during the treatment period.
17. **FACS**: including CD19 and other circulating B-cell subsets, T-cells, natural killer cells, and other leukocytes. On infusion visits, blood samples should be collected prior to the infusion of methylprednisolone.
18. **Routine safety lab:** hematology, chemistry and urinalysis: on infusion visits, all urine and blood samples should be collected prior to the infusion of methylprednisolone. At other times, samples may be collected at any time during the visit.
19. **Type I interferon neutralizing antibody assay:** At baseline (visit 2), sample should be collected before first Rebif®/Rebif® placebo injection. At subsequent visits, samples should be collected at least 36 hours following last injection of Rebif®/Rebif® placebo.
20. All patients receive **prophylactic treatment** with 100 mg of methylprednisolone i.v. prior to infusion of ocrelizumab /ocrelizumab placebo. In the rare case when the use of methylprednisolone is contraindicated for the patient, use of an equivalent dose of an alternative steroid should be used as premedicationprior to the infusion. It is also recommended that patients receive an analgesic/antipyretic such as acetaminophen/paracetamol (1 g) and an i.v. or oral antihistaminic such as diphenhydramine 50 mg 30-60 minutes prior to ocrelizumab/ ocrelizumab placebo.
21. **Administration (infusion) of i.v. ocrelizumab/ocrelizumab placebo**: the Treating Investigator must review the clinical and laboratory re-treatment criteria prior to subsequent infusion of ocrelizumab/ocrelizumab placebo.
22. **A delayed dosing visit** will be performed and recorded in the Delayed Dosing Visit eCRF form when dosing cannot be administered at the scheduled dosing visit. Other tests or assessments may be done as appropriate.

## Table 4: Schedule of Assessments: Screening through the End of Double-Blind, Double-Dummy Treatment Period (Cont.)

23. **Unscheduled Visit:** assessments performed at unscheduled (non-dosing) visits will depend on the clinical needs of the patient. All patients with new neurological symptoms suggestive of relapse should have EDSS performed by examining Investigator, *whenever possible within 7 days of the onset of the relapse*. Other tests/assessments may be done as appropriate. Please note: in case of alanine aminotransferase (ALT) elevations dose modification should be necessary, additional visits may be required for dispensing of study medication.
24. At the Week 84 Visit, a discussion with the patient regarding participation in the Open-Label Extension Phase should occur if the Treating Investigator is of the opinion that the patient may benefit from treatment with ocrelizumab. An informed consent for the OLE Phase should be provided to the patient. It is recommended that the Informed Consent Form of the Open-Label Extension Phase be signed at the Week 96 Visit.
25. If the patient enters OLE Phase Screening Period, Rebif® / Rebif® placebo should be provided to the patient at the Week 96 Visit (please see Section 5.10.1).

Please note: based on local Ethics Committees or National Competent Authority requirements, additional diagnostic testing may be required for selected patients or selected centers to exclude tuberculosis, Lyme disease, HTLV-1 associated myelopathy (HAM), acquired immune deficiency syndrome (AIDS), hereditary disorders, connective tissue disorders, or sarcoidosis. Other specific diagnostic tests may be requested when deemed necessary by the Investigator.

## Table 5: Schedule of Assessments: Safety Follow-Up (including prolonged B-cell monitoring if required)

| | Safety Follow-Up | Prolonged B-cell Monitoring[1] | End of observation or withdrawal from Safety Follow up |
|---|---|---|---|
| **Assessments** | *Visits every 12 weeks (±7 days)* [2] | *Visits every 24 weeks (±7 days)* | |
| Urine pregnancy test | x | x | x |
| Routine Safety Labs[3] | x | x | x |
| FACS [4] | x | x | x |
| Total Ig, IgA, IgG, IgM | x[10] | x | x |
| HAHA [5] | x[10] | x | x |
| Plasma/urine banking for JCV | x | x | x |
| Antibody titers | x[10] | x | x |
| Hepatitis B viral DNA[6] | (x) | (x) | (x) |
| Protein biomarker sampling [8] | x[10] | x | x |
| Vital Signs | x | x | x |
| EDSS | x | | x |
| Neurological examination | x | x | x |
| Physical examination | x[10] | x | x |
| Potential relapses recorded | x | x | x |
| Adverse events | x | x | x |
| Concomitant Medication | x | x | x |
| Telephone interview [9] | x | x | |

CD=cluster of differentiation; DNA=deoxyribonucleic acid; EDSS=Expanded Disability Status Scale; EDTA=ethylenediaminetetraacetic acid; FACS=fluorescence activated cell sorting; HAHA=human anti-human antibodies; HbcAb=hepatitis C antibody; HbsAg=hepatitis B surface antigen; HBV=hepatitis B virus; Ig=immunoglobulin; IgA=immunoglobulin A; IgG=immunoglobulin G; IgM=immunoglobulin M; i.v.=intravenous; JCV=JC virus; RNA=ribonucleic acid.

**Table 5: Schedule of Assessments: Safety Follow-Up (including prolonged B-cell monitoring if required) (Cont.)**

5. **Prolonged B-cell monitoring**: patients whose B-cells have not been repleted after 48 weeks of Safety Follow-Up Period will continue with visits every 24 weeks (± 7 days) until B-cell repletion.
6. **Safety Follow-Up** will be carried out for at least 48 weeks starting from the date of last infusion of ocrelizumab. Visits will be performed at 12-week intervals starting from the date of the patient's Withdrawal From Treatment Visit. *Safety Follow-Up applies to study patients who have completed the blinded treatment period (or open label treatment period, if applicable) and to patients who withdraw early from treatment. If B-cells have returned to normal levels at this visit, then the 48 week Safety Follow-up visit will become the End of Observation visit and the patient will have completed the study. An End of observation visit will be performed only in case of occurrence of new adverse event and/or if the investigator considers the prior safety assessment (laboratory, other) as abnormal and clinically significant.*
7. **Routine safety lab**: hematology, chemistry and urinalysis.
8. **FACS** including CD19 and other circulating B-cell subsets, T cells, natural killer cells and other leukocytes.
9. **HAHA**: two serum samples are required.
10. **Hepatitis monitoring:** hepatitis to be monitored only in patients with screening results of HbsAg negative, HBcAb positive and HBV DNA negative, inclusive.
11. **Protein biomarker sampling**: 6 mL blood sample in a plain tube without EDTA for serum isolation will be collected from all patients for analysis of protein biomarkers.
12. **A structured telephone interview** will be performed by site personnel every 4 weeks (± 3 days) between visits until 48 weeks after the last infusion to identify any new or worsening neurological symptoms that warrant an unscheduled visit and collect data on possible events of infections. If prolonged B-cell monitoring is required beyond 48 weeks after the last infusion, telephone interviews will be done every 12 weeks (± 7 days) between visits.
13. Needs to be assessed only every 24 weeks.

Please note: patients in Safety Follow-Up who receive other B-cell targeted therapies will only be followed for 48 weeks from the date of the last infusion of the study drug regardless of their B-cell count.

## Table 6: Schedule of Assessments: Open Label Extension Phase

| | OLE Screen | Open-Label Extension Phase[1] | | | | | | | | | | | Delayed dosing Visit[20] | Unscheduled Visit[21] | Withdrawal from Treatment Visit |
|---|---|---|---|---|---|---|---|---|---|---|---|---|---|---|---|
| **Cycle[1]** | | **5** | | | **6[1]** | | **7[1]** | | **8[1]** | | **N[1,3]** | | | | |
| **Visit** | | **12** | **13** | **14** | **15** | **16** | **17** | **18** | **19** | **20** | **…** | **…** | | | |
| **Week in OLE Phase (window in days)** | | *0[2]* | *2 (± 2)* | *12 (± 7)* | *22 (± 7)* | *24 (±5)* | *46 (± 7)* | *48 (± 5)* | *70 (± 7)* | *72 (± 5)* | *n-2 wk (± 7)* | *n (± 7)* | | | |
| Informed consent[4] | x | | | | | | | | | | | | | | |
| Review of eligibility criteria | x | x | | | | | | | | | | | | | |
| Review of retreatment criteria | | x | x | | | x | | x | | x | | x | x | | |
| Administration of s.c. Rebif® / Rebif® placebo 3x/wk [5] | x | | | | | | | | | | | | | | |
| Assessment of s.c. Rebif®/ Rebif® placebo compliance | | x | | | | | | | | | | | | | |
| Pre-treatment with i.v. methylprednisolone[6] | | x | x | | | x | | x | | x | | x | x | | |
| Administration of i.v. ocrelizumab[7] | | x | x | | | x | | x | | x | | x | x | | |
| Physical examination | | x | x | | | x | | x | | x | | x | x | | x |
| Vital signs, weight[8] | | x | x | x | | x | | x | | x | | x | x | x | x |
| 12-lead ECG (pre- and post-dose, once early)[9] | | x | x | | | | | | | x | | (x) | | | x |
| Neurological exam and EDSS | | x | | x | | x | | x | | x | | x | | x | x |
| Routine safety labs[10] | | x | | x | x | | x | | x | | x | | | x | x |
| Adverse events | | x | x | x | x | x | x | x | x | x | x | x | x | x | x |
| Concomitant treatments | | x | x | x | x | x | x | x | x | x | x | x | x | x | x |
| Potential relapses recorded | | x | x | x | x | x | x | x | x | x | x | x | x | x | x |
| Pregnancy test[11] | | x | x | x | | x | | x | | x | | x | x | | x |
| Antibody titers[12] | | x | | x | x | | x | | x | | x | | | | x |
| Total Ig, IgA, IgG, IgM | | x | | | x | | x | | x | | x | | | | |
| CD4 count | | x | x | | x | | x | | x | | x | | | | |

## Table 6: Schedule of Assessments: Open Label Extension Phase (Cont.)

| Cycle[1] / Visit / Week in OLE Phase (window in days) | OLE Screen | Open-Label Extension Phase[1] | | | | | | | | | | | Delayed dosing Visit[20] | Unscheduled Visit[21] | Withdrawal from Treatment Visit |
|---|---|---|---|---|---|---|---|---|---|---|---|---|---|---|---|
| Cycle | | 5 | | | 6[1] | | 7[1] | | 8[1] | | N[1,3] | | | | |
| Visit | | 12 | 13 | 14 | 15 | 16 | 17 | 18 | 19 | 20 | … | … | | | |
| Week (window in days) | | *0*[2] | *2 (± 2)* | *12 (± 7)* | *22 (± 7)* | *24 (±5)* | *46 (± 7)* | *48 (± 5)* | *70 (± 7)* | *72 (± 5)* | *n-2 wk (± 7)* | *n (± 7)* | | | |
| HAHA[22] | | x | | | x | | x | | x | | x | | | | x |
| *Ocrelizumab concentration sample*[22] | | *x* | | | *x* | | *x* | | *x* | | *x* | | | | *x* |
| Telephone interview (every 4 weeks)[13] | | | | - - - - - - | - - - - - - | - - - - - - | - - - - - - | - - - - - - | - - - - - - | - - - - - - | - - - - - -▶ | | | | |
| EQ-5D (once yearly) | | x | | | | | x | | | | (x) | | | | |
| Protein biomarker sampling[15] | | x | | x | x | | x | | x | | x | | | | |
| FACS[16] | | | x | x | x | | x | | x | | x | | | | |
| Hepatitis B virus DNA[17] | | | | (x) | (x) | | (x) | | (x) | | (x) | | | | |
| MRI (once yearly)[18] | | | | | | | x | | | | (x) | | | | |
| Patient's Assessment of Treatment Benefit (once yearly) | | | | | | | x | | | | (x) | | | | |
| Plasma/urine banking for JCV[19] | | x | | x | x | | x | | x | | x | | | | x |

CD4 = cluster of differentiation; DNA = deoxyribonucleic acid; ECG = electrocardiogram; eCRF = electronic case report form; EDSS = Expanded Disability Status Scale; EQ-5D = EuroQoL; FACS = fluorescence activated cell sorting; HAHA = human anti-human antibodies; Ig = immunoglobulin; IgA = immunoglobulin A; IgG = immunoglobulin G; IgM = immunoglobulin M; i.v. = intravenous; JCV = JC virus; MRI = magnetic resonance imaging; n = number; OLE = Open-Label Extension; RCR = Roche Clinical Repository; s.c. = subcutaneous.

1. **The OLE Phase** can terminate at any moment or cycle (please refer to End of Study Section 3.1.4). In case the study is ended, a Withdrawal from Treatment Visit should occur.
2. **The OLE Phase** starts at the first infusion of Cycle 5. The OLE Phase Screening Period will start after all assessments at the Week 96 Visit have been performed, and it should last approximately 4 weeks. It is possible that the OLE Phase Screening Period could be longer than 4 weeks. If a prolongation of the OLE Phase Screening Period is needed, it should be discussed with the Sponsor on a case-by-case basis.
3. The assessments requested for N represent the typical schedule of assessments during a cycle.
4. The **informed consent** should have been provided to the patient at the Week 84 Visit and signed by the patient at the Week 96 Visit.

## Table 6: Schedule of Assessments: Open Label Extension Phase (Cont.)

5. During the OLE Phase Screening Period, s.c. administration Rebif® / Rebif® placebo 3 times per week should occur until one day prior to the first infusion of ocrelizumab of Cycle 5 (beginning of OLE Phase). If during this period, the patient decides not to participate in the OLE Phase, then administration of Rebif® / Rebif® placebo 3 times per week should stop immediately, and the patient will be entered in the Safety Follow-Up Period.
6. All patients receive prophylactic treatment with 100 mg of methylprednisolone i.v. prior to infusion of ocrelizumab. In the rare case when the use of methylprednisolone is contraindicated for the patient, use of an equivalent dose of an alternative steroid should be used as premedication prior to the infusion. It is also recommended that patients receive an analgesic/antipyretic such as acetaminophen/paracetamol (1 g) and an i.v. or oral antihistaminic such as diphenhydramine 50 mg 30-60 minutes prior to ocrelizumab.
7. **Administration (infusion) of i.v. ocrelizumab**: the Investigator must review the clinical and laboratory re-treatment criteria prior to subsequent infusion of ocrelizumab.
8. **Vital signs** (i.e., pulse rate, systolic and diastolic blood pressure, respiration rate, and temperature) will be obtained while the patient is in the semi-supine position (after 5 minutes). Vital signs should be collected within 45 minutes prior to the methylprednisolone infusion in all patients. In addition, vital signs should be obtained prior to ocrelizumab infusion, then every 15 minutes (± 5 minutes) for the first hour; then every 30 minutes (± 10 minutes) until 1 hour after the end of the infusion.
9. **ECGs** (pre- and post-dose): ECG should be performed within 45 minutes prior to the methylprednisolone infusion in all patients, and within 60 minutes after completion of the ocrelizumab infusion.
10. **Routine safety lab**: hematology, chemistry, and urinalysis. On infusion visits at Cycle 5, all urine and blood samples should be collected prior to the infusion of methylprednisolone. At other times, samples may be collected at any time during the visit.
11. **Urine β-hCG** [sensitivity of at least 25 mIU/mL] will be performed. On infusion visits, the urine pregnancy test should be performed prior to methylprednisolone infusion in all women of childbearing potential. If positive, the patient will not receive the scheduled dose and for confirmation a serum pregnancy test will be performed.
12. **Antibody Titers**: measurement of antibody titers against common antigens (mumps, rubella, varicella, and Streptococcus pneumoniae) will be performed.
13. A structured **telephone interview** will be conducted by site personnel every 4 weeks (± 3 days) from Cycle 5 *(Week 8 of OLE Phase)* through the study to identify any new or worsening neurological symptoms that warrant an unscheduled visit and collect data on possible events of infections.
14. **Protein biomarker sampling**: one serum sample (6 mL) will be collected from all patients for analysis of protein biomarkers. On infusion visits, samples should be collected 5-30 minutes prior to methylprednisolone infusion.
15. **FACS**: including CD19 and other circulating B-cell subsets, T-cells, natural killer cells, and other leukocytes.
16. **Hepatitis monitoring**: For those patients enrolled with negative HBsAg and positive total HBcAb, HB virus DNA (PCR) must be repeated every 24 weeks during the treatment period.
17. **MRI**: brain MRI scans *should occur within a window of ± 4 weeks of the scheduled visit. Also, brain MRI scans* will be obtained in patients withdrawn from the OLE Phase (at a withdrawal visit) if not performed during last 4 weeks.
18. **Plasma and urine samples for JCV** will be collected at specified time points
19. A **delayed dosing visit** will be performed and recorded in the Delayed Dosing Visit eCRF form when dosing cannot be administered at the scheduled dosing visit. Other tests or assessments may be done as appropriate.

## Table 6: Schedule of Assessments: Open Label Extension Phase (Cont.)

20. **Unscheduled Visit**: assessments performed at unscheduled (non-dosing) visits will depend on the clinical needs of the patient. All patients with new neurological symptoms suggestive of relapse should have EDSS performed*, whenever possible within 7 days of the onset of the relapse*. Other tests/assessments may be done as appropriate.
21. ***HAHA and Ocrelizumab drug concentration samples****: At the infusion Visit 12 (Cycle 5), a blood sample should be collected before the methylprednisolone infusion. At non-infusion visits, samples may be collected at any time during the visit.*

## 5.1 Screening Examination and Eligibility Screening Form

All patients must sign and date the most current Institutional Review Board/Institutional Ethics Committee's (IRB/IEC) approved written informed consent before any study specific assessments or procedures are performed.

Consenting patients will enter the 2-week screening period to be evaluated for eligibility. Please refer to "Schedule of Assessments: Screening through the End of Double-Blind, Double-Dummy Treatment Period" - Table 4 for details. Patient must fulfill all entry criteria for participation in the study.

The screening period can be extended to a total period of 8 weeks in cases when a laboratory blood test or MRI scan need to be repeated for confirmation during the screening interval, or for other relevant clinical, administrative, or operational reasons.

Please note that based on local Ethics Committees or National Competent Authority requirements, additional diagnostic testing may be required for selected patients or selected centers to exclude tuberculosis, Lyme disease, HAM, AIDS, hereditary disorders, connective tissue disorders, or sarcoidosis.

An Eligibility Screening Form [ESF] documenting the Investigator's assessment of each screened patient with regard to the protocol's inclusion and exclusion criteria is to be completed by the Investigator.

Each patient screened must be registered in the IxRS by the Investigator or the Investigator's research staff at screening. A screen failure record must be maintained by the Investigator, and reasons must be captured in the IxRS.

It should be stated in the medical record that the patient is participating in this clinical study.

## 5.2 Procedures for Enrollment of Eligible Subjects

Once a patient has fulfilled all eligibility criteria, he or she will be randomized via IxRS to one of two treatment groups: ocrelizumab 600 mg (given as 300 mg × 2 14 days apart for the first 24 weeks and 600 mg × 1 every 24 weeks thereafter) or Rebif®.

Patient eligibility information will be provided to the IxRS by the Investigator or the Investigator's research staff at randomization. The patient will be randomized and assigned a unique treatment box number (medication number) and randomization number. As confirmation, the site will be provided with a verification of each patient's randomization.

The patient randomization numbers will be generated by Roche or its designee and incorporated into the double-blind labeling.

The patient randomization numbers are to be allocated sequentially in the order in which the patients are enrolled according to the specification document agreed with the external randomization company/center.

Treatment with the first study drug infusion should occur within 24 hours of randomization. In exceptional cases where all baseline assessments cannot be completed

within 24 hours, the first study drug infusion can be administered within 48 hours of randomization provided that the Investigator assures that all inclusion and exclusion criteria are still met on the day of dosing. In particular, there should be no evidence of an ongoing infection at the time of dosing.

No patient may begin treatment prior to randomization and assignment of a medication number.

## 5.3 Clinical Assessments and Procedures During the Double-Blind, Double-Dummy Treatment Period

This is an assessor blinded study. During the double-blind, double-dummy treatment period, each site will have two Investigators: a principal or Treating Investigator and an Examining Investigator or rater.

- **The Treating Investigator** is the physician responsible for the patient care and should be a neurologist experienced in the care of MS patients. The Treating Investigator will have access to safety and blinded efficacy data and will make treatment decisions based on the patient's clinical response and laboratory findings.
- **The Examining Investigator** should be a neurologist or other health care practitioner and must be trained and certified in administering the Neurostatus Functional System Scores (FSS) and Expanded Disability Status Scale (EDSS) examination prior to study start.

The Examining Investigator will perform the neurological examination, document the FSS scores, and assess EDSS scores and the Karnofsky Performance Status Scale. The Examining Investigator or a qualified designee will also be responsible for performing and documenting results from the following: MSFCS, low-contrast visual acuity testing, and the Symbol Digit Modalities Test. They will only have access to data from the assessments listed above. Every effort will be made to ensure that there is no change in the EDSS rater throughout the course of the study for any individual patient. Whenever possible, the same person should perform the examination for the full study duration.

All efforts should be made to keep the Examining Investigator blinded to the treatment assignment during the double-blind, double-dummy treatment period. Patients will be instructed not to discuss any symptoms related to the study treatment with the Examining Investigator; the Examining Investigator should remind the patient at the start of the examination. In view of the extended duration of this study, each site will identify a primary and back-up for Treating and Examining Investigator. **The Treating Investigator and the Examining Investigator will not be allowed to switch roles**.

### 5.3.1 Overview of Clinical Visits During the Double-Blind, Double-Dummy Treatment Period

After the screening visit, patients fulfilling the entry criteria will be scheduled for the baseline assessments. Randomization will occur only after the patient meets all inclusion and exclusion criteria on Day 1. Visits will take place as described in the Schedule of Assessments (Table 4).

Visits should be scheduled with reference to the date of the baseline visit (Day 1). A minimum interval of 20 weeks kept between the ocrelizumab/ocrelizumab placebo second infusion of Cycle 1 (i.e., infusion Week 2) and the next infusion on Cycle 2 (Week 24). A minimum of 22 weeks must occur between ocrelizumab/ ocrelizumab placebo single infusions administered during Weeks 24, 48, and 72.

At infusion visits, patients treated with ocrelizumab /ocelizumab placebo should remain in observation for at least 1 hour after the completion of the infusion.

If for logistical reasons the ocrelizumab/ocrelizumab placebo infusion at Week 24, 48, or 72 cannot be administered on the same study visit day, the infusion should be given within the next 24 hours provided that the patient still meets re-treatment criteria (refer to Section 6.1.4).

Patients who cannot receive their infusion at the scheduled visit or within 24 hours of the visit should be re-scheduled for a delayed dosing visit– see Section 5.3.1.1. Additional unscheduled visits for the assessment of potential relapses, new neurological symptoms, safety events or for dispensing Rebif®/Rebif® placebo if down titration is needed may occur at any time.

#### 5.3.1.1 Delayed Dosing Visit

Delayed dosing visits may be scheduled only if the infusion cannot be administered at the time points defined in Schedule of Assessments -Table 4. Thus, a patient who had all assessments of a dosing visit performed, but could not receive his/her infusion, should be re-scheduled for the infusion. Delayed dosing visit should not be scheduled for the first infusion of the first treatment cycle (Day 1), as treatment with the first study drug infusion should occur within 24 hours of randomization (in exceptional cases within 48 hours of randomization provided that the Investigator assures that all inclusion and exclusion criteria are still met on the day of dosing), see Section 5.2.

In unforeseen situations, if the infusion of the first treatment cycle (Day 1) is delayed, then the visit for the second infusion should be scheduled 14 days after the delayed first infusion (± 2 days). In the event any subsequent infusion needs to be delayed, a minimum interval of 20 weeks between the second infusion of cycle 1 (Week 2) and the next infusion on cycle 2 (Week 24) is required; a minimum of 22 weeks must occur between infusions administered during Weeks 24, 48, and 72.

At the delayed dosing visit, additional tests or assessments, such as routine safety laboratory tests, may be performed when the Investigator judges that these are warranted.

#### 5.3.1.2 Unscheduled Visits

Patients developing new or worsening neurological symptoms should be seen at the investigational site as soon as possible regardless of the treatment group to which they were randomized, regardless of the dates of their pre-planned, scheduled study visits, and regardless of the study period. Assessments performed at unscheduled (non-dosing) visits will depend on the clinical needs of the patient.

Patients with new neurological symptoms suggestive of relapse should have an EDSS performed by Examining Investigator, *whenever possible within 7 days of the onset of the*

*relapse*. Other tests/assessments may be done as appropriate. Please note: if, during the double-blind, double-dummy treatment period, Rebif®/Rebif® placebo dose modification *is* necessary in case of ALT elevations, then unscheduled visits may be required for dispensing of study medication (see Section 6.2).

Please refer also to Section 7.3.4.1 for guidance on the diagnosis of progressive multifocal leukoencephalopathy (PML).

#### 5.3.1.3 Withdrawal Visits

At the moment a patient meets one or more of the withdrawal criteria (Section 4.6), this patient is regarded withdrawn from treatment. Patient who withdraw from ocrelizumab treatment will need to complete all assessments as shown in Schedule of Assessments and will enter the Safety Follow-up.

*At the termination of the study (see also Section 3.1.4 for definition of end of the study), the patients will receive either commercial ocrelizumab (if available in that country) or will enter the Safety Follow-up. All patients will undergo a complete final evaluation according to the 'Withdrawal from Treatment Visit' in the Schedule of Assessments, Table 6. Thereafter, all patients will be treated according to individual center practice.*

For patients who have withdrawn from the double-blind, double-dummy treatment period, OLE Phase, or the OLE Phase Screening Period or who are not eligible for treatment with ocrelizumab, it is at the discretion of the Investigator to decide on further treatment of the underlying disease. However, immunosuppressants, lymphocyte-depleting therapies, and lymphocyte trafficking blockers may increase the risk of infections while patients remain B-cell depleted and therefore are not allowed during the Safety Follow-Up Period.

Please note: at the Withdrawal from Double-Blind, Double-Dummy Treatment Period Visit *or OLE Phase*, an MRI scan will be required only if not performed in the prior 4 weeks.

### 5.3.2 Assessment of Efficacy

#### 5.3.2.1 Assessment of Relapse

All new or worsening neurological events consistent with MS representing a clinical relapse are to be reported on the dedicated page of eCRF. Patients with clinical relapses should be referred to the Examining Investigator who will assess the FSS/EDSS independently to allow confirmation as to whether or not the clinical relapse(s) meet the criteria for protocol-defined relapse(s).

**Protocol-defined relapse** is the occurrence of new or worsening neurological symptoms attributable to MS. Symptoms must persist for >24 hours and should not be attributable to confounding clinical factors (e.g., fever, infection, injury, adverse reactions to medications) and immediately preceded by a stable or improving neurological state for at least 30 days. The new or worsening neurological symptoms must be accompanied by objective neurological worsening consistent with an increase of at least half a step on the EDSS scale, or 2 points on one of the appropriate FSS, or 1 point on two or more of the appropriate FSS. The change must affect the selected FSS (i.e., pyramidal, ambulation,

cerebellar, brainstem, sensory, or visual). Episodic spasms, sexual dysfunction, fatigue, mood change, or bladder or bowel urgency or incontinence will not suffice to establish a relapse. NB: Sexual dysfunction and Fatigue will not be scored. Please note: adjudication of protocol-defined relapses will be performed by the Sponsor based on pre-specified criteria, applied to data collected by Investigator, in a blinded fashion.

**All patients with new neurological symptoms suggestive of a relapse should be referred to the Examining Investigator for EDSS assessment*, whenever possible within 7 days of the onset of the relapse*. Any patient, complaining of a neurological symptom, defined at a visit or over the phone, should be referred to the Examining Investigator unless the Treating Investigator determines that the symptom is due to mitigating circumstances (such as an intensification of neurological symptoms from a transient systemic infection).**

Please note: clinical relapses (i.e., regardless of whether they meet criteria for a protocol-defined relapse) will be recorded on a pre-specified eCRF "MS relapse" eform. MS relapses should not be reported on Adverse Event eform of eCRF.

##### 5.3.2.2 Assessment of Disability

**Disability progression** has been defined as an increase of ≥1.0 point from the baseline EDSS score that is not attributable to another etiology (e.g., fever, concurrent illness, or concomitant medication) when the baseline score is 5.5 or less, and ≥0.5 when the baseline score is above 5.5. Disability progression is considered *confirmed* when the increase in the EDSS is confirmed at a regularly scheduled visit at least 12 weeks or 24 weeks, after the initial documentation of neurological worsening.

*Confirmed* disability progression, confirmed for 12 weeks, after the initial documentation of neurological worsening, will be analyzed as key secondary endpoint. *Additionally, confirmed disability progression for 24 weeks will be assessed.* The initial event of neurological worsening must occur during the 96-week, double-blind, double-dummy, treatment period.

##### 5.3.2.3 Kurtzke Expanded Disability Status Scale

The EDSS is based on a standard neurological examination, incorporating the following functional systems (pyramidal, cerebellar, brainstem, sensory, bowel and bladder, visual, and cerebral [or mental]) and ambulation rated and scored as functional system scores (FSS). Each FSS is an ordinal clinical rating scale ranging from 0 to 5 or 6. These ratings are then used in conjunction with observations and information concerning ambulation and use of assistive devices to determine the EDSS score. The EDSS is a disability scale that ranges in 0.5-point steps from 0 (normal) to 10 (death) [58].

The EDSS will be assessed by the Examining Investigator. **All patients with new neurological symptoms suggestive of relapse should have EDSS performed during an unscheduled visit.**

##### 5.3.2.4 The Multiple Sclerosis Functional Composite Scale

The Multiple Sclerosis Functional Composite Scale (MSFCS) consists of three subscales, including the 9-Hole Peg Test, Paced Auditory Serial Addition Test (PASAT), and Timed

25-Foot Walk (25-TW), which provide a global quantitative estimate of MS *disability* progression [59].

The MFSCS will be performed by the Examining Investigator or a qualified designee who must remain blinded to the treatment assignment.

#### *5.3.2.5 Low-Contrast Visual Acuity Testing*

Low-contrast letter acuity charts (Sloan charts) have gained validity in the assessment of visual dysfunction in patients with MS not readily apparent on commonly used high-contrast acuity tests. Reductions in low-contrast letter acuity are associated with MS and correlate with increasing disability, MRI abnormalities, and reduced retinal nerve fiber layer (RNFL) thickness as measured by OCT.

LCVA testing will be performed using low contrast letter acuity charts (low contrast Sloan letter charts) by the Examining Investigator or a qualified designee at the timepoints indicated in the Schedule of Assessments -Table 4.

#### *5.3.2.6 The Symbol Digit Modalities Test*

The SDMT has demonstrated sensitivity in detecting not only the presence of cognitive impairment, but also changes in cognitive functioning over time and in response to treatment. The SDMT is brief, easy to administer, and involves a simple substitution task that normal children and adults can easily perform. Using a reference key, the examinee has 90 seconds to pair specific numbers with given geometric figures. Responses can be written or oral, and for either response mode, administration time is just 5 minutes.

SDMT will be administered by the Examining Investigator or a qualified designee at the timepoints indicated in the Schedule of Assessments -Table 4.

### 5.3.3 Brain Magnetic Resonance Imaging

MRI is a useful tool for monitoring CNS lesions in MS. Different MRI derived parameters have been related to clinical activity and T1 weighted Gd-enhancing lesions or new and/or enlarging hyperintense T2 lesions have been related to relapses. It is hypothesized that changes in brain volume may reflect brain atrophy as a result of MS-related tissue loss and may thereby correlate with long-term clinical outcome in these patients.

Brain MRI scans will be obtained in all patients as detailed in the Schedule of Assessments -Table 4. In addition, brain MRI scans will be obtained in patients withdrawn from the double-blind, double-dummy treatment period (at the withdrawal visit) if not performed during the previous 4 weeks.

Scans will be performed by trained and certified MRI technicians. The following time windows apply:

- “Baseline” MRI should be performed after screening visit, but at least 10 days prior to the baseline visit.
- MRI at visits scheduled at Weeks: 24, 48, 96 or at withdrawal visit (if applicable) should be performed within a window of ± 4 weeks of the scheduled visit.

- In the OLE Phase, MRI should be performed yearly (e.g., Cycles 7, 9, etc.) *within a window of ± 4 weeks of the scheduled visit*.

If patients receive corticosteroids for an MS relapse, every effort should be made to obtain the scan prior to the first steroid dose if the pre-steroid scan is within 1 week of the scheduled visit. In patients receiving corticosteroids for an MS relapse, there should be an interval of 3 weeks between the last dose of corticosteroids and the scan.

The MRI will include the acquisition of scans at each time point with and/or without intravenously administered Gd contrast enhancement.

MRI scans will be read by a centralized reading center for efficacy endpoints. The centralized reading center is blinded to the treatment assignment and the reading is performed in the absence of clinical information. Further details on scanning acquisition sequences, methods, handling and transmission of the scans, certification of site MRI radiologist/technicians, and the procedures for the blinded analysis of the scans at the central reading center are described in a separate MRI Acquisition Procedures Manual.

All MRI scans will also be reviewed locally by a radiologist for safety. During the double-blind, double-dummy treatment period, the MRI scan report containing only non-MS pathology will be provided to the Treating Investigator (see Section 5.3 for definition). At the investigational site, only the local radiologist/technician assigned to this study may have access to the MRI scans post-randomization; the Treating Investigator should not review the MRI scans obtained after randomization unless a safety concern arises. In the event that the Treating Investigator does become aware of these MRI results, this should be documented in the eCRF, indicating the reason.

Note that during the OLE Phase, it is possible for the Treating Investigator to have access to MRI scans performed during the OLE Phase.

### 5.3.4 Safety

Adverse events, vital signs, weight, physical and neurological examination, clinical laboratory tests (including pregnancy tests), 12-lead ECG, locally reviewed MRI for safety (non-MS CNS pathology), and data on concomitant medications and diseases will be collected throughout the study.

Please note: On the infusion days, the vital signs should be taken within 45 minutes prior to the methylprednisolone infusion in all patients. In addition, the vital signs should be obtained prior to the study drug infusion, then every 15 minutes (± 5 minutes) for the first hour; then every 30 minutes (± 10 minutes) until 1 hour after the end of the infusion. On non-infusion days, the vital signs may be taken at any time during the visit. Additional vital signs readings may be taken at the discretion of the Investigator in the event of an infusion related reaction or if clinically indicated and should be recorded on the unscheduled vital signs eCRF.

Please refer to relevant sections of protocol for more details.

#### *5.3.4.1 Electrocardiogram*

A 12-lead ECG should be taken at the visits indicated in the Schedule of Assessments - Table 4. Comments generated automatically by the ECG machine should not be recorded in the eCRF unless confirmed by a physician. An ECG is also required if the patient prematurely withdraws from the study.

#### *5.3.4.2 Physical Examination*

The physical examination will be performed as per Schedule of Assessments. Diagnosis of new abnormalities or clinically significant worsening of pre-existing abnormalities should be recorded as adverse events if appropriate.

#### *5.3.4.3 Neurological Examination*

A neurological examination will be performed at every planned visit and at unscheduled visit if applicable.

- In the presence of newly identified or worsening neurological symptoms*at any given time in the study (double-blind treatment, safety follow-up or open label extension period)*, a neurological evaluation should be scheduled promptly. In case of events suggestive of relapse the Treating Investigator should request EDSS to be performed by the Examining Investigator*, whenever possible within 7 days of the onset of the relapse.*

Study Investigators will screen patients for signs and symptoms of PML by evaluating neurological deficits localized to the cerebral cortex, such as cortical symptoms/signs, behavioral and neuropsychological alteration, retrochiasmal visual defects, hemiparesis, cerebellar symptoms/signs (e.g., gait abnormalities, limb incoordination). A brain MRI scan and CSF analysis may be warranted to assist in the diagnosis of PML. See Section 7.3.4.1 for guidance on the diagnosis of PML.

Patients with suspected PML, defined as a new or worsening neurological symptom which necessitates MRI and or lumbar puncture and CSF analyses to rule out PML, should be withheld from study treatment until PML is ruled out by complete clinical evaluation and appropriate diagnostic testing (see Section 7.3.4.1) The Sponsor's Medical Responsible and Medical Monitor should be contacted by email. In addition Sponsor medical responsible person should be immediately contacted by phone.

A patient with confirmed PML should be withdrawn from treatment. PML should be reported as an SAE (with all available information) with immediate notification of the Medical Monitor (see also Section 7.1.1.3).

#### *5.3.4.4 Telephone Interviews*

The purpose of this semi-structured interview is to identify new or worsening neurological symptoms that warrant an unscheduled visit and collect information on possible events of infections. The telephone interview will be conducted by site personnel familiar with the patient(s) every 4 weeks (± 3 days) between the study visits during the double-blind, double-dummy treatment period, OLE Phase Screening Period, OLE Phase, and Safety Follow-Up Period starting from Week 8, until 48 weeks after the

last infusion. Thereafter, for those patients who require prolonged B-cell monitoring, telephone interviews will continue every 12 weeks (±7 days) between regular visits.

The site will record in the eCRF the telephone interview as “Done” or “Not Done” and documentation of the interview will be maintained in the patient’s study file.

Please refer to Appendix 4 for detailed information.

#### 5.3.4.5 *Columbia-Suicide Severity Rating Scale*

The Columbia-Suicide Severity Rating Scale (C-SSRS) will be used during the double-blind, double-dummy treatment period and the OLE Phase Screening Period for prospective suicidality assessment. C-SSRS is a tool used to assess the lifetime suicidality of a patient and to track suicidal events through the treatment. The structured interview prompts recollection of suicidal ideation, including the intensity of the ideation, behavior and attempts with actual/potential lethality.

The scale will be administered by the Treating Physician or a qualified designee at the timepoints indicated in the Schedule of Assessments. The C-SSRS “baseline” will be collected at baseline and the C-SSRS “since last visit” will be collected at subsequent visits.

Please note: assessing the risk of suicide is a difficult and complex task when applied to the individual patient. Certainly, no single clinical scale can replace a thorough medical examination and suicide risk assessment. Ultimately, the determination of the presence of suicidality depends on clinical judgment.

### 5.3.5 The Karnofsky Performance Scale (Clinician-Reported Version)

The Karnofsky Performance Scale score allows patients to be classified as to their functional impairment. This scale is usually used to compare effectiveness of different therapies and to assess the prognosis in individual patients. The lower the Karnofsky score, the worse the survival for most serious illnesses.

The scale will be administered by Examining Investigator at the time points indicated in the Schedule of Assessments -Table 4.

## 5.4 Laboratory Assessments

Roche Clinical Repository biomarker samples will be shipped directly to Roche Clinical Sample Operations unit. All other lab samples collected during the study will be shipped to Central Laboratory.

The procedures for the collection, handling and shipping of laboratory samples are specified in the Laboratory Manual.

The samples for this study should be classified, packed and shipped as UN3373 Biological Substance, Category B.

Full details of the central laboratory sample handling, shipment and reporting of results will be described in the Laboratory Manual.

During the double-blind treatment period of the study, the total volume of blood loss for laboratory assessments will be approximately 341 mL over 2 years. The amount of blood taken at each visit will vary, but will be no more than 56 mL. During the OLE Phase and in the Safety Follow-Up Period, the amount of blood taken at each visit will be no more than 41 mL. Patients consenting for RCR project may have additional blood samples taken – please refer to Section 5.5.1 for more details.

### 5.4.1 Standard Laboratory Assessments

Please note: Some laboratory parameters that could reveal patient's allocation to study treatment, such as FACS cell counts, absolute neutrophil counts, Ig levels, and type I interferon neutralizing antibody levels, will be blinded. In order to ensure patients' safety in the study and to allow for assessments of the re-treatment criteria, a central laboratory will provide study Investigators and Medical Monitors with reflex messages triggered by critical blinded laboratory results. Investigators notified of their patient's critical laboratory test results will be instructed to suspend further treatment with study drug until the patient becomes eligible for re-treatment. The reflex messages from a central laboratory, together with non-blinded laboratory results, should be carefully reviewed at every visit before continuing with study treatment. The reflex messages will occur during the double-blind, double-dummy treatment period until the fifth cycle (first cycle of OLE Phase). The reflex messages will not be in effect from the sixth cycle onward. Further details will be provided in Laboratory Manual.

**Hematology:** Hemoglobin, hematocrit, red blood cells (RBC), white blood cells (WBC) (absolute and differential), absolute neutrophil count, and quantitative platelet count.

**Blood chemistry:** AST/SGOT, ALT/SGPT, GGT, alkaline phosphatase, amylase, lipase, total protein, albumin, cholesterol, total bilirubin, urea, uric acid, creatinine, random glucose, potassium, sodium, calcium, phosphorus, lactic dehydrogenase, creatine phosphokinase, and triglycerides.

**Thyroid function test:** sTSH will be tested at screening, and yearly during the double-blind, double–dummy Treatment Period. Thyroid autoantibodies will be assayed only at screening.

**FACS** will include (but is not limited to) the following cells:

- Total B cells ($CD19^{pos}$)
- Total T cell ($CD3^{pos}$)
- T helper cells ($CD3^{pos}$, $CD4^{pos}$)
- $T_{CTL}$ ($CD3^{pos}$, $CD8^{pos}$)
- NK Cells ($CD3^{neg}$, $CD16/56^{pos}$)
- B-cell subsets:
  - memory B-cells ($CD19^{pos}$, $CD27^{pos}$, $CD38^{neg}$)
  - naïve B-cells ($CD19^{pos}$, $CD27^{neg}$, $IgD^{pos}$)

– plasmablasts ($CD19^{lo}$, $CD27^{pos}$, $CD38^{hi}$,)

**Quantitative Immunoglobulin:** Ig levels (including Total Ig, IgG, IgM, and IgA isotypes).

**Antibody titers:** Measurement of antibody titers to common antigens (mumps, rubella, varicella, *S. pneumoniae*) will be performed. This information is used to assess the effect of ocrelizumab on specific humoral immunity to bacterial and viral antigens.

**HAHA:** Serum samples will be collected for determination of antibodies against ocrelizumab (HAHA*; also known as ADA*). Since ocrelizumab concentrations affect the HAHA assay, the concentration of ocrelizumab will be measured as well at all timepoints with HAHA assessment to enable interpretation of the results (PK sample). For details please refer to Schedule of Assessments.

**Pregnancy Test**: All women of childbearing potential must have regular pregnancy tests. At screening, a serum pregnancy test will be performed in central laboratory. During the double-blind, double-dummy treatment period and Safety Follow up, a urine pregnancy test (sensitivity of at least 25 mIU/mL β-hCG) will be performed locally at the time points shown in Schedule of Assessments -Table 4. On infusion visits, the urine pregnancy test should be performed prior to the methylprednisolone infusion. A positive urine pregnancy test should be confirmed with a serum test through the central laboratory prior to any further dosing with ocrelizumab.

Please note: additional laboratory tests will be performed at screening in order to verify eligibility criteria. Please refer to Table 4 for further details.

### 5.4.2 Hepatitis Screening and Liver Function Monitoring

Patients with recurrent or chronic hepatitis B or history/presence of hepatitis C infection must be excluded from enrollment into the study (see Section 4.3). In addition, hepatitis B and C serology will be performed at screening. A positive result to either hepatitis surface antigen (HBsAg), or hepatitis B core antibody (total HBcAb) associated with positive viral DNA titres as measured by PCR, or a positive result for hepatitis C antibody (HepCAb) should result in the patient's exclusion. Patients with evidence of past resolved hepatitis B infection (i.e., positive total hepatitis B core antibody associated with a negative viral DNA) can be enrolled, and will have the hepatitis B viral DNA checked *regularly* as per Schedule of Assessment. Patients in whom the viral DNA becomes positive but in whom the quantity is at the lower limit of detection of the assay should have the test repeated as soon as possible. Patients found to have a confirmed viral DNA-positive test should be referred to a hepatologist for immediate assessment. These patients will not receive further infusions of ocrelizumab and will enter the Safety Follow-Up Period.

Liver function, i.e., ALT/SGPT, AST/SGOT, gamma glutamyl transferase (GGT), alkaline phosphatase, total bilirubin, should be reviewed throughout the study. Patients developing evidence of liver dysfunction should be assessed for viral hepatitis and, if necessary, referred to a hepatologist or other appropriately qualified expert. Study drug should be withheld until the diagnosis of viral hepatitis has been excluded. Patients developing hepatitis B or C should be withdrawn from the study and should enter

the Safety Follow up period. Should treatment be prescribed, this will be recorded in the eCRF. Patients with viral hepatitis due to other agents, such as hepatitis A, may resume treatment after the patient's recovery.

Please refer also to Section 6.2.2 for further guidelines on liver function monitoring.

#### 5.4.3 Plasma and Urine Banking for JC Virus

Long-term storage of plasma samples and urine is planned for JC virus DNA and/or other relevant tests for JC virus, independent of an occurrence of suspected PML case (see Section 7.3.4.1). Plasma samples (5 mL) and urine samples (10 mL) will be collected as per Schedule of Assessments. As the assay of the virus DNA has not been standardized, and a correlation between viremia and onset of PML has not been established, the JC virus assessments in plasma and urine will be performed if deemed necessary in the future and not on an ongoing basis.

#### 5.4.4 Pharmacokinetic/Pharmacodynamic Assessments

Blood samples will be collected to evaluate the pharmacokinetics and pharmacodynamics of ocrelizumab as described in the Schedule of Assessments (Table 4). The blood volume collected for pharmacokinetic assessments will be approximately 2 mL per sample. These samples will be assayed for ocrelizumab concentration using an enzyme-linked immunosorbent assay (ELISA).

Serum samples for determination of ocrelizumab concentrations will be collected at the time points detailed in the Schedule of Assessments (Table 4). On the infusion visit at week 72, two serum samples should be collected, one 5-30 minutes prior to the methylprednisolone infusion and the second one 30 minutes (±10 minutes) following the completion of the ocrelizumab infusion. For all other infusion visits, a blood sample should be taken 5 - 30 minutes before the methylprednisolone infusion. At other times (non-infusion visits), samples may be taken at any time during the visit.

For sampling procedures, storage conditions, and shipment instructions, see the Sample Handling and Logistics Manual, which will be provided to each site.

#### 5.4.5 Type I Interferon Neutralizing Antibody Assay

Type I interferon neutralizing antibody assay will be performed during the Double-Blind, Double-Dummy Treatment Period - please see Table 4 - Schedule of Assessments.

### 5.5 Roche Clinical Repository Specimen(s)

Please note: the Roche Clinical Repository research is contingent on review and approval for the exploratory biomarker assessments by an appropriate regulatory body (depending on the country where the study is performed) and a site's Institutional Review Board/ Ethics Committee. Written patient's informed consent to RCR project is also required. If a regulatory body or site's Institutional Review Board / Ethics Committee does not approve the extended analysis and long term storage of the biomarker samples, this section of protocol will not be applicable.

Specimens for dynamic (non-inherited) biomarker discovery and validation will be collected only from patients consenting to RCR.

These specimens will be used for research purposes to identify dynamic biomarkers that are predictive of response to ocrelizumab treatment (in terms of dose, safety and tolerability) and will help to better understand the pathogenesis, course and outcome of multiple sclerosis and related diseases. Specimens for dynamic biomarker discovery will be single coded like any other clinical sample (labeled and tracked using the patient's study identification number (see Section 17).

The results of specimen analysis from the RCR will facilitate the rational design of new pharmaceutical agents and the development of diagnostic tests, which may allow for individualized drug therapy for patients in the future.

All RCR specimens will be destroyed no later than 15 years after the final freeze of the respective clinical database unless regulatory authorities require that specimens be maintained for a longer period. The specimens in the RCR will be made available for future biomarker research towards further understanding of MS treatment with ocrelizumab, related diseases and adverse events and for the development of potential associated diagnostic assays. The implementation and use of the RCR specimens is governed by the Roche Clinical Repository policy to ensure the appropriate use of the RCR specimens.

### 5.5.1 Specimen Types

Exploratory Biomarkers (non-DNA):

– **Plasma assays**

Blood (one, approximately 6 mL sample in EDTA) for plasma isolation will be obtained at various time points as shown in Schedule of Assessments (Table 4). These samples will be used for biomarker assays which may include chemokines and other candidate biomarkers in multiple sclerosis. For sampling procedures, storage conditions and shipment instructions see study Sample Handling and Logistics Manual.

– **Blood for RNA expression profiling**

Blood (2 × approximately 2.5 mL collected in PAXgene vacutainers) for RNA isolation will be obtained at various time points as shown in Schedule of Assessments (Table 4). The samples may be tested using techniques such as a micro array profiling system and/or RT PCR to study the expression profile of genes known to be involved with multiple sclerosis, and any other differentially expressed genes relative to treatment response or re-treatment. For sampling procedures, storage conditions and shipment instructions see study Sample Handling and Logistics Manual.

Exploratory Biomarkers (DNA): one sample of 6 mL of blood will be taken *in the double-blind, double-dummy treatment phase of the study* as per Schedule of Assessments (Table 4). A 6 mL whole blood sample will be obtained only from patients consenting to RCR for pharmacogenetic and genetic analysis. If not done at Baseline (Visit 2), the sample may be collected at the next visit.

For all samples, dates of specimen collection should be recorded on the associated RCR page of the eCRF and/or in the clinical database.

## 5.6 Protein Biomarker Samples

Specimens for protein biomarker discovery and validation will be collected from all patients. These specimens will be used for research purposes to identify and/or verify protein biomarkers that are predictive of response to ocrelizumab treatment (in terms of dose, safety and tolerability) and will help to understand the pathogenesis, course and outcome of relapsing MS and related diseases. Identification of patient subgroups with increased response to therapy or increased progression rates would provide information of significant clinical value to guide treatment decisions and aid in the appropriate use of the therapy. Analyses will include but are not limited to *interleukin-6*.

A 6 mL sample of whole blood will be collected in a plain tube without EDTA for serum isolation. For sampling procedures, storage conditions and shipment instructions see study Sample Handling and Logistics Manual, which will be provided to each study site.

Blood specimens for protein biomarker discovery and validation will be collected from all patients as per Schedule of Assessments. These specimens will be stored for 5 years after the end of the study and then destroyed, unless a different regulation for storage time is in place at a given site.

## 5.7 Patient Reported Outcome(s)

PRO data will be collected at the study visit with an electronic tablet device during the double-blind, double-dummy treatment period. The tablet with the PRO instruments will be distributed by the Investigator staff and completed in their entirely by the patient.

Please note: all PROs are required to be administered prior to administration of study drug and prior to any other study assessment(s) to ensure the validity of the instruments is not compromised, and data quality meet requirements of regulatory authorities and best practices [60, 61].

PRO data will be elicited from patients in this study to better characterize the clinical profile of ocrelizumab. These PRO measurements are described in Sections 5.7.1, 5.7.2, and 5.7.3. Please note that the methods for collecting and analyzing PRO data are different from those for the ascertainment of observed or volunteered adverse events. Due to these differences, PRO data will not be reported as adverse events and no attempt will be made to resolve any noticeable discrepancies between PRO data and observed or volunteered adverse events.

Patients who are unable to complete exploratory assessments (e.g., ePROs) due to physical/disease limitations will not be excluded from the study.

### 5.7.1 Modified Fatigue Impact Scale

The Modified Fatigue Impact Scale (MFIS) will assess change in the level of fatigue during the double-blind, double-dummy treatment period. The MFIS is a 21-item instrument that asks patients to rate their fatigue over the past four weeks on a 5-point Likert scale, indicating “Never” to “Almost always.” Four scores can be derived from the MFIS, including a total score as well as scores for three subscales: physical, cognitive, and psychosocial functioning. Changes from baseline will be calculated for the total scale scores as well as for the subscale scores.

English version of MFIS is provided in Appendix 5.

### 5.7.2 The Center for Epidemiologic Studies Depression Scale

The Center for Epidemiologic Studies Depression Scale (CES-D) will be used to evaluate patients for depressive symptoms during the double-blind, double-dummy treatment period. The CES-D is a 20-item self-report instrument that asks patients to rate their feelings and behaviors over the past week on a 4-point Likert scale, from "Rarely or none of the time (less than one day)" to "Most or all of the time (5-7 days)." Only a total scale score is calculated for the CES-D.

English version of CES-D is provided in Appendix 6.

### 5.7.3 The Short Form Health Survey

The Short Form (SF-36 *v2*) Health Survey is a generic quality of life instrument that has been widely tested for its psychometric properties and is widely used in clinical and epidemiological studies. The SF-36*v2* contains 36 items and measures eight health domains: vitality, physical functioning, bodily pain, general health perceptions, physical role functioning, emotional role functioning, social role functioning, and mental health. The SF-36*v2* yields a score for each domain, as well as summary scores for the physical and mental dimensions. It can be completed in 5-10 minutes.

English version of the Short Form (SF-36*v2*) Health Survey is provided in Appendix 7.

### 5.7.4 Patient's Assessment of Treatment Benefit

The Patient Perception of Treatment Questionnaire will be assessed *after* administration of PRO instruments at each visit in which PRO assessments are made, except for baseline. Specifically, patients will be asked whether they think their MS has become better, become worse or been stable since baseline. This question will serve as a global assessment from the patient perspective and will provide a useful anchor to help interpret the clinical meaningfulness of PRO results.

## 5.8 Pharmacoeconomic Assessments/EQ-5D

Pharmacoeconomic assessments will be included for purposes of deriving health utilities for economic modeling. The EQ-5D will be used to derive utilities for health states included in MS economic models and will be administered as per Schedule of Assessments.

The EQ-5D (formerly known as EuroQOL) is a generic, preference-based health-related quality of life instrument. It has five dimensions assessing mobility, self-care, usual activities, pain/discomfort and anxiety /depression. Each dimension has 3 possible levels. Different combinations of responses are utility-weighted to produce a single health utility index. The Visual Analog Scale (VAS) measures self-reported health on a scale between "worst imaginable" and "best imaginable" health states.

EQ-5D is a patient reported outcome and should be performed before any other study assessments and before administration of study drug in order to minimize bias.

## 5.9 Optional Exploratory Substudies

Patients who are randomized to the main study protocol WA21092 have the option to participate in exploratory substudies upon separate consent and fulfillment of additional exploratory substudy protocol criteria. *The Optical Coherence Tomography Exploratory Substudy (OCT) is included in the main study protocol (see Section 5.9.1 and Appendix 8). Other* exploratory substudies are *run under separate study protocols: Substudy of brain and spinal cord MRI in patients with MS participating in the OPERA clinical trial (BE29352); B cell and T cell repertoires in ocrelizumab-treated MS patients (BE29353); brain myelin mapping to quantify demyelination and repair in MS in a Phase 3 trial of ocrelizumab (BE29340); and assessment of ocrelizumab treatment effects on disability of MS patients enrolled in the Phase 3 Orchestra program using multimodal evoked potentials (mEP) and high-resolution electroencephalogram (EEG; BE29354). Substudies will be run only at the specific assigned sites that are referred to in the substudy protocols.*

### 5.9.1 Optical Coherence Tomography Exploratory Substudy

A Roche-sponsored, multi-center, Optical Coherence Tomography (OCT) Exploratory Substudy is being conducted. OCT is a noninvasive imaging tool capable of measuring changes in structural architecture of the retina and retinal nerve fiber sensitively and rapidly [62] OCT can be of particular interest in MS, because optic neuritis is often the pivotal event in establishing the diagnosis of MS. Optic nerve dysfunction is characterized by optic disc pallor, loss of contrast sensitivity, and visual field defects and may occur subclinically in many other patients. It is estimated that nearly 20% of all patients with MS present initially with optical neuritis, and an additional 30%–100% will have optical neuritis at some point in their disease course [63]. OCT outcome measures such as RNFL thickness and macular volumes have been shown to correlate with clinical measures of vision loss and may facilitate visualization of any process of neurodegeneration or repair as part of natural history of MS or as a consequence of neuroprotective interventions [64].

The procedures and schedule of assessment are specified in the substudy protocol (Appendix 8) and in the Independent Review Committee Charter.

## 5.10 Open-Label Extension Phase

### 5.10.1 Open-Label Extension Phase Screening Period

Patients who have completed the 96-week double-blind, double-dummy treatment period, and who, in the opinion of the Treating Investigator, may benefit from treatment with ocrelizumab, will be offered the opportunity to participate in the OLE Phase of the study. Eligible patients who are not willing to participate in the OLE Phase of the study will be entered into the Safety Follow-Up Period (see below).

It should be noted that in the case of a patient who initially declines participation in the OLE Phase and subsequently reconsiders the decision, the patient will have up to 24 weeks after the Week 96 Visit to enter the OLE Phase. In this instance, he or she should not have taken any prohibited medication as specified in Section 4.5.2.1.

Patients who have consented to participate in the OLE Phase will enter an OLE Phase Screening Period to be evaluated for eligibility. The OLE Phase Screening Period will start after all assessments at the Week 96 Visit have been performed, and it should last *up to* 4 weeks. It is possible that the OLE Phase Screening Period could be longer than 4 weeks. If a prolongation of the OLE Phase Screening Period is needed, it should be discussed with the Sponsor on a case-by-case basis.

Information from assessments performed during the Week 96 Visit will be used to verify the eligibility of the patient for the OLE Phase of the study. Please refer to Section 4.4.

During the OLE Phase Screening Period, all patients should receive Rebif®/Rebif® placebo (depending on initial arm assigned to) until the first infusion of Cycle 5. Please refer to Table 7 and Table 10 for more details regarding the Rebif®/Rebif® placebo regimen.

Patients who withdraw from the OLE Phase Screening Period will also be entered into the Safety Follow-Up Period (see below).

### 5.10.2 Open-Label Extension Phase

The OLE Phase starts on the first infusion of Cycle 5 with continued dosing cycles every 24 weeks and up to the end of study (see Section 3.1.4). Patient treatment allocation during the double-blind, double-dummy treatment period will not be unblinded *until the the last data point from the last patient is received, as required for statistical analysis defined in Section 8.2 and the SAP* (see *also* Sections 3.1.3 and 6.4).

Study procedures at this visit will also include the following, but are not limited to:

- Physical examination;
- Neurological exam;
- Routine safety lab (blood and urine sampling), Hep B virus DNA (if applicable), FACS analysis, immunoglobulin levels.

The first cycle of the OLE Phase (Cycle 5) will consist of two i.v. infusions of 300 mg ocrelizumab separated by 14 days. Subsequent cycles (Cycles 6, 7, 8, etc.) will be with single i.v. infusions of 600 mg ocrelizumab to all patients enrolled in OLE Phase of the study.

Note: This initial dual infusion is designed to both optimize the initial treatment effect and to minimize any infusion-related reactions for patients initially assigned to the ocrelizumab placebo/Rebif® verum arm. In order to avoid unblinding of the assigned arm in the double-blind, double-dummy treatment period, both arms should receive dual infusion as described above.

### 5.10.3 Overview of Schedule of Assessments in the Open-Label Extension Phase

Patients participating in OLE Phase will be assessed at clinical visits every 24 weeks as per the Schedule of Assessments (see). For the description of the assessments, please refer to Section 5.3.2.

The mechanisms necessary to guarantee assessor blindness are not necessary during the OLE Phase. All required assessments during the OLE Phase should occur as described in Section 5. It is recommended that the same EDSS assessor perform the test throughout the OLE Phase.

Visits should be scheduled with reference to the date of first infusion during the OLE Phase (Cycle 5). The visit for the second infusion should be scheduled 14 days after the first infusion of Cycle 5. A minimum interval of 20 weeks should be kept between the ocrelizumab second infusion of Cycle 5 and the next infusion at Cycle 6. A minimum of 22 weeks should occur between ocrelizumab single infusions administered during Cycle 6 onward. In the unforeseen cases that an infusion is delayed, additional tests or assessments, such as routine safety laboratory tests, may be performed when the Investigator judges that these are warranted. At infusion visits, patients should remain in observation for at least 1 hour after the completion of the infusion.

In order to verify re-treatment criteria for infusions in Cycle 6 onwards, patients should attend a scheduled visit 2 weeks prior to the infusion visit.

Additional unscheduled visits for the assessment of potential MS relapses, new neurological symptoms, or safety events may occur at any time. Assessments performed at unscheduled (non-dosing) visits will depend on the clinical needs of the patient.

Patients with new neurological symptoms suggestive of MS relapse should have an EDSS performed by the Examining Investigator, *whenever possible within 7 days of the relapse onset date*. Other tests/assessments may be done as appropriate.

Please refer also to Section 7.3.4.1 for guidance on the diagnosis of PML.

#### 5.10.3.1 Delayed Dosing Visit in the Open-Label Extension Phase

Delayed dosing visits may be scheduled if the infusion cannot be administered at the time points defined in the Schedule of Assessments -Table 6. Thus, a patient who had all assessments of a dosing visit performed, but could not receive his/her infusion, should be re-scheduled for the infusion.

At the delayed dosing visit, additional tests or assessments, such as routine safety laboratory tests, may be performed when the Investigator judges that these are warranted.

#### 5.10.3.2 Unscheduled Visits in the Open-Label Extension Phase

Patients who develop new or worsening neurological symptoms should be seen at the investigational site as soon as possible regardless of the dates of their pre-planned, scheduled study visits. Assessments performed at unscheduled (non-dosing) visits will depend on the clinical needs of the patient.

Patients with new neurological symptoms suggestive of relapse should have an EDSS performed by the Examining Investigator, *whenever possible within 7 days of the relapse onset date*. Other tests/assessments may be done as appropriate. It should be noted if, during the *Open Label Extension screening* period, Rebif®/Rebif® Placebo dose modification is necessary in the case of ALT elevations, then unscheduled visits may be required for dispensing of study medication (please refer to Section 6.2).

Please refer also to Section 7.3.4.1 for guidance on the diagnosis of PML.

#### 5.10.3.3 Withdrawal Visits in the Open-Label Extension Phase

At the moment a patient meets one or more of the withdrawal criteria (Section 4.6), this patient is regarded as withdrawn from treatment. Patients who withdraw from ocrelizumab treatment will need to complete all assessments as shown in Schedule of Assessments - Table 6 and will be entered into the Safety Follow-Up Period.

For patients who have withdrawn from the OLE Phase or who are not eligible for treatment with ocrelizumab, it is at the discretion of the Investigator to decide on further treatment of the underlying disease. However, immunosuppressants, lymphocyte depleting therapies, and lymphocyte trafficking blockers may increase the risk of infections while patients remain B-cell depleted and therefore are not allowed during the Safety Follow-Up Period.

## 6. INVESTIGATIONAL MEDICINAL PRODUCT

During the double-blind, double-dummy treatment period, patients will be randomly assigned into one of two treatment groups:

– **Group A** – Ocrelizumab 600 mg regimen (given as dual infusions of 300 mg of ocrelizumab 14 days apart for the first 24 weeks and single infusions of 600 mg every 24 weeks thereafter)

– **Group B** – Rebif® subcutaneous injections, 3x weekly

**Table 7: Treatment Groups and Schedule of Study Medication During the Double-Blind, Double-Dummy Treatment Period**

| Treatment Group | Schedule of Study Medication |
|---|---|
| **Group A**<br>Ocrelizumab 600 mg regimen | Two i.v. infusions of ocrelizumab 300 mg separated by 14 days for the first 24 weeks, followed by single i.v. infusions of ocrelizumab 600 mg every 24 weeks thereafter<br>AND<br>placebo Rebif® subcutaneous injections, 3x weekly. |
| **Group B**<br>Rebif® | Rebif® subcutaneous injections, 3x weekly<br>AND<br>Two i.v. infusions of placebo ocrelizumab 300 mg separated by 14 days for the first 24 weeks, followed by single i.v. infusions of placebo ocrelizumab 600 mg every 24 weeks thereafter. |

The first i.v. infusion of ocrelizumab or placebo, or the first subcutaneous injection of Rebif® or placebo will be administered on study Day 1.

During the OLE Phase Screening Period, patients will continue to receive blinded treatment according to their initial assignment:

- **Group A** – Rebif® placebo s.c. injections, 3x weekly

- **Group B** – Rebif® s.c. injections, 3x weekly

During the OLE Phase, all patients will receive ocrelizumab i.v. infusions every 24 weeks. At Cycle 5, patients will receive 2 i.v. infusions of 300 mg ocrelizumab separated by 14 days during the 24-week cycle. Beginning with Cycle 6, patients will receive ocrelizumab 600 mg every 24 weeks.

Please note: 100 mg of methylprednisolone i.v. will be administered prior to each i.v. infusion of ocrelizumab/ocrelizumab placebo during the double-blind, double-dummy treatment period and prior to each i.v. infusion of ocrelizumab during the OLE Phase. In the event that the use of methylprednisolone is contraindicated for the patient, use of an equivalent dose of an alternative steroid is allowed as premedication.

## 6.1 Ocrelizumab

### 6.1.1 Preparation and Administration of Ocrelizumab Infusions

**Detailed instructions for the preparation of the infusion bags containing the study drug will be provided separately in the Dose Preparation Guidelines.**

Although ocrelizumab may be administered on an outpatient basis, patients may be hospitalized for observation at the discretion of the Investigator (in some countries this is the standard procedure). The study drug infusions should always be administered in a hospital or clinic environment under close supervision of the Investigator or a medically qualified staff member with immediate availability of full resuscitation facilities.

**Preparation of infusion**

Ocrelizumab drug product must be diluted before administration. Solutions of ocrelizumab for i.v. administration are prepared by dilution of the drug product or ocrelizumab-matching placebo into an infusion bag containing 0.9% sodium chloride, to a final drug concentration of approximately 1.16 mg/mL. Specific instructions are provided separately in the Dose Preparation Guidelines. It is important not to use evacuated glass containers (to prepare the infusion), which require vented administration sets because this causes foaming as air bubbles pass through the solution.

Prior to the start of the infusion, please ensure that the content of the bags is at room temperature to avoid an infusion reaction due to the administration of the solution at low temperatures.

**Infusion procedures**

Ocrelizumab should be given as a slow i.v. infusion. It must not be administered as an i.v. push or bolus. Well adjusted infusion pumps should be used to control the infusion rate and the study drug should be infused through a dedicated line.

All patients should receive pre-treatment before the infusion (see Section 6.1.2).

**Dual infusion cycle (Cycle 1 *and OLE Cycle* 5)**

The first cycle *of the double-blind double dummy treatment period (Cycle 1) and OLE Phase (Cycle 5)* will consist of 2 infusions of 300 mg ocrelizumab administered 14 days apart. For each infusion it is necessary to prepare a single infusion bag containing 300 mg ocrelizumab. Specific instructions will be provided separately in the Dose Preparation Guidelines and must be followed exactly. The infusion should be started at a rate of 32 mL/h. This should be escalated at the rates shown in Table 8.

**Table 8: Infusions of Ocrelizumab 300 mg**

| Time (Minutes) | Infusion Rate (mL/hr) | Maximum Dose per Interval (mg) | Cumulative Dose (mg) |
|---|---|---|---|
| 0-30 | 32 | 18.75 | 18.75 |
| 31-60 | 65 | 37.5 | 56.25 |
| 61-90 | 97 | 56.25 | 112.5 |
| 91-120 | 129 | 75 | 187.5 |
| 121-150* | 194 | 112.5 | 300* |

*Infusion of 300 mg of ocrelizumab should be completed at approximately 150 minutes (~2.5 hours).

**Single infusion cycles (Cycles 2 - 4*, and OLE Cycle 6 onwards*)**

Cycles 2 through 4 *of the double-blind, double dummy treatment period and Cycle 6 onwards of OLE Phase* will consist of one infusion of 600 mg ocrelizumab administered on Day 1 of each cycle. For each cycle it is necessary to prepare a single infusion bag containing a total of 600 mg ocrelizumab/ocrelizumab placebo. Specific instructions will be provided separately in the Dose Preparation Guidelines and must be followed exactly. The infusion should be started at a rate of 40 mL/h. This should be escalated at the rates shown in Table 9.

**Table 9: Subsequent Infusions of Ocrelizumab 600 mg**

| Time (Minutes) | Infusion Rate (mL/hr) | Maximum Dose per Interval (mg) | Cumulative Dose (mg) |
|---|---|---|---|
| 0-30 | 40 | 23.18 | 23.18 |
| 31-60 | 85 | 49.27 | 72.45 |
| 61-90 | 130 | 75.36 | 147.81 |
| 91-120 | 169 | 98.05 | 245.86 |
| 121-215* | 200 | 354.14** | 600.00 |

*The infusion of 600 mg ocrelizumab should be completed at approximately 215 minutes (~3.6 hours).
**This last interval is approximately 95 minutes, delivering a max dose of 115.94 mg per 0.5 hour.

Because of possible need to vary infusion rates depending on tolerance of the infusion, the total infusion time may exceed the time stated. UNLESS AN INFUSION REACTION OCCURS NECESSITATING DISCONTINUATION, THE ENTIRE CONTENT OF INFUSION BAG MUST BE ADMINISTERED TO THE PATIENT.

After completion of the infusion, the i.v. cannula should remain in situ for at least 1 hour in order to be able to administer drugs intravenously, if necessary in the event of a delayed reaction. If no adverse events occur during this period of time, the i.v. cannula may be removed and the patient may be discharged.

Because ocrelizumab solutions for infusion do not contain a preservative, the i.v. bags containing ocrelizumab diluted solutions should be stored refrigerated at 2-8°C. They may be stored under refrigerated conditions for up to 24 hours prior to use. Bags containing ocrelizumab diluted solutions for i.v. infusion need to be used within 48 hours of preparation (i.e., stable 24 hours at 2-8°C plus 24 hours at room temperature). As noted above, the diluted infusion bags should be at room temperature prior to administration to the patient.

### 6.1.2 Prevention and Treatment of Infusion Related Reactions

Methylprednisolone has been shown to decrease the incidence and the severity of infusion reactions. In RA patients treated with a similar agent, rituximab, the rate and severity of infusion reactions markedly decreased with i.v. corticosteroid pre-medication [65]. To reduce potential infusion reactions, all patients will receive prophylactic treatment with 100 mg of methylprednisolone, administered by slow i.v. infusion, to be completed approximately 30 minutes before the start of each ocrelizumab infusion. In the event that the use of methylprednisolone is contraindicated, use of an equivalent dose of alternative steroid should be used as premedication prior to the infusion.

It is also recommended that the infusion is accompanied by prophylactic treatment with an analgesic/antipyretic such as acetaminophen/paracetamol (1 g) and an i.v. or oral antihistaminic (such as i.v. diphenhydramine 50 mg; or equivalent dose of alternative) 30 to 60 minutes prior to the start of an infusion to reduce potential infusion reactions. Patients administered a sedating antihistaminic for the treatment or prevention of infusion reactions should be given appropriate warnings concerning drowsiness and potential impairment of ability to drive or operate machinery.

Since transient hypotension may occur during ocrelizumab infusion, the Investigator may wish to withhold anti-hypertensive medications 12 hours prior to ocrelizumab infusion.

IRRs should be treated symptomatically with oral acetaminophen/paracetamol (1 g), and intramuscular or slow i.v. antihistamine administration, such as diphenhydramine (25 mg to 100 mg). Acetaminophen/paracetamol and diphenhydramine dosing should be repeated as clinically indicated. Non-allergic events should be treated symptomatically as judged clinically relevant by the Investigator.

**In patients with associated respiratory symptoms (stridor, wheeze or bronchospasm), additional treatment with bronchodilators may be indicated.**

One patient with well-controlled asthma at baseline experienced an acute asthma attack following their first rituximab infusion. Physicians should therefore monitor patients with a history of asthma carefully and institute an appropriate treatment if signs and symptoms of asthma are noticed.

Section 6.1.3 details the reduction, interruption or discontinuation of the infusion in the event of an infusion reaction.

### 6.1.3 Ocrelizumab Dose Modifications, Interruptions and Delays

No ocrelizumab dose modifications are foreseen.

Slowing of the infusion rate or interruption of the infusion may be necessary in the event of an infusion reaction. In rare patients, ocrelizumab treatment may need to be discontinued. Guidance is provided below.

**Handling infusion reactions:**

Handling of IRRs will depend on the intensity of symptoms (see also Section 7.1.1.1 for grading of intensity of IRRs).

In the event that a patient experiences a (CTCAE Grade 1 or 2 – Appendix 3) infusion-related event, the infusion rate should be reduced to half the rate being given at the time of onset of the event (e.g., from 50 mL/hr to 25 mL/hr or from 100 mL/hr to 50 mL/hr). Once the event has resolved, the Investigator should wait for 30 minutes while delivering the infusion at the reduced rate. If tolerated, the infusion rate may then be increased to the next closest rate on the patient's infusion schedule and the rate increments resumed.

Patients who experience an infusion-related event (CTCAE Grade 3) or flushing, fever and throat pain cluster should have their infusion interrupted immediately and should receive aggressive symptomatic treatment. The infusion should be re-started only after all the symptoms have disappeared. The initial infusion rate at restart should be half of the infusion rate that was in progress at the time of onset of the reaction.

**Please note: patients who experience a life threatening infusion-related event (CTCAE Grade 4) during an infusion should have their infusion immediately stopped and should receive appropriate treatment (including use of resuscitation medications and equipment that must be available and used as clinically indicated). These patients should be withdrawn from treatment and should enter the Safety Follow-up Period.**

### 6.1.4 Criteria for Re-Treatment with Ocrelizumab

Prior to re-treatment with ocrelizumab, patients will be evaluated for the following conditions and laboratory abnormalities. If any of these are present prior to re-dosing, further administration of ocrelizumab should be suspended until resolved or held indefinitely:

- Life threatening (CTCAE Grade 4) infusion-related event that occurred during a previous ocrelizumab infusion
  - Any significant or uncontrolled medical condition or treatment-emergent, clinically significant laboratory abnormality
  - Active infection
  - Absolute neutrophil count < $1.5 \times 10^3/\mu L$
  - CD4 cell count < $250/\mu L$

- Hypogammaglobulinemia IgG < 3.3 g/L
- Ongoing pregnancy (for female patients)

Please note: any critical blinded laboratory values for IgG, absolute neutrophil count and CD4 will be provided to the Treating Investigator and the Medical Monitor. Investigators notified of their patient's critical laboratory test result will be instructed to suspend further treatment with study drug until the patient can be further evaluated. A repeat laboratory test may be necessary to confirm the results. Patients with values below these critical values should not be retreated until the re-treatment criteria are met and these laboratory values have normalized.

## 6.2 Rebif®

### 6.2.1 Dose and Schedule of Rebif®

Please refer to Table 10 for overview of Rebif® regimen.

The first subcutaneous injection of Rebif®/placebo will be administered on Study Day 1. Patients will be instructed by a nurse or Investigator how to self-administer the injections; the first dose of Rebif®/placebo will be self-administered under the supervision of a nurse or physician. Thereafter, patients will self-administer their Rebif®/placebo treatment three times weekly. Rebif®/placebo must be administered, if possible, at the same time (preferably in the late afternoon or evening) on the same 3 days (e.g., Monday, Wednesday, and Friday) at least 48 hours apart. Patients must be instructed in the use of aseptic techniques when administering Rebif®/placebo injections. Patient understanding and use of aseptic self-injection techniques and procedures must be periodically re-evaluated.

Since Rebif® needs to be stored at 2-8°C, it is recommended to remove the syringe from refrigerator at least 30 minutes prior to use. Patient should be reminded not to heat or microwave a syringe.

When starting treatment with Rebif®, the dose will be gradually escalated (please refer to Table 10). The Rebif®/placebo initiation package corresponds to the patient needs for the first month of treatment.

**Table 10: Overview of Rebif® Dosing Regimen ***

| | **Treatment Initiation** | | **Treatment Continuation** | **Dose modification (if required)** |
|---|---|---|---|---|
| **Week** | **Weeks 1- 2** | **Weeks 3-4** | **Week 5 onwards** | – |
| Study Day | 1-14 | 15-28 | 29+ | At any time >Day 29 |
| **Dose of Rebif®** | **Rebif® 8.8 µg** (1 pre-filled syringe [0.2 mL] containing 2.4 MIU of interferon β-1a) s.c. **3x per week** | **Rebif® 22 µg** (1 pre-filled syringe [0.5 mL] containing 6 MIU of interferon β-1a) s.c. **3x per week** | **Rebif® 44 µg** (1 pre-filled syringe [0.5 mL] containing 12 MIU of interferon β-1a) s.c. **3x per week** | **Rebif® 22 µg** (1 pre-filled syringe [0.5 mL] containing 6 MIU of interferon β-1a) s.c. **3x per week** |

* provided in blinded fashion

Non-steroid anti-inflammatory drugs (ibuprofen) or acetaminophen are recommended in case of injection site reaction; Investigator should follow local label for further information.

Note: During the OLE Phase Screening Period, administration of Rebif® / Rebif® placebo subcutanousely 3 times per week should occur. If during this period, the patient decides not to participate in the OLE Phase then administration of Rebif® / Rebif® placebo 3 times per week should be terminated.

### 6.2.2 Rebif® Dose Modifications, Interruptions and Delays

Rebif®/ Rebif® placebo should be taken three times a week. Rebif®/ Rebif® placebo should never be taken on two consecutive days. If a patient misses a dose, then the next dose must be taken as soon as possible. The patient should avoid taking Rebif®/ Rebif® placebo on the following day. The patient should return to their regular schedule the following week. If a patient takes more than the prescribed dose or takes it on 2 consecutive days, they should inform the Investigator immediately.

The Rebif®/Rebif® placebo dosage can be modified at the discretion of the Treating Investigator due to safety reasons at any time; the Treating Investigator should follow information on the local label for further information.

Asymptomatic increases in laboratory parameters of hepatic function have been associated with Rebif®.

In case of elevation of liver function tests the following rules will apply:

| | |
|---|---|
| – **ALT ≥ 10 × ULN** OR jaundice or other clinical symptoms of liver dysfunction | In case of detection of elevated **ALT ≥ 10 × ULN**, jaundice or other clinical symptoms of liver dysfunction the injections of Rebif®/Rebif® placebo must be discontinued permanently. The monitoring of liver function tests should be continued on a monthly basis until return to normal baseline levels or CTCAE v.4.0 Grade 1 toxicity (ALT: >ULN - 3.0 × ULN). A consultation with hepatologist is recommended. Patients should move to Safety Follow-Up Period. |
| – **ALT ≥ 5 × ULN** | In case of detection of elevated **ALT ≥ 5 × ULN** (but below 10 xULN) the injections of Rebif®/Rebif® placebo must be discontinued temporarily. Additional blood chemistry panel including AST, ALP, GGT, and bilirubin should be performed biweekly until no further increase is observed. Subsequently, ALT analysis has to be performed every month until return to normal baseline levels or CTCAE v.4.0 Grade 1 toxicity (ALT >ULN - 3.0 × ULN). A consultation with hepatologist should be considered as per Investigator judgment.<br>If causes of toxicity other than possible treatment with Rebif® are excluded, the patient may then be cautiously re-challenged with Rebif®/Rebif® placebo 22µg provided in a blinded fashion upon request to IxRS. The monitoring of liver function tests should continue on a monthly basis. If there is no further recurrence of toxicity, patient may continue treatment with |

| | |
|---|---|
| | Rebif®/Rebif® placebo 44 µg provided in a blinded fashion upon Investigator's request to IxRS. **In case of recurrence of toxicity (ALT > 3 xULN, or other clinical symptoms of liver dysfunction) the injections of Rebif®/Rebif® placebo should be discontinued permanently.** Patients should move to Safety Follow-Up Period.<br><br>Please note: Re-initiation of therapy with Rebif® following elevation of liver function tests can only be considered once. |
| – **ALT > 3 × ULN** | In case of detection of elevated **ALT > 3 × ULN** (but below 5 × ULN) additional blood chemistry panel including AST, ALP, GGT and bilirubin should be performed biweekly until no further increase is observed. Subsequently, ALT analysis has to be performed every month until return to normal baseline levels or CTCAE v.4.0 Grade 1 toxicity (ALT > ULN - 3.0 × ULN). |

## 6.3 Formulation, Packaging and Labeling

Study drug packaging will be overseen by the Roche clinical trial supplies department and bear a label with the identification required by local law, the protocol number, drug identification and dosage.

The packaging and labeling of the study medication will be in accordance with Roche standards and local regulations.

Upon arrival of investigational products at the site, site personnel should check them for damage and verify proper identity, quantity, integrity of seals and temperature conditions, and report any deviations or product complaints to the monitor upon discovery.

### 6.3.1 Ocrelizumab

**Formulation**

Ocrelizumab is manufactured as a sterile, clear, colorless, preservative free liquid intended for dilution for i.v. administration.

Ocrelizumab is supplied as a liquid formulation containing 30 mg/mL ocrelizumab in 20 mM sodium acetate at pH 5.3, with 4% (106 mM) trehalose dihydrate and 0.02% polysorbate 20. The drug product is provided as a single-use liquid formulation in a 15 cc Type I USP glass vial, fitted with a 20 mm fluoro-resin laminated stopper and an aluminum seal with a flip-off plastic cap and contains a nominal 200 mg ocrelizumab. No preservative is used as each vial is designed for single use.

Ocrelizumab-matching placebo is also supplied in 15 cc single-use vials. Placebo has the same composition and configuration as the drug product, but does not contain ocrelizumab.

**Packaging**

The hospital units/pharmacy will receive study medication kits for each patient.

For the double-blind treatment in Cycle 1, consisting of two 300 mg infusions 14 days apart, the study medication kit will contain 2 single-use liquid vials with ocrelizumab (or ocrelizumab placebo).

For each of the subsequent Cycles 2 to 4, consisting of a single 600 mg infusion, two kits will be dispensed. Each kit will contain 2 single-use liquid vials with ocrelizumab (or ocrelizumab placebo), from which only 3 vials should be used.

*For the OLE Phase, each study medication kit will contain 1 single-use vial. For OLE Phase Cycle 5, consisting of two 300 mg infusion 14 days apart, 2 study medication kits will be supplied per infusion. For OLE Phase Cycle 6 and each of the subsequent cycles consisting of 600 mg infusions, three study medication kits will be dispensed. Detailed instructions are provided separately in the Dose Preparation Guidelines.*

**Storage of Ocrelizumab and Placebo Vials for Infusion:**

Ocrelizumab and placebo vials are stable at 2-8°C (refrigerated storage). They should not be used beyond the expiration date stamped on the carton. Expiration dating may be extended during the trial; the Sponsor will provide documentation. Ocrelizumab vials should not be frozen or shaken and should be protected from direct sunlight.

The study medication labels will be produced in accordance with the local requirements.

### 6.3.2 Rebif®

**Formulation and packaging**

Rebif® (IFN β-1a) will be supplied as a liquid formulation for injection in pre-filled syringes.

The liquid formulation is supplied in syringes containing 0.2 mL or 0.5 mL of solution. These commercially available syringes will be provided to the sites by the Sponsor and re-labeled as investigational medicinal product.

The placebo to Rebif® is provided as a liquid formulation in a pre-filled syringe containing 0.2 mL or 0.5 mL of 0.9% sodium chloride solution without any active substance.

The study medication kits, which will be used for the initial 4 weeks of treatment, will contain 12 pre-filled syringes, either 6x 8.8 µg and 6x 22 µg OR placebo. The study medication kits that will be used for treatment continuation will contain 12 pre-filled syringes 12x44 µg of IFN β-1a or 12x22 µg of IFN β-1a or placebo

The Rebif® and Rebif® placebo pre-filled syringes are for subcutaneous use only.

Please note: if Rebif® dose modification is required due to lab abnormalities possibly related to the treatment with Rebif®, the Investigator (the treating physician) will need to notify IxRS and the blinded study medication (Rebif® placebo or Rebif® verum) will be dispensed accordingly. In addition, to ensure patient safety in the study, unscheduled visits may be required for additional assessments, monitoring and for dispensing study medication.

The study medication labels will be produced in accordance with the local requirements. The strength will be presented as follows: 44 µg / 22 µg / placebo.

**Storage of Rebif®**

Rebif®/Rebif® placebo pre-filled syringes need to be stored in a refrigerator at 2-8°C, in the original package in order to protect from light. The patient may remove Rebif® from the refrigerator and store it not above 25°C for one single period of up to 14 days. Rebif® must then be returned to the refrigerator and used before the expiry date.

## 6.4 Blinding and Unblinding

The Patient Randomization List will be generated by IxRS using a pre-defined randomization specification. The Randomization List will not be available at the study center, to the Roche monitors, project Statisticians or to the Sponsor's project team. Unblinding of treatment assignment should not occur except in the case of emergency situations, where the knowledge of what study medication the patient is receiving is critical for clinical management. Treating Investigators are asked to contact the Roche Medical Monitor, prior to unblinding any patient, in order to discuss the medical necessity for unblinding. Any request from the Investigator for information about the treatment administered to study patients for another purpose must be discussed with Roche. Unblinding will be performed by means of an Interactive Web Response System (IxRS). As per regulatory reporting requirement, Roche will unblind the identity of the study medication for serious adverse events that are considered by the Investigator or the Sponsor to be related to study drug, that are unexpected as per safety reference document(s), e.g., IB, CDS, and SPC, and that are not exempted from unblinding as per Section 7.2.2.2. Details of patients who are unblinded during the study will be included in the Clinical Study Report.

Unblinding for analysis of biological samples, *pharmacokinetic data analysis,* or ongoing safety monitoring by a Data Monitoring Committee [DMC], will be performed according to procedures in place to ensure integrity of the data.

*Patient treatment allocation during the double-blind, double-dummy treatment period will not be unblinded until the the last data point from the last patient is received, as required for statistical analysis defined in Section 8.2 and the SAP* (see *also* Section 3.1.3).

## 6.5 Accountability of Investigational Medicinal Product and Assessment of Compliance

### 6.5.1 Accountability of Investigational Medicinal Product

The Investigator is responsible for the control of drugs under investigation. Adequate records for the receipt and disposition of the study drug must be maintained. Accountability will be assessed by maintaining adequate drug dispensing and return records.

Accurate records must be kept for each study drug provided by the Sponsor. These records must contain the following:

- Documentation of drug shipments received from the Sponsor (date received and quantity)
- Disposition of unused study drug not dispensed to patient.

A Drug Dispensing Log must be kept current and should contain the following information:

- the identification of the patient to whom the study medication was dispensed
- the date[s] and quantity of the study medication dispensed *to* the patient
- the date[s] and quantity of the study medication returned *by* the patient.

All records and drug supplies must be available for inspection/accountability by the Monitor at every monitoring visit.

#### 6.5.2 Assessment of Compliance

Patient compliance will be assessed by maintaining adequate study drug dispensing records. The Investigator is responsible for ensuring that dosing is administered in compliance with the protocol. Delegation of this task must be clearly documented and approved by the Investigator.

The study pharmacist should keep all ocrelizumab/ocrelizumab placebo vials to measure compliance. All patients will be asked to return on regular intervals all used and unused Rebif®/Rebif® placebo containers to the site as a measure of compliance.

### 6.6 Destruction of the Investigational Medicinal Product/Comparator

Local or institutional regulations may require immediate destruction of used IMP for safety reasons. In these cases, it may be acceptable for investigational site staff to destroy dispensed IMP before a monitoring inspection provided that source document verification is performed on the remaining inventory and reconciled against the documentation of quantity shipped, dispensed, returned and destroyed. Written authorization must be obtained from the Sponsor at study start up before destruction.

Written documentation of destruction must contain the following:

- Identity (batch numbers or medication numbers) of IMP and comparators destroyed
- Quantity of IMP destroyed
- Date of destruction
- Method of destruction
- Name and signature of responsible person who destroyed the IMP.

Wherever possible, preferably drug should be destroyed locally on site according to their local policies and procedures once drug accountability has been completed by the monitor.

# 7. SAFETY INSTRUCTIONS AND GUIDANCE

## 7.1 Adverse Events and Laboratory Abnormalities

### 7.1.1 Clinical Adverse Events

According to the International Conference of Harmonisation (ICH), an AE is any untoward medical occurrence in a patient or clinical investigation patient administered a pharmaceutical product and which does not necessarily have a causal relationship with this treatment. An AE can therefore be any unfavorable and unintended sign, including an abnormal laboratory finding, symptom, or disease temporally associated with the use of a (investigational) medicinal product, whether or not considered related to the medicinal (investigational) product. Pre-existing conditions which worsen during a study are to be reported as AEs.

In the eCRF, adverse events will be reported at each visit.

**Clinical relapses** will be recorded only on a pre-specified eCRF "MS relapse" eform.

Infusion-related reactions will be recorded only on a pre-specified eCRF "Infusion Related Reaction" eform.

B-cell depletion is the expected outcome of ocrelizumab treatment and is not an adverse event. However, patients may be at risk for infections and particular attention should be directed toward early identification and treatment of infections. During the study, Investigators are requested to promptly investigate patients reporting signs or symptoms of infection, to take appropriate specimens for identification of the pathogen and to treat infections aggressively (see Section 7.3.1). Prior to enrollment into the study, it is recommended that the Investigators review and, if warranted, update patient's immunizations in accordance with country medical immunization guidelines (see also Section 4.5.3).

#### *7.1.1.1 Intensity of Clinical Adverse Events*

Adverse events will be graded according to CTCAE, version 4 and is provided to the investigator in a separate handout entitled "Common Terminology Criteria for Adverse Events v4.0"- see Appendix 3.

Adverse events not listed by the CTCAE *and IRRs as a comprehensive entity* will be graded using the following criteria:

Grade 1: Discomfort noticed but no disruption of normal daily activity

Grade 2: Discomfort sufficient to reduce or affect normal daily activity

Grade 3: Inability to work or perform normal daily activity

Grade 4: Represents an immediate threat to life.

Any Grade 4 adverse event, either by CTCAE criteria or the additional criteria listed *in Section 7.1.1.3*, should be reported as an SAE.

#### *7.1.1.2 Drug - Adverse Event Relationship*

**Relationship** of the adverse event to the treatment should always be assessed by the Investigator. The causality relationship of study drug to the adverse event will be assessed by the Investigator as either: Yes or No. Please refer to Appendix 1 for more details.

#### *7.1.1.3 Serious Adverse Events (Immediately Reportable to Sponsor)*

A Serious Adverse Event is any experience that suggests a significant hazard, contraindication, side effect or precaution. It is any adverse event that, at any dose, fulfils at least one of the following criteria:

- is fatal; (results in **death***; please note: death is an outcome, not an event)
- is Life-Threatening (please note: the term "Life-Threatening" refers to an event in which the patient was at immediate risk of death at the time of the event; it does not refer to an event which could hypothetically have caused a death had it been more severe).
- required in-patient hospitalization or prolongation of existing hospitalization;
- results in persistent or significant disability/incapacity;
- is a congenital anomaly/birth defect;
- is medically significant or requires intervention to prevent one or other of the outcomes listed above.

***The term sudden death should only be used when the cause is of a cardiac origin as per standard definition. The terms death and sudden death are clearly distinct and must not be used interchangeably**.

The exception to this definition of an SAE is in the rare event that a patient is hospitalized following an MS relapse, as long as the reason for hospitalization is to receive standard treatment with i.v. methylprednisolone. The rationale for this exception is that some countries and/or clinical sites routinely hospitalize patients who require administration of methylprednisolone in the event of an MS relapse. Thus, the serious adverse event criteria for "hospitalization" would be met on the basis of local practice and would not reflect the seriousness of the event.

When the MS relapse results in hospitalization for any reason other than for routine treatment of the relapse (such as for a treatment course beyond the standard treatment described in (see Section 4.5.2) or when hospitalization is prolonged, the MS relapse should be considered a serious adverse event.

The study will comply with all local regulatory requirements and will adhere to the full requirements of the ICH Guideline for Clinical Safety Data Management, Definitions and Standards for Expedited Reporting, Topic E2 (see Appendix 2).

### 7.1.2 Treatment and Follow-up of Adverse Events

Adverse events should be followed up until they have stabilized or have returned to baseline status (in the event of an exacerbation of a pre-existing condition). This is

especially important for those events where the reported causal relationship to study medication(s) is “related”. If a clear explanation is established, it should be recorded on the eCRF.

If after study completion or withdrawal, return to baseline status or stabilization cannot be established an explanation should be recorded on the eCRF.

### 7.1.3 Laboratory Test Abnormalities

Laboratory test results will appear on electronically produced laboratory reports submitted directly from the central laboratory.

Any treatment-emergent abnormal laboratory result which is clinically significant, i.e., meeting one or more of the following conditions, should be recorded as a single diagnosis on the adverse event eform in the eCRF:

- Accompanied by clinical symptoms,
- Leading to a change in study medication (e.g., dose modification, interruption or permanent discontinuation),
- Requiring a change in concomitant therapy (e.g., addition of, interruption of, discontinuation of, or any other change in a concomitant medication, therapy or treatment).

Any laboratory result abnormality fulfilling the criteria for an SAE should be reported as such, in addition to being recorded as an AE in the eCRF.

#### *7.1.3.1 Follow-up of Abnormal Laboratory Test Values*

In the event of medically significant unexplained abnormal laboratory test values, the tests should be repeated and followed up until they have returned to the normal range and/or an adequate explanation of the abnormality is found. If a clear explanation is established it should be recorded on the eCRF.

B-cell depletion is a pharmacodynamic effect and is not an adverse event.

During the double-blind, dummy-dummy treatment period, blinded laboratory values for IgG, absolute neutrophil count and CD4 will be provided to the Investigator and the Medical Monitor. Investigators notified of their patient’s critical laboratory test result will be instructed to suspend further treatment with study drug until the patient can be further evaluated. A repeat laboratory test may be necessary to confirm the results. Patients with values below these critical values should not be re-treated until the re-treatment criteria are met (see Section 6.1.4) and these laboratory values have normalized. During the OLE Phase from Cycle 6 onward, these laboratory values will not be blinded.

## 7.2 Handling of Safety Parameters

### 7.2.1 Reporting of Adverse Events

All adverse events will be documented in the eCRF.

New or worsening neurological symptoms not considered MS-related should be recorded on an AE page and the monitor should be informed.

### 7.2.2 Reporting of Serious Adverse Events

#### *7.2.2.1 Immediate Reporting to the Sponsor*

Any clinical adverse event or abnormal laboratory test value that is serious (as defined in Section 7.1.1.3), which occurs during the course of the study, regardless of the treatment group, must be reported to the Sponsor **within 24 hours** of the Investigator becoming aware of the event (expedited reporting). In addition, for fatal and life-threatening events, the Roche Medical Monitor should be contacted immediately. Contact numbers for the Roche Medical Monitor (including after hours cover) will be provided to the site before any patients are screened.

After the patient signs the Informed Consent, but prior to initiation of study medication, only serious adverse events caused by a protocol-mandated intervention will be collected (e.g., serious adverse events related to MRI exam). After first dose of study medication, all serious adverse events must be reported.

Related serious adverse events **MUST** be collected and reported regardless of the time elapsed from the last study drug administration, even if the study has been closed.

Unrelated serious adverse events must be collected and reported during the study through the end of the Safety Follow-up Period, which is at least 48 weeks after the last infusion but may be extended in patients whose B-cells take longer to replete.

The Investigator must complete the serious adverse event reporting form in the eCRF. Relevant follow-up information should be submitted as soon as it becomes available. Only if a technical failure prevents the ability to report a serious adverse event in the eCRF, then the paper serious adverse event Reporting Form provided by the Sponsor must be completed and faxed to the number provided.

A death occurring during the study or information related to such occurrence that comes to the attention of the Investigator during the study must be reported immediately to the Sponsor, whether considered treatment-related or not.

The following are not considered as a serious adverse event:

- Elective hospitalizations or surgical procedures that are a result of a patient's pre-existing condition(s) that have not worsened since receiving trial medication. Examples may include, but are not limited to, cholecystectomy for gallstones, and diagnostic testing. Such events should still be recorded as medical procedures in the eCRF.
- Hospitalization to receive trial medication such as infusions of ocrelizumab unless this is prolonged (more than 24 hours).
- Hospitalization following an MS relapse as long as the reason for hospitalization is to receive standard treatment with i.v. methylprednisolone

Of specific importance is the prompt reporting of serious infections. In particular, PML should be reported as a serious adverse event (with all available information) with immediate notification of the Medical Monitor.

This study adheres to the definition and reporting requirements of ICH Guideline for Clinical Safety Data Management, Definitions, and Standards for Expedited Reporting, Topic E2 (see Appendix 2).

#### 7.2.2.2 *Emergency Medical Contacts*

To ensure the safety of the study patients, an Emergency Medical Call Center Help Desk will access the Roche Medical Emergency List, escalate emergency medical calls, provide medical translation services (if necessary), connect the Investigator with a Roche Medical Monitor, and track all calls. The Emergency Medical Call Center Help Desk will be available 24 hours per day, 7 days per week. Toll-free numbers for the Help Desk and Medical Monitor contact information will be distributed to all Investigators (see "Protocol Administrative and Contact Information and List of Investigators").

#### 7.2.2.3 *Expedited Reporting to Health Authorities, Investigators, Institutional Review Boards, and Ethics Committees*

The Sponsor will promptly evaluate all reported serious adverse events against cumulative product experience to identify and expeditiously communicate possible new safety findings to Investigators, IRBs, ECs, and relevant health authorities based on applicable legislation.

Reporting requirements will be based on the Investigator's assessment of causality and seriousness, with allowance for upgrading by the Sponsor as needed. To determine reporting requirements for single adverse event cases, the Sponsor will also assess the expectedness of the event on the basis of the Investigator's Brochure.

In principle, adverse events which are serious, related and unexpected will be reported in an expedited manner within 15 days (non-fatal/non-life-threatening) or 7 days (fatal or life-threatening).

Only those adverse events qualifying for expedited reporting occurring in patients on active treatment will be sent in an expedited timeframe to Health Authorities. This requires unblinding of patient treatment allocation. Investigators, Institutional Review Boards, and Ethics Committees will receive blinded reports unless local regulations require that unblinded expedited reports are sent.

For certain types of adverse events, the relation to study medication cannot be assessed based on single case evaluation. Therefore, in order to prevent unnecessary unblinding of study participants, the following events are exempted from expedited reporting:

– Neoplasms benign, malignant, and unspecified (including cysts and polyps),

– Infections and infestations with the exception of opportunistic infections (including PML and reactivation of viral infections).

The DMC will review adverse events at *periodic* meetings and assess their relation to study medication based on review of aggregate unblinded safety information. *The DMC will continue safety monitoring during the OLE phase.*

### 7.2.3 Pregnancy and Lactation

Female patients should take all appropriate precautions to avoid becoming pregnant during this study and for the entire duration of B-cell depletion. As such, women of childbearing potential should use adequate contraception for the duration of the trial and for 48 weeks after receiving their last infusion of ocrelizumab, or until their B-cells have repleted whichever is the longer. Regular pregnancy tests will be performed during the study. In case of an ongoing pregnancy during the study, no further infusions of ocrelizumab should be administered.

Effects on pregnancies from the female partners of B-cell depleted males have not been studied. Therefore, it is required that male patients also use reliable contraception while receiving ocrelizumab treatment in this study for 24 weeks after receiving their last infusion of ocrelizumab.

Reproductive toxicology studies of ocrelizumab conducted in cynomolgus monkeys are described in the IB. Studies of the effect of ocrelizumab on human reproduction have not been performed. It is not known whether ocrelizumab can cause fetal harm when administered to pregnant women or whether it can affect reproductive capacity. However, since IgG molecules such as ocrelizumab are known to cross the placenta, ocrelizumab may cause fetal CD20 B-cell depletion. It is not known whether ocrelizumab is excreted in breast milk, and what effect this might have on the breast feeding infant. However, since immunoglobulins are found in breast milk, breast feeding mothers are excluded from participation in the study.

Regardless of the treatment assignment, a female patient must be instructed to immediately inform the Investigator if she becomes pregnant during the study (including the Safety Follow-Up Period). In case of pregnancy, patient must be withdrawn from treatment and she should enter the Safety Follow-Up Period. The Investigator should report all pregnancies within 24 hours to the Sponsor by means of an eCRF Pregnancy Reporting Form.

As ocrelizumab may cross the placenta and cause B-cell depletion in the neonate, babies born to mothers participating in this study should have an assessment of their lymphocyte counts and be carefully followed until these are within the normal range for the age of the infant. The Investigator should counsel the patient as to the risks of continuing with the pregnancy and the possible effects on the fetus. Monitoring of the patient should continue until conclusion of the pregnancy. Informed consent will be sought for the Sponsor to collect information on the health and well being of the baby.

Whether the drug is excreted in the semen is unknown. Therefore, pregnancy occurring in the partner of a male patient participating in the study should also be reported to the Investigator, and the Investigator should inform the Sponsor. If appropriate, an additional consent form will be provided (subject to ethics committee review) to solicit information about the pregnancy.

## 7.3 Warnings and Precautions

### 7.3.1 Ocrelizumab

Patients should be informed of the risks associated with taking ocrelizumab. Below are listed specific major risks of which the patients should be made aware. Further information on ocrelizumab is given in the current version of IB.

**Infusion-Related Reactions**

All CD20 depleting agents including ocrelizumab have been associated with acute infusion-related reactions (fever, urticaria/rash, chills, rigors, sneezing, angioneurotic edema, throat irritation, nausea, fatigue, headache, dyspnea, rhinitis, vomiting, or flushing cough and bronchospasm, with or without associated hypotension or hypertension). Some of these events have been severe enough to warrant interruption or discontinuation of the infusion. Symptoms are often reversible if the infusion is interrupted and/or patients receive additional treatment with an antihistaminic, acetaminophen, epinephrine or an i.v. corticosteroid. Please refer to Sections 6.1.2 and 6.1.3 for further information.

**Infection Risks**

Prolonged peripheral B-cell depletion is the expected outcome of ocrelizumab treatment. Infection is a potentially serious complication of B-cell depleting therapy and thus requires vigilant attention and prompt investigation and treatment in patients that exhibit signs of infection at any time following anti-CD20 antibody therapy.

Data on the long term risk of infection in MS patients treated with ocrelizumab are not available at this time however in review of the Phase II data in patients with RRMS no imbalance in the overall number of infections or serious infections between placebo and active ocrelizumab arms was observed at week 24. The rate of infections did not increase in ocrelizumab-treated patients at 96 weeks compared with 24 weeks. There was no trend of increase of risk of infection or serious infection for ocrelizumab treated patients with previous interferon treatment (Avonex® for 6 months). There was no trend of increase risk of infections or serious infections with high dose. Please see Section 1.1.4 and the ocrelizumab IB for details.

Rarely, cases of hepatitis B reactivation, including fulminant hepatitis which have occasionally been fatal, have been reported in NHL patients receiving rituximab. A case of hepatitis B reactivation in a Hep B core Ab positive patient with RA treated with ocrelizumab has been reported (see Section 5.4.2 for hepatitis screening and monitoring of liver function).

Other serious, opportunistic and fatal infections have occurred in patients with lupus and RA treated with ocrelizumab in Phase III clinical trials. Data from completed studies regarding infection risks with ocrelizumab treatment in these patient populations are provided in the Investigator Brochure (IB).

Ocrelizumab should not be administered to patients with an active infection. Physicians should exercise caution when considering the use of ocrelizumab in patients with underlying conditions that may predispose patients to serious infection. Patients who develop signs/symptoms of infection while participating in this trial should be seen

immediately, samples taken for appropriate microbiological analysis and appropriate treatment instituted promptly.

Patients should be screened for tuberculosis according to national guidelines. As with other infections, patients with active tuberculosis should not be enrolled, patients with latent tuberculosis should be treated prior to enrollment.

Patients should be warned that the risk of serious infection may be increased by exposure to the medications to be used in this study and should be asked to contact the clinic staff if they start to develop signs of infection. Patients will be provided with a warning card which specifically delineates this risk, which is to be carried on their person at all times in case they are admitted to a hospital which is not participating in the study.

Please refer to the ocrelizumab IB for further information on infection risks.

**Prolonged B-cell Depletion**

In patients with RA that were treated with rituximab, prolonged peripheral B-cell depletion has been reported up to 4 years following a single course of therapy. It is not known whether this will occur following use of ocrelizumab. Patients with prolonged B-cell depletion should be monitored until their B-cells have repleted (see Section 3.1).

**Progressive multifocal leukoencephalopathy**

To date there have been no confirmed cases of PML in any MS patient treated with either rituximab or ocrelizumab. No confirmed cases of PML have been observed in any patient receiving ocrelizumab, for any indication.

Among patients treated with rituximab, cases of PML have been observed, in oncology and other autoimmune diseases. The vast majority of these cases have occurred in patients being treated for hematological malignancy.

There is no currently accepted screening test for PML, nor are there known interventions that can reliably prevent PML or adequately treat PML. See also section 7.3.4 for more details. Guidance for diagnosis is given in Section 7.3.4.1.

**Cardiovascular Disorders**

Rarely, cardiac arrhythmias, cardiac ischemia and death due to myocardial dysfunction have been associated with rituximab administration in patients with oncologic disorders. In these cases, the presumed cause was decompensated cardiac disease as a result of cytokine release and/or infusion associated reactions. Patients with a history of cardiac disease (i.e., angina pectoris, cardiac arrhythmias, or congestive heart failure) should be monitored closely during and following infusions. It should be noted that the exclusion criteria exclude enrollment of patients with significant cardiac diseases and congestive heart failure (NYHA III or IV) – see Section 4.3.

**Immunogenicity**

Positive HAHA responses were observed and were most frequent in the lower dose groups in both RA Phase I/II studies; no HAHA responses were observed in the NHL study. In Study ACT2847g, which included doses of 10 mg × 2, 50 mg × 2, 200 mg × 2,

500 mg×2, and 1000 mg × 2, HAHA was observed in 19% and 10% of patients receiving 10 mg × 2 and 50 mg × 2, respectively, versus 0% to 5% of patients receiving 200−1000 mg × 2. In Study WA18230, which included doses of 400, 1000, 1500, and 2000 mg, HAHA was observed in 10% and 5% of patients receiving 400 mg and 1000 mg, respectively, and in none of the patients receiving 1500 mg and 2000 mg.

In the RRMS Phase II study (WA21493), no new HAHA seropositivity occurred after initiation of ocrelizumab treatment (300 mg × 2 or 1000 mg × 2).

The clinical significance of positive HAHA is unknown at this time.

**Immunization**

The effect of ocrelizumab on the response to immunization is not known – please refer to Section 4.5.3 for more details; patients receiving ocrelizumab may not mount a humoral response to recall antigens during B-cell depletion. Physicians should review the patient's vaccine history, and be aware that immune response to vaccination could be reduced. Current administration of live vaccines during the Treatment Period and thereafter when B-cells remain depleted is not allowed.

### 7.3.2 Rebif®

Patients should be informed of the risks associated with taking Rebif®. The most frequent Rebif® adverse reactions of which the patients should be made aware have been summarized in Section 1.1.5.

Depression and suicide ideation are known to occur in increased frequency in the MS and in association with interferon use. Therefore all patients should be advised to immediately report any symptoms of depression and/or suicidal ideation to Investigator. Patients exhibiting depression should be monitored closely and treated appropriately. Cessation of double-blind treatment should be considered.

Injection site necrosis has been reported in patients using Rebif®. To minimize the risk of injection site necrosis patients should be advised to:

- use an aseptic injection technique,
- rotate the injection sites with each dose.

If the patient experiences any break in the skin, which may be associated with swelling or drainage of fluid from the injection site, the patient should be advised to consult with their physician before continuing injections with Rebif®/ Rebif® placebo. If the patient has multiple lesions, injections should be discontinued until healing has occurred. Patients with single lesions may continue provided that the necrosis is not too extensive.

Rebif®, like other interferons β, has a potential for causing severe liver injury including acute hepatic failure. The mechanism for the rare symptomatic hepatic dysfunction is not known. No specific risk factors have been identified. Please refer to Section 6.2.2 for additional guidelines.

### 7.3.3 Corticosteroids

Systemic corticosteroids, such as methylprednisolone, can cause immunosuppression, hypertension, diabetes mellitus, cataract, glaucoma, bruising, thinning of the skin, weight gain, psychological changes including psychosis, osteoporosis, accelerated atherosclerosis, increased risk of gastrointestinal bleeding, aseptic necrosis of bone and adrenal insufficiency. Although rare, corticosteroid induced hypersensitivity reactions may occur. They range from minor rashes to the more serious cardiovascular collapse. For additional safety data, refer to the local prescribing information.

### 7.3.4 Progressive Multifocal Leukoencephalopathy

Progressive multifocal leukoencephalopathy (PML) is a potentially fatal neurological condition linked to reactivation of a polyomavirus (JC virus) and active viral replication in the brain. Polyomavirus infection is acquired in childhood and up to 80% of adults demonstrate serological evidence of past infection. Reactivation of JC virus replication with transient viremia or viruria unassociated with clinical symptoms may occur spontaneously in healthy persons. Less frequently, central nervous system symptoms associated with active viral replication in brain tissue is observed. The clinical syndrome is significantly more frequent among immune suppressed patients.

To date there have been no confirmed cases of PML in any MS patient treated with either rituximab or ocrelizumab and no confirmed cases in any patient taking ocrelizumab for any indication. Cases of PML have been reported in patients receiving rituximab in oncology and other autoimmune indications. The vast majority of these cases have occurred in patients being treated for hematological malignancy and many of these patients were also HIV positive. The majority of these patients received rituximab in combination with chemotherapy or as part of a haematopoietic stem cell transplant.

Physicians should consider the diagnosis of PML in any patient presenting with new and/or progressive neurological deficits localized to the cerebral cortex, such as cortical symptoms/signs, behavioral and neuropsychological alteration, retrochiasmal visual defects, hemiparesis, cerebellar symptoms/signs (e.g., gait abnormalities, limb incoordination), at each visit.

If PML is considered, a neurological consultation should be obtained and treatment suspended until PML has been ruled out. If PML is confirmed in a patient receiving ocrelizumab, no further infusions should be administered and the patient will be withdrawn from treatment (see Section 4.6). No known interventions can reliably prevent PML or adequately treat PML, if it occurs.

It is not known whether the risk of PML is altered by anti-CD20 treatment given as monotherapy. Please refer to Section 7.3.4.1 for guidance on the diagnosis of PML.

PML should be reported as an SAE (with all available information) with immediate notification of the Medical Monitor. Study drug should be withheld and patients with confirmed PML should *enter the safety follow-up phase of* the study.

There is no known treatment or cure for PML. Treatment considerations are discussed in the medical literature [66].

#### *7.3.4.1 Guidance for Diagnosis of Progressive Multifocal Leukoencephalopathy*

The following safety monitoring algorithm (Figure 3) will be implemented in this study. This algorithm was implemented in the Phase I/II studies with rituximab in patients with MS and is consistent with the algorithm used in natalizumab studies.

Comprehensive neurological assessments will be performed every 12 weeks at the regular study visits. Patients will be required to undergo a neurological exam for calculation of an Expanded Disability Status Scale (EDSS) score every 12 weeks. This requires that Functional System Score (FSS) also be determined. The examination to calculate the FSS includes cognitive, visual and motor assessments, the neurological systems most often affected by PML, as well as assessments of other neurological systems.

In the eCRF, the Investigator will record the presence or absence of neurological deficits localized to the cerebral cortex (e.g., cortical symptoms/signs, behavioral and neuropsychological alteration, retrochiasmal visual defects, hemiparesis), cerebellar symptoms/signs (e.g., gait abnormalities, limb incoordination), at each visit. Presence of such neurological findings will be recorded as adverse events. If a diagnosis for the deficits is identified, the symptoms should be replaced by the diagnosis in the adverse event eCRF.

In addition to the neurological evaluation at regular visits, patients will undergo a telephone interview between the study visits by site personnel familiar with the patient(s). The purpose of this interview is to identify new or worsening neurological symptoms that warrant an unscheduled visit (Appendix 4). Partners or caregivers of study patients, if applicable, will be informed on symptoms and signs that may be suggestive of PML and should be instructed to contact the site, should any such signs or symptoms appear.

In the event that new or worsening neurological symptoms are considered during the telephone interview, a neurological evaluation will be conducted. Should a non MS etiology, such as PML, be considered, further assessments should be done. The evaluation of PML may include a brain MRI scan and CSF analysis per the proposed treatment algorithm (see Figure 3).

**<u>The following clinical guidance is provided:</u>**

**Treatment of Relapse and Other Neurological Symptoms**

- As in all MS studies, new or recurrent neurological symptoms occurring in study patients should prompt careful clinical evaluation.
- Given the occurrence of PML in immunocompromised patients who had received rituximab, PML should be considered in patients who develop worsening neurological signs or symptoms.
- There are no pathognomonic signs or symptoms that distinguish MS from PML, but there are certain clinical features that may help differentiate between the two conditions (see Table 12).
- In addition to PML and MS, other CNS conditions (e.g., stroke, migraine, etc.) should be considered when evaluating a patient with new neurological changes.

- Relapses should be managed according to the study protocol.
- Corticosteroid treatment should only be considered for cases in which PML is unlikely on clinical grounds and when the severity of the relapse warrants such treatment. Lack of response to corticosteroids should trigger further investigation.

**Action Steps if PML is Suspected**

- If the clinical presentation is suggestive of PML, further investigations should include brain MRI evaluation as soon as possible. If MRI evaluation reveals lesions suspicious for PML (see Figure 3) a lumbar puncture with evaluation of the cerebrospinal fluid (CSF) for the detection of JCV DNA should be undertaken. A diagnosis of PML can potentially be made by evaluating clinical and MRI findings plus the identification of JCV in the CSF.

Please note: In the event that PML is suspected, an additional plasma, urine, as well as a CSF sample should be obtained for JCV analysis. CSF samples will be analyzed upon receipt and the results will be provided directly to the investigational site and to the Sponsor. The additional plasma and urine samples will be stored together with the routine JCV samples. Storage conditions and shipment instructions will be provided.

**MRI Assessment**

- Although there are no pathognomonic findings that differentiate PML from MS, a brain MRI scan that includes fluid-attenuated inversion recovery (FLAIR) and T2-weighted and T1-weighted sequences, with and without Gd, should be performed to assess patients with neurological changes suggestive of PML – see Figure 3.
- Comparison with a baseline scan may assist with interpretation of the findings on the newly acquired MRI (see Table 12 for differences in lesion characteristics that may help differentiate between PML and MS).

**CSF Assessment**

- The detection of JCV DNA in the CSF of a patient with clinical and MRI features suggestive of PML establishes the diagnosis of PML.
- If JCV DNA is not detected in CSF and if clinical suspicion of PML remains high, a repeat lumbar puncture should be performed.
- If diagnosis remains uncertain and suspicion of PML remains high, a brain biopsy may be considered to establish a definitive diagnosis.

**Figure 3: Diagnostic Algorithm for PML**

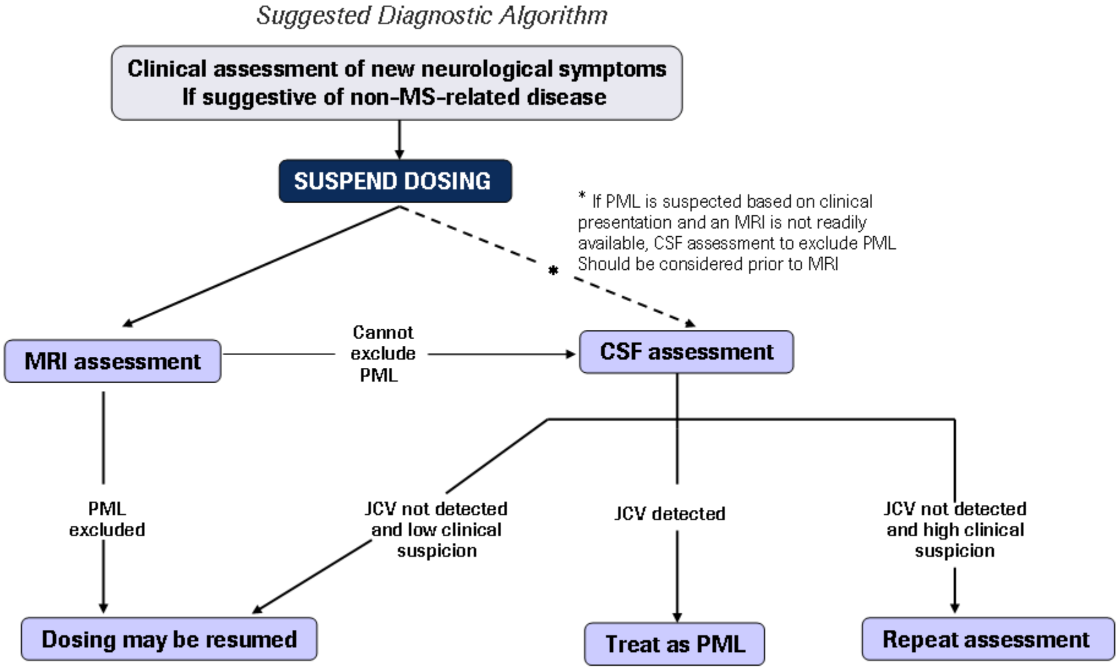


**Table 11: Clinical features to distinguish between MS relapse and PML***

| | MS relapse | PML |
|---|---|---|
| **Onset** | Acute | Subacute |
| **Evolution** | - Over hours to days<br>- Normally stabilizes<br>- Resolves spontaneously or with treatment | - Over weeks<br>- Progressive |
| **Clinical presentation** | - Optic neuritis<br>- Incomplete myelopathy or partial myelitis | - Cortical signs and symptoms<br>- Behavioral and neuropsychological alterations<br>- Retrochiasmal visual deficits<br>- Hemiparesis |

*Adapted from Kappos L et al [67]

## Table 12: MRI Lesion Characteristics Typical of PML and MS

| Feature | MS (relapse) | PML |
|---|---|---|
| **Location of new lesions** | Mostly focal; affect entire brain and spinal chord, in white and possibly gray matter | Diffuse lesions, mainly subcortical and rarely periventricular, located almost exclusively in white matter, although occasional extension to gray matter has been seen; posterior fossa frequently involved (cerebellum) |
| **Borders** | Sharp edges; mostly round or finger-like in shape (especially periventricular lesions), confluent with other lesions; U-fibers may be involved | Ill-defined edges; irregular in shape; confined to white matter; sparing gray matter; pushing against the cerebral cortex; U-fibers destroyed |
| **Mode of extension** | Initially focal; lesions enlarge within days or weeks and later decrease in size within months | Lesions are diffuse and asymmetric, extending homogeneously; no confluence with other lesions; confined to white-matter tracks, sparing the cortex; continuous progression |
| **Mass effect** | Acute lesions show some mass effect | No mass effect even in large lesions (but lesion slightly abuts cerebral cortex) |
| **On T2-weighted sequence** | - Acute lesions: hyperintense center, isointense ring, discrete hyperintensity outside the ring structure<br>- Subacute and chronic lesions: hyperintense with no ring structure | Diffuse hyperintensity, slightly increased intensity of newly involved areas compared with old areas, little irregular signal intensity of lesions |
| **On T1-weighted sequence** | Acute lesions: densely hypointense (large lesions) or isointense (small lesions); increasing signal intensity over time in 80%; decreasing signal intensity (axonal loss) in about 20% | Slightly hypointense at onset, with signal intensity decreasing over time and along the affected area; no reversion of signal intensity |
| **On FLAIR sequence** | Hyperintense, sharply delineated | Hyperintensity more obvious; true extension of abnormality more clearly visible than in T2-weighted images |
| **With enhancement** | - Acute lesions: dense homogeneous enhancement, sharp edges<br>- Subacute lesions: ring enhancement<br>- Chronic lesions: no enhancement | Usually no enhancement, even in large lesions; in patients with HIV, some peripheral enhancement is possible, especially under therapy. |
| **Atrophy** | Focal atrophy possible due to focal white-matter degeneration; no progression | No focal atrophy |

Adapted from Yousry TA et al [68]

# 8. STATISTICAL CONSIDERATIONS AND ANALYTICAL PLAN

Full details of all statistical issues and planned statistical analyses will be specified in a separate *Statistical* Analysis Plan (*S*AP), which will be finalized prior to the locking and unblinding of the study database.

## 8.1 Study Endpoints

### 8.1.1 Primary Efficacy Endpoint

The primary efficacy endpoint is annualized protocol-defined relapse rate by two years (96 weeks).

Protocol-defined relapse is defined as the occurrence of new or worsening neurological symptoms attributable to MS. Symptoms must persist for >24 hours and should not be attributable to confounding clinical factors (e.g., fever, infection, injury, adverse reactions to medications) and immediately preceded by a stable or improving neurological state for least 30 days. The new or worsening neurological symptoms must be accompanied by objective neurological worsening consistent with an increase of at least half a step on the EDSS scale, or 2 points on one of the appropriate FSS, or 1 point on two or more of the appropriate FSS. The change must affect the selected FSS (i.e., pyramidal, ambulation, cerebellar, brainstem, sensory, or visual). Episodic spasms, sexual dysfunction, fatigue, mood change or bladder or bowel urgency or incontinence will not suffice to establish a relapse. Please note: Sexual dysfunction and Fatigue will not be scored.

Adjudication of protocol-defined relapses will be performed by the Sponsor based on pre-specified criteria, applied to data collected by Investigator, in a blinded fashion.

### 8.1.2 Secondary Efficacy Endpoints

The *key* secondary efficacy endpoints are:

- The time to onset of *confirmed* disability progression for at least 12 weeks *with the* initial event of neurological worsening occur*ring* during the 96-week, double-blind, double-dummy, treatment period (see Section 5.3.2.2 for the definition of *confirmed* disability progression).
- *The total number of T1 Gd-enhancing lesions as detected by brain MRI at Weeks 24, 48, and 96*
- The total number of new, and/or enlarging T2 hyperintense lesions as detected by brain MRI at Weeks 24, 48, and 96.
- *The proportion of patients who have confirmed disability improvement for at least 12 weeks, with the initial event of neurological improvement occurring during the 96-week double-blind, double-dummy treatment period.*
- The time to onset of *confirmed* disability progression for at least 24 weeks *with the* initial event of neurological worsening occur*ring* during the 96-week, double-blind, double-dummy, treatment period (see Section 5.3.2.2 for the definition of *confirmed* disability progression).
- *The total number of T1-hypo-intense lesions (Chronic Black Holes) at Weeks 24, 48, and 96*
- The change in MSFCS score from baseline to Week 96.

- The *percentage* change in brain volume as detected by brain MRI from Week 24 to Week 96.
- *The change in SF-36 PCS Score from baseline to Week 96*
- *The proportion of patients who have NEDA by Week 96*

### 8.1.3 Exploratory Efficacy Endpoints

The exploratory efficacy endpoints in this study *may* include, but may not be limited to:

- The change in low contrast visual acuity from baseline to Weeks 48 and 96.
- The change in the Symbol Digit Modalities Test from baseline to Weeks 48 and 96.
- *The proportion of relapse free patients by 96 weeks*
- *The change in total T2 hyperintense lesion volume as detected by brain MRI from baseline to Week 96*
- The annualized relapse rate, based on *all* clinical at the end of the 96-week comparative treatment period *(protocol-defined relapses are a subset of all clinical relapses).*
- *The ARR of relapses requiring IV steroid therapy.*
- *The ARR of severe relapses.*
- The *percentage* change in brain volume as detected by brain MRI from baseline to Week 96.
- The change in Multiple Sclerosis Functional Composite Scale (MSFCS) score from baseline to Week 48.
- The cumulative change in EDSS scores, measured in area under the curve (AUC) by Week 96.
- The change in EDSS from baseline to Week 96.
- The change in timed 25-foot walk from baseline to Week 96.
- The change in 9-hole peg test from baseline to Week 96.
- The change in paced auditory serial addition test (PASAT) from baseline to Weeks 48 and 96.
- The time to onset of sustained 20% increase in 9-hole peg test for at least 12 weeks.
- The time to onset of sustained 20% increase in timed 25 foot walk for at least 12 weeks.
- *The change in fatigue, as measured by the MFIS total score from baseline to Week 96.*
- *The change from baseline in patient-reported depressive symptoms, as measured by the CES-D, from baseline to Week 96.*
- *Analyses of EQ-5D, collected at baseline, Week 48, and Week 96.*
- The change in Karnofsky Performance Status Scale from baseline to Week 96.
- *The percentage change in cortical grey matter volume from baseline to Week 96.*
- *The percentage change in white matter volume from baseline to Week 96.*
- *The proportion of patients who have disability improvement confirmed for at least 24 weeks, with the initial event of neurological improvement occurring during the 96-week double-blind double-dummy treatment period.*
- *The proportion of patients who have disability improvement sustained for at least 12 weeks and sustained until the end of the 96-week, double-blind,*

*double-dummy treatment period, with the initial event of neurological improvement occurring during the 96-week, double-blind, double-dummy treatment period.*

- *The duration of the confirmed disability improvement.*
- *The proportion of patients who at Week 96 have improved, stable or worsened disability, compared to baseline.*
- *The change in Quality of Life, as measured by the Short Form 36 version 2 Mental Component Summary (MCS) Score from baseline to Week 96.*
- To evaluate the long-term safety, tolerability, and efficacy of ocrelizumab in patients with relapsing form of MS who are enrolled in the OLE Phase.

### 8.1.4 Safety

Safety will be assessed through regular neurological and physical examinations, vital signs, ECG, and the occurrence of adverse events. In addition, the following will be examined:

- Non-MS pathology in all available MRI scans.
- Columbia-suicide severity rating scale (C-SSRS).
- *Standard* hematology, chemistry, and urinalysis *assessments*;
- Circulating B-cell *total and* subsets, T cells, natural killer cells and other leukocytes;
- Plasma immunoglobulins;
- HAHA;
- Antibody titers for mumps, rubella, varicella, and Streptococcus pneumoniae;
- *MS relapses classified as serious*
- Serial pregnancy tests [serum/urine β subunit human chorionic gonadotropin (β hCG)] will be performed in women of child bearing potential.
- JC virus (JCV) plasma/urine sampling only if deemed necessary.

## 8.2 Statistical and Analytical Methods

Prior to unblinding the treatment groups, a *S*AP will be produced that will contain full details of all planned analyses. An outline of the planned analyses is described below.

*After the Week 96 visit of the last patient randomised, approximately 12 weeks may be needed to allow the confirmation of the last event of the 12 week-confirmed disability progression. Therefore the clinical cut-off date will occur approximately 12 weeks after the last patient's Week 96 visit when the status is clarified for each patient. Database lock and unblinding of the Sponsor will occur several weeks after the clinical cut-off in order to clarify all outstanding queries. The sites and EDSS raters will remain blinded until approximately 24 weeks after the Week 96 visit of the last patient randomised to allow the confirmation of the last 24 week-confirmed disability progression event, in case an updated analysis of this endpoint is requested at a later point.*

The time to onset of *confirmed* disability progression for at least 12 weeks during the 96-week comparative treatment period*, the proportion of patients who have confirmed disability improvement for at least 12 weeks with the initial event of neurological*

*improvement occurring during the 96-week double-blind, double-dummy treatment period,* and the time to onset of *confirmed* disability progression for at least 24 weeks during the 96-week comparative treatment period will be analyzed using pooled data across the two identical studies *running* as a part of the Phase III program, with respect to ocrelizumab group versus Rebif® group.

All eligible patients will be randomized to treatment stratified by region (United States versus ROW) and baseline EDSS (<4.0 versus ≥4.0). All analyses will also be stratified by region (United States versus ROW) and baseline EDSS (<4.0 versus ≥4.0).

*Some efficacy analyses will be undertaken for some subgroups, as agreed with regulatory authorities. Details of these subgroup analyses are presented in the SAP.*

All analyses, summaries and listings will be performed using SAS® software (Version 9.2 or higher in a UNIX environment).

### 8.2.1 Primary Efficacy Analysis

The primary efficacy analysis for this trial will compare annualized protocol-defined relapse rate by 96 weeks between ocrelizumab group and Rebif® group. The annualized relapse rates by 96 weeks will be *analyzed* using negative binomial model, adjusting for region (United States versus ROW) and baseline EDSS (<4.0 versus ≥4.0). The adjusted annualized relapse rates and the two-sided 95% confidence intervals for the relapse rates will be presented along with the p-value.

Other sensitivity analyses may also be performed for the primary efficacy endpoint (and documented in the SAP).

### 8.2.2 Secondary Efficacy Analyses

*The statistical testing strategy for all secondary efficacy endpoints, the testing hierarchy and the rationale for this hierarchical order of secondary endpoints is fully explained in the SAP.*

Secondary efficacy endpoints will be tested in hierarchal order, all at alpha = 0.05 level. The first secondary efficacy endpoint will be tested if and only if the primary endpoint has reached the significant level at 0.05 (e.g., P-value ≤0.05). *With the exception of the three secondary efficacy endpoints which will be analyzed at the pooled level (see Section 8.2), all* secondary *efficacy* endpoints will be tested if and only if the secondary endpoint list*ed* ahead of it has reached the significance level at 0.05.

*Additional pooled analyses are also described in a separate SAP (called the pooled SAP). This document describes solely those analyses that will be performed on the two studies combined.*

#### *8.2.2.1 The Time to Onset of Confirmed Disability Progression for At Least 12 Weeks During the 96-Week Comparative Treatment Period*

Time to *confirmed* disability progression (12 week confirmation) is defined as the time from Baseline to the first disability progression, which is confirmed at the next regularly

scheduled visit ≥84 days after the initial disability progression. Disability progression is defined as an increase of ≥1.0 point from baseline EDSS, if the baseline EDSS is between 0 and 5.5 points (inclusive), or an increase of ≥ 0.5 points, if the baseline EDSS is >5.5 points. Please note that the inclusion criteria of EDSS (0–5.5) only applies to screening EDSS. It is still possible that a patient's baseline EDSS (derived based on both screening and day 1 EDSS results) is >5.5. The non-confirmatory EDSS assessments (if any) between the initial and confirmation of disability progression should be at least as high as the minimum change required for progression. All initial disability progression events up to Week 96 with corresponding confirmation visits at the next scheduled visit (see Schedule of Assessments in Section 5) will be taken into account for the statistical analysis irrespective of whether or not the confirmation visit occurred during the treatment phase or after study drug discontinuation*, or during the OLE phase*. Thus, patients who prematurely discontinue study drug treatment should be kept in the study, and every effort should be made to follow up their EDSS status at the next scheduled visit. Patients who according to the above definition did not have *confirmed* disability progression by Week 96 visit, time of early discontinuation of treatment, or loss to follow up will be censored at the date of their last EDSS assessment.

Data from the two studies with respect to ocrelizumab group vs Rebif® group will be pooled for analysis of this endpoint. To assess the validity of pooling data across the two RMS studies, demographic and baseline characteristics will be compared by trials. The treatment effect (hazard ratio and CI) for *confirmed* disability progression within each trial will be compared between the two trials. In interpreting the trial comparisons with a view toward assessing the validity of the pooled dataset, the primary interest is in confirming that the treatment effect is qualitatively similar across the two studies – positive treatment effects (the estimated hazard to have *confirmed disability* progression in patients treated with OCR is numerically smaller than that in patients treated with Rebif) are shown in both studies. If the results from the two studies are not qualitatively similar (e.g., positive treatment effect is only shown in one study), data will not be pooled. *Further details regarding the assessment of poolability are provided in the SAP.*

Time to *confirmed* disability progression for ocrelizumab group and Rebif® group (across the studies) will be compared using a two-sided log-rank test stratifying by region (United States versus ROW), baseline EDSS (<4.0 versus ≥4.0). The proportion of patients with *confirmed* disability progression will be estimated using Kaplan-Meier methodology. The overall hazard ratio will be estimated using a stratified Cox regression model with the same stratification factors used in the stratified log-rank test above. *More details are provided in the SAP for the study.*

#### *8.2.2.2 Total Number of T1 Gadolinium-Enhanced Lesions as Detected by Brain MRI at Weeks 24, 48, and 96*

*The total number of T1 gadolinium (Gd)-enhanced lesions will be calculated as the sum of the individual number of T1 Gd-enhanced lesions at Weeks 24, 48 and 96. Data from other unscheduled assessments will not be included in this summary or analysis.*

*A negative binomial model will be used to compare the difference between ocrelizumab and Rebif groups.*

#### 8.2.2.3 The Total Number of New, and/or Enlarging T2 Hyperintense Lesions as Detected by Brain Magnetic Resonance Imaging at Week 24, Week 48 and Week 96

*The same approach will be used for the statistical analysis as for the total number of T1 Gadolinium-enhanced lesions.*

#### 8.2.2.4 Proportion of Patients who have Disability Improvement Confirmed for At Least 12 Weeks

*This endpoint will be analyzed only for the subgroup of patients with a baseline EDSS score ≥2.0. Exactly the same approach to data derivation will be used for disability improvement as for disability progression (refer to Section 8.2.2.1, although note that, here, the endpoint is a binary improved/not improved variable rather than a time-to-event endpoint). In particular, the same approach to the timing of the confirmation of disability improvement will be applied as for disability progression. The baseline EDSS score is the average of the EDSS score at the Screening Period and the EDSS score at the baseline visit, without rounding. For patients with a baseline EDSS score ≥2 and ≤5.5, disability improvement is defined as a reduction in EDSS score ≥1.0 compared to baseline EDSS score. For patients with a baseline EDSS score >5.5, disability improvement is defined as a reduction in EDSS score of 0.5. All patients without disability improvement will be counted as not improved, independent of follow-up time.*

*Data from the two studies with respect to ocrelizumab group vs Rebif® group will be pooled for analysis of this endpoint.*

*The proportions in treatment groups will be compared using the Cochran-Mantel-Haenszel (CMH) $\chi^2$ test stratified by geographical region (United States vs ROW) and baseline EDSS score (<4.0 vs ≥4.0).*

#### 8.2.2.5 The Time to Onset of *Confirmed* Disability Progression for At Least 24 Weeks During the 96-Week Comparative Treatment Period

Time to *confirmed* disability progression between ocrelizumab group and Rebif® group using a 24-week confirmation window for disability progression will be compared using the same analysis method for time to *confirmed* disability progression using a 12-week confirmation window. Time to *confirmed* disability progression (24-week confirmation) is defined as the time from Baseline (Day 1) to the first disability progression, which is confirmed at the next regularly scheduled visit ≥ 161 days after the initial disability progression. All initial disability progression events up to Week 96 with corresponding confirmation visits at the next schedule visit (see Schedule of Assessments in Section 5) will be taken into account for the statistical analysis. The same analysis principles as described in Section 8.2.2.1 will be applied to the 24-week disability endpoint.

*Data from the two studies with respect to ocrelizumab group versus Rebif® group will be pooled for analysis of this endpoint.*

#### 8.2.2.6 *Total Number of T1-Hypo-Intense Lesions (Chronic Black Holes) at Weeks 24, 48, and 96*

*The same approach will be used for the statistical analysis as for the totoal number of T1 Gadolinium-enhanced lesions.*

#### 8.2.2.7 The Change in Multiple Sclerosis Functional Composite Scale (MSFCS) Score from Baseline to Weeks 96

The change in MSFCS from baseline to Week 96 will be compared between ocrelizumab group and Rebif® group *using a Mixed-Effect Model Repeated Measures (MMRM) analysis, adjusting for baseline MSFCS, region (United States versus ROW), and baseline EDSS (<4.0 versus ≥4.0).* .

#### 8.2.2.8 The *Percentage* Change in Brain Volume as Detected by Brain Magnetic Resonance Imaging Scan from Week 24 to Week 96

The change in brain volume as detected by brain MRI from week 24 to Week 96 will be compared between ocrelizumab group and Rebif® group using *an MMRM analysis. Baseline covariates here will be as follows: brain volume at Week 24, baseline Gd lesion (present or not), region (United States versus ROW), and baseline EDSS score (<4.0 vs ≥ 4.0).*

#### 8.2.2.9 *Change in Quality of Life, as Measured by the Short Form 36 version 2 Physical Component Summary (PCS) Score from Baseline to Week 96*

*The change in quality of life, as measured by the SF-36 PCS score from baseline to week 96 will be compared between ocrelizumab group and Rebif group using a MMRM analysis. Baseline covariates here will be as follows: baseline PCS score, region (United States versus ROW), and baseline EDSS (< 4.0 vs ≥ 4.0).*

#### 8.2.2.10 *Proportion of Patients Who Have No Evidence of Disease Activity (NEDA) by Week 96*

*This endpoint will be defined only for those patients with a baseline EDSS score ≥ 2.0. All available data during the 96-week treatment period will be used for the analysis. Patients who complete the 96-week treatment period will be considered as having evidence of disease activity if at least one protocol defined relapse, a CDP event having occurred or at least one MRI scan showing MRI activity (defined as Gd-enhancing T1 lesions, or new or enlarging T2 lesions) was reported during the 96-week treatment period, otherwise the patient will be considered as having no evidence of disease activity (NEDA). Patients who discontinue treatment early with at least one event before early discontinuation will be considered as having evidence of disease activity.*

*Even if an event was not reported before early discontinuation, the patient will be considered as having evidence of disease activity if the reason for early discontinuation is lack of efficacy or death; otherwise, it will be considered a missing observation.*

*The proportions within treatment groups will be compared using the Cochran-Mantel-Haenszel (CMH) $\chi^2$ test stratified by region (United States versus ROW) and baseline EDSS (<4.0 versus ≥4.0).*

### 8.2.3 Exploratory Analyses

The exploratory endpoints will be summarized using tables, listings and graphs, where appropriate. Full details of the derivations and analyses of exploratory endpoints will be provided in the SAP.

### 8.2.4 Sample Size

The sample size for this study has been estimated based on data from previous RRMS trials, with the use of two-sided tests with an experiment-wise alpha of 0.05. The annualized rate of relapse among patients receiving ocrelizumab at 96 weeks is predicted to be 0.165 (standard deviation of approximately 0.60), as compared with 0.33 (standard deviation of approximately 0.80) among patients receiving the control treatment, Rebif® (this represents a relative reduction of 50% on ocrelizumab compared to the active comparator). For the annualized relapse rate, a t-test has been used to determine the sample size between ocrelizumab and the control arm. The sample size of 400 patients per arm provides 84 percent power, maintaining the type I error rate of 0.05, and assuming a drop out rate of 20 percent approximately (assuming relative reduction among patients drop out is 25%).

For *confirmed disability* progression, a two group test of equal exponential survival with exponential dropout is used to determine the sample size. Assuming the 2-year *confirmed* disability progression rate is 18% for the Rebif® arm and 12.6% for the ocrelizumab arm (this represents a relative reduction of 30% on ocrelizumab compared to the active comparator), and assuming a drop out rate of 20 percent over 2 years approximately, the sample size of 400 per arm will provide 80 percent power, maintaining the type I error rate of 0.05 based on the pooled analysis of two RMS trials (800 patients treated with ocrelizumab 600 mg and 800 patients treated with Rebif®).

### 8.2.5 Hypothesis Testing

The hypotheses to be tested are:

$H_0$ (null hypothesis): There is no statistically significant difference in annualized protocol-defined relapse rate at 2 years between ocrelizumab group and Rebif® group.

$H_1$ (alternative hypothesis): There is a statistically significant difference in annualized protocol-defined relapse rate at 2 years between ocrelizumab group and Rebif® group.

Annualized protocol-defined relapse rate at 2 years between the ocrelizumab group and Rebif® group will be compared using negative binomial model adjusting region (United States versus ROW) and baseline EDSS (<4.0 versus ≥4.0). If the test result for comparing 600 mg ocrelizumab and Rebif® groups is statistically significant at $\alpha<0.05$ level (two-sided test), we will conclude that the 600 mg ocrelizumab group demonstrated a superior effect of reducing Annualized protocol defined relapse rate, when compared to Rebif® group.

Similar hypotheses will also be tested for the secondary efficacy parameters. Methods for handling multiplicity issues related to secondary endpoints will be described in the SAP.

### 8.2.6 Analysis Populations

One patient population will be defined for the purpose of the safety analysis and two for the efficacy analysis. All efficacy analyses will be performed using the intent-to-treat (ITT) population. The per-protocol (PP) population will be used for all primary efficacy

analyses in order to evaluate the influence of major protocol violators and as a sensitivity check to the ITT analysis.

#### *8.2.6.1 Safety Population*

This population will be used for all summaries of safety data. The safety population will include all patients who received any study drug. Randomized patients that receive incorrect therapy from that intended will be summarized in the group according to the therapy actually received. Patients who are not randomized, but who receive study drug will be included in the safety population and summarized according to the therapy actually received.

#### *8.2.6.2 Intent-to-Treat Population*

All randomized patients will be included in the intent–to-treat population. Patients who prematurely withdraw from the study for any reason and for whom an assessment is not performed for whatever reason will still be included in the ITT analysis. Patients who receive an incorrect therapy from that which is intended will be summarized according to their randomized treatment.

#### *8.2.6.3 Per Protocol Population*

The per protocol population will include all patients in the ITT population adhering to the protocol. Patients may be excluded if they significantly violate the inclusion/exclusion criteria or deviate from the study plan. Specific reasons for warranting exclusion will be agreed and documented in the *S*AP prior to unblinding of the treatment groups. Only those patients with violations that are deemed to potentially affect the efficacy of study treatment will be excluded from the per protocol population. Patients who receive an incorrect therapy from that intended will be excluded from the per protocol population.

### 8.2.7 Interim Analysis

No formal efficacy interim analyses are planned.

### 8.2.8 Safety Data Analysis

All safety parameters will be summarized and presented in tables based on *the* safety population. The safety data will be listed and summarized at determined cut off points, e.g., using data for each patient up to Week 96, using all available data at the Week 96 database lock for the primary analysis.

All adverse events will be coded and tabulated by system organ class and preferred term for individual events within each system organ class, and will be presented in descending frequency. Adverse events will also be tabulated by severity and relationship to the study medication. Serious adverse events will be summarized separately. Results of C-SSRS will be *summarized by treatment group*.

Non-MS pathology reported by local safety radiologist will be summarized by treatment group.

Associated laboratory parameters such as hepatic function, renal function and hematology values will be grouped and presented together. Correlation between low IgG and IgM and infections will be presented separately. Marked abnormalities will also be

flagged. Marked abnormalities will be tabulated for each laboratory test by treatment group.

Analysis of HAHA to ocrelizumab will be summarized graphically and descriptively. Correlation between presence of HAHA and IRR/B-cell depletion will be presented descriptively.

The results of vital sign, physical examination and ECG will be included in individual patient listings. Change from baseline in vital signs will be summarized by groups.

*Type I interferon neutralizing antibody data will be summarized in listings and additional analysis will be performed as appropriate.*

An external, independent DMC will *periodically* review safety data throughout the study. *The DMC will continue safety monitoring during the OLE phase.* Analyses required for the DMC data review will be performed as described in the DMC Charter and DMC data handling plan.

### 8.2.9 Safety Follow-up Period

Data from this period will be analyzed to provide information on the maintenance effect and the potential withdrawal effect of ocrelizumab. In addition, data will be analyzed to provide information concerning the long-term safety of ocrelizumab. Data will be summarized and tables and listings will be produced.

### 8.2.10 Open-Label Extension Phase

Data from this period of the study will be analyzed in order to characterize the long-term safety and efficacy of patients treated with ocrelizumab beyond the double-blind, double-dummy treatment period of the study. The data will be summarized according to the randomized treatment groups of the double-blind, double-dummy treatment period of the study. Details of the statistical analyses will be provided in a separate *S*AP.

### 8.2.11 Other Analyses

#### *8.2.11.1 Pharmacokinetic Analysis*

**Pharmacokinetic Parameters**

Ocrelizumab serum concentration-time data will be modeled using a population approach. The primary population PK parameters (Clearances and Volumes) for ocrelizumab will be estimated by means of NONMEM analysis of the sparse PK data. Clearances with associated inter-patient variability may be characterized by a saturable and non-saturable clearance as well as an intercompartmental clearance depending on the final structural model. Volumes with associated inter-patient variability may be characterized by central and peripheral volumes depending on the final structural model. Exposure (AUC) to ocrelizumab will be estimated. The selection of other parameters will depend on the final PK model used for this analysis.

**Pharmacokinetic Analysis**

Nonlinear mixed effects modeling (with software NONMEM [69]) will be used to analyze the sparse sampling dose-concentration-time data of ocrelizumab. Patients who

have measurable concentrations of ocrelizumab will be included in the PK analysis unless major protocol deviations or unavailability of information (e.g., exact blood sampling time) occurred which may interfere with PK evaluation. The PK data of this study may be pooled with more extensive data from other studies. Population PK parameters (Clearances and Volumes) will be estimated and the influence of covariates, such as age, gender, weight, HAHA, and baseline CD19 lymphocytes, on these parameters will be investigated.

Details of the mixed-effects modeling analyses will be described in a Modeling and Simulation Analysis Plan and results will be reported separately.

#### *8.2.11.2 Pharmacodynamic Analysis*

The relationship between individual ocrelizumab exposure and selected safety and efficacy parameters will be analyzed and explored, in order to characterize the exposure/dose response curve of ocrelizumab. This may include but is not limited to annualized relapse rate, T1 and T2 lesions at week 96, IRRs, infections, and other AEs or safety parameters of interest. Other exploratory analyses may be performed to assess the possible relationship between PD markers e.g., CD19 count, PK, and clinical response.

#### *8.2.11.3 Roche Clinical Repository / Protein Biomarker Samples*

Additional blood samples for serum and/or plasma analyses will be taken for research purposes subject to discretionary approval from each center's IRB/IEC and the patient's specific written consent. These samples will be used to identify dynamic biomarkers to help us better understand the pathogenesis of RMS and response to treatment with ocrelizumab. Such future biomarkers have yet to be determined but may include circulating biochemical markers in blood including cytokines as well as peripheral blood gene expression patterns. Exploratory statistical data analyses may include assessments for possible relationships between these biomarker levels, PK and clinical response.

# 9. DATA COLLECTION, MANAGEMENT AND QUALITY ASSURANCE

The overall procedures for quality assurance of clinical study data are described in the Sponsor's (or designee) Standard Operational Procedures.

Data for this study will be recorded via an Electronic Data Capture (EDC) system using electronic Case Report Forms. It will be transcribed by the site from the paper source documents onto the eCRF. In addition, EDSS, MSFC, C-SSRS, Karnofsky Performance Status Scale and patient reported outcomes will be collected via an electronic interface. The data will be transmitted from the electronic interface to a central database that will later be transferred to the Sponsor (or designee).

Accurate and reliable data collection will be assured by verification and cross–check of the eCRFs against the Investigator's records by the study monitor (source document verification), and the maintenance of a drug–dispensing log by the Investigator.

A comprehensive validation check program utilizing front-end checks in the eCRF/electronic interface and back-end checks in the data base will verify the data and discrepancies will be generated accordingly. These are transferred electronically to the site for resolution by the Investigator.

Throughout the study the Study Management Team (SMT) will review data according to the EDC Cleaning Process as described in the Data Management Plan.

## 9.1 Assignment of Preferred Terms and Original Terminology

For classification purposes, preferred terms will be assigned by the Sponsor to the original terms entered on the eCRF, using the most up-to-date version of the Medical Dictionary for Regulatory Activities (MedDRA) terminology for adverse events and diseases and the International Non-proprietary Name (INN) Drug Terms and Procedures Dictionary for treatments and surgical and medical procedures.

# 10. STUDY COMMITTEES

**Steering Committee**

An external Steering Committee will provide general guidance, assist with liaison to Investigators and oversee any external communication of the results of the study.

**Data Monitoring Committee (DMC)**

An external independent Data Monitoring Committee (DMC) will be chartered to review safety data throughout the study and make recommendations regarding continuation, termination, or modification of the study. *During the double-blind, double-dummy phase of the study,* regularly scheduled safety data reviews will occur at least three times per year after the first patient is enrolled. *The DMC will continue safety monitoring during the OLE phase.*

Any safety event that requires unblinding of study treatment allocation will be immediately reported to the DMC and to the health authorities in an expedited safety report. The DMC may request and review any additional reports outside of the planned analyses at any time if deemed necessary to ensure the safety of patients. The safety evaluations will be conducted on parameters specified within the DMC charter and may vary depending on the requirements and requests of the DMC.

The details of the DMC roles and responsibilities, scope of work and the logistics of the DMC activities will be outlined in a DMC Charter. The purpose of the DMC interim analyses is primarily safety evaluation, and the study may be stopped or amended because of significant safety concerns.

# 11. REFERENCES

1. Neurological Disorders: Public health Challenges WHO, WHO Press, 1211 Geneva 27, Switzerland, assessed on line on July 6, 2010. http://www.who.int/mental_health/neurology/chapter_3_a_neuro_disorders_public_h_challenges.pdf

2. McDonald WI, Compston A, Edan G, et al. Recommended diagnostic criteria for multiple sclerosis: guidelines from the International Panel on the Diagnosis of Multiple Sclerosis. Ann Neurol 2001;50:121–7.

3. Polman CH, Reingold SC, Banwell B, et al. Diagnostic criteria for multiple sclerosis: 2010 revisions to the "McDonald Criteria". Ann Neurol 2011;69:292–302.

4. Lublin FD and Reingold SC. Defining the clinical course of multiple sclerosis: results of an international survey. National Multiple Sclerosis Society (USA) Advisory Committee on Clinical Trials of New Agents in Multiple Sclerosis. Neurology 1996;46:907–11.

5. Filippini G, Munari L, Incorvaia B, Ebers GC, Polman C, D'Amico R, et al. Interferons in relapsing remitting multiple sclerosis: a systematic review. Lancet 2003;361:545–52.

6. Compston A, Coles A. Multiple sclerosis. Lancet 2008; 372:1502-17.

7. O'Connor P, Wolinsky JS, Confavreux C, et al. Randomized trial of oral teriflunomide for relapsing multiple sclerosis. N Engl J Med 2011;365:1293-1303.

8. Kappos, et al. The efficacy and safety of teriflunomide in patients with relapsing MS: results from TOWER, a phase III, placebo-controlled study [abstract]. ECTRIMS 2012. 2012:Abstract 153.

9. A multicenter, randomized, parallel-group, rater-blinded study comparing the effectiveness and safety of teriflunomide and interferon beta-1a in patients with relapsing multiple sclerosis. [TENERE]; Date of access 04 Mar 2013. http://clinicaltrials.gov/ct2/show/results/NCT00883337.

10. Hauser SL, Waubant E, Arnold DL, et al; HERMES Trial Group. B-cell depletion with rituximab in relapsing-remitting multiple sclerosis. N Engl J Med 2008;358:676–88.

11. Sidén A. Isoelectric focusing and crossed immunoelectrofocusing of CSF immunoglobulins in MS. J Neurol 1979;221:39–51.

12. Meinl E, Krumbholz M, Hohlfeld R. B lineage cells in the inflammatory central nervous system environment: migration, maintenance, local antibody production, and therapeutic modulation. Ann Neurol 2006;59:880–92.

13. Franciotta D, Salvetti M, Lolli F, Serafini B, Aloisi F. B cells and multiple sclerosis. Lancet Neurol 2008;7:852-8.

14. McFarland HF. The B cell--old player, new position on the team. N Engl J Med 2008;358:664–5.

15. Owens GP, Kraus H, Burgoon MP, et al. Restricted use of VH4 germline segments in an acute multiple sclerosis brain. Ann Neurol 1998;43:236–43.

16. Baranzini SE, Jeong MC, Butunoi C, Murray RS, Bernard CC, Oksenberg JR. B cell repertoire diversity and clonal expansion in multiple sclerosis brain lesions. J Immunol 1999;163:5133-44.

17. Colombo M, Dono M, Gazzola P, et al. Accumulation of clonally related B lymphocytes in the cerebrospinal fluid of multiple sclerosis patients. J Immunol 2000;164:2782-9.

18. Ritchie AM, Gilden DH, Williamson RA, et al. Comparative analysis of the CD19+ and CD138+ cell antibody repertoires in the cerebrospinal fluid of patients with multiple sclerosis. J Immunol 2004;173:649–56.

19. Lambracht-Washington D, O'Connor KC, et al. Antigen specificity of clonally expanded and receptor edited cerebrospinal fluid B cells from patients with relapsing remitting MS. J Neuroimmunol 2007;186:164–76.

20. Owens GP, Winges KM, Ritchie AM, et al. VH4 gene segments dominate the intrathecal humoral immune response in multiple sclerosis. J Immunol 2007;179:6343–51.

21. Monson NL, Brezinschek HP, Brezinschek RI, et al. Receptor revision and atypical mutational characteristics in clonally expanded B cells from the cerebrospinal fluid of recently diagnosed multiple sclerosis patients. J Neuroimmunol 2005;158:170–81.

22. Obermeier B, Mentele R, Malotka J, et al. Matching of oligoclonal immunoglobulin transcriptomes and proteomes of cerebrospinal fluid in multiple sclerosis. Nat Med 2008;14:688–93.

23. Reindl M, Linington C, Brehm U, et al. Antibodies against the myelin oligodendrocyte glycoprotein and the myelin basic protein in multiple sclerosis and other neurological diseases: a comparative study. Brain 1999;122:2047–56.

24. Egg R, Reindl M, Deisenhammer F, et al. Anti-MOG and anti-MBP antibody subclasses in multiple sclerosis. Mult Scler 2001;7:285–9.

25. Andersson M, Yu M, Söderström M, et al. Multiple MAG peptides are recognized by circulating T and B lymphocytes in polyneuropathy and multiple sclerosis. Eur J Neurol 2002;9:243–51.

26. Genain CP, Cannella B, Hauser SL, et al. Identification of autoantibodies associated with myelin damage in multiple sclerosis. Nat Med 1999;5:170–5.

27. Serafini B, Rosicarelli B, Magliozzi R, et al. Detection of ectopic B-cell follicles with germinal centers in the meninges of patients with secondary progressive multiple sclerosis. Brain Pathol 2004;14:164–74.

28. Magliozzi R, Howell O, Vora A, et al. Meningeal B-cell follicles in secondary progressive multiple sclerosis associate with early onset of disease and severe cortical pathology. Brain 2007;130:1089–104.

29. Qin Y, Duquette P, Zhang Y, et al. Clonal expansion and somatic hypermutation of V(H) genes of B cells from cerebrospinal fluid in multiple sclerosis. J Clin Invest 1998;102:1045–50.

30. Aloisi F and Pujol-Borrell R. Lymphoid neogenesis in chronic inflammatory diseases. Nat Rev Immunol 2006;6:205–17.

31. Howell OW, Reeves C, Magliozzi R, et al. The incidence of meningeal B-cell follicles in secondary progressive multiple sclerosis: a neuropathological study of 96 cases (abstract). Mult Scler 2009;15:S5.

32. Kappos L, Li D, Calabresi PA, et al. Ocrelizumab in relapsing-remitting multiple sclerosis: a phase 2, randomised, placebo-controlled, multicentre trial. Lancet 2011;378(9805):1779-87.

33. Kappos L, et al. Long-term safety and efficacy of ocrelizumab in patients with relapsing-remitting multiple sclerosis: Week 144 results of a phase II, randomised, multicentre trial [abstract]. ECTRIMS 2012. 2012:abstract 362.

34. *Bar-Or A, Calabresi PA, Arnold D, et al. Rituximab in relapsing-remitting multiple sclerosis: a 72-week, open-label, Phase I trial. Ann Neurol 2008;63:395–400.*

35. Hawker K, O'Connor P, Freedman M, et al. Rituximab in patients with primary progressive multiple sclerosis: results of a randomized double-blind placebo-controlled multicenter trial. Ann Neurol 2009;66:460-71.

36. PRISMS Study Group. Randomized double-blind placebo controlled study of interferon-beta-1a in relapsing/remitting multiple sclerosis. Lancet 1998;352:1498–504.

37. PRISMS Study Group, University of British Columbia MS/MRI Analysis Group. PRISMS-4: long-term efficacy of interferon-beta-1a in relapsing MS. Neurology 2001;56:1628–36.

38. Panitch H, Goodin DS, Francis G, Chang P, Coyle PK, O'Connor P, Monaghan E, Li D, Weinshenker B; EVIDENCE Study Group. Evidence of Interferon Dose-response: European North American Comparative Efficacy; University of British Columbia MSMRI Research Group. Randomized, comparative study of interferon beta-1a treatment regimens in MS: The EVIDENCE Trial. Neurology 2002;59(10):1496-506.

39. Secondary Progressive Efficacy Clinical Trial of Recombinant Interferon-Beta-1a in MS (SPECTRIMS) Study Group. Randomized controlled trial of interferon- beta-1a in secondary progressive MS: Clinical results. Neurology 2001;56(11):1496-504.

40. European Public Assessments reports, Rebif Summary of product characteristic, January 27, 2010.

41. REBIF U.S. Physician Prescribing Information, Revised: July 2009.

42. Lim SY, Constantinescu CS. Current and future disease-modifying therapies in multiple sclerosis. Int J Clin Pract. 2010;64(5):637-50.

43. Polman C et al; Recommendations for clinical use of data on neutralizing antibodies to interferon-beta therapy in multiple sclerosis. Lancet Neurol 2010;9:740–50.

44. Polman CH, Reingold SC, Barkhof F, et al. Ethics of placebo-controlled clinical trials in multiple sclerosis: a reassessment. Neurology 2008;70:1134–40.

45. Rio J, Comabella M, Montalban X. Multiple sclerosis: current treatment algorithms. Curr Opin Neurol 2011:24;230-7.

46. Lindberg RL, De Groot CJ, Certa U, et al. Multiple sclerosis as a generalized CNS disease – comparative microarray analysis of normal appearing white matter and lesions in secondary progressive MS. J Neuroimmunol 2004;152:154-67.

47. Trapp, BD, Peterson J, Ransohoff RM, et al. Axonal transection in the lesions of multiple sclerosis. N Engl J Med 1998;338:278-85.

48. Freedman MS. Long-term follow-up of clinical trials of multiple sclerosis therapies. Neurology 2011:76; S26-34.

49. Scalfari A, Neuhaus A, Degenhardt A, et al. The natural history of multiple sclerosis: a geographically based study 10: relapses and long-term disability. Brain 2010: 133;1914-29.

50. Day RO, Williams KM. Open-label extension studies: do they provide meaningful information on the safety of new drugs? Drug Saf 2007;30:93-105.

51. Kap YS, van Driel N, Blezer E, et al. Late B Cell Depletion with a Human Anti-Human CD20 IgG1 κ Monoclonal Antibody halts the Development of Experimental Autoimmune Encephalomyelitis in Marmosets. J Immunol 2010: 185; 3990-4003.

52. Gong Q, Ou Q, Ye S, et al. Importance of Cellular Microenvironment and Circulatory Dynamics in B Cell Immunotherapy. J Immunol 2005:174;817-26.

53. Ahuja A, Shupe J, Dunn R, et al. Depletion of B Cells in Murine Lupus: Efficacy and Resistance. J Immunol 2007:179;3351–61.

54. Gelinck LBS, et al. Poor serological responses upon influenza vaccination in patients with rheumatoid arthritis treated with rituximab. Ann Rheum Dis 2007; 66:1402-3.

55. van Assen S, Holvast A, Benne CA, et al. Humoral responses after influenza vaccination are severely reduced in patients with rheumatoid arthritis treated with Rituximab. Arthritis & Rheumatism 2010; 62:75-81.

56. Oren S. Vaccination against influenza in rheumatoid arthritis patients: the effect of rituximab on the humoral response. Ann Rheum Dis published online November 2, 2007.

57. Bingham C, Looney R, Deodhar A, et al. Results from a controlled clinical trial (SIERRA) to evaluate primary and recall responses to immunizations in RA patients treated with rituximab. Arthritis Rheum 2008; 58:900-901[abstract#1999]. http://acr.confex.com/acr/2008/webprogram/Paper3941.html assessed on December 17, 2009

58. Cohen JA, Fischer JS, Bolibrush DM, et al. Intrarater and interrater reliability of the MS functional composite outcome measure. Neurology 2000;54:802-6.

59. Fischer JS, Rudick RA, Cutter GR, Reingold SC. The Multiple Sclerosis Functional Composite Measure (MSFC): an integrated approach to MS clinical outcome assessment. National MS Society Clinical Outcomes Assessment Task Force. Mult Scler 1999;5:244-50.

60. Guidance for Industry: Patient-reported outcome measures: Use in medical product development to support labeling claims, FDA 2009, accessed at http://www.fda.gov/downloads/Drugs/GuidanceComplianceRegulatoryInformation/Guidances/UCM193282.pdf

61. Fairclough DL, Design and analysis of quality of life studies in clinical trials. Boca Raton, FL: CRC Press; 2010.

62. Frohman EM, Fujimoto JG, Frohman TC, et al. Optical coherence tomography: a window into the mechanisms of multiple sclerosis. Nature Clinical Practice 2008;4:664-75.

63. Sergott RC, Frohman E, Glanzman R, et al. The role of optical coherence tomography in multiple sclerosis: expert panel consensus. J Neurol Sci 2007;263:3-14.

64. Costello F, Hodge W, Pan YI, et al. Tracking retinal nerve fiber layer loss after optic neuritis: a prospective study using optical coherence tomography. Multiple Sclerosis 2008;14:893-905.

65. Emery P, Fleischmann R, Filipowicz-Sosnowska A, et al. The efficacy and safety of rituximab in patients with active rheumatoid arthritis despite methotrexate treatment: results of a phase IIB randomized, double-blind, placebo-controlled, dose-ranging trial. Arthritis Rheum 2006;54:1390-400.

66. Calabrese LH, Molloy ES, Huang D, Ransohoff RM. Progressive multifocal leukoencephalopathy in rheumatic diseases: evolving clinical and pathologic patterns of disease. Arthritis Rheum 2007;56:2116-28.

67. Kappos L, Bates D, Hartung HP, et al. Natalizumab treatment for multiple sclerosis: recommendations for patient selection and monitoring. Lancet Neurol 2007;6:431-41.

68. Yousry TA, Major EO, Ryschkewitsch C, et al. Evaluation of patients treated with natalizumab for progressive multifocal leukoencephalopathy. N Engl J Med 2006;354:924-33.

69. Beal SL, Boeckman AJ, Sheiner LB. NONMEM User's Guide, Parts I-VIII San Francisco: Division of Clinical Pharmacology- University of California, 1992.

# PART II: ETHICS AND GENERAL STUDY ADMINISTRATION

## 12. ETHICAL ASPECTS

### 12.1 Local Regulations/Declaration of Helsinki

The Investigator will ensure that this study is conducted in full conformance with the principles of the "Declaration of Helsinki" or with the laws and regulations of the country in which the research is conducted, whichever affords the greater protection to the individual. The study must fully adhere to the principles outlined in "Guideline for Good Clinical Practice" ICH Tripartite Guideline or with local law if it affords greater protection to the patient. For studies conducted in the EU/EEA countries, the Investigator will ensure compliance with the EU Clinical Trial Directive [2001/20/EC]. For studies conducted in the USA or under US IND, the Investigator will additionally ensure adherence to the basic principles of "Good Clinical Practice" as outlined in the current version of 21 CFR, subchapter D, part 312, "Responsibilities of Sponsors and Investigators", part 50, "Protection of Human Subjects", and part 56, "Institutional Review Boards".

In other countries where a "Guideline for Good Clinical Practice" exists, Roche and the Investigators will strictly ensure adherence to the stated provisions.

### 12.2 Informed Consent

#### 12.2.1 Study Informed Consent

**It is the responsibility of the Investigator, or a person designated by the Investigator [if acceptable by local regulations], to obtain signed informed consent for the double-blind, double-dummy treatment period and the OLE Phase of the study from each patient prior to participating in either the double-blind, double-dummy treatment period or the OLE Phase of this study after adequate explanation of the aims, methods, anticipated benefits, and potential hazards of the study**.

The Investigator or designee must also explain that the patients are completely free to refuse to enter any period of the study or to withdraw from it at any time, for any reason.

The electronic Case Report Forms for this study contain a section for documenting patient informed consent, and this must be completed appropriately. If new safety information results in significant changes in the risk/benefit assessment, the consent form should be reviewed and updated if necessary. All patients (including those already being treated) should be informed of the new information, given a copy of the revised form and give their consent to continue in the study.

For the patient not qualified or incapable of giving legal consent, written consent must be obtained from the legally acceptable representative. In the case where both the patient and his/her legally acceptable representative are unable to read, an impartial witness should be present during the entire informed consent discussion. After the patient and representative have orally consented to participation in the trial, the witness' signature on the form will attest that the information in the consent form was accurately explained and understood.

**For U.S.-IND studies:** In a life-threatening situation where a patient is unconscious or otherwise unable to communicate, the emergency is such that there is not enough time to obtain consent from the patient's legally acceptable representative, and there is no other or better treatment available, it is permissible to treat the patient under protocol with consent of both the Investigator and another physician not involved in the study, with appropriate documentation submitted to the IRB within 5 days. If this collaboration is not immediately possible, there must be a written evaluation by a physician independent of the study and the appropriate documentation be submitted to the IRB within 5 days of treating the patient. In addition, the patient or his/her legally acceptable representative should be informed about the trial as soon as possible and consent to continue, giving written consent as described above.

**For non-U.S.-IND studies:** In a life-threatening situation where a patient is unconscious or otherwise unable to communicate, the emergency is such that there is not enough time to obtain consent from the patient's legally acceptable representative, and there is no other or better treatment available, it is permissible to treat the patient under protocol with consent of the Investigator, with appropriate documentation that the IEC had approved the procedures used to enroll patients in such situations. In addition, the patient or his/her legally acceptable representative should be informed about the trial as soon as possible and consent to continue, giving written consent as described above.

### 12.2.2 RCR Informed Consent

It is the responsibility of the Investigator, or a person designated by the Investigator (if acceptable under local regulations), to obtain written informed consent from each individual who has consented to RCR sampling after adequate explanation of the aims, methods, objectives and potential hazards. Subjects must receive an explanation that they are completely free to refuse to provide the RCR specimen(s) and may withdraw his/ her sample at any time and for any reason during the study or 15 year storage period of the specimen(s). The Informed Consent for an **optional** specimen donation will be incorporated as a specific section into the main Clinical Trial Informed Consent Form (ICF). A second, separate, specific signature consenting to specimen donation will be required to document the study participant’s agreement to provide an **optional** specimen; if the participant declines, he/ she will check a “no” box in the appropriate section and not provide a second signature.

The patient does not have to provide a separate consent for protein biomarker RCR sampling.

The eCRF for the associated clinical study contains a page for documenting patient informed consent to the RCR, and this must be completed appropriately.

### 12.2.3 Death or Loss of Competence of Participant who has donated a specimen(s) that is stored in the RCR

In case the Informed Consent Form and/or the Study Protocol do not provide any specific provisions for death or loss of competence, specimen and data will continue to be used as part of RCR research.

In the event of the death of a participant of a Roche Clinical Trial or Experimental Medicine Research study or if a participant is legally incompetent at the time of the specimen and data procurement, or becomes legally incompetent thereafter, applicable provisions as stated for such situations in the respective Informed Consent Form and/or the Study Protocol shall become effective and be followed accordingly.

Additional procurement of assent from legally incompetent persons and minors shall take place according to local laws and international best practice, as it applies to the specific case.

## 12.3 Independent Ethics Committees (IEC) and Institutional Review Board (IRB)

The protocol, informed consent and any accompanying material provided to the patient in the U.S. will be submitted by the Investigator to an IRB for review. For EEA member states, the Sponsor will submit to the Competent Authority and IEC, the protocol and any accompanying material provided to the patient. In both the US and EEA member states, the accompanying material may include patient information sheets, descriptions of the study used to obtain informed consent and terms of any compensation given to the patient as well as advertisements for the trial.

An approval letter or certificate (specifying the protocol number and title) from the IEC/IRB must be obtained before study initiation by the Investigator specifying the date on which the committee met and granted the approval. This applies whenever subsequent amendments/modifications are made to the protocol.

Any modifications made to the protocol, informed consent or material provided to the patient after receipt of the IEC/IRB approval must also be submitted by the Investigator in the U.S. and by the Sponsor in the EEA member states in accordance with local procedures and regulatory requirements.

When no local review board exists, the Investigator is expected to submit the protocol to a regional committee. If no regional committee exists, Roche will assist the Investigator in submitting the protocol to the European Ethics Review Committee.

Sampling for the RCR is contingent on review and approval for the exploratory biomarker assessments and written informed consent by an appropriate regulatory body (depending on the country where the study is performed) and a site's Institutional Review Board (IRB) / Ethics Committee (EC). If a regulatory or site's IRB/EC does not approve the sampling for the exploratory assessments the section on biomarker sampling will not be applicable.

Roche shall also submit an Annual Safety Report once a year to the IEC and Competent Authorities (CAs) according to local regulatory requirements and timelines of each country participating in the study. In the U.S. Roche submits an IND Annual Report to the FDA according to local regulatory requirements and timelines.

## 12.4 Role of the Science and Ethics Advisory Group (SEAG)

A Science and Ethics Advisory Group consisting of experts in the fields of biology, ethics, sociology and law will advise Roche regarding the use of specimens stored in the RCR and on the scientific and ethical aspects of handling genetic information. The SEAG is independent of Roche.

# 13. CONDITIONS FOR MODIFYING THE PROTOCOL

Requests from Investigators to modify the protocol to ongoing studies will be considered only by consultation between an appropriate representative of the Sponsor and the Investigator [Investigator representative(s) in the case of a multicenter trial]. Protocol modifications must be prepared by a representative of the Sponsor and initially reviewed and approved by the Clinical Science Leader and Biostatistician.

All protocol modifications must be submitted to the appropriate Independent Ethics Committee or Institutional Review Board for information and approval in accordance with local requirements, and to Regulatory Agencies if required. Approval must be obtained before any changes can be implemented, except for changes necessary to eliminate an immediate hazard to trial patients, or when the change(s) involves only logistical or administrative aspects of the trial (e.g., change in monitor[s], change of telephone number[s]).

# 14. CONDITIONS FOR TERMINATING THE STUDY

Both the Sponsor and the Investigator reserve the right to terminate the study at any time. Should this be necessary, both parties will arrange the procedures on an individual study basis after review and consultation. In terminating the study, Roche and the Investigator will assure that adequate consideration is given to the protection of the patients' interests. The appropriate IRB/EC and Regulatory Agencies should be informed accordingly.

# 15. STUDY DOCUMENTATION, CRFS AND RECORD KEEPING

## 15.1 Investigator's Files / Retention of Documents

The Investigator must maintain adequate and accurate records to enable the conduct of the study to be fully documented and the study data to be subsequently verified. These documents should be classified into two different separate categories [1] Investigator's Study File, and [2] patient clinical source documents.

The Investigator's Study File will contain the protocol/amendments, eCRF and schedule of assessments, Independent Ethics Committee/Institutional Review Board and governmental approval with correspondence, sample informed consent, drug records, staff curriculum vitae and authorization forms and other appropriate documents/correspondence, etc. In addition at the end of the study the Investigator will receive the patient data, which includes an audit trail containing a complete record of all changes to data, query resolution correspondence and reasons for changes, in human readable format on CD which also has to be kept with the Investigator's Study File.

Subject clinical source documents (usually defined by the project in advance to record key efficacy/safety parameters independent of the eCRFs) would include patient hospital/clinic records, physician's and nurse's notes, appointment book, original

laboratory reports, ECG, EEG, X-ray, pathology and special assessment reports, signed informed consent forms, consultant letters, and patient screening and enrollment logs. The Investigator must keep the two categories of documents as described above (including the archival CD) on file for at least 15 years after completion or discontinuation of the study. After that period of time the documents may be destroyed, subject to local regulations.

Should the Investigator wish to assign the study records to another party or move them to another location, Roche must be notified in advance.

If the Investigator can not guarantee this archiving requirement at the investigational site for any or all of the documents, special arrangements must be made between the Investigator and Roche to store these in a sealed container(s) outside of the site so that they can be returned sealed to the Investigator in case of a regulatory audit. Where source documents are required for the continued care of the patient, appropriate copies should be made for storing outside of the site.

ICH GCP guidelines require that Investigators maintain information in the study patient's records which corroborate data collected on the eCRF(s). Completed eCRF will be transferred to Sponsor.

## 15.2 Source Documents and Background Data

The Investigator shall supply the Sponsor on request with any required background data from the study documentation or clinic records. This is particularly important when errors in data transcription are suspected. In case of special problems and/or governmental queries or requests for audit inspections, it is also necessary to have access to the complete study records, provided that patient confidentiality is protected.

## 15.3 Audits and Inspections

The Investigator should understand that source documents for this trial should be made available to appropriately qualified personnel from the Roche Pharma Development Quality Assurance Unit or its designees, or to health authority inspectors after appropriate notification. The verification of the eCRF data must be by direct inspection of source documents.

## 15.4 Electronic Case Report Forms

Data for this study will be captured via an Electronic Data Capture (EDC) system by using eCRFs. An audit trail will maintain a record of initial entries and changes made; reasons for change; time and date of entry; and user name of person authorizing entry or change. The Investigator must update eCRF and connect on a regular basis.

For each patient enrolled, an eCRF must be completed and electronically signed by the principal Investigator or authorized delegate from the study staff. This also applies to records for those patients who fail to complete the study (even during a pre-randomization screening period if an eCRF was initiated). If a patient withdraws from the study, the reason must be noted on the eCRF. If a patient is withdrawn from the study because of a treatment-limiting AE, thorough efforts should be made to clearly document the outcome.

The Investigator should ensure the accuracy, completeness and timeliness of the data reported to the Sponsor in the eCRFs and in all required reports.

### 15.5 Financial Disclosure

The Investigator(s) will provide the Sponsor with sufficient accurate financial information (PD35) to allow the Sponsor to submit complete and accurate financial certification or disclosure statements to the appropriate regulatory authorities. The Investigator is responsible to promptly update any information provided to the Sponsor if relevant changes occur in the course of the investigation and for 1 year following the completion of the study (last patient, last visit).

## 16. MONITORING THE STUDY

It is understood that the responsible Roche monitor [or designee] will contact and visit the Investigator regularly and will be allowed, on request, to inspect the various records of the trial (eCRFs and other pertinent data) provided that patient confidentiality is maintained in accord with local requirements.

It will be the monitor's responsibility to inspect the eCRFs at regular intervals throughout the study, to verify the adherence to the protocol and the completeness, consistency and accuracy of the data being entered on them. The monitor must verify that the patient received the study drug assigned by the randomization center (by controlling the written confirmation of the randomization by IxRS). The monitor should have access to laboratory test reports and other patient records needed to verify the entries in the eCRF. The Investigator (or deputy) agrees to cooperate with the monitor to ensure that any problems detected in the course of these monitoring visits are resolved.

Roche Clinical Repository specimens will at all times be tracked in a manner consistent with Good Clinical Practice, by a quality controlled, auditable and validated Laboratory Information Management System, to ensure compliance with data confidentiality as well as adherence to authorized use of specimens as specified in the study protocol and ICF, respectively. Roche monitors and auditors will have direct access to appropriate parts of records relating to patients participating in this study for the purposes of verifying the data provided to Roche. The site will permit monitoring, audits, Institutional Review Board/Independent Ethics Committee (IRB/IEC) review, and regulatory inspections by providing direct access to source data and documents related to the RCR Research Project.

## 17. CONFIDENTIALITY OF TRIAL DOCUMENTS AND SUBJECT RECORDS

The Investigator must assure that patients' anonymity will be maintained and that their identities are protected from unauthorized parties. On CRFs or other documents submitted to the Sponsor, patients should not be identified by their names, but by an identification code. The Investigator should keep a patient enrollment log showing codes, names and addresses. The Investigator should maintain documents not for submission to Roche, e.g., patients' written consent forms, in strict confidence.

Roche already maintains rigorous confidentiality standards for clinical studies by "coding" (i.e., assigning a unique patient ID number at the Investigator site) all patients enrolled in Roche clinical studies. This means that patient names are not included in data sets that are transmitted to any Roche location. Given the sensitive nature of genetic data, Roche has implemented a number of additional processes to assure patient confidentiality. All specimens taken for inherited genetic research that will be stored in the RCR (see Section 5.5) undergo a second level of "coding". At Roche, the specimen is transferred to a new tube and labeled with a new random number. This is referred to as "Double Coding (De-Identification)". Data generated following the use of these specimens and all clinical data transferred from the clinical study database and considered relevant, will also be labeled with this same code. The "linking key" between the participant's identification number and this new independent code will be stored in a secure database system. Access to the table linking the participant identification number to the specimen code will be strictly limited and monitored by audit trail. Legitimate operational reasons for accessing the "linking key" will be documented in a standard operating procedure. Access to the "linking key" for any other reason will require written approval from the Governance Committee responsible for the specimen(s).

## 18. Clinical Study Report (CSR)

A clinical study report will be written and distributed to Health Authorities as required by applicable regulatory requirements.

## 19. Publication of Data and Protection of Trade Secrets

Roche will comply with the requirements for publication of study results.

The results of this study may be published or presented at scientific meetings. If this is foreseen, the Investigator agrees to submit all manuscripts or abstracts to Roche prior to submission. This allows the Sponsor to protect proprietary information and to provide comments based on information from other studies that may not yet be available to the Investigator.

In accordance with standard editorial and ethical practice, Roche will generally support publication of multicenter trials only in their entirety and not as individual center data. In this case, a coordinating Investigator will be designated by mutual agreement.

Authorship will be determined by mutual agreement and in line with International Committee of Medical Journal Editors (ICMJE) authorship requirements. Any formal publication of the study in which contribution of Roche personnel exceeded that of conventional monitoring will be considered as a joint publication by the Investigator and the appropriate Roche personnel.

Data derived from RCR specimen analysis on individual patients will not be provided to study Investigators, except where explicitly stipulated in a study protocol (e.g., if the result is an enrollment criterion). Exceptions may be granted (e.g., if biomarker data would be linked to safety issues). The aggregate results of any research conducted using RCR specimens will be available in accordance with the effective Roche policy on study data publication.

Any inventions and resulting patents, improvements and / or know- how originating from the use of the RCR will become and remain the exclusive and unburdened property of Roche, except where agreed otherwise.

## Appendix 1: AEs Categories for Determining Relationship to Test Drug

The causality relationship of study drug to the adverse event will be assessed by the Investigator as either: Yes or No.

If there is a reasonable suspected causal relationship to the study medication, i.e., there are facts (evidence) or arguments to suggest a causal relationship, drug-event relationship should be assessed as Yes.

**The following criteria should be considered in order to assess the relationship as Yes:**

- Reasonable temporal association with drug administration
- It may or may not have been produced by the subject's clinical state, environmental or toxic factors, or other modes of therapy administered to the subject.
- Known response pattern to suspected drug
- Disappears or decreases on cessation or reduction in dose
- Reappears on rechallenge

**The following criteria should be considered in order to assess the relationship as No:**

- It does not follow a reasonable temporal sequence from administration of the drug.
- It may readily have been produced by the subject's clinical state, environmentalor toxic factors, or other modes of therapy administered to the subject.
- It does not follow a known pattern of response to the suspected drug.
- It does not reappear or worsen when the drug is readministered.

## Appendix 2: ICH Guidelines for Clinical Safety Data Management, Definitions and Standards for Expedited Reporting, Topic E2

A serious adverse event is any experience that suggests a significant hazard, contraindication, side effect or precaution. It is any AE that at any dose fulfills at least one of the following criteria:

– is fatal; [results in death] [**NOTE**: death is an outcome, not an event]

– is Life-Threatening [**NOTE**: the term "Life-Threatening" refers to an event in which the patient was at immediate risk of death at the time of the event; it does not refer to an event which could hypothetically have caused a death had it been more severe]

– requires in-patient hospitalization or prolongation of existing hospitalization

– results in persistent or significant disability/incapacity

– is a congenital anomaly/birth defect

– is medically significant or requires intervention to prevent one or other of the outcomes listed above.

Medical and scientific judgment should be exercised in deciding whether expedited reporting to the Sponsor is appropriate in other situations, such as important medical events that may not be immediately life-threatening or result in death or hospitalization but may jeopardize the patient or may require intervention to prevent one of the outcomes listed in the definitions above. These situations should also usually be considered serious.

Examples of such events are intensive treatment in an emergency room or at home for allergic bronchospasm; blood dyscrasias or convulsions that do not result in hospitalization; or development of drug dependency or drug abuse.

An unexpected AE is one in which the nature or severity is not consistent with the applicable product information.

Causality is initially assessed by the Investigator. For Serious Adverse Events, possible causes of the event are indicated by selecting one or more options. (Check all that apply)

– Pre-existing/Underlying disease - specify

– Study treatment - specify the drug(s) related to the event

– Other treatment (concomitant or previous) - specify

– Protocol-related procedure

– Other (e.g., accident, new or intercurrent illness) - specify

The term severe is a measure of intensity, thus a severe AE is not necessarily serious. For example, nausea of several hours' duration may be rated as severe, but may not be clinically serious.

## Appendix 2: ICH Guidelines for Clinical Safety Data Management, Definitions and Standards for Expedited Reporting, Topic E2 (Cont.)

A serious adverse event occurring during the study or which comes to the attention of the Investigator within 15 days after stopping the treatment or during the protocol-defined follow-up period, if this is longer, whether considered treatment-related or not, must be reported. In addition, a serious adverse event that occurs after this time, if considered related to test "drug", should be reported.

Such preliminary reports will be followed by detailed descriptions later which will include copies of hospital case reports, autopsy reports and other documents when requested and applicable.

For serious adverse events, the following must be assessed and recorded on the AEs eform of the eCRF: intensity, relationship to test substance, action taken, and outcome to date.

The Investigator must notify the Ethics Review Committee/Institutional Review Board of a serious adverse event in writing as soon as is practical and in accordance with international and local laws and regulations.

ROCHE LOCAL COUNTRY CONTACT for SAEs: Local Monitor:

See attached *Protocol Administrative and Contact Information & List of Investigators Form,[ gcp_for000227]*, for details of administrative and contact information.

ROCHE HEADQUARTERS CONTACT for SAEs and other medical emergencies: Clinical Operations/Clinical Science:

See attached *Protocol Administrative and Contact Information & List of Investigators form,[gcp_for000227]*, for details of administrative and contact information.

24 HOUR MEDICAL COVERAGE:

Identification of a contact for 24 Hour Medical Coverage is mandatory to be compliant with worldwide regulatory agencies and to ensure the safety of study patients.

An Emergency Medical Call Center Help Desk will access the Roche Medical Emergency List, escalate emergency medical calls, provide medical translation service (if necessary), connect the Investigator with the Roche medical contact for this study and track all calls. The Emergency Medical Call Center Help Desk will be manned 24 hours 7 days a week. Toll free numbers will be distributed to all Investigators running Roche Pharma Development clinical trials. The Help Desk will be used for medical emergencies outside regular business hours, or when the regular Clinical Science Leader cannot be reached.

See the attached *Protocol Administrative and Contact Information & List of Investigators form [gcp_for000227]*, for details of administrative, contact information, and Emergency Medical Call Center Help Desk toll-free numbers.

## Appendix 3: Common Terminology Criteria (CTCAE)

In the present study, toxicities will be graded according to the Common Terminology Criteria for Adverse Events (CTCAE), version 4.0.

The Common Terminology Criteria for Adverse Events v4.0 (CTCAE) can be found in the Roche hand-out entitled: " Common Terminology Criteria for Adverse Events v4.0" or via the following web-site: http://ctep.cancer.gov

## Appendix 4: Telephone Interviews

The purpose of this interview is to identify any new or worsening neurological symptoms that warrant an unscheduled visit and to collect data on possible events of infections. Telephone interviews should be performed by study personnel every 4 weeks between clinic visits (with exemption of prolonged B-cell monitoring period when telephone interviews need to be performed every 12 weeks) – see also Section 5.3.4.4.

**Please ask the following questions and record patient's answers during the Telephone Interview:**

| Questions | No | Yes |
|---|---|---|
| **1. Since your last visit or telephone interview, have you had any new or worsening medical problems (such as sudden changes in your thinking, alterations in your behavior, visual disturbances, extremity weakness, limb coordination problems, or gait abnormalities) that have persisted over several days?** | | |
| **2. Since your last visit or telephone interview, have you taken any new medicines to treat cancer or MS or any other new medicines that weaken your immune system?** | | |
| **3. Since your last visit or telephone interview, other than for the treatment of a recent relapse, have you taken any of the following medicines: methylprednisone, (e.g., Depo-Medrol®, Solu-Medrol®), dexamethasone (e.g., Decadron®), prednisolone, or other steroid medicine?** | | |
| **4. Since your last visit or telephone interview, have you had any signs or symptoms of infection?** | | |

If the patient answered YES to any question, contact the Treating Investigator and review the patient's answers. The Investigator can determine if an unscheduled visit is required.

**Record any pertinent comments made by the patient during the interview:**

_______________________________________________

_______________________________________________

_______________________________________________

NAME:______________________________ Date:______________________________
*Name of person completing the telephone interview*

## Appendix 4: Telephone Interviews (Cont.)

**Below is a sample list of medications that can weaken the immune system. This list does not include all drugs that can suppress the immune system.**

**Approved MS Therapies:**

Glatiramer acetate (Copaxone®)

Interferon β-1a (Rebif®, AVONEX®)

Interferon β-1b (Betaseron®)

Mitoxantrone (Novantrone®)

Natalizumab (Tysabri®)

Fingolimod (Gilenya®) – if relevant

**Immunosuppressants/Antineoplastics:**

Azathioprine (Imuran®, Azasan®)

Cladribine (Leustatin®)

Cyclophosphamide (Cytoxan®, Neosar®)

Cyclosporine (Sandimmune®, Neoral®)

Fludarabine phosphate (Fludara®)

Leflunomide (Arava®)

Mercaptopurine (Purinethol®)

Methotrexate (Methotrex®, Rheumatrex®, Trexall®)

Mycophenolate mofetil (CellCept®)

Pemetrexed (Alimta®)

**Additional Immunomodulators and Immunosuppressants:**

Other interferons (Actimmune®, Infergen®, Intron® A,

Pegasys®, PEG-Intron®, Rebetron®, Roferon®-A)

Adalimumab (Humira®)

Alefacept (Amevive®)

Alemtuzumab (Campath®)

Anakinra (Kineret®)

Daclizumab (Zenapax®)

Etanercept (Enbrel®)

Infliximab (Remicade®)

Intravenous immunoglobulin (IVIG)

Ofatumumab (Arzerra®)

Rituximab (Rituxan/MabThera®)

Trastuzumab (Herceptin®)

## Appendix 5: Modified Fatigue Impact Scale (MFIS)

MFIS

### MODIFIED FATIGUE IMPACT SCALE (MFIS)

Following is a list of statements that describe how fatigue may affect a person. Fatigue is a feeling of physical tiredness and lack of energy that many people experience from time to time. In medical conditions like MS, feelings of fatigue can occur more often and have a greater impact than usual. Please read each statement carefully, and then SELECT THE ONE NUMBER that best indicates how often fatigue has affected you in this way during the PAST 4 WEEKS. (If you need help in marking your responses, TELL THE INTERVIEWER THE NUMBER of the best response.) PLEASE ANSWER EVERY QUESTION. If you are not sure which answer to select, please choose the one answer that comes closest to describing you. The interviewer can explain any words or phrases that you do not understand.

| Because of my fatigue during the PAST 4 WEEKS.... | Never | Rarely | Sometimes | Often | Almost always |
|---|---|---|---|---|---|
| * 1. I have been less alert. | 0 | 1 | 2 | 3 | 4 |
| * 2. I have had difficulty paying attention for long periods of time. | 0 | 1 | 2 | 3 | 4 |
| * 3. I have been unable to think clearly. | 0 | 1 | 2 | 3 | 4 |
| * 4. I have been clumsy and uncoordinated. | 0 | 1 | 2 | 3 | 4 |
| * 5. I have been forgetful. | 0 | 1 | 2 | 3 | 4 |
| * 6. I have had to pace myself in my physical activities. | 0 | 1 | 2 | 3 | 4 |

Back | Next

## Appendix 5: Modified Fatigue Impact Scale (MFIS) (Cont.)

MFIS

**MODIFIED FATIGUE IMPACT SCALE (MFIS)**

| Because of my fatigue during the PAST 4 WEEKS.... | Never | Rarely | Sometimes | Often | Almost always |
|---|---|---|---|---|---|
| * 7. I have been less motivated to do anything that requires physical effort. | ○ 0 | ○ 1 | ○ 2 | ○ 3 | ○ 4 |
| * 8. I have been less motivated to participate in social activities. | ○ 0 | ○ 1 | ○ 2 | ○ 3 | ○ 4 |
| * 9. I have been limited in my ability to do things away from home. | ○ 0 | ○ 1 | ○ 2 | ○ 3 | ○ 4 |
| * 10. I have had trouble maintaining physical effort for long periods. | ○ 0 | ○ 1 | ○ 2 | ○ 3 | ○ 4 |
| * 11. I have had difficulty making decisions. | ○ 0 | ○ 1 | ○ 2 | ○ 3 | ○ 4 |
| * 12. I have been less motivated to do anything that requires thinking. | ○ 0 | ○ 1 | ○ 2 | ○ 3 | ○ 4 |
| * 13. my muscles have felt weak. | ○ 0 | ○ 1 | ○ 2 | ○ 3 | ○ 4 |
| * 14. I have been physically uncomfortable. | ○ 0 | ○ 1 | ○ 2 | ○ 3 | ○ 4 |

Back | Next

# Appendix 5: Modified Fatigue Impact Scale (MFIS) (Cont.)

MFIS

## MODIFIED FATIGUE IMPACT SCALE (MFIS)

| Because of my fatigue during the PAST 4 WEEKS.... | Never | Rarely | Sometimes | Often | Almost always |
|---|---|---|---|---|---|
| * 15. I have had trouble finishing tasks that require thinking. | 0 | 1 | 2 | 3 | 4 |
| * 16. I have had difficulty organizing my thoughts when doing things at home or at work. | 0 | 1 | 2 | 3 | 4 |
| * 17. I have been less able to complete tasks that require physical effort. | 0 | 1 | 2 | 3 | 4 |
| * 18. my thinking has been slowed down. | 0 | 1 | 2 | 3 | 4 |
| * 19. I have had trouble concentrating. | 0 | 1 | 2 | 3 | 4 |
| * 20. I have limited my physical activities. | 0 | 1 | 2 | 3 | 4 |
| * 21. I have needed to rest more often or for longer periods. | 0 | 1 | 2 | 3 | 4 |

Back Next

## Appendix 6: The Center for Epidemiologic Studies Depression Scale (CES-D)

**CES-D Scale**

### Center for Epidemiologic Studies Depression Scale (CES-D, NIMH)

Below is a list of the ways you might have felt or behaved. Please tell me how often you have felt this way in the last week.

| | During the Past Week | | | |
|---|---|---|---|---|
| | Rarely or none of the time (less than 1 Day) | Some or a little of the time (1-2 days) | Occasionally or a moderate amount of time (3-4 days) | Most or all of the time (5-7 days) |
| * 1. I was bothered by things that usually don't bother me. | ○ | ○ | ○ | ○ |
| * 2. I did not feel like eating: my appetite was poor. | ○ | ○ | ○ | ○ |
| * 3. I felt that I could not shake off the blues even with help from my family or friends. | ○ | ○ | ○ | ○ |
| * 4. I felt that I was just as good as other people. | ○ | ○ | ○ | ○ |
| * 5. I had trouble keeping my mind on what I was doing. | ○ | ○ | ○ | ○ |
| * 6. I felt depressed. | ○ | ○ | ○ | ○ |
| * 7. I felt that everything I did was an effort. | ○ | ○ | ○ | ○ |
| * 8. I felt hopeful about the future. | ○ | ○ | ○ | ○ |
| * 9. I thought my life had been a failure. | ○ | ○ | ○ | ○ |
| * 10. I felt fearful. | ○ | ○ | ○ | ○ |

Back | Next

**CES-D Scale**

## Center for Epidemiologic Studies Depression Scale (CES-D, NIMH)

Below is a list of the ways you might have felt or behaved. Please tell me how often you have felt this way in the last week.

| | During the Past Week | | | |
|---|---|---|---|---|
| | Rarely or none of the time (less than 1 Day) | Some or a little of the time (1-2 days) | Occasionally or a moderate amount of time (3-4 days) | Most or all of the time (5-7 days) |
| * 11. My sleep was restless. | ○ | ○ | ○ | ○ |
| * 12. I was happy. | ○ | ○ | ○ | ○ |
| * 13. I talked less than usual. | ○ | ○ | ○ | ○ |
| * 14. I felt lonely. | ○ | ○ | ○ | ○ |
| * 15. People were unfriendly. | ○ | ○ | ○ | ○ |
| * 16. I enjoyed life. | ○ | ○ | ○ | ○ |
| * 17. I had crying spells. | ○ | ○ | ○ | ○ |
| * 18. I felt sad. | ○ | ○ | ○ | ○ |
| * 19. I felt that people dislike me. | ○ | ○ | ○ | ○ |
| * 20. I could not get "going". | ○ | ○ | ○ | ○ |

SCORING: zero for answers in the first column, 1 for answers in the second column, 2 for answers in the third column, 3 for answers in the fourth column. The scoring of positive items is reversed. Possible range of scores is zero to 60, with the higher scores indicating the presence of more symptomatology.

Back | Next

# Appendix 7: The Short Form (SF-36) Health Survey

**SF-36**

## Your Health and Well-Being

This survey asks for your views about your health. This information will help keep track of how you feel and how well you are able to do your usual activities. Thank you for completing this survey!

For each of the following questions, please SELECT the response that best describes your answer.

*** 1. In general, would you say your health is:**

| Excellent | Very good | Good | Fair | Poor |
|---|---|---|---|---|
| ◯ 1 | ◯ 2 | ◯ 3 | ◯ 4 | ◯ 5 |

*** 2. COMPARED TO ONE YEAR AGO, how would you rate your health in general NOW?**

| Much better now than one year ago | Somewhat better now than one year ago | About the same as one year ago | Somewhat worse now than one year ago | Much worse now than one year ago |
|---|---|---|---|---|
| ◯ 1 | ◯ 2 | ◯ 3 | ◯ 4 | ◯ 5 |

SF-36v2® Health Survey © 1992, 2002, 2009 Medical Outcomes Trust and QualityMetric Incorporated. All rights reserved.
SF-36® is a registered trademark of Medical Outcomes Trust.

Back | Next

## SF-36

3. The following questions are about activities you might do during a typical day. Does YOUR HEALTH NOW LIMIT YOU in these activities? If so, how much?

| | Yes, limited a lot | Yes, limited a little | No, not limited at all |
|---|---|---|---|
| * a. VIGOROUS ACTIVITIES, such as running, lifting heavy objects, participating in strenuous sports. | ◯ 1 | ◯ 2 | ◯ 3 |
| * b. MODERATE ACTIVITIES, such as moving a table, pushing a vacuum cleaner, bowling, or playing golf. | ◯ 1 | ◯ 2 | ◯ 3 |
| * c. Lifting or carrying groceries. | ◯ 1 | ◯ 2 | ◯ 3 |
| * d. Climbing SEVERAL flights of stairs. | ◯ 1 | ◯ 2 | ◯ 3 |
| * e. Climbing ONE flight of stairs. | ◯ 1 | ◯ 2 | ◯ 3 |
| * f. Bending, kneeling, or stooping. | ◯ 1 | ◯ 2 | ◯ 3 |
| * g. Walking MORE THAN A MILE. | ◯ 1 | ◯ 2 | ◯ 3 |
| * h. Walking SEVERAL HUNDRED YARDS. | ◯ 1 | ◯ 2 | ◯ 3 |
| * i. Walking ONE HUNDRED YARDS. | ◯ 1 | ◯ 2 | ◯ 3 |
| * j. Bathing or dressing yourself. | ◯ 1 | ◯ 2 | ◯ 3 |

SF-36v2® Health Survey © 1992, 2002, 2009 Medical Outcomes Trust and QualityMetric Incorporated. All rights reserved.
SF-36® is a registered trademark of Medical Outcomes Trust.

**Back** **Next**

**SF-36**

**4. During the PAST 4 WEEKS, how much of the time have you had any of the following problems with your work or other regular daily activities AS A RESULT OF YOUR PHYSICAL HEALTH?**

| | All of the time | Most of the time | Some of the time | A little of the time | None of the time |
|---|---|---|---|---|---|
| * a. Cut down on the AMOUNT OF TIME you spent on work or other activities. | ○ 1 | ○ 2 | ○ 3 | ○ 4 | ○ 5 |
| * b. ACCOMPLISHED LESS than you would like. | ○ 1 | ○ 2 | ○ 3 | ○ 4 | ○ 5 |
| * c. Were limited in the KIND of work or other activities. | ○ 1 | ○ 2 | ○ 3 | ○ 4 | ○ 5 |
| * d. Had DIFFICULTY performing the work or other activities (for example, it took extra effort). | ○ 1 | ○ 2 | ○ 3 | ○ 4 | ○ 5 |

**5. During the PAST 4 WEEKS, how much of the time have you had any of the following problems with your work or other regular daily activities AS A RESULT OF ANY EMOTIONAL PROBLEMS (such as feeling depressed or anxious)?**

| | All of the time | Most of the time | Some of the time | A little of the time | None of the time |
|---|---|---|---|---|---|
| * a. Cut down on the AMOUNT OF TIME you spent on work or other activities. | ○ 1 | ○ 2 | ○ 3 | ○ 4 | ○ 5 |
| * b. ACCOMPLISHED LESS than you would like. | ○ 1 | ○ 2 | ○ 3 | ○ 4 | ○ 5 |
| * c. Did work or other activities LESS CAREFULLY THAN USUAL. | ○ 1 | ○ 2 | ○ 3 | ○ 4 | ○ 5 |

SF-36v2® Health Survey © 1992, 2002, 2009 Medical Outcomes Trust and QualityMetric Incorporated. All rights reserved.
SF-36® is a registered trademark of Medical Outcomes Trust.

**Back** **Next**

**SF-36**

* 6. During the PAST 4 WEEKS, to what extent has your PHYSICAL HEALTH OR EMOTIONAL PROBLEMS interfered with your normal social activities with family, friends, neighbors, or groups?

| Not at all | Slightly | Moderately | Quite a bit | Extremely |
|---|---|---|---|---|
| ○ 1 | ○ 2 | ○ 3 | ○ 4 | ○ 5 |

* 7. How much BODILY pain have you had during the PAST 4 WEEKS?

| None | Very mild | Mild | Moderate | Severe | Very severe |
|---|---|---|---|---|---|
| ○ 1 | ○ 2 | ○ 3 | ○ 4 | ○ 5 | ○ 6 |

* 8. During the PAST 4 WEEKS, how much did PAIN interfere with your normal work (including both work outside the home and housework)?

| Not at all | A little bit | Moderately | Quite a bit | Extremely |
|---|---|---|---|---|
| ○ 1 | ○ 2 | ○ 3 | ○ 4 | ○ 5 |

SF-36v2® Health Survey © 1992, 2002, 2009 Medical Outcomes Trust and QualityMetric Incorporated. All rights reserved.
SF-36® is a registered trademark of Medical Outcomes Trust.

Back | Next

**SF-36**

9. These questions are about how you feel and how things have been with you DURING THE PAST 4 WEEKS. For each question, please give the one answer that comes closest to the way you have been feeling. How much of the time during the PAST 4 WEEKS...

| | All of the time | Most of the time | Some of the time | A little of the time | None of the time |
|---|---|---|---|---|---|
| * a. Did you feel full of life? | ○ 1 | ○ 2 | ○ 3 | ○ 4 | ○ 5 |
| * b. Have you been very nervous? | ○ 1 | ○ 2 | ○ 3 | ○ 4 | ○ 5 |
| * c. Have you felt so down in the dumps that nothing could cheer you up? | ○ 1 | ○ 2 | ○ 3 | ○ 4 | ○ 5 |
| * d. Have you felt calm and peaceful? | ○ 1 | ○ 2 | ○ 3 | ○ 4 | ○ 5 |
| * e. Did you have a lot of energy? | ○ 1 | ○ 2 | ○ 3 | ○ 4 | ○ 5 |
| * f. Have you felt downhearted and depressed? | ○ 1 | ○ 2 | ○ 3 | ○ 4 | ○ 5 |
| * g. Did you feel worn out? | ○ 1 | ○ 2 | ○ 3 | ○ 4 | ○ 5 |
| * h. Have you been happy? | ○ 1 | ○ 2 | ○ 3 | ○ 4 | ○ 5 |
| * i. Did you feel tired? | ○ 1 | ○ 2 | ○ 3 | ○ 4 | ○ 5 |

SF-36v2® Health Survey © 1992, 2002, 2009 Medical Outcomes Trust and QualityMetric Incorporated. All rights reserved.
SF-36® is a registered trademark of Medical Outcomes Trust.

Back | Next

**SF-36**

* 10. During the PAST 4 WEEKS, how much of the time has your PHYSICAL HEALTH OR EMOTIONAL PROBLEMS interfered with your social activities (like visiting with friends, relatives, etc.)?

| All of the time | Most of the time | Some of the time | A little of the time | None of the time |
|---|---|---|---|---|
| ○ 1 | ○ 2 | ○ 3 | ○ 4 | ○ 5 |

11. How TRUE or FALSE is EACH of the following statements for you?

| | Definitely true | Mostly true | Don't know | Mostly false | Definitely false |
|---|---|---|---|---|---|
| * a. I seem to get sick a little easier than other people. | ○ 1 | ○ 2 | ○ 3 | ○ 4 | ○ 5 |
| * b. I am as healthy as anybody I know. | ○ 1 | ○ 2 | ○ 3 | ○ 4 | ○ 5 |
| * c. I expect my health to get worse. | ○ 1 | ○ 2 | ○ 3 | ○ 4 | ○ 5 |
| * d. My health is excellent. | ○ 1 | ○ 2 | ○ 3 | ○ 4 | ○ 5 |

SF-36v2® Health Survey © 1992, 2002, 2009 Medical Outcomes Trust and QualityMetric Incorporated. All rights reserved.
SF-36® is a registered trademark of Medical Outcomes Trust.

Back | Next

## Appendix 8: Optical Coherence Tomography Exploratory Substudy

*Introduction*

Optical coherence tomography (OCT) is a noninvasive imaging tool capable of sensitive, reproducible and rapid measuruments of structural architecture of the retina and retinal nerve fiber layer [1]. OCT can be of particular interest in multiple sclerosis (MS), because optic neuritis is often the pivotal event in establishing the diagnosis of MS. Optic nerve dysfunction is characterized by optic disc pallor and loss of contrast sensitivity, and visual field defects, which may occur subclinically in many patients. It is estimated that nearly 20% of all patients with MS present initially with optic neuritis, and an additional 30% – 100% will have optic neuritis at some point in their disease course [2]. OCT outcome measures such as retinal nerve fiber layer (RNFL) thickness and macular volumes have been shown to correlate with clinical measures of vision loss and may facilitate visualization of any process of neurodegeneration or repair as part of natural history of MS or as a consequence of neuroprotective interventions [3].

This substudy is part of three ongoing Phase III studies that serve to evaluate the neuroprotective effect of ocrelizumab in MS as measured by RNFL thickness and macular volume in both eyes of patients who participate in the confirmatory pivotal studies in patients with relapsing multiple sclerosis (RMS; Studies WA21092 and WA21093) or patients with primary progressive multiple sclerosis (PPMS; Study WA25046).

***Objectives***

***Efficacy Objectives***

The primary efficacy objectives for this substudy are as follows:

- To evaluate the neuroprotective effect of ocrelizumab therapy as measured by macular volume and RNFL over time
- To characterize the time-course of changes in RNFL that imply axonal loss in patients with both RMS and PPMS with or without ocrelizumab treatment

The secondary efficacy objectives for this study are as follows:

- In the case of RMS patients (Studies WA21092 and WA21093), the study will assess whether ocrelizumab 600 mg has superior neuroprotective effect compared with Rebif® as measured by RNFL thickness.
- In the case of PPMS patients (Study WA25046), the study will assess whether ocrelizumab 600 mg has superior neuroprotective effect compared with placebo as measured by RNFL thickness.

***Safety Objectives***

The safety objectives for this study are as follows:

- To evaluate the ophthalmological safety of ocrelizumab therapy in patients with MS, focusing on serious adverse events (SAEs)

***Exploratory Objectives***

The exploratory objectives for this study are as follows:

- To evaluate if OCT outcomes can serve as a reliable and predictive measure of response to ocrelizumab therapy in MS patients or progression to a more severe disease state
- To evaluate the relationship of OCT outcomes with outcomes from ocrelizumab Phase III pivotal studies, such as
  - Change in brain volume as measured by brain magnetic resonance imaging (MRI)
  - *Confirmed* disability progression
  - T2 lesion volume
  - Number of T1 Gd-enhanced lesions
  - Change in Multiple Sclerosis Functional Composite Scale score

***<u>Study Design</u>***

***Description of Study***

*<u>Overview</u>*

The current substudy is an add-on, multicenter, longitudinal study to the ongoing Phase III ocrelizumab Studies WA21092, WA21093, and WA25046 to evaluate the neuroprotective effects of ocrelizumab treatment as measured by OCT. OCT will be performed in parallel to the ocrelizumab pivotal Phase III studies.

Patients can be enrolled at any time during the first 48 weeks after enrollment in the main pivotal studies. However, all attempts should be made to enroll the patient at the time of screening of the main pivotal study. Patients will undergo an ophthalmological examination prior to first OCT scan and at the end of the study. Patients will also undergo at least three OCT scans at 24-week intervals (see schedule of assessments of this substudy, Appendix A).

For patients participating in Studies WA21092 and WA21093:

- The patient should undergo OCT measurement every 24 weeks after the first OCT visit (Visit 1 of the OCT substudy). If the patient is enrolled into the OCT substudy at baseline of the main study, the following visits should occur: Visit 1 (Week 0; occurring at the baseline visit of the main study), Visit 2 (Week 24; occurring at Visit 5 of the main study), Visit 3 (Week 48; occurring at Visit 7 of the main study), Visit 4 (Week 72; occurring at Visit 9 of the main study) and Visit 5 (Week 96; occurring at Visit 11 of the main study). If the patient is enrolled after baseline of the main study, OCT visits should occur every 24 weeks after the first OCT visit (Visit 1, Week 0).

For patients participating in Study WA25046:

- The patient should undergo OCT measurement every 24 weeks after the first OCT visit (Visit 1 of the OCT substudy). If the patient is enrolled into the OCT substudy at baseline of the main study, the following visits should occur: Visit 1 (Week 0; occurring at the baseline visit of the main study), Visit 2 (Week 24, occurring at Visit 5 of the main study), Visit 3 (Week 48, occurring at Visit 8 of the main study), Visit 4 (Week 72, occurring at Visit 11 of the main study), Visit 5 (Week 96, occurring at Visit 14 of the main study), and Visit 6 (Week 120, occurring at Visit 17 of the main study). If the patient is enrolled after baseline of the main study, OCT visits should occur every 24 weeks after the first OCT visit (Visit 1, Week 0).

If the patient is withdrawn from study treatment in the main protocol, an OCT visit should occur if it has not been performed during the previous 4 weeks. Patients should then have an OCT measurement at the end of the safety follow-up of the main protocol.

If a patient decides to participate in the Open-Label Extension Phase of the main protocol, OCT measurements should continue to occur every 24 weeks during the OLE Phase.

A schedule of assessments is provided in Appendix A of this substudy.

*Independent Review Committee (IRC)*

A masked, central OCT reading center will review and analyze OCT endpoints. The Independent Review Committee (IRC) membership and procedures will be detailed in an IRC charter.

***End of Study***

The end of the study is defined as the date when the last patient, last visit (LPLV) of this substudy occurs. LPLV of this substudy is expected to occur at the same time of LPLV of the last ongoing Phase III pivotal ocrelizumab studies (i.e., either WA21092, WA21093, or WA25046), or earlier.

### *Outcome Measures*

*Efficacy Outcome Measures*

The efficacy outcome measures for this study are as follows:

- Overall and quadrant RNFL thickness measured by OCT
- Macular volume maps as measured by OCT

*Safety Outcome Measures*

The safety outcome measures for this study are as follows:

- Incidence, nature, and severity of ophthalmological adverse events (AEs)

### ***Materials and Methods***

### *Patients*

Adult patients who fulfill eligibility criteria for one of the main Phase III ocrelizumab pivotal studies (i.e., WA21092, WA21093, or WA25046) and the eligibility criteria outlined in the following inclusion and exclusion sections of this substudy can be enrolled into the study.

*Inclusion Criteria*

Patients must meet the following criteria for study entry:

- Able and willing to provide written informed consent and comply with the study protocol
- Be a participant in one of the following studies: WA21092, WA21093, or WA25046

*Exclusion Criteria*

Patients who meet any of the following criteria will be excluded from study entry:

- Medical history of macular degeneration, retinopathy, glaucoma, amblyopia, diabetes, or any other documented cause of vision loss
- Inability to undergo reliable OCT testing
- More than 48 weeks have lapsed since randomization

***Study Assessments***

*Screening and Baseline Examination and Eligibility Screening Form*

All patients must sign and date the most current Institutional Review Board/Institutional Ethics Committee's approved written informed consent before any study specific assessments or procedures are performed.

Consenting patients must also have signed the informed consent and be eligible for the main pivotal Phase III study. Patients will receive an ophthalmological and eye examination to be evaluated for eligibility to participate in this substudy. If the patient is eligible, this ophthalmological examination will be considered the baseline ophthalmological measure (OCT Visit 1).

An Eligibility Screening Form documenting the Investigator's assessment of each screened patient with regard to the protocol's inclusion and exclusion criteria is to be completed by the Investigator.

It should be stated in the medical record that the patient is participating in this clinical study.

Once a patient has fulfilled all eligibility criteria, he or she will undergo OCT according to schedule of assessments (Appendix A of this substudy).

*Procedure in Case of Delayed Dosing Visit, Relapse or Unscheduled Visits, and Withdrawal Visits in the Main Phase III Ocrelizumab Pivotal Studies*

The main ocrelizumab pivotal studies have mechanisms set up regarding delayed dosing visits, unscheduled visits due to relapse, and withdrawal visits.

OCT measurements should occur at initially scheduled times and should not be rescheduled due to delayed dosing schedule or unscheduled visit due to relapse. In case there is a relapse during an OCT schedule*d* visit, the Investigator should document all ocular symptoms, including ocular neuritis, in the electronic Case Report Form as well as communicate them to the Principal Investigator of the main study. If a patient suffers a relapse with symptoms acute optic neuritis during the month before OCT study, a second OCT must be performed 1 month later to account for the effect of papilledema.

If the patient is withdrawn from study treatment in the main protocol, an OCT visit should occur if it has not been performed during the previous 4 weeks. Patients should then have an OCT measurement every 24 weeks until the end of the safety follow up of the main protocol.

It is possible that the patient withdraws from only the OCT Exploratory substudy. In this case, an OCT visit should occur if it has not been performed during the previous 4 weeks.

*Description of Study Assessments*

*Medical History and Demographic Data*

Medical history and demographic data will be captured in main study in which the patient is enrolled. Further medical history includes clinically significant diseases that may cause vision loss of the affected eye that have been documented by the ophthalmologist.

*Ophthalmological and Eye Examination.*

The presence of any visual abnormalities will be established in a full eye exam. This will include an examination performed by an ophthalmologist during Visit 1 (Week 0) and completion/withdrawal visit. This will include best corrected visual acuity evaluation (with ETDRS standardized eye chart); color vision test (Hardy Rand Rittler pseudoisochormatic plate); visual field measurement; intraocular pressure; slitlamp examination to examine the anterior parts (cornea, lens, and sclera); and a dilated ophthalmoscopic examination of retina, optic nerve, retinal blood vessels, and macula. The eye chart chosen for a patient must be consistently used throughout the study as well as the use of corrective lenses during the ophthalmological exam.

*Visual Evoked Potential*

Visual Evoked Potential (VEP) is a sensitive test of visual pathway function and a marker of optic nerve involvement in MS, and it has been used as a diagnostic tool for RMS and PPMS [4]. It can be used to supplement information provided by a clinical examination to provide objective evidence of a second lesion provided that the only clinically expressed lesion did not affect the visual pathways. VEP measurement should occur during OCT Visit 1 (Week 0). If available at the center, multifocal VEP will be performed instead of full-field VEP.

Note: In the rare cases that the selected site cannot deliver VEP data for any reason, VEP measurements will not be necessary after discussion with the Sponsor. However, all efforts should be made to collect VEP data during Visit 1 (Week 0).

*Optical Coherence Tomography*

OCT images will be acquired in all patients as detailed in the schedule of assessments (Appendix A of this substudy). In addition, an OCT scan will be performed in patients withdrawn from the main study treatment if it has not been performed within the previous 4 weeks.

## Appendix 8: Optical Coherence Tomography Exploratory Substudy (Cont.)

All OCT images will be performed by certified personnel. The following time windows apply:

- OCT Visit 1 (Week 0) should occur prior to baseline visit of the primary study and at Visit 2 (Week 24), Visit 3 (Week 48), Visit 4 (Week 72), and Visit 5 (Week 96).
- In case the patient is enrolled after the baseline visit of main study, OCT Visit 1 should occur at time of enrollment into the substudy. All further OCT scans should occur every 24 weeks thereafter.
- *If a patient enrolls into the Open Label Extension Phase of the main study, OCT assessments should continue to occur every 24 weeks.*

During OCT Visit 1 (Week 0), the OCT scan should be performed twice in order to control for test-retest reliability.

OCT images will be read by a centralized reading center. The reading will be performed in a masked fashion in the absence of clinical information. Further details on the OCT protocols and standardization of machines are described in the separate Independent Review Committee Charter.

*Procedure in Case of Ocular Neuritis or Relapse*

If the patient presents with optic neuritis, the affected eye should be documented. The patient should also be referred to the Principal Investigator of the main study for examination. If the patient suffers a relapse with symptoms of acute optic neuritis during the month before an OCT scan, a second OCT scan must be performed 1 month later to account for the effect of papilledema.

### ***Assessment of Safety***

#### ***Safety Plan***

Any AE regarding ocular findings should be reported to the Principal Investigator of the main study in which the subject is participating, as well as the Sponsor. Please refer to main protocol Section 7 regarding the procedures related to reporting of AEs and SAEs.

### ***Statistical Considerations and Analysis Plan***

Full details of all statistical issues and planned statistical analyses will be specified in the IRC charter.

#### ***Sample Size***

A total of approximately 300 patients will be enrolled in this substudy, and the total study duration will be approximately 96 weeks, depending on the time of enrollment.

## Appendix 8: Optical Coherence Tomography Exploratory Substudy (Cont.)

***Efficacy Analyses***

The purpose of this study is to estimate the neuroprotective effect of ocrelizumab treatment in patients with MS relative to Rebif in the case of Studies WA21092 and WA21093 and relative to placebo in the case of Study WA25046. Point and interval estimates of the decrease of RNFL thickness and macular volume will be obtained.

*Primary Efficacy Endpoint*

The primary efficacy endpoint is the decrease in RNFL thickness over time during the duration of the main study.

*Secondary and Exploratory Efficacy Endpoints*

Secondary and exploratory endpoints are:

- Determination of macular volume over time during the duration of the study
- Exploratory correlation analyses to determine the predictive value of OCT measures (RNFL thickness and macular volume) with outcomes measured in the main study, such as (but not restricted to):
  - Change in brain volume as measured by brain MRI
  - *Confirmed* disability progression
  - T2 lesion volume
  - Number of T1 Gd-enhanced lesions
  - Change in Multiple Sclerosis Functional Composite Scale score

***Safety Analyses***

No safety analyses are planned for this substudy. Any safety event noted during this substudy will be forward to the principal Investigator of the main study. Please refer to Section 7 of the main protocol.

References


1. Frohman EM, Fujimoto JG, Frohman TC, et al. Optical coherence tomography: a window into the mechanisms of multiple sclerosis. Nature Clinical Practice 2008;4:664-75.

2. Sergott RC, Frohman E, Glanzman R, et al. The role of optical coherence tomography in multiple sclerosis: expert panel consensus. J Neurol Sci 2007;263:3-14.

3. Costello F, Hodge W, Pan YI, et al. Tracking retinal nerve fiber layer loss after optic neuritis: a prospective study using optical coherence tomography. Multiple Sclerosis 2008;14:893-905.

4. Polman CH, Reingold SC, Edan G, et al. Diagnostic criteria for multiple sclerosis: 2005 revisions to the "McDonald Criteria". Ann Neurol 2005;58:8406.

## Appendix 8: Optical Coherence Tomography Exploratory Substudy (Cont.)

### Appendix A OPTICAL COHERENCE TOMOGRAPHY EXPLORATORY SUBSTUDY SCHEDULE OF ASSESSMENTS

Schedule of Assessments

| | Treatment Period [a] | | | | | Open-Label Extension Phase (for OPERA study)[e] | Completion Visit | Safety Follow-Up | Withdrawal Visit [a] |
|---|---|---|---|---|---|---|---|---|---|
| Visit | 1 | 2 | 3 | 4 | 5 | | | | |
| Week (window in days) | 0 | 24 (±14) | 48 (±14) | 72 (±14) | 96 (±14) | | | | |
| Informed consent | x | | | | | | | | |
| Ophthalmological and Eye Examination | x | | | | | | x | | x |
| Visual Evoked Potential | x | | | | | | x | | x |
| Medical History | x | | | | | | x | | x |
| Baseline Conditions | x | | | | | | x | | x |
| OCT[b] | x | x | x | x | x | x | x | x[c] | |
| Adverse events [d] | x | x | x | x | x | x | x | x | x |

OCT = optical coherence tomography

a) Treatment period is variable, as it depends on the time of enrollment within the main study.

b) In cases where the initially submitted OCT visit scan is inadequate/not evaluable by Bern Photographic Reading Centre, the site should make all efforts to request the patient to come back to site to perform a new OCT scan.

c) Safety follow-up: OCT assessment should continue every 48 weeks counting after last OCT assessment visit.

d) Any adverse events should be reported to the principal investigator of the study.

*e) If a patient enrolls in the OLE Phase of the main study, OCT assessments should occur every 24 weeks.*

## WA21093: Changes in Conduct of Study or Planned Analyses

There were three protocol amendments to the original Protocol WA21093 Version A released on 27 May 2011.

### Protocol WA21093 Version B

Study Protocol WA21093 Version B was released on 15 June 2012. The main changes to the protocol were as follows:

- Dosing preparation and infusion guidance were revised to simplify the preparation of infusion bags and dosing procedures
- Specific eligibility cutoff values for immunoglobulin M (IgM) and immunoglobulin G (IgG) and the re-treatment criteria for IgG were modified to reflect the central lab reference ranges
- Inclusion/exclusion criteria were revised to:
    - Exclude enrollment of patients who had undergone experimental procedures for treating MS
    - Allow patients who were unable to receive gadolinium contrast for magnetic resonance imagining (MRI) to participate in the study (such patients could participate in the study, but not receive gadolinium during an MRI scan
    - Allow patients to continue previous MS therapies with β-interferons, glatiramer acetate and  other permitted immunomodulatory therapies until randomization
    - Allow patients previously treated with dimethyl fumarate (Tecfidera)to enter the study following an adequate washout period (24 weeks)
    - Allow patients with contraindication to methylprednisolone to enter the study; these patients thus received an equivalent dose of an alternative corticosteroid as premedication prior to ocrelizumab infusion
- Sites were informed of additional, optional sub-studies conducted at select centers in which patients could be eligible to participate

### Protocol WA21093 Version C

Study protocol WA21093 Version C was released on 28 March 2013. The main changes to the protocol were as follows:

- Inclusion of an OLE phase under the same protocol
- Addition of the following exploratory objectives: proportion of disease activity free patients, defined as absence of both relapses and sustained accumulation of disability, and absence of MRI activity by Week 96; evaluation of long term safety and efficacy of ocrelizumab in the OLE study phase
- Clarification on how sustained disability progression is calculated

### Protocol WA21093 Version D

Study Protocol WA21093 Version D was released on 4 September 2014. The main changes to the protocol were as follows:

- An update to the Statistical Considerations and Analytical Plan section of the protocol in line with the SAP amendment to implement European Medicines Agency (EMA) Scientific Advice and to increase statistical rigor. This included:
    - Modifications to the secondary efficacy endpoints, their definitions and hierarchical order
    - Modifications to the exploratory efficacy endpoints and their definitions
    - Modifications to statistical methodology (addition of the Mixed-Effect Model Repeated Measures (MMRM) method)
    - Clarifications to various aspects of the safety analysis, including change to the definition of safety population
    - Change to the timing of database lock to allow for 12-week confirmation of all initial disability progression events during the 96-week comparative treatment period
    - Clarification of the calculation of the baseline EDSS value, the timing of unblinding of the sites and EDSS examining investigators
- Conversion of the optional investigator-sponsored Roche-supported exploratory substudies to Roche-sponsored studies as per current policy of the sponsor
- Revision of the pre-specified mandatory biomarker

# STATISTICAL ANALYSIS PLAN

| | |
|---|---|
| **TITLE:** | **A RANDOMIZED, DOUBLE-BLIND, DOUBLE-DUMMY, PARALLEL-GROUP STUDY TO EVALUATE THE EFFICACY AND SAFETY OF OCRELIZUMAB IN COMPARISON TO INTERFERON BETA-1A (REBIF®) IN PATIENTS WITH RELAPSING MULTIPLE SCLEROSIS** |
| **PROTOCOL NUMBER:** | WA21093 |
| **STUDY DRUG:** | Ocrelizumab |
| **IND NUMBER:** | 100,593 |
| **SPONSOR:** | F.Hoffmann-La Roche Ltd<br>Genentech, Inc. |
| **PLAN PREPARED BY:** | Jiameng Zhang, Ph.D. |
| **DATE FINAL:** | 8 March 2011 |

**CONFIDENTIAL**

This is a Genentech, Inc. document that contains confidential information. Nothing herein is to be disclosed without written consent from Genentech, Inc.

TABLE OF CONTENTS **Page**

1. BACKGROUND .......... 5

2. STUDY DESIGN .......... 6

2.1 Protocol Synopsis .......... 6
2.2 Outcome Measures .......... 6
2.2.1 Primary Efficacy Outcome Measures .......... 6
2.2.2 Secondary Efficacy Outcome Measures .......... 6
2.2.3 Exploratory Efficacy Outcome Measures .......... 6
2.2.4 Pharmacokinetic and Pharmacodynamic Efficacy Outcome Measures .......... 7
2.2.5 Safety Outcome Measures .......... 7
2.3 Determination of Sample Size .......... 7
2.4 Analysis Timing .......... 8

3. STUDY CONDUCT .......... 8

3.1 Randomization Issues .......... 8
3.2 Independent Review Facility .......... 8
3.3 Data Monitoring .......... 8

4. STATISTICAL METHODS .......... 9

4.1 Analysis Populations .......... 9
4.1.1 Intent-to-Treat Population .......... 9
4.1.2 Per Protocol Population .......... 9
4.1.3 Pharmacokinetic Evaluable Population .......... 9
4.1.4 Safety Population .......... 9
4.2 Analysis of Study Conduct .......... 9
4.3 Analysis of Treatment Group Comparability .......... 10
4.3.1 Demography .......... 10
4.3.2 Baseline Disease Characteristics .......... 10
4.4 Efficacy Analysis .......... 11
4.4.1 Primary Efficacy Endpoint .......... 11
4.4.1.1 Significance Level .......... 11
4.4.1.2 Definition .......... 11
4.4.1.3 Analysis Methods .......... 12
4.4.1.4 Sensitivity and Robustness Checks .......... 12
4.4.2 Secondary Efficacy Endpoints .......... 13
4.4.2.1 The Time to Onset of Sustained Disability Progression for at Least 12 Weeks during the 96-Week Comparative Treatment Period .......... 13
4.4.2.2 The Time to Onset of Sustained Disability Progression for at Least 24 Weeks during the 96-Week Comparative Treatment Period .......... 15
4.4.2.3 The Proportion of Relapse-Free Patients by 96 Weeks .......... 15

TABLE OF CONTENTS (cont'd) Page


4.4.2.4 The Change in Total T2 Lesion Volume as Detected by Brain MRI from Baseline to Week 96 ........................................ 15
4.4.2.5 The Total Number of New, and/or Enlarging T2 Hyperintense Lesions as Detected by Brain MRI at Week 24, Week 48, and Week 96 ........................................ 16
4.4.2.6 The Change in Multiple Sclerosis Functional Composite Scale Score from Baseline to Week 96 ........................................ 16
4.4.2.7 The Change in Brain Volume as Detected by Brain MRI from Week 24 to Week 96 ........................................ 18
4.4.3 Exploratory Efficacy Endpoints ........................................ 18
4.4.3.1 The Annualized Relapse Rate, Based on Clinical and Protocol-Defined Relapse at the End of the 96-Week Comparative Treatment Period ........................................ 18
4.4.3.2 The Total Number of T1 Gadolinium-Enhanced Lesions As Detected by Brain MRI at Week 24, 48, and 96 .................... 19
4.4.3.3 The Change in Brain Volume as Detected by Brain MRI from Baseline to Week 96 ........................................ 19
4.4.3.4 The Change in Brain Volume as Detected by Brain MRI from Week 48 to Week 96 ........................................ 19
4.4.3.5 The Change in Multiple Sclerosis Functional Composite Scale (Msfcs) Score from Baseline to Week 48 .......................... 19
4.4.3.6 The Cumulative Change in EDSS Scores, Measured in Area under the Curve (AUC) by Week 96 ................................. 20
4.4.3.7 The Change in Edss from Baseline to Weeks 48 and 96 ............ 20
4.4.3.8 The Change in Timed 25 Foot Walk from Baseline to Weeks 48 and 96 ........................................ 20
4.4.3.9 The Change in 9-Hole Peg Test from Baseline to Weeks 48 and 96 ........................................ 21
4.4.3.10 The Change in Paced Auditory Serial Addition Test (Pasat) from Baseline to Weeks 48 and 96 ................................ 21
4.4.3.11 The Time to Onset of Sustained 20% Increase in 9-Hole Peg Test for at Least 12 Weeks ........................................ 21
4.4.3.12 The Time to Onset of Sustained 20% Increase in Timed 25 Foot Walk for at Least 12 Weeks ........................................ 22
4.4.3.13 Change in the MFIS from Baseline to Weeks 48 and 96 ............ 23
4.4.3.14 Change in the CES-D from Baseline to Weeks 48 and Week 96 ........................................ 24
4.4.3.15 Euro-Quality of Life 5 Dimensions (EQ-5D) Questionnaire ........................................ 24
4.4.3.16 The Short Form (SF-36) Health Survey ...................................... 25
4.4.3.17 Patient Perception of Treatment Questionnaire .......................... 25
4.4.3.18 The Change in Karnofsky Performance Status Scale (Clinician-Reported Version) from Baseline to Weeks 24, 48, 72, and 96 ........................................ 25

## TABLE OF CONTENTS (cont'd) Page


4.4.4 Subgroup Analyses ....26
4.4.5 Data from Safety Follow Up ....26
4.5 Pharmacokinetic and Pharmacodynamic Analyses ....26
4.5.1 Pharmacokinetic Analysis ....26
4.5.2 Pharmacodynamic Analysis ....27
4.6 Safety Analyses ....27
4.6.1 Exposure of Study Medication ....27
4.6.2 Columbia-Suicide Severity Rating Scale (C-SSRS) ....27
4.6.3 Adverse Events ....27
4.6.4 MRI Data ....29
4.6.5 Laboratory Data ....29
4.6.6 Other ....29

5. REFERENCES ....30


## LIST OF APPENDICES


Appendix 1: Protocol Synopsis

# 1. BACKGROUND

A Randomized, Double-Blind, Double-Dummy, Parallel-Group Study to Evaluate the Efficacy and Safety of Ocrelizumab (300 mg×2 q24 weeks) in Comparison to Interferon Beta-1a (Rebif®) in Patients with Relapsing Multiple Sclerosis (RMS).

An ongoing Phase II study (WA21493) in Relapsing Remitting Multiple Sclerosis (RRMS), which was unblinded in November 2009 and has an open label phase which will be completed in 2011, has revealed a rapid effect on B-cell depletion on MRI and clinical activity. After 24 weeks of treatment both doses of ocrelizumab (2×300 mg and 2×1000 mg) showed a strong effect with a highly statistically significant reduction in signs of disease activity as measured by MRI lesions versus placebo, the primary endpoint. Both ocrelizumab dose regimen groups showed statistically significant differences in total number of Gd+ T1 lesions at weeks 12, 16, 20 and 24 versus placebo with relative reductions in number of lesions of 89% and 96% vs. placebo (2×300 mg and 2×1000 mg ocrelizumab, respectively). Both ocrelizumab doses showed also a statistically significant reduction in the Annualized Relapse Rate (ARR) compared with placebo. Moreover, both ocrelizumab doses were superior to the active comparator, Avonex, for the magnetic resonance imaging (MRI) primary endpoint and ARR.

Although the Phase II ocrelizumab study which consisted of approximately 50 patients per arm demonstrated significant treatment effects in both MRI and ARR at Week 24 versus Avonex, the sample size required to demonstrate a statistically significant difference in disability progression between ocrelizumab and an active comparator is estimated to be much greater, approximately 800 patients per arm. To demonstrate significant treatment benefit of ocrelizumab in disability progression, the Sponsor plans to pool data from two Phase III studies, which will both include approximately 400 patients per ocrelizumab or Rebif treatment arm, have identical inclusion/exclusion criteria, and will be implemented concurrently. The Food and Drug Administration agrees that demonstration of a statistically and clinically significant benefit by pooling of data across the two Phase III studies will support a disability claim for ocrelizumab if the trials are double blinded.

# 2. Study Design

## 2.1 Protocol Synopsis

The protocol synopsis is in Appendix A. For additional details, see the Study Flowchart, also included in Appendix A.

## 2.2 Outcome Measures

### 2.2.1 Primary Efficacy Outcome Measures

The primary efficacy endpoint is annualized protocol-defined relapse rate by two years (96 weeks).

### 2.2.2 Secondary Efficacy Outcome Measures

- The time to onset of sustained disability progression for at least 12 weeks during the 96-week comparative treatment period.
- The time to onset of sustained disability progression for at least 24 weeks during the 96-week comparative treatment period.
- The proportion of relapse-free patients by 96 weeks.
- The change in total T2 lesion volume as detected by brain MRI from baseline to Week 96.
- The total number of new, and/or enlarging T2 hyperintense lesions as detected by brain MRI at Week 24, Week 48, and Week 96.
- The change in Multiple Sclerosis Functional Composite Scale (MSFCS) score from baseline to Week 96.
- The change in brain volume as detected by brain MRI from Week 24 to Week 96

### 2.2.3 Exploratory Efficacy Outcome Measures

- The annualized relapse rate, based on clinical and protocol-defined relapses at the end of the 96-week comparative treatment period.
- The total number of T1 gadolinium-enhanced lesions as detected by brain MRI at Weeks 24, 48, and 96.
- The change in brain volume as detected by brain MRI from baseline to Week 96.
- The change in brain volume as detected by brain MRI from Week 48 to Week 96.
- The change in Multiple Sclerosis Functional Composite Scale (MSFCS) score from baseline to Week 48.
- The cumulative change in Expanded Disability Status Scale (EDSS) scores, measured in area under the curve (AUC) by Week 96.
- The change in EDSS from baseline to Weeks 48 and 96.
- The change in timed 25-foot walk from baseline to Weeks 48 and 96.
- The change in 9-hole peg test from baseline to Weeks 48 and 96.
- The change in paced auditory serial addition test (PASAT) from baseline to Weeks 48 and 96.
- The time to onset of sustained 20% increase in 9-hole peg test for at least 12 weeks.
- The time to onset of sustained 20% increase in timed 25 foot walk for at least 12 weeks.

- Patient Reported Outcomes (PROs): Modified Fatigue Impact Scale (MFIS), EuroQol instrument (EQ-5D), Center for Epidemiology Studies Depression Scale (CES-D) and Medical Outcomes Study 36-Item Short Form Survey Instrument (SF-36) at baseline, Week 48 and Week 96.
- The change in Karnofsky Performance Status Scale (clinician-reported version) from baseline to Weeks 24, 48, 72, and 96.

#### 2.2.4 Pharmacokinetic and Pharmacodynamic Efficacy Outcome Measures

Pharmacokinetics:

- Develop a population PK model to describe the PK of ocrelizumab in MS patients and to estimate inter- and intra-patient variability
- Determine individual post-hoc estimates to derive PK exposure measures
- Explore and quantify the potential influence of covariates contributing to the inter-patient variability in PK parameters
- Explore the relationship between ocrelizumab exposure and efficacy endpoints
- Explore the relationship between ocrelizumab exposure and selected safety parameters

Pharmacodynamics:

- CD19 count as PD marker

#### 2.2.5 Safety Outcome Measures

Safety will be assessed through regular neurological and physical examinations, vital signs, ECG, and the occurrence of adverse events. In addition, the following will be examined:

- Non-MS pathology at all available MRI scans;
- Columbia-suicide severity rating scale (C-SSRS);
- Complete routine hematology, chemistry and urinalyses;
- Circulating B-cell subsets, T cells, natural killer cells and other leukocytes;
- Plasma immunoglobulins;
- HAHA;
- Antibody titers for mumps, rubella, varicella, and Streptococcus pneumoniae;
- Serial pregnancy tests [serum/urine β subunit human chorionic gonadotropin (β hCG)] performed in women of child bearing potential.
- JC virus (JCV) plasma/urine (sampling).

### 2.3 Determination of sample size

The sample size for this study has been estimated based on data from previous RRMS trials, with the use of two-sided tests with an experiment-wise alpha of 0.05. The annualized rate of relapse among patients receiving ocrelizumab at 96 weeks is predicted to be 0.165 (standard deviation of approximately 0.60), as compared with 0.33 (standard deviation of approximately 0.80) among patients receiving the control treatment, Rebif (this represents a relative reduction of 50% on ocrelizumab compared to the active comparator). For the annualized relapse rate, a t-test has been used to determine the sample size between ocrelizumab and the control arm. The sample size of 400 patients per arm provides 84 percent power, maintaining the type I error rate of 0.05, and assuming a drop out rate of 20 percent approximately (assuming relative reduction among patients drop out is 25%).

For sustained disease progression, a two-group test of equal exponential survival with exponential dropout is used to determine the sample size. Assuming the 2 year sustained disability progression rate is 18% for the Rebif arm and 12.6% for the ocrelizumab arm (this represents a relative reduction of 30% on ocrelizumab compared to the active comparator), and assuming a drop out rate of 20 percent over 2 years approximately, the sample size of 400 per arm will provide 80 percent power, maintaining the two-sided type I error rate of 0.05 based on the pooled analysis of two RMS trials (800 patients treated with ocrelizumab 600 mg and 800 patients treated with Rebif).

## 2.4 Analysis Timing

Database lock for analysis will happen after last patient enrolled finishes the Week 96 visit.

# 3. STUDY CONDUCT

## 3.1 Randomization Issues

Patients will be randomized in 2 groups in a 1:1 ratio. An independent Interactive Voice and Web Response System (IxRS) provider will conduct randomization (using blocked randomization) and hold the treatment assignment code. Patients will be stratified by region (U.S. vs. Rest of World) and baseline EDSS ($< 4$ and $\geq 4$).

The Patient Randomization List will be generated by IxRS using a pre-defined randomization specification. The Randomization List will not be available at the study center, to the Roche monitors, project Statisticians or to the Sponsor's project team. Unblinding of ocrelizumab dose should not occur except in the case of emergency situations. Any request from the investigator for information about the treatment administered to study patients for another purpose must be discussed with Roche. Unblinding will be performed by means of an IxRS. As per regulatory reporting requirement, Roche will unblind the identity of the study medication for all unexpected serious adverse events that are considered by the investigator to be related to study drug as per safety reference document(s), e.g., IB, CDS, and SPC. Details of patients who are unblinded during the study will be included in the Clinical Study Report.

## 3.2 Independent Review Facility

MRI scans will be read by a centralized reading center. The centralized reading center is blinded to the treatment assignment and the reading is performed in the absence of clinical information. Further details on scanning acquisition sequences, methods, handling and transmission of the scans, certification of site MRI radiologist/technicians, and the procedures for the blinded analysis of the scans at the central reading center are described in a separate MRI technical manual.

All EDSS results will be checked using algorithm provided by NEUROSTATUS. Queries from these checks will be sent out to sites.

## 3.3 Data Monitoring

A Data Monitoring Committee (DMC) will review cumulative data from the trial at approximately four month intervals until the final analysis is completed. The DMC will review both efficacy and safety data; however, studies will not be stopped for efficacy reasons.

# 4. STATISTICAL METHODS

## 4.1 Analysis Populations

One patient population will be defined for the purpose of the safety analysis and two for the efficacy analysis. All efficacy analyses will be performed using the intent-to-treat (ITT) population. The per-protocol population will be used for all primary and secondary efficacy analyses in order to evaluate the influence of major protocol violators and as a sensitivity check to the ITT analysis.

### 4.1.1 Intent-to-Treat Population

All randomized patients who have received any study drug will be included in the ITT population. Patients who prematurely withdraw from the study for any reason and for whom an assessment is not performed for whatever reason will still be included in the ITT analysis. Patients who receive an incorrect therapy from that which is intended will be summarized according to their randomized treatment.

### 4.1.2 Per Protocol Population

The per-protocol population will include all patients in the ITT population adhering to the protocol. Patients may be excluded if they significantly violate the inclusion/exclusion criteria or deviate from the study plan. Specific reasons for warranting exclusion will be agreed and documented in the Data Analysis Plan based on the final version of the protocol prior to unblinding of the treatment groups. Only those patients with violations that are deemed to potentially affect the efficacy of study treatment will be excluded from the per protocol population. Patients who receive an incorrect therapy from that intended will be excluded from the per-protocol population.

### 4.1.3 Pharmacokinetic Evaluable Population

The PK population will include all patients who received any study drug and provided at least one measurable concentration value.

### 4.1.4 Safety Population

The safety population will include all patients who received any study drug and provided at least one assessment of safety. Randomized patients that receive incorrect therapy from that intended will be summarized in the group according to the therapy actually received. Patients who are not randomized, but who receive study drug, will be included in the safety population and summarized according to the therapy actually received. Patients who receive more than one study therapy will be summarized in the ocrelizumab group.

## 4.2 Analysis of study conduct

The following analysis will be conducted:

- Summary of protocol deviation
- Summary of ITT/per-protocol defined/safety population
- Summary of subject disposition
- Summary/plot of time to discontinuation of treatment/study

## 4.3 Analysis of treatment group comparability

### 4.3.1 Demography

- Age (years) – the summary statistics calculated will be the mean, median, SD, minimum and maximum. Percentage and number in each category (18–39, 40–54, ≥55) will be displayed.
- Sex – the number and percentage of male and female patients.
- Race – The number and percentage of White, Black or African American, Asian (Indian Subcontinent, Other than Indian subcontinent), American Indian or Alaskan native, Native Hawaiian or other Pacific Islander, and Other.
- Ethnicity – The number and percentage of Hispanic or Latino and non–Hispanic or Latino.
- Weight (kg) – summary statistics as described for age.
- Height (cm) – summary statistics as described for age.

### 4.3.2 Baseline Disease Characteristics

- Baseline EDSS – the number and percentage of patients in each EDSS category (<2.5, 2.5–4, ≥4); EDSS mean, median, SD, minimum, and maximum.
- Number of relapses in the past year – the number and percentage of patients in each category (0, 1, 2, 3, ≥4); a relapse is considered to happen in the past year if the date of first treatment – date of relapse is equal or less than 365.
- Number of relapses in the past two years – the number and percentage of patients in each category (0, 1, 2, 3, ≥4); a relapse is considered to happen in the past two years if the date of first treatment – the date of relapse is equal or less than 365×2.
- Time since last onset of MS relapse prior to randomization (year) – the number and percentage of patients in each category (more than 6 months prior to randomization; within the last 6 months from randomization); the summary statistics calculated will be the mean, median, SD, minimum and maximum (calculated in years, i.e., divide by 365.25). Time is calculated as the date of first treatment – the date of last relapse.
- Baseline FS score (Pyramidal, Cerebellar, Brainstem, Sensory, Bowel/bladder, Visual, Cerebral) – the number and percentage of patients in each category (0–6, depends on the domain of FS score).
- Baseline MFIS total score – the summary statistics calculated will be the mean, median, SD, minimum, and maximum.
- Baseline SF-36 total score – the summary statistics calculated will be the mean, median, SD, minimum, and maximum.
- Baseline CES-D score – the summary statistics calculated will be the mean, median, SD, minimum, and maximum.
- Baseline EQ-5D score – the summary statistics calculated will be the mean, median, SD, minimum, and maximum.

- Duration since MS symptom onset (years) – the summary statistics calculated will be th mean, median, SD, minimum, and maximum (calculated in years, i.e., divide by 365.25).

  Duration since MS symptom/diagnosis onset will be calculated up to first screening date. The missing month of symptom/diagnosis onset date will be imputed by January. The missing day of symptom/diagnosis onset date will be imputed by 1st
- Duration since MS diagnosis (years) -- the summary statistics calculated will be the mean, median, SD, minimum and maximum (calculated in years, i.e., divide by 365.25).
- Prior treatment with any immuno-modulatory treatment (Yes [treatment experienced patient]; No [naïve patients])
- Number of Gadolinium Enhancing T1 lesions at baseline – the number and percentage of patients in each category (0, 1, 2, 3, ≥4); the summary statistics calculated will be the mean, median, SD, minimum and maximum.
- Volume of T2 lesions at baseline –the summary statistics calculated will be the mean, median, SD, minimum and maximum.
- Number of Previous T2 lesions in the past 12 months – the number and percentage of patients in each category (0-5, 6-9, >9)

## 4.4 Efficacy Analysis

### 4.4.1 Primary Efficacy Endpoint

#### *4.4.1.1 Significance level*

The null hypothesis will be tested at alpha=0.05 level (two-sided test).

$H_0$ (null hypothesis): There is no statistically significant difference in annualized protocol-defined relapse rate at two years between the ocrelizumab group and Rebif group.

$H_1$ (alternative hypothesis): There is a statistically significant difference in annualized protocol-defined relapse rate at two years between the ocrelizumab group and Rebif group.

Annualized protocol-defined relapse rate at two years between the ocrelizumab dose groups and Rebif group will be compared using the Poisson model, adjusting geographical region and baseline EDSS. If the test result for comparing 600 mg ocrelizumab and Rebif groups is statistically significant at $\alpha < 0.05$ level (two-sided test), we will conclude that the 600-mg ocrelizumab group demonstrated a superior effect of reducing Annualized protocol defined relapse rate, when compared to Rebif group.

#### *4.4.1.2 Definition*

A protocol-defined relapse is defined as the occurrence of new or worsening neurological symptoms attributable to MS and immediately preceded by a relatively stable or improving neurological state of least 30 days. Symptoms must persist for >24 hours and should not be attributable to confounding clinical factors (e.g., fever, infection, injury, adverse reactions to concomitant medications). The new or worsening neurological symptoms must be accompanied by objective neurological worsening consistent with an increase of at least half a step on the EDSS, or 2 points on one of the appropriate Functional System Scores (FSS), or 1 point on two or more of the appropriate FSS. The change must affect the selected FSS

(i.e., pyramidal, gait, cerebellar, brainstem, sensory, or visual). Sensory changes, episodic spasms, fatigue, mood change, or bladder or bowel urgency or incontinence will not suffice to establish a relapse.

Information related to a protocol-defined relapse will be captured on a Clinical relapse event CRF page If the following criteria are satisfied, the clinical relapse will qualify as a protocol defined relapse:

1. Check box of "*Did symptom persist for >24 hours and were not being attributable…*" on the Clinical MS relapse event is checked.
2. EDSS from a visit occurring at (or near after) the onset date of relapse needs to be at least half a step from the previous EDSS; OR for FSS domains involved in the relapse event (indicated in the Clinical MS relapse page), they need to be at least 2 points increase on one appropriate FSS domain or at least 1 point on two or more appropriate FSS domains.
3. There is no protocol defined relapse within 30 days before the start date of the clinical relapse.

#### 4.4.1.3 Analysis Methods

The annualized protocol-defined relapse rate by Week 96 will be calculated using Poisson regression, offsetting for exposure time in years, accounting for over-dispersion and adjusting for geographical region and baseline EDSS (<4, ≥4). The number of protocol-defined relapse will be counted. The exposure time will be calculated as (early treatment discontinued date/date cut date for Week 96 – date of study day 1 +1)/365.

All available data during the 96-week treatment period will be used for the analysis.
For patients discontinued the treatment early, only their data before the early treatment discontinuation will be used in this analysis.

#### 4.4.1.4 Sensitivity and Robustness Checks

Poisson models adjusting for number of relapses occurring within the 2 years prior to study entry, baseline presence of gadolinium lesions (present or absent), prior immunomodulatory treatment, age (<40, ≥40), baseline EDSS, and geographical region will also be fitted.

Diagnostic tests will be conducted to ensure the assumptions concerning Poisson regression are satisfied. If the scale estimate (as a ratio of the deviance or the Pearson Chi-Square to its associated degrees of freedom) from the Poisson model is much larger than 1, then negative binomial regression or ANCOVA using patient relapse rate (calculated as the number of relapses for each patient divided by the number of years followed in the study for that patient) will be used to replace Poisson model with same multivariate.

Sensitivity analyses will be done including all available data at or before Week 96 (for patients discontinued the treatment early, safety follow up data before Week 96 will be included).

### 4.4.2 Secondary Efficacy Endpoints

Secondary efficacy endpoints will be tested in hierarchal order (listed as following), all at alpha=0.05 level. The first secondary efficacy endpoint will be tested if and only if the primary endpoint has reached the significant level at 0.05 (e.g., P-value ≤0.05). The rest of the secondary endpoints will be tested if and only if the secondary endpoint list ahead of it has reached the significant level at 0.05.

#### *4.4.2.1 The time to onset of sustained disability progression for at least 12 weeks during the 96-week comparative treatment period*

Disability progression as measured by EDSS will be assessed in all patients by the independent examining investigator at screening, Week 0, and every 12 weeks throughout the study until the Observation Period, at which time disability progression is assessed every 24 weeks. Additional EDSS assessments for individual patients may be requested between visits (i.e., during an MS relapse).

The EDSS is based on a standard neurological examination; the seven categories of the EDSS representing functional systems (pyramidal, cerebellar, brainstem, sensory, bowel and bladder, visual, and cerebral [or mental], plus "other") are rated and scored (collectively, functional system scores [FSS]). Each score of the FSS is an ordinal clinical rating scale ranging from 0 to 5 or 6. **These ratings are then used in conjunction with observations and information concerning ambulation** and use of assistive devices (which will also be scored) to determine the EDSS score. The EDSS is a disability scale that ranges in 0.5-point steps from 0 (normal) to 10.0 (death).

Due to the variability of EDSS (EDSS is captured electronically and blinded EDSS raters will not be able to review previous EDSS records), a single measurement at Week 0 might not provide a sufficiently reliable baseline EDSS. Therefore, using an average score from two separate measurements may improve the reliability of baseline EDSS and the reliability of sustained disability progression seen on study. Inclusion criteria require neurological stability for 30 days prior to both screening and Week 0. Generally, it takes 2 weeks for screening. There should not be any change in EDSS excepting the measurement variability during the screening period, assuming patients are neurologically stable during this period.

If the EDSS rater for the screening EDSS is different from the EDSS rater for the Week 0 and remaining measurements during the trial, the Week 0 EDSS will be used for baseline EDSS.

A rounding up method is proposed for the calculation of average EDSS (round 0.25 to 0.5; round 0.75 to 1) to decrease the false positive rate of sustained disability progression.

Progression is defined a ≥ 1 point increase in the EDSS from baseline EDSS 1.0–5.5 and a 0.5 increase from baseline EDSS 6.0 or higher. Confirmation requires the sustained change in EDSS for at least 12 weeks from the initial progression. Initial progression can happen at any visit. Confirmation of disease progression must occur at the regularly scheduled visit that is at least 12 weeks (84 days) after initial progression. If a subject has a missing EDSS at the scheduled visit taking place at least 84 days after an initial progression, then the confirmation of the disease progression must be based on the assessment at the next scheduled visit. There may be EDSS assessments at unscheduled or scheduled visits that are <84 days after

the initial progression, which are between the initial progression visit and the confirmation visit. Disease progression cannot be confirmed unless the EDSS values meet the minimum change required for progression. Subjects who complete the 96-week treatment period without confirmed progression of disease will be censored at the date of their last EDSS assessment during the treatment period. Subjects who subsequently discontinue from the treatment, with/without an initial assessment of disease progression, will not be considered to have confirmed disease progression and will be censored at the date of their last EDSS assessment during the treatment period. Subjects who receive exclusionary MS therapies prior to experiencing the confirmed disease progression in the treatment period will be censored at the time of the last EDSS assessment prior to receiving the exclusionary MS medication. The exclusive MS therapies include natalizumab, interferon beta, interferon beta-1a, interferon beta-1b, glatiramer acetate, and mitoxantrone hydrochloride, cyclophosphamide, mycophenolate mofetil, azathioprine, alemtuzumab, cyclosporine, cladribine, plasmapheresis, immune globulin (IVIG), and fingolimod.

Data from the two studies with respect to ocrelizumab group vs. Rebif group will be pooled for analysis of this endpoint at alpha=0.05 level. Only data collected during the treatment period will be used for this analysis.

Time to sustained disability progression for ocrelizumab group and Rebif group (across the studies) will be compared using a two-sided log-rank test stratifying by geographic region, baseline EDSS, and study. The proportion of patients with sustained disability progression will be estimated using Kaplan-Meier methodology. The overall hazard ratio will be estimated using a stratified Cox regression model with the same stratification factors used in the stratified log-rank test above.

To assess the validity of pooling data across the two RMS studies, demographic and baseline characteristics will be compared by trials. The treatment effect (hazard ratio and CI) for sustained disability progression within each trial will be displayed in a forest plot and compared between the two trials. In interpreting the trial comparisons with a view toward assessing the validity of the pooled dataset, the primary interest is in confirming that the treatment effect is qualitatively similar across the two studies. If the results from the two studies are not qualitatively similar, data will not be pooled.

The following sensitivity analyses will be done:

- Using last available EDSS before first dose as EDSS baseline.
- Using average score of screening and Week 0 EDSS with rounding down method (round 0.25 to 0.0; round 0.75 to 0.5) as EDSS baseline.
- For the confirmation of disease progression, sensitivity analyses will be done to exclude any EDSS assessments at scheduled visit but within 30 days after a protocol defined relapse.
- Use all available data during the treatment period without censoring patients with exclusionary MS medication.
- Use all available data at or before Week 96 (for patients who discontinued the treatment early, safety follow up data before week 96 will be included).

- Sensitivity analysis uses the same tests above but adjusting for number of relapses occurring within the 2 year prior to study entry, baseline presence of gadolinium lesions (present or absent), prior immunomodulatory treatment, age (<40, ≥40), baseline EDSS, and geographical region.

#### 4.4.2.2 The time to onset of sustained disability progression for at least 24 weeks during the 96-week comparative treatment period

This is the same as the 12-week sustained disability progression except that the confirmation of disease progression must occur at the regularly scheduled visit that is at least 24 weeks after initial progression. Because the scheduled visits can happen outside the visit window specified in the protocol (protocol specified visit window is ± 4 days of the expected date), a cutoff of 161 days will be used for 24 weeks CDP analysis to capture more events. A sensitivity analysis using 168 days as cutoff will also be done.

#### 4.4.2.3 The proportion of relapse-free patients by 96 weeks

All available data during the 96-week treatment period will be used for the analysis. For patients who complete the 96-week treatment period, they will be considered as not relapse-free if they had at least one protocol defined relapse during the 96-week treatment period, otherwise they will be considered as relapse-free. Patients who discontinued the treatment early with at least one relapse before the early discontinuation will be considered as not relapse-free. Even if they did not have a relapse before the early discontinuation, they will be considered as having relapse if the reason for early discontinuation is lack of efficacy or death; otherwise, they will be considered as missing observations.

The proportions will be analyzed using Cochran-Mantel-Haenszel $\chi^2$ test stratified by geographical region and baseline EDSS (<4, ≥4). Logistic model adjusting for adjusting for number of relapses occurring within the 2 years prior to study entry, baseline presence of gadolinium lesions (present or absent), prior immunomodulatory treatment, age (<40, ≥40), baseline EDSS and geographical region will also be fitted. Log-rank test will also be used to get k-m estimates of proportion of relapse-free patients at Week 96. In this analysis, time to first relapse will used in the test. For those patients who didn’t have relapse by Week 96 or early discontinuation, their time to first relapse will be censored at Week 96 or early discontinuation visit.

Sensitivity analyses will be done including all available data at or before Week 96 (for patients discontinued the treatment early, safety follow up data before Week 96 will be included). In this analysis, for patients who have discontinued the study before Week 96, if they didn’t have any relapse before the discontinuation of the study, they will be considered as having relapses if the reason for early discontinuation of study is lack of efficacy or death; otherwise they will be considered as missing observations.

#### 4.4.2.4 The change in total T2 lesion volume as detected by brain MRI from baseline to Week 96

The change in total volume of T2 lesions on MRI scans of the brain from baseline to Week 96 will be computed. Missing values will be imputed using last-observation-carried-forward (LOCF) methodology. Baseline values will not be carried forward.

Ranked ANCOVA[1] adjusting baseline T2 lesion volume, baseline Gd lesion (present or not), geographical region and baseline EDSS will also be will be applied to compare the differences between ocrelizumab and Rebif groups. Change from baseline and baseline values will be ranked first. Ranks of change from baseline will replace change from baseline in the ANCOVA model. Residual from the regression model for ranks of change from baseline and ranks of baseline will then be used in the ANCOVA model to replace baseline values.

Sensitivity analysis will be done using observed data with no imputation.

#### *4.4.2.5 The total number of new, and/or enlarging T2 hyperintense lesions as detected by brain MRI at Week 24, Week 48, and Week 96*

The total number of new and/or enlarging T2 lesions will be calculated as the sum of the individual number of new and/or enlarging lesions at Weeks 24, 48, and 96. A missing value for T2 lesions at a particular week will be imputed by the average number of new and/or enlarging lesions on available scans obtained during the 96 weeks of treatment.

Negative binomial model adjusting baseline Gd lesion (present or not), geographical region, and baseline EDSS will be applied to compare the difference between ocrelizumab and Rebif groups.

Sensitivity analysis will be done using observed data with no imputation and with the van Elteren test stratified by geographical region and baseline EDSS.

#### *4.4.2.6 The change in Multiple Sclerosis Functional Composite Scale score from baseline to Week 96*

There are three primary measures in a Multiple Sclerosis Functional Composite (MSFC):1) Timed 25-Foot walk; 2) 9-Hole Peg Test (9-HPT); and 3) Paced Auditory Serial Addition Test (PASAT-3" version). The MSFC is based on the concept that scores for these three dimensions—arm, leg, and cognitive function are combined to create a single score (the MSFC) that can be used to detect change over time in a group of multiple sclerosis patients. Since the three primary measures are different in what they actually measure (time for the 9HPT and 25-Foot Timed Walk, but number of correct answers for the PASAT-3), a common metric for these variables has be used to create a composite score for the three different measures. Z-score was selected for this purpose. The results from each of these three tests are transformed into Z-scores and averaged to yield a composite score for each patient at each time point.

MSFC Score = {Zarm, average + Zleg, average + Zcognitive} / 3.0

Where Zxxx =Z-score

[1] Categorical Data Analysis using the SAS system. Stokes, Davis, and Koch

The following are MSFC components:

1. Trial 1, Timed 25-Foot Walk

2. Trial 2, Timed 25-Foot Walk

3. Trial 1, Dominant Hand, 9-HPT

4. Trial 2, Dominant Hand, 9-HPT

5. Trial 1, Non-Dominant Hand, 9-HPT

6. Trial 2, Non-Dominant Hand, 9-HPT

7. PASAT-3

There are three derived results for three tests: (1) the average (reciprocal) scores from the four trials on the 9-HPT (the two trials for each hand are averaged, converted to the reciprocals of the mean times for each hand and then the two reciprocals are averaged); (2) the average scores of two 25-Foot Timed Walk trials; (3) the number correct from the PASAT-3. The Z-score will be created for these three derived results to yield the composite score by the following formula:

| Formula For Creating The MSFC Score to Compare Groups Within a Study<br>(The Preferred Method) |
|---|
| MSFC Score ={ (Average (1/9-HPT) - Baseline Mean (1/9-HPT)<br>/ Baseline Std Dev (1/9-HPT)<br>+ { - (Average 25-Foot Walk - Baseline Mean 25-Foot Walk)<br>/Baseline Std-Dev 25-Foot Walk}<br>+ (PASAT-3 - Baseline Mean PASAT-3)<br>/Baseline Std Dev PASAT-3} / 3.0 |
| *Note:* "Average (1/9-HPT)" is the average of the inverse (reciprocal) for the mean time of the two trials on the right hand and reciprocal of the mean time of the two left-hand trials from the test patient, Baseline Mean (1/9-HPT) and Std Dev (1/9-HPT) are the baseline values from each patient in all study groups combined at the baseline assessment; "Average 25-Foot Walk" is the mean time from the two trials of the 25-foot timed walk; and we take the negative value of the Z-score to make the direction of change the same as the other components. Similarly, the Baseline Mean and Std Dev 25-Foot Walk are of all Baseline Groups combined; and "PASAT-3" is the score from the test patient, and the Baseline Mean PASAT-3 and Std Dev PASAT-3 of the combined baseline assessments. These are discussed below. |

Please note the changing the signs of the Z-scores for the 25-Foot Walk and using inverse value of 9-HPT to ensure that a negative value implies worsening and a positive value implies improvement. More details can be found at MSFC Manual.

Missing data imputation for results of the same test:

25-Foot Timed Walk trials: If the test results are not available due to "physical limitation", impute a large number (777 according to manual). If the test results are not available NOT due to "physical limitation," use the result from the other trial to impute the missing value.

9-HPT: If the test results are not available due to "physical limitation," impute a large number (777 according to manual). If the test results are not available but NOT due to "physical limitation," impute the missing test score using the result from the other trial of the same hand. If the result from the other trial of the same hand is also missing, use the average score from the other hand (or available score from one trial if only result from one trial is available).

Missing data imputation for the composite score:

If there is a protocol-defined relapse that has happened within 30 days before the scheduled Week 96 visit, the MSFCS results will not be used. The last observed value (at stable condition, e.g. at least 30 days after the start date of a protocol defined relapse) will be used to input missing or unusable MSFCS value.

Ranked ANCOVA[2] adjusting baseline MSFCS, geographical region and baseline EDSS will also be will be applied to compare the differences between ocrelizumab and Rebif groups.

Sensitivity analysis will be done using observed data with no imputations.

##### *4.4.2.7 The change in brain volume as detected by brain MRI from Week 24 to Week 96*

The change in brain volume on MRI scans of the brain from Week 24 to Week 96 will be computed. Missing values will be imputed using LOCF methodology. Baseline values will not be carried forward.

Ranked ANCOVA[2] adjusting baseline brain volume, baseline Gd lesion (present or not), geographical region and baseline EDSS will also be modeled to compare the differences between ocrelizumab and Rebif groups. Sensitivity analysis will be done using observed data with no imputation.

#### 4.4.3 Exploratory Efficacy Endpoints

##### *4.4.3.1 The annualized relapse rate, based on clinical and protocol-defined relapse at the end of the 96-week comparative treatment period*

All clinical relapses reported on the MS relapse page will be used for this analysis (protocol defined relapse is a subset of clinical relapse).

The annualized clinical relapse rate by Week 96 will be calculated using Poisson regression, offsetting for exposure time in years, accounting for over-dispersion and adjusting for geographical region and baseline EDSS (<4, ≥4). The number of protocol defined relapse will be counted. The exposure time will be calculated as (early treatment discontinued date/data cut date for Week 96 – date of study day 1 +1)/365.

[2] Categorical Data Analysis using the SAS system. Stokes, Davis, and Koch.

All available data during the 96-week treatment period will be used for the analysis. For patients who discontinued the treatment early, only their data before the early treatment discontinuation will be used in the analysis.

#### *4.4.3.2 The total number of T1 gadolinium-enhanced lesions as detected by brain MRI at Week 24, 48, and 96*

The total number of T1 gadolinium-enhanced lesions will be calculated as the sum of the individual number of T1 gadolinium-enhanced lesions at Weeks 24, 48, and 96. A missing value for T1 gadolinium-enhanced lesions at a particular week will be imputed by the average number of new and/or enlarging lesions on available scans obtained during the 96 weeks of treatment.

Negative binomial model adjusting baseline Gd lesion (present or not), geographical region and baseline EDSS (<4, ≥4) will be applied to compare the differences between ocrelizumab and Rebif groups.

#### *4.4.3.3 The change in brain volume as detected by brain MRI from baseline to Week 96*

The change in brain volume on MRI scans of the brain from baseline to Week 96 will be calculated. Missing values will be imputed using last-observation-carried-forward (LOCF) methodology. Baseline values will not be carried forward.

Ranked ANCOVA adjusting baseline brain volume, baseline Gd lesion (present or not), geographical region and baseline EDSS (<4, ≥4) will be applied to compare the differences between ocrelizumab and Rebif groups.

#### *4.4.3.4 The change in brain volume as detected by brain MRI from Week 48 to Week 96*

The change in brain volume on MRI scans of the brain from Week 48 to Week 96 will be calculated. Missing values will be imputed using last-observation-carried-forward (LOCF) methodology. Baseline values will not be carried forward.

Ranked ANCOVA[3] adjusting baseline brain volume, baseline Gd lesion (present or not), geographical region, and baseline EDSS (<4, ≥4) will be applied to compare the differences between ocrelizumab and Rebif groups.

#### *4.4.3.5 The change in Multiple Sclerosis Functional Composite Scale (MSFCS) score from baseline to Week 48*

This is the same as the change in Multiple Sclerosis Functional Composite Scale (MSFCS) score from baseline to Week 96.

---

[3] Categorical Data Analysis using the SAS system. Stokes, Davis, and Koch.

### 4.4.3.6 The cumulative change in EDSS scores, measured in area under the curve (AUC) by Week 96

Only EDSS from scheduled visits will be used for the calculation of AUC. The planned scheduled visits with EDSS measure are baseline, Week 12, Week 24, Week 36, Week 48, Week 60, Week 72, Week 84, and Week 96. If a protocol-defined relapse occurred within 30 days before the scheduled visit, the EDSS results from that scheduled visit will not be used. If the EDSS value for a scheduled visit is missing or not usable, the value will be imputed using last observed available EDSS from the scheduled visit before the time point for the missing value. If the date of a scheduled visit is missing, it will be imputed using last available date of scheduled visit (e.g., date of Week 24 visit=date of week 12 visit + 84). After the imputation, the AUC will be calculated by the following formula:

$$AUC_{baseline-week96} = \sum\left(\frac{EDSS_{baseline} + EDSS_{week12}}{2} \bullet (date_{week12} - date_{baseline})\right)$$

$$+\left(\frac{EDSS_{week12} + EDSS_{week24}}{2} \bullet (date_{week24} - date_{week12})\right) + \cdots$$

$$+\left(\frac{EDSS_{week84} + EDSS_{week96}}{2} \bullet (date_{week96} - date_{week84})\right)$$

Ranked ANCOVA[4] adjusting baseline EDSS, geographical region and baseline EDSS (<4, ≥4) will be applied to compare the differences in AUC between ocrelizumab and Rebif groups.

### 4.4.3.7 The change in EDSS from baseline to Weeks 48 and 96

If a protocol-defined relapse occurred within 30 days before the scheduled Week 48 or Week 96 visit, then the EDSS results will not be used. The last observed value which is at least 30 days after a protocol defined relapse will be used to input missing or unusable EDSS value.

Ranked ANCOVA[4] adjusting baseline EDSS, geographical region, and baseline EDSS (<4 ≥4) will be applied to compare the differences in change from baseline to Week 48 (96) between ocrelizumab and Rebif groups.

### 4.4.3.8 The change in timed 25 foot walk from baseline to Weeks 48 and 96

Average time (for 25-foot walk) from two trials will be used in this analysis. Imputation method for missing values in any of the two trials will be same as in 4.4.2.6.

If a protocol-defined relapse occurred within 30 days before the scheduled Week 48 or Week 96 visit, then the timed 25-foot walk results will not be used. The last observed value which is at least 30 days after a protocol defined relapse will be used to input missing or unusable EDSS value.

[4] Categorical Data Analysis using the SAS system. Stokes, Davis, and Koch.

Ranked ANCOVA[5] adjusting baseline timed 25 foot walk, geographical region and baseline EDSS (<4, ≥4) will be applied to compare the differences in change from baseline to Week 48 (96) between ocrelizumab and Rebif groups.

#### *4.4.3.9 The change in 9-hole peg test from baseline to Weeks 48 and 96*

Average score of 9-hole peg test from four trials will be used in this analysis. Imputation method for missing values in any of the four trials will be same as in 4.4.2.6.

If a protocol-defined relapse occurred within 30 days before the scheduled Week 48 or Week 96 visit, then the 9-hole peg test results will not be used. The last observed value which is at least 30 days after a protocol defined relapse will be used to input missing or unusable EDSS value.

Van Elteren test stratified by geographical region and baseline EDSS (<4, ≥4) will be applied to compare the differences in change from baseline to Week 48 (96) between ocrelizumab and Rebif groups.

Ranked ANCOVA[5] adjusting baseline 9-hole peg test, geographical region and baseline EDSS (<4, ≥4) will be applied to compare the differences in change from baseline to Week 48 (96) between ocrelizumab and Rebif groups.

#### *4.4.3.10 The change in paced auditory serial addition test (PASAT) from baseline to Weeks 48 and 96*

If a protocol-defined relapse occurred within 30 days before the scheduled Week 48 or Week 96 visit, then the PASAT test results will not be used. Last observed value which is at least 30 days after a protocol defined relapse will be used to input missing or unusable EDSS value.

Ranked ANCOVA[5] adjusting baseline PASAT, geographical region and baseline EDSS (<4, ≥4) will be applied to compare the differences in change from baseline to Week 48 (96) between ocrelizumab and Rebif groups.

#### *4.4.3.11 The time to onset of sustained 20% increase in 9-hole peg test for at least 12 weeks*

Average score of 9-hole peg test from four trials will be used in this analysis. Imputation method for missing values in any of the four trials will be same as in 4.4.2.6.

Progression is defined as a ≥ 20% worsening in the 9-hole peg test from baseline ([Average score of 9-hole peg test_wkXX- Average score of 9-hole peg test_baseline] / Average score of 9-hole peg test at baseline ≥ 20%). Confirmation requires the sustained change in 9-hole peg test for at least 12 weeks from the initial progression. Initial disease progression can happen at any visit. Confirmation of disease progression must occur at the regularly scheduled visit that is at least 12 weeks (84 days) after initial progression. If a subject has a missing 9-hole peg test at the scheduled visit taking place at least 84 days after an initial progression, then the confirmation of the disease progression must be based on the

---

[5] Categorical Data Analysis using the SAS system. Stokes, Davis, and Koch.

assessment at the following scheduled visit. There may be 9-hole peg test assessments (at unscheduled or scheduled visits that are <84 days after the initial progression) between the initial progression visit and the confirmation visit. These 9-hole peg test values should be at least as high as the minimum change required for progression; otherwise, the initial progression cannot be confirmed at the confirmatory visit. Subjects who complete the 96-week treatment period without confirmed progression of disease will be censored at the date of their last 9-hole peg test assessment during the treatment period. Subjects with/without an initial assessment of disease progression who subsequently discontinue from the treatment will not be considered to have confirmed disease progression, and will be censored at the date of their last 9-hole peg test assessment during the treatment period. Subjects who receive exclusionary MS therapies prior to experiencing the confirmed disease progression in the treatment period will be censored at the time of the last 9-hole peg test assessment prior to receiving the exclusionary MS medication. The exclusive MS therapies include natalizumab, interferon beta, interferon beta-1a, interferon beta-1b, glatiramer acetate, and mitoxantrone hydrochloride, cyclophosphamide, mycophenolate mofetil, azathioprine, alemtuzumab, cyclosporine, cladribine, plasmapheresis, immune globulin (IVIG), and fingolimod.

Time to sustained disability progression for ocrelizumab group and Rebif group will be compared using a two-sided log-rank test stratifying by geographic region, baseline EDSS (<4, ≥4). The proportion of patients with sustained disability progression will be estimated using Kaplan-Meier methodology. The overall hazard ratio will be estimated using a stratified Cox regression model with the same stratification factors used in the stratified log-rank test above.

#### *4.4.3.12 The time to onset of sustained 20% increase in timed 25 foot walk for at least 12 weeks*

Average score of timed 25-foot walk from two trials will be used in this analysis. Imputation method for missing values in any one of the trials will be same as in 4.4.2.6.

Progression is defined a ≥ 20% worsening in the timed 25 foot walk from baseline ([Average score of timed 25-foot walk _wkXX- Average score of timed 25 foot walk _baseline] / Average score of timed 25-foot walk _baseline ≥ 0.2 ). Confirmation requires the sustained change in timed 25-foot walk for at least 12 weeks from the initial progression. Initiation of disease progression can happen at any visit. Confirmation of disease progression must occur at the regularly scheduled visit that is at least 12 weeks (84 days) after initial progression. If a subject has a missing timed 25-foot walk at the scheduled visit taking place at least 84 days after an initial progression, then the confirmation of the disease progression must be based on the assessment at the following scheduled visit. There may be timed 25-foot walk assessments (at unscheduled or scheduled visits that are <84 days after the initial progression) between the initial progression visit and the confirmation visit. These timed 25-foot walk values should be at least as high as the minimum change required for progression; otherwise, the initial progression cannot be confirmed at the confirmatory visit. Subjects who complete the 96-week treatment period without confirmed progression of disease will be censored at the date of their last timed 25-foot walk assessment during the treatment period. Subjects with/without an initial assessment of disease progression who subsequently discontinues from the treatment will not be considered to have confirmed disease

progression, and will be censored at the date of their last timed 25-foot walk assessment during the treatment period. Subjects who receive exclusionary MS therapies prior to experiencing the confirmed disease progression in the treatment period will be censored at the time of the last timed 25 foot walk assessment prior to receiving the exclusionary MS medication. The exclusive MS therapies include natalizumab, interferon beta, interferon beta-1a, interferon beta-1b, glatiramer acetate, and mitoxantrone hydrochloride, cyclophosphamide, mycophenolate mofetil, azathioprine, alemtuzumab, cyclosporine, cladribine, plasmapheresis, immune globulin (IVIG), and fingolimod.

Time to sustained disability progression for ocrelizumab group and Rebif group will be compared using a two-sided log-rank test stratifying by geographic region, baseline EDSS a. The proportion of patients with sustained disability progression will be estimated using Kaplan-Meier methodology. The overall hazard ratio will be estimated using a stratified Cox regression model with the same stratification factors used in the stratified log-rank test above.

#### 4.4.3.13 Change in the MFIS from Baseline to Weeks 48 and 96

The items of the MFIS can be aggregated into three subscales (Physical, Cognitive, Psychosocial), as well as into a total MFIS score. All items are scaled so that higher scores indicate a greater impact of fatigue on a patient's activities.

*Physical Subscale:*

This scale can range from 0-36. It is computed by adding raw scores on the following items: 4+6+7+10+13+14+17+20+21.

*Cognitive Subscale:*

This scale can range from 0–40. It is computed by adding raw scores on the following items: 1+2+3+5+11+12+15+16+18+19.

*Psychosocial Subscale:*

This scale can range from 0–8. It is computed by adding raw scores on the following items: 8+9.

*Total MFIS Score:*

The Total MFIS score can range from 0=84. It is computed by adding scores on the Physical+Cognitive+Psychosocial subscales.

Change in the MFIS (Total MFIS Score, and three subscale score) from baseline to Week 48 and change in the MFIS from baseline to Week 96 will be analyzed separately using the same statistical method. Missing values will be imputed using last-observation-carried-forward (LOCF; for each item) methodology. Ranked ANOVA test adjusting for geographical region, baseline EDSS (<4, ≥4), and baseline values MFIS will be used to compare the ocrelizumab group versus Rebif group.

#### 4.4.3.14 Change in the CES-D from Baseline to Weeks 48 and Week 96

Each of the 20 items in this instrument is assigned one value of 0, 1, 2, or 3. For all but four items (see below), the values are assigned as follows:

Rarely or none of the time (less than 1 day) = 0

Some or a little of the time (1–2 days) = 1

Occasionally or a more moderate amount of the time (3–4 days) = 2

More or all of the time (5–7 days) = 3

The four items with reversed scoring are the following:

4. I felt that I was just as good as other people.

8. I felt hopeful about the future.

12. I was happy.

16. I enjoyed life.

For the four items with reversed scoring, the values should be assigned as follows:

Rarely or none of the time (less than 1 day) = 3

Some or a little of the time (1–2 days) = 2

Occasionally or a more moderate amount of the time (3–4 days) = 1

More or all of the time (5–7 days) = 0

Once you have assigned a value for each item, compute a total, adding the values for each of the 20 items. The resulting score should range between 0 and 60.

Change in the CES-D (Total Score) from baseline to Week 48 and Week 96 will be analyzed separately using the same statistical method. Missing values will be imputed using LOCF (for each item) methodology. Ranked ANOVA test adjusting geographical region, baseline EDSS (<4, ≥4), and baseline values CES-D will be used to compare the ocrelizumab group versus Rebif group.

#### 4.4.3.15 Euro-Quality of Life 5 Dimensions (EQ-5D) Questionnaire

The Short Form (SF-36) Health Survey is a generic quality of life instrument that has been widely tested for its psychometric properties and is widely used in clinical and epidemiological studies. The SF-36 contains 36 items and measures eight health domains: vitality, physical functioning, bodily pain, general health perceptions, physical role functioning, emotional role functioning, social role functioning, and mental health. The SF-36 yields a score for each domain, as well as summary scores for the physical and mental dimensions. The details of calculation of the scores are in the manual (3).

Change in the SF-36 (total, domain and summary scores) from baseline to Week 48 and Week 96 will be analyzed separately using the same statistical method. Missing values will

be imputed using LOCF (for each item) methodology. Ranked ANOVA test adjusting geographical region, baseline EDSS (<4, ≥4), and baseline values will be used to compare the Ocrelizumab group versus Rebif group.

#### *4.4.3.16 The Short Form (SF-36) Health Survey*

The EQ-5D is a generic preference-based health-related quality of life questionnaire completed by the patient that provides a single index value for health status. It is included in the study solely to derive utilities for pharmacoeconomic evaluations rather than as a direct measure of treatment benefit. The EQ-5D consists of two parts. The first part, health state classification, contains five dimensions of health: mobility, self-care, usual activities, pain/discomfort, and anxiety/depression. Each dimension has 3 possible levels. Different combinations of responses are utility-weighted to produce a single health utility index. The second part consists of a visual analog scale (VAS) measuring current health on a scale between “worst imaginable“ and “best imaginable“ health states.

Since the EQ-5D is included to inform pharmacoeconomic evaluations but not as a direct measure of treatment benefit, descriptive results of the EQ-5D data during different phases of the study (at baseline, Weeks 48 and 96) will be presented. Change from baseline in EQ-5D score will not be evaluated.

#### *4.4.3.17 Patient Perception of Treatment Questionnaire*

The Patient Perception of Treatment Questionnaire will be assessed before administration of PRO instruments at each visit in which PRO assessments are made. Specifically, patients will be asked two questions:

1) Which treatment do they think is more effective, the one they get or the treatment in the other study group;

2) Whether they think their MS has become better, become worse or been stable since baseline.

The number and percentage of patients in each category at each visit will be summarized by treatment group for the above two questions. The proportions between the two treatment groups at each visit will be compared using Cochran-Mantel-Haenszel $\chi^2$ test stratified by geographical region and baseline EDSS (<4, >=4).

#### *4.4.3.18 The change in Karnofsky Performance Status Scale (clinician-reported version) from baseline to Weeks 24, 48, 72, and 96*

The Karnofsky Performance Scale Index allows patients to be classified as to their functional impairment. The Karnofsky score is range from 0–100. The lower the Karnofsky score, the worse outcome of the disease.

Change in the Karnofsky score from baseline to Week 24, Week 48, Week 72 and Week 96 will be analyzed separately using the same statistical method. Missing values will be imputed using LOCF methodology. Ranked ANOVA test adjusting geographical region, baseline EDSS (<4, ≥4), and baseline values will be used to compare the ocrelizumab group versus Rebif group at each time point.

### 4.4.4 Subgroup Analyses

The primary/key secondary endpoints (12/24 week CDP) will be summarized by the following subgroups,

- Age (≥40 versus <40; by baseline median);
- Sex
- Race (White versus other)
- Baseline EDSS (<4 versus ≥4; by baseline median)
- Baseline Gd lesion (0 versus >0)
- Region (North American versus other )
- Historical MS therapy (immuno-modulatory therapy at any time Yes / No)
- History of relapse during 2 year prior to randomization (<2, 2, >2)
- History of relapse during 1 year prior to randomization (<1, 1, >1)
- More than 2 relapses during 2 years prior to randomization, or more than 1 relapse during 1 year prior to randomization or Baseline Gd lesion >0 (Yes/No)
- HAHA (positive (had positive values anytime after first dose of study treatment and before Week 96; negative)

Note: Clinical Science will provide a list of common key previous non-steroid treatments for MS and Biostatistics will provide an actual list of treatments on the database for Science review.

### 4.4.5 Data from Safety Follow Up

For the primary endpoint, ocrelizumab data from treatment period and safety follow-up will be compared to assess maintenance effect and the potential withdrawal effect of ocrelizumab. Moreover, safety follow-up data from ocrelizumab and Rebif arms will be compared (for the primary endpoint too). Same analysis method will be used as the primary analysis.

## 4.5 Pharmacokinetic and Pharmacodynamic Analyses

### 4.5.1 Pharmacokinetic Analysis

Ocrelizumab serum concentration-time data will be modeled using a population approach. The primary population PK parameters (Clearances and Volumes) for ocrelizumab will be estimated by means of NONMEM analysis of the sparse PK data. Clearances with associated inter-patient variability may be characterized by a saturable and non-saturable clearance as well as an intercompartmental clearance depending on the final structural model. Volumes with associated inter-patient variability may be characterized by central and peripheral volumes depending on the final structural model. Exposure (AUC) to ocrelizumab will be estimated. The selection of other parameters will depend on the final PK model used for this analysis.

Nonlinear mixed effects modeling (with software NONMEM [1]) will be used to analyze the sparse sampling dose-concentration-time data of ocrelizumab. Patients who have measurable concentrations of ocrelizumab will be included in the PK analysis unless major protocol deviations or unavailability of information (e.g., exact blood sampling time) occurred which may interfere with PK evaluation. The PK data of this study may be pooled with more extensive data from other studies. Population PK parameters (Clearances and Volumes) will be estimated and the influence of covariates, such as age, gender, weight, HAHA, and baseline CD19 lymphocytes, on these parameters will be investigated.

Details of the mixed-effects modeling analyses will be described in a Modeling and Simulation Analysis Plan and results will be reported separately.

#### 4.5.2 Pharmacodynamic Analysis

The relationship between individual ocrelizumab exposure and selected safety and efficacy parameters will be analyzed and explored, in order to characterize the exposure/dose response curve of ocrelizumab. This may include but is not limited to annualized relapse rate, T1 and T2 lesions at Week 96, infusion related reactions (IRRs), infections, and other AEs or safety parameters of interest. Other exploratory analyses may be performed to assess the possible relationship between PD markers, e.g., CD19 count, PK, and clinical response.

### 4.6 Safety Analyses

Safety data include but are not restricted to MRI data (non-MS findings), adverse event data, laboratory data, previous and concomitant treatment data, infusion information including infusion related reactions data, withdrawal data, death data, vital signs and dosing information. The safety analyses will include all safety data available (up to the date of data cut for the database lock). This will include any double blind and safety follow-up data.

#### 4.6.1 Exposure of Study Medication

The amount (mg) of ocrelizumab infused at each infusion will be listed and summarized using descriptive statistics. The amount (mg) of Rebif administrated will be listed and summarized using descriptive statistics.

#### 4.6.2 Columbia-suicide severity rating scale (C-SSRS)

Results of C-SSRS will be listed for each visit by patient.

#### 4.6.3 Adverse Events

Adverse events will include all those recorded on the “adverse event” and “infusion related reaction (IRR)” CRF pages.

For each adverse event (AE) recorded, the term entered by the investigator describing the event (the “reported term”) will be assigned a standardized term (the “preferred term”) based on the MedDRA World Health Organization (WHO) dictionary of terms. All analyses of adverse event data will be performed using the preferred terms unless otherwise specified.

All AEs will be mapped to preferred terms and super class terms. For all summary tables, the adverse events will be sorted by body system (in decreasing order of overall incidence), and then by preferred term (in decreasing order of overall incidence).

All summaries and listings of adverse events will be based on the safety population, unless otherwise stated. Summaries of adverse events will be generated summarizing the incidence of treatment-emergent adverse events only. Treatment-emergent events are defined as those AEs with observed or imputed onset date on or after the start date of trial treatment. Only where the most extreme intensity is greater than the initial intensity will events with an onset date prior to the start of trial treatment (and with an end date on or after the start of trial treatment) be considered treatment-emergent. An AE with a completely missing non-imputed start date will be assumed to be treatment-emergent unless the AE has a complete non-imputed end date that is prior to study Day 1.

For each treatment group, the incidence count for each adverse event preferred term will be defined as the number of patients reporting at least one treatment-emergent occurrence of the event. The incidence rate will be calculated as the incidence count divided by the total number of patients in the population. Each table will also present the overall number of patients experiencing at least one AE and the total number of adverse events reported (multiple occurrences of the same adverse event in one patient will be counted only once).

Serious infections will be presented with and without the inclusion of non serious infections requiring IV anti-infective treatments. If the conclusions drawn from both analyses differ then case by case adjudication of the seriousness of the non serious infections requiring IV anti-infectives will be performed and discussed.

Rate per 100 patient years by treatment group (along with the 95% CI) will be calculated for infections and serious infections.

- The number of AEs per 100 patient years is then calculated as:

  (total number of adverse events/total number of patient years)*100

- The 95% confidence interval for the number of AEs per 100 patient years is calculated as:

$$100 \times \exp\left[\ln\left(\frac{numAE}{numyear}\right) \pm 1.96 \times \sqrt{\frac{1}{numAE}}\right]$$

Where *numAE* is the total number of AEs and *numyear* is the total number of patient years.

For IRR, the number and percentage of patients with at least one infusion reaction by infusion will be presented (patients with multiple events within an infusion will count only once). In addition, the total number of infusion related reactions will be summarized (multiple events will be counted). The total and percentage (based on the total number of patients with at least one IRR) by most extreme intensity will be summarized. For multiple events within a patient by intensity only, the most extreme intensity will be taken and the total of each intensity will only sum to the total number of patients with at least one IRR if there are no missing extreme intensities.

For AEs leading to death, the most extreme intensity will be overwritten by CTC Grade 5 (death).

### 4.6.4 MRI data

Non-MS pathology reported by local safety radiologist will be summarized by treatment group.

### 4.6.5 Laboratory Data

Abnormal lab outcomes will be reported. A summary of the number and percentage of patients with abnormal lab outcomes along with each CTC grade per laboratory parameter will be produced by treatment group.

Absolute value and change from baseline values at each visit will be summarized for Circulating B-cell subsets, T cells, natural killer cells, other leukocytes, and plasma immunoglobulins.

HAHA and antibody titers for mumps, rubella, varicella, and Streptococcus pneumoniae will be summarized at each visit.

Any JCV plasma/urine results will be listed.

### 4.6.6 Other

The results of vital sign, physical examination, and ECG will be included in individual patient listings. Change from baseline in vital signs will be summarized by groups.

## 5. REFERENCES


1. Categorical Data Analysis using the SAS system. Stokes, Davis, and Koch.

2. Beal SL., Boeckman AJ., Sheiner LB. NONMEM User's Guide, Parts I-VIII San Francisco: Division of Clinical Pharmacology- University of California, 1992.

3. Ware JE, Kosinski M & Gandek B. SF-36 Health Survey. Manual & Interpretation Guide. Quality Metric Incorporated. Lincoln, RI. 2000.

# Appendix A Protocol Synopsis

# SYNOPSIS OF PROTOCOL NUMBER WA21093

| | | | |
|---|---|---|---|
| TITLE | **A Randomized, Double-Blind, Double-Dummy, Parallel-Group Study to Evaluate the Efficacy and Safety of Ocrelizumab in Comparison to Interferon Beta-1a (Rebif®) in Patients with Relapsing Multiple Sclerosis** | | |
| SPONSOR | F.Hoffmann-La Roche Ltd<br>Genentech Inc | CLINICAL PHASE | III |
| INDICATION | Relapsing Multiple Sclerosis | | |

OBJECTIVES

**Primary:**

The primary objective of this study is to assess whether the efficacy of ocrelizumab 600 mg (given as dual infusions of 300 mg ocrelizumab 14 days apart) intravenously every 24 weeks is superior to Rebif® as measured by the annualized protocol-defined* relapse rate by two years (96 weeks) in patients with relapsing multiple sclerosis.

**Secondary:**

To evaluate whether the efficacy of ocrelizumab is superior to Rebif®, as reflected by the following measures:

- The time to onset of sustained disability progression for at least 12 weeks during the 96-week comparative treatment period.*
- The time to onset of sustained disability progression for at least 24 weeks during the 96-week comparative treatment period.*
- The proportion of relapse-free patients by 96 weeks.
- The change in total T2 lesion volume as detected by brain MRI from baseline to Week 96.
- The total number of new, and/or enlarging T2 hyperintense lesions as detected by brain MRI at weeks 24, 48 and 96.
- The change in Multiple Sclerosis Functional Composite Scale (MSFCS) score from baseline to Week 96.
- The change in brain volume as detected by brain MRI from Week 24 to Week 96.

**See section: “Assessments of efficacy” for the definition*

**Safety:**

To evaluate the safety and tolerability of ocrelizumab 600 mg (given as dual infusions of 300 mg 14 days apart) every 24 weeks, in patients with relapsing MS.

**Pharmacokinetics/Pharmacodynamics:**

To explore the pharmacokinetics, immunogenicity and pharmacodynamics of ocrelizumab in patients with relapsing MS.

**Exploratory objectives:**

- The annualized relapse rate, based on clinical and protocol-defined relapses at the end of the 96-week comparative treatment period.
- The total number of T1 gadolinium-enhanced lesions as detected by brain MRI at weeks 24, 48 and 96.
- The change in brain volume as detected by brain MRI from baseline to Week 96.
- The change in brain volume as detected by brain MRI from week 48 to Week 96.
- The change in Multiple Sclerosis Functional Composite Scale (MSFCS) score from baseline to Week 48.

| | |
|---|---|
| | • The cumulative change in EDSS scores, measured in area under the curve (AUC) by Week 96.<br>• The change in EDSS from baseline to Weeks 48 and 96.<br>• The change in timed 25 foot walk from baseline to Weeks 48 and 96.<br>• The change in 9-hole peg test from baseline to Weeks 48 and 96.<br>• The change in paced auditory serial addition test (PASAT) from baseline to Weeks 48 and 96.<br>• The time to onset of sustained 20% increase in 9-hole peg test for at least 12 weeks.<br>• The time to onset of sustained 20% increase in timed 25 foot walk for at least 12 weeks.<br>• Patient Reported Outcomes (PROs): Modified Fatigue Impact Scale (MFIS), EuroQol instrument (EQ-5D), Center for Epidemiology Studies Depression Scale (CES-D) and Medical Outcomes Study 36-Item Short Form Survey Instrument (SF-36) at baseline, Week 48 and Week 96.<br>• The change in Karnofsky Performance Status Scale (clinician-reported version) from baseline to Weeks 48 and 96. |
| TRIAL DESIGN | Multicentre, randomized, double-blind, double-dummy, parallel-group study |
| NUMBER OF SUBJECTS | 800 patients in total, 400 patients per group using a 1:1 randomization ratio. Please refer to Sample Size and Statistical Analyses section of the synopsis for more details. |
| TARGET POPULATION | **Inclusion criteria:**<br>1. Ability to provide written, informed consent and to be compliant with the schedule of protocol assessments.<br>2. Ages 18-55 years at screening, inclusive.<br>3. Diagnosis of MS, in accordance with the revised McDonald criteria (2005).<br>4. At least 2 documented clinical attacks within the last 2 years prior to screening or one clinical attack in the year prior to screening (but not within 30 days prior to screening).<br>5. Neurological stability for ≥ 30 days prior to both screening and baseline.<br>6. EDSS, at screening, from 0 to 5.5 inclusive.<br>7. Documented MRI of brain with abnormalities consistent with MS prior to screening.<br>8. Patients of reproductive potential must use reliable means of contraception as described below as a minimum (adherence to local requirements, if more stringent, is required*):<br>• Two methods of contraception throughout the trial, including the active treatment phase AND for 48 weeks after the last dose of ocrelizumab, or until their B-cells have repleted, whichever is longer.<br>Acceptable methods of contraception include one primary (e.g., systemic hormonal contraception or tubal ligation of the female partner, vasectomy of the male partner) AND one secondary barrier method (e.g., latex condoms, spermicide) OR a double barrier method (e.g., latex condom, intrauterine device, vaginal ring or pessary plus spermicide [e.g., foam, vaginal suppository, gel, cream]). |

9. For patients of <u>non reproductive potential (</u>adherence to local requirements, if more stringent, is required*):
   - Women may be enrolled if postmenopausal (i.e., spontaneous amenorrhea for the past year confirmed by an FSH level greater than 40 mIU/mL) unless the patient is receiving a hormonal therapy for their menopause or surgically sterile (i.e., hysterectomy, complete bilateral oophorectomy);
   - Men may be enrolled if they are surgically sterile (castration).

* Based on local Ethics Committees or National Competent Authority feedback, additional requirements to assure contraception or to confirm menopause may be required (e.g. serum estradiol compatible with post-menopause status, longer duration of amenorrhea, higher level of FSH).

**<u>Exclusion Criteria</u>**

Patients who meet the following criteria must be excluded from study entry:

1. Diagnosis of primary progressive MS.
2. Disease duration of more than 15 years in patients with an EDSS ≤ 2.0 at screening.
3. Inability to complete an MRI (contraindications for MRI include but are not restricted to claustrophobia, weight ≥ 140 kg, pacemaker, cochlear implants, presence of foreign substances in the eye, intracranial vascular clips, surgery within 6 weeks of entry into the study, coronary stent implanted within 8 weeks prior to the time of the intended MRI, etc).
4. Known presence of other neurological disorders which may mimic MS including but not limited to: neuromyelitis optica, Lyme disease, untreated vitamin B12 deficiency, neurosarcoidosis and cerebrovascular disorders.

**<u>Exclusions Related to General Health</u>**

5. Pregnancy or lactation.
6. Any concomitant disease that may require chronic treatment with systemic corticosteroids or immunosuppressants during the course of the study.
7. History or currently active primary or secondary immunodeficiency.
8. Lack of peripheral venous access.
9. History of severe allergic or anaphylactic reactions to humanized or murine monoclonal antibodies.
10. Significant or uncontrolled somatic disease or any other significant disease that may preclude patient from participating in the study.
11. Congestive heart failure (NYHA III or IV functional severity).
12. Known active bacterial, viral, fungal, mycobacterial infection or other infection, excluding fungal infection of nail beds.

13. Infection requiring hospitalization or treatment with i.v. antibiotics within 4 weeks prior to baseline visit or oral antibiotics within 2 weeks prior to baseline visit.
14. History or known presence of recurrent or chronic infection (e.g., hepatitis B or C, HIV, syphilis, tuberculosis).
15. History of progressive multifocal leukoencephalopathy (PML)
16. History of malignancy, including solid tumors and hematological malignancies, except basal cell carcinoma, *in situ* squamous cell carcinoma of the skin, and *in situ* carcinoma of the cervix of the uterus that have been previously completely excised with documented, clear margins.
17. History of alcohol or drug abuse within 24 weeks prior to baseline.
18. History or laboratory evidence of coagulation disorders.

**Exclusions Related to Medications***

19. Receipt of a live vaccine within 6 weeks prior to baseline.

*In rare cases when patient requires vaccination with a live vaccine, the screening period may be extended but cannot exceed 8 weeks.*

20. Treatment with any investigational agent within 24 weeks of screening (Visit 1) or five half-lives of the investigational drug (whichever is longer).
21. Contraindications to or intolerance of oral or i.v. corticosteroids, including methylprednisolone administered i.v., according to the country label, including:
    a) Psychosis not yet controlled by a treatment;
    b) Hypersensitivity to any of the constituents.
22. Contraindication to Rebif® or incompatibility with Rebif® use, including:
    a) Current severe depression and/or suicidal ideation;
    b) Hypersensitivity to natural or recombinant interferon-β, or to any excipients;
    c) Previous suboptimal response to High Dose High Frequency (HDHF) interferon or cessation of HDHF interferon therapy due to poor tolerability;
    d) Prior cessation of Rebif® therapy due to toxicity, which is likely to recur.
23. Treatment with β interferons (with exemptions for HDHF interferon as listed above), glatiramer acetate, plasmapheresis, or other immunomodulatory therapies within 4 weeks prior to baseline.
24. Treatment with dalfamipridine (Ampyra®) unless on stable dose for ≥ 30 days prior to screening. Patients should remain on stable doses throughout the 96 week treatment period.
25. Previous treatment with B-cell targeted therapies (i.e. rituximab, ocrelizumab, atacicept, belimumab or ofatumumab).
26. Systemic corticosteroid therapy within 4 weeks prior to screening**.
27. Any previous treatment with alemtuzumab (Campath), anti-CD4, cladribine, mitoxantrone, daclizumab, BG12, teriflunomide, laquinimod, total body irradiation or bone marrow transplantation.

28. Treatment with cyclophosphamide, azathioprine, mycophenolate mofetil (MMF), cyclosporine, methotrexate or natalizumab within 24 months prior to screening. NB. P*atients previously treated with natalizumab will be eligible for this study only if duration of treatment with natalizumab was < 1 year.*
29. Treatment with fingolimod (FTY720, Gilenya®) or other S1P receptor modulator (i.e., BAF312) within 24 weeks prior to screening. NB.*Only patients with T lymphocyte count ≥ LLN will be eligible for this study.*
30. Treatment with i.v. immunoglobulin within 12 weeks prior to baseline.

* *Patients screened for this study should not be withdrawn from therapies for the sole purpose of meeting eligibility for the trial. Patients, who discontinue their current therapy for non-medical reasons, should specifically be informed before deciding to enter the study of their treatment options.*

** *The screening period may be extended (but cannot exceed 8 weeks) for patients who have used systemic corticosteroids for their MS before screening. For a patient to be eligible, systemic corticosteroids should not have been administered also between screening and baseline.*

**Exclusions Related to Laboratory Findings***

31. Positive serum β hCG measured at screening.
32. Positive screening tests for hepatitis B (hepatitis B surface antigen [HBsAg] positive, or positive hepatitis B core antibody [total HBcAb] confirmed by a positive viral deoxyribonucleic acid [DNA] polymerase chain reaction [PCR]) or hepatitis C (HepCAb).
33. Positive rapid plasma reagin (RPR).
34. CD4 count < 300/µL.
35. AST/SGOT or ALT/SGPT ≥ 2.0 Upper Limit of Normal (ULN).
36. Platelet count <100,000/µL (<100 x $10^9$/L).
37. Levels of serum IgG <5.65 g/L.
38. Levels of serum IgM < 0.55 g/L.
39. Total neutrophil count <1.5 x $10^3$/µL.

**Re-testing before baseline: in rare cases in which the screening laboratory samples are rejected by the central laboratory (example: hemolyzed sample) or the results are not assessable (example: indeterminate) or abnormal, the tests need to be repeated within 4 weeks. The last value before randomization must meet study criteria. In such circumstances, the screening period may need to be prolonged but should not exceed 8 weeks.*

Please note: based on local Ethics Committees or National Competent Authority requirements, additional diagnostic testing may be required for selected patients or selected centers to exclude tuberculosis, Lyme disease, HTLV-1 associated myelopathy (HAM), acquired immune deficiency syndrome (AIDS), hereditary disorders, connective tissue disorders, or sarcoidosis. Other specific diagnostic tests may be requested when deemed necessary by the investigator.

| | |
|---|---|
| LENGTH OF STUDY | The study will consist of the following periods:<br>- **Screening period**: 2 weeks.<br>*In rare case (i.e., if the screening laboratory samples are rejected by the central laboratory) the screening period may be extended but cannot exceed 8 weeks.*<br>**- Treatment period:** 96 weeks<br>The double-blind comparative treatment period will consist of 4 treatment cycles 24 weeks apart.<br>*Patients who complete the 96 week treatment period may become eligible for a separate, open-label extension study, under a separate protocol.*<br>**-Safety Follow up Period**: at least 48 weeks;<br>Patients who withdraw from study treatment will be observed for a period of at least 48 weeks counting from the date of the last infusion of study drug. If at this time the peripheral blood B-cells remain depleted patient should continue to be monitored at 24-week intervals until B-cell count has returned to the baseline value or to the lower limit of the normal range (whichever is the lower).<br>See section "Procedures" below for more details. |
| END OF STUDY | The end of the study has been defined as the date at which the last data point from the last patient, which was required for statistical analysis as defined in Data Analysis Plan (DAP), was received. |
| INVESTIGATIONAL MEDICAL PRODUCT(S) DOSE/ ROUTE/ REGIMEN | – Group A (Ocrelizumab): Ocrelizumab 600 mg (given as a dual i.v. infusions of 300 mg 14 days apart) every 24 weeks.<br>– Group B (Interferon beta-1a (Rebif®))<br>Rebif® will be administered as follows:<br>▪ Treatment initiation:<br>⇒ during weeks one and two, Rebif® 8.8 µg (one pre-filled syringe (0.2 ml ) containing 8.8 µg (2.4 MIU) of interferon beta-1a given via subcutaneous injection three times per week<br>⇒ during weeks three and four, Rebif® 22 µg (one pre-filled syringe (0.5 ml ) containing 22 µg (6 MIU) of interferon beta-1a) given via subcutaneous injection three times per week<br>▪ Treatment continuation:<br>⇒ From the fifth week onwards, Rebif® 44 µg (one pre-filled syringe (0.5 ml ) containing 44 µg (12 MIU) of interferon beta-1a) given via subcutaneous injection three times per week<br>⇒ A lower dose of 22 µg, given via subcutaneous injection three times per week, will be available for patients who cannot tolerate the 44 µg dose of Rebif®. *Please refer to detailed guidelines in the study protocol.*<br>Patients randomized to active ocrelizumab group will also receive dummy placebo of Rebif® (administered via subcutaneous injection three times per week).<br>Patients randomized to active Rebif® group will also receive dummy placebo of ocrelizumab (administered as intravenous infusions at similar time points to those of the ocrelizumab group). |

| | |
|---|---|
| | Dummy placebos of ocrelizumab and of Rebif® will be similar in appearance and administration as the investigational product. |
| NON-INVESTIGATIONAL MEDICAL PRODUCT(S) | Ocrelizumab/ocrelizumab dummy placebo: Thirty minutes prior to the start of each infusion, patients will receive a methylprednisolone 100 mg i.v. infusion. Pre-infusion treatment with an oral analgesic/antipyretic (e.g.: acetaminophen), and an oral antihistamine (e.g. diphenhydramine) is also recommended.<br>Rebif®/ Rebif® dummy placebo: Pre-treatment with non-steroid anti-inflammatory drugs (ibuprofen) or acetaminophen are recommended; investigators should follow their local label for further information. |
| ASSESSMENTS OF:<br>- EFFICACY | – **Assessment of clinical and protocol defined relapses**<br>▪ Protocol-defined relapse is the occurrence of new or worsening neurological symptoms attributable to MS. Symptoms must persist for >24 hours and should not be attributable to confounding clinical factors (e.g. fever, infection, injury, adverse reactions to medications) and immediately preceded by a stable or improving neurological state for least 30 days. The new or worsening neurological symptoms must be accompanied by objective neurological worsening consistent with an increase of at least half a step on the EDSS scale, or 2 points on one of the appropriate FSS, or 1 point on two or more of the appropriate FSS. The change must affect the selected FSS (i.e., pyramidal, ambulation, cerebellar, brainstem, sensory, or visual). Episodic spasms, sexual dysfunction, fatigue, mood change or bladder or bowel urgency or incontinence will not suffice to establish a relapse. NB: Sexual dysfunction and Fatigue will not be scored.<br>⇨ *Please note: adjudication of protocol-defined relapses will be performed by the Sponsor based on pre-specified criteria, applied to data collected by investigator, in a blinded fashion*.<br>– **Brain MRI acquisition sequences**<br>▪ T1-weighted MRI (without gadolinium-enhancement)<br>▪ T1-weighted MRI (with gadolinium-enhancement)<br>▪ Fluid-attenuated, inversion recovery (FLAIR), Proton-density–weighted and/or T2-weighted MRI<br>– **Assessment of sustained disability progression**<br>▪ Disability progression is defined as an increase of ≥ 1.0 point from the baseline EDSS score that is not attributable to another etiology (e.g. fever, concurrent illness, or concomitant medication) when the baseline score is 5.5 or less, and ≥ 0.5 when the baseline score is above 5.5. Disability progression is considered sustained when the increase in the EDSS is confirmed at regularly scheduled visits at least 12 and 24 weeks after the initial documentation of neurological worsening.<br>▪ Sustained disability progression, confirmed for both 12 and 24 weeks after the initial documentation of neurological worsening, will be analyzed.<br>The following instruments will be used to assess the patient's functional |

| | |
|---|---|
| | ability: The Kurtzke Expanded Disability Status Scale (EDSS), Multiple Sclerosis Functional Composite Scale (MSFCS) and Karnofsky Performance Status Scale (clinician-reported version). |
| - SAFETY | Adverse events, vital signs, weight, physical and neurological examination, clinical laboratory tests, 12 lead ECG, locally reviewed MRI for safety (non-MS CNS pathology), concomitant medications.<br>Pregnancy tests [serum/urine beta subunit human chorionic gonadotropin (beta hCG)] will be performed in women of childbearing potential. Plasma and urine samples will be collected for JCV assessments.<br>Columbia-Suicide Severity Rating Scale (C-SSRS) will be used for prospective suicidality assessment. |
| - PHARMACOKINETICS/ PHARMACODYNAMICS | Blood samples will be taken for measurement of ocrelizumab serum concentration at the time points detailed in the Schedule of Assessments. Nonlinear mixed effects modeling will be used to analyze the sparse sampling dose-concentration-time data of ocrelizumab in order to assess the pharmacokinetics. Population PK parameters will be estimated and the influence of covariates, such as age, gender, weight, HAHA, and CD19 lymphocyte count, investigated. The relationship between ocrelizumab exposure and selected safety and efficacy parameters will be analyzed and explored in order to characterize the exposure-response curve of ocrelizumab. |
| – IMMUNOGENICITY | Serum samples for measurement of HAHA (Human Anti-human Antibodies) to ocrelizumab are collected according to the Schedule of Assessments. |
| – PATIENT REPORTED OUTCOMES (PRO) | ▪ Modified Fatigue Impact Scale (MFIS Standard)<br>▪ Center for Epidemiology Studies Depression Scale (CES-D3)<br>▪ Medical Outcomes Study 36-Item Short Form Survey Instrument (SF-36: SF-36v2 Standard)<br>▪ EuroQol instrument (EQ-5D-3L-Tablet) |
| – PROTEIN BIOMARKER SAMPLES | Specimens for protein biomarker discovery and validation will be collected from all patients. These specimens will be used for research purposes to identify and/or verify protein biomarkers that are predictive of response to ocrelizumab treatment (in terms of dose, safety and tolerability) and will help understand the pathogenesis, course and outcome of multiple sclerosis and related diseases. In addition, screening technologies for larger numbers of proteins and antibodies may also be used to discover novel antibody associations with MS, disease progression and response to therapy.<br>Analyses will include but may not be limited to Complement Factor H (CFH) and the B-cell activating factor (BAFF).<br>6 ml sample of whole blood will be collected in a plain tube without EDTA for serum isolation. Blood specimens for protein biomarker discovery and validation will be collected from all patients as per Schedule of Assessments. These specimens will be stored for 5 years after the end of the study and then destroyed. |
| - EXPLORATORY BIOMARKERS (non-DNA) | Roche Clinical Repository (RCR) non-DNA, (dynamic, non-inherited) RNA specimen and plasma for biomarker discovery and validation will be collected only from patients consenting to RCR as per Schedule of Assessments. RCR samples (2 x approximately 2.5 mL of blood collected in PAXgene vacutainers and 6 ml of blood collected in a tube with EDTA for plasma isolation) will be collected to promote, facilitate and improve individualized healthcare by better understanding/predicting ocrelizumab efficacy, dose responses, safety, ocrelizumab mode of action, progression of multiple sclerosis and associated diseases. These specimens may be stored for up to 15 years after the end of the study. |

- EXPLORATORY BIOMARKERS (DNA)

All patients who have been enrolled in the study will be asked to donate an optional DNA specimen (by written informed consent) for pharmacogenetic and genetic research.

RCR DNA sampling will involve taking one sample of 6 mL of blood taken as per Schedule of Assessments. The study protocol which includes RCR sampling is submitted to the concerned Ethics Committee and is available for Competent Authority review upon request. These specimens will be stored for up to 15 years after the end of the study.

PROCEDURES (summary):

**Figure 1 Overview of Study Design**

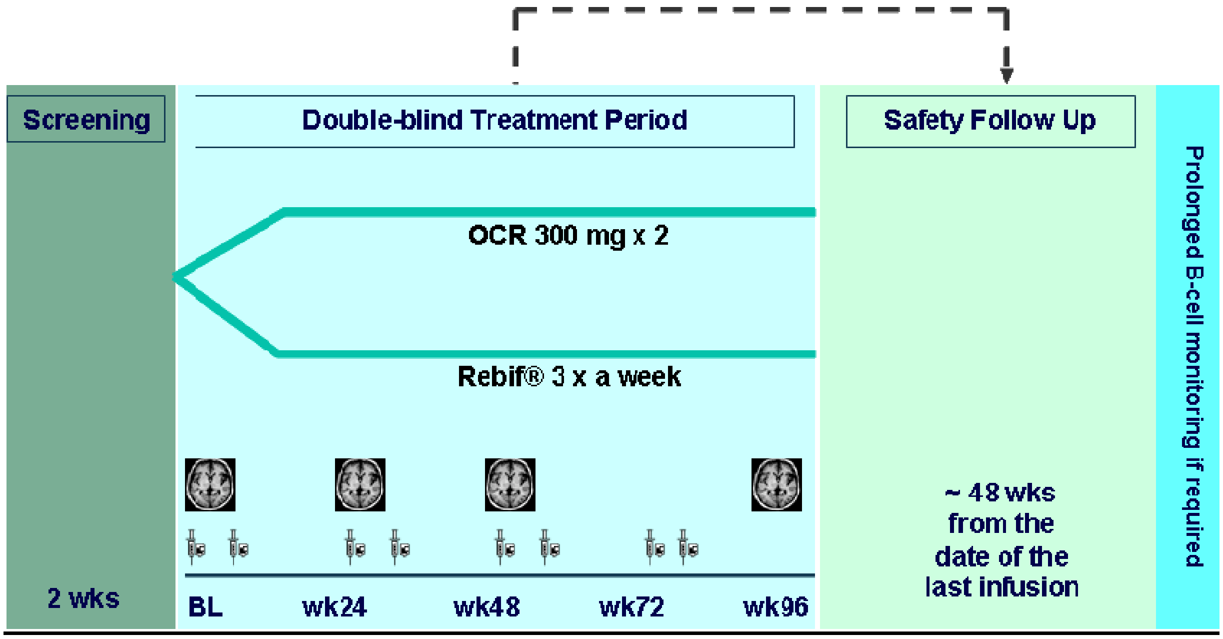


Please note: patients who complete the 96 week treatment period may become eligible for a separate, open-label extension study, under a separate protocol.

## Screening:

Consenting patients will enter the 2-week screening period to be evaluated for eligibility. Procedures at screening will include collecting medical history, medical examination including thorough neurological exam, EDSS score, MSFCS score, ECG, blood and urine sampling. Please see Table 3 - "Schedule of Assessments: Screening Through the End of Double-Blind Treatment Period" for further details.

*Please note that based on local Ethics Committees or National Competent Authority requirements, additional diagnostic testing may be required for selected patients or selected centers to exclude tuberculosis, Lyme disease, HTLV-1 associated myelopathy (HAM), acquired immune deficiency syndrome (AIDS), hereditary disorders, connective tissue disorders, or sarcoidosis.*

## Treatment Period:

Eligible patients will be randomized via IxRS into one of two treatment groups: ocrelizumab 600 mg regimen (group A) or interferon beta-1a (Rebif®) (group B) – please refer to Table 1 and Table 2 for more details.

To prevent potential unblinding as a result of adverse events or changes to laboratory results, the following, additional measures have been implemented:

- **The Examining Investigator/EDSS assessor** will perform the neurological examination, document the Kurtzke Functional Systems (KFS) scores and assess EDSS scores. The examining physician/EDSS assessor will not be involved with any aspect of medical management of the patient and will not have access to patient data. Every effort will be made to ensure that there is no change in the examining physician/EDSS assessor throughout the course of the study for any individual patient. The examining physician/EDSS assessors will be trained and instructed not to discuss what adverse effects (if any) the patient is experiencing from their medication. Examining physician/EDSS assessors will receive training in performing EDSS assessments prior to the beginning of the study and must have successfully passed an examination on performance of the *Neurostatus EDSS examination* within 24 months of participation. All examining physician/EDSS assessors will receive ongoing training on performance of the *Neurostatus EDSS examination* throughout the course of the study.

- **Patient education:** Prior to being examined by the Examining Investigator/EDSS assessor, patients will be instructed not to discuss what (if any) adverse effects they may be experiencing. Treating physicians and/or study coordinators should remind patients of these instructions prior to EDSS assessments and this should be documented in the source documents.

- **Blinded, central MRI assessments:** A blinded, central MRI reader will assess all on-study MRI scans. These assessments will provide independent confirmation of the relative changes in immune-mediated, CNS damage.

- **Blinding of laboratory parameters:** Laboratory parameters that may lead to unblinding to treatment assignment, such as FACS cell counts including $CD19^+$ cells, lymphocyte count, IgM and IgG levels, and type I interferon neutralizing antibody levels will be blinded in all patients, except those meeting unblinding criteria for safety reasons.

During the treatment period, patients will be assessed at clinical visits as per Schedule of Assessments: Screening Through the End of Treatment Period – please refer to Table 3 for further details.

Prior to the next infusion of study drug, patients will be evaluated for pre-specified conditions and laboratory abnormalities to allow for re-treatment.

**Patients who complete the 96 week treatment period may become eligible for a separate open-label extension study, under a separate protocol.**

## Safety Follow up Period

Patients who discontinue treatment with study drug will enter the Safety Follow up Period for at least 48 weeks counting from the date of the last infusion of the ocrelizumab/placebo. However, if after this time the peripheral blood B-cells remain depleted, patient should continue to be monitored at 24-week intervals until B-cell count has returned to the baseline value or to the lower limit of the normal range (whichever is the lower). Please refer to Figure 2 for more details.

**Figure 2 Safety Follow up – Prolonged B-cell Monitoring Period**

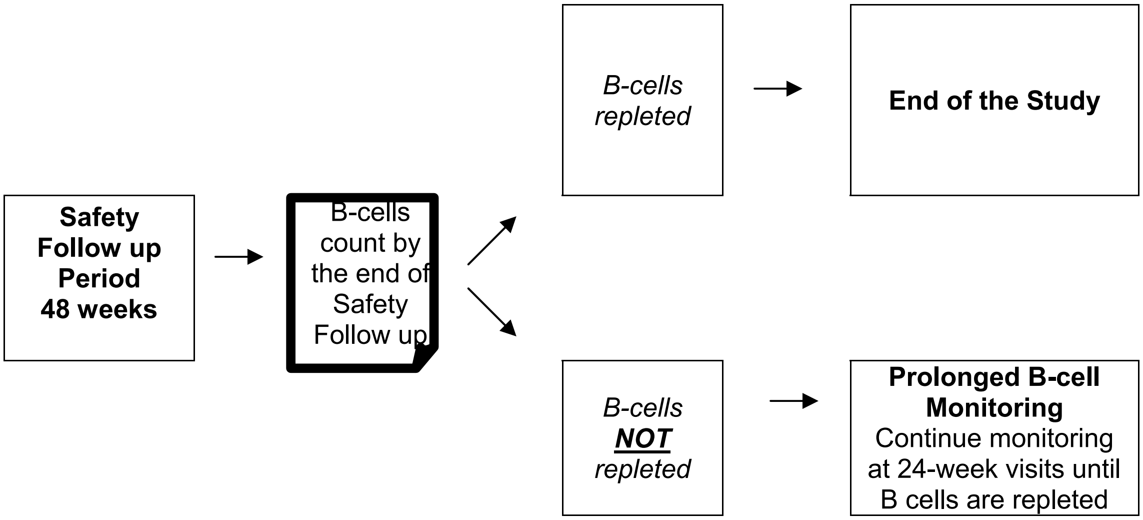


*Please note: patients in Safety Follow up who receive other B-cell targeted therapies will only be followed for 48 weeks from the date of the last infusion of the study drug regardless of their B-cell count.*

During Safety Follow up patients will be assessed at clinical visits every 12 weeks as per Schedule of Assessments. Telephone interviews will be performed every 4 weeks. If prolonged B-cell monitoring is required, patients will be assessed at clinical visits every 24 weeks (as per Schedule of Assessments) and telephone interviews will be performed every 12 weeks. Please refer to Table 4 for further details.

Please note: It is important to distinguish between "withdrawal from treatment" and "withdrawal from study". Patients who withdraw from treatment should be encouraged to remain in the study for the full duration of the Safety Follow Up Period (minimum of 48 weeks following the last infusion).

**Every effort should be made to have patients withdrawing from the treatment complete the Safety Follow up Period and all related assessments, regardless of whether or not they receive alternative treatment for MS.**

**Table 1 Overview of Dosing Regimen**

| Group | Treatment Period[1,2] | | | | | | | |
|---|---|---|---|---|---|---|---|---|
| | 1st Cycle (Weeks 1-24) | | 2nd Cycle[3] (Weeks 24-48) | | 3rd Cycle[3] (Weeks 48-72) | | 4th Cycle[3] (Weeks 72-96) | |
| | **Day 1 Infusion** | **Day 15 Infusion** | **Week 24 Infusion** | **Week 26 Infusion** | **Week 48 Infusion** | **Week 50 Infusion** | **Week 72 Infusion** | **Week 74 Infusion** |
| **A Ocrelizumab 600 mg regimen** | **Ocrelizumab** 300 mg i.v. | **Ocrelizumab** 300 mg i.v. | **Ocrelizumab** 300 mg i.v. | **Ocrelizumab** 300 mg i.v. | **Ocrelizumab** 300 mg i.v. | **Ocrelizumab** 300 mg i.v. | **Ocrelizumab** 300 mg i.v. | **Ocrelizumab** 300 mg i.v. |
| **B Rebif® regimen[4]** | **Rebif®** s.c. three times per week | → | → | → | → | → | → | → |

1. The treatment period consists of 96 weeks of treatment; patients will receive a maximum of 4 treatment cycles.
2. Each treatment cycle has a duration of 24 weeks, and consists of two i.v. infusions separated by 14 days.
3. Prior to each infusion, a clinical evaluation will be performed to ensure that the patient remains eligible for treatment.
4. Please refer to table 2 for detailed Rebif® dosing regimen.

Please note: 100 mg of methylprednisolone i.v. will be administered in both treatment arms prior to each infusions of ocrelizumab/ ocrelizumab placebo.

## Table 2 Overview of Rebif® Dosing Regimen

| | Treatment Initiation | | Treatment Continuation | Dose modification (if required) |
|---|---|---|---|---|
| **Week** | **Weeks 1- 2** | **Weeks 3-4** | **Week 5 onwards** | – |
| *Study Day* | *1-14* | *15-28* | *29+* | At any time >29 |
| **Dose of Rebif®** | **Rebif® 8.8µg**<br>(one pre-filled syringe [0.2 ml] containing 2.4 MIU of interferon beta-1a) s.c.<br>**3x per week** | **Rebif® 22 µg**<br>(one pre-filled syringe [0.5 ml] containing 6 MIU of interferon beta-1a) s.c.<br>**3x per week** | **Rebif® 44 µg**<br>(one pre-filled syringe [0.5 m]) containing 12 MIU of interferon beta-1a) s.c.<br>**3x per week** | **Rebif® 22 µg**<br>(one pre-filled syringe [0.5 ml] containing 6 MIU of interferon beta-1a) s.c.<br>**3x per week** |

Please note: If Rebif® dose modification is required due to laboratory abnormalities possibly related to the treatment with Rebif®, the investigator (the treating physician) will need to notify IxRS and the blinded study medication (Rebif® placebo or Rebif® verum) will be dispensed accordingly. In addition, to ensure patient safety in the study, unscheduled visits may be required for additional assessments, monitoring and for dispensing study medication.

**In case of elevation of liver function tests the following rules will apply:**

| | |
|---|---|
| ⇨ **ALT ≥ 10 xULN**, jaundice or other clinical symptoms of liver dysfunction | In case of detection of elevated **ALT ≥ 10 xULN**, jaundice or other clinical symptoms of liver dysfunction the injections of Rebif®/Rebif® placebo must be discontinued permanently. The monitoring of liver function tests should be continued on a monthly basis until return to normal baseline levels or CTCAE v.4.0 grade 1 toxicity (ALT: >ULN - 3.0 x ULN). A consultation with hepatologist is recommended. Patients should move to Safety Follow up Period. |
| ⇨ **ALT ≥ 5 xULN** | In case of detection of elevated **ALT ≥ 5 xULN** (but below 10 xULN) the injections of Rebif®/Rebif® placebo must be discontinued temporarily. Additional blood chemistry panel including AST, ALP, GGT and bilirubin should be performed biweekly until no further increase is observed. Subsequently, ALT analysis has to be performed every month until return to normal baseline levels or CTCAE v.4.0 grade 1 toxicity (ALT >ULN - 3.0 x ULN). A consultation with hepatologist should be considered as per investigator judgment.<br>If causes of toxicity other than possible treatment with Rebif® are excluded, the patient may then be cautiously re-challenged with Rebif®/Rebif® placebo 22µg provided in a blinded fashion upon request to IxRS. The monitoring of liver function tests should continue on a monthly basis. If there is no further recurrence of toxicity, patient may continue treatment with Rebif®/Rebif® placebo 44 µg provided in a blinded fashion upon investigator's request to IxRS. **In case of recurrence of toxicity (ALT > 3 xULN, or other clinical symptoms of liver dysfunction) the injections of Rebif®/Rebif® placebo should be discontinued permanently.** Patients should move to Safety Follow up Period.<br><br>Please note: Re-initiation of therapy with Rebif® following elevation of liver function tests can only be considered once. |
| ⇨ **ALT > 3 xULN** | In case of detection of elevated **ALT > 3 xULN** (but below 5x ULN) additional blood chemistry panel including AST, ALP, GGT and bilirubin should be performed biweekly until no further increase is observed. Subsequently, ALT analysis has to be performed every month until return to normal baseline levels or CTCAE v.4.0 grade 1 toxicity (ALT>ULN - 3.0 x ULN). |

## Table 3. Schedule of Assessments: Screening Through the End of Double-Blind Treatment Period

| | Screen | Treatment Period | | | | | | | | | | | | | Delayed Dosing Visit [22] | Unscheduled Visit [23] | Withdrawal from Treatment Visit |
|---|---|---|---|---|---|---|---|---|---|---|---|---|---|---|---|---|---|
| **Visit** | **1** | **2 BL** | **3** | **4** | **5** | **6** | **7** | **8** | **9** | **10** | **11** | **12** | **13** | **14** | | | |
| **Week** | **-2** | **-** | **w2** | **w12** | **w24** | **w26** | **w36** | **w48** | **w50** | **w60** | **w72** | **w74** | **w84** | **w96** | | | |
| *Study Day (window in days)* | *-14* | *1* | *15 (± 1)* | *85 (±4)* | *169 (±1)* | *14 days post w24 Visit (±1)* | *253 (±4)* | *337 (±1)* | *14 days post w48 Visit (±1)* | *421 (±4)* | *505 (±1)* | *14 days post w72 Visit (±1)* | *589 (±4)* | *673 (±1)* | | | |
| | | | | | | | | | | | | | | | | | |
| Informed consent [1] | x | | | | | | | | | | | | | | | | |
| Medical history | x | | | | | | | | | | | | | | | | |
| Review of eligibility criteria | x | x | | | | | | | | | | | | | | | |
| CES-D, MFIS, EQ-5D, SF-36 | | x | | | | | | x | | | | | | x | | | x |
| Patient's Assessment of Treatment Benefit | | | | | | | | x | | | | | | x | | | x |
| C-SSRS | | x | | x | x | | x | x | | x | x | | x | x | | x | x |
| Physical examination | x | x | x | | x | x | | x | x | | x | x | | x | x | | x |
| Vital signs [2] | x | x | x | x | x | x | x | x | x | x | x | x | x | x | x | x | x |
| 12 lead ECG (pre- and post-dose) [3] | x | x | | | | | | | | | | x | | | | | x |
| Height | x | | | | | | | | | | | | | | | | |
| Weight | x | | | | | | | | | | x | | | x | | | x |
| EDSS, MSFCS, neurological exam | x | x | | x | x | | x | x | | x | x | | x | x | | x | x |
| Karnofsky Performance Status Scale | | x | | | x | | | x | | | x | | | x | | | x |
| MRI [4] | | x | | | x | | | x | | | | | | x | | | x |
| Concomitant Treatment | | x | x | x | x | x | x | x | x | x | x | x | x | x | x | x | x |
| Adverse Events | *Only SAEs* | x | x | x | x | x | x | x | x | x | x | x | x | x | x | x | x |
| Potential relapses recorded | | x | x | x | x | x | x | x | x | x | x | x | x | x | x | x | x |
| Telephone interview (every 4 wks) [5] | | | | → | → | → | → | → | → | → | → | → | → | → | | | x |

## Table 3. Schedule of Assessments: Screening Through the End of Double-Blind Treatment Period (Cont.)

| | Screen | Treatment Period | | | | | | | | | | | | | Delayed Dosing Visit [22] | Unscheduled Visit [23] | Withdrawal from Treatment Visit |
|---|---|---|---|---|---|---|---|---|---|---|---|---|---|---|---|---|---|
| **Visit** | **1** | **2 BL** | **3** | **4** | **5** | **6** | **7** | **8** | **9** | **10** | **11** | **12** | **13** | **14** | | | |
| **Week** | **-2** | **-** | **w2** | **w12** | **w24** | **w26** | **w36** | **w48** | **w50** | **w60** | **w72** | **w74** | **w84** | **w96** | | | |
| *Study Day (window in days)* | *-14* | *1* | *15 (± 1)* | *85 (±4)* | *169 (±1)* | *14 days post w24 Visit (±1)* | *253 (±4)* | *337 (±1)* | *14 days post w48 Visit (±1)* | *421 (±4)* | *505 (±1)* | *14 days post w72 Visit (±1)* | *589 (±4)* | *673 (±1)* | | | |
| | | | | | | | | | | | | | | | | | |
| Pregnancy test [6] | x | x | x | x | x | x | x | x | x | x | x | x | x | x | x | | x |
| Antibody Titers [7] | | x | | x | x | | | x | | | x | | | x | | | x |
| RCR (non-DNA) [8] | | x | | x | x | | | x | | | x | | | x | | | x |
| RCR (DNA) [9] | | x | | | | | | | | | | | | | | | |
| Protein biomarker sampling [10] | | x | | x | x | | | x | | | x | | | x | | | x |
| HAHA [11] | | x | | | x | | | x | | | x | | | x | | | x |
| Plasma/ urine banking for JCV[12] | | x | | x | x | | x | x | | x | x | | x | x | | | x |
| PK Samples [13] | | x | | | x | | | x | | | x[13] | x[13] | x | x | | | x |
| Thyroid function tests [14] | x | | | | x | | | x | | | x | | | x | | | x |
| FSH [15] | x | | | | | | | | | | | | | | | | |
| Hepatitis Screening [16] | x | | | | | | | | | | | | | | | | |
| Hepatitis B virus DNA[16] | x | x | | (x) | (x) | | (x) | (x) | | (x) | (x) | | (x) | (x) | | | (x) |
| RPR | x | | | | | | | | | | | | | | | | |
| CD4 count | x | | | x | | | x | | | x | | | x | | | | |
| IgG | | | | x | | | x | | | x | | | x | | | | |
| Total Ig, IgA, IgG, IgM | x | | | | x | | | x | | | x | | | x | | | x |
| FACS [17] | | x | x | x | x | | | x | | | x | | | x | | | x |
| Routine safety lab [18] | x | x | x | x | x | | x | x | | x | x | | x | x | | | x |
| Type I interferon neutralizing antibody assay [19] | | x | | | x | | | x | | | x | | | x | | | x |

## Table 3. Schedule of Assessments: Screening Through the End of Double-Blind Treatment Period (Cont.)

| | Screen | Treatment Period | | | | | | | | | | | | | Delayed Dosing Visit [22] | Unscheduled Visit[23] | Withdrawal from Treatment Visit |
|---|---|---|---|---|---|---|---|---|---|---|---|---|---|---|---|---|---|
| Visit | 1 | 2 BL | 3 | 4 | 5 | 6 | 7 | 8 | 9 | 10 | 11 | 12 | 13 | 14 | | | |
| Week | -2 | - | w2 | w12 | w24 | w26 | w36 | w48 | w50 | w60 | w72 | w74 | w84 | w96 | | | |
| *Study Day (window in days)* | *-14* | *1* | *15 (± 1)* | *85 (±4)* | *169 (±1)* | *14 days post w24 Visit (±1)* | 253 (±4) | *337 (±1)* | *14 days post w48 Visit (±1)* | 421 (±4) | 505 (±1) | *14 days post w72 Visit (±1)* | 589 (±4) | 673 (±1) | | | |
| Pre-treatment with i.v. methylprednisolone [20] | | x | x | | x | x | | x | x | | x | x | | | x | | |
| Administration of i.v. ocrelizumab / ocrelizumab placebo [21] | | x | x | | x | x | | x | x | | x | x | | | x | | |
| Assessment of s.c. Rebif®/ Rebif® placebo compliance | | x | x | x | x | x | x | x | x | x | x | x | x | x | | x | x |
| Administration of s.c. Rebif® / Rebif® placebo 3x/wk | | x | x | x | x | x | x | x | x | x | x | x | x | | | | |

1. **Informed Consent** must be obtained in written form from all patients at screening (prior to any study–related procedure) in order to meet eligibility for the study.
2. **Vital signs** (i.e., pulse rate, systolic and diastolic blood pressure, respiration rate and temperature) will be obtained while the patient is in the semi supine position (after 5 minutes). On infusion visits, the vital signs should be taken within 45 minutes prior to the methylprednisolone infusion in all patients. In addition, vital signs should be obtained prior to ocrelizumab/ocrelizumab placebo infusion, then every 15 minutes (± 5 minutes) for the first hour; then every 30 minutes (± 10 minutes) until 1 hour after the end of the infusion. On non-infusion days, the vital signs may be taken at any time during the visit.
3. **ECG (pre- and post-dose):** on infusion visits ECG should be taken within 45 minutes prior to the methylprednisolone infusion in all patients, and within 60 minutes after completion of ocrelizumab/ocrelizumab placebo infusion. On non-infusion days, the ECG may be taken at any time during the visit.
4. **MRI**: brain MRI scans will be obtained in patients withdrawn from the treatment period (at a withdrawal visit) if not performed during last 4 weeks.

**Table 3. Schedule of Assessments: Screening Through the End of Double-Blind Treatment Period (Cont.)**

5. **A structured telephone interview** will be conducted by site personnel every 4 weeks (± 3 days) from Week 8 through the study to identify any new or worsening neurological symptoms that warrant an unscheduled visit and collect data on possible events of infections.
6. **Serum β-hCG** must be performed at screening in women of childbearing potential. Subsequently, urine β-hCG [sensitivity of at least 25 mIU/mL] will be performed. On infusion visits, the urine pregnancy test should be performed prior to methylprednisolone infusion in all women of child-bearing potential. If positive, the patient will not receive the scheduled dose and confirmation, a serum pregnancy test, will be performed.
7. **Antibody Titers**: measurement of antibody titers against common antigens (mumps, rubella, varicella and Streptococcus pneumoniae) will be performed.
8. **RCR - Roche Clinical Repository non-DNA (RNA – and protein):** for RNA 2x 2.5 ml whole blood samples to be taken from consenting patients only for expression profiling analysis. For protein: 6 ml blood samples in EDTA tube for plasma samples will be taken from consenting patients only for analysis of protein biomarkers. On infusion visits, ALL samples should be taken 5-30 minutes prior to methylprednisolone infusion.
9. **RCR - Roche Clinical Repository (DNA):** 6 ml whole blood sample to be taken from only from patients consenting to RCR for pharmacogenetic and genetic analysis. If not done at Baseline (Visit 2), sample may be collected at next visit.
10. **Protein biomarker sampling:** one serum sample (6 ml) will be taken from all patients for analysis of protein biomarkers. On infusion visits, samples should be taken 5-30 minutes prior to methylprednisolone infusion.
11. **HAHA**: On infusion visits, serum samples are collected 5-30 minutes prior to the methylprednisolone infusion.
12. **Plasma and urine samples for JCV will be collected** at specified time points and analyzed in batches.
13. **PK samples:** on the infusion days at week 72 and week 74, two serum samples should be collected, one 5-30 minutes prior to the methylprednisolone infusion and the second one 30 minutes (±10 minutes) following the completion of the ocrelizumab/ocrelizumab placebo infusion. For all other infusion visits, a blood sample should be taken 5 – 30 minutes before the methylprednisolone infusion. At other times (non-infusion visits) samples may be taken at any time during the visit.
14. **sTSH** will be tested at screening and during double-blind treatment period. Thyroid autoantibodies will be assayed only at screening.
15. **FSH**: <u>only applicable to women</u> to confirm the post-menopausal status.
16. **Hepatitis** screening & monitoring: all patients must have negative HBsAg result and negative HepCAb screening tests prior to enrollment. If total HBcAb is positive at screening, HB virus DNA measured by PCR must be negative to be eligible. For those patients enrolled with negative HBsAg and positive total HBcAb, HB virus DNA (PCR) must be repeated every 12 weeks during the treatment period.
17. **FACS**: including CD19 and other circulating B-cell subsets, T-cells, natural killer cells and other leukocytes. On infusion visits, blood samples should be collected prior to the infusion of methylprednisolone.

## Table 3. Schedule of Assessments: Screening Through the End of Double-Blind Treatment Period (Cont.)

18. **Routine safety lab:** hematology, chemistry and urinalysis: on infusion visits, all urine and blood samples should be collected prior to the infusion of methylprednisolone. At other times, samples may be taken at any time during the visit.
19. **Type I interferon neutralizing antibody assay:** samples should be taken at least 36 hours following last injection of Rebif®/Rebif® placebo.
20. All patients receive **prophylactic treatment** with 100 mg of methylprednisolone i.v. prior to infusion of ocrelizumab /ocrelizumab placebo. It is also recommended that patients receive an analgesic/antipyretic such as acetaminophen/paracetamol (1 g) and an i.v. or oral antihistamine such as diphenhydramine 50 mg 30-60 minutes prior to ocrelizumab/ ocrelizumab placebo.
21. **Administration (infusion) of i.v. ocrelizumab/ocrelizumab placebo**: the Treating Investigator must review the clinical and laboratory re-treatment criteria prior to subsequent infusion of ocrelizumab/ocrelizumab placebo.
22. **A delayed dosing visit** will be performed and recorded in the Delayed Dosing Visit eCRF form when dosing cannot be administered at the scheduled dosing visit. Other tests or assessments may be done as appropriate.
23. **Unscheduled Visit**: assessments performed at unscheduled (non-dosing) visits will depend on the clinical needs of the patient. All patients with new neurological symptoms suggestive of relapse should have EDSS performed by examining investigator. Other tests/assessments may be done as appropriate. Please note: in case of ALT elevations dose modification should be necessary, additional visits may be required for dispensing of study medication.

<u>Please note:</u> based on local Ethics Committees or National Competent Authority requirements, additional diagnostic testing may be required for selected patients or selected centers to exclude tuberculosis, Lyme disease, HTLV-1 associated myelopathy (HAM), acquired immune deficiency syndrome (AIDS), hereditary disorders, connective tissue disorders, or sarcoidosis. Other specific diagnostic tests may be requested when deemed necessary by the investigator.

**Table 4. Schedule of Assessments: Safety Follow up (including prolonged B-cell monitoring if required)**

| | **Safety Follow up** | ***Prolonged B-cell Monitoring*[1]** | ***End of observation or withdrawal from Safety Follow up*** |
|---|---|---|---|
| **Assessments** | *Visits every 12 weeks (±7 days)* [2] | *Visits every 24 weeks (±7 days)* | |
| Urine pregnancy test | x | x | x |
| Routine Safety Labs[3] | x | x | x |
| FACS [4] | x | x | x |
| Total Ig, IgA, IgG, IgM | x[10] | x | x |
| HAHA[5] | x[10] | x | x |
| Plasma/urine banking for JCV | x | x | x |
| Antibody titers | x[10] | x | x |
| Hepatitis B viral DNA[6] | (x) | (x) | (x) |
| RCR non-DNA [7] | x[10] | x | x |
| Protein biomarker sampling [8] | x[10] | x | x |
| Vital Signs | x | x | x |
| EDSS | x | | x |
| Neurological examination | x | x | x |
| Physical examination | x[10] | x | x |
| Potential relapses recorded | x | x | x |
| Adverse events | x | x | x |
| Concomitant Medication | x | x | x |
| Telephone interview [9] | x | x | |

**Table 4. Schedule of Assessments: Safety Follow up (including prolonged B-cell monitoring if required) (Cont.)**

1. **Prolonged B-cell monitoring:** patients whose B-cells have not been repleted after 48 weeks of Safety Follow up period will continue with visits every 24 weeks (± 7 days) until B-cell repletion.
2. Visits will be performed at 12-week intervals counting from the date of last infusion of ocrelizumab.
3. **Routine safety lab**: hematology, chemistry and urinalysis.
4. **FACS** including CD19 and other circulating B-cell subsets, T cells, natural killer cells and other leukocytes.
5. **HAHA**: two serum samples are required.
6. **Hepatitis monitoring:** hepatitis to be monitored only in patients with screening results of HbsAg negative, HBcAb positive and HBV DNA negative, inclusive.
7. **RCR (Roche Clinical Repository) non-DNA (RNA and protein):** for RNA 2x 2.5 ml whole blood samples to be taken from consenting patients only for expression profiling analysis. For protein 6 ml blood samples in EDTA tube for plasma samples to be taken from consenting patients only for analysis of protein biomarkers.
8. **Protein biomarker sampling**: 6 ml blood sample in a plain tube without EDTA for serum isolation will be taken from all patients for analysis of protein biomarkers.
9. **A structured telephone interview** will be performed by site personnel every 4 weeks (± 3 days) between visits until 48 weeks after the last infusion to identify any new or worsening neurological symptoms that warrant an unscheduled visit and collect data on possible events of infections. If prolonged B-cell monitoring is required beyond 48 weeks after the last infusion, telephone interviews will be done every 12 weeks (± 7 days) between visits.
10. Needs to be assessed only every 24 weeks.

Please note: patients in Safety Follow up who receive other B-cell targeted therapies will only be followed for 48 weeks from the date of the last infusion of the study drug regardless of their B-cell count.

## SAMPLE SIZE AND STATISTICAL ANALYSES

The sample size for this study has been estimated based on data from previous RRMS trials, with the use of two-sided tests with an experiment-wise alpha of 0.05. The annualized rate of relapse among patients receiving ocrelizumab at 96 weeks is predicted to be 0.165 (standard deviation of approximately 0.60), as compared with 0.33 (standard deviation of approximately 0.80) among patients receiving the control treatment, Rebif® (this represents a relative reduction of 50% on ocrelizumab compared to the active comparator). For the annualized relapse rate, a t-test has been used to determine the sample size between ocrelizumab and the control arm. The sample size of 400 patients per arm provides 84 percent power, maintaining the type I error rate of 0.05, and assuming a drop out rate of 20 percent approximately (assuming relative reduction among patients drop out is 25%). For sustained disease progression, a two group test of equal exponential survival with exponential dropout is used to determine the sample size. Assuming the 2 year sustained disability progression rate is 18% for the Rebif® arm and 12.6% for the ocrelizumab arm (this represents a relative reduction of 30% on ocrelizumab compared to the active comparator), and assuming a drop out rate of 20 percent over 2 years approximately, the sample size of 400 per arm will provide 80 percent power, maintaining the type I error rate of 0.05 based on the pooled analysis of two identical RMS trials (800 patients treated with ocrelizumab 600 mg and 800 patients treated with Rebif®).

All eligible patients will be randomized to treatment (with 1:1 ratio to ocrelizumab 600 mg regimen or the control arm) stratified by geographical region ( US versus ROW) and baseline EDSS (< 4.0 versus ≥ 4.0).

The primary efficacy endpoint is the annualized relapse rate by 96 weeks. The annualized relapse rates at 96 weeks will be calculated using Poisson regression, offsetting for exposure time in years, accounting for over-dispersion and adjusting for region and baseline EDSS. The adjusted annualized relapse rates and the 95% confidence intervals for the relapse rates will be presented along with the p-value.

Summaries of safety data will be produced using data from all patients who have received any study treatment and provided at least one assessment of safety.

## STATISTICAL ANALYSIS PLAN

| | |
|---|---|
| **TITLE:** | **A RANDOMIZED, DOUBLE-BLIND, DOUBLE-DUMMY, PARALLEL-GROUP STUDY TO EVALUATE THE EFFICACY AND SAFETY OF OCRELIZUMAB IN COMPARISON TO INTERFERON BETA-1A (REBIF®) IN PATIENTS WITH RELAPSING MULTIPLE SCLEROSIS** |
| **PROTOCOL NUMBERS:** | **WA21092 and WA21093** |
| **STUDY DRUG:** | **Ocrelizumab (RO4964913)** |
| **VERSION NUMBER:** | **4** |
| **IND NUMBER:** | **100,593** |
| **EUDRACT NUMBERS:** | **2010-020337-99 and 2010-020315-36** |
| **SPONSOR:** | F. Hoffmann-La Roche Ltd |
| **PLAN PREPARED BY:** | Gaelle Klingelschmitt |
| **DATE FINAL:** | 8 March 2011 |
| **DATES AMENDED:** | Version 2: 14 January 2014<br>Version 3: 3 September 2014<br>Version 4: See electronic date stamp below. |

### STATISTICAL ANALYSIS PLAN AMENDMENT APPROVAL

| **Name** | **Reason for Signing** | **Date and Time** (UTC) |
|---|---|---|
| Burger,Hans_Ulrich | Company Signatory | 14-May-2015 10:43:32 |


**CONFIDENTIAL**

This is an F. Hoffmann-La Roche Ltd document that contains confidential information. Nothing herein is to be disclosed without written consent from F. Hoffmann-La Roche Ltd.

## STATISTICAL ANALYSIS PLAN AMENDMENT RATIONALE

**January 2014**

Study WA21093 number for pooling was not included in the first version. Several exploratory and sensitivity analyses have been removed in order to focus on the analyses to be performed for the regulatory submission. The hierarchy of the secondary efficacy endpoints has been modified. Efficacy analyses in some subgroups have been added for EU regulatory purposes. The intent-to-treat population includes all patients randomized to adhere to the standard definition.

**August 2014**

This Statistical Analysis Plan (SAP) was amended to implement EMA Scientific Advice and to increase statistical rigor (for example, replacing ranked ANCOVA analysis with Mixed-Effect Model Repeated Measures analysis (MMRM) in relevant analyses). This SAP has also been updated to align with the amended study protocols. In both the protocols and this SAP, the secondary and exploratory efficacy endpoints have been extensively modified to reflect the latest clinical and scientific thinking in the field of MS, and also to increase rigor (for example, the proportion of relapse-free patients was changed from a secondary to an exploratory efficacy endpoint, since it is closely related to the primary efficacy endpoint, hence adds only limited value to our understanding of efficacy).

More details have been added to specify the calculation of the baseline Expanded Disability Status Scale (EDSS) value. The average value of the screening EDSS score and the value at the baseline visit used as reference for the analyses of increase or decrease EDSS score (i.e., the Confirmed Disability Progression, Confirmed Disability Improvement (CDI), and mean change of EDSS over time). However, for the stratification, the value is rounded up to the next EDSS score (e.g., 3.75 is rounded up to 4. and 3.25 is rounded up to 3.5), in order to present a valid score for the baseline characteristics and randomization scheme. The definition of the confirmed disability progression has been slightly modified to take into account the existence of a baseline EDSS of 5.75, with no impact to the identification of the progression. The definition of the Confirmed Disability Improvement has been added. In order to simplify the definition of the safety population and adhere to the standard, the safety population now includes all patients who received any study drug.

This Statistical Analysis Plan covers analyses performed at the level of each individual study. The scope of this statistical analysis plan includes:

- All data collected in the 96-week comparative treatment phase

- All data collected in the Open-Label Extension (OLE) phase up to the date of clinical cutoff applied for the analysis of the 96-week comparative treatment phase
- All data collected in the Safety Follow-Up (SFU) phase up to the date of clinical cutoff applied for the analysis of the 96-week comparative treatment phase

Note that substudies conducted at certain selected sites, such as the substudy collecting Optical Coherence Tomography (OCT) scans, are not within the scope of this statistical analysis plan. These analyses will be planned, executed, and reported separately.

Analyses performed upon data from both studies combined are described in a separate analysis plan (i.e., the “Pooled SAP”).

It is planned that this statistical analysis plan will be reviewed, to include the SAS code for primary and secondary analysis per FDA request, once more before the first of the two study database locks for the primary analysis, to ensure it remains fit for purpose and provides sufficient detail.

Protocol synopsis and schedule of assessment have been removed from the SAP and are available in the protocols.

Additional minor changes have been made to improve clarity and consistency.

**May 2015**

Updates made at this time include the following major aspects. The rationale for the updates is to provide full and complete information on these important aspects prior to database lock:

- Subgroup analyses for EU planning purposes have been removed; these are now covered in the pooled SAP.
- Detailed updates have been made to the safety analysis section.
- SAS code has been included for statistical models for primary and secondary analyses.
- Derivation of protocol-defined relapses has been added.
- Reasons for patient exclusion from per-protocol population has been added.
- Sensitivity analyses have been added to describe alternative analyses when negative binomial models do not fit appropriately
- Methods have been added to handle MSFC values outside of normal ranges

Additional minor changes have been made to improve clarity and consistency.

## TABLE OF CONTENTS


STATISTICAL ANALYSIS PLAN AMENDMENT RATIONALE ............................ 2

1. BACKGROUND ............................................................................. 9

2. WA21092 AND WA21093 STUDY DESIGN ............................................ 9

2.1 Protocol Synopsis ......................................................................... 13

2.2 Outcome Measures ........................................................................ 13

2.2.1 Primary Efficacy Outcome Measure ................................................ 13

2.2.2 Secondary Efficacy Outcome Measures ........................................... 13

2.2.3 Exploratory Efficacy Outcome Measures ........................................ 13

2.2.4 Pharmacokinetic Analysis: Objectives and Outcome Measures ........................................................................ 14

2.2.5 Pharmacodynamic Outcome Measure ............................................. 15

2.2.6 Safety Outcome Measures .............................................................. 15

2.3 Determination of Sample Size ......................................................... 15

2.4 Analysis Timing ............................................................................. 16

3. STUDY CONDUCT ............................................................................ 16

3.1 Randomization ................................................................................ 16

3.2 Independent Review Facility ............................................................. 16

3.3 Expanded Disability Status Scale Cleaning Process ......................................................................................... 17

3.4 Data Monitoring ............................................................................... 17

4. STATISTICAL METHODS .................................................................... 17

4.1 Analysis Populations ........................................................................ 17

4.1.1 Intent-to-Treat Population ............................................................. 17

4.1.2 Per-Protocol Population ................................................................. 17

4.1.3 Pharmacokinetic Evaluable Population ............................................ 19

4.1.4 Safety Population ........................................................................... 19

4.2 Analysis of Study Conduct ................................................................ 19

4.3 Analysis of Treatment Group Comparability ........................................ 20

4.3.1 Demography .................................................................................. 20

4.3.2 Baseline Disease Characteristics .................................................... 21

4.3.3 Multiple Sclerosis Disease History .................................................. 21

4.3.4 Non-MS Disease History ........ 22
4.3.5 Prior Treatments for Multiple Sclerosis ........ 22
4.3.6 Baseline Magnetic Resonance Imaging Data ........ 22
4.4 Efficacy Analysis ........ 22
4.4.1 Primary Efficacy Endpoint, Annualized Protocol-Defined Relapse Rate by 2 Years (96 weeks) ........ 23
4.4.2 Secondary Efficacy Endpoints ........ 27
4.4.2.1 Rationale for Hierarchy of Secondary Efficacy Endpoints ........ 27
4.4.2.2 Statistical Testing Strategy for Secondary Efficacy Endpoints ........ 28
4.4.2.3 Time to Onset of Confirmed Disability Progression for at Least 12 Weeks with the Initial Event of Neurological Worsening Occurring during the 96-Week, Double-Blind, Double-Dummy Treatment Period ........ 31
4.4.2.4 Total Number of T1 Gadolinium–Enhanced Lesions as Detected by Brain Magnetic Resonance Imaging at Weeks 24, 48, and 96 ........ 35
4.4.2.5 Total Number of New and/or Enlarging T2 Hyperintense Lesions as Detected by Brain Magnetic Resonance Imaging at Weeks 24, 48, and 96 ........ 36
4.4.2.6 Proportion of Patients Who Have Confirmed Disability Improvement Sustained for at Least 12 Weeks with the Initial Event of Neurological Improvement Occurring during the 96-Week, Double-Blind, Double-Dummy Treatment Period ........ 36
4.4.2.7 Time to Onset of Confirmed Disability Progression for at Least 24 Weeks with the Initial Event of Neurological Worsening Occurring during the 96-Week, Double-Blind, Double-Dummy Treatment Period ........ 37
4.4.2.8 Total Number of New T1-Hypo-Intense Lesions (Chronic Black Holes) at Weeks 24, 48, and 96 ........ 38
4.4.2.9 Change in Multiple Sclerosis Functional Composite Scale Score from Baseline to Week 96 ........ 38

4.4.2.10 Percentage Change in Brain Volume as Detected by Brain Magnetic Resonance Imaging Scan from Week 24 to Week 96 .... 41
4.4.2.11 Change in Quality of Life, as Measured by the Short Form 36 Version 2 Physical Component Summary Score from Baseline to Week 96 .... 42
4.4.2.12 Proportion of Patients Who Have No Evidence of Disease Activity by Week 96 .... 43
4.4.3 Exploratory Efficacy Endpoints .... 44
4.4.3.1 Proportion of Relapse-Free Patients by 96 Weeks .... 44
4.4.3.2 Percentage Change in Total T2 Lesion Volume as Detected by Brain Magnetic Resonance Imaging from Baseline to Week 96 .... 44
4.4.3.3 Annualized Relapse Rate, Based on All Clinical Relapses at the End of the 96-Week Comparative Treatment Period .... 45
4.4.3.4 Annualized Protocol-Defined Relapse Rate by 2 Years (96 weeks) for Relapses Requiring Intravenous Steroid Therapy .... 45
4.4.3.5 Annualized Protocol-Defined Relapse Rate by 2 Years (96 weeks) for Severe Relapses .... 45
4.4.3.6 Percentage Change in Brain Volume as Detected by Brain Magnetic Resonance Imaging from Baseline to Week 96 .... 45
4.4.3.7 Change in Timed 25-Foot Walk from Baseline to Week 96 .... 46
4.4.3.8 Change in 9-Hole Peg Test from Baseline to Week 96 .... 46
4.4.3.9 Change in Fatigue, as Measured by the Modified Fatigue Impact Scale Total Score from Baseline to Week 96 .... 47
4.4.3.10 Change in Patient-Reported Depressive Symptoms, as Measured by the Center for Epidemiologic Studies Depression Scale, from Baseline to Week 96 .... 47
4.4.3.11 Change in Karnofsky Performance Status Score from Baseline to Week 96 .... 48

4.4.3.12 Percentage Change in Cortical Grey Matter Volume as Detected by Brain MRI from Baseline to Week 96 ..... 49
4.4.3.13 Percentage Change in White Matter Volume as Detected by Brain MRI from Baseline to Week 96 ..... 49
4.4.3.14 Proportion of Patients Who Have Disability Improvement Sustained for At Least 24 Weeks with the Initial Event of Neurological Improvement Occurring during the 96-Week, Double-Blind, Double-Dummy Treatment Period ..... 50
4.4.3.15 Proportion of Patients Who have Disability Improvement Sustained for at Least 12 Weeks and Sustained until the End of the 96-Week, Double-Blind, Double-Dummy Treatment Period, with the Initial Event of Neurological Improvement Occurring during the 96-Week, Double-Blind, Double-Dummy Treatment Period ..... 50
4.4.3.16 Duration of Confirmed Disability Improvement ..... 50
4.4.3.17 Proportion of Patients Who at Week 96 Have Improved, Stable or Worsened Disability, Compared with Baseline ..... 50
4.4.3.18 Change in EDSS Score from Baseline to Week 96 ..... 51
4.4.3.19 Change in Quality of Life, as Measured by the Short Form 36 Version 2 Mental Component Summary Score from Baseline to Week 96 ..... 51
4.4.4 Subgroup Analyses ..... 51
4.4.4.1 Efficacy Data from the Safety Follow-Up Phase ..... 52
4.4.4.2 Efficacy Data from the Open-Label Extension Phase ..... 52
4.5 Pharmacokinetic and Pharmacodynamic Analyses ..... 52
4.5.1 Pharmacokinetic Analyses ..... 52
4.5.2 Pharmacodynamic Analyses ..... 52
4.6 Safety Analyses ..... 53
4.6.1 Exposure of Study Medication ..... 53
4.6.2 Columbia-Suicide Severity Rating Scale ..... 54
4.6.3 Adverse Events ..... 54
4.6.3.1 Selected Adverse Events ..... 56

4.6.4 Magnetic Resonance Imaging Data ..... 57
4.6.5 Laboratory Data ..... 57
4.6.6 Vital Signs ..... 58
4.6.7 Safety by Treatment and Period ..... 58
4.6.8 Type I Interferon-Neutralizing Antibody Data ..... 59
4.7 Missing Data ..... 59
4.8 Interim Analyses ..... 59
5. REFERENCES ..... 60

## LIST OF TABLES

Table 1 Overview of Dosing Regimen in the Double-Blind, Double-Dummy Treatment Period ..... 11
Table 2 Overview of Dosing Regimen in the OLE Phase Screening Period and the OLE Phase ..... 12
Table 3 Center for Epidemiologic Studies Depression Scale ..... 48

## LIST OF FIGURES

Figure 1 Hierarchical Order of Key Efficacy Endpoints ..... 31

## LIST OF APPENDICES

Appendix 1 Standard Operating Procedure “EDSS Assessment Check for the Roche Trials WA25046, WA21092, and WA21093” ..... 61

# 1. BACKGROUND

Studies WA21092 and WA21093 are two Phase III studies with identical study design. This statistical analysis plan (SAP) describes the analyses that will be performed for each study independently. The analyses will be conducted in exactly the same way for both studies; therefore, only one statistical analysis plan has been written to be applied to both studies. The analysis of some secondary efficacy endpoints will necessitate the pooling of data from both studies in order to have sufficient statistical power to detect relevant treatment differences. Analyses of pooled data are described separately in the "Pooled SAP."

Study WA21493 is an ongoing double-blind, double-dummy, parallel-group, Phase II study of the efficacy and safety of ocrelizumab compared with interferon beta-1a (intramuscular) IM (Avonex®) in patients with relapsing remitting multiple sclerosis (RRMS). The study was unblinded in November 2009 and has an open-label phase that is ongoing. After 24 weeks of treatment, both doses of ocrelizumab (600 mg and 2000 mg) demonstrated a strong effect with a highly statistically significant reduction versus placebo in signs of paraclinical disease activity as measured by the number of gadolinium (Gd)–enhancing brain lesions in magnetic resonance imaging (MRI), the primary endpoint of the study, at Weeks 12, 16, 20, and 24. Compared with placebo, this represents a relative reduction in the number of lesions of 89% and 96%, for the 600-mg and 2000-mg ocrelizumab groups respectively. In addition, a statistically significant reduction in the annualized relapse rate (ARR) was observed with both ocrelizumab doses compared with placebo. Moreover, both ocrelizumab doses were superior to the active comparator, Avonex, for both the MRI primary endpoint and the ARR.

Although Study WA21493 (which enrolled approximately 50 patients per arm) demonstrated significant treatment effects in both MRI and ARR endpoints at Week 24 versus Avonex, the sample size required to demonstrate a statistically significant difference in disability progression between ocrelizumab and an active comparator is estimated to be much greater (i.e., approximately 800 patients per arm). To demonstrate significant treatment benefit of ocrelizumab in disability progression, the Sponsor plans to pool data from two Phase III studies (WA21092 and WA21093), which both include approximately 400 patients per treatment arm (ocrelizumab or interferon beta-1a 44 μg subcutaneous [SC] [Rebif®]), have identical inclusion and exclusion criteria, and were implemented concurrently.

# 2. WA21092 AND WA21093 STUDY DESIGN

The primary objective of Studies WA21092 and WA21093 is to assess whether the efficacy of ocrelizumab 600 mg (given as dual infusions of 300 mg on Days 1 and 15 of the first 24-week Dose and as a single infusion of 600 mg on Day 1 of each 24-week Dose thereafter) intravenously every 24 weeks is superior to interferon beta-1a 44 μg

subcutaneous (SC) as measured by the annualized protocol-defined relapse rate (see Section 4.4.1 for the definition of protocol-defined relapse) by 2 years (96 weeks) in patients with relapsing forms of multiple sclerosis (RMS).

The studies consist of the following periods:

### Screening:

Consenting patients entered a screening period to be evaluated for eligibility. The screening period lasted approximately 2 weeks, but it may have been prolonged for up to 8 weeks for relevant clinical, administrative, or operational reasons.

### Treatment Period:

Double-blind, double-dummy, 96-week comparative treatment period

Eligible patients were randomized via an Interactive Voice and Web Response System (IxRS) into one of two treatment groups: ocrelizumab 600 mg regimen (Group A) or interferon beta-1a 44 μg SC (Group B).

Open-Label Extension Phase

Patients who complete the 96-week treatment period may become eligible for the open-label extension (OLE) phase of the study. The OLE phase will be ongoing until ocrelizumab is commercially available in the patient’s country or as per local regulation (not to exceed 4 years after the last patient completes the Week 96 visit) or the Sponsor’s decision to terminate the ocrelizumab program for multiple sclerosis (MS).

### Safety Follow-Up Period:

Patients who discontinue treatment for any reason during the following periods will be entered into the Safety Follow-Up Period:

- During or after completion of the 96-week, double-blind, double-dummy treatment period
- During the OLE Phase Screening Period
- During the OLE Phase
- Patients who choose not to enter the OLE Phase or are not eligible for the OLE Phase after completing the 96-week, double-blind, double-dummy treatment period

Table 1 and Table 2 give an overview of the study design and dosing regimen during the double-blind double-dummy treatment period and OLE, respectively.

**Table 1 Overview of Dosing Regimen in the Double-Blind, Double-Dummy Treatment Period**

| Study Medication | Double-Blind, Double-Dummy Treatment Period [a, b] | | | | |
|---|---|---|---|---|---|
| | 1st Dose [c] (Weeks 1–24) | | 2nd Dose [c] (Weeks 24–48) | 3rd Dose [c] (Weeks 48–72) | 4th Dose [c] (Weeks 72–96) |
| | Day 1 | Day 15 | Week 24 | Week 48 | Week 72 |
| A: Ocrelizumab 600 mg regimen | 300 mg IV | 300 mg IV | 600 mg IV | 600 mg IV | 600 mg IV |
| B: Interferon beta-1a 44 µg SC regimen [d] | Interferon beta-1a 44 µg SC 3 times per week | → | → | → | → |

IV = intravenous; SC = subcutaneous.

Note: 100 mg of methylprednisolone IV will be administered in both treatment arms prior to each infusion of ocrelizumab/ocrelizumab placebo.

[a] The double-blind, double-dummy, treatment period consists of 96 weeks of treatment (four Doses).

[b] The first Dose consists of two 300-mg ocrelizumab IV infusions separated by 14 days. Doses 2–4 consist of a single IV infusion of 600-mg ocrelizumab.

[c] Prior to each infusion, a clinical evaluation will be performed to ensure that the patient remains eligible for treatment.

[d] Refer to the protocol for detailed interferon beta-1a 44 µg SC dosing regimen.

**Table 2 Overview of Dosing Regimen in the OLE Phase Screening Period and the OLE Phase**

| Study Medication | OLE Phase Screening Period | OLE Phase [a] | | | | |
|---|---|---|---|---|---|---|
| | | 5th Dose [b, c,d] | | 6th Dose [b, c, d] | 7th Dose [b, c, d] | Nth Dose [b, c, d] |
| | | Day 1 Infusion | Day 15 Infusion | | | |
| Ocrelizumab 600 mg regimen | NA [e] | 300 mg IV | 300 mg IV | 600 mg IV | 600 mg IV | 600 mg IV |
| Interferon beta-1a 44 μg SC regimen | Interferon beta-1a 44 μg SC 3 times per week[f] | NA [g] | NA [g] | NA [g] | NA [g] | NA [g] |

IV = intravenous; N = "nth" Dose; NA = not applicable; OLE = open-label extension; SC = subcutaneous.

Note: 100 mg of methylprednisolone IV will be administered prior to each infusion of ocrelizumab.

To keep the blinding, all patients will receive the first dose of ocrelizumab in the open-label phase in a split form, even if they had been treated with ocrelizumab in the previous blinded phase

[a] The OLE Phase can terminate at any moment.

[b] The assessments requested for N represent the typical schedule of assessments.

[c] Prior to each infusion, a clinical evaluation will be performed to ensure that the patient remains eligible for treatment.

[d] The first Dose of the OLE Phase consists of two 300-mg ocrelizumab IV Infusions that are to be separated by 14 days. Dose 6 onward consists of a single IV infusion of 600 mg ocrelizumab.

[e] During the OLE Phase Screening Period there will be no administration of ocrelizumab.

[f] Refer to the protocol for detailed interferon beta-1a 44 μg SC dosing regimen.

[g] During the OLE, there will be no administration of interferon beta-1a 44 μg SC; patients previously assigned to interferon beta-1a 44 μg SC receive ocrelizumab.

## 2.1 PROTOCOL SYNOPSIS

The protocol synopsis and the Schedule of Assessments are included in the protocols.

## 2.2 OUTCOME MEASURES

### 2.2.1 Primary Efficacy Outcome Measure

The primary efficacy endpoint is the annualized protocol-defined relapse rate at 2 years (96 weeks).

### 2.2.2 Secondary Efficacy Outcome Measures

The secondary efficacy endpoints are as follows:

- The time to onset of confirmed disability progression (CDP) for at least 12 weeks, with the initial event of neurological worsening occurring during the 96-week, double-blind, double-dummy treatment period
- The total number of T1 Gd-enhancing lesions as detected by brain MRI at Weeks 24, 48, and 96
- The total number of new and/or enlarging T2 hyperintense lesions as detected by brain MRI at Weeks 24, 48, and 96
- The proportion of patients who have confirmed disability improvement for at least 12 weeks, with the initial event of neurological improvement occurring during the 96-week, double-blind, double-dummy treatment period
- The time to onset of confirmed disability progression for at least 24 weeks, with the initial event of neurological worsening occurring during the 96-week, double-blind, double-dummy treatment period
- The total number of new T1–hypo-intense lesions (chronic black holes) at Weeks 24, 48, and 96
- The change in Multiple Sclerosis Functional Composite Scale (MSFCS) score from baseline to Week 96
- The percentage change in brain volume as detected by brain MRI from Week 24 to Week 96
- The change in SF-36 Physical Component Summary (PCS) Score from baseline to Week 96
- The proportion of patients who have no evidence of disease activity (NEDA) by Week 96

### 2.2.3 Exploratory Efficacy Outcome Measures

The exploratory efficacy endpoints are as follows:

- The proportion of relapse-free patients by Week 96
- The percentage change in total T2 hyperintense lesion volume as detected by brain MRI from baseline to Week 96

- The ARR, based on all clinical relapses at the end of the 96-week comparative treatment period (protocol-defined relapses are a subset of all clinical relapses)
- The ARR of relapses requiring IV steroids therapy
- The ARR of severe relapses
- The percentage change in brain volume as detected by brain MRI from baseline to Week 96
- The change in timed 25-foot walk from baseline to Week 96
- The change in 9-hole peg test from baseline to Week 96
- The change in fatigue, as measured by the Modified Fatigue Impact Scale (MFIS) total score from baseline to Week 96
- The change from baseline in patient-reported depressive symptoms, as measured by the Center for Epidemiologic Studies Depression Scale (CES-D), from baseline to Week 96
- The change in Karnofsky Performance Status Scale from baseline to Week 96
- The percentage change in cortical gray matter volume from baseline to Week 96
- The percentage change in white matter volume from baseline to Week 96
- The proportion of patients who have confirmed disability improvement for at least 24 weeks, with the initial event of neurological improvement occurring during the 96-week, double-blind, double-dummy treatment period
- The proportion of patients who have disability improvement sustained for at least 12 weeks and sustained until the end of the 96-week, double-blind, double-dummy treatment period, with the initial event of neurological improvement occurring during the 96-week, double-blind, double-dummy treatment period
- The duration of the confirmed disability improvement
- The proportion of patients who at Week 96 have improved, stable, or worsened disability, compared to baseline
- The change in Expanded Disability Status Scale (EDSS) Score from baseline to Week 96
- The change in Quality of Life, as measured by the Short Form 36 Version 2 (SF-36 v2) Mental Component Summary Score from baseline to Week 96

### 2.2.4 Pharmacokinetic Analysis: Objectives and Outcome Measures

The pharmacokinetic (PK) endpoints are as follows:

- Develop a population PK model to describe the pharmacokinetics of ocrelizumab in patients with MS and to estimate inter- and intrapatient variability
- Determine individual post hoc estimates to derive PK exposure measures
- Explore and quantify the potential influence of covariates contributing to the interpatient variability in PK parameters

- Explore the relationship between ocrelizumab exposure and selected efficacy endpoints (e.g., annualized relapse rate)
- Explore the relationship between ocrelizumab exposure and appropriate safety parameters

### 2.2.5 Pharmacodynamic Outcome Measure

The pharmacodynamics (PD) endpoint is the CD19 count, as a PD marker.

### 2.2.6 Safety Outcome Measures

Safety will be assessed through regular neurological and physical examinations, vital signs, ECGs, and the occurrence of adverse events. In addition, the following will be examined:

- Non-MS pathology at all available MRI scans
- Columbia-Suicide Severity Rating Scale (C-SSRS)
- Standard hematology, chemistry, and urinalysis assessments
- Circulating B-cell total and subsets, T cells, natural killer cells, and other leukocytes
- Plasma immunoglobulins
- Anti-drug antibodies (ADAs), also called human anti-human antibodies (HAHAs)
- Antibody titers for mumps, rubella, varicella, and Streptococcus pneumonia
- MS relapses classified as serious

## 2.3 DETERMINATION OF SAMPLE SIZE

The sample size for this study was estimated on the basis of data from previous RRMS trials, with use of two-sided tests with an experiment-wise alpha of 0.05. The ARR at 96 weeks in patients receiving ocrelizumab is predicted to be 0.165 (SD of approximately 0.60), compared with 0.33 (SD of approximately 0.80) in patients receiving the control treatment (interferon beta-1a 44 μg SC); this represents a relative reduction of approximately 50% with ocrelizumab treatment compared with the active comparator. For the ARR, a t-test was used to determine the sample size between ocrelizumab and interferon beta-1a 44 μg SC. The sample size of 400 patients per treatment group provides 84% power, maintaining the type I error rate of 0.05 and with the assumption of a dropout rate of approximately 20% (with the assumption that relative reduction among patient dropout is 25%).

For confirmed disability progression, a two group test of equal exponential survival with exponential dropout was used to determine the sample size. Assuming the 2-year confirmed disability progression rate is 18% for the interferon beta-1a 44 μg SC arm and 12.6% for the ocrelizumab arm (this represents a relative reduction of 30% on ocrelizumab compared to the active comparator), and assuming a dropout rate of 20% over 2 years approximately, the sample size of 400 per arm will provide 80% power, maintaining the type I error rate of 0.05 based on the pooled analysis of two RMS

studies (800 patients treated with ocrelizumab 600 mg and 800 patients treated with interferon beta-1a 44 μg SC).

### 2.4 ANALYSIS TIMING

After the 96-week visit of the last randomized patient, approximately 12 weeks may be needed to allow the confirmation of the latest event of the 12-week confirmed disability progression. Therefore, the clinical cut-off date will be approximately 12 weeks after the last patient's 96-week visit when the status is clarified for each patient. Database lock and unblinding of the Sponsor will occur several weeks after the clinical cut-off to clarify all outstanding queries.

The sites and EDSS raters will remain blind until approximately 24 weeks after the 96-week visit of the last patient randomized, to allow the confirmation of the last 24-week confirmed disability progression, in case an updated analysis of this endpoint is requested at a later point.

## 3. STUDY CONDUCT

### 3.1 RANDOMIZATION

Patients were randomized into two groups (Group A or Group B) in a 1:1 ratio. An independent IxRS provider conducted randomization (with use of blocked randomization with a block size of 4) and holds the treatment assignment code. Patients were stratified by geographical region (United States vs. rest of world [ROW]) and baseline EDSS score ($<$4.0 and $\geq$4.0). (Note: If both screening EDSS and randomization EDSS scores were collected, the average of both values will be considered as baseline EDSS score for all analyses and for the stratification factor, unless otherwise specified.)

The patient randomization list was generated by the IxRS with use of a pre-defined randomization specification. The randomization list will not be available to the study center, study monitors, project statisticians, or the Sponsor's project team. Unblinding of individual patients should not occur except in the case of emergency situations. Any request from the investigator for information about the treatment administered to study patients for another purpose must be discussed with the Sponsor. Unblinding will be performed by means of the IxRS. In accordance with regulatory reporting requirements, the Sponsor will unblind the identity of the study medication for all unexpected serious adverse events that are considered by the investigator to be related to study drug per safety reference document(s), (e.g., Investigator's Brochure, Core Data Sheet, and Summary of Product Characteristics). Details of patients who are unblinded during the study will be included in the Clinical Study Report.

### 3.2 INDEPENDENT REVIEW FACILITY

MRI scans will be read by a centralized reading center. The centralized reading center is blinded to the treatment assignment, and the reading is performed in the absence of clinical information. Further details on scanning acquisition sequences; methods,

handling, and transmission of the scans; certification of site MRI radiologist/technicians; and the procedures for the blinded analysis of the scans at the central reading center are described in a separate MRI technical manual.

### 3.3 EXPANDED DISABILITY STATUS SCALE CLEANING PROCESS

EDSS assessments are performed by a qualified examining investigator (a person other than the treating investigator), and the results are entered into an electronic device, and transferred to a central database. All EDSS results will then be checked in accordance with the Standard Operating Procedure (SOP) entitled “EDSS Assessment Check for the Roche Trials WA25046, WA21092, and WA21093” (see Appendix 1).

### 3.4 DATA MONITORING

An independent Data Monitoring Committee (iDMC) is reviewing cumulative data from the studies at approximately 4-month intervals until the primary analysis is completed. After all patients have completed the 96-week comparative treatment period, the iDMC will review cumulative data less frequently, but it is anticipated at least once per year. The iDMC is reviewing both efficacy and safety data; however, the studies will not be stopped for efficacy reasons.

## 4. STATISTICAL METHODS

### 4.1 ANALYSIS POPULATIONS

One patient population will be defined for the purpose of the safety analysis, and two patient populations will be defined for the efficacy analysis. All efficacy analyses will be performed using the intent-to-treat (ITT) population. The per-protocol population will be used for the primary analysis of the primary endpoint (ARR at 96 weeks) and the primary analysis of the first secondary endpoint (time to onset of confirmed disability progression at a minimum of 12 weeks with the initial event of neurological worsening occurring during the 96-week, double-blind, double-dummy treatment period) in order to evaluate the influence of major protocol violators and as a sensitivity check to the ITT analysis.

#### 4.1.1 Intent-to-Treat Population

All randomized patients will be included in the ITT population. Patients who prematurely withdrew from the study for any reason and for whom an assessment was not performed for whatever reason will still be included in the ITT analysis. Patients who received an incorrect therapy from that which was intended will be summarized according to their randomized treatment.

#### 4.1.2 Per-Protocol Population

The per-protocol population will include all patients in the ITT population who adhere to the protocol. Patients may be excluded if they significantly violated the inclusion or exclusion criteria or deviated from the study plan. Specific reasons for warranting exclusion will be agreed to and documented in the Data Analysis Plan on the basis of

the final version of the protocol prior to unblinding of the treatment groups. Only those patients with violations that are deemed to potentially affect the efficacy of study treatment will be excluded from the per-protocol population. Patients who received an incorrect therapy from that which was intended will be excluded from the per-protocol population.

The following patients will also be excluded from the per-protocol population:

- Diagnosis of primary progressive MS
- Disease duration from the onset of MS symptoms of more than 10 years in patients with an EDSS ≤2.0 at screening
- Known presence of other neurological disorders that may mimic MS
- Pregnancy or lactation
- Any concomitant disease that may require chronic treatment with systemic corticosteroids or immunosuppressants during the course of the study
- History or currently active primary or secondary immunodeficiency
- Treatment with any investigational agent within 24 weeks of screening or five half-lives of the investigational drug (whichever is longer); or treatment with any experimental procedures for MS
- Contraindication to interferon beta-1a 44 μg SC or incompatibility with interferon beta-1a 44 μg SC use
- Previous treatment with B-cell targeted therapies
- Any previous treatment with alemtuzumab (Campath), anti-CD4, cladribine, mitoxantrone, daclizumab, teriflunomide, laquinimod, total body irradiation, or bone marrow transplantation
- Treatment with cyclophosphamide, azathioprine, mycophenolate mofetil (MMF), cyclosporine, MTX, or natalizumab within 24 months prior to screening
- Treatment with fingolimod (FTY720, Gilenya®) or other S1P receptor modulator (i.e., BAF312) or with BG12, within 24 weeks prior to screening
- Treatment with IV immunoglobulin within 12 weeks prior to baseline
- No diagnosis of relapsing MS, in accordance with the revised McDonald criteria (2010)

- Less than two documented clinical attacks within the last 2 years prior to screening and no clinical attack in the year prior to screening; or at least one clinical attack within 30 days prior to screening

- Neurological status instability within 30 days prior to both screening and baseline.

- Received no dose of ocrelizumab / ocrelizumab placebo and interferon beta-1a 44 μg SC / interferon beta-1a 44 μg SC placebo

- Received ocrelizumab + interferon beta-1a 44 μg SC placebo / ocrelizumab placebo + interferon beta-1a 44 μg SC verum but was not randomized

- Received ocrelizumab + interferon beta-1a 44 μg SC placebo / ocrelizumab placebo + interferon beta-1a 44 μg SC verum other than the group to which the patient was randomized at any point during the study

- Received study medication that has been mishandled (e.g., incorrect storage temperature) and was not approved subsequently for use

#### 4.1.3 Pharmacokinetic Evaluable Population

The PK population will include all patients in the ocrelizumab treatment arm who had at least one measurable concentration value.

#### 4.1.4 Safety Population

The Safety Population will include all patients who received any study drug. Randomized patients who receive an incorrect therapy from that which was intended will be summarized in the group according to the therapy actually received. Patients who are not randomized but who receive study drug will be included in the safety population and summarized according to the therapy actually received. Patients who received more than one study therapy will be summarized in the ocrelizumab group. Patients who received only ocrelizumab placebo but not interferon beta-1a 44 μg SC will be included in the interferon beta-1a44 μg SC–arm group as a conservative approach.

### 4.2 ANALYSIS OF STUDY CONDUCT

All data up to the point of the clinical cutoff date for each study will be included to evaluate study conduct. This will include complete data from the 96-week, double-blind, double-dummy treatment period and all available data from the OLE and SFU phases at the time of cutoff for the primary analysis. Data from Study WA21092 that relate to the period after the Study WA21092 clinical cutoff date but before the Study WA21093 clinical cutoff date will not be included in the Study WA21092 database that will be used in the primary analysis.

The following analyses will be performed to evaluate the study conduct:

- Summary of protocol violations
- Summaries of ITT, per-protocol-defined, and safety populations, including numbers of patients in each population, and reasons for exclusion from the per-protocol population
- Summary of patient disposition, including the number of Doses received, the number of patients entering into the SFU phase, the number of patients entering into the OLE phase, and the number of patients entering B-cell monitoring
- Summary and Kaplan-Meier plots of the following:

    Time to discontinuation of study treatment during the 96-week, double-blind, double-dummy treatment period

    Time to discontinuation from the study (available data from all study phases to be included)

## 4.3 ANALYSIS OF TREATMENT GROUP COMPARABILITY

For continuous variables, the mean, median, SD, minimum, and maximum will be calculated. For categorical variables, number and percentage in each category will be displayed. The units/categories to be used are indicated within the brackets and separated by commas.

Except where stated, all assessments of treatment group comparability will utilize the date of the baseline visit (not the date of the screening visit) as the reference point in time.

Summaries will be presented for ITT and Safety Populations.

### 4.3.1 Demography

- Age (years): summary statistics calculated will include mean, median, SD, minimum, and maximum, percentage, and number in each category (18–39, ≥40, also age categories <18, 18–65, >65, used in the Development Safety Update Report [DSUR]) will be displayed (age at randomization will be used)
- Sex: the number and percentage of male and female patients
- Race: the number and percentage of White; Black or African American; Asian (Indian Subcontinent, Other than Indian subcontinent), American Indian or Alaskan native; Native Hawaiian or other Pacific Islander; and Other
- Stratification Factor: (Geographical region) the number and percentage of United States and Rest of World patients

    Sub-region: the number and percentage of patients from European Union/Switzerland/Norway, Latin America, Non-EU/Israel/Africa, and USA/Canada/Australia

- Ethnicity: the number and percentage of Hispanic or Latino and not Hispanic or Latino

- Weight (kg): summary statistics as described for age
- Body Mass Index (BMI): summary statistics as described for age

### 4.3.2 Baseline Disease Characteristics

- Stratification Factor (Baseline EDSS): the number and percentage of patients in each EDSS category ($<4.0$, and $\geq 4.0$); EDSS mean, median, SD, minimum, and maximum will be summarized. The baseline EDSS is defined as the average EDSS score from the screening and baseline visit EDSS scores (only values recorded up to and including the date of randomization will be used). For the analysis of mean change of EDSS, disability progression, and disability improvement, the exact value of the average EDSS score will be used. However, for the stratification factor, and for the randomization algorithm, the average will be rounded up to the next EDSS score, in order to assign a valid score (e.g., round 3.75 to 4.0, round 5.25 to 5.5) If one of the EDSS scores from the screening or baseline visit is missing, the other will be used for baseline EDSS. If EDSS score is missing at both the screening and baseline visit, the patient should not be randomized. Unless otherwise specified, this definition of baseline EDSS is used throughout.
- Number of relapses in the past year: the number and percentage of patients in each category (0, 1, 2, 3, and $\geq 4$); a relapse is considered to happen in the past year if: date of randomization $-$ date of relapse is $\leq 365$ days
- Number of relapses in the past 2 years: the number and percentage of patients in each category (0, 1, 2, 3, and $\geq 4$); a relapse is considered to happen in the past 2 years if: date of randomization $-$ the date of relapse is $\leq (365 \times 2)$ days
- Time since last onset of MS relapse prior to randomization (year): the number and percentage of patients in each category ($>6$ months prior to randomization, within the last 6 months from randomization); summary statistics calculated will include mean, median, SD, minimum, and maximum (calculated in years, i.e., divided by 365.25). Time is calculated as: date of randomization $-$ the date of last relapse
- Baseline Functional Systems Scores (FSSs) for each category (pyramidal, cerebellar, brainstem, sensory, bowel and bladder, visual, cerebral [or mental]; rated 0–5 or 0–6 depending on the domain of FSS) and for ambulation (categorical; rated 0–12)
- Baseline MSFCS score, raw results for each component: summary statistics calculated will include mean, median, SD, minimum, and maximum

### 4.3.3 Multiple Sclerosis Disease History

- Duration since MS symptom onset (years): summary statistics calculated will include mean, median, SD, minimum, and maximum (calculated in years; i.e., divide by 365.25)
- Duration since MS diagnosis (years): summary statistics calculated will include mean, median, SD, minimum, and maximum (calculated in years; i.e., divide by 365.25)

- Duration since MS symptom/diagnosis onset will be calculated up to the randomization date. If the month of symptom/diagnosis onset date is missing, the month of January will be used. If the day of symptom/diagnosis onset date is missing, the first ($1^{st}$) of the month will be used

### 4.3.4 Non-MS Disease History

- Did patient have a history of any non-MS diseases?: number and percentage of patients, (yes or no)
- Each non-MS disease: number and percentage of patients with a history of each disease

### 4.3.5 Prior Treatments for Multiple Sclerosis

- Did patient receive any prior treatment for MS?: number and percentage of patients, (yes or no)
- Did patient receive any prior treatment for MS with any interferon or glatiramer acetate?: number and percentage of patients, (yes or no)
- Each prior treatment for MS: number and percentage of patients receiving each treatment

### 4.3.6 Baseline Magnetic Resonance Imaging Data

- Number of Gd-enhancing T1 lesions at baseline: the number and percentage of patients in each category (0, 1, 2, 3, and $\geq 4$); summary statistics calculated will include mean, median, SD, minimum, and maximum
- Volume of T2 lesions at baseline: summary statistics calculated will include mean, median, SD, minimum, and maximum
- Number of T2 lesions at baseline: summary statistics calculated will include mean, median, SD, minimum, and maximum. Also the number and percentage of patients in each category (0–5, 6–9, and $>9$) will be presented.
- Normalized brain volume at baseline: summary statistics calculated will include mean, median, SD, minimum, and maximum
- Number of T1 hypo intense lesion (black holes) count at baseline: summary statistics calculated will include mean, median, SD, minimum, and maximum

Past MRI Data (recorded at Screening):

- Number of previous Gd-enhancing T1 lesions: the number and percentage of patients in each category (i.e., 0, 1, $>1$, and not evaluable)
- Number of previous T2 lesions: the number and percentage of patients in each category (0–5, 6–9, and $>9$)

## 4.4 EFFICACY ANALYSIS

All statistical hypotheses for the primary and secondary endpoints and treatment comparisons will be tested at the 5% significance level ($\alpha = 0.05$) against two-sided alternatives.

For all assessments, the baseline value will be used as the last non-missing value on or before the date of the first infusion of study medication. See Section 4.3.2 for baseline EDSS and Section 4.4.2.4 for time-to-confirmed sustained disability progression.

All primary and secondary efficacy endpoints will be analyzed stratified by geographical region (United States vs. ROW) and baseline EDSS ($<$4.0 vs. $\geq$4.0).

A pooled analysis of efficacy of the two studies, WA21092 and WA21093, is described in a separate pooled SAP.

### 4.4.1 Primary Efficacy Endpoint, Annualized Protocol-Defined Relapse Rate by 2 Years (96 weeks)

#### Significance Level

The null hypothesis will be tested at the $\alpha = 0.05$ level (two-sided test).

- $H_0$ (null hypothesis): there is no statistically significant difference between the ocrelizumab group and interferon beta-1a 44 μg SC group in the annualized protocol-defined relapse rate at 2 years.
- $H_1$ (alternative hypothesis): there is a statistically significant difference between the ocrelizumab group and interferon beta-1a 44 μg SC group in the annualized protocol-defined relapse rate at 2 years.

Annualized protocol-defined relapse rate at 2 years between the ocrelizumab dose groups and interferon beta-1a 44 μg SC group will be compared using the negative binomial model, adjusting for geographical region (United States vs. ROW) and baseline EDSS score ($<$4.0 vs. $\geq$4.0). If the test result for comparing the 600-mg ocrelizumab group and the interferon beta-1a 44 μg SC group is statistically significant at $\alpha < 0.05$ level (two-sided test), it will be concluded that the 600-mg ocrelizumab group demonstrated a superior effect of reducing the annualized protocol-defined relapse rate when compared with the interferon beta-1a 44 μg SC group.

#### Protocol-Defined Relapse

A protocol-defined relapse is defined as the occurrence of new or worsening neurological symptoms attributable to MS and immediately preceded by a relatively stable or improving neurological state of least 30 days. Symptoms must persist for $>$24 hours and should not be attributable to confounding clinical factors (e.g., fever, infection, injury, or adverse reactions to concomitant medications). The new or worsening neurological symptoms must be accompanied by objective neurological worsening consistent with an increase of at least half a step on the EDSS score, or 2 points on one of the appropriate FSS, or 1 point on two or more of the appropriate FSS. The change must affect the selected FSS (i.e., Pyramidal, Gait, Cerebellar, Brainstem, Sensory, or Visual). Episodic spasms, sexual dysfunction, fatigue, mood change, or bladder or bowel urgency or incontinence will not suffice to establish the diagnosis of a relapse.

Information related to a protocol-defined relapse will be captured on a clinical relapse event case report form (CRF) page. If the following criteria are satisfied, the clinical relapse will qualify as a protocol-defined relapse:

- Check-box of "Did symptoms persist for > 24 hours and were not being attributable…" on the Clinical MS relapse event is checked.
- EDSS scores from a visit occurring on or after the onset date of relapse needs to be at least half a step from the previous EDSS score; OR for FSS domains involved in the relapse event (indicated on the Clinical MS Relapse page), an increase of at least 2 points on one appropriate FSS domain or at least 1 point on two or more appropriate FSS domains.
- There is no protocol-defined relapse within 30 days before the start date of the clinical relapse.

Protocol defined relapse will be derived following the steps below:

1. Clinical relapse is reported on eCRF.
2. "Did symptoms persist for > 24 hours and were not being attributable…" is checked 'Yes' on the Clinical MS relapse event form.
3. Check if the first EDSS assessment at a visit (unscheduled or scheduled) on or after the onset date of the relapse is increased by ≥ 0.5 steps from the previous EDSS; OR SELECTED FSS domains relevant to the relapse event (pyramidal, ambulation, cerebellar, brainstem, sensory, or converted visual) are increased by ≥ 2 points on one domain or ≥ 1 point on two or more domains. When deriving this step do the following:
    - Take the last EDSS/FSS score before each clinical relapse onset date
    - Take the first EDSS/FSS score on or after each clinical relapse onset date
    - Calculate the difference between the two scores
    - Select clinical relapses where there is an increase of ≥ 0.5 in EDSS OR ≥ 2 on one appropriate FSS domain OR ≥ 1 on two or more appropriate FSS domains
4. For each relapse that satisfies the 3 criteria above, check if the following relapses are within 30 days (i.e., the onset dates are ≤ 30 days apart). If they are within 30 days, then the later relapses are not protocol-defined relapses.

### Analysis Methods

The ITT Population analysis will be presented.

The total number of protocol-defined relapses for each patient will be counted. The exposure time will be calculated as follows:

Exposure Time = (Early treatment discontinued date or date of Week 96 visit)–date of study Day 1 + 1)/365.25

All available data during the 96-week treatment period will be used for the analysis. For patients who discontinue the treatment early, only data that are collected before the early treatment discontinuation will be used in this analysis (with the exception of subsequent data used to confirm disability progression post-discontinuation of study treatment).

The annualized protocol-defined relapse rate at Week 96 will be analyzed using a negative binomial model, fitted in SAS using the GENMOD procedure. The model will include, for each patient, the total number of protocol-defined relapses with onset between randomization date and early treatment discontinued date/date of Week 96 as response variable and treatment group, baseline EDSS score ($<4.0$ vs. $\geq 4.0$), and geographical region (United States vs. ROW) as covariates. In order to account for different study treatment exposure durations among patients, log-transformed exposure time will be included in the model as an “offset” variable for appropriate computation of relapse rate. The rate ratio and the associated two-sided 95% confidence interval will be provided to compare the ocrelizumab and interferon beta-1a 44 μg SC groups. The estimated relapse rate and its two-sided 95% confidence intervals will be provided for each treatment group.

Sample SAS code can be found below (SAS code is regarded as “draft” until fully validated at the analysis stage):

```
proc genmod data=ARR;
   class ARMCD REGION BEDSSCAT;
   model N_PDR = ARMCD REGION BEDSSCAT / offset=EXPLOG
   link=log dist=negbin type3;
   lsmeans ARMCD / exp cl;
   ods output lsmeans=lsm;
   ods output ParameterEstimates=est;
run;
```

The unadjusted annualized protocol-defined relapse rate at Week 96 will also be presented for each treatment group. This is defined for each treatment group as the total number of protocol-defined relapses for all patients in the treatment group, divided by the total number of patient-years of exposure to that study treatment.

### Sensitivity and Robustness Checks

The primary analysis described above will be repeated for the per-protocol population as a sensitivity analysis.

The primary analysis described above will be repeated for the ITT population but will exclude patients who received no study medication, as a sensitivity analysis.

As a sensitivity analysis, the primary analysis negative binomial model described above will be adjusted by the following additional covariates: number of relapses occurring within the 2 years prior to study entry, baseline presence of Gd lesions (present or absent), prior MS treatment, and age ($<40$, $\geq 40$).

A Poisson model with the same covariates as the primary analysis negative binomial model will be fitted.

Three further sensitivity analyses will be undertaken:

- The annualized protocol-defined relapse rate at Week 96 will be calculated including all protocol-defined relapses occurring during the 96-week, double-blind, double-dummy phase or the SFU Phase, up to 96 weeks after randomization. In this analysis, the exposure time will be calculated as follows:

  Exposure Time = ((Date of Week 96 visit or date of last visit up to 96 weeks after randomization) − date of study Day 1 + 1)/365.25

- A sensitivity analysis for the annualized protocol-defined relapse rate at Week 96 using multiple imputations will be performed to explore the potential influence of informative dropouts on the results of the primary efficacy analyses. The multiple imputations will be used to impute the events for patients who discontinued treatment early during the 96-week, double-blind, double-dummy phase without any protocol-defined relapse during the 30 days prior to discontinuation of study treatment. Multiple imputation inference involves three distinct phases:

  5. The missing data are filled in "m" times to generate "m" complete data sets. Instead of filling in a single value for each missing value, multiple imputation replaces each missing value with a set of "m" plausible values that represent the uncertainty about the right value to impute.
  6. The "m" complete data sets are analyzed using standard statistical analyses.
  7. The results from the "m" complete data sets are combined to produce inferential results.

  In this analysis, 50% of the patients who discontinued treatment early during the 96-week, double-blind, double-dummy phase without any protocol-defined relapse during the 30 days prior to discontinuation of study treatment will be randomly assigned to have an event at the date of treatment discontinuation; the other 50% of these patients will be censored at the date of treatment discontinuation. A total of 1000 (m = 1000) imputed datasets will be produced.

  The primary model described in the "Analysis Methods" section above will be applied to each of the imputed datasets. Each imputed dataset will produce an estimate of the difference between ocrelizumab and interferon beta-1a 44 μg SC. The multiple imputation estimator of the difference between ocrelizumab and interferon beta-1a 44 μg SC is the average of the individual 1000 estimators. The variance of the estimator is the combination of the between- and within-imputation variability (Carpenter and Kenward 2007).

- The annualized protocol-defined relapse rate at Week 96 will be calculated such that patients who discontinued treatment early during the 96-week, double-blind, double-dummy phase without any protocol-defined relapse during the 30 days prior to discontinuation of study treatment will be counted as having had a relapse on the date of treatment discontinuation. This approach is similar to above, but here the

described approach is applied to 100% of such patients (hence no imputation), rather than randomly to 50% of such patients (with multiple imputation).

### 4.4.2 Secondary Efficacy Endpoints

#### 4.4.2.1 Rationale for Hierarchy of Secondary Efficacy Endpoints

The hierarchical order of secondary efficacy endpoints is shown below, with endpoints listed in descending order of importance. The rationale for this hierarchical order of secondary endpoints is based primarily on clinical meaningfulness (i.e., those endpoints that are clinically more meaningful are listed higher in the hierarchy). In situations where endpoints have similar clinical relevance, those endpoints with a greater chance of achieving a statistically significant treatment difference are listed higher in the hierarchy. Established, rather than novel, endpoints are generally given higher priority within the hierarchy.

For patients, reduction in disability progression is a highly meaningful clinical outcome, reflecting the degree to which they are able to maintain independence and quality of life.

The following is list of all secondary endpoints, also called key endpoints:

- The time to onset of confirmed disability progression for at least 12 weeks, with the initial event of neurological worsening occurring during the 96-week, double-blind, double-dummy treatment period is listed as the first secondary efficacy endpoint on the basis of its clinical meaningfulness.
- The second and third secondary efficacy endpoints listed in the hierarchy are MRI parameters (T1 Gd-enhancing lesions and new and/or enlarging T2 hyperintense lesions) that historically have shown to correlate well with the clinical course.
- The fourth secondary efficacy endpoint, disability improvement, is a clinical endpoint that, if achieved, would have even greater impact on the quality of life of patients than reduction in disability progression. However, it represents a novel endpoint. Therefore, this endpoint is placed lower within the hierarchy than the above endpoints.
- The fifth secondary efficacy endpoint is the time to onset of confirmed disability progression for at least 24 weeks, with the initial event of neurological worsening occurring during the 96-week, double-blind, double-dummy treatment period, but the modality being tested here (EDSS) is identical to that of the first secondary endpoint. Given that the number of 24-week CDP events will be lower than the number of 12 week CDP events, there is likely a lower chance of achieving a statistically significant treatment difference, and hence, the 24 week CDP is listed lower than the 12 week CDP. It is, however, listed relatively higher in the hierarchy as the fifth endpoint, given the greater clinical meaningfulness of disability progression than the remaining endpoints.

- The sixth and eighth secondary endpoints, chronic black holes and brain volume, are indicators of brain tissue loss. The possible correlation of such brain-atrophy measures and long-term clinical outcomes has been suggested in other studies (Barkhof et al. 2009). Therefore, because they are imaging modalities and the correlation to clinical outcomes is weaker than the imaging endpoints that are listed higher in the hierarchy, these endpoints are listed lower in the hierarchy than the clinical endpoints.
- The EDSS does not adequately assess upper limb function and cognitive impairment; therefore, the MSFC scale is used to address this gap in clinical disability assessment. However, since the MSFC scale is not as widely used in other studies and not as well-accepted as a validated endpoint, it is listed seventh in the hierarchy.
- The SF-36 is one of the most widely used and validated instruments for measuring quality of life in patients with MS. It is, however, listed as ninth in the hierarchy below all the other clinical measures because quality-of-life instruments in general are not as widely used in confirmatory clinical studies compared with the other clinical measures.
- The tenth and final secondary endpoint is a composite measure encompassing the absence of disease activity based on clinical and MRI parameters (i.e., consisting of the primary endpoint and secondary endpoints 1, 2, and 3 above). It is a highly meaningful endpoint for patients but is listed as last in the hierarchy because it is a combination of previous secondary endpoints.

#### 4.4.2.2 Statistical Testing Strategy for Secondary Efficacy Endpoints

Secondary efficacy endpoints will be tested in the sequence presented in the hierarchical order listed in Figure 1, all at the $\alpha = 0.05$ level.

All p-values will be reported and will be interpreted as either confirmatory or non-confirmatory (i.e., descriptive only). The circumstances for confirmatory and for non-confirmatory interpretation of p-values are described below, and the sequence of the confirmatory tests is illustrated in Figure 1.

The first secondary endpoint in the hierarchy is the time to onset of confirmed disability progression for at least 12 weeks with the initial event of neurological worsening occurring during the 96-week, double-blind, double-dummy treatment period, and the fifth secondary endpoint is the same, except progression is defined as sustained for at least 24 weeks during the 96-week comparative treatment period. The fourth secondary endpoint is the proportion of patients who have confirmed disability improvement for at least 12 weeks with the initial event of neurological improvement occurring during the 96-week, double-blind, double-dummy treatment. For these three endpoints, only when data from both Studies WA21092 and WA21093 are combined will there be sufficient statistical power to detect relevant treatment differences. For the other secondary efficacy endpoints and the primary efficacy endpoint (annualized protocol-defined relapse rate by 2 years), there is sufficient statistical power within each study to detect relevant treatment differences, without needing to combine data from the two studies.

As a consequence of this, for analyses of secondary efficacy endpoints at the individual study level:

- For the first secondary efficacy endpoint (the time to onset of confirmed disability progression for at least 12 weeks), the study-level p-value will be interpreted as non-confirmatory, due to inadequate statistical power at the study level to detect relevant treatment differences.
- The second secondary efficacy endpoint (total number of T1 Gd-enhancing lesions at Weeks 24, 48, and 96) will be tested in a confirmatory manner if and only if, in the analysis of both studies combined, the first secondary efficacy endpoint reaches a significance level of 0.05 (i.e., pooled analysis $p \leq 0.05$). If, in the analysis of the combined studies, the first secondary efficacy endpoint pooled analysis $p > 0.05$, then the second and subsequent secondary efficacy endpoint p-values within the hierarchy will be interpreted as non-confirmatory.
- The third secondary efficacy endpoint (total number of new and/or enlarging T2 hyperintense lesions at Weeks 24, 48, and 96) will be tested in a confirmatory manner if and only if the second secondary efficacy endpoint (total number of T1 Gd-enhancing lesions at Weeks 24, 48, and 96) reaches a significance level of 0.05 (i.e., $p \leq 0.05$). If the second secondary efficacy endpoint $p > 0.05$, then the third and subsequent secondary efficacy endpoint p-values within the hierarchy will be interpreted as non-confirmatory.
- For the fourth (proportion of patients who have confirmed disability improvement for at least 12 weeks) and fifth (time to onset of confirmed disability progression for at least 24 weeks) secondary efficacy endpoints, the study-level p-value will be interpreted as non-confirmatory, due to inadequate statistical power at the study level to detect relevant treatment differences.
- The sixth secondary efficacy endpoint (total number of new T1-hypo-intense lesions (chronic black holes) at Weeks 24, 48, and 96) will be tested in a confirmatory manner if and only if, in the analysis of the combined studies, the fifth secondary efficacy endpoint (time to onset of confirmed disability progression for at least 12 weeks) reaches a significance level of 0.05 (i.e., pooled analysis $p \leq 0.05$). If, in the analysis of both studies combined, the fifth secondary efficacy endpoint pooled analysis $p > 0.05$, then the sixth and subsequent secondary efficacy endpoint p-values within the hierarchy will be interpreted as non-confirmatory.
- The seventh (and subsequent) secondary efficacy endpoint will be tested in a confirmatory manner if and only if the sixth (or immediately previous) secondary efficacy endpoint reaches a significance level of 0.05 (i.e., $p \leq 0.05$). If the sixth (or immediately previous) secondary efficacy endpoint $p > 0.05$, then the seventh (or current) and all subsequent secondary efficacy endpoint p-values within the hierarchy will be interpreted as non-confirmatory.

Furthermore, for analyses of secondary efficacy endpoints where data from both studies are combined (so there is sufficient statistical power for all primary and secondary efficacy endpoint comparisons):

- The first secondary efficacy endpoint (the time to onset of confirmed disability progression for at least 12 weeks) will be tested in a confirmatory manner if and only if the primary efficacy endpoint (annualized protocol-defined relapse rate by 2 years), reaches a significance level of 0.05 (i.e., pooled analysis $p \leq 0.05$). If the primary efficacy endpoint pooled analysis $p > 0.05$, then all secondary efficacy endpoint pooled analysis p-values within the hierarchy will be interpreted as non-confirmatory.
- The second (and subsequent) secondary efficacy endpoint will be tested in a confirmatory manner if and only if the first (or immediately previous) secondary efficacy endpoint reaches a significance level of 0.05 (i.e., pooled analysis $p \leq 0.05$). If the first (or immediately previous) secondary efficacy endpoint pooled analysis $p > 0.05$, then the second (or current) and all subsequent secondary efficacy endpoint pooled analysis p-values within the hierarchy will be interpreted as non-confirmatory.

The sequence of the confirmatory testing is illustrated in Figure 1 below.

The arrows on the left illustrate the hierarchy of the statistical tests of Study WA21092. The arrows on the right illustrate the hierarchy of the statistical tests of Study WA21093. The boxes in the middle highlight the analyses performed in the pooled data set of WA21092 and WA21093.

For example, for Study WA21092, the primary endpoint ARR should be positive in both studies ($p \leq 0.05$). After the poolability of both Studies WA21092 and WA21093 has been observed (see more details in the Pooled SAP), the first secondary endpoint is tested in the pooled dataset WA21092 and WA21093. Then, the analysis of T1 Gd-enhancing lesions is performed for Study WA21092. If this is statistically significant ($p \leq 0.05$), the T2 hyperintense lesions are analyzed for Study WA21092. If this is statistically significant ($p \leq 0.05$), the analysis of confirmed disability improvement is done in the pooled data of Studies WA21092 and WA21093. If this is statistically significant ($p \leq 0.05$), then the analysis of Confirmed Disability Progression for at least 24 weeks is performed in the pooled data. If this is statistically significant ($p \leq 0.05$), the Chronic Black Holes endpoint is analyzed in Study WA21092. Then, the sequence of tests continues in the order of MSFC, brain volume, SF-36 PCS, and NEDA for Study WA21092.

**Figure 1 Hierarchical Order of Key Efficacy Endpoints**

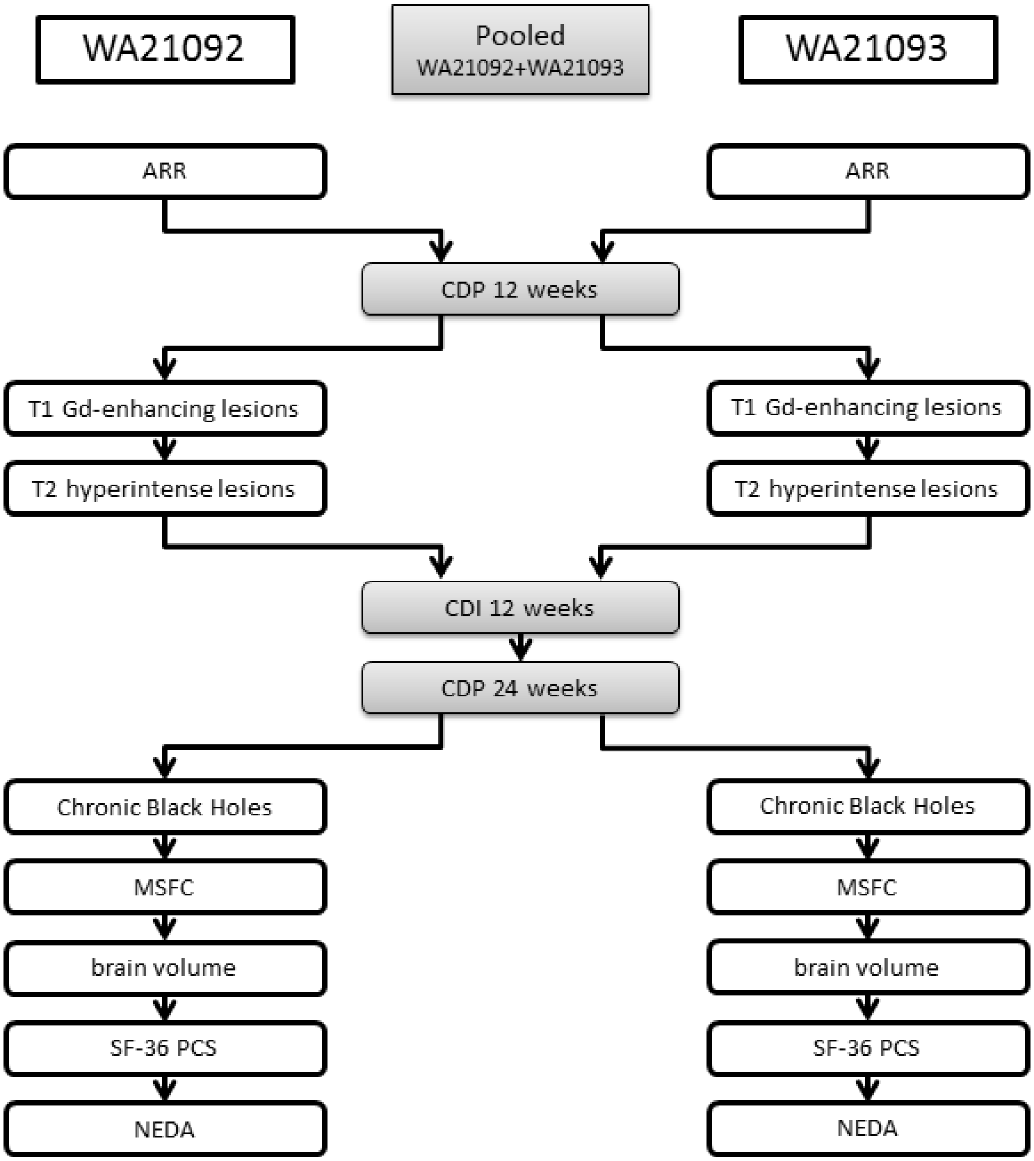


ARR = annualized relapse rate; CDI = Confirmed Disability Improvement; CDP = confirmed disability progression; Gd = gadolinium; MSFC = Multiple Sclerosis Functional Composite Scale; NEDA = no evidence of disease activity; SF-36 PCS = short form 36 Physical Component Summary.

#### 4.4.2.3 Time to Onset of Confirmed Disability Progression for at Least 12 Weeks with the Initial Event of Neurological Worsening Occurring during the 96-Week, Double-Blind, Double-Dummy Treatment Period

Disability progression as measured by EDSS will be assessed in all patients by the independent examining investigator at screening and baseline visit, and every 12 weeks throughout the study until the end of the double-blind, double-dummy treatment period at

Week 96, after which disability progression is assessed at Weeks 12 and 24 in the OLE and then every 24 weeks thereafter. Additional EDSS assessments for individual patients may be requested between visits (i.e., during an MS relapse).

Section 2.4 provides additional information on the timing of this analysis.

The EDSS is based on a standard neurological examination; the seven categories of the EDSS representing functional systems (Pyramidal, Cerebellar, Brainstem, Sensory, Bowel and Bladder, Visual, plus “Other”) are rated and scored (collectively, FSS). Each score of the FSS is an ordinal clinical rating scale ranging from 0 to 5 or 6. These ratings are then used in conjunction with observations and information concerning ambulation and use of assistive devices (which will also be scored) to determine the EDSS score. The EDSS is a disability scale that ranges in 0.5-point steps from 0 (normal) to 10.0 (death).

Because of the variability of EDSS, a single measurement at the baseline visit might not provide a sufficiently reliable baseline EDSS. Therefore, using an average score from two separate measurements may improve the reliability of baseline EDSS and the reliability of confirmed disability progression seen in the study. Inclusion criteria require neurological stability for 30 days prior to both screening and baseline visits. Generally, it takes 2 weeks for screening. There should be little change in EDSS except for the measurement variability during the screening period, assuming patients are clinically neurologically stable during this period.

In the derivation of time-to-onset confirmed-disability progression, baseline EDSS is defined as the average of the EDSS scores at the screening and baseline visits, without rounding.

Progression is defined a $\geq$1–point increase in EDSS score from a baseline EDSS score of 0.0–5.5 inclusive, and a 0.5-increase from a baseline EDSS score higher than 5.5.

For example, for a patient with a baseline EDSS score of 5.25 or 5.5, the progression is defined as an EDSS score of at least 6.5. For a patient with a baseline EDSS score of 5.75 or 6.0, the progression is defined as an EDSS score of at least 6.5.

The inclusion criterion of EDSS (0–5.5) only applies to screening EDSS. Hence, it is still possible for a patient’s baseline EDSS score (derived from both screening and Day 1 EDSS results) to be $>$5.5. Confirmation requires the sustained change in EDSS for at least 12 weeks from the initial progression. Initial progression can happen at any visit during the 96-week, double-blind, double-dummy treatment period.

Confirmation of disability progression must occur at the regularly scheduled visit that is at least 12 weeks (84 days) after initial progression. If a patient has a missing EDSS at the scheduled visit occurring at least 84 days after an initial progression or the

scheduled visit occurs several days before the 84-day window after an initial progression (e.g., the visit window is ±4 days), confirmation of the disability progression must be on the basis of the assessment at the next scheduled visit. There may be EDSS assessments at unscheduled or scheduled visits that are <84 days after the initial progression that are between the initial progression visit and the confirmation visit. Disability progression cannot be confirmed unless the EDSS values meet the minimum change required for progression. The non-confirmatory EDSS assessments (if any) between the initial disability progression and the confirmation of disability progression should be at least as high as the minimum change required for progression. All initial disability progression events up to Week 96 with corresponding confirmation visits at a subsequent scheduled visit will be taken into account for the statistical analysis, irrespective of whether the confirmation visit occurred during the treatment phase or in the SFU phase or OLE phase after study-drug discontinuation. Thus, patients who prematurely discontinue study drug treatment should remain in the SFU phase of the study, and every effort should be made to obtain a follow-up EDSS status at the next scheduled visit. Patients who, according to the above definition, did not have onset of confirmed disability progression by the Week–96 visit, by the time of the early discontinuation of treatment, or are lost to follow up will be censored at the date of the last EDSS assessment during the 96-week, double-blind, double-dummy treatment period.

Data from the two studies (with respect to the ocrelizumab group vs. the interferon beta-1a 44 μg SC group) will be pooled for analysis of this endpoint at the $\alpha = 0.05$ level. Only data collected during the 96-week treatment period will be used for this analysis, although confirmation of disability progression during the 96-week, double-blind, double-dummy treatment period may occur during a subsequent study phase (i.e., during the SFU phase or the OLE phase).

Time to confirmed disability progression in the ocrelizumab group and in the interferon beta-1a 44 μg SC group will be compared using a two-sided log-rank test stratifying by geographical region (United States vs. ROW), and baseline EDSS (<4.0 vs. ≥4.0). The proportion of patients with confirmed disability progression will be estimated using Kaplan-Meier methodology. A by-treatment Kaplan-Meier plot will be presented for this endpoint. The overall hazard ratio will be estimated using a stratified Cox regression model with treatment group as covariate and the same stratification factors used in the stratified log-rank test above.

Sample SAS code can be found below (SAS code is regarded as “draft” until fully validated at the analysis stage):

```
* Log-rank test;
   proc lifetest data=CDP12;
      time CDP12TIME*CNSR(1);
      strata REGION BEDSSCAT / group=ARMCD;
      ods output HomTests =_logrank;
   run;

* Cox regression model;
proc phreg data=CDP12;
      class ARMCD / descending;
      model CDP12TIME*CNSR(1) = ARMCD / rl;
      strata REGION BEDSSCAT;
      ods output ParameterEstimates=est;
   run;
```

The analysis described above will be repeated for the per-protocol population as a sensitivity analysis.

Additionally, the following sensitivity analyses will be performed:

- Use of two different methods for handling of missing data:

  Method 1: with use of the same kind of multiple imputation approach described at the end of Section 4.4.1, patients with an initial disability progression during the 96-week, double-blind, double-dummy treatment period who discontinue the treatment early and do not have a subsequent visit with EDSS measurement will be randomly imputed as an event of confirmed disability progression at the date of initial disability progression with a probability of 50%; the other 50% of these patients will be censored at the date of initial disability progression. A total of 1000 (m = 1000) imputed datasets will be produced.

  Method 2: Patients with an initial disability progression during the 96-week, double-blind, double-dummy treatment period who discontinue the treatment early and do not have a subsequent visit with EDSS measurement will be imputed as an event of confirmed disability progression at the date of initial disability progression.

- Adjustment for some additional baseline factors: Sensitivity analysis will use the same tests above but will, in addition, adjust for the number of relapses occurring within a 2-year period prior to study entry, baseline presence of Gd lesions (present or absent), prior MS treatment, and age ($<40$ vs. $\geq 40$ years).

#### 4.4.2.4 Total Number of T1 Gadolinium–Enhanced Lesions as Detected by Brain Magnetic Resonance Imaging at Weeks 24, 48, and 96

The total number of T1 Gd–enhanced lesions will be calculated as the sum of the individual number of T1 Gd–enhanced lesions at Weeks 24, 48, and 96. Data from other unscheduled assessments will not be included in this summary or analysis.

The same approach to analysis will be applied here as described in Section 4.4.1, "Analysis Methods" section, with the following exceptions:

- In order to account for patients receiving varying numbers of brain MRI scans during the study, the log-transformed number of brain MRI scans received will be included in the model as an "offset" variable for appropriate computation. This approach is preferable to imputation of missing values using the average of non-missing observations, because only three timepoints would give a high variability in the imputed average values. Additionally, the "offset" variable approach aligns more closely with approaches to analysis used a) in regulatory submissions for other MS therapies, b) for the primary endpoint, and c) in this program's Phase II study.
- Baseline covariates here will be as follows: baseline Gd lesion (present or not), treatment group, baseline EDSS score ($<$4.0 vs. $\geq$4.0), and geographical region (United States vs. ROW).

Sample SAS code can be found below (SAS code is regarded as "draft" until fully validated at the analysis stage):

```
proc genmod data=MRIT1;
   class ARMCD REGION BEDSSCAT BGDLESFL;
   model N_MRIT1 = ARMCD REGION BEDSSCAT BGDLESFL /
   offset=MRILOG link=log dist=negbin type3;
   lsmeans ARMCD / exp cl;
   ods output lsmeans=lsm;
   ods output ParameterEstimates=est;
run;
```

If the model fails to converge due to high number of zero T1 lesion counts, a logistic regression model will be performed on Gd lesion (present or not) adjusted for the same baseline variables. Patients withdrawn from treatment and having no T1 lesions: those withdrawn because of lack of efficacy or death are considered as having T1 lesions; otherwise, it will be considered a missing observation:

```
proc logistic data=MRIT1;
   class ARMCD REGION BEDSSCAT BGDLESFL;
   model N_MRIT1_CAT = ARMCD REGION BEDSSCAT BGDLESFL;
   oddsratio ARMCD /cl=wald;
   ods output OddsRatiosWald = oddsr;
run;
```

#### 4.4.2.5 Total Number of New and/or Enlarging T2 Hyperintense Lesions as Detected by Brain Magnetic Resonance Imaging at Weeks 24, 48, and 96

The total number of new and/or enlarging T2 lesions will be calculated as the sum of the individual number of new and/or enlarging lesions at Weeks 24, 48, and 96. Data from other unscheduled assessments will not be included in this summary or analysis.

The same approach to analysis will be applied here as described in Section 4.4.2.4, with the following exception.

- Baseline covariates here will be as follows: baseline T2 lesion count, treatment group, baseline EDSS score ($<4.0$ vs. $\geq 4.0$) and geographical region (United States vs. ROW).

Sample SAS code can be found below (SAS code is regarded as “draft” until fully validated at the analysis stage):

```
proc genmod data=MRIT2;
   class ARMCD REGION BEDSSCAT;
   model N_MRIT2 = ARMCD REGION BEDSSCAT BT2LES /
   offset=MRILOG link=log dist=negbin type3;
   lsmeans ARMCD / exp cl;
   ods output lsmeans=lsm;
   ods output ParameterEstimates=est;
run;
```

If the model fails to converge due to a high number of zero T2 lesion counts, a logistic regression model will be performed on new/enlarging T2 lesion (present or not) adjusted for the same baseline variables. Patients withdrawn from treatment and having no T2 lesions: those withdrawn due to lack of efficacy or death are considered as having T2 lesions; otherwise, it will be considered a missing observation.

#### 4.4.2.6 Proportion of Patients Who Have Confirmed Disability Improvement Sustained for at Least 12 Weeks with the Initial Event of Neurological Improvement Occurring during the 96-Week, Double-Blind, Double-Dummy Treatment Period

This endpoint will be analyzed only for the subgroup of patients with a baseline EDSS score $\geq 2.0$. Exactly the same approach to data derivation will be used for disability improvement as for disability progression (refer to Section 4.4.2.3, although note that here the endpoint is a binary improved/not improved variable, rather than a time-to-event endpoint). In particular, the same approach to the timing of the confirmation of disability improvement will be applied as for disability progression. The baseline EDSS is the average of the screening EDSS score and the score of the baseline visit, without rounding. For patients with a baseline EDSS score $\geq 2$ and $\leq 5.5$, disability improvement is defined as a reduction in EDSS score $\geq 1.0$ compared to baseline EDSS score. For patients with a baseline EDSS score $>5.5$, disability improvement is defined as a reduction in EDSS score of $\geq 0.5$. All patients without disability improvement will be counted as not improved, independent of follow-up time

This analysis requires both Studies WA21092 and WA21093 to be combined to have sufficient statistical power to detect relevant treatment differences. In the study-level analysis, the p-value for this treatment comparison will be interpreted as non-confirmatory.

The proportions in treatment groups will be compared using the Cochran-Mantel-Haenszel (CMH) $\chi^2$ test stratified by geographical region (United States vs. ROW), and baseline EDSS score ($<$4.0 vs. $\geq$4.0).

Sample SAS code can be found below (SAS code is regarded as “draft” until fully validated at the analysis stage):

```
proc freq data=CDI12;
   tables REGION*BEDSSCAT*ARMCD*CDI12 / cmh;
   ods output CMH=pval;
   ods output CommonRelRisks=risks;
run;
```

#### 4.4.2.7 Time to Onset of Confirmed Disability Progression for at Least 24 Weeks with the Initial Event of Neurological Worsening Occurring during the 96-Week, Double-Blind, Double-Dummy Treatment Period

This is the same as the 12-week confirmed disability progression, except that the confirmation of disability progression must occur at the regularly scheduled visit that is $\geq$24 weeks ($\geq$161 days) after initial disability progression. Section 2.4 provides additional information on the timing of this analysis.

All initial disability progression events up to Week 96 with corresponding confirmation visits at the next schedule visit will be taken into account for the statistical analysis. The same analysis principles as described in Section 4.4.2.3 will be applied to the 24-week disability endpoint. The sensitivity analyses will be performed for the 24-week disability endpoint in the same way as for the 12-week endpoint.

Sample SAS code can be found below (SAS code is regarded as “draft” until fully validated at the analysis stage):

```
* Log-rank test;
   proc lifetest data=CDP24;
      time CDP24TIME*CNSR(1);
      strata REGION BEDSSCAT / group=ARMCD;
      ods output HomTests =_logrank;
   run;
```

```
* Cox regression model;
   proc phreg data=CDP24;
      class ARMCD / descending;
      model CDP24TIME*CNSR(1) = ARMCD / rl;
      strata REGION BEDSSCAT;
      ods output ParameterEstimates=est;
   run;
```

#### 4.4.2.8 Total Number of New T1-Hypo-Intense Lesions (Chronic Black Holes) at Weeks 24, 48, and 96

The total number of new T1-hypo-intense lesions (chronic black holes) will be calculated as the sum of the individual number of new lesions at Weeks 24, 48, and 96. Data from other unscheduled assessments will not be included in this summary or analysis.

The same approach to analysis will be applied here as described in Section 4.4.2.4, with the following exception:

- Baseline covariates here will be as follows: treatment group, baseline T1-hypo-intense lesions count, baseline EDSS score ($<$4.0 vs. $\geq$4.0) and geographical region (United States vs. ROW).

Sample SAS code can be found below (SAS code is regarded as “draft” until fully validated at the analysis stage):

```
   proc genmod data=MRICBH;
      class ARMCD REGION BEDSSCAT;
      model N_MRICBH = ARMCD REGION BEDSSCAT BT1LES /
      offset=MRILOG link=log dist=negbin type3;
      lsmeans ARMCD / exp cl;
      ods output lsmeans=lsm;
      ods output ParameterEstimates=est;
   run;
```

If the model fails to converge due to high number of zero chronic black hole counts, a logistic regression model will be performed on chronic black hole (present or not), adjusted for the same baseline variables. Patients withdrawn from treatment and having no chronic black holes: those withdrawn due to lack of efficacy or death are considered as having chronic black holes; otherwise, it will be considered a missing observation.

Note that the protocol and previous versions of this SAP did not make it clear that only new T1 hypo-intense lesions would be considered.

#### 4.4.2.9 Change in Multiple Sclerosis Functional Composite Scale Score from Baseline to Week 96

There are three primary measures in a MSFC score: 1) Timed 25-Foot walk; 2) 9-Hole Peg Test (9-HPT); and 3) Paced Auditory Serial Addition Test (PASAT-3 version). The MSFCS is based on the concept that scores for these three dimensions— (arm, leg, and cognitive function) are combined to create a single score (the MSFC) that can be used to detect change over time in a group of patients with MS. Since the three primary measures differ in what they actually measure (time for the 9-HPT and 25-Foot Timed Walk, but number of correct answers for the PASAT-3), a common metric for these

variables has been used to create a composite score for the three different measures. Z-score was selected for this purpose. The results from each of these three tests are transformed into Z-scores and averaged to yield a composite score for each patient at each timepoint.

MSFC Score = $\{Z_{arm}$, average + $Z_{leg}$, average + $Z_{cognitive}\}$ / 3.0

Where Zxxx = Z-score

The following are MSFC components:

- Trial 1, Timed 25-Foot Walk
- Trial 2, Timed 25-Foot Walk
- Trial 1, Dominant Hand, 9-HPT
- Trial 2, Dominant Hand, 9-HPT
- Trial 1, Non-Dominant Hand, 9-HPT
- Trial 2, Non-Dominant Hand, 9-HPT
- PASAT-3

There are three derived results for three tests: 1) the average (reciprocal) scores from the four trials on the 9-HPT (the two trials for each hand are averaged, converted to the reciprocals of the mean times for each hand and then the two reciprocals are averaged); 2) the average scores of two 25-Foot Timed Walk trials; and 3) the number correct from the PASAT-3. The Z-score will be created for these three derived results to yield the composite score by the following formula.

Formula for Creating the MSFC Score to Compare Groups within a Study (The Preferred Method)

MSFC Score = $\{ \{(\text{Average } (_{1/9\text{-HPT}}) - \text{Baseline Mean } (_{1/9\text{-HPT}}))/ \text{ Baseline Std Dev } (_{1/9\text{ HPT}})\}$
$+ \{(\text{Average }_{25\text{-Foot Walk}} - \text{Baseline Mean }_{25\text{-Foot Walk}})/\text{Baseline Std-Dev }_{25\text{-Foot Walk}}\}$
$+ \{(\text{PASAT-3} - \text{Baseline Mean }_{\text{PASAT-3}}) /\text{Baseline Std Dev }_{\text{PASAT-3}}\} \} / 3.0$

Note: “Average (1/9-HPT)” is the average of the inverse (reciprocal) for the mean time of the two trials on the right hand and reciprocal of the mean time of the two left-hand trials from the test patient; Baseline Mean (1/9-HPT) and Std Dev (1/9-HPT) are the baseline values from each patient in all study groups combined at the baseline assessment; “Average 25-Foot Walk” is the mean time from the two trials of the 25-foot timed walk; and we take the negative value of the Z-score to make the direction of change the same as the other components. Similarly, the Baseline Mean and Std Dev 25-Foot Walk are of all Baseline Groups combined; and “PASAT-3” is the score from the test patient, and the Baseline Mean PASAT-3 and Std Dev PASAT-3 of the combined baseline assessments. These are discussed below.

Note that changing the signs of the Z-scores for the 25-Foot Walk and using inverse value of 9-HPT to ensure that a negative value implies worsening, and a positive value implies improvement. More details can be found in the MSFC Manual.

Missing data will be imputed as follows:

- For results within a given test:

  25-Foot Timed Walk trials: If the test results are not available due to a "physical limitation," impute the maximum possible value for the scale. If the test results are not available but it is NOT due to a "physical limitation," use the result from the other trial to impute the missing value.

  9-HPT: If the test results are not available due to a "physical limitation," impute the maximum possible value for the scale. If the test results are not available but it is NOT due to a "physical limitation," impute the missing test score using the result from the other trial of the same hand. If the result from the other trial of the same hand is also missing, use the average score from the other hand (or available score from one trial if the result from one trial only is available).

- For the composite score:

  All available data will be used for analysis regardless of the timing of any protocol-defined relapse.

Outliers will be applied with the following rules:

- For 9-HPT, the lower bound is set to be 10 seconds (Oxford Grice et al. 2003), and the upper bound is set to be 300 seconds, according to the MSFC Manual.
- For 25-FWT, the lower bound is set to be 2.2 seconds (Bohannon 1997), and upper bound is set to be 180 seconds, according to the MSFC Manual.
- For PASAT, the lower and upper bounds are set to be 0 and 60 according to the MSFC Manual.
- For values outside the lower and upper bounds, they will be treated as missing, and the imputation rule applied as defined above.

The MSFC score will be summarized at baseline and at Weeks 12, 24, 36, 48, 60, 72, 84, and 96 using descriptive statistics for each treatment group. Data from other unscheduled assessments will not be included in this summary or analysis. The change from baseline to Weeks 12, 24, 36, 48, 60, 72, 84, and 96 in MSFC score will be computed and summarized using descriptive statistics for each treatment group.

For the assessment of differences in the mean change from baseline in MSFC score up to Week 96, a Mixed-Effect Model Repeated Measures analysis (MMRM) incorporating post-randomization data collected up to 96 weeks of treatment will be used to assess all data collected over time with consideration of the variance−covariance matrix of the

repeated measures. This method allows for a general unstructured variance−covariance matrix and will include data from patients with incomplete data from some scheduled timepoints.

The model will be implemented in SAS using the MIXED procedure and will include the change from baseline in MSFC score as the dependent variable. The fixed effects in the model will include independent variables of randomized treatment, visit (nominal post ≤baseline visits as per the Schedule of Assessments) and treatment-by-visit interaction, along with the following baseline covariates: baseline MSFC score, baseline MSFC score by visit interaction, geographical region (United States vs. ROW), and baseline EDSS score ($<$4.0 vs. $\geq$4.0). Visit will be treated as a repeated variable within a patient. Patient, treatment, and visit will be treated as factor variables. An unstructured variance−covariance structure will be applied to model the within-patient errors. The model will be fitted using the Restricted Maximum Likelihood method (REML). Denominator degrees of freedom will be estimated using Satterthwaite's approximation.

To estimate the difference between the ocrelizumab and interferon beta-1a 44 μg SC groups in mean change from baseline to Week 96, a treatment-by-visit interaction contrast will be constructed (i.e., the treatment group contrast at Week 96). On the basis of this analysis, least square means, standard errors, and the 95% confidence interval for the treatment difference will be reported.

Graphical presentations for least square means and 95% confidence intervals will be used to illustrate trends over time.

Sample SAS code can be found below (SAS code is regarded as "draft" until fully validated at the analysis stage):

```
proc mixed data=MSFCS method=REML;
   class REGION BEDSSCAT ARMCD VISIT USUBJID;
   model change  =  BFCS  REGION  BEDSSCAT  ARMCD  VISIT
    BFCS*VISIT  ARMCD*VISIT / ddfm= satterthwaite;
   repeated VISIT / type=un subject = USUBJID;
   lsmeans ARMCD*VISIT / pdiff cl;
   ods output lsmeans = lsm;  * contains the adjusted means;
   ods output diffs = dif;  * contains treatment differences;
run;
```

#### 4.4.2.10 Percentage Change in Brain Volume as Detected by Brain Magnetic Resonance Imaging Scan from Week 24 to Week 96

Brain volume is recorded as an absolute "normalized" value at the baseline visit then recorded at subsequent visits as a percentage change relative to the absolute value at the baseline visit. Therefore, brain volume at Week 24 will be calculated as the brain volume at the baseline visit multiplied by 1+([percentage change in brain volume from baseline visit to Week 24]/100). This value will be used to determine the percentage change in brain volume at Weeks 48 and 96 relative to Week 24.

Percentage change in brain volume relative to Week 24, as detected by brain MRI scan, will be computed and summarized at Weeks 48 and 96 using descriptive statistics for each treatment group. Data from other unscheduled assessments will not be included in this summary or analysis.

For the assessment of differences in the mean percentage change in brain volume on MRI scans from Week 24 to Week 96, an MMRM analysis will be undertaken using the same approach as described in Section 4.4.2.9, except for the following:

- The response variable will be the percentage change from Week 24 to subsequent visits (Weeks 48 and 96). Baseline brain volume data will not feature in this analysis.
- Baseline covariates here will be as follows: brain volume at Week 24, geographical region (United States vs. ROW), and baseline EDSS score (<4.0 vs ≥4.0)
- To estimate the difference between the ocrelizumab and interferon beta-1a 44 μg SC groups in mean change from Week 24 to Week 96, a treatment-by-visit interaction contrast will be constructed (i.e., the treatment group contrast at Week 96)

Sample SAS code can be found below (SAS code is regarded as “draft” until fully validated at the analysis stage):

```
proc mixed data=MRINBV method=REML;
   class REGION BEDSSCAT ARMCD VISIT USUBJID;
   model change  =  W24NBV  REGION  BEDSSCAT  ARMCD  VISIT
    W24NBV*VISIT  ARMCD*VISIT / ddfm= satterthwaite;
   repeated VISIT / type=un subject = USUBJID;
   lsmeans ARMCD*VISIT / pdiff cl;
   ods output lsmeans = lsm;  * contains the adjusted means;
   ods output diffs = dif;  * contains treatment differences;
run;
```

The choice of Week 24 as “baseline” in this analysis, rather than the baseline visit, is a widely accepted approach to establish a stable baseline and avoid the effect of pseudoatrophy commonly reported for immunomodulatory therapies.

#### 4.4.2.11 Change in Quality of Life, as Measured by the Short Form 36 Version 2 Physical Component Summary Score from Baseline to Week 96

The Short Form 36 Version 2 (SF-36v2) is a 36-item, self-reported, generic measure of quality of life that has been widely used in multiple disease areas. It is composed of eight health domains: Physical Functioning (PF), Role-Physical (RP), Bodily Pain (BP), General Health (GH), Vitality (VT), Social Functioning (SF), Role-Emotional (RE), and Mental Health (ME). On the basis of these domain scores, the PCS Score and the Mental Component Summary (MCS) Score will be computed. The standard form of the instrument, with a 4-week recall, is being administered in the studies.

Scoring and rules for missing items will follow the SF-36v2 User's Manual. In brief, scoring for each health domain scale involves (a) recoding item response values, (b) summing recoded response values for all items in a given scale to obtain the scale raw score, and (c) transforming the scale raw score to a 0–100 score. The PCS score is computed by (a) multiplying each health domain Z-score by a scale-specific physical factor score coefficient, (b) summing the resulting products, and (c) converting the product total to a T score. The MCS score is computed in the same manner, using scale-specific mental factor score coefficients.

The PCS scores will be summarized at baseline and Weeks 48 and 96 using descriptive statistics for each treatment group. Data from other unscheduled assessments will not be included in this summary or analysis. The change from baseline to Weeks 48 and 96 in PCS scores will be computed and summarized using descriptive statistics for each treatment group.

For the assessment of differences in the mean change in PCS score during the 96-week comparative treatment period from baseline up to Week 96, MMRM analyses will be undertaken using the same approach as described above in Section 4.4.2.9, except baseline covariates here will be as follows: Baseline PCS score, geographical region (United States vs. ROW), and baseline EDSS score ($<$4.0 vs. $\geq$4.0).

Sample SAS code can be found below (SAS code is regarded as "draft" until fully validated at the analysis stage):

```
proc mixed data=PCSS method=REML;
   class REGION BEDSSCAT ARMCD VISIT USUBJID;
   model change  =  BPCS  REGION  BEDSSCAT  ARMCD  VISIT
    BPCS*VISIT  ARMCD*VISIT / ddfm= satterthwaite;
   repeated VISIT / type=un subject = USUBJID;
   lsmeans ARMCD*VISIT / pdiff cl;
   ods output lsmeans = lsm;  * contains the adjusted means;
   ods output diffs = dif;  * contains treatment differences;
run;
```

#### 4.4.2.12 Proportion of Patients Who Have No Evidence of Disease Activity by Week 96

This endpoint will be defined only for those patients with a baseline EDSS score $\geq$2.0. All available data during the 96-week treatment period will be used for the analysis. Patients who complete the 96-week treatment period will be considered as having evidence of disease activity if at least one protocol-defined relapse, a CDP event or at least one MRI scan showing MRI activity (defined as Gd-enhancing T1 lesions, or new or enlarging T2 lesions) was reported during the 96-week treatment period, otherwise the patient will be considered as having NEDA. Patients who discontinue treatment early with at least one event before early discontinuation will be considered as having evidence of disease activity.

Even if an event was not reported before early discontinuation, the patient will be considered as having evidence of disease activity if the reason for early discontinuation is lack of efficacy or death; otherwise, it will be considered a missing observation.

Only data collected during the 96-week treatment period will be used for this analysis, although confirmation of disability progression during the 96-week, double-blind, double-dummy treatment period may occur during a subsequent study phase (i.e., during the SFU phase or the OLE phase).

The proportion of patients with NEDA within treatment groups will be compared using the CMH $\chi^2$ test stratified by geographical region (United States vs. ROW), and baseline EDSS score ($<$4.0 vs. $\geq$4.0).

Sample SAS code can be found below (SAS code is regarded as “draft” until fully validated at the analysis stage):

```
proc freq data=NEDA;
   tables REGION*BEDSSCAT*ARMCD*NEDA / cmh;
   ods output CMH=pval;
   ods output CommonRelRisks=risks;
run;
```

### 4.4.3 Exploratory Efficacy Endpoints

#### 4.4.3.1 Proportion of Relapse-Free Patients by 96 Weeks

All available data during the 96-week treatment period will be used for the analysis. Patients who complete the 96-week treatment period will be considered as not relapse-free if at least one protocol-defined relapse was reported during the 96-week treatment period; otherwise, the patient will be considered as relapse-free. Patients who discontinued treatment early with at least one relapse before early discontinuation will be considered as not relapse-free. Even if the patient did not have a relapse before early discontinuation, if the reason for early discontinuation is lack of efficacy or death, the patient will be considered as not relapse-free; otherwise, it will be considered as a missing observation.

The proportions within treatment groups will be compared using CMH $\chi^2$ test stratified by geographical region (United States vs. ROW), and baseline EDSS score ($<$4.0 vs. $\geq$4.0).

#### 4.4.3.2 Percentage Change in Total T2 Lesion Volume as Detected by Brain Magnetic Resonance Imaging from Baseline to Week 96

The total volume of T2 lesions on MRI scans of the brain will be summarized at baseline and Weeks 24, 48, and 96 using descriptive statistics for each treatment group. Data from other unscheduled assessments will not be included in this summary or analysis. The percentage change from baseline to Weeks 24, 48, and 96 in total volume of T2 lesions on MRI scans of the brain will be computed and summarized using descriptive statistics (e.g., mean, SD, median, minimum, maximum, and n) for each treatment group.

For the assessment of differences in the mean percentage change in total T2 lesion volume during the 96-week comparative treatment period from baseline up to Week 96, an MMRM analysis will be undertaken using the same approach as described in Section 4.4.2.9, except baseline covariates here will be as follows:  Baseline T2 lesion volume, geographical region (United States vs. ROW), and baseline EDSS ($<$4.0 vs. $\geq$4.0).

#### 4.4.3.3 Annualized Relapse Rate, Based on All Clinical Relapses at the End of the 96-Week Comparative Treatment Period

All clinical relapses reported on the MS relapse page will be used for this analysis (protocol-defined relapse is a subset of clinical relapse).

The annualized clinical relapse rate by Week 96 will be analyzed using the same general approach as described in Section 4.4.1, "Analysis Methods" for protocol-defined relapse (primary efficacy endpoint).  However, the sensitivity and robustness checks described in Section 4.4.1 will not be performed here.

#### 4.4.3.4 Annualized Protocol-Defined Relapse Rate by 2 Years (96 weeks) for Relapses Requiring Intravenous Steroid Therapy

All protocol-defined relapses requiring treatment with IV steroids will be used for this analysis.

The annualized relapse rate by Week 96 will be analyzed using the same general approach as described in Section 4.4.1, "Analysis Methods" for protocol-defined relapse (primary efficacy endpoint).  However, the sensitivity and robustness checks described in Section 4.4.1 will not be performed here.

#### 4.4.3.5 Annualized Protocol-Defined Relapse Rate by 2 Years (96 weeks) for Severe Relapses

All severe or life threatening protocol-defined relapses will be used for this analysis.

The annualized relapse rate by Week 96 will be analyzed using the same general approach as described in Section 4.4.1, "Analysis Methods" for protocol-defined relapse (primary efficacy endpoint).  However, the sensitivity and robustness checks described in Section 4.4.1 will not be performed here.

#### 4.4.3.6 Percentage Change in Brain Volume as Detected by Brain Magnetic Resonance Imaging from Baseline to Week 96

Percentage change in brain volume relative to the baseline visit, as detected by brain MRI scan, will be computed and summarized at Weeks 24, 48 and 96 using descriptive statistics for each treatment group.  Data from other unscheduled assessments will not be included in this summary or analysis.

For the assessment of differences in the mean percentage change in brain volume on MRI scans during the 96-week comparative treatment period from baseline up to

Week 96, an MMRM analysis will be undertaken using the same approach as described in Section 4.4.2.9, except baseline covariates here will be as follows: baseline brain volume, geographical region (United States vs. ROW), and baseline EDSS score ($<$4.0 vs. $\geq$4.0).

#### 4.4.3.7 Change in Timed 25-Foot Walk from Baseline to Week 96

The average time (for the 25-foot walk) from two trials will be used in this analysis. The imputation method for missing values in either of the two trials will be same as described in Section 4.4.2.9.

All available data at Weeks 12, 24, 36, 48, 60, 72, 84 and 96 will be used for analysis, regardless of the timing of any protocol-defined relapses.

The timed 25-foot walk will be summarized at baseline and at Weeks 12, 24, 36, 48, 60, 72, 84, and 96 using descriptive statistics for each treatment group. Data from other unscheduled assessments will not be included in this summary or analysis. The change from baseline to Weeks 12, 24, 36, 48, 60, 72, 84, and 96 in the timed 25-foot walk will be computed and summarized using descriptive statistics for each treatment group.

For the assessment of differences in the mean change in the timed 25-foot walk during the 96-week comparative treatment period from baseline up to Week 96, MMRM analyses will be undertaken using the same approach as described in Section 4.4.2.9.

Baseline covariates here will be as follows: Baseline timed 25-foot walk, geographical region (United States vs. ROW), and baseline EDSS score ($<$4.0 vs. $\geq$4.0)

#### 4.4.3.8 Change in 9-Hole Peg Test from Baseline to Week 96

The average score of the 9-hole peg test from four trials will be used in this analysis. The imputation method for missing values from any of the four trials will be same as in Section 4.4.2.9.

All available data at Weeks 12, 24, 36, 48, 60, 72, 84, and 96 will be used for analysis regardless of the timing of any protocol-defined relapses.

The 9-hole peg test will be summarized at baseline and at Weeks 12, 24, 36, 48, 60, 72, 84, and 96 using descriptive statistics for each treatment group. Data from other unscheduled assessments will not be included in this summary or analysis. The change from baseline to Weeks 12, 24, 36, 48, 60, 72, 84, and 96 in the 9-hole peg test will be computed and summarized using descriptive statistics for each treatment group.

For the assessment of differences in the mean change in the 9-hole peg test during the 96-week comparative treatment period from baseline up to Week 96, MMRM analyses will be undertaken using the same approach as described in Section 4.4.2.9.

Baseline covariates here will be as follows: baseline 9-hole peg test, geographical region (United States vs. ROW), and baseline EDSS score ($<$4.0 vs. $\geq$4.0)

#### 4.4.3.9 Change in Fatigue, as Measured by the Modified Fatigue Impact Scale Total Score from Baseline to Week 96

The Modified Fatigue Impact Scale (MFIS) measures fatigue effects in terms of physical, cognitive, and psychological impact. A higher score indicates greater impact of fatigue on a patient's activities.

- The Physical Subscale is the sum of items 4, 6, 7, 10, 13, 14, 17, 20, and 21 on the MFIS. Range is 0–36.
- The Cognitive Subscale is the sum of items 1, 2, 3, 5, 11, 12, 15, 16, 18, and 19. Range is 0–40.
- The Psychosocial Subscale is the sum of items 8 and 9. Range is 0–8.
- The Total MFIS score is the sum of all items. Range is 0–84.

Subscale scores will not be computed for patients with missing items on the subscale. Per the developer of the questionnaire, the Total MFIS score may be computed if up to two items are missing by first dividing the total score by the number of items answered and then multiplying by the number of total items in the scale. If more than two items are missing, the form will be set to missing.

The MFIS total score will be summarized at baseline and Weeks 48 and 96 using descriptive statistics for each treatment group. Data from other unscheduled assessments will not be included in this summary or analysis. The change from baseline to Weeks 48 and 96 in the MFIS total score will be computed and summarized using descriptive statistics for each treatment group. The MFIS subscale scores will only be listed.

For the assessment of differences in the mean change in MFIS total during the 96-week comparative treatment period from baseline up to Week 96, MMRM analyses will be undertaken using the same approach as described above in Section 4.4.2.9, except baseline covariates here will be as follows: baseline total MFIS score, geographical region, and baseline EDSS score ($<$4.0 vs. $\geq$4.0).

#### 4.4.3.10 Change in Patient-Reported Depressive Symptoms, as Measured by the Center for Epidemiologic Studies Depression Scale, from Baseline to Week 96

The CES-D is a 20-item self-report questionnaire measuring depressive symptomology. It includes mood, well-being, somatic, and interpersonal domains.

Items are scored using the following table:

**Table 3 Center for Epidemiologic Studies Depression Scale**

| CES-D Items | Rarely or none of the time | Some or a little of the time | Occasionally or a moderate amount of time | Most or all of the time |
|---|---|---|---|---|
| 4, 8, 12, and 16 | 3 | 2 | 1 | 0 |
| All other items | 0 | 1 | 2 | 3 |

CES-D = Center for Epidemiologic Studies Depression Scale.

A total CES-D score is computed by summing the scored items. The total score ranges 0–60. If four or fewer items on a patient's questionnaire are missing, the computational mean is used for imputation. If more than four items are missing, the form is set to missing.

The total CES-D score will be summarized at baseline and Weeks 48 and 96 using descriptive statistics for each treatment group. Data from other unscheduled assessments will not be included in this summary or analysis. The change from baseline to Weeks 48 and 96 in total CES-D score will be computed and summarized using descriptive statistics for each treatment group.

For the assessment of differences in the mean change in total CES-D score during the 96-week comparative treatment period from baseline up to Week 96, an MMRM analysis will be undertaken using the same approach as described in Section 4.4.2.9, except baseline covariates here will be as follows: baseline total CES-D score, geographical region (United States vs. ROW), and baseline EDSS score ($<4.0$ vs. $\geq 4.0$).

#### 4.4.3.11 Change in Karnofsky Performance Status Score from Baseline to Week 96

The Karnofsky Performance Scale allows for the classification of patients according to their functional impairment. This scale is usually used to compare effectiveness of different therapies and to assess the prognosis in individual patients. The Karnofsky score ranges 100–0, where 100 is "perfect" health (normal, no complaints, or no evidence of disease) and 0 is death. Scores at 10-point intervals between 100 and 0 are assigned to represent health status.

The Karnofsky Performance Status Score will be summarized at baseline and Weeks 24, 48, 72, and 96 using descriptive statistics for each treatment group. Data from other unscheduled assessments will not be included in this summary or analysis. The change from baseline to Weeks 24, 48, and 96 in Karnofsky Performance Status Score will be computed and summarized using descriptive statistics for each treatment group (i.e., frequency counts or summary statistics, as appropriate).

For the assessment of differences in the mean change in Karnofsky Performance Status Score during the 96-week comparative treatment period from baseline up to Week 96, an MMRM analysis will be undertaken using the same approach as described in Section 4.4.2.9, except baseline covariates here will be as follows: baseline Karnofsky Performance Status Score, geographical region (United States vs. ROW), and baseline EDSS score ($<$4.0 vs. $\geq$4.0).

#### 4.4.3.12 Percentage Change in Cortical Grey Matter Volume as Detected by Brain MRI from Baseline to Week 96

Cortical grey matter volume as assessed on MRI scans of the brain will be summarized at baseline and Weeks 24, 48, and 96 using descriptive statistics for each treatment group. Data from other unscheduled assessments will not be included in this summary or analysis. The percentage change from baseline to Weeks 24, 48, and 96 in cortical grey matter volume on MRI scans of the brain will be computed and summarized using descriptive statistics for each treatment group.

For the assessment of differences in the mean percentage change in cortical grey matter volume on MRI scans during the 96-week comparative treatment period from baseline up to Week 96, an MMRM analysis will be undertaken using the same approach as described in Section 4.4.2.9, except baseline covariates here will be as follows: baseline cortical grey matter volume, geographical region (United States vs. ROW), and baseline EDSS score ($<$4.0 vs. $\geq$4.0).

#### 4.4.3.13 Percentage Change in White Matter Volume as Detected by Brain MRI from Baseline to Week 96

White matter volume as assessed on MRI scans of the brain will be summarized at baseline and Weeks 24, 48, and 96 using descriptive statistics for each treatment group. Data from other unscheduled assessments will not be included in this summary or analysis. The percentage change from baseline to Weeks 24, 48, and 96 in white matter volume on MRI scans of the brain will be computed and summarized using descriptive statistics for each treatment group.

For the assessment of differences in the mean percentage change in white matter volume on MRI scans during the 96-week comparative treatment period from baseline up to Week 96, an MMRM analysis will be undertaken using the same approach as described above in Section 4.4.2.9, except baseline covariates here will be as follows: baseline white matter volume, geographical region (United States vs. ROW), and baseline EDSS score ($<$4.0 vs. $\geq$4.0).

#### 4.4.3.14 Proportion of Patients Who Have Disability Improvement Sustained for At Least 24 Weeks with the Initial Event of Neurological Improvement Occurring during the 96-Week, Double-Blind, Double-Dummy Treatment Period

This endpoint is the same as the 12-week confirmed disability improvement endpoint (refer to Section 4.4.2.6), but based instead on a 24-week period of disability improvement.

The same approach to analysis as described in Section 4.4.2.6 will be applied to the 24-week confirmed disability improvement endpoint.

#### 4.4.3.15 Proportion of Patients Who have Disability Improvement Sustained for at Least 12 Weeks and Sustained until the End of the 96-Week, Double-Blind, Double-Dummy Treatment Period, with the Initial Event of Neurological Improvement Occurring during the 96-Week, Double-Blind, Double-Dummy Treatment Period

This endpoint is similar to the 12-week confirmed disability improvement endpoint (see Section 4.4.2.6), but it is required that improvement is sustained until the end of the 96-week double-blind double-dummy treatment period. Otherwise, patients will be classified as not improved.

The same approach to analysis as described in Section 4.4.2.6 will be applied here.

#### 4.4.3.16 Duration of Confirmed Disability Improvement

This analysis will be performed for the patients with a Confirmed Disability Improvement for 12 weeks identified as in Section 4.4.2.6.

The duration of the Confirmed Disability Improvement is the time from the date of onset of the event, until the first visit where the EDSS score no longer fulfills the criteria for disability improvement. For the patients who have a sustained improvement until after the end of the double-blind treatment period, the duration of the confirmed disability improvement will be censored at the last EDSS score measured during the 96week, double-blind, double-dummy treatment period. For patients with the onset of the CDI at the last visit during this period and confirmed in the open-label or safety follow-up phase, the duration of the CDI will be censored at Day 1. The Kaplan-Meier estimates of the duration of CDI will be calculated. No comparison between the randomized arms will be made, since this analysis is performed only for the patients with a CDI.

#### 4.4.3.17 Proportion of Patients Who at Week 96 Have Improved, Stable or Worsened Disability, Compared with Baseline

Stable disability is defined as a difference in EDSS score of no greater than $\pm 0.5$ comparing the Week 96 and baseline scores. Worsened disability is defined as an increase in EDSS score of $> 0.5$ at Week 96 compared to baseline. Improved disability is defined as a reduction in EDSS score of $> 0.5$ at Week 96 compared with baseline. The proportion of patients with improved, stable, or worsened disability compared with

baseline in each treatment group will be reported. In addition, the proportion of patients with improved or stable disability (combining the proportion or patients improved and the proportion stable) in each treatment group will be reported.

Patients with missing EDSS data at baseline or Week 96 are excluded from this analysis. Interval EDSS scores are not taken into account in this analysis.

The proportions with improved, stable, and worsened disability within treatment groups will be analyzed using multinomial logistic regression with baseline covariates, geographical region (United States vs. ROW), and baseline EDSS score ($<$4.0 vs. $\geq$4.0).

#### 4.4.3.18 Change in EDSS Score from Baseline to Week 96

The EDSS scores will be summarized at baseline (without rounding) and Weeks 48 and 96 using descriptive statistics for each treatment group. Data from other unscheduled assessments will not be included in this summary or analysis. The change from baseline to Weeks 48 and 96 in EDSS scores will be computed and summarized using descriptive statistics for each treatment group.

For the assessment of differences in the mean change in EDSS score during the 96-week comparative treatment period from baseline up to Week 96, MMRM analyses will be undertaken using the same approach as described above in Section 4.4.2.9, except baseline covariates here will be as follows: geographical region (United States vs. ROW) and baseline EDSS (continuous without rounding).

Graphical presentations for least square means and 95% confidence intervals will be used to illustrate trends over time.

#### 4.4.3.19 Change in Quality of Life, as Measured by the Short Form 36 Version 2 Mental Component Summary Score from Baseline to Week 96

The derivation of the SF-36 MCS Score was described previously in Section 4.4.2.11.

The MCS Score will be summarized and analyzed in exactly the same way as the SF-36 PCS Score, as described in Section 4.4.2.11.

### 4.4.4 Subgroup Analyses

The primary and some secondary efficacy endpoints (12 and 24 week CDP) will be summarized and analyzed by the following subgroups:

- Demography: age ($\geq$40 vs. $<$40 years)
- Demography: sex (male vs. female)
- Demography: race (White vs. Other)
- Body weight: $\geq$75 kg versus $<$75 kg
- Stratification factor: geographical region (United States vs. ROW)

- Stratification factor: baseline EDSS score (< 4.0 vs. ≥ 4.0)
- Previous lesions: baseline Gd-enhancing lesion (0 vs. > 0)

Estimates of treatment effect and associated 95% CIs will be presented in forest plots. Unadjusted p-values will also be presented for these analyses.

#### 4.4.4.1 Efficacy Data from the Safety Follow-Up Phase

Exploratory analyses of efficacy data collected in the safety follow-up phase are not envisioned.

#### 4.4.4.2 Efficacy Data from the Open-Label Extension Phase

Exploratory analyses of efficacy data collected in the open-label extension phase are envisioned. Additional analysis will be undertaken after efficacy data are more mature.

## 4.5 PHARMACOKINETIC AND PHARMACODYNAMIC ANALYSES

### 4.5.1 Pharmacokinetic Analyses

Ocrelizumab serum concentration–time data will be analyzed using a population approach. The primary population PK parameters (clearances and volumes) for ocrelizumab will be estimated by means of a non-linear mixed effect model fitted to the PK data collected in studies WA21092, WA21093, and WA21493. Clearances with associated inter-patient variability may be characterized by a saturable and non-saturable clearance as well as an intercompartmental clearance depending on the final structural model. Volumes with associated inter-patient variability may be characterized by central and peripheral volumes depending on the final structural model. Exposure (area under the concentration–time curve [AUC]) to ocrelizumab will be estimated. The selection of other parameters will depend on the final PK model used for this analysis.

Patients who have measurable concentrations of ocrelizumab will be included in the PK analysis unless major protocol deviations or unavailability of information (e.g., exact blood sampling time) occurred that may interfere with PK evaluation. The influence of covariates, such as age, gender, weight, ADA, and baseline CD19+ cell count on these individual PK parameter estimates will be investigated.

Details will be described in a Modeling and Simulation Analysis Plan, and results will be reported separately from the main CSR.

### 4.5.2 Pharmacodynamic Analyses

The correlation between individual ocrelizumab exposure and selected safety and efficacy parameters will be explored in order to characterize the exposure and /or dose-response relationship. This may include but is not limited to CD19+ cell count, ARR, infusion-related reactions (IRRs), serious infections, and other safety or efficacy parameters if of interest. Any such analyses will be reported separately in the PK report.

## 4.6 SAFETY ANALYSES

Safety data include but are not restricted to adverse event data, laboratory data, previous and concomitant treatment data, infusion information including IRR data, withdrawal data, fatalities, ECG, vital signs, ADA data, and dosing information.

This SAP describes the analyses of all available safety data collected up to the clinical cutoff date (study CCOD). The safety data will be summarized descriptively for the following periods:

(1) the controlled treatment period, defined as the period until completion of treatment or safety follow-up until 96 weeks after the first dose of study treatment (patient CCOD);

(2) double-blind period + OLE period (see Section 4.6.7 for details)

### 4.6.1 Exposure of Study Medication

This section deals with exposure in the context of safety analyses. In the context of efficacy analyses, exposure time is as defined above in Section 4.4.

Exposure of ocrelizumab/ocrelizumab placebo and interferon beta-1a 44 μg SC / interferon beta-1a 44 μg SC placebo over the course of the study will be summarized using descriptive statistics.

"Duration of Observation" and "Duration of Exposure" are used interchangeably within this SAP.

Definition of the Dose: A Dose of ocrelizumab can be given as one infusion or two infusions administered 2 weeks apart.

Patients will be considered to have received a Dose of treatment if at least part of one infusion of that Dose (either Day 1 or Day 15 for dual infusions) was given. If a Dose is completely missed instead of delayed, the next Dose number will be based on the number of previous doses received.

The duration of observation for a patient will be calculated as:

(Date of last contact* − date of first Dose) + 1

* Earliest of 1) date of CCOD for the reporting event, 2) date of last contact from the study completion page, or 3) date of death.

For the controlled treatment period, the CCOD will be variable for each patient at 96 weeks from treatment.

For patients who are receiving interferon beta-1a 44 μg SC and who switch to active ocrelizumab treatment, exposure of interferon beta-1a 44 μg SC will end on the study day prior to their first active Dose of ocrelizumab.

The duration of observation, within a Dose is defined in a similar manner as:

$$(\text{Day prior to first infusion in } n+1^{th} \text{ Dose}^* - \text{date of first infusion in } n^{th} \text{ Dose}) + 1$$

*With the exception of the last Dose received by the patient where date of last contact is used as defined above.

Exposure to ocrelizumab and exposure to interferon beta-1a 44 μg SC during the controlled treatment phase will be summarized. For ocrelizumab, the treatment duration, number of infusions received, and the total cumulative dose (derived to mg) will be summarized by study treatment arm. An additional summary will be presented, summarizing at each infusion the percentage of patients who received $<80\%$ and $\geq 80\%$ of the planned infusion. The number and percentage of infused patients who were pre-treated with steroids will be summarized. For interferon beta-1a 44 μg SC ,the number of Doses and dose intensity (based on the number of syringes received rather than the actual Dose) will be presented by study treatment arm.

### 4.6.2 Columbia-Suicide Severity Rating Scale

The number and percentage of patients with suicidal ideation, suicidal behavior, and self-injurious behavior without suicidal intent at any time after the start of study treatment, based on the Columbia Suicide Severity Rating Scale (C-SSRS), will be presented. In addition, shift tables will be presented to show changes from baseline in C-SSRS categories. A listing of patients with suicidal ideation, suicidal behavior, or self-injurious behavior, based on the C-SSRS, will be presented.

### 4.6.3 Adverse Events

Adverse events will be defined as all adverse events including IRRs and serious MS relapses, but excluding non-serious MS relapses. Therefore, those adverse events recorded on the “adverse event” and “infusion-related reaction” CRF pages will be included.

For each adverse event recorded, the term entered by the investigator describing the event (the “reported term”) will be assigned a standardized term (the “preferred term” [PT]) based on the Medical Dictionary for Regulatory Activities (MedDRA) World Health Organization (WHO) dictionary of terms. All analyses of adverse event data will be performed using the PTs unless otherwise specified.

All adverse events will be mapped to PTs and super class terms. For all summary tables, the adverse events will be sorted by body system (in decreasing order of overall incidence) and then by PT (in decreasing order of overall incidence). Additionally, the most frequent adverse events ($\geq$5% in any treatment arm) will be presented by PT.

Adverse events will be assigned to a Dose if the adverse event onset date is on or after the date of the first infusion of that Dose, but before the first infusion of the next treatment Dose. Adverse events that are reported during the safety follow-up period or B-cell monitoring period will be assigned to the last Dose received. Hence the last Dose for a patient may be of a variable length from 24 to 72 weeks or longer. Adverse events that start prior to the first Dose and worsen during treatment (i.e. treatment emergent) will be assigned to the first treatment Dose.

All summaries and listings of adverse events will be based on the safety population unless otherwise stated. Summaries of adverse events will be generated by summarizing the incidence of treatment-emergent adverse events only. Treatment-emergent events are defined as those adverse events with observed or imputed onset date on or after the start date of study treatment. Only where the most extreme intensity is greater than the initial intensity will events with an onset date prior to the start of study treatment (and with an end date on or after the start of study treatment) be considered treatment emergent. An adverse event with a completely missing non-imputed start date will be assumed to be treatment emergent unless the adverse event has a complete non-imputed end date that is prior to start of study treatment.

For each treatment group, the incidence count for each adverse event PT will be defined as the number of patients reporting at least one treatment emergent occurrence of the event. The incidence rate will be calculated as the incidence count divided by the total number of patients in the population. Each table will also present the overall number of patients experiencing at least one adverse event and the total number of adverse events reported (multiple occurrences of the same adverse event in 1 patient will be counted only once).

The number of patients who experienced a related adverse event will be summarized by system organ class (SOC) and PT.Adverse events will be summarized by SOC and PT by intensity grade. Multiple occurrences of the same event within a patient will be counted once at the greatest intensity/highest grade for this PT. For adverse events leading to death, the most extreme intensity will be overwritten by Grade 5 (death). Any adverse events and the SOC overall rows of the summary table will count patients according to adverse events by intensity/grades.

All patient deaths, regardless of treatment received, will be listed.

Serious adverse events will be defined as all serious adverse events including serious MS relapses and serious IRRs. The number of patients who experienced a serious adverse event will be summarized by SOC and PT. Related serious adverse events will be summarized by SOC and PT. Additionally, the most frequent serious adverse events ($\geq$1%) will be presented by PT.

A separate listing with relapses considered as serious adverse events will be presented.

A patient may experience an adverse event that leads to the discontinuation of their study treatment. Discontinuation of study treatment for an adverse event may not necessarily lead to discontinuation from the study because patients can enter the safety follow-up periods of the protocol. Only adverse events that led to the discontinuation of study treatment are of interest. Patients who withdraw early from the study because of adverse events will be summarized under disposition. Because of the double-dummy design, the study-drug regimen consists of more than one combination of two treatments (ocrelizumab and interferon beta-1a 44 μg SC). An analysis of adverse events leading to study drug discontinuation will not be performed for the individual treatments of the regimen within one study arm, because the investigators were instructed to discontinue both treatments. The number of patients who experienced an adverse event that led to discontinuation of study treatment will be summarized by SOC and PT.

The number of patients who experienced an adverse event that led to modification or interruption of study drug will be summarized by SOC and PT.

#### 4.6.3.1 Selected Adverse Events

**Infection**

Infections will be defined from the adverse event data using the MedDRA SOC of "Infections and Infestations." Adverse events that are reported as an infection by the investigator will also be included.

An infection will be defined as serious if the event is a serious adverse event or if the non-serious infection was treated with an IV anti-infective.

The number of infections and serious infections per 100 patient-years will be calculated and summarized separately. The ninety-five percent CI of the incidence rates will be calculated using exact method based on Poisson distribution.

Summary of infections will be presented by SOC and PT, severity, and Dose. Similar summary tables will be also produced by serious infections.

Summary tables of infections and serious infections by pathogen information codes as well as listing of infections/serious infections with pathogen codes will be produced.

**Infusion-Related Reactions**

An IRR and its corresponding symptoms are collected on the dedicated eCRF.

The symptom(s) of an IRR and the IRR itself may be of different intensities. As other symptoms can be recorded as free-text on the eCRF page, symptoms will be coded in the MedDRA and summarized by PTs.

IRRs are categorized by the time of the event occurring (1) during the infusion, (2) after completion of the infusion while patient is in the clinic, and (3) within 24 hours of completion of the infusion and patient is not in the clinic. The number and percentage of

patients with at least one IRR will be presented by infusion (patients with multiple events within an infusion will count only once). In addition, the total number of IRRs will be summarized (multiple events will be counted). The total and percentage (based on the total number of patients with at least one IRR) will be summarized by most extreme intensity.

IRRs will be summarized by intensity and by infusion. For multiple events within a patient, the most extreme intensity will be taken.

Symptoms of IRR and symptoms of serious IRR will also be presented by infusion. This analysis will be produced for both the controlled treatment period and the OLE period.

The number of patients with at least one IRR, the total number of IRRs, and the number of IRRs will be summarized by the time of events.

### Pregnancies

Any pregnancies that occur during the study will be listed.

### 4.6.4 Magnetic Resonance Imaging Data

Non-MS pathology that is reported by the local safety radiologist will be listed by treatment group.

### 4.6.5 Laboratory Data

### General laboratory evaluation

All laboratory assessments will be summarized or listed when applicable.

Absolute value and change from baseline values at each visit will be summarized. The baseline value will be the last value prior to the first dose of study medication. When laboratory data are presented over time, these laboratory values will be time-windowed into a common visit structure. If multiple values of the same laboratory parameter occur within the same time window, the worst value for that parameter will be presented in the summary table.

A summary of the number and percentage of patients with abnormal laboratory outcomes will be produced by treatment group for each parameter.

In addition, for the liver laboratory parameters, the number and percentage of patients with an elevated post-baseline AST or ALT level during the 96-week controlled treatment period will be summarized by treatment.

### CD19

The median CD19 cell count will be displayed graphically over time from the first infusion of study medication. The absolute CD19 count and change from baseline in the CD19 count will be summarized over time. At each timepoint, the number and percentage of patients whose CD19 counts have repleted will be presented. Repletion is defined as

the CD19 count having returned to their baseline value or lower limit of normal (≥80 cells/μL), whichever is lowest.

### Immunoglobulins

The mean immunoglobulin levels (IgG, IgM, and total Ig) will be displayed graphically over time from the first infusion of study medication. The absolute values and change from baseline will be summarized over time. At each timepoint, the number and percentage of patients with immunoglobulin levels lower than the lower limit of normal will be presented.

### Anti-Drug Antibodies

Anti-drug antibodies (ADAs), also called human anti-human antibodies (HAHAs), will be summarized over the 96-week controlled treatment period by treatment group. The baseline prevalence and post-baseline incidence of ADAs will be displayed. The number of patients with treatment-induced ADA will also be displayed. A table will be presented that summarizes ocrelizumab serum concentrations (μg/mL) at timepoints where ADA samples were collected and analyzed. A listing by treatment of anti-ocrelizumab antibody data will be presented for patients with at least one ADA sampled datum.

### Antibody Titers

If data are available, antibody titers for mumps, rubella, varicella, and *Streptococcus pneumoniae* will be summarized for number and percentages of patients with positive level.

## 4.6.6 Vital Signs

Vital signs, physical examination, and ECG results will be presented in patient listings. Change from study baseline in vital signs at each visit will be summarized by treatment group. Changes in vital signs from baseline to post-infusion timepoints will also be summarized for each infusion.

## 4.6.7 Safety by Treatment and Period

To explore safety by treatment and period, all of the available safety data collected up to study CCOD will be presented by treatment regimen and by period (i.e. double-blind period and OLE period).

For the double-blind period, the interferon beta-1a 44 μg SC and ocrelizumab treatment groups will present all safety data prior to the first active dose of ocrelizumab in OLE, including the SFU data post-Week 96 until the start of OLE and SFU data post-Week 96 for patients who withdrew during the double-blind period. For the OLE period, the interferon beta-1a 44 μg SC /OLE ocrelizumab or ocrelizumab/OLE ocrelizumab treatment groups will present all data collected after the first dose of OLE ocrelizumab, including all SFU data for patients who withdraw during the OLE.

The adverse event profile will be produced by treatment regimen for each period separately. The number of events per 100 patient-years will be presented to account for differing amounts of exposure.

Adverse events and serious adverse events will be presented by SOC and PTs for each period separately. Also, infection and serious infection will be presented by SOC and PTs for each period separately.

#### 4.6.8 Type I Interferon-Neutralizing Antibody Data

Type I interferon-neutralizing antibody data will be summarized in listings and additional analysis will be performed as appropriate.

### 4.7 MISSING DATA

All methods for handling missing data and associated sensitivity analyses are described above, section by section, for each endpoint.

### 4.8 INTERIM ANALYSES

No interim efficacy analyses are planned.

## 5. REFERENCES

Barkhof F, Calabresi PA, Miller DH, et al. Imaging outcomes for neuroprotection and repair in multiple sclerosis trials. Nat Rev Neurol 2009; 5:256–266.

Bohannon RW. Comfortable and maximum walking speed of adults aged 20–79 years: reference values and determinants. Age Ageing 1997;26:15–19.

Carpenter JR, Kenward MG. Missing data in randomised controlled trials: A practical guide. 2007. Final report available at http://missingdata.lshtm.ac.uk/downloads/rm04_jh17_mk.pdf.

Oxford Grice KA, Vogel KA, Le V, et al. Adult Norms for a Commercially Available Nine Hole Peg Test for Finger Dexterity. Am J Occup Ther 2003;57:570–573.

# Appendix 1
# Standard Operating Procedure “EDSS Assessment Check for the Roche Trials WA25046, WA21092, and WA21093”



Standard Operating Procedure (SOP)

| | | | |
|---|---|---|---|
| EDSS assessment check for the<br>Roche trials WA25046, WA21092 and WA21093<br>SD02 | | | |
| Attachments<br>Curriculum vitae of the USB Experts<br>Contract of the USB Experts in copy<br>Conflicts of interests of USB Experts | | | |
| Version: 1.0 | | | |
| Target group: Roche, Neurostatus Systems GmbH, USB EDSS Expert Team | | | |
| Function | Name, Company | Date | Signature |
| Author | Oezguer Yaldizli, USB | | |
| Approved | Prof. David Leppert, GDTL, Roche | | |
| Approved | Prof. Ludwig Kappos, USB Chair of the Department of Neurology | | |
| Approved | Franz Schnyder Neurostatus Systems GmbH | | |
| Approved | Pia Steinger Quality, USB | | |

# Appendix 1
# Standard Operating Procedure "EDSS Assessment Check for the Roche Trials WA25046, WA21092, and WA21093" (cont.)

**Contents**

1. Aim 2

2. Background 2

3. Processes 3

3.1. Flow chart 3

3.2. Process in written form 4

3.3 Handling of EDSS assessments exposed to previous process (dates)

4. Abbreviations 5

5. References 5

6. History of amendments 5

## 1. Aim

The aim of this SOP is to describe the process and the roles and responsibilities for the review of the Expanded Disability Status Scale (EDSS) assessments. In addition this SOP further outlines how to detect and handle inconsistent EDSS assessments and John Kurtzke's Functional Systems scores (FSS) in the Roche pivotal Multiple Sclerosis trials WA25046, WA21092 and WA21093 according to ICH guidelines.

## 2. Background

Currently, John Kurtzke's Functional Systems and the EDSS are the most widely accepted clinical outcome measures for the evaluation of neurological impairment and disability in Multiple Sclerosis (MS) clinical trials. The determination of the EDSS step is primarily based on the individual scores of the 7 Functional Systems including visual, brainstem, pyramidal, cerebellar, sensory, bowel and bladder, and cerebral, as well as on the walking range and the assistance needed to ambulate (Kurtzke JF et al. 1955; Kurtzke JF 1983). During the past years standardized training programs and certification of EDSS evaluating physician (EDSS assessor) have been developed and introduced to improve inter-rater and intra-rater reliability. For the Roche trials WA25046, WA21092 and WA21093 the EDSS step is assessed by trained neurologists who refer to the Neurostatus definitions in the booklet version of 04/10.2. Between Jan 1, 2011 and Jan 31, 2012 University Hospital Basel (USB) Experts reviewed 1082 EDSS assessments rated by 267 examining investigators at 160 study sites participating in the Roche trials WA25046, WA21092 and WA21093. They found in 23% of the cases inconsistencies

in the last step of the assessment, namely the combination of the Functional Systems and the ambulation scores to the final EDSS step.

## 3. Processes

### 3.1. Flow Chart on Query Resolution (Detail in 3.3)

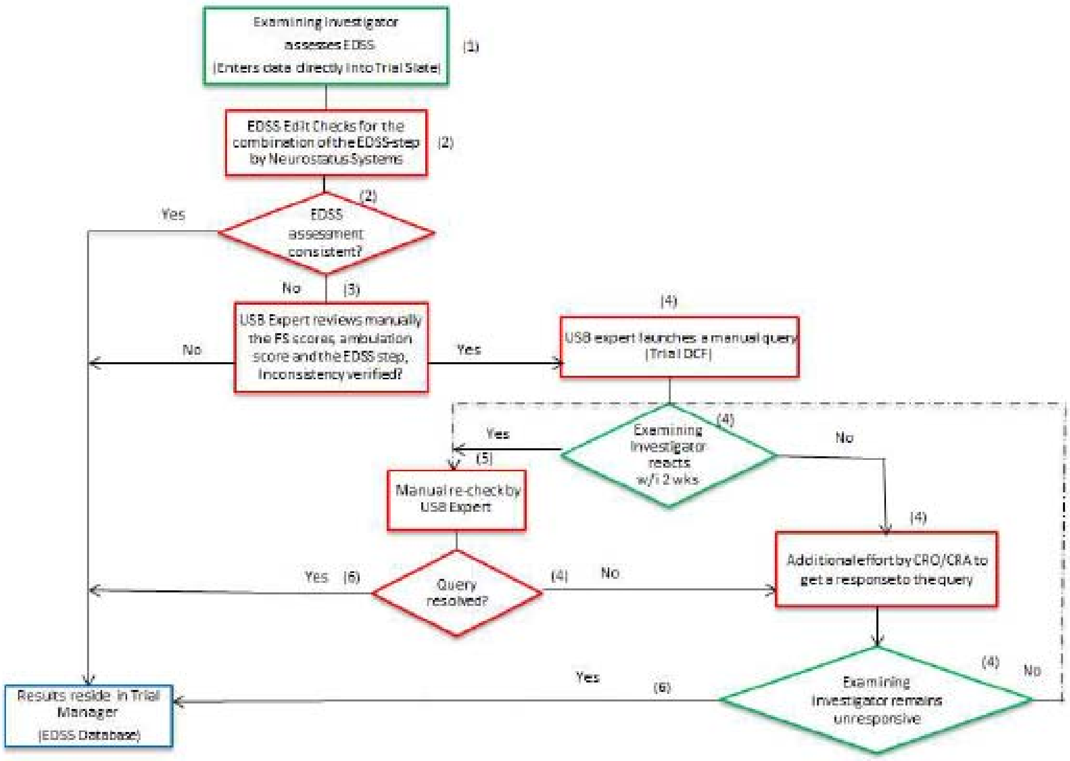


### 3.2. Roles & Responsibilities

| Responsible | Role |
|---|---|
| EDSS Evaluating Physician | Examining investigator / EDSS assessor who performs the neurological examination, documents the FS scores and assess EDSS steps. The examining Investigator will not be involved with any aspect of medical management of the patient and will not have access to patient data. |

| | |
|---|---|
| CRF Health (CRFHealth eCOA Solutions Company) | Provider of the TrialSlate and associated web portal (TrialManager). CRF Health is responsible for processing all data clarification forms (TrialDCFs) related to the EDSS assessment captured in the TrialManager. |
| Neurostatus Systems GmbH | Company responsible for the technical and administrative implementation of training and certification of physicians participating in projects using EDSS in multiple sclerosis.<br><br>Neurostatus Systems is responsible to check for plausibility and inconsistencies of the EDSS assessments. |
| USB Expert | USB Experts are medical doctors working at the Department of Neurology of the USB specialized in the assessments of the EDSS. For contracts see attachment. Prof Kappos has the oversight of the Expert Review process. He will appoint a named person as one of the USB Experts who will act as a single point of contact for Roche. |
| | USB expert are reviewing EDSS assessment of the scoring sheet.<br><br>The USB expert is responsible for the content of the TrialDCFs.<br><br>The USB expert is responsible for being available via email / telephone in order to respond to questions by the EDSS physician. |

| | |
|---|---|
| Monitor/CRA | Clinical Research Associate employed by a Clinical Research Organisation (CRO).<br><br>Monitor / CRA log into TrialManager to check for TrialDCFs and to notify sites that a response is required from the examining investigator. |
| Roche Study Management Team (SMT) | The Study Management Team (SMT) reviews unresolved TrialDCFs and decides to close or continue to follow up the outstanding TrialDCFs. |

3.3. Reporting and Review Process in written form

Step 1: EDSS assessment by the examining investigator at the study sites

Functional system score, ambulation score and EDSS step (EDSS assessment) are assessed by examining investigators based on a standardized neurological examination using the Neurostatus Scoring booklet, version 04/10.2. These data are captured by using TrialSlate (an electronic data capturing device). Range checks are performed during data entry onto the device. The data are then transferred via LAN or Mobile network to TrialManager web portal (the database).

Step 2: Automated Consistency Check

Neurostatus Systems checks the data on TrialManager for plausibility and inconsistencies of the EDSS using automated consistency checks. The rules for these checks are given in the Neurostatus Scoring booklet, version 04/10.2. A scoring sheet consisting of the results of the EDSS assessments is generated by Neurostatus and uploaded to TrialManager. The scoring sheet will flag inconsistencies in the EDSS assessment.

If the scoring sheet doesn't identify inconsistencies, the assessment remains unchanged in TrialManager.

Step 3: Manual consistency check

The USB expert will review the scoring sheet with the EDSS assessments within 2 working days from upload to TrialManager.

The EDSS assessments with flagged inconsistencies in the scoring sheet are manually reviewed by the USB Expert.

- If after review by the USB expert, the flagged EDSS assessment is determined to be consistent then it will remain on TrialManager unchanged.

- If after review by the USB expert, the flagged EDSS assessments are confirmed to be inconsistent a manual query (TrialDCF) will be generated in TrialManager. The query in TrialManager will be reviewed and responded to by the examining investigator (See Step 4)

Step 4: TrialDCF

The TrialDCF are queries described in the study manual query process.

- The USB expert is responsible for the content of the TrialDCF
- CRF Health is responsible for processing all TrialDCFs related to the EDSS assessment captured in TrialManager.

- The Monitor / CRA will log into TrialManager at least once per week for any new TrialDCFs. If new TrialDCFs have been issued, sites will be notified by the monitor/ CRA that a response to the TrialDCF is required by the examining investigator within two weeks via TrialManager.

  - If the examining investigator does not respond to the initial TrialDCF within the two weeks as stated above, the monitor/CRA will contact the examining investigator to request to resolve the outstanding TrialDCF as per the agreed monitoring plan.

  - The Examining investigator and/or monitor/CRA can ask for support by the USB expert via email or telephone to ask any question to enable resolution of the TrialDCF.

- Call service times are weekly for European countries on every Tuesday 3-4 p.m.
  CET, biweekly on Tuesdays 9-10 a.m. CET for Australia and Asia and 5-6 p.m. CET for the Americas. The content of these discussions during the calls will be documented and archived by the USB Expert.

  - Detailed information on call in numbers and email information will be provided by the USB expert.

  - All outstanding TrialDCFs will be followed up by the monitor/CRA as per the agreed monitoring plan. The Study Management Team (SMT) will review unresolved TrialDCFs (as defined in study Integrated Data Review Plan (iDRP)) and will decide to close or continue to follow up the outstanding TrialDCFs.

  - Any TrialDCFs that are not answered/resolved will have their status changed as defined by CRFHealth (following authorisation from the Roche SMT during the course of the study (see Step 6).

Step 5: Manual re-check

All answered TrialDCFs will be re-checked by the same USB Expert who issued the initial TrialDCF. TrialManger will generate a report to notify the USB expert of the response from examining investigator at the site and he/she will review the response.

Step 6: Resolution of TrialDCF in TrialManager

If the TrialDCFs is resolved, the EDSS assessment will be considered final and CRFHealth will implement and verify the change in TrialManger

The final status of a TrialDCF will be either

1) "Resolved with change and closed" – TrialDCF is answered and revised data are entered by the examining investigator into TrialManager

2) "Resolved without change and closed" – TrialDCF is answered by the Examining Investigator who confirms his original assessment in the TrialDCF.

3) "Unresolved and closed" – TrialDCF is not answered and the SMT authorises to close due to unresponsiveness

In all cases the examining investigator remains the final decision maker on the EDSS assessment.

3.4 Handling of EDSS assessments checked prior to the implementation of the process outlined in this SOP

All previous EDSS assessments, that had gone through the previous automated TrialDCF process, whether they had been changed or not as a result of this process will go through this new revised Neurostatus Systems edit check process again and the process steps outlined in this current SOP 2-6 will be applied. Automated and new TrialDCF data will be stored in TrialManager.

4. Glossary & Definitions

| | |
|---|---|
| ICH GCP guideline | International Conference on Harmonisation good clinical practice guidelines |
| CRF Health | CRFHealth eCOA Solutions Company |
| CRA | Clinical research associate |
| CRO | Contract research organisation |

| | |
|---|---|
| EDSS | Expanded Disability Status Scale |
| FSS | Functional system score |
| iDRP | Integrated Data Review Plan |
| MS | Multiple sclerosis |
| Neurostatus Systems GmbH | Company responsible for the technical and administrative implementation of training and certification of physicians participating in projects using EDSS in MS. |
| Principal Investigator | Lead site investigator |
| SMT | Study Management Team |
| TrialSlate | An electronic data capture device provided by CRFHealth |
| Trial DCF | Trial data clarification form (this resides in TrialManager) |
| TrialManager | Webportal Database which collects all questionnaires completed on the trialslate including EDSS assessments of the study patients |
| USB | University Hospital Basel |
| USB expert | Medical doctor who works at the University Hospital Basel (USB), specialized in the assessment of the EDSS and responsible for the quality control of inconsistencies, queries, teaching of the EDSS and certification process. |

5. References


Kurtzke JF. A new scale for evaluating disability in multiple sclerosis. Neurology 1955;5:580-583.

Kurtzke JF. Rating neurologic impairment in multiple sclerosis: an expanded disability status scale (EDSS). Neurology 1983;33(11):1444-1452.

Neurostatus, Version 04/10.2. www.Neurostatus.net

# STATISTICAL ANALYSIS PLAN

## POOLED ANALYSIS OF STUDIES WA21092 AND WA21093

| | |
|---|---|
| **TITLE:** | **A RANDOMIZED, DOUBLE-BLIND, DOUBLE-DUMMY, PARALLEL-GROUP STUDY TO EVALUATE THE EFFICACY AND SAFETY OF OCRELIZUMAB IN COMPARISON TO INTERFERON BETA-1A (REBIF®) IN PATIENTS WITH RELAPSING MULTIPLE SCLEROSIS** |
| **PROTOCOL NUMBERS:** | **WA21092 and WA21093** |
| **STUDY DRUG:** | **Ocrelizumab (RO4964913)** |
| **VERSION NUMBER:** | **1** |
| **IND NUMBER:** | **100,593** |
| **EUDRACT NUMBERS:** | **2010-020337-99 and 2010-020315-36** |
| **SPONSOR:** | F. Hoffmann-La Roche Ltd |
| **PLAN PREPARED BY:** | Gaelle Klingelschmitt |
| **DATE FINAL:** | See electronic date stamp below. |

### STATISTICAL ANALYSIS PLAN APPROVAL

| Name | Reason for Signing | Date and Time (UTC) |
|---|---|---|
| Burger,Hans_Ulrich | Company Signatory | 03-Sep-2014 10:13:23 |

**CONFIDENTIAL**

This is an F. Hoffmann-La Roche Ltd document that contains confidential information. Nothing herein is to be disclosed without written consent from F. Hoffmann-La Roche Ltd.

## TABLE OF CONTENTS

1. BACKGROUND .......... 4

2. WA21092/WA21093 STUDY DESIGN .......... 4

2.1 Protocol Synopsis .......... 4

2.2 Outcome Measures .......... 4

2.2.1 Primary Efficacy Outcome Measures .......... 4

2.2.2 Secondary Efficacy Outcome Measures .......... 4

2.2.3 Exploratory Efficacy Outcome Measures .......... 5

2.2.4 Pharmacokinetic Analysis: Objectives and Outcome Measures .......... 5

2.2.5 Pharmacodynamic Outcome Measures .......... 5

2.2.6 Safety Outcome Measures .......... 5

2.3 Determination of Sample Size .......... 6

2.4 Analysis Timing .......... 6

3. STUDY CONDUCT .......... 6

4. STATISTICAL METHODS .......... 6

4.1 Analysis Populations .......... 6

4.2 Analysis of Study Conduct .......... 6

4.3 Analysis of Treatment Group Comparability .......... 6

4.4 Efficacy Analysis .......... 7

4.4.1 Primary Efficacy Endpoint, Annualized Protocol-Defined Relapse Rate by 2 Years (96 Weeks) .......... 7

4.4.2 Secondary Efficacy Endpoints .......... 9

4.4.2.1 Hierarchy of and Statistical Testing Strategy for Secondary Efficacy Endpoints .......... 9

4.4.2.2 Time to Onset of Confirmed Disability Progression for at Least 12 Weeks with the Initial Event of Neurological Worsening Occurring during the 96-Week, Double-Blind, Double-Dummy Treatment Period .......... 10

4.4.2.3 Total Number of T1 Gadolinium– Enhancing Lesions as Detected by Brain Magnetic Resonance Imaging at Weeks 24, 48, and 96 .......... 11

4.4.2.4 Total Number of New and/or Enlarging T2 Hyperintense Lesions as Detected by Brain Magnetic Resonance Imaging at Week 24, 48, and 96 ..... 11
4.4.2.5 Proportion of Patients Who Have Confirmed Disability Improvement for at Least 12 Weeks with the Initial Event of Neurological Improvement Occurring during the 96-week Double-Blind, Double-Dummy Treatment Period ..... 11
4.4.2.6 Time to Onset of Confirmed Disability Progression for at Least 24 Weeks with the Initial Event of Neurological Worsening Occurring during the 96-Week Double-Blind, Double-Dummy Treatment Period ..... 11
4.4.2.7 Total Number of T1-Hypo-Intense Lesions (Chronic Black Holes) at Weeks 24, 48, and 96 ..... 12
4.4.2.8 Change in Multiple Sclerosis Functional Composite Scale Score from Baseline to Week 96 ..... 12
4.4.2.9 Percentage Change in Brain Volume as Detected by Brain Magnetic Resonance Imaging Scan from Week 24 to Week 96 ..... 12
4.4.2.10 Change in Quality of Life, as Measured by the Short Form 36 Version 2 Physical Component Summary Score from Baseline to Week 96 ..... 12
4.4.2.11 Proportion of Patients Who Have No Evidence of Disease Activity by Week 96 ..... 12
4.4.3 Exploratory Efficacy Endpoints ..... 13
4.4.4 Subgroup Analyses ..... 13
4.4.5 Efficacy Data from the Safety Follow-Up Phase ..... 14
4.4.6 Efficacy Data from the Open-Label Extension Phase ..... 14
4.5 Pharmacokinetic and Pharmacodynamic Analyses ..... 15
4.5.1 Pharmacokinetic Analyses ..... 15
4.5.2 Pharmacodynamic Analyses ..... 15
4.6 Safety Analyses ..... 15
4.7 Missing Data ..... 15
4.8 Interim Analyses ..... 15

# 1. BACKGROUND

Studies WA21092 and WA21093 are two Phase III trials with identical study design. There is a separate statistical analysis plan (SAP) describing the analyses to be performed for each study independently. This "pooled" SAP describes solely those analyses that will be performed on the two studies combined. The same statistical methods will be used for the pooled and individual study analyses, but some additional considerations are required for the pooled analyses. For example, the study name is added as a stratification factor or covariate in some analyses. The analysis of some secondary efficacy endpoints, Confirmed Disability Progression and Confirmed Disability Improvement, cannot be tested in a confirmatory manner in the individual trials and will necessitate the pooling of both trials in order to have sufficient statistical power to detect relevant treatment differences, as mentioned in the protocol. The analysis of the primary efficacy endpoint, secondary endpoints, and safety analyses are done in the pooled dataset to summarize the safety and efficacy of ocrelizumab in RMS collected in WA21092 and WA21093.

Further background details for studies WA21092 and WA21093 can be found in the study-level SAP.

# 2. WA21092/WA21093 STUDY DESIGN

Refer to the individual study-level SAP, or the individual study protocols, for full details on study design and assessments for Studies WA21092 and WA21093.

## 2.1 PROTOCOL SYNOPSIS

For the protocol synopsis and Schedule of Assessments, refer to the individual study protocols.

## 2.2 OUTCOME MEASURES

### 2.2.1 Primary Efficacy Outcome Measures

The primary efficacy endpoint is the annualized protocol-defined relapse rate at 2 years (96 weeks).

### 2.2.2 Secondary Efficacy Outcome Measures

All secondary efficacy endpoints (also called key efficacy endpoints) described in the study level SAP are also analyzed in the pooled data and are as follows:

- The time to onset of confirmed disability progression (CDP) for at least 12 weeks, with the initial event of neurological worsening occurring during the 96-week, double-blind, double-dummy treatment period
- The total number of T1 gadolinium (Gd)–enhancing lesions as detected by brain magnetic resonance imaging (MRI) at Weeks 24, 48, and 96
- The total number of new and/or enlarging T2 hyperintense lesions as detected by brain MRI at Weeks 24, 48, and 96

- The proportion of patients who have confirmed disability improvement (CDI) for at least 12 weeks, with the initial event of neurological improvement occurring during the 96-week, double-blind, double-dummy treatment period
- The time to onset of CDP for at least 24 weeks, with the initial event of neurological worsening occurring during the 96-week, double-blind, double-dummy treatment period
- The total number of T1-hypo-intense lesions (chronic black holes) at Weeks 24, 48, and 96
- The change in Multiple Sclerosis Functional Composite Scale (MSFCS) score from baseline to Week 96
- The percentage change in brain volume as detected by brain MRI from Week 24 to Week 96
- The change in Short Form 36 (SF-36) Physical Component Summary (PCS) Score from baseline to Week 96
- The proportion of patients who have no evidence of disease activity (NEDA) by Week 96

### 2.2.3 Exploratory Efficacy Outcome Measures

The exploratory efficacy endpoints analyzed at the individual study level will not be included in the pooled analysis of Studies WA21092 and WA21093, with the exception of the following three endpoints:

- The proportion of patients who have confirmed disability improvement for at least 24 weeks, with the initial event of neurological improvement occurring during the 96-week, double-blind, double-dummy treatment period
- The duration of the confirmed disability improvement (Kaplan Meier estimate)
- The proportion of patients who at Week 96 have improved, stable, or worsened disability, compared to baseline

### 2.2.4 Pharmacokinetic Analysis: Objectives and Outcome Measures

Refer to the individual study-level SAP for a description of the pharmacokinetic (PK) analysis.

### 2.2.5 Pharmacodynamic Outcome Measures

Refer to the individual study-level SAP and the Modeling and Simulation Plan for a description of the PD analysis of pooled data that will be presented in a separate report.

### 2.2.6 Safety Outcome Measures

The study-level SAP covers safety analyses in the areas of exposure to study medication, Columbia-Suicide Severity Rating Scale, adverse events, MRI data, ADA, laboratory data, and vital signs to be performed for each study individually.

## 2.3 DETERMINATION OF SAMPLE SIZE

The sample size for Studies WA21092 and WA21093 was determined in the same way for both studies. Further details of this sample size determination are provided in the study-level SAP and the protocols.

## 2.4 ANALYSIS TIMING

The primary pooled analysis of Studies WA21092 and WA21093 will be conducted immediately after the completion of the individual study analyses of Studies WA21092 and WA21093.

# 3. STUDY CONDUCT

For details of randomization, independent review facility, Expanded Disability Status Scale (EDSS) cleaning process, and data monitoring, refer to the individual study-level SAP.

# 4. STATISTICAL METHODS

## 4.1 ANALYSIS POPULATIONS

All analysis populations used in the pooled analysis are defined in the same way as the analyses of the individual studies. Details are provided in the individual study-level SAP.

## 4.2 ANALYSIS OF STUDY CONDUCT

The individual study-level SAP contains details of the data to be included in the individual study-level databases. The database used for the pooled analysis will simply be the consolidation of the individual databases for Studies WA21092 and WA21093.

No summaries or analyses will be performed at the pooled level to evaluate study conduct (i.e., those performed at the individual study level will be sufficient to evaluate study conduct).

## 4.3 ANALYSIS OF TREATMENT GROUP COMPARABILITY

The individual study-level SAP contains details of the summaries to be presented for individual Studies WA21092 and WA21093 in the following areas:

- Demography
- Baseline disease characteristics
- Multiple sclerosis (MS) disease history
- Non-MS disease history
- Prior treatments for MS
- Baseline MRI data (including previous MRI data collected at screening)

The summaries listed above will be presented for the pooled analysis of both studies in exactly the same way as for each individual study.

To assess the validity of pooling data across the two studies, the demographic and baseline characteristics will be visually compared for Studies WA21092 and WA21093 and for the pooled database of both studies.

## 4.4 EFFICACY ANALYSIS

Except where stated, statistical hypotheses for the primary and secondary endpoints and treatment comparisons will be tested at the 5% significance level ($\alpha = 0.05$) against two-sided alternatives.

For all assessments, the baseline value will be used as the last non-missing value on or before the date of the first infusion of study medication. See Section 4.4.1 for baseline EDSS and Section 4.4.2.2 for time to onset of confirmed disability progression and Section 4.4.2.5 for the proportion of patients with confirmed disability improvement.

All primary and secondary efficacy endpoints will be analyzed, stratified or adjusted by geographical region (United States vs. rest of world [ROW]) and baseline EDSS score ($<$4.0 vs. $\geq$4.0, stratification factor).

Unless otherwise specified, no sensitivity analyses will be performed as part of this pooled analysis of the efficacy endpoints; only the primary analysis for the primary efficacy endpoint and secondary endpoints will be performed (primary analysis in "Analysis Methods" part of Section 4.4.1 of the individual study-level SAP, and the first analysis of each secondary endpoint described in the various subsections in Section 4.4.2 of the individual study-level SAP).

In pooling Studies WA21092 and WA21093, it is important to assess whether the treatment difference between ocrelizumab and Rebif® is broadly consistent between the two studies. The poolability will be examined for the primary endpoint ARR and the first secondary endpoint 12 week CDP. See the Section 4.4.1 and Section 4.4.2.2, respectively.

If the primary endpoint (annualized protocol-defined relapse rate by 2 years) and the first secondary efficacy endpoint (time to onset of confirmed disability progression for at least 12 weeks with the initial event of neurological worsening occurring during the 96-week double-blind, double-dummy treatment period) for the intent-to-treat population can validly be pooled for the two studies, this will be taken as a general indication that it is similarly valid to pool the primary and all secondary efficacy endpoint analyses, and to undertake the subgroup analyses specified in Section 4.4.4 on the two studies pooled.

### 4.4.1 Primary Efficacy Endpoint, Annualized Protocol-Defined Relapse Rate by 2 Years (96 Weeks)

For the primary efficacy endpoint, the combined pooled analysis for Studies WA21092 and WA21093 will follow the same general approach described in the individual study-level SAP, with exceptions as noted below.

No sensitivity analyses will be performed as part of this pooled analysis of the primary efficacy endpoint; only the primary analysis for the primary efficacy endpoint will be performed (described in the "Analysis Methods" part of Section 4.4.1 of the individual study-level SAP).

The poolability assessment will be achieved via the inclusion in statistical models of an interaction term between study and treatment group in the analysis of the primary endpoint ARR.

Considering the interaction between study and treatment group, a quantitative interaction occurs when the difference between the two treatments is different between the two studies but both within-study treatment differences are in the same direction. That is, ocrelizumab shows a better response than Rebif® in both studies but to a differing extent, or alternatively, Rebif® shows a better response than ocrelizumab in both studies but to a differing extent. A qualitative interaction occurs when the difference between the two treatments is different between the two studies and these treatment differences are in opposite directions. That is, in one study, ocrelizumab shows a better response than Rebif® and in the other study, Rebif® shows a better response than ocrelizumab.

With a 10% significance level, if $p \leq 0.10$ for study-by-treatment group interaction and the interaction is qualitative rather than quantitative, it will be concluded that it is not appropriate to pool the two studies, because there is evidence of fundamentally different treatment effects in the two studies. In this situation, the study-by-treatment interaction term will be reported in the pooled clinical study report (CSR), but no statistical table will be presented since the pooled analysis is not considered valid. The two individual study-level analyses represent the only valid treatment comparisons for the primary efficacy endpoint, but if these results are in disagreement, interpretation of the treatment effect is problematic.

In all other cases (i.e., $p > 0.10$ for study-by-treatment group interaction, or $p \leq 0.10$ for study-by-treatment group interaction but the interaction is quantitative rather than qualitative), it will be concluded that it is valid to pool the two studies since there is no evidence of fundamentally different treatment effects in the two studies. In this situation, the study-by-treatment interaction term will be reported in the statistical table but then removed from the model and the resulting model (original individual study-level model plus study effect) will be presented. This is a valid statistical model for the two pooled studies and will be interpreted appropriately within the pooled CSR.

### 4.4.2 Secondary Efficacy Endpoints

#### 4.4.2.1 Hierarchy of and Statistical Testing Strategy for Secondary Efficacy Endpoints

As stated in the individual study-level SAP, the hierarchical order of secondary efficacy endpoints is shown below, with endpoints listed in descending order of importance:

- The time to onset of confirmed disability progression (CDP) for at least 12 weeks, with the initial event of neurological worsening occurring during the 96-week, double-blind, double-dummy treatment period
- The total number of T1 gadolinium (Gd)–enhancing lesions as detected by brain magnetic resonance imaging (MRI) at Weeks 24, 48, and 96
- The total number of new and/or enlarging T2 hyperintense lesions as detected by brain MRI at Weeks 24, 48, and 96
- The proportion of patients who have confirmed disability improvement (CDI) for at least 12 weeks, with the initial event of neurological improvement occurring during the 96-week, double-blind, double-dummy treatment period
- The time to onset of CDP for at least 24 weeks, with the initial event of neurological worsening occurring during the 96-week, double-blind, double-dummy treatment period
- The total number of T1-hypo-intense lesions (chronic black holes) at Weeks 24, 48, and 96
- The change in Multiple Sclerosis Functional Composite Scale (MSFCS) score from baseline to Week 96
- The percentage change in brain volume as detected by brain MRI from Week 24 to Week 96
- The change in Short Form 36 (SF-36) Physical Component Summary (PCS) Score from baseline to Week 96
- The proportion of patients who have no evidence of disease activity (NEDA) by Week 96

The rationale for this hierarchical order of secondary endpoints is described in detail in the study protocols and the individual study-level SAP.

Secondary efficacy endpoints will be tested in the sequence presented in the hierarchical order listed above, all at the $\alpha = 0.05$ level.

All p-values will be reported and will be interpreted as either confirmatory or non-confirmatory (i.e., descriptive only). The circumstances for confirmatory and for non-confirmatory interpretation of p-values are described below:

- The first secondary efficacy endpoint (the time to onset of confirmed disability progression for at least 12 weeks) will be tested in a confirmatory manner if and only if the primary efficacy endpoint (annualized protocol-defined relapse rate by 2 years) reaches a significance level of 0.05 (i.e., pooled analysis $p \leq 0.05$). If the primary

efficacy endpoint pooled analysis $p > 0.05$, then all secondary efficacy endpoint pooled analysis p-values within the hierarchy will be interpreted as non-confirmatory.

- The second (and subsequent) secondary efficacy endpoint will be tested in a confirmatory manner if and only if the first (or immediately previous) secondary efficacy endpoint reaches a significance level of 0.05 (i.e., pooled analysis $p \leq 0.05$). If the first (or immediately previous) secondary efficacy endpoint pooled analysis $p > 0.05$, then the second (or current) and all subsequent secondary efficacy endpoint pooled analysis p-values within the hierarchy will be interpreted as non-confirmatory.

#### 4.4.2.2 Time to Onset of Confirmed Disability Progression for at Least 12 Weeks with the Initial Event of Neurological Worsening Occurring during the 96-Week, Double-Blind, Double-Dummy Treatment Period

For this secondary efficacy endpoint, the pooled analysis for Studies WA21092 and WA21093 will follow the same general approach described in the individual study-level SAP, with exceptions as noted below.

The analysis for this endpoint is described in Section 4.4.2.3 of the individual study-level SAP.

To assess the validity of pooling data across the two studies, the treatment effect (hazard ratio and CI) for this endpoint within each trial from individual cox-regression will be displayed in a forest plot. In interpreting the trial comparisons with a view toward assessing the validity of the pooled dataset, the primary interest is in confirming whether the treatment effect is qualitatively similar across the two studies (i.e., positive treatment effects are shown in both studies).

For the pooled analysis of this endpoint, the log-rank test and Cox regression model will use the same stratification factors described for this analysis in the individual study-level SAP, except that an additional term for stratification by study will be included.

In addition, the following sensitivity analyses will be performed (see Section 4.4.2.3 of the individual study-level SAP):

- The following methods for the handling of missing data will be used:

  Method 1: with use of the same kind of multiple imputation approach described at the end of Section 4.4.1 of the individual study-level SAP, patients with an initial disability progression during the 96-week, double-blind, double-dummy treatment period who discontinue the treatment early and who do not have a subsequent visit with an EDSS measurement will be randomly imputed as an event of confirmed disability progression at the date of initial disability progression with a probability of 50%; the other 50% of these patients will be censored at the date of initial disability progression. A total of 1000 ($m = 1000$) imputed datasets will be produced.

Method 2: Patients with an initial disability progression during the 96-week, double-blind, double-dummy treatment period who discontinue treatment early and who do not have a subsequent visit with an EDSS measurement will be imputed as an event of confirmed disability progression at the date of initial disability progression.

- Adjustment for additional baseline factors: Sensitivity analysis will be performed using the same tests described above adjusting for the following baseline factors:

  Number of relapses occurring within a 2-year period prior to study entry

  Baseline presence of Gd lesions (present or absent)

  Prior immunomodulatory treatment

  Age ($<40$, $\geq 40$ years)

#### 4.4.2.3 Total Number of T1 Gadolinium– Enhancing Lesions as Detected by Brain Magnetic Resonance Imaging at Weeks 24, 48, and 96

The statistical analysis of this endpoint will follow the same approach that is used for each study, as described in the individual study-level SAP. The only exception is that in this pooled analysis a study main effect term will also be included in the statistical model.

#### 4.4.2.4 Total Number of New and/or Enlarging T2 Hyperintense Lesions as Detected by Brain Magnetic Resonance Imaging at Week 24, 48, and 96

The statistical analysis of this endpoint will follow the same approach that is used for each study, as described in the individual study-level SAP. The only exception is that in this pooled analysis a study main effect term will also be included in the statistical model.

#### 4.4.2.5 Proportion of Patients Who Have Confirmed Disability Improvement for at Least 12 Weeks with the Initial Event of Neurological Improvement Occurring during the 96-week Double-Blind, Double-Dummy Treatment Period

The statistical analysis of this endpoint will follow the same approach that is used for each study, as described in the individual study-level SAP. The only exception is that in this pooled analysis a study main effect term will also be included as a stratification factor in the Cochran-Mantel-Haenszel (CMH) $\chi^2$ test.

#### 4.4.2.6 Time to Onset of Confirmed Disability Progression for at Least 24 Weeks with the Initial Event of Neurological Worsening Occurring during the 96-Week Double-Blind, Double-Dummy Treatment Period

This endpoint is the same as the 12-week confirmed disability progression except that the confirmation of disability progression must occur at the regularly scheduled visit that is at least 24 weeks ($\geq$161 days) after initial disability progression. See Section 2.4 for additional information on the timing of this analysis.

For this secondary efficacy endpoint, the pooled analysis for Studies WA21092 and WA21093 will follow the same general approach described in the individual study-level SAP, with exceptions as noted below.

For this endpoint, the log-rank test and Cox regression model will use the same stratification factors described in the individual study-level SAP, except that an additional term for study main effect will be included.

#### 4.4.2.7 Total Number of T1-Hypo-Intense Lesions (Chronic Black Holes) at Weeks 24, 48, and 96

The statistical analysis of this endpoint will follow the same approach that is used for each study, as described in the individual study-level SAP. The only exception is that in this pooled analysis a study main effect term will also be included in the statistical model.

#### 4.4.2.8 Change in Multiple Sclerosis Functional Composite Scale Score from Baseline to Week 96

The statistical analysis of this endpoint will follow the same approach that is used for each study, as described in the individual study-level SAP. The only exception is that in this pooled analysis a study main effect term will also be included in the statistical model.

#### 4.4.2.9 Percentage Change in Brain Volume as Detected by Brain Magnetic Resonance Imaging Scan from Week 24 to Week 96

The statistical analysis of this endpoint will follow the same approach that is used for each study, as described in the individual study-level SAP. The only exception is that in this pooled analysis a study main effect term will also be included in the statistical model.

#### 4.4.2.10 Change in Quality of Life, as Measured by the Short Form 36 Version 2 Physical Component Summary Score from Baseline to Week 96

The statistical analysis of this endpoint will follow the same approach that is used for each study, as described in the individual study-level SAP. The only exception is that in this pooled analysis a study main effect term will also be included in the statistical model.

#### 4.4.2.11 Proportion of Patients Who Have No Evidence of Disease Activity by Week 96

The statistical analysis of this endpoint will follow the same approach that is used for each study, as described in the individual study-level SAP. The only exception is that in this pooled analysis a study main effect term will additionally be included as a stratification factor in the CMH $\chi^2$ test.

### 4.4.3 Exploratory Efficacy Endpoints

The individual study-level SAP describes analyses planned for exploratory efficacy endpoints. The three listed below will be repeated in the pooled analysis of Studies WA21092 and WA21093.

- The proportion of patients who have confirmed disability improvement for at least 24 weeks, with the initial event of neurological improvement occurring during the 96-week, double-blind, double-dummy treatment period
- The duration of the confirmed disability improvement (Kaplan Meier estimate)
- The proportion of patients who at Week 96 have improved, stable, or worsened disability, compared to baseline

### 4.4.4 Subgroup Analyses

The primary and two of the key secondary endpoints (12- and 24-week confirmed disability progression) will be summarized and analyzed by the following subgroups:

- Demography: age ($\geq$40 vs. $<$40 years)
- Demography: sex (male vs. female)
- Demography: race (White vs. other)
- Body weight: $\geq$75 kg versus $<$75 kg
- Stratification factor: geographical region (United States vs. ROW)
- Stratification factor: baseline EDSS score ($<$4.0 vs. $\geq$4.0)
- Previous lesions: baseline Gd-enhancing lesion (0 vs. $>$0)

The subgroups below will be analyzed only for E.U. regulatory purposes only:

- Previous treatments: previously treated with interferon-beta or glatiramer acetate (yes vs. no)
- Previous treatments: previously treated with interferon-beta or glatiramer acetate for at least 6 months (yes vs. no)
- Previous treatments/relapses: previously treated with interferon-beta or glatiramer acetate for at least 6 months and had at least one relapse while on treatment (yes vs. no)
- Previous relapses: patients with $\geq$2 relapses in the year prior to randomization (yes vs. no)
- Previous relapses/lesions: patients with $\geq$1 relapses in the last year prior to randomization and $\geq$1 baseline Gd-enhancing lesion (yes vs. no)
- Previous relapses: patients with $\geq$2 relapses in the previous 2 years and $\geq$1 relapses in the last year prior to randomization (yes vs. no)
- Previous relapses/lesions: patients with $\geq$2 relapses in the previous 2 years and $\geq$1 relapse in the last year prior to randomization and not previously treated with interferon-beta or glatiramer acetate (yes vs. no)

- Previous lesions: ≥1 baseline Gd-enhancing lesion (yes vs. no)
- Non-responders: patients treated with interferon-beta or glatiramer acetate for at least 1 year, and had at least, 1 relapse in the previous year and have at least 9 T2-hyperintense lesions or ≥1 Gd lesions (yes vs. no)
- Non-responders: patients treated with interferon-beta or glatiramer acetate for at least 1 year, with unchanged relapse rate compared with previous year (i.e., same relapse rate), or increased relapse rate compared with previous year (at least +1 relapse) (yes vs. no)
- Non-responders: patients with at least one relapse in the previous year while receiving therapy with interferon-beta or glatiramer acetate, and have at least 9 T2 hyperintense lesions in a cranial MRI or at least 1 Gd-enhancing lesion or patient with an unchanged or increased relapse rate, as compared with the previous year (i.e., patients included in at least one of the two non-responder subgroups listed immediately above) (yes vs. no)
- Patients with rapidly evolving severe relapsing remitting multiple sclerosis (RRMS): patients with ≥2 relapses in the last year prior to randomization and ≥1 baseline Gd-enhancing lesion or increase in T2 lesion load at the baseline visit (changing from 0–5 to 6–9 lesions or from 6–9 lesions to >9 lesions), compared with the prior MRI (yes vs. no)

Estimates of treatment effect and associated 95% CI will be presented in forest plots. Unadjusted p-values will also be presented for these analyses.

It is not expected that there will be a fundamental difference of treatment benefit in those subgroups, compared with the main analysis (i.e., the full intent-to-treat population). If important differences of treatment benefit are observed (e.g., in the forest plots), further analyses will be performed. For example, demographic and baseline characteristics will be summarized. In case of imbalance, further sensitivity analyses of the main efficacy endpoints would be performed for these subgroups, adjusting for the demographic and baseline characteristics that are not balanced between the treatment groups.

### 4.4.5 Efficacy Data from the Safety Follow-Up Phase

Efficacy data from the safety follow-up phase will not be included in this pooled analysis of Studies WA21092 and WA21093. Exploratory analyses of efficacy data collected in the safety follow-up phase are envisioned. More details will be provided in a further version of the SAP.

### 4.4.6 Efficacy Data from the Open-Label Extension Phase

Efficacy data from the open-label extension phase will not be included in this pooled analysis of Studies WA21092 and WA21093. Exploratory analyses of efficacy data collected in the open-label extension phase are envisioned. More details will be provided in a further version of the SAP

## 4.5 PHARMACOKINETIC AND PHARMACODYNAMIC ANALYSES

### 4.5.1 Pharmacokinetic Analyses

The individual study-level SAP provides some detail on the planned PK analyses on pooled data. Further details will be described in a Modeling and Simulation Analysis Plan, and results will be reported separately.

### 4.5.2 Pharmacodynamic Analyses

The individual study-level SAP provides some detail on the planned PD analyses on pooled data, and the results of the analyses on the pooled data will be reported in the PK report.

## 4.6 SAFETY ANALYSES

The individual study-level SAP provides information regarding safety analyses including exposure to study medication, Columbia–Suicide Severity Rating Scale, adverse events, laboratory data, and vital signs to be performed for each study individually. The pooled safety analyses to be performed for Studies WA21092 and WA21093 combined will be provided in a subsequent version of this analysis plan, prior to the first of the two study database locks for the primary analysis.

## 4.7 MISSING DATA

All methods for handling missing data and associated sensitivity analyses are described in the individual study-level SAP, section by section, for each endpoint. This pooled analysis will use the same approach to missing data as described in the individual study-level SAP.

## 4.8 INTERIM ANALYSES

No interim analyses of efficacy data are planned.

# STATISTICAL ANALYSIS PLAN

## POOLED ANALYSIS OF STUDIES WA21092 AND WA21093

| | |
|---|---|
| **TITLE:** | **A RANDOMIZED, DOUBLE-BLIND, DOUBLE-DUMMY, PARALLEL-GROUP STUDY TO EVALUATE THE EFFICACY AND SAFETY OF OCRELIZUMAB IN COMPARISON TO INTERFERON BETA-1A (REBIF®) IN PATIENTS WITH RELAPSING MULTIPLE SCLEROSIS** |
| **PROTOCOL NUMBERS:** | **WA21092 and WA21093** |
| **STUDY DRUG:** | **Ocrelizumab (RO4964913)** |
| **VERSION NUMBER:** | **2** |
| **IND NUMBER:** | **100,593** |
| **EUDRACT NUMBERS:** | **2010-020337-99 and 2010-020315-36** |
| **SPONSOR:** | F. Hoffmann-La Roche Ltd |
| **PLAN PREPARED BY:** | Gaelle Klingelschmitt |
| **DATE FINAL:** | 3 September 2014 |
| **DATE AMENDED** | See electronic date stamp below. |

**STATISTICAL ANALYSIS PLAN AMENDMENT APPROVAL**

| Name | Reason for Signing | Date and Time (UTC) |
|---|---|---|
| Burger,Hans_Ulrich | Company Signatory | 14-May-2015 16:35:55 |

**CONFIDENTIAL**

This is an F. Hoffmann-La Roche Ltd document that contains confidential information. Nothing herein is to be disclosed without written consent from F. Hoffmann-La Roche Ltd.

# STATISTICAL ANALYSIS PLAN AMENDMENT RATIONALE

Updates to the amended SAP include the following major changes.

The Sponsor has modified the subgroups for analysis to take into account:

- Interactions with the CHMP on the need for subgroups to include both active and highly active patient populations to allow comparison between ocrelizumab and other approved multiple sclerosis (MS) therapies
- Finalization of the MS guideline EMA/CHMP/771815/2011, Rev 2 (26 March 2015), which outlines the need for subgroups to allow for a separate risk-benefit assessment for different degrees of disease activity in MS

Detailed updates have been made to the safety analysis section.

The SAS code has been included for the primary analysis.

Additional minor changes have been made to improve clarity and consistency.

## TABLE OF CONTENTS

STATISTICAL ANALYSIS PLAN AMENDMENT RATIONALE ............................ 2

1. BACKGROUND ........................................................................... 6

2. WA21092/WA21093 STUDY DESIGN ....................................................... 6

2.1 Protocol Synopsis ........................................................ 6

2.2 Outcome Measures ......................................................... 6

2.2.1 Primary Efficacy Outcome Measures ..................................... 6

2.2.2 Secondary Efficacy Outcome Measures ................................... 6

2.2.3 Exploratory Efficacy Outcome Measures ................................. 7

2.2.4 Pharmacokinetic Analysis: Objectives and Outcome Measures ............. 7

2.2.5 Pharmacodynamic Outcome Measures ...................................... 7

2.2.6 Safety Outcome Measures ............................................... 7

2.3 Determination of Sample Size ............................................. 8

2.4 Analysis Timing .......................................................... 8

3. STUDY CONDUCT ........................................................................ 8

4. STATISTICAL METHODS .................................................................. 8

4.1 Analysis Populations ...................................................... 8

4.2 Analysis of Study Conduct ................................................. 8

4.3 Analysis of Treatment Group Comparability ................................ 8

4.4 Efficacy Analysis .......................................................... 9

4.4.1 Primary Efficacy Endpoint, Annualized Protocol-Defined Relapse Rate by 2 Years (96 Weeks) ...................... 10

4.4.2 Secondary Efficacy Endpoints .......................................... 11

4.4.2.1 Hierarchy of and Statistical Testing Strategy for Secondary Efficacy Endpoints ............................................ 11

4.4.2.2 Time to Onset of Confirmed Disability Progression for at Least 12 Weeks with the Initial Event of Neurological Worsening Occurring during the 96-Week, Double-Blind, Double-Dummy Treatment Period ........................................ 12

4.4.2.3 Total Number of T1 Gadolinium– Enhancing Lesions as Detected by Brain Magnetic Resonance Imaging at Weeks 24, 48, and 96 ....................... 13

4.4.2.4 Total Number of New and/or Enlarging T2 Hyperintense Lesions as Detected by Brain Magnetic Resonance Imaging at Week 24, 48, and 96 ..... 14
4.4.2.5 Proportion of Patients Who Have Confirmed Disability Improvement for at Least 12 Weeks with the Initial Event of Neurological Improvement Occurring during the 96-Week Double-Blind, Double-Dummy Treatment Period ..... 14
4.4.2.6 Time to Onset of Confirmed Disability Progression for at Least 24 Weeks with the Initial Event of Neurological Worsening Occurring during the 96-Week Double-Blind, Double-Dummy Treatment Period ..... 14
4.4.2.7 Total Number of New T1-Hypo-Intense Lesions (Chronic Black Holes) at Weeks 24, 48, and 96 ..... 14
4.4.2.8 Change in Multiple Sclerosis Functional Composite Scale Score from Baseline to Week 96 ..... 14
4.4.2.9 Percentage Change in Brain Volume as Detected by Brain Magnetic Resonance Imaging Scan from Week 24 to Week 96 ..... 15
4.4.2.10 Change in Quality of Life, as Measured by the Short Form 36 Version 2 Physical Component Summary Score from Baseline to Week 96 ..... 15
4.4.2.11 Proportion of Patients Who Have No Evidence of Disease Activity by Week 96 ..... 15
4.4.3 Exploratory Efficacy Endpoints ..... 15
4.4.4 Subgroup Analyses ..... 15
4.4.5 Efficacy Data from the Safety Follow-Up Phase ..... 16
4.4.6 Efficacy Data from the Open-Label Extension Phase ..... 16
4.5 Pharmacokinetic and Pharmacodynamic Analyses ..... 16
4.5.1 Pharmacokinetic Analyses ..... 16
4.5.2 Pharmacodynamic Analyses ..... 16
4.6 Safety Analyses ..... 16
4.6.1 Pool A: Phase III Relapsing Multiple Sclerosis Controlled Treatment Population ..... 16

4.6.2 Exposure to the Drug ........ 17
4.6.3 Treatments ........ 18
4.6.4 Common Adverse Events ........ 18
4.6.4.1 Frequency Counts of Adverse Events ........ 19
4.6.4.2 Number of Adverse Events per 100 Patient-Years ........ 19
4.6.4.3 Adverse Events per Treatment Dose ........ 20
4.6.4.4 Related Adverse Events ........ 20
4.6.4.5 Adverse Events by Intensity ........ 20
4.6.5 Deaths ........ 21
4.6.6 Serious Adverse Events ........ 21
4.6.7 Adverse Events That Lead to Withdrawal of Study Treatment ........ 21
4.6.8 Adverse Events Leading to Modification or Interruption of Study Treatment ........ 22
4.6.9 Selected Adverse Events ........ 22
4.6.9.1 Infusion-Related Reactions ........ 22
4.6.9.2 Infections ........ 23
4.6.9.3 Malignancies ........ 25
4.6.9.4 Clinical Laboratory Evaluations ........ 25
4.6.9.5 General Laboratory Evaluations ........ 26
4.6.9.6 CD19 Cell Counts ........ 26
4.6.9.7 Anti-Drug Antibodies ........ 26
4.6.10 Subgroup Analyses ........ 26
4.7 Missing Data ........ 27
4.8 Interim Analyses ........ 27
4.9 Subgroup Analyses for European Union Regulatory Purposes ........ 27

# 1. BACKGROUND

Studies WA21092 and WA21093 are two Phase III studies with identical study design. There is a separate statistical analysis plan (SAP) describing the analyses to be performed for each study independently. This “pooled” SAP describes solely those analyses that will be performed on the two studies combined. The same statistical methods will be used for the pooled and individual study analyses, but some additional considerations are required for the pooled analyses. For example, the study name is added as a stratification factor or covariate in some analyses. The analysis of some secondary efficacy endpoints, Confirmed Disability Progression and Confirmed Disability Improvement, cannot be tested in a confirmatory manner in the individual studies and will necessitate the pooling of both studies in order to have sufficient statistical power to detect relevant treatment differences, as mentioned in the protocol. The analysis of the primary efficacy endpoint, secondary endpoints, and safety analyses are done in the pooled dataset to summarize the safety and efficacy of ocrelizumab in RMS collected in Studies WA21092 and WA21093.

Further background details for Studies WA21092 and WA21093 can be found in the study-level SAP.

# 2. WA21092/WA21093 STUDY DESIGN

Refer to the individual study-level SAP, or the individual study protocols, for full details on study design and assessments for Studies WA21092 and WA21093.

## 2.1 PROTOCOL SYNOPSIS

For the protocol synopsis and Schedule of Assessments, refer to the individual study protocols.

## 2.2 OUTCOME MEASURES

### 2.2.1 Primary Efficacy Outcome Measures

The primary efficacy endpoint is the annualized protocol-defined relapse rate at 2 years (96 weeks).

### 2.2.2 Secondary Efficacy Outcome Measures

All secondary efficacy endpoints (also called key efficacy endpoints) described in the study level SAP are also analyzed in the pooled data and are as follows:

- The time to onset of confirmed disability progression (CDP) for at least 12 weeks, with the initial event of neurological worsening occurring during the 96-week, double-blind, double-dummy treatment period
- The total number of T1 gadolinium (Gd)–enhancing lesions as detected by brain magnetic resonance imaging (MRI) at Weeks 24, 48, and 96
- The total number of new and/or enlarging T2 hyperintense lesions as detected by brain MRI at Weeks 24, 48, and 96

- The proportion of patients who have confirmed disability improvement (CDI) for at least 12 weeks, with the initial event of neurological improvement occurring during the 96-week, double-blind, double-dummy treatment period
- The time to onset of CDP for at least 24 weeks, with the initial event of neurological worsening occurring during the 96-week, double-blind, double-dummy treatment period
- The total number of new T1-hypo-intense lesions (chronic black holes) at Weeks 24, 48, and 96
- The change in Multiple Sclerosis Functional Composite Scale (MSFCS) score from baseline to Week 96
- The percentage change in brain volume as detected by brain MRI from Week 24 to Week 96
- The change in Short Form 36 (SF-36) Physical Component Summary (PCS) Score from baseline to Week 96
- The proportion of patients who have no evidence of disease activity (NEDA) by Week 96

### 2.2.3 Exploratory Efficacy Outcome Measures

The exploratory efficacy endpoints analyzed at the individual study level will not be included in the pooled analysis of Studies WA21092 and WA21093, with the exception of the following three endpoints:

- The proportion of patients who have confirmed disability improvement for at least 24 weeks, with the initial event of neurological improvement occurring during the 96-week, double-blind, double-dummy treatment period
- The duration of the confirmed disability improvement (Kaplan Meier estimate)
- The proportion of patients who, at Week 96, have improved, stable, or worsened disability, compared to baseline

### 2.2.4 Pharmacokinetic Analysis: Objectives and Outcome Measures

Refer to the individual study-level SAP for a description of the pharmacokinetic (PK) analysis.

### 2.2.5 Pharmacodynamic Outcome Measures

Refer to the individual study-level SAP and the Modeling and Simulation Plan for a description of the PD analysis of pooled data that will be presented in a separate report.

### 2.2.6 Safety Outcome Measures

The study-level SAP covers safety analyses in the areas of exposure to study medication, Columbia-Suicide Severity Rating Scale, adverse events, anti-drug antibody (ADA), laboratory data, and vital signs to be performed for each study individually.

## 2.3 DETERMINATION OF SAMPLE SIZE

The sample size for Studies WA21092 and WA21093 was determined in the same manner for both studies. Further details of this sample size determination are provided in the study-level SAP and in the study protocols.

## 2.4 ANALYSIS TIMING

The primary pooled analysis of Studies WA21092 and WA21093 will be conducted immediately after the completion of the individual study analyses of Studies WA21092 and WA21093.

# 3. STUDY CONDUCT

For details of randomization, independent review facility, Expanded Disability Status Scale (EDSS), cleaning process, and data monitoring, refer to the individual study-level SAP.

# 4. STATISTICAL METHODS

## 4.1 ANALYSIS POPULATIONS

All analysis populations used in the pooled analysis are defined in the same manner as the analyses of the individual studies. Details are provided in the individual study-level SAP.

## 4.2 ANALYSIS OF STUDY CONDUCT

The individual study-level SAP contains details of the data to be included in the individual study-level databases. The database that is used for the pooled analysis will simply be the consolidation of the individual databases for Studies WA21092 and WA21093.

No summaries or analyses will be performed at the pooled level to evaluate study conduct (i.e., those performed at the individual study level will be sufficient to evaluate study conduct).

## 4.3 ANALYSIS OF TREATMENT GROUP COMPARABILITY

The individual study-level SAP contains details of the summaries to be presented for individual Studies WA21092 and WA21093 in the following areas:

- Demography
- Baseline disease characteristics
- Multiple sclerosis (MS) disease history
- Non-MS disease history
- Prior treatments for MS
- Baseline MRI data (including previous MRI data collected at screening)

The summaries that are listed above will be presented for the pooled analysis of both studies in exactly the same manner as for each individual study for both Intent-to-Treat (ITT) and Safety Populations.

To assess the validity of pooling data across the two studies, the demographic and baseline characteristics will be visually compared for Studies WA21092 and WA21093 and for the pooled database of both studies.

## 4.4 EFFICACY ANALYSIS

Except where stated, statistical hypotheses for the primary and secondary endpoints and treatment comparisons will be tested at the 5% significance level ($\alpha = 0.05$) against two-sided alternatives.

For all assessments, the baseline value will be used as the last non-missing value on or before the date of the first infusion of study medication. See Section 4.4.1 for baseline EDSS and Section 4.4.2.2 for time to onset of confirmed disability progression and Section 4.4.2.5 for the proportion of patients with confirmed disability improvement.

All primary and secondary efficacy endpoints will be analyzed, stratified or adjusted by study (WA21092 vs. WA21093), geographical region (United States vs. rest of world [ROW]) and baseline EDSS score ($<$4.0 vs. $\geq$4.0, stratification factor).

Unless otherwise specified, no sensitivity analyses will be performed as part of this pooled analysis of the efficacy endpoints; only the primary analysis for the primary efficacy endpoint and secondary endpoints will be performed (primary analysis in "Analysis Methods" part of Section 4.4.1 of the individual study-level SAP, and the first analysis of each secondary endpoint described in the various subsections in Section 4.4.2 of the individual study-level SAP).

In pooling Studies WA21092 and WA21093, it is important to assess whether the treatment difference between ocrelizumab and interferon beta-1a (Rebif®) 44 μg subcutaneous (SC) is broadly consistent between the two studies. The poolability will be examined for the primary endpoint annualized relapse rate (ARR) and the first secondary endpoint 12 week CDP; See Section 4.4.1 and Section 4.4.2.2, respectively.

If the primary endpoint (annualized protocol-defined relapse rate by 2 years) and the first secondary efficacy endpoint (time to onset of confirmed disability progression for at least 12 weeks with the initial event of neurological worsening occurring during the 96-week double-blind, double-dummy treatment period) for the ITT population can validly be pooled for the two studies, this will be taken as a general indication that it is similarly valid to pool the primary and all secondary efficacy endpoint analyses and to undertake the subgroup analyses specified in Section 4.4.4 on the two studies pooled.

### 4.4.1 Primary Efficacy Endpoint, Annualized Protocol-Defined Relapse Rate by 2 Years (96 Weeks)

For the primary efficacy endpoint, the combined pooled analysis for Studies WA21092 and WA21093 will follow the same general approach described in the individual study-level SAP, with exceptions noted below.

No sensitivity analyses will be performed as part of this pooled analysis of the primary efficacy endpoint; only the primary analysis for the primary efficacy endpoint will be performed (described in the “Analysis Methods” part of Section 4.4.1 of the individual study-level SAP).

Sample SAS code can be found below (SAS code is regarded as “draft” until fully validated at the analysis stage):

```
proc genmod data=ARR;
   class ARMCD STUDY REGION BEDSSCAT;
   model N_PDR = ARMCD STUDY REGION BEDSSCAT / offset=EXPLOG
link=log dist=negbin type3;
   lsmeans ARMCD / exp cl;
   ods output lsmeans=lsm;
   ods output ParameterEstimates=est;
run;
```

The poolability assessment will be achieved via the inclusion in statistical models of an interaction term between study and treatment group in the analysis of the primary endpoint ARR.

```
proc genmod data=ARR;
   class ARMCD STUDY REGION BEDSSCAT;
   model N_PDR = ARMCD STUDY ARMCD*STUDY REGION BEDSSCAT /
offset=EXPLOG link=log dist=negbin type3;
run;
```

Considering the interaction between study and treatment group, a quantitative interaction occurs when the difference between the two treatments is different between the two studies but both within–study treatment differences are in the same direction. That is, ocrelizumab shows a better response than interferon beta-1a 44 μg SC in both studies but to a differing extent, or alternatively, interferon beta-1a 44 μg SC shows a better response than ocrelizumab in both studies but to a differing extent. A qualitative interaction occurs when the difference between the two treatments is different between the two studies and these treatment differences are in opposite directions. That is, in one study, ocrelizumab shows a better response than interferon beta-1a 44 μg SC, and in the other study, interferon beta-1a 44 μg SC shows a better response than ocrelizumab.

With a 10% significance level, if $p \leq 0.10$ for study-by-treatment group interaction and the interaction is qualitative rather than quantitative, it will be concluded that it is not appropriate to pool the two studies, because there is evidence of fundamentally different treatment effects in the two studies. In this situation, the study-by-treatment interaction term will be reported in the pooled clinical study report (CSR), but no statistical table will be presented because the pooled analysis is not considered valid. The two individual study-level analyses represent the only valid treatment comparisons for the primary efficacy endpoint, but if these results are in disagreement, interpretation of the treatment effect is problematic.

In all other cases (i.e., $p > 0.10$ for study-by-treatment group interaction, or $p \leq 0.10$ for study-by-treatment group interaction but the interaction is quantitative rather than qualitative), it will be concluded that it is valid to pool the two studies because there is no evidence of fundamentally different treatment effects in the two studies. In this situation, the study-by-treatment interaction term will be reported in the statistical table but then removed from the model and the resulting model (original individual study-level model plus study effect) will be presented. This is a valid statistical model for the two pooled studies and will be interpreted appropriately within the pooled CSR.

### 4.4.2 Secondary Efficacy Endpoints

#### 4.4.2.1 Hierarchy of and Statistical Testing Strategy for Secondary Efficacy Endpoints

As stated in the individual study-level SAP, the hierarchical order of secondary efficacy endpoints is shown below, with endpoints listed in descending order of importance:

- The time to onset of confirmed disability progression (CDP) for at least 12 weeks, with the initial event of neurological worsening occurring during the 96-week, double-blind, double-dummy treatment period
- The total number of T1 gadolinium (Gd)–enhancing lesions as detected by brain magnetic resonance imaging (MRI) at Weeks 24, 48, and 96
- The total number of new and/or enlarging T2 hyperintense lesions as detected by brain MRI at Weeks 24, 48, and 96
- The proportion of patients who have confirmed disability improvement (CDI) for at least 12 weeks, with the initial event of neurological improvement occurring during the 96-week, double-blind, double-dummy treatment period
- The time to onset of CDP for at least 24 weeks, with the initial event of neurological worsening occurring during the 96-week, double-blind, double-dummy treatment period
- The total number of new T1-hypo-intense lesions (chronic black holes) at Weeks 24, 48, and 96
- The change in Multiple Sclerosis Functional Composite Scale (MSFCS) score from baseline to Week 96

- The percentage change in brain volume as detected by brain MRI from Week 24 to Week 96
- The change in Short Form 36 (SF-36) Physical Component Summary (PCS) Score from baseline to Week 96
- The proportion of patients who have no evidence of disease activity (NEDA) by Week 96

The rationale for this hierarchical order of secondary endpoints is described in detail in the study protocols and the individual study-level SAP.

Secondary efficacy endpoints will be tested in the sequence presented in the hierarchical order listed above, all at the $\alpha = 0.05$ level.

All p-values will be reported and will be interpreted as either confirmatory or non-confirmatory (i.e., descriptive only). The circumstances for confirmatory and for non-confirmatory interpretation of p-values are described below:

- The first secondary efficacy endpoint (the time to onset of confirmed disability progression for at least 12 weeks) will be tested in a confirmatory manner if and only if the primary efficacy endpoint (annualized protocol-defined relapse rate by 2 years) reaches a significance level of 0.05 (i.e., pooled analysis $p \leq 0.05$). If the primary efficacy endpoint pooled analysis $p > 0.05$, then all secondary efficacy endpoint pooled analysis p-values within the hierarchy will be interpreted as non-confirmatory.
- The second (and subsequent) secondary efficacy endpoint will be tested in a confirmatory manner if and only if the first (or immediately previous) secondary efficacy endpoint reaches a significance level of 0.05 (i.e., pooled analysis $p \leq 0.05$). If the first (or immediately previous) secondary efficacy endpoint pooled analysis $p > 0.05$, then the second (or current) and all subsequent secondary efficacy endpoint pooled analysis p-values within the hierarchy will be interpreted as non-confirmatory.

#### 4.4.2.2 Time to Onset of Confirmed Disability Progression for at Least 12 Weeks with the Initial Event of Neurological Worsening Occurring during the 96-Week, Double-Blind, Double-Dummy Treatment Period

For this secondary efficacy endpoint, the pooled analysis for Studies WA21092 and WA21093 will follow the same general approach described in the individual study-level SAP, with exceptions as noted below.

The analysis for this endpoint is described in Section 4.4.2.3 of the individual study-level SAP. Sample SAS code is as per individual SAP with additional terms for STUDY.

To assess the validity of pooling data across the two studies, the treatment effect (hazard ratio and CI) for this endpoint within each trial from individual cox-regression will be displayed in a forest plot. In interpreting the trial comparisons with a view toward assessing the validity of the pooled dataset, the primary interest is in confirming whether

the treatment effect is qualitatively similar across the two studies (i.e., positive treatment effects are shown in both studies).

For the pooled analysis of this endpoint, the log-rank test and Cox regression model will use the same stratification factors described for this analysis in the individual study-level SAP, except that an additional term for stratification by study will be included.

In addition, the following sensitivity analyses will be performed (see Section 4.4.2.3 of the individual study-level SAP):

- The following methods for the handling of missing data will be used:

  Method 1: With use of the same kind of multiple imputation approach described at the end of Section 4.4.1 of the individual study-level SAP, patients with an initial disability progression during the 96-week, double-blind, double-dummy treatment period who discontinue the treatment early and who do not have a subsequent visit with an EDSS measurement will be randomly imputed as an event of confirmed disability progression at the date of initial disability progression with a probability of 50%; the other 50% of these patients will be censored at the date of initial disability progression. A total of 1000 ($m = 1000$) imputed datasets will be produced.

  Method 2: Patients with an initial disability progression during the 96-week, double-blind, double-dummy treatment period who discontinue treatment early and who do not have a subsequent visit with an EDSS measurement will be imputed as an event of confirmed disability progression at the date of initial disability progression.

- Adjustment for additional baseline factors: Sensitivity analysis will be performed using the same tests described above adjusting for the following baseline factors:

  Number of relapses occurring within a 2-year period prior to study entry

  Baseline presence of Gd lesions (present or absent)

  Prior MS treatment

  Age ($<40$, $\geq 40$ years)

#### 4.4.2.3 Total Number of T1 Gadolinium– Enhancing Lesions as Detected by Brain Magnetic Resonance Imaging at Weeks 24, 48, and 96

The statistical analysis of this endpoint will follow the same approach that is used for each study, as described in the individual study-level SAP. The only exception is that in this pooled analysis, a study main effect term will also be included in the statistical model.

#### 4.4.2.4 Total Number of New and/or Enlarging T2 Hyperintense Lesions as Detected by Brain Magnetic Resonance Imaging at Week 24, 48, and 96

The statistical analysis of this endpoint will follow the same approach that is used for each study, as described in the individual study-level SAP. The only exception is that in this pooled analysis, a study main effect term will also be included in the statistical model.

#### 4.4.2.5 Proportion of Patients Who Have Confirmed Disability Improvement for at Least 12 Weeks with the Initial Event of Neurological Improvement Occurring during the 96-Week Double-Blind, Double-Dummy Treatment Period

The statistical analysis of this endpoint will follow the same approach that is used for each study, as described in the individual study-level SAP. The only exception is that in this pooled analysis, a study main effect term will also be included as a stratification factor in the Cochran-Mantel-Haenszel (CMH) $\chi^2$ test.

#### 4.4.2.6 Time to Onset of Confirmed Disability Progression for at Least 24 Weeks with the Initial Event of Neurological Worsening Occurring during the 96-Week Double-Blind, Double-Dummy Treatment Period

This endpoint is the same as the 12-week confirmed disability progression except that the confirmation of disability progression must occur at the regularly scheduled visit that is at least 24 weeks ($\geq$161 days) after initial disability progression. See Section 2.4 for additional information on the timing of this analysis.

For this secondary efficacy endpoint, the pooled analysis for Studies WA21092 and WA21093 will follow the same general approach described in the individual study-level SAP, with exceptions as noted below.

For this endpoint, the log-rank test and Cox regression model will use the same stratification factors described in the individual study-level SAP, except that an additional term for study main effect will be included.

#### 4.4.2.7 Total Number of New T1-Hypo-Intense Lesions (Chronic Black Holes) at Weeks 24, 48, and 96

The statistical analysis of this endpoint will follow the same approach that is used for each study, as described in the individual study-level SAP. The only exception is that in this pooled analysis, a study main effect term will also be included in the statistical model.

#### 4.4.2.8 Change in Multiple Sclerosis Functional Composite Scale Score from Baseline to Week 96

The statistical analysis of this endpoint will follow the same approach that is used for each study, as described in the individual study-level SAP. The only exception is that in this pooled analysis, a study main effect term will also be included in the statistical model.

#### 4.4.2.9 Percentage Change in Brain Volume as Detected by Brain Magnetic Resonance Imaging Scan from Week 24 to Week 96

The statistical analysis of this endpoint will follow the same approach that is used for each study, as described in the individual study-level SAP. The only exception is that in this pooled analysis, a study main effect term will also be included in the statistical model.

#### 4.4.2.10 Change in Quality of Life, as Measured by the Short Form 36 Version 2 Physical Component Summary Score from Baseline to Week 96

The statistical analysis of this endpoint will follow the same approach that is used for each study, as described in the individual study-level SAP. The only exception is that in this pooled analysis, a study main effect term will also be included in the statistical model.

#### 4.4.2.11 Proportion of Patients Who Have No Evidence of Disease Activity by Week 96

The statistical analysis of this endpoint will follow the same approach that is used for each study, as described in the individual study-level SAP. The only exception is that in this pooled analysis, a study main effect term will additionally be included as a stratification factor in the CMH $\chi^2$ test.

### 4.4.3 Exploratory Efficacy Endpoints

The individual study-level SAP describes analyses planned for exploratory efficacy endpoints. The three listed below will be repeated in the pooled analysis of Studies WA21092 and WA21093.

- The proportion of patients who have confirmed disability improvement for at least 24 weeks, with the initial event of neurological improvement occurring during the 96-week, double-blind, double-dummy treatment period
- The duration of the confirmed disability improvement (Kaplan Meier estimate)
- The proportion of patients who at Week 96 have improved, stable, or worsened disability, compared to baseline

### 4.4.4 Subgroup Analyses

The primary and two of the key secondary endpoints (12- and 24-week confirmed disability progression) will be summarized and analyzed by the following subgroups:

- Demography: age ($\geq$40 vs. $<$40 years)
- Demography: sex (male vs. female)
- Demography: race (White vs. other)
- Body weight: $\geq$75 kg versus $<$75 kg
- Stratification factor: geographical region (United States vs. ROW)
- Stratification factor: baseline EDSS score ($<$4.0 vs. $\geq$4.0)
- Previous lesions: baseline Gd-enhancing lesion (0 vs. $>$0)

Estimates of treatment effect and associated 95% CI will be presented in forest plots. Unadjusted p-values will also be presented for these analyses.

#### 4.4.5 Efficacy Data from the Safety Follow-Up Phase

Exploratory analyses of efficacy data collected in the safety follow-up phase are not envisioned for this pooled analysis.

#### 4.4.6 Efficacy Data from the Open-Label Extension Phase

Exploratory analyses of efficacy data collected in the open-label extension phase are envisioned. Additional analysis will be undertaken after efficacy data are more mature.

## 4.5 PHARMACOKINETIC AND PHARMACODYNAMIC ANALYSES

### 4.5.1 Pharmacokinetic Analyses

The individual study-level SAP provides some detail on the planned PK analyses on pooled data. Further details will be described in a Modeling and Simulation Analysis Plan, and results will be reported separately.

### 4.5.2 Pharmacodynamic Analyses

The individual study-level SAP provides some detail on the planned PD analyses on pooled data, and the results of the analyses on the pooled data will be reported in the PK report.

## 4.6 SAFETY ANALYSES

The individual study-level SAP provides information regarding safety analyses including exposure to study medication, Columbia–Suicide Severity Rating Scale, adverse events, laboratory data, and vital signs to be performed for each study individually.

The Summary of Clinical Safety SAP defines five data poolings, denoted Pool A to Pool E, which will be used to report clinical safety for the ocrelizumab program. Only one of these, Pool A, will be used here. Other data pools do not apply here.

### 4.6.1 Pool A: Phase III Relapsing Multiple Sclerosis Controlled Treatment Population

The purpose of the Phase III RMS controlled treatment population is to provide combined comparative safety data of ocrelizumab 600 mg relative to interferon beta-1a 44 μg SC within the RMS indication.

This population is defined in the same way as the Safety Population within the individual primary study CSRs.

Baseline measurement is defined as the last available measurement prior to the first study drug administration.

Summaries will be presented by ocrelizumab 600 mg and interferon beta-1a 44 μg SC for the pooled data. These summaries will contain data from the following:

- The 96-week controlled treatment period of the study
- Safety follow-up data up to 96 weeks after first dose of study treatment for those patients who withdrew early

The summaries will not include the open-label extension (OLE) data. These are the same data (i.e., variable clinical cut-off dates [patient CCODs] for each patient 96 weeks from treatment start) as for the individual primary CSRs.

#### 4.6.2 Exposure to the Drug

"Duration of observation" and "duration of exposure" are used interchangeably within this SAP.

The definition of dose is ocrelizumab given as one infusion or two infusions administered 2 weeks apart.

These studies are designed to collect safety data from all patients for a minimum of 48 weeks after the last ocrelizumab/ocrelizumab placebo infusion. B-cell monitoring and safety follow-up of each patient will last until the B-cell count has returned to the baseline value or to the lower limit of the normal range (whichever is lower). Adverse events and other safety data are being collected during this extended period as part of the protocol.

To avoid exclusion of any adverse events recorded within the studies (in particular infections), observation years will be used to calculate the rates of events per 100 patient-years (see Section 4.6.4.2). It is recognized that the number of events that occur could decline during the safety follow-up period, thus potentially lowering this rate. However, for as long as B cells are not repleted, ocrelizumab is considered to exert its pharmacodynamic effect (albeit to a lesser extent as the time interval since last infusion increases). As a consequence, a patient is considered to be potentially at risk for as long as B cells are not repleted. Therefore, the rate on the control arm is expected to be lowered to a greater extent than on the active ocrelizumab arm, thus widening the treatment difference in favor of the control arm. This would still be a conservative approach for ocrelizumab in any comparison to interferon beta-1a 44 μg SC for those risks known to be associated with interferon beta-1a, as more observation time is being included within this treatment arm while the patient has been off treatment. For patients who withdraw early during the controlled treatment phase of the studies, observation time in safety follow-up will be included.

Patients will be considered to have received a treatment dose if at least part of one infusion of that dose (either Day 1, or Day 15 for dual infusions) was given. If a dose is completely missed instead of delayed, the next dose number will be on the basis of the number of previous doses received.

The duration of observation for a patient will be calculated as:

(Date of last contact $-$ date of first dose) $+1$

Here, the date of last contact is defined as the earliest of the:

- date of CCOD for the reporting event (for the controlled treatment period, the CCOD will be variable for each patient at 96 weeks from treatment),
- date of last contact from the study completion page (for Studies WA21092 and WA21093); or,
- date of death

For patients who are receiving interferon beta-1a 44 μg SC who switch to active ocrelizumab treatment, interferon beta-1a exposure will end on the study day prior to their first active dose of ocrelizumab.

The duration of observation within a dose is defined in a similar way:

(Day prior to first infusion in $n+1^{th}$ dose $-$ date of first infusion in $n^{th}$ dose) $+1$

An exception exists if the last dose received by the patient is on the basis of the date of last contact as defined above.

Exposure to ocrelizumab and exposure to interferon beta-1a during the controlled treatment phase will be summarized. For ocrelizumab, the treatment duration, number of doses received, and the total cumulative dose (defined in mg) will be summarized by study treatment arm. An additional summary will be presented, summarizing at each dose the percentage of patients who received $<80\%$ and $\geq 80\%$ of the planned infusion. The number and percentage of infused patients who were pre-treated with steroids will be summarized. For interferon beta-1a, the number of doses and dose intensity (based on the number of syringes received rather than the actual dose) will be presented by study treatment arm.

#### 4.6.3 Treatments

All treatments that started prior to the first dose of study drug and were ongoing at study baseline (prior, concurrent) plus all treatments that started on or after the first dose of study medication (concomitant) will be summarized by treatment group, by class, and by preferred term (PT).

Additionally, prior treatments for MS and prior MS treatments that are currently ongoing at study baseline will be presented separately, by class and PT.

#### 4.6.4 Common Adverse Events

Adverse events will be defined as all adverse events, infusion-related reactions (IRR), and serious MS relapses, excluding non-serious MS relapses. A separate listing with relapses that are considered to be serious adverse events will be presented.

For each adverse event recorded, the term entered by the investigator describing the event (the “reported term”) will be assigned a standardized term (the “preferred term”) and assigned to a superclass term on the basis of the Medical Dictionary for Regulatory Activities (MedDRA) World Health Organization (WHO) dictionary of terms. All studies to be pooled together will be re-coded to a common version of MedDRA, which may be a later version than that used to report the adverse events in the CSRs. All analyses of adverse event data will be performed using the PTs, unless otherwise specified.

All treatment emergent adverse events will be included from the start of study treatment through the entire recorded observation for the patient. An adverse event is considered to be treatment emergent if the adverse event started on or after the start date of study treatment or if the intensity worsened during study treatment. An adverse event with a completely missing non-imputed start date will be assumed to be treatment emergent unless the adverse event has a complete non-imputed end date that is prior to the start of study treatment.

An adverse event profile table will be produced specifically for the body of the safety summary. The table will contain frequencies and percentages.

An additional adverse event profile table presenting the number of events per 100 patient-years will be produced.

#### 4.6.4.1 Frequency Counts of Adverse Events

All adverse events will be summarized by system organ class (SOC) and PT.

For frequency counts by PT, multiple occurrences of the same adverse event in a patient are counted only once. These tables will also be presented by SOC only for the body of the safety summary. Additionally, the most frequent adverse events ($\geq$5% in any treatment arm) will be presented by PT for the same populations.

#### 4.6.4.2 Number of Adverse Events per 100 Patient-Years

The number of adverse events per 100 patient-years observation, including 95% CI will be presented by SOC and PT.

The CI will be calculated using the exact method based on the Poisson distribution.

The number of adverse events per 100 patient-years will be calculated as:

- (Number of adverse events observed/ Total patient-years observation) $\times$ 100

Multiple occurrences of the same adverse event in one patient will be counted multiple times. Total patient-years observation will be calculated as the sum over patients of the time intervals (in years) between the start of study therapy to the end of the adverse event reporting period. This approach assumes that during an adverse event, patients remain at risk for additional events.

#### 4.6.4.2.1 Incidence Rate Ratio

The relative safety of ocrelizumab 600 mg to interferon beta-1a 44 μg SC will be compared using an incidence rate ratio for any adverse event and a 95% CI adjusted for the study.

The incidence rate ratio of ocrelizumab 600 mg to interferon beta1-a 44 μg SC will be calculated using a Poisson regression model, including treatment group and study as covariates.

#### 4.6.4.2.2 Difference of Incidence Rate

The combined incidence rate difference of any adverse event and 95% CI will be presented by calculating the weighted average of the rate differences estimated for each study.

The inverse variance method will be used to weight the study estimates of the rate difference by the inverse of its variance (i.e., a fixed-effects summary-level meta-analysis). These results will be presented using a meta-analysis forest plot.

### 4.6.4.3 Adverse Events per Treatment Dose

Adverse events will be assigned to a dose if the adverse event onset date is on or after the date of the first infusion of that dose, but before the first infusion of the next treatment dose. Adverse events that are reported during the safety follow-up period or B-cell monitoring period will be assigned to the last dose received. Hence, the last dose for a patient may be of a variable length from 24 to 72 weeks or longer. Adverse events that start prior to first dose and worsen during treatment (i.e., treatment emergent) will be assigned to the first treatment dose.

The number of any adverse events per 100 patient-years observation will be summarized by dose and based on the number of patient-years observation for the specific dose (see Section 4.6.4.2 for definition) to assess whether the rate of any adverse event is increasing by dose.

### 4.6.4.4 Related Adverse Events

The number of patients who experienced a related adverse event will be summarized by SOC and PT.

### 4.6.4.5 Adverse Events by Intensity

Adverse events will be summarized by SOC, and PT by intensity grade.

Multiple occurrences of the same event within a patient will be counted once, at the greatest intensity/highest grade for this PT. For adverse events leading to death, the most extreme/greatest intensity will be overwritten by Grade 5 (death). Any adverse events and the rows of the overall SOC of the summary table will count patients according to adverse events by intensity/grades.

#### 4.6.5 Deaths

All patient deaths, regardless of treatment received, will be listed by study.

#### 4.6.6 Serious Adverse Events

Serious adverse events will be defined as all serious adverse events and serious IRRs, including serious MS relapses.

The number of patients who experienced a serious adverse event and the number of serious adverse events per 100 patient-years observation will be summarized.

Related serious adverse events will be summarized by SOC and PT. Additionally, the most frequent serious adverse events ($\geq$1%) will be presented by PT.

The number of any serious adverse events per 100 patient-years observation will be summarized by dose and will be on the basis of the number of patient-years observation for the specific dose (see Section 4.6.4.2 for definition). The incidence rate ratio of any serious adverse events and 95% CI adjusted for study will be presented; the incidence rate ratio of ocrelizumab to interferon beta1-a 44 $\mu$g SC will be calculated using a Poisson regression model, including treatment group and study as covariates.

#### 4.6.7 Adverse Events That Lead to Withdrawal of Study Treatment

A patient may experience an adverse event that leads to the discontinuation of his or her study treatment. Discontinuation of study treatment for an adverse event may not necessarily lead to discontinuation from the study because patients can enter the safety follow-up periods of the protocols. For this section, only adverse events that lead to the discontinuation of study treatment are of interest. Patients who withdraw early from the study because of adverse events will be summarized under disposition.

Because of the double-dummy design of the Phase III RMS Studies WA21092 and WA21093, the study drug regimen consists of more than one combination of two treatments (ocrelizumab and interferon beta-1a 44 $\mu$g SC). An analysis of adverse events leading to study drug discontinuation will not be performed for the individual treatments of the regimen within one study arm for the controlled treatment population, as the investigators were instructed to discontinue both treatments.

The number of patients who experienced an adverse event that led to discontinuation of study treatment will be summarized by SOC and PT.

Summaries will also be presented by dose. Adverse events that lead to withdrawal will be assigned to dose based on their last infusion received prior to the event. It is assumed that adverse events that occur on the day of an infusion will be post-infusion.

### 4.6.8 Adverse Events Leading to Modification or Interruption of Study Treatment

The number of patients who experienced an adverse event that leads to modification or interruption of study drug will be summarized by SOC and PT.

Summaries will be presented overall and by dose. Adverse events will be assigned to a dose on the basis of the start date of the adverse event in relation to the date of the first infusion of the dose. It is assumed that adverse events that occur on the day of an infusion will be post-infusion.

### 4.6.9 Selected Adverse Events

The following sections cover the selected adverse event for the ocrelizumab RMS program, identified prior to the unblinding of the first Phase III study.

Some analyses that are pre-specified within this section of the SAP may not be generated if the number of events is considered to be too small for meaningful interpretation. In these cases, any changes to the analyses will be justified in the report.

#### 4.6.9.1 Infusion-Related Reactions

Data for IRRs and their corresponding symptoms are collected on the dedicated electronic case report form (eCRF).

The symptom(s) of an IRR and the IRR itself may be of differing intensities. As other symptoms can be recorded as free-text on this page, symptoms will be coded using MedDRA. Symptoms will be summarized by PT (not the pre-printed terms on the eCRF; thus, they can be combined across studies).

IRRs are categorized on the eCRF as occurring 1) ‘during infusion,’ 2) ‘after completion of infusion while patient is in clinic,’ and 3) ‘within 24 hours of completion of the infusion and patient is not in clinic.’

For IRRs that lead to death, the most extreme intensity will be overwritten by Grade 5 (death). These rules will only apply to the IRR events and not the symptoms of the IRR.

The following IRR and symptoms of IRR outputs will be presented by infusion.

- Percentages for the number of patients with at least one IRR will be based on the number of patients within the respective population that received that specific infusion.
- The proportion of patients with an IRR per infusion will be presented. The number of serious IRRs will also be presented.
- The number of patients with at least one IRR, the total number of IRRs, and the number of IRRs that occurred 1) during the infusion, 2) after the infusion while the patient was in the clinic, or 3) within 24 hours of completion of the infusion (outside of the clinic) will be summarized by infusion

- The number of patients with at least one IRR, the total number of IRRs, and the intensity of the IRR will be presented by infusion and by the time of onset as recorded on the eCRF 1) during the infusion, 2) after infusion while the patient is in clinic, or 3) after the patient leaves the clinic)

Additionally, similar tables will be presented by the pre-medication subgroup (methylprednisolone alone, methylprednisolone plus analgesic/antipyretics, methylprednisolone plus antihistamines, methylprednisolone plus analgesic/antipyretic and antihistamines).

Symptoms of IRRs and symptoms of serious IRRs will be presented by infusion. Symptoms of IRRs that led to modification of study medication or symptoms of IRRs that led to discontinuation of study medication will also be presented. Symptoms of IRRs that were experienced by patients during an infusion, after the infusion, and after the patient leaves the clinic will also be presented.

IRRs and their symptoms will be listed.

Adverse events other than IRRs experienced by the patient within 24 hours of an infusion will be presented by infusion. Adverse events will be included if the onset date is on the day of an infusion or the following day. The onset date of the adverse event will be matched to the date of an infusion; onset dates will not be imputed.

The number of IRRs per patient will be summarized by treatment only.

This summary will be limited to patients who received the first four doses of treatment.

#### 4.6.9.2 Infections

Infections will be defined using the adverse event data and the MedDRA SOC of infections and infestations. Adverse events that are reported by the investigator as an infection will also be included.

An infection will be defined as serious if the event is a serious adverse event as defined in Section 4.6.6 or if the non-serious infection was treated with an intravenous (IV) anti-infective. A listing will be presented of non-serious infections treated with an IV anti-infective.

Infections will be classified according to the pathogen type (e.g., bacterial, fungal, viral, parasitical, unknown, or other).

Opportunistic infections (OI) will be defined using the ocrelizumab MS specific adverse event glossary of terms (AEGT) of opportunistic infections.

The analyses to be performed are:

- The number of patients who experienced an infection, summarized by SOC and PT

- Infections experienced by more than 5% of patients
- The number of any infection events per 100 patient-years observation (rates will not be presented by PT)*
- The number of patients who experienced a serious infection, summarized by SOC and PT
- The number of any serious infection events per 100 patient-years observation (rates will not be presented by PT)*
- The number of any serious infection events per 100 patient-years observation, summarized by dose and based on the number of patient-years observation for the specific dose

*For these analyses, the incidence rate ratio with 95% CI, adjusted for study via a Poisson regression, will be presented. In addition, the combined incidence rate difference with 95% CI will be presented by calculating the weighted average of the rate differences estimated by each study (see Section 4.6.4.2).

Infections and serious infections will be summarized according to suspected or identified pathogen type.

Infections will also be categorized into the following types using ocrelizumab MS specific AEGT of the same name:

- Upper respiratory tract infections
- Lower respiratory tract infections
- Urinary tract infections
- Skin infections
- CNS infections
- Herpes virus-associated infections

Infections will be summarized by these types, and the number of any infection events of these types per 100 patient-years observation will be presented. These summaries will be presented overall and by dose for infections and serious infections separately.

Infections will be summarized by SOC and PT by intensity (see Section 4.6.4.5).

The following analyses regarding OI will be performed:

- Number of patients who experienced an OI, summarized by SOC and PT
- Number of any OI events per 100 patient-years observation*

  *For these analyses for the controlled treatment population, the incidence rate ratio with 95% CI, adjusted for study via a Poisson regression, will be presented.

#### 4.6.9.3 Malignancies

Malignancy selected adverse events will be defined using the Standard MedDRA Query of "Malignant tumours (narrow)."

The analyses to be performed are:

- Number of patients with a malignancy, summarized by SOC and PT
- Number of any malignancy events per 100 patient-years observation

The incidence rate ratio and 95% CI, adjusted for study via a Poisson regression model, will also be presented, along with the combined incidence rate difference and 95% CI calculated by the weighted average of the rate differences estimated by each study (see Section 4.6.4).

The following analyses will be performed:

- Summaries of malignancies presented by dose; the percentages will be based on the number of patients that received treatment in that specific dose
- Number of any malignancy events per 100 patient-years observation by dose and based on the number of patient-year observations for the specific dose

#### 4.6.9.4 Clinical Laboratory Evaluations

This section of the SAP covers the analysis of the standard hematology parameters (CBC), liver laboratory parameters, and the following laboratory tests of special interest for patients treated with ocrelizumab: CD19 B-cell counts and the development of ADAs.

Summaries will use all laboratory data from scheduled, unscheduled, and repeat tests; this includes laboratory data collected within the safety follow-up or prolonged B-cell monitoring periods of a study.

When laboratory data are presented over time, these laboratory values will be time windowed into a common visit structure. If multiple values of the same laboratory parameter occur within the same time window, the worst value for that parameter will be presented in the summary table.

Laboratory parameters will be presented over time by treatment group. The baseline value will be the last value prior to the first dose of study medication.

#### 4.6.9.5 General Laboratory Evaluations

The following laboratory parameters will be summarized:

- Hematology/ CBC laboratory parameters:  hemoglobin, hematocrit, RBC, WBC (absolute and differential), ANC, and quantitative platelet count
- Liver laboratory parameters:  AST/SGOT, ALT/SGPT, and total bilirubin
- Other:  creatinine

The number and percentage of patients with a laboratory abnormality will be summarized for each parameter.  In addition, for the liver laboratory parameters, the number and percentage of patients with an elevated post-baseline AST or ALT level during the controlled treatment period will be summarized by treatment.

#### 4.6.9.6 CD19 Cell Counts

The median CD19 cell count will be displayed graphically over time from the first dose of study medication.  The absolute CD19 count and change from baseline in the CD19 count will be summarized over time.  At each timepoint, the number and percentage of patients whose CD19 counts have repleted will be presented.  Repletion is defined as the CD19 counts having returned to their baseline value or to the lower limit of normal ($\geq$80 cells/μl), whichever is lowest.

#### 4.6.9.7 Anti-Drug Antibodies

ADA (also called human anti-human antibodies [HAHAs]) results will be summarized. The baseline prevalence and post-baseline incidence of ADAs will be displayed on this table.

Additional analyses to explore whether the development of positive ADA following ocrelizumab exposure is associated with changes in safety will only be performed if a meaningful number of patients experience an ADA post-ocrelizumab exposure.  In this case, the overall adverse event profile tables will be presented by ADA status.  Adverse events will be listed for patients who experience a treatment-emergent ADA, and adverse events pre and post this positive result will be flagged.

### 4.6.10 Subgroup Analyses

For each subgroup, the adverse event profile table presenting the number of events per 100 patient-years will be presented.

This profile table will be supplemented by the number of patients who experience adverse events, serious adverse events, infections, and serious infections, which will be presented by SOC and PT.

The subgroups to be explored are as follows:

- Sex (male, female)
- Race (White, Black, and other)

- Body weight (< 75 kg, ≥ 75 kg)
- Geographical region (United States, ROW)
- Geographical region 2 (European Union/Switzerland/Norway, Latin America, Non-EU Europe/Israel/Africa, United States/Canada/Australia)
- Prior cardiovascular disease (yes, no)
- Prior diabetes (yes, no)

## 4.7 MISSING DATA

All methods for handling missing data and associated sensitivity analyses are described in the individual study-level SAP, section by section, for each endpoint. This pooled analysis will use the same approach to missing data as described in the individual study-level SAP.

## 4.8 INTERIM ANALYSES

No interim analyses of efficacy data are planned.

## 4.9 SUBGROUP ANALYSES FOR EUROPEAN UNION REGULATORY PURPOSES

Subgroup analyses requested by the European Medicines Agency, consisting of patients who have shown clinical and/or MRI activity in the year prior to randomization, will be undertaken to support the European filing, as follows:

- Active inadequate responder subgroup: patients treated with interferon or glatiramer acetate for ≥ 1 year and:

  Have had at least one relapse in the last year prior to randomization or at least one baseline Gd-enhancing lesion

- Highly active inadequate-responder subgroup: patients treated with interferon or glatiramer acetate for ≥ 1 year and had at least one relapse in the previous year and:

  Have at least nine T2-lesions* or

  Have at least one Gd–enhancing lesion*

  * at baseline

- Active treatment-naive subgroup: treatment-naive patients with at least two relapses in the previous 2 years and at least one relapse in the last year prior to randomization
- Highly active treatment-naive subgroup: treatment-naive patients with at least two relapses in the last year prior to randomization and:

  ≥ 1 baseline Gd lesion or

  Increase in T2 lesion count at baseline visit (changing from 0–5 to 6–9 lesions or from 6–9 lesions to > 9 lesions), as compared with the prior MRI

For these subgroups, the following efficacy endpoints will be summarized and analyzed:

- Annualized protocol-defined relapse rate by 2 years (96 Weeks)
- Time to onset of CDP for at least 12 weeks, with the initial event of neurological worsening occurring during the 96-week, double-blind, double-dummy treatment period
- Total number of T1 gadolinium (Gd)–enhancing lesions as detected by brain MRI at Week 96
- Proportion of patients who have CDI for at least 12 weeks, with the initial event of neurological improvement occurring during the 96-week, double-blind, double-dummy treatment period
- Time to onset of CDP for at least 24 weeks, with the initial event of neurological worsening occurring during the 96-week, double-blind, double-dummy treatment period

In addition, for safety purposes, the adverse event profile table presenting the number of events per 100 patient-years will be presented for each subgroup. This profile table will be supplemented by the number of patients who experience death, adverse events, serious adverse events, IRRs, serious IRRs, infections and serious infections.
